# Underpinning beneficial maize response to application of minimally processed homogenates of red and brown seaweeds

**DOI:** 10.3389/fpls.2023.1273355

**Published:** 2023-11-30

**Authors:** Pradipkumar Vaghela, Grishma Gandhi, Khanjan Trivedi, K. G. Vijay Anand, Dhruvil Chavda, Moutusi Manna, Tanmaye Seth, Abhiram Seth, Munisamy Shanmugam, Arup Ghosh

**Affiliations:** ^1^ Academy of Scientific and Innovative Research (AcSIR), Ghaziabad, India; ^2^ Council of Scientific & Industrial Research (CSIR) - Central Salt and Marine Chemicals Research Institute, GB Marg, Bhavnagar, Gujarat, India; ^3^ Aquagri Processing Private Limited, Delhi, India

**Keywords:** tandem mass spectroscopy, reactive oxygen species, biostimulants, maize, untargeted metabolites, *Kappaphycus alvarezii*, *Sargassum wightii*

## Abstract

Sap from the fresh seaweed *Kappaphycus alvarezii* (KA) has been reported to improve crop growth, quality, and stress alleviation. However, limited studies are reported for the minimally processed aqueous homogenates (MPHs) derived from dry seaweeds. The present investigation was envisaged to characterize the MPHs from the red seaweed KA and a brown seaweed *Sargassum wightii* (SW) and also assess the effect of foliar application on maize (*Zea mays*) crop performance when applied alone or in proportions ranging from 0% to 100%. Two doses (0.35% and 0.7%) were compared with control. Both the MPHs contained several compounds like retronecine, tyrosyl-glycine, hexyl 2-furoate, 1-phosphatidyl-1D-myo-inositol, 12-(2,3-dihydroxycyclopentyl)-2-dodecanone, and trihomomethionine and many others that have known bioactivity for enhancing plant growth and providing stress tolerance. Both doses of MPHs enhanced crop growth and yield; however, the best response was in general observed at a lower dose. The MPH of SW at 100% gave the highest seed yield at a lower dose, which was also on par with that obtained under a lower dose of 100% KA. Other combinations, 80:20 and 40:60 KA : SW, were also found to give comparable yields. The highest dose of 100% MPH of SW was found on par with control, a phenomenon that was investigated in detail with respect to metabolites and antioxidant profile in leaves as well as membrane modeling. Higher ROS and certain sugar and organic acids were observed in 100% MPH of SW at a higher dose, although none of the antioxidant enzymes were significantly affected, nor was there any change in membrane characteristics of the leaf with respect to control as well as lower dose. Improvements in the seed yield were attributed to improved photosynthate production on account of higher dry matter accumulation in the MPH-treated plants, which may also be attributed to the presence of bioactive compounds in the biostimulants. In the future, it is imperative to direct scientific investigations towards the quantification and identification of the most effective concentrations of these compounds within MPHs to optimize plant responses. The study indicated the beneficial use of the MPHs towards increasing crop production by employing optimum dose as foliar spray to crops.

## 1 Introduction

As the world’s population continues to grow and the demand for food increases, there has been a significant rise in the consumption of conventional chemical fertilizers. This has led to growing concerns regarding its impact on the environment, soil health, human health, and the economy (Eissa et al., 2017; Jjagwe et al., 2020). With the aim of promoting sustainable agricultural practices, many farmers have shifted away from using synthetic fertilizers and turned to organic farming methods. As part of this movement, the use of seaweed-based biostimulants has gained popularity as a natural and environment-friendly way to boost agricultural productivity (Singh et al., 2015; Singh et al., 2018). Seaweed extracts, or various formulations derived from red, brown, or green algae, among other taxonomically diverse types of algae, have been used as biostimulants (Del Buono et al., 2021). Some of the most used species as sources of biostimulants include *Ascophyllum nodosum, Ecklonia maxima, Sargassum* spp., *Gracilaria* spp., and *Kappaphycus alvarezii* (KA). These seaweeds are widely available on the market and are used to produce a range of commercial products (Sharma et al., 2014). The use of seaweed as a plant biostimulant is currently one of the most promising applications. Despite being used in agriculture since the Roman era, the mechanism by which seaweed-based biostimulants enhance growth and production is not yet fully understood (Calvo et al., 2014). KA belongs to the Rhodophyceae family and is currently one of the most widely cultivated seaweed species globally (Ferdouse et al., 2018). Its cultivation technology for tropical seas has been well standardized (Mantri et al., 2017). Given that many of these organisms can be easily cultivated or harvested from their natural habitats, they offer a cost-effective source of feedstock for the production of biostimulants that can enhance the growth, yield, and resilience of agricultural crops (Ragaza et al., 2015). The biostimulant obtained from KA has a low carbon dioxide emission footprint (Ghosh et al., 2015). The tropical seaweeds KA and *Sargassum wightii* (SW) are promising sources of biostimulants that can enhance crop productivity. Several studies have demonstrated the benefits of using seaweed-based biostimulants to improve the yields of important cash crops like maize (Singh et al., 2015; Trivedi et al., 2018), rice (Sharma et al., 2017), sugarcane (Singh et al., 2018), pulses (Biswajit et al., 2013), oilseeds (Rathore et al., 2009), medicinal plants (Elansary et al., 2016), and horticultural crops (Battacharyya et al., 2015). Seaweeds contain various bioactive ingredients including plant growth regulator (Prasad et al., 2010; Vaghela et al., 2023); quaternary ammonium compounds like glycine betaine, choline chloride, and cetrimonium (Trivedi et al., 2017; Trivedi et al., 2022; Vaghela et al., 2023); polyphenols (Rengasamy et al., 2015; Vaghela et al., 2023); oligosaccharides and polysaccharides (González et al., 2013; Shukla et al., 2016); amino acid, amine derivative, kinetin, and canavanine; and many other classes of compounds (Vaghela et al., 2022; Vaghela et al., 2023). These compounds either acting alone or synergistically have been implicated in eliciting the observed crop physiological responses to seaweed biostimulant application including KA and SW extracts.

Fresh sap derived from *K. alvarezii* has been extensively studied as a biostimulant. However, reports on minimally processed aqueous homogenates (MPHs) obtained from dry seaweeds are limited. The objective of this study was to investigate two MPHs derived from KA and SW in order to identify untargeted metabolites present in them using high-resolution mass spectrometry (HRMS) and as well as to investigate their potential physiological role in growth, development, and yield of maize crop when applied exogenously either alone or in combinations.

## 2 Materials and methods

### 2.1 Preparation of minimally processed homogenates of seaweeds

The preparation of minimally processed homogenate(s) or MPH(s) of the respective seaweeds has already been described in Vaghela et al. (2023). Briefly, the sun-dried algae (1 kg) were shredded into 10-cm pieces and rehydrated with tap water (6 L) by soaking them in ambient conditions in the ratio of 1:6 (w/v of seaweed: tapwater). The resulting mixture was filtered twice using a 200-mesh filter and spray dried using a triple effect evaporator into dry powders.

### 2.2 Sample processing for liquid chromatography

Extraction of the samples for liquid chromatography was carried out using the procedure described by Vaghela et al. (2022). In brief, 2.5 g of MPHs of seaweed was mixed with 98% v/v methanol, maintaining a solid/solvent ratio of 1:10 w/v, and stirred at 200 rpm for 24 h using a magnetic stirrer at room temperature. The extracts of both MPHs were then filtered through Whatman No. 1 filter paper, and the obtained filtrates (crude extract) were collected. Residues left on the filter papers were re-extracted with 10 mL of 98% v/v methanol, and the filtrates were pooled. The pooled filtrates were then concentrated to a volume of 5 mL using a rotary evaporator (Buchi-US, vacuum pressure 500 bar, water bath temperature 63°C). The samples were filtered using a 0.22-micron syringe filter, and further concentrated using nitrogen gas flow until a sample volume of 2 mL was achieved. The resulting samples were stored at −20°C for subsequent analysis by Q-TOF-HRMS (Quadrupole-Time of Flight-High Resolution Mass Spectroscopy) at the Sophisticated Analytical Instrument Facility (SAIF), IIT, Powai, Mumbai.

#### 2.2.1 LC-Q-TOF-MS instrument setup condition and data acquisition

The liquid chromatographic separations were conducted using a reversed-phase C18 analytical column with TMS end-capping, measuring 150 mm × 2 mm and with a 5-µm particle size (Luna ®, Phenomenex, Torrance, USA). The column temperature was set at 40°C and the sample injection volume was 5 µL. The mobile phase, composed of 0.1% formic acid in water (A) and acetonitrile (B), was run for a total of 30 min (**Supplementary Table 1**), with the flow rate maintained at 0.3 mL min^−1^ using a binary pump (Model G4220B). The sampler temperature was set at 4°C. The chromatographic system was coupled to a quadrupole time-of-flight high-resolution mass spectrometer (Agilent Technologies, model – G6550A Q-TOF-MS) equipped with DUAL AJS electro-spray Jet Stream Technology. The MS analysis was performed in ESI-positive and -negative ionization modes with the following parameters: gas temperature: 250°C; drying gas flow: 13 L min^−1^; nebulizer pressure: 35 psig; sheath gas temperature: 300°C; sheath gas flow: 11 L min^−1^. The set scan source parameters included capillary voltage: 3,500 V; nozzle voltage: 1,000 V, fragmentor voltage: 175 V; skimmer voltage: 65 V; and octopole RF peak: 750 V. The Q-TOF-HRMS recorded accurate mass spectra in the range of 120–1,100 m/z with a scanning rate of one spectrum per second. The MS Abs threshold value was set at 200, while MS Rel. threshold (%) was set at 0.010. Auto MS/MS analysis was conducted in ESI-positive and -negative ionization modes using the direct injection technique, with a flow rate of 30 µL min^−1^. The MS/MS Abs threshold value was set at 5, while MS/MS Rel. threshold (%) was set at 0.010. The MS/MS scanning rate was set at one spectrum per second.

#### 2.2.2 Data processing and molecular feature extraction and annotation of metabolites

The Q-TOF-HRMS (Quadrupole-Time of Flight-High Resolution Mass Spectrometry) instrument was used for untargeted data acquisition, with Agilent MassHunter™ B.06 software version B.05.01 (B5125) (Agilent Technologies, Santa Clara, CA, USA) employed for this purpose. The resulting data were processed with MassHunter Qualitative Analysis software (Agilent Technologies). Using the Molecular Feature Extractor (MFE), which is a proprietary untargeted data-mining algorithm, the total ion chromatogram (TIC) data were deconvoluted into individual chemical compound peaks. The MFE algorithm utilized the accuracy of the mass measurements to group-related ions, based on their isotopic distribution, charge-state envelope, and/or the presence of adducts, dimers, or trimers. It assigns multiple species (ions) related to the same neutral molecule to a single compound, called a “feature”, and could identify multiple compounds within a single chromatographic peak. The intensity of each compound peak was calculated as the sum of its isotopic peaks, adduct ion peaks, and molecular ion peaks. The putative compound annotation was performed using the METLIN Personal Metabolite Database (DB) and Molecular Formula Generation (MFG) software (Agilent Technologies). Molecular formula candidates were derived and ranked based on their relative probabilities, using MassHunter MFG software, which calculated an abundance-weighted, combined cross-species score for each molecular formula based on accurate mass measurements and additional information on covariant species of the features deduced from the isotopic abundance and distribution. The provisionally annotated compounds were those with an accurate mass within the specified mass tolerance window, along with a corresponding empirical formula assigned to the feature by a match to an annotated METLIN database (DB). The compounds that matched one or more annotations in the database were reported, showing the best metabolite hit for each mass, along with the number of hits in the DB and the corresponding mass differences with respect to the DB (referred to as DB difference and expressed in ppm).

### 2.3 Study area and experimental design

The study was conducted during the *rabi* season (November 2019 to February 2020) at the Central Salt and Marine Chemicals Research Institute (CSMCRI), Bhavnagar, Gujarat, India, in a net house facility located at 21° 44′ 57.6′′ N–72° 08′ 39.3′′ E. Sandy loam soil with an initial pH of 7.88 and an electrical conductivity of 0.24 dS m^−1^ was used. The soil had an organic carbon content of 1.71%, while the available N, P, and K were 110, 15, and 166 kg ha^−1^, respectively. Seeds of *Zea mays* L. Sugar 75 hybrid (Syngenta) were used as the test crop, and the greenhouse experiment was laid out in a two-factor Completely Randomized Block Design (F-CRBD) with 18 treatments (**Supplementary Table 7**) and five replications under irrigated conditions. The first factor included six levels of MPHs: T1-100:0 KA : SW, T2-80:20 KA : SW, T3-60:40 KA : SW, T4-40:60 KA : SW, T5-20:80 KA : SW, and T6-0:100 KA : SW. The combinations were prepared by reconstituting MPHs in 3.5% (w/v) solution and mixing each of the extracts in appropriate amounts, i.e., 80 mL of KA extract solution and 20 mL of SW extract solution to obtain 80:20 combination and so on. Each of these six levels was applied as foliar spray at 0%, 10%, and 20% (v/v) concentrations of the spray volume, respectively, which is equivalent to a concentration of 0.35% or 0.7% (w/v) on total solid content basis of the MPHs, and this formed the second factor. The reconstituted MPHs were in suspension and were thoroughly mixed homogeneously with slight agitation. Ninety earthen pots were filled with a soil mixture (32 kg) prepared in a 6:4:1 proportion of black soil:red soil:farmyard manure. Each pot was sown with four seeds, which was later thinned to a single plant per pot, which constituted a single replication. The treatments were applied thrice at 31 days after sowing (DAS) (V5; 5th leaf collar), 56 DAS (V10; 10th leaf collar), and 85 DAS (V15; 15th leaf collar) in pots, with a spray volume of 20, 29, and 32.5 mL per plant required, respectively, in maize. Fertilizers were applied at the rate of 120:60:40 N:P:K kg ha^−1^, which was 1.7 g N (in two splits, the first at the time of sowing and the second before V10 stage), 0.857 g P_2_O_5_, and 0.57 g K_2_O to each of the pots through urea, single super phosphate, and muriate of potash (IFFCO brand), respectively. Before the experiment, all plants received irrigation as per the regular schedule, at the rate of 2 L of water per pot applied manually every second day as a drench till until harvest (92 DAS). No pesticides were applied and weeding was done manually as and when required.

### 2.4 Growth, yield, and photosynthetic attributes

The height of the plants was measured from the soil surface up to the tassel during the harvest, while the stem diameter was measured at 3 cm above the soil surface. After the harvest, the fresh and sundried weights of various plant parts (such as leaf, stem, and root) were recorded. The roots were carefully removed from the soil and cleaned by hand to remove soil particles. To obtain any remaining small roots in the soil, the soil was sifted. Any soil particles attached to the root fibers were removed using a damp paper towel. The fresh weight of the roots was then recorded.

After harvest, the leaf, stem, and root were sun-dried to measure the dry matter accumulation and expressed in grams per plant.

Yield and yield attributes were measured right after harvesting the fresh cobs. Cob length and fill length were measured without leaf cover. The grains were separated from the cobs after sun-drying, counted, weighed, and expressed as grams per plant. The number of seeds, test weight (per 100 seeds), and the total seed weight per plant were also determined.

The photosynthetic rate was measured using an infrared gas analyzer system (IRGA; Model-Li-6400XT, LI-COR, USA) on the last fully matured leaf. The photosynthetic photon flux density (PPFD) was maintained at 1,000 µmol m^−2^ s^−1^, and a fixed CO_2_ concentration was maintained in the leaf chamber by continuously allowing air to pass through from an open end.

### 2.5 Maize leaf biochemical analysis

Leaf samples were collected from maize plants after V10 treatments (56 DAS) for biochemical analysis. The leaves were immediately snap-frozen in liquid nitrogen and stored at −80°C until further analysis.

#### 2.5.1 Antioxidant enzyme extraction

All procedures were performed under chilled conditions of 0–4°C. Leaf tissue weighing 0.1 g fresh weight was rapidly frozen in liquid nitrogen and pulverized in a cold mortar and pestle. The extraction buffer (pH 7.5) that consisted of 50 mM Tris, 0.1 mM ethylene-diamine tetra acetic acid (EDTA), 0.2% Triton X-100, 1 mM polymethyl sulfonyl fluoride (PMSF), and 2 mM dithiothreitol was used to homogenize the samples. After vigorous vortexing, the homogenates were centrifuged at 18,000 × *g* for 30 min at 4°C. The supernatant was carefully collected and stored at −80°C for further analysis.

For the ascorbate peroxidase (APX) extraction, 50 mM potassium phosphate buffer (pH 7.0) containing 2 mM ascorbate and all the previously mentioned ingredients except Tris was used to extract the sample. Ascorbate was added to prevent APX inactivation during isolation (Bradford, 1976) and used to quantify the protein, and bovine serum albumin (BSA) was used as a reference. Unless stated otherwise, all biochemical assays were conducted using a Shimadzu 3600 UV-Vis spectrophotometer (Shimadzu, Japan) with three replicates for each treatment. The chemicals utilized in this experiment were procured from Sigma-Aldrich or Merck, Germany. Superoxide dismutase (SOD) (EC 1.15.1.1) activity was measured following the manufacturer’s protocol, utilizing a commercial SOD kit (19160) (Sigma-Aldrich). The absorbance was measured at 440 nm, and SOD activity was expressed as the percentage of water-soluble tetrazolium salt (WST-1) inhibition. Catalase (CAT) (EC 1.11.1.6) activity was measured following the procedure described by Aebi (1984) with modifications.

CAT activity was determined at 25°C in a reaction mixture of 50 mM potassium phosphate buffer (pH 7.0), 10 µL of extract, and 10 mM hydrogen peroxide (H_2_O_2_) in a final volume of 1 mL. The reaction was initiated by the addition of H_2_O_2_, and the decrease in absorbance at 240 nm was measured for 130 s against an extract-free blank. Enzyme activity was calculated using the molar coefficient 0.043 mM^−1^ cm^−1^, with one unit of catalase defined as the amount of enzyme that decomposes 1 µmol of H_2_O_2_ per minute per mL at 25°C, and expressed in units per mg of protein.

APX (EC 1.11.1.1) activity was measured according to Nakano and Asada (1981) by monitoring the decrease in absorbance at 290 nm over 130 s in a reaction mixture consisting of 50 mM potassium phosphate buffer (pH 7.0), 0.5 mM ascorbate, 0.1 mM EDTA, and 1.2 mM H_2_O_2_ at the final concentration, along with 10 µL of extract. The extinction coefficient of 2.8 mM^−1^ cm^−1^ was used to calculate the concentration of oxidized ascorbate. Enzyme activity was expressed in units per mg protein, with one unit of APX defined as 1 mM mL^−1^ ascorbate oxidized per minute.

Glutathione reductase (GR) (EC 1.6.4.2) activity was determined according to Edwards et al. (1990). The oxidation of NADPH dependent on GSSG (oxidized glutathione) was monitored by measuring the decrease in absorbance at 340 nm at a temperature of 25°C. A 1-mL assay mixture was used, consisting of 100 mM N−2−hydroxyethyl−piperazine−N−2−ethanesulfonic acid [HEPES] buffer (pH 7.8), 1 mM EDTA, 3 mM MgCl_2_, 0.5 mM GSSG at the final concentration, and 10 μL of extract. The reaction was initiated by adding NADPH (0.2 mM at final concentration). Corrections were made for non-enzymatic reduction of GSSG by NADPH. The activity was calculated using an extinction coefficient of 6220 M^−1^ cm^−1^ for NADPH. One unit of GR was defined as 1 μM of NADPH oxidized per minute per mL at 25°C and expressed in units per mg protein.

#### 2.5.2 Determination of total reactive oxygen species

Total reactive oxygen species (ROS) was measured using a modified 2’,7’-dichlorofluorescein diacetate (DCFDA; Sigma-Aldrich, Germany) assay (Jambunathan, 2010). To extract the leaf tissue, approximately 100 mg of leaf tissue ground with liquid nitrogen was mixed with 1 mL of 10 mM Tris-HCl buffer (pH 7.2) and then centrifuged at 12,000 × *g* for 20 min at 4°C. The resulting supernatant was diluted 1:9 with 10 mM Tris-HCl buffer (pH 7.2). Next, a stock solution of 1 mM DCFDA was added to the diluted supernatant, resulting in a final concentration of 10 µM. The assay mixture was incubated at room temperature in complete darkness for 10 min. Fluorescence was measured using a spectrofluorophotometer (Shimadzu RF-5301 PC, Kyoto, Japan) at 490 nm excitation and 525 nm emission.

#### 2.5.3 Endogenous metabolites composition using GC-MS analysis

The metabolite extractions were as performed by the method used by Vaghela et al. (2023).

To extract metabolites from maize fresh tissue, 250 mg of ground powder was mixed with pre-chilled 1.4 mL of methanol containing 0.1 mL of adinotol (0.2 mg mL^−1^) as an internal standard. The mixture was incubated for 15 min at 70°C with shaking (200 rpm) in the dark. Then, equal amounts of water and chloroform (750 µL) were added and mixed vigorously after each addition. The mixture was centrifuged at 22,000 × *g* at room temperature for 15 min. Next, 200 µL of the resulting supernatant was transferred to a fresh tube, dried under vacuum, and derivatized.

For derivatization, the vacuum-dried residues were re-dissolved in 40 µL of methoxyamine hydrochloride (20 mg mL^−1^ in pyridine) and incubated at 37°C for 2 h with shaking. After that, 60 µL of N,O-Bis(trimethylsilyl)trifluoroacetamide (BSFTA) was added and incubated for 30 min at 37°C. The derivatives were analyzed by GC-MS using a Shimadzu GC/MS-QP2010 system connected to an SH-Rxi-5 ms column (30 m, 0.25 µm df, Shimadzu, USA) with split injection mode. The injector temperature was maintained at 250°C, and helium was used as the carrier gas with a flow rate of 1 mL min^−1^. The ion source was tuned to 250°C, and the transfer line was set at 300°C with a rate of 14.5°C s^−1^. The mass spectra were recorded at a rate of eight scans per second with a scanning range of 70–700 m/z. Metabolites were identified by comparing their relative retention time and mass spectra with those of standards and NIST 2014 libraries.

#### 2.5.4 Fatty acid profiling

The lipid extraction was carried out according to Bligh and Dyer (1959). To analyze the fatty acids present in fresh maize leaves, 500 mg of the sample was used. The extraction process involved adding 2 mL of a chloroform/methanol solution (1/2, v/v) to the leaves and vortexing for 30 min. The mixture was then centrifuged at 10,000 rpm for 10 min at room temperature, and the resultant supernatant was collected. This step was repeated thrice, with 2 mL of a chloroform/methanol solution (1/1; v/v) added each time, followed by centrifugation. All of the supernatants were combined and then an equal volume of Milli-Q water was added. The mixture was then centrifuged at 5,000 rpm for 10 min, and the lower organic phases were collected. These were evaporated to dryness using nitrogen gas, and the total lipid content was determined gravimetrically.

##### 2.5.4.1 Preparation of FAMEs

The fatty acid analysis involved the conversion of the fatty acids to their fatty acid methyl esters (FAMEs) through transmethylation of the lipid samples obtained via solvent extraction. To do this, 1 mL of 1% NaOH in MeOH was added to the samples and heated for 15 min at 55°C. This was followed by the addition of 2 mL of 5% methanolic HCl and another heating step for 15 min at 55°C. Subsequently, 1 mL of Milli-Q water was added to the samples. FAME was extracted by adding hexane (4 × 1 mL) followed by centrifugation of the mixture to collect the upper layer. A 50-µL mixture of FAMEs standard was added to the samples for analysis. The samples were then evaporated to dryness under nitrogen (Carreau and Dubacq, 1978). They were redissolved in 150 µL of hexane and stored in −20°C in glass vials until analyzed by GC-MS.

##### 2.5.4.2 Lipid composition and preparation of model membrane

**Figure 1:**
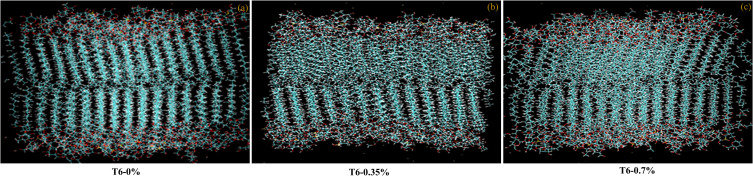
Snapshots of bilayer membranes with different concentrations of MPH treatment (T6-0:100 KA : SW): **(a)** 0% treatment (Control), **(b)** 0.35% treatment, and **(c)** 0.7% treatment.

The lipid composition of the maize leaves was experimentally derived in this study. In general, thylakoid membranes of chloroplast are the main intracellular membranes in leaves and the lipid content of the leaves as a whole is dominated by the lipid composition of the thylakoid membranes. Thylakoid membranes are characterized by their unique composition, which includes the glycolipids monogalactosyldiacylglycerol (MGDG), digalactosyldiacylglycerol (DGDG), and sulfoquinovosyl-diacylglycerol (SQDG), which are not found in extrachloroplastidic membranes. Therefore, the presence of these glycolipids in high levels nearly represents the thylakoid membranes in maize leaves. In this study, we have prepared the model thylakoid membrane composed of four unique polar lipids: MGDG, DGDG, SQDG, and phosphatidylglycerol (PG) (Rathod et al., 2023). The most abundant acyl tails found in the lipid composition of maize leaves were palmitic acid (16:0), oleic acid (18:1), and linolenic acid (18:2). The lipid compositions of the *in silico* membrane models for different applied concentrations of MPH-treated maize plant leaf membranes are presented in **Supplementary Table 2**. To perform the molecular dynamic simulations, the lipid bilayers were solvated with water, and Na^+^ counter ions were added to ensure overall charge neutrality. To achieve a salt concentration of 0.15 M, NaCl was added. The membranes were composed of different amounts of lipid mixtures, and each bilayer leaflet contained 128 lipids. The representative snapshots of the membrane can be seen in **Figure 1**, which displays the bilayer membranes with different concentrations of only SW MPH treatment T6 (KA : SW 0:100): (a) 0% treatment (Control), (b) 0.35% treatment, and (c) 0.7% treatment.

We used three solvated model bilayer membranes of maize plant in this study, representing 0%, 0.35%, and 0.7% treatments of MPHs of seaweed formulations. Each solvated bilayer was first energy minimized and then subjected to short simulations of 1 ns with harmonic position restraints on lipid glycerol carbon atoms. After releasing all restraints, the system was subjected to production simulations at room temperature (30°C). To ensure the reliability of the results, we conducted three independent simulations of the membranes at a given temperature, with each production simulation running for 1500 ns. The cumulative length of the atomistic simulation used in this study is ~4.5 μs. VMD (Humphrey et al., 1996) was used for trajectory visualizations.

###### 2.5.4.2.1 Simulation protocol

All simulations were performed with all-atom CHARMM36 force field parameters for systems and CHARMM-modified TIP3P model for water (Jorgensen et al., 1983; Klauda et al., 2010; Klauda et al., 2012). These sets of parameters had been used in many recent computer simulations of lipid membranes (Li et al., 2018; Manna and Murarka, 2021). All simulations were conducted using GROMACS version 2020.4 (Van Der Spoel et al., 2005; Abraham et al., 2015) and under an isothermal–isochoric (NPT) ensemble. A time step of 2 fs was employed for integrating the equations of motion. Linear Constraint Solver (LINCS) algorithm (Hess et al., 1997) was used to constrain the covalent bond lengths of hydrogen atoms. The Nose-Hoover thermostat with a 1.0-ps coupling constant was employed to maintain the constant temperature of the system. The system’s pressure is controlled semi-isotropically at 1 bar using a (Parrinello and Rahman, 1981) barostat with a 5.0-ps coupling constant. Periodic boundary conditions are applied in all three directions. The long-range electrostatic interactions were treated with the particle mesh Ewald (PME) (Darden et al., 1993) method with an actual space cutoff of 12 Å. The van der Waals interactions were computed using the Lennard-Jones potential and using a cutoff distance of 12 Å. The neighbor lists were updated every 20 steps. The same simulation protocol was used in another recent work on a realistic model of thylakoid membrane in algae (Rathod et al., 2023).

### 2.6 Statistical analysis

Data obtained on maize growth and yield parameters were analyzed using a two- factor completely randomized block design (F-CRBD) for analysis of variance. Parameters that met the assumptions of normality and homogeneity of variance were subjected to analysis of variance using MSTATC software (Michigan, USA). Mean comparisons were performed using Duncan’s Multiple Range Test (DMRT) at a significance level of *p*< 0.05.

## 3 Result

In the present study, identification of untargeted metabolites of the MPHs of seaweeds was carried out. Furthermore, these MPHs were assessed for their impact on maize growth, yield, and biochemical parameters.

### 3.1 LC-Q-TOF-HRMS analysis in MPHs of seaweeds

The ESI+ and ESI− ionization modes were employed for the methanolic extract analysis of MPH derived from KA and SW using the optimized MS-Q-TOF. The resulting mass spectra provided information on the number and intensity of detected peaks, which were further annotated.

#### 3.1.1 LC-Q-TOF-HRMS analysis MPH of *Kappaphycus alvarezii*

**Figure 2:**
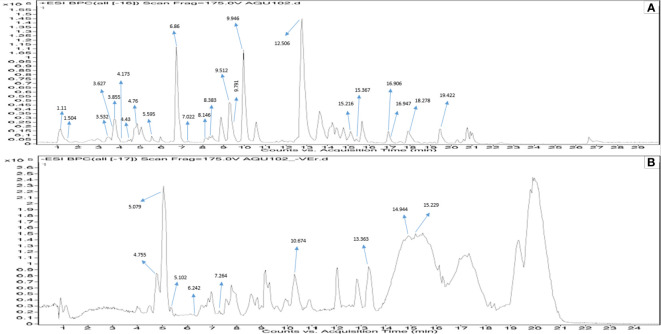
Q-TOF-HRMS profiles with major peaks in the TICs. **(A)** Positive ESI and **(B)** negative ESI of MPH of KA.

A total of 181 metabolites were detected with 80 of them in ESI-positive mode and the rest in ESI-negative mode. The compounds were annotated by comparing their measured accurate mass with those found in the database within 5 ppm mass difference, while considering spectral features, isotopic patterns, and fragmentation patterns. The acceptable mass error considered in the present study ranged between −4.64 and 4.98 ppm. After applying these cutoffs, 23 putative compounds were found (**Table 1**). Similarly, in ESI-negative ionization mode, out of a total of 101 metabolite peaks detected, 9 putative compounds were identified (**Table 2**). The results of the Q-TOF-HRMS TICs in both ionization modes are shown in **Figures 2A, B**.

**Table 1:** Putative compounds identified using ESI positive ionization mode Q-TOF-HRMS-MS/MS in methanolic extract of MPH of KA.

List of compounds	Compound name	Retention time (min)	Compounds’ mass without adduct ions	Ion attribution	Observed Mass	MFG formula	DB formula	DB Diff (ppm)	DB Hits	MS/MS fragments
1	Retronecine	1.11	155.0943	(M+H)^+^	156.1016	C_8_H_13_NO_2_	C_8_H_13_NO_2_	2.22	3	130.0605, 132.103, 144.1005, 145.1043, 155.081, 156.1013
2	Pirbuterol	1.504	240.1467	(M+H)^+^	241.1541	C_12_H_20_N_2_O_3_	C_12_H_20_N_2_O_3_	2.82	10	126.0578, 136.0629, 138.0568, 167.0812, 174.1489, 218.0945, 241.1543
3	Isocarbostyril	3.532	145.0521	(M+H)^+^	146.0595	C_9_H_7_NO	C_9_H_7_NO	4.32	10	123.0793, 127.0823, 146.0597
4	Fasoracetam	3.627	196.1209	(M+H)^+^	197.1279	C_10_H_16_N_2_O_2_	C_10_H_16_N_2_O_2_	1.34	6	145.0671, 150.1259, 168.1476, 172.094, 188.162, 196.0964, 197.1265
5	Sinapoylputrescine	3.855	294.1565	(M+H)^+^	295.1639	C_15_H_22_N_2_O_4_	C_15_H_22_N_2_O_4_	4.95	10	121.0757, 137.0609, 168.149, 190.0487, 197.1274, 224.1275, 230.1166, 276.119, 294.1322, 295.1645
6	4-Dodecylbenzenesulfonic acid	4.173	326.1907	(M+H)^+^	327.199	C_18_H_30_O_3_S	C_18_H_30_O_3_S	2.8	10	134.0463, 137.0851, 202.1257, 205.0629, 211.0564, 225.1202, 273.0511, 298.1666, 327.1988
7	Tyrosyl-Glycine	4.43	238.0944	(M+H)^+^	239.1021	C_11_H_14_N_2_O_4_	C_11_H_14_N_2_O_4_	3.9	10	121.0389, 147.0903, 184.1105, 193.0964, 197.1322, 221.0892, 222.1111, 239.1465
8	L,L-Cyclo(leucylprolyl)	4.76	210.1361	(M+H)^+^	211.1433	C_11_H_18_N_2_O_2_	C_11_H_18_N_2_O_2_	3.57	3	139.0741, 159.0443, 183.1472, 192.0622, 211.1425
9	Hexyl 2-furoate	5.595	196.1096	(M+H)^+^	197.1171	C_11_H_16_O_3_	C_11_H_16_O_3_	1.74	4	120.085, 133.1004, 153.1125, 179.1063, 197.1165
10	DG(20:5(5Z,8Z,11Z,14Z,17Z)/20:2(11Z,14Z)/0:0)	6.86	666.5245	(M+2Na)^+2^	356.2519	C_43_H_70_O_5_	C_43_H_70_O_5_	-3.33	10	132.0998, 171.1485, 172.1521, 239.2117, 354.207, 355.2033, 355.2212, 356.1931, 356.2539
11	Sulfoglycolithocholate	7.022	513.2751	(M+H)^+^	514.2826	C_26_H_43_NO_7_S	C_26_H_43_NO_7_S	1.74	4	136.062, 171.1479, 178.1198, 183.0753, 207.1414, 227.1742, 261.1531, 264.2049, 315.2631, 514.2826
12	1-Phosphatidyl-1D-myo- inositol 3-phosphate	8.146	470.0227	(M+Na)^+^	493.0134	C_11_H_20_O_16_P_2_	C_11_H_20_O_16_P_2_	-0.19	2	120.0804, 148.0254, 199.0334, 203.1434, 224.1422, 228.1562, 252.2416, 283.0868, 356.2081, 493.0151
13	2,3-Dihydro-6-methyl-5- propanoyl-1H-pyrrolizine	8.383	177.115	(M+H)^+^	178.1224	C_11_H_15_NO	C_11_H_15_NO	1.84	10	128.0604, 133.0648, 134.0669, 177.1255, 178.1227
14	Protorifamycin I	9.512	639.3066	(M+H)^+^	640.3136	C_35_H_45_NO_10_	C_35_H_45_NO_10_	-3.56	2	120.0802, 123.0797, 253.2138, 278.1945, 285.1762, 286.083, 308.2098, 309.2133, 351.2126, 579.3476, 640.3136
15	Candoxatrilat	9.791	399.2269	(M+Na)^+^	422.216	C_20_H_33_NO_7_	C_20_H_33_NO_7_	-3.02	1	122.0925, 196.1377, 222.1088, 224.1281, 255.2092, 288.2895, 318.8813, 341.2048, 422.216
16	Anapheline	9.946	224.1881	(M+H)^+^	225.1954	C_13_H_24_N_2_O	C_13_H_24_N_2_O	3.4	2	121.0644,123.1151,137.0954, 142.0786, 203.1439, 223.1293, 224.1276, 225.1952
17	12-(2,3-Dihydroxycyclopentyl)- 2-dodecanone	12.506	284.2361	(M+Na)^+^	307.2252	C_17_H_32_O_3_	C_17_H_32_O_3_	-3.27	10	128.0634, 169.0484, 224.1297, 235.2063, 278.2478, 279.2302, 306.2085, 306.2425, 307.2249
18	Idoxuridine	15.216	353.9727	(M+H)^+^	354.9818	C_9_H_11_IN_2_O_5_	C_9_H_11_IN_2_O_5_	-4.03	1	145.9478, 172.9793, 173.9784, 175.9927, 206.9677, 207.9654, 231.0359, 291.0372, 354.9845
19	Chlorfenvinphos	15.367	357.9706	(M+H)^+^	358.9778	C_12_H_14_C_l3_O_4_P	C_12_H_14_C_l3_O_4_P	-2.95	1	123.0797, 149.0227, 165.052, 203.1088, 260.1623, 295.1679, 339.3058, 354.9827, 358.9768
20	1,1’-Bis(2-hydroxy-3- methylcarbazole)	16.906	392.1524	(M+Na)^+^	415.1413	C_26_H_20_N_2_O_2_	C_26_H_20_N_2_O_2_	0.23	10	124.086, 175.1105, 179.1241, 207.0639, 382.3066, 383.3119, 411.2642, 415.1412
21	5-Decanoyl-2-nonylpyridine	16.947	359.3189	(M+Na)^+^	382.3081	C_24_H_41_NO	C_24_H_41_NO	-0.25	2	160.1202, 223.1473, 256.2643, 300.2303, 301.2346, 327.2687, 352.3174, 382.3078
22	2,4,14-Eicosatrienoic acid isobutylamide	18.278	361.3342	(M+Na)^+^	384.3233	C_24_H_43_NO	C_24_H_43_NO	0.65	2	122.0963, 149.0231, 166.0855, 284.295, 309.2744, 339.3183, 368.4223, 370.3263, 384.3207
23	Octadecyl fumarate	19.422	368.2927	(M+Na)^+^	391.282	C_22_H_40_O_4_	C_22_H_40_O_4_	-0.18	10	124.0869, 149.0229, 150.0254, 167.0343, 295.1738, 338.3414, 391.2818

NB: The results include the empirical formula for which the number of unique METLIN Personal metabolite database matches (< 5 ppm tolerance)

**Table 2:** Putative compounds identified using ESI negative ionization mode in methanolic extract of MPH of KA.

List of compounds	Compound name	Retention time (min)	Compounds’ mass without adduct ions	Ion attribution	Observed Mass	MFG formula	DB formula	DB Diff (ppm)	DB Hits	MS/MS fragments
1	4,4’-(2-Methylpropylidene)bisphenol	4.051	242.1318	(M-H)^-^	241.1246	C_16_H_18_O_2_	C_16_H_18_O_2_	-4.64	8	160.847, 162.8428, 164.8377, 195.8157, 197.8128, 199.8086, 215.1441, 227.1439, 241.1234
2	Simetryn	5.079	213.1052	(M-H)^-^	212.0977	C_8_H_15_N_5_ S	C_8_H_15_N_5_ S	-2.03	4	130.0923, 195.8154, 197.1329, 197.8115, 212.0977
3	guaiazulene	5.102	198.1412	(M-H)^-^	197.1337	C_15_H_18_	C_15_H_18_	-1.67	1	116.0527, 127.056, 130.089, 160.8457, 162.8431, 197.1339
4	Sethoxydim	6.242	327.1867	(M+CH_3_COO)^-^	386.2013	C_17_H_29_N O_3_S	C_17_H_29_N O_3_S	0.26	8	146.0487, 160.8444, 162.8435, 164.839, 195.8154, 197.8114, 199.8092, 214.1164, 257.1557, 277.1631, 386.2013
5	2-Deoxystreptidine	7.264	246.1434	(M+HCOO)^-^	291.1413	C_8_H_18_N_6_ O_3_	C_8_H_18_N_6_ O_3_	2.73	4	115.09, 180.0655, 180.0844, 195.815, 197.8131, 285.1916
6	Tragopogonsaponin B	10.674	926.4647	(M-H)^-^	925.4572	C_50_H_70_O_16_	C_50_H_70_O_16_	1.84	2	160.8473, 164.8395, 179.0925, 195.8145, 197.8115, 311.1761, 311.1927, 327.224, 405.2208, 630.2531, 925.4578
7	Carteolol	13.363	292.1781	(M+CH_3_COO)^-^	351.1922	C_16_H_24_N_2_O_3_	C_16_H_24_N_2_O_3_	1.88	6	160.8455, 162.8423, 197.8135, 209.1223, 271.2331, 311.1737, 325.1919, 351.1912
8	Hydroquinidine	14.944	326.1984	(M-H)^-^	325.1915	C_20_H_26_N_2_O_2_	C_20_H_26_N_2_O_2_	3.22	9	160.8455, 162.8412, 183.0161, 184.0197, 195.815, 209.1224, 325.191
9	Cucurbitacin E	15.229	556.3016	(M-H)^-^	555.2949	C_32_H_44_O_8_	C_32_H_44_O_8_	3.6	6	125.0272, 160.8464, 162.8426, 164.9899, 225.0126, 255.2377, 325.1909, 326.1947, 342.2719, 555.2937

NB: The results include the empirical formula for which the number of unique METLIN Personal metabolite database matches (< 5 ppm tolerance)

The detailed data on the observed mass, database mass, molecular formula, compound’s exact mass, database difference, database hits, and MS/MS fragments are summarized in **Tables 1** , **2**.

A total of 27 molecular families were detected in the methanolic extract of MPH of KA. They covered a wide range and were composed of alkaloid, pyridine and derivative, carboxylic acid and derivative, phenolamides, benzene and substituted derivative, dipeptide, amino acid and derivative, furan, phospholipid, bile salt, polyols, pyrrolizine, phenylpropanoid and polyketide, piperidine alkaloid, oraganooxygen compound, pyrimidine nucleoside, indoles and derivative, fatty acyl, fatty acid ester, diarylmethane, methylthiotriazine, sesquiterpene, organic hydroxyl compound, amino cyclitol, triterpene saponin, quinoline, and steroid and derivative. These are elaborated in the ensuing sections.

##### 3.1.1.1 Classes (family) of compounds in MPH of *Kappaphycus alvarezii*

**Figure 3:**
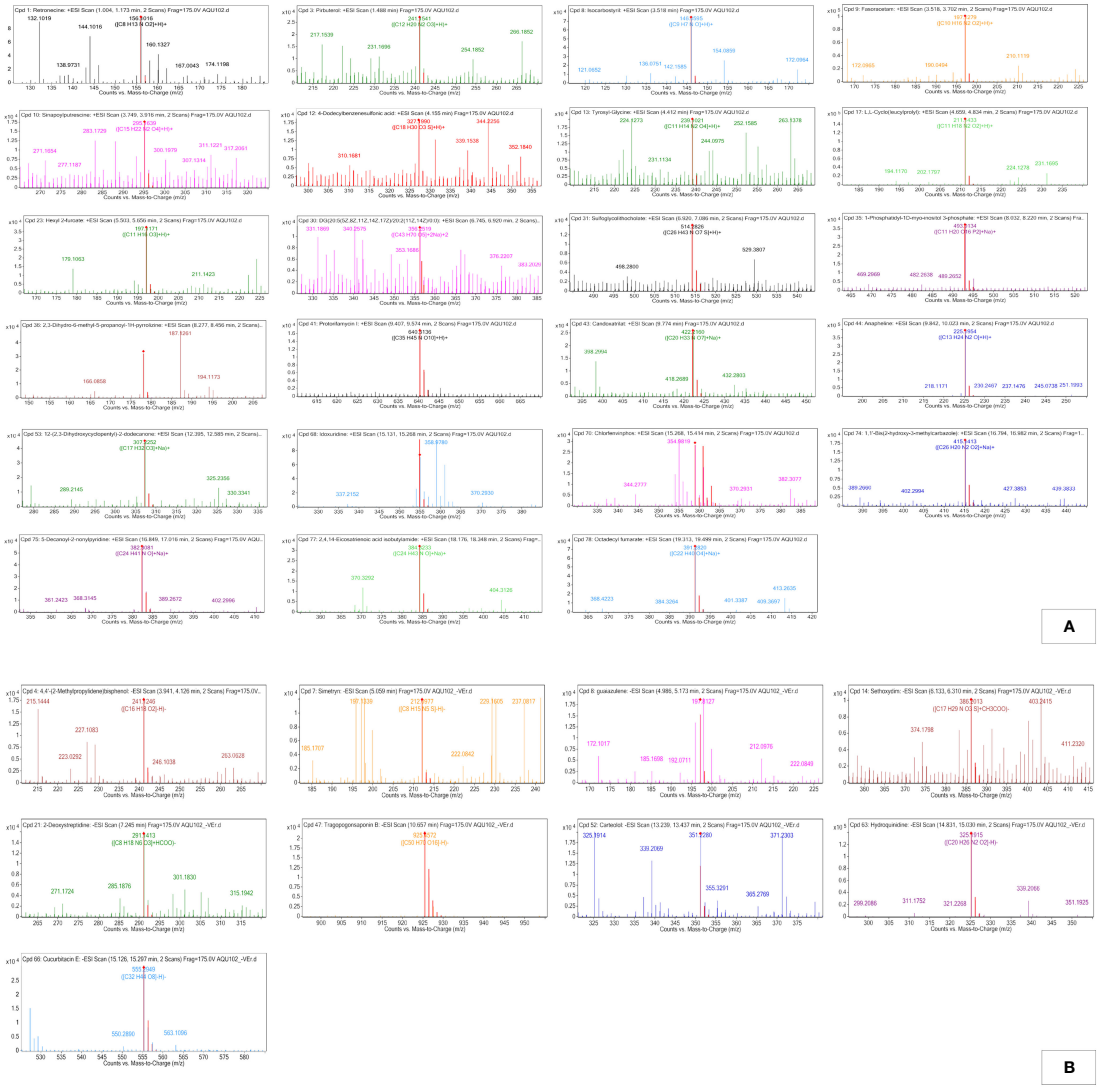
**(A)** Positive electro-spray ionization MFE-MS spectrum of all screened annotated compounds in MPH of KA. **(B)** Negative electro-spray ionization MFE-MS spectrum of all screened annotated compounds in MPH of KA.

The peaks were annotated and classified into different classes of compounds. The observed m/z values have been described in **Figures 3A, B**, while the known biological role of the compounds has been described in **Supplementary Table 3**. Furthermore, MS/MS data obtained for the annotated compounds have been depicted in **Tables 1**, **2**. The MS/MS spectra have been given in **Supplementary Figures 1**, **2**.

###### 3.1.1.1.1 Alkaloid, piperidine alkaloid

Three compounds detected in MPH of KA belonged to the class alkaloid. Retronecine and isocarbostyril eluted at a retention time of 1.11 and 3.532 min in ESI-positive ionization mode, respectively, at m/z 156.1016 (peak 1) as [M+H]^+^ with MS/MS fragmentation: 156 → 155 → 145 → 144 → 132 → 130 and m/z 146.0595 (peak 3) as [M+H]^+^ with MS/MS fragmentation: 146 → 127 → 123. Another alkaloid compound in ESI-negative ionization, hydroquinidine (peak 8), was eluted at 14.944 min at m/z 325.1915 as [M-H]^−^ with MS/MS fragmentation: 325 → 209 → 196 → 184 → 183 → 163 → 161.

In ESI+, peak 16 is characterized as piperidine alkaloid at m/z 225.1954 as [M+H]^+^ with MS/MS fragmentation: 225 → 224 → 223 → 203 → 142 → 137 → 123 → 121, which is annotated as anapheline (C_13_H_24_N_2_O).

###### 3.1.1.1.2 Pyridine and derivative, pyrimidine nucleoside

One pyridine and derivative detected in MPH of KA (peak 2) at m/z 241.1541 as [M+H]^+^ with MS/MS fragmentation: 241 → 218→ 174 → 167 → 138 → 136→ 126 is annotated as pirbuterol.

One pyrimidine nucleoside class of compound that was detected in MPH of KA (peak 18) at m/z 354.9818 as [M+H]^+^ with MS/MS fragmentation: 355 → 291→ 231 → 208 → 207 → 176→ 174 → 173 → 146 is annotated as idoxuridine (C_9_H_11_IN_2_O_5_).

###### 3.1.1.1.3 Carboxylic acid and derivative, amino acid and derivative

Two compounds belonging to carboxylic acid and derivative were characterized between 3.627 and 9.791 min in the sample analyzed. Thus, peak 4 with m/z 197.1279 as [M+H]^+^ with MS/MS fragmentation: 197 → 196→ 188 → 172 → 168 → 150→ 145 is annotated as fasoracetam and peak 15 at m/z 422.216 as [M+Na]^+^ with MS/MS fragmentation: 422 → 341 → 319 → 288 → 255 → 224→ 222 → 196 → 122 is annotated as candoxatrilat (C_20_H_33_NO_7_).

One amino acid and derivative class of compound was detected in MPH of KA (peak 8) at m/z 211.1433 as [M+H]^+^ with MS/MS fragmentation: 211 → 192 → 183 → 159 → 139, which is annotated as L,L-Cyclo(leucylprolyl).

###### 3.1.1.1.4 Phenol-amide, phenylpropanoid and polyketide, amino cyclitol, dipeptide

Peak 5 is characterized as phenol-amide at m/z 295.1639 as [M+H]^+^ with MS/MS fragmentation: 295 → 294 → 276 → 230 → 224→ 197 → 190 → 168 → 137→ 121, which is annotated as sinapoylputrescine.

One phenylpropanoid and polyketide was detected in MPH of KA (peak 14) at m/z 640.3136 as [M+H]^+^ with MS/MS fragmentation: 640 → 579 → 351 → 309 → 308→ 286 → 285 → 278 → 253 → 123 → 120, which is annotated as protorifamycin I (C_35_H_45_NO_10_).

One amino cyclitol was detected in MPH of KA (peak 5) at m/z 291.1413 as [M+HCOO]^−^ with MS/MS fragmentation: 285 → 198 → 196 → 180 → 115, annotated as 2-Deoxystreptidine.

One dipeptide class of compound was also detected in MPH of KA (peak 7) at m/z 239.1021 as [M+H]^+^ with MS/MS fragmentation: 239 → 222 → 221 → 197 → 193 → 184 → 147 → 121, which is annotated as tyrosyl-glycine (C_11_H_14_N_2_O_4_).

###### 3.1.1.1.5 Benzene-substituted derivative, furan, bile salt

Benzene-substituted derivative peaks 6 and 19 were detected in MPH of KA. Under this class, compounds such as 4-Dodecylbenzenesulfonic acid (m/z 327.199) as [M+H]^+^ with MS/MS fragmentation: 327 → 298 → 273 → 225 → 211 → 205 → 202 → 137 → 134 and Chlorfenvinphos (m/z 358.9778) as [M+H]^+^ with MS/MS fragmentation: 359 → 355 → 339 → 295 → 260 → 203 → 165 → 149 → 123 were detected.

One furan class of compound was detected in MPH of KA (peak 9) at m/z 197.1171 as [M+H]^+^ with MS/MS fragmentation: 197 → 179 → 153 → 133 → 120, annotated as hexyl 2-furoate (C_11_H_16_O_3_).

One bile salt class of compound was also detected in MPH of KA (peak 11) at m/z 514.2826 as [M+H]^+^ with MS/MS fragmentation: 514 → 315 → 264 → 261 → 227 → 207 → 183 → 178 → 171 → 136, annotated as sulfoglycolithocholate (C_26_H_43_NO_7_S).

###### 3.1.1.1.6 Phospholipid, fatty acyl, and fatty acid ester

One phospholipid was detected in MPH of KA (peak 10) at m/z 356.2519 as [M+2Na]^2+^ with MS/MS fragmentation: 356 → 355 → 354 → 239 → 172 → 171 → 132, annotated as DG(20:5(5Z,8Z,11Z,14Z,17Z)/20:2(11Z,14Z)/0:0) (C_43_H_70_O_5_).

Peak 22 is characterized as fatty acyl at m/z 384.3233 as [M+Na]^+^ with MS/MS fragmentation: 384 → 370 → 368 → 339 → 309 → 284 → 166 → 149 → 122, annotated as 2,4,14-eicosatrienoic acid isobutyl amide.

One fatty acid ester class of compound was also detected in MPH of KA (peak 23) at m/z 391.282 as [M+Na]^+^ with MS/MS fragmentation: 391 → 338 → 295 → 167 → 150 → 149 → 124 and was annotated as octadecyl fumarate (C_22_H_40_O_4_).

###### 3.1.1.1.7 Alcohol and polyol, pyrrolizine, quinolone

Peak 12 annotated belonged to alcohol and polyol class and peak 13 to pyrrolizine class of compound in ESI+. Peak 12 that gave [M+Na]^+^ ion at m/z 493.0134 with MS/MS fragmentation: 493 → 356 → 283 → 252 → 228 → 224 → 203 → 199 → 148 → 120 is annotated as 1-phosphatidyl-1D-myo-inositol 3-phosphate and peak 13 that gave [M+H]^+^ at m/z 178.1224 with MS/MS fragmentation: 178 → 177 → 134 → 133 → 128 is annotated as 2,3-dihydro-6-methyl-5-propanoyl-1H-pyrrolizine. In ESI−, one quinolone class of compound was detected in MPH of KA (peak 7) at m/z 351.1922 as [M+CH_3_COO]^−^ with MS/MS fragmentation: 351 → 325 → 311 → 271 → 209 → 198 → 163 → 161, which was annotated as carteolol (C_16_H_24_N_2_O_3_).

###### 3.1.1.1.8 Organooxygen compound, organic hydroxy compound

Two organooxygen compounds were detected in MPH of KA. 12-(2,3-Dihydroxycyclopentyl)-2-dodecanone and 5-Decanoyl-2-nonylpyridine eluted at a retention time of 12.506 and 16.947 min, respectively, at m/z 307.2252 (peak 17) as [M+Na]^+^ with MS/MS fragmentation: 307 → 306 → 279 → 278 → 235 → 224 → 169 → 128 and m/z 382.3081 (peak 21) as [M+Na]^+^ with MS/MS fragmentation: 382 → 352 → 327 → 301 → 300 → 256 → 223 → 160. One organic hydroxy compound detected in ESI-negative mode in MPH of KA (peak 4) at m/z 386.2013 as [M+CH_3_COO]^−^ with MS/MS fragmentation: 386 → 277 → 257 → 214 → 200 → 198 → 196 → 165 → 163 → 161 → 146 is annotated as sethoxydim.

###### 3.1.1.1.9 Indole and derivative, steroid and steroid derivative

Peak 20 is characterized as indole and derivative at m/z 415.1413 as [M+Na]^+^ with MS/MS fragmentation: 415 → 411 → 383 → 382 → 207 → 179 → 175 → 124 and annotated as 1,1’-bis(2-hydroxy-3-methylcarbazole).

One steroid and steroid derivative class of compound was also detected in MPH of KA (peak 9) at m/z 555.2949 as [M-H]^−^ with MS/MS fragmentation: 555 → 342 → 326 → 325 → 255 → 225 → 165 → 163 → 161 → 125 and annotated as Cucurbitacin E (C_32_H_44_O_8_).

###### 3.1.1.1.10 Diarylmethane, methylthiotriazine

One diarylmethane class of compound was detected in MPH of KA (peak 1) at m/z 241.1246 as [M-H]^−^ with MS/MS fragmentation: 241 → 227 → 215 → 200 → 198 → 196 → 165 → 163 → 161, annotated as 4,4’-(2-methylpropylidene) bisphenol (C_16_H_18_O_2_).

One methylthiotriazine class of compound was also detected in MPH of KA (peak 2) at m/z 212.0977 as [M-H]^−^ with MS/MS fragmentation: 212 → 198 → 197 → 196 → 130, annotated as simetryn.

###### 3.1.1.1.11 Sequiterpene, triterpene saponin

Sequiterpene was detected in MPH of KA (peak 3) at m/z 197.1337 as [M-H]^−^ with MS/MS fragmentation: 197 → 163 → 161 → 130 → 127 → 116, annotated as guaiazulene.

Peak 6 was characterized as triterpene saponin (m/z 925.4572) as [M-H]^−^ with MS/MS fragmentation: 925 → 630 → 405 → 327 → 311 → 198 → 196 → 179 → 165 → 161 and attributed to be tragopogonsaponin B.

#### 3.1.2 LC-Q-TOF-HRMS analysis MPH of *Sargassum wightii*

**Figure 4:**
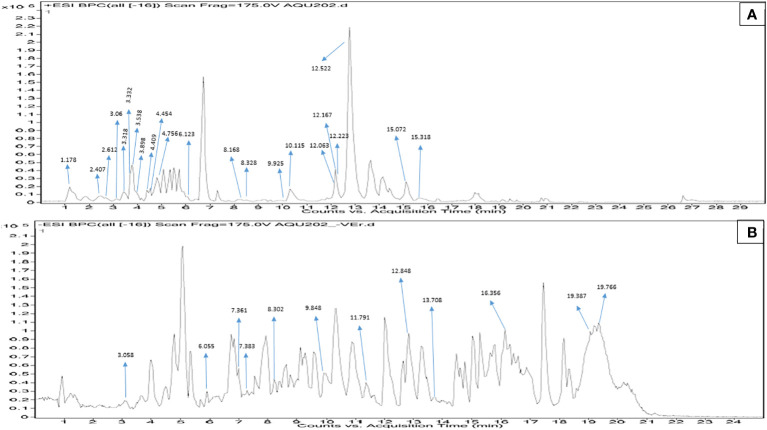
**(A)** Positive electro-spray ionization MFE-MS spectrum of all screened annotated compounds in MPH of SW. **(B)** Negative electro-spray ionization MFE-MS spectrum of all screened annotated compounds in MPH of SW.

In MPH of SW, out of a total of 181 metabolite peaks, 80 were detected in ESI-positive mode and the remaining peaks were detected in ESI-negative mode. Applying the cutoffs on the annotated compounds, 22 and 12 metabolites were putatively identified in ESI-positive and -negative mode, respectively (**Tables 3**, **4**). The acceptable shifts considered in the present study ranged between −3.79 and 4.98. The results of the Q-TOF-HRMS TICs in both ionization modes are shown in **Figures 4A, B**.

**Table 3:** Putative compounds identified using ESI positive ionization mode Q-TOF-HRMS-MS/MS in methanolic extract of MPH of SW.

List of compounds	Compound name	RT (min)	Compound mass	Adduct ions	Compound + adduct	Formula	DB Diff (ppm)	DB Hits	MS/MS fragments
1	Retronecine	1.178	155.0945	(M+H)^+^	156.1018	C_8_H_13_NO_2_	0.87	3	144.1009, 146.1168, 156.1007
2	Metanephrine	2.407	197.1048	(M+Na)^+^	220.0965	C_10_H_15_NO_3_	2.02	10	124.0767, 188.0695, 98.0773, 204.0877, 205.0733, 208.1322, 219.1121, 220.0976
3	d-Dethiobiotin	2.612	214.1311	(M+H)^+^	215.1383	C_10_H_18_N_2_O_3_	2.86	10	130.0874, 142.0937, 153.1257, 170.0827, 177.0708, 198.0756, 208.132, 209.1257, 215.1383
4	Trihomomethionine	3.06	191.0971	(M+Na)^+^	214.0855	C_8_H_17_NO_2_S	4.9	9	120.081, 121.0642, 160.0968, 170.082, 181.0942, 198.0732, 214.0867
5	Butyl 2-aminobenzoate	3.318	193.1093	(M+H)^+^	194.117	C_11_H_15_NO_2_	4.98	10	130.1605, 135.0447, 146.0597, 150.9845, 194.1183
6	Salicylanilide	3.332	213.0789	(M+H)^+^	214.0856	C_13_H_11_NO_2_	0.6	9	132.1004, 145.075, 146.0583, 168.1009, 186.0745, 196.0964, 209.1274, 214.0832
7	Isocarbostyril	3.538	145.0523	(M+H)^+^	146.0596	C_9_H_7_NO	3.4	10	146.0597
8	Fasoracetam	3.898	196.1208	(M+H)^+^	197.128	C_10_H_16_N_2_O_2_	1.87	6	124.1116, 152.1079, 169.1334, 197.1263
9	2,4’-Diphenyldiamine	4.409	184.0997	(M+H)^+^	185.1067	C_12_H_12_N_2_	1.72	8	132.1014, 142.0845, 184.1043, 185.1062
10	Pirbuterol	4.454	240.1482	(M+Na)^+^	263.1376	C_12_H_20_N_2_O_3_	-3.21	10	120.0803, 136.0741, 175.0642, 184.0748, 216.1025, 263.1361
11	L,L-Cyclo(leucylprolyl	4.756	210.1362	(M+H)^+^	211.1433	C_11_H_18_N_2_O_2_	3.21	3	129.0704, 154.0715, 183.1492, 211.1455
12	Mycinamicin IV	6.123	695.4262	(M+H)^+^	696.4335	C_37_H_61_NO_11_	-2.51	1	120.0808, 133.085, 142.1572, 166.0835, 202.1219, 213.16, 291.1335, 303.19, 598.3021, 696.4322
13	1-Phosphatidyl-1D-myo-inositol 3-phosphate	8.168	470.023	(M+Na)^+^	493.014	C_11_H_20_O_16_P_2_	-0.7	2	120.0821, 121.0666, 185.0783, 207.1726, 277.2138, 280.1048, 304.1553, 436.3209, 456.321, 493.0136
14	Istamycin A1	8.328	417.2603	(M+H)^+^	418.2672	C_18_H_35_N_5_O_6_	-3.79	2	254.1544, 267.1574, 318.1584, 325.1769, 357.2334, 368.21, 397.2376, 400.2686, 417.2164, 418.2672
15	Fleroxacin	9.925	369.13	(M+H)^+^	370.1368	C_17_H_18_F_3_N_3_O_3_	0.02	2	211.0488, 272.1294, 274.2694, 280.2056, 301.2123, 333.2034, 370.1368
16	Dibenzo[a,e]pyrene	10.115	302.1087	(M+Na)^+^	325.0987	C_24_H_14_	2.69	10	152.0694, 185.1311, 191.0692, 205.0878, 224.1275, 231.1375, 279.0931, 325.0987
17	16-Oxo-palmitate	12.063	270.22	(M+Na)^+^	293.2092	C_16_H_30_O_3_	-1.7	10	125.0601, 137.06, 162.075, 165.1239, 224.1302, 275.1977, 279.232, 292.205, 293.2092
18	3b,17a,21-Trihydroxypregnenone	12.167	348.2299	(M+Na)^+^	371.2197	C_21_H_32_O_4_	0.32	10	123.0796, 143.0868, 161.1301, 177.0935, 187.1459, 327.0468, 335.2173, 370.3066, 371.219
19	6-Oxabicyclo[3.1.0]hexane-2-undecanoic acid methyl ester	12.223	282.2202	(M+Na)^+^	305.2092	C_17_H_30_O_3_	-2.41	10	137.059, 149.1309, 222.1102, 239.1637, 288.2492, 305.2086
20	12-(2,3-Dihydroxycyclopentyl)-2-dodecanone	12.522	284.2362	(M+Na)^+^	307.2253	C_17_H_32_O_3_	-3.56	10	139.0737, 145.1043, 158.0976, 279.2282, 305.2113, 307.2235
21	Chlorfenvinphos	15.072	357.9707	(M+H)^+^	358.978	C_12_H_14_Cl_3_O_4_P	-3.3	1	137.0951, 149.0228, 228.2299, 242.2838, 283.2606, 293.1724, 354.9805, 355.9822, 358.975
22	Idoxuridine	15.318	353.9704	(M+H)^+^	354.9818	C_9_H_11_IN_2_O_5_	2.36	1	149.0241, 172.9799, 179.069, 261.9973, 281.2429, 291.0361, 354.9793

NB: The results include the empirical formula for which the number of unique METLIN Personal metabolite database matches (< 5 ppm tolerance)

**Table 4:** Putative compounds identified using ESI negative ionization mode Q-TOF-HRMS-MS/MS in methanolic extract of MPH of SW.

List of compounds	Compound name	RT (min)	Compound mass	Adduct ions	Compound + adduct	Formula	DB Diff (ppm)	DB Hits	MS/MS fragments
1	(S)-Menthone 8-thioacetate	3.058	228.1178	(M+HCOO)^-^	273.1155	C_12_H_20_O_2_S	2.81	10	124.9958, 126.9098, 128.04, 157.1376, 175.0636, 185.0886, 195.8152, 201.1279, 208.0692, 227.1092, 273.1155
2	Sethoxydim	6.055	327.186	(M+HCOO)^-^	372.1861	C_17_H_29_NO_3_S	2.37	10	195.8161, 228.1777, 229.1102, 238.1607, 243.1769, 257.1555, 277.1614, 278.1665, 328.1941, 329.1788, 372.1861
3	2-Deoxystreptidine	7.361	246.1441	(M+HCOO)^-^	291.1422	C_8_H_18_N_6_O_3_	-0.17	4	115.0899, 124.9959, 219.1579, 235.0698, 291.142
4	Oxybutynin	7.383	357.2312	(M-H)^-^	356.2276	C_22_H_31_NO_3_	-2.28	4	116.0746, 185.1695, 279.1384, 284.24, 331.1888, 342.2456, 356.2255
5	(1R,2S,4R,5S)-2,5-Fenchanediol 2-O-b-D-glucoside	8.302	332.1826	(M+HCOO)^-^	377.1802	C_16_H_28_O_7_	2.72	10	130.0929, 131.0925, 160.8468, 197.8146, 305.1558, 370.2405, 377.1802
6	Ethamoxytriphetol	9.848	419.2462	(M-H)^-^	418.2439	C_27_H_33_NO_3_	-0.31	6	130.0898, 140.0758, 160.8452, 164.0741, 227.1705, 242.1929, 353.2402, 368.1342, 369.2371, 417.1976, 418.2439
7	Aspidospermine	11.791	354.2312	(M-H)^-^	353.2239	C_22_H_30_N_2_O_2_	-1.26	5	118.0682, 160.8462, 162.8436, 172.9641, 195.8149, 235.1933, 293.1802, 294.1878, 304.1644, 309.2117, 337.2466, 351.2253, 353.2239
8	Pithecolobine	12.848	382.3659	(M+CH_3_COO)^-^	441.3796	C_22_H_46_N_4_O	3.39	10	159.1181, 160.1204, 160.8464, 201.1264, 269.2197, 341.2404, 342.245, 441.3814
9	Bryodulcosigenin	13.708	474.3692	(M-H)^-^	473.3621	C_30_H_50_O_4_	3.7	10	159.1193, 160.8446, 164.8411, 197.8136, 311.2293, 334.3187, 339.2193, 379.378, 415.2819, 423.3664, 473.3621
10	10-Deoxymethymycin	16.356	453.3102	(M+CH_3_COO)^-^	512.3261	C_25_H_43_NO_6_	-2.54	2	160.847, 162.8447, 195.8154, 197.8132, 206.0878, 271.2355, 272.2394, 281.2581, 379.2231, 476.3446, 512.3261
11	Atenolol	19.387	2165.1548	(M-H)-	265.1558	C_14_H_22_N_2_O_3_	4.85	9	116.9327, 134.8985, 135.8991, 136.8957, 197.8135, 235.1545, 266.1555,
12	Practolol	19.766	266.1619	(M-H)-	265.1549	C_14_H_22_N_2_O_3_	4.28	9	112.9868, 116.932, 132.9039, 134.8983, 135.8969, 136.8954, 153.8721, 265.1542

NB: The results include the empirical formula for which the number of unique METLIN Personal metabolite database matches (< 5 ppm tolerance).

The detailed data on the observed mass, database mass, molecular formula, compound’s exact mass, database difference, database hits, error tolerance, and MS/MS fragments are summarized in **Tables 3**, **4**.

A total of 23 classes of compounds were detected in the methanolic extract of MPH of SW. They covered a wide range and were composed of alkaloid, phenol, fatty acyl, carboxylic acid and derivative, benzene and substituted derivative, isoquinoline and derivative, biphenyl and derivative, pyridine and derivative, macrolide, alcohol and polyol, amino glycoside, polycyclic aromatic hydrocarbon, fatty acid and conjugate, steroid and steroid derivative, fatty acid, organooxygen compound, pyrimidine nucleoside, prenol lipid, cyclohexanone, amino cyclitol, terpene glycoside, tri-terpenoid, and glycoside. These are elaborated in the ensuing sections.

##### 3.1.2.1 Class (family) of compounds in MPH of *Sargassum wightii*

**Figure 5:**
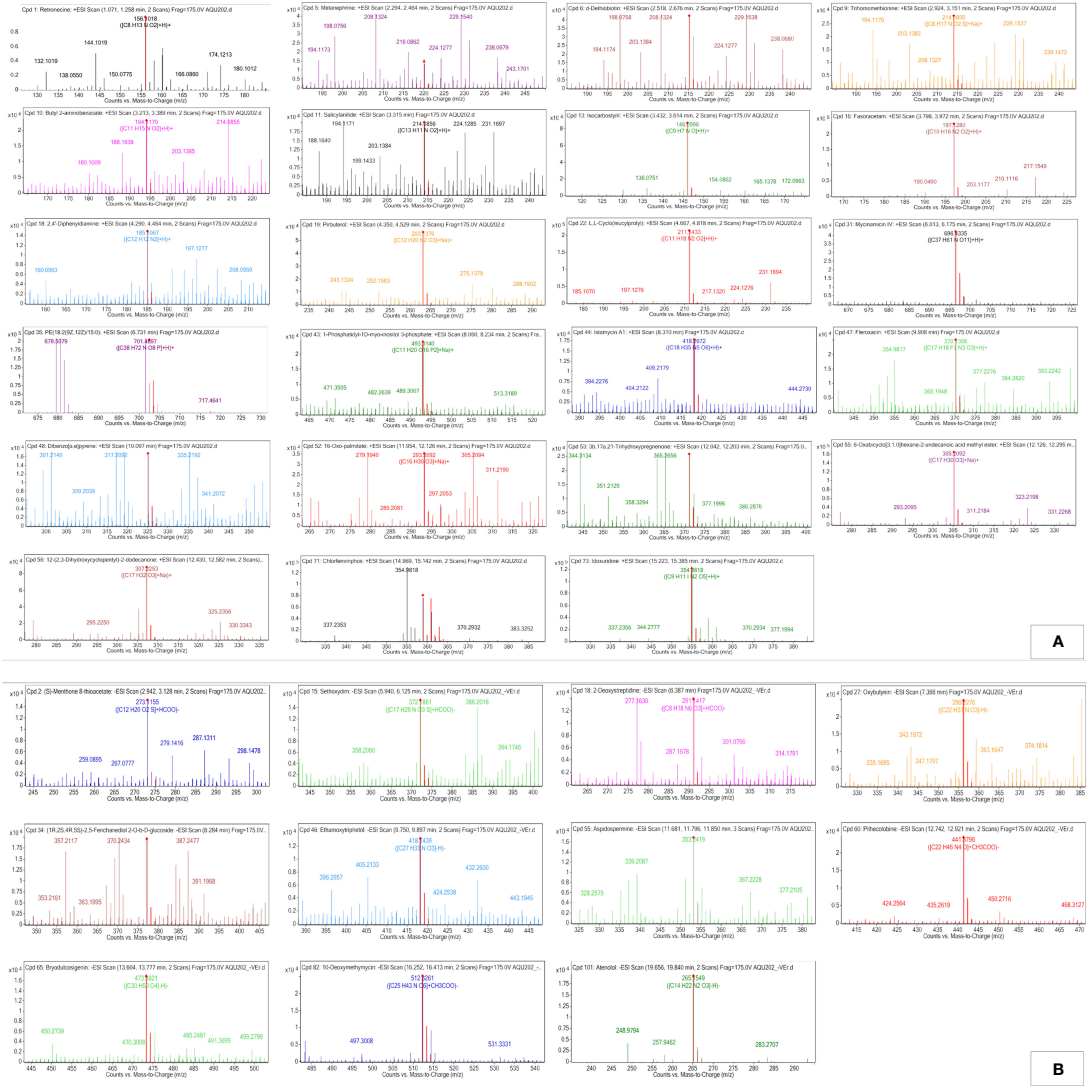
Q-TOF-HRMS profiles with major peaks in the TICs. **(A)** Positive ESI and **(B)** negative ESI of MPH of SW.

The peaks were annotated and classified into different classes of compounds. Both the ESI modes derived m/z values are presented in **Figures 5A, B**, while the known biological roles of the annotated compounds are described accordingly in **Supplementary Table 3**. Further MS/MS data obtained for the annotated compounds are depicted in **Tables 3**, **4**. The MS/MS spectra are given in **Supplementary Figures 3**, **4**.

###### 3.1.2.1.1 Alkaloid, steroid and steroid derivative, glycoside

Three alkaloid classes of compounds were detected in MPH of SW: Retronecine was annotated in ESI+ mode while Aspidospermine and Pithecolobine were annotated in ESI− mode eluted at a retention time of 1.178, 11.791, and 12.848 min. respectively. Peak 1 with m/z 156.1018 as [M+H]^+^ with MS/MS fragmentation: 156 → 146 → 144 is annotated as retronecine. Peak 7 that gave [M-H]^−^ ion at m/z 353.2239 with MS/MS fragmentation: 353 → 351 → 337 → 309 → 304 → 294 → 293 → 235 → 196 → 173 → 163 → 161 → 118 is annotated as aspidospermine and peak 8 that gave [M+CH_3_COO]^−^ at m/z 441.3796 with MS/MS fragmentation: 441 → 342 → 341 → 269 → 201 → 161 → 160 → 159 is annotated as pithecolobine.

One steroid and its derivative was detected in MPH of SW (peak 18) at m/z 371.2197 as [M+Na]^+^ with MS/MS fragmentation: 371 → 370 → 335 → 327 → 187 → 177 → 161 → 143 → 123, and annotated as 3b,17a,21-trihydroxypregnenone (C_21_H_32_O_4_).

One glycoside was also detected in MPH of SW (peak 10) at m/z 512.3261 as [M+CH_3_COO]^−^ with MS/MS fragmentation: 512 → 476 → 379 → 281 → 272 → 271 → 206 → 198 → 196 → 163 → 161, and was annotated as 10-Deoxymethymycin (C_25_H_43_NO_6_).

###### 3.1.2.1.2 Phenol, biphenyl, and derivative

Two compounds belonging to a class of phenol were detected in MPH of SW (peak 2) at m/z 220.0965 as [M+Na]^+^ with MS/MS fragmentation: 220 → 219 → 208 → 205 → 204 → 198 → 188 → 124, which is annotated as metanephrine, and peak 6 in ESI-negative mode at m/z 418.2439 as [M-H]^−^ with MS/MS fragmentation: 418 → 417 → 369 → 368 → 353 → 242 → 227 → 164 → 161 → 140 → 130 is annotated as ethamoxytriphetol (C_27_H_33_NO_3_).

One biphenyl and derivative class of compound was also detected in MPH of SW (peak 9) at m/z 185.1067 [M+H]^+^ with MS/MS fragmentation: 185 → 184 → 142 → 132, which is annotated as 2,4’-diphenyldiamine.

###### 3.1.2.1.3 Fatty acyl, fatty acid and conjugate, fatty acid

Peak 3 is characterized as fatty acyl at m/z 215.1383 as [M+H]^+^ with MS/MS fragmentation: 215 → 209 → 208 → 198 → 177 → 170 → 153 → 142 → 130, which is annotated as D-dethiobiotin (C_10_H_18_N_2_O_3_).

Peak 17 is characterized as fatty acid and conjugate at m/z 293.2092 as [M+Na]^+^ with MS/MS fragmentation: 293 → 292 → 279 → 275 → 224 → 165 → 162 → 137 → 125, which is annotated as16-oxo-palmitate.

One fatty acid was also detected in MPH of SW (peak 19) at m/z 305.2092 as [M+Na]^+^ with MS/MS fragmentation: 305 → 288 → 239 → 222 → 149 → 137, annotated as 6-oxabicyclo[3.1.0]hexane-2-undecanoic acid methyl ester (C_17_H_30_O_3_).

###### 3.1.2.1.4 Carboxylic acid and derivative, organooxygen compound

Three carboxylic acids and derivative were detected in the MPH of SW (4, 8, and 11), which eluted between 3.06 and 4.756 min. Peak 4 was detected as [M+Na]^+^ of m/z 214.0855 with MS/MS fragmentation: 214 → 198 → 181 → 170 → 160 → 121 → 120, and annotated as trihomomethionine. Peak 8 was detected as [M+H]^+^ of m/z 197.128 with MS/MS fragmentation: 197 → 169 → 152 → 124, and annotated as fasoracetam, and peak 11 was detected as [M+H]^+^ of m/z 211.1433 with MS/MS fragmentation: 211 → 183 → 154 → 129, and annotated as L,L-Cyclo(leucylprolyl (C_11_H_18_N_2_O_2_).

One organooxygen compound was also detected in MPH of SW (peak 20) as [M+Na]^+^ at m/z 307.2253 with MS/MS fragmentation: 307 → 305 → 279 → 158 → 145 → 139, and annotated as 12-(2,3-dihydroxycyclopentyl)-2-dodecanone.

###### 3.1.2.1.5 Pyridine and derivative, pyrimidine nucleoside, macrolide

One pyridine and derivative compound was detected in MPH of SW (peak 10) as [M+Na]^+^ at m/z 263.1376 with MS/MS fragmentation: 263 → 216 → 184 → 175 → 136 → 120, and annotated as pirbuterol (C_12_H_20_N_2_O_3_).

Peak 22 is characterized as pyrimidine nucleoside at m/z 354.9818 as [M+H]^+^ with MS/MS fragmentation: 355 → 291 → 281 → 262 → 179 → 173 → 149, and annotated as idoxuridine (C_9_H_11_IN_2_O_5_).

One macrolide compound was also detected in MPH of SW (peak 12) as [M+H]^+^ at m/z 696.4335 with MS/MS fragmentation: 696 → 598 → 303 → 291 → 213 → 202 → 166 → 142 → 133 → 120, and annotated as mycinamicin IV (C_37_H_61_NO_11_).

###### 3.1.2.1.6 Alcohol and polyol, amino glycoside, isoquinoline and derivative, polycyclic aromatic hydrocarbon

Peak 13 annotated belonged to alcohol and polyol class and peak 14 to amino glycoside classes of compound. Peak 18 that gave [M+Na]+ ion at m/z 493.014 with MS/MS fragmentation: 493→ 456 → 436 → 304 → 280 → 277 → 207 → 185 → 121 → 120 is annotated as 1-phosphatidyl-1D-myoinositol 3-phosphate and peak 14 that gave [M+H]^+^ at m/z 418.2672 with MS/MS fragmentation: 418 → 417 → 400 → 397 → 368 → 357 → 325 → 318 → 267 → 254 is annotated as istamycin A1 (C_18_H_35_N_5_O_6_).

Two isoquinolines and a derivative class of compounds were detected in MPH of SW. Isocarbostyril and Fleroxacin eluted at a retention time of 3.538 to 9.925 min. respectively, at m/z 146.0596 (peak 7) as [M+H]^+^ with MS/MS fragmentation: 146 and m/z 370.1368 (peak 15) as [M+H]^+^ with MS/MS fragmentation: 370 → 333 → 301 → 280 → 274 → 272 → 211. One polycyclic aromatic hydrocarbon was also detected in MPH of SW (peak 16) at m/z 325.0987 as [M+Na]^+^ with MS/MS fragmentation: 325→ 279 → 231→ 224 → 205 → 191→ 185 → 152, annotated as dibenzo[a,e]pyrene (C_24_H_14_).

###### 3.1.2.1.7 Benzene and substituted derivatives, cyclohexenone

Six benzene and substituted derivative were detected in MPH of SW. Peak numbers 5, 6, and 21 were annotated in ESI+ and peak numbers 4, 11, and 12 were annotated in ESI−. Peak 5 as [M+H]^+^ of m/z 194.117 with MS/MS fragmentation: 194 → 151 → 146 → 135 → 130 is annotated as butyl 2-aminobenzoate. Peak 6 that gave [M+H]^+^ of m/z 214.0856 with MS/MS fragmentation: 214 → 209 → 196 → 186 → 168 → 146 → 132 is annotated as salicylanilide. Peak 21 that gave [M+H]^+^ of m/z 358.978 with MS/MS fragmentation: 359 → 356 → 293 → 283 → 242 → 228 → 149 → 137 is annotated as chlorfenvinphos. In ESI-negative ionization, peak 4 that gave [M-H]^−^ of m/z 356.2276 with MS/MS fragmentation: 356 → 342 → 331 → 284 → 279 → 185 → 116 is annotated as oxybutynin. Peaks 11 and 12 that gave [M-H]^−^ of m/z 265.1558 and 265.1549 with MS/MS fragmentation: 266 → 235 → 198 → 137 → 136 → 117, and 265 → 154 → 137 → 138 → 133 → 117 → 113 are annotated as atenolol and practolol, respectively.

### 3.2 Growth attributes

In order to evaluate the efficacy of the combination of the two algal extracts in different proportions, trials were laid out in a two-factor design. The maize plants were foliar sprayed with three different concentrations [0, 0.35% (lower dose), and 0.7% (higher dose)] of the six different combinations prepared from MPH of the two seaweeds (MPHs of KA and SW) by blending in six different proportions ranging from 100:0 to 0:100. The results in growth parameters of maize data are illustrated in **Table 5**.

**Table 5:** The effects MPHs of seaweeds on maize growth.

Parameters		Plant height with tassel (cm)	Stem Dia. (mm)	Dry matter accumulation (g plant^-1^)			Cob parameter	
				Leaf	Stem	Root	Cob length with husk (cm plant^-1^)	cob length up to grain filling (cm plant^-1^)
Factor A								
T1 (KA : SW 100:0)		183.6 ± 15.1^a^	17.1 ± 1.5^b^	36.6 ± 4.1^b^	40.7 ± 7.9^b^	17.3 ± 6.0^a^	21.6 ± 2.0^a^	10.5 ± 1.2^ab^
T2 (KA : SW 80:20)		186.2 ± 14.3^a^	18.3 ± 1.4^a^	40.6 ± 5.2^a^	45.7 ± 7.5^ab^	16.9 ± 3.4^a^	20.9 ± 2.2^ab^	10.6 ± 0.9^ab^
T3 (KA : SW 60:40)		177.4 ± 19.4^b^	17.4 ± 1.8^ab^	38.2 ± 4.9^ab^	44.4 ± 6.9^ab^	18.1 ± 4.0^a^	20.5 ± 1.8^ab^	10.0 ± 1.2^ab^
T4 (KA : SW 40:60)		185.9 ± 11.8^a^	18.0 ± 1.7^ab^	40.3 ± 5.3^a^	46.9 ± 9.7^a^	18.1 ± 6.3^a^	20.9 ± 2.5^ab^	10.7 ± 1.2^a^
T5 (KA : SW 20:80)		183.0 ± 14.5^ab^	17.9 ± 1.2^ab^	39.8 ± 4.4^a^	42.2 ± 4.4^ab^	17.9 ± 5.2^a^	20.6 ± 1.9^ab^	9.97 ± 0.6^b^
T6 (KA : SW 0:100)		177.1 ± 20.1^b^	18.1 ± 2.0^ab^	38.3 ± 7.4^ab^	43.6 ± 7.9^ab^	18.1 ± 4.6^a^	20.1 ± 2.3^b^	10.3 ± 0.7^ab^
Factor B	Foliar applications							
	0%	167.4 ± 8.9^c^	16.7 ± 1.0^b^	34.2 ± 2.1^b^	40.2 ± 7.5^b^	15.7 ± 3.6^b^	19.2 ± 1.7^b^	9.7 ± 0.9^b^
	0.35%	191.3 ± 10.1^a^	18.5 ± 1.6^a^	42.0 ± 4.5^a^	46.4 ± 5.9^a^	18.5 ± 4.7^a^	21.8 ± 1.7^a^	10.8 ± 0.9^a^
	0.7%	187.9 ± 16.3^b^	18.3 ± 1.6^a^	41.0 ± 5.4^a^	45.4 ± 8.1^a^	19.1 ± 5.7^a^	21.4 ± 2.1^a^	10.5 ± 0.9^a^
Interaction A × B	Applied Conc. Of MPHs of KA & SW							
T1 (KA : SW 100:0)	0%, 0%	166.8 ± 6.1^gh^	15.81 ± 0.8^d^	32.2 ± 1.7^g^	35.2 ± 8.6^d^	13.8 ± 2.8^d^	20.1 ± 1.5^bcd^	9.4 ± 0.7^e^
	0.35%, 0%	189.3 ± 10.6^bcd^	17.572 ± 1.8^abcd^	37.6 ± 1.9^cdef^	44 ± 6.7^bcd^	14.3 ± 3.9^d^	22.26 ± 1.6^ab^	10.7 ± 0.8^abcde^
	0.7%, 0%	194.6 ± 10.1^abc^	18.024 ± 0.5^abc^	39.8 ± 3.6^bcde^	43 ± 6.5^bcd^	23.8 ± 4.7^a^	22.4 ± 2.3^ab^	11.4 ± 1.2^ab^
T2 (KA: SW 80:20)	0%, 0%	169.1 ± 6.2^g^	17.44 ± 0.5^bcd^	34.6 ± 1.6^fg^	39.4 ± 7.1^cd^	15.4 ± 3.5^cd^	18.74 ± 1.1^d^	9.9 ± 0.5^de^
	0.28%, 0.07%	198 ± 6.3^ab^	19.308 ± 1.2^ab^	43.6 ± 3.8^ab^	48.9 ± 7.7^ab^	17.8 ± 3.3^abcd^	23 ± 1.3^a^	11.3 ± 1.0^abc^
	0.56%, 0.14%	191.6 ± 8.0^abcd^	18.184 ± 1.6^abc^	43.8 ± 2.6^ab^	48.7 ± 3.6^abc^	17.6 ± 3.5^abcd^	21 ± 1.5^abcd^	10.46 ± 0.6^abcde^
T3 (KA : SW 60:40)	0%, 0%	155.3 ± 10.6^i^	15.81 ± 0.4^d^	33.2 ± 1.7^fg^	36.8 ± 3.4^d^	14.7 ± 1.9^d^	19 ± 1.2^cd^	9.4 ± 1.4^e^
	0.21%, 0.14%	182 ± 9.5^def^	17.93 ± 2.1^abc^	40.8 ± 2.8^abcde^	47.1 ± 3.7^abc^	21.3 ± 2.6^abc^	20.1 ± 1.3^bcd^	10.4 ± 1.2^abcde^
	0.42%, 0.28%	194.8 ± 10.0^abc^	18.31 ± 1.6^abc^	41.0 ± 4.9^abcde^	49.4 ± 5.2^ab^	18.3 ± 4.3^abcd^	22.4 ± 1.1^ab^	10.2 ± 1.1^bcde^
T4 (KA : SW 40:60)	0%, 0%	175.1 ± 9.3^efg^	16.668 ± 0.7^cd^	34.2 ± 1.5^fg^	40.2 ± 8.8^bcd^	14.6 ± 4.2^d^	19.3 ± 2.1^cd^	10 ± 1.4^cde^
	0.14%, 0.21%	193.2 ± 7.6^abcd^	18.514 ± 1.9^abc^	43.8 ± 4.1^ab^	47.3 ± 8.3^abc^	17.5 ± 6.3^abcd^	22.9 ± 0.7^a^	11.52 ± 0.8^a^
	0.28%, 0.42%	189.4 ± 10.8^bcd^	18.67 ± 1.6^abc^	43.4 ± 2.7^ab^	53.3 ± 8.7^a^	22.1 ± 6.7^ab^	20.6 ± 3.0^abcd^	10.6 ± 0.9^abcde^
T5 (KA : SW 20:80)	0%, 0%	166.6 ± 4.3^gh^	17.492 ± 0.3^abcd^	36.4 ± 1.6^defg^	40.9 ± 4.2^bcd^	16.9 ± 4.0^bcd^	18.65 ± 1.4^d^	9.5 ± 0.5^e^
	0.07%, 0.28%	185 ± 5.5^cde^	18.042 ± 0.7^abc^	41.4 ± 3.8^abcd^	42.9 ± 3.4^bcd^	18 ± 5.5^abcd^	21 ± 1.1^abcd^	10.2 ± 0.6^bcde^
	0.14%, 0.56%	197.4 ± 9.2^ab^	18.246 ± 2.0^abc^	42.2 ± 5.3^abc^	42.8 ± 6.0^bcd^	18.7 ± 6.8^abcd^	22.1 ± 1.5^ab^	10.2 ± 0.4^bcde^
T6 (KA : SW 0:100)	0%, 0%	171.5 ± 2.2^fg^	17.206 ± 1.4^bcd^	35.8 ± 1.7^efg^	48.4 ± 6.4^abc^	19 ± 3.3^abcd^	19.4 ± 2.8^cd^	9.9 ± 0.7d^e^
	0%, 0.35%	202.6 ± 5.0^a^	19.628 ± 1.3^a^	46 ± 5.9^a^	47.7 ± 4.1^abc^	21.8 ± 2.9^abc^	21.5 ± 2.1^abc^	11 ± 0.7^abcd^
	0%, 0.7%	157.2 ± 5.7^hi^	17.55 ± 2.3^abcd^	33.6 ± 7.1^fg^	34.8 ± 3.5^d^	13.4 ± 3.0^d^	19.5 ± 1.9^cd^	10.1 ± 0.2^bcde^
Analysis of variance								
Treatments (factor A)		**	ns	ns	ns	ns	ns	ns
Foliar appli. (factor B)		***	***	***	***	**	***	***
Factor (A × B)		***	ns	*	**	**	ns	ns

KA, *Kappaphycus alvarezii*; SW, *Sargassum wightii*; ns, non-significant. Values represented are mean of 5 replicates. Values followed by different alphabets in the top of the bar are significantly different at *p* *** significant at *p* < 0.001, ** significant at *p* < 0.05, * significant at *p* < 0.5 using Duncan's Multiple Range test (DMRT).

The analysis of variance of maize plant height showed that both the main effects (combinations of MPHs and concentrations) and their interactions varied significantly. The interaction results further revealed that compared to their respective controls, the plant height increased (10%–25%) in each of the different combinations applied at either lower or higher doses except in case of 100% MPH of SW applied at higher dose, wherein it decreased by 8%. The highest improvement of 25% was recorded in plant height under KA : SW at 60:40 when applied at the highest dose of 0.7%. The main effect revealed that the plant stem diameter did not significantly vary due to the different combination, while it increased significantly over control due to application at either of the doses. The dry matter accumulation in leaf increased significantly by 17%–28% over their respective controls, under all the combinations containing MPH of KA up to 40% (T1–T4) at either of the doses, while in case of KA : SW of 80:20, the higher concentration was effective. SW when applied alone (100% SW) at the highest concentration did not improve leaf dry matter content over control in contrast to significant improvement observed under lower dose. The interaction results revealed that compared to their respective controls, a significant improvement in DMA in stem was obtained in the proportion 80:20 KA : SW (T2) when applied at the lower dose, in 60:40 KA : SW when applied at both the doses, and 40:60 KA : SW when applied at the highest dose, improvements being in the range of 24%–34%. No change was observed with respect to control in other treatments at any of the doses except when 100% SW was applied at the highest dose.

Improvements in DMA in roots over their respective controls were observed using a lower dose of KA : SW at 60:40 and the higher dose of 100:0 and 40:60. The main effects revealed that no differences were found among the different seaweed extract combinations (T1–T6) with respect to DMA in different plant parts, and significant improvements in them were found due to the lower as well as higher dose over control. Application of higher dose though did not result in any improvement in DMA over the lower results as both these levels were on par with each other.

### 3.3 Yield Attributes and yield

With respect to cob length, among the main effects and their interaction, only the concentration factor was found to be significant (**Table 6**). Improvements in total length of cob (12%–14%) and the fill length of the cob (8%–11%) were found over control by either of the MPH concentrations used for the foliar spray, both of which were on par with each other. The lowest number of seeds was observed in all the respective controls of the combination treatments (246–287), while the highest was found in T2 (368) applied at a lower dose that was, however, on par with T6 and T4, also at a lower dose. The main effect of concentration revealed that the 100-seed weight varied from 7% to 12% over control; however, no effect was found with respect to factor 2 (concentration) of MPH combination or at the interaction level. The main effects as well as interaction were found significant for seed yield. Compared to their respective controls, 100% MPH of KA (T1) increased the seed yield per plant at a lower as well as a higher dose (38%–42%), while 100% SW (T6) increased it (38%) only at a lower dose. A higher dose of SW was found on par with the control for seed yield. Among the combinations, all of them gave numerically higher seed yield than their respective controls at both doses, but significant improvements were observed at lower doses of T2 (80: 20 KA : SW) and T4 (40:60 KA : SW), all of which were on par with each other (**Table 6**). The interaction as well as the main effect on concentration revealed no additional improvement over control by applying a higher dose of the different MPAH combination treatments.

**Table 6:** The effects MPHs of seaweeds on maize yield and yield attributes.

Parameters		Yield attributes		
		No. of seed per cob	100 seed weight (g)	Total seed weight (g)
Factor A				
T1 (KA : SW 100:0)		286.9 ± 37.9^b^	10.7 ± 1.3^a^	31.4 ± 7.8^a^
T2 (KA : SW 80:20)		316.3 ± 47.9^a^	9.6 ± 1.9^a^	31.0 ± 8.0^a^
T3 (KA : SW 60:40)		293.1 ± 23.7b	9.8 ± 1.8^a^	28.4 ± 6.1^a^
T4 (KA : SW 40:60)		302.3 ± 52.4^ab^	10.5 ± 1.3^a^	31.7 ± 6.3^a^
T5 (KA : SW 20:80)		315.7 ± 37.6^a^	10.0 ± 1.5^a^	31.2 ± 6.7^a^
T6 (KA : SW 0:100)		317.6 ± 45.4^a^	9.9 ± 1.2^a^	30.8 ± 6.3^a^
Factor B	Foliar applications			
	0%	269.9 ± 30.7^c^	9.4 ± 1.5^b^	25.4 ± 5.5^b^
	0.35%	334.3 ± 41.6^a^	10.1 ± 1.6^a^	34.0 ± 6.4^a^
	0.7%	311.8 ± 25.5^b^	10.6 ± 1.2^a^	33.2 ± 5.2^a^
Interaction A × B	Applied Conc. of MPHs of KA & SW			
T1 (KA : SW 100:0)	0%, 0%	250 ± 22.1^h^	9.85 ± 0.6^ab^	24.8 ± 4.6^de^
	0.35%, 0%	283.6 ± 18.0^fgh^	11.35 ± 1.9^a^	34.1 ± 6.2^ab^
	0.7%, 0%	327.2 ± 21.4^bcde^	10.83 ± 0.6^a^	35.1 ± 8.5^a^
T2 (KA: SW 80:20)	0%, 0%	271.4 ± 23.6^gh^	8.38 ± 2.1^b^	23.95 ± 8.9^e^
	0.28%, 0.07%	368.2 ± 24.8^a^	9.69 ± 1.5^ab^	35.8 ± 6.6^a^
	0.56%, 0.14%	309.4 ± 30.3^defg^	10.8 ± 1.4^a^	33.1 ± 2.3^abcd^
T3 (KA : SW 60:40)	0%, 0%	278.8 ± 25.1^gh^	9.26 ± 2.0^ab^	25.5 ± 6.3^cde^
	0.21%, 0.14%	298.4 ± 26.1^defg^	9.44 ± 2.4^ab^	28.1 ± 6.9^abcdef^
	0.42%, 0.28%	302 ± 16.4^defg^	10.69 ± 0.8^a^	31.7 ± 4.0^abcde^
T4 (KA : SW 40:60)	0%, 0%	246.2 ± 30.2^h^	9.83 ± 1.6^ab^	25.3 ± 5.7^cde^
	0.14%, 0.21%	354.2 ± 30.1^abc^	10.73 ± 0.7^a^	36.0 ± 3.9^a^
	0.28%, 0.42%	306.4 ± 21.9^defg^	11.04 ± 1.4^a^	33.7 ± 3.7^abc^
T5 (KA : SW 20:80)	0%, 0%	286.6 ± 29.8^efgh^	9.37 ± 1.8^ab^	26.4 ± 5.0^bcde^
	0.07%, 0.28%	337.8 ± 31.9^abcd^	9.78 ± 1.7^ab^	33.4 ± 8.9^abc^
	0.14%, 0.56%	322.8 ± 36.4^cdef^	10.77 ± 1.0^a^	33.7 ± 3.4^abc^
T6 (KA : SW 0:100)	0%, 0%	286.2 ± 36.7^efgh^	9.90 ± 1.0^ab^	26.5 ± 3.6^bcde^
	0%, 0.35%	363.4 ± 03^ab^	10.12 ± 1.2^ab^	36.5 ± 3.2^a^
	0%, 0.7%	303.2 ± 23.9^defg^	9.62 ± 1.6^ab^	29.4 ± 7.0^abcde^
Analysis of variance Treatments (Factor A) Foliar Application (Factor B) Factor (A × B)				
		***	ns	*
		***	**	***
		***	ns	**

Data are presented as mean ± SD (n = 5). Values in the same column with different superscript letters are significantly different based on Duncan’s Multiple Range Test (DMRT) test. KA, Kappaphycus alvarezii; SW, Sargassum wightii; ns, non-significant,

*p< 0.05, **p< 0.01, ***p< 0.001.

### 3.4 Gas exchange parameters

Photosynthetic rate and other gas exchange parameters like Fv/Fm ratio, Ci, phi PS2, qP, NPQ, ETR, TR, WUE, and Ci/Ca were measured. No significant variations were found in any of these gas exchange parameters due to the two main effects as well as their interaction (**Supplementary Table 4**).

### 3.5 Total ROS, antioxidant enzymes, and metabolite analysis

**Figure 6:**
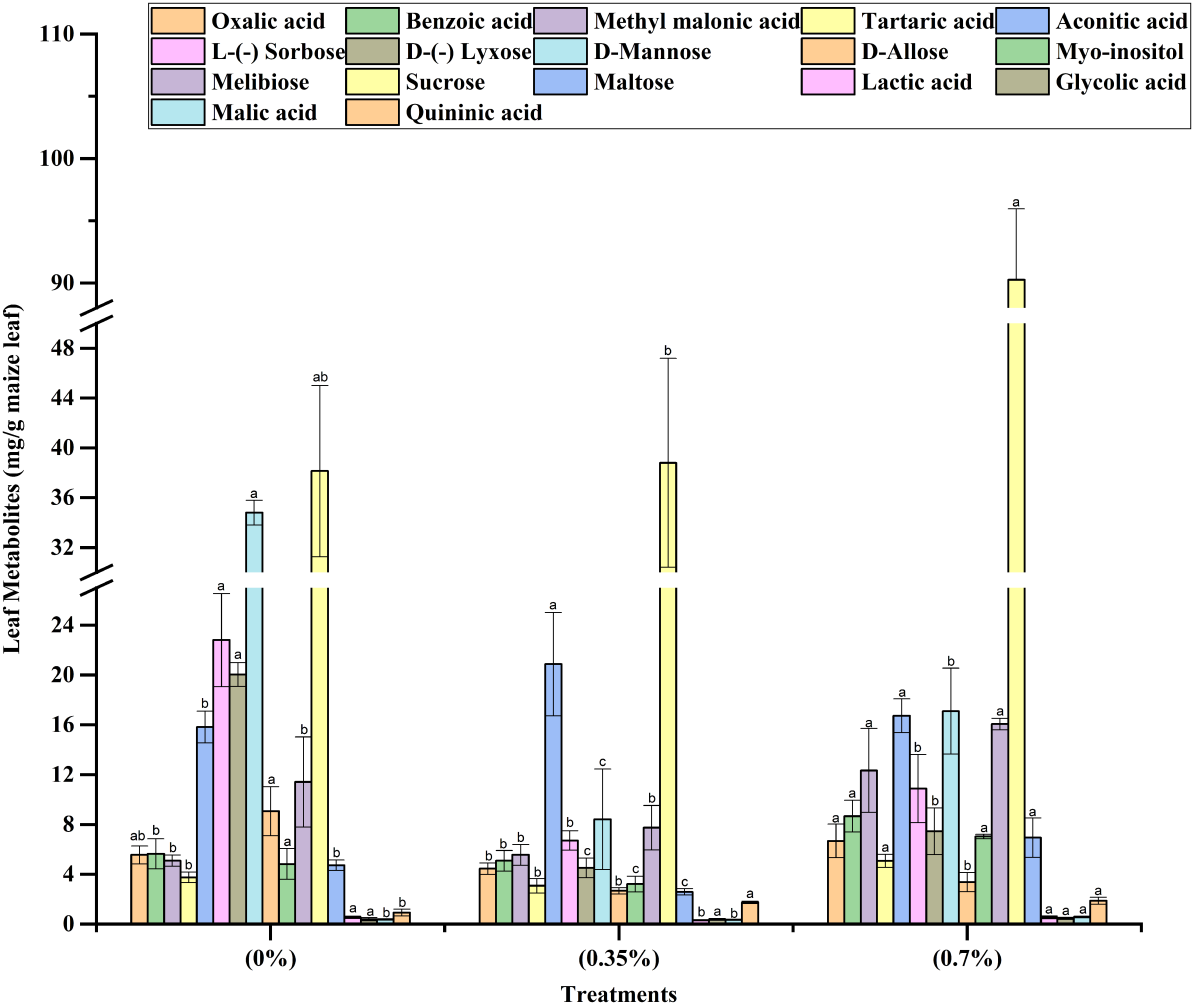
Effects of MPHs on maize leaf metabolite concentrations. Values represented are mean of 5 replicates. Values followed by different alphabets in the top of the bar are significantly different at p < 0.05 using Duncan's Multiple Range test (DMRT).

**Figure 7:**
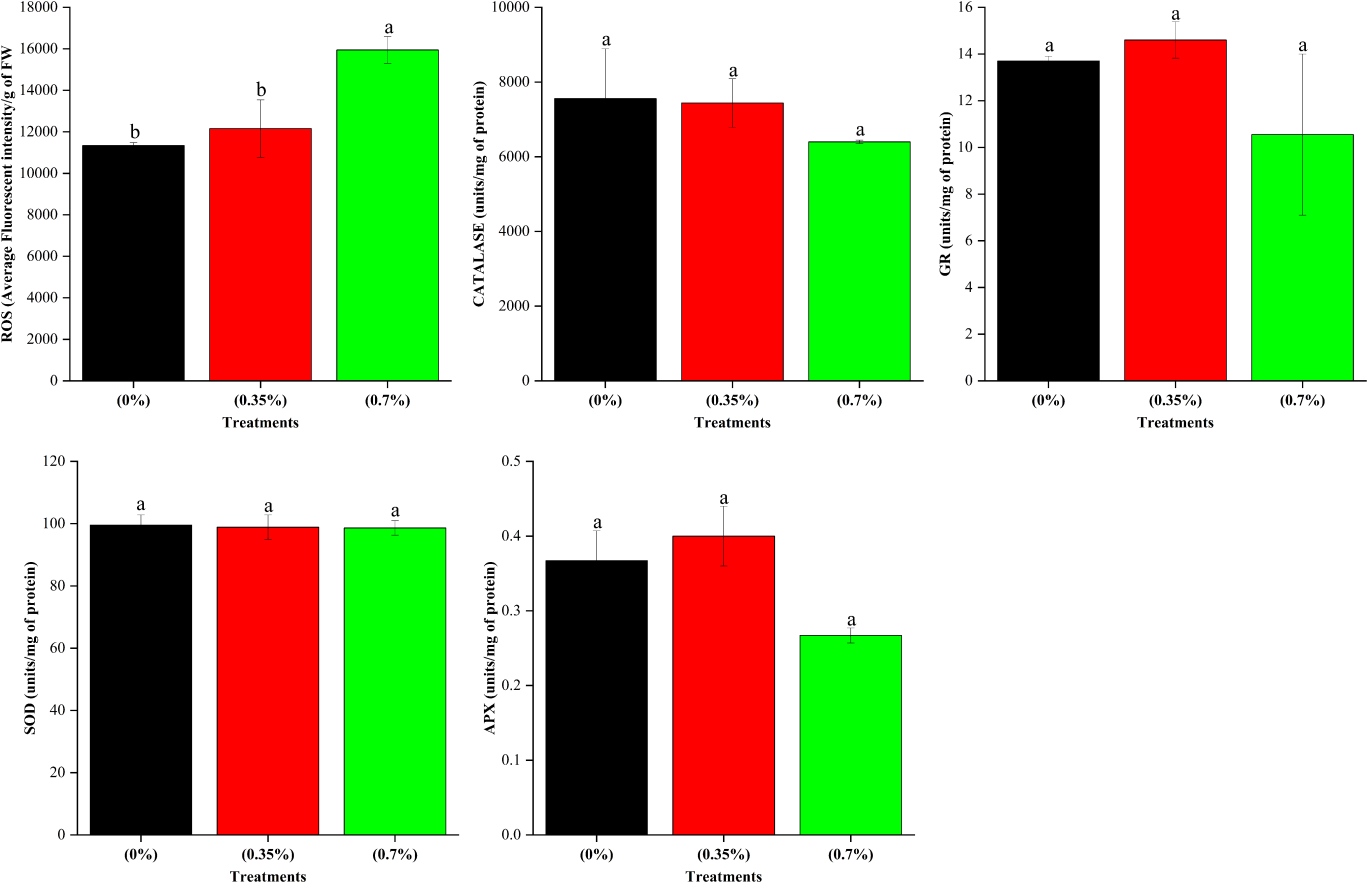
Effects of MPHs on antioxidant enzymes and total ROS in maize leaf. Values represented are mean of 5 replicates. Values followed by different alphabets in the top of the bar are significantly different at p < 0.05 using Duncan's Multiple Range test (DMRT).

The results on height and dry matter accumulation of stem revealed that while the lower dose of 100% MPH of SW enhanced these parameters over control, the higher dose brought about a decrease. Thus, in order to decipher any underlying mechanism, we analyzed the leaf metabolites including total ROS and antioxidants in the three treatments, namely, control, low dose, and high dose of 100% MPH. It was found that out of 17 metabolites that were analyzed using GC-MS, 10 were found in the highest quantity under the high dose of 100% MPH of SW (**Figure 6**). These include benzoic acid, methyl malonic acid, tartaric acid, melibiose, and malic acid. The metabolites myoinositol, sucrose, oxalic acid, and lactic acid were found to be higher at a high dose compared to a low dose of MPH of SW, but were on par with control. Glycolic acid did not differ with any of the treatments. No statistical change in antioxidant enzymes, namely, catalase, GR, SOD, and ascorbate peroxidase, was observed in these three treatments; however, a significant increase in ROS was found in the high dose compared to that in low MPH of SW as well as control (**Figure 7**).

### 3.6 Fatty acid analysis and computational maize leaf membrane composition

In order to understand any impact of higher dose on leaf cell membrane, the content of saturated and unsaturated fatty acids was analyzed for the three treatments, namely control, low dose, and high dose of 100% MPH of SW ( **Supplementary Tables 5A, B**). The data were further used for computational modeling of model cell membrane and understanding its properties as described in Section 3.6.1.

#### 3.6.1 Effect of MPHs on lipid composition and model membranes

##### 3.6.1.1 Area per lipid molecule

To observe the effect of algal sap on the membranes, the area per lipid molecule (AL) is a crucial variable for characterizing the membrane structure (**Supplementary Figure 5A**). As shown in plots, the average AL was found to be similar (~46 Å) for all three different model membranes. This also suggests that varying sap concentrations have no significant effect on maize thylakoid membranes.

##### 3.6.1.2 Bilayer thickness

Furthermore, the lipid bilayer thickness was calculated, described by the distance between the average positions of the lipid phosphate groups in two bilayer leaflets. Similar to area per lipid, there was no significant difference in the thickness of all membranes (**Supplementary Figure 5B**).

##### 3.6.1.3 Order parameter

To quantify the order of the membrane, the order parameter of lipid acyl chains, $-S_{CD}$ , was calculated using the following equation:


(1) $$-S_{CD}=\frac{1}{2}\;\langle \;3{\text{cos}}^{2}Ө\;-\;1\;\rangle$$

where θ is the angle of a C−H vector with respect to the bilayer normal. The higher the value of $-S_{CD}$ , the more ordered the lipid membrane will be. The results show that compared to control, 100% MPH at low or high dose has no significant impact on the ordering of the bilayer membrane. Among the three different lipid acyl chain types included in the study, as expected, the saturated palmitic acid chains were found to be the most ordered ones, followed by monounsaturated oleic chains, while the lowest-order parameters were observed for polyunsaturated linolenic acid chains (**Supplementary Figure 6**) in three systems. For all three systems studied here, the $-S_{CD}$ values of the fatty acid tails were found to be in the following order: lipid with dipalmitoyl (DP) chains > palmitoyl-oleoyl (PO) chains > palmitoyl-linoleoyl (PL) chains.

## 4 Discussion

The results on growth and yield parameters, in general, revealed that the maize plants elicited response at the lower level itself (0.35%), when compared over control. This also corroborated the hypothesis in an earlier study with MPHs of KA and SW in tomato, where it was inferred that the MPHs have sufficient amounts of bioactive ingredients to be physiologically relevant even if it is applied at a still lower concentration than 0.8% (Vaghela et al., 2023). Furthermore, it was also observed that the application of the MPHs at a higher level did not bring about any significant additional advantage over the penultimate dose. This might be explained on the basis of the law of diminishing marginal returns (Patton, 1926) wherein there is a limit to how much a particular crop can benefit from additional inputs before reaching a point where plants are alleviated off all the limiting factors, such as pest pressure, stress, physiological processes, etc. Beyond this limit, the crop may not be able to utilize the extra inputs efficiently and thus not result in a proportional increase in crop yield. This is in agreement with a study in tomato where tomato yield was favorably influenced at lower doses (0.8%) of MPH of KA and SW or their combinations, while no incremental yield was recorded by raising the dose (Vaghela et al., 2023). Since, in many of the MPH treatments, a slight although non-significant decrease in the growth and yield parameters was observed by applying a higher dose of MPHs compared to that in lower dose, an attempt was made to investigate whether the higher doses tend to interfere with the leaf membrane configuration. This was very prominent in the treatment employing 100% MPH of SW, wherein a slight decrease in height, stem, and root weight was also observed. The leaf membrane modeling study conducted on the basis of different lipid composition of the control, low, and high MPH of SW revealed that there was no change in the membranes among the higher or lower MPH-treated leaves, both of which were also found on par with respect to area per lipid molecule, membrane thickness, and other membrane order parameters (**Supplementary Figures A, B**, **6**). There was also no significant change in the gas exchange parameters in these treatments. Investigations were also carried out to decipher whether there were any changes in the metabolites, antioxidant status, and ROS production due to the variable doses of the MPHs of SW on leaves of maize plants. Although no changes were observed in any of the antioxidant enzymes studied, namely, APX, SOD, catalase, and GR due to lower or higher MPH of SW, when compared to control, the ROS content under high MPH dose was enhanced over both control and low MPH dose. The metabolite profile also revealed that majority of the metabolites including sugar and organic acids like benzoic acid, methyl malonic acid, tartaric acid, melibiose, maltose, and malic acids were high at a higher dose of MPH. It is hypothesized that a higher dose of MPH of SW, through some unknown mechanism, might trigger ROS formation, the perception of which might have induced higher accumulation of these compounds as a stress response for achieving metabolic adaptation and cellular homeostasis. Some of these metabolites, like benzoic acid, also act as an antioxidant that has a protective response to mitigate oxidative stress and reduce cellular damage. Some of the molecules like maltose and myoinositol that were found high at the higher dose compared to control and low dose also act as signaling molecules in response to stress and can activate stress-related pathways. Nevertheless, no significant reduction in seed yield over control was observed in any of the treatments at the higher dose, suggesting no drastic response in plants.

In the present study, compared to control, no significant improvement due to any of the treatments was found in net photosynthetic rate of the MPH-treated plants (**Supplementary Table 4**). However, there were significant improvement in seed yield obtained in these treatments. This might be due to enhanced leaf dry matter accumulations, which were observed in the corresponding treatments where good yield response was there; as a result, the total photosynthate production was enhanced, which was eventually partitioned into the seeds to give higher seed yields due to the application of the MPHs. Improved dry matter accumulation might be due to the presence of several bioactive ingredients present in the MPHs of KA and SW, which influence crop growth and quality. For example, both MPH of KA and SW contains 5.92 and 1.49 mg g^−1^ mannitol (Vaghela et al., 2023), which has a role in enhancing growth, biomass, and alleviating stress (Habiba et al., 2019). The MPHs also have organic acids like oxalic acid, propionic acid, tartaric acid, malic acid, and glutamic acid (Vaghela et al., 2023), which have known bioactivity towards increasing crop growth and root activity (Awad-Allah and Elsokkary, 2020). Similarly, the tyramine present in both the MPH (Vaghela et al., 2023) has a role in positively modulating crop growth and development. The MPH also contains glycine (Vaghela et al., 2023), which has been reported to increase the growth of coriander when applied at a very low concentration of 5 ppm on the plants (Mohammadipour and Souri, 2019).

The present work sought to investigate the effect of application of MPH of KA and SW either alone or in blended form in certain proportions, while also characterizing the metabolites present in the two MPHs. Although a detailed characterization of both the MPHs were reported earlier (Vaghela et al., 2023), this study attempted to additionally characterize the untargeted metabolites in them using liquid chromatography with high-resolution mass spectrometry, which detected a number of compounds hitherto undetected. Several classes of compounds were identified that might be involved to enhance the growth and yield of maize. The identified compounds have significant biological involvement towards improving plant growth and stress response. The known bioactivities of the annotated compounds found in the two MPHs are described in **Supplementary Table 3**. Among the annotated bioactive compounds, Retroscine, whose presence was identified in both the MPHs, has been reported to have antifungal activity against plant pathogen (Pedras and Yaya, 2015). Tyrosyl-glycine (Dipeptide) act as signal molecules to trigger cell–cell signaling for plant growth and defense mechanisms (Hu et al., 2018). This is in agreement with an earlier report (Vaghela et al., 2022) wherein this peptide compound was also identified in KA methanolic extract. Hexyl 2-furoate has been found to have antioxidant activity (Zannou and Koca, 2019). 1-Phosphatidyl-1D-myo-inositol, which is a class of phosphatidylinositol, was associated with plant responses to various ecological stimuli (Meijer and Munnik, 2003; Krinke et al., 2006; Im et al., 2010; Im et al., 2011) and was identified in MPH of KA. Our previous study also identified this compound in KA extract (Vaghela et al., 2022). The phosphorylated phosphatidylinositol lipids biosynthesized from phosphatidyl-1D-myo-inositol serve as a secondary messenger in signal transduction and have been implicated in vesicle trafficking (Brochet et al., 2014). The 12-(2,3-Dihydroxycyclopentyl)-2-dodecanone compound plays a vital role against herbivory (Perera et al., 2017). One insecticidal compound, chlorfenvinphos, identified in MPH of KA has pesticidal properties (Lukaszewicz-Hussain, 2008), while guaiazulene has herbicidal properties (Bakun et al., 2021). In MPH of SW, the trihomomethionine compound was identified, which is the key molecule to produce glucosinolates that have the property to mitigate abiotic and biotic stresses (Zang et al., 2008; Chowdhury, 2022). Butyl 2-aminobenzoate has insect-repellent properties (Ida, 2015) and salicylamide has been used for fungicidal products (Kraushaar, 1954). One organooxygen compound 12-(2,3-dihydroxycyclopentyl)-2-dodecanone was identified. It has insecticidal activities against *Tribolium castameum* and *Lasioderma* adult insects (Wang et al., 2019). These compounds probably work in unison to provide boost to plant growth, while also warding off biotic and abiotic stress, thus increasing productivity as also evident in the results of the study. Further studies should focus on quantification of these compounds in the MPHs and determine the effective concentration eliciting plant response.

## 5 Conclusion

This study details the effect of foliar application of minimally processed aqueous homogenates (MPHs) derived from dry KA and SW (either alone or in certain proportions on maize plants). The study also involved comprehensive characterization of compounds, hitherto not reported in the two formulations using the LC-HRMS-MS/MS approach. The study indicated that the bioactive compounds in MPHs of KA and SW play a significant role in promoting plant growth, yield, and metabolic changes in maize crop. A lower dose of 0.35% of the MPHs, either alone or in certain proportions, was instrumental in eliciting favorable growth and yield of maize compared to control, while a higher dose of 0.7% gave no additional improvement in yield over that obtained by lower dose. Future research efforts should prioritize the measurement and quantification of these compounds. It is essential to determine the precise concentration of these compounds that effectively trigger a favorable response in plants. By establishing the optimal concentration, we can better understand and harness the potential benefits of these compounds for plant growth and development.

## Data availability statement

All the data used for this manuscript including total ion chromatogram and spectra are given in supplementary files of this manuscript.

## Author contributions

PV: Data curation, Formal analysis, Writing – original draft. GG: Formal analysis, Writing – original draft. KT: Data curation, Writing – review & editing. KV: Writing – review & editing, Data curation. DC: Writing – review & editing, Formal analysis, Software. MM: Writing – review & editing. TS: Writing – review & editing, Resources. AS: Resources, Writing – review & editing. MS: Resources, Writing – review & editing, Methodology. AG: Writing – review & editing, Conceptualization, Data curation, Funding acquisition, Supervision, Validation.
