# Supplementary material for: Underpinning beneficial maize response to application of minimally processed homogenates of red and brown seaweeds
Source: Front Plant Sci. 2023 Nov 30;14:1273355. doi: 10.3389/fpls.2023.1273355 (PMC10723902; doi:10.3389/fpls.2023.1273355)
Supplement: Supplementary file 1 [file DataSheet_1.zip › Supplementary Table 1.DOCX]

**Supplementary Table 1: Flow rate and solvent composition of the procedure in Q-TOF-HRMS**

| **Time**  **(minutes)** | **Solvent (A)**  **(%)** | **Solvent (B)**  **(%)** | **Flow rate (mL min-1)** | **Pressure (bar)** |
| --- | --- | --- | --- | --- |
| 1.00 | 95.00 | 5.00 | 0.300 | 1200.00 |
| 20.00 | 0.00 | 100.00 | 0.300 | 1200.00 |
| 25.00 | 0.00 | 100.00 | 0.300 | 1200.00 |
| 26.00 | 95.00 | 5.00 | 0.300 | 1200.00 |
| 30.00 | 95.00 | 5.00 | 0.300 | 1200.00 |
