# Supplementary material for: Underpinning beneficial maize response to application of minimally processed homogenates of red and brown seaweeds
Source: Front Plant Sci. 2023 Nov 30;14:1273355. doi: 10.3389/fpls.2023.1273355 (PMC10723902; doi:10.3389/fpls.2023.1273355)
Supplement: Supplementary file 1 [file DataSheet_1.zip › Supplementary Table 2.DOCX]

**Supplementary Table 2: Lipids of simulated model bilayer membranes**

| **Sr. No.** | **Polar headgroups** | **Acyl chains** | **No of lipids in**  **0%**  **treatment of MPHs** | **No of lipids in**  **0.35%**  **treatment of MPHs** | **No of lipids in**  **0.7%**  **treatment of MPHs** |
| --- | --- | --- | --- | --- | --- |
| 1 | MGDG | DP (16:0/16:0) | 50 | 48 | 44 |
| 2 |  | PO (18:1/16:0) | 6 | 6 | 6 |
| 3 |  | PL (18:2/16:0) | 8 | 10 | 14 |
| 4 | DGDG | DP (16:0/16:0) | 30 | 28 | 26 |
| 5 |  | PO (18:1/16:0) | 4 | 4 | 4 |
| 6 |  | PL (18:2/16:0) | 4 | 4 | 8 |
| 7 | SQDG | DP (16:0/16:0) | 10 | 10 | 10 |
| 8 |  | PO (18:1/16:0) | 2 | 2 | 2 |
| 9 |  | PL (18:2/16:0) | 2 | 2 | 2 |
| 10 | PG | DP (16:0/16:0) | 8 | 10 | 8 |
| 11 |  | PO (18:1/16:0) | 2 | 2 | 2 |
| 12 |  | PL (18:2/16:0) | 2 | 2 | 2 |

(MGDG- Monogalactosyldiacylglycerol, DGDG- Digalactosyldiacylglycerol, SQDG- Sulfoquinovosyldiacylglycerol, PG- Phosphatidylglycerol, DP- Di-palmitoyl acid, PO- Palmitoyl and Oleoyl acid, PL- Palmitoyl and Linoleoyl acid)
