# Supplementary material for: Underpinning beneficial maize response to application of minimally processed homogenates of red and brown seaweeds
Source: Front Plant Sci. 2023 Nov 30;14:1273355. doi: 10.3389/fpls.2023.1273355 (PMC10723902; doi:10.3389/fpls.2023.1273355)
Supplement: Supplementary file 1 [file DataSheet_1.zip › Supplementary Table 4.DOCX]

**Supplementary Table 4: Gas exchange parameters of maize as treated with MPHs of *Kappaphycus alvarezii* and *Sargassum wightii* in pot experiment**

| **Treatments** | **Applied Conc. Of MPHs of KA & SW** | **PR** | **Fv/Fm ratio** | **C*i (*µmol CO_2_ m^-2^s^-1^)** | **øPS2** | **qP** | **qN** | **NPQ** | **ETR** | **TR (mmol H_2_O m^-2^ s^-1^)** | **WUE (µmol CO_2_/mmol H_2_O)** | **Ci/Ca** |
| --- | --- | --- | --- | --- | --- | --- | --- | --- | --- | --- | --- | --- |
| T1 (KA:SW 100:0) | 0%, 0% | 19.9±3.6^a^ | 0.8±0.1^ab^ | 74.4±8.9^bcd^ | 0.3±0.1^a^ | 0.6±0.1^abc^ | 0.8±0^ab^ | 1.5±0.1^ab^ | 108.3±21.5^a^ | 3.1±0.8^ab^ | 6.7±1.6^a^ | 0.3±0.2^abc^ |
|  | 0.35%, 0% | 23±7.3^a^ | 0.8±0.1^ab^ | 91.4±5.8^abc^ | 0.3±0^a^ | 0.6±0^ab^ | 0.8±0^ab^ | 1.5±0.1^ab^ | 118.1±7.6^a^ | 3.4±0.6^ab^ | 7±2.6^a^ | 0.5±0.1^a^ |
|  | 0.7%, 0% | 23±1.9^a^ | 0.7±0^b^ | 112.9±12.2^abc^ | 0.3±0.1^a^ | 0.6±0.1^abc^ | 0.8±0.1^ab^ | 1.4±0.4^ab^ | 111.2±23.9^a^ | 3.8±0.6^a^ | 6.1±2.2^a^ | 0.4±0.1^ab^ |
| T2  (KA: SW 80:20) | 0%, 0% | 18±8.3^a^ | 0.8±0.1^ab^ | 67.5±25.4^d^ | 0.2±0^a^ | 0.5±0^bc^ | 0.7±0^b^ | 1.2±0.1^ab^ | 105.6±8.4^a^ | 2.9±0.2^ab^ | 6.1±2.6^a^ | 0.3±0.1^abc^ |
|  | 0.28%, 0.07% | 17.3±2.7^a^ | 0.8±0.1^ab^ | 102.6±20.8^a^ | 0.3±0.1^a^ | 0.6±0.1^abc^ | 0.8±0.1^ab^ | 1.4±0.2^ab^ | 107.3±20.1^a^ | 2.8±1^b^ | 6.4±1.2^a^ | 0.4±0.1^ab^ |
|  | 0.56%, 0.14% | 20.8±6.8^a^ | 0.8±0.1^ab^ | 109.8±17.6^a^ | 0.3±0^a^ | 0.6±0^ab^ | 0.7±0^ab^ | 1.4±0.2^ab^ | 117.0±8^a^ | 3.4±0.3^ab^ | 6.1±2.1^a^ | 0.4±0.1^ab^ |
| T3 (KA:SW 60:40) | 0%, 0% | 21.5±5.2^a^ | 0.8±0.1^ab^ | 58.9±9.9^d^ | 0.2±0^a^ | 0.5±0.1^bc^ | 0.8±0^ab^ | 1.4±0.2^ab^ | 104.5±10.1^a^ | 2.8±0.4^b^ | 7.6±0.8^a^ | 0.2±0.1^bc^ |
|  | 0.21%, 0.14% | 21.3±4.6^a^ | 0.8±0.1^ab^ | 69.1±5.8^cd^ | 0.2±0^a^ | 0.5±0.1^c^ | 0.7±0.1^b^ | 1.2±0.3^ab^ | 98.6±11.2^a^ | 2.9±0.6^ab^ | 7.4±0.7^a^ | 0.3±0.1^abc^ |
|  | 0.42%, 0.28% | 23.8±0.6^a^ | 0.8±0.1^ab^ | 61.8±16.5^d^ | 0.3±0^a^ | 0.5±0^abc^ | 0.7±0^b^ | 1.2±0.2^b^ | 107.6±6.8^a^ | 3.2±0.2^ab^ | 7.5±0.7^a^ | 0.4±0.1^a^ |
| T4 (KA:SW 40:60) | 0%, 0% | 16.3±5^a^ | 0.8±0^ab^ | 66.9±6.9^d^ | 0.2±0^a^ | 0.5±0^bc^ | 0.8±0^a^ | 1.6±0.2^a^ | 97.6±13^a^ | 2.7±0.3^b^ | 6.2±2.4^a^ | 0.3±0.1^abc^ |
|  | 0.14%, 0.21% | 19.7±3.1^a^ | 0.8±0^ab^ | 71±8^bcd^ | 0.2±0^a^ | 0.6±0^abc^ | 0.8±0.1^ab^ | 1.4±0.3^ab^ | 106.2±12.1^a^ | 3±0.5^ab^ | 6.9±1.9^a^ | 0.4±0.1^ab^ |
|  | 0.28%, 0.42% | 22.1±3.3^a^ | 0.8±0^ab^ | 74.6±6.4^bcd^ | 0.3±0.1^a^ | 0.6±0.1^abc^ | 0.8±0^ab^ | 1.4±0.1^ab^ | 114.8±23^a^ | 3.4±0.7^ab^ | 6.8±1.9^a^ | 0.4±0.1^ab^ |
| T5 (KA:SW 20:80) | 0%, 0% | 22.9±3.5^a^ | 0.9±0^a^ | 93.7±10.3^ab^ | 0.3±0^a^ | 0.6±0^a^ | 0.8±0^ab^ | 1.4±0.1^ab^ | 122.4±4.2^a^ | 3.5±0.7^ab^ | 6.8±2.1^a^ | 0.3±0.1^abc^ |
|  | 0.07%, 0.28% | 23.1±1.5^a^ | 0.8±0.1^ab^ | 74.5±6.9^bcd^ | 0.3±0^a^ | 0.6±0^abc^ | 0.8±0^ab^ | 1.5±0.2^ab^ | 111.5±2^a^ | 3.1±0.2^ab^ | 7.4±0.3^a^ | 0.3±0.1^abc^ |
|  | 0.14%, 0.56% | 22.8±1.3^a^ | 0.8±0.1^ab^ | 76.7±6.2^bcd^ | 0.3±0^a^ | 0.6±0^abc^ | 0.8±0^ab^ | 1.4±0.1^ab^ | 112.5±3.6^a^ | 3.1±0.2^ab^ | 7.4±0.3^a^ | 0.3±0.1^abc^ |
| T6 (KA:SW 0:100) | 0%, 0% | 23.2±3.1^a^ | 0.8±0.1^ab^ | 53.3±8.9^d^ | 0.2±0^a^ | 0.5±0^bc^ | 0.8±0^ab^ | 1.3±0^ab^ | 14.3±12.5^a^ | 3±0.3^ab^ | 7.8±0.6^a^ | 0.2±0.1^bc^ |
|  | 0%, 0.35% | 24.1±4.4^a^ | 0.7±0^ab^ | 57.3±9.9^d^ | 0.3±0^a^ | 0.6±0.1^abc^ | 0.7±0^ab^ | 1.3±0.1^ab^ | 111.7±7.2^a^ | 3.1±0.4^ab^ | 7.7±0.5^a^ | 0.3±0^abc^ |
|  | 0%, 0.7% | 20.8±2.8^a^ | 0.8±0^ab^ | 65.9±6.3^d^ | 0.2±0^a^ | 0.5±0.1^c^ | 0.8±0^ab^ | 1.4±0.1^ab^ | 97.8±17.7^a^ | 2.8±0.3^b^ | 7.5±0.2^a^ | 0.2±0^c^ |
| P-value |  | ns | ns | 0.0055 | ns | 0.3138 | ns | ns | ns | ns | ns | ns |

Values represented are mean of 5 replicates in pot trial; values followed by different alphabets in the rows are significantly different at P < 0.05 using DMRT. PR, Photosynthesis rate; Ci, Intercellular CO_2_ concentration; øPS_2_, Quantum yield of PS II electron transport; qP, Photochemical quenching; qN, Non-photochemical quenching; NPQ, Non-photochemical fluorescence quenching; ETR, Photosynthetic electron transport rate; TR, Transpiration rate; WUE, Water use efficiency; Ci /Ca, Intercellular CO_2_ conc./Ambient CO_2_
