# Supplementary material for: Underpinning beneficial maize response to application of minimally processed homogenates of red and brown seaweeds
Source: Front Plant Sci. 2023 Nov 30;14:1273355. doi: 10.3389/fpls.2023.1273355 (PMC10723902; doi:10.3389/fpls.2023.1273355)
Supplement: Supplementary file 1 [file DataSheet_1.zip › Supplementary Table 5.DOCX]

**Table 5A: Saturated fatty acids composition in maize leaf**

| **Treatments** | **Conc. of MPHs** | **Capric acid methyl ester**  **(µg g^-1^)** | **Methyl undecanoate**  **(µg/gm)** | **Lauric acid**  **(µg/gm)** | **Myristic acid**  **(µg/gm)** | **Palmitic acid**  **(µg/gm)** | **Margaric acid methyl ester (µg/gm)** | **Methyl stearate (µg/gm)** | **TSFA** |
| --- | --- | --- | --- | --- | --- | --- | --- | --- | --- |
|  |  | **C10:0** | **C11:0** | **C12:0** | **C14:0** | **C16:0** | **C17:0** | **C18:0** |  |
| T6 (KA:SW 0:100) | 0% | 179.54±64.6^c^ | 94.03±3.4^ab^ | 73.96±17.0^b^ | 227.35±17.5^a^ | 913.0±40.2^c^ | 82.97±7.5^cd^ | 79.63±17.5^b^ | 1650.48 |
|  | 0.35% | 275.89±89.9^ab^ | 145.31±57.1^a^ | 154.11±67.2^a^ | 245.58±137.0^a^ | 1676.47±241.4^ab^ | 187.77±39.2^abcd^ | 161.73±56.0^ab^ | 2846.86 |
|  | 0.7% | 268.25±69.5^abc^ | 143.03±37.5^a^ | 157.37±32.8^a^ | 280.90±91.3^a^ | 2020.11±457.3^a^ | 250.12±94.5^a^ | 176.86±63.8^a^ | 3028.39 |
| P-value |  | 0.5689 | ns | 0.0670 | ns | 0.0061 | 0.0898 | 0.4029 |  |

TSFA=Total Saturated Fatty acids

**Table 5B**: **Unsaturated fatty acids composition in maize leaf**

| **Treatments** | **Conc. of MPHs** | **MUFA(µg g^-1^)** | **PUFA (µg g^-1^)** | **Palmitoleate**  **(µg g^-1^)** | **gamma.-Linolenic acid, methyl ester**  **(µg g^-1^)** | **9,12-Octadecadienoic acid, methyl ester (µg g^-1^)** | **Eladic acid, methyl ester (µg g^-1^)** | **Arachidonic acid methyl ester**  **(µg g^-1^)** | **Methyl nervonate (µg g^-1^)** |
| --- | --- | --- | --- | --- | --- | --- | --- | --- | --- |
|  |  |  |  | **C16:1** | **C18:3** | **C18:2** | **C18:1** | **C24:4** | **C24:01** |
| T6 (KA:SW 0:100) | 0% | 205.96 | 277.28 | 82.47±40.2^a^ | 38.96±3.9^b^ | 201.49±102.1^d^ | 100.41±50.5^c^ | 36.83±17.1^a^ | 23.08±2.6^bc^ |
|  | 0.35% | 424.65 | 692.31 | 82.55±20.1^a^ | 0.0 | 618.45±18.5^abcd^ | 302.21±58.5^abc^ | 73.86±36.5^a^ | 39.89±5.7^ab^ |
|  | 0.7% | 471.58 | 865.26 | 0.0 | 0.0 | 791.10±388.7^a^ | 421.30±251.2^ab^ | 74.16±24.6^a^ | 50.28±11.5^a^ |
| P-value |  |  |  | Ns | Ns | 0.1506 | 0.2144 | ns | 0.2390 |

MUFA=Mono Unsaturated Fatty acids; PUFA=Poly Unsaturated Fatty acids
