# Supplementary material for: Underpinning beneficial maize response to application of minimally processed homogenates of red and brown seaweeds
Source: Front Plant Sci. 2023 Nov 30;14:1273355. doi: 10.3389/fpls.2023.1273355 (PMC10723902; doi:10.3389/fpls.2023.1273355)
Supplement: Supplementary file 1 [file DataSheet_1.zip › Supplementary Table 6.DOCX]

**Supplementary Table 6: The experimental site's meteorological data**

|  | **Temperature (°C) mean maximum** | **Temperature (°C) mean**  **minimum** | **Relative Humidity**  **(%)** | **Wind Speed (m/s)** | **Solar Radiation**  **(W/m^2^)** | **Rainfall**  **(mm)** |
| --- | --- | --- | --- | --- | --- | --- |
| **2019-20** |  |  |  |  |  |  |
| November-19 | 31.5 | 21.8 | 49.0 | 1.82 | 157 | 3.00 |
| December-19 | 28.3 | 18.0 | 49.0 | 2.88 | 112 | 3.00 |
| January-20 | 26.9 | 14.0 | 40.4 | 3.04 | 99 | 0.00 |
| February-20 | 30.0 | 16.0 | 35.5 | 2.74 | 173 | 0.00 |
| March-20 | 33.3 | 16.0 | 34.1 | 2.98 | 202 | 0.18 |
