# Supplementary material for: Underpinning beneficial maize response to application of minimally processed homogenates of red and brown seaweeds
Source: Front Plant Sci. 2023 Nov 30;14:1273355. doi: 10.3389/fpls.2023.1273355 (PMC10723902; doi:10.3389/fpls.2023.1273355)
Supplement: Supplementary file 1 [file DataSheet_1.zip › Supplementary Table 7.DOCX]

**Supplementary Table 7: Treatment description of experiment**

| **Treatments No.** | **Treatments define** | **Foliar Application Concentration** |
| --- | --- | --- |
| T1 | Water (Control) | 0% |
| T2 | (100:0); MPHs of KA: SW | 0.35% |
| T3 | (100:0); MPHs of KA: SW | 0.7% |
| T4 | Water (Control) | 0% |
| T5 | (80:20); MPHs of KA: SW | 0.35% |
| T6 | (80:20); MPHs of KA: SW | 0.7% |
| T7 | Water (Control) | 0% |
| T8 | (60:40); MPHs of KA: SW | 0.35% |
| T9 | (60:40); MPHs of KA: SW | 0.7% |
| T10 | Water (Control) | 0% |
| T11 | (40:60); MPHs of KA: SW | 0.35% |
| T12 | (40:60); MPHs of KA: SW | 0.7% |
| T13 | Water (Control) | 0% |
| T14 | (20:80); MPHs of KA: SW | 0.35% |
| T15 | (20:80); MPHs of KA: SW | 0.7% |
| T16 | Water (Control) | 0% |
| T17 | (0:100); MPHs of KA: SW | 0.35% |
| T18 | (0:100); MPHs of KA: SW | 0.7% |

KA=*Kappaphycus alvarezii*; SW=*Sargassum wightii*; MPH=Minimally Processed Homogenate
