# Supplementary material for: Underpinning beneficial maize response to application of minimally processed homogenates of red and brown seaweeds
Source: Front Plant Sci. 2023 Nov 30;14:1273355. doi: 10.3389/fpls.2023.1273355 (PMC10723902; doi:10.3389/fpls.2023.1273355)
Supplement: Supplementary file 1 [file DataSheet_1.zip › Supplementary captions.docx]

**Figure captions**

**Figure 1:** Snapshots of bilayer membranes with different concentrations of MPHs treatment (T6- 0:100 KA:SW): (a) 0% treatment (Control), (b) 0.35% treatment, and (c) 0.7% treatment.

**Figure 2:** Q-TOF-HRMS profiles with major peaks in the TICs (A) Positive ESI (B) Negative ESI of MPH of KA

**Figure 3:** (**A)** Positive electro-spray ionization MFE-MS spectrum of all screened annotated compounds in MPH of KA. **(B)** Negative electro-spray ionization MFE-MS spectrum of all screened annotated compounds in MPH of KA

**Figure 4:** Q-TOF-HRMS profiles with major peaks in the TICs (A) Positive ESI (B) Negative ESI of MPH of SW

**Figure 5:** (**A)** Positive electro-spray ionization MFE-MS spectrum of all screened annotated compounds in MPH of SW. **(B)** Negative electro-spray ionization MFE-MS spectrum of all screened annotated compounds in MPH of SW

**Figure 6:** Effects of MPHs on maize leaf metabolites concentrations

**Figure 7:** Effects of MPHs on antioxidant enzymes and total ROS in maize leaf

**Supplementary Figure 1:** Positive electro-spray ionization MS/MS spectrum of all screened annotated compounds in MPH of KA

**Supplementary Figure 2:** Negative electro-spray ionization MS/MS spectrum of all screened annotated compounds in MPH of KA

**Supplementary Figure 3:** Positive electro-spray ionization MS/MS spectrum of all screened annotated compounds in MPH of SW

**Supplementary Figure 4:** Negative electro-spray ionization MS/MS spectrum of all screened annotated compounds in MPH of SW

**Supplementary Figure 5:** (a) Area per lipid molecule (AL), (b) membrane bilayer thickness

**Supplementary Figure 6:** Membrane order parameters
