# Supplementary material for: Underpinning beneficial maize response to application of minimally processed homogenates of red and brown seaweeds
Source: Front Plant Sci. 2023 Nov 30;14:1273355. doi: 10.3389/fpls.2023.1273355 (PMC10723902; doi:10.3389/fpls.2023.1273355)
Supplement: Supplementary file 1 [file DataSheet_1.zip › Supplementary Table 3.docx]

**Supplementary Table 3: Bioactivities of all screened annotated compounds in MPHs of *Kappaphycus alvarezii* and *Sargassum wightii***

| **Compound name** | **Bioactivities** | **Reference** |
| --- | --- | --- |
| Retronecine | Anti-fungal activity against plant pathogens | (Pedras and Yaya, 2015) |
| Pirbuterol | It is used beta-adrenergic drugs. | (Bethesda, 2017) |
| Isocarbostyril | Anticancer activity. | (Bingham et al., 2019) |
| Fasoracetam | - |  |
| Sinapoylputrescine | It is also identified in Rice. | (Dong et al., 2015) |
| 4-Dodecylbenzenesulfonic acid | - |  |
| Tyrosyl-Glycine | Peptides secreted as signal molecules to trigger cell-to-cell signalling are indispensable for plant growth and defense processes. | (Hu et al., 2018) |
| L,L-Cyclo(leucylprolyl) | Anti-bacterial activity. | (Zaher et al., 2015) |
| Hexyl 2-furoate | It is identified in medicinal plants and has antioxidant activity. | (Zannou and Koca, 2019) |
| DG(20:5(5Z,8Z,11Z,14Z,17Z)/ 20:2(11Z,14Z)/0:0) | It does indeed act as a signalling molecule during plant development and in response to certain environmental stimuli | (Dong et al., 2012) |
| Sulfoglycolithocholate | Sulfoglycolithocholate, a secondary bile salts metabolite, a plant-derived antimicrobial activity. | (Khosravi et al., 2014) |
| 1-Phosphatidyl-1D-myo- inositol 3-phosphate | It also found in *Kappaphycus alvarezii* extract. Involved in gene expression modulation, hormonal signalling, guard cell  movement, stress response, disease resistance, and membrane and cell wall biogenesis. It has use in surfactants, emulsifiers, food additives, nutritional supplements, membrane stabilizers. | (Vaghela et al., 2022) |
| 2,3-Dihydro-6-methyl-5- propanoyl-1H-pyrrolizine | - |  |
| Protorifamycin I | It is enriched in the biosynthesis of ansamycins and biosynthesis of antibiotics. | (Nuli et al., 2019) |
| Candoxatrilat | - | - |
| Anapheline | - | - |
| 12-(2,3-Dihydroxycyclopentyl)- 2-dodecanone | It is found in *Ruta graveolens* Essential Oil and play a vital role defence strategy against herbivory. | (Perera A.G.W.U. and S.D.M, 2017) |
| Idoxuridine | Anti-viral compound. | (Vishwanadham et al., 2013) |
| Chlorfenvinphos | Chlorfenvinphos (CFVF) belongs to organophosphate insecticides and was commonly used to control household pests, such as flies, fleas, and mites. | (Lukaszewicz-Hussain, 2008) |
| 1,1'-Bis(2-hydroxy-3- methylcarbazole) | It is identified in herbs and spices. | FDB020592 |
| 5-Decanoyl-2-nonylpyridine | It is identified in the roots of *Gymnotheca* *chinensis*. | Xiao, 2016 |
| 2,4,14-Eicosatrienoic acid isobutylamide | - |  |
| Octadecyl fumarate | - |  |
| 4,4'-(2-Methylpropylidene) bisphenol | - |  |
| Simetryn | Simetryn is used to control broadleaf weeds in mixtures with other herbicides that are active against grasses. | (Müller, 2008) |
| Guaiazulene | Antiallergic, antibacterial, and anti-inflammatory activities. | (Bakun et al., 2021) |
| Sethoxydim | Used as a post-emergence herbicide. | (Campbell and Penner, 1985) |
| 2-Deoxystreptidine | Antimicrobial activity. | US6759523B2 |
| Tragopogonsaponin B | It is identified and isolated from *Tragopogon porrifolius* L. | (Warashina et al., 1991) |
| Carteolol | It is used for pharmaceutical sectors. | (Stewart and Castelli, 1996) |
| Hydroquinidine | Antiarrhythmic activity. | (Angelini et al., 2020) |
| Cucurbitacin E | Cucurbitacin E has been reported to possess anti-inflammatory, anti-angiogenic, immunomodulatory, cytotoxic, cytostatic and hepatoprotective properties in both in vitro and in vivo model | (Abdelwahab et al., 2011; Attard and Martinoli, 2015; Arjaibi et al., 2017) |
| Retronecine | Anti-fungal activity against plant pathogens | (Pedras and Yaya, 2015) |
| Metanephrine | Metanephrines in biological fluids is important for clinical screening of pheochromocytoma/paraganglioma and diagnosis of overtraining syndrome in athletes. | (Dikunets et al., 2020) |
| d-Dethiobiotin | Dethiobiotin is the true precursor to biotin. | (Lezius et al., 1963) |
| Trihomomethionine | Trihomomethionine that is used in the biosynthesis of C4 and C5 glucosinolates, which mitigates abiotic and biotic stress in *Brassicaceae*. | (Zang et al., 2008; Chowdhury, 2022) |
| Butyl 2-aminobenzoate | It is also called Butyl anthranilate. It is used as a flavouring and fragrance agent and exhibits insect repellent properties. It has a role as a flavoring agent, an insect repellent, a fragrance, and a plant metabolite. | PCID24433  (Ida Emilie Steinmark, 2015) |
| Salicylanilide | Salicylanilides are a very large group of compounds, originally developed as fungicides for topical use and as antimicrobial agents in soaps. | (Kraushaar, 1954) |
| Isocarbostyril | Anticancer activity. | (Bingham et al., 2019) |
| Fasoracetam | - |  |
| 2,4'-Diphenyldiamine | - |  |
| Pirbuterol | It is used beta-adrenergic drugs. | (Bethesda, 2017) |
| L,L-Cyclo(leucylprolyl | Anti-bacterial activity. | (Zaher et al., 2015) |
| Mycinamicin IV | They were first discovered in 1980 from Micromonospora griseorubida and were shown to inhibit the growth of Gram-positive bacteria, including drug-resistant *Staphylococcus aureus* (SA) strains. | (Satoi et al., 1980) |
| 1-Phosphatidyl-1D-myo-inositol 3-phosphate | It also found in *Kappaphycus alvarezii* extract. Involved in gene expression modulation, hormonal signalling, guard cell  movement, stress response, disease resistance, and membrane and cell wall biogenesis. It has use in surfactants, emulsifiers, food additives, nutritional supplements, membrane stabilizers. | (Vaghela et al., 2022) |
| Istamycin A1 | Antimicrobial activity. | (Yogesh Murti and Tarun Agrawal, 2010) |
| Fleroxacin | Bactericidal activity. | (Naber, 1996) |
| Dibenzo[a,e]pyrene | - |  |
| 16-Oxo-palmitate | Major fatty acid in plant membrane. | (Deas et al., 1974) |
| 3b,17a,21-Trihydroxypregnenone | It is an intermediate in C21-Steroid hormone metabolism. | HMDB0006762 |
| 6-Oxabicyclo [3.1.0] hexane-2-undecanoic acid methyl ester | - |  |
| 12-(2,3-Dihydroxycyclopentyl)-2-dodecanone | It is also known is 2-dodecanone. It exhibited potent insecticidal and repellent activities against the *Tribolium castaneum, Lasioderma serricorne*, and *Liposcelis bostrychophila* adult insects. | (Wang et al., 2019) |
| Chlorfenvinphos | Chlorfenvinphos (CFVF) belongs to organophosphate insecticides and was commonly used to control household pests, such as flies, fleas and mites. | (Lukaszewicz-Hussain, 2008) |
| Idoxuridine | Anti-viral compound. | (Vishwanadham et al., 2013) |
| (S)-Menthone 8-thioacetate | - |  |
| Sethoxydim | Sethoxydim is a postemergence herbicide used to control grass weeds. | (Weber et al., 1988) |
| 2-Deoxystreptidine | - |  |
| Oxybutynin | Oxybutynin is an anticholinergic medication indicated in patients with overactive bladder or symptoms of detrusor over activity, including urinary frequency and urgency. | (Jennifer et al., 2022) |
| (1R,2S,4R,5S)-2,5-Fenchanediol 2-O-b-D-glucoside | - |  |
| Ethamoxytriphetol | - |  |
| Aspidospermine | It is antiparasitic compound extracted from *Aspidosperma polyneuron.* | (Coatti et al., 2016) |
| Pithecolobine | - |  |
| Bryodulcosigenin | Bryodulcosigenin (BDG) a cucurbitane-type triterpenoid has been isolated from the roots of *Bryonia dioca* and possesses marked anti-inflammatory effects. | (Li et al., 2022) |
| 10-Deoxymethymycin | - |  |
| Atenolol | It found in root and leaves in radish, lamb’s lettuce and spinach. | (Kodešová et al., 2019) |
| Practolol | - |  |

References:

Abdelwahab, S. I., Hassan, L. E. A., Sirat, H. M., Yagi, S. M. A., Koko, W. S., Mohan, S., et al. (2011). Anti-inflammatory activities of cucurbitacin E isolated from Citrullus lanatus var. citroides: Role of reactive nitrogen species and cyclooxygenase enzyme inhibition. *Fitoterapia* 82, 1190–1197. doi: 10.1016/j.fitote.2011.08.002.

Angelini, F., Pourshayesteh, S., Gastino, E., Cingolani, M. ., Castagno, D., Cerrato, N., et al. (2020). Long-term efficacy and safety of hydroquinidine in patients with Brugada syndrome. *Eur. Heart J.* 41. doi: 10.1093/ehjci/ehaa946.0397.

Arjaibi, H. M., Ahmed, M. S., and Halaweish, F. T. (2017). Mechanistic investigation of hepato-protective potential for cucurbitacins. *Med. Chem. Res.* 26, 1567–1573. doi: 10.1007/s00044-017-1872-3.

Attard, E., and Martinoli, M.-G. (2015). Cucurbitacin E, An Experimental Lead Triterpenoid with Anticancer, Immunomodulatory and Novel Effects Against Degenerative Diseases. A Mini-Review. *Curr. Top. Med. Chem.* 15, 1708–1713. doi: 10.2174/1568026615666150427121331.

Bakun, P., Czarczynska-Goslinska, B., Goslinski, T., and Lijewski, S. (2021). In vitro and in vivo biological activities of azulene derivatives with potential applications in medicine. *Med. Chem. Res.* 30, 834–846. doi: 10.1007/s00044-021-02701-0.

Bethesda (2017). *LiverTox: Clinical and Research Information on Drug-Induced Liver Injury*. , ed. National Institute of Diabetes and Digestive and Kidney Diseases National Library of Medicine. https://www.ncbi.nlm.nih.gov/books/NBK548685/.

Bingham, T. W., Hernandez, L. W., Olson, D. G., Svec, R. L., Hergenrother, P. J., and Sarlah, D. (2019). Enantioselective Synthesis of Isocarbostyril Alkaloids and Analogs Using Catalytic Dearomative Functionalization of Benzene. *J. Am. Chem. Soc.* 141, 657–670. doi: 10.1021/jacs.8b12123.

Campbell, J. R., and Penner, D. (1985). Abiotic Transformations of Sethoxydim. *Weed Sci.* 33, 435–439. doi: 10.1017/S0043174500082606.

Chowdhury, P. (2022). “Glucosinolates and Its Role in Mitigating Abiotic and Biotic Stress in Brassicaceae,” in doi: 10.5772/intechopen.102367.

Coatti, G. C., Marcarini, J. C., Sartori, D., Fidelis, Q. C., Ferreira, D. T., and Mantovani, M. S. (2016). Cytotoxicity, genotoxicity and mechanism of action (via gene expression analysis) of the indole alkaloid aspidospermine (antiparasitic) extracted from Aspidosperma polyneuron in HepG2 cells. *Cytotechnology* 68, 1161–1170. doi: 10.1007/s10616-015-9874-9.

Deas, A. H. B., Baker, E. A., and Holloway, P. J. (1974). Identification of 16-hydroxyoxohexadecanoic acid monomers in plant cutins. *Phytochemistry* 13, 1901–1905. doi: 10.1016/0031-9422(74)85111-3.

Dikunets, M., Dudko, G., Glagovsky, P., and Mamedov, I. (2020). Simultaneous Quantification of Plasma Catecholamines and Metanephrines by LC‑MS/MS. *J. Braz. Chem. Soc.* doi: 10.21577/0103-5053.20200033.

Dong, W., Lv, H., Xia, G., and Wang, M. (2012). Does diacylglycerol serve as a signaling molecule in plants? *Plant Signal. Behav.* 7, 472–475. doi: 10.4161/psb.19644.

Dong, X., Gao, Y., Chen, W., Wang, W., Gong, L., Liu, X., et al. (2015). Spatiotemporal Distribution of Phenolamides and the Genetics of Natural Variation of Hydroxycinnamoyl Spermidine in Rice. *Mol. Plant* 8, 111–121. doi: 10.1016/j.molp.2014.11.003.

Hu, Z., Zhang, H., and Shi, K. (2018). Plant peptides in plant defense responses. *Plant Signal. Behav.*, 1–5. doi: 10.1080/15592324.2018.1475175.

Ida Emilie Steinmark (2015). News Fruity alternative to toxic insecticides. *Chemistryworld*.

Jennifer Dwyer; Sean M. Tafuri; Chad A. LaGrange. (2022). Oxybutynin. *StatPearls*.

Khosravi, Y., Dieye, Y., Loke, M. F., Goh, K. L., and Vadivelu, J. (2014). Streptococcus mitis Induces Conversion of Helicobacter pylori to Coccoid Cells during Co-Culture In Vitro. *PLoS One* 9, e112214. doi: 10.1371/journal.pone.0112214.

Kodešová, R., Klement, A., Golovko, O., Fér, M., Nikodem, A., Kočárek, M., et al. (2019). Root uptake of atenolol, sulfamethoxazole and carbamazepine, and their transformation in three soils and four plants. *Environ. Sci. Pollut. Res.* 26, 9876–9891. doi: 10.1007/s11356-019-04333-9.

kraushaar, A. (1954). [Chemotherapeutic activity of halogenated salicylanilides in relation to their constitution]. *Arzneimittelforschung.* 4, 548–51.

Lezius, A., Ringelmann, E., And Lynen, F. (1963). [On the biochemical function of biotin. IV. The biosynthesis of biotin]. *Biochem. Z.* 336, 510–25. German. PMID: 13930373.

Li, R., Chen, C., Liu, B., Shi, W., Shimizu, K., and Zhang, C. (2022). Bryodulcosigenin a natural cucurbitane-type triterpenoid attenuates dextran sulfate sodium (DSS)-induced colitis in mice. *Phytomedicine* 94, 153814. doi: 10.1016/j.phymed.2021.153814.

Lukaszewicz-Hussain, A. (2008). Subchronic intoxication with chlorfenvinphos, an organophosphate insecticide, affects rat brain antioxidative enzymes and glutathione level. *Food Chem. Toxicol.* 46, 82–86. doi: 10.1016/j.fct.2007.06.038.

Müller, G. (2008). “History of the Discovery and Development of Triazine Herbicides,” in *The Triazine Herbicides* (Elsevier), 13–29. doi: 10.1016/B978-044451167-6.50005-2.

Naber, K. G. (1996). Fleroxacin Overview. *Chemotherapy* 42, 1–9. doi: 10.1159/000239485.

Nuli, R., Azhati, J., Cai, J., Kadeer, A., Zhang, B., and Mohemaiti, P. (2019). Metagenomics and Faecal Metabolomics Integrative Analysis towards the Impaired Glucose Regulation and Type 2 Diabetes in Uyghur-Related Omics. *J. Diabetes Res.* 2019, 1–15. doi: 10.1155/2019/2893041.

Pedras, M. S. C., and Yaya, E. E. (2015). Plant chemical defenses: are all constitutive antimicrobial metabolites phytoanticipins? *Nat. Prod. Commun.* 10, 209–18.

Perera A.G.W.U., K. M. M. S. C., and S.D.M, C. (2017). Bio-efficacy of Ruta graveolens Essential Oil and Its Long Chain Aliphatic 2-Methlyl Ketone Constituents on the Egg Hatchability of Corcyra cephalonica (Stainton). in *International Forestry and Environment Symposium* (Department of Forestry and Environmental Science, University of Sri Jayewardenepura, Sri Lanka: Session VIII – Environmental Engineering and Green Technology), 127.

Satoi, S., Muto, N., Hayashi, M., Fujii, T., And Otani, M. (1980). Mycinamicins, new macrolide antibiotics. I. Taxonomy, production, isolation, characterization and properties. *J. Antibiot. (Tokyo).* 33, 364–376. doi: 10.7164/antibiotics.33.364.

Shikha Singh, M. K. S., Pal, S. K., Thakur, R., Zodape, S. T., and And Ghosh, A. (2015). Use of seaweed sap for sustainable productivity of maize. Bioscan 10, 1349–1355

Stewart, W. C., and Castelli, W. P. (1996). Systemic side effects of topical beta-adrenergic blockers. *Clin. Cardiol.* 19, 691–697. doi: 10.1002/clc.4960190904.

Vaghela, P., Das, A. K., Trivedi, K., Anand, K. G. V., Shinde, P., and Ghosh, A. (2022). Characterization and metabolomics profiling of Kappaphycus alvarezii seaweed extract. *Algal Res.* 66, 102774. doi: 10.1016/j.algal.2022.102774.

Vishwanadham Yerragunta, Prathima Patil, V.Anusha, T. K., and D.Suman, T. S. (2013). Pyrimidine and Its Biological Activity: A Review. *PharmaTutor Mag.* 1, 39–44.

Wang, Y., Zhang, L.-T., Feng, Y.-X., Guo, S.-S., Pang, X., Zhang, D., et al. (2019). Insecticidal and repellent efficacy against stored-product insects of oxygenated monoterpenes and 2-dodecanone of the essential oil from Zanthoxylum planispinum var. dintanensis. *Environ. Sci. Pollut. Res.* 26, 24988–24997. doi: 10.1007/s11356-019-05765-z.

Warashina, T., Miyase, T., And Ueno, A. (1991). Novel acylated saponins from Tragopogon porrifolius L. Isolation and the structures of tragopogonsaponins A-R. *Chem. Pharm. Bull.* 39, 388–396. doi: 10.1248/cpb.39.388.

Weber, A., Fischer, E., Branitz, H. S. von, and Lüttge, U. (1988). The Effects of the Herbicide Sethoxydim on Transport Processes in Sensitive and Tolerant Grass Species I. Effects on the Electrical Membrane Potential and Alanine Uptake. *Zeitschrift für Naturforsch. C* 43, 249–256. doi: 10.1515/znc-1988-3-416.

Yogesh Murti and Tarun Agrawal (2010). Marine derived pharmaceuticalsDevelopment of natural health products from marine biodiversity. *Int. J. ChemTech Res.* 2, 2198–2217.

Zaher, A. M., Moharram, A. M., Davis, R., Panizzi, P., Makboul, M. A., and Calderón, A. I. (2015). Characterisation of the metabolites of an antibacterial endophyte Botryodiplodia theobromae Pat. of Dracaena draco L. by LC–MS/MS. *Nat. Prod. Res.* 29, 2275–2281. doi: 10.1080/14786419.2015.1012715.

Zang, Y.-X., Kim, J.-H., Park, Y.-D., Kim, D.-H., and Hong, S.-B. (2008). Metabolic engineering of aliphatic glucosinolates in Chinese cabbage plants expressing Arabidopsis MAM1, CYP79F1, and CYP83A1. *BMB Rep.* 41, 472–478. doi: 10.5483/BMBRep.2008.41.6.472.

Zannou, O., and Koca, I. (2019). Aroma and Bioactive Compounds of Some Medicinal Plants’ Leaves Used as Traditional Tea in Benin Republic. *Turkish J. Sci. Rev.* 12, 16–25.
