# Supplementary figures and images for: Underpinning beneficial maize response to application of minimally processed homogenates of red and brown seaweeds
Source: Front Plant Sci. 2023 Nov 30;14:1273355. doi: 10.3389/fpls.2023.1273355 (PMC10723902; doi:10.3389/fpls.2023.1273355)

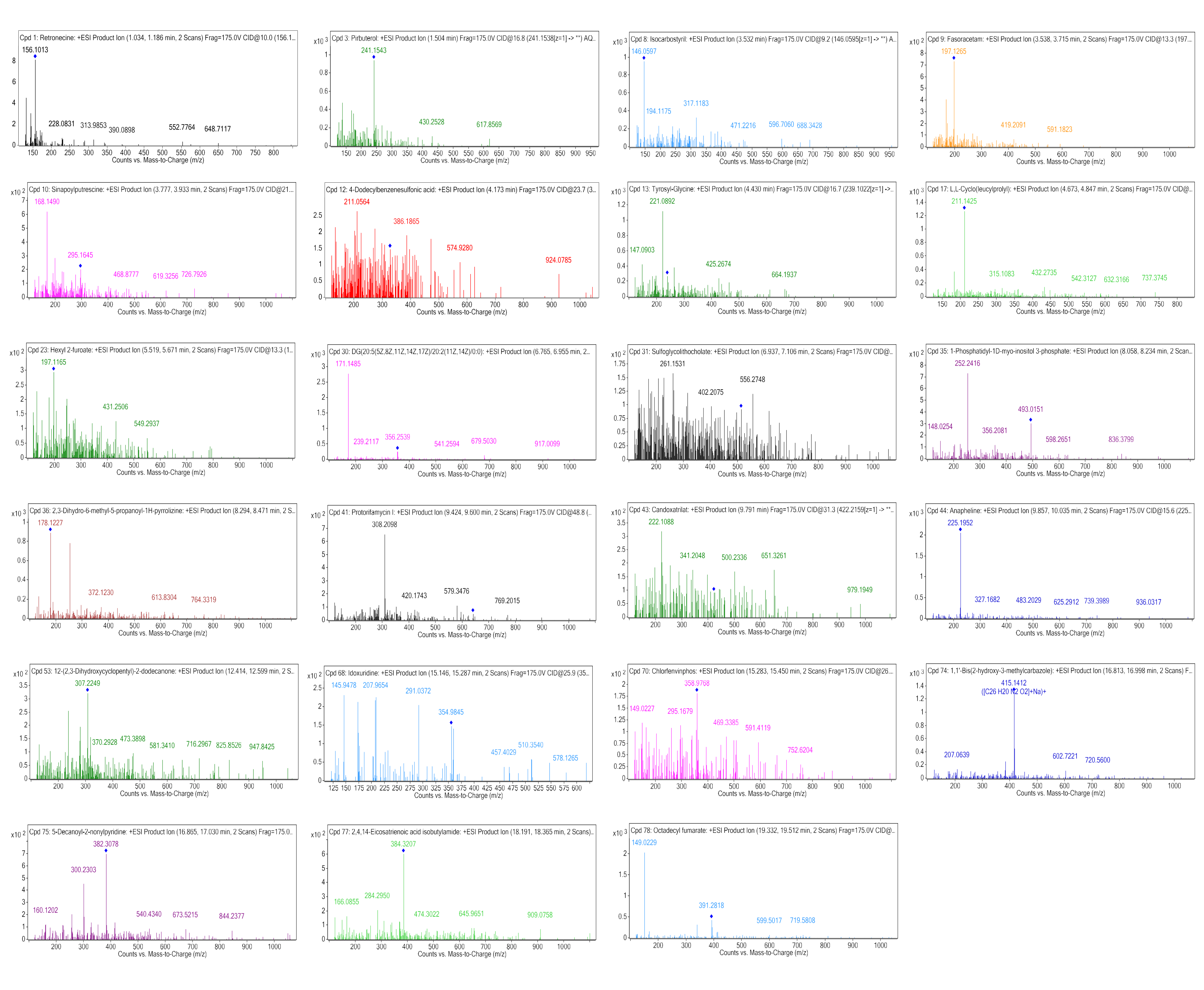

Supplement: Supplementary file 1 [file DataSheet_1.zip › Supplementary Figure 1.TIF]

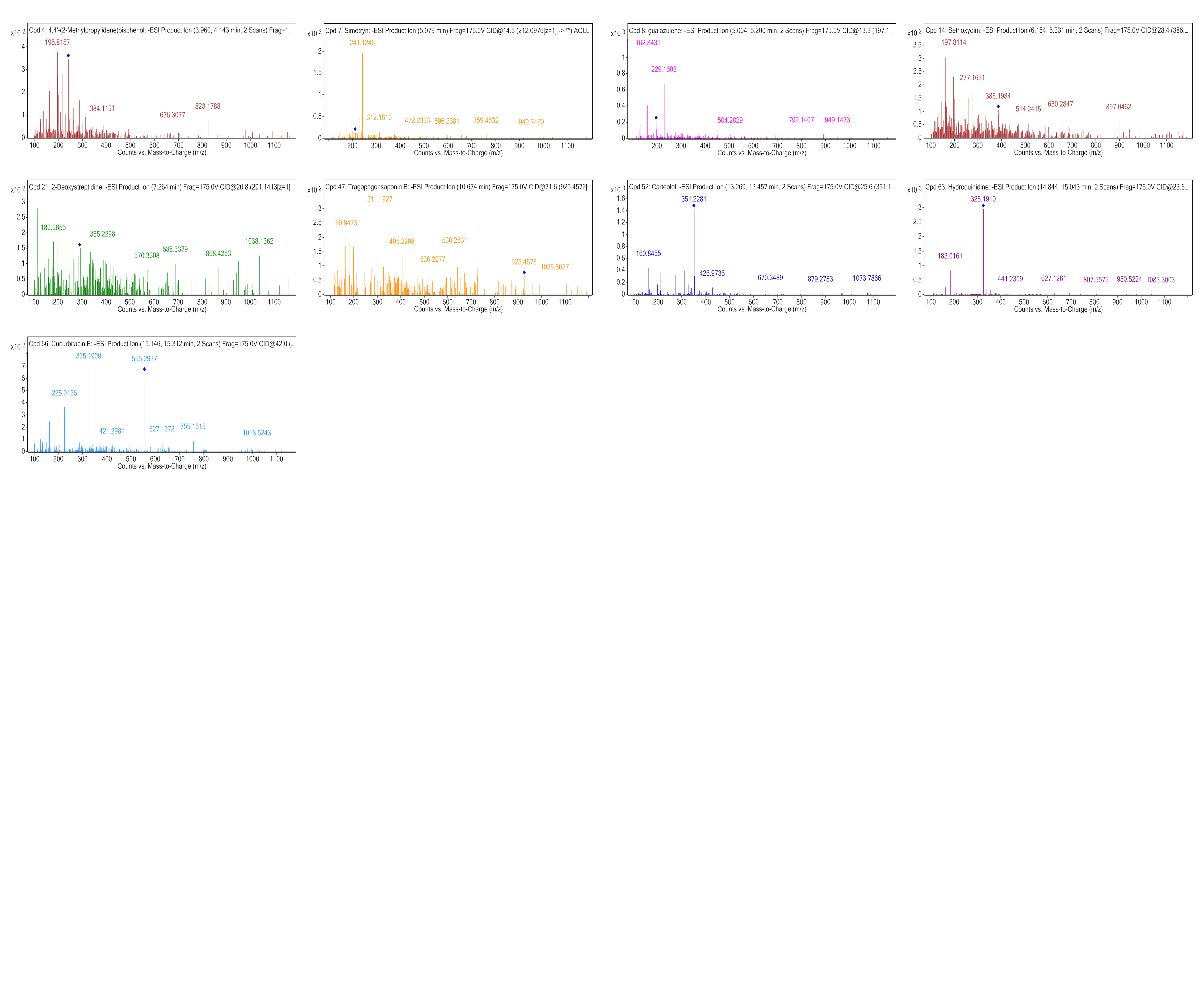

Supplement: Supplementary file 1 [file DataSheet_1.zip › Supplementary Figure 2.TIF]

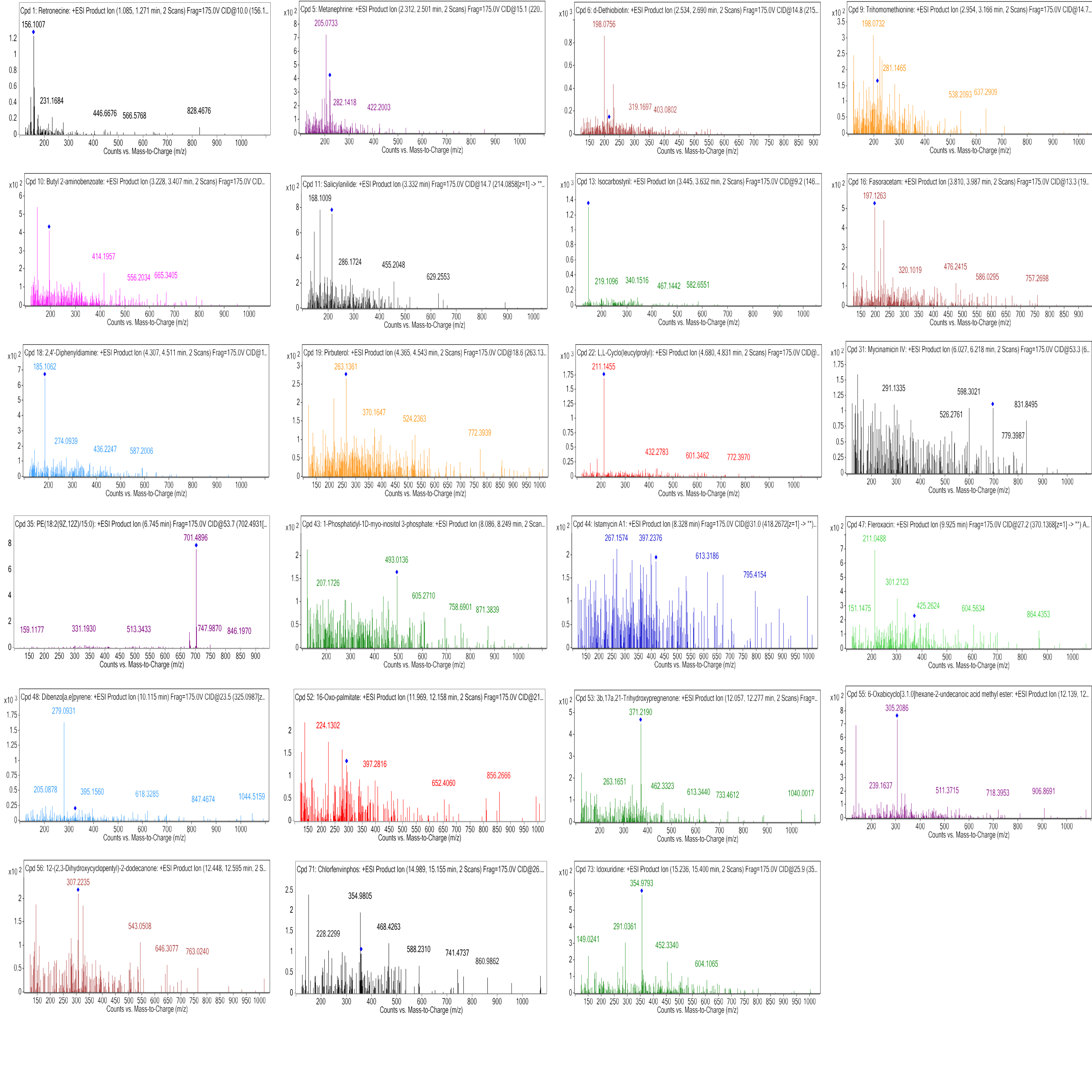

Supplement: Supplementary file 1 [file DataSheet_1.zip › Supplementary Figure 3.TIF]

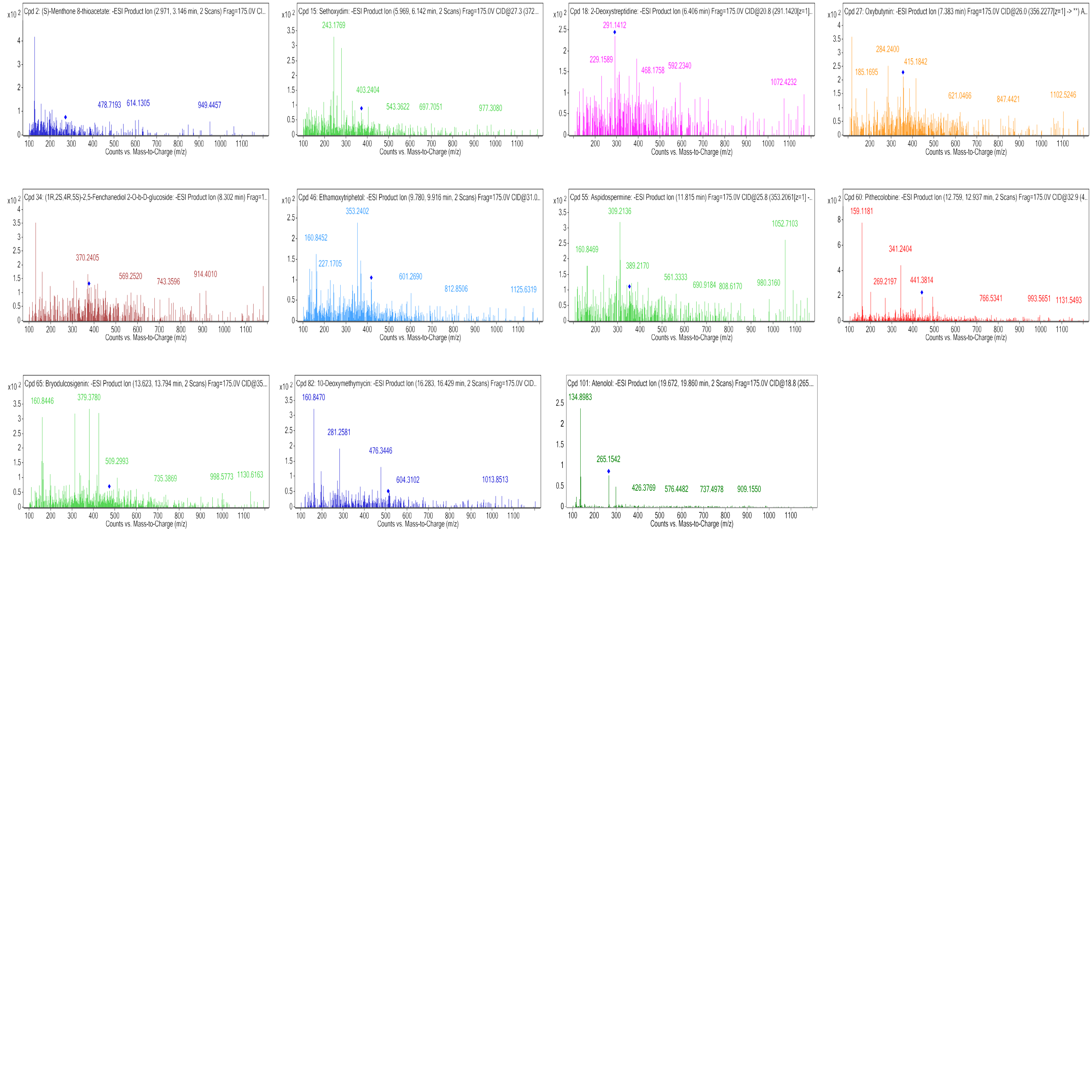

Supplement: Supplementary file 1 [file DataSheet_1.zip › Supplementary Figure 4.TIF]

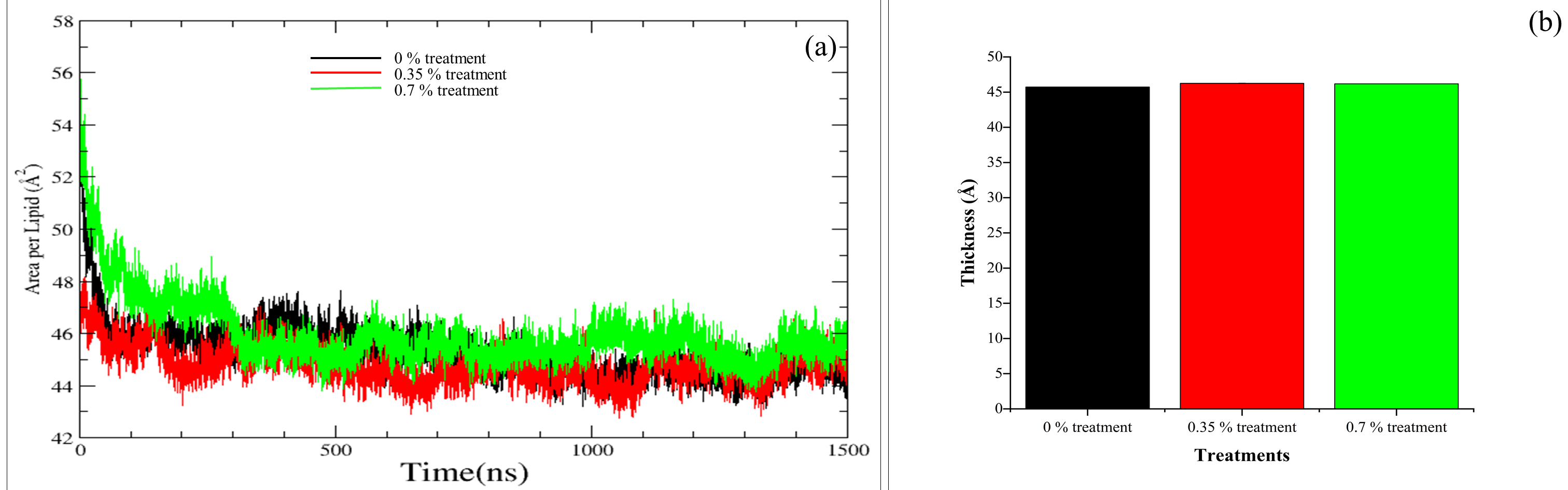

Supplement: Supplementary file 1 [file DataSheet_1.zip › Supplementary Figure 5.TIF]

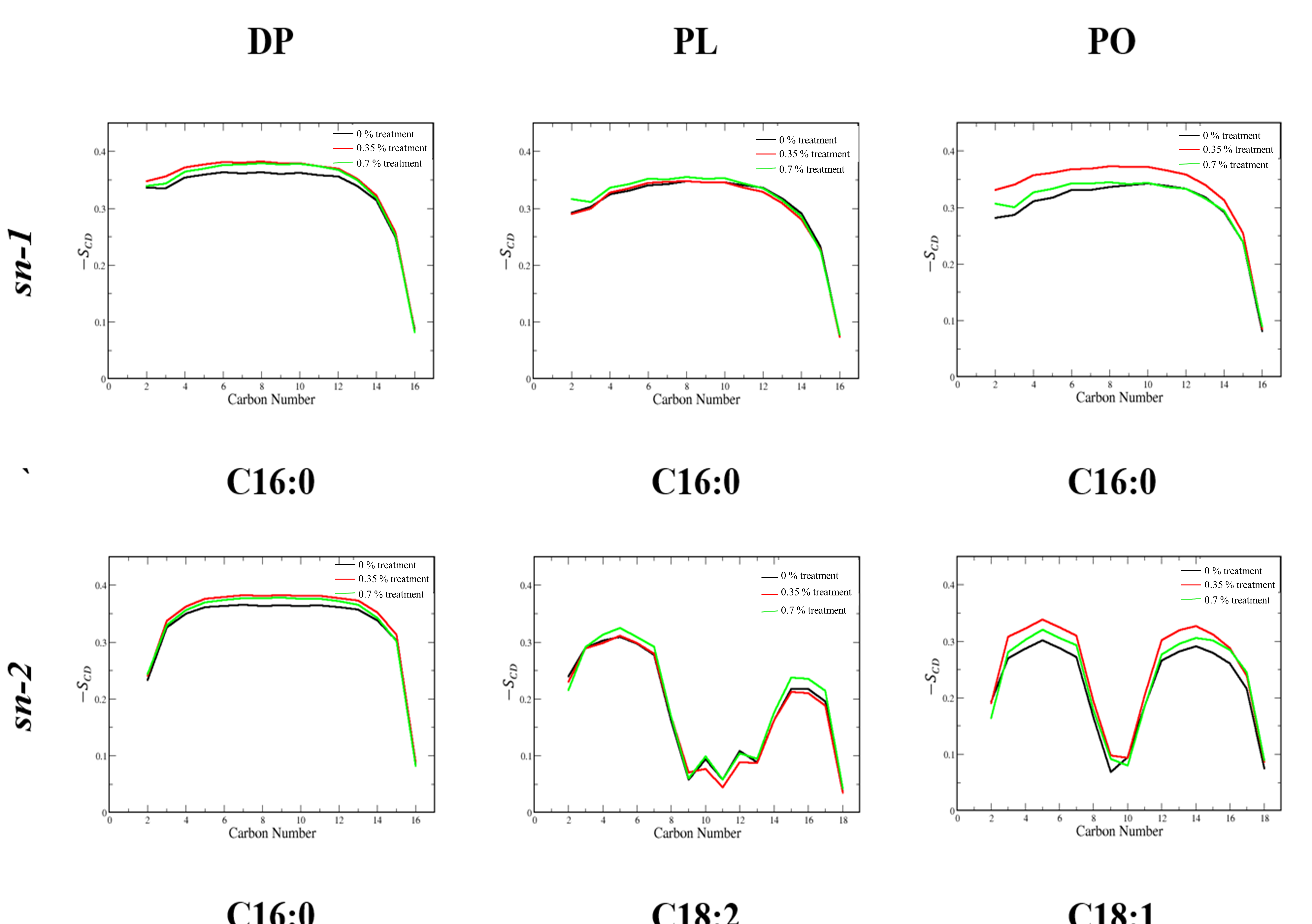

Supplement: Supplementary file 1 [file DataSheet_1.zip › Supplementary Figure 6.TIF]
