# Supplementary material for: Underpinning beneficial maize response to application of minimally processed homogenates of red and brown seaweeds
Source: Front Plant Sci. 2023 Nov 30;14:1273355. doi: 10.3389/fpls.2023.1273355 (PMC10723902; doi:10.3389/fpls.2023.1273355)
Supplement: Supplementary file 2 [file DataSheet_2.pdf]

Qualitative Compound Report

**Data File**	AQU102.d	**Sample Name**	AQU102
**Sample Type**	Sample	**Position**	P1-B5
**Instrument Name**	QTOF	**User Name**	
**Acq Method**	metabolite_ESI_+VE_MSMS.m	**Acquired Time**	4/18/2022 7:10:42 PM
**IRM Calibration Status**	Success	**DA Method**	default.m
**Comment**			

**Sample Group**		**Info.**
**Acquisition SW Version**	6200 series TOF/6500 series Q-TOF B.05.01 (B5125.3)	

Compound Table

Compound Label	RT	Mass	Abund	Name	Formula	MFG Formula	DB Formula	DB Diff (ppm)	Hits (DB)
Cpd 1: Retronecine	1.11	155.0943	99775	Retronecine	C8 H13 N O2	C8 H13 N O2	C8 H13 N O2	2.22	3
Cpd 2: Indospicine	1.35	173.1152	35781	Indospicine	C7 H15 N3 O2	C7 H15 N3 O2	C7 H15 N3 O2	6.98	3
Cpd 3: Pirbuterol	1.504	240.1467	27776	Pirbuterol	C12 H20 N2 O3	C12 H20 N2 O3	C12 H20 N2 O3	2.82	10
Compound 4	1.513		27162						
Cpd 5: Phencyclidine	1.522	243.1962	19183	Phencyclidine	C17 H25 N	C17 H25 N	C17 H25 N	10.25	1
Compound 6	2.68		19254						
Compound 7	3.032		45373						
Cpd 8: Isocarbostyril	3.532	145.0521	76125	Isocarbostyril	C9 H7 N O	C9 H7 N O	C9 H7 N O	4.32	10
Cpd 9: Fasoracetam	3.627	196.1209	102932	Fasoracetam	C10 H16 N2 O2	C10 H16 N2 O2	C10 H16 N2 O2	1.34	6
Cpd 10: Sinapoylputrescine	3.855	294.1565	18014	Sinapoylputrescine	C15 H22 N2 O4	C15 H22 N2 O4	C15 H22 N2 O4	4.95	10
Cpd 11: Fasoracetam	3.876	196.1208	172449	Fasoracetam	C10 H16 N2 O2	C10 H16 N2 O2	C10 H16 N2 O2	2.16	6
Cpd 12: 4-Dodecylbenzenesulfonic acid	4.173	326.1907	18702	4-Dodecylbenzenesulfonic acid	C18 H30 O3 S	C18 H30 O3 S	C18 H30 O3 S	2.8	10
Cpd 13: Tyrosyl-Glycine	4.43	238.0944	20542	Tyrosyl-Glycine	C11 H14 N2 O4	C11 H14 N2 O4	C11 H14 N2 O4	3.9	10
Cpd 14: Methohexital	4.466	262.1304	25069	Methohexital	C14 H18 N2 O3	C14 H18 N2 O3	C14 H18 N2 O3	5.05	10
Compound 15	4.487		28913						
Cpd 16: Sinapoylputrescine	4.726	294.1564	17254	Sinapoylputrescine	C15 H22 N2 O4	C15 H22 N2 O4	C15 H22 N2 O4	5.46	10
Cpd 17: L,L-Cyclo(leucylprolyl)	4.76	210.1361	156361	L,L-Cyclo(leucylprolyl)	C11 H18 N2 O2	C11 H18 N2 O2	C11 H18 N2 O2	3.57	3
Cpd 18: Istamycin C1	4.778	431.2704		Istamycin C1	C19 H37 N5 O6	C19 H37 N5 O6	C19 H37 N5 O6	9.25	1
Cpd 19: L,L-Cyclo(leucylprolyl)	5.099	210.1361	94597	L,L-Cyclo(leucylprolyl)	C11 H18 N2 O2	C11 H18 N2 O2	C11 H18 N2 O2	3.63	3
Cpd 20: trans,trans-Farnesyl phosphate	5.124	302.1614	30742	trans,trans-Farnesyl phosphate	C15 H27 O4 P	C15 H27 O4 P	C15 H27 O4 P	10.79	1
Compound 21	5.303		37125						
Cpd 22: 17beta-Nitro-5alpha-androstane	5.39	305.2322	18961	17beta-Nitro-5alpha-androstane	C19 H31 N O2	C19 H31 N O2	C19 H31 N O2	10.71	5
Cpd 23: Hexyl 2-furoate	5.595	196.1096	38479	Hexyl 2-furoate	C11 H16 O3	C11 H16 O3	C11 H16 O3	1.74	4
Cpd 24: 3-Oxo-12,18-ursadien-28-oic acid	5.764	452.3332	31616	3-Oxo-12,18-ursadien-28-oic acid	C30 H44 O3	C30 H44 O3	C30 H44 O3	-9.18	10
Cpd 25: Hexyl 2-furoate	5.986	196.1102	25591	Hexyl 2-furoate	C11 H16 O3	C11 H16 O3	C11 H16 O3	-1.32	4
Cpd 26: 2-Pentadecylfuran	6.069	278.2575	24674	2-Pentadecylfuran	C19 H34 O	C19 H34 O	C19 H34 O	12.38	5
Compound 27	6.676								
Compound 28	6.712								
Compound 29	6.715								
Cpd 30: DG(20:5(5Z,8Z,11Z,14Z,17Z)/20:2(11Z,14Z)/0:0)	6.86	666.5245	11825	DG(20:5(5Z,8Z,11Z,14Z,17Z)/20:2(11Z,14Z)/0:0)	C43 H70 O5	C43 H70 O5	C43 H70 O5	-3.33	10
Cpd 31: Sulfoglycolithocholate	7.022	513.2751	14358	Sulfoglycolithocholate	C26 H43 N O7 S	C26 H43 N O7 S	C26 H43 N O7 S	1.74	4
Compound 32	7.069		44501						
Compound 33	7.286		25425						
Cpd 34: Dihydrocapsaicin	7.465	307.2109	19487	Dihydrocapsaicin	C18 H29 N O3	C18 H29 N O3	C18 H29 N O3	12.49	3
Cpd 35: 1-Phosphatidyl-1D-myo-inositol 3-phosphate	8.146	470.0227		1-Phosphatidyl-1D-myo-inositol 3-phosphate	C11 H20 O16 P2	C11 H20 O16 P2	C11 H20 O16 P2	-0.19	2
Cpd 36: 2,3-Dihydro-6-methyl-5-propanoyl-1H-pyrrolizine	8.383	177.115		2,3-Dihydro-6-methyl-5-propanoyl-1H-pyrrolizine	C11 H15 N O	C11 H15 N O	C11 H15 N O	1.84	10
Cpd 37: 1-Phenyl-5-propyl-1H-pyrazole	8.504	186.1184	49975	1-Phenyl-5-propyl-1H-pyrazole	C12 H14 N2	C12 H14 N2	C12 H14 N2	-14.63	3
Cpd 38: 1-Phenyl-5-propyl-1H-pyrazole	8.779	186.118	27453	1-Phenyl-5-propyl-1H-pyrazole	C12 H14 N2	C12 H14 N2	C12 H14 N2	-12.33	3
Compound 39	8.875		127864						
Compound 40	9.251		246217						
Cpd 41: Protorifamycin I	9.512	639.3066		Protorifamycin I	C35 H45 N O10	C35 H45 N O10	C35 H45 N O10	-3.56	2
Compound 42	9.62		62902						
Cpd 43: Candoxatrilat	9.791	399.2269	26541	Candoxatrilat	C20 H33 N O7	C20 H33 N O7	C20 H33 N O7	-3.02	1
Cpd 44: Anapheline	9.946	224.1881	403305	Anapheline	C13 H24 N2 O	C13 H24 N2 O	C13 H24 N2 O	3.4	2
Cpd 45: 25-Methylgramisterol	9.972	426.3921		25-Methylgramisterol	C30 H50 O	C30 H50 O	C30 H50 O	-13.9	10
Cpd 46: Anapheline	10.277	224.1879	90036	Anapheline	C13 H24 N2 O	C13 H24 N2 O	C13 H24 N2 O	4.26	2
Cpd 47: Rhipocephalin	10.329	376.1938	23158	Rhipocephalin	C21 H28 O6	C21 H28 O6	C21 H28 O6	-13.75	7
Compound 48	10.552		98316						
Compound 49	10.714		20319						
Compound 50	10.786		37061						
Compound 51	11.85		24609						
Cpd 52: 5-Tricosyl-1,3-benzenediol	12.192	432.392	22667	5-Tricosyl-1,3-benzenediol	C29 H52 O2	C29 H52 O2	C29 H52 O2	11	2
Cpd 53: 12-(2,3-Dihydroxycyclopentyl)-2-dodecanone	12.506	284.2361	46170	12-(2,3-Dihydroxycyclopentyl)-2-dodecanone	C17 H32 O3	C17 H32 O3	C17 H32 O3	-3.27	10
Cpd 54: Ximenoylacetone	12.701	434.4078		Ximenoylacetone	C29 H54 O2	C29 H54 O2	C29 H54 O2	10.62	2
Cpd 55: Ximenoylacetone	13.06	434.408		Ximenoylacetone	C29 H54 O2	C29 H54 O2	C29 H54 O2	10	2
Cpd 56: Stigmastanol	13.425	416.3973	46719	Stigmastanol	C29 H52 O	C29 H52 O	C29 H52 O	10.79	2
Cpd 57: Stigmastanol	13.728	416.3972		Stigmastanol	C29 H52 O	C29 H52 O	C29 H52 O	11.04	2
Cpd 58: Stigmastanol	14.07	416.3972		Stigmastanol	C29 H52 O	C29 H52 O	C29 H52 O	10.97	2
Cpd 59: (Z)-22-Hentriacontene-2,4-dione	14.07	462.439		(Z)-22-Hentriacontene-2,4-dione	C31 H58 O2	C31 H58 O2	C31 H58 O2	10.23	1
Cpd 60: Hydroflumethiazide	14.18	330.9945	130245	Hydroflumethiazide	C8 H8 F3 N3 O4 S2	C8 H8 F3 N3 O4 S2	C8 H8 F3 N3 O4 S2	-11.12	1
Cpd 61: Methyl 2-furoate	14.407	126.034	108757	Methyl 2-furoate	C6 H6 O3	C6 H6 O3	C6 H6 O3	-18.25	9

Cpd 62: (Z)-22-Hentriacontene-2,4-dione	14.408	462.4391		(Z)-22-Hentriacontene-2,4-dione	C31 H58 O2	C31 H58 O2	C31 H58 O2	9.82	1
Cpd 63: Hydroflumethiazide	14.533	330.9945		Hydroflumethiazide	C8 H8 F3 N3 O4 S2	C8 H8 F3 N3 O4 S2	C8 H8 F3 N3 O4 S2	-11.1	1
Compound 64	14.819								
Cpd 65: Ascorbyl stearate	14.902	442.2873		Ascorbyl stearate	C24 H42 O7	C24 H42 O7	C24 H42 O7	12.9	1
Compound 66	14.998		33902						
Cpd 67: Chlorfenvinphos	15.061	357.9707	87531	Chlorfenvinphos	C12 H14 Cl3 O4 P	C12 H14 Cl3 O4 P	C12 H14 Cl3 O4 P	-3.3	1
Cpd 68: Idoxuridine	15.216	353.9727	71220	Idoxuridine	C9 H11 I N2 O5	C9 H11 I N2 O5	C9 H11 I N2 O5	-4.03	1
Compound 69	15.299								
Cpd 70: Chlorfenvinphos	15.367	357.9706	28521	Chlorfenvinphos	C12 H14 Cl3 O4 P	C12 H14 Cl3 O4 P	C12 H14 Cl3 O4 P	-2.95	1
Cpd 71: (E,E)-1,6-bis(4-methoxyphenyl)-1,5-hexadiene	15.418	294.1638	29638	(E,E)-1,6-bis(4-methoxyphenyl)-1,5-hexadiene	C20 H22 O2	C20 H22 O2	C20 H22 O2	-6.09	10
Cpd 72: Undecylprodigiosin	15.662	393.2757	122506	Undecylprodigiosin	C25 H35 N3 O	C25 H35 N3 O	C25 H35 N3 O	5.89	1
Cpd 73: Undecylprodigiosin	15.909	393.2758	44696	Undecylprodigiosin	C25 H35 N3 O	C25 H35 N3 O	C25 H35 N3 O	5.72	1
Cpd 74: 1,1'-Bis(2-hydroxy-3-methylcarbazole)	16.906	392.1524	18860	1,1'-Bis(2-hydroxy-3-methylcarbazole)	C26 H20 N2 O2	C26 H20 N2 O2	C26 H20 N2 O2	0.23	10
Cpd 75: 5-Decanoyl-2-nonylpyridine	16.947	359.3189	57178	5-Decanoyl-2-nonylpyridine	C24 H41 N O	C24 H41 N O	C24 H41 N O	-0.25	2
Cpd 76: 2,4,14-Eicosatrienoic acid isobutylamide	17.907	361.3342	74225	2,4,14-Eicosatrienoic acid isobutylamide	C24 H43 N O	C24 H43 N O	C24 H43 N O	0.69	2
Cpd 77: 2,4,14-Eicosatrienoic acid isobutylamide	18.278	361.3342	32767	2,4,14-Eicosatrienoic acid isobutylamide	C24 H43 N O	C24 H43 N O	C24 H43 N O	0.65	2
Cpd 78: Octadecyl fumarate	19.422	368.2927		Octadecyl fumarate	C22 H40 O4	C22 H40 O4	C22 H40 O4	-0.18	10
Compound 79	20.954								
Compound 80	21.035		31023						

Compound Label	Name	*m/z*	RT	Algorithm	Mass
Cpd 1: Retronecine	**Retronecine**	156.1016	1.11	Auto MS/MS	155.0943

MS Spectrum

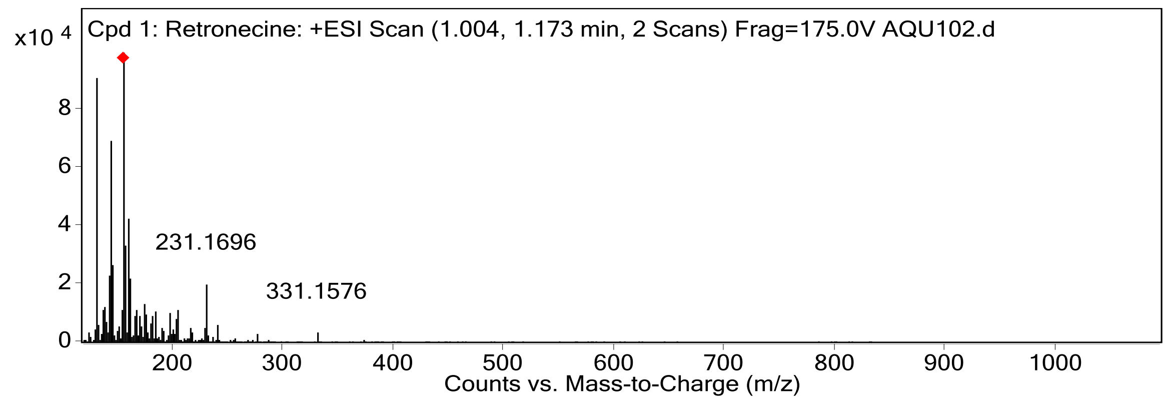

MS Zoomed Spectrum

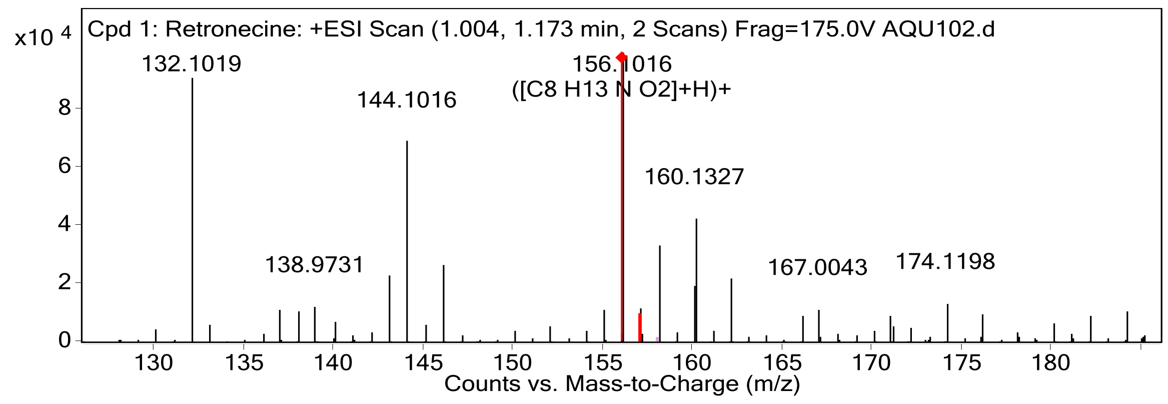

MS Spectrum Peak List

m/z	*Calc m/z*	Diff(ppm)	z	Abund	Formula	Ion
132.1019			1	90805.86		
143.0813			1	23180.17		
144.1016			1	69291.25		
146.1171			1	26577.35		
156.1016	156.1019	1.72	1	99775.47	C8 H13 N O2	(M+H)+
157.1041	157.1051	6.35	1	11691.39	C8 H13 N O2	(M+H)+
158.1168			1	33616.21		
160.1327			1	42820.3		
162.1123			1	22318.96		
231.1696			1	20292.71		

MSMS Spectrum

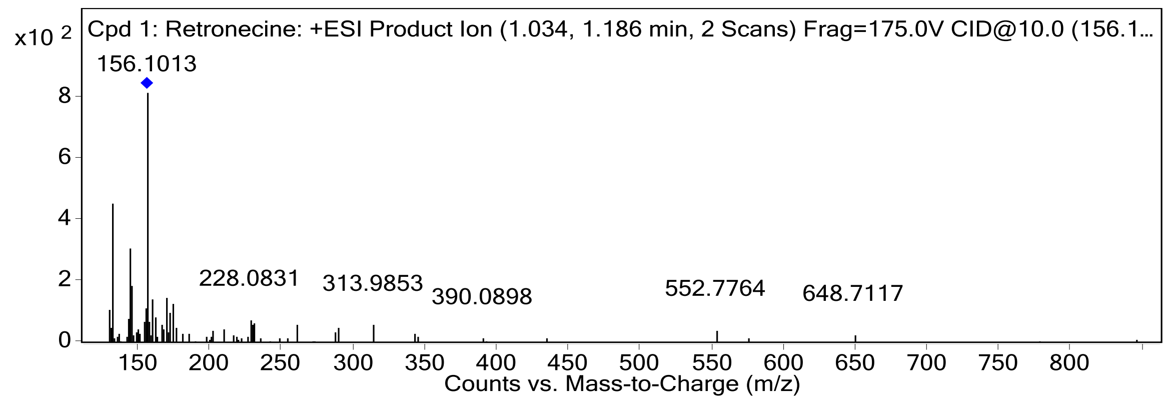

MS/MS Spectrum Peak List

m/z	z	Abund
130.0605		106.61
132.103		454.7
144.1005		308.94
145.1043		183.28
155.081	1	113.53
156.1013	1	817.02
160.0963		99.74
160.1323		140.58
170.1142		148.46
174.1266		127.7

Compound Structure

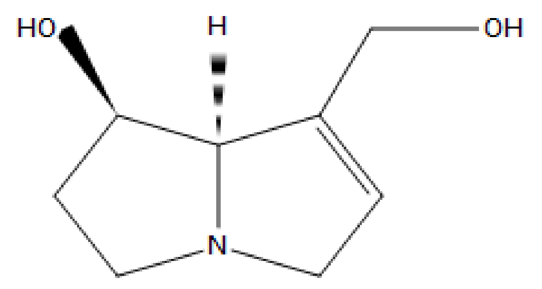

Compound Label	Name	*m/z*	RT	Algorithm	Mass
Cpd 2: Indospicine	**Indospicine**	174.1226	1.35	Auto MS/MS	173.1152

MS Spectrum

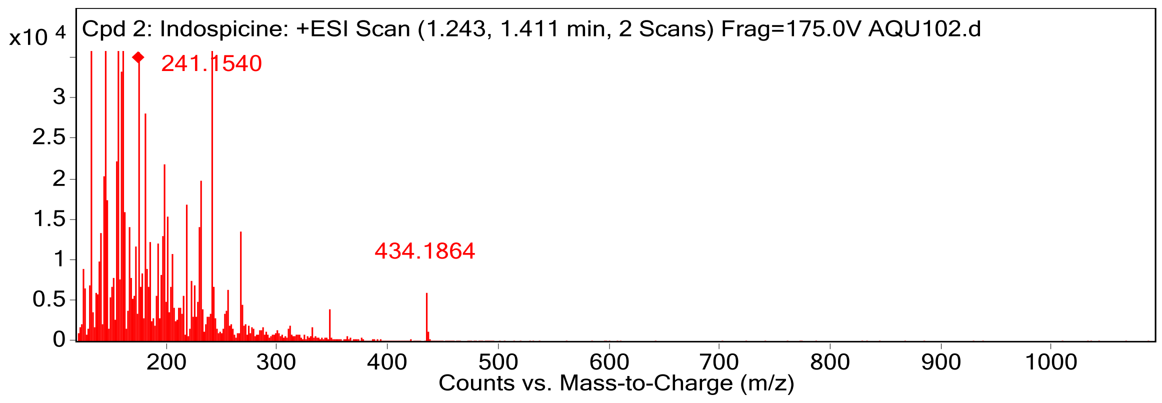

MS Zoomed Spectrum

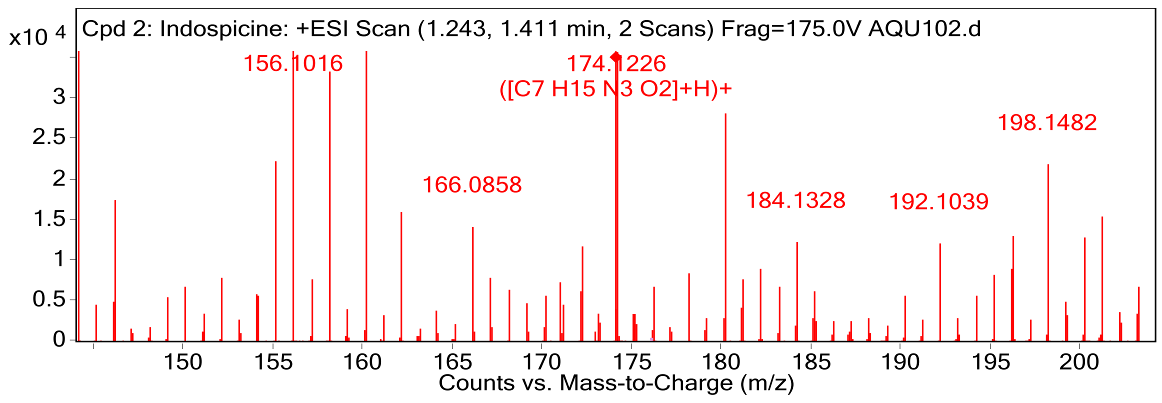

MS Spectrum Peak List

m/z	*Calc m/z*	Diff(ppm)	z	Abund	Formula	Ion
132.1018			1	47790.86		
144.1017			1	45461.89		
155.0921				22291.66		
156.1016			1	66653.04		
158.1172			1	33474.82		
160.1326				41143.45		
174.1226	174.1237	6.45		35780.66	C7 H15 N3 O2	(M+H)+
175.1239	175.1263	14.13	1	2211.87	C7 H15 N3 O2	(M+H)+
180.1013			1	28179.17		
241.154			1	36200.97		

MSMS Spectrum

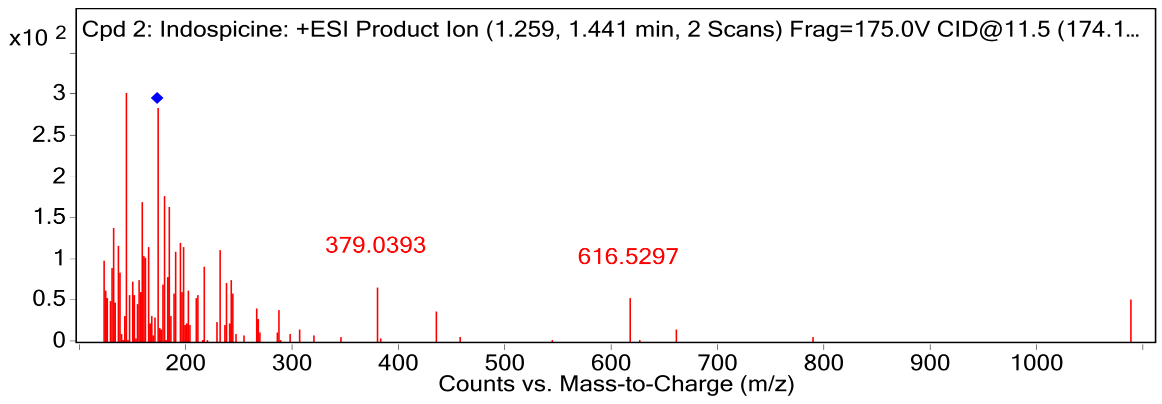

MS/MS Spectrum Peak List

m/z	z	Abund
132.1008		139.98
136.078		118.12
144.1028		303.04
158.1155		169.32
174.1235		283.44
174.1484	1	273.14
180.102		177.47
184.0944		164.81
194.1173		121.98
198.1497	1	116.62

Compound Structure

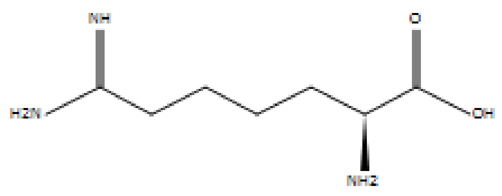

Compound Label	Name	*m/z*	RT	Algorithm	Mass
Cpd 3: Pirbuterol	**Pirbuterol**	241.1541	1.504	Auto MS/MS	240.1467

MS Spectrum

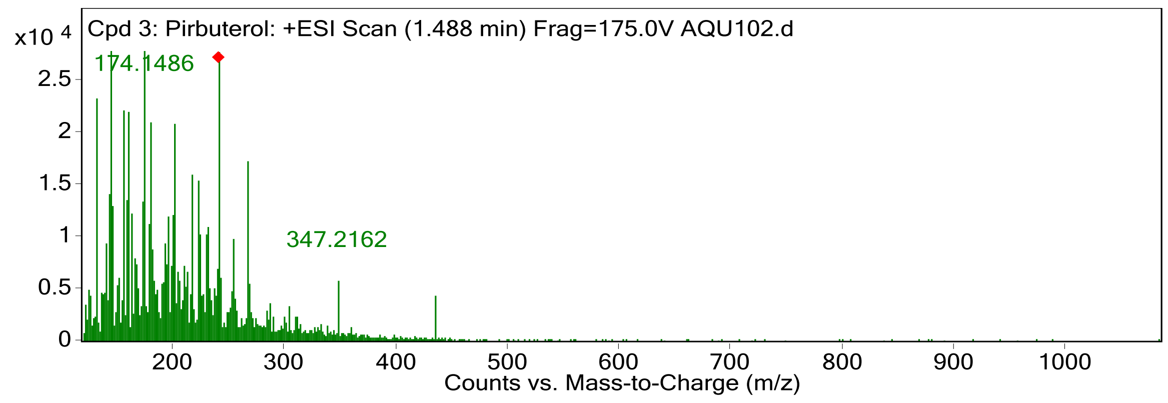

MS Zoomed Spectrum

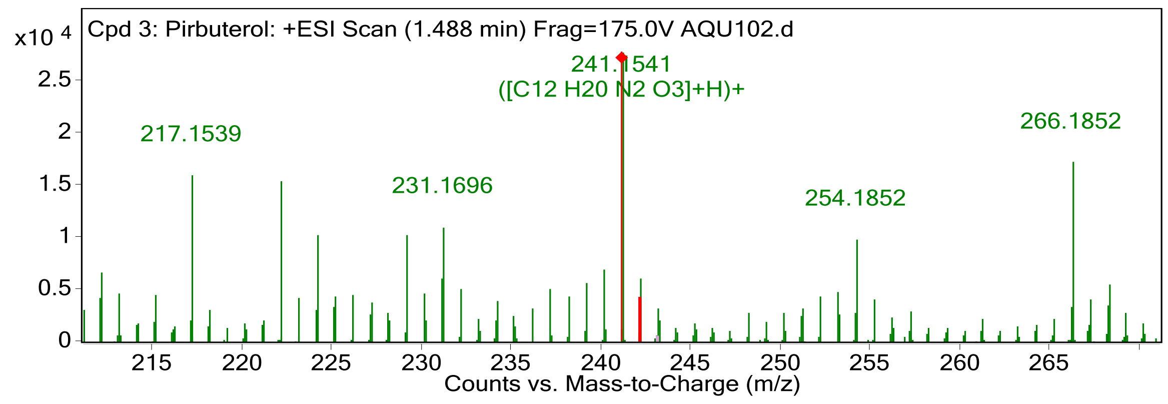

MS Spectrum Peak List

m/z	*Calc m/z*	Diff(ppm)	z	Abund	Formula	Ion
132.1021				23334.17		
144.0658				19492.68		
144.1018				30860.44		
155.0918				22251.08		
160.0971				22116.27		
174.1486				33077.1		
180.1011				21048.38		
201.0714				20925.83		
241.1541	241.1547	2.49	1	27776.15	C12 H20 N2 O3	(M+H)+
242.1567	242.1578	4.21	1	6202.51	C12 H20 N2 O3	(M+H)+

MSMS Spectrum

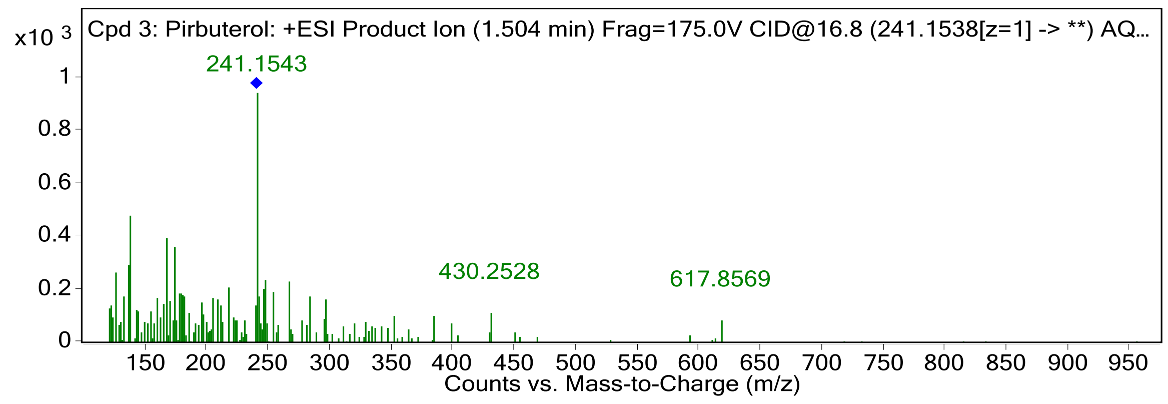

MS/MS Spectrum Peak List

m/z	z	Abund
126.0578		264.66
136.0629		291.95
138.0568		480.37
167.0812		392.3
174.1489	1	363.47
218.0945		210.18
241.1543	1	944.2
247.0563		221.72
247.1509		235.15
267.1967		229

Compound Structure

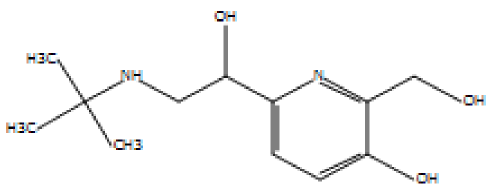

Compound Label	*m/z*	RT	Algorithm
Compound 4	174.1486	1.513	Auto MS/MS

MS Spectrum

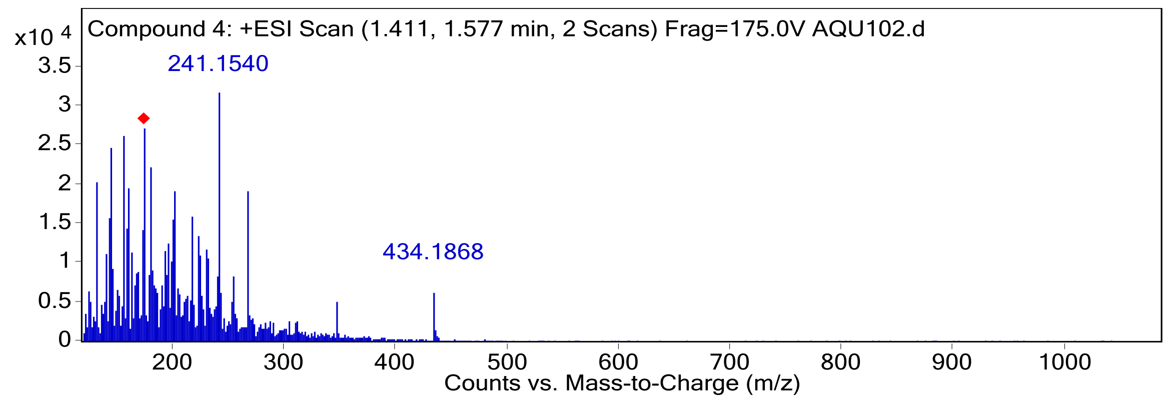

MS Zoomed Spectrum

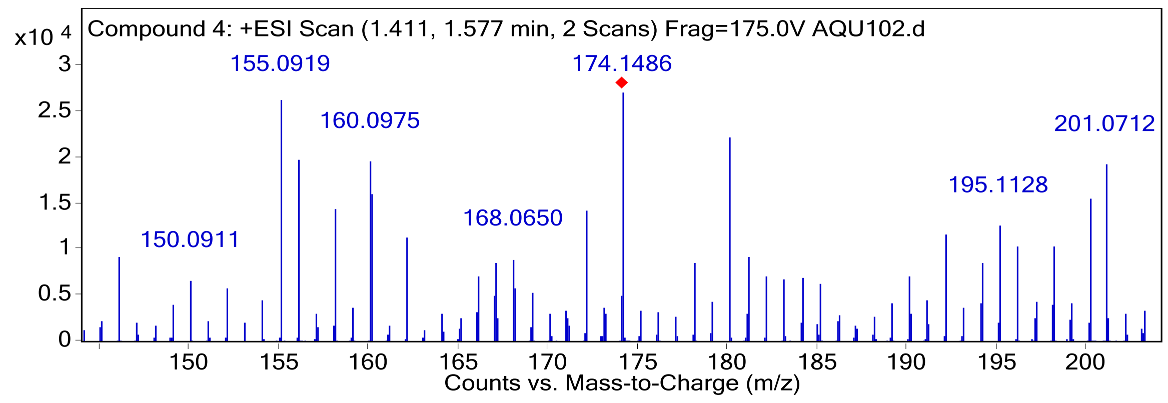

MS Spectrum Peak List

m/z	z	Abund
132.102		20234.7
144.1017		24630.8
155.0919		26227.12
156.1018		19876.29
160.0975		19604.12
174.1223		19643.64
174.1486		27161.67
175.152	1	3338.94
180.1014		22255.21
241.154	1	31756.87

MSMS Spectrum

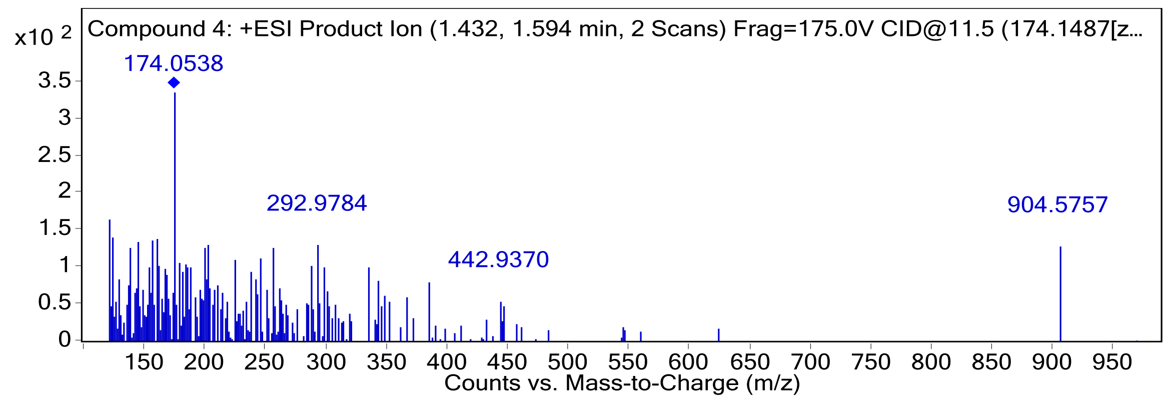

MS/MS Spectrum Peak List

m/z	Abund
121.0652	164.96
123.0917	141.37
144.1022	133.77
156.101	136.36
160.0947	138.48
174.0538	336.18
174.1478	306.88
202.1416	129.96
292.9784	130.89
904.5757	128.81

Compound Label	Name	*m/z*	RT	Algorithm	Mass
Cpd 5: Phencyclidine	**Phencyclidine**	266.1857	1.522	Auto MS/MS	243.1962

MS Spectrum

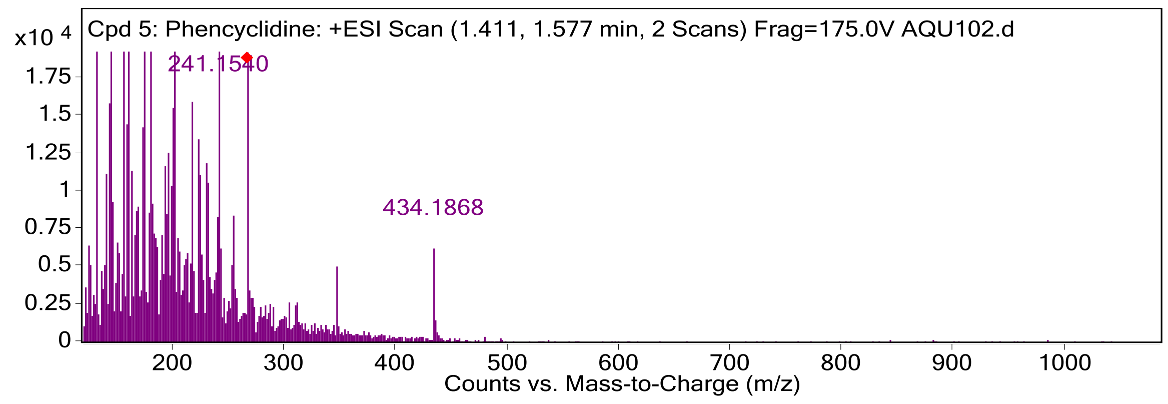

MS Zoomed Spectrum

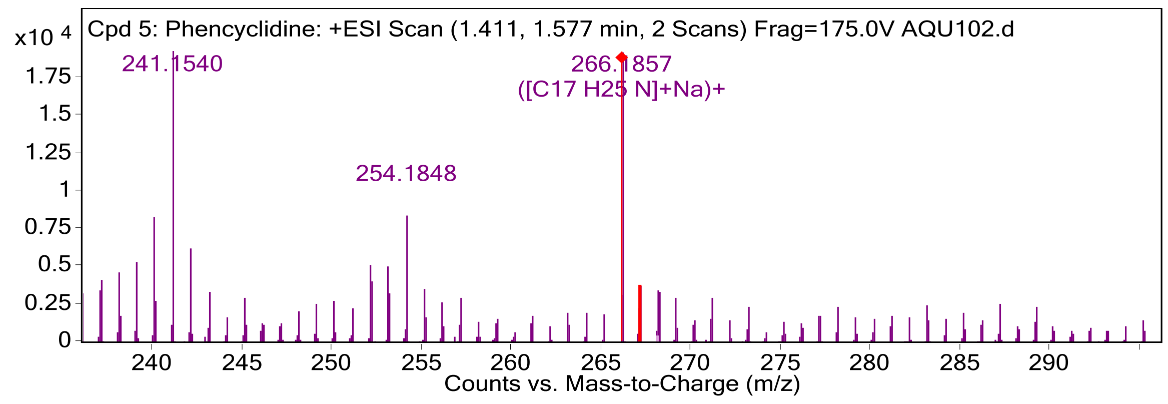

MS Spectrum Peak List

m/z	*Calc m/z*	Diff(ppm)	z	Abund	Formula	Ion
132.102				20234.7		
144.1017				24630.8		
155.0919				26227.12		
156.1018				19876.29		
174.1223				19643.64		
174.1486				27161.67		
180.1014				22255.21		
241.154			1	31756.87		
266.1857	266.1879	8.45		19182.65	C17 H25 N	(M+Na)+
267.1873	267.1912	14.43	1	3381.35	C17 H25 N	(M+Na)+

MSMS Spectrum

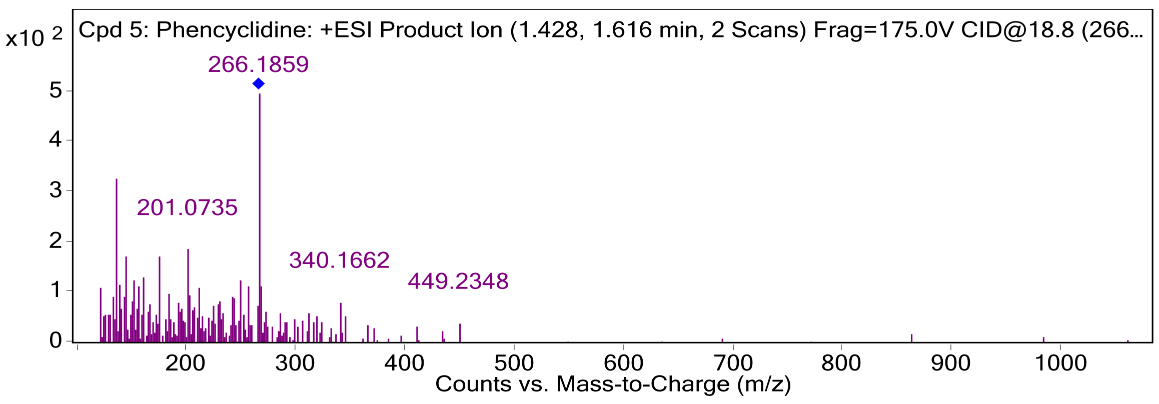

MS/MS Spectrum Peak List

m/z	Abund
136.0623	326.02
138.0541	116.94
144.1022	172.49
151.0839	124.97
160.096	132.03
174.1479	172.19
201.0735	186.62
249.1262	126.02
256.1386	113.95
266.1859	496.49

Compound Structure

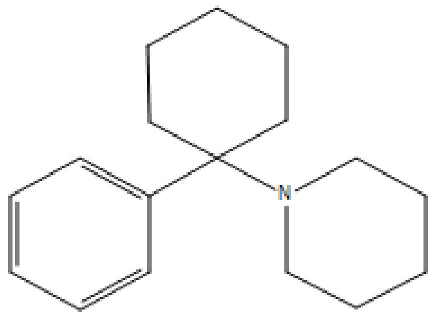

Compound Label	*m/z*	RT	Algorithm
Compound 6	186.1479	2.68	Auto MS/MS

MS Spectrum

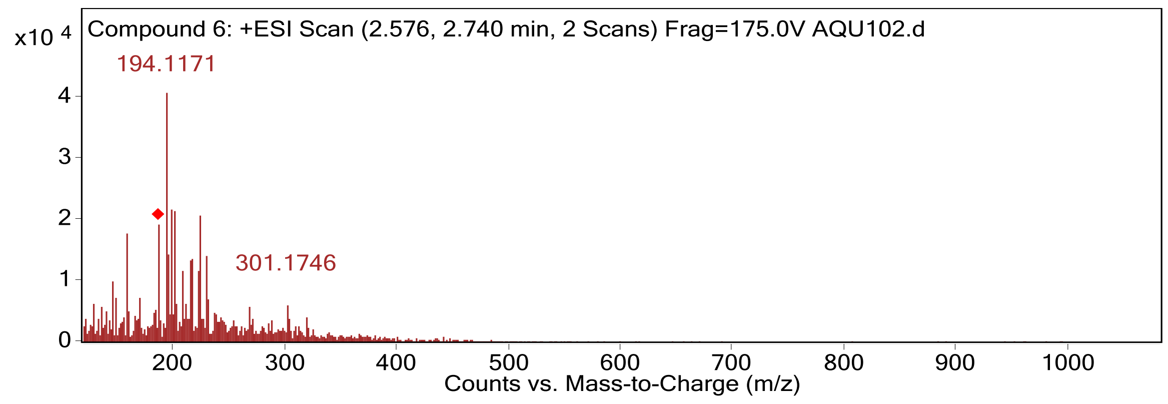

MS Zoomed Spectrum

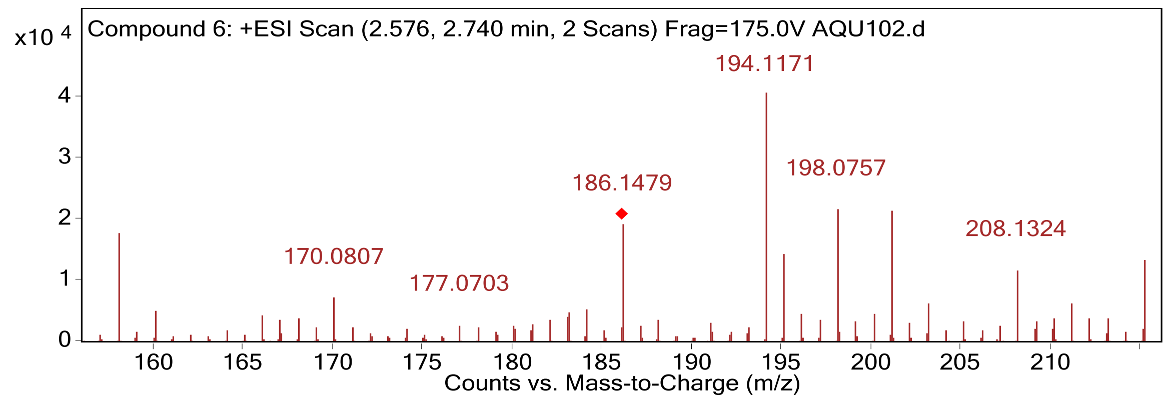

MS Spectrum Peak List

m/z	z	Abund
158.0809		17713.9
186.1479		19254
187.1497	1	2607
188.1622	1	300.71
194.1171	1	40830.17
195.1148	1	14412.47
198.0757	1	21717.59
201.1164	1	21489.5
224.1276	1	20675.71
229.1537		14055.27

MSMS Spectrum

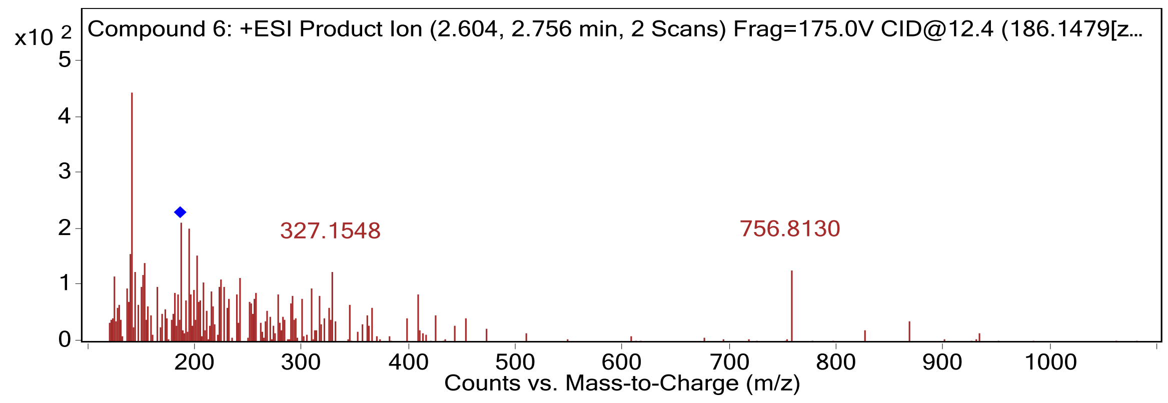

MS/MS Spectrum Peak List

m/z	z	Abund
139.073		155.98
139.0936		122.71
140.1434	1	445.15
143.0015		124.6
153.0553		141.38
186.1485		211.58
194.1163	1	201.68
201.1164		153.91
327.1548		124.14
756.813		127.92

Compound Label	*m/z*	RT	Algorithm
Compound 7	186.1481	3.032	Auto MS/MS

MS Spectrum

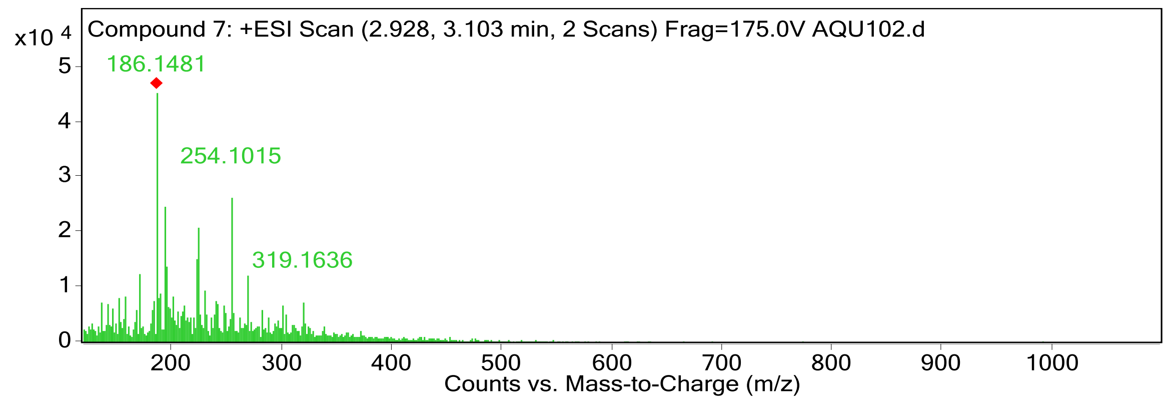

MS Zoomed Spectrum

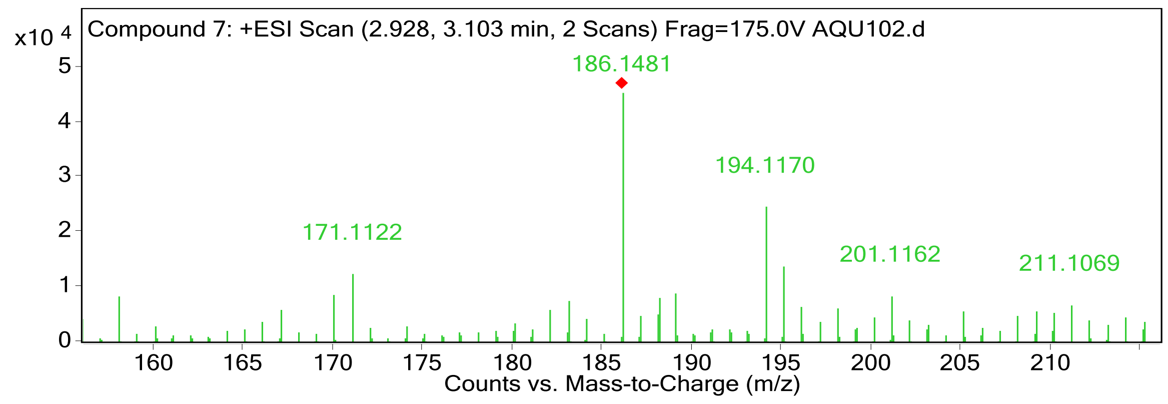

MS Spectrum Peak List

m/z	z	Abund

171.1122		12481.29
186.1481	1	45372.8
187.1512	1	4809.36
194.117		24673.28
195.1139		13799.54
222.1113	1	15202.27
224.1276	1	20923.57
229.1179		9529.15
254.1015	1	26407.94
268.2006		12263.89

MSMS Spectrum

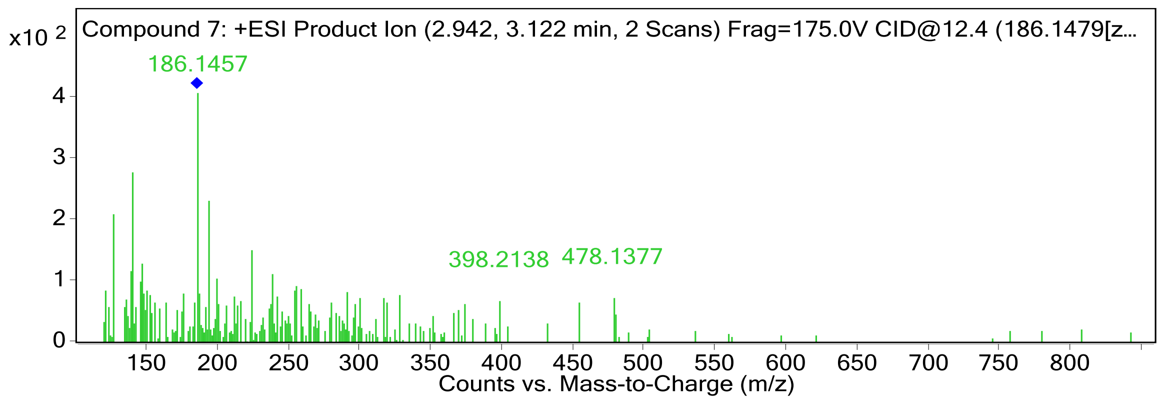

MS/MS Spectrum Peak List

m/z	z	Abund
127.0396		209.92
139.0768		116.3
140.0325		185.58
140.1421		276.5
147.0863		128.41
186.1457	1	407.04
194.1137		231.14
199.0883		105.11
224.1254		151.14
238.1536		112.1

Compound Label	Name	*m/z*	RT	Algorithm	Mass
Cpd 8: Isocarbostyril	**Isocarbostyril**	146.0595	3.532	Auto MS/MS	145.0521

MS Spectrum

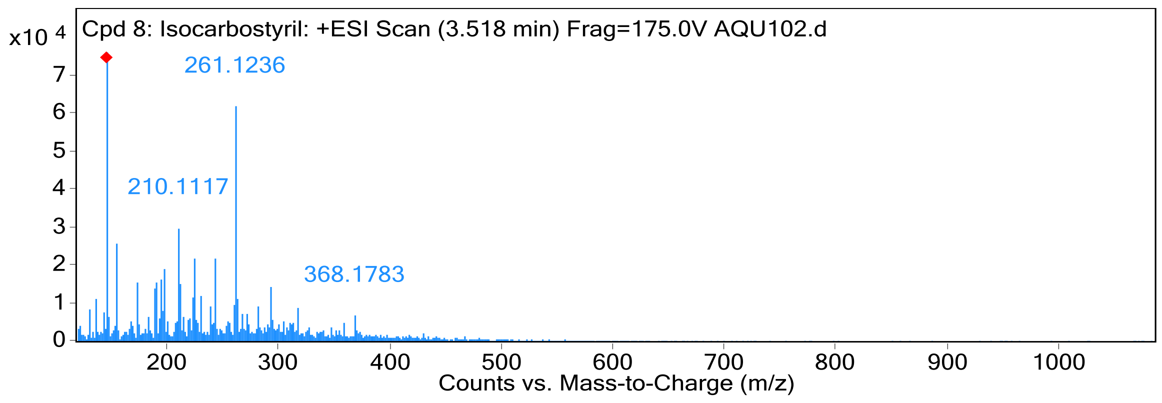

MS Zoomed Spectrum

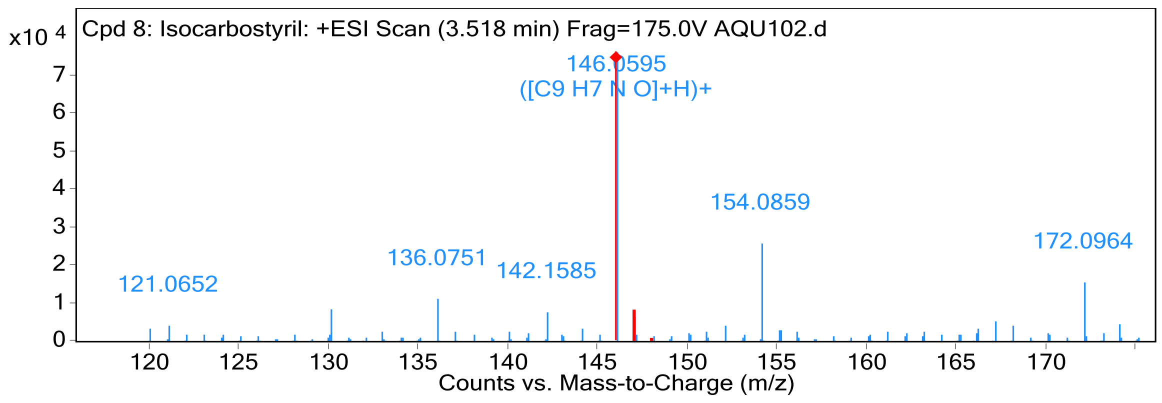

MS Spectrum Peak List

m/z	*Calc m/z*	Diff(ppm)	z	Abund	Formula	Ion
146.0595	146.06	4.03	1	76125.09	C9 H7 N O	(M+H)+
147.0635	147.0632	-2.33	1	6686.58	C9 H7 N O	(M+H)+
148.0593	148.0657	42.99	1	1584.23	C9 H7 N O	(M+H)+
154.0859				25875.55		
194.1171			1	16553.53		
197.1282				19283.56		
210.1117				29975.53		
224.1277			1	22010.03		
243.1334			1	21785.85		
261.1236			1	62002.68		

MSMS Spectrum

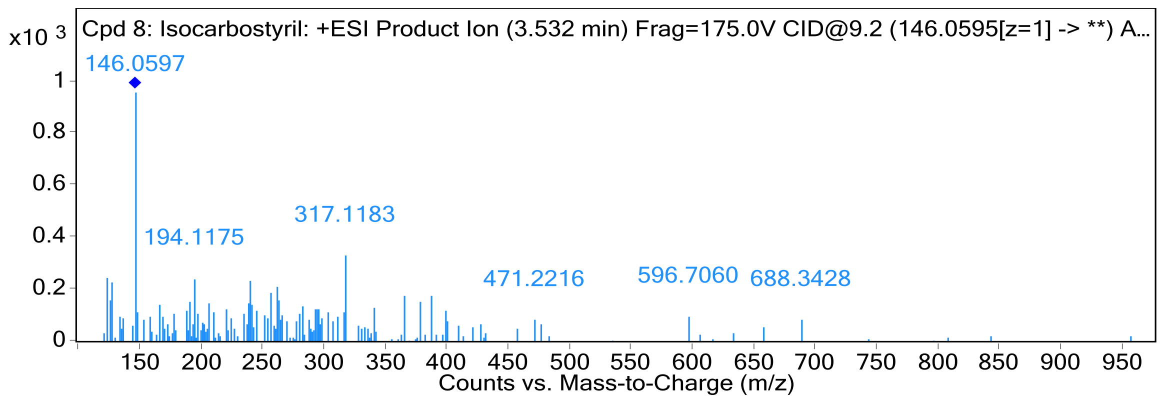

MS/MS Spectrum Peak List

m/z	Abund
123.0793	244.12
127.0823	227.48
146.0597	959.12
194.1175	243.23

239.1364	235.62
256.1727	186.52
261.1217	210.01
317.1183	331.15
365.0748	177.47
387.2418	177.23

Compound Structure

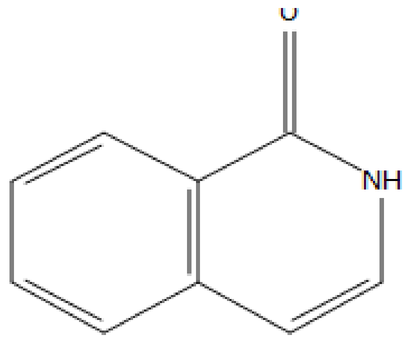

Compound Label	Name	*m/z*	RT	Algorithm	Mass
Cpd 9: Fasoracetam	**Fasoracetam**	197.1279	3.627	Auto MS/MS	196.1209

MS Spectrum

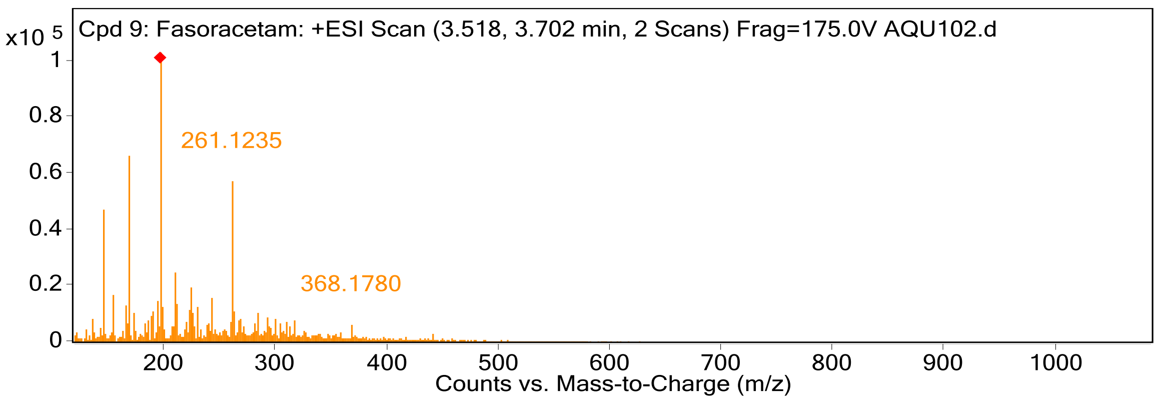

MS Zoomed Spectrum

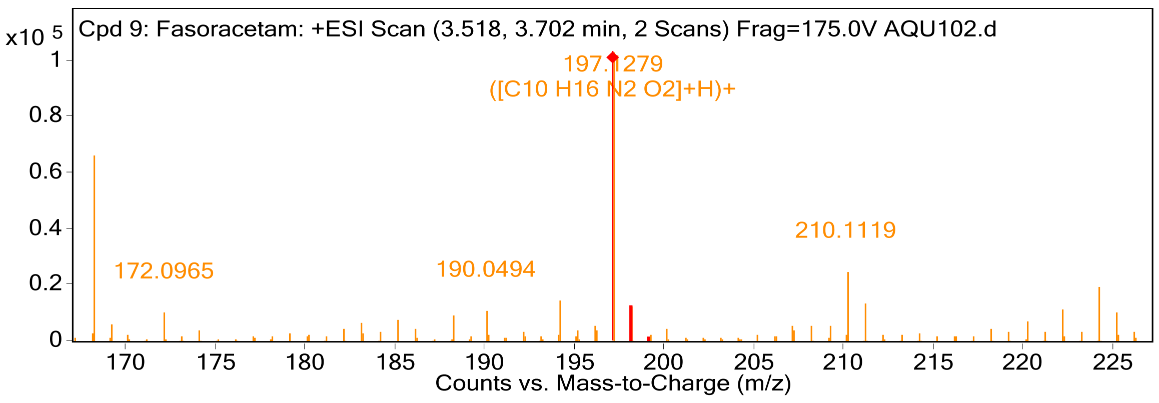

MS Spectrum Peak List

m/z	*Calc m/z*	Diff(ppm)	z	Abund	Formula	Ion
146.0595				47258.68		
154.086				16984.42		
168.1492			1	66398.37		
197.1279	197.1285	2.58	1	102931.91	C10 H16 N2 O2	(M+H)+
198.1315	198.1315	0.08	1	12518.82	C10 H16 N2 O2	(M+H)+
199.1425	199.1338	-44.04	1	2483.48	C10 H16 N2 O2	(M+H)+
210.1119				24989.7		
224.1278			1	19850.57		
243.1332				15785.47		
261.1235			1	57077.59		

MSMS Spectrum

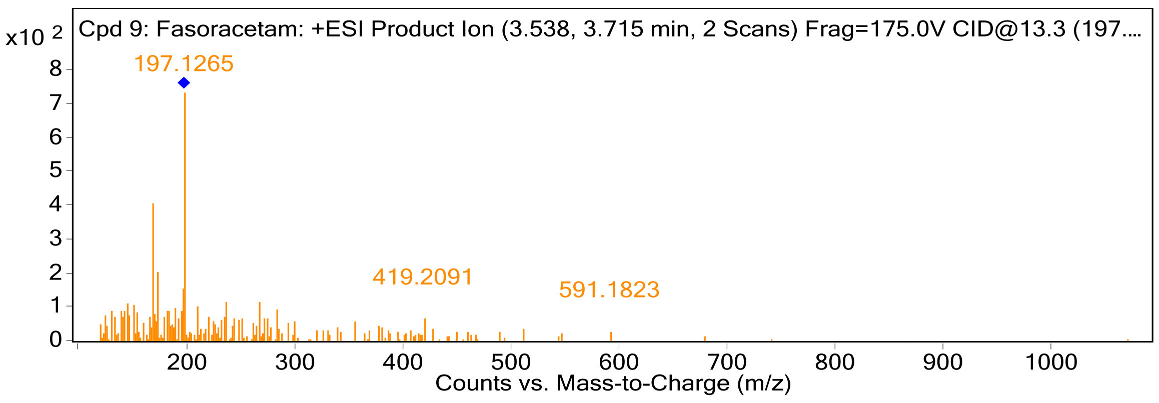

MS/MS Spectrum Peak List

m/z	z	Abund
145.0671		112.26
150.1259		108.5
168.1476	1	410.53
172.094		207.46
188.162		102.12
196.0964		157.5
197.1265	1	736.12
210.1093		106.36
236.1069		117.23
266.1392		117.04

Compound Structure

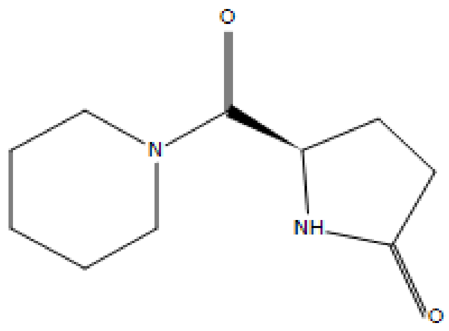

Compound Label	Name	*m/z*	RT	Algorithm	Mass
Cpd 10: Sinapoylputrescine	**Sinapoylputrescine**	295.1639	3.855	Auto MS/MS	294.1565

MS Spectrum

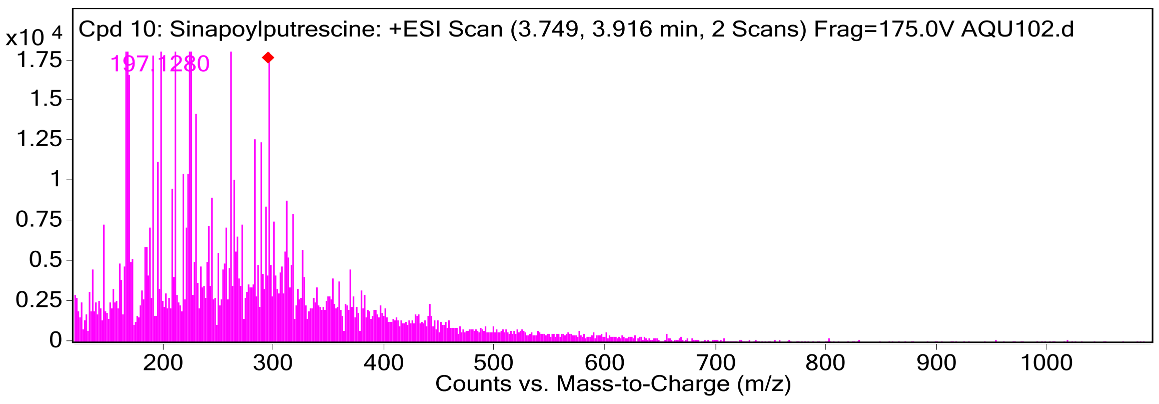

MS Zoomed Spectrum

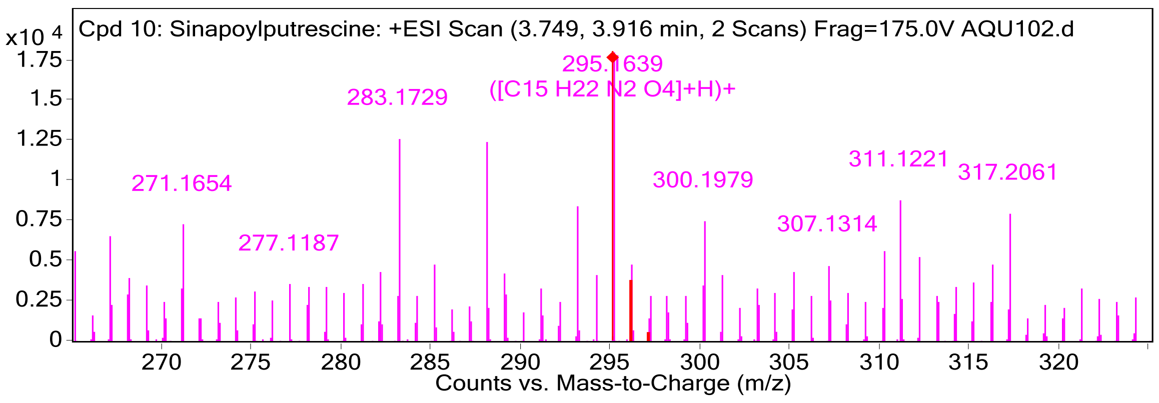

MS Spectrum Peak List

m/z	*Calc m/z*	Diff(ppm)	z	Abund	Formula	Ion
166.1225				23154.38		
168.1493			1	162244		
197.128			1	183680.81		
198.1312			1	22202.94		
210.1119			1	23405.82		
224.1277			1	20709.23		
261.1239			1	24087.21		
295.1639	295.1652	4.39	1	18013.87	C15 H22 N2 O4	(M+H)+
296.1641	296.1684	14.55	1	4789.86	C15 H22 N2 O4	(M+H)+
297.173	297.1708	-7.4	1	2924.6	C15 H22 N2 O4	(M+H)+

MSMS Spectrum

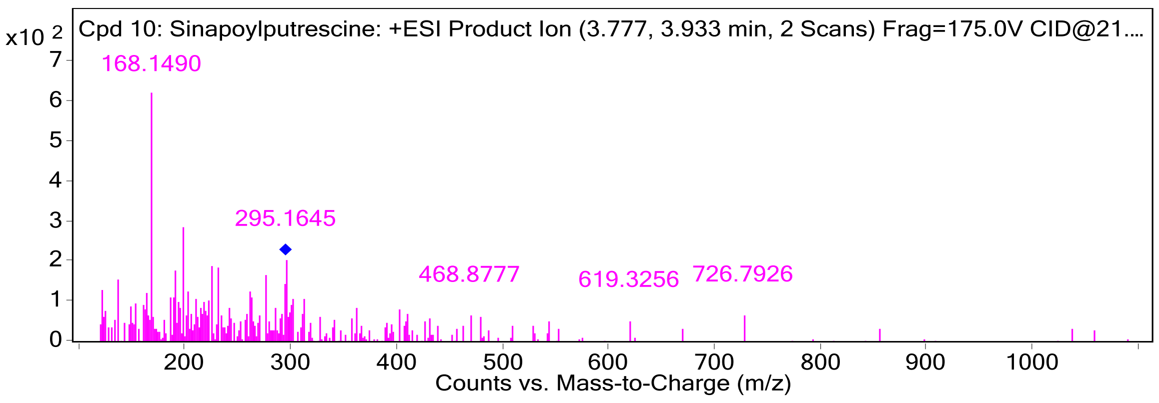

MS/MS Spectrum Peak List

m/z	z	Abund
121.0757		130.5
137.0609		155.66
168.149		623.94
190.0487		180.75
197.1274		285.92
224.1275		189.06
230.1166		185.73
276.119		166.99
294.1322		143.79
295.1645	1	205.39

Compound Structure

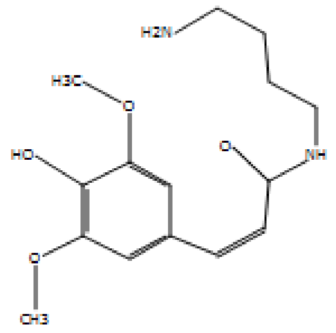

Compound Label	Name	*m/z*	RT	Algorithm	Mass

Cpd 11: Fasoracetam	**Fasoracetam**	197.1279	3.876	Auto MS/MS	196.1208

MS Spectrum

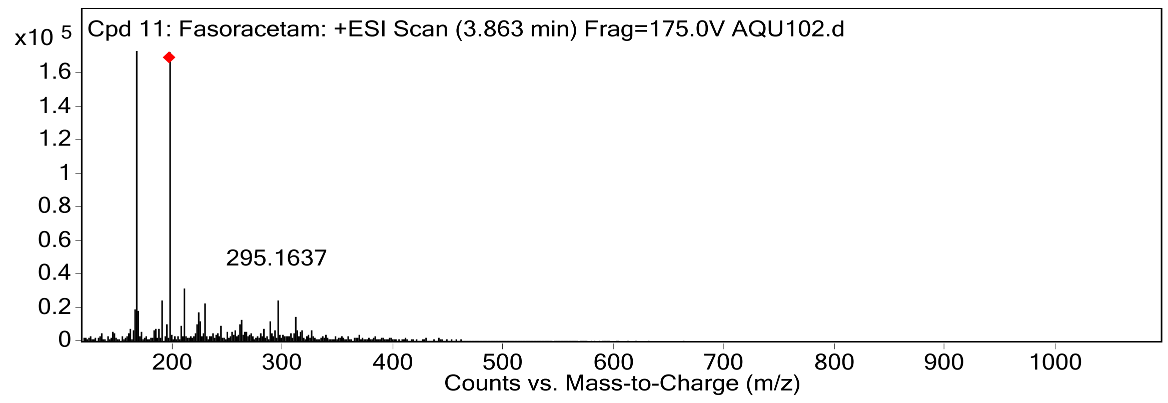

MS Zoomed Spectrum

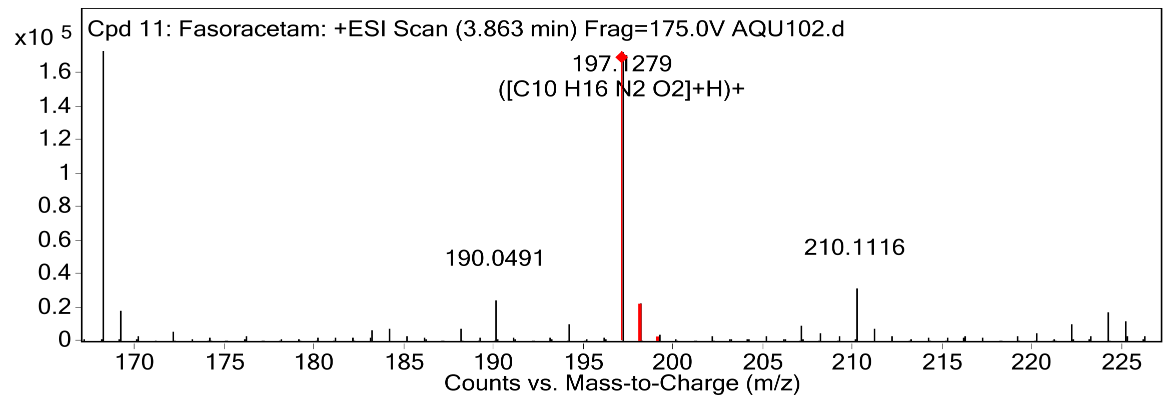

MS Spectrum Peak List

m/z	*Calc m/z*	Diff(ppm)	z	Abund	Formula	Ion
166.1223				19192.46		
168.1492			1	186277.38		
169.1526			1	18454		
190.0491				25068.22		
197.1279	197.1285	2.95	1	172449.08	C10 H16 N2 O2	(M+H)+
198.1309	198.1315	2.99	1	23480.18	C10 H16 N2 O2	(M+H)+
199.1412	199.1338	-37.44	1	4073.85	C10 H16 N2 O2	(M+H)+
210.1116			1	31590.01		
229.1175			1	22909.42		
295.1637			1	25291.29		

MSMS Spectrum

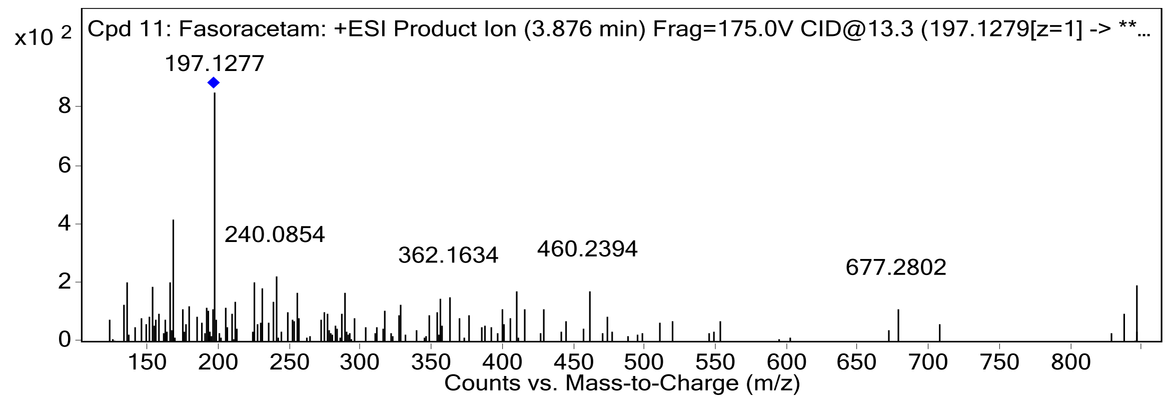

MS/MS Spectrum Peak List

m/z	z	Abund
135.0823		206.76
153.1278		189.02
165.0909	1	203.54
168.1505		418.29
197.1277	1	855.22
224.1274		202.11
230.119		181.82
240.0854		225.63
460.2394		176.24
844.7692		192.33

Compound Structure

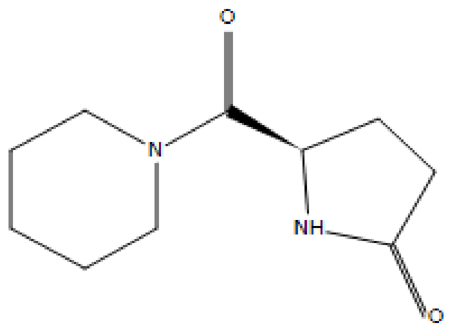

Compound Label	Name	*m/z*	RT	Algorithm	Mass
Cpd 12: 4-Dodecylbenzenesulfonic	**4-Dodecylbenzenesulfoni**	327.199	4.173	Auto MS/MS	326.1907

MS Spectrum

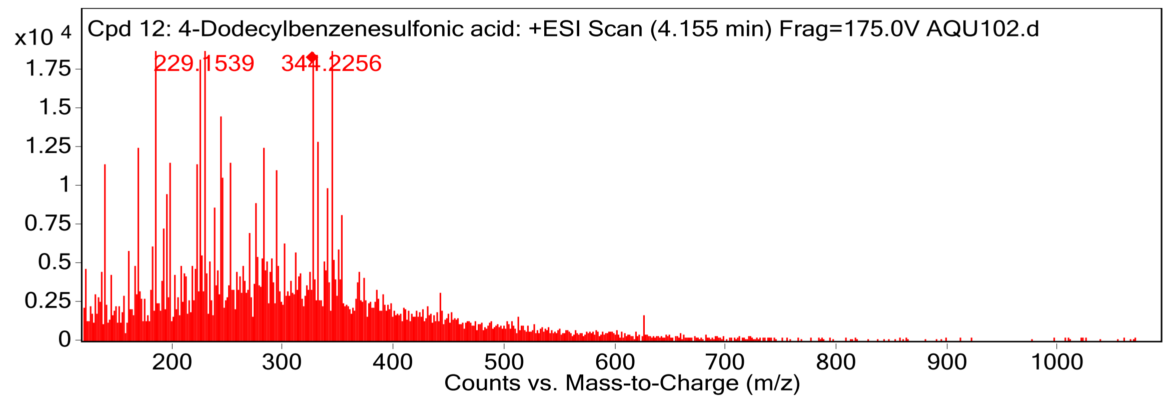

MS Zoomed Spectrum

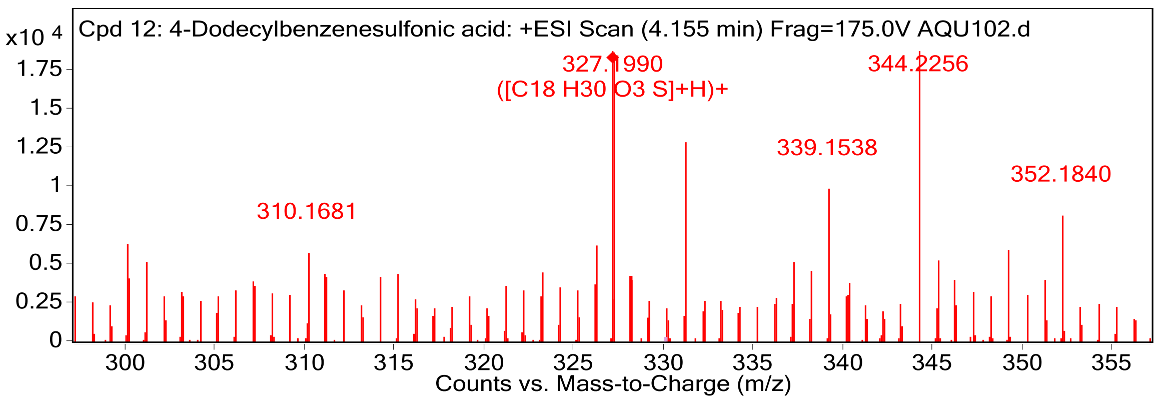

MS Spectrum Peak List

m/z	*Calc m/z*	Diff(ppm)	z	Abund	Formula	Ion
185.1275				18850.26		
224.1276				18239.35		
229.1539			1	34142.95		
243.1332				14548.67		
282.1694			1	12521.51		
327.199	327.1988	-0.47	1	18701.84	C18 H30 O3 S	(M+H)+
328.2023	328.2021	-0.49	1	4047.81	C18 H30 O3 S	(M+H)+
329.1885	329.1984	30.07	1	2727.96	C18 H30 O3 S	(M+H)+
331.2204				12958.61		
344.2256			1	37558.09		

MSMS Spectrum

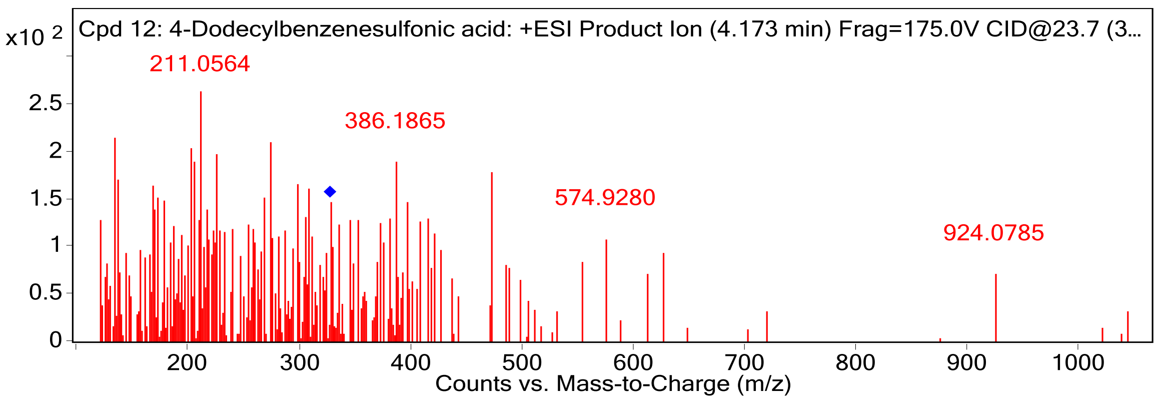

MS/MS Spectrum Peak List

m/z	Abund
134.0463	216.02
137.0851	172.04
202.1257	204.01
205.0629	190.93
211.0564	263.89
225.1202	198.16
273.0511	210.61
298.1666	166.17

m/z	Abund
386.1865	190.11
472.265	180.13

Compound Structure

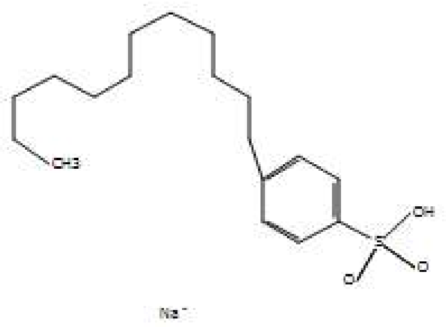

Compound Label	Name	*m/z*	RT	Algorithm	Mass
Cpd 13: Tyrosyl-Glycine	**Tyrosyl-Glycine**	239.1021	4.43	Auto MS/MS	238.0944

MS Spectrum

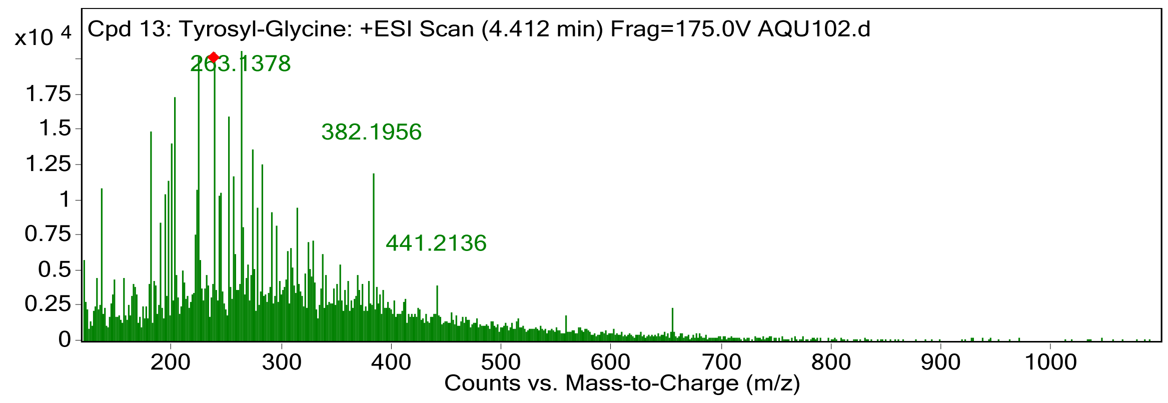

MS Zoomed Spectrum

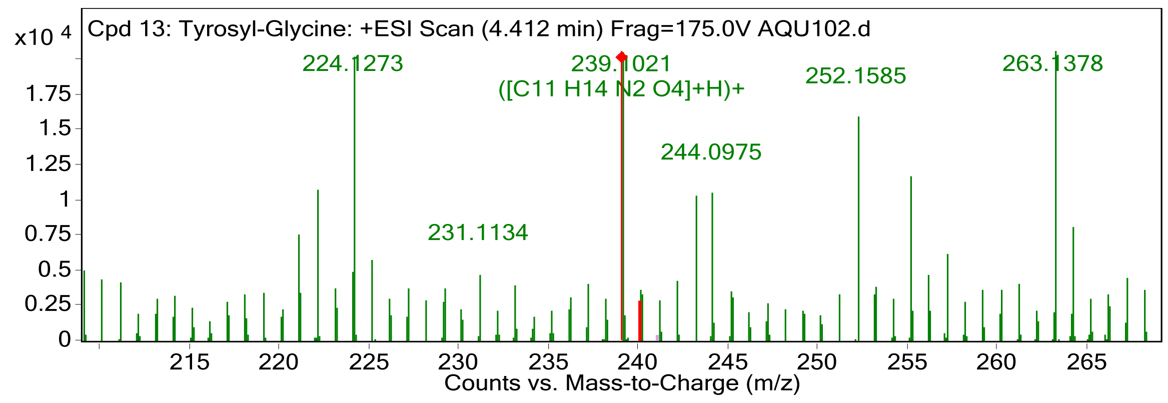

MS Spectrum Peak List

m/z	*Calc m/z*	Diff(ppm)	z	Abund	Formula	Ion
181.0965				14933.3		
200.1065				14046.47		
202.1796				17357.24		
224.1273			1	20177.24		
239.1021	239.1026	2.43	1	20542.13	C11 H14 N2 O4	(M+H)+
240.1029	240.1057	11.77	1	3746.4	C11 H14 N2 O4	(M+H)+
252.1585			1	15958.92		
263.1378			1	46204.53		
273.2159				13691.7		
281.1491				12581.84		

MSMS Spectrum

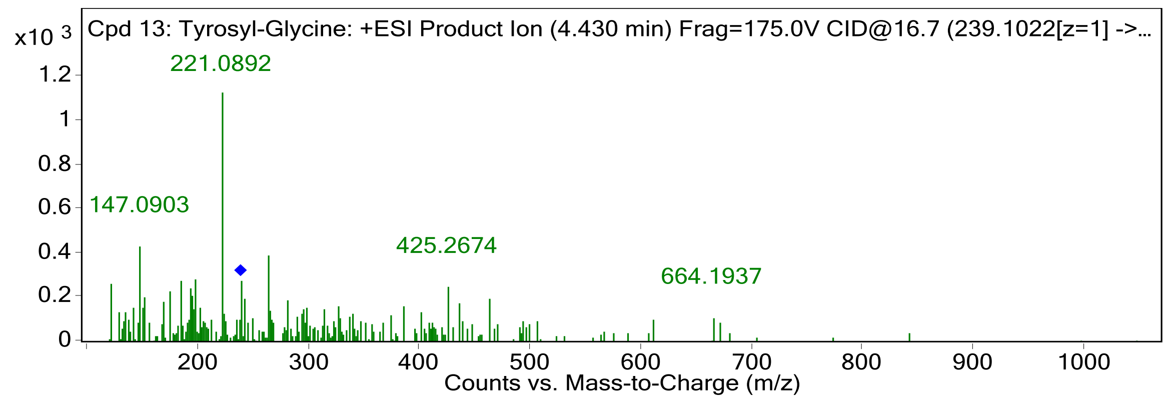

MS/MS Spectrum Peak List

m/z	z	Abund
121.0389		264.26
147.0903		433.11
184.1105		277.72
193.0964	1	240.93
197.1322		283.45
221.0892		1129.28
222.1111		265.82
239.1465		277.12
263.1377		390.34
425.2674		248.38

Compound Structure

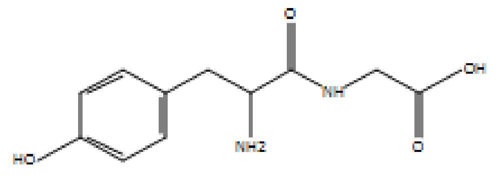

Compound Label	Name	*m/z*	RT	Algorithm	Mass
Cpd 14: Methohexital	**Methohexital**	263.1377	4.466	Auto MS/MS	262.1304

MS Spectrum

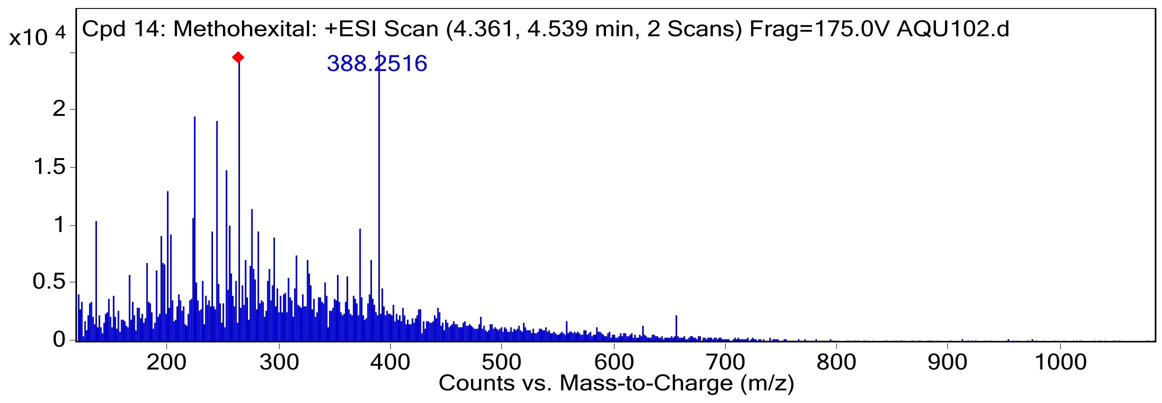

MS Zoomed Spectrum

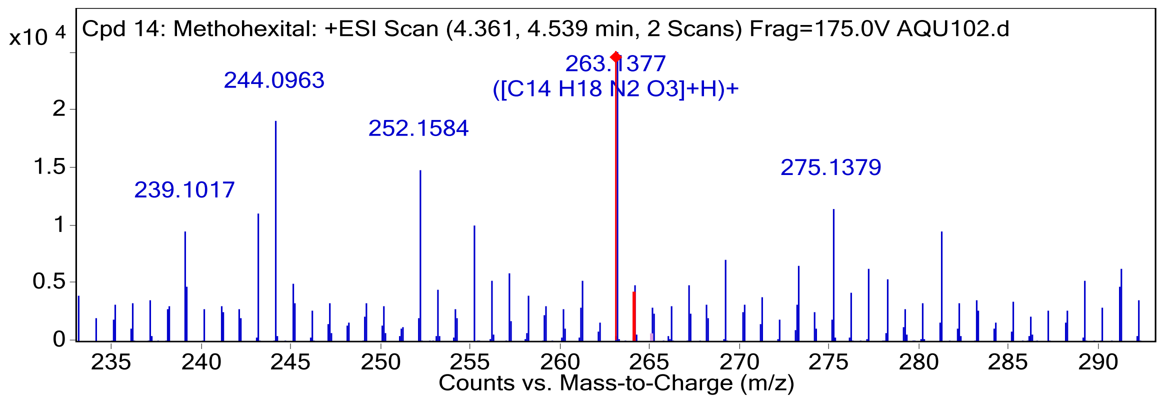

MS Spectrum Peak List

m/z	*Calc m/z*	Diff(ppm)	z	Abund	Formula	Ion
200.1063				13089.33		
222.1118			1	10738.85		
224.1273			1	19449.85		
243.1329				11097.78		
244.0963			1	19080.73		
252.1584			1	14911.77		
263.1377	263.139	5.04	1	25068.78	C14 H18 N2 O3	(M+H)+
264.1408	264.1421	4.96	1	4958.79	C14 H18 N2 O3	(M+H)+
275.1379			1	11510.98		
388.2516			1	29265.55		

MSMS Spectrum

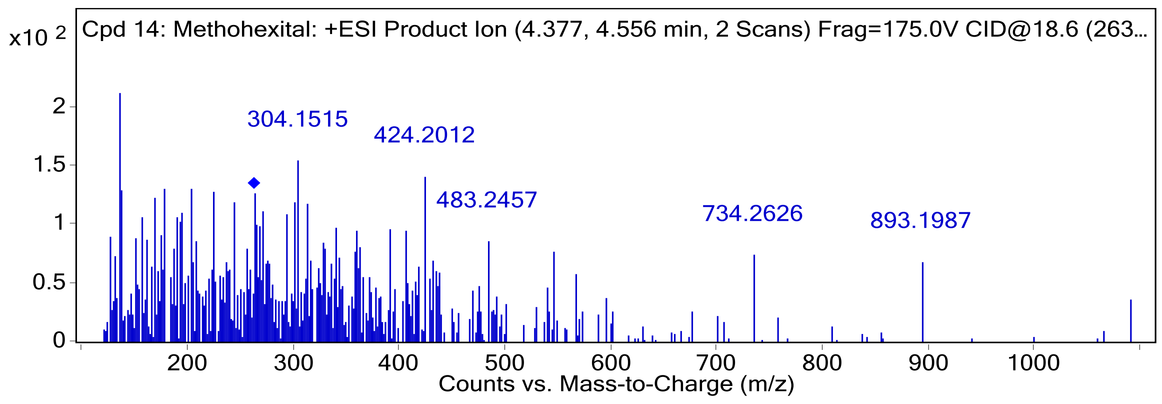

MS/MS Spectrum Peak List

m/z	Abund
136.0756	212.63
137.0721	129.85
168.1043	123.54
177.0934	131.07
202.1783	130.9
224.1263	128.36
263.1773	126.46
300.1845	119.97
304.1515	154.94
424.2012	141.44

Compound Structure

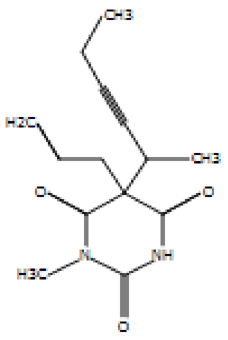

Compound Label	*m/z*	RT	Algorithm
Compound 15	388.2512	4.487	Auto MS/MS

MS Spectrum

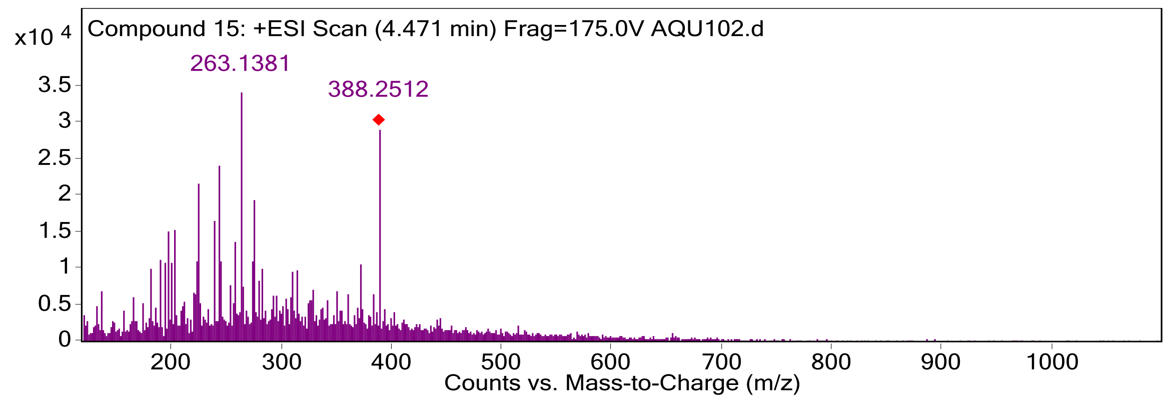

MS Zoomed Spectrum

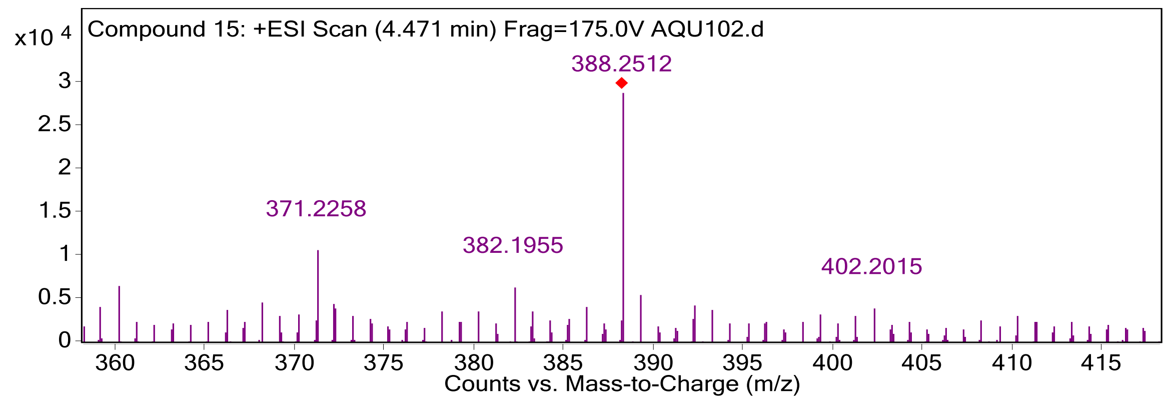

MS Spectrum Peak List

m/z	z	Abund
197.1278		15190.46
202.1796		15316.66
224.1272	1	21577.14
239.1017		16434.77
243.1332		24055.87
263.1381	1	34153.13
275.1378	1	19364.44
388.2512	1	28912.95
389.2542	1	5456.96
390.2522	1	1292.41

MSMS Spectrum

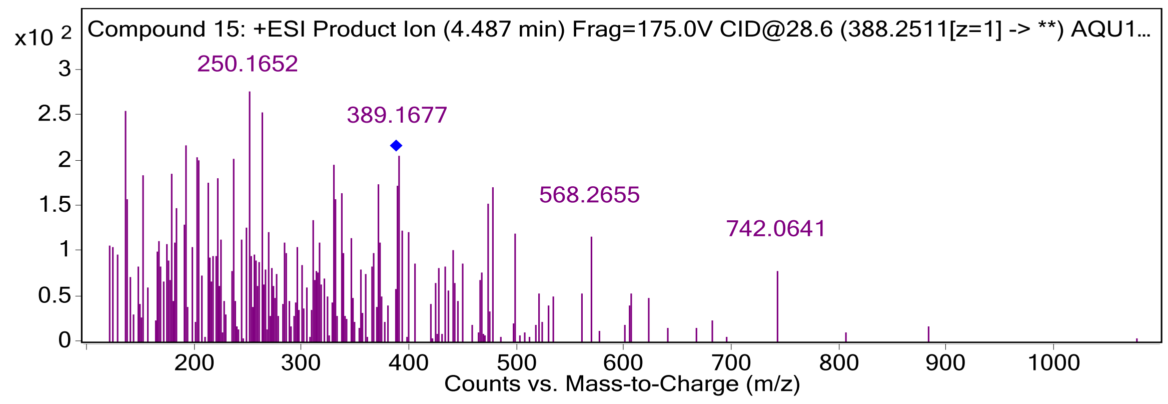

MS/MS Spectrum Peak List

m/z	z	Abund
136.0619		255.08
191.0783		217.33
202.1802		204.08
204.1014		200.51
236.131		203.41
250.1652		276.2
263.144	1	254.28
329.1486		196.02
389.1677		205.35
389.2585		188.18

Compound Label	Name	*m/z*	RT	Algorithm	Mass
Cpd 16: Sinapoylputrescine	**Sinapoylputrescine**	295.1641	4.726	Auto MS/MS	294.1564

MS Spectrum

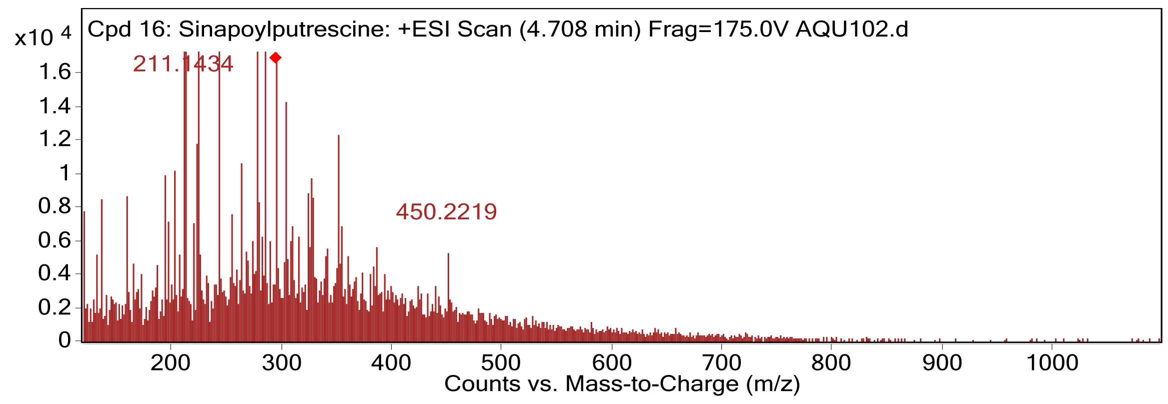

MS Zoomed Spectrum

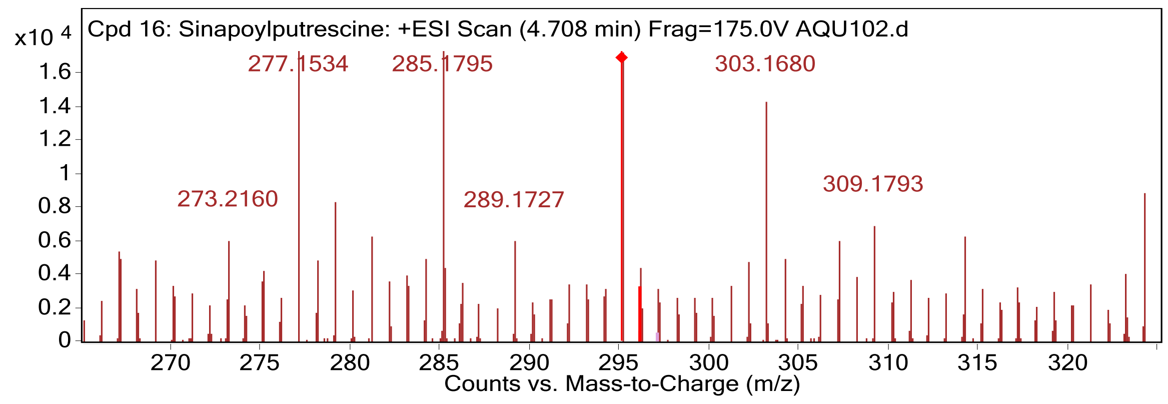

MS Spectrum Peak List

m/z	*Calc m/z*	Diff(ppm)	z	Abund	Formula	Ion
211.1434			1	180274		
212.1464			1	23154.48		
224.1276			1	18031.07		
243.1331			1	20366.51		
277.1534			1	21892		
285.1795				22336.21		
295.1641	295.1652	3.8	1	17253.5	C15 H22 N2 O4	(M+H)+
296.1649	296.1684	11.84	1	4486.21	C15 H22 N2 O4	(M+H)+
303.168			1	14332.31		
351.2252			1	12341.42		

MSMS Spectrum

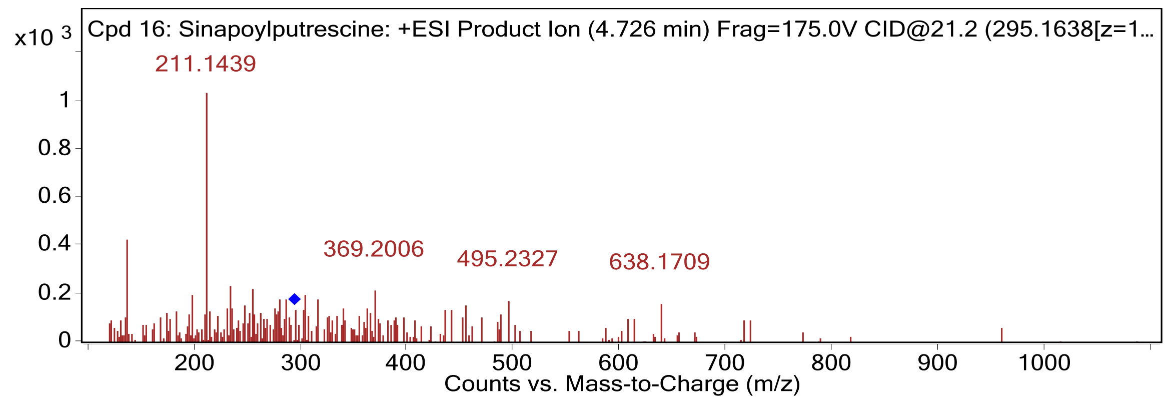

MS/MS Spectrum Peak List

m/z	Abund
136.0745	430.28
198.11	195.22
211.1439	1036.24
234.1267	233.32
254.1019	219.97
279.1731	178.56
285.1801	181.42
303.1654	201.02
316.1953	178.79
369.2006	215.47

Compound Structure

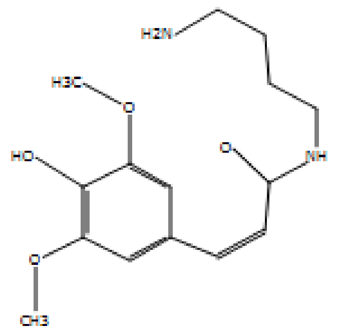

Compound Label	Name	*m/z*	RT	Algorithm	Mass
Cpd 17: L,L-Cyclo(leucylprolyl)	**L,L-Cyclo(leucylprolyl)**	211.1433	4.76	Auto MS/MS	210.1361

MS Spectrum

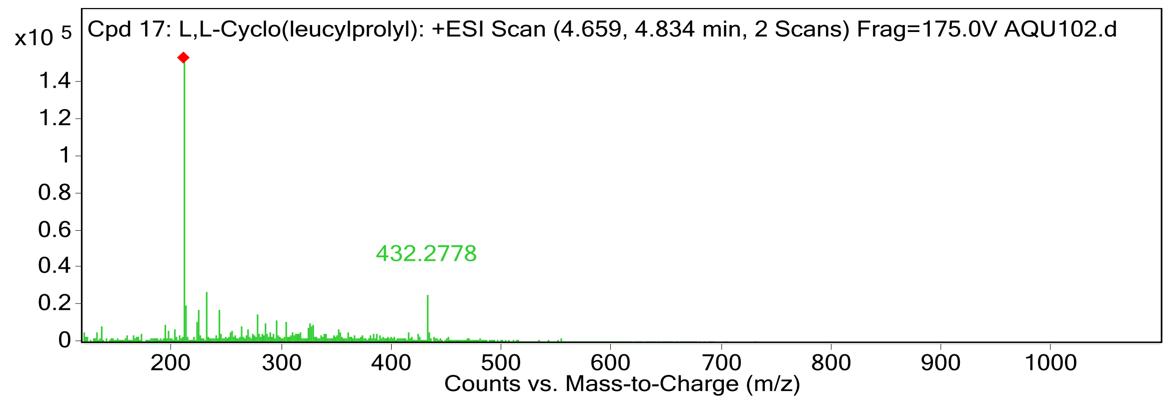

MS Zoomed Spectrum

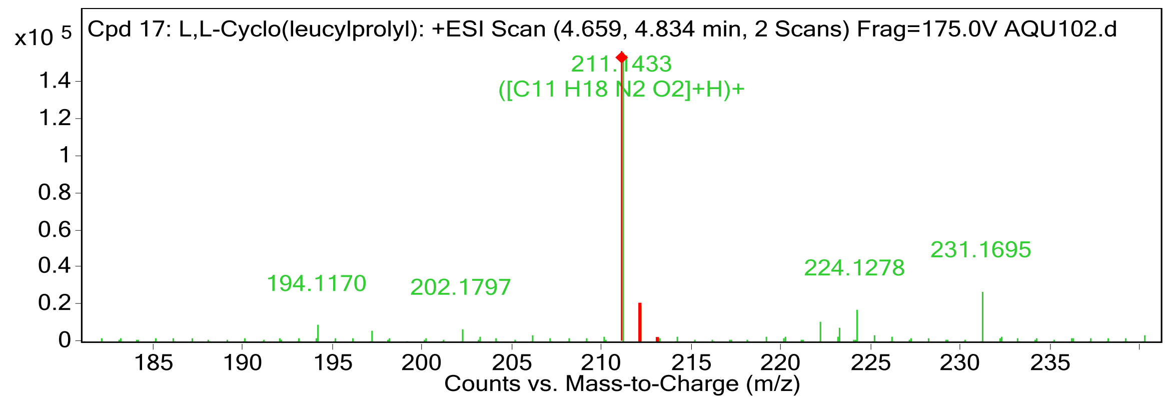

MS Spectrum Peak List

m/z	*Calc m/z*	Diff(ppm)	z	Abund	Formula	Ion
211.1433	211.1441	3.86	1	156360.61	C11 H18 N2 O2	(M+H)+
212.1464	212.1472	3.48	1	20241.91	C11 H18 N2 O2	(M+H)+
213.1519	213.1495	-11.29	1	2793.88	C11 H18 N2 O2	(M+H)+
222.1478				11368.15		
224.1278			1	17757.22		
231.1695				27572.02		
243.1322			1	17972.8		
277.1532			1	15530.69		
295.1639			1	12216.27		
432.2778			1	25558.64		

MSMS Spectrum

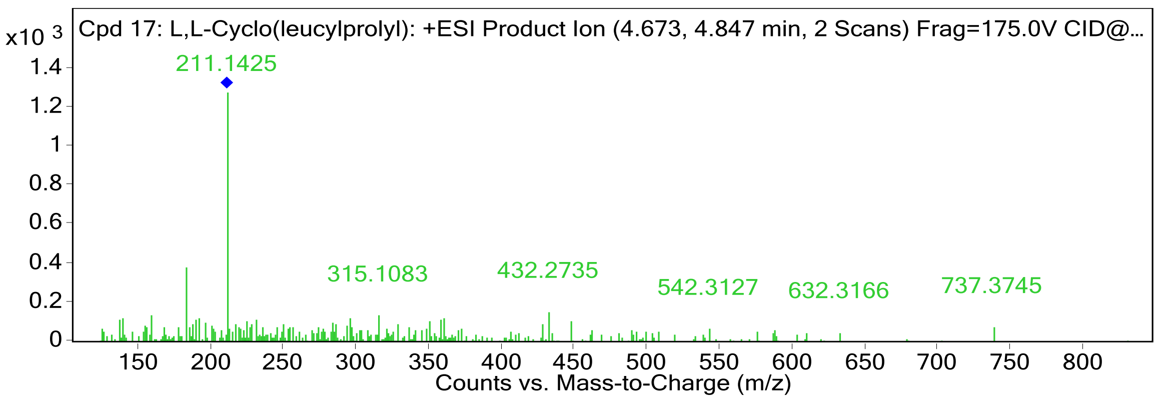

MS/MS Spectrum Peak List

m/z	z	Abund
139.0741		121.43
159.0443		139.76
183.1472		381.55
192.0622		119.34
211.1425	1	1276.47
295.1618		122.05
315.1083		134.61
358.0969		117.09
360.1984		122.39
432.2735		153.11

Compound Structure

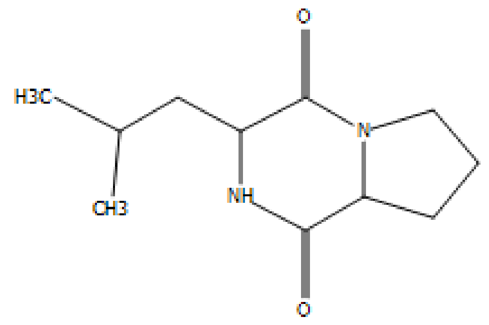

Compound Label	Name	*m/z*	RT	Algorithm	Mass
Cpd 18: Istamycin C1	**Istamycin C1**	432.2778	4.778	Auto MS/MS	431.2704

MS Spectrum

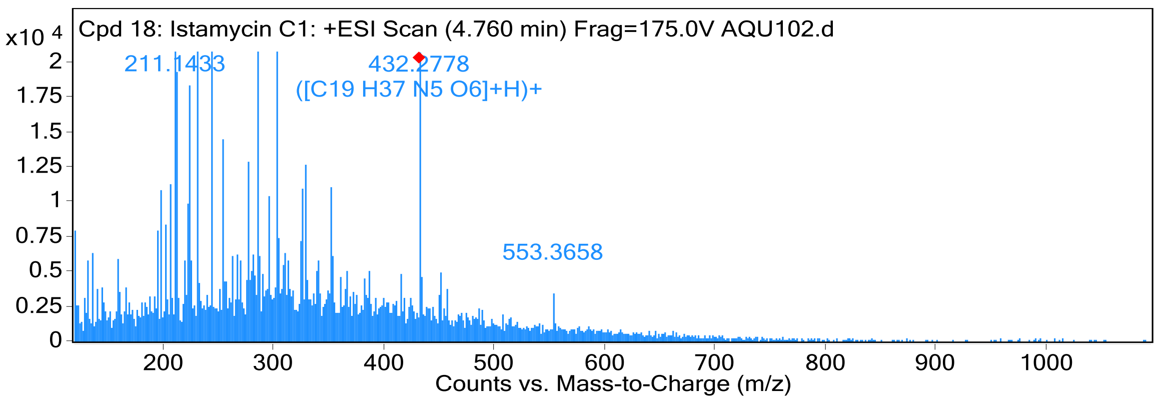

MS Zoomed Spectrum

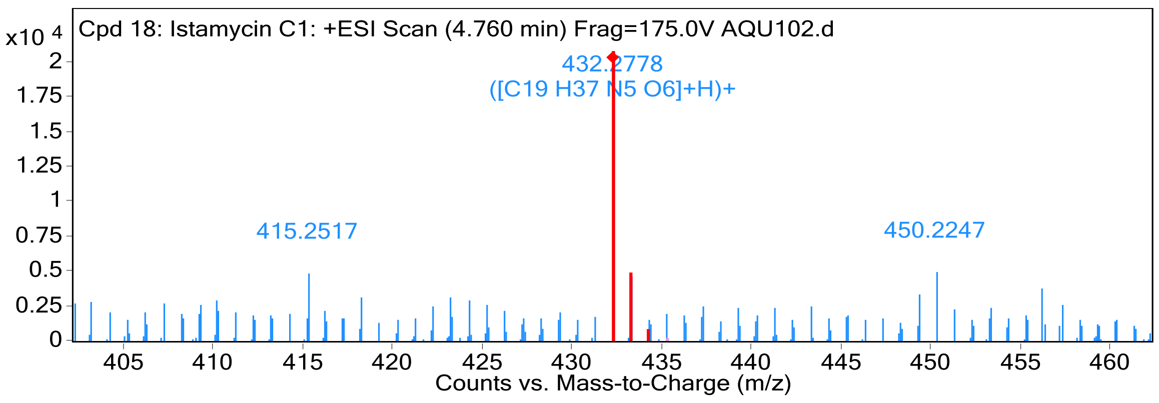

MS Spectrum Peak List

m/z	*Calc m/z*	Diff(ppm)	z	Abund	Formula	Ion
211.1433			1	187614.45		
212.1464			1	19357.99		
224.1277			1	18346.85		
231.1696			1	21748.46		
243.133			1	42324.64		
285.1797			1	36350.84		
303.1699			1	25293.95		
432.2778	432.2817	9.01	1	20712.63	C19 H37 N5 O6	(M+H)+
433.2816	433.2846	6.94	1	4667.78	C19 H37 N5 O6	(M+H)+
434.2779	434.287	20.93	1	1315.01	C19 H37 N5 O6	(M+H)+

MSMS Spectrum

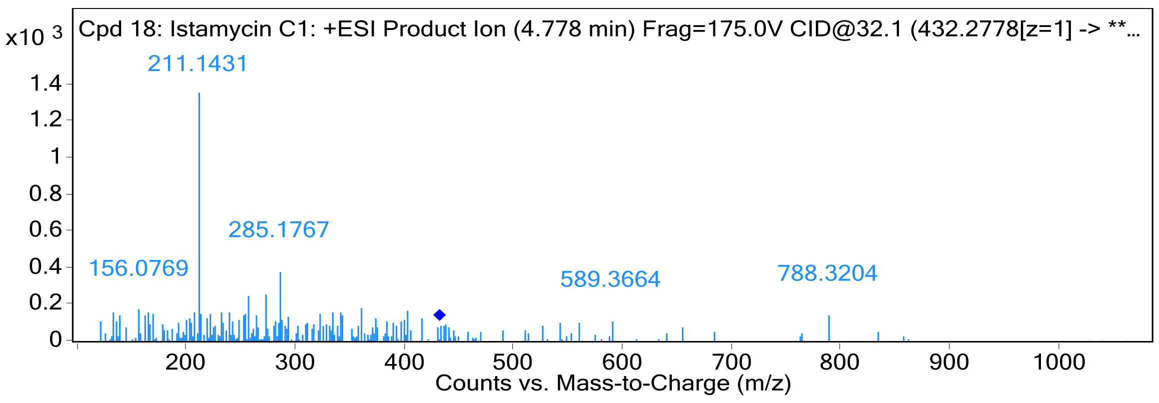

MS/MS Spectrum Peak List

m/z	z	Abund
156.0769		174.94
211.1431		1359.8

232.0601		164.06
238.1149		163.56
256.1671		252
272.1619		256.36
285.1767	1	385.82
334.1254		162.88
360.207		185.05
401.1607		173.79

Compound Structure

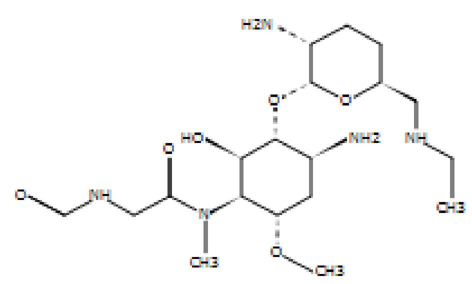

Compound Label	Name	*m/z*	RT	Algorithm	Mass
Cpd 19: L,L-Cyclo(leucylprolyl)	**L,L-Cyclo(leucylprolyl)**	211.1433	5.099	Auto MS/MS	210.1361

MS Spectrum

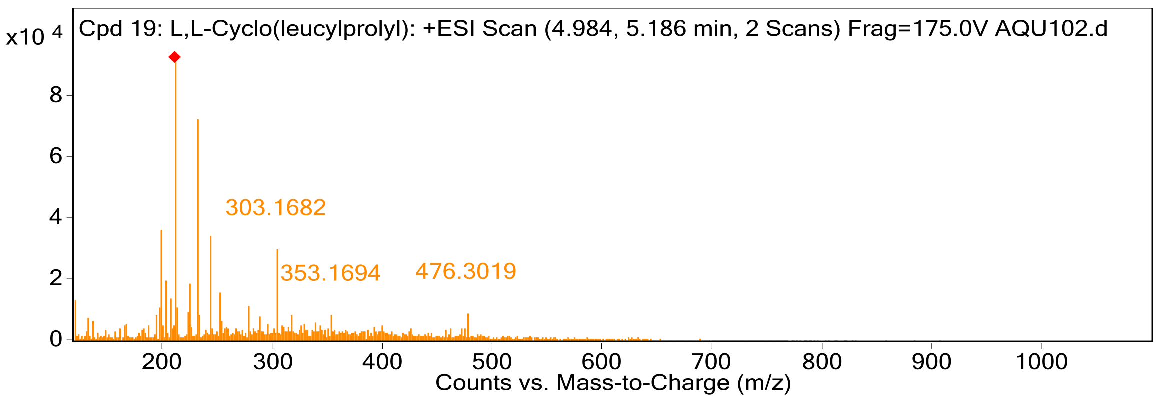

MS Zoomed Spectrum

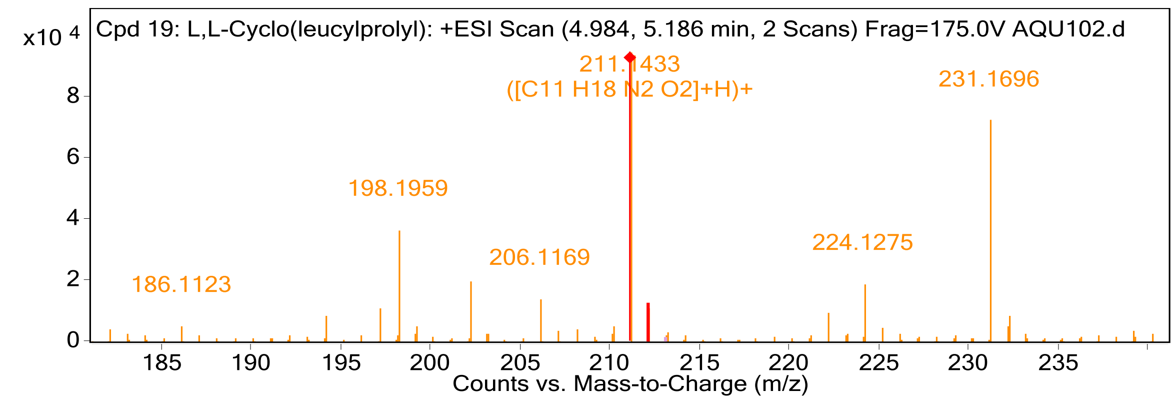

MS Spectrum Peak List

m/z	*Calc m/z*	Diff(ppm)	z	Abund	Formula	Ion
198.1959			1	36801.41		
202.1796				19844.53		
206.1169			1	14223.43		
211.1433	211.1441	3.63	1	94597.45	C11 H18 N2 O2	(M+H)+
212.1464	212.1472	3.59	1	11111.33	C11 H18 N2 O2	(M+H)+
224.1275			1	19103.19		
231.1696			1	72547.38		
243.133			1	34398.95		
252.0857				16319.68		
303.1682			1	30238.89		

MSMS Spectrum

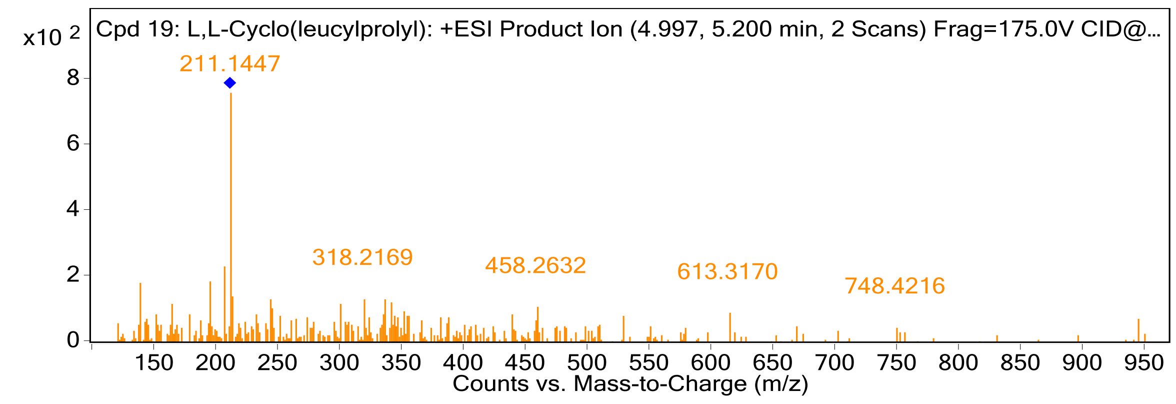

MS/MS Spectrum Peak List

m/z	z	Abund
138.1274		184.24
163.0759		119.4
194.1174		187.13
206.1212		231
211.1447	1	762.17
212.1423	1	140.48
243.1306		132.54
318.2169		132.64
335.1152		132.2
341.1528	1	121.22

Compound Structure

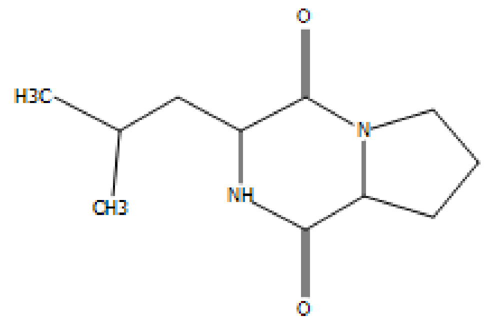

Compound Label	Name	*m/z*	RT	Algorithm	Mass
Cpd 20: trans,trans-Farnesyl phosphate	**trans,trans-Farnesyl phosphate**	303.1692	5.124	Auto MS/MS	302.1614

MS Spectrum

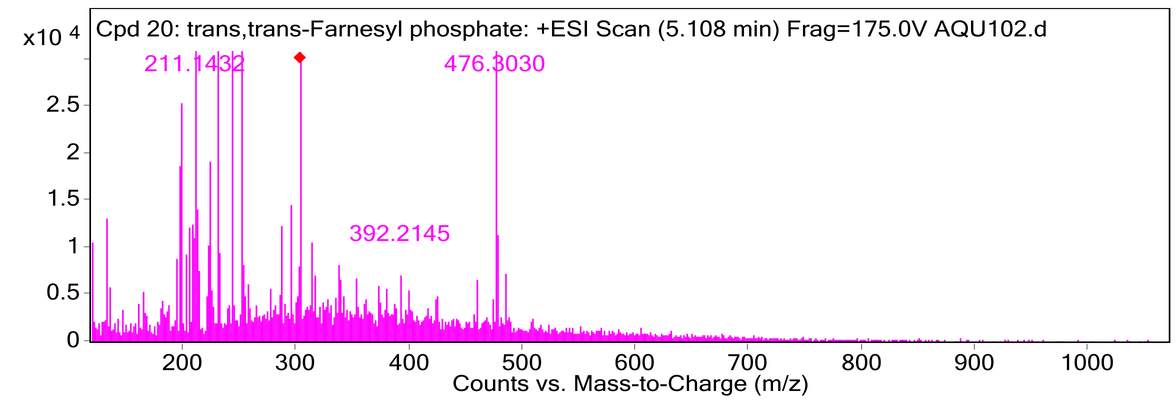

MS Zoomed Spectrum

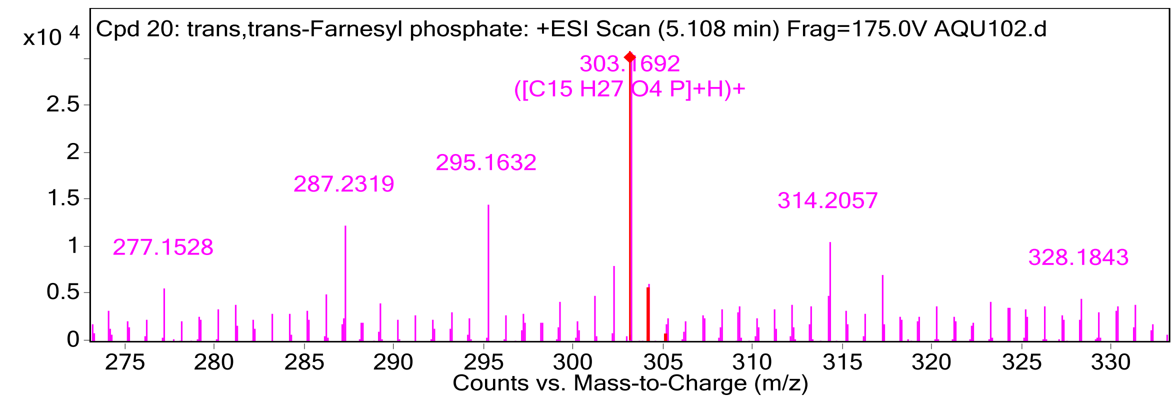

MS Spectrum Peak List

m/z	*Calc m/z*	Diff(ppm)	z	Abund	Formula	Ion

198.1958			1	25346.03		
211.1432			1	115681.91		
224.1276			1	19139.12		
231.1693			1	30840.5		
243.1333			1	86355.98		
252.0859			1	48242.75		
303.1692	303.172	9.3	1	30742.21	C15 H27 O4 P	(M+H)+
304.1731	304.1754	7.59	1	6161.93	C15 H27 O4 P	(M+H)+
305.1669	305.1778	35.85	1	2564.22	C15 H27 O4 P	(M+H)+
476.303			1	40952.27		

MSMS Spectrum

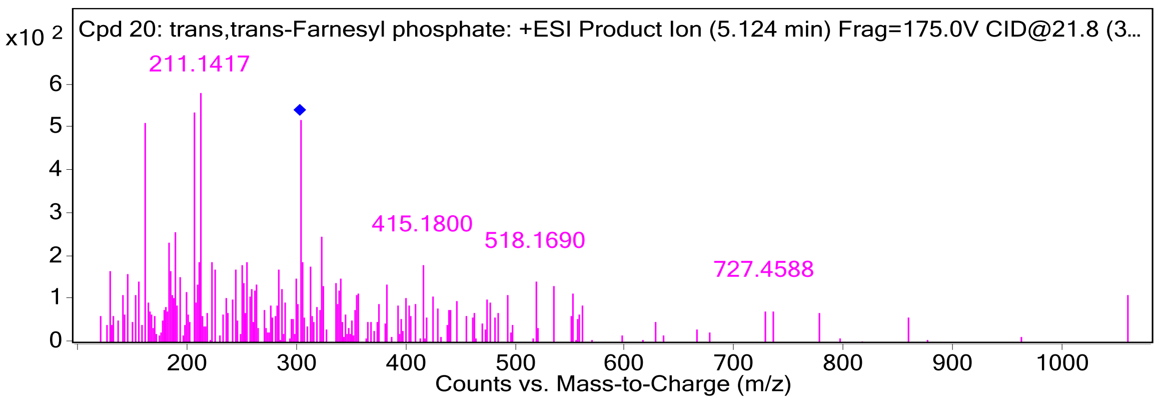

MS/MS Spectrum Peak List

m/z	z	Abund
160.1099		512.68
182.0967		234.43
188.0894	1	256.5
206.1166	1	537.52
210.1517		188.83
211.1417		582.76
253.1042		188.04
303.1684	1	519.12
304.185	1	189.07
322.1045		246.45

Compound Structure

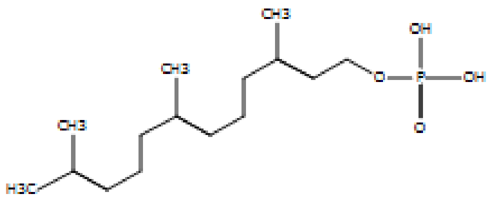

**Compound Label**	***m/z***	**RT**	**Algorithm**
Compound 21	520.3293	5.303	Auto MS/MS

MS Spectrum

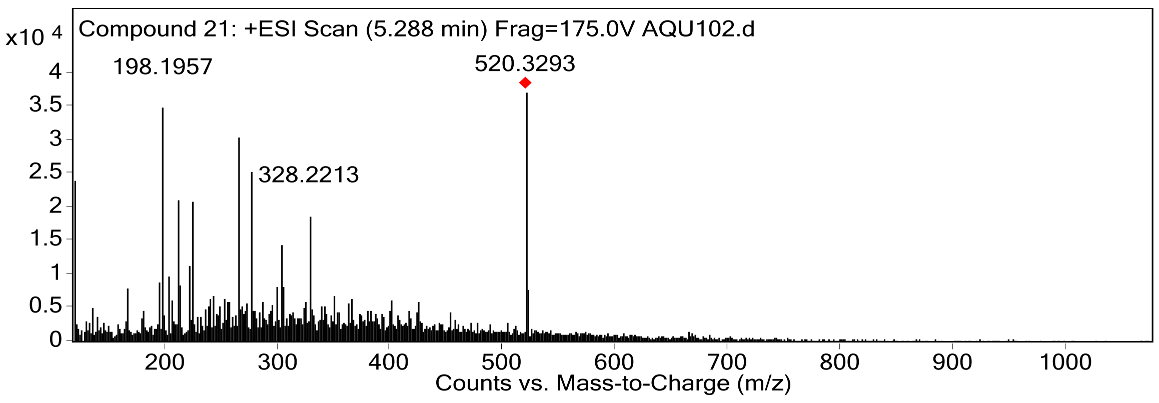

MS Zoomed Spectrum

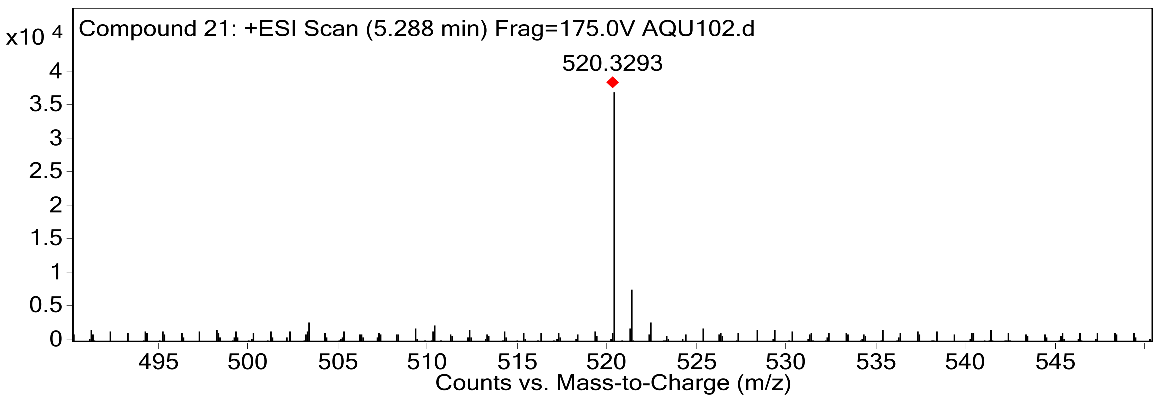

MS Spectrum Peak List

m/z	z	Abund
120.0809		24046.66
198.1957	1	34828.11
211.1431	1	21125.97
224.128	1	20824
265.1534	1	30311.63
277.1174	1	25249.04
520.3293	1	37124.65
521.3329	1	7803.45
522.3324	1	2978.92
523.3427	1	418.99

MSMS Spectrum

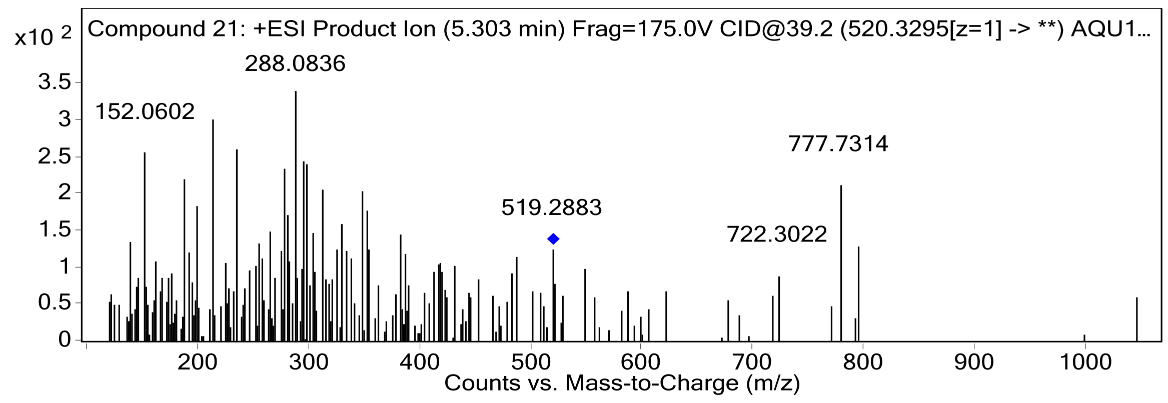

MS/MS Spectrum Peak List

m/z	Abund
152.0602	256.88
186.954	220.31
213.1619	301.48
234.1259	261.35
277.1165	235.69
287.1382	245.18
288.0836	339.53
294.1903	244.72
297.0892	241.88
777.7314	213.09

Compound Label	Name	*m/z*	RT	Algorithm	Mass
Cpd 22: 17beta-Nitro-5alpha-androstane	**17beta-Nitro-5alpha-androstane**	328.2215	5.39	Auto MS/MS	305.2322

MS Spectrum

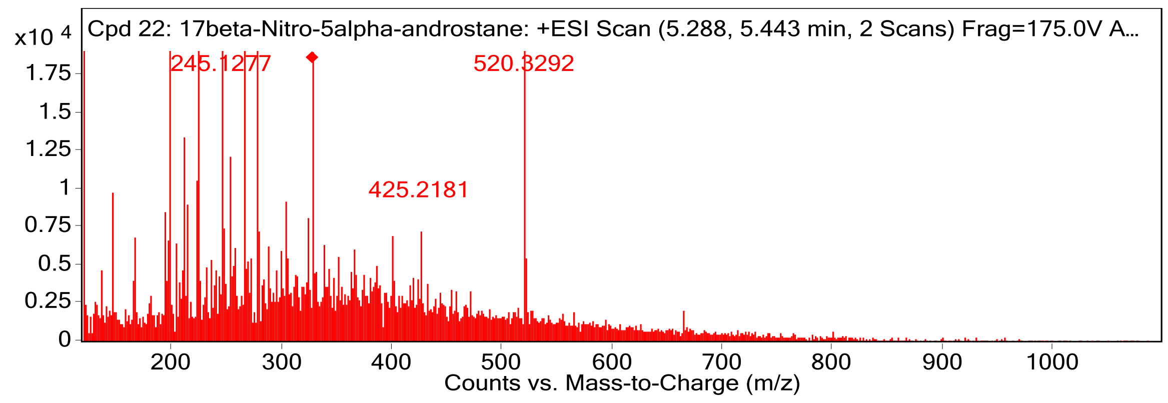

MS Zoomed Spectrum

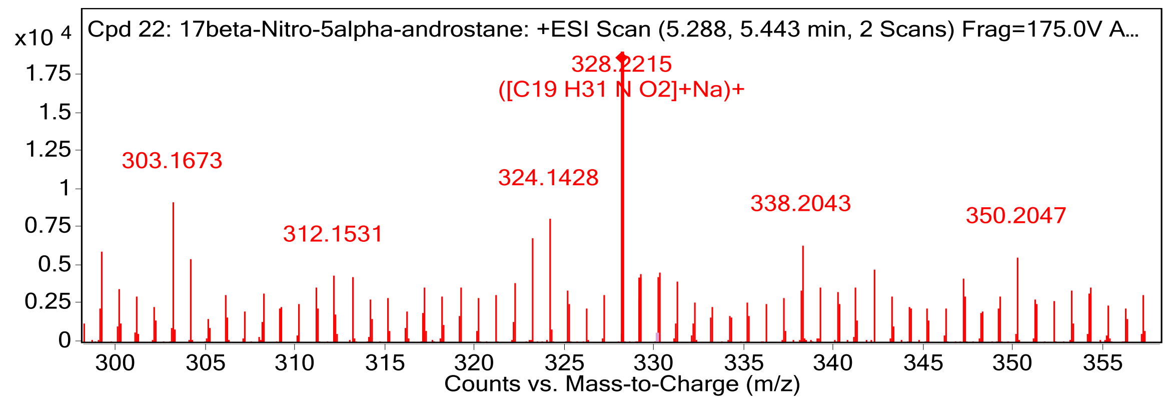

MS Spectrum Peak List

m/z	*Calc m/z*	Diff(ppm)	z	Abund	Formula	Ion
120.0809				24679.52		
198.1958				20600.99		
224.1277			1	19093.21		
245.1277			1	86271.3		
246.1315			1	16141.34		
265.1533			1	20642.69		
277.1172			1	37370.32		
328.2215	328.2247	9.74	1	18960.85	C19 H31 N O2	(M+Na)+
329.2244	329.228	10.86	1	4186.41	C19 H31 N O2	(M+Na)+
520.3292			1	21559.74		

MSMS Spectrum

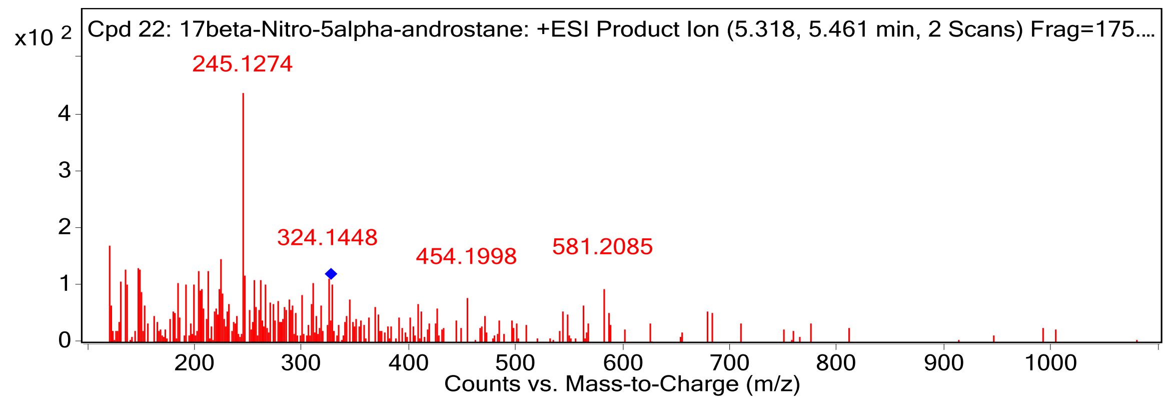

MS/MS Spectrum Peak List

m/z	z	Abund
120.0802		170.02
135.0806		127.6
146.0573		132.08
148.0603		128.59
203.1221		125.03
211.178		124.87
224.1281		145.84
245.1274	1	438.88
246.1376	1	117.31
324.1448		111.51

Compound Structure

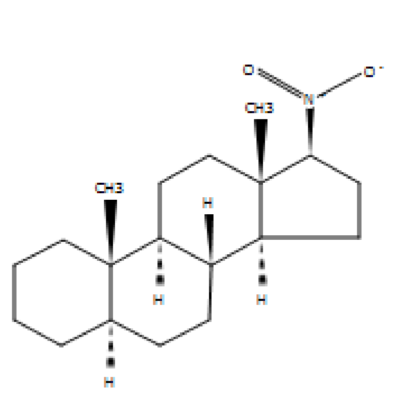

Compound Label	Name	*m/z*	RT	Algorithm	Mass
Cpd 23: Hexyl 2-furoate	**Hexyl 2-furoate**	197.1171	5.595	Auto MS/MS	196.1096

MS Spectrum

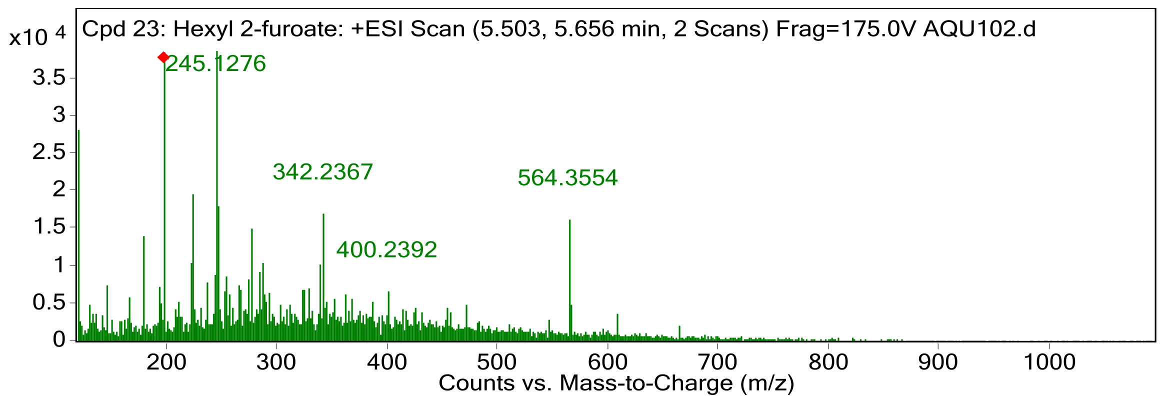

MS Zoomed Spectrum

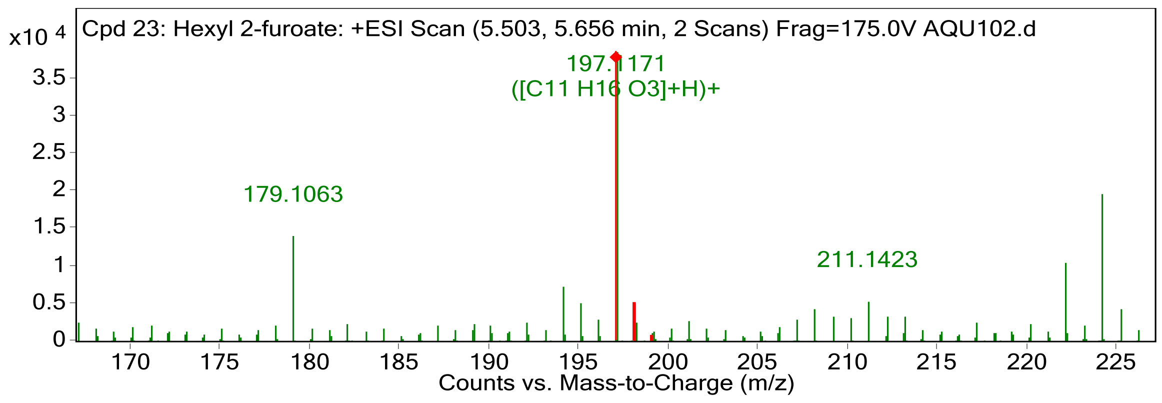

MS Spectrum Peak List

m/z	*Calc m/z*	Diff(ppm)	z	Abund	Formula	Ion
120.0808				28150.41		
197.1171	197.1172	0.62	1	38479.11	C11 H16 O3	(M+H)+
198.1197	198.1206	4.74	1	5251.13	C11 H16 O3	(M+H)+
199.119	199.1228	19.33	1	1478.16	C11 H16 O3	(M+H)+
224.1274			1	19572.19		
245.1276			1	96695.23		
246.1311			1	18062.62		
277.1172				15127.89		
342.2367			1	17101.88		
564.3554			1	16314.56		

MSMS Spectrum

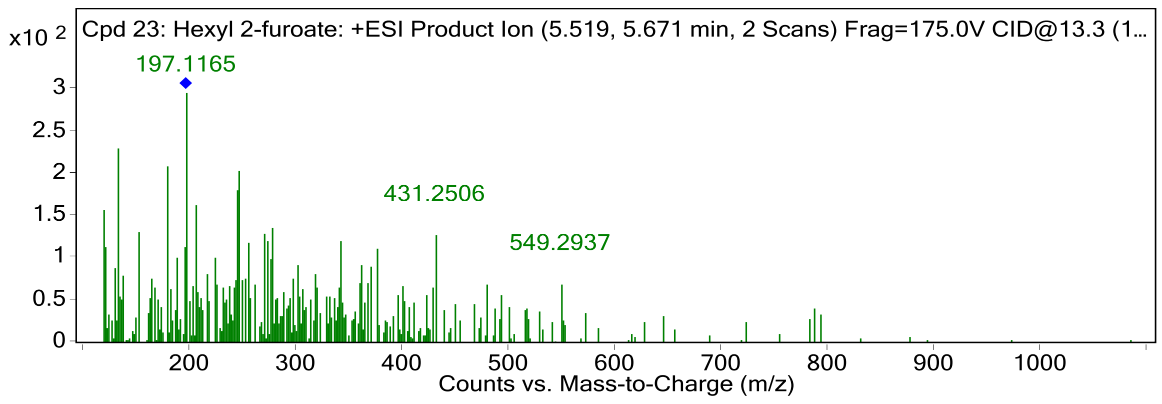

MS/MS Spectrum Peak List

m/z	Abund
120.085	158.22
133.1004	229.6
153.1125	131.43
179.1063	209.27
197.1165	295.95
207.112	163.25
245.1281	180.75
246.1284	203.22
270.1843	129.89
278.1364	136.01

Compound Structure

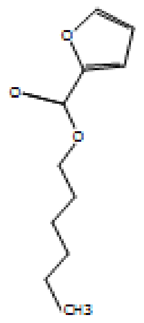

Compound Label	Name	*m/z*	RT	Algorithm	Mass

Cpd 24: 3-Oxo-12,18-ursadien-28-oic acid	**3-Oxo-12,18-ursadien-28-oic acid**	453.3405	5.764	Auto MS/MS	452.3332

MS Spectrum

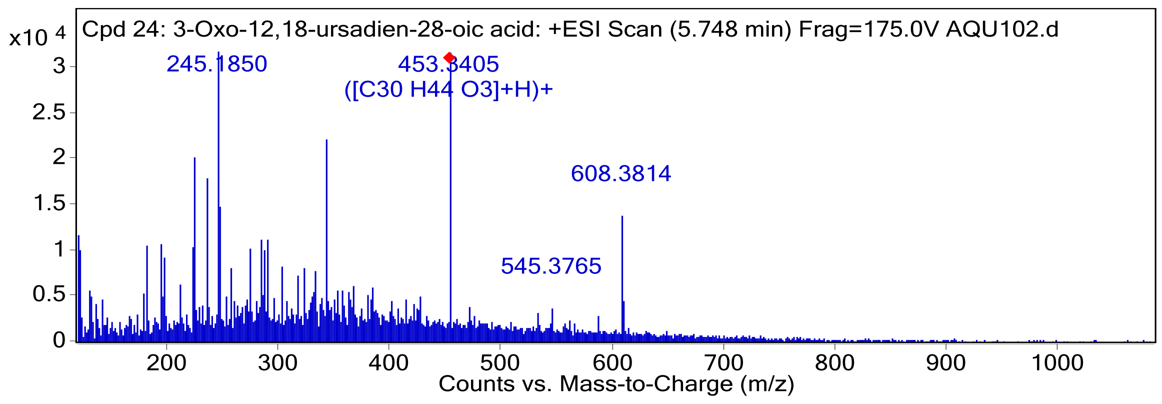

MS Zoomed Spectrum

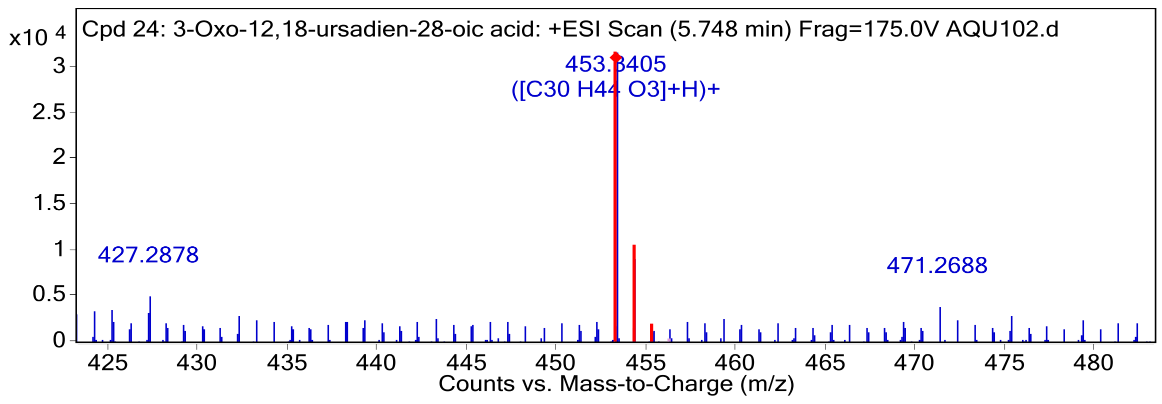

MS Spectrum Peak List

m/z	*Calc m/z*	Diff(ppm)	z	Abund	Formula	Ion
224.1274			1	20222.91		
236.1635			1	17888.31		
245.1276			1	32131.32		
245.185			1	77162.96		
247.1426				14888.96		
342.237			1	22136.72		
453.3405	453.3363	-9.31	1	31616.45	C30 H44 O3	(M+H)+
454.3442	454.3397	-9.88	1	9081.8	C30 H44 O3	(M+H)+
455.3429	455.3429	0.01	1	1271	C30 H44 O3	(M+H)+
608.3814			1	13918.83		

MSMS Spectrum

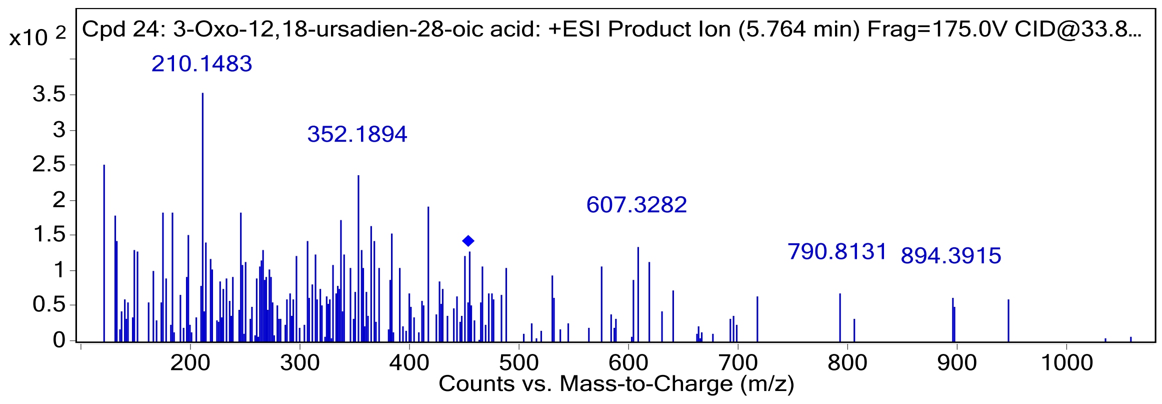

MS/MS Spectrum Peak List

m/z	Abund
120.0805	252.08
130.0649	180.55
174.0373	183.78
182.1523	184.97
209.168	200.73
210.1483	355.38
245.1861	184.88
336.2256	173.83
352.1894	237.01
415.2946	194.13

Compound Structure

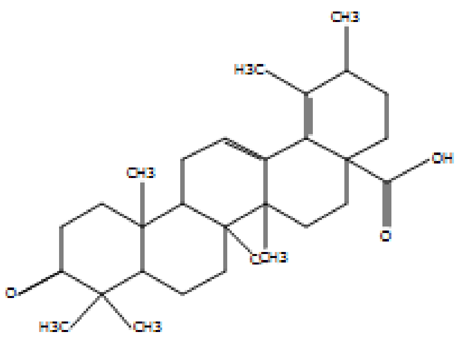

Compound Label	Name	*m/z*	RT	Algorithm	Mass
Cpd 25: Hexyl 2-furoate	**Hexyl 2-furoate**	197.1177	5.986	Auto MS/MS	196.1102

MS Spectrum

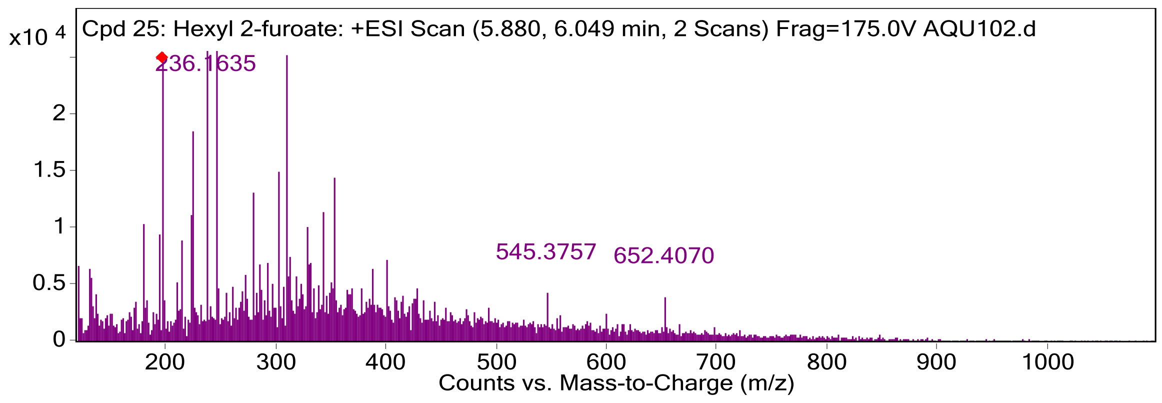

MS Zoomed Spectrum

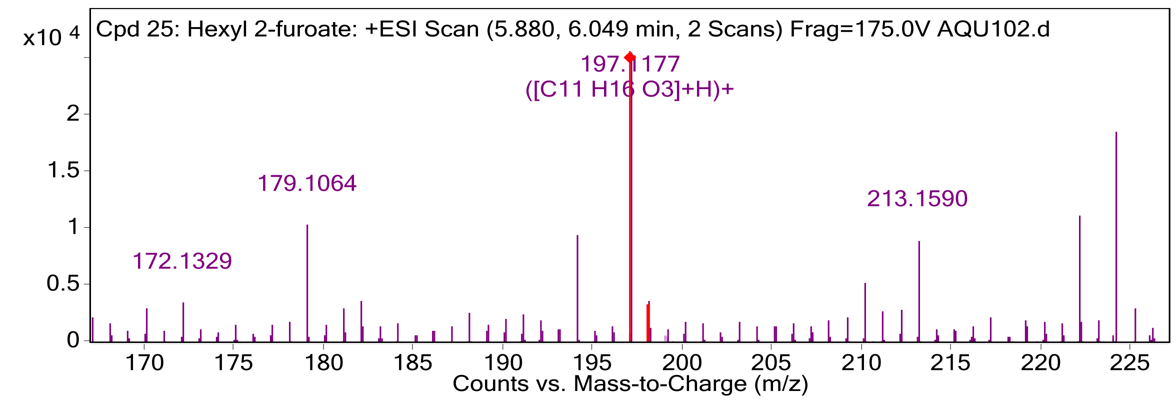

MS Spectrum Peak List

m/z	*Calc m/z*	Diff(ppm)	z	Abund	Formula	Ion
197.1177	197.1172	-2.59	1	25591.1	C11 H16 O3	(M+H)+
198.1191	198.1206	7.63	1	3744.01	C11 H16 O3	(M+H)+
224.1276				18636.96		
236.1635			1	28359.13		
245.1849			1	27253.25		
279.1684				13174.5		
301.2472				15030.41		
309.179			1	25371.27		
342.236				11430.76		
352.2211			1	14517.1		

MSMS Spectrum

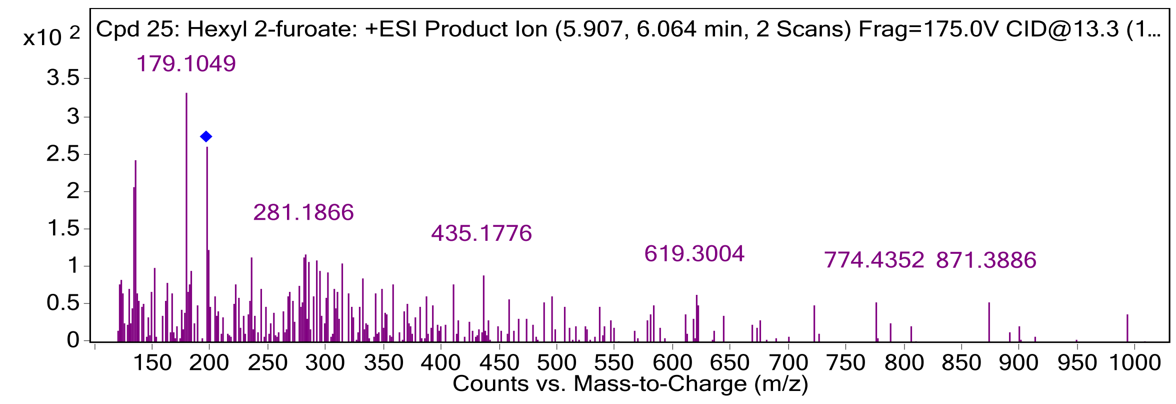

MS/MS Spectrum Peak List

m/z	z	Abund
133.101	1	208.19
135.1168		243.93
179.1049	1	333.61
197.12		260.65
198.1318		123.72
198.1974		108.79
235.1096		114.46

m/z	z	Abund
280.118		113.28
281.1866		118.12
291.1474		110.46

Compound Structure

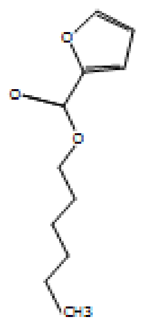

Compound Label	Name	*m/z*	RT	Algorithm	Mass
Cpd 26: 2-Pentadecylfuran	**2-Pentadecylfuran**	301.2469	6.069	Auto MS/MS	278.2575

MS Spectrum

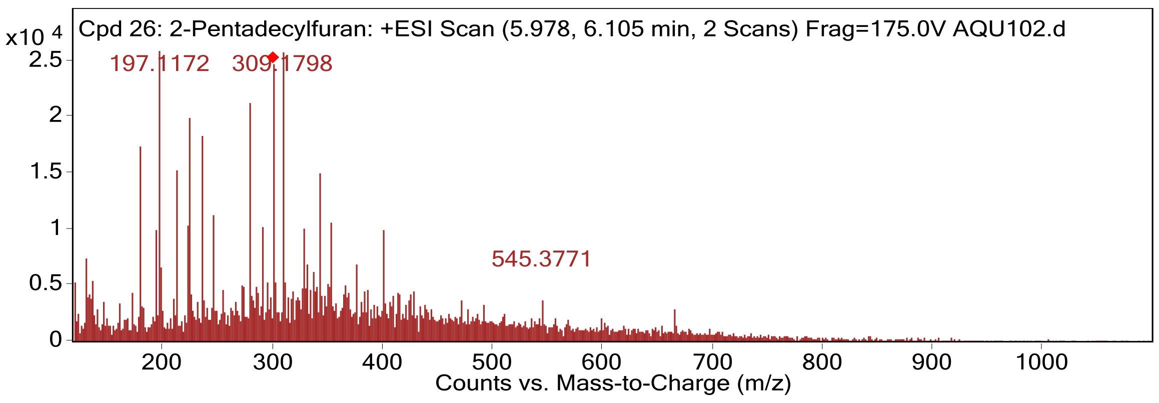

MS Zoomed Spectrum

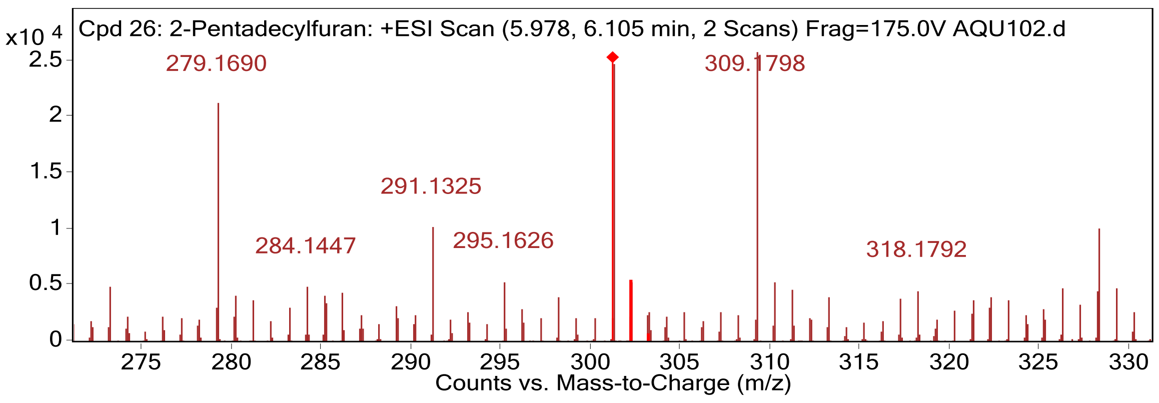

MS Spectrum Peak List

m/z	*Calc m/z*	Diff(ppm)	z	Abund	Formula	Ion
179.1062				17337.28		
197.1172			1	50753.2		
213.1589				15204.33		
224.1275			1	19866.42		
236.1636			1	18362.97		
279.169			1	21209.56		
301.2469	301.2502	10.93	1	24673.59	C19 H34 O	(M+Na)+
302.2497	302.2536	12.74	1	5284.03	C19 H34 O	(M+Na)+
303.2519	303.2568	16.09	1	1037.2	C19 H34 O	(M+Na)+
309.1798			1	25731.68		

MSMS Spectrum

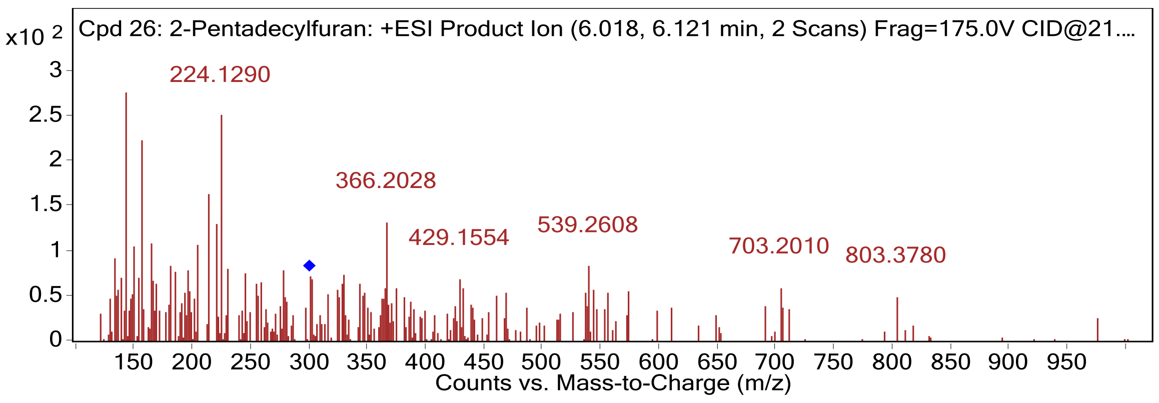

MS/MS Spectrum Peak List

m/z	Abund
133.1007	92.4
142.1602	276.71
149.0244	105
156.1738	223.98
165.055	109.18
204.0944	107.56
213.1599	163.04
220.1167	129.82
224.129	250.55
366.2028	132.79

Compound Structure

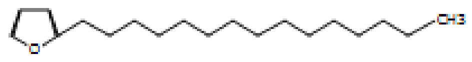

Compound Label	*m/z*	RT	Algorithm
Compound 27	701.4903	6.676	Auto MS/MS

MS Spectrum

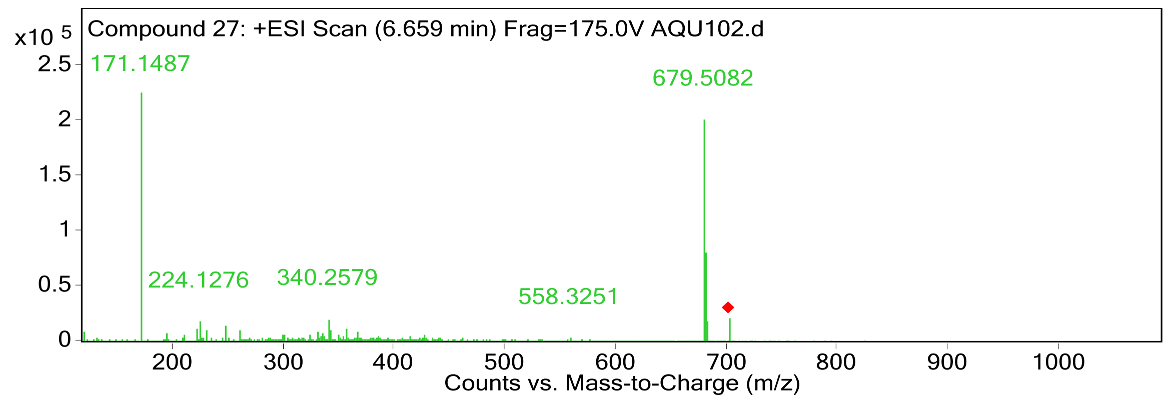

MS Zoomed Spectrum

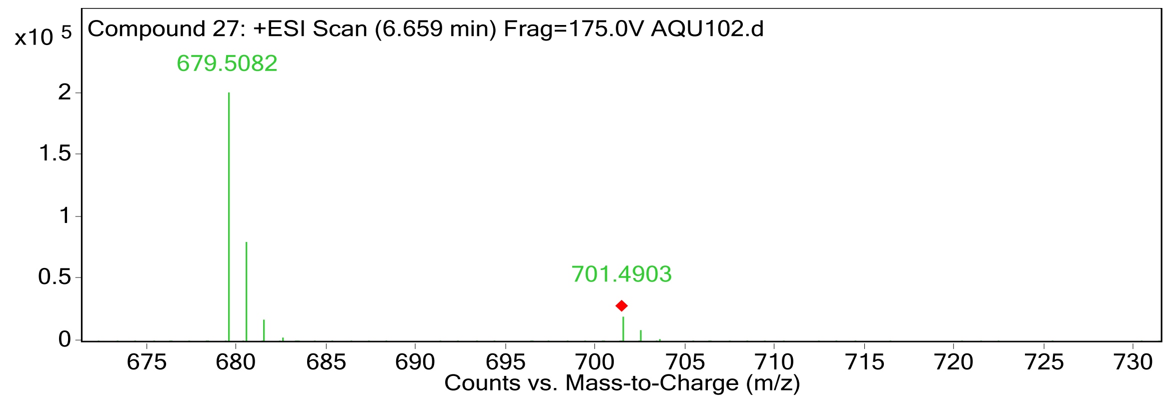

MS Spectrum Peak List

m/z	z	Abund
171.1487	1	225645.06
172.1519	1	26311.09
340.2579	2	20684.95
679.5082	1	201390.45
680.5112	1	80709.67
681.5142	1	18506.81
701.4903	1	21013.31
702.4922	1	9876.73
703.4951	1	1949.01
704.5063	1	302.01

MSMS Spectrum

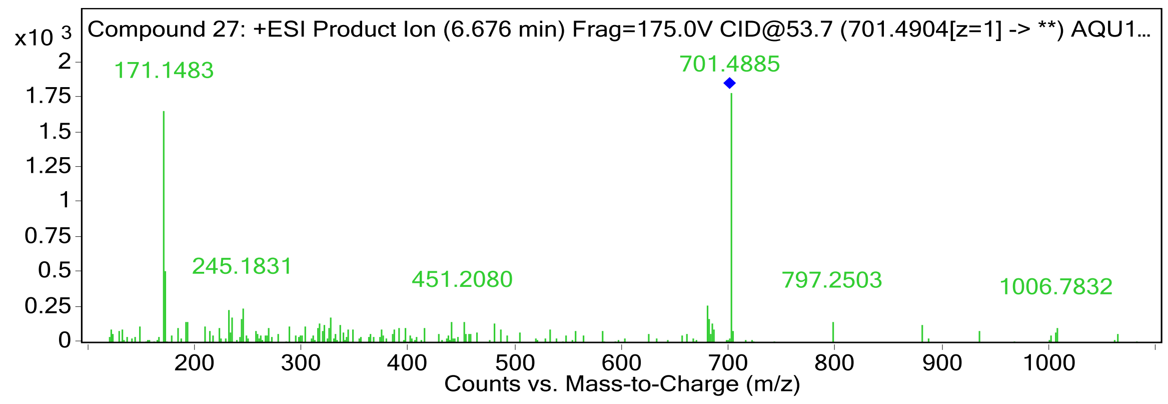

MS/MS Spectrum Peak List

m/z	z	Abund
171.1483	1	1653.43
172.1512	1	508.66
231.1692		235.38
235.0922		185.43
243.1082		170.19
245.1831		249.33
327.1901		179.88
679.5018	1	263.12
680.4977	1	166.09
701.4885		1787.69

Compound Label	*m/z*	RT	Algorithm
Compound 28	171.1488	6.712	Auto MS/MS

MS Spectrum

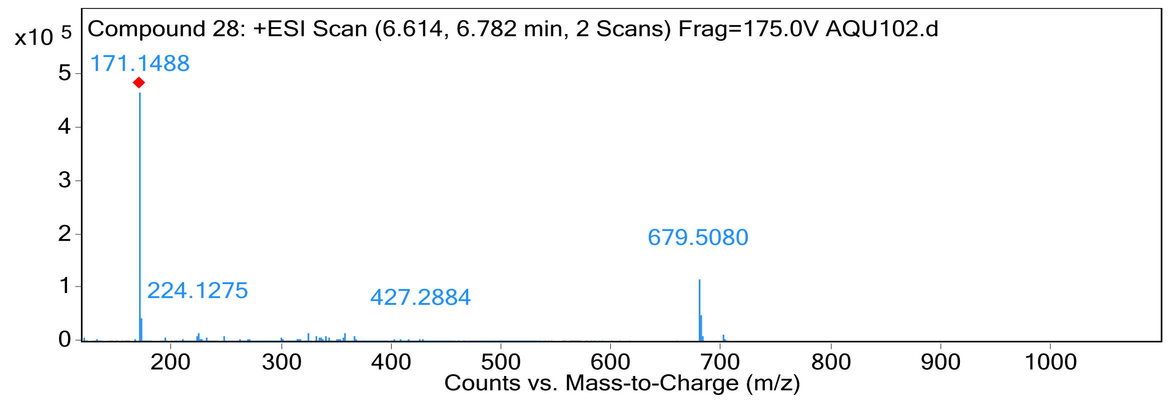

MS Zoomed Spectrum

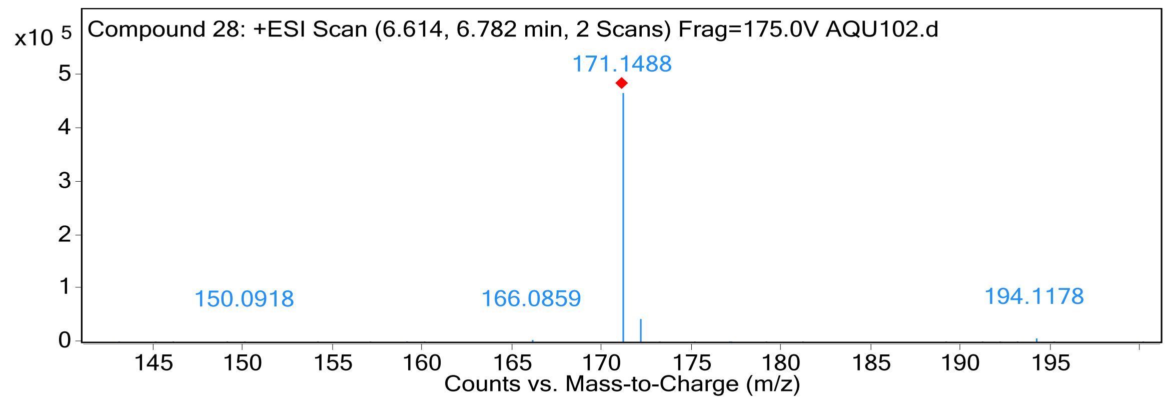

MS Spectrum Peak List

m/z	z	Abund
171.1488	1	468192.88
172.152	1	44406.45
224.1275	1	18110.47
247.1429		12223.28
323.1936	1	17858.57
340.2573	2	12531.61
356.2521	1	15778.3
679.508	1	118018.53
680.511	1	51048.66
701.4892	1	14284.95

MSMS Spectrum

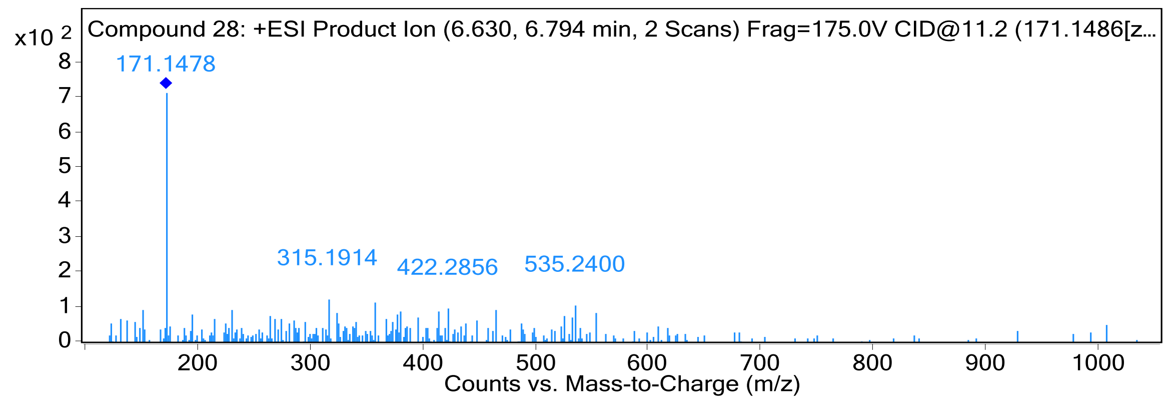

MS/MS Spectrum Peak List

m/z	z	Abund
150.1263		94.55
171.1478	1	715.31
172.1517	1	141.52
229.1507		95.87
315.1914		124.06
356.252		113.91
379.1919		91.59
422.2856		96.69
464.0797		93.28
535.24		105.75

Compound Label	*m/z*	RT	Algorithm
Compound 29	679.508	6.715	Auto MS/MS

MS Spectrum

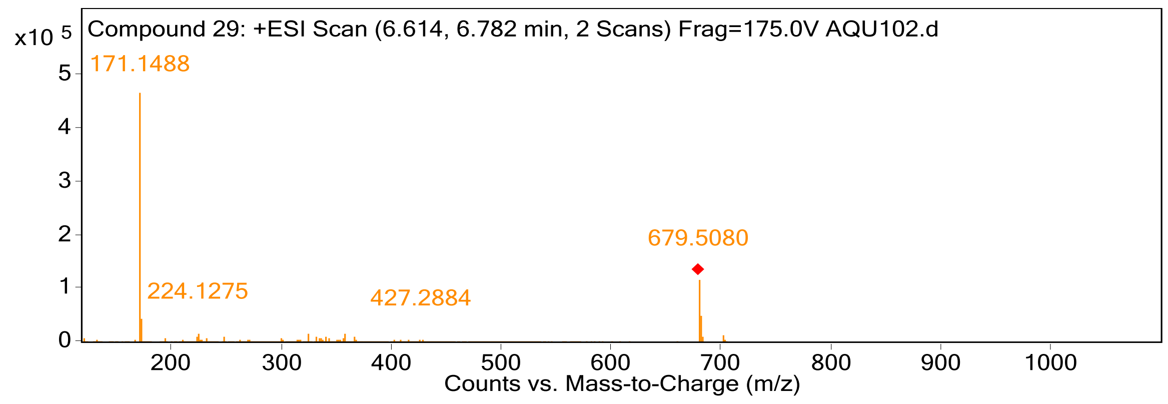

MS Zoomed Spectrum

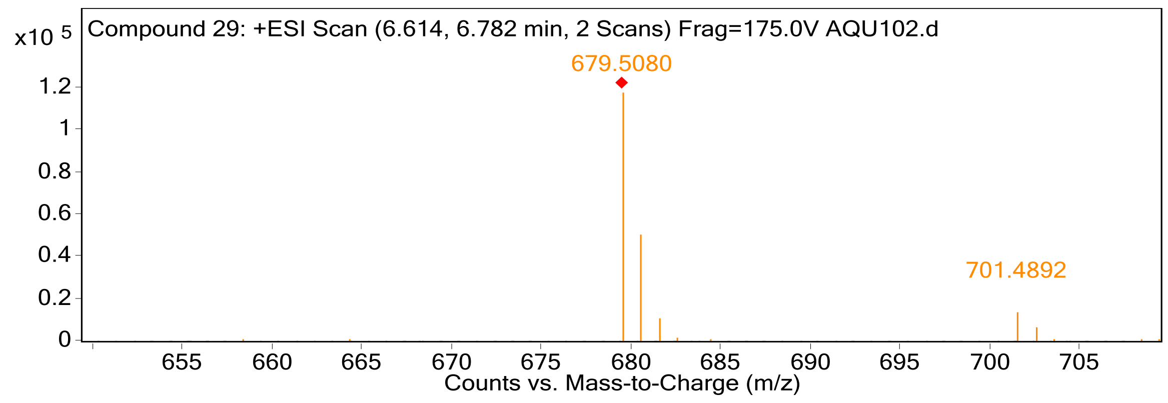

MS Spectrum Peak List

m/z	z	Abund
171.1488	1	468192.88

172.152	1	44406.45
224.1275	1	18110.47
323.1936	1	17858.57
356.2521	1	15778.3
679.508	1	118018.53
680.511	1	51048.66
681.5139	1	11014.94
682.5176	1	2005.47
701.4892	1	14284.95

MSMS Spectrum

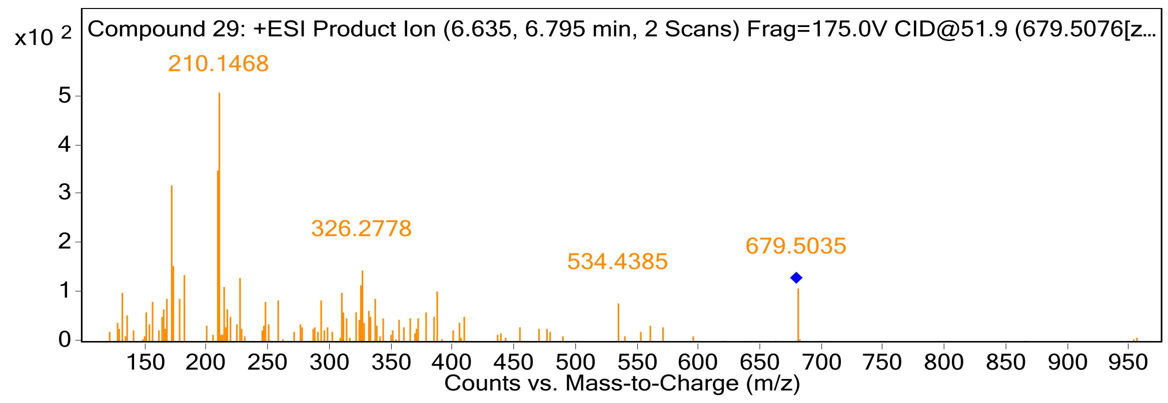

MS/MS Spectrum Peak List

m/z	Abund
171.149	319.98
172.151	155.55
182.1536	138.45
209.1651	351.38
210.1468	509.27
214.0875	113.08
226.1893	130.44
325.0735	116.5
326.2778	146.68
679.5035	111.1

Compound Label	Name	*m/z*	RT	Algorithm	Mass
Cpd 30: DG(20:5(5Z,8Z,11Z,14Z,17Z)/20:2(11Z,14Z)/0:0)	**DG(20:5(5Z,8Z,11Z,14Z,17Z)/20:2(11Z,14Z)/0:0)**	356.2519	6.86	Auto MS/MS	666.5245

MS Spectrum

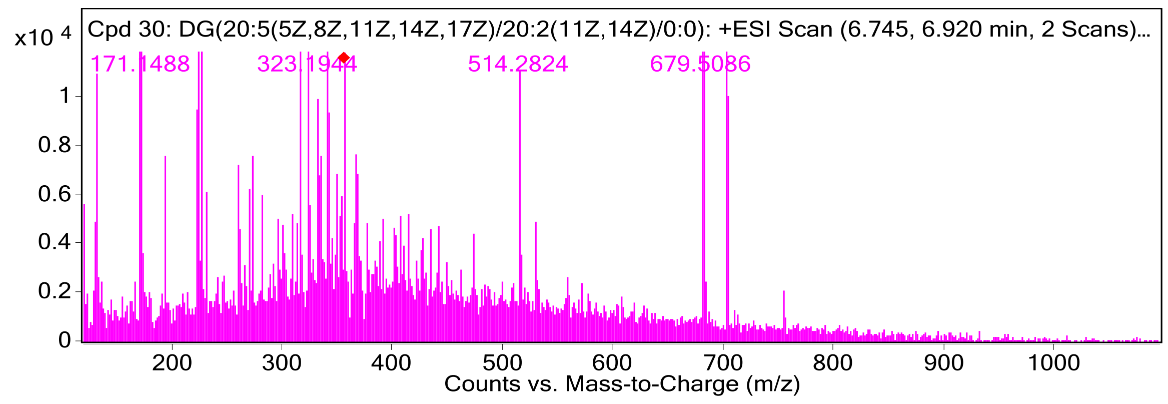

MS Zoomed Spectrum

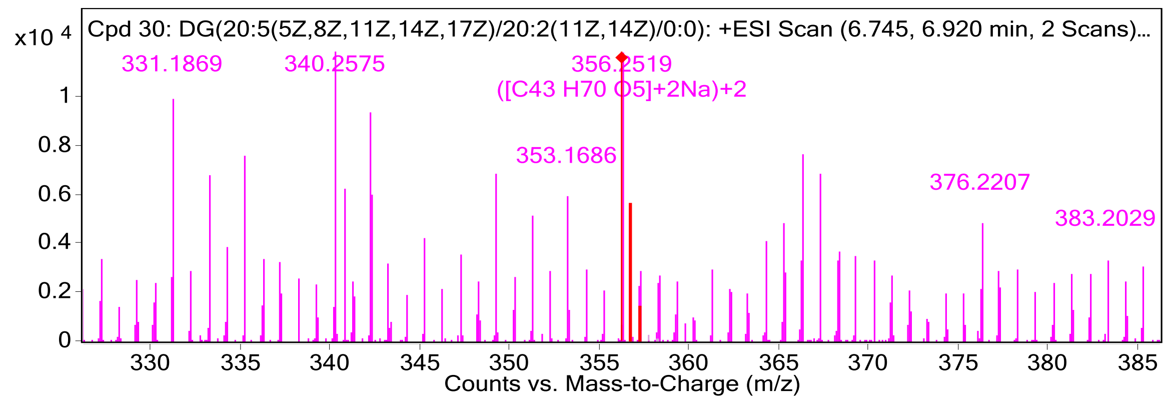

MS Spectrum Peak List

m/z	*Calc m/z*	Diff(ppm)	z	Abund	Formula	Ion
171.1488			1	653409.44		
172.152			1	67635.43		
323.1944			1	19666.58		
356.2519	356.2504	-4.22	1	11824.82	C43 H70 O5	(M+2Na)+2
356.7421	356.7521	27.88	2	250.39	C43 H70 O5	(M+2Na)+2
357.2541	357.2537	-1.25	1	2897.55	C43 H70 O5	(M+2Na)+2
679.5086			1	187677.36		
680.5114			1	75295.96		
681.5143			1	17986.12		
701.4901			1	24996.52		

MSMS Spectrum

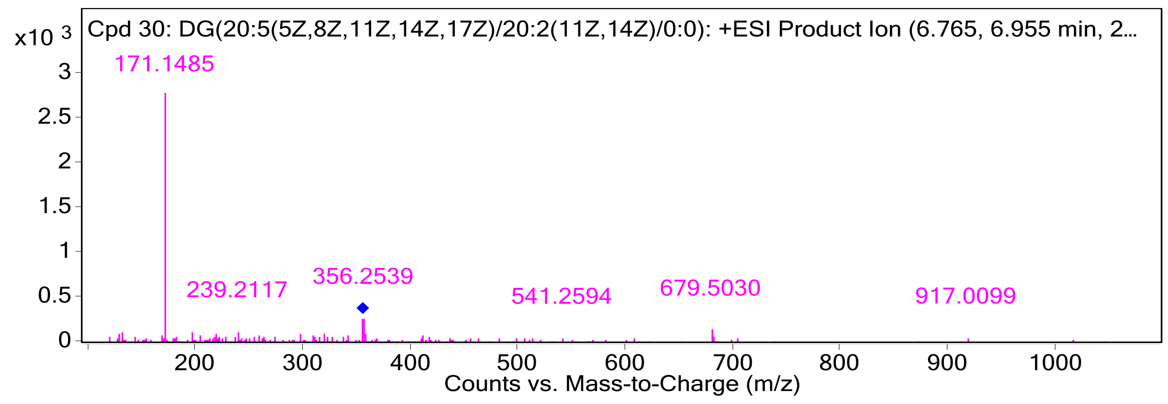

MS/MS Spectrum Peak List

m/z	z	Abund
132.0998		119.39
171.1485	1	2785.45
172.1521	1	253.48

239.2117		123.83
354.207		169.05
355.2033		261.54
355.2212	1	142.64
356.1931		154.04
356.2539		274.07
679.503	1	143.26

Compound Structure

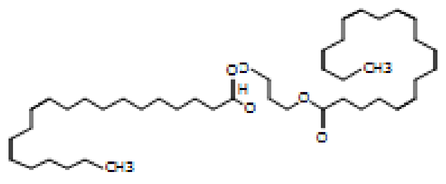

Compound Label	Name	*m/z*	RT	Algorithm	Mass
Cpd 31: Sulfoglycolithocholate	**Sulfoglycolithocholate**	514.2826	7.022	Auto MS/MS	513.2751

MS Spectrum

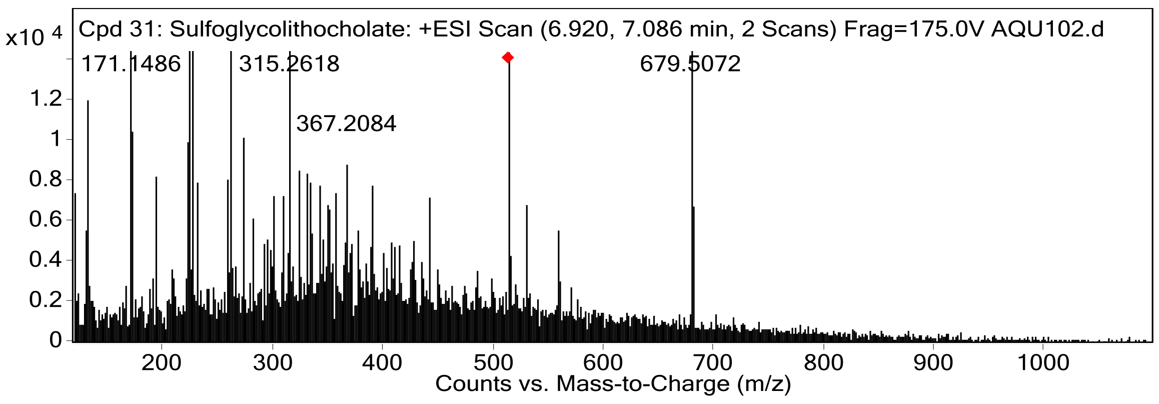

MS Zoomed Spectrum

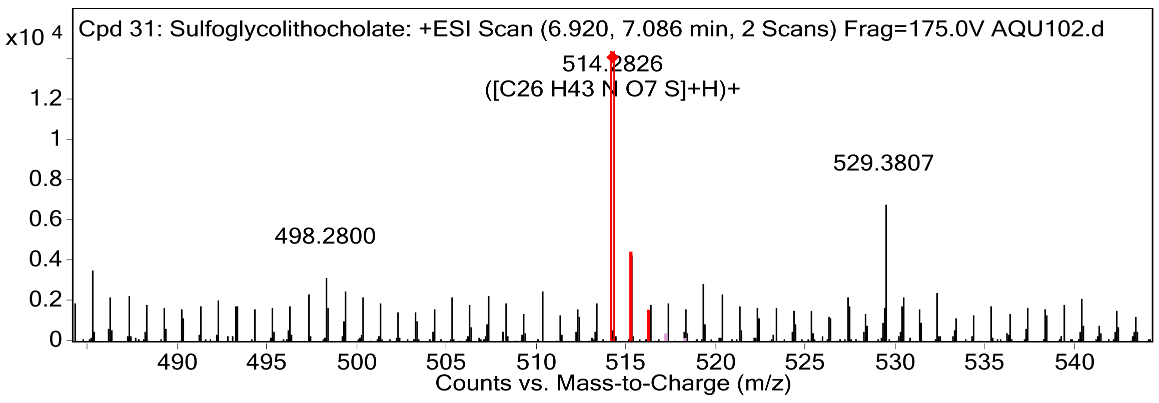

MS Spectrum Peak List

m/z	*Calc m/z*	Diff(ppm)	z	Abund	Formula	Ion
132.1017				11995.19		
171.1486			1	102688.05		
224.1275			1	18421.77		
227.1744				15564.33		
261.1585			1	15173.36		
315.2618				14478.72		
514.2826	514.2833	1.45	1	14357.55	C26 H43 N O7 S	(M+H)+
515.2869	515.2865	-0.75	1	4279	C26 H43 N O7 S	(M+H)+
516.28	516.2848	9.4	1	1855.43	C26 H43 N O7 S	(M+H)+
679.5072			1	16479.12		

MSMS Spectrum

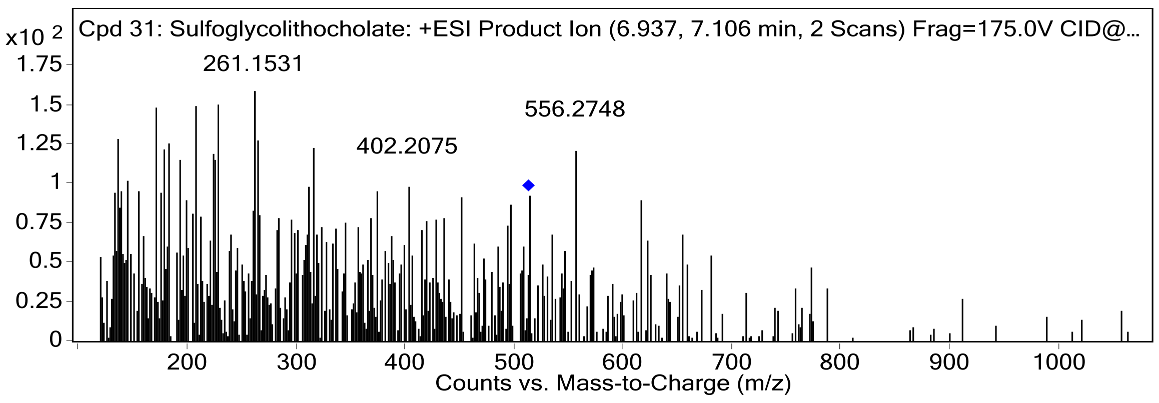

MS/MS Spectrum Peak List

m/z	Abund
136.062	128.41
171.1479	149.06
178.1198	121.85
183.0753	125.85
207.1414	149.61
227.1742	150.26
261.1531	158.72
264.2049	127.82
315.2631	123.09
556.2748	121.71

Compound Structure

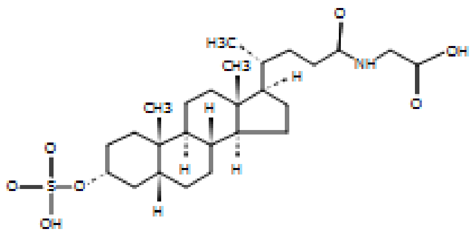

Compound Label	*m/z*	RT	Algorithm
Compound 32	171.1486	7.069	Auto MS/MS

MS Spectrum

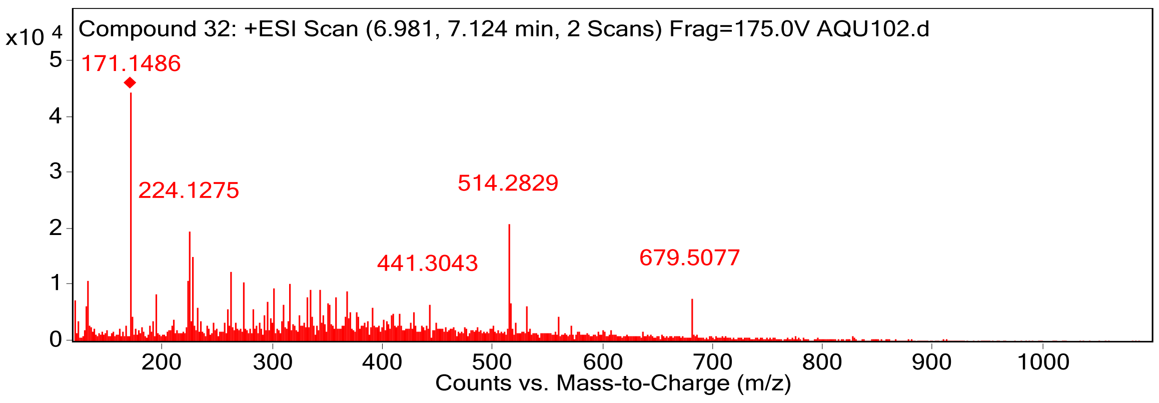

MS Zoomed Spectrum

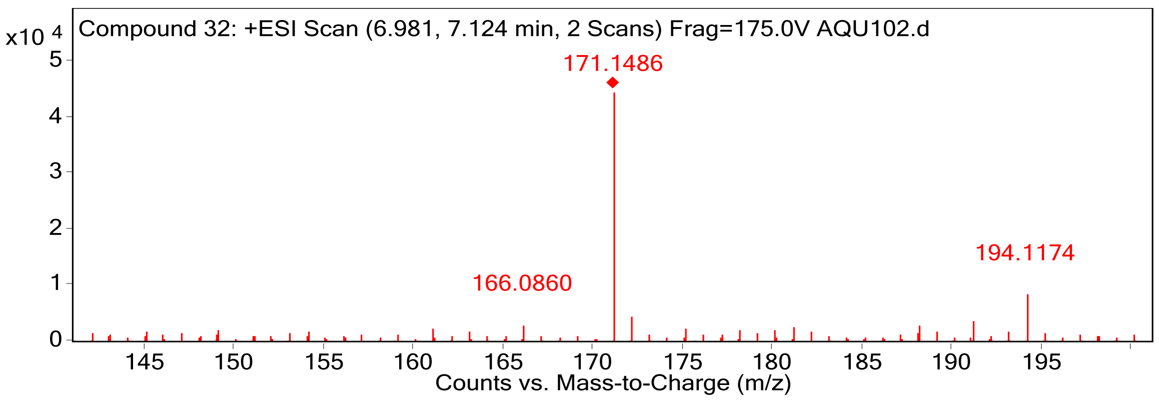

MS Spectrum Peak List

m/z	z	Abund
132.1019		10969.12
171.1486	1	44501.01
172.1518	1	4569.11
222.1118		10845.75
224.1275	1	19633.5
227.1745		15085.17
261.1577	1	12524.47
273.1809		10579.01
315.262		10377.65
514.2829	1	20929.72

MSMS Spectrum

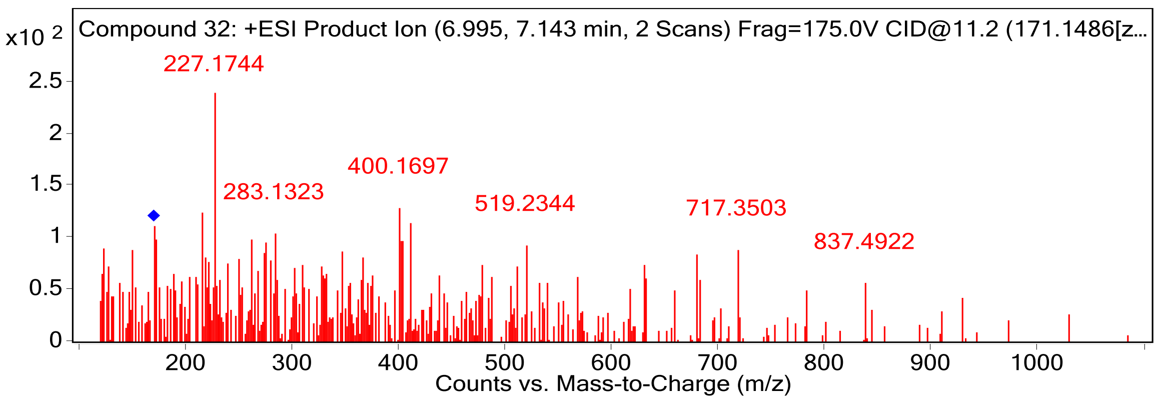

MS/MS Spectrum Peak List

m/z	Abund
171.0788	112.34
172.1696	99.28
214.8855	124.74
227.1744	240.17
261.152	98.91
283.1323	104.95
400.1697	129.7
402.2514	97.42
403.2334	96.9
411.2386	114.69

Compound Label	*m/z*	RT	Algorithm
Compound 33	453.3408	7.286	Auto MS/MS

MS Spectrum

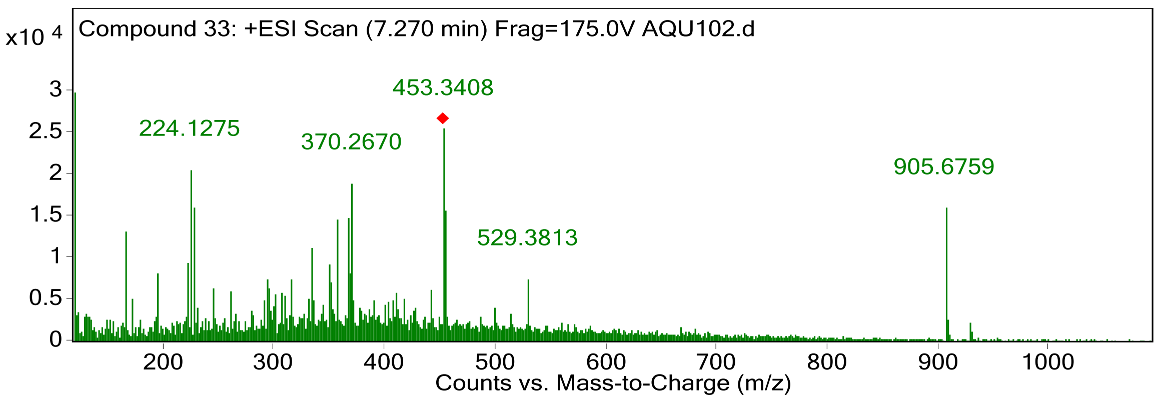

MS Zoomed Spectrum

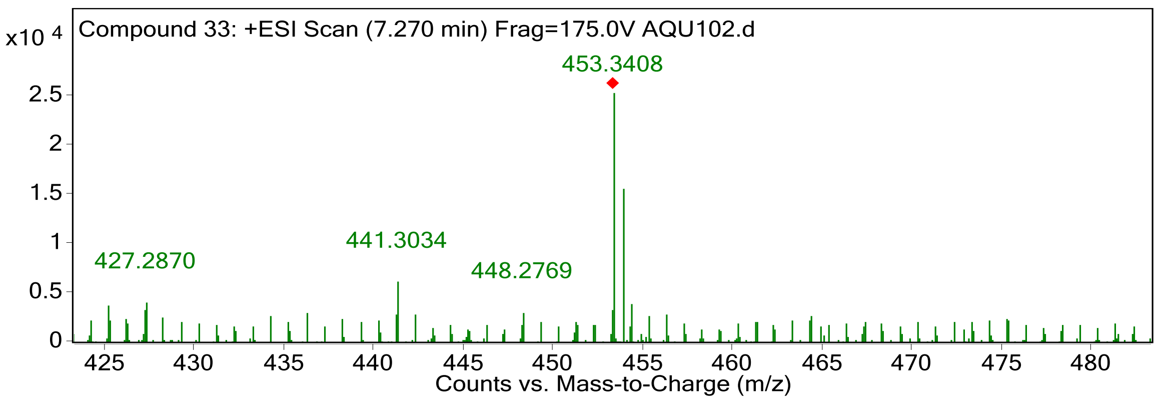

MS Spectrum Peak List

m/z	z	Abund
120.0808	1	29797.5
224.1275	1	20558.26
227.1742		16077.26
367.1844		14789.16
370.267	1	18942.86
453.3408	2	25424.85
453.8419	2	15658.37
454.3433	2	3953.09
454.8457	2	920.02
905.6759	1	15964.34

MSMS Spectrum

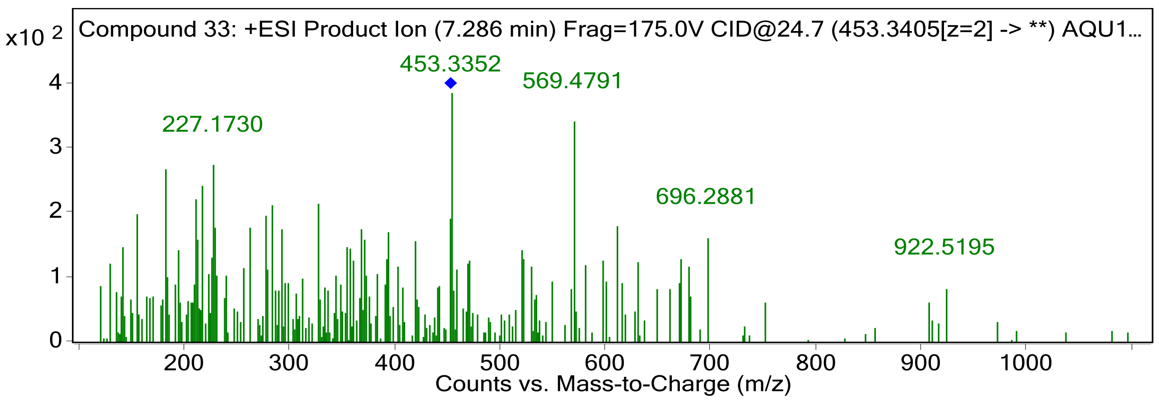

MS/MS Spectrum Peak List

m/z	Abund
154.0944	198.06
182.1546	268.18
210.1507	222.74
216.1357	242.47
227.173	273.73
282.1151	212.72
282.172	198.29
326.2743	214.21
453.3352	386.64
569.4791	341.09

Compound Label	Name	*m/z*	RT	Algorithm	Mass

Cpd 34: Dihydrocapsaicin	**Dihydrocapsaicin**	308.2202	7.465	Auto MS/MS	307.2109

MS Spectrum

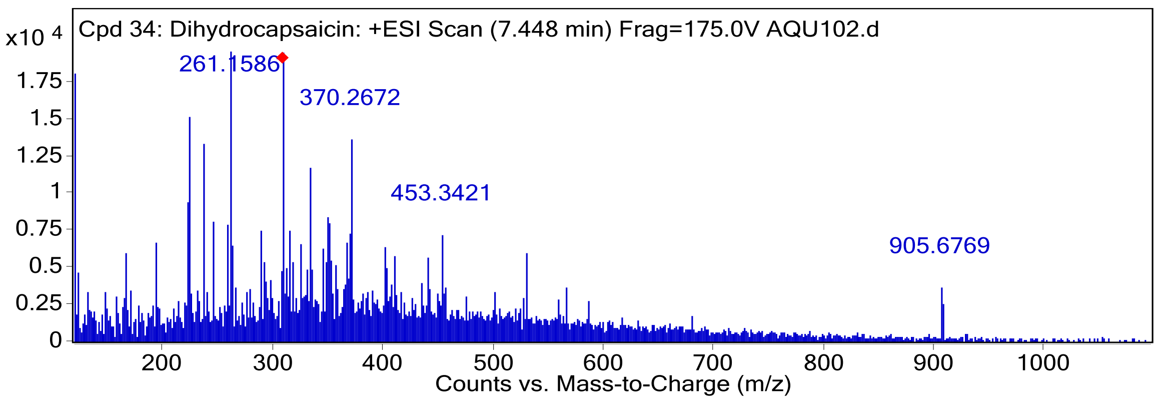

MS Zoomed Spectrum

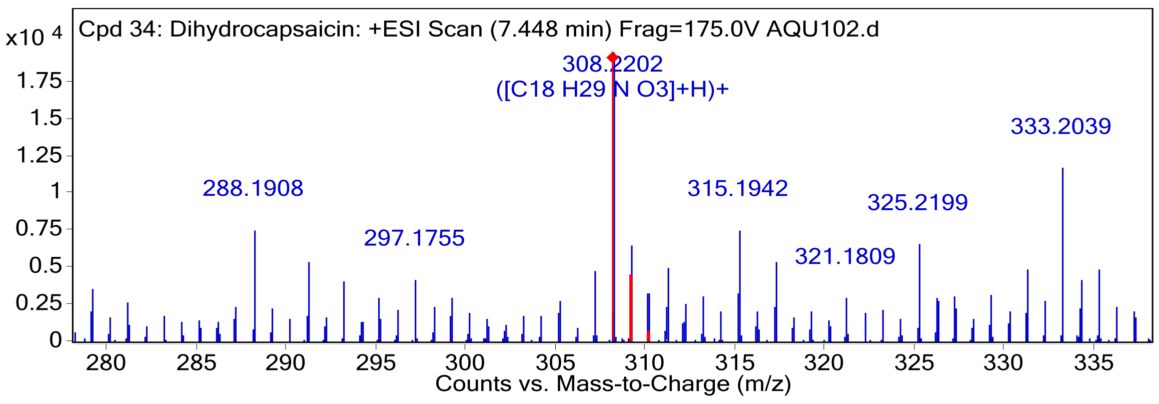

MS Spectrum Peak List

m/z	*Calc m/z*	Diff(ppm)	z	Abund	Formula	Ion
120.0809				18032.27		
222.1115				9433.25		
224.1272			1	15174.25		
237.1477				13351.83		
261.1586			1	34174.73		
308.2202	308.222	5.92	1	19486.97	C18 H29 N O3	(M+H)+
309.2218	309.2253	11.52	1	4961.11	C18 H29 N O3	(M+H)+
310.2075	310.228	66.24	1	2409.9	C18 H29 N O3	(M+H)+
333.2039			1	11750.07		
370.2672			1	13706.9		

MSMS Spectrum

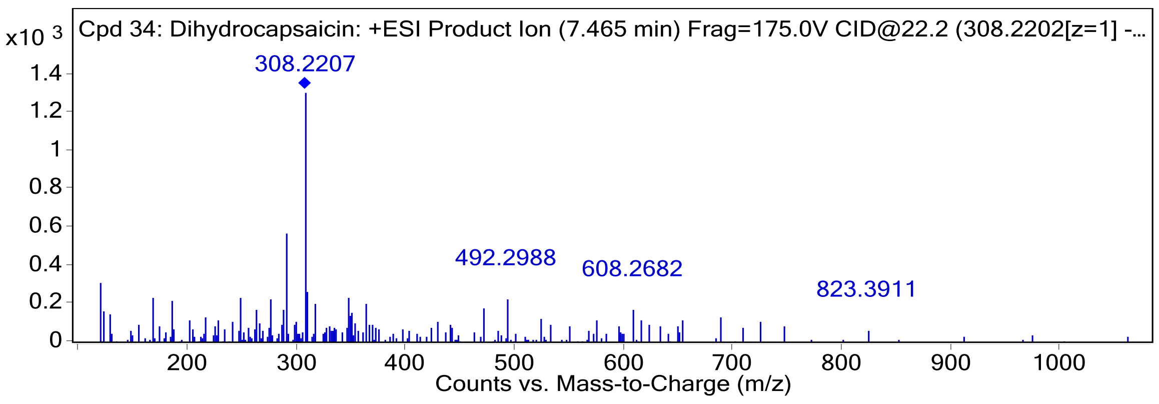

MS/MS Spectrum Peak List

m/z	z	Abund
120.0806		311.34
168.1395		236.39
185.0687		215.21
247.9678		237.72
275.198		227.65
290.2078		567.02
308.2207	2	1304.76
309.2226	2	269.02
347.1768		231.14
492.2988		225.59

Compound Structure

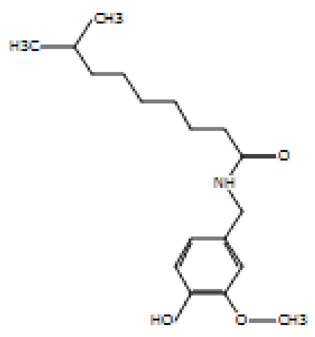

Compound Label	Name	*m/z*	RT	Algorithm	Mass
Cpd 35: 1-Phosphatidyl-1D-myo-inositol 3-phosphate	**1-Phosphatidyl-1D-myo-inositol 3-phosphate**	493.0134	8.146	Auto MS/MS	470.0227

MS Spectrum

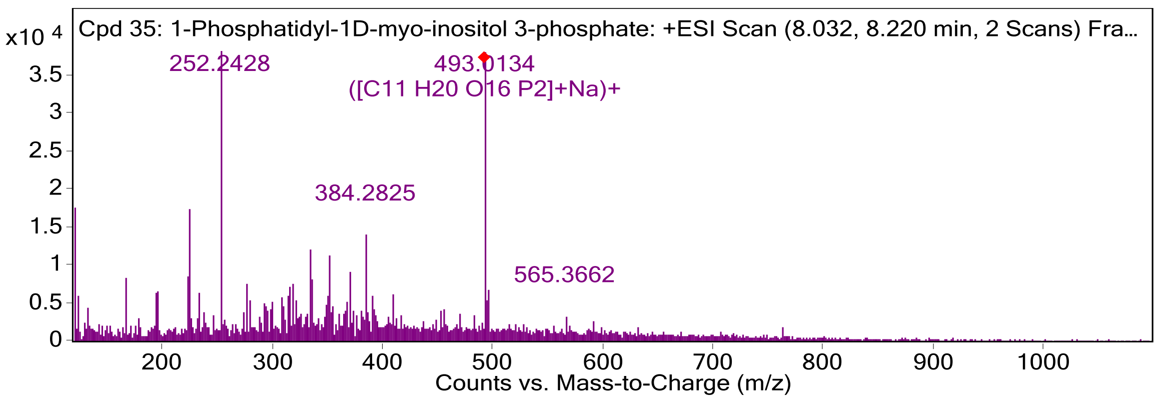

MS Zoomed Spectrum

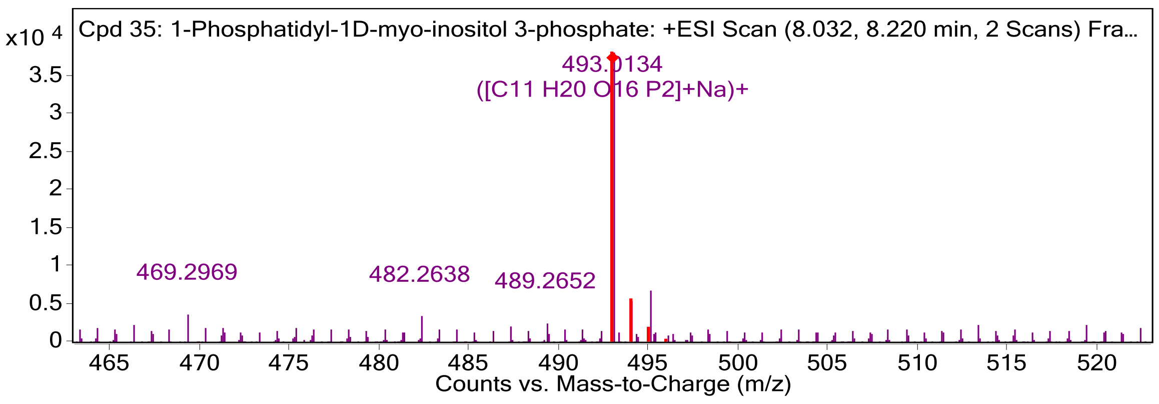

MS Spectrum Peak List

m/z	*Calc m/z*	Diff(ppm)	z	Abund	Formula	Ion
120.0809				17615.44		
224.1274			1	17541.91		
252.2428			1	215501.42		
253.2456			1	37810.39		
333.2044			1	12132.88		
384.2825			1	14120.8		
493.0134	493.0119	-3.15	1	38049.03	C11 H20 O16 P2	(M+Na)+
494.0154	494.0153	-0.1	1	5470.87	C11 H20 O16 P2	(M+Na)+
495.0098	495.0166	13.85	1	6820.08	C11 H20 O16 P2	(M+Na)+
496.0113	496.0197	16.92	1	952.44	C11 H20 O16 P2	(M+Na)+

MSMS Spectrum

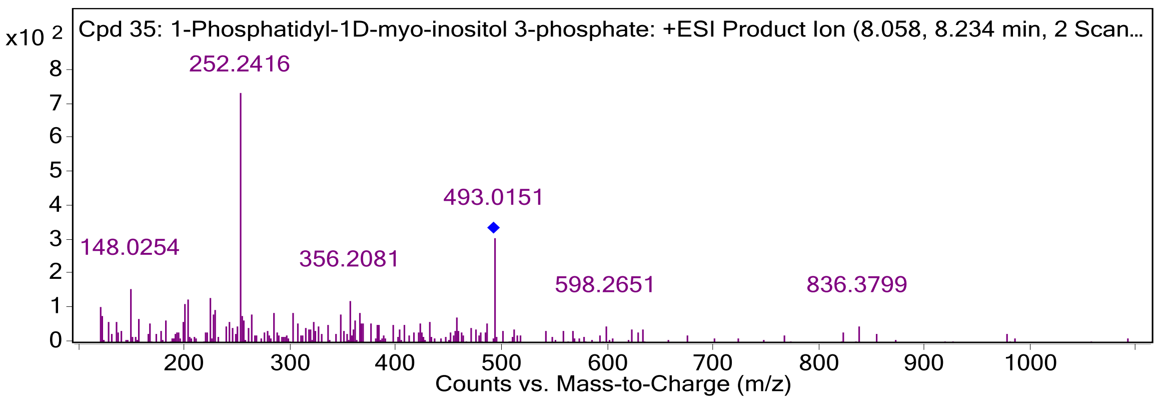

MS/MS Spectrum Peak List

m/z	z	Abund
120.0804		106.01
148.0254		158.2
199.0334		115.44
203.1434		129.03
224.1422		130.69
228.1562		96.69
252.2416	1	734.71
283.0868		89.85
356.2081		123.77
493.0151		305.5

Compound Structure

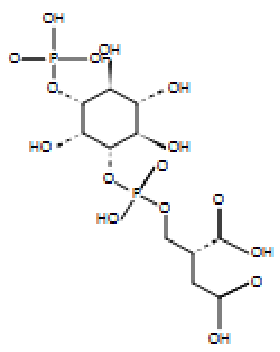

Compound Label	Name	*m/z*	RT	Algorithm	Mass
Cpd 36: 2,3-Dihydro-6-methyl-5-propanoyl-1H-pyrrolizine	**2,3-Dihydro-6-methyl-5-propanoyl-1H-pyrrolizine**	178.1224	8.383	Auto MS/MS	177.115

MS Spectrum

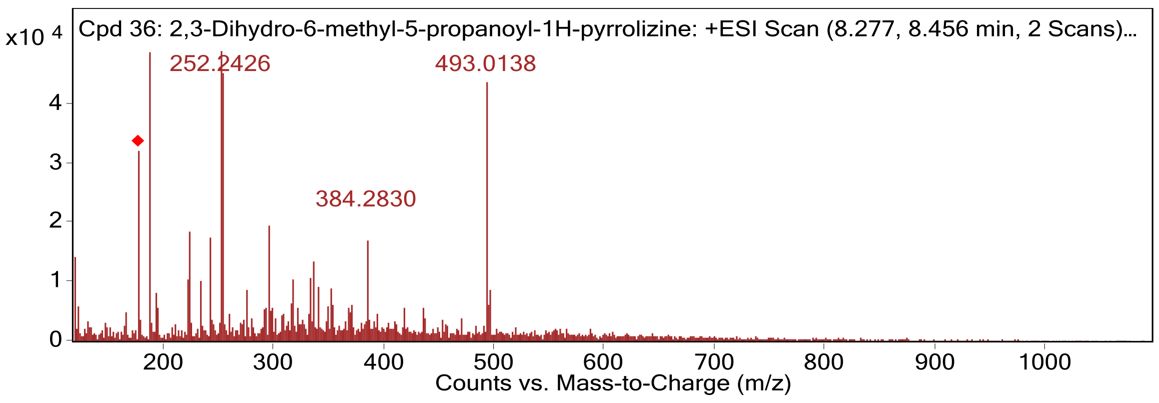

MS Zoomed Spectrum

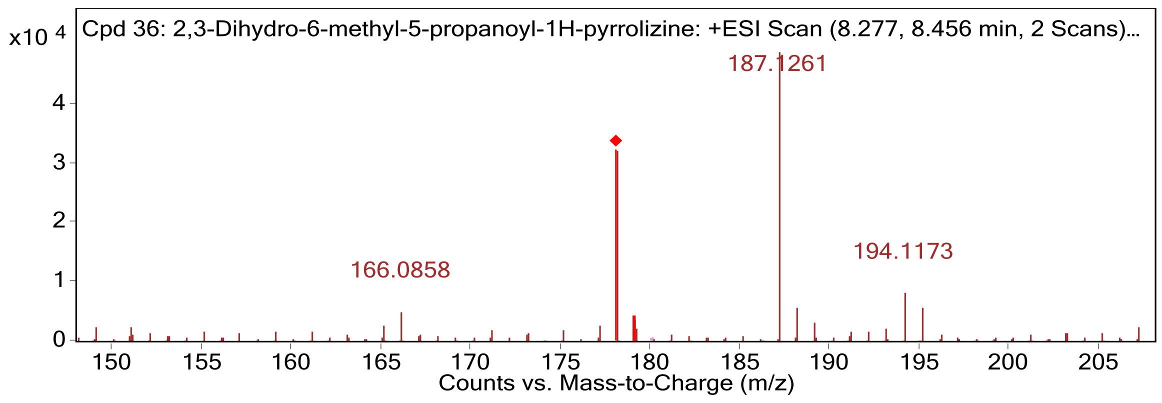

MS Spectrum Peak List

m/z	*Calc m/z*	Diff(ppm)	z	Abund	Formula	Ion
178.1224	178.1226	1.14	1	32016.82	C11 H15 N O	(M+H)+
179.1245	179.1259	7.78	1	3663.55	C11 H15 N O	(M+H)+
187.1261			1	48710.61		
224.1276			1	18642.07		
242.283			1	17479.63		
252.2426			1	292984.63		
253.2456			1	45304		
295.1695			1	19704.67		
384.283			1	17094.86		
493.0138			1	43565.7		

MSMS Spectrum

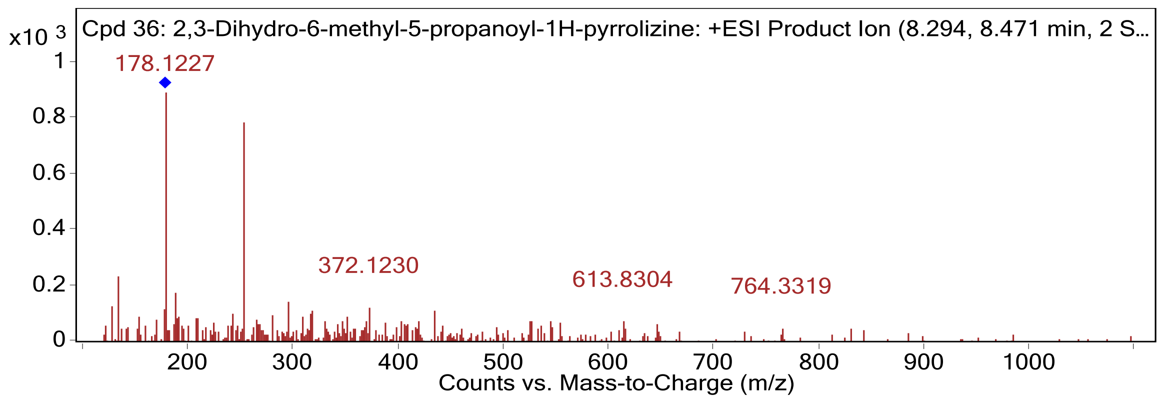

MS/MS Spectrum Peak List

m/z	z	Abund
128.0604		130.07
133.0648		232.78
134.0669		120.87
177.1255		115.03
178.1227	1	892.7
179.124	1	163.75
187.1257		177.51
252.2415	1	784.83
295.164		144.93
372.123		124.61

Compound Structure

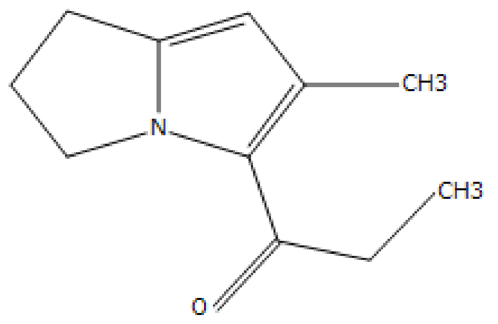

Compound Label	Name	*m/z*	RT	Algorithm	Mass
Cpd 37: 1-Phenyl-5-propyl-1H-pyrazole	**1-Phenyl-5-propyl-1H-pyrazole**	187.1261	8.504	Auto MS/MS	186.1184

MS Spectrum

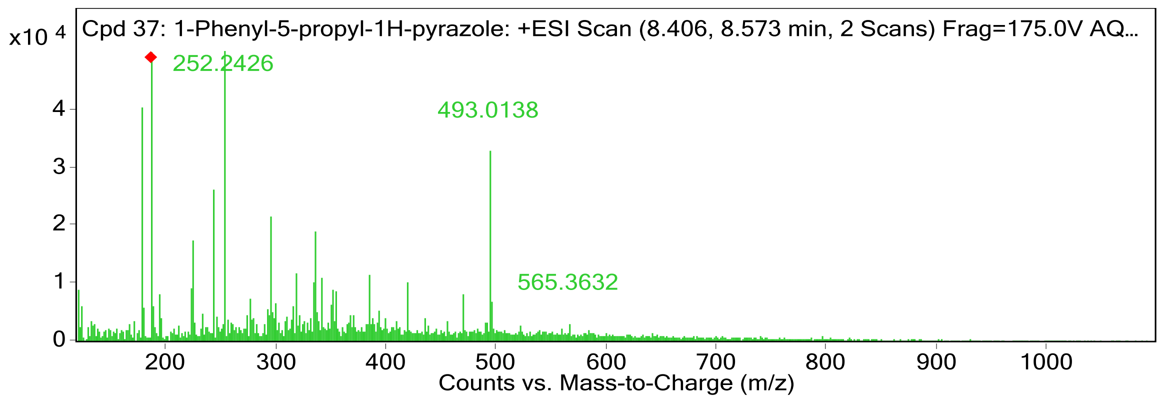

MS Zoomed Spectrum

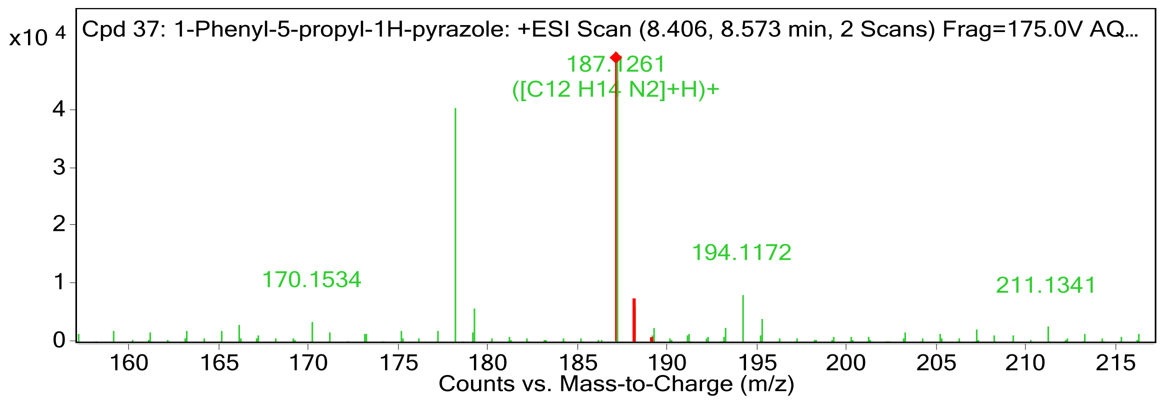

MS Spectrum Peak List

m/z	*Calc m/z*	Diff(ppm)	z	Abund	Formula	Ion
178.1222			1	40371		

187.1261	187.123	-16.53	1	49975.34	C12 H14 N2	(M+H)+
188.1285	188.126	-13.26	1	6100.78	C12 H14 N2	(M+H)+
189.1254	189.1291	19.22	1	2694.69	C12 H14 N2	(M+H)+
224.1276			1	17539.83		
242.283			1	26159.92		
252.2426			1	79697.45		
295.1702			1	21572.2		
335.2193			1	19004.54		
493.0138			1	32886.89		

MSMS Spectrum

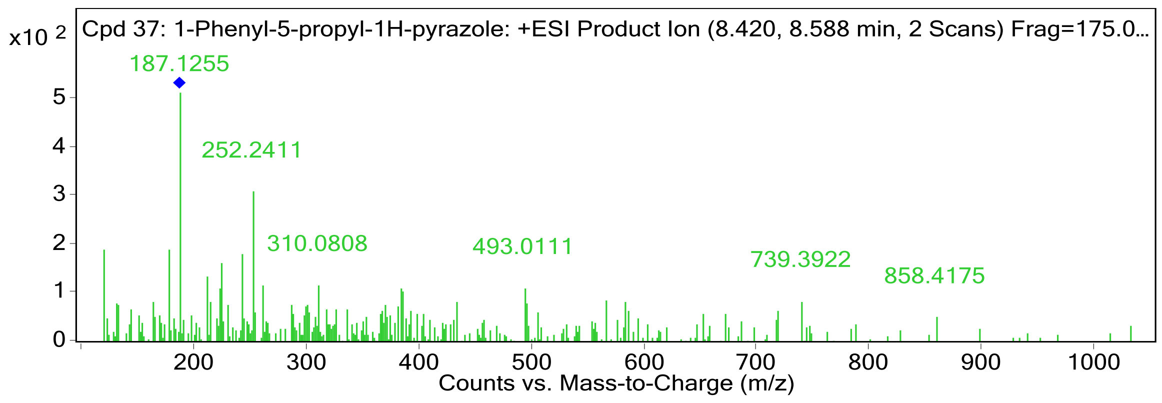

MS/MS Spectrum Peak List

m/z	z	Abund
120.0808		189.29
178.1207		189.48
187.1255		514.3
211.1454		134.29
224.1293	1	163.71
242.2816	1	182.61
252.2411	1	310.35
260.2181		115.49
310.0808		117.42
493.0111	1	111.18

Compound Structure

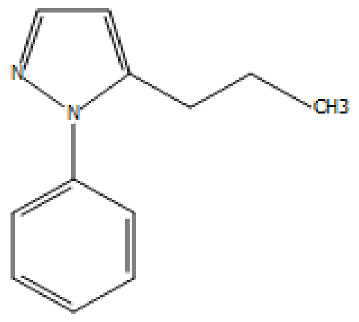

Compound Label	Name	*m/z*	RT	Algorithm	Mass
Cpd 38: 1-Phenyl-5-propyl-1H-pyrazole	**1-Phenyl-5-propyl-1H-pyrazole**	187.1259	8.779	Auto MS/MS	186.118

MS Spectrum

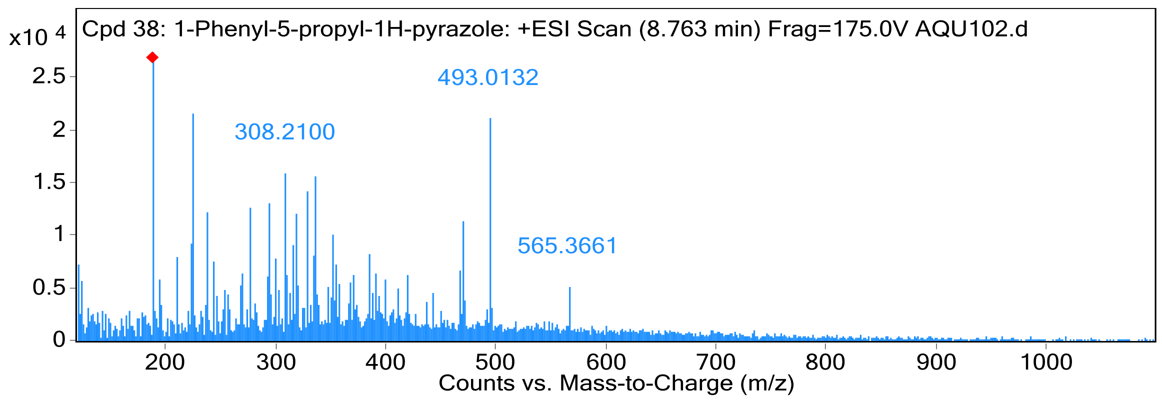

MS Zoomed Spectrum

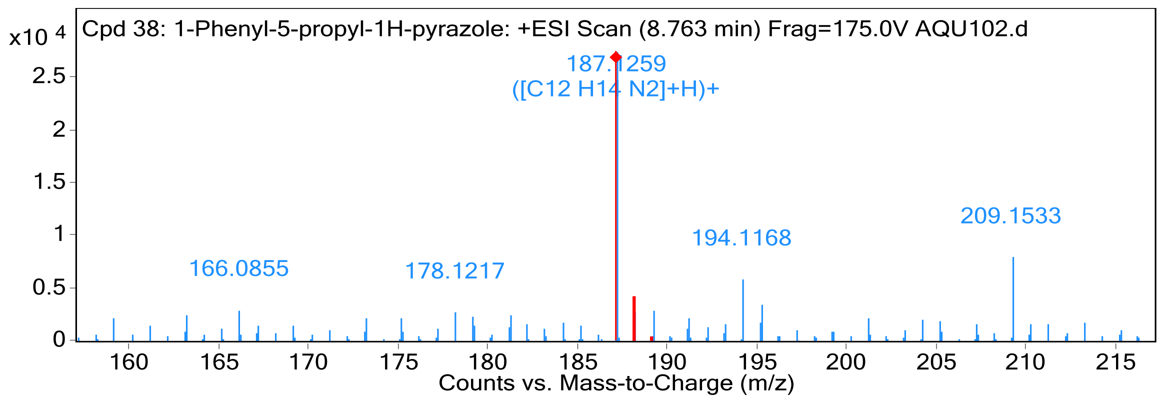

MS Spectrum Peak List

m/z	*Calc m/z*	Diff(ppm)	z	Abund	Formula	Ion
187.1259	187.123	-15.79	1	27452.59	C12 H14 N2	(M+H)+
188.1286	188.126	-13.9	1	2831.05	C12 H14 N2	(M+H)+
189.1263	189.1291	14.74	1	2964.09	C12 H14 N2	(M+H)+
190.1238	190.1321	43.6	1	378.49	C12 H14 N2	(M+H)+
224.1274				21615.47		
293.2089				13150.41		
308.21			1	16009.87		
327.1901			1	14250.96		
335.2199			1	15739.7		
493.0132				21177.14		

MSMS Spectrum

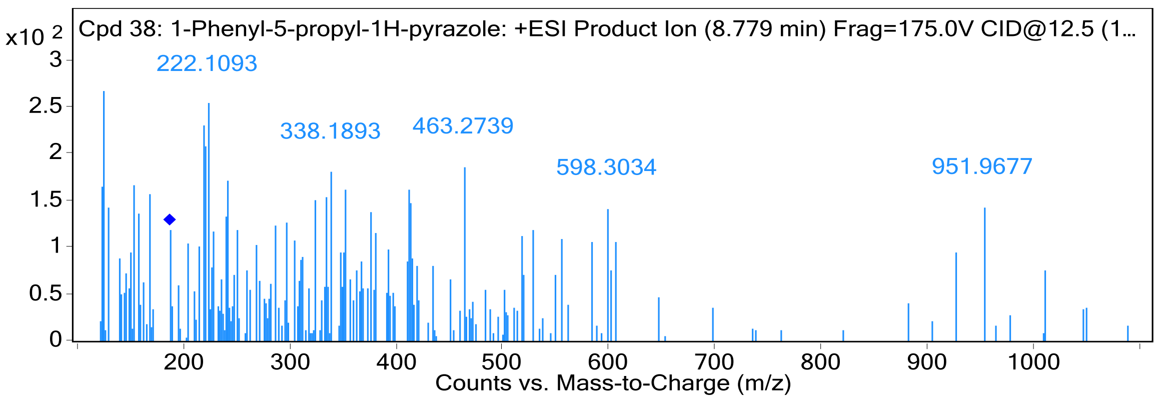

MS/MS Spectrum Peak List

m/z	Abund
123.1164	166.34
124.086	267.09
152.0718	168
218.1151	231.59
219.1355	173.35
220.1456	209.24
222.1093	255.17
241.1871	172.85
338.1893	180.94
463.2739	186.51

Compound Structure

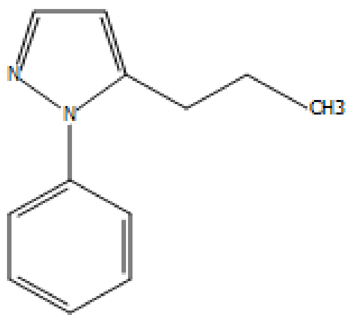

Compound Label	*m/z*	RT	Algorithm
Compound 39	308.2106	8.875	Auto MS/MS

MS Spectrum

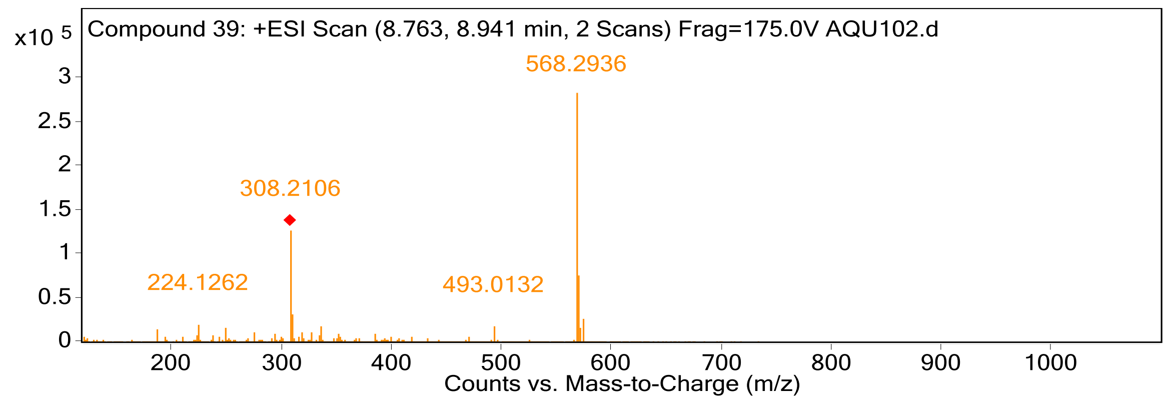

MS Zoomed Spectrum

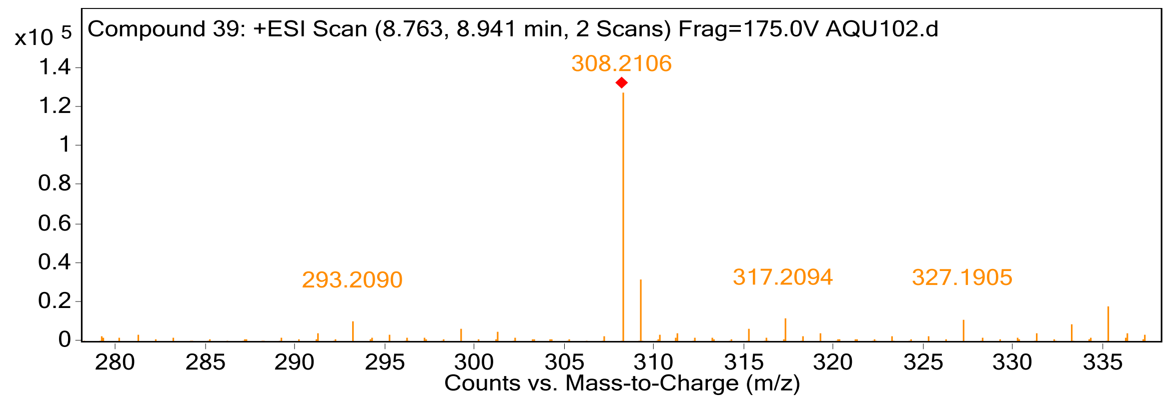

MS Spectrum Peak List

m/z	z	Abund
224.1262	1	20850.41
249.1478	1	17081.28
308.2106	1	127864.48
309.2129	1	32198.23
310.2144	1	3909.21
335.2196	1	18189.21
493.0132		18474.02
568.2936	1	283419.22
569.2965	1	75768.77
573.2483	1	26390.9

MSMS Spectrum

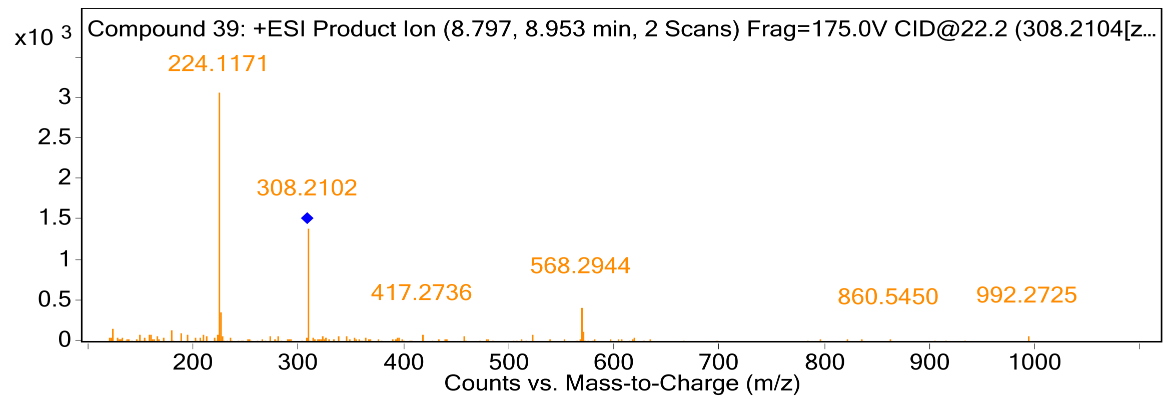

MS/MS Spectrum Peak List

m/z	z	Abund
123.0802		166.56
178.1221		138.82
187.1286		102.56
194.1114		99.11
223.1084		96.88
224.1171	1	3074.52
225.1237	1	363.31
308.2102	1	1388.52
568.2944	1	427.34
569.2938	1	132.96

Compound Label	*m/z*	RT	Algorithm
Compound 40	308.2109	9.251	Auto MS/MS

MS Spectrum

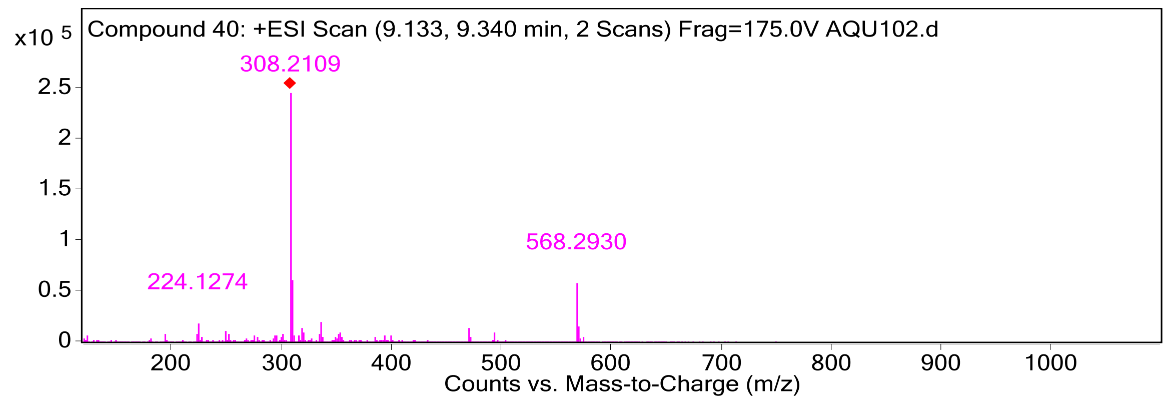

MS Zoomed Spectrum

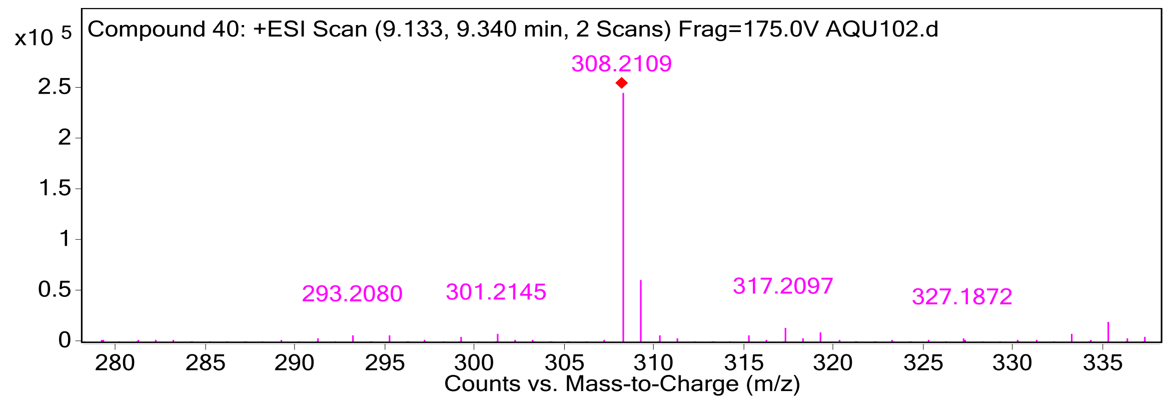

MS Spectrum Peak List

m/z	z	Abund

224.1274	1	18988.23
249.1841	1	11246.09
308.2109	1	246217.17
309.2134	1	62494.65
310.2156	1	7523.97
317.2097	1	14586.29
335.2189	1	19906
469.2993	1	14388.69
568.293	1	58479.65
569.2969	1	16843

MSMS Spectrum

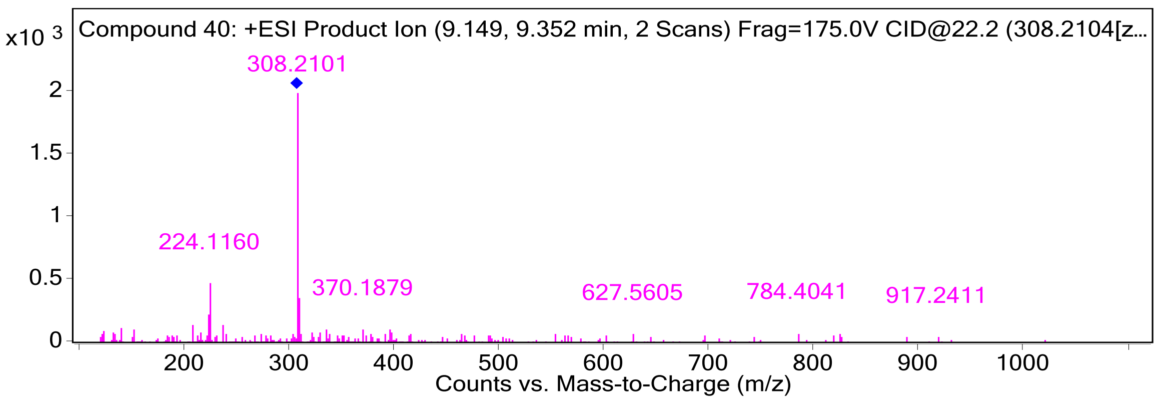

MS/MS Spectrum Peak List

m/z	z	Abund
139.0727		117.44
152.0539		105.31
208.0864		143.3
223.1074		230.16
224.116		473.18
236.114		145.16
308.2101	1	1994.41
309.2105	1	352.06
370.1879		105.68
395.212		105.33

Compound Label	Name	*m/z*	RT	Algorithm	Mass
Cpd 41: Protorifamycin I	**Protorifamycin I**	640.3136	9.512	Auto MS/MS	639.3066

MS Spectrum

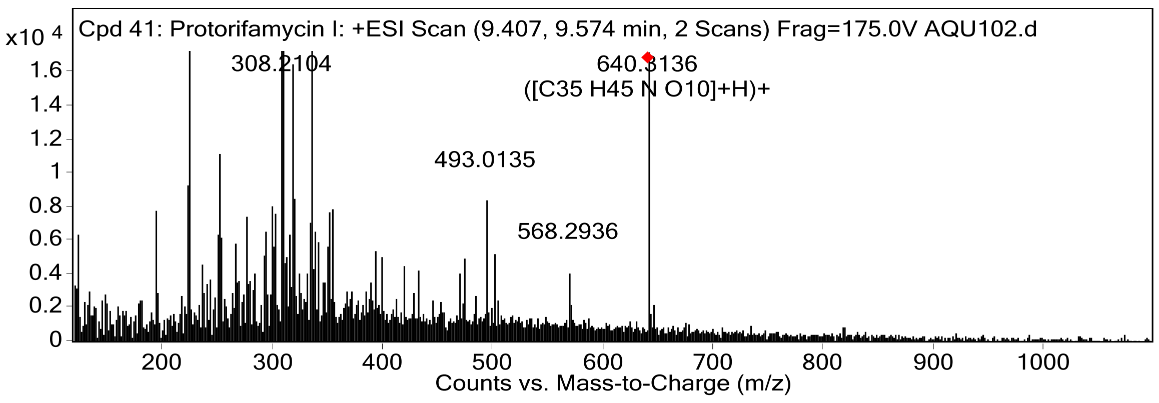

MS Zoomed Spectrum

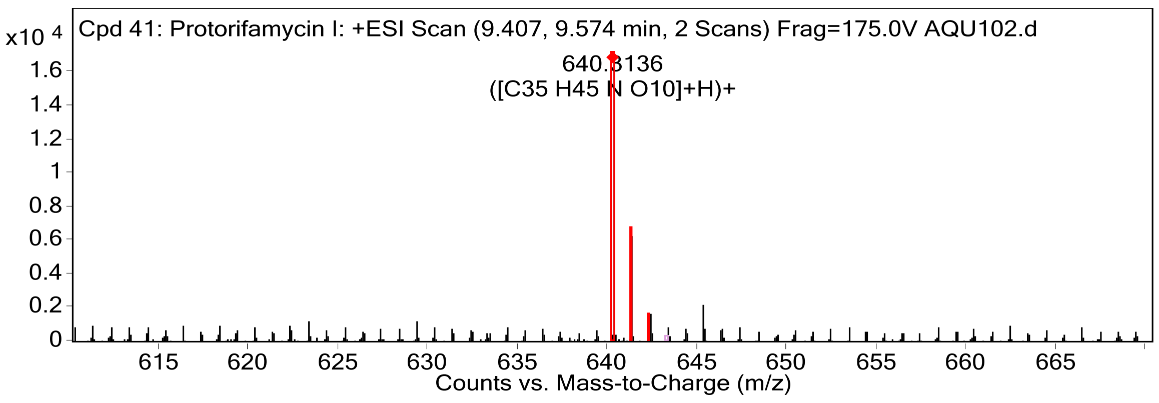

MS Spectrum Peak List

m/z	*Calc m/z*	Diff(ppm)	z	Abund	Formula	Ion
222.1115				9271.99		
224.1276				19816.53		
251.1991				11160.61		
308.2104			1	173122.03		
309.2133			1	40374.11		
317.2091			1	16473.21		
335.2199			1	17655.93		
640.3136	640.3116	-3.16	1	17192.67	C35 H45 N O10	(M+H)+
641.3173	641.315	-3.56	1	6290.31	C35 H45 N O10	(M+H)+
642.3224	642.3178	-7.25	1	1705.48	C35 H45 N O10	(M+H)+

MSMS Spectrum

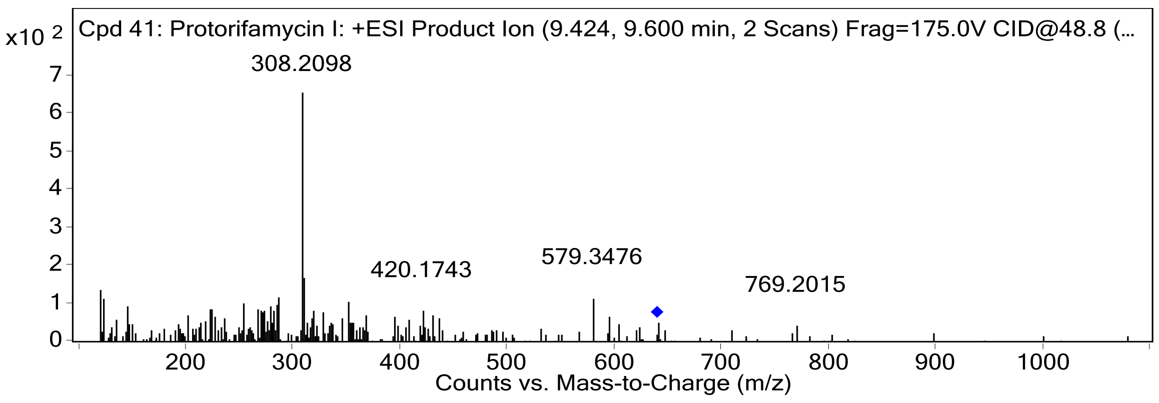

MS/MS Spectrum Peak List

m/z	z	Abund
120.0802		139.58
123.0797		114.27
253.2138		101.43
278.1945		93.99

285.1762		97.36
286.083		116.61
308.2098	1	657.99
309.2133	1	169.65
351.2126		104.49
579.3476		115.43

Compound Structure

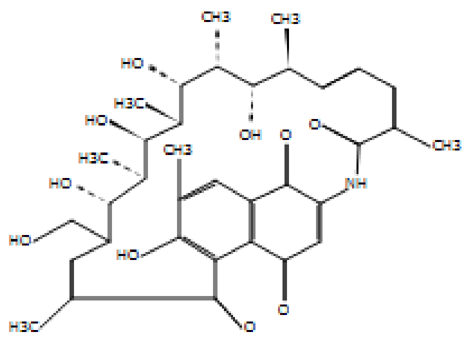

Compound Label	*m/z*	RT	Algorithm
Compound 42	308.2101	9.62	Auto MS/MS

MS Spectrum

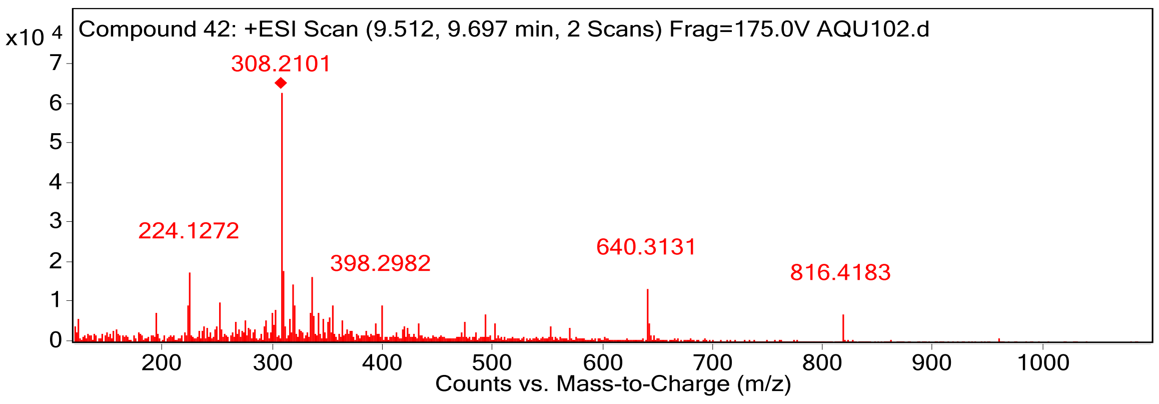

MS Zoomed Spectrum

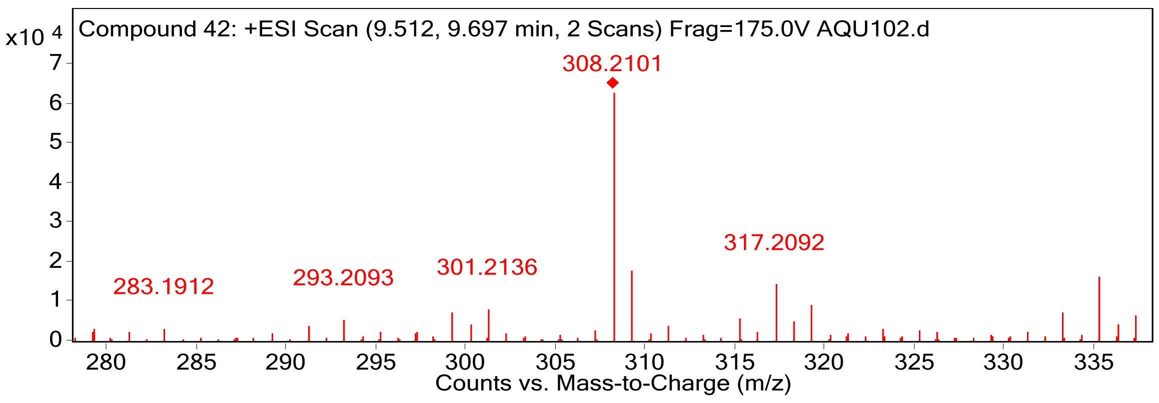

MS Spectrum Peak List

m/z	z	Abund
224.1272		17661.52
251.1991		10301.66
308.2101	1	62902.05
309.2123	1	18200.27
310.2136	1	2174.54
317.2092	1	14600.72
319.2247		9428.73
335.2192	1	16468.19
398.2982		9443.57
640.3131	1	13597.76

MSMS Spectrum

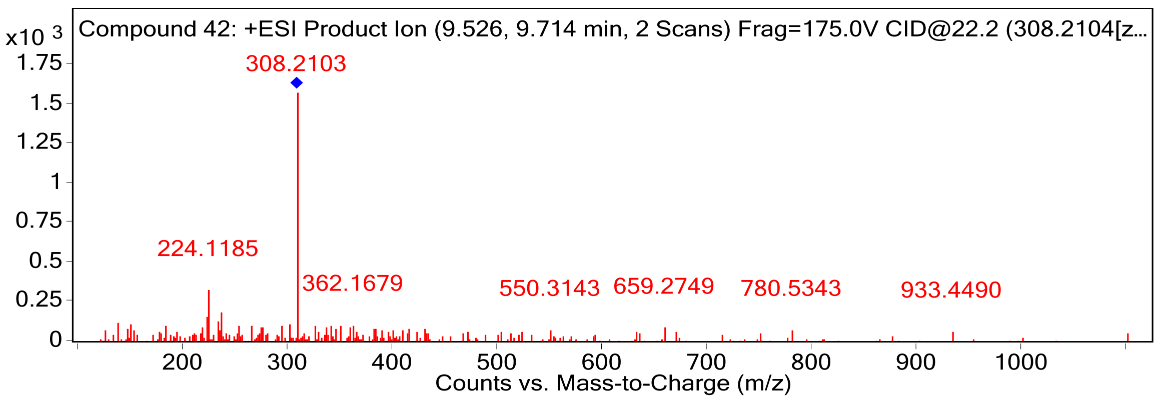

MS/MS Spectrum Peak List

m/z	z	Abund
137.0598		118.73
150.0879		111.79
222.1593		110.02
223.1222		157.03
224.1185		327.46
233.1534		128.1
236.1182		184.02
301.1987		108.42
308.2103	1	1573.32
309.2124	1	123.61

Compound Label	Name	*m/z*	RT	Algorithm	Mass
Cpd 43: Candoxatrilat	**Candoxatrilat**	422.216	9.791	Auto MS/MS	399.2269

MS Spectrum

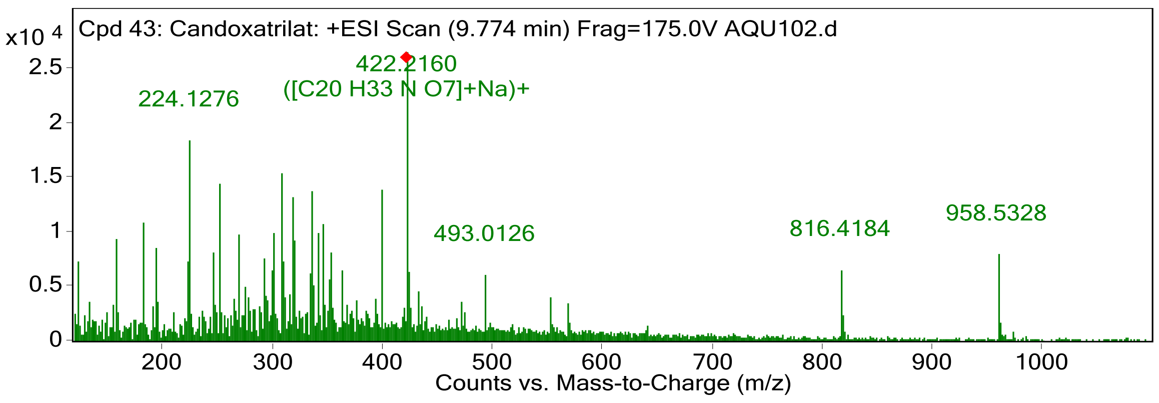

MS Zoomed Spectrum

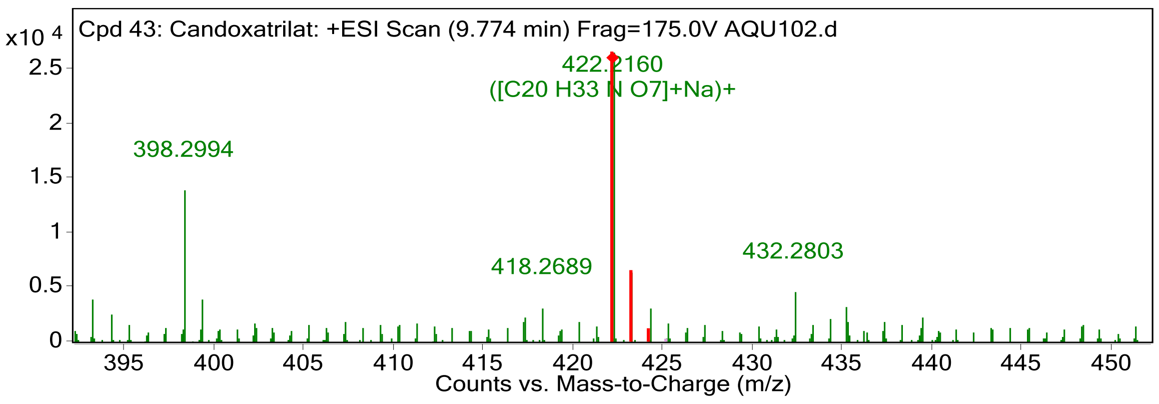

MS Spectrum Peak List

m/z	*Calc m/z*	Diff(ppm)	z	Abund	Formula	Ion
182.1536				10973.05		
224.1276			1	18497.24		
251.1995			1	14552.85		
308.2103				15448.45		
317.2102				13218.28		
335.2194			1	13778.9		
398.2994			1	13919.65		
422.216	422.2149	-2.56	1	26541.4	C20 H33 N O7	(M+Na)+
423.2223	423.2182	-9.51	1	6481.6	C20 H33 N O7	(M+Na)+
424.2172	424.2207	8.12	1	3213.16	C20 H33 N O7	(M+Na)+

MSMS Spectrum

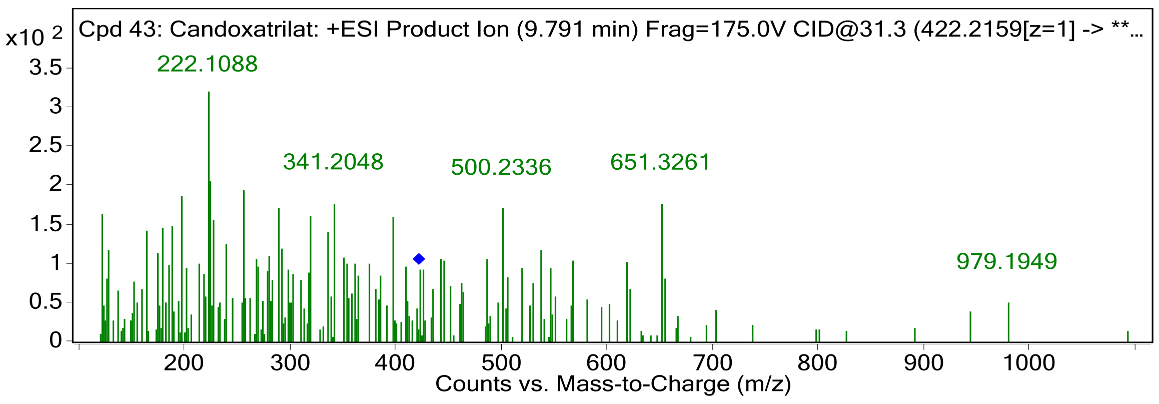

MS/MS Spectrum Peak List

m/z	Abund
122.0925	164.38
196.1377	188.31
222.1088	320.33
224.1281	205.74
255.2092	194.95
288.2895	171.66
318.8813	162.38
341.2048	178.37
500.2336	171.47
651.3261	178.06

Compound Structure

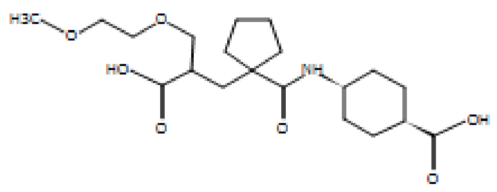

Compound Label	Name	*m/z*	RT	Algorithm	Mass
Cpd 44: Anapheline	**Anapheline**	225.1954	9.946	Auto MS/MS	224.1881

MS Spectrum

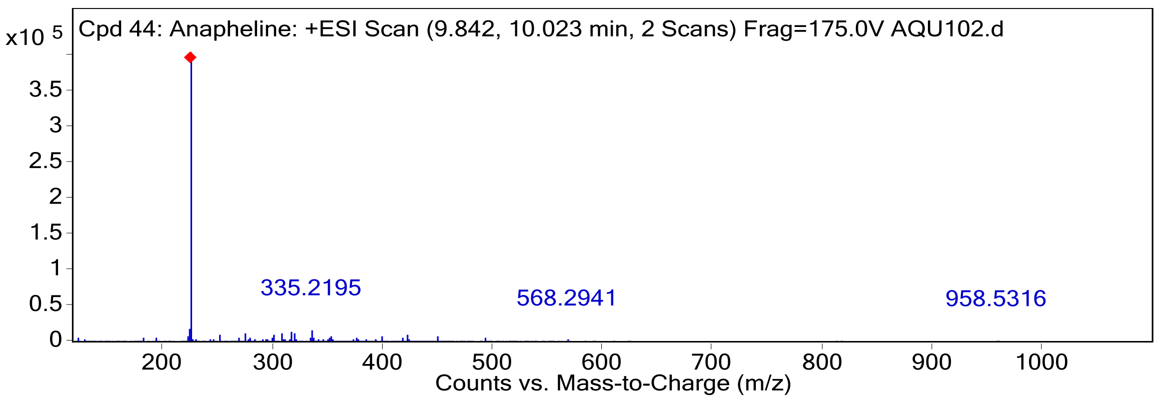

MS Zoomed Spectrum

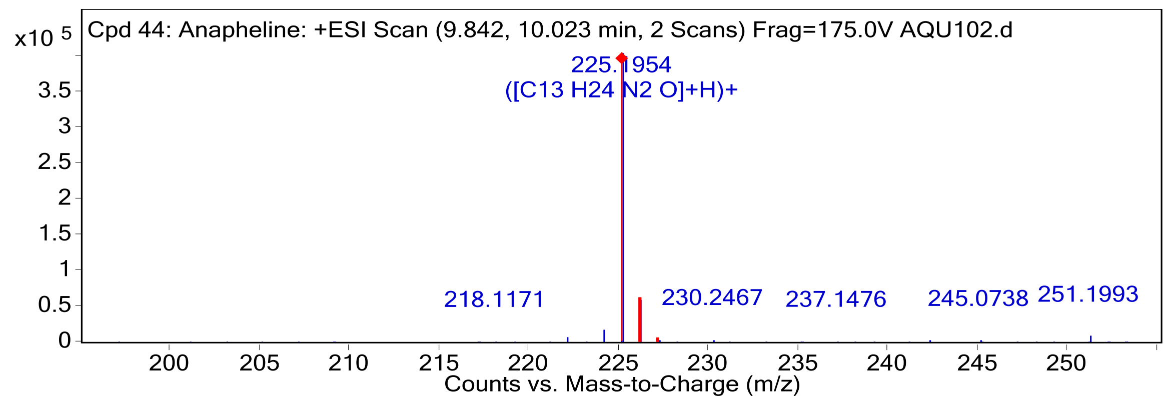

MS Spectrum Peak List

m/z	*Calc m/z*	Diff(ppm)	z	Abund	Formula	Ion
224.1276			1	17953.2		
225.1954	225.1961	3.44	1	403305.19	C13 H24 N2 O	(M+H)+
226.1986	226.1992	2.93	1	59329.98	C13 H24 N2 O	(M+H)+
227.2018	227.202	1	1	4740.09	C13 H24 N2 O	(M+H)+
274.2727			1	12126.85		
308.2104			1	11776.7		
317.2097			1	14469.54		
319.2248			1	11705.25		
335.2195			1	17537.02		
422.2163			1	11051.84		

MSMS Spectrum

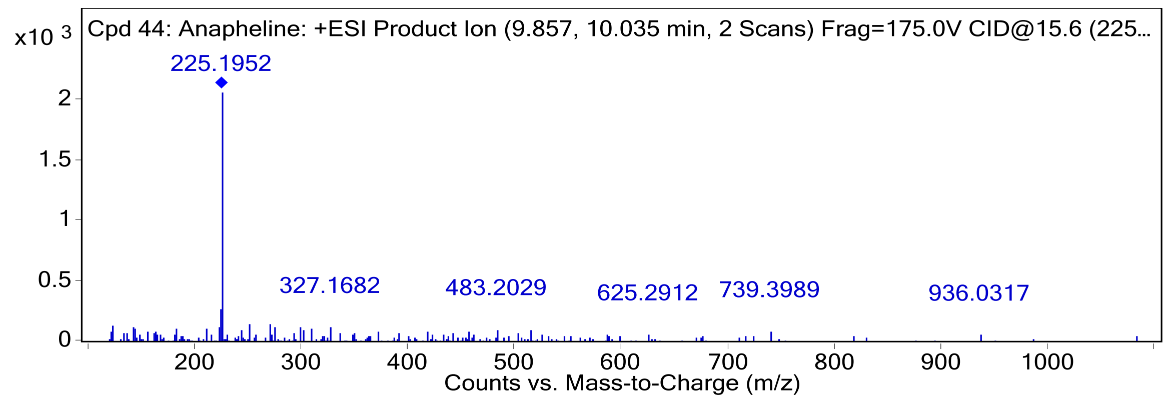

MS/MS Spectrum Peak List

m/z	z	Abund
123.1151		130.5
142.0786		128.78
223.1293		128.06
224.1276	1	278.01
225.1952	1	2069.59
226.1968	1	298.16
251.2006		142.56
270.1761		146.48
274.2708		121.88
327.1682		122.38

Compound Structure

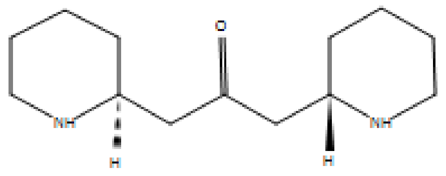

Compound Label	Name	*m/z*	RT	Algorithm	Mass
Cpd 45: 25-Methylgramisterol	**25-Methylgramisterol**	449.3815	9.972	Auto MS/MS	426.3921

MS Spectrum

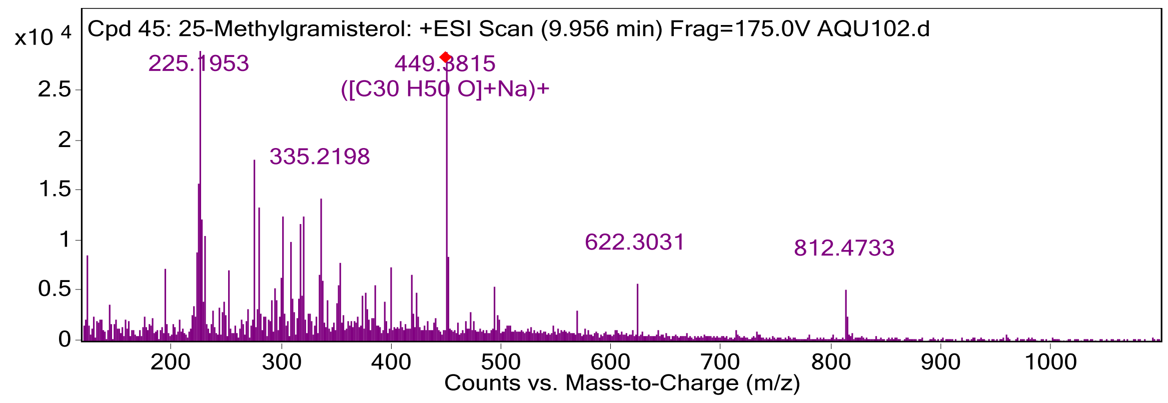

MS Zoomed Spectrum

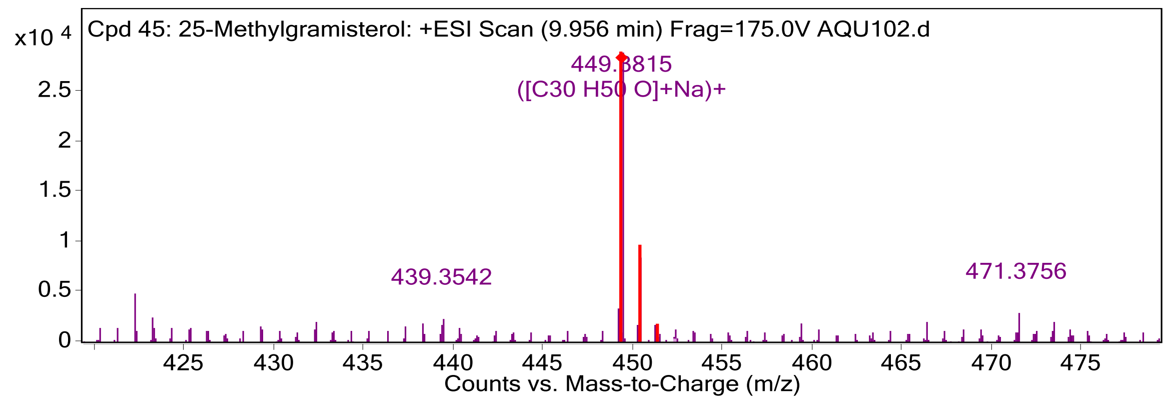

MS Spectrum Peak List

m/z	*Calc m/z*	Diff(ppm)	z	Abund	Formula	Ion
224.1275			1	15740.66		

225.1953			1	1089342.88		
226.1988			1	159804.2		
274.2727			1	18229.47		
279.0923			1	13393.99		
319.2245			1	12588.65		
335.2198			1	14369.5		
449.3815	449.3754	-13.5	1	28921.78	C30 H50 O	(M+Na)+
450.3843	450.3788	-12.28	1	8502.87	C30 H50 O	(M+Na)+
451.3876	451.3821	-12.22	1	927.57	C30 H50 O	(M+Na)+

MSMS Spectrum

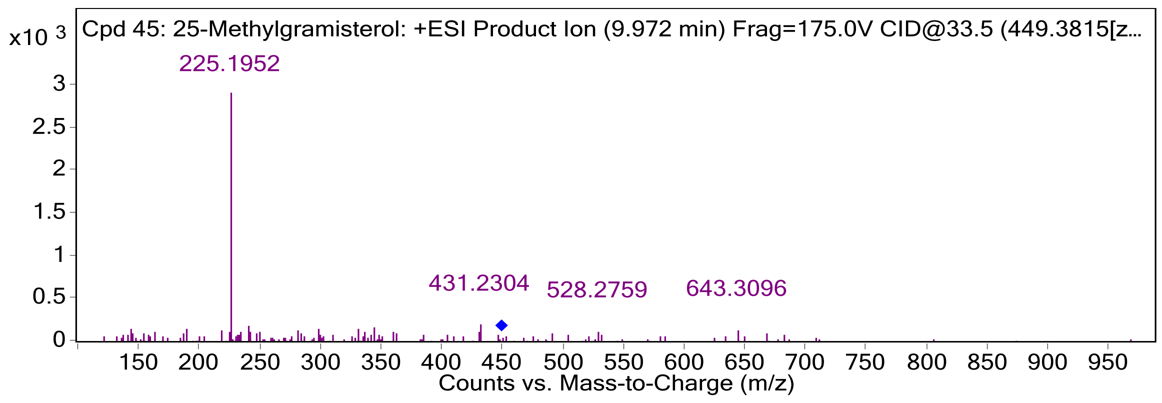

MS/MS Spectrum Peak List

m/z	z	Abund
143.0661		150.68
189.1641		153.94
225.1952	1	2915.97
226.1978	1	734.07
240.1391		194.66
281.069		142.04
297.2839		153.6
330.1223		160
344.0802		181.52
431.2304		201.41

Compound Structure

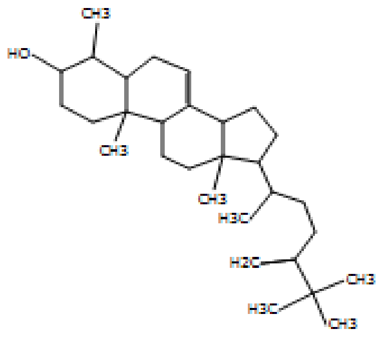

Compound Label	Name	*m/z*	RT	Algorithm	Mass
Cpd 46: Anapheline	**Anapheline**	225.1952	10.277	Auto MS/MS	224.1879

MS Spectrum

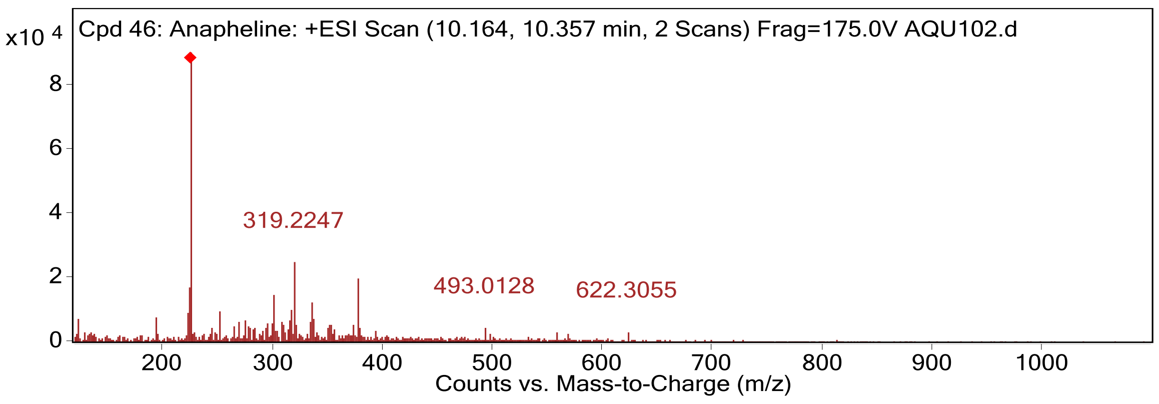

MS Zoomed Spectrum

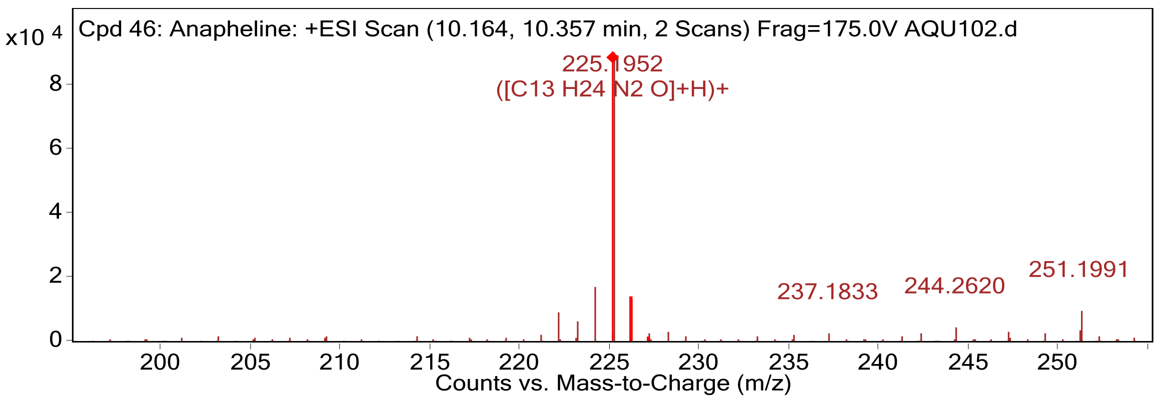

MS Spectrum Peak List

m/z	*Calc m/z*	Diff(ppm)	z	Abund	Formula	Ion
224.1274			1	17339.44		
225.1952	225.1961	4.37	1	90035.88	C13 H24 N2 O	(M+H)+
226.1985	226.1992	3.52	1	13533.45	C13 H24 N2 O	(M+H)+
227.2012	227.202	3.82	1	1005.37	C13 H24 N2 O	(M+H)+
251.1991				9633.34		
301.214			1	14904.58		
317.209			1	10045.74		
319.2247			1	25048.22		
335.2197			1	12555.59		
377.201			1	19859.57		

MSMS Spectrum

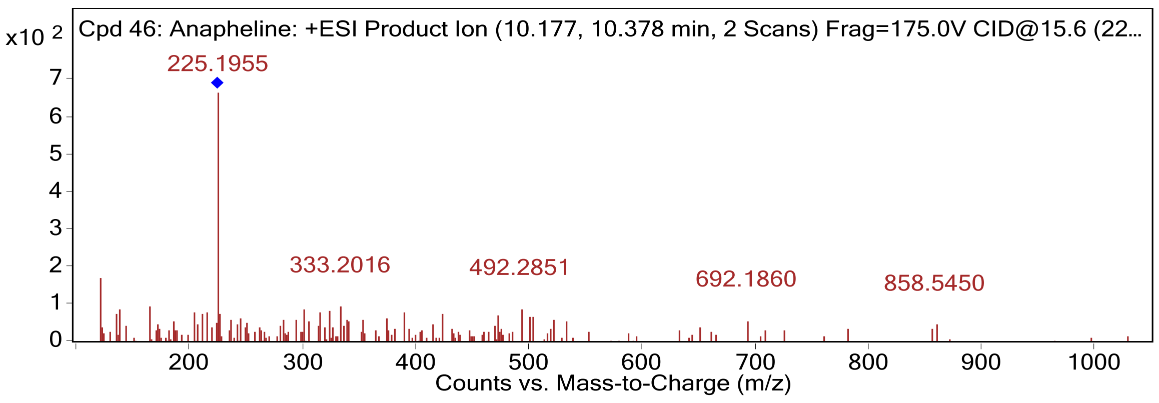

MS/MS Spectrum Peak List

m/z	z	Abund
121.0644		172.22
137.0954		88.7
165.0516		93.8
203.1439		80.58
224.128		187.78
225.1955	1	666.92
301.2143		87.29
323.1114		83.75
333.2016		94.98
492.2851		88.14

Compound Structure

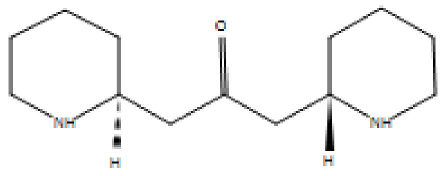

Compound Label	Name	*m/z*	RT	Algorithm	Mass

Cpd 47: Rhipocephalin	**Rhipocephalin**	377.2008	10.329	Auto MS/MS	376.1938

MS Spectrum

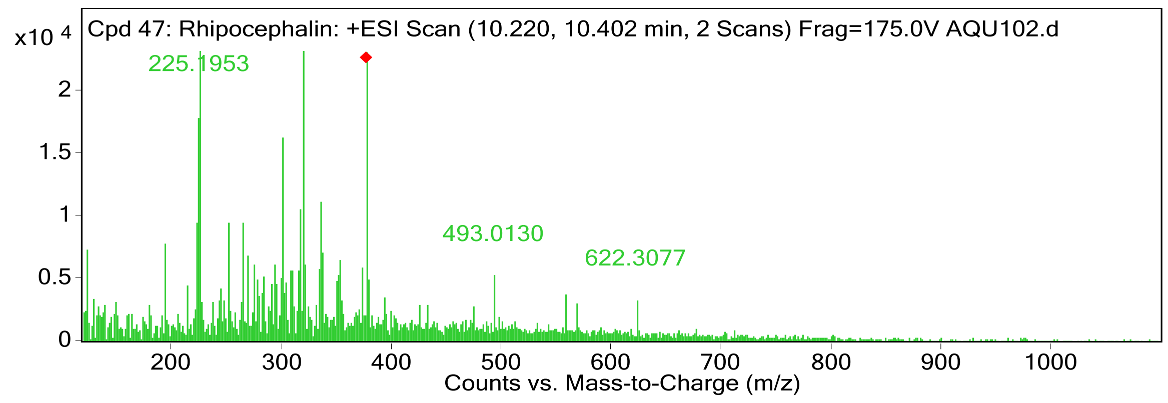

MS Zoomed Spectrum

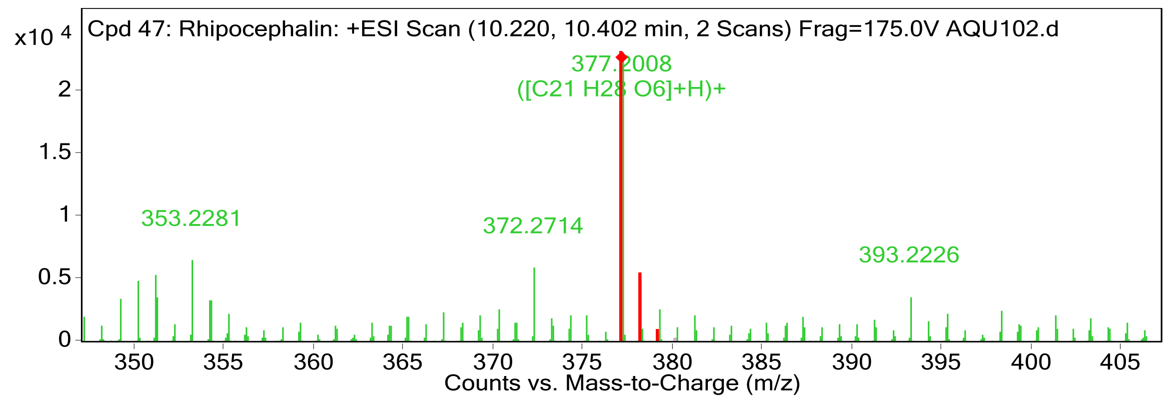

MS Spectrum Peak List

m/z	*Calc m/z*	Diff(ppm)	z	Abund	Formula	Ion
222.1116				9532.58		
224.1271			1	17866.73		
225.1953			1	46952.73		
301.2147			1	16348.38		
317.2091			1	10573.57		
319.2251			1	27066.72		
335.2205			1	11248.3		
377.2008	377.1959	-12.96	1	23157.7	C21 H28 O6	(M+H)+
378.2049	378.1993	-14.9	1	5063.78	C21 H28 O6	(M+H)+
379.2111	379.2019	-24.4	1	1102.42	C21 H28 O6	(M+H)+

MSMS Spectrum

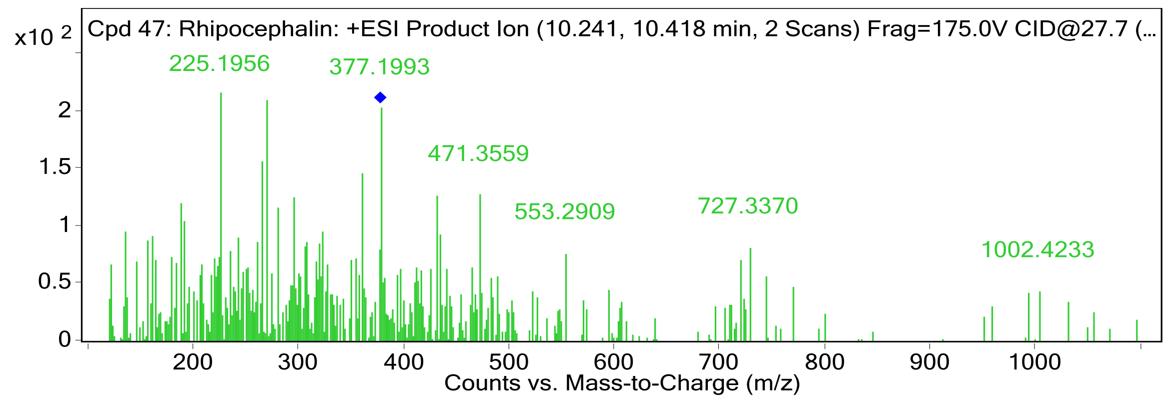

MS/MS Spectrum Peak List

m/z	z	Abund
187.1464		121.05
225.1956		216.81
265.1774		156.46
269.2093		210.15
279.194		116.27
295.0095		125.48
359.1888		146.14
377.1993	1	202.95
430.2245		126.8
471.3559		127.57

Compound Structure

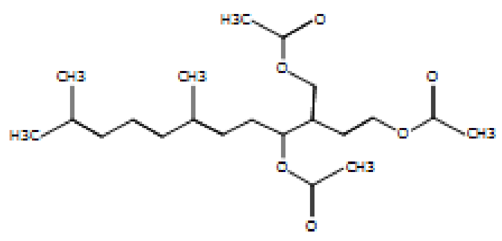

**Compound Label**	*m/z*	**RT**	**Algorithm**
Compound 48	306.2887	10.552	Auto MS/MS

MS Spectrum

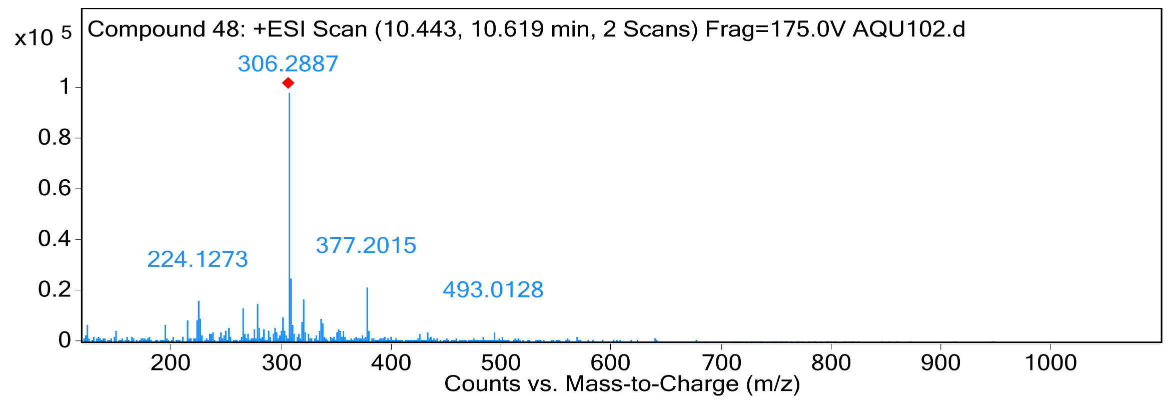

MS Zoomed Spectrum

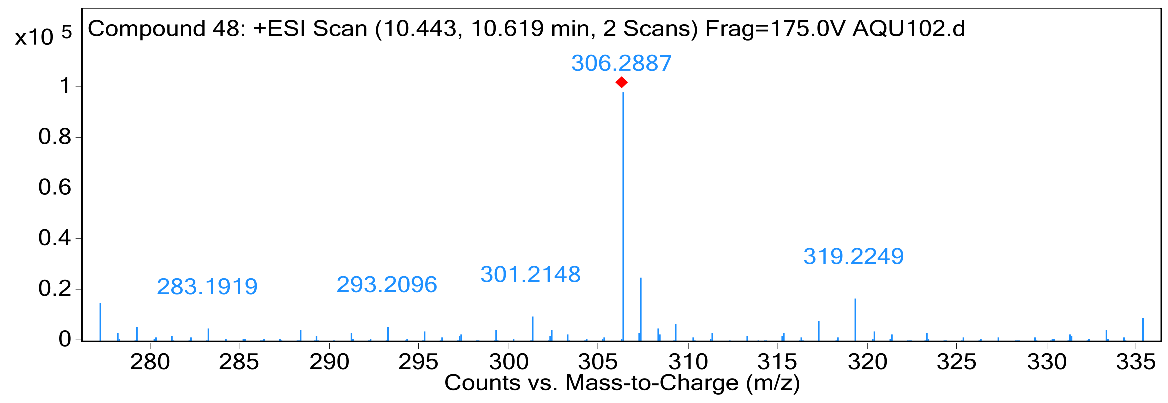

MS Spectrum Peak List

m/z	z	Abund
224.1273	1	16391.01
225.195		9679.98
265.1788	1	13605.45
277.1783	1	15192.53
301.2148	1	10068.42
306.2887	1	98316.45
307.292	1	25520.11
308.2946	1	3157.5
319.2249	1	17166.71
377.2015	1	21843.5

MSMS Spectrum

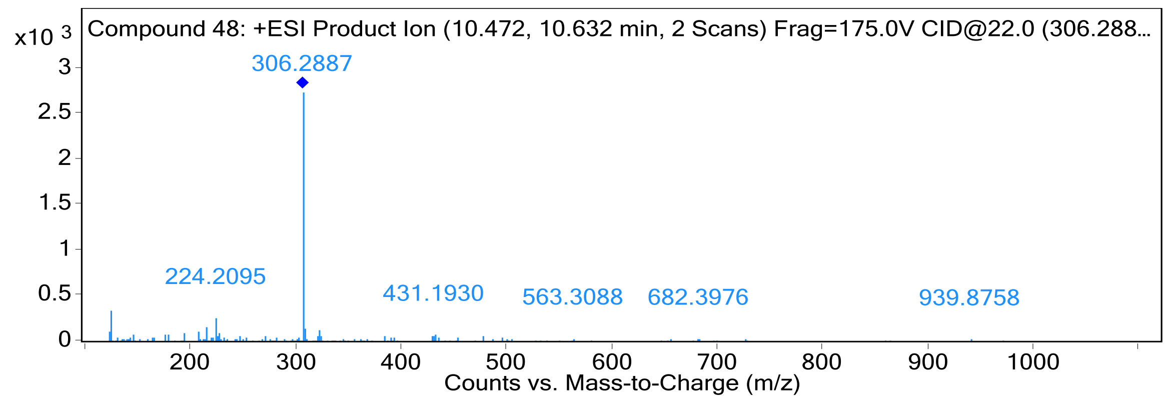

MS/MS Spectrum Peak List

m/z	z	Abund
123.077	1	113.44
125.1064		342.09
214.25		168.63
224.121		121.02
224.2095	1	260.02
306.2887	1	2735.73
307.2917	1	175.23
308.2098		144.55
308.2934	1	138.8
321.2049		139.08

**Compound Label**	***m/z***	**RT**	**Algorithm**
Compound 49	448.2147	10.714	Auto MS/MS

MS Spectrum

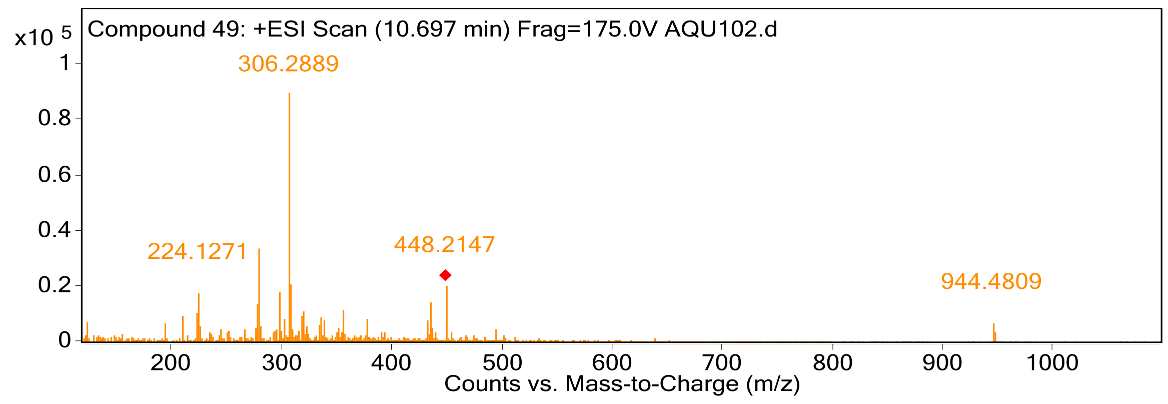

MS Zoomed Spectrum

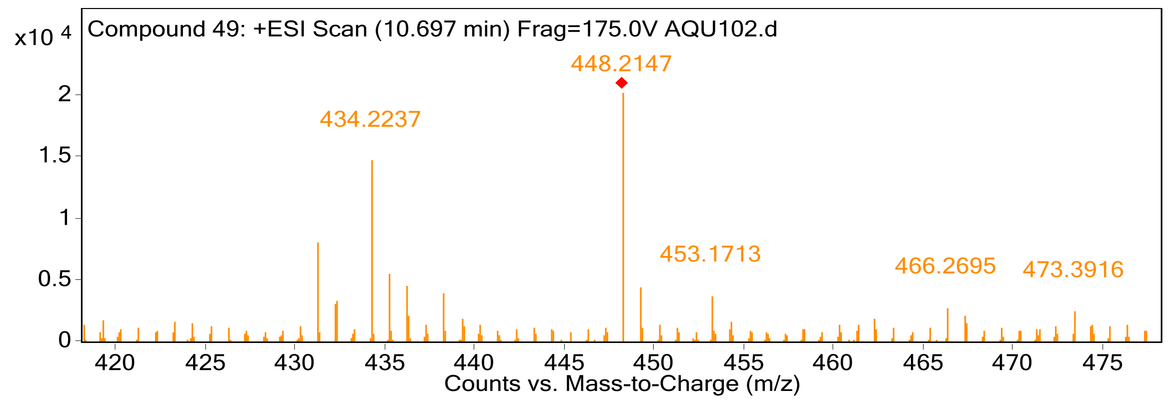

MS Spectrum Peak List

m/z	z	Abund
224.1271		17863.38
277.1786	1	13830.17
279.2305	1	34144.77
297.2409	1	18512.16
306.2889	1	90047.02
307.2923	1	20882.24
434.2237	1	14775.69
448.2147	1	20318.51
449.2182	1	4551.31
450.2189	1	1160.75

MSMS Spectrum

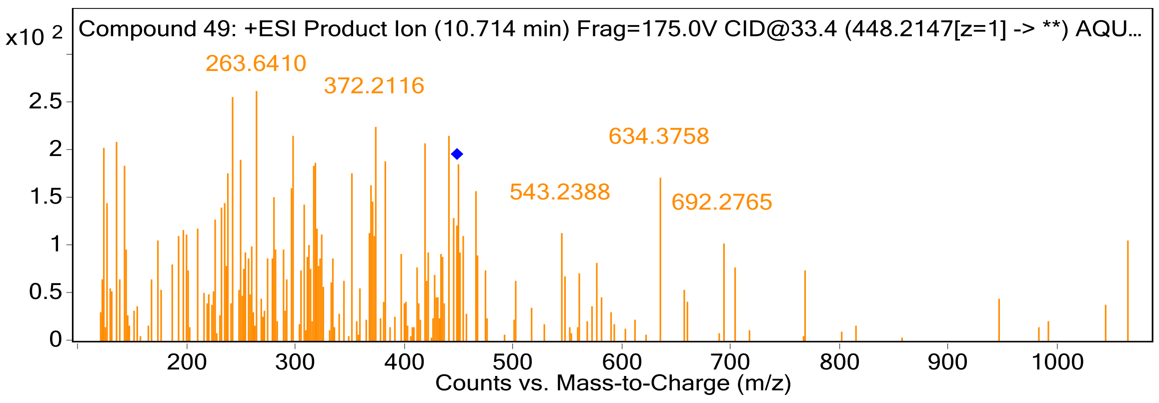

MS/MS Spectrum Peak List

m/z	Abund
122.0983	203.65
135.0806	209.09
241.2162	256.01
248.1315	190.54
263.641	262.25
297.2407	216.51
372.2116	225.33
381.2619	189.32
418.2321	208.32
440.3335	216.17

Compound Label	*m/z*	RT	Algorithm
Compound 50	436.1948	10.786	Auto MS/MS

MS Spectrum

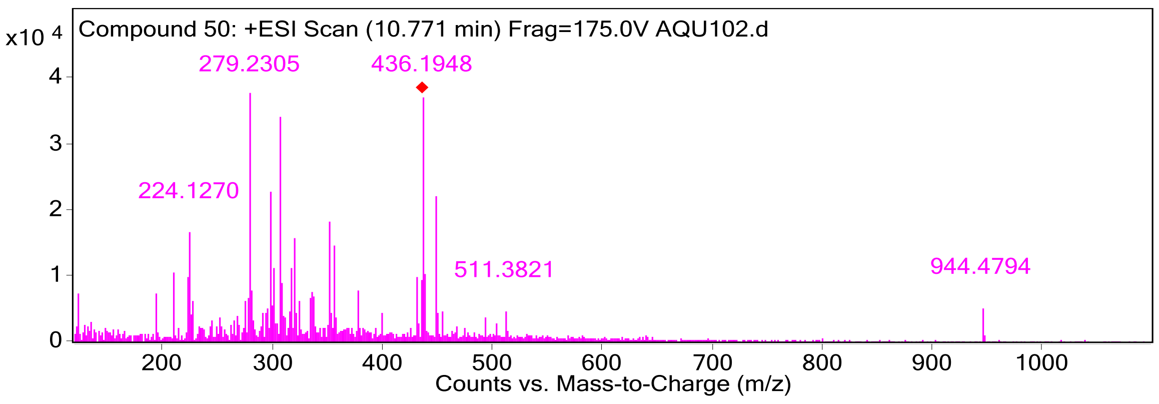

MS Zoomed Spectrum

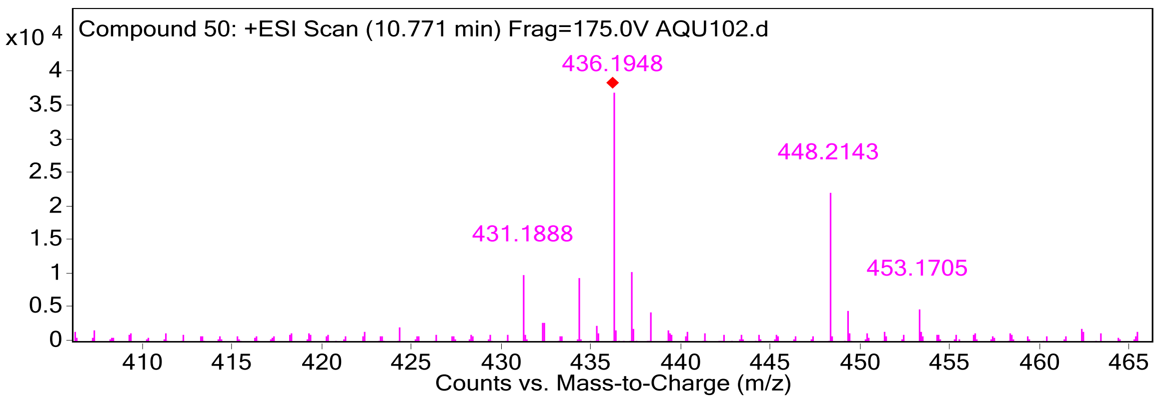

MS Spectrum Peak List

m/z	z	Abund
224.127	1	16743.18
279.2305	1	37902.55
297.2411	1	22860.23
306.2886	1	34196.25
319.2248	1	15822.33
351.2564	1	18346.12
436.1948	1	37061.23
437.1984	1	10354.32
438.2024	1	4440.16
448.2143	1	22105.69

MSMS Spectrum

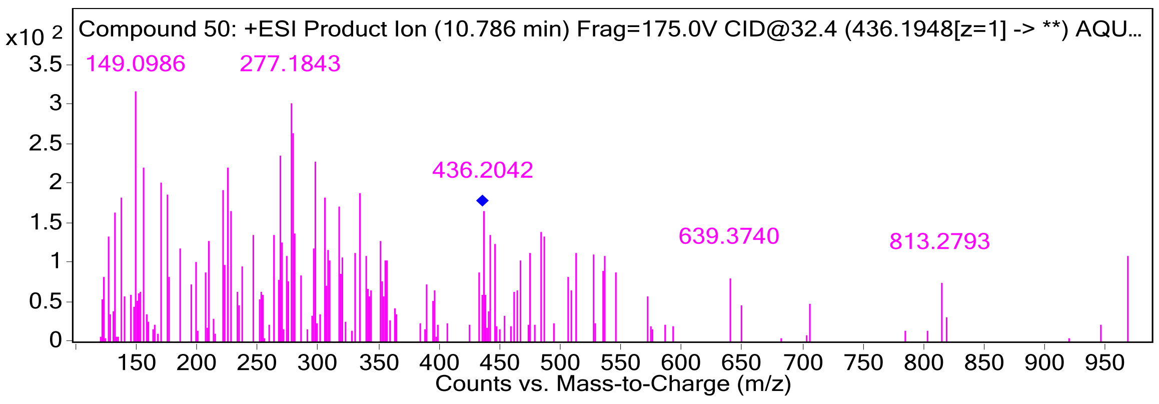

MS/MS Spectrum Peak List

m/z	z	Abund
149.0986		316.28
155.0713		220.41
169.1021		201.7
221.1556		192.88
224.1281		220.28
268.0581		235.83
277.1843		302.36
278.205		264.88
297.2409		227.86
333.168	1	189.38

Compound Label	*m/z*	RT	Algorithm
Compound 51	304.2979	11.85	Auto MS/MS

MS Spectrum

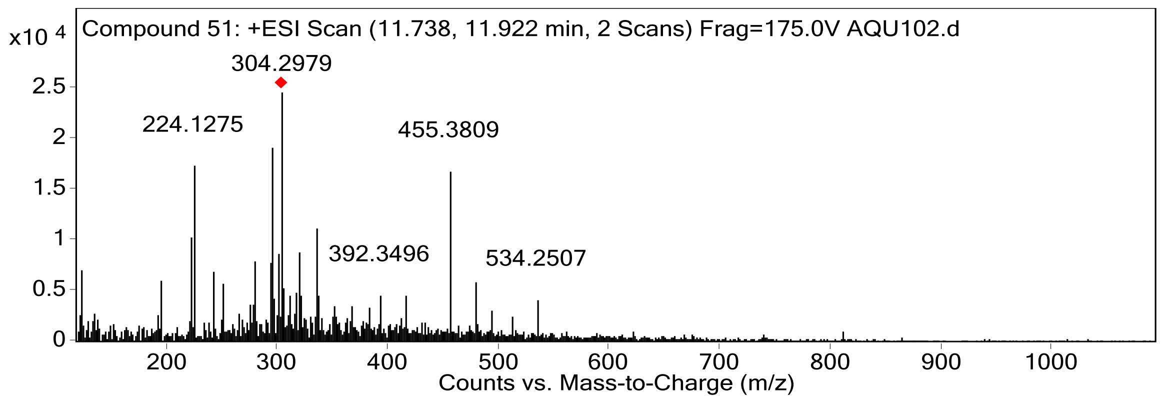

MS Zoomed Spectrum

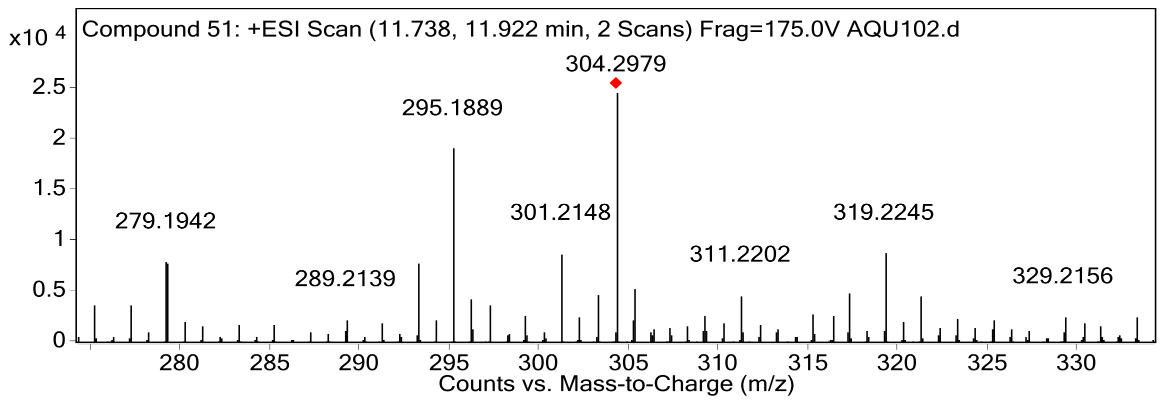

MS Spectrum Peak List

m/z	z	Abund
222.1114		10334.86
224.1275	1	17380.98
295.1889	1	19059.02
301.2148	1	8749.03
304.2979	1	24609.19
305.301	1	5285.57
306.2943	1	1349
319.2245	1	8757.17
335.2193	1	11169.34
455.3809	1	16838.56

MSMS Spectrum

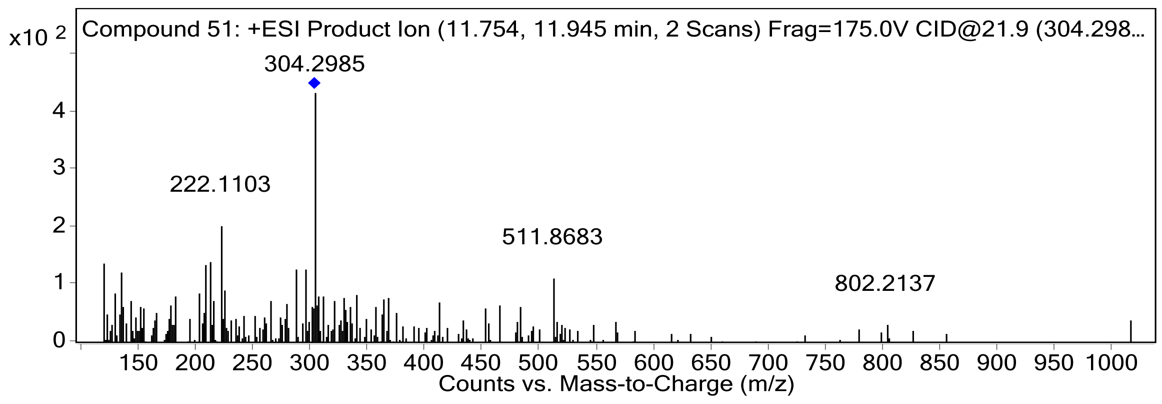

MS/MS Spectrum Peak List

m/z	Abund
120.08	136.97
135.0786	120.69
135.1205	105.73
208.8012	134.05
212.2345	140.58
222.1103	203.1
288.1259	125.86
295.1885	128.05
304.2985	434.17
511.8683	111.58

Compound Label	Name	*m/z*	RT	Algorithm	Mass
Cpd 52: 5-Tricosyl-1,3-benzenediol	**5-Tricosyl-1,3-benzenediol**	455.3809	12.192	Auto MS/MS	432.392

MS Spectrum

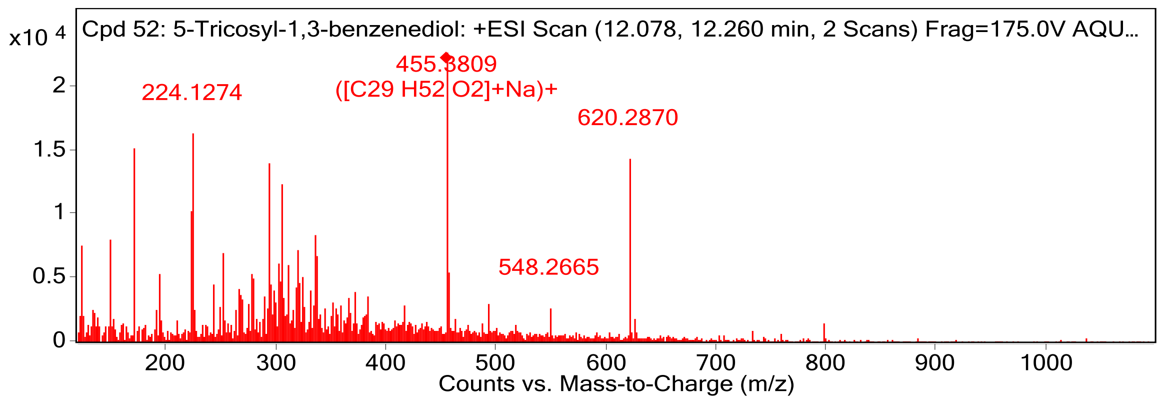

MS Zoomed Spectrum

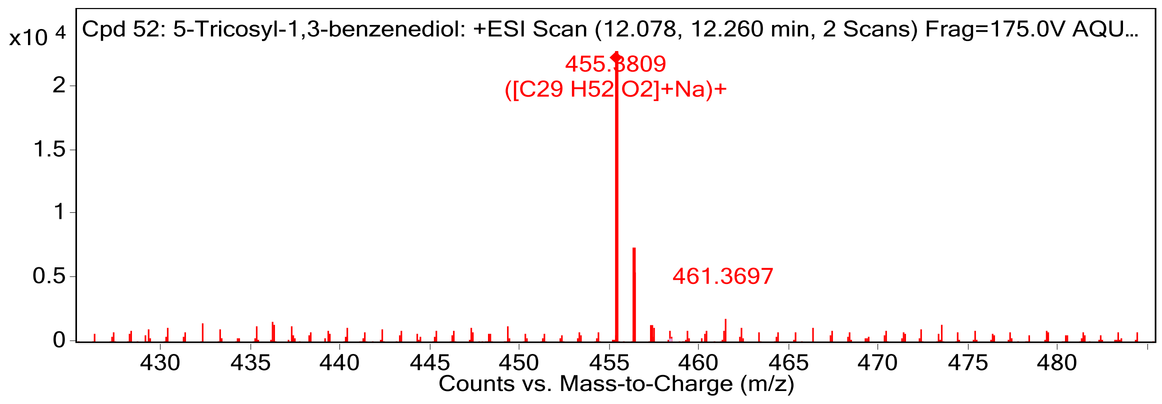

MS Spectrum Peak List

m/z	*Calc m/z*	Diff(ppm)	z	Abund	Formula	Ion
170.0961			1	15156.87		
222.1112				10299.34		
224.1274			1	16331.14		
293.2091			1	14044.06		
305.2092			1	12427.99		
335.2195			1	8453.46		
455.3809	455.386	11	1	22666.5	C29 H52 O2	(M+Na)+
456.3853	456.3894	8.82	1	5530.59	C29 H52 O2	(M+Na)+
457.389	457.3926	7.81	1	1197.48	C29 H52 O2	(M+Na)+
620.287			1	14364.89		

MSMS Spectrum

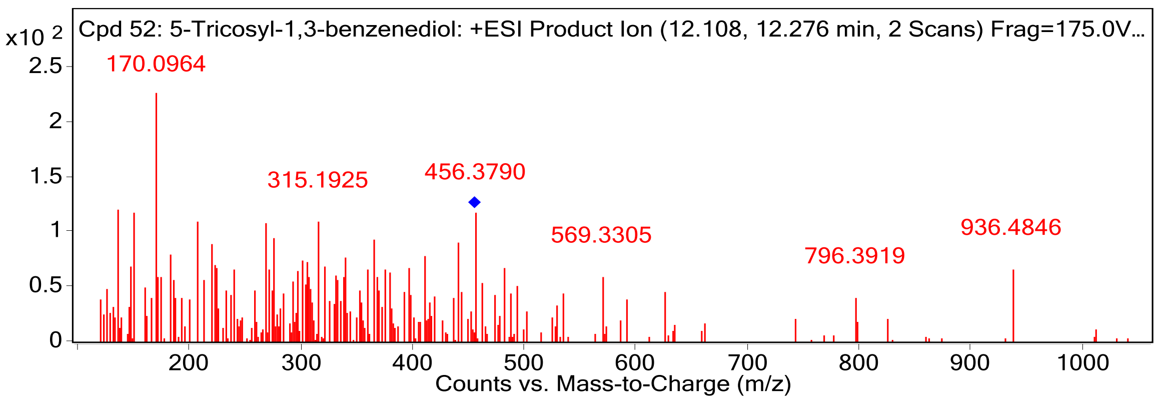

MS/MS Spectrum Peak List

m/z	z	Abund
136.0621		121.55
149.0592		117.82
170.0964	1	227.41
207.1349		109.96
267.1559		109.32
274.2779		95.22
315.1925		110.14
365.262		93.38
455.3818		98.14
456.379		117.59

Compound Structure

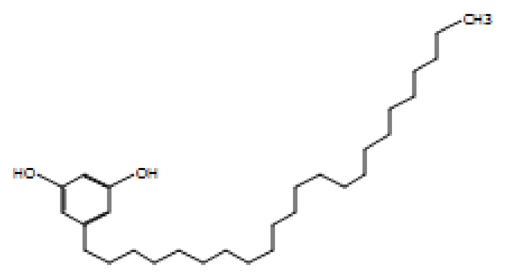

Compound Label	Name	*m/z*	RT	Algorithm	Mass
Cpd 53: 12-(2,3-Dihydroxycyclopentyl)-2-dodecanone	**12-(2,3-Dihydroxycyclopentyl)-2-dodecanone**	307.2252	12.506	Auto MS/MS	284.2361

MS Spectrum

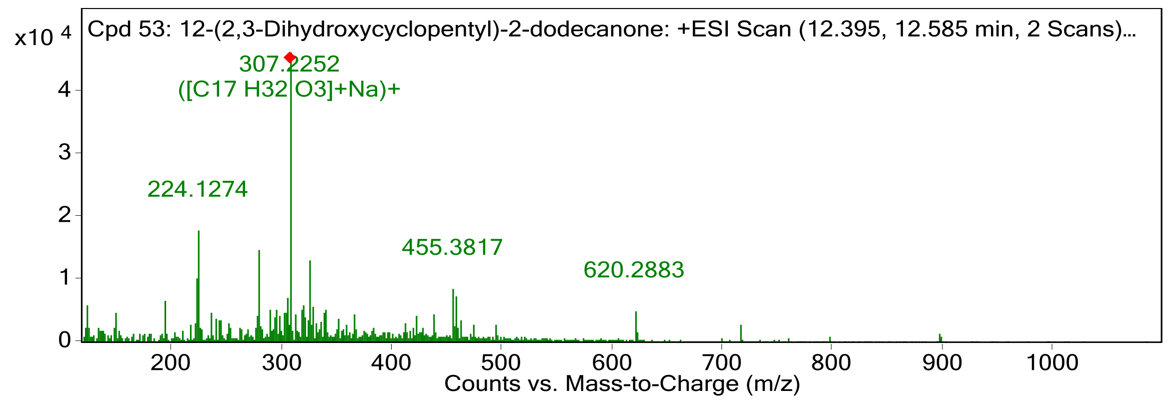

MS Zoomed Spectrum

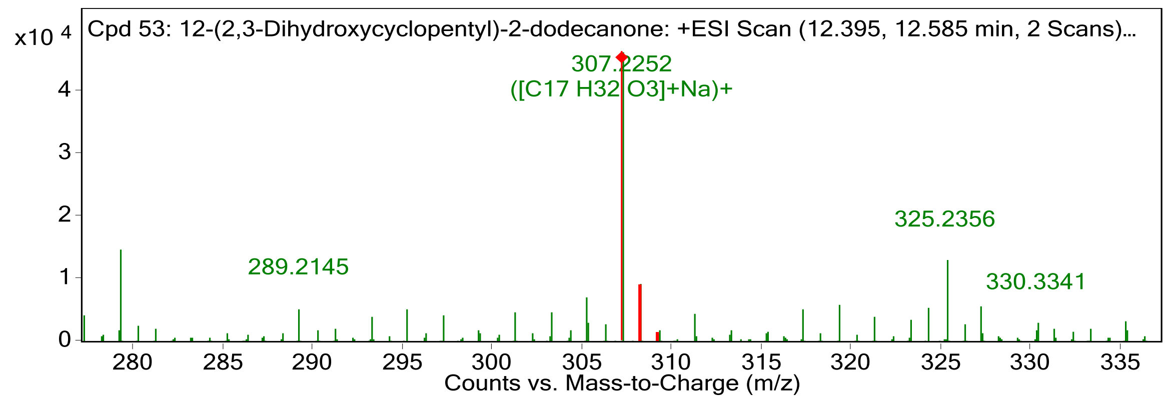

MS Spectrum Peak List

m/z	*Calc m/z*	Diff(ppm)	z	Abund	Formula	Ion
222.1116				10178.98		
224.1274			1	17812.06		
279.2306			1	14689.35		
305.2095				7137.34		
307.2252	307.2244	-2.61	1	46170.02	C17 H32 O3	(M+Na)+
308.228	308.2278	-0.66	1	9188.57	C17 H32 O3	(M+Na)+
309.2385	309.2305	-25.74	1	1824.53	C17 H32 O3	(M+Na)+
325.2356			1	12991.55		
455.3817			1	8533.88		
457.3964			1	7423.08		

MSMS Spectrum

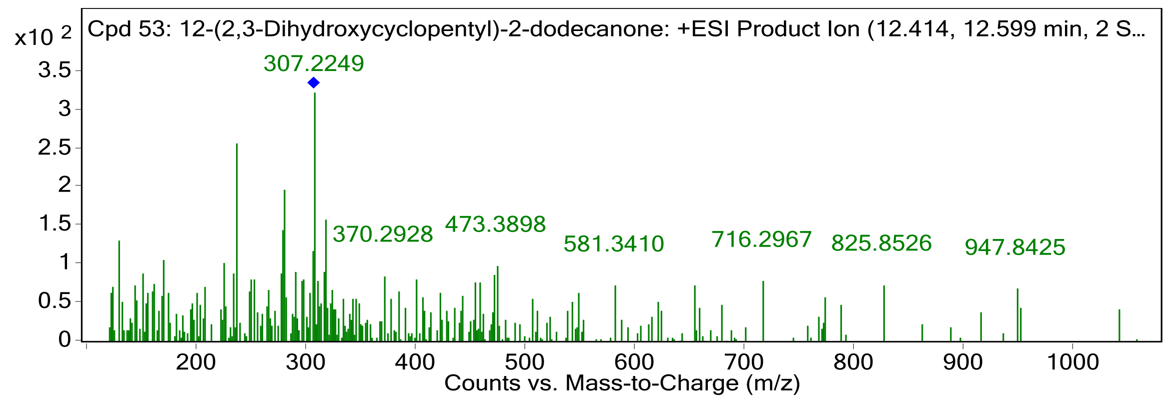

MS/MS Spectrum Peak List

m/z	Abund
128.0634	130.53
169.0484	105.87
224.1297	101.98
235.2063	256.19
278.2478	144.78
279.2302	197.09
306.2085	103.99
306.2425	117.49
307.2249	323.17
317.2094	158.46

Compound Structure

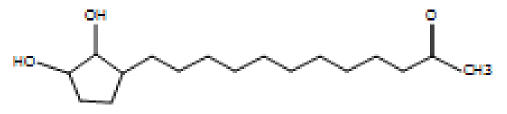

Compound Label	Name	*m/z*	RT	Algorithm	Mass
Cpd 54: Ximenoylacetone	**Ximenoylacetone**	457.397	12.701	Auto MS/MS	434.4078

MS Spectrum

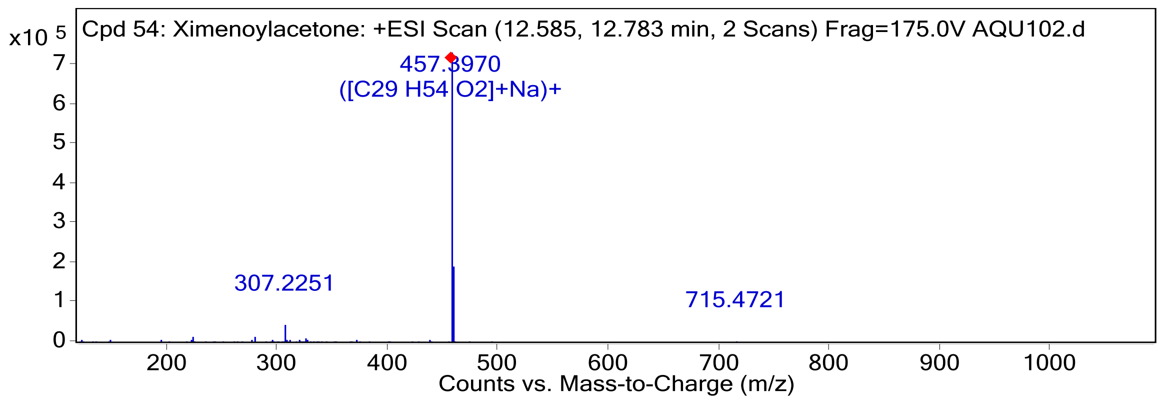

MS Zoomed Spectrum

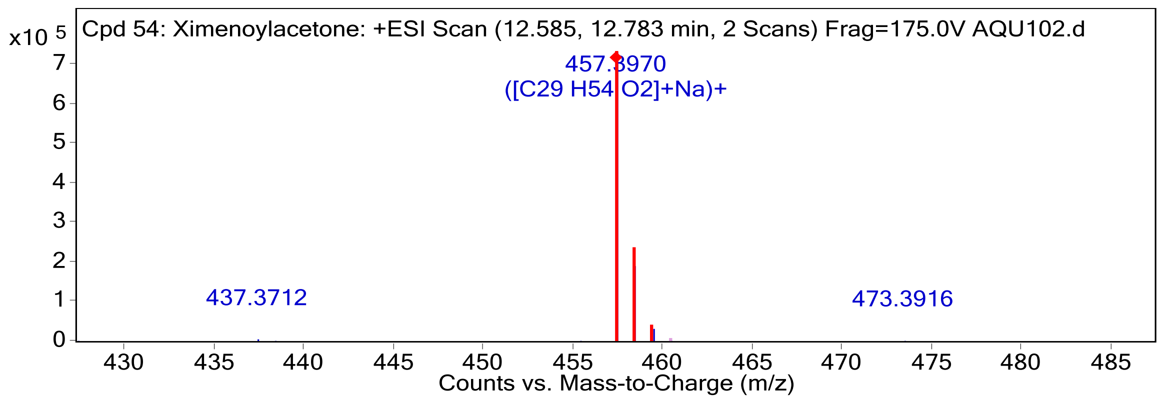

MS Spectrum Peak List

m/z	*Calc m/z*	Diff(ppm)	z	Abund	Formula	Ion
222.1114				9405.99		
224.1272			1	16482.14		
279.2305			1	15755.42		
307.2251			1	44894.21		
308.2278			1	9012.91		
311.1678				7299.16		
325.2355			1	10578.93		
457.397	457.4016	10.14	1	731881.19	C29 H54 O2	(M+Na)+
458.4005	458.405	9.74	1	193175.03	C29 H54 O2	(M+Na)+
459.4031	459.4082	11.14	1	33352.68	C29 H54 O2	(M+Na)+

MSMS Spectrum

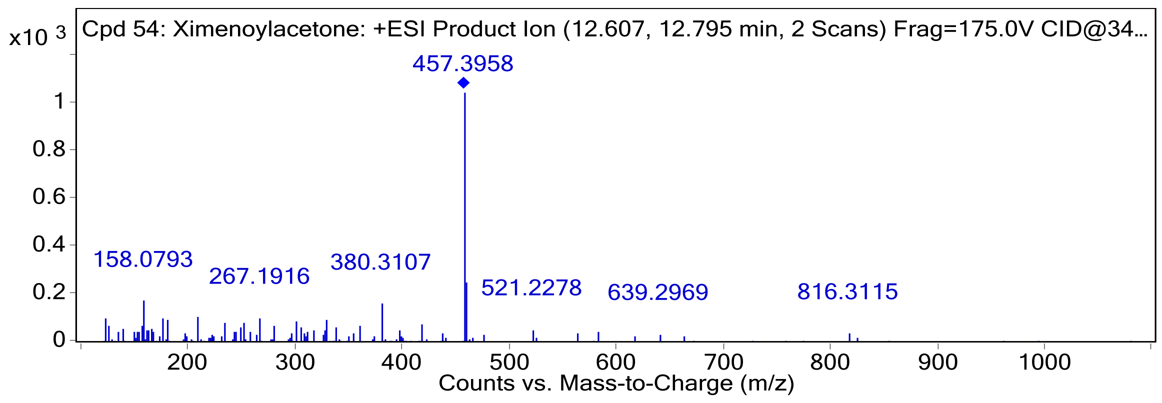

MS/MS Spectrum Peak List

m/z	z	Abund
123.0808		98.99
158.0793		171.97
176.1452		99.77
180.8902		93.6
209.1267		106.06
267.1916		100.97
329.1809		93.93
380.3107		160.29
457.3958	1	1043.54
458.4003	1	251.09

Compound Structure

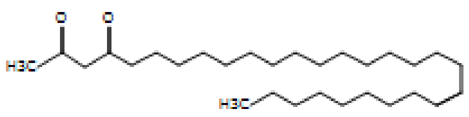

Compound Label	Name	*m/z*	RT	Algorithm	Mass
Cpd 55: Ximenoylacetone	**Ximenoylacetone**	457.3974	13.06	Auto MS/MS	434.408

MS Spectrum

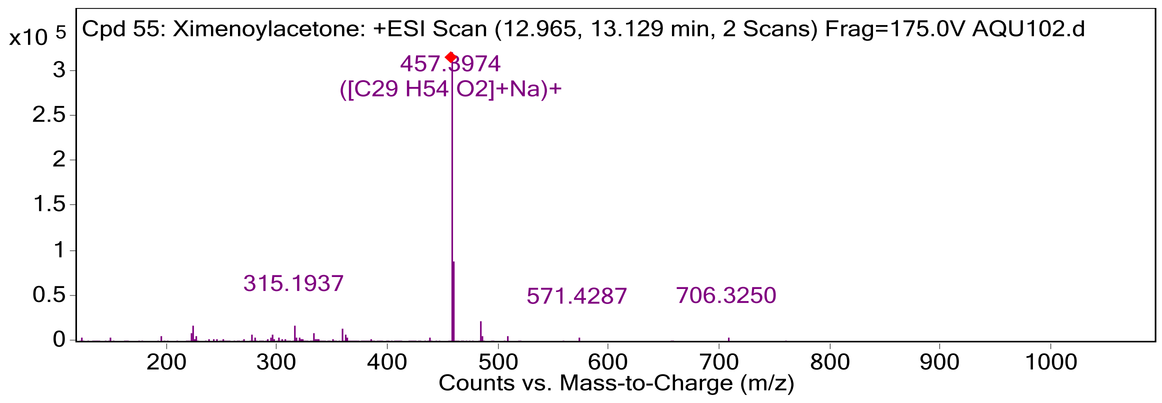

MS Zoomed Spectrum

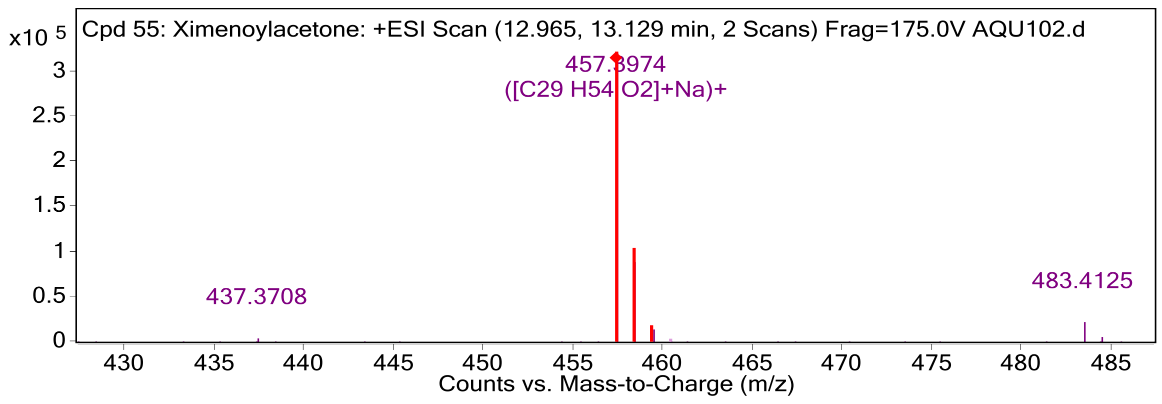

MS Spectrum Peak List

m/z	*Calc m/z*	Diff(ppm)	z	Abund	Formula	Ion
222.1115				9243.25		
224.1273			1	18278.61		
295.1712			1	8662.36		
315.1937			1	18625.79		
332.3293			1	9208.18		
358.9771			1	14285.76		
457.3974	457.4016	9.26	1	321428.78	C29 H54 O2	(M+Na)+
458.4004	458.405	10.1	1	88649.86	C29 H54 O2	(M+Na)+
459.4032	459.4082	11.01	1	15112.96	C29 H54 O2	(M+Na)+
483.4125			1	22617.66		

MSMS Spectrum

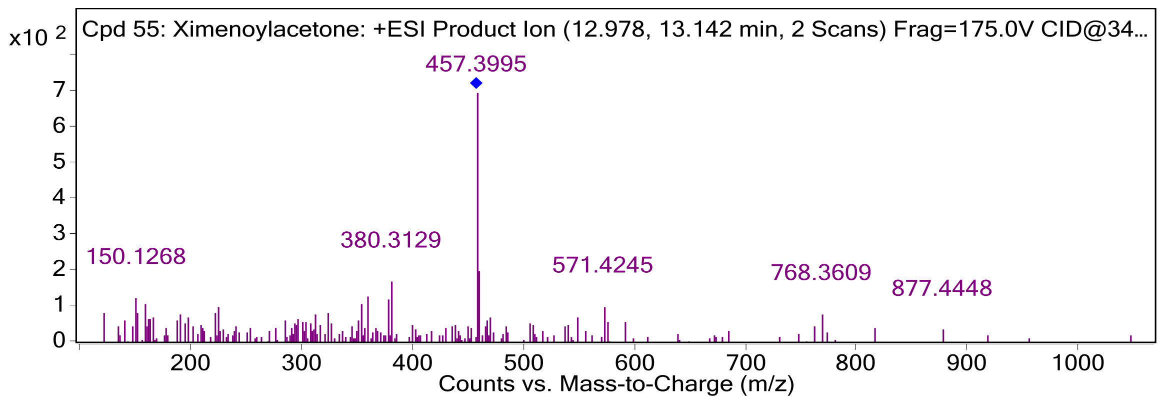

MS/MS Spectrum Peak List

m/z	z	Abund
150.1268		124.61
158.0804		107.12
224.1277		101.04
353.2682		106.56
358.9751		127.58
377.2666		119.48
380.3129		170.65
457.3995	1	698.35
458.3991	1	200.13
571.4245	1	100.58

Compound Structure

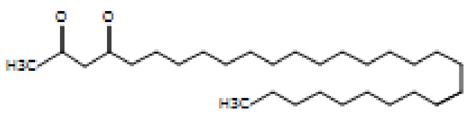

Compound Label	Name	*m/z*	RT	Algorithm	Mass
Cpd 56: Stigmastanol	**Stigmastanol**	439.3865	13.425	Auto MS/MS	416.3973

MS Spectrum

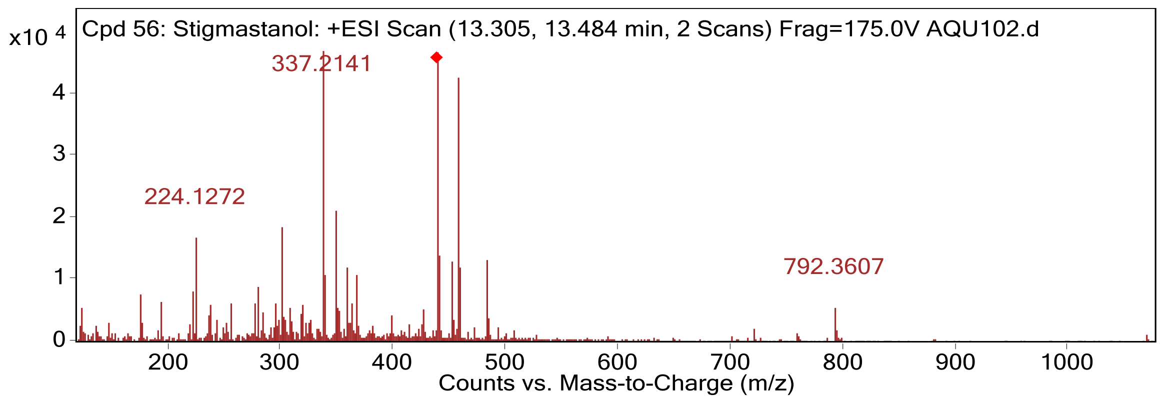

MS Zoomed Spectrum

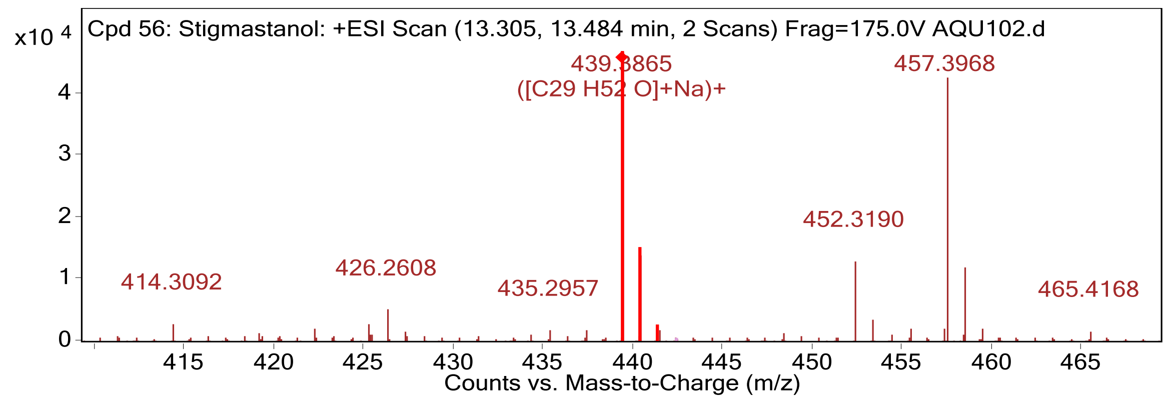

MS Spectrum Peak List

m/z	*Calc m/z*	Diff(ppm)	z	Abund	Formula	Ion
224.1272			1	16772.89		
301.2145			1	18645.15		
337.2141			1	47139.05		
349.2142			1	21204.42		
439.3865	439.391	10.27	1	46719.24	C29 H52 O	(M+Na)+
440.39	440.3944	10.12	1	13924.18	C29 H52 O	(M+Na)+
441.3933	441.3978	10.18	1	1913.31	C29 H52 O	(M+Na)+
452.319			1	13082.49		
457.3968			1	42524.97		
483.4122			1	13132.16		

MSMS Spectrum

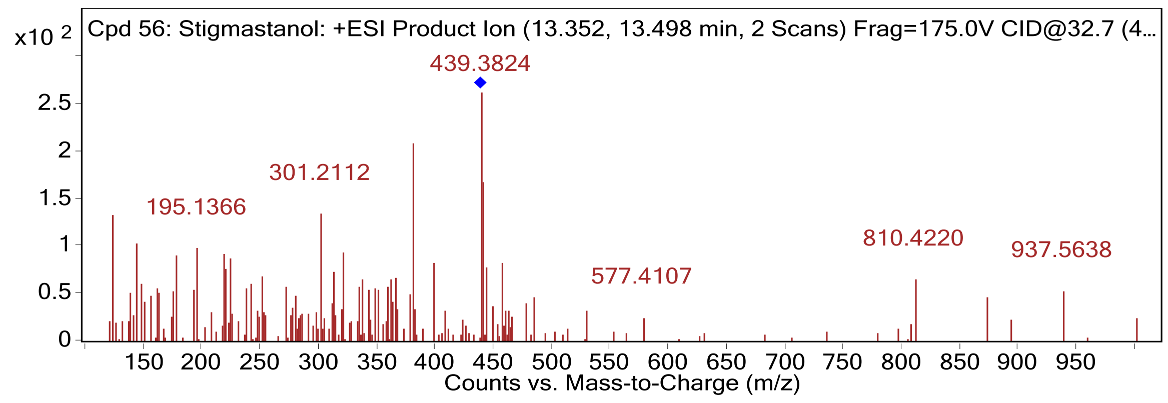

MS/MS Spectrum Peak List

m/z	z	Abund
123.0805		133.32
144.0792		103.34
177.1261		91.55
195.1366		98.52
219.135		93.5
301.2112		134.92
321.2478		94.22
380.3104	1	208.77
439.3824		263.29
440.3886	2	168.43

Compound Structure

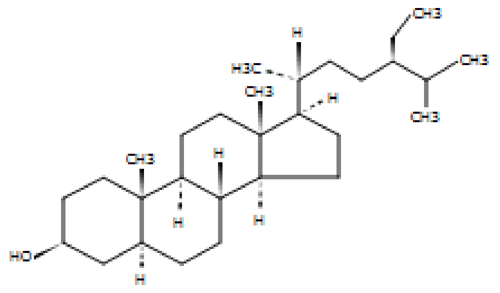

Compound Label	Name	*m/z*	RT	Algorithm	Mass
Cpd 57: Stigmastanol	**Stigmastanol**	439.3865	13.728	Auto MS/MS	416.3972

MS Spectrum

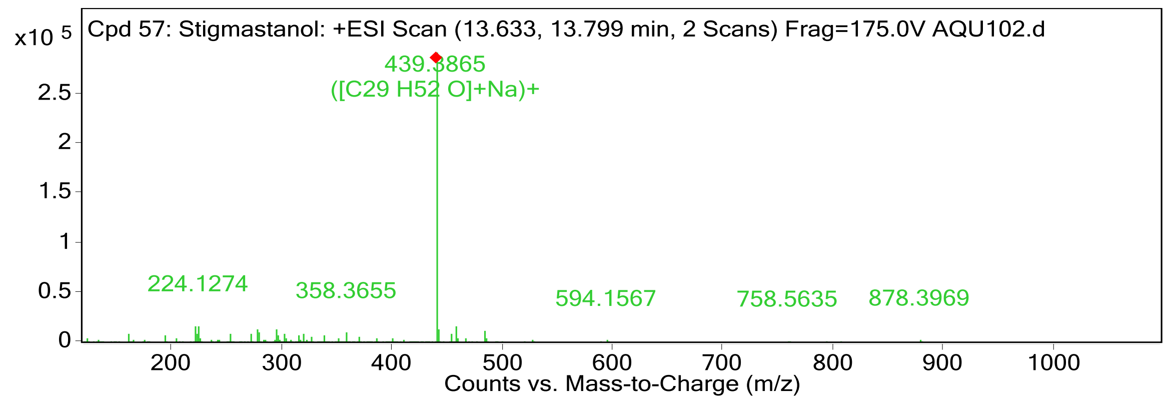

MS Zoomed Spectrum

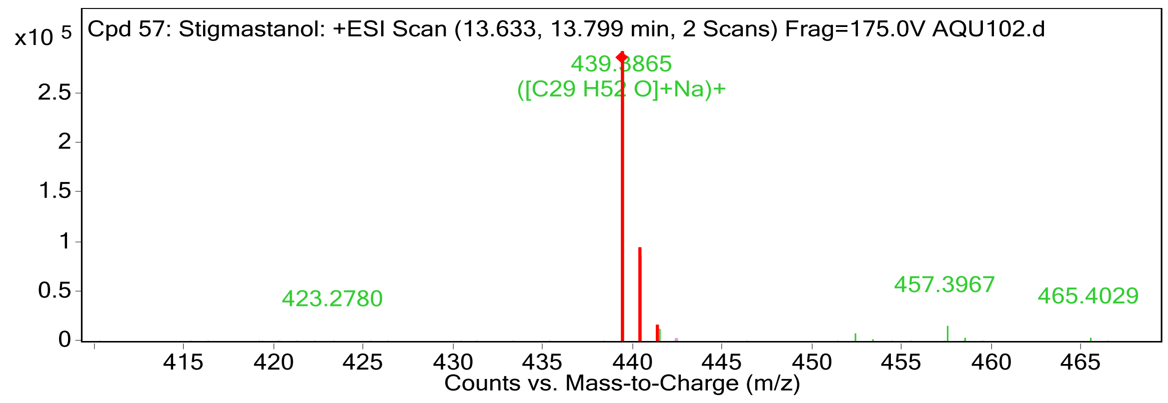

MS Spectrum Peak List

m/z	*Calc m/z*	Diff(ppm)	z	Abund	Formula	Ion
221.1161				16566.13		
224.1274			1	17240.94		

277.2169			1	13516.06		
279.2303			1	11209.7		
294.2048				13029.79		
439.3865	439.391	10.22	1	292397.84	C29 H52 O	(M+Na)+
440.3896	440.3944	10.91	1	88148.22	C29 H52 O	(M+Na)+
441.3924	441.3978	12.11	1	13948.8	C29 H52 O	(M+Na)+
457.3967			1	15940.15		
483.4124			1	12525.09		

MSMS Spectrum

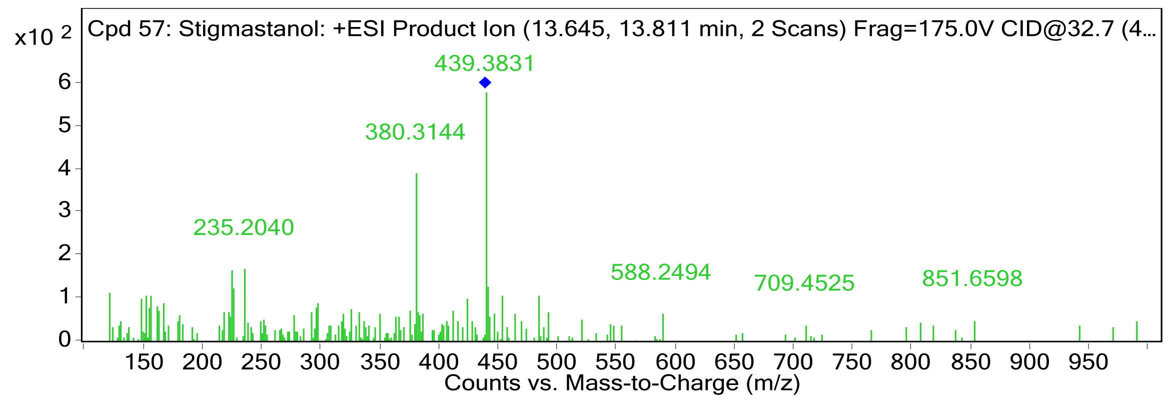

MS/MS Spectrum Peak List

m/z	z	Abund
121.0595		115.61
152.0565		107
224.128		166.91
225.2184		124.32
235.204		169.19
380.3144	1	391.6
439.3831	1	580.1
440.3846	1	127.48
452.2677		109.07
483.4164		108.74

Compound Structure

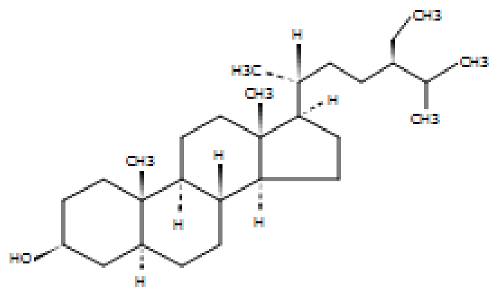

Compound Label	Name	*m/z*	RT	Algorithm	Mass
Cpd 58: Stigmastanol	**Stigmastanol**	439.3866	14.07	Auto MS/MS	416.3972

MS Spectrum

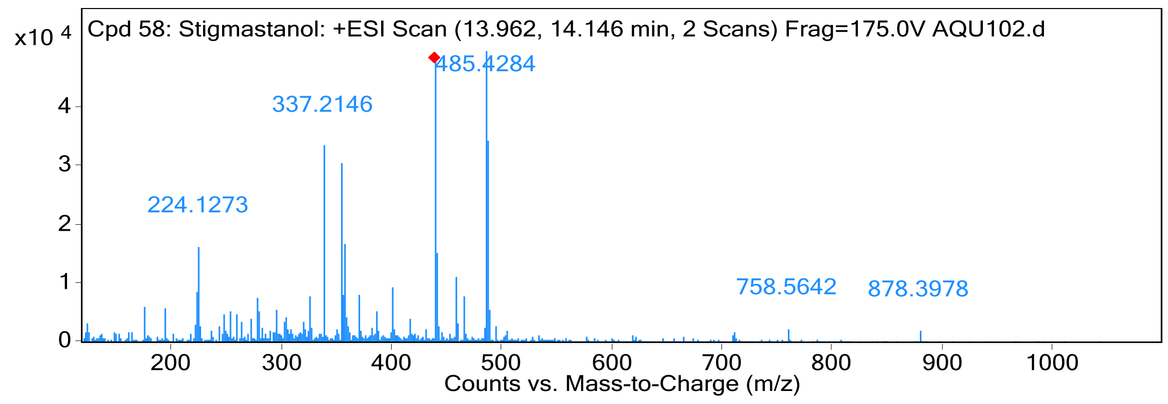

MS Zoomed Spectrum

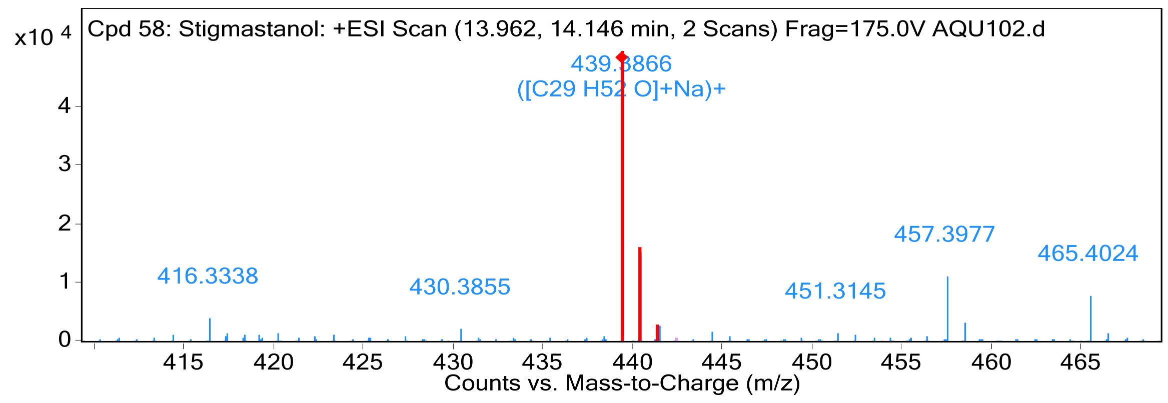

MS Spectrum Peak List

m/z	*Calc m/z*	Diff(ppm)	z	Abund	Formula	Ion
224.1273			1	16404.22		
337.2146			1	33599.65		
353.9837			1	30509.86		
355.9796			1	16707.8		
439.3866	439.391	10.19	1	49451.81	C29 H52 O	(M+Na)+
440.3898	440.3944	10.47	1	15393.32	C29 H52 O	(M+Na)+
441.3918	441.3978	13.6	1	2735.36	C29 H52 O	(M+Na)+
457.3977			1	11311.46		
485.4284			1	104819.71		
486.4312			1	34347.93		

MSMS Spectrum

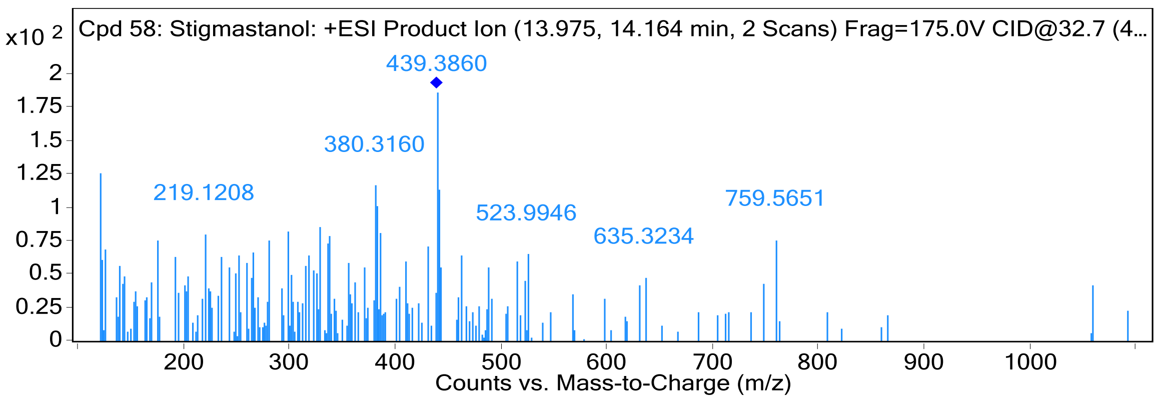

MS/MS Spectrum Peak List

m/z	Abund
121.0405	126.59
121.1	104.84
219.1208	80.8
298.273	82.33
327.176	86.35
380.316	117.08
381.3154	101.72
385.3314	81.68
439.386	187.19
440.2173	113.7

Compound Structure

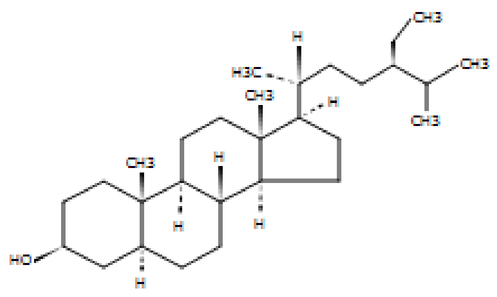

Compound Label	Name	*m/z*	RT	Algorithm	Mass
Cpd 59: (Z)-22-Hentriacontene-2,4-dione	**(Z)-22-Hentriacontene-2,4-dione**	485.4284	14.07	Auto MS/MS	462.439

MS Spectrum

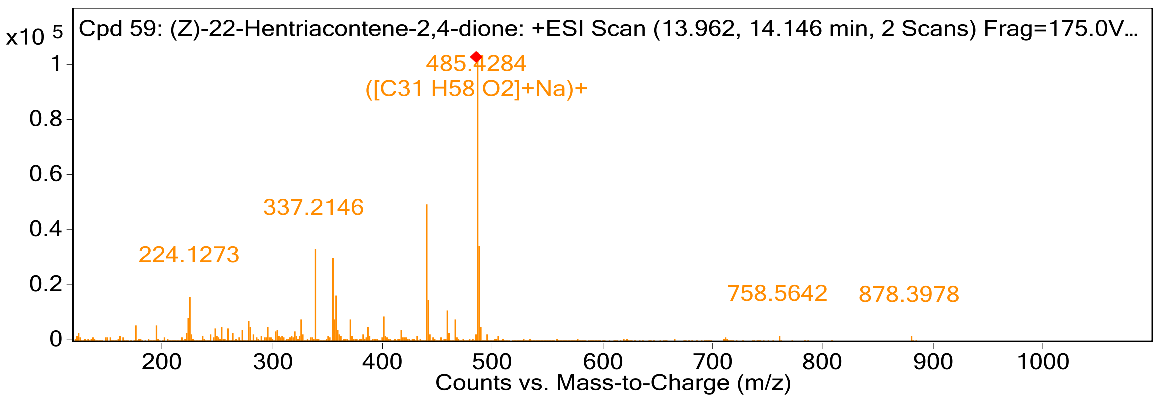

MS Zoomed Spectrum

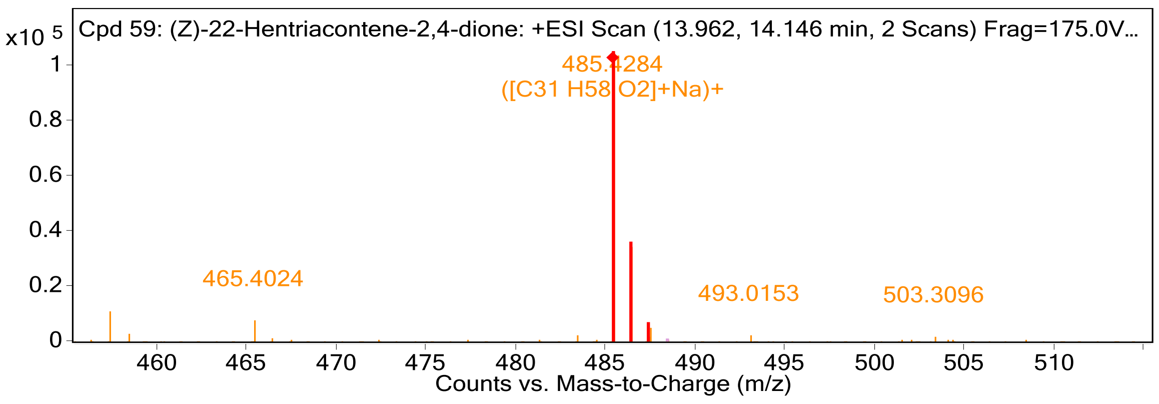

MS Spectrum Peak List

m/z	*Calc m/z*	Diff(ppm)	z	Abund	Formula	Ion
224.1273			1	16404.22		
337.2146			1	33599.65		
353.9837			1	30509.86		
355.9796			1	16707.8		
439.3866			1	49451.81		
440.3898			1	15393.32		
457.3977			1	11311.46		
485.4284	485.4329	9.25	1	104819.71	C31 H58 O2	(M+Na)+
486.4312	486.4363	10.61	1	34347.93	C31 H58 O2	(M+Na)+
487.4331	487.4396	13.24	1	5608.66	C31 H58 O2	(M+Na)+

MSMS Spectrum

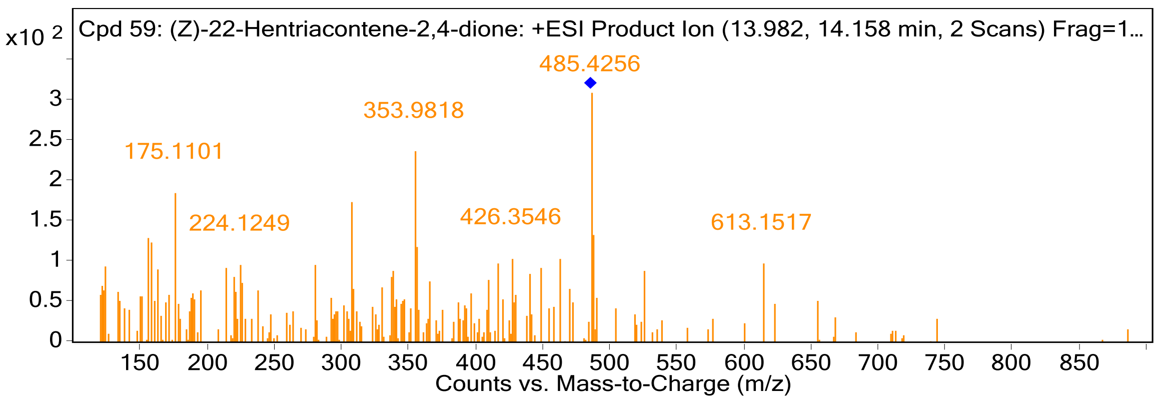

MS/MS Spectrum Peak List

m/z	z	Abund
155.0698		129.06
158.0811		123.77
175.1101		185.81
307.2596		173.77
353.9818		236.76
355.9781		117.81
426.3546	1	104.52
462.1973		104.01
485.4256	1	310.16
486.4275	1	134.16

Compound Structure

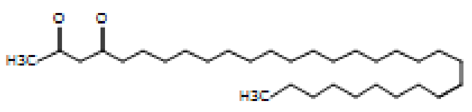

Compound Label	Name	*m/z*	RT	Algorithm	Mass
Cpd 60: Hydroflumethiazide	**Hydroflumethiazide**	353.9837	14.18	Auto MS/MS	330.9945

MS Spectrum

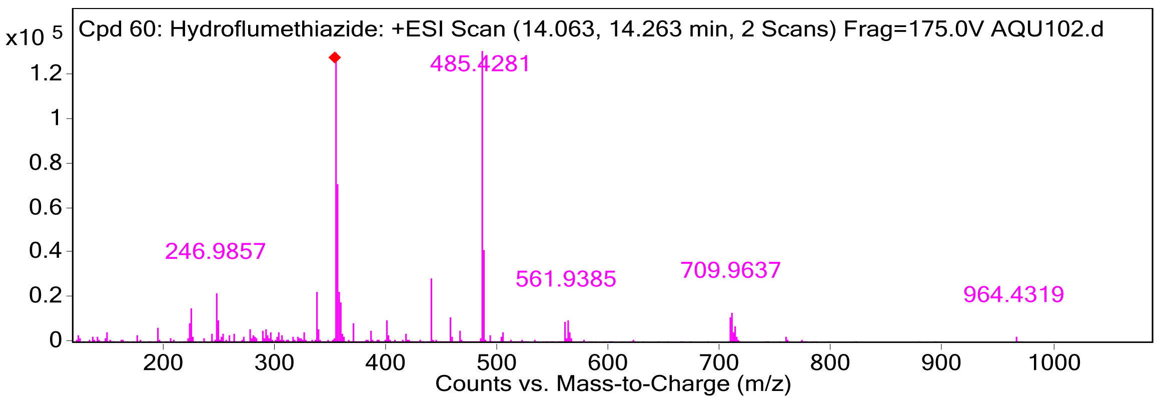

MS Zoomed Spectrum

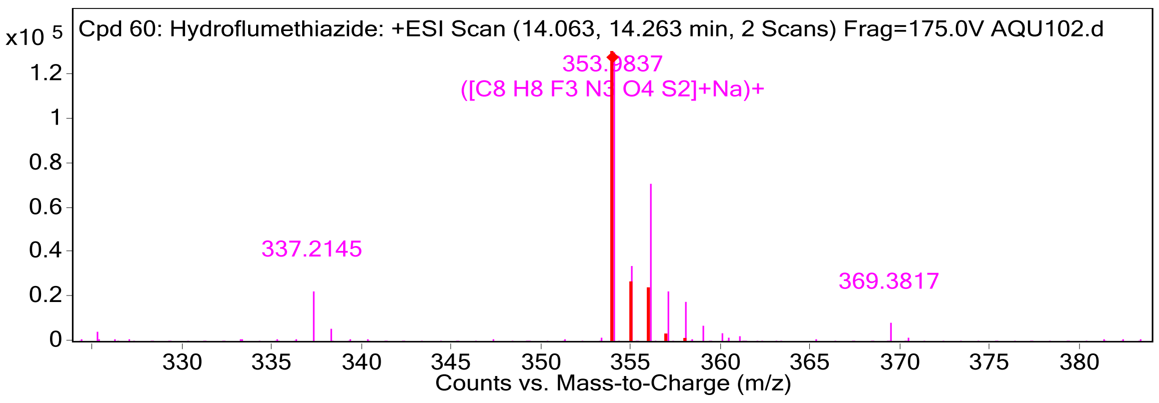

MS Spectrum Peak List

m/z	*Calc m/z*	Diff(ppm)	z	Abund	Formula	Ion

246.9857			1	22243.84		
337.2145			1	23119.12		
353.9837	353.9801	-10.29	1	130244.89	C8 H8 F3 N3 O4 S2	(M+Na)+
354.9887	354.9823	-17.9	1	34216.6	C8 H8 F3 N3 O4 S2	(M+Na)+
355.9795	355.977	-6.99	1	71052.36	C8 H8 F3 N3 O4 S2	(M+Na)+
356.9844	356.9792	-14.53	1	22584.94	C8 H8 F3 N3 O4 S2	(M+Na)+
357.9769	357.9752	-4.54	1	17916.48	C8 H8 F3 N3 O4 S2	(M+Na)+
439.3861			1	28682.55		
485.4281			1	132398.53		
486.4314			1	41626.08		

MSMS Spectrum

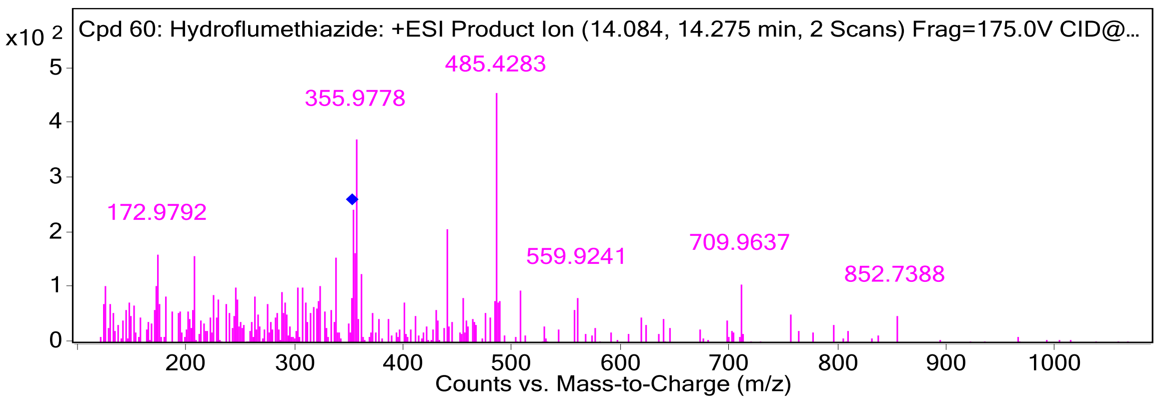

MS/MS Spectrum Peak List

m/z	z	Abund
172.9792		162.18
206.9667		158.31
337.2145		156.71
353.9792		242.3
354.983		164.58
355.9778	1	370.81
359.9722		126.54
439.3854		208.4
485.4283	1	456.38
709.9637		107.31

Compound Structure

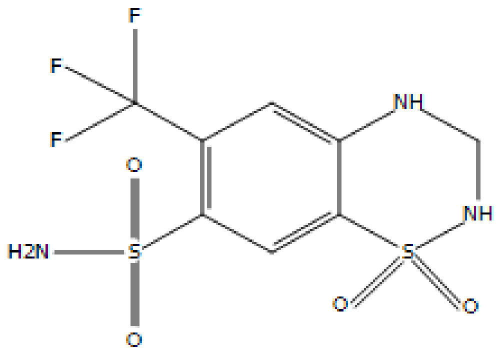

Compound Label	Name	*m/z*	RT	Algorithm	Mass
Cpd 61: Methyl 2-furoate	**Methyl 2-furoate**	149.0232	14.407	Auto MS/MS	126.034

MS Spectrum

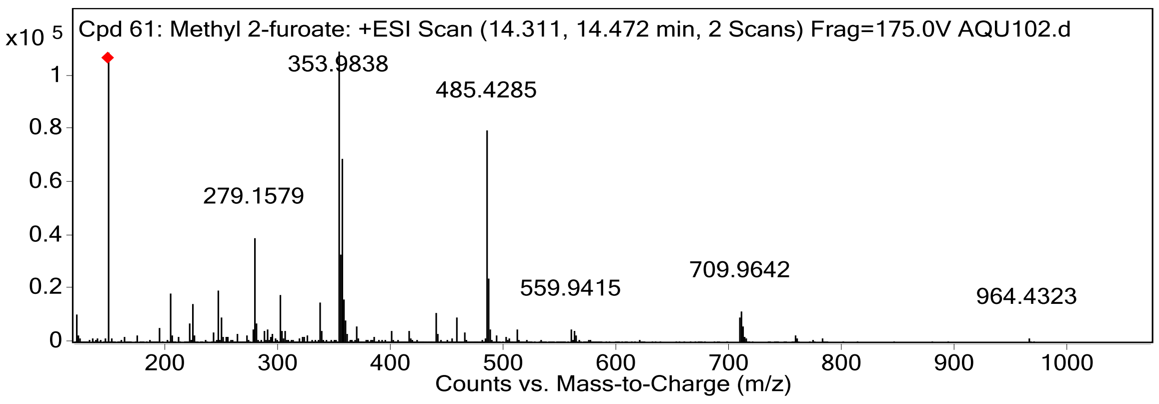

MS Zoomed Spectrum

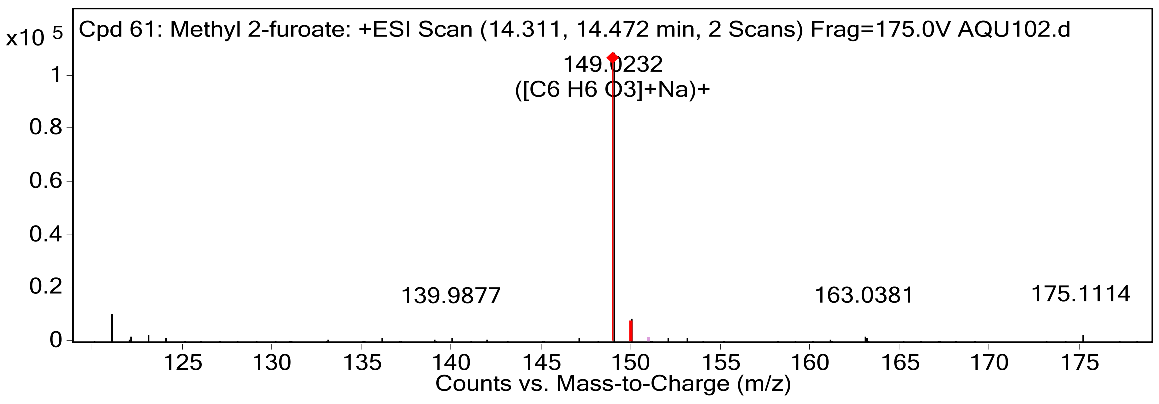

MS Spectrum Peak List

m/z	*Calc m/z*	Diff(ppm)	z	Abund	Formula	Ion
149.0232	149.0209	-15.51	1	108756.96	C6 H6 O3	(M+Na)+
150.0265	150.0243	-14.61	1	9014.11	C6 H6 O3	(M+Na)+
246.986				19883.99		
279.1579			1	39457.87		
353.9838			1	126808.2		
354.9889			1	32998.69		
355.9796			1	68949.56		
356.9838			1	20922.1		
485.4285			1	79356.68		
486.4314			1	24047.63		

MSMS Spectrum

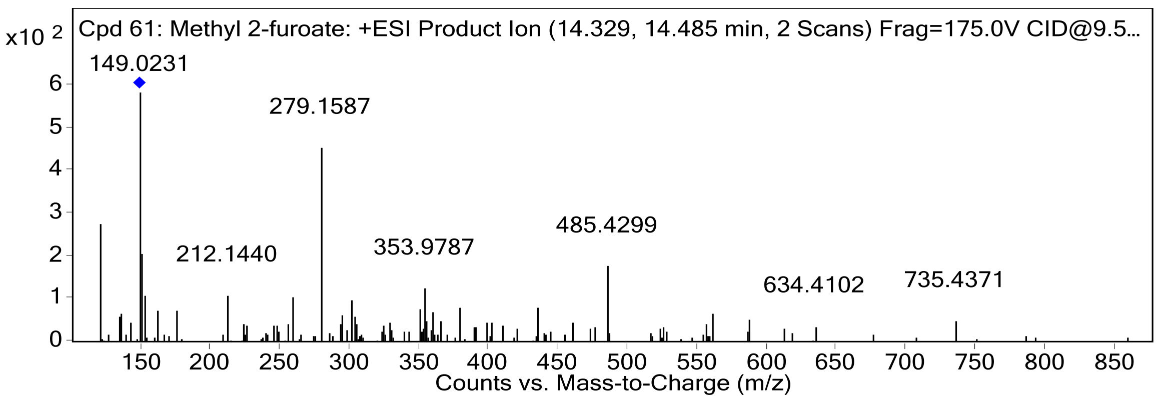

MS/MS Spectrum Peak List

m/z	z	Abund
121.0275		274.75
149.0231		584.26
150.0243	1	205.81
153.0664		108.6
212.144		108.58
259.1689		105.58
279.1587		455
301.1437		98.9
353.9787	1	124.79
485.4299		176.59

Compound Structure

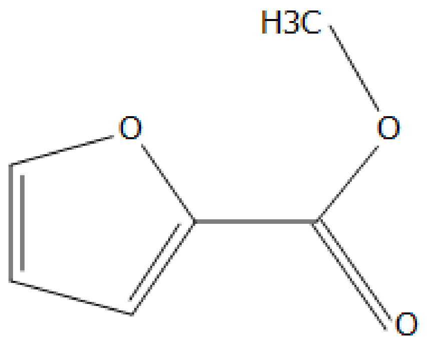

Compound Label	Name	*m/z*	RT	Algorithm	Mass
Cpd 62: (Z)-22-Hentriacontene-2,4-dione	**(Z)-22-Hentriacontene-2,4-dione**	485.4285	14.408	Auto MS/MS	462.4391

MS Spectrum

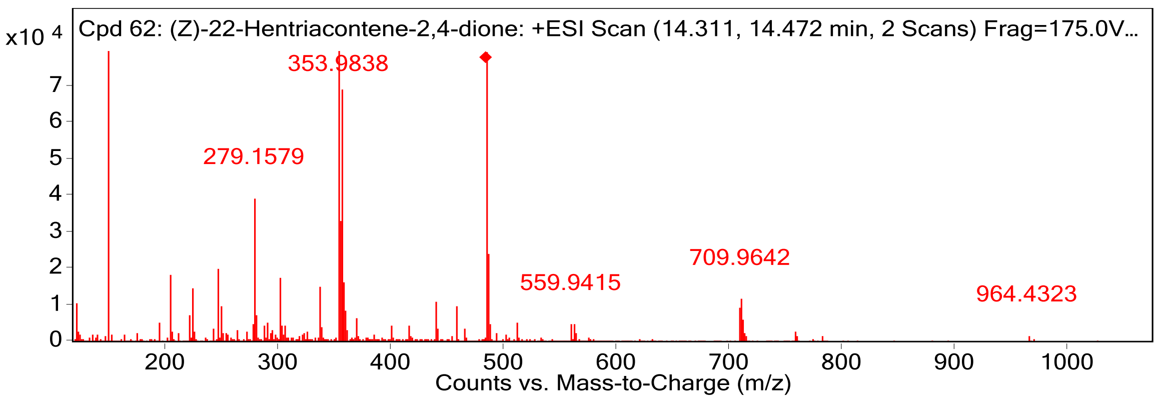

MS Zoomed Spectrum

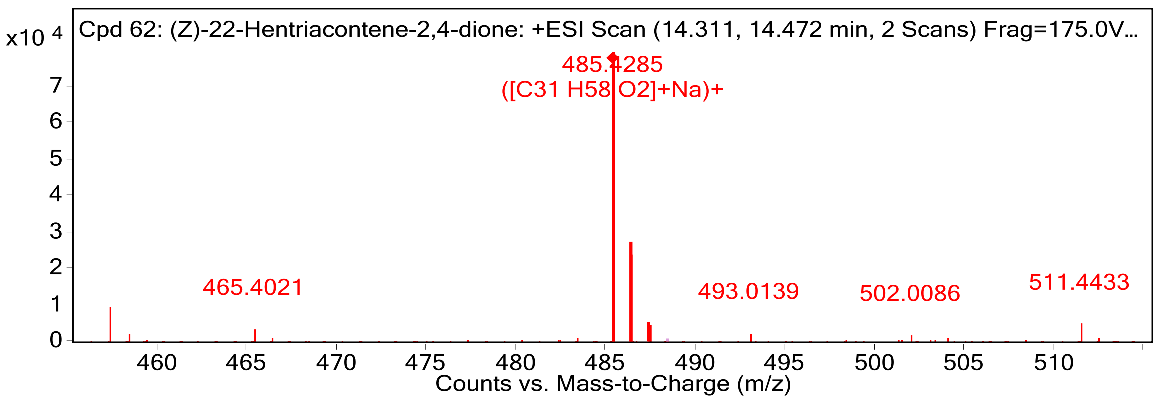

MS Spectrum Peak List

m/z	*Calc m/z*	Diff(ppm)	z	Abund	Formula	Ion
149.0232			1	108756.96		
246.986				19883.99		
279.1579			1	39457.87		
353.9838			1	126808.2		
354.9889			1	32998.69		
355.9796			1	68949.56		
356.9838			1	20922.1		
485.4285	485.4329	9.04	1	79356.68	C31 H58 O2	(M+Na)+
486.4314	486.4363	10.14	1	24047.63	C31 H58 O2	(M+Na)+
487.4341	487.4396	11.16	1	4775.04	C31 H58 O2	(M+Na)+

MSMS Spectrum

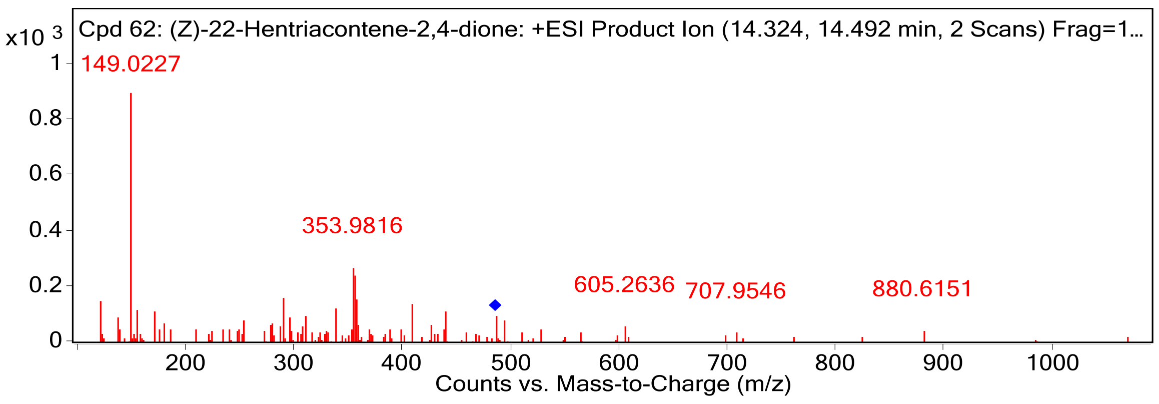

MS/MS Spectrum Peak List

m/z	z	Abund
121.0285		151.76

149.0227		898.58
153.9828		117.12
290.0082		160.84
337.2096		123
353.9816	1	271.33
355.9765	1	243.35
356.9773		154.03
408.3448		137.97
439.3857		115.04

Compound Structure

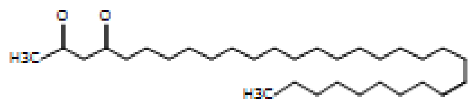

Compound Label	Name	*m/z*	RT	Algorithm	Mass
Cpd 63: Hydroflumethiazide	**Hydroflumethiazide**	353.9837	14.533	Auto MS/MS	330.9945

MS Spectrum

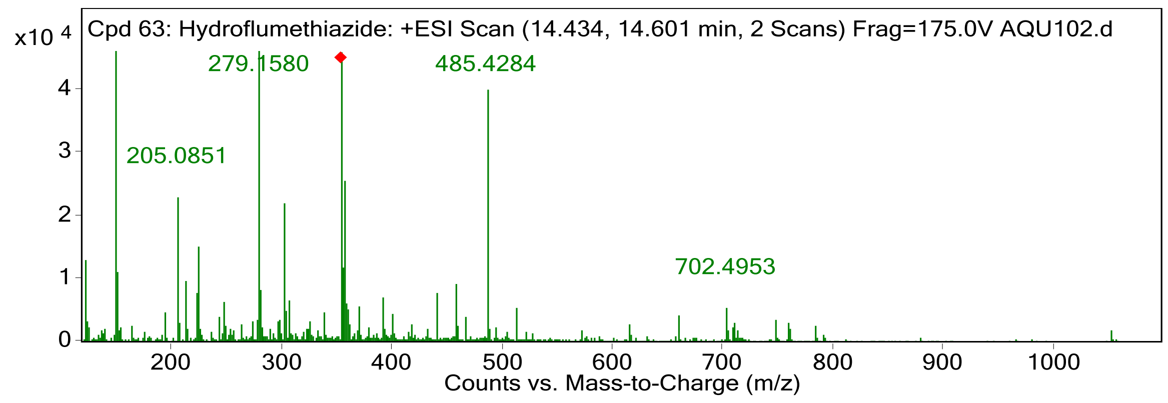

MS Zoomed Spectrum

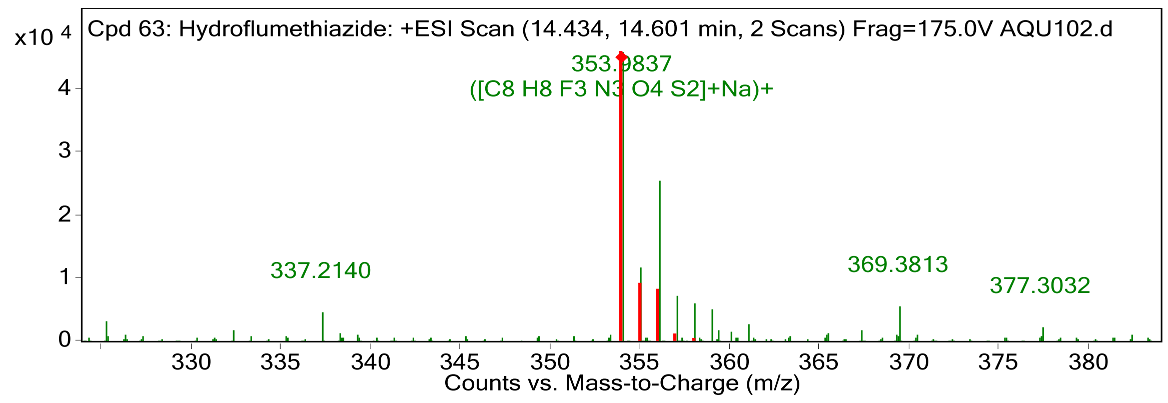

MS Spectrum Peak List

m/z	*Calc m/z*	Diff(ppm)	z	Abund	Formula	Ion
149.0232			1	134461.5		
205.0851			1	23046.25		
279.158			1	46662.95		
301.1399			1	22058.96		
353.9837	353.9801	-10.42	1	45998.08	C8 H8 F3 N3 O4 S2	(M+Na)+
354.9889	354.9823	-18.71	1	11769.71	C8 H8 F3 N3 O4 S2	(M+Na)+
355.9795	355.977	-6.88	1	25533.81	C8 H8 F3 N3 O4 S2	(M+Na)+
356.9839	356.9792	-13.09	1	7356.03	C8 H8 F3 N3 O4 S2	(M+Na)+
357.9771	357.9752	-5.23	1	6145.47	C8 H8 F3 N3 O4 S2	(M+Na)+
485.4284			1	40036.63		

MSMS Spectrum

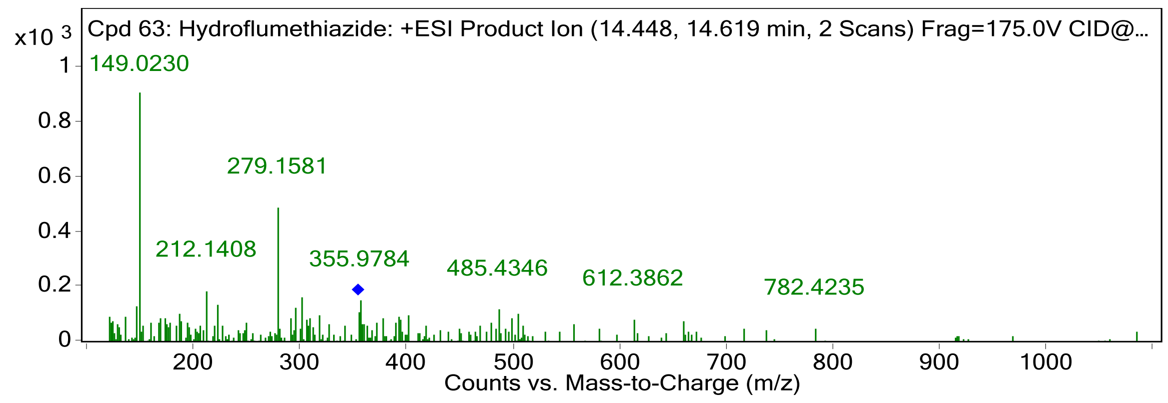

MS/MS Spectrum Peak List

m/z	z	Abund
145.9547		128.04
149.023	1	911
212.1408		186.83
222.1146		137.7
279.1581	1	490.99
295.1675		124.35
301.1396		163.72
353.9821	1	106.5
355.9784		151.42
485.4346		117.59

Compound Structure

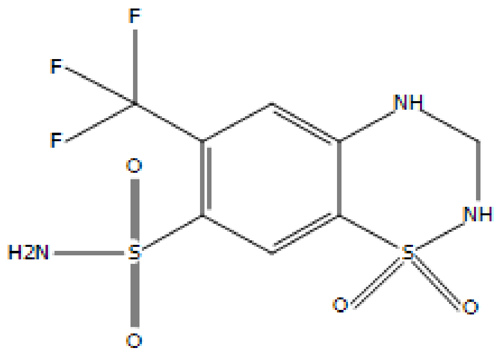

Compound Label	m/z	RT	Algorithm
Compound 64	526.392	14.819	Auto MS/MS

MS Spectrum

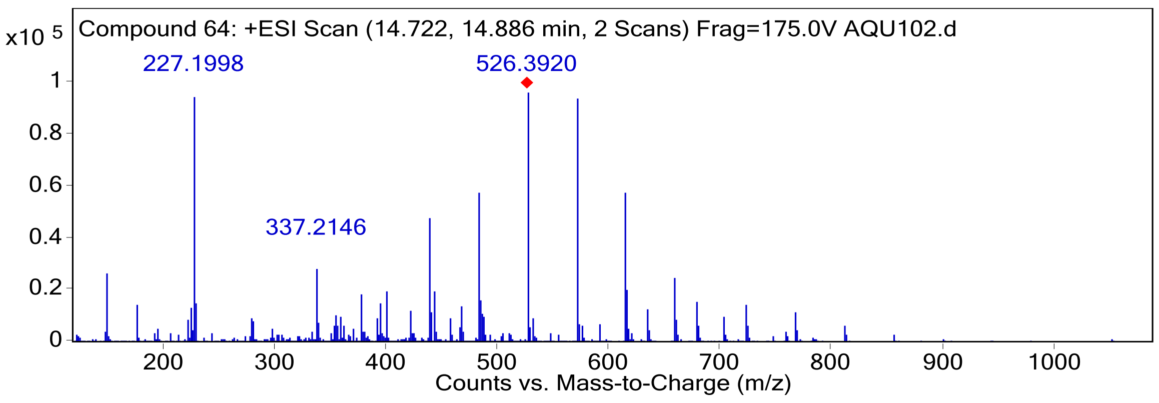

MS Zoomed Spectrum

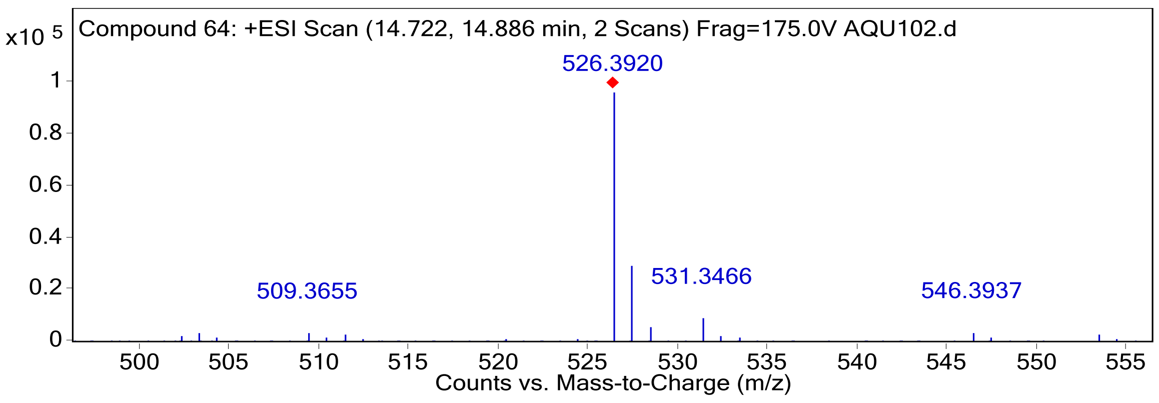

MS Spectrum Peak List

m/z	z	Abund
227.1998	1	94399.54
337.2146	1	28251.37
438.3396	1	48014.5
482.3658	1	57582.17
526.392	1	96269.88
527.395	1	29124.9
528.3976	1	5506.35
570.4179	1	93614.58
571.4213	1	30239.22
614.4441	1	57743.71

MSMS Spectrum

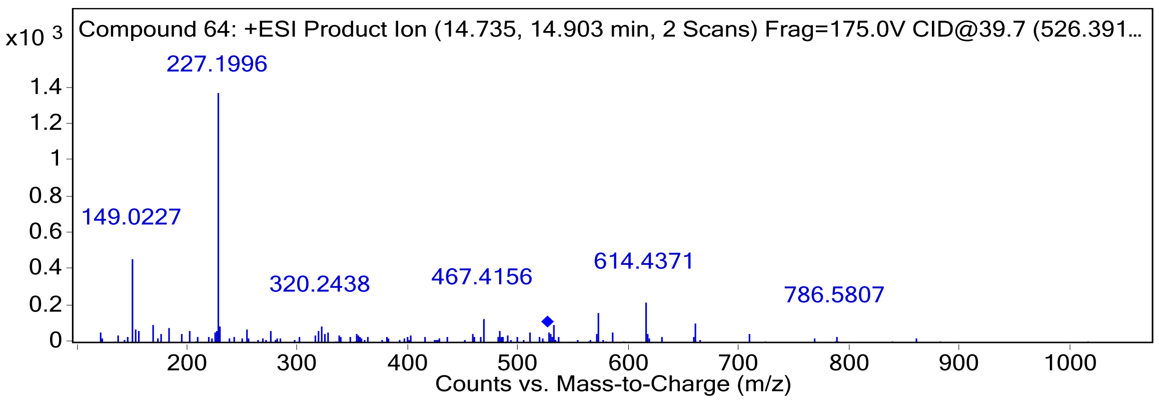

MS/MS Spectrum Peak List

m/z	z	Abund
149.0227		463.65
167.996		101.39
227.1996	1	1375.83
228.2027	1	89.57
320.2438	1	93.16
467.4156		133.57
531.3494		101.5
571.4231		160.53
614.4371		219.95
658.4679		103.2

Compound Label	Name	m/z	RT	Algorithm	Mass
Cpd 65: Ascorbyl stearate	**Ascorbyl stearate**	443.2947	14.902	Auto MS/MS	442.2873

MS Spectrum

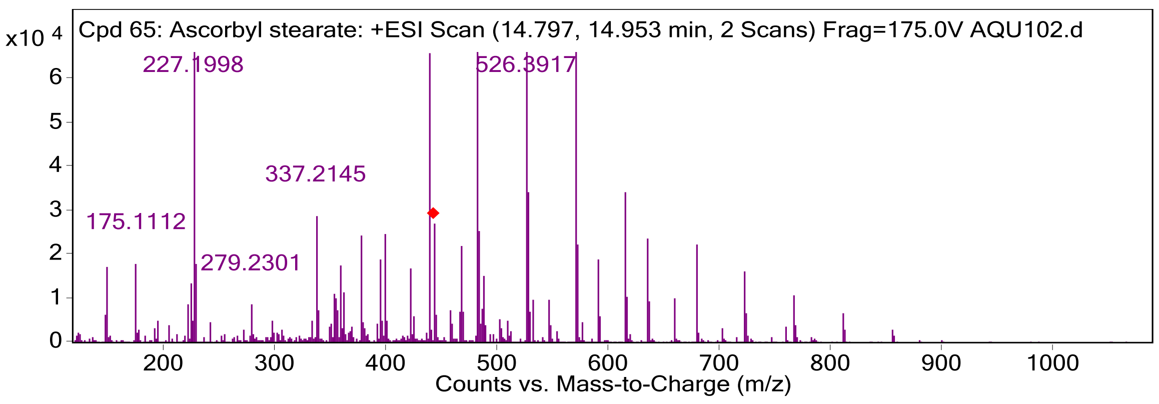

MS Zoomed Spectrum

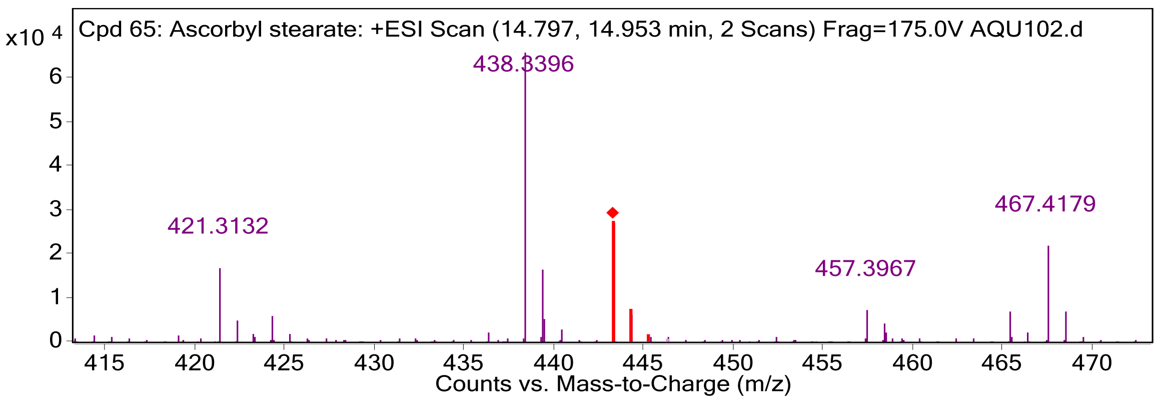

MS Spectrum Peak List

m/z	*Calc m/z*	Diff(ppm)	z	Abund	Formula	Ion
227.1998			1	128494.13		
438.3396			1	65848.92		
443.2947	443.3003	12.69	1	27076.3	C24 H42 O7	(M+H)+
444.2976	444.3037	13.91	1	6382.73	C24 H42 O7	(M+H)+
445.3012	445.3064	11.57	1	1304.81	C24 H42 O7	(M+H)+
482.3655			1	90821.28		
526.3917			1	116433.05		
527.3945			1	34273.56		
570.4173			1	73530.1		
614.4435			1	34117.73		

MSMS Spectrum

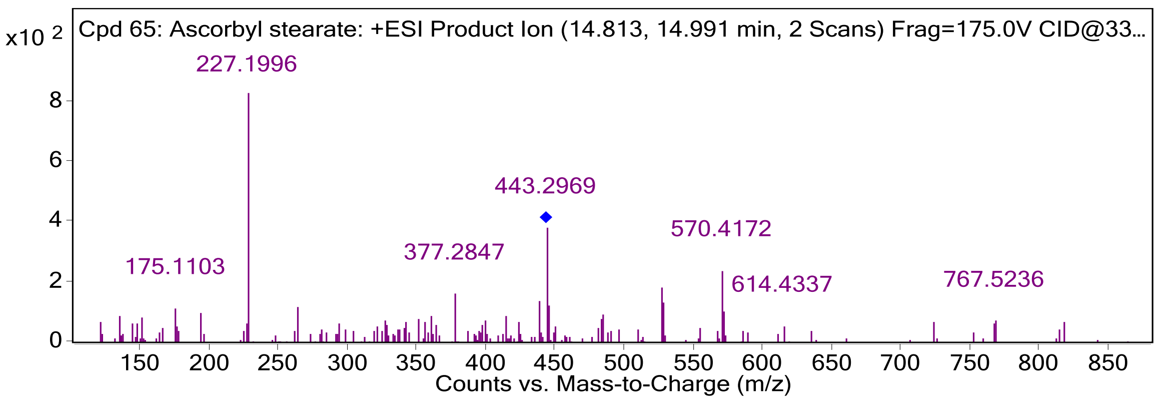

MS/MS Spectrum Peak List

m/z	z	Abund
175.1103		114.54
227.1996	1	830.08
263.144		121.11
377.2847		163
438.3389	1	139.69

m/z	z	Abund
443.2969	1	383.13
444.304	1	123.7
526.3926		183.99
527.3942		135.59
570.4172	1	240.65

Compound Structure

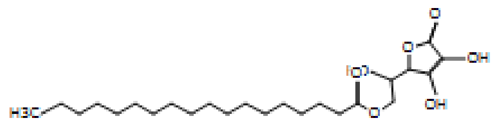

Compound Label	*m/z*	RT	Algorithm
Compound 66	467.4177	14.998	Auto MS/MS

MS Spectrum

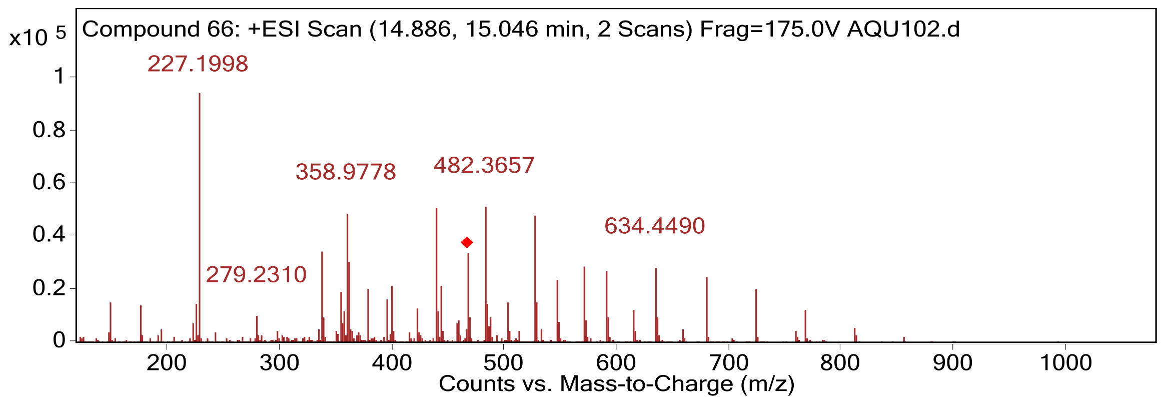

MS Zoomed Spectrum

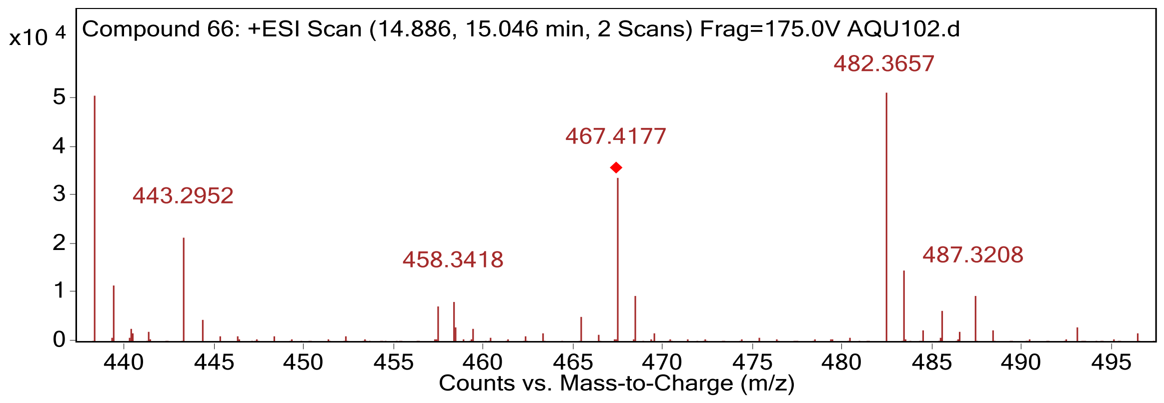

MS Spectrum Peak List

m/z	z	Abund
227.1998	1	94570.09
337.2145	1	34506.55
358.9778	1	48920.44
438.3396	1	50642.03
467.4177	1	33901.63
468.4209	1	9625.67
469.4243	1	1879.65
470.4239	1	421.91
482.3657	1	51440.09
526.3919	1	48250.07

MSMS Spectrum

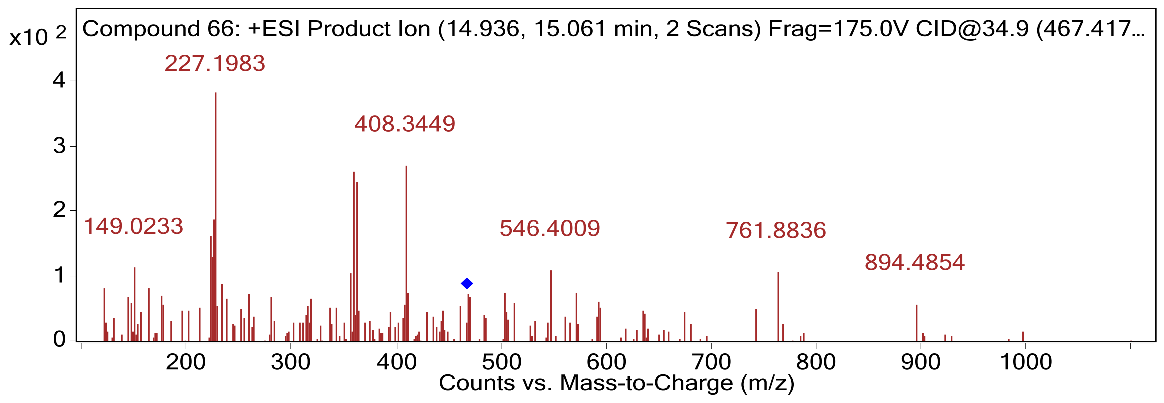

MS/MS Spectrum Peak List

m/z	z	Abund
149.0233		114.19
222.1123	1	164.34
224.1269		130.26
225.1289		188.1
227.1983		385.15
358.9753		261.61
360.9747		245.79
408.3449	1	272.35
546.4009		110.88
761.8836		107.86

Compound Label	Name	*m/z*	RT	Algorithm	Mass
Cpd 67: Chlorfenvinphos	**Chlorfenvinphos**	358.978	15.061	Auto MS/MS	357.9707

MS Spectrum

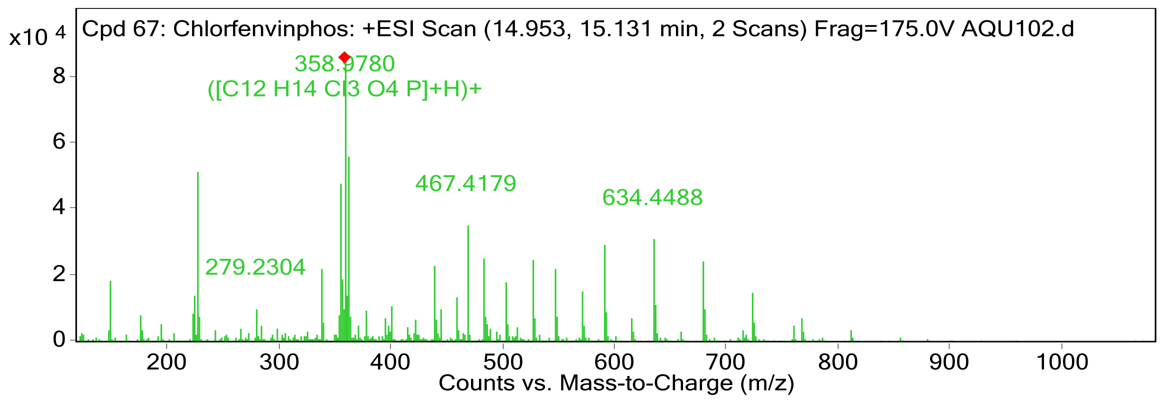

MS Zoomed Spectrum

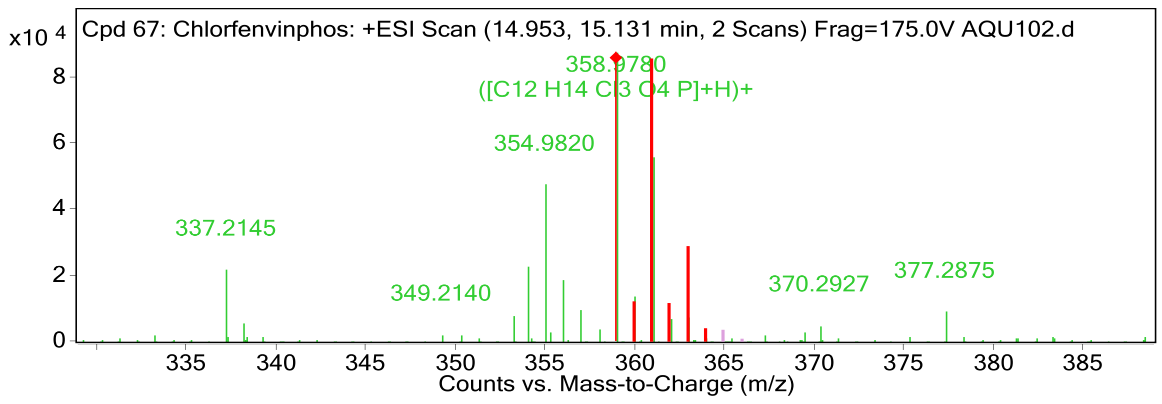

MS Spectrum Peak List

m/z	*Calc m/z*	Diff(ppm)	z	Abund	Formula	Ion
227.1997			1	51397.29		
354.982			1	47755.25		
358.978	358.9768	-3.34	1	87531.38	C12 H14 Cl3 O4 P	(M+H)+
359.9801	359.9802	0.23	1	13973.92	C12 H14 Cl3 O4 P	(M+H)+
360.9756	360.974	-4.43	1	55961.26	C12 H14 Cl3 O4 P	(M+H)+
361.9784	361.9773	-2.87	1	7190.1	C12 H14 Cl3 O4 P	(M+H)+
362.972	362.9713	-1.76	1	7752.8	C12 H14 Cl3 O4 P	(M+H)+
363.9751	363.9746	-1.44	1	1384.26	C12 H14 Cl3 O4 P	(M+H)+
467.4179			1	35356.21		
634.4488			1	31121.18		

MSMS Spectrum

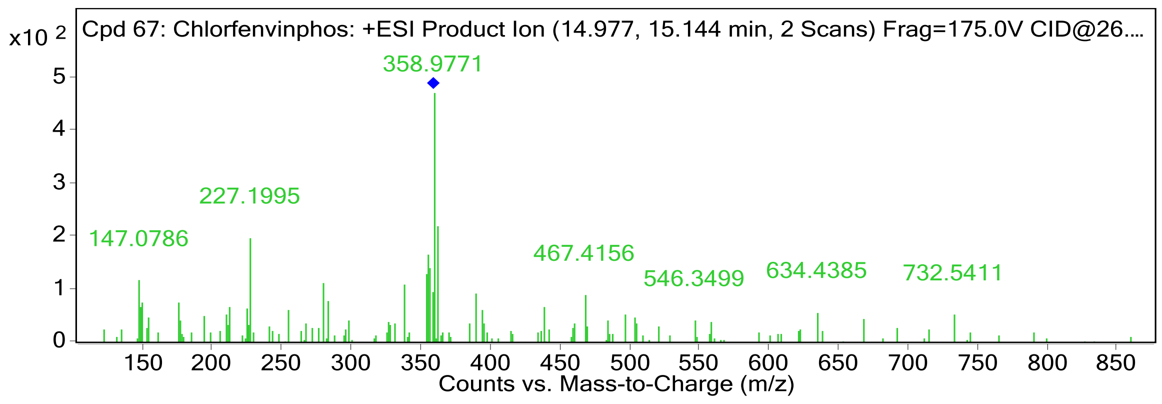

MS/MS Spectrum Peak List

m/z	Abund
147.0786	118.02
227.1995	198.76
279.2296	112.72
337.2121	110.96
353.9823	130.51
354.9801	167.39
355.9841	141.06
358.366	96.88
358.9771	472.38
360.9752	220.57

Compound Structure

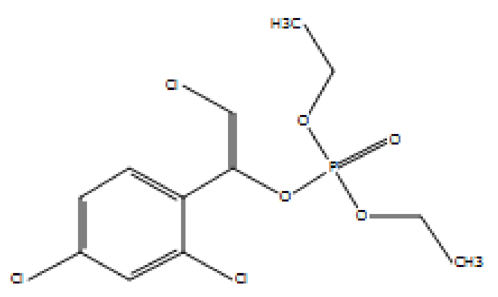

Compound Label	Name	*m/z*	RT	Algorithm	Mass
Cpd 68: Idoxuridine	**Idoxuridine**	354.9818	15.216	Auto MS/MS	353.9727

MS Spectrum

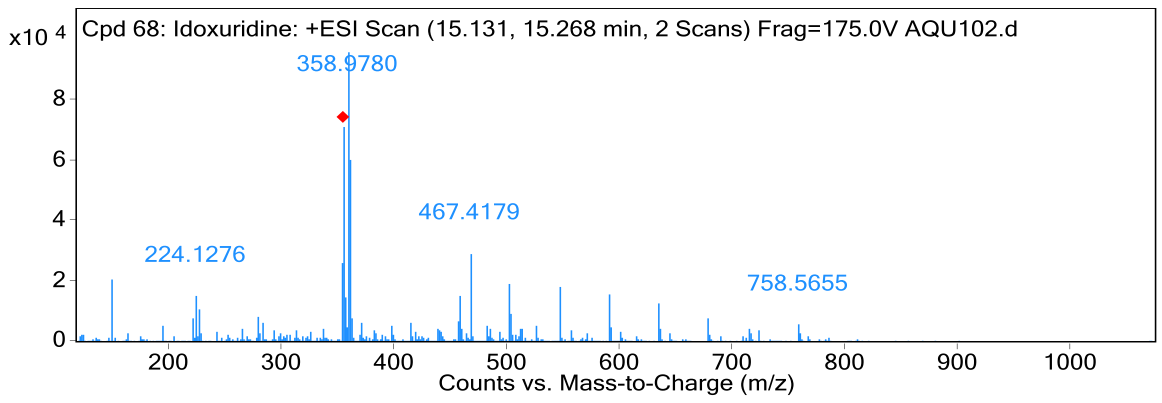

MS Zoomed Spectrum

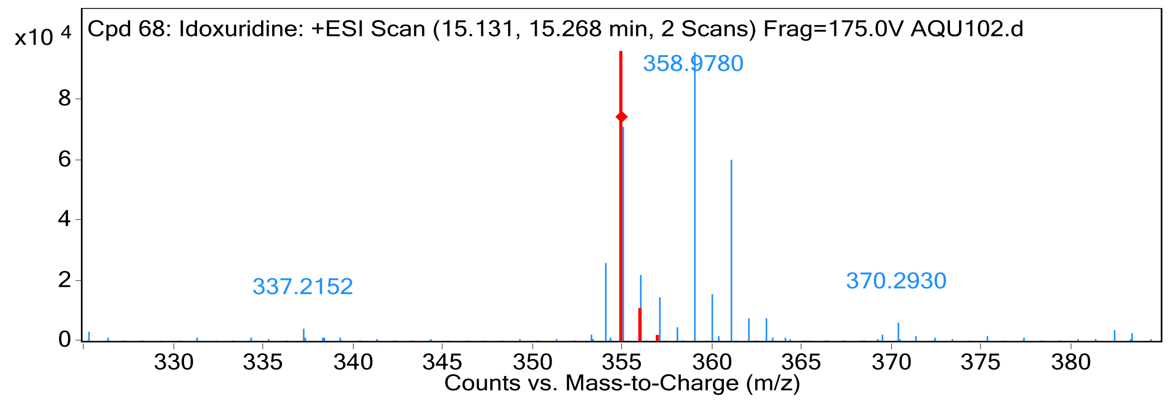

MS Spectrum Peak List

m/z	*Calc m/z*	Diff(ppm)	z	Abund	Formula	Ion
149.0232				20973.68		
353.9835				26026.38		
354.9818	354.9785	-9.18	1	71219.59	C9 H11 I N2 O5	(M+H)+
355.9811	355.9815	1.27	1	22299.3	C9 H11 I N2 O5	(M+H)+
356.9787	356.9834	13.09	1	14651.15	C9 H11 I N2 O5	(M+H)+
358.978			1	96065.89		
360.9757			1	60439.48		
467.4179			1	29456.79		
502.3704			1	19432.11		
546.3967			1	18093.55		

MSMS Spectrum

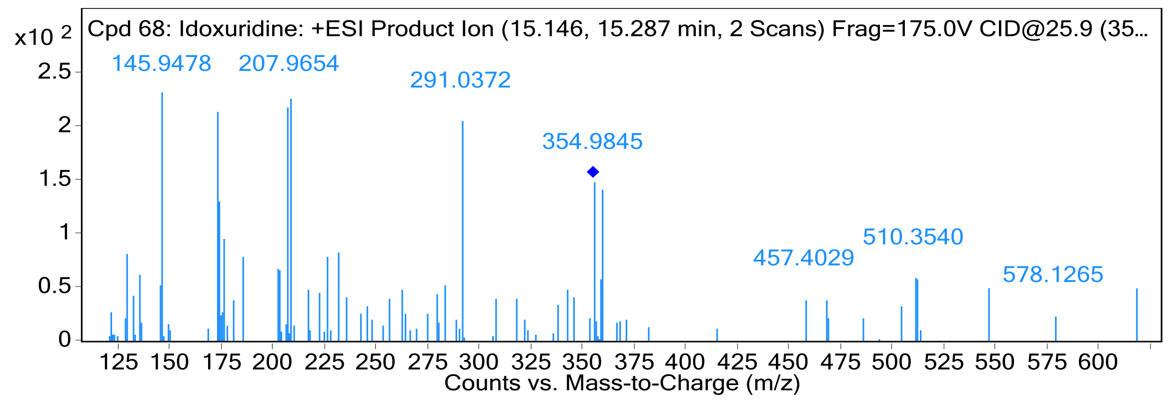

MS/MS Spectrum Peak List

m/z	z	Abund
145.9478		232.22
172.9793		214.06
173.9784	1	131.07
175.9927		95.9
206.9677		218.38
207.9654		226.15
231.0359		83.52
291.0372		205.07
354.9845	1	147.89
358.9803		141.67

Compound Structure

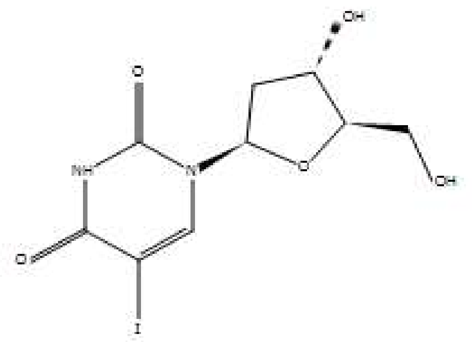

**Compound Label**	***m/z***	**RT**	**Algorithm**
Compound 69	467.418	15.299	Auto MS/MS

MS Spectrum

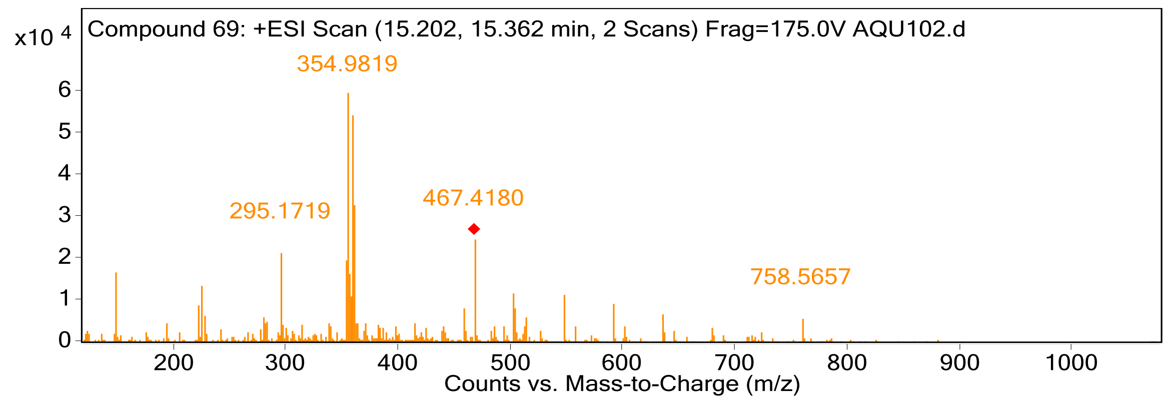

MS Zoomed Spectrum

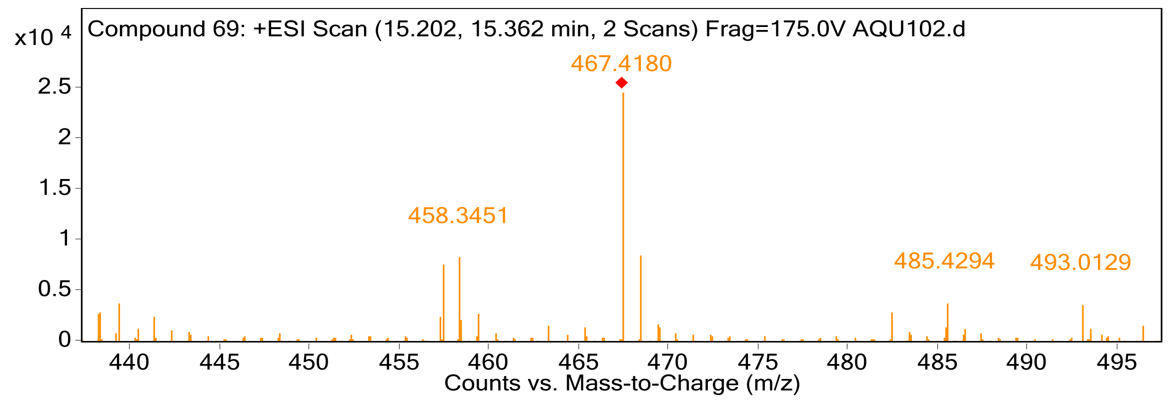

MS Spectrum Peak List

m/z	z	Abund
149.023		16794.09
295.1719	1	21473.59

353.9835		19418.87
354.9819	1	59559.09
358.978	1	54148.39
360.9759	1	32826.76
467.418	1	24586.02
468.4209	1	8497.8
469.4204	1	1490.85
470.4245	1	273.05

MSMS Spectrum

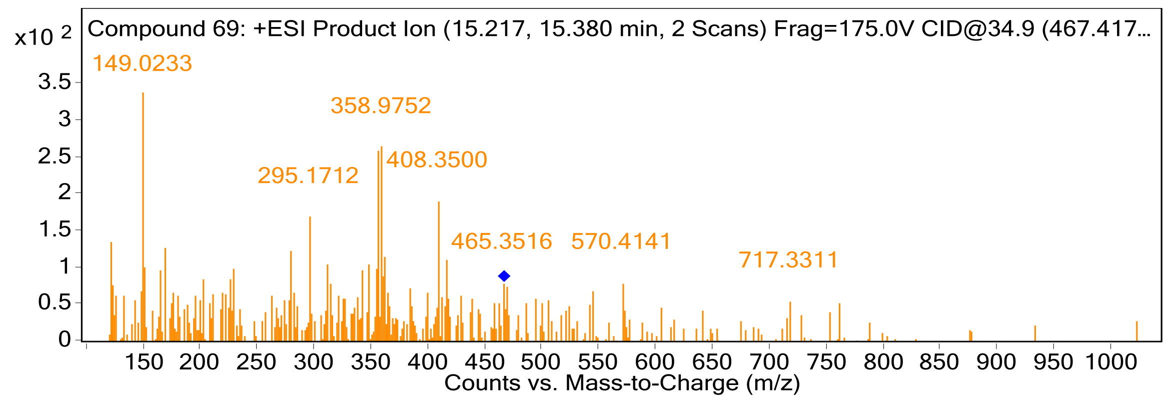

MS/MS Spectrum Peak List

m/z	z	Abund
121.0274		135.34
149.0233		339.45
168.0899		128.09
279.2292		123.22
295.1712	1	169.7
354.9804	1	260.48
358.9752	1	264.95
360.9718	1	115.54
408.35	1	191.29
415.3128		112.28

Compound Label	Name	*m/z*	RT	Algorithm	Mass
Cpd 70: Chlorfenvinphos	**Chlorfenvinphos**	358.9778	15.367	Auto MS/MS	357.9706

MS Spectrum

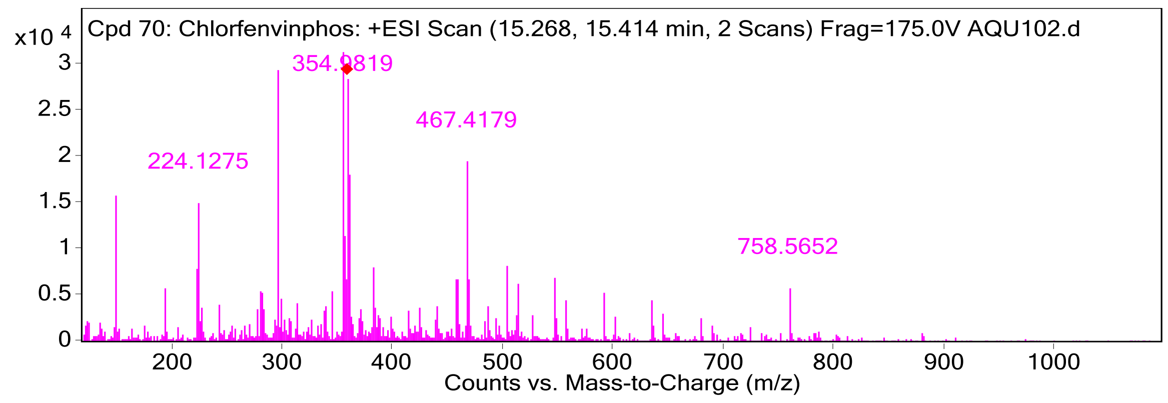

MS Zoomed Spectrum

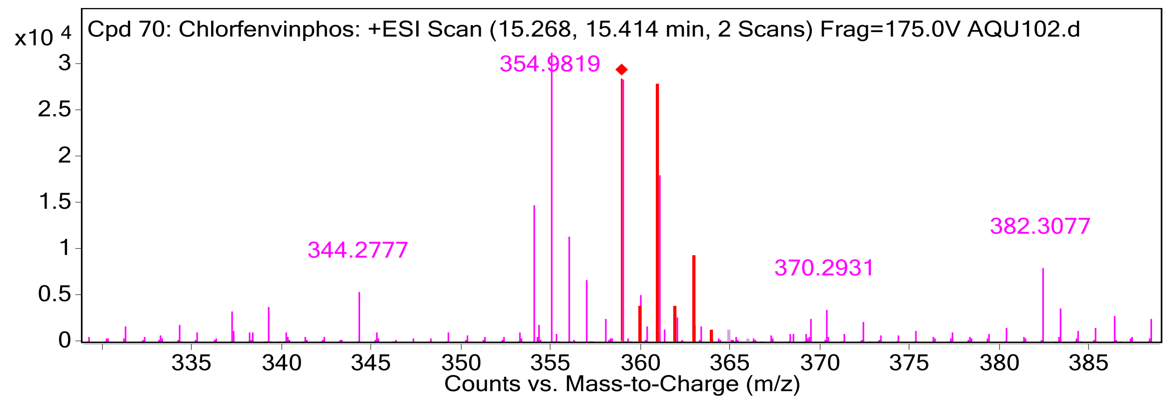

MS Spectrum Peak List

m/z	*Calc m/z*	Diff(ppm)	z	Abund	Formula	Ion
149.0232				15921.35		
295.171			1	29386.93		
354.9819			1	31373.54		
358.9778	358.9768	-2.7	1	28521.46	C12 H14 Cl3 O4 P	(M+H)+
359.9797	359.9802	1.53	1	5231.07	C12 H14 Cl3 O4 P	(M+H)+
360.9757	360.974	-4.79	1	18091.21	C12 H14 Cl3 O4 P	(M+H)+
361.9776	361.9773	-0.62	1	2825.26	C12 H14 Cl3 O4 P	(M+H)+
362.9724	362.9713	-2.9	1	1882.26	C12 H14 Cl3 O4 P	(M+H)+
363.9775	363.9746	-8.05	1	302.76	C12 H14 Cl3 O4 P	(M+H)+
467.4179			1	19581.04		

MSMS Spectrum

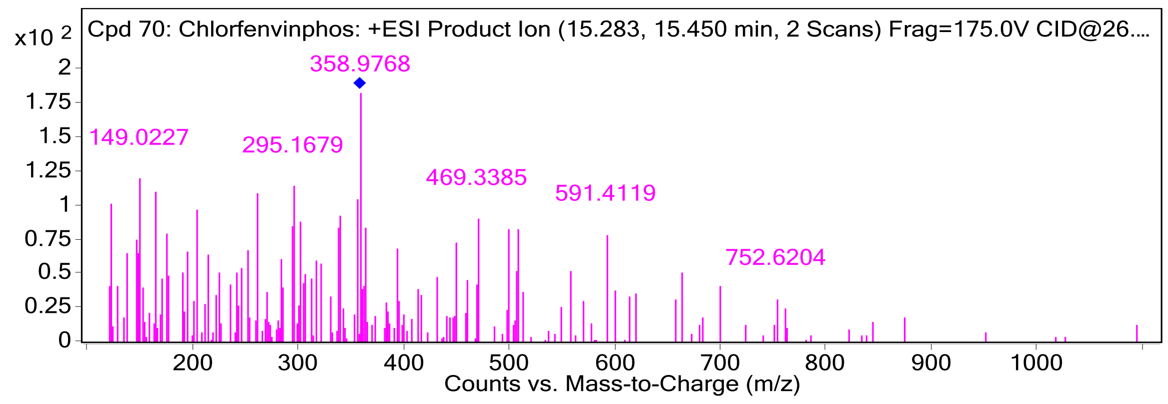

MS/MS Spectrum Peak List

m/z	z	Abund
123.0797		101.64
149.0227		120.21
165.052		110.39
203.1088		97.38
260.1623		109.1

295.1679		114.61
339.3058		93.29
354.9827		104.69
358.9768	1	183.48
469.3385		90.73

Compound Structure

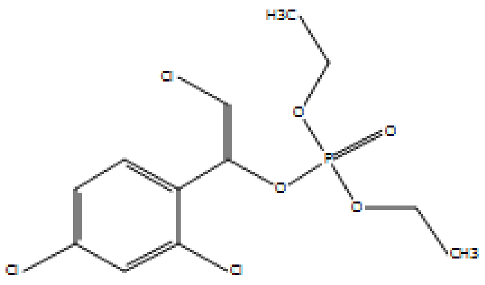

Compound Label	Name	*m/z*	RT	Algorithm	Mass
Cpd 71: (E,E)-1,6-bis(4-methoxyphenyl)-1,5-hexadiene	**(E,E)-1,6-bis(4-methoxyphenyl)-1,5-hexadiene**	295.1715	15.418	Auto MS/MS	294.1638

MS Spectrum

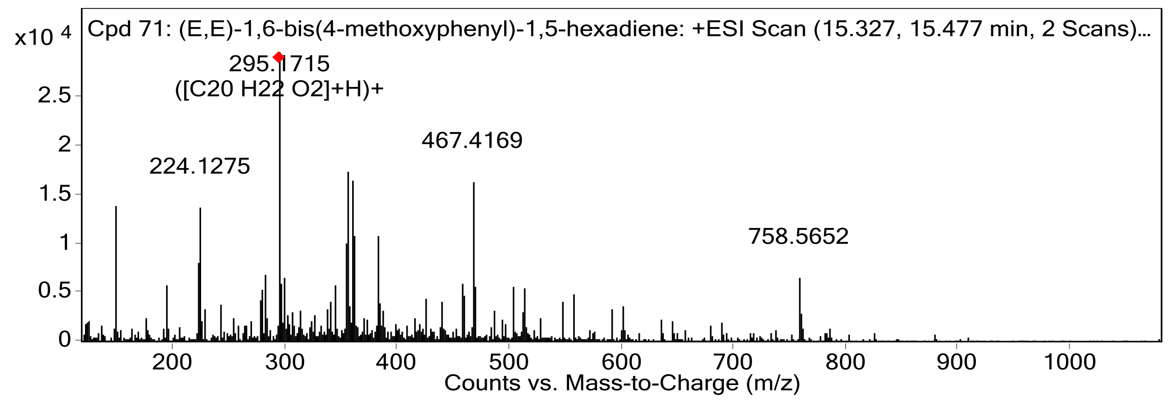

MS Zoomed Spectrum

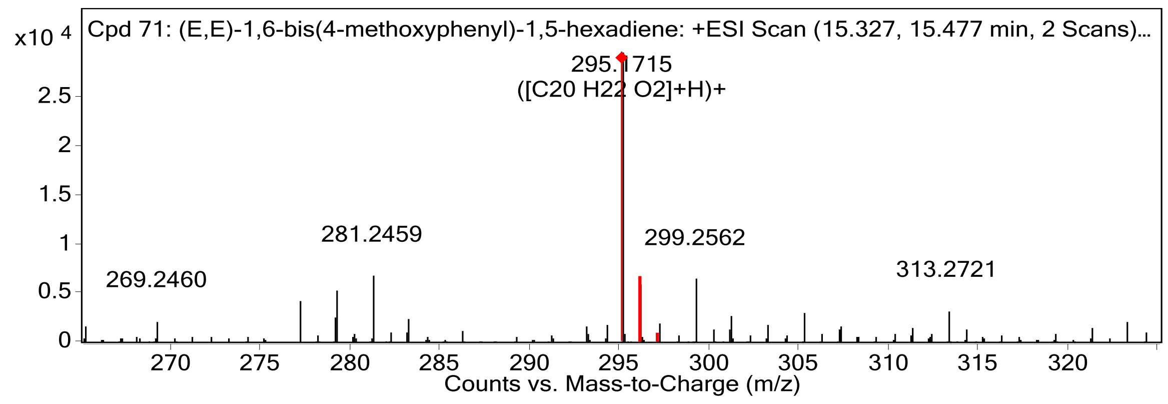

MS Spectrum Peak List

m/z	*Calc m/z*	Diff(ppm)	z	Abund	Formula	Ion
149.0232				13949.59		
224.1275			1	13754.19		
295.1715	295.1693	-7.72	1	29637.56	C20 H22 O2	(M+H)+
296.1743	296.1726	-5.65	1	5928.81	C20 H22 O2	(M+H)+
297.1706	297.1757	17.19	1	2001.83	C20 H22 O2	(M+H)+
354.9826			1	17405.95		
358.9776			1	16545.65		
360.9757			1	10913.54		
382.3081			1	10804.62		
467.4169			1	16395.25		

MSMS Spectrum

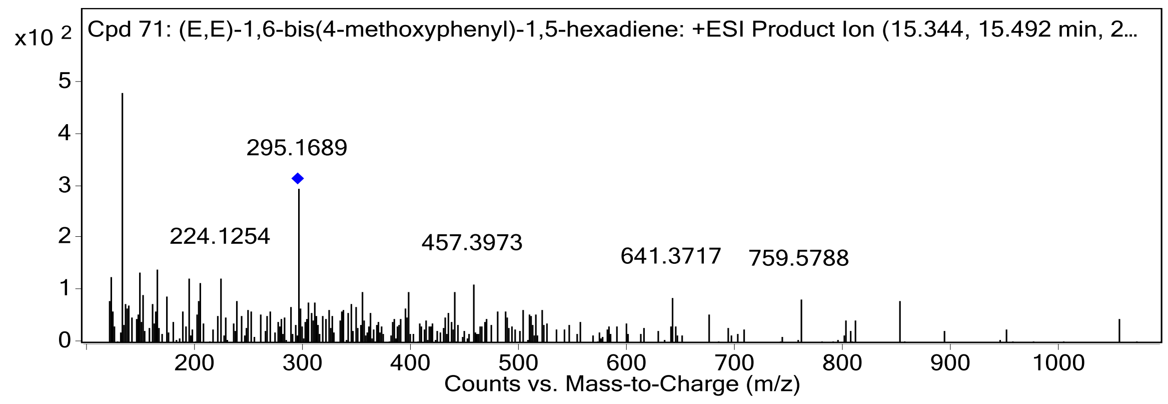

MS/MS Spectrum Peak List

m/z	Abund
123.0797	127.72
133.0662	481.37
149.0228	135.84
165.0348	140.31
194.1458	124.47
205.0881	113.79
224.1254	124.89
295.1689	295.67
397.2839	98.24
457.3973	112.58

Compound Structure

Qualitative Compound Report

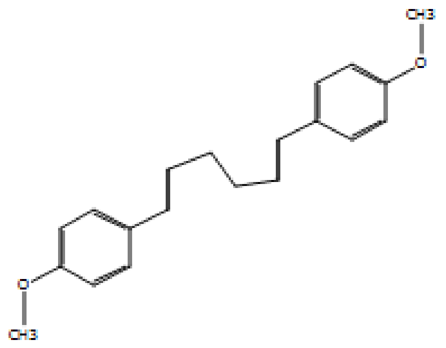

Compound Label	Name	*m/z*	RT	Algorithm	Mass
Cpd 72: Undecylprodigiosin	**Undecylprodigiosin**	394.283	15.662	Auto MS/MS	393.2757

MS Spectrum

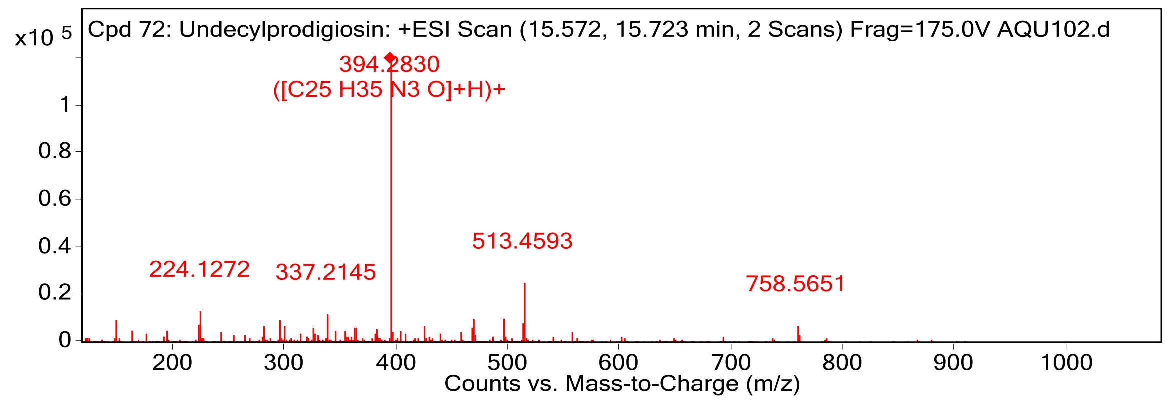

MS Zoomed Spectrum

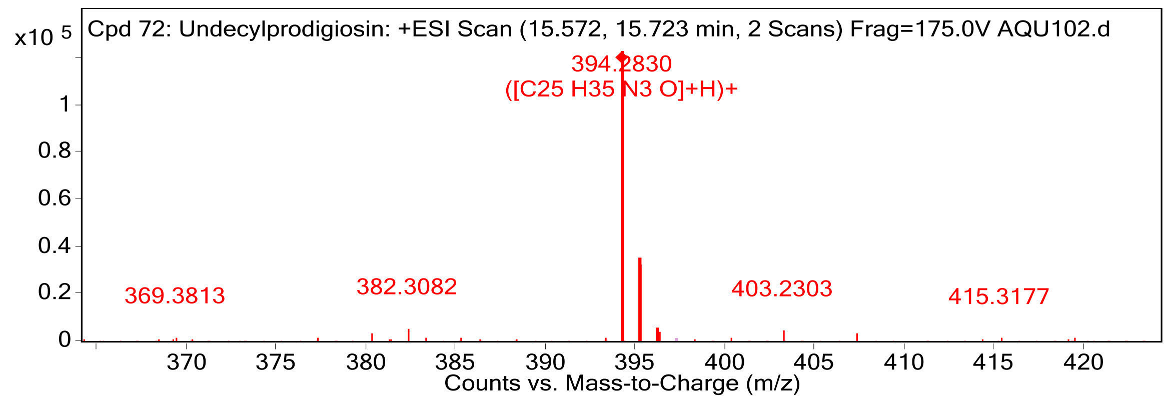

MS Spectrum Peak List

m/z	*Calc m/z*	Diff(ppm)	z	Abund	Formula	Ion
149.023				9237.79		
224.1272			1	13357.2		
295.1723			1	9170.63		
337.2145			1	12102.17		
394.283	394.2853	5.87	1	122506.06	C25 H35 N3 O	(M+H)+

m/z	*Calc m/z*	Diff(ppm)	z	Abund	Formula	Ion
395.286	395.2884	6.12	1	32590.92	C25 H35 N3 O	(M+H)+
396.2893	396.2915	5.43	1	4389.73	C25 H35 N3 O	(M+H)+
468.3873			1	9843.04		
496.3363			1	9790.44		
513.4593			1	25301.39		

MSMS Spectrum

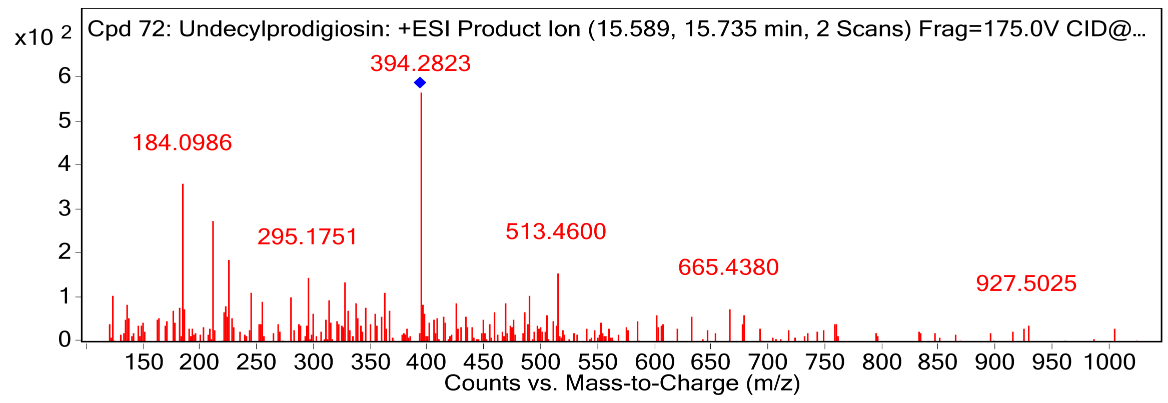

MS/MS Spectrum Peak List

m/z	z	Abund
122.0959		106.17
184.0986	1	359.22
211.0853		274.21
224.1261		186.92
244.214		113.59
295.1751		144.67
326.2673		134.33
361.2338		110.57
394.2823	1	567.8
513.46		156.59

Compound Structure

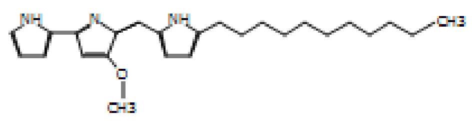

Compound Label	Name	*m/z*	RT	Algorithm	Mass
Cpd 73: Undecylprodigiosin	**Undecylprodigiosin**	394.2828	15.909	Auto MS/MS	393.2758

MS Spectrum

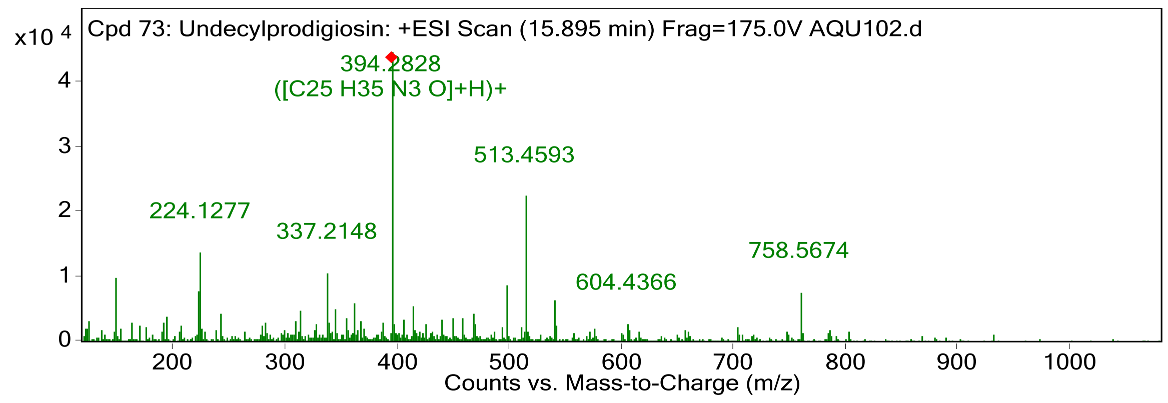

MS Zoomed Spectrum

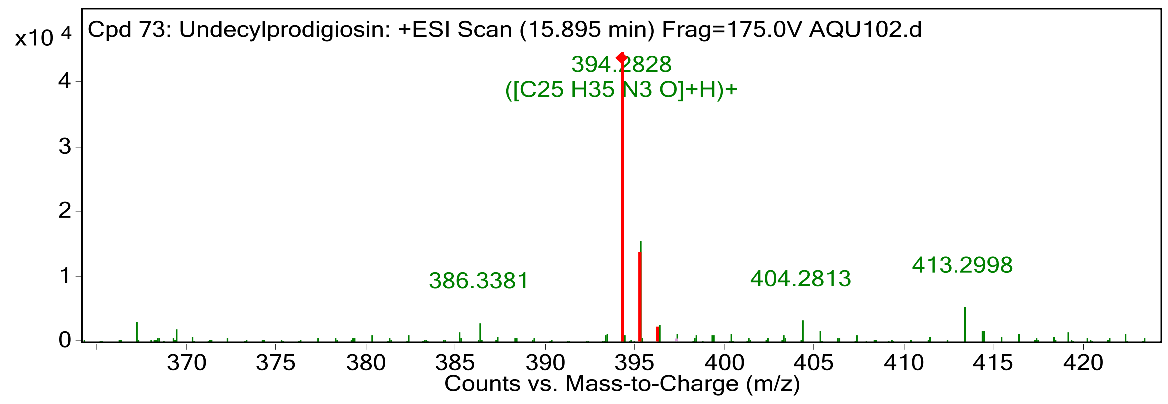

MS Spectrum Peak List

m/z	*Calc m/z*	Diff(ppm)	z	Abund	Formula	Ion
149.0233				9862.14		
222.1121				7751.32		
224.1277			1	13837.88		
337.2148			1	10664.63		
394.2828	394.2853	6.19	1	44695.83	C25 H35 N3 O	(M+H)+
395.2867	395.2884	4.51	1	15574.61	C25 H35 N3 O	(M+H)+
396.2899	396.2915	3.97	1	2810.24	C25 H35 N3 O	(M+H)+
496.336			1	8826.46		
513.4593			1	22497.22		
514.4621			1	7719.76		

MSMS Spectrum

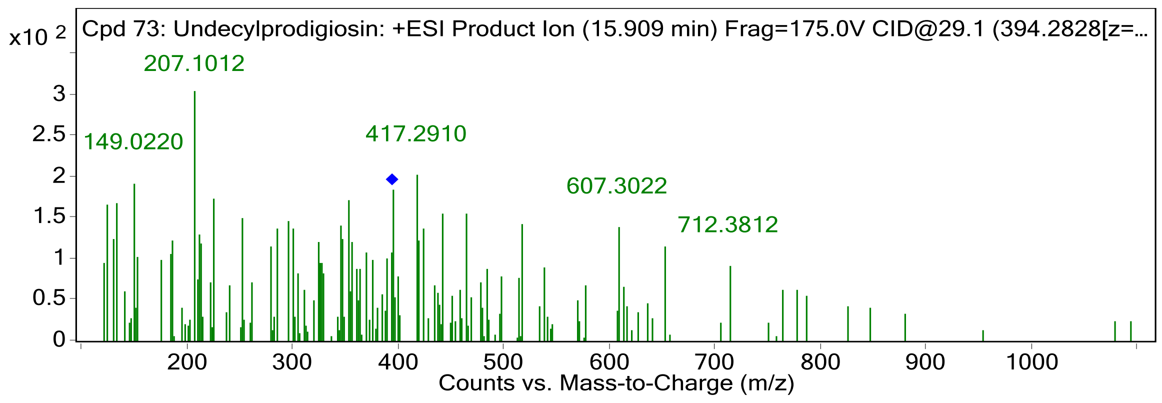

MS/MS Spectrum Peak List

m/z	Abund
123.1147	166.63
133.0968	168.55
149.022	193.45
207.1012	304.57
225.1926	175.01
352.281	171.94
394.2765	185.01
417.291	203.18
441.3333	155.76
464.2472	156.13

Compound Structure

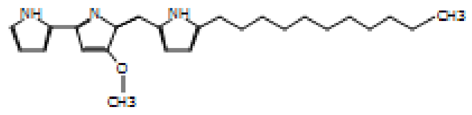

Compound Label	Name	*m/z*	RT	Algorithm	Mass
Cpd 74: 1,1'-Bis(2-hydroxy-3-	**1,1'-Bis(2-hydroxy-3-methylcarbazole)**	415.1413	16.906	Auto MS/MS	392.1524

MS Spectrum

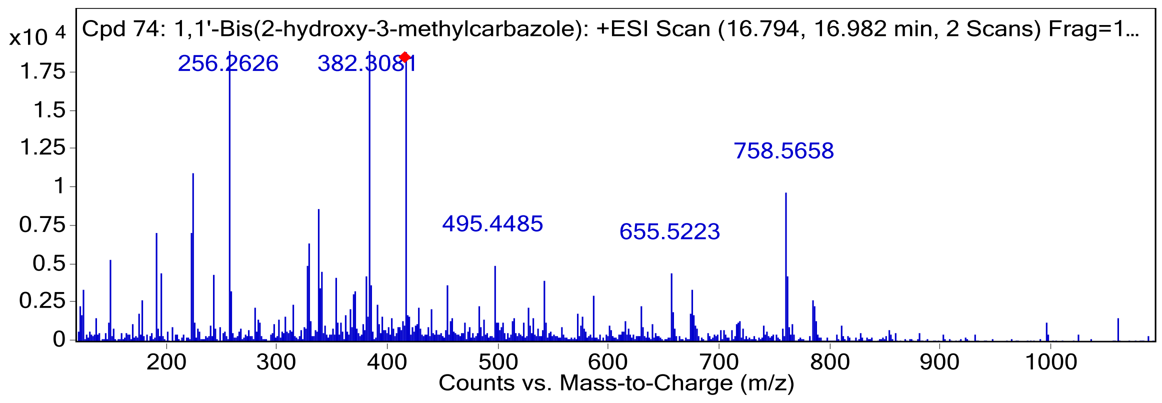

MS Zoomed Spectrum

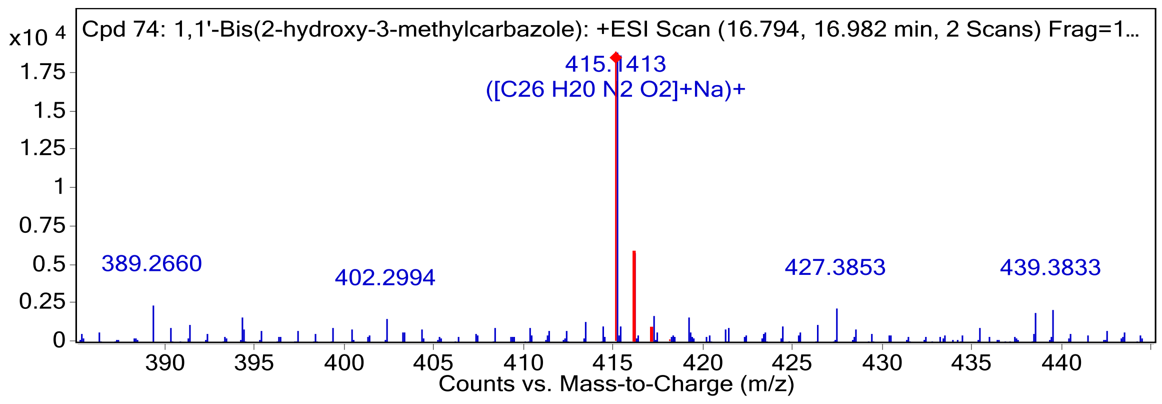

MS Spectrum Peak List

m/z	*Calc m/z*	Diff(ppm)	z	Abund	Formula	Ion
224.1275			1	10968.88		
256.2626			1	19955.12		
337.2145			1	8694.85		
382.3081			1	61366.36		
383.3121			1	20148.93		
415.1413	415.1417	1.05	1	18860.2	C26 H20 N2 O2	(M+Na)+
416.144	416.1449	2.2	1	5870.16	C26 H20 N2 O2	(M+Na)+
417.1523	417.1479	-10.46	1	1791.36	C26 H20 N2 O2	(M+Na)+
418.1597	418.1508	-21.36	1	365.14	C26 H20 N2 O2	(M+Na)+
758.5658			1	9748.44		

MSMS Spectrum

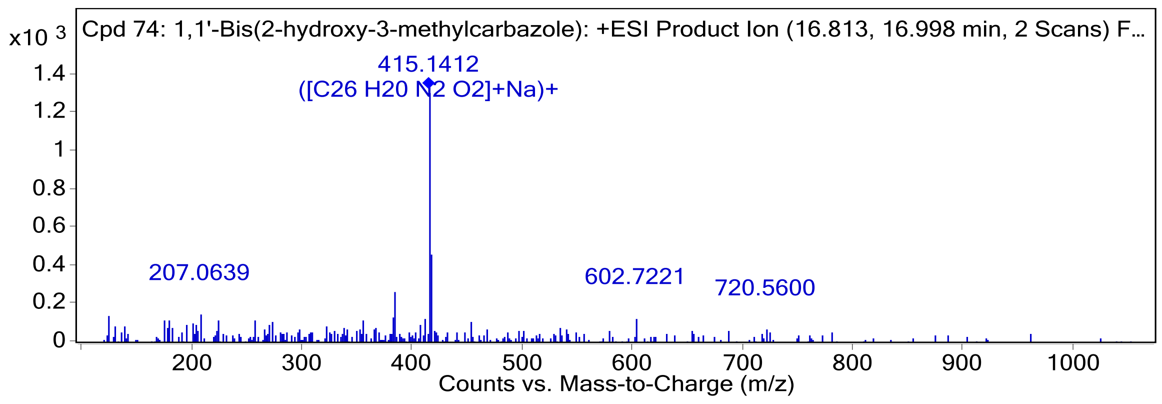

MS/MS Spectrum Peak List

m/z	*Calc m/z*	Diff (ppm)	z	Abund	Formula	Ion
124.086				139.69		

175.1105				120.45		
179.1241				120.25		
207.0639				148.62		
382.3066				132.19		
383.3119				263.57		
411.2642				124.4		
415.1412	415.1417	1.1	1	1306.07	C26 H20 N2 O2	(M+Na)+
416.1457			1	457.34		
602.7221				127.84		

Compound Structure

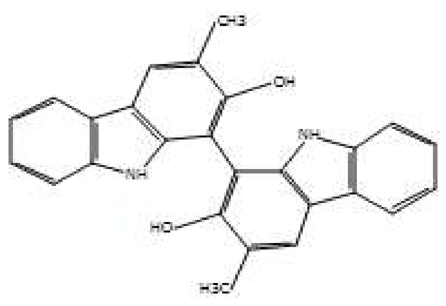

Compound Label	Name	*m/z*	RT	Algorithm	Mass
Cpd 75: 5-Decanoyl-2-nonylpyridine	**5-Decanoyl-2-nonylpyridine**	382.3081	16.947	Auto MS/MS	359.3189

MS Spectrum

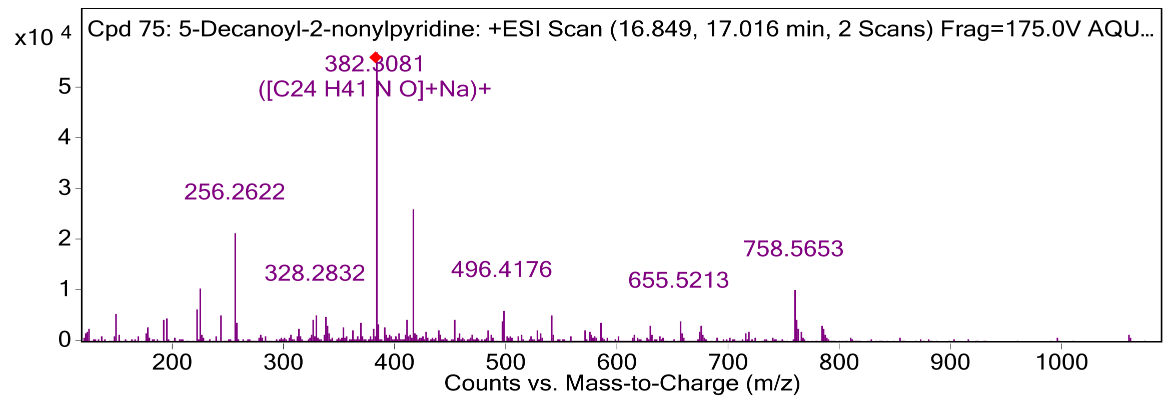

MS Zoomed Spectrum

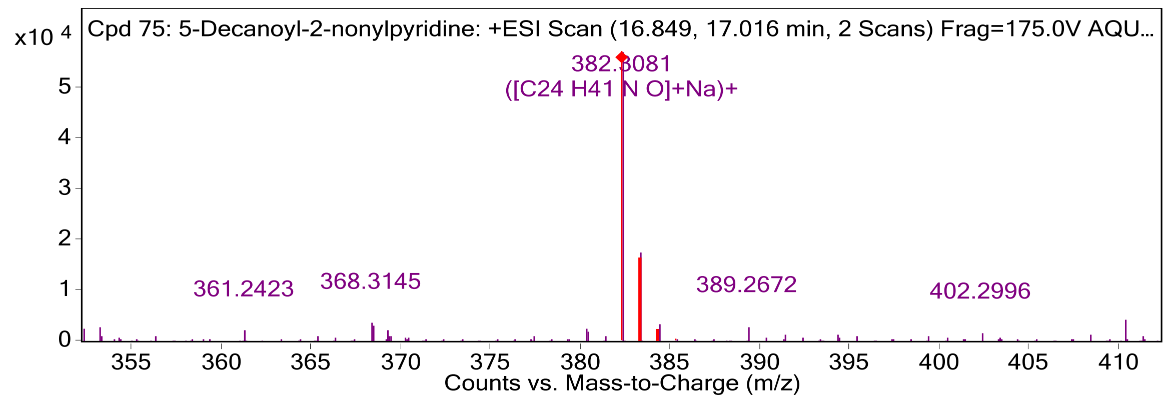

MS Spectrum Peak List

m/z	*Calc m/z*	Diff(ppm)	z	Abund	Formula	Ion
222.1116				6430.31		
224.1274			1	10522.92		
256.2622			1	21456.27		
382.3081	382.308	-0.2	1	57177.81	C24 H41 N O	(M+Na)+
383.3114	383.3114	-0.22	1	17546.5	C24 H41 N O	(M+Na)+
384.3155	384.3145	-2.4	1	3397.34	C24 H41 N O	(M+Na)+
385.3155	385.3176	5.43	1	620.99	C24 H41 N O	(M+Na)+
415.1415			1	26167.39		
416.1446			1	8751.62		
758.5653			1	10205.03		

MSMS Spectrum

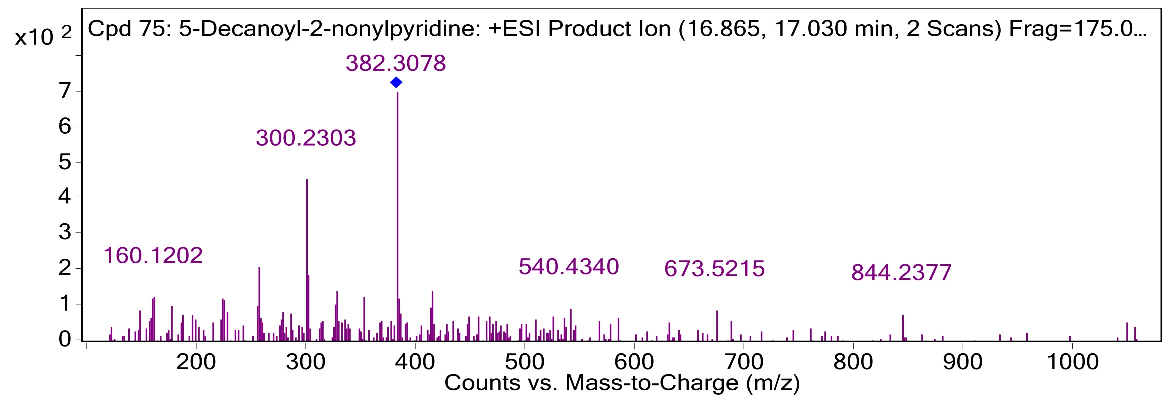

MS/MS Spectrum Peak List

m/z	*Calc m/z*	Diff (ppm)	z	Abund	Formula	Ion
160.1202				125.02		
223.1473				123.17		
256.2643			1	208.16		
300.2303			1	455.24		
301.2346			1	188.28		
327.2687			1	141.57		
352.3174				126.93		
382.3078				699.89		
383.3114	383.3114	-0.03	1	510.17	C24 H41 N O	(M+Na)+
415.138			1	142.12		

Compound Structure

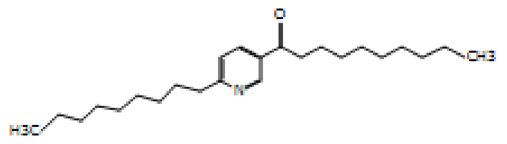

Compound Label	Name	*m/z*	RT	Algorithm	Mass
Cpd 76: 2,4,14-Eicosatrienoic acid isobutylamide	**2,4,14-Eicosatrienoic acid isobutylamide**	384.3234	17.907	Auto MS/MS	361.3342

MS Spectrum

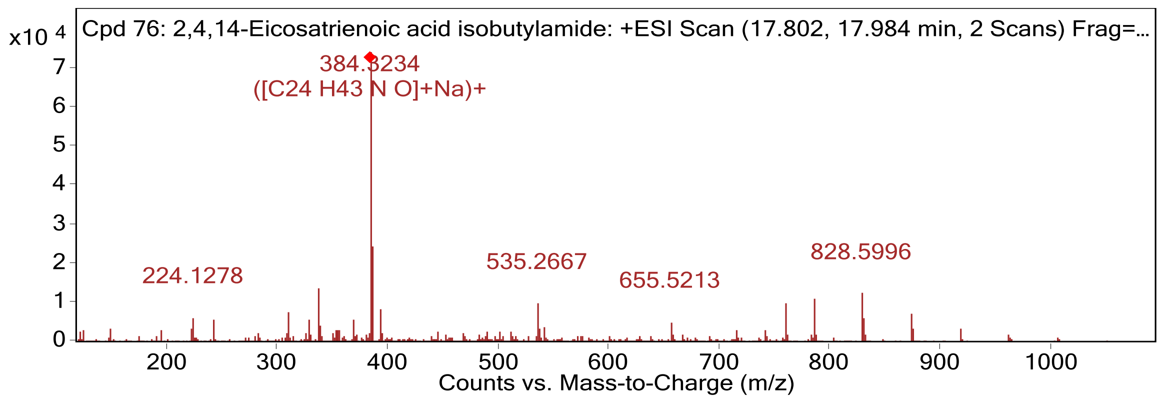

MS Zoomed Spectrum

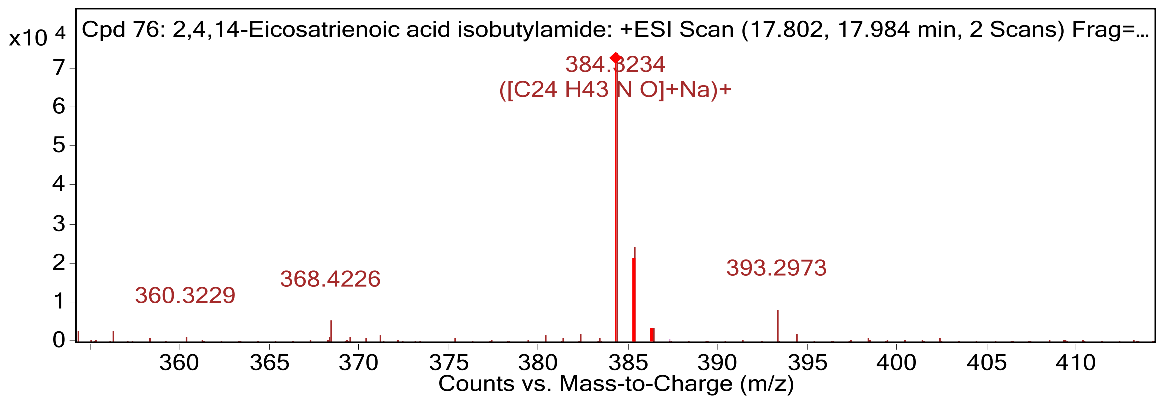

MS Spectrum Peak List

m/z	*Calc m/z*	Diff(ppm)	z	Abund	Formula	Ion
310.309				7590.55		
337.2137			1	13912.51		
384.3234	384.3237	0.75	1	74225.09	C24 H43 N O	(M+Na)+
385.3268	385.327	0.57	1	24554.26	C24 H43 N O	(M+Na)+
386.3306	386.3302	-0.92	1	4009.37	C24 H43 N O	(M+Na)+
393.2973			1	8429.57		
535.2667			1	9929.25		
758.5645			1	9982.17		
784.5752			1	11238.43		
828.5996			1	12448.67		

MSMS Spectrum

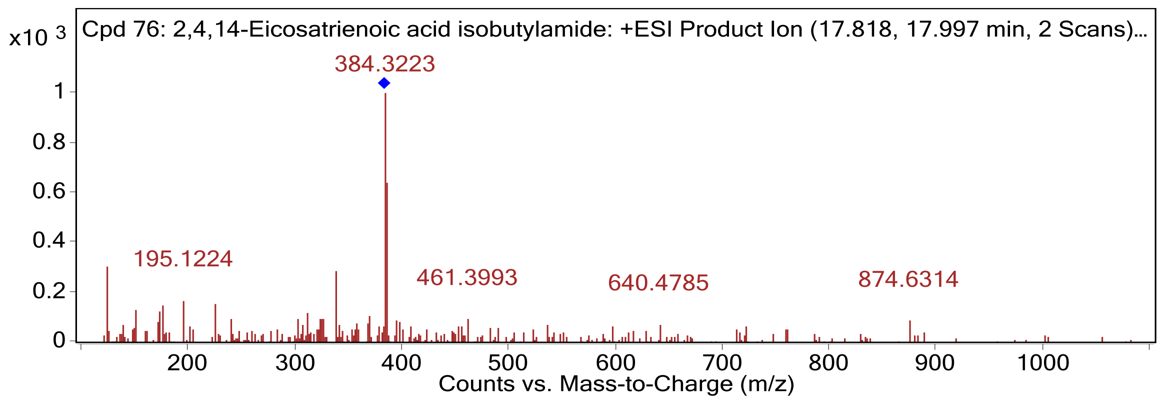

MS/MS Spectrum Peak List

m/z	Abund
123.115	305.25
150.0512	129.44
173.13	127.57
175.1122	149.63
195.1224	169.12
224.1265	154.48
310.3081	121
337.2115	286.56
384.3223	1002.13
385.3254	638.48

Compound Structure

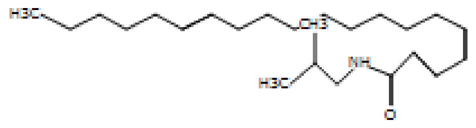

Compound Label	Name	*m/z*	RT	Algorithm	Mass
Cpd 77: 2,4,14-Eicosatrienoic acid isobutylamide	**2,4,14-Eicosatrienoic acid isobutylamide**	384.3233	18.278	Auto MS/MS	361.3342

MS Spectrum

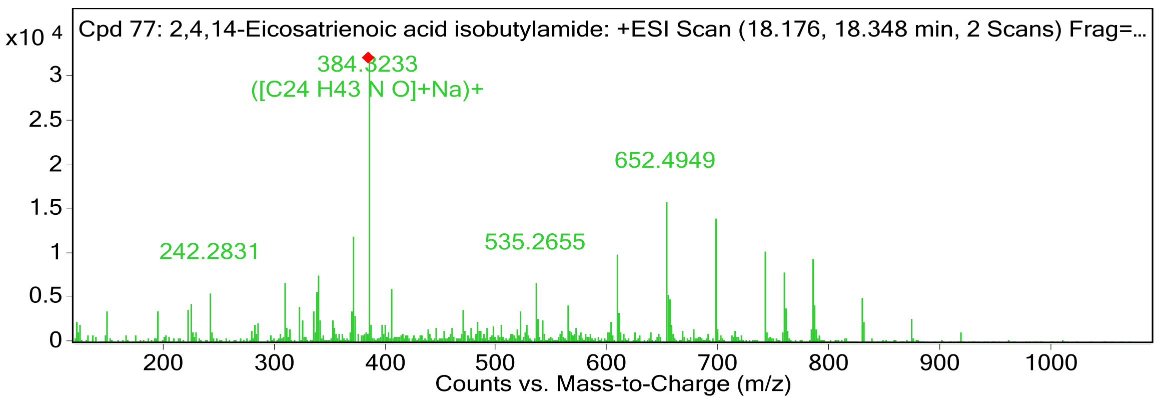

MS Zoomed Spectrum

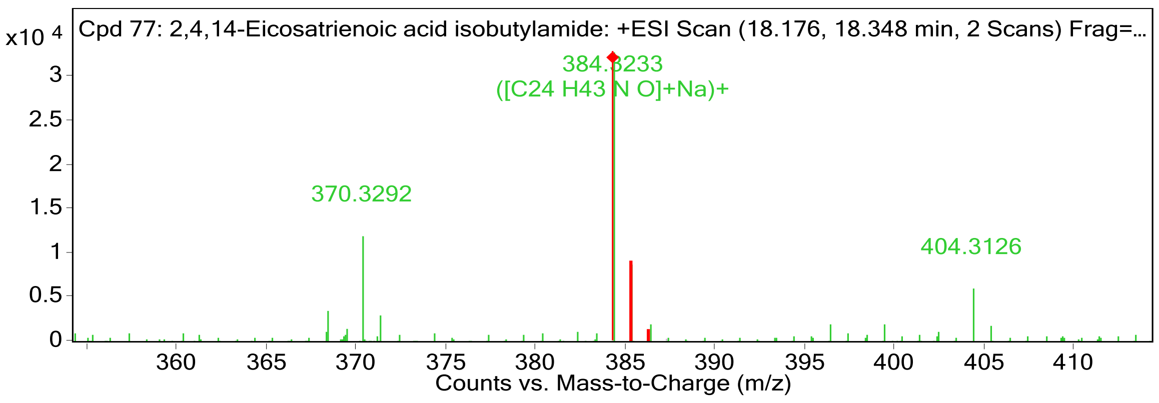

MS Spectrum Peak List

m/z	*Calc m/z*	Diff(ppm)	z	Abund	Formula	Ion
370.3292			1	12074.82		
384.3233	384.3237	0.98	1	32767.23	C24 H43 N O	(M+Na)+
385.327	385.327	0.13	1	8828.23	C24 H43 N O	(M+Na)+
386.3315	386.3302	-3.46	1	1970.71	C24 H43 N O	(M+Na)+
608.4692			1	9922.53		
652.4949			1	15932.74		
696.5225			1	13935.13		
740.5472			1	10338.81		
758.5655			1	7886.3		
784.5757			1	9442.83		

MSMS Spectrum

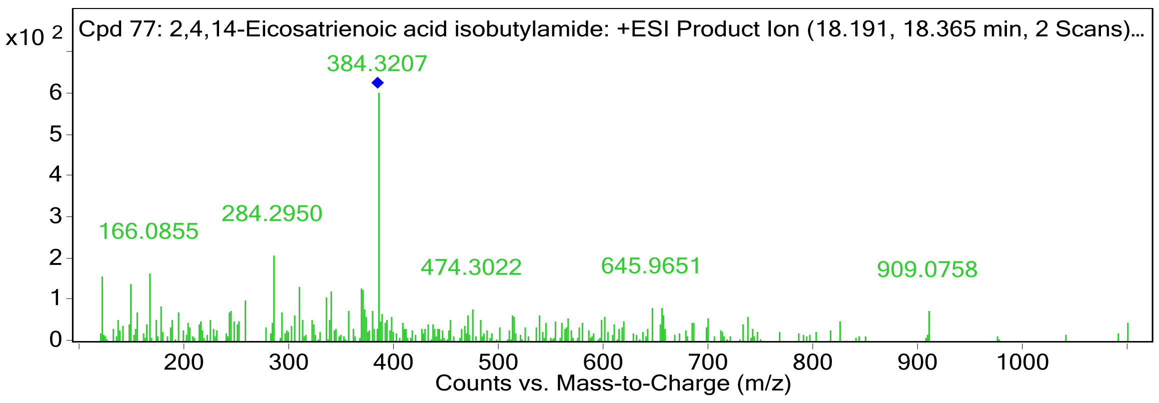

MS/MS Spectrum Peak List

m/z	z	Abund
122.0963		158.32
149.0231		140.34
166.0855		167.84
284.295		210.87
309.2744		134.85
339.3183		122.39
368.4223	1	129.81
370.3263		125.75
384.3207		604.98
385.3265	1	360.97

Compound Structure

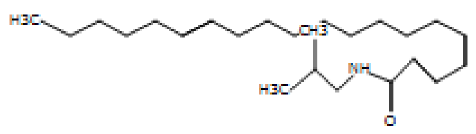

Compound Label	Name	*m/z*	RT	Algorithm	Mass
Cpd 78: Octadecyl fumarate	**Octadecyl fumarate**	391.282	19.422	Auto MS/MS	368.2927

MS Spectrum

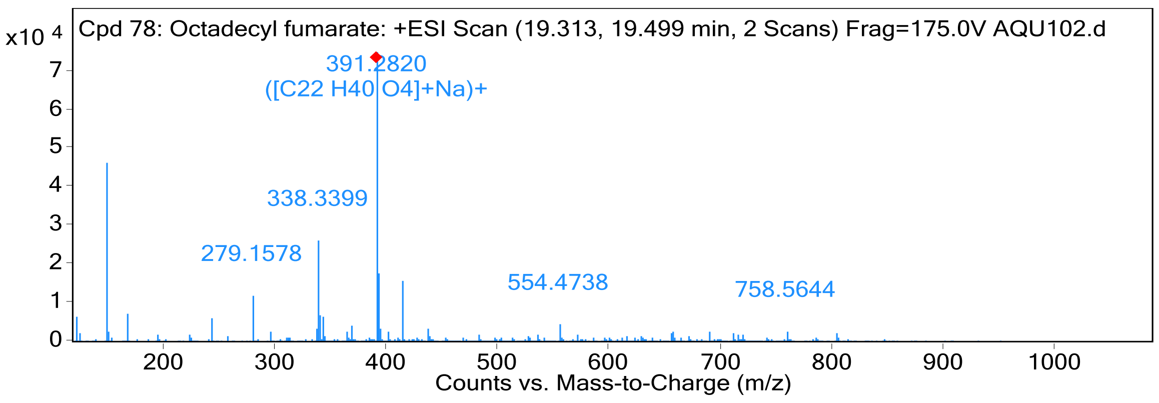

MS Zoomed Spectrum

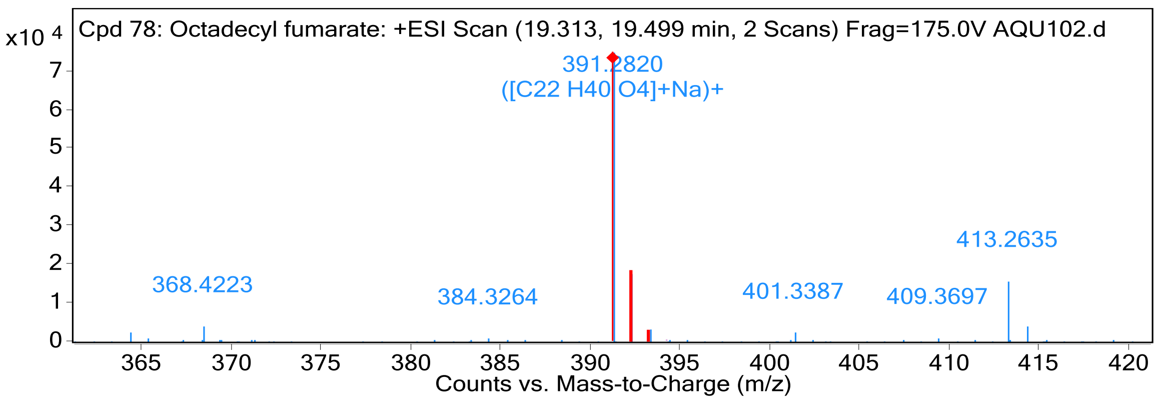

MS Spectrum Peak List

m/z	*Calc m/z*	Diff(ppm)	z	Abund	Formula	Ion
121.0283				6681.98		
149.0231			1	46147.37		
167.0328				7300.37		
279.1578			1	12122.5		
338.3399			1	26413.12		
339.3433			1	7060.82		
391.282	391.2819	-0.42	1	74901.38	C22 H40 O4	(M+Na)+
392.2849	392.2853	1.04	1	17685.35	C22 H40 O4	(M+Na)+
393.2886	393.2881	-1.2	1	3284.52	C22 H40 O4	(M+Na)+
413.2635			1	15936.13		

MSMS Spectrum

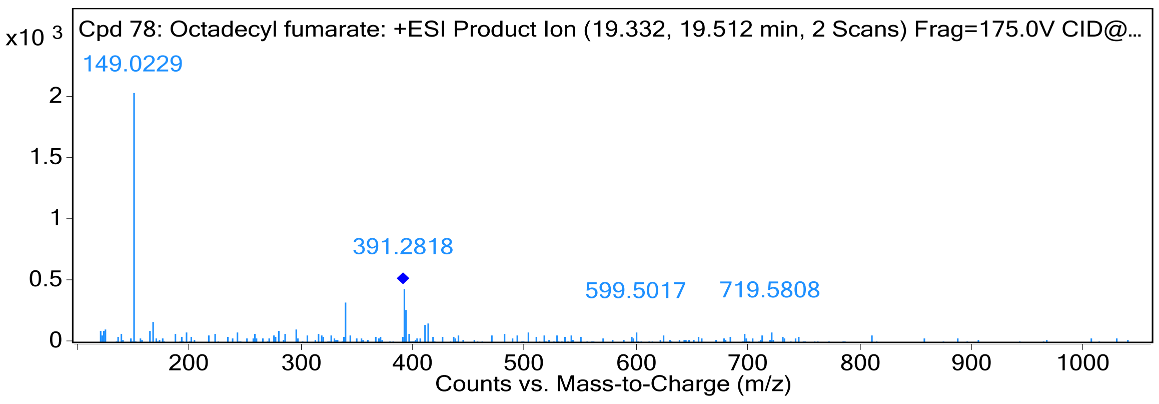

MS/MS Spectrum Peak List

m/z	z	Abund
124.0869		106.08
149.0229	1	2035.1
150.0254	1	166.77
167.0343		166.07
295.1738	1	114.66
338.3414		324.37
391.2818		443.83
392.2877		272.49
409.3794		141.68

m/z	z	Abund
413.2611		161.2

Compound Structure

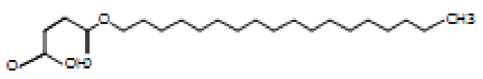

Compound Label	*m/z*	RT	Algorithm
Compound 79	664.5319	20.954	Auto MS/MS

MS Spectrum

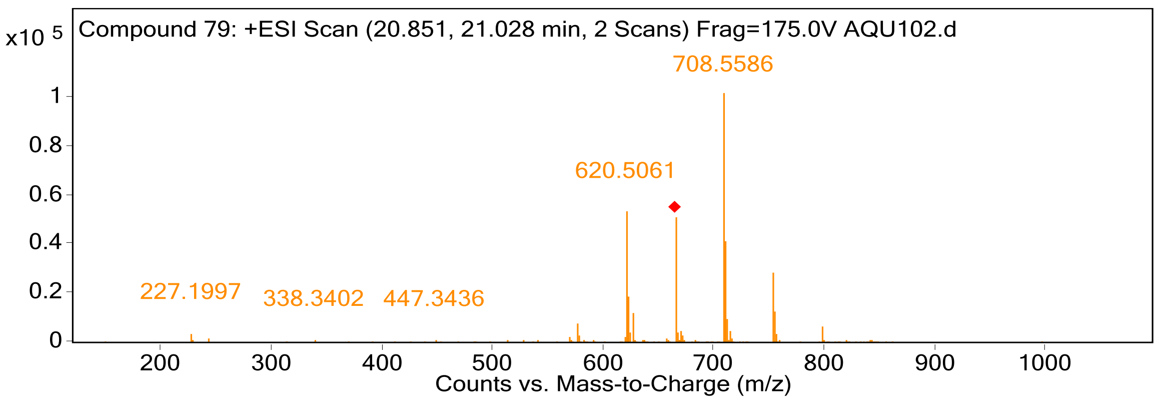

MS Zoomed Spectrum

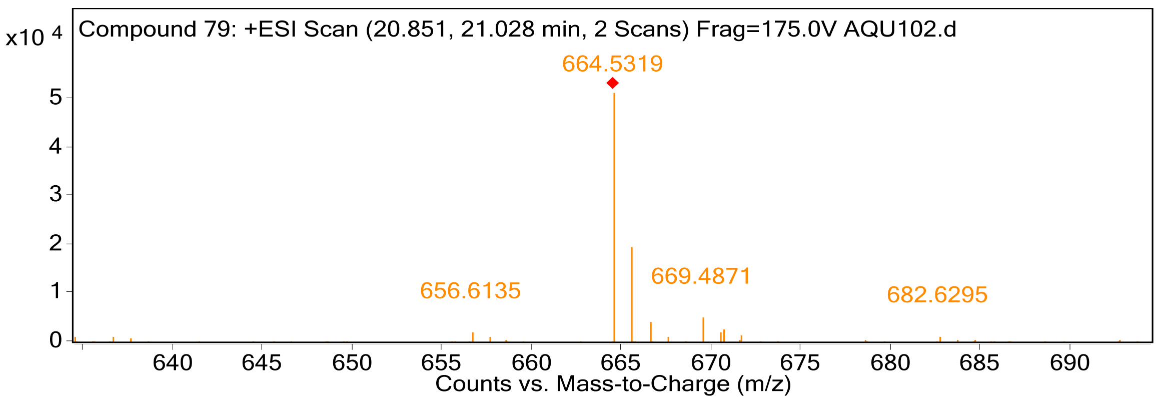

MS Spectrum Peak List

m/z	z	Abund
620.5061	1	53392.51
621.5092	1	18702.67
664.5319	1	51263.67
665.5353	1	19484.98
666.5386	1	4228.59
667.5437	1	1140.81
708.5586	1	101939.62
709.5622	1	41720.01
752.5845	1	28924.04
753.5883	1	12656.48

MSMS Spectrum

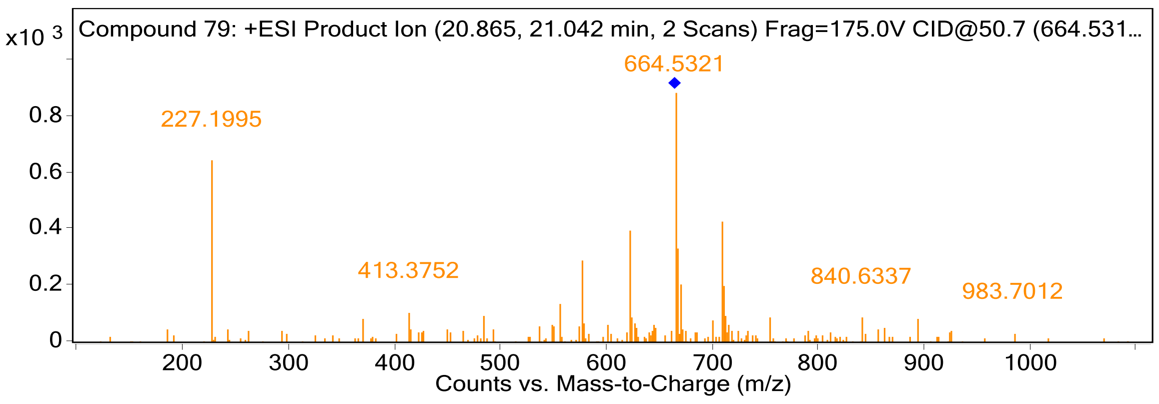

MS/MS Spectrum Peak List

m/z	z	Abund
227.1995		644.97
413.3752		107.94
555.4854		136.31
576.481		289.95
620.5078	1	394.32
664.5321	1	885
665.5401	1	333.69
669.4967	1	204.43
708.5584	1	430.96
709.5661	1	202.06

Compound Label	*m/z*	RT	Algorithm
Compound 80	620.5057	21.035	Auto MS/MS

MS Spectrum

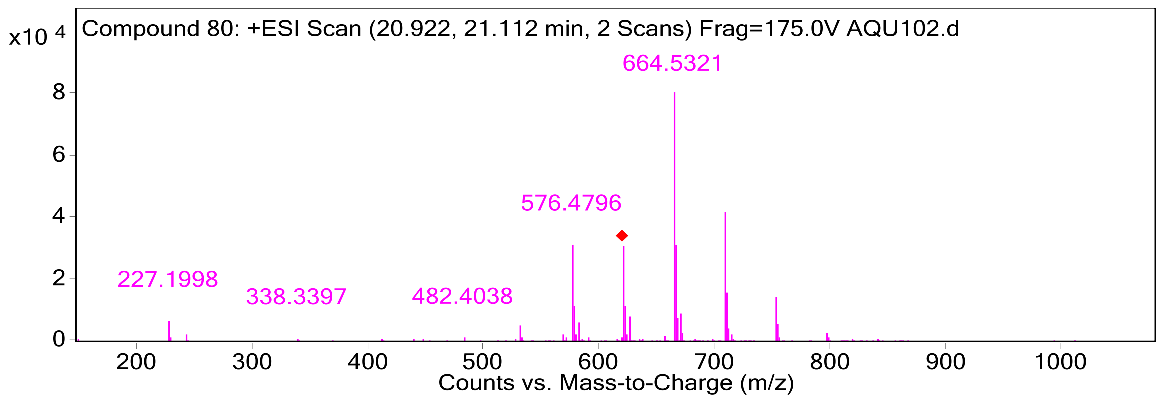

MS Zoomed Spectrum

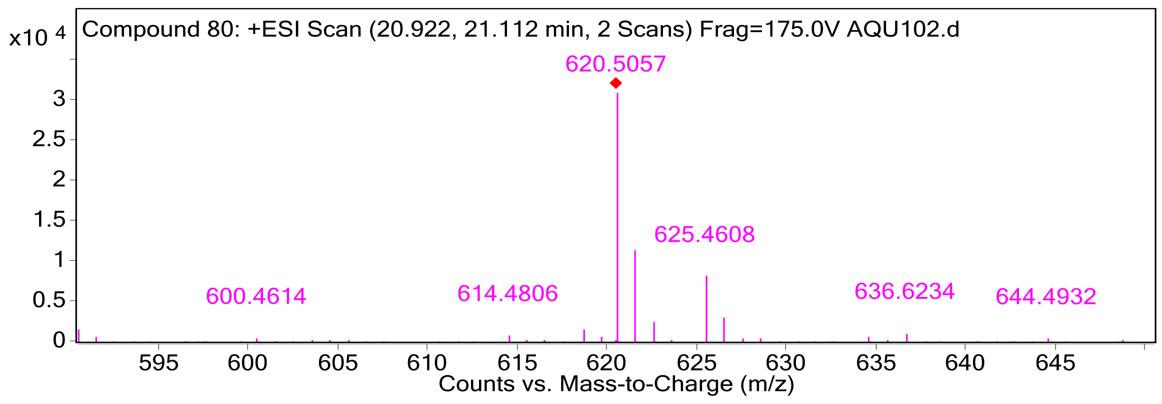

MS Spectrum Peak List

m/z	z	Abund
576.4796	1	31429.67
620.5057	1	31023.28
621.5092	1	11455.15
622.5126	1	2564.43
623.5103	1	373.96
664.5321	1	80690.98
665.5352	1	31160.93
708.5576	1	42195.5
709.5613	1	15987.3
752.5843	1	14297.15

MSMS Spectrum

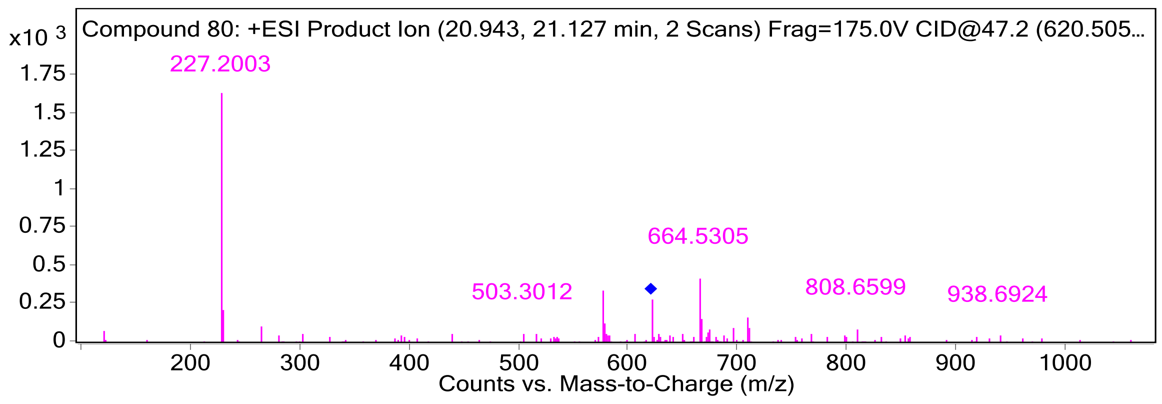

MS/MS Spectrum Peak List

m/z	z	Abund
227.2003	1	1637.5
228.2026	1	217.89
264.2702		111.13
576.4781	1	346.92
577.4868	1	126.56
620.5039	1	281.73
664.5305	1	425.48
665.5416	1	156.66
695.5277		101.55
708.5594	1	168.78

--- End Of Report ---

Qualitative Compound Report

**Data File**	AQU102_-VEr.d	**Sample Name**	AQU102
**Sample Type**	Sample	**Position**	P1-B5
**Instrument Name**	QTOF	**User Name**	
**Acq Method**	30min_-ve_ESI_plant extract_MSMS.m	**Acquired Time**	4/19/2022 1:51:33 PM
**IRM Calibration Status**	Success	**DA Method**	default.m
**Comment**			

Sample Group **Info.**

Acquisition SW Version 6200 series TOF/6500 series Q-TOF B.05.01 (B5125.3)

Compound Table

Compound Label	RT	Mass	Abund	Name	Formula	Tgt Mass	Diff (ppm)	MFG Formula	DB Formula	DB Diff (ppm)	Hits (DB)
Cpd 2: 1-[2-Bromo-1-(4-chlorophenyl)ethenyl]-2-chlorobenzene	0.924	325.9289		1-[2-Bromo-1-(4-chlorophenyl)ethenyl]-2-chlorobenzene	C14 H9 Br Cl2			C14 H9 Br Cl2	C14 H9 Br Cl2	-7.57	1
Compound 3	1.15										
Cpd 4: 4,4'-(2-Methylpropylidene)bisphenol	4.051	242.1318	18039	4,4'-(2-Methylpropylidene)bisphenol	C16 H18 O2			C16 H18 O2	C16 H18 O2	-4.64	8
Cpd 5: 4,4'-(2-Methylpropylidene)bisphenol	4.396	242.1319	20362	4,4'-(2-Methylpropylidene)bisphenol	C16 H18 O2			C16 H18 O2	C16 H18 O2	-4.89	8
Cpd 6: 4,4'-(2-Methylpropylidene)bisphenol	4.755	242.1318		4,4'-(2-Methylpropylidene)bisphenol	C16 H18 O2			C16 H18 O2	C16 H18 O2	-4.62	8
Cpd 7: Simetryn	5.079	213.1052		Simetryn	C8 H15 N5 S			C8 H15 N5 S	C8 H15 N5 S	-2.03	4
Cpd 8: guaiazulene	5.102	198.1412	15354	guaiazulene	C15 H18			C15 H18	C15 H18	-1.67	1
Cpd 9: 4,4'-(2-Methylpropylidene)bisphenol	5.133	242.132		4,4'-(2-Methylpropylidene)bisphenol	C16 H18 O2			C16 H18 O2	C16 H18 O2	-5.46	8
Cpd 10: 1,7-Diphenyl-4-hepten-3-one	5.319	264.1531	20835	1,7-Diphenyl-4-hepten-3-one	C19 H20 O			C19 H20 O	C19 H20 O	-6.28	3
Compound 11	5.928		14292								
Cpd 12: Lamtidine	5.98	344.2254	11743	Lamtidine	C18 H28 N6 O			C18 H28 N6 O	C18 H28 N6 O	20.51	5
Cpd 13: Momilactone B	6.116	330.1865	10162	Momilactone B	C20 H26 O4			C20 H26 O4	C20 H26 O4	-10.13	10
Cpd 14: Sethoxydim	6.242	327.1867	10334	Sethoxydim	C17 H29 N O3 S			C17 H29 N O3 S	C17 H29 N O3 S	0.26	8
Cpd 15: Lamtidine	6.329	344.226	13989	Lamtidine	C18 H28 N6 O			C18 H28 N6 O	C18 H28 N6 O	18.67	5
Compound 16	6.528		11901								
Cpd 17: Spirasine I	6.54	355.2181	20664	Spirasine I	C22 H29 N O3			C22 H29 N O3	C22 H29 N O3	-9.34	4
Cpd 18: Spirasine I	6.791	355.2175	28290	Spirasine I	C22 H29 N O3			C22 H29 N O3	C22 H29 N O3	-7.86	4
Cpd 19: Momilactone B	6.797	330.1866	25727	Momilactone B	C20 H26 O4			C20 H26 O4	C20 H26 O4	-10.61	10
Cpd 20: Eseramine	7.211	318.1664	11112	Eseramine	C16 H22 N4 O3			C16 H22 N4 O3	C16 H22 N4 O3	8.73	4
Cpd 21: 2-Deoxystreptidine	7.264	246.1434	16033	2-Deoxystreptidine	C8 H18 N6 O3			C8 H18 N6 O3	C8 H18 N6 O3	2.73	4
Cpd 22: 16-Methyl-epi-nigakihemiacetal B	7.554	404.2233	12569	16-Methyl-epi-nigakihemiacetal B	C23 H32 O6			C23 H32 O6	C23 H32 O6	-8.37	10
Cpd 23: Spirasine I	7.592	355.2171	24524	Spirasine I	C22 H29 N O3			C22 H29 N O3	C22 H29 N O3	-6.64	4
Cpd 24: β-estradiol	7.822	272.1797		β-estradiol	C18 H24 O2			C18 H24 O2	C18 H24 O2	-7.64	9
Cpd 25: Spirasine I	7.839	355.2184	54445	Spirasine I	C22 H29 N O3			C22 H29 N O3	C22 H29 N O3	-10.18	4
Cpd 26: 3alpha,7beta,12alpha-Trihydroxy-5alpha-cholan-24-oic Acid	7.886	408.2826	16582	3alpha,7beta,12alpha-Trihydroxy-5alpha-cholan-24-oic Acid	C24 H40 O5			C24 H40 O5	C24 H40 O5	12.16	10
Cpd 27: β-estradiol	8.083	272.1796	22352	β-estradiol	C18 H24 O2			C18 H24 O2	C18 H24 O2	-7.37	9
Cpd 28: Europine	8.163	329.1814	19130	Europine	C16 H27 N O6			C16 H27 N O6	C16 H27 N O6	7.42	4
Compound 29	8.276		11774								
Cpd 30: Cavipetin C	8.456	388.2543	10228	Cavipetin C	C24 H36 O4			C24 H36 O4	C24 H36 O4	18.29	2
Compound 31	8.497		24991								
Cpd 32: 3alpha,7beta,12alpha-Trihydroxy-5alpha-cholan-24-oic Acid	8.594	408.2827	26798	3alpha,7beta,12alpha-Trihydroxy-5alpha-cholan-24-oic Acid	C24 H40 O5			C24 H40 O5	C24 H40 O5	12	10
Compound 33	8.743		21518								
Cpd 34: 3alpha,7beta,12alpha-Trihydroxy-5alpha-cholan-24-oic Acid	8.967	408.2828	30116	3alpha,7beta,12alpha-Trihydroxy-5alpha-cholan-24-oic Acid	C24 H40 O5			C24 H40 O5	C24 H40 O5	11.66	10
Compound 35	9.061		25735								
Compound 36	9.116		16132								
Cpd 37: Laccarin	9.155	194.102		Laccarin	C10 H14 N2 O2			C10 H14 N2 O2	C10 H14 N2 O2	18.22	3
Compound 38	9.23		26853								
Cpd 39: sn-3-O-(geranylgeranyl)glycerol 1-phosphate	9.299	444.2604	10322	sn-3-O-(geranylgeranyl)glycerol 1-phosphate	C23 H41 O6 P			C23 H41 O6 P	C23 H41 O6 P	8.36	9
Cpd 40: 3alpha,7beta,12alpha-Trihydroxy-5alpha-cholan-24-oic Acid	9.352	408.2831	33063	3alpha,7beta,12alpha-Trihydroxy-5alpha-cholan-24-oic Acid	C24 H40 O5			C24 H40 O5	C24 H40 O5	10.91	10
Compound 41	9.426		26393								
Compound 42	9.493		15944								
Compound 43	9.673		17492								
Cpd 44: 20-Hydroxy-PGF2a	9.775	370.2421	24583	20-Hydroxy-PGF2a	C20 H34 O6			C20 H34 O6	C20 H34 O6	-17.74	3
Compound 45	9.794		19474								
Compound 46	10.238		56985								
Cpd 47: Tragopogonsaponin B	10.674	926.4647		Tragopogonsaponin B	C50 H70 O16			C50 H70 O16	C50 H70 O16	1.84	2
Compound 48	12.034		33067								
Cpd 49: alpha-Tocopherolquinone	12.816	446.3715		alpha-Tocopherolquinone	C29 H50 O3			C29 H50 O3	C29 H50 O3	10.15	8
Cpd 50: alpha-Tocopherolquinone	13.104	446.3708		alpha-Tocopherolquinone	C29 H50 O3			C29 H50 O3	C29 H50 O3	11.67	8
Compound 51	13.263		59924								
Cpd 52: Carteolol	13.363	292.1781		Carteolol	C16 H24 N2 O3			C16 H24 N2 O3	C16 H24 N2 O3	1.88	6
Compound 53	13.396										
Cpd 54: Hydroxystenozole	13.503	326.232	19762	Hydroxystenozole	C21 H30 N2 O			C21 H30 N2 O	C21 H30 N2 O	11.81	2
Compound 55	13.528		28435								
Cpd 56: Bryodulcosigenin	13.719	474.3654	12328	Bryodulcosigenin	C30 H50 O4			C30 H50 O4	C30 H50 O4	11.7	10
Cpd 57: Hydroquinidine	14.195	326.1984		Hydroquinidine	C20 H26 N2 O2			C20 H26 N2 O2	C20 H26 N2 O2	3.22	9
Cpd 58: Hydroxystenozole	14.279	326.2322	28347	Hydroxystenozole	C21 H30 N2 O			C21 H30 N2 O	C21 H30 N2 O	10.94	2
Compound 1	14.439	290.0801	1075		C15 H14 O6	290.079	3.8	C15 H14 O6	C15 H14 O6		
Cpd 59: Hydroquinidine	14.574	326.1983		Hydroquinidine	C20 H26 N2 O2			C20 H26 N2 O2	C20 H26 N2 O2	3.33	9
Cpd 60: Schidigeragenin C	14.628	430.3127	15895	Schidigeragenin C	C27 H42 O4			C27 H42 O4	C27 H42 O4	-10.09	10
Compound 61	14.767		11115								
Cpd 62: 5'-Methoxybilobetin	14.812	582.1237		5'-Methoxybilobetin	C32 H22 O11			C32 H22 O11	C32 H22 O11	-12.93	1
Cpd 63: Hydroquinidine	14.944	326.1984		Hydroquinidine	C20 H26 N2 O2			C20 H26 N2 O2	C20 H26 N2 O2	3.22	9

Cpd 64: UDP-2,4-bis(acetamido)-2,4,6-trideoxy-beta-L-altropyranose	15.065	632.1216		UDP-2,4-bis(acetamido)-2,4,6-trideoxy-beta-L-altropyranose	C19 H30 N4 O16 P2			C19 H30 N4 O16 P2	C19 H30 N4 O16 P2	-13.24	1
Cpd 65: 5'-Methoxybilobetin	15.177	582.1239		5'-Methoxybilobetin	C32 H22 O11			C32 H22 O11	C32 H22 O11	-13.29	1
Cpd 66: Cucurbitacin E	15.229	556.3016		Cucurbitacin E	C32 H44 O8			C32 H44 O8	C32 H44 O8	3.6	6
Cpd 67: Hydroquinidine	15.286	326.1985		Hydroquinidine	C20 H26 N2 O2			C20 H26 N2 O2	C20 H26 N2 O2	2.92	9
Cpd 68: UDP-2,4-bis(acetamido)-2,4,6-trideoxy-beta-L-altropyranose	15.403	632.1217		UDP-2,4-bis(acetamido)-2,4,6-trideoxy-beta-L-altropyranose	C19 H30 N4 O16 P2			C19 H30 N4 O16 P2	C19 H30 N4 O16 P2	-13.52	1
Cpd 69: Hydroquinidine	15.63	326.1985	139897	Hydroquinidine	C20 H26 N2 O2			C20 H26 N2 O2	C20 H26 N2 O2	2.81	9
Compound 70	15.766										
Cpd 71: UDP-2,4-bis(acetamido)-2,4,6-trideoxy-beta-L-altropyranose	15.778	632.1217		UDP-2,4-bis(acetamido)-2,4,6-trideoxy-beta-L-altropyranose	C19 H30 N4 O16 P2			C19 H30 N4 O16 P2	C19 H30 N4 O16 P2	-13.48	1
Cpd 72: Hydroquinidine	16.016	326.1983		Hydroquinidine	C20 H26 N2 O2			C20 H26 N2 O2	C20 H26 N2 O2	3.37	9
Compound 73	16.14		68592								
Compound 74	16.326										
Cpd 75: Hydroquinidine	16.371	326.1983	63675	Hydroquinidine	C20 H26 N2 O2			C20 H26 N2 O2	C20 H26 N2 O2	3.37	9
Compound 76	16.438		41693								
Compound 77	16.686										
Compound 78	16.687		41591								
Cpd 79: Hydroquinidine	16.741	326.1983	79831	Hydroquinidine	C20 H26 N2 O2			C20 H26 N2 O2	C20 H26 N2 O2	3.4	9
Compound 80	16.82										
Compound 81	17.053										
Cpd 82: Hydroquinidine	17.112	326.1982	110803	Hydroquinidine	C20 H26 N2 O2			C20 H26 N2 O2	C20 H26 N2 O2	3.65	9
Compound 83	17.201										
Compound 84	17.219										
Cpd 85: Hydroquinidine	17.428	326.1986		Hydroquinidine	C20 H26 N2 O2			C20 H26 N2 O2	C20 H26 N2 O2	2.44	9
Compound 86	17.511										
Cpd 87: Hydroquinidine	17.721	326.1985		Hydroquinidine	C20 H26 N2 O2			C20 H26 N2 O2	C20 H26 N2 O2	2.97	9
Cpd 88: 3-O-Mycarosylerythronolide B	17.857	546.3457	15236	3-O-Mycarosylerythronolide B	C28 H50 O10			C28 H50 O10	C28 H50 O10	-9.78	4
Cpd 89: 24-Methylcycloart-23-en-3beta-yl acetate	18.606	482.4176	15274	24-Methylcycloart-23-en-3beta-yl acetate	C33 H54 O2			C33 H54 O2	C33 H54 O2	-10.82	2
Cpd 90: Atenolol	18.648	266.1605		Atenolol	C14 H22 N2 O3			C14 H22 N2 O3	C14 H22 N2 O3	9.5	8
Cpd 91: 22,23-Dihydroergosterol	18.793	398.3477	20233	22,23-Dihydroergosterol	C28 H46 O			C28 H46 O	C28 H46 O	17.99	10
Cpd 92: Atenolol	19.009	266.161		Atenolol	C14 H22 N2 O3			C14 H22 N2 O3	C14 H22 N2 O3	7.85	8
Compound 93	19.105										
Compound 94	19.261										
Cpd 95: Atenolol	19.378	266.1611		Atenolol	C14 H22 N2 O3			C14 H22 N2 O3	C14 H22 N2 O3	7.44	8
Compound 96	19.494										
Compound 97	19.659										
Cpd 98: Atenolol	19.767	266.1609		Atenolol	C14 H22 N2 O3			C14 H22 N2 O3	C14 H22 N2 O3	8.17	8
Compound 99	20.041										
Compound 100	20.426										
Compound 101	20.821										

Compound Label	Name	*m/z*	RT	Algorithm	Mass
Cpd 2: 1-[2-Bromo-1-(4-chlorophenyl)ethenyl]-2-chlorobenzene	**1-[2-Bromo-1-(4-chlorophenyl)ethenyl]-2-chlorobenzene**	384.9426	0.924	Auto MS/MS	325.9289

MS Spectrum

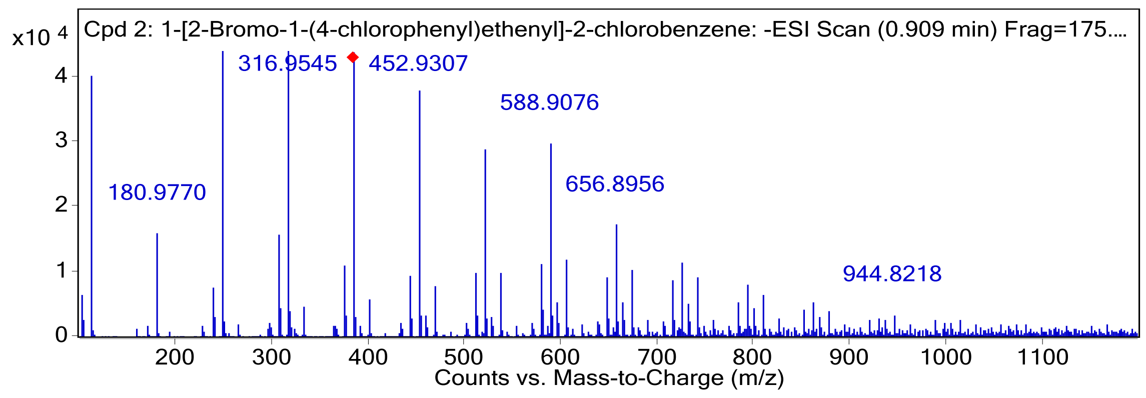

MS Zoomed Spectrum

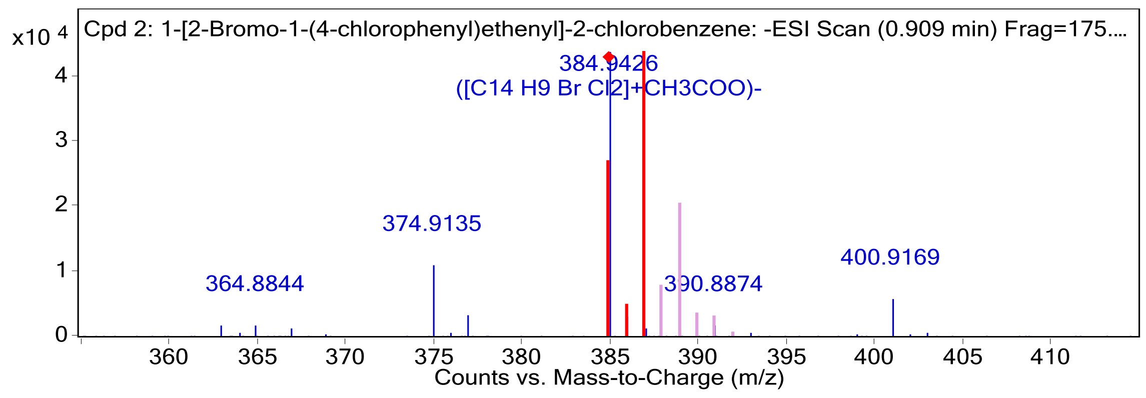

MS Spectrum Peak List

m/z	*Calc m/z*	Diff(ppm)	z	Abund	Formula	Ion
112.9891				40143.55		
248.9658			1	52525.14		
316.9545			1	59867.59		
384.9426	384.9403	-5.85	1	43820.34	C14 H9 Br Cl2	(M+CH3COO)-
385.9462	385.9437	-6.37	1	3153.78	C14 H9 Br Cl2	(M+CH3COO)-
386.9476	386.938	-24.7	1	1372.74	C14 H9 Br Cl2	(M+CH3COO)-
452.9307			1	37986.38		
520.9195			1	28825.09		
588.9076			1	29898.61		
656.8956			1	17316.74		

MSMS Spectrum

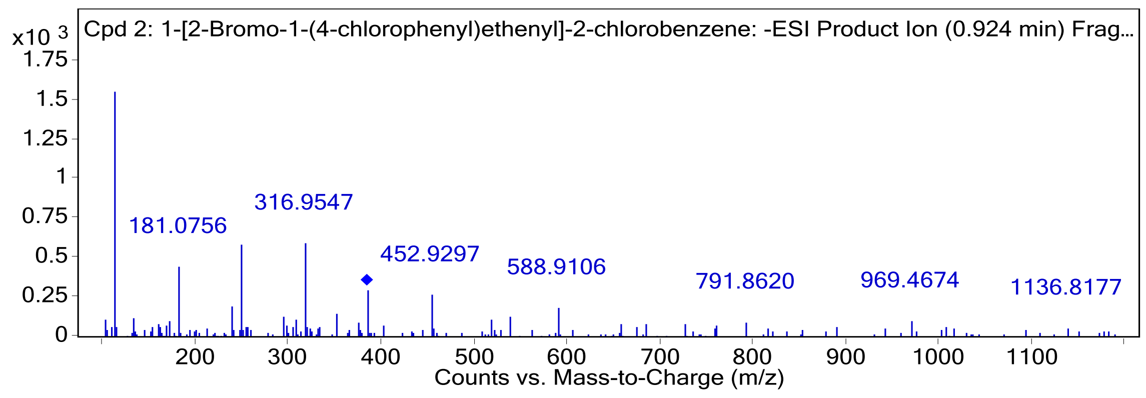

MS/MS Spectrum Peak List

m/z	z	Abund
112.9892	1	1555.14
180.9758		267.24
181.0756	1	447.03
238.9376		190.68
248.9643	1	581.45
316.9547	1	596.01
351.2535		150.9
384.9424		300.2
452.9297		267.5
588.9106		185.39

Compound Structure

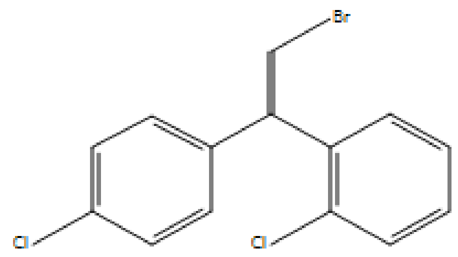

Compound Label	*m/z*	RT	Algorithm
Compound 3	266.8096	1.15	Auto MS/MS

MS Spectrum

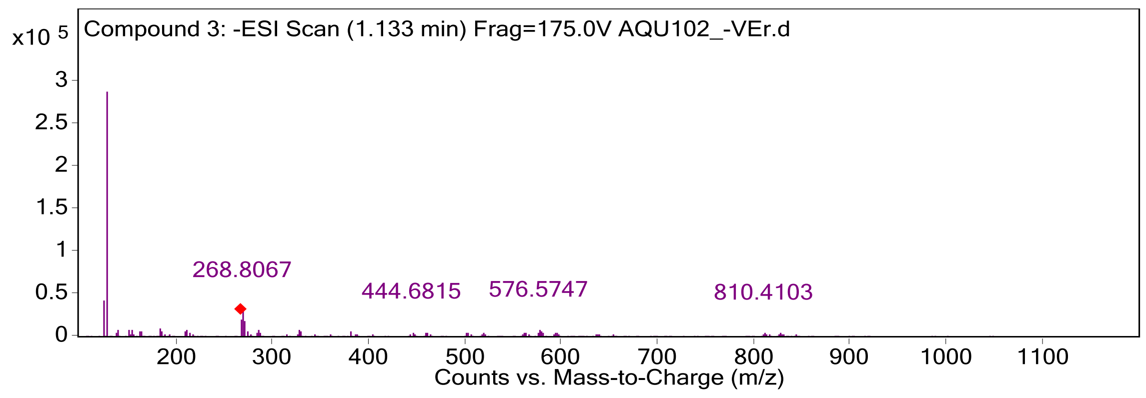

MS Zoomed Spectrum

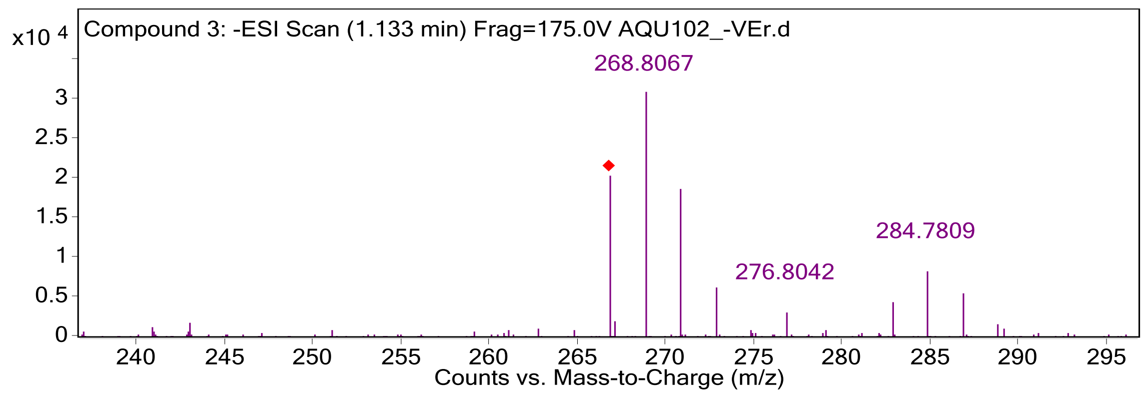

MS Spectrum Peak List

m/z	Abund
124.9948	43016.53
126.9082	287888.25
150.8897	8486.44
152.8872	8016.19
182.9542	9965.96
210.8473	9215.3
266.8096	20267.35
268.8067	30910.64
270.8035	18596.25
284.7809	8251

MSMS Spectrum

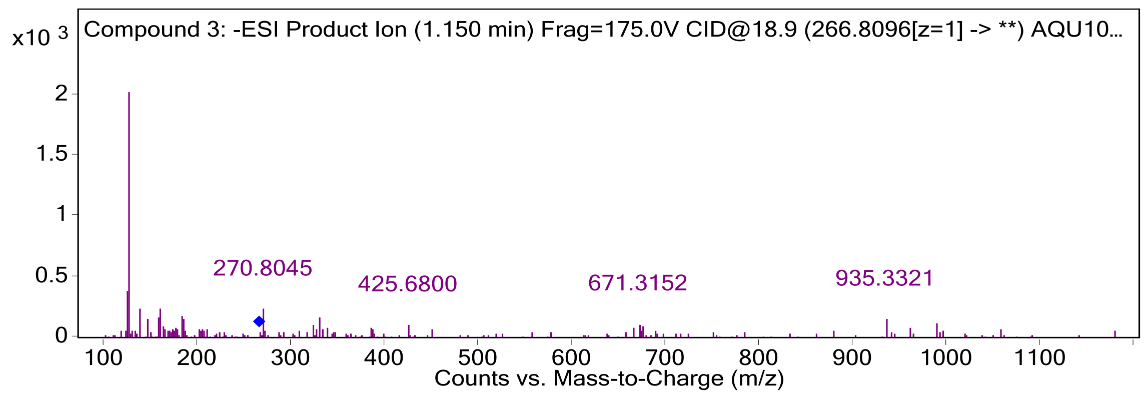

MS/MS Spectrum Peak List

m/z	Abund
124.9941	381.51
126.9076	2024.19
139.0096	242.48
146.9868	160.1
158.923	174.98
160.8473	242.19
182.9552	184.24
186.05	162.04
270.8045	236.4

330.7586	163.72

Compound Label	Name	*m/z*	RT	Algorithm	Mass
Cpd 4: 4,4'-(2-Methylpropylidene)bisphe	**4,4'-(2-Methylpropylidene)bis**	241.1246	4.051	Auto MS/MS	242.1318

MS Spectrum

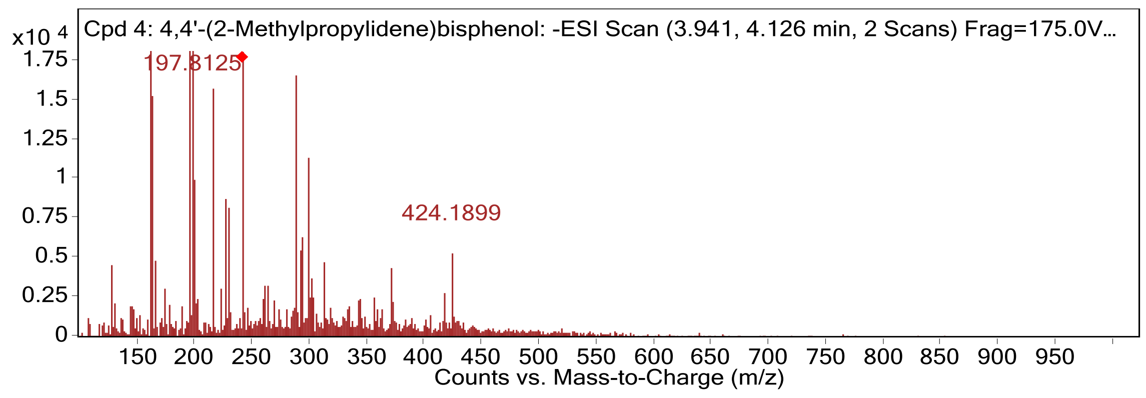

MS Zoomed Spectrum

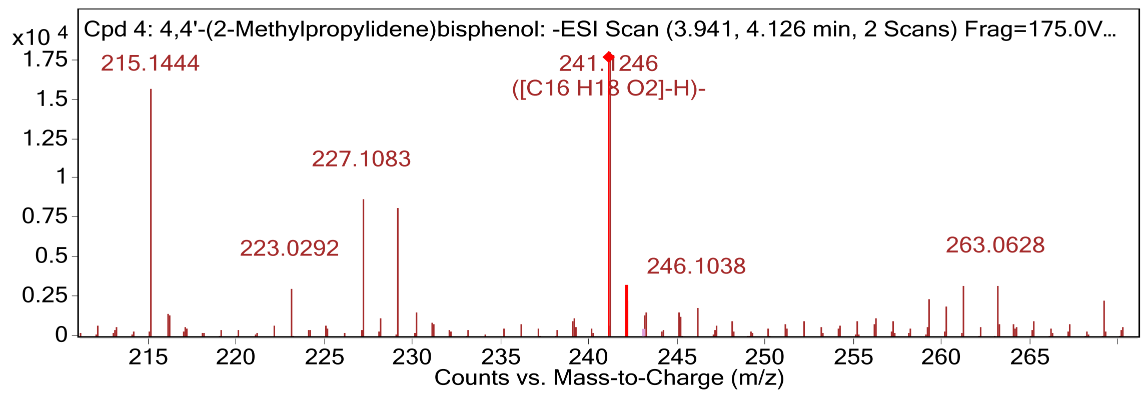

MS Spectrum Peak List

m/z	*Calc m/z*	Diff(ppm)	z	Abund	Formula	Ion
160.846				19201.2		
162.8428				15231.88		
195.8153				19442.1		
197.8125				20260.79		
199.8093				9909.83		
215.1444			1	15668.17		
241.1246	241.1234	-4.79	1	18038.55	C16 H18 O2	(M-H)-
242.1277	242.1268	-3.71	1	2292.43	C16 H18 O2	(M-H)-
287.1306			1	16575.95		
298.1471			1	11366.32		

MSMS Spectrum

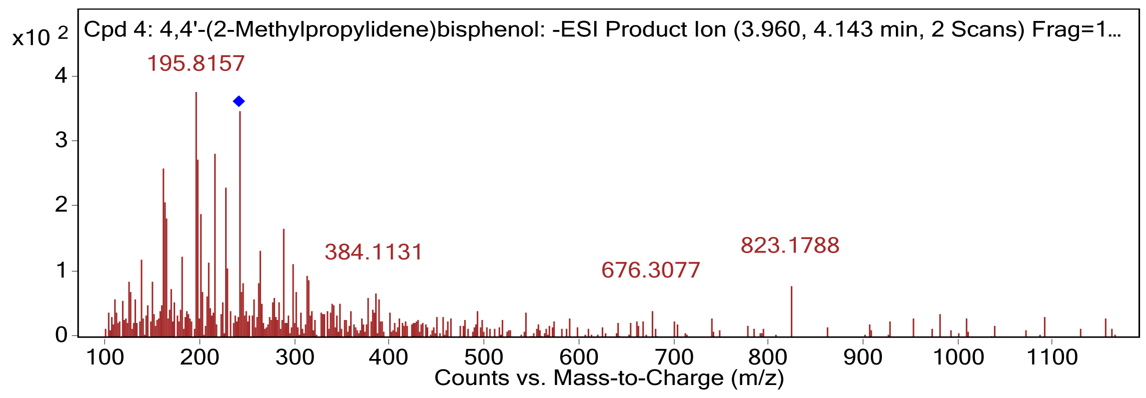

MS/MS Spectrum Peak List

m/z	z	Abund
160.847		259.67
162.8428		206.61
164.8377		183.91
195.8157		377.78
197.8128		273.08
199.8086		189.78
215.1441		282.4
227.1439		230.93
241.1234		347.59
287.1293	1	166.84

Compound Structure

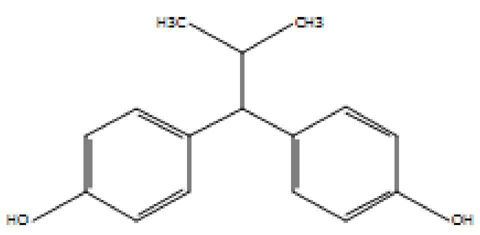

Compound Label	Name	*m/z*	RT	Algorithm	Mass
Cpd 5: 4,4'-(2-Methylpropylidene)bisphe	**4,4'-(2-Methylpropylidene)bis**	241.1246	4.396	Auto MS/MS	242.1319

MS Spectrum

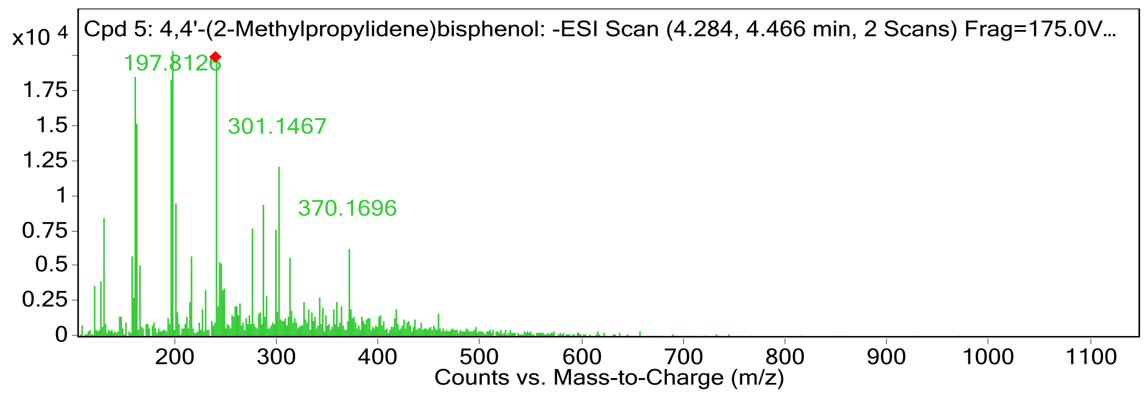

MS Zoomed Spectrum

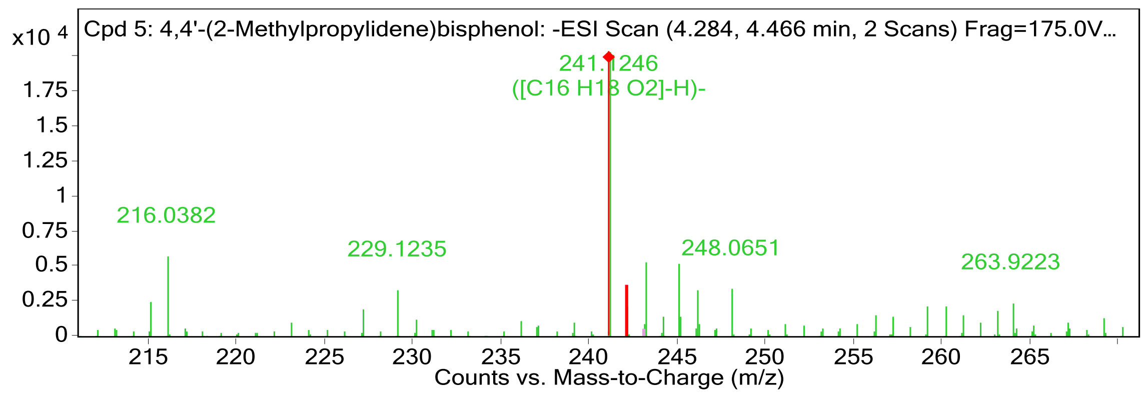

MS Spectrum Peak List

m/z	*Calc m/z*	Diff(ppm)	z	Abund	Formula	Ion
130.0906				8465.82		
160.846				18544.91		
162.8427				15238.48		
195.8154				18380.36		
197.8126				21646.93		
199.8096				9517.49		
241.1246	241.1234	-4.95	1	20362.49	C16 H18 O2	(M-H)-
242.1278	242.1268	-4.3	1	2238.26	C16 H18 O2	(M-H)-
287.1308			1	9402.25		
301.1467			1	12155.35		

MSMS Spectrum

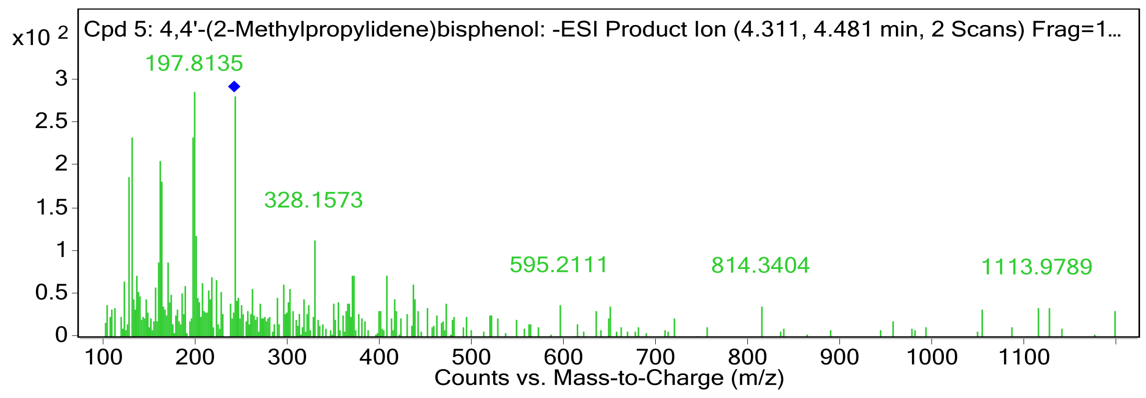

MS/MS Spectrum Peak List

m/z	z	Abund
126.9079		186.13
130.0896		232.83
160.8453		205.17
162.8426		181.5
195.8154		232.44
197.1333		265.98
197.8135		287.02
198.1366		133.09
199.8085		117.87
241.1235	1	280.78

Compound Structure

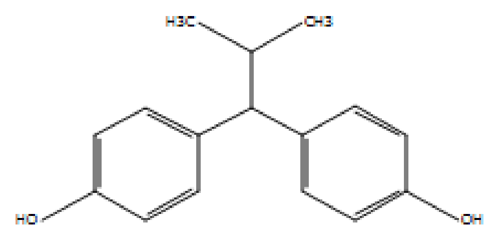

Compound Label	Name	*m/z*	RT	Algorithm	Mass
Cpd 6: 4,4'-(2-Methylpropylidene)bisphe	**4,4'-(2-Methylpropylidene)bis**	241.1245	4.755	Auto MS/MS	242.1318

MS Spectrum

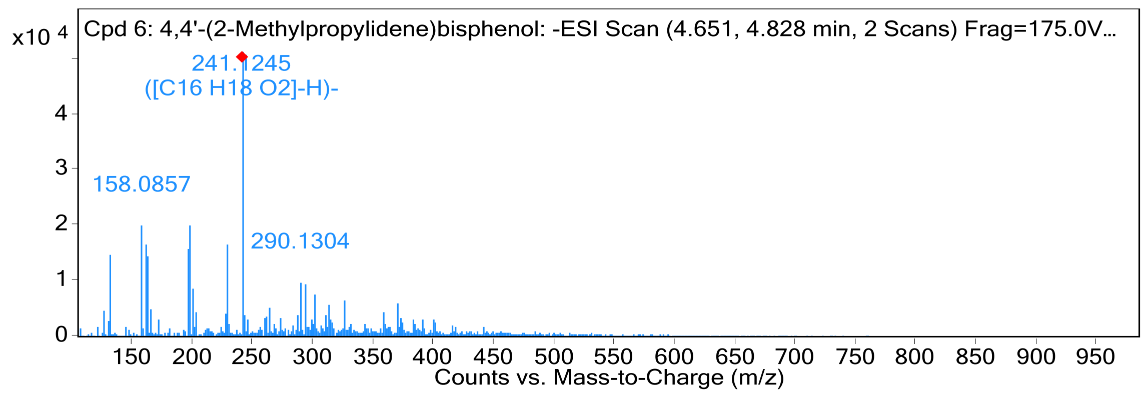

MS Zoomed Spectrum

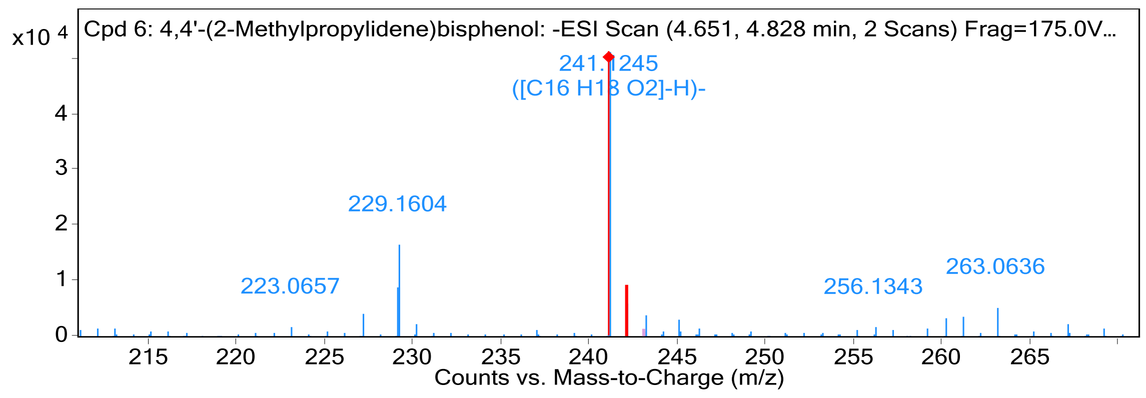

MS Spectrum Peak List

m/z	*Calc m/z*	Diff(ppm)	z	Abund	Formula	Ion
131.075				14858.08		
158.0857			1	20149.63		
160.846				16748.37		
162.8431				14637.35		
195.8153				15729.56		
197.8125				19979.81		
229.1604			1	16619.72		
241.1245	241.1234	-4.7	1	51238.03	C16 H18 O2	(M-H)-

242.1277	242.1268	-3.94	1	6185.47	C16 H18 O2	(M-H)-
290.1304				9887.7		

MSMS Spectrum

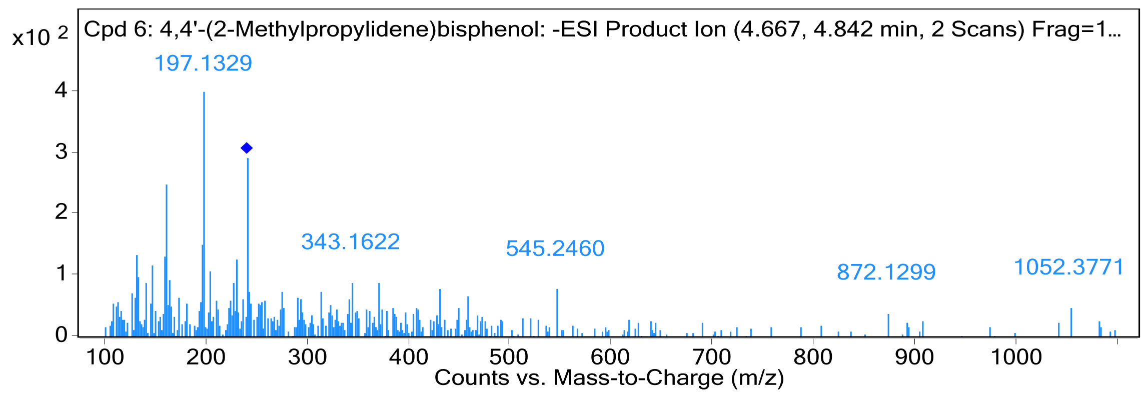

MS/MS Spectrum Peak List

m/z	z	Abund
131.0732		134.73
132.0778		97.69
146.8673		116.31
158.0859		132.33
160.8446		249.28
195.814		150.46
197.1329		400
203.0941		106.45
229.1596		125.9
241.1218	1	291.2

Compound Structure

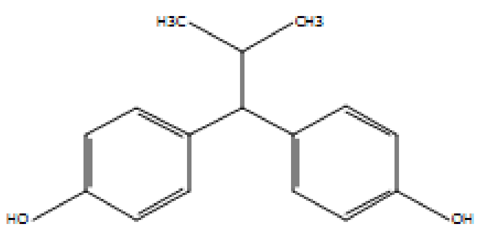

Compound Label	Name	*m/z*	RT	Algorithm	Mass
Cpd 7: Simetryn	**Simetryn**	212.0977	5.079	Auto MS/MS	213.1052

MS Spectrum

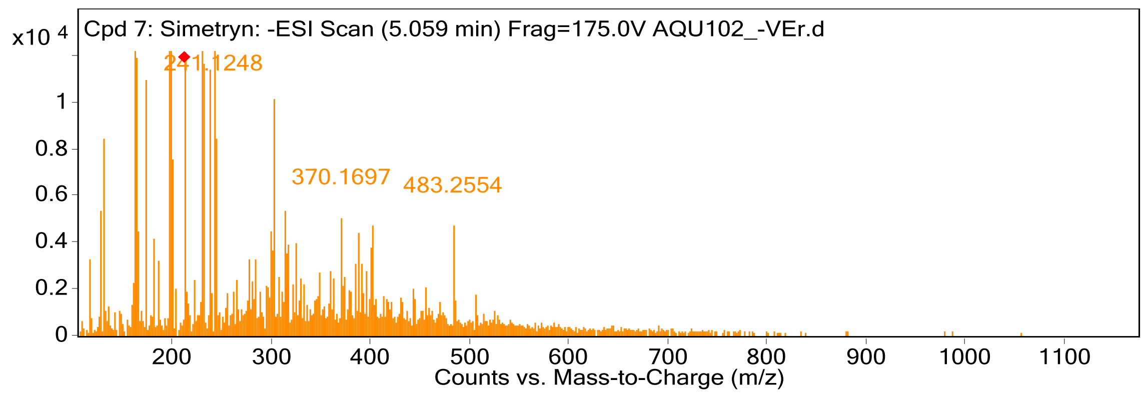

MS Zoomed Spectrum

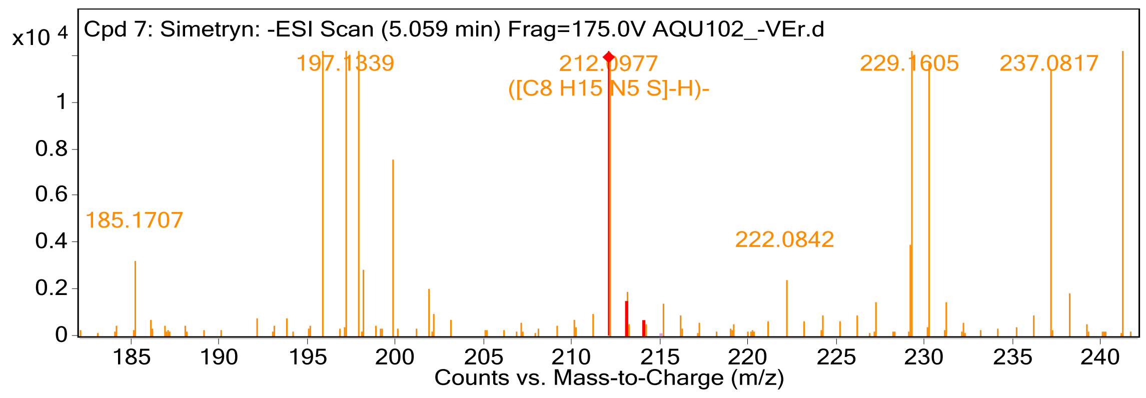

MS Spectrum Peak List

m/z	*Calc m/z*	Diff(ppm)	z	Abund	Formula	Ion
160.8463				14612.48		
195.8154				14891.39		
197.1339			1	31422.04		
197.8127				16768.11		
212.0977	212.0975	-0.68	1	12175.74	C8 H15 N5 S	(M-H)-
213.0987	213.0997	4.64	1	1953.11	C8 H15 N5 S	(M-H)-
214.1063	214.0943	-55.92	1	540.97	C8 H15 N5 S	(M-H)-
229.1605			1	73467.98		
241.1248			1	229542.72		
242.1277			1	25989.01		

MSMS Spectrum

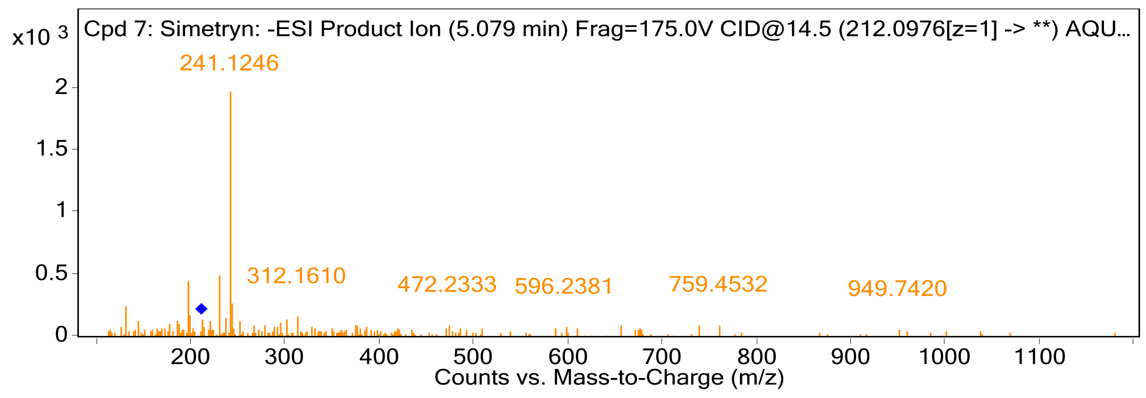

MS/MS Spectrum Peak List

m/z	z	Abund
130.0923		245.01
195.8154		178.74
197.1329		444.18
197.8115		176.84
229.1264		157.97
229.162		491
241.1246	1	1974.97
242.127	1	266.13
243.0919		174.88
312.161		162.07

Compound Structure

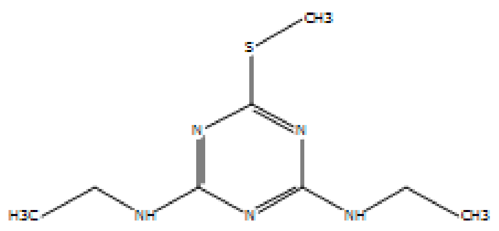

Compound Label	Name	*m/z*	RT	Algorithm	Mass
Cpd 8: guaiazulene	**guaiazulene**	197.1337	5.102	Auto MS/MS	198.1412

MS Spectrum

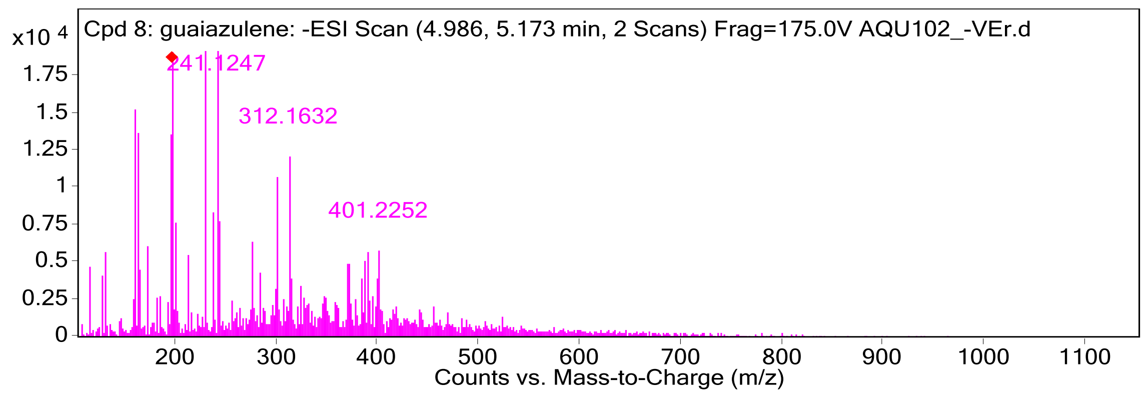

MS Zoomed Spectrum

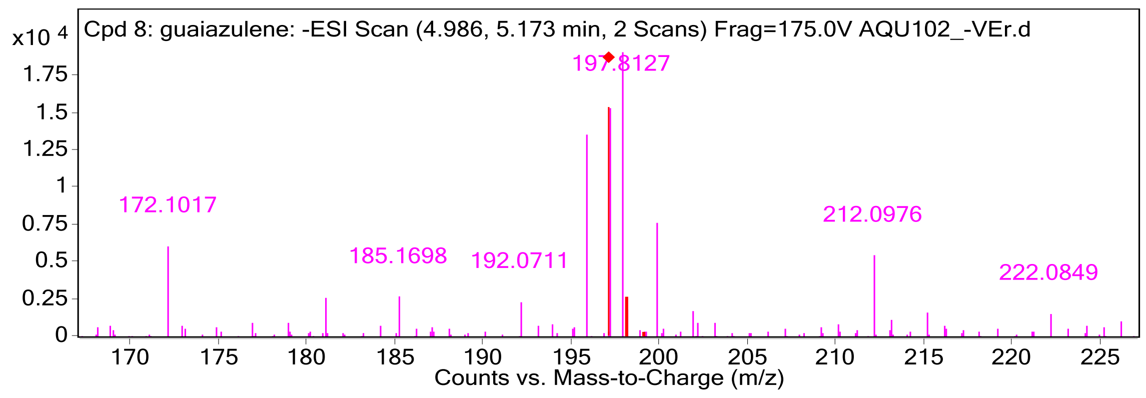

MS Spectrum Peak List

m/z	*Calc m/z*	Diff(ppm)	z	Abund	Formula	Ion
160.8459				15226.44		
162.8431				13694.37		
195.8154				13534.75		
197.1337	197.1336	-0.71	1	15354.02	C15 H18	(M-H)-
197.8127				19090.02		
198.1373	198.137	-1.62	1	1826.86	C15 H18	(M-H)-
199.1492	199.1404	-44.2	1	350.85	C15 H18	(M-H)-
229.1606			1	60420.93		
241.1247			1	111979.5		
242.1275			1	13244.06		

MSMS Spectrum

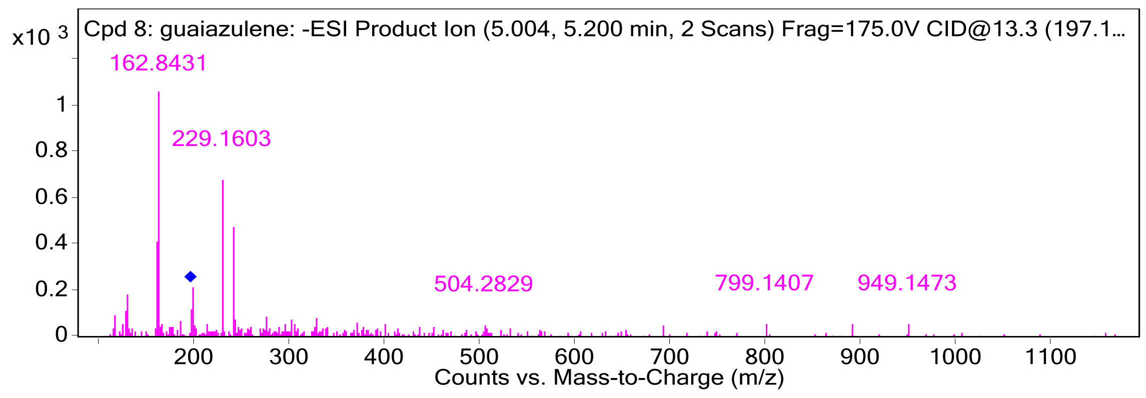

MS/MS Spectrum Peak List

m/z	z	Abund
116.0527		93.91
127.056		116.08
130.089		182.28
160.8457		415.56
162.8431		1063.8
197.1339		122.33
197.8127		216.41
229.1603	1	683.15
241.1243	1	479.89
275.0648		91.92

Compound Structure

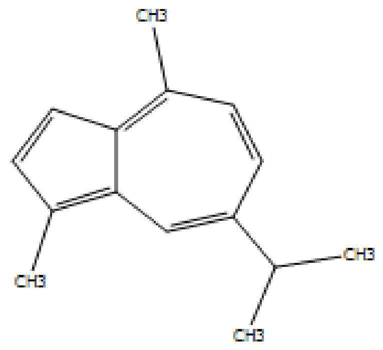

Compound Label	Name	*m/z*	RT	Algorithm	Mass
Cpd 9: 4,4'-(2-Methylpropylidene)bisphe	**4,4'-(2-Methylpropylidene)bis**	241.1247	5.133	Auto MS/MS	242.132

MS Spectrum

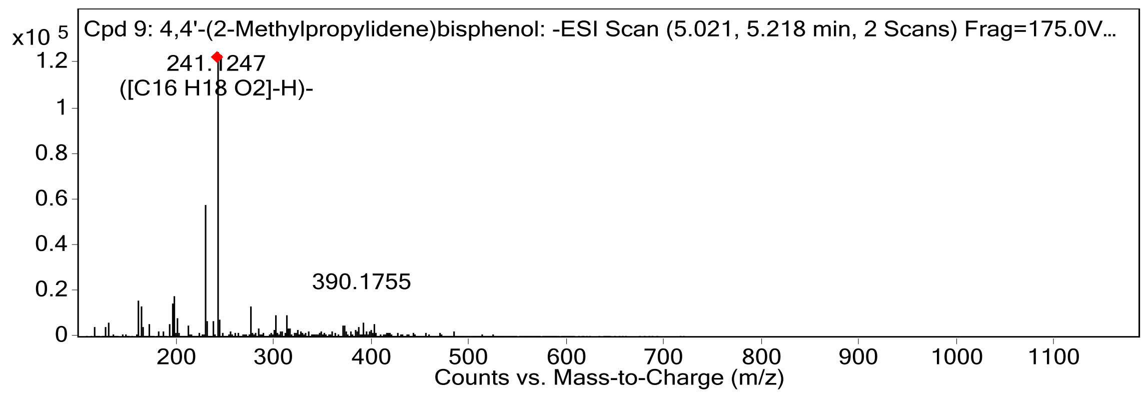

MS Zoomed Spectrum

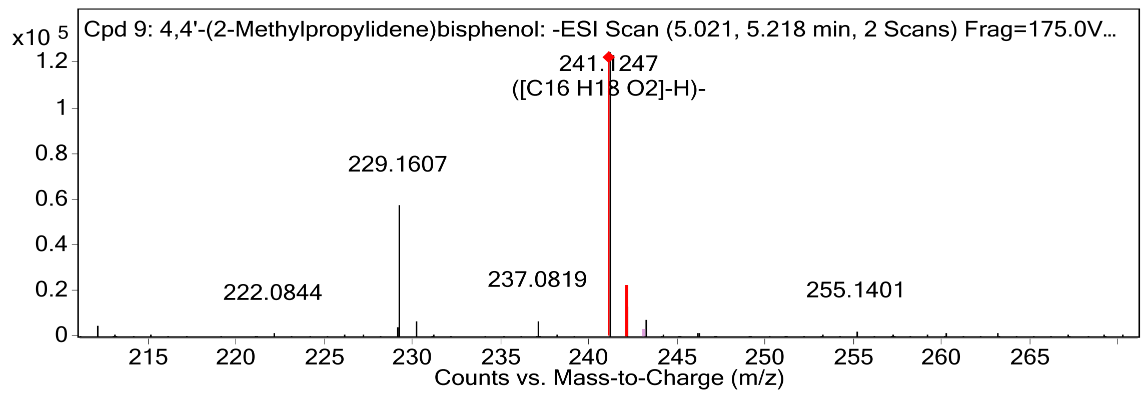

MS Spectrum Peak List

m/z	*Calc m/z*	Diff(ppm)	z	Abund	Formula	Ion
160.846				16249.35		
162.843				13711.55		
195.8155				15045.51		
197.1338			1	14301.6		
197.8128				18000.81		
229.1607			1	58014.77		
241.1247	241.1234	-5.54	1	124802.34	C16 H18 O2	(M-H)-
242.128	242.1268	-4.93	1	13599.72	C16 H18 O2	(M-H)-
275.1097			1	13277.88		
312.1631			1	9949.65		

MSMS Spectrum

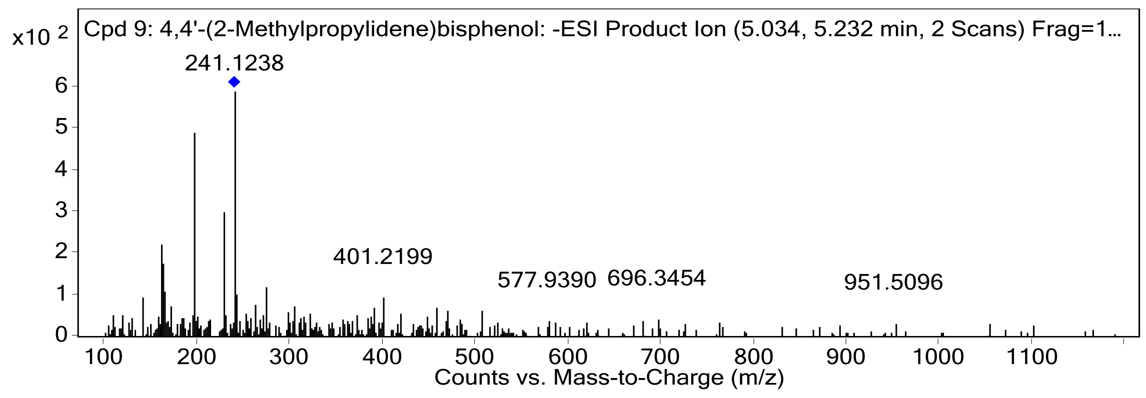

MS/MS Spectrum Peak List

m/z	z	Abund
141.1065		95.4
160.8461		223.06
162.844		162.48
164.0744		177.64
164.838		108
197.1333	1	491.11
229.161	1	298.93
241.1238	1	591.02
242.1275	1	101.64
275.1086		121.5

Compound Structure

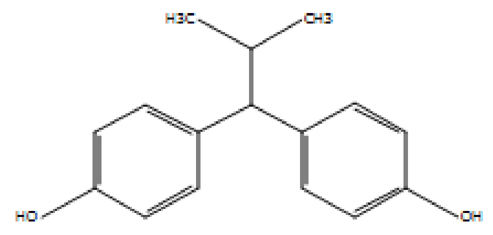

Compound Label	Name	*m/z*	RT	Algorithm	Mass
Cpd 10: 1,7-Diphenyl-4-hepten-3-one	**1,7-Diphenyl-4-hepten-3-one**	263.1458	5.319	Auto MS/MS	264.1531

MS Spectrum

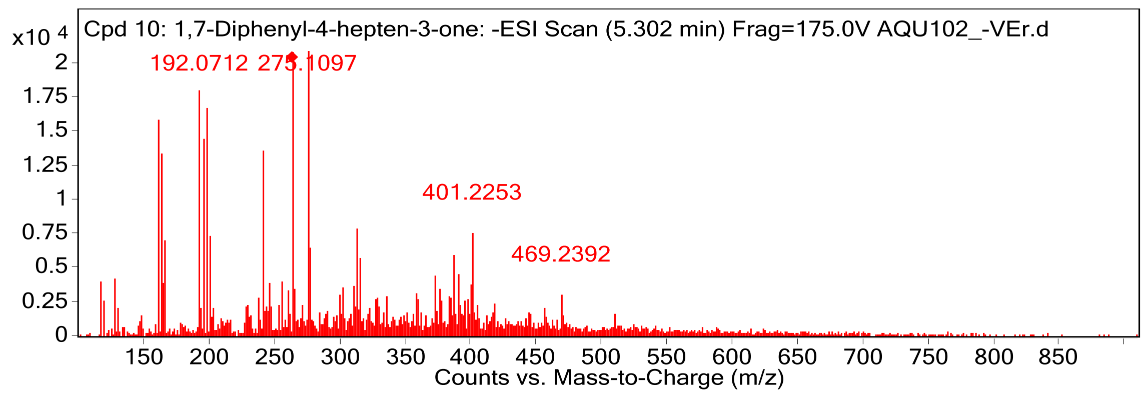

MS Zoomed Spectrum

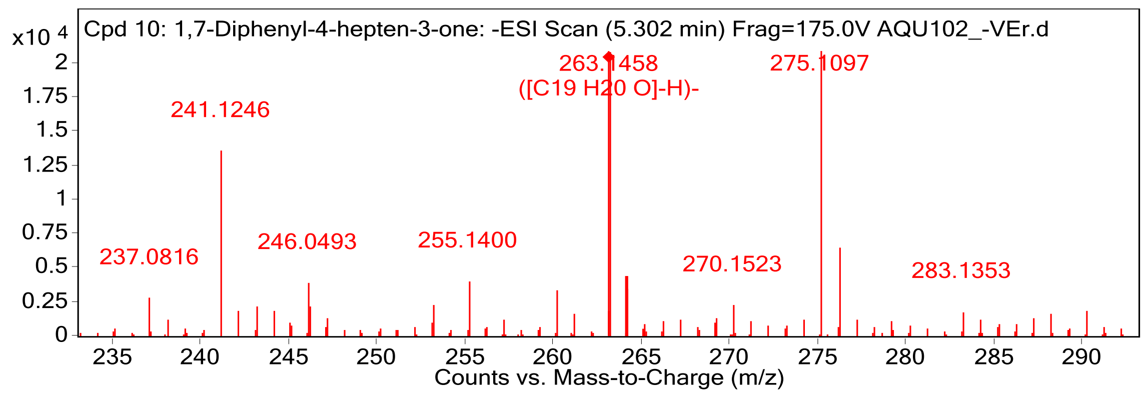

MS Spectrum Peak List

m/z	*Calc m/z*	Diff(ppm)	z	Abund	Formula	Ion
160.8462				15843.37		
162.8432				13405.39		
192.0712			1	18000.21		
195.8155				14509.43		
197.8127				16720.8		
241.1246			1	13662.61		
263.1458	263.1441	-6.39	1	20835.34	C19 H20 O	(M-H)-
264.1491	264.1475	-6.13	1	3506.01	C19 H20 O	(M-H)-
265.152	265.1507	-4.78	1	394.65	C19 H20 O	(M-H)-
275.1097			1	43436.88		

MSMS Spectrum

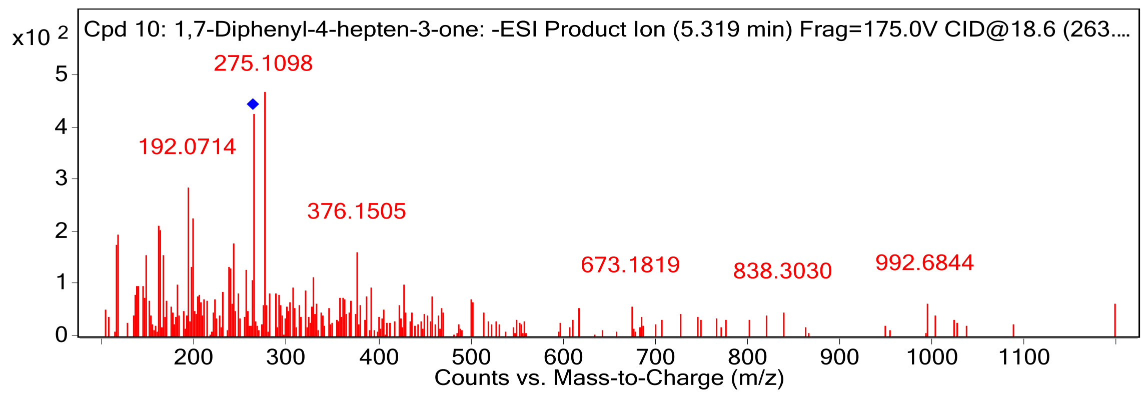

MS/MS Spectrum Peak List

m/z	*Calc m/z*	Diff (ppm)	Abund	Formula	Ion
115.0913			177.24		
116.0541			195.33		
160.8448			213.13		
162.8449			204.75		
192.0714			286.83		
197.8133			228.32		
241.1246			178.82		
263.1457	263.1441	-6.01	427.52	C19 H20 O	(M-H)-
275.1098			470.42		
376.1505			163.07		

Compound Structure

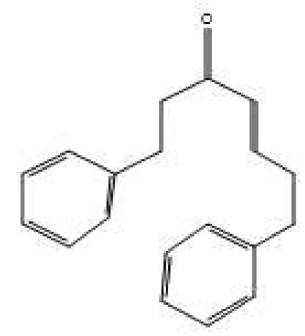

Compound Label	*m/z*	RT	Algorithm
Compound 11	217.1128	5.928	Auto MS/MS

MS Spectrum

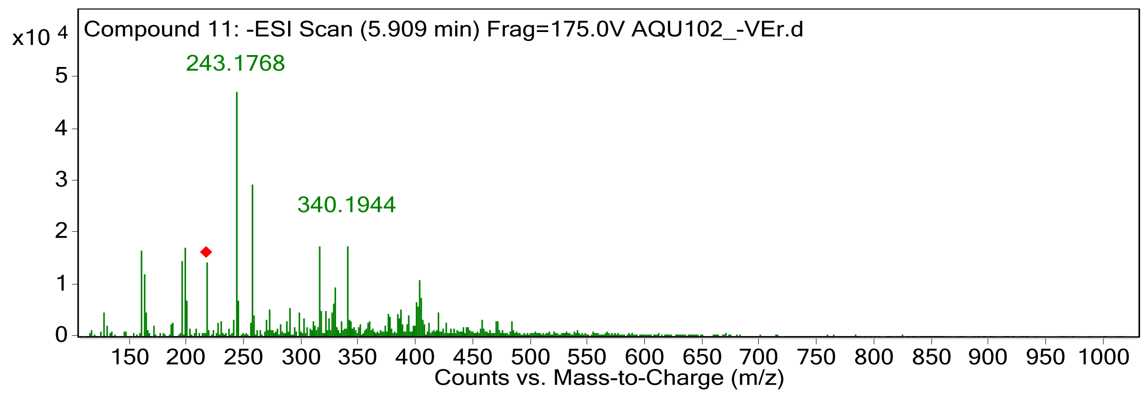

MS Zoomed Spectrum

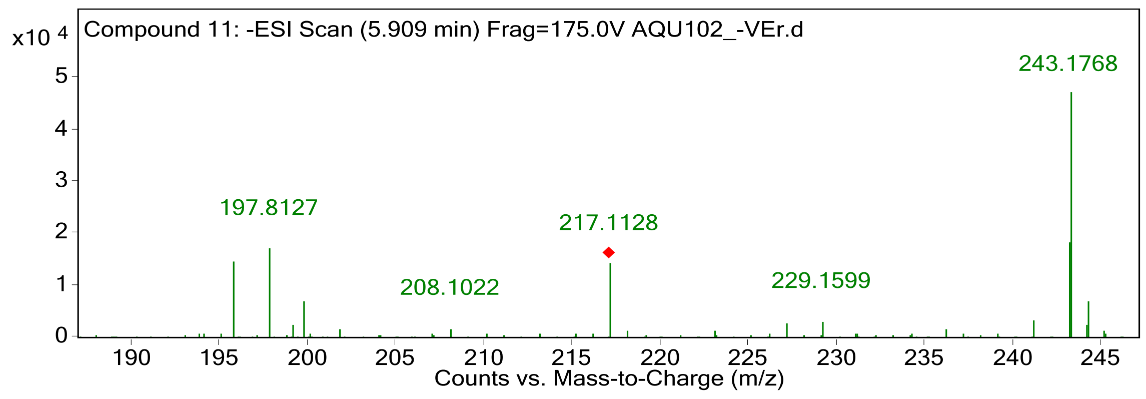

MS Spectrum Peak List

m/z	z	Abund
160.8461		16746.9
197.8127		17203.57
217.1128		14292.01
218.1169	1	1314.86
219.1167	1	518.91
243.1403	1	18311.42
243.1768	1	47327.3
257.1559	1	29470.75
315.1625	1	17404.75

340.1944	1	17658.68

MSMS Spectrum

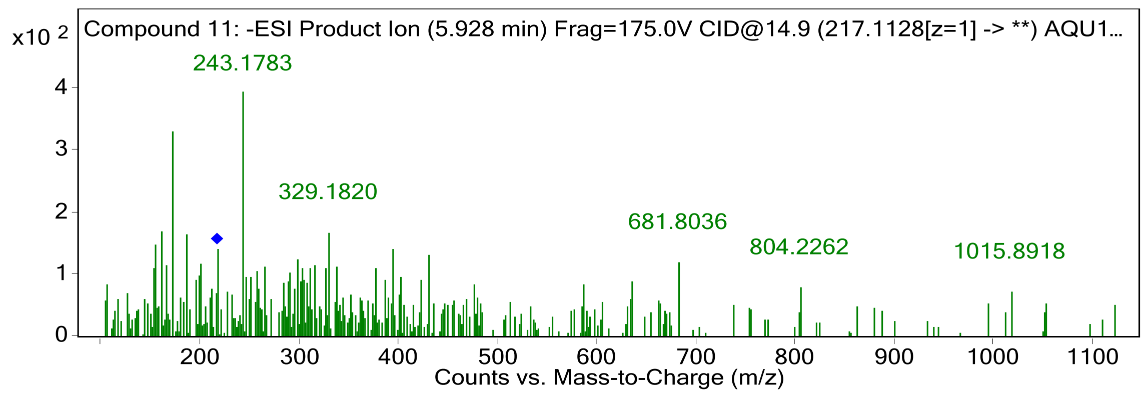

MS/MS Spectrum Peak List

m/z	Abund
155.1095	148.88
160.8465	170.26
171.1058	330.86
186.1154	166.1
217.1123	142.19
243.1396	203.11
243.1783	396.44
329.182	168.52
393.1708	140.89
429.222	131.79

Compound Label	Name	*m/z*	RT	Algorithm	Mass
Cpd 12: Lamtidine	**Lamtidine**	403.2413	5.98	Auto MS/MS	344.2254

MS Spectrum

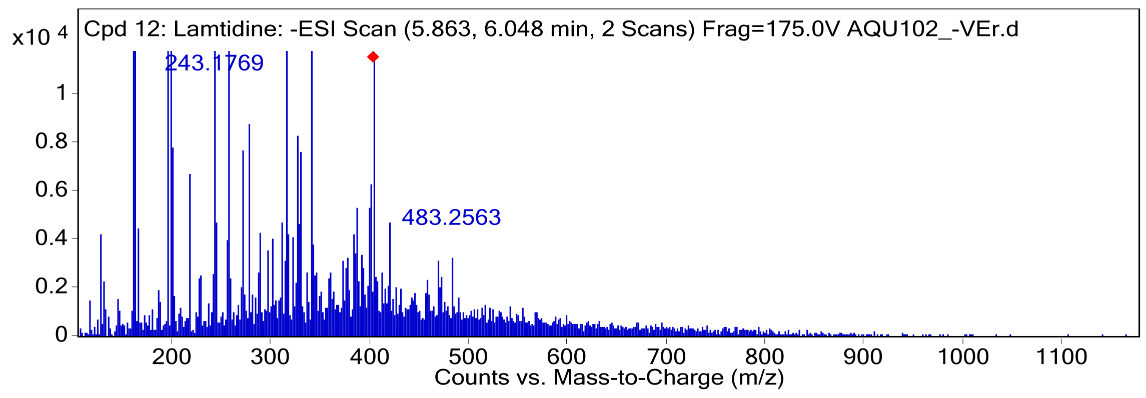

MS Zoomed Spectrum

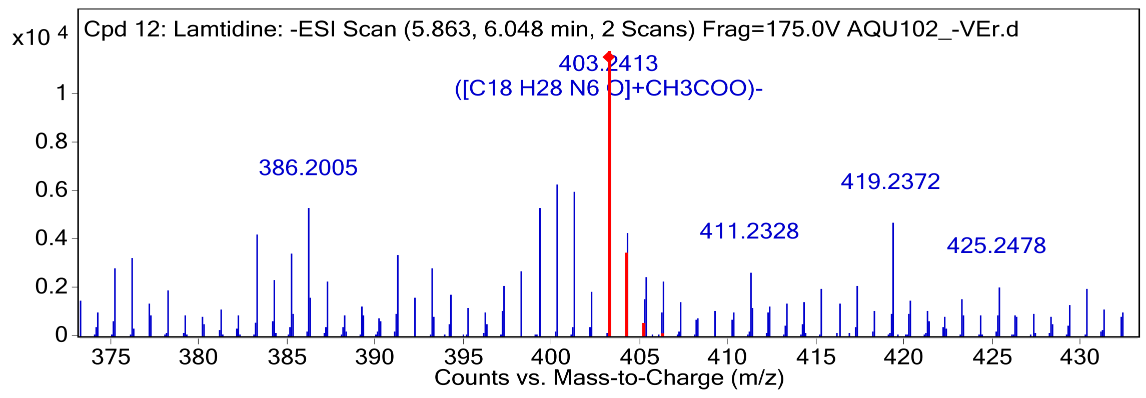

MS Spectrum Peak List

m/z	*Calc m/z*	Diff(ppm)	z	Abund	Formula	Ion
160.8459				17820.11		
162.8429				15902.75		
197.8126				17221.19		
243.1403				15786.28		
243.1769			1	31407.18		
257.1559			1	17221.33		
403.2413	403.2463	12.32	1	11742.88	C18 H28 N6 O	(M+CH3COO)-
404.2449	404.2491	10.43	1	3090.73	C18 H28 N6 O	(M+CH3COO)-
405.2331	405.2517	45.9	1	2463.07	C18 H28 N6 O	(M+CH3COO)-
406.24	406.2542	34.97	1	668.12	C18 H28 N6 O	(M+CH3COO)-

MSMS Spectrum

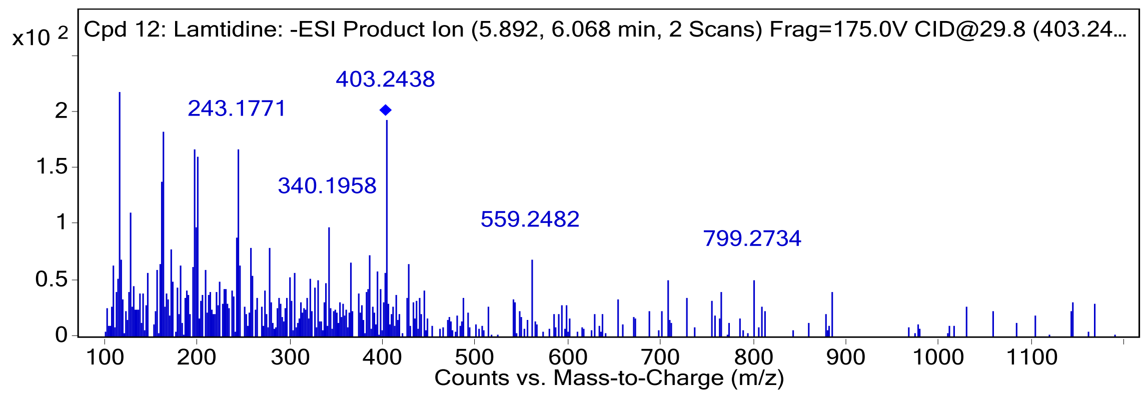

MS/MS Spectrum Peak List

m/z	Abund
115.043	218.83
126.907	110.95
160.8449	138.74
162.842	182.81
195.8145	166.68
197.8124	97.94
199.1873	160.91
243.1771	167.64
340.1958	98.61
403.2438	193.67

Compound Structure

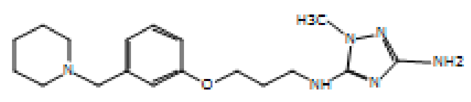

Compound Label	Name	*m/z*	RT	Algorithm	Mass
Cpd 13: Momilactone B	**Momilactone B**	329.1791	6.116	Auto MS/MS	330.1865

MS Spectrum

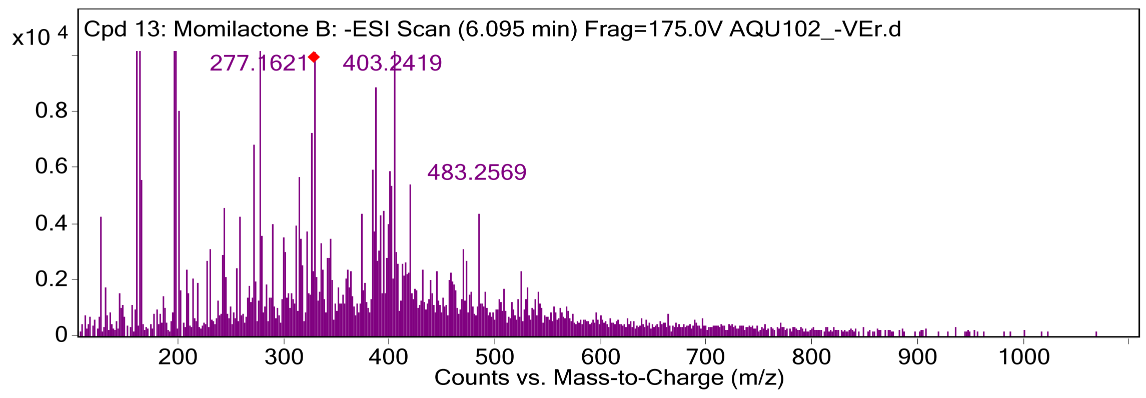

MS Zoomed Spectrum

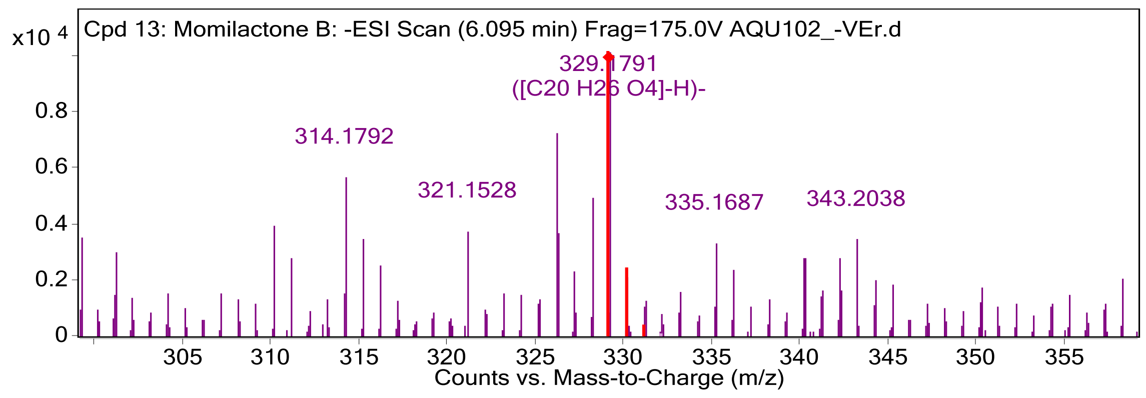

MS Spectrum Peak List

m/z	*Calc m/z*	Diff(ppm)	z	Abund	Formula	Ion
160.8459				15466.22		
162.8428				15004.59		
195.8156				14910.81		
197.8124				17273.34		
277.1621			1	20181.12		
329.1791	329.1758	-10.01	1	10162.01	C20 H26 O4	(M-H)-
330.1808	330.1792	-4.75	1	2164.39	C20 H26 O4	(M-H)-
331.1885	331.182	-19.77	1	1332.86	C20 H26 O4	(M-H)-
386.2007			1	8886.49		
403.2419			1	16165.31		

MSMS Spectrum

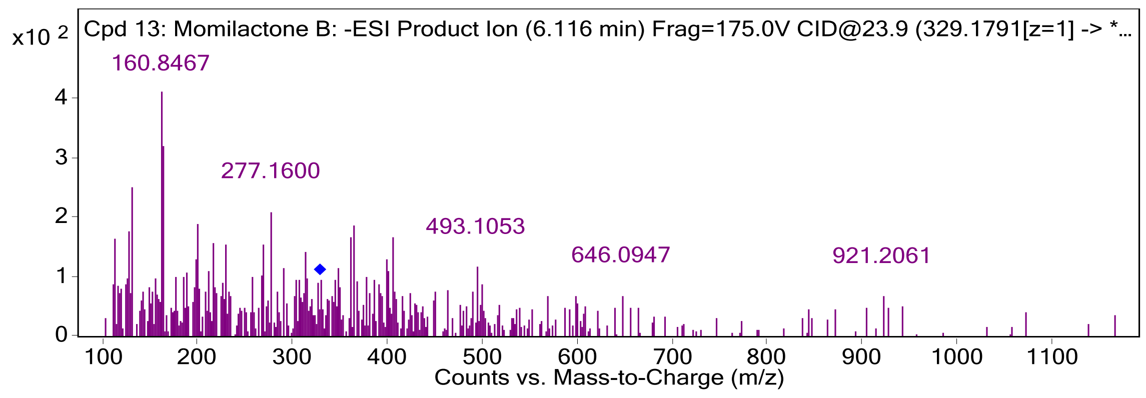

MS/MS Spectrum Peak List

m/z	Abund
111.8644	165.56
126.9071	179.13
130.0893	253.18
160.8467	414.67
162.8433	321.83
199.8092	189.62
277.16	211.65
360.17	168.42
363.07	189.39
404.245	167.75

Compound Structure

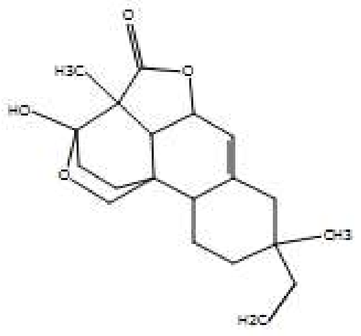

Compound Label	Name	*m/z*	RT	Algorithm	Mass
Cpd 14: Sethoxydim	**Sethoxydim**	386.2013	6.242	Auto MS/MS	327.1867

MS Spectrum

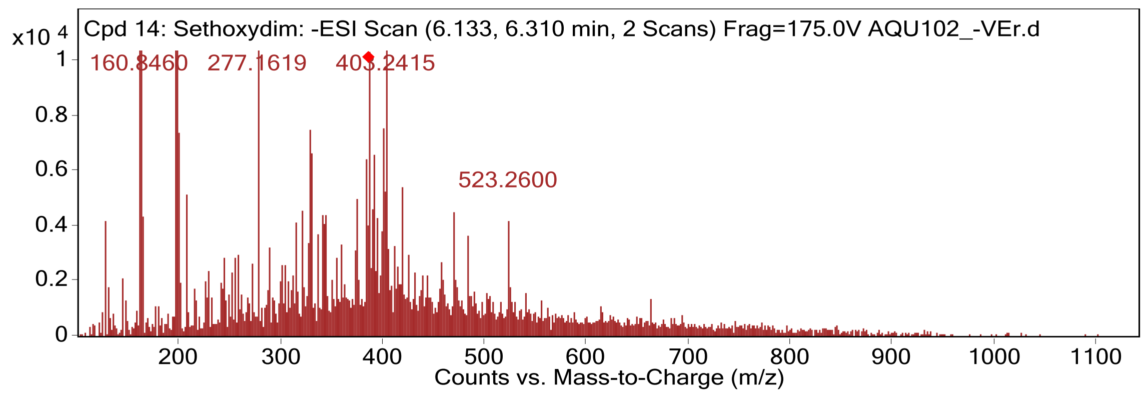

MS Zoomed Spectrum

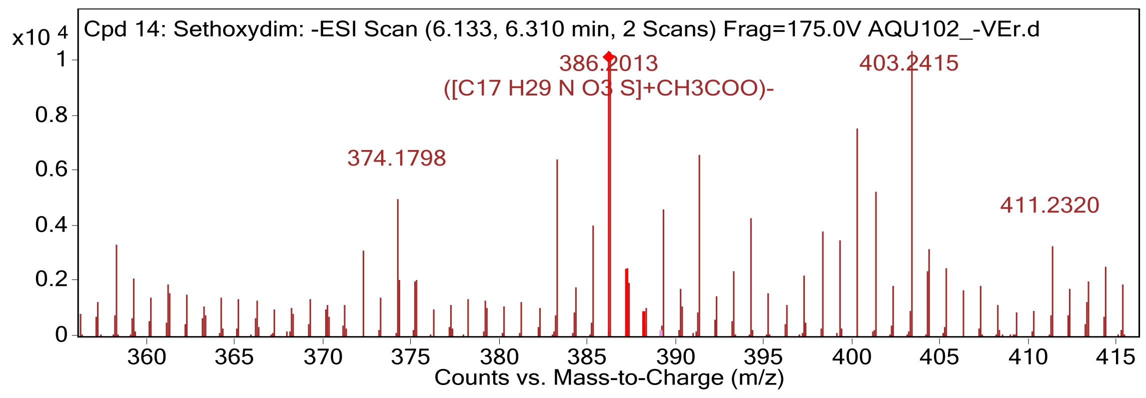

MS Spectrum Peak List

m/z	*Calc m/z*	Diff(ppm)	z	Abund	Formula	Ion
160.846				17104.66		
162.8428				14683.33		
195.8156				13916.2		
197.8127				16223.06		
277.1619			1	14837		
386.2013	386.2007	-1.51	1	10333.79	C17 H29 N O3 S	(M+CH3COO)-
387.2027	387.2038	2.95	1	2493.73	C17 H29 N O3 S	(M+CH3COO)-
388.1964	388.2007	11.05	1	1053.72	C17 H29 N O3 S	(M+CH3COO)-

400.2165				7564.19		
403.2415			1	15866.98		

MSMS Spectrum

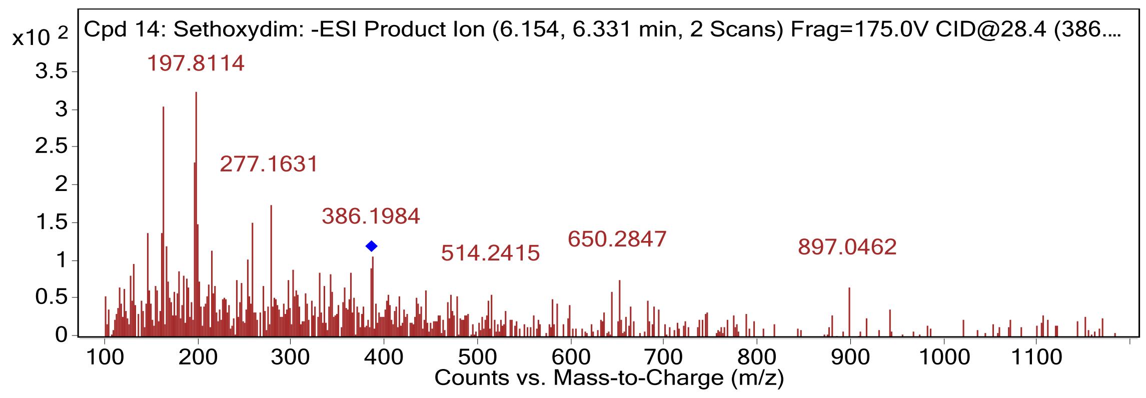

MS/MS Spectrum Peak List

m/z	Abund
146.0487	137.59
160.8444	138.1
162.8435	305.05
164.839	120.35
195.8154	231.52
197.8114	325.08
199.8092	149.36
214.1164	115.01
257.1557	151.98
277.1631	175.31

Compound Structure

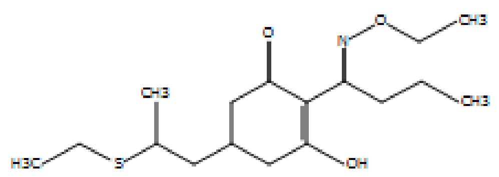

Compound Label	Name	*m/z*	RT	Algorithm	Mass
Cpd 15: Lamtidine	**Lamtidine**	403.2413	6.329	Auto MS/MS	344.226

MS Spectrum

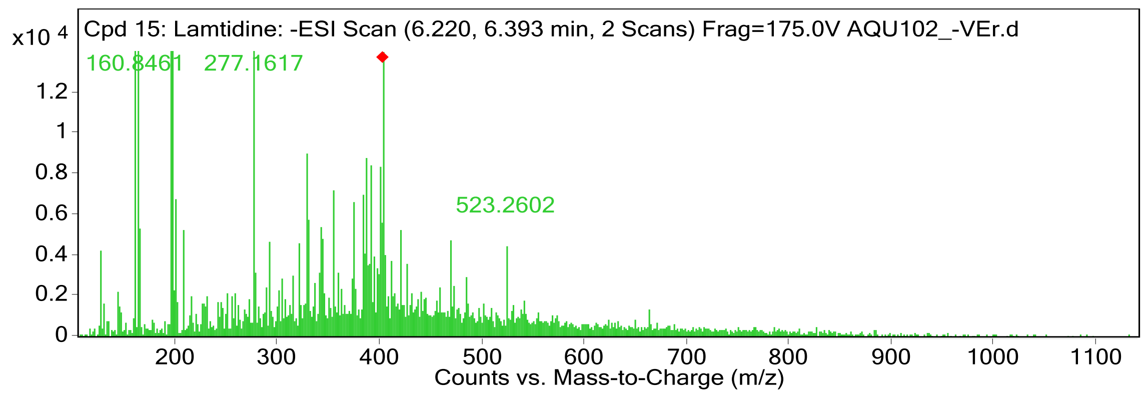

MS Zoomed Spectrum

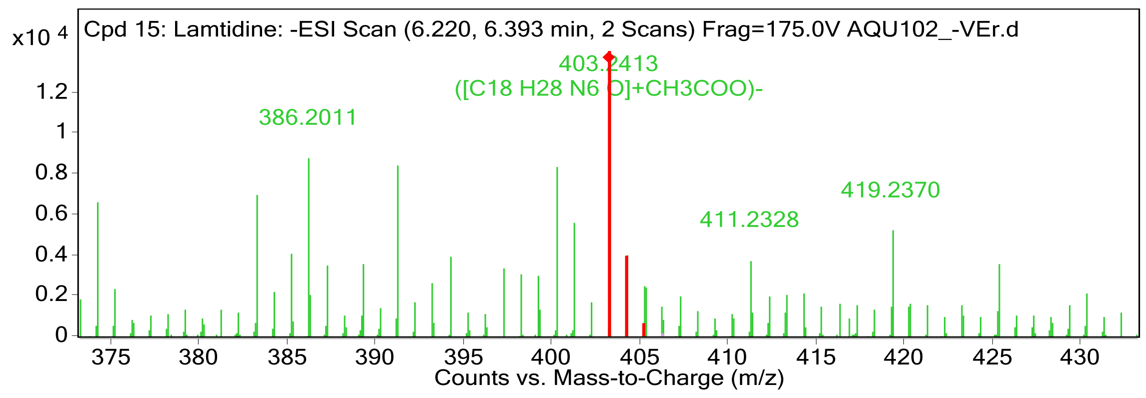

MS Spectrum Peak List

m/z	*Calc m/z*	Diff(ppm)	z	Abund	Formula	Ion
160.8461				16245.04		
162.8429				14283.99		
195.8155				14344.65		
197.8126				15276.1		
277.1617			1	18004.92		
328.1945				9009.24		
386.2011			1	8807.13		
403.2413	403.2463	12.43	1	13988.74	C18 H28 N6 O	(M+CH3COO)-
404.2446	404.2491	11.36	1	4038.09	C18 H28 N6 O	(M+CH3COO)-
405.2341	405.2517	43.44	1	2440.24	C18 H28 N6 O	(M+CH3COO)-

MSMS Spectrum

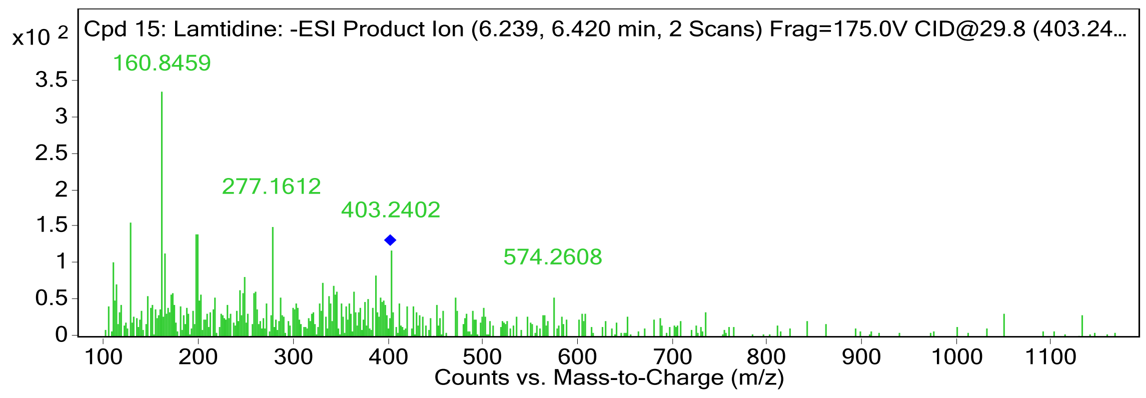

MS/MS Spectrum Peak List

m/z	z	Abund
109.0685		103.49
126.9079		157.11
160.8459		336.65
162.8414		115.32
195.8146		141.47
197.8128		140.65

246.9827		81.95
277.1612		150.44
386.1985		85.2
403.2402	1	118.25

Compound Structure

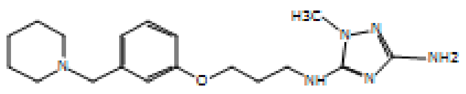

Compound Label	*m/z*	RT	Algorithm
Compound 16	187.1015	6.528	Auto MS/MS

MS Spectrum

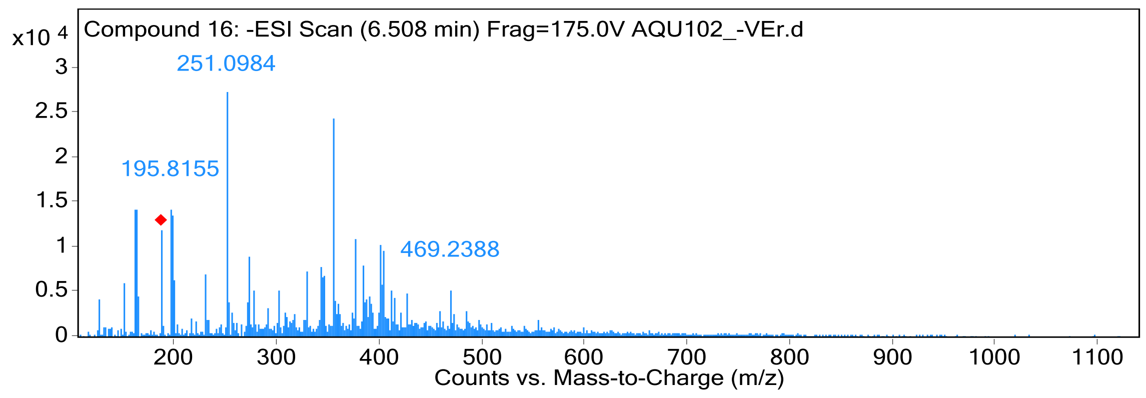

MS Zoomed Spectrum

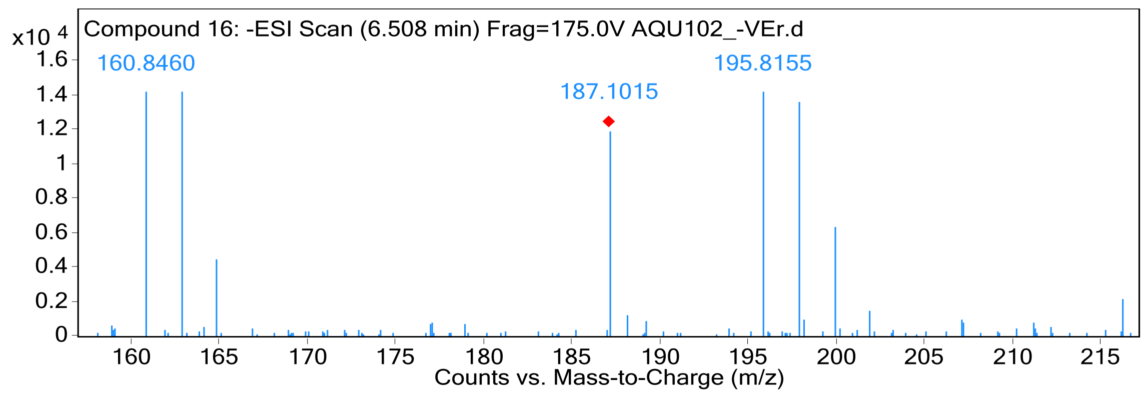

MS Spectrum Peak List

m/z	z	Abund
160.846		14272.33
162.843		14219.37
187.1015		11901.01
188.1055	1	1269.69
189.0969	1	955.19
195.8155		14278.81
197.8125		13650.31
251.0984	1	27423.52
354.2102	1	24471.8
375.2207	1	11056.22

MSMS Spectrum

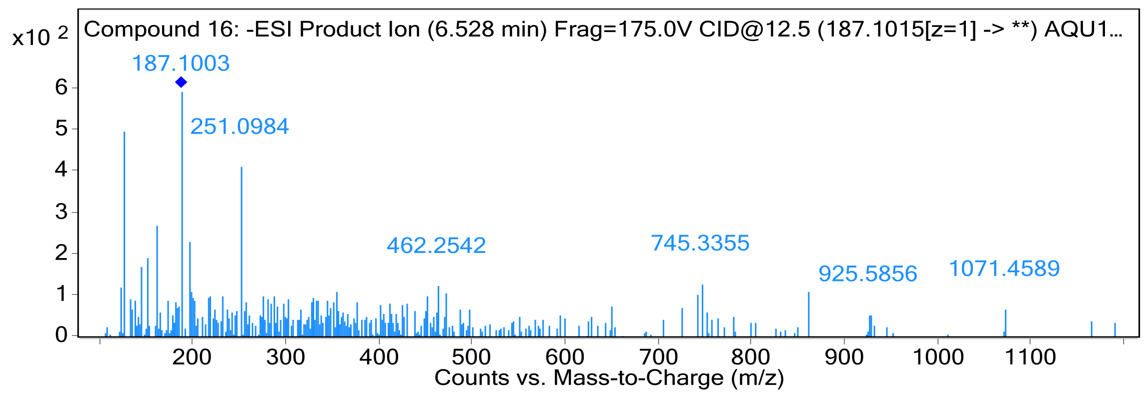

MS/MS Spectrum Peak List

m/z	z	Abund
122.6864		121.24
125.101		497.08
143.8626		170
151.042		192.18
160.846		270.98
187.1003		595.04
195.8154		231.67
251.0984	1	412
462.2542		123
745.3355		129.09

Compound Label	Name	*m/z*	RT	Algorithm	Mass
Cpd 17: Spirasine I	**Spirasine I**	354.2104	6.54	Auto MS/MS	355.2181

MS Spectrum

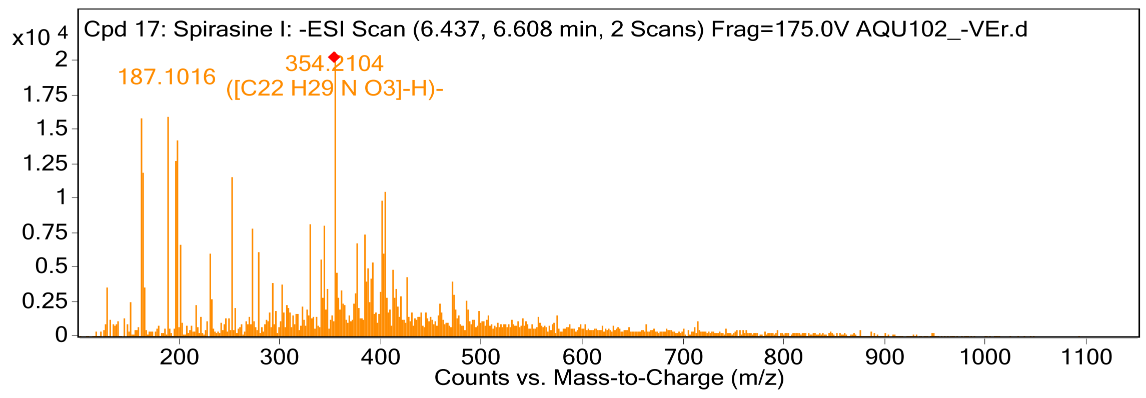

MS Zoomed Spectrum

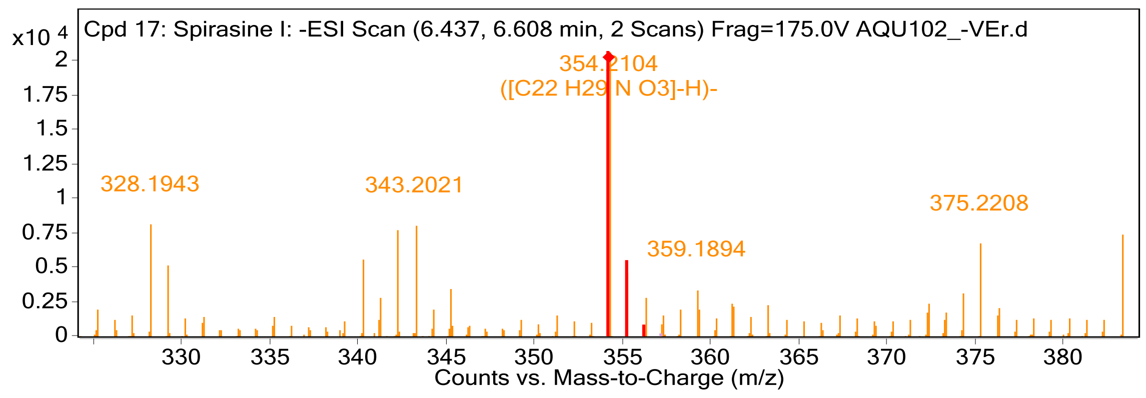

MS Spectrum Peak List

m/z	*Calc m/z*	Diff(ppm)	z	Abund	Formula	Ion
160.8462				15896.36		
162.843				11919.1		
187.1016				15945.35		
195.8156				12745.67		
197.8126				14228.74		
251.0983				11591.39		
354.2104	354.2075	-8.41	1	20664.23	C22 H29 N O3	(M-H)-
355.2125	355.2108	-4.97	1	4673.5	C22 H29 N O3	(M-H)-
356.2219	356.2137	-23.11	1	2889	C22 H29 N O3	(M-H)-
403.2406			1	10564.04		

MSMS Spectrum

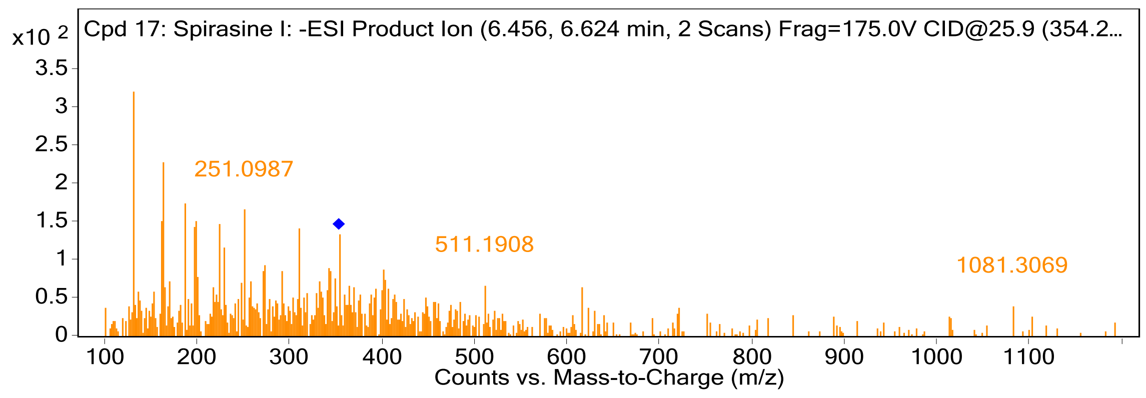

MS/MS Spectrum Peak List

m/z	Abund
130.0921	321.08
160.8452	151.47
162.8427	227.96
187.1003	174.3
195.8143	144.35
197.8122	151.05
223.1113	147.87
251.0987	166.24
310.2183	141.75
354.2117	134.6

Compound Structure

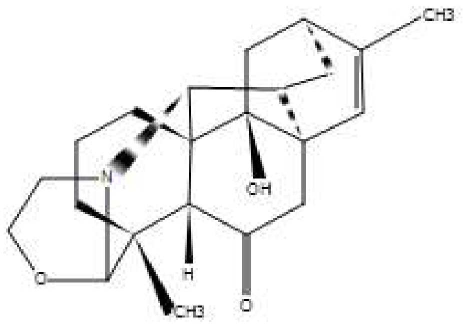

Compound Label	Name	*m/z*	RT	Algorithm	Mass
Cpd 18: Spirasine I	**Spirasine I**	354.2105	6.791	Auto MS/MS	355.2175

MS Spectrum

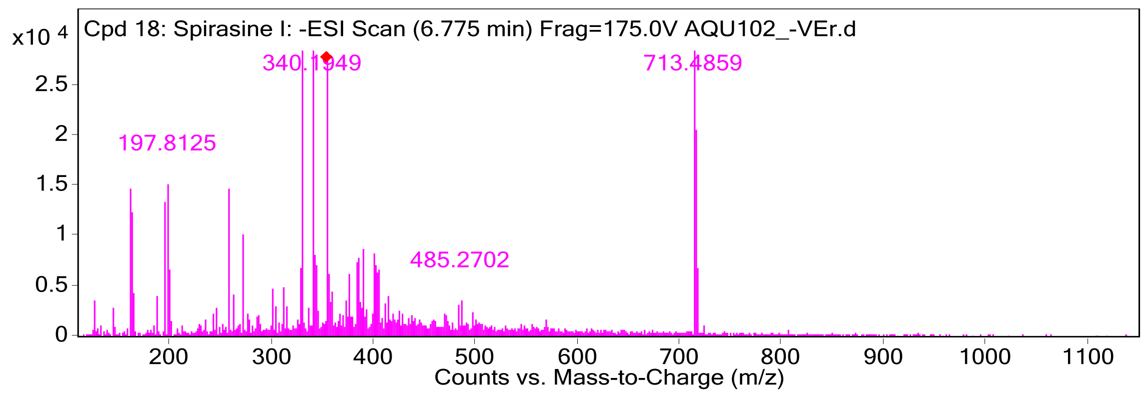

MS Zoomed Spectrum

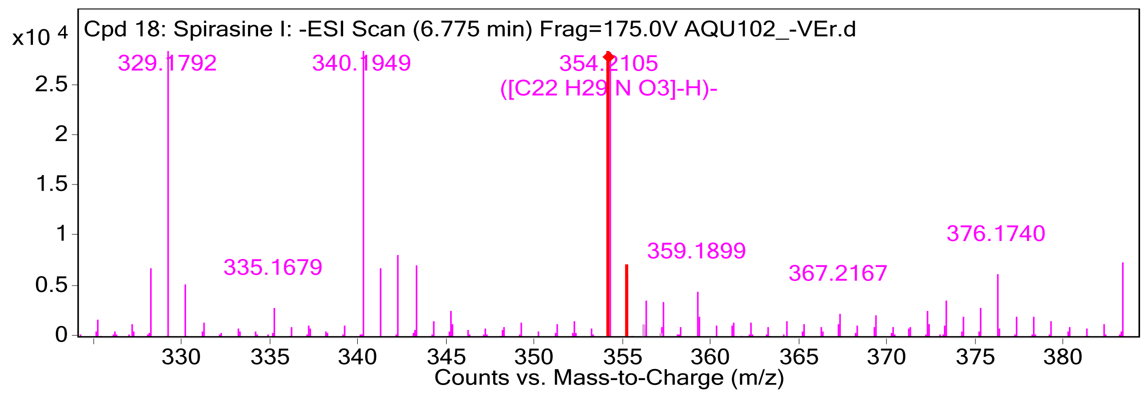

MS Spectrum Peak List

m/z	*Calc m/z*	Diff(ppm)	z	Abund	Formula	Ion
160.8462				14772.97		
197.8125				15219.92		
257.1558			1	14688.64		
329.1792			1	28692.08		
340.1949			1	31195.93		
354.2105	354.2075	-8.44	1	28290.12	C22 H29 N O3	(M-H)-
355.2127	355.2108	-5.43	1	6335.75	C22 H29 N O3	(M-H)-
713.4859			1	50881.84		

714.4889			1	20508.12		
715.4847			1	17931.87		

MSMS Spectrum

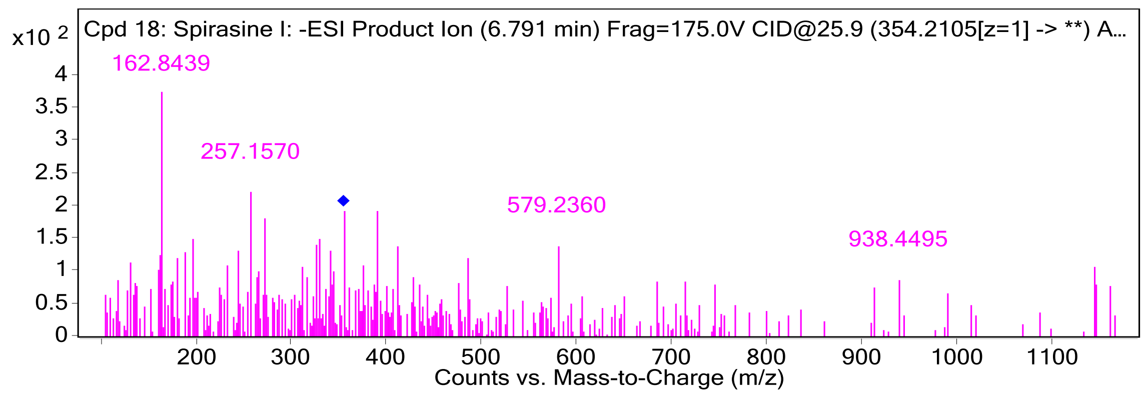

MS/MS Spectrum Peak List

m/z	Abund
162.8439	375.97
195.8153	150.48
257.157	221.9
271.173	182.02
325.1793	140.94
329.1737	150.33
354.2095	193.91
389.1955	193.45
411.1075	139.06
579.236	139.79

Compound Structure

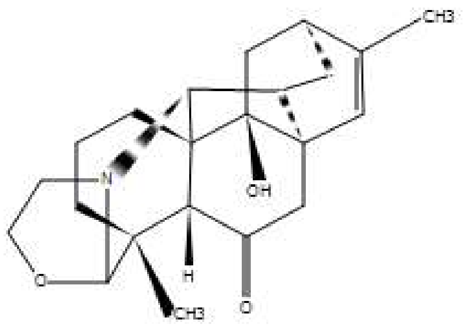

Compound Label	Name	*m/z*	RT	Algorithm	Mass
Cpd 19: Momilactone B	**Momilactone B**	329.1791	6.797	Auto MS/MS	330.1866

MS Spectrum

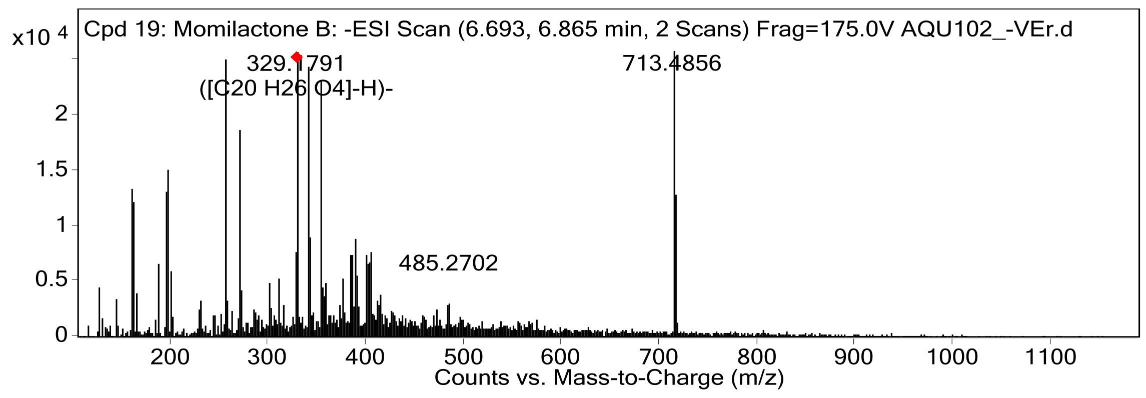

MS Zoomed Spectrum

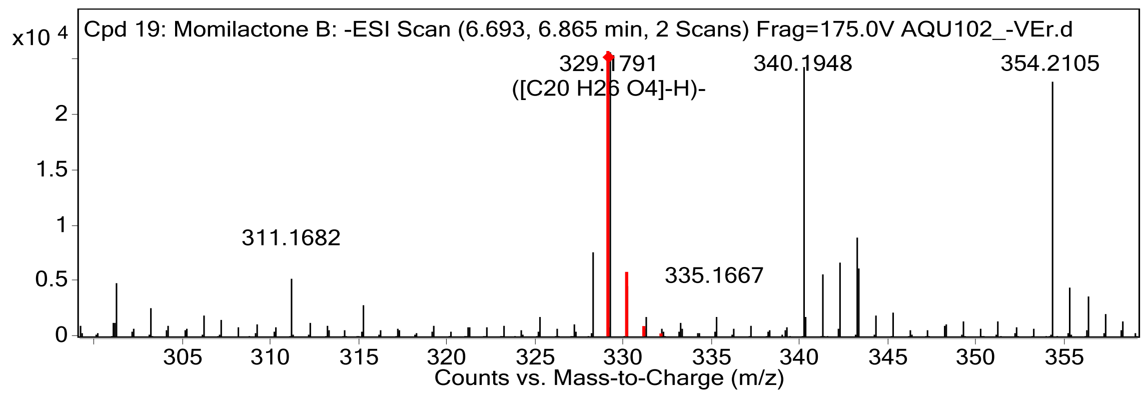

MS Spectrum Peak List

m/z	*Calc m/z*	Diff(ppm)	z	Abund	Formula	Ion
197.8126				15147.72		
257.1559			1	25127.01		
271.1723			1	18666.45		
329.1791	329.1758	-9.87	1	25726.86	C20 H26 O4	(M-H)-
330.1821	330.1792	-8.57	1	4670.98	C20 H26 O4	(M-H)-
331.1907	331.182	-26.24	1	1859.98	C20 H26 O4	(M-H)-
332.1884	332.1847	-11.37	1	477.49	C20 H26 O4	(M-H)-
340.1948			1	24453.46		
354.2105			1	23033.71		
713.4856			1	38156.31		

MSMS Spectrum

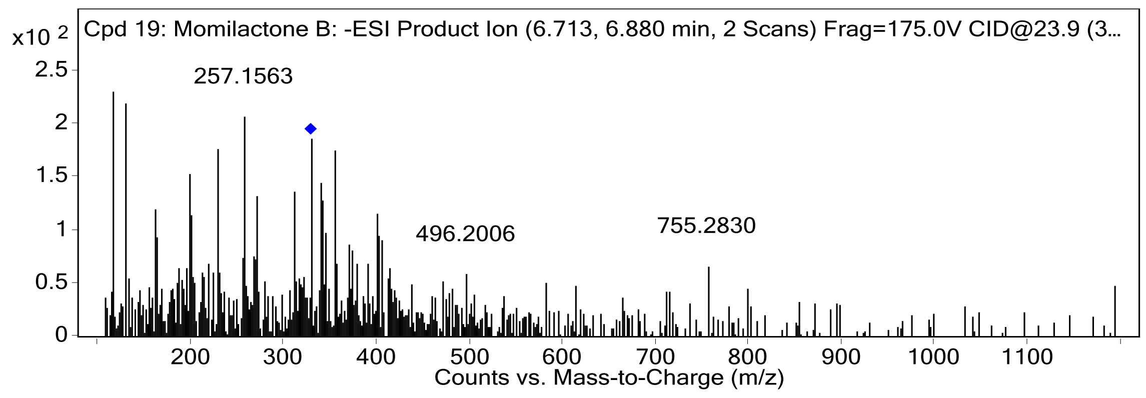

MS/MS Spectrum Peak List

m/z	z	Abund
116.0742		231.18
130.0918		219.36
197.8134		152.99
229.1619		177.01
257.1563	1	207.3
271.1706	1	132.12

311.1679	1	137.24
329.1782	1	185.87
340.1936		145.59
354.2081		174.92

Compound Structure

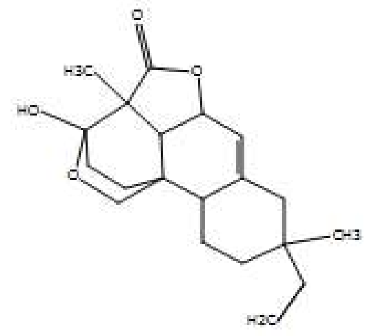

Compound Label	Name	*m/z*	RT	Algorithm	Mass
Cpd 20: Eseramine	**Eseramine**	363.1639	7.211	Auto MS/MS	318.1664

MS Spectrum

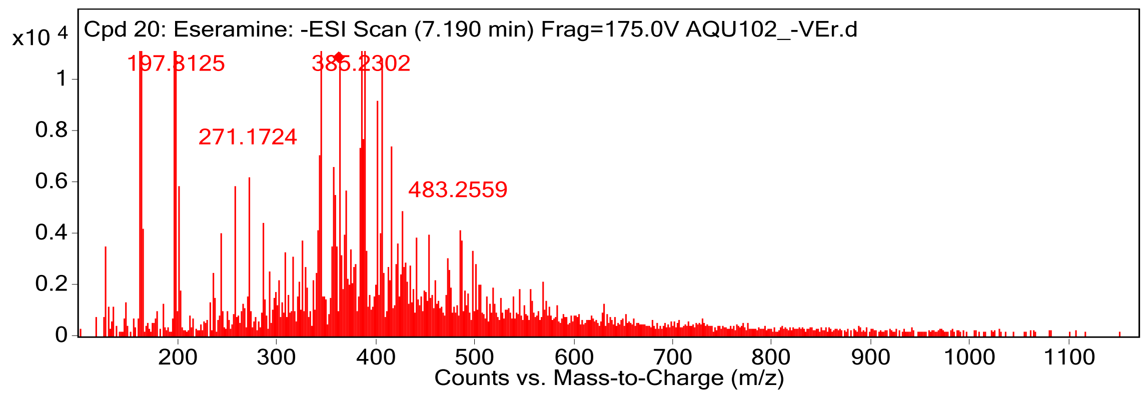

MS Zoomed Spectrum

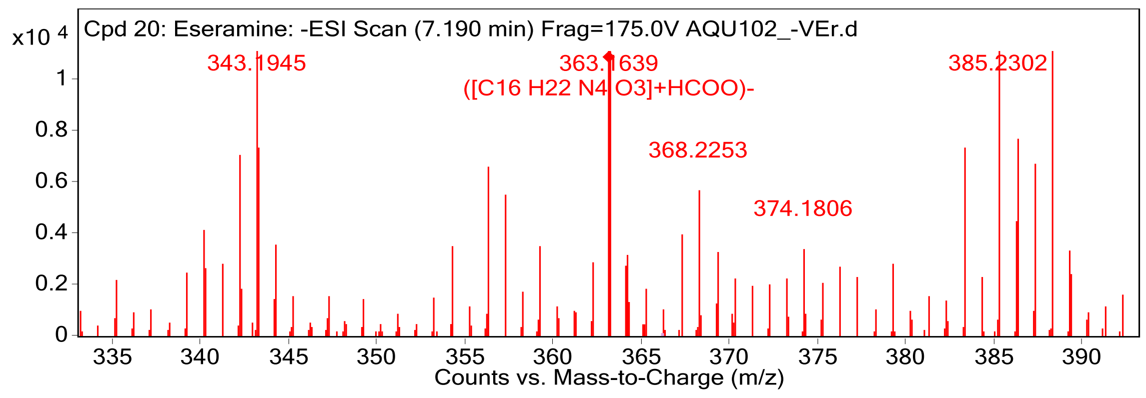

MS Spectrum Peak List

m/z	*Calc m/z*	Diff(ppm)	z	Abund	Formula	Ion
160.8464				14317.29		
162.843				13314.49		
195.8154				11955.61		
197.8125				15338.48		
343.1945			1	14855.01		
363.1639	363.1674	9.51	1	11111.77	C16 H22 N4 O3	(M+HCOO)-
364.1662	364.1703	11.28	1	3220.48	C16 H22 N4 O3	(M+HCOO)-
365.1762	365.1727	-9.47	1	1898.9	C16 H22 N4 O3	(M+HCOO)-
385.2302			1	17052.89		
388.196			1	12876.34		

MSMS Spectrum

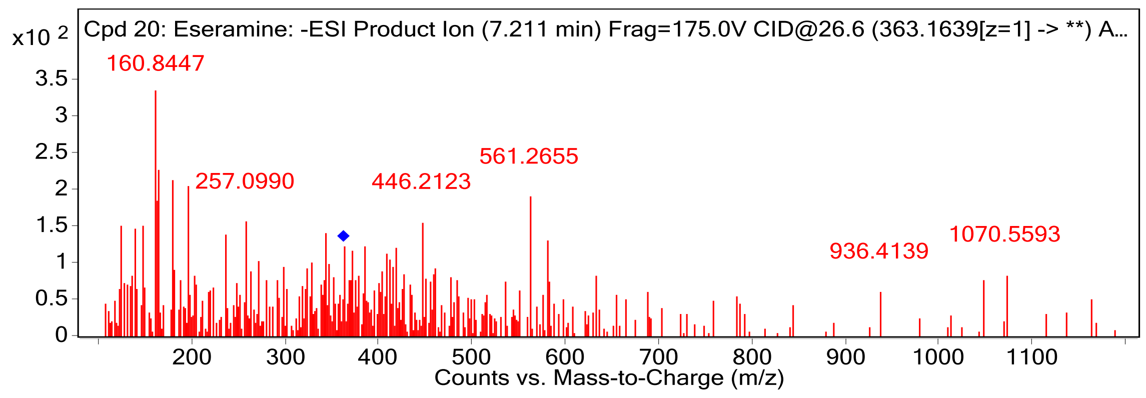

MS/MS Spectrum Peak List

m/z	Abund
123.2116	152.93
160.8447	334.9
162.0721	185.11
162.8429	195.9
164.0741	227.72
179.0859	214.59
195.8153	205.62
257.099	157.01
446.2123	156.56
561.2655	191.03

Compound Structure

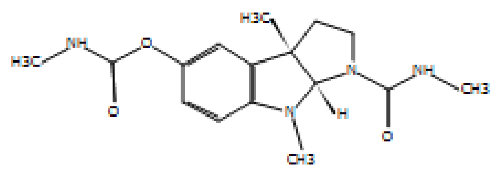

Compound Label	Name	*m/z*	RT	Algorithm	Mass
Cpd 21: 2-Deoxystreptidine	**2-Deoxystreptidine**	291.1413	7.264	Auto MS/MS	246.1434

MS Spectrum

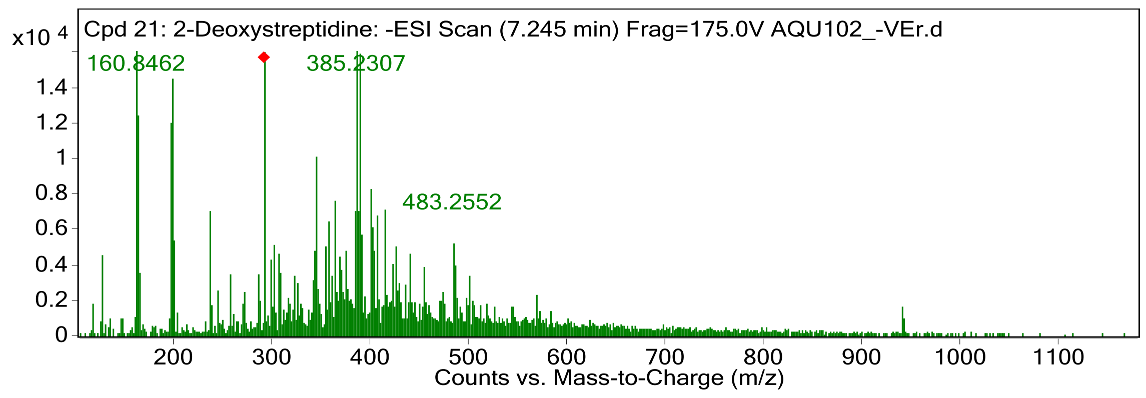

MS Zoomed Spectrum

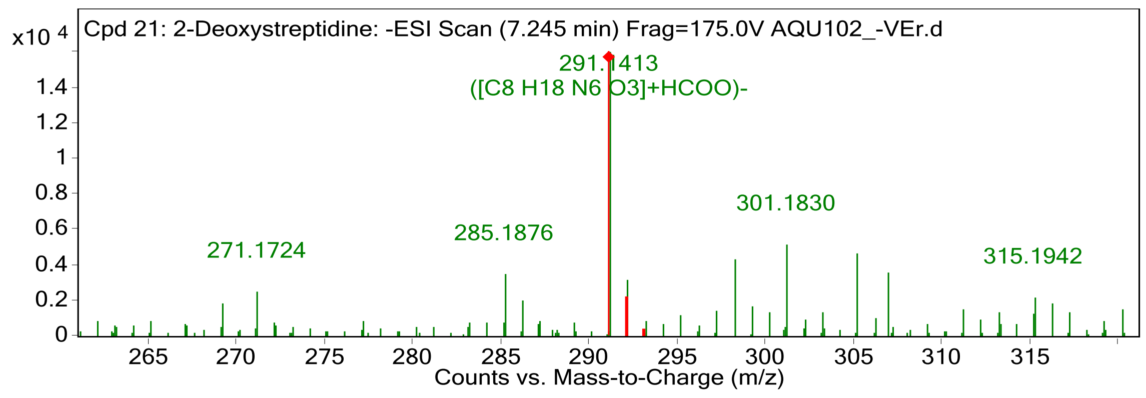

MS Spectrum Peak List

m/z	*Calc m/z*	Diff(ppm)	z	Abund	Formula	Ion
160.8462				16128.78		
162.8429				12475.88		
195.8155				12078.95		
197.8126				14513.79		
291.1413	291.1422	3.2	1	16033.38	C8 H18 N6 O3	(M+HCOO)-
292.1447	292.1445	-0.54	1	3245.32	C8 H18 N6 O3	(M+HCOO)-
293.1478	293.1466	-4.21	1	885.93	C8 H18 N6 O3	(M+HCOO)-
343.1943			1	10139.75		
385.2307			1	17761.9		
388.1953			1	15945.78		

MSMS Spectrum

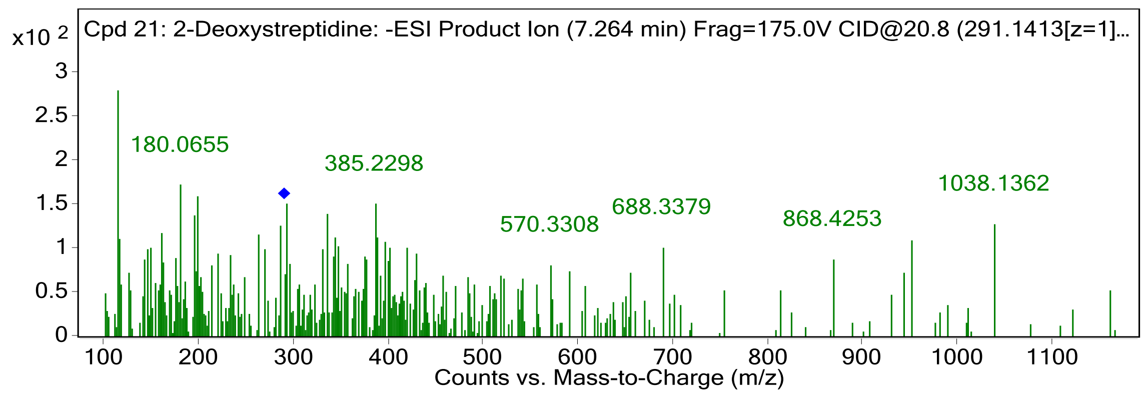

MS/MS Spectrum Peak List

m/z	z	Abund
115.09		279.06
180.0655		172.41
180.0844		130.38
195.815		137.49
197.8131		160.11
285.1916		127.35
292.1438		151.04
335.177		140.26
385.2298	1	151
1038.1362		128.35

Compound Structure

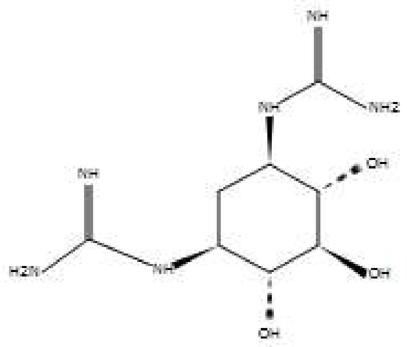

Compound Label	Name	*m/z*	RT	Algorithm	Mass
Cpd 22: 16-Methyl-epi-nigakihemiacetal B	**16-Methyl-epi-nigakihemiacetal B**	403.2167	7.554	Auto MS/MS	404.2233

MS Spectrum

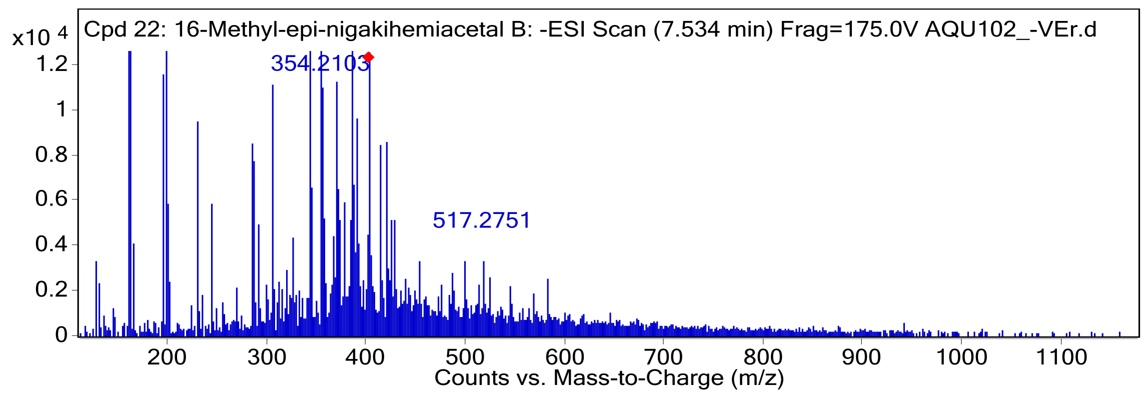

MS Zoomed Spectrum

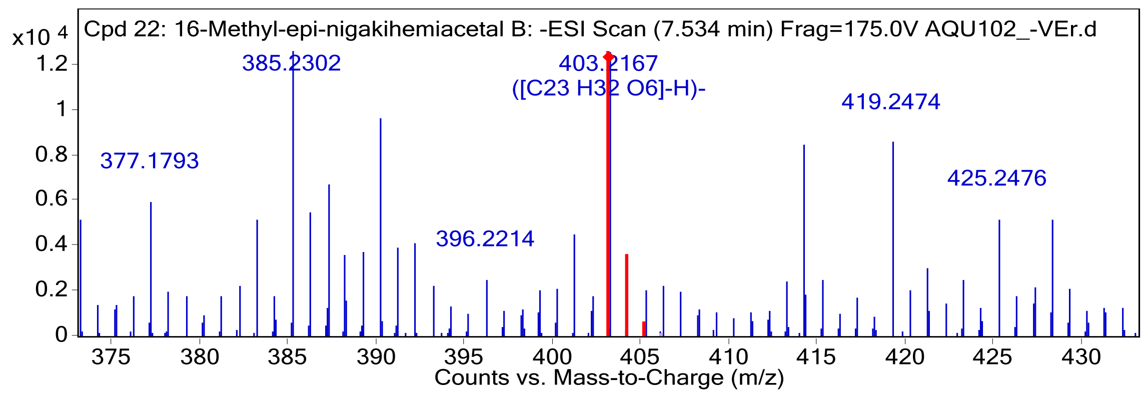

MS Spectrum Peak List

m/z	*Calc m/z*	Diff(ppm)	z	Abund	Formula	Ion
160.846				15627.37		
162.8431				13633.07		
195.8155				11620.14		
197.8127				14034.77		
343.1942			1	16119.64		
354.2103			1	41680.09		
385.2302			1	15760.28		
403.2167	403.2126	-10.13	1	12569.21	C23 H32 O6	(M-H)-
404.2211	404.216	-12.63	1	3630.88	C23 H32 O6	(M-H)-
405.2148	405.2187	9.64	1	2085.45	C23 H32 O6	(M-H)-

MSMS Spectrum

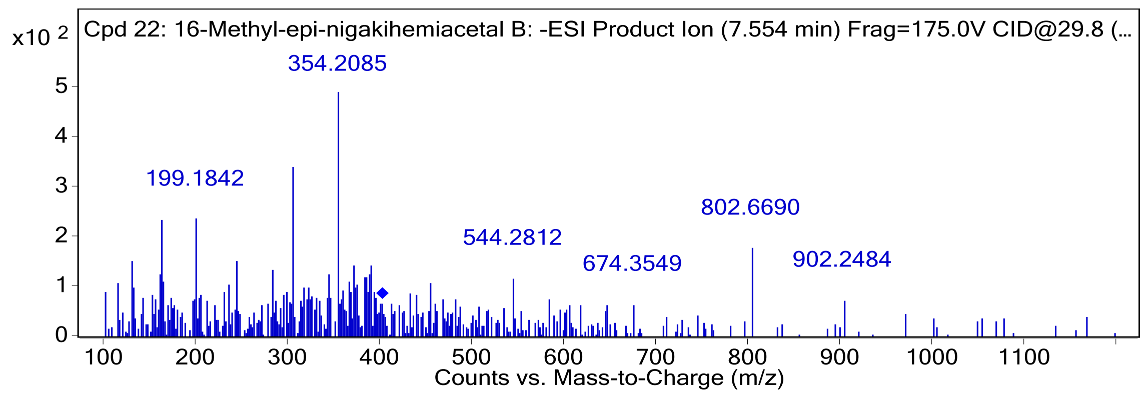

MS/MS Spectrum Peak List

m/z	Abund
131.0388	153.15
162.8412	237.12
199.1842	237.31
243.1739	154.22
282.1177	136.2
305.158	340.43
354.2085	493.1
370.2424	144.1
390.186	143.9
802.669	179.04

Compound Structure

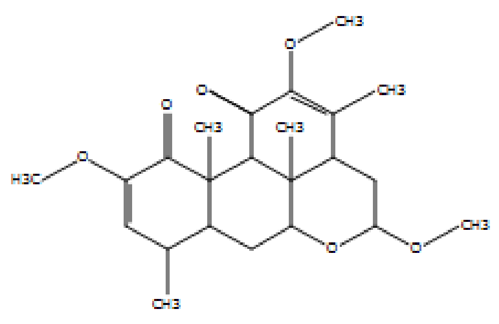

Compound Label	Name	*m/z*	RT	Algorithm	Mass
Cpd 23: Spirasine I	**Spirasine I**	354.2106	7.592	Auto MS/MS	355.2171

MS Spectrum

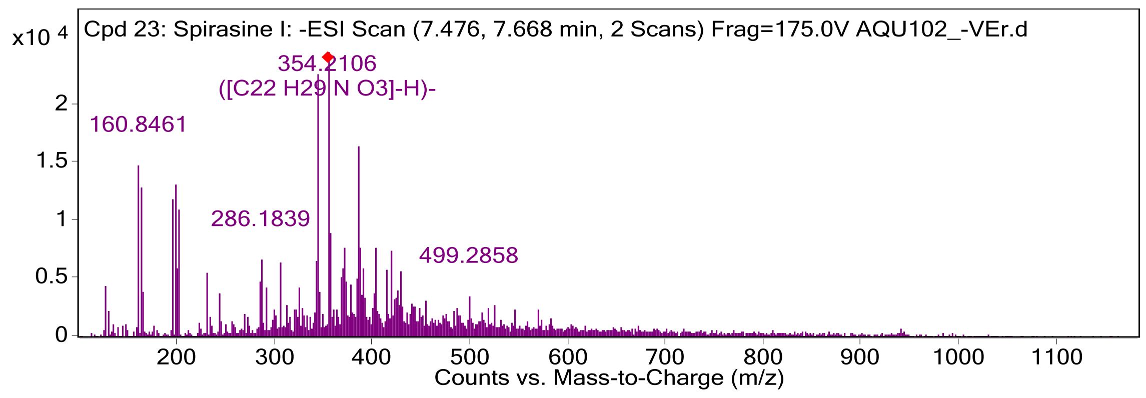

MS Zoomed Spectrum

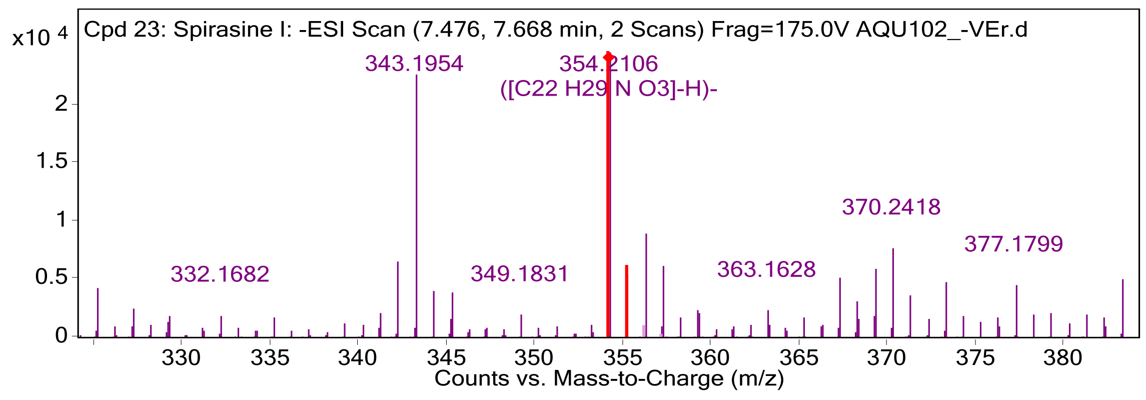

MS Spectrum Peak List

m/z	*Calc m/z*	Diff(ppm)	z	Abund	Formula	Ion
160.8461				14828.78		
162.843				12865.76		
195.8155				11847.22		
197.8127				13119.52		
201.1179				10946.59		
343.1954			1	22617.99		
354.2106	354.2075	-8.74	1	24524.04	C22 H29 N O3	(M-H)-
355.2098	355.2108	2.76	1	5467.18	C22 H29 N O3	(M-H)-
356.2262			1	9038.31		

385.2308			1	16374.04		

MSMS Spectrum

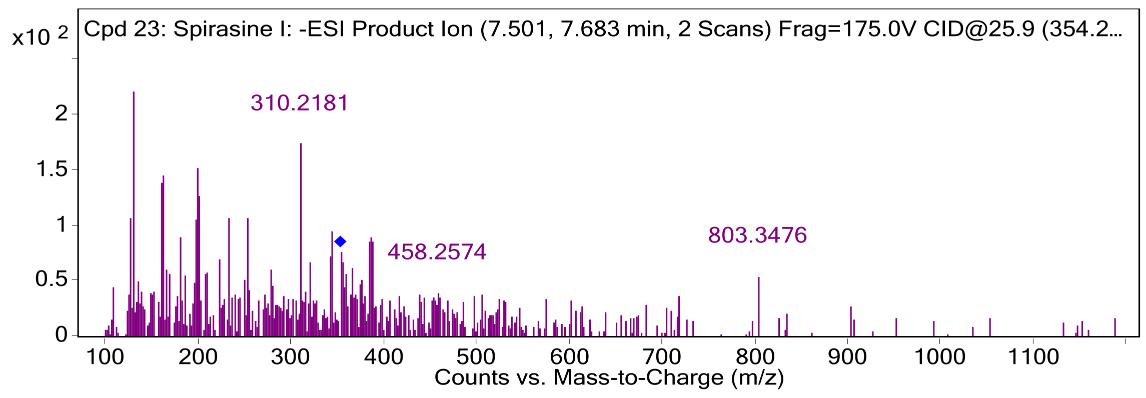

MS/MS Spectrum Peak List

m/z	z	Abund
127.0556		107.86
130.0901		222.05
160.8455		139.84
162.8433		146.48
197.8131		105.67
199.186		152.78
201.1175		127.59
233.136		107.23
253.1508	1	106.93
310.2181		174.73

Compound Structure

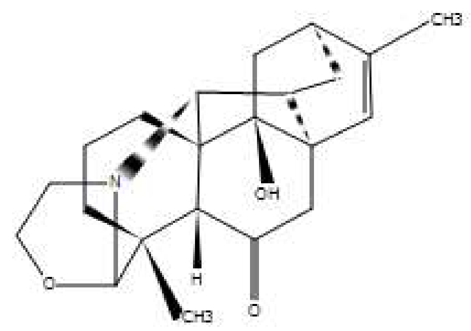

**Compound Label**	**Name**	***m/z***	**RT**	**Algorithm**	**Mass**
Cpd 24: β-estradiol	**β-estradiol**	271.1725	7.822	Auto MS/MS	272.1797

MS Spectrum

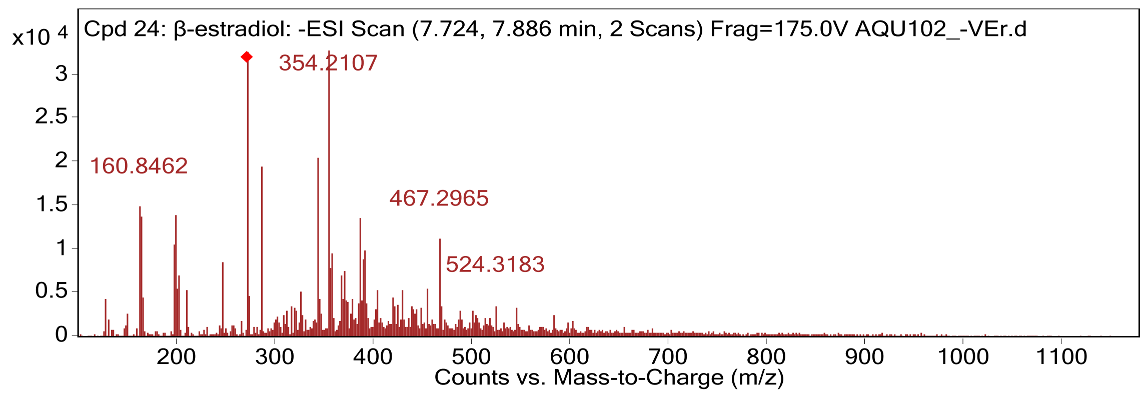

MS Zoomed Spectrum

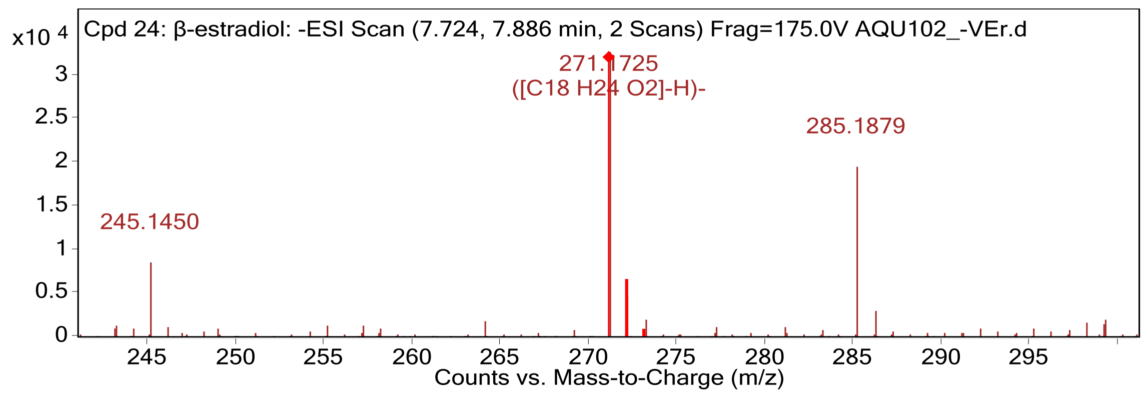

MS Spectrum Peak List

m/z	*Calc m/z*	Diff(ppm)	z	Abund	Formula	Ion
160.8462				14922.85		
162.8429				13848.27		
197.8129				13992.03		
271.1725	271.1704	-8.04	1	32636.94	C18 H24 O2	(M-H)-
272.1757	272.1738	-7.11	1	4772.58	C18 H24 O2	(M-H)-
273.1772	273.1767	-1.98	1	2022.01	C18 H24 O2	(M-H)-
285.1879			1	19540.02		
343.1956			1	20446.43		
354.2107			1	34984.68		
385.2316			1	13668.39		

MSMS Spectrum

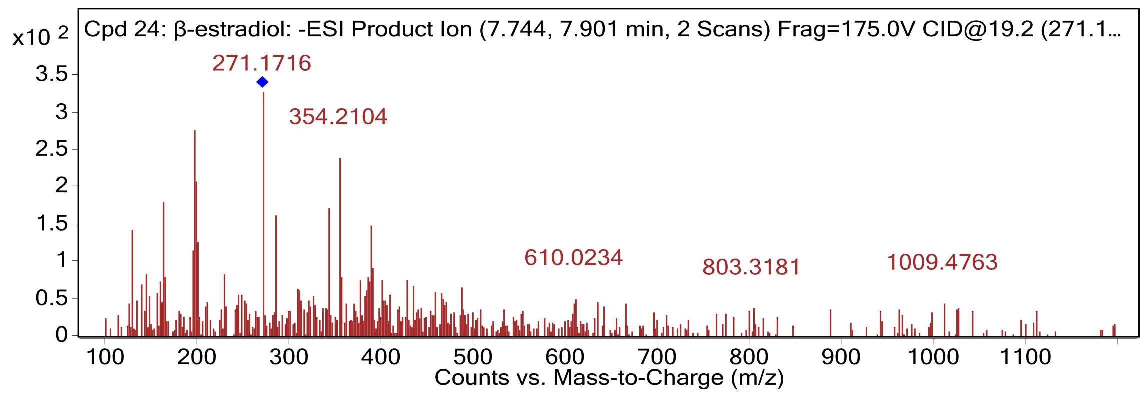

MS/MS Spectrum Peak List

m/z	z	Abund
130.0882		144.43
162.8425		180.54
197.8131		277
199.1861		209.6
201.1182		128.31
271.1716	1	329.37
285.1898		163.19

343.1946		173.77
354.2104	1	241.06
388.1929		149.09

Compound Structure

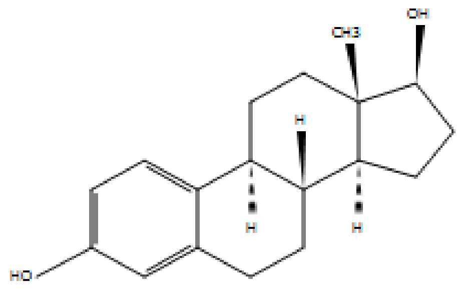

Compound Label	Name	*m/z*	RT	Algorithm	Mass
Cpd 25: Spirasine I	**Spirasine I**	354.2109	7.839	Auto MS/MS	355.2184

MS Spectrum

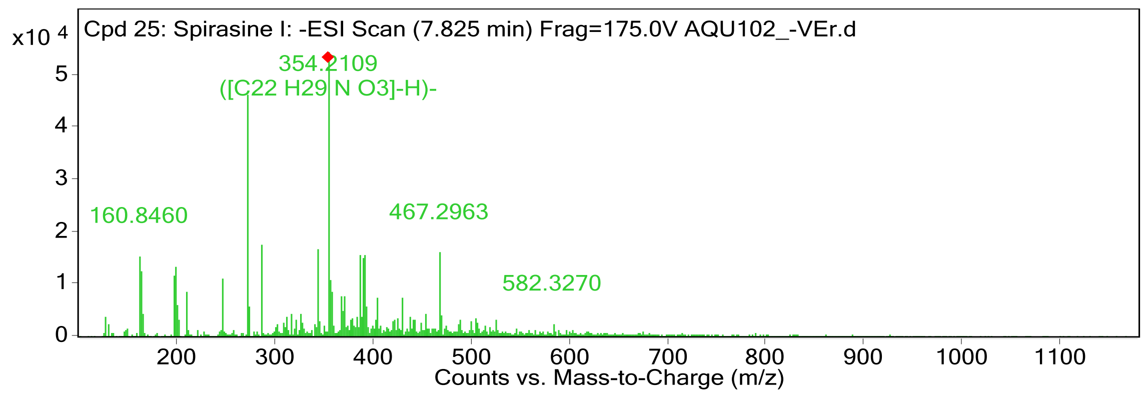

MS Zoomed Spectrum

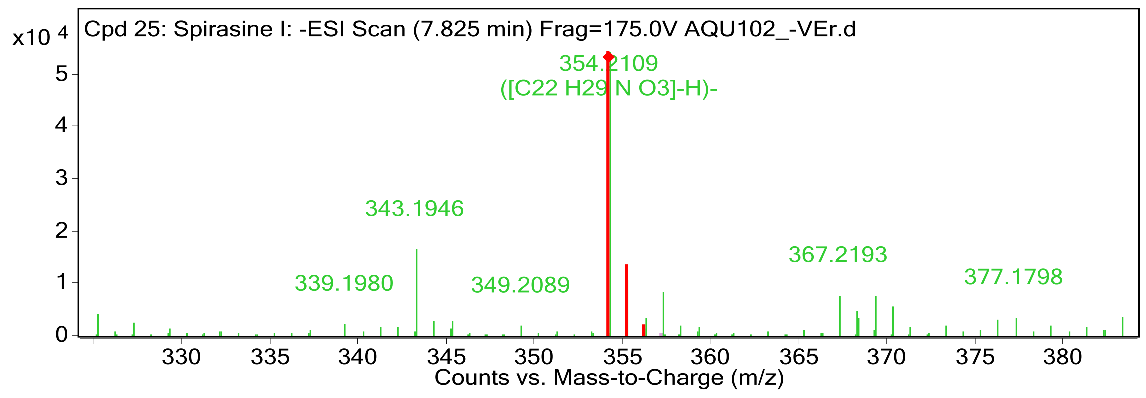

MS Spectrum Peak List

m/z	*Calc m/z*	Diff(ppm)	z	Abund	Formula	Ion
160.846				15454.62		
271.1723			1	46234.56		
285.1877			1	17610.39		
343.1946			1	16755.63		
354.2109	354.2075	-9.66	1	54445.14	C22 H29 N O3	(M-H)-
355.2134	355.2108	-7.44	1	11056.79	C22 H29 N O3	(M-H)-
356.2235	356.2137	-27.57	1	3545.39	C22 H29 N O3	(M-H)-
385.231			1	15677.06		
390.1891			1	15814.57		
467.2963			1	16151.37		

MSMS Spectrum

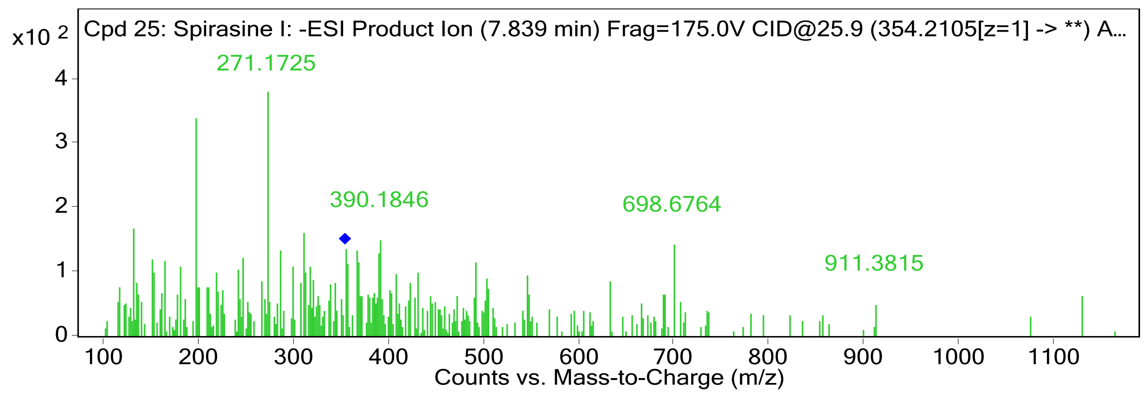

MS/MS Spectrum Peak List

m/z	z	Abund
130.0904		169.02
197.1335	1	339.33
271.1725		382.04
285.186		134.25
310.2182		162.56
354.2087		136.34
366.1888		135.06
388.1934		129.85
390.1846		150.04
698.6764		143

Compound Structure

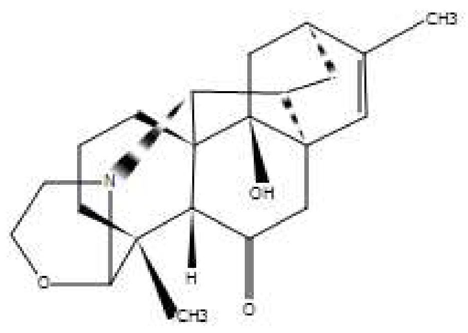

Compound Label	Name	*m/z*	RT	Algorithm	Mass
Cpd 26: 3alpha,7beta,12alpha-Trihydroxy-5alpha-cholan-	**3alpha,7beta,12alpha-Trihydroxy-5alpha-cholan-24-oic Acid**	467.2966	7.886	Auto MS/MS	408.2826

MS Spectrum

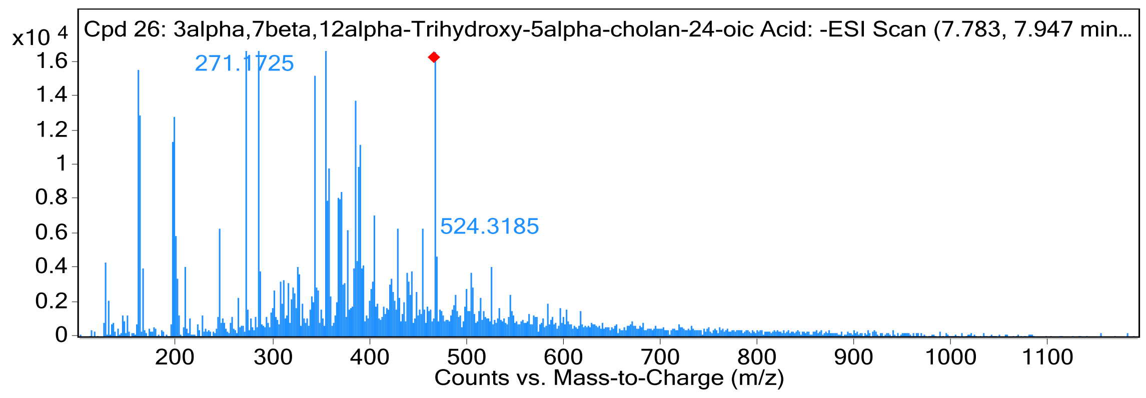
Cpd 26: 3alpha,7beta,12alpha-Trihydroxy-5alpha-cholan-24-oic Acid: -ESI Scan (7.783, 7.947 min...
x10 4
1.6
1.4
1.2
1
0.8
0.6
0.4
0.2
0
271.1725
524.3185
200
300
400
500
600
700
800
900
1000
1100
Counts vs. Mass-to-Charge (m/z)

MS Zoomed Spectrum

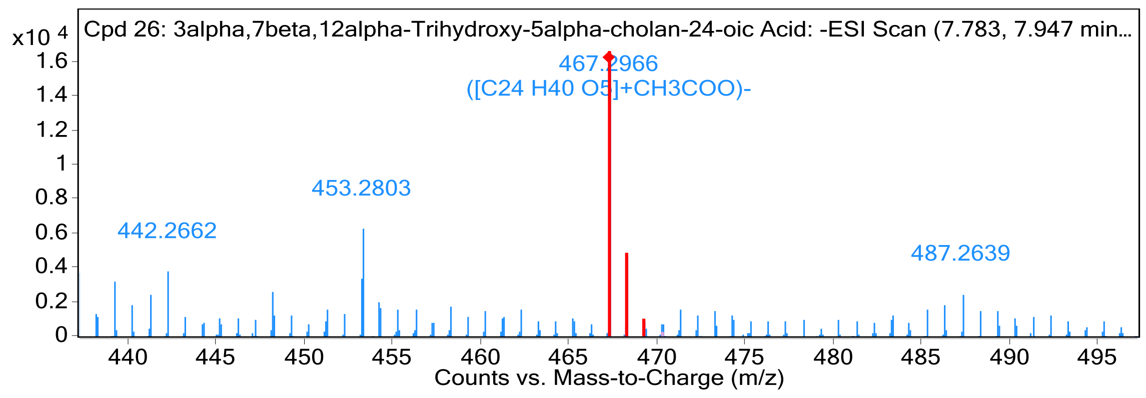

MS Spectrum Peak List

m/z	*Calc m/z*	Diff(ppm)	z	Abund	Formula	Ion
160.8463				15585.45		
162.8431				12877.17		
271.1725			1	41644.57		
285.1879			1	22696.71		
343.1949			1	15205.86		
354.2108			1	38902.89		
385.2318			1	13780.37		
467.2966	467.3014	10.3	1	16582.08	C24 H40 O5	(M+CH3COO)-
468.2995	468.3048	11.48	1	4697.68	C24 H40 O5	(M+CH3COO)-
469.3019	469.3076	12.16	1	493.04	C24 H40 O5	(M+CH3COO)-

MSMS Spectrum

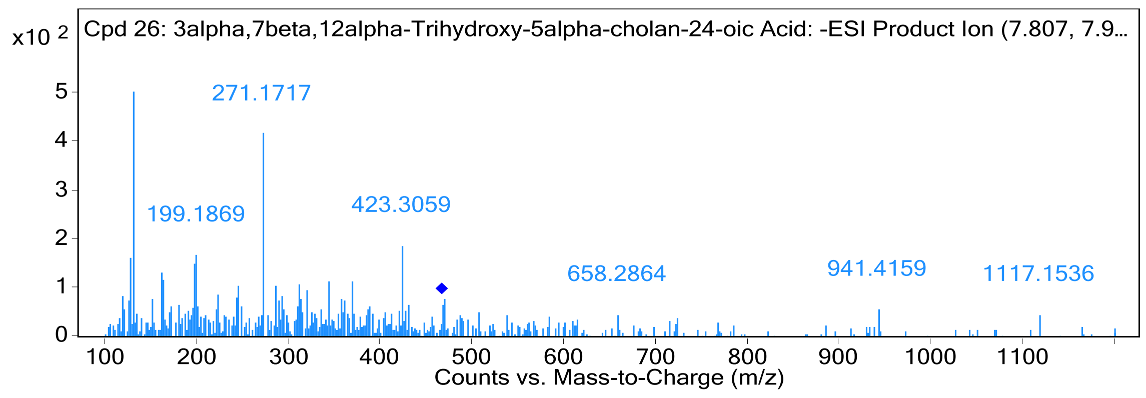

MS/MS Spectrum Peak List

m/z	Abund
127.0542	162.19
130.0901	504.56
160.8467	134.15
162.8432	116.55
197.8128	152.09
199.1869	168.82
271.1717	417.84
343.1938	113.42
369.2321	115
423.3059	188.28

Compound Structure

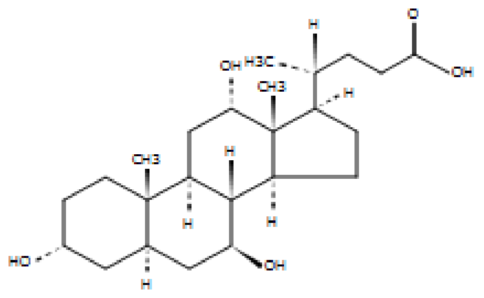

Compound Label	Name	*m/z*	RT	Algorithm	Mass
Cpd 27: β-estradiol	**β-estradiol**	271.1725	8.083	Auto MS/MS	272.1796

MS Spectrum

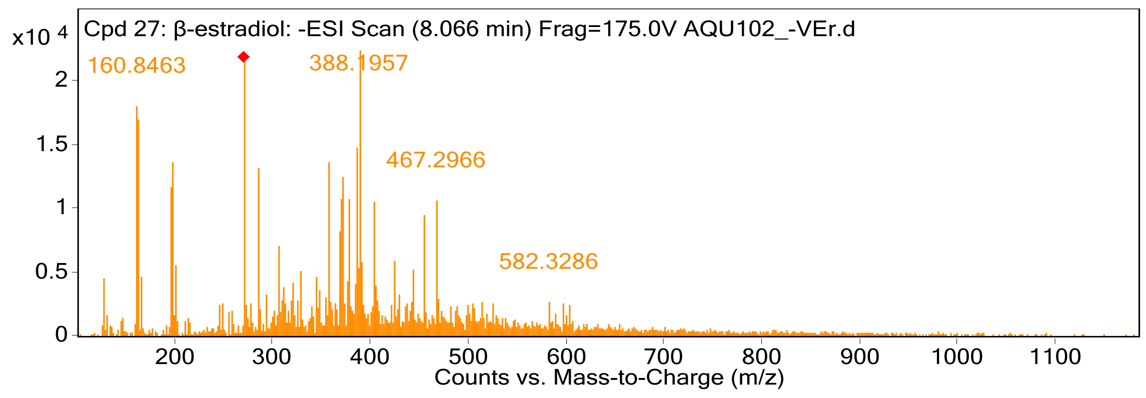

MS Zoomed Spectrum

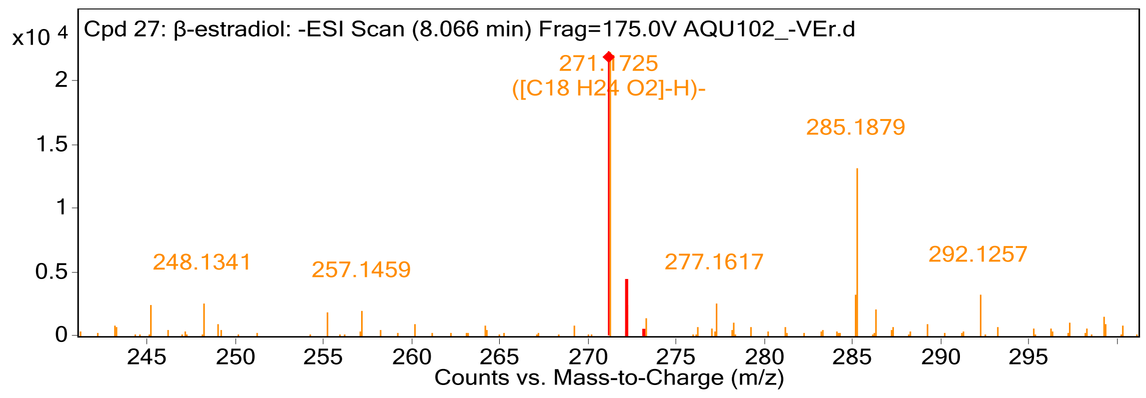

MS Spectrum Peak List

m/z	*Calc m/z*	Diff(ppm)	z	Abund	Formula	Ion
160.8463				18129.1		
162.8435				17100.82		
197.8129				13721.98		
271.1725	271.1704	-7.88	1	22352.16	C18 H24 O2	(M-H)-
272.1756	272.1738	-6.85	1	2547	C18 H24 O2	(M-H)-
273.177	273.1767	-1.08	1	1456.85	C18 H24 O2	(M-H)-
285.1879			1	13278.13		
357.2111			1	13658.5		

385.2316			1	14844.15		
388.1957			1	25895.24		

MSMS Spectrum

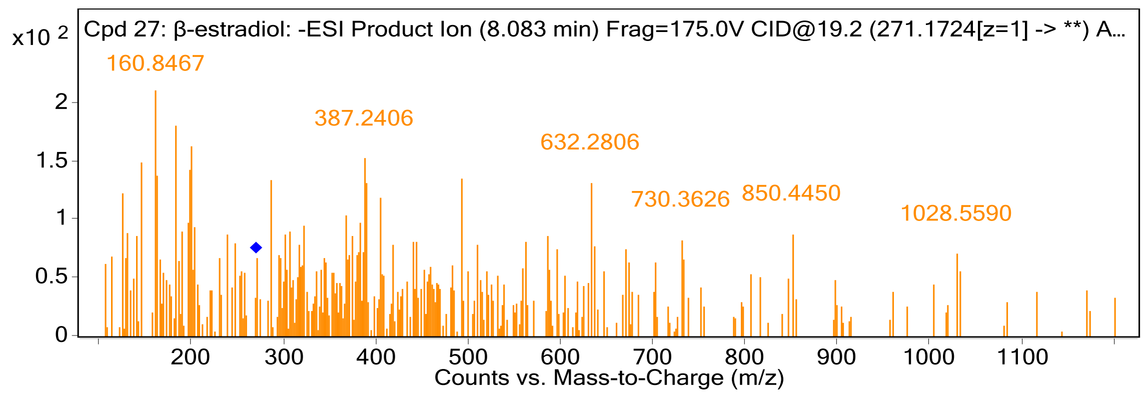

MS/MS Spectrum Peak List

m/z	Abund
144.8716	149.53
160.8467	210.54
162.8432	138.62
182.1605	181.16
197.8134	143.32
199.1828	163.66
285.1861	134.39
387.2406	152.56
491.1648	136.07
632.2806	132.06

Compound Structure

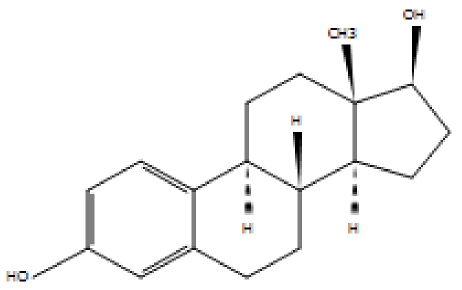

Compound Label	Name	*m/z*	RT	Algorithm	Mass
Cpd 28: Europine	**Europine**	388.1957	8.163	Auto MS/MS	329.1814

MS Spectrum

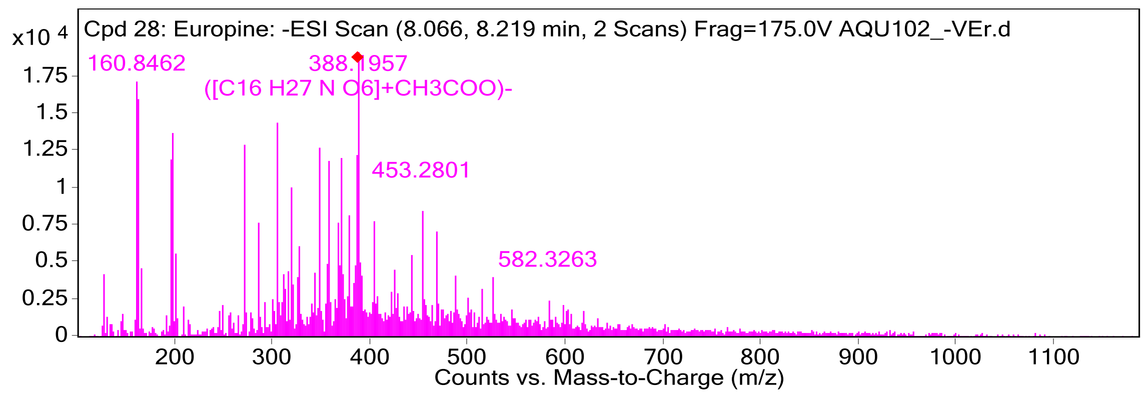

MS Zoomed Spectrum

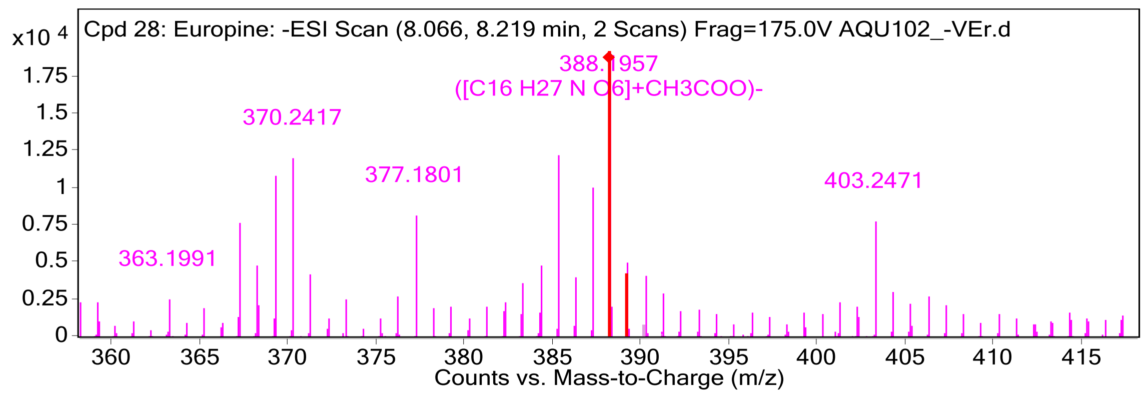

MS Spectrum Peak List

m/z	*Calc m/z*	Diff(ppm)	z	Abund	Formula	Ion
160.8462				17150.29		
162.8432				15958.75		
197.8129				13743.3		
271.1723				12944.12		
305.1573			1	14415.08		
347.1513			1	12692.69		
370.2417			1	12049.79		
385.2316			1	12181.61		
388.1957	388.1977	5.19	1	19129.82	C16 H27 N O6	(M+CH3COO)-
389.1969	389.201	10.5	1	4989.39	C16 H27 N O6	(M+CH3COO)-

MSMS Spectrum

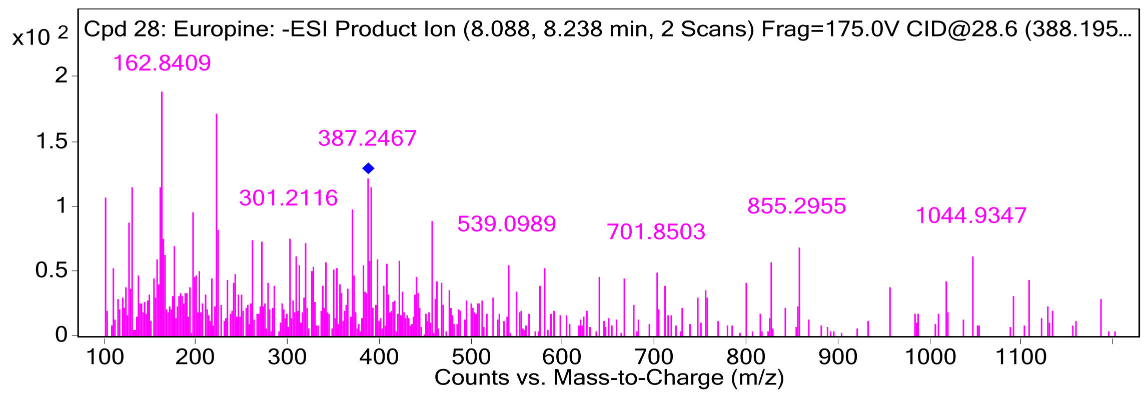

MS/MS Spectrum Peak List

m/z	z	Abund
101.0639		107.79
130.0913		115.97
160.8449		115.86
162.8409		189.62
195.8161		96.74
222.1294		171.83

369.2337	1	98.23
387.2467	1	121.88
390.2081		115.79
456.2743		89.5

Compound Structure

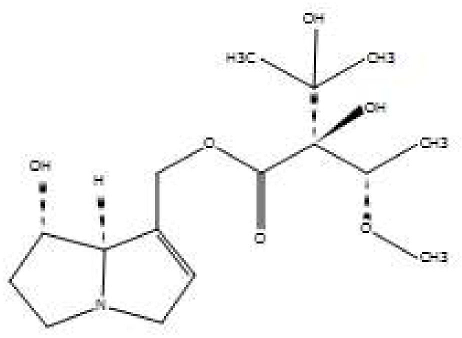

Compound Label	*m/z*	RT	Algorithm
Compound 29	327.2246	8.276	Auto MS/MS

MS Spectrum

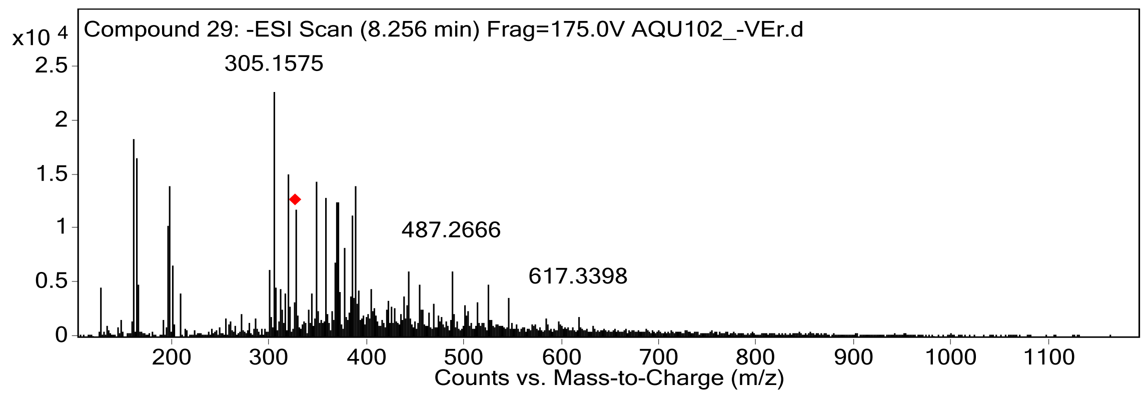

MS Zoomed Spectrum

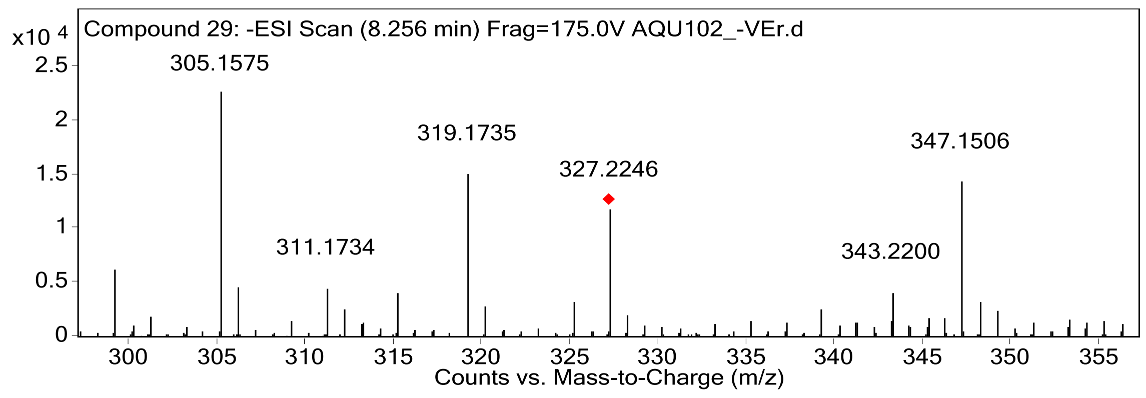

MS Spectrum Peak List

m/z	z	Abund
160.8462		18387.37
162.843		16551.72
197.8127		14026.82
305.1575	1	22756.99
319.1735	1	15138.17
327.2246	1	11774.37
328.2272	1	1976.58
347.1506	1	14382.15
357.2101	1	12928.36
387.2465	1	14011.71

MSMS Spectrum

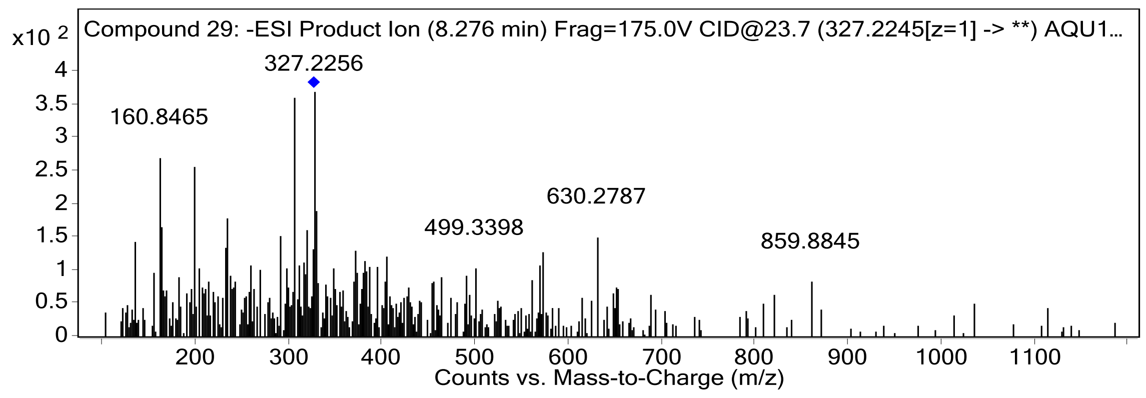

MS/MS Spectrum Peak List

m/z	Abund
160.8465	271.03
162.8423	166.92
197.8128	256.1
233.1609	179.01
291.2013	152.86
305.1537	360.33
319.1727	162.27
327.2256	370.68
328.2256	190.64
630.2787	151.25

Compound Label	Name	*m/z*	RT	Algorithm	Mass
Cpd 30: Cavipetin C	**Cavipetin C**	387.2466	8.456	Auto MS/MS	388.2543

MS Spectrum

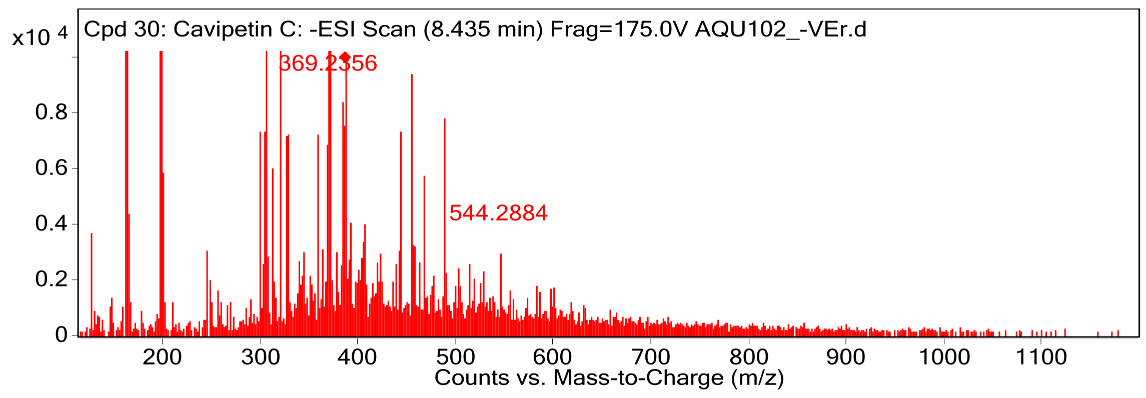

MS Zoomed Spectrum

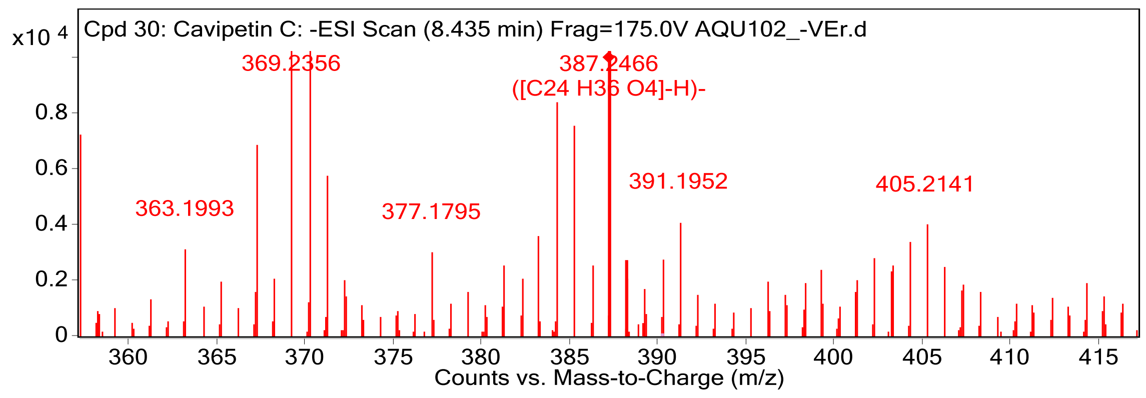

MS Spectrum Peak List

m/z	*Calc m/z*	Diff(ppm)	z	Abund	Formula	Ion
160.8463				17866.37		
162.8429				16432.35		
197.8126				11564.89		
305.1574			1	18361.24		
319.1732			1	12977.86		
369.2356			1	27920.12		
370.2406			1	11300.47		
387.2466	387.2541	19.27	1	10228.13	C24 H36 O4	(M-H)-
388.2495	388.2575	20.69	1	2088.31	C24 H36 O4	(M-H)-
389.2602	389.2604	0.6	1	835.78	C24 H36 O4	(M-H)-

MSMS Spectrum

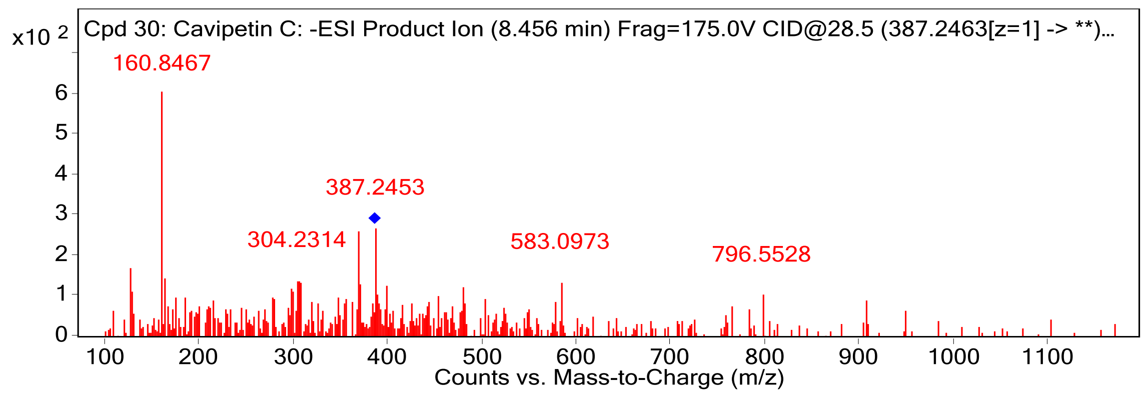

MS/MS Spectrum Peak List

m/z	z	Abund
126.9094		168.93
160.8467		607.86
162.8408		144.13
304.2314		139.68
305.2311		139.54
307.1671		136.12
369.2365	1	260.94
370.2352	1	129.77
387.2453	1	269.59
583.0973		135.18

Compound Structure

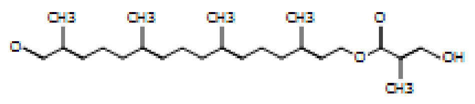

Compound Label	*m/z*	RT	Algorithm
Compound 31	369.2358	8.497	Auto MS/MS

MS Spectrum

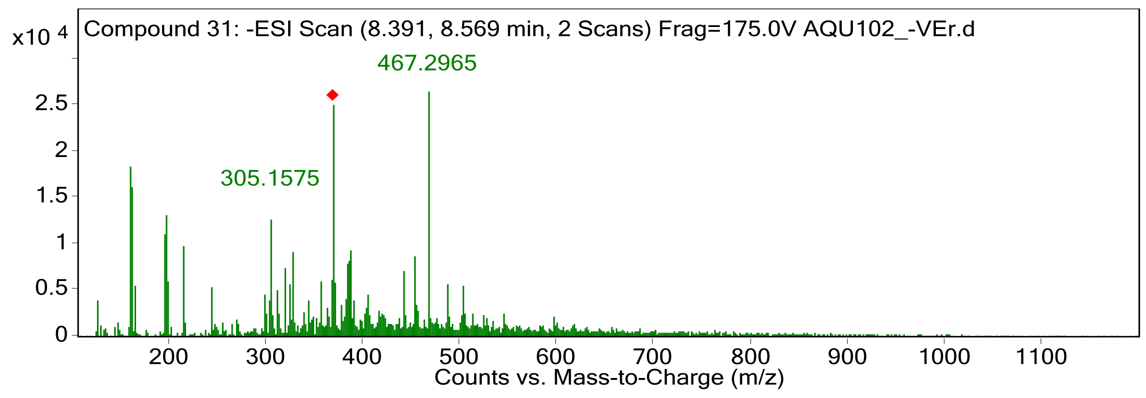

MS Zoomed Spectrum

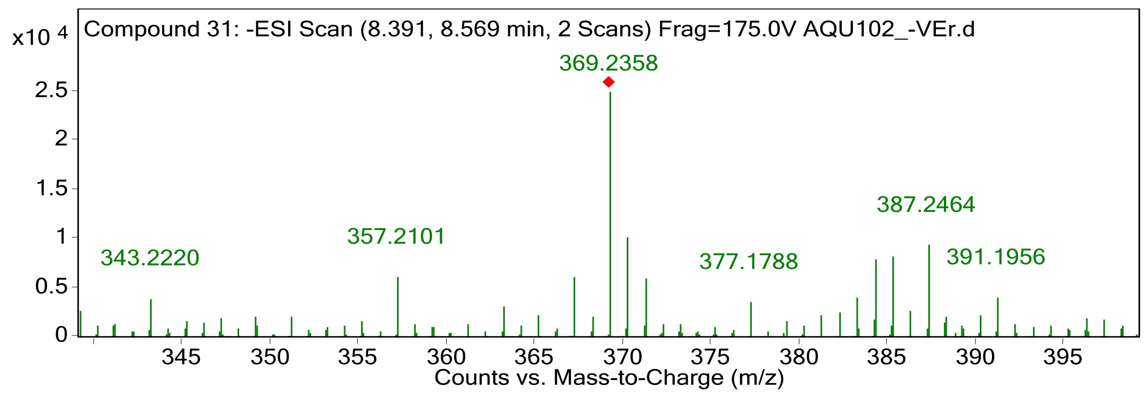

MS Spectrum Peak List

m/z	z	Abund
160.8462		18330.98
162.8431		16197.44
195.8157		11063.62
197.8129		13111.26
305.1575	1	12741.47
369.2358	1	24990.88
370.2408	1	10118.97
371.2444	1	5912.29

372.2525	1	1339.59
467.2965	1	26518.69

MSMS Spectrum

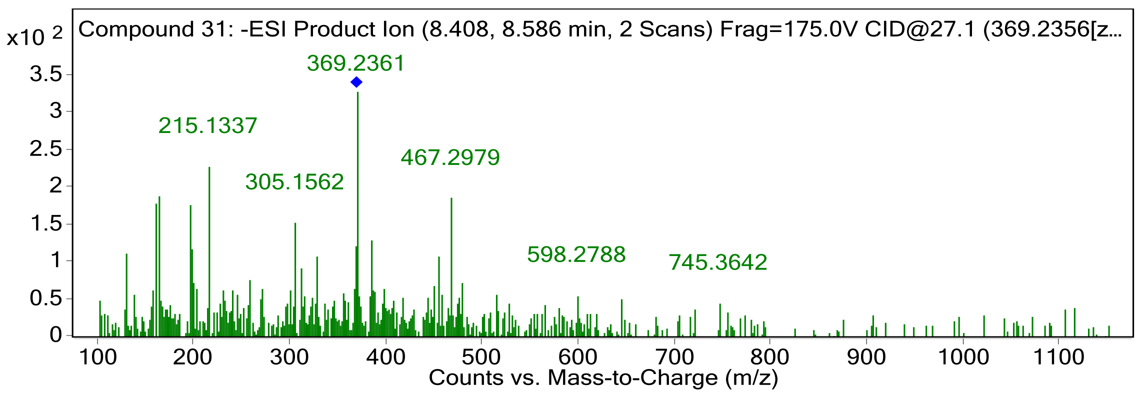

MS/MS Spectrum Peak List

m/z	z	Abund
160.846		179.59
162.8422		187.76
195.8151		177.48
215.1337		228.76
305.1562		153.22
367.2206		121.79
369.2361	1	328.77
384.2528		129.21
466.9844		130.67
467.2979		185.93

Compound Label	Name	*m/z*	RT	Algorithm	Mass
Cpd 32: 3alpha,7beta,12alpha-Trihydroxy-5alpha-cholan-	**3alpha,7beta,12alpha-Trihydroxy-5alpha-cholan-24-oic Acid**	467.2964	8.594	Auto MS/MS	408.2827

MS Spectrum

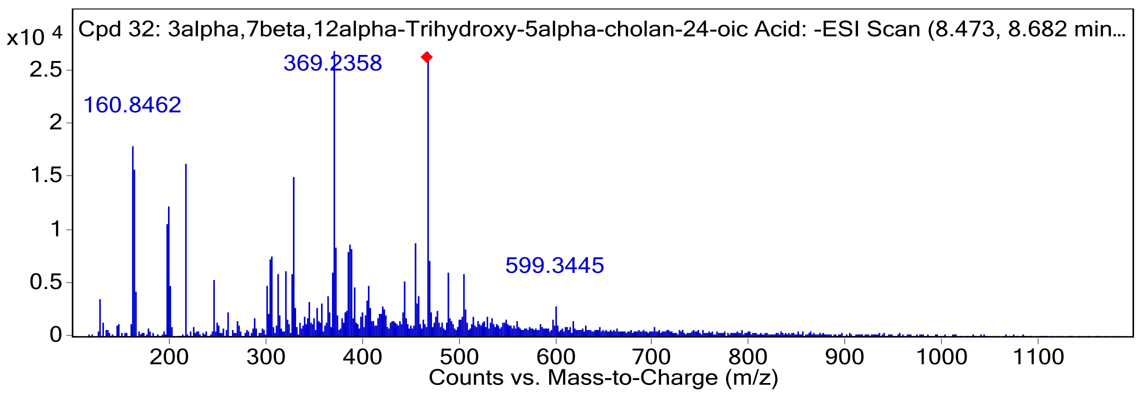

MS Zoomed Spectrum

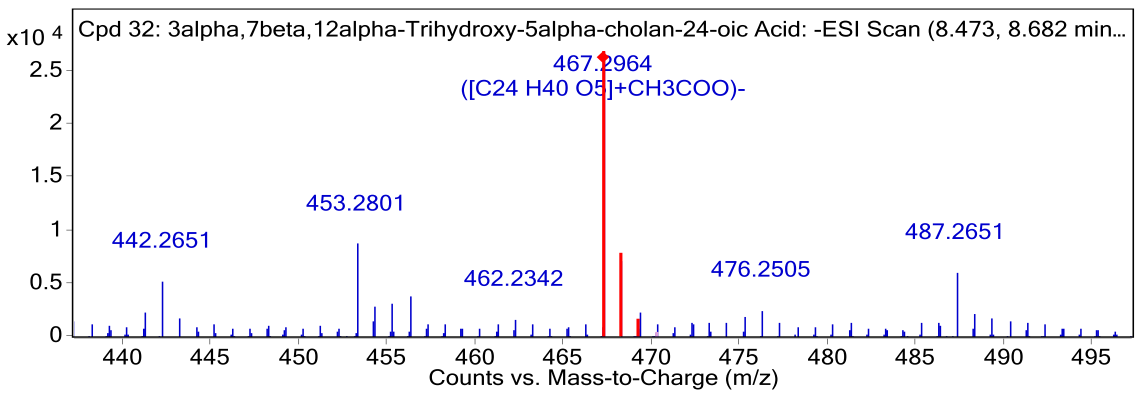

MS Spectrum Peak List

m/z	*Calc m/z*	Diff(ppm)	z	Abund	Formula	Ion
160.8462				18019.94		
162.8431				15707.22		
197.8127				12306.27		
215.1336			1	16237.69		
327.2245			1	15088.46		
369.2358			1	26987.94		
370.2411			1	13680.33		
467.2964	467.3014	10.84	1	26797.51	C24 H40 O5	(M+CH3COO)-
468.2998	468.3048	10.77	1	7135.86	C24 H40 O5	(M+CH3COO)-
469.305	469.3076	5.39	1	2333.45	C24 H40 O5	(M+CH3COO)-

MSMS Spectrum

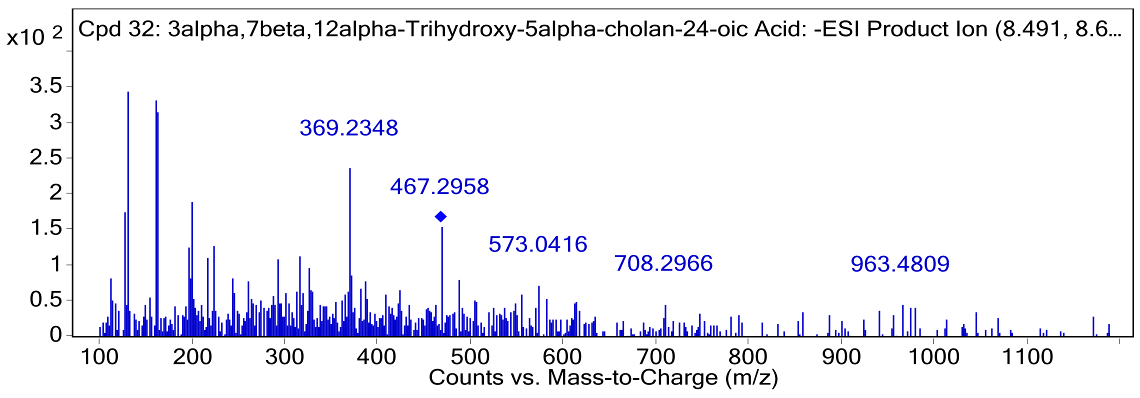

MS/MS Spectrum Peak List

m/z	z	Abund
127.0546		175.26
130.0916		345.32
160.8461		333.14
162.8435		316.15
195.8156		125.48
199.185		190.28
222.1279		127.3
315.1905		113.41
369.2348	1	236.58
467.2958	1	154.04

Compound Structure

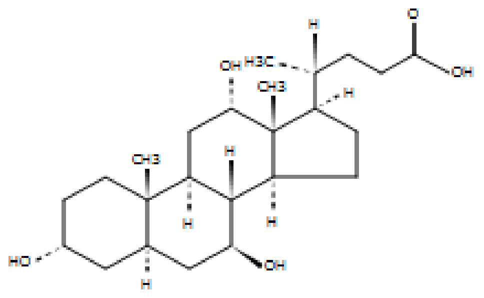

Compound Label	*m/z*	RT	Algorithm
Compound 33	327.2244	8.743	Auto MS/MS

MS Spectrum

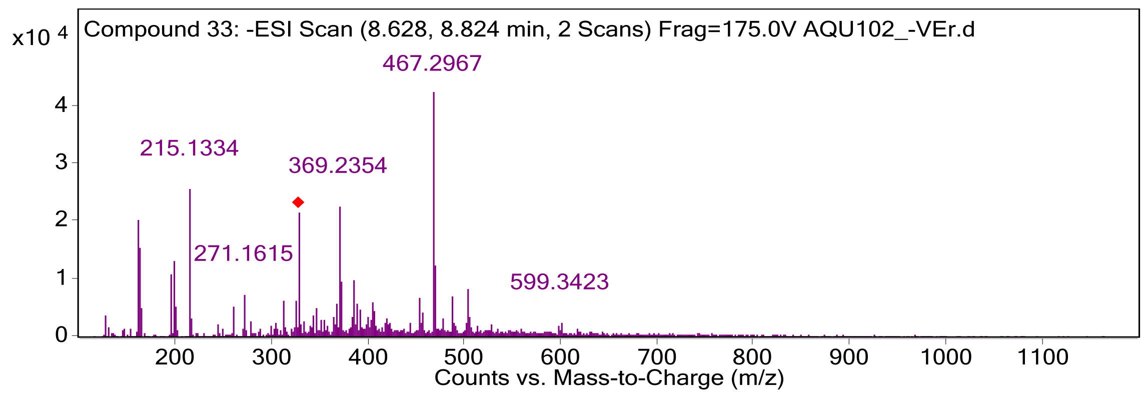

MS Zoomed Spectrum

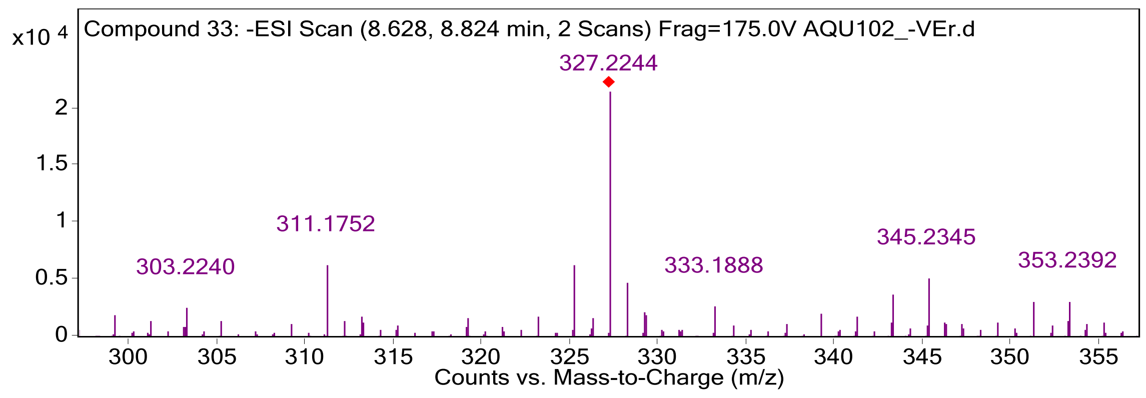

MS Spectrum Peak List

m/z	z	Abund
160.846		20470.47
162.8429		15544.88
197.8125		13187.41
215.1334	1	25719.22
327.2244	1	21518.22
328.2274	1	4713.1
329.2145	1	2243.57
369.2354		22701.62
370.241		13607.46
467.2967	1	42529.08

MSMS Spectrum

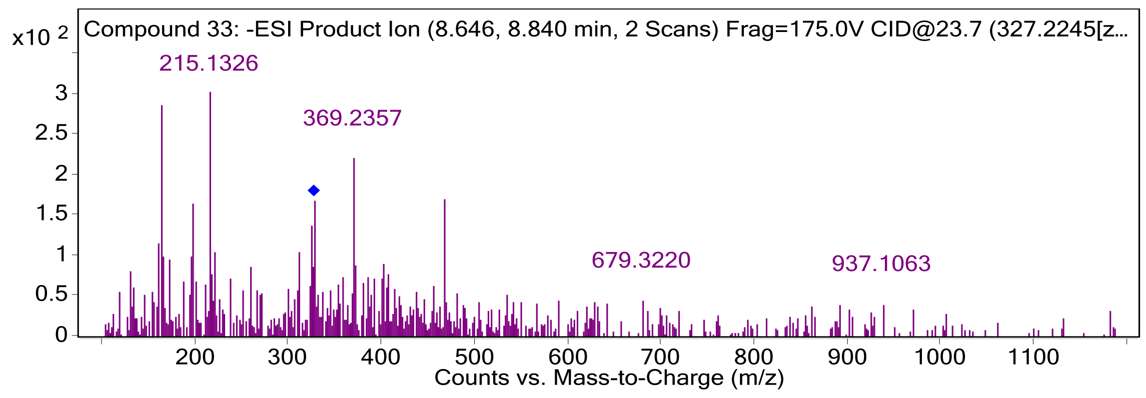

MS/MS Spectrum Peak List

m/z	z	Abund
160.8459		115.39
162.8433		286.09
197.8119		164.95
215.1326	1	303.57
221.122		106.02
311.1734		106
325.1877		137.42
327.2236	1	169.04
369.2357	1	220.53
467.2977	1	170.28

Compound Label	Name	*m/z*	RT	Algorithm	Mass
Cpd 34: 3alpha,7beta,12alpha-Trihydroxy-5alpha-cholan-	**3alpha,7beta,12alpha-Trihydroxy-5alpha-cholan-24-oic Acid**	467.2966	8.967	Auto MS/MS	408.2828

MS Spectrum

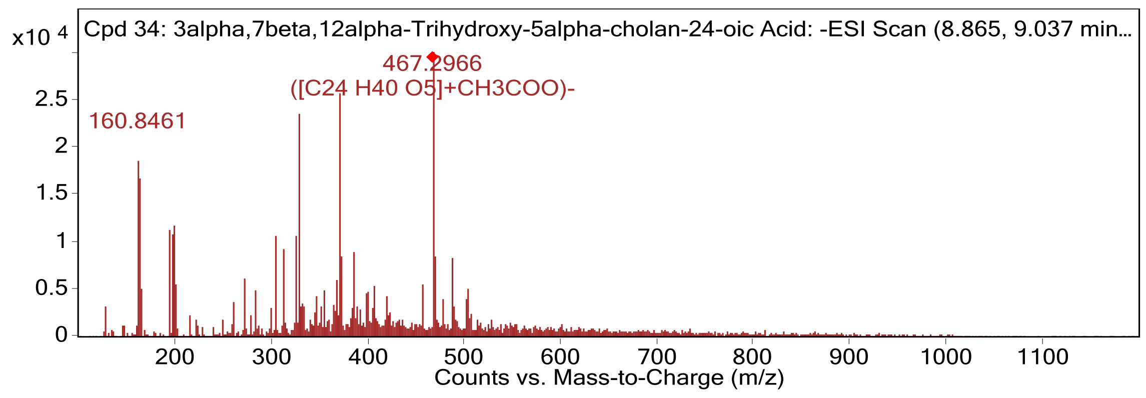

MS Zoomed Spectrum

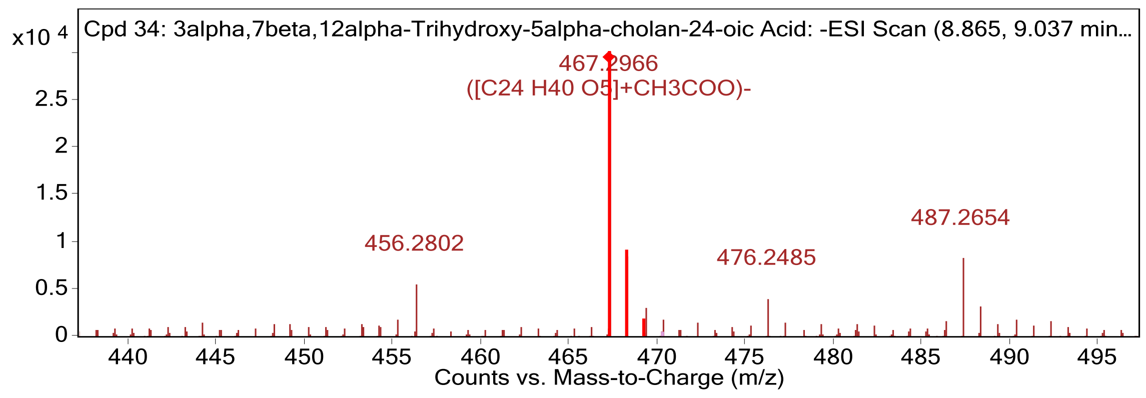

MS Spectrum Peak List

m/z	*Calc m/z*	Diff(ppm)	z	Abund	Formula	Ion
160.8461				18556.85		
162.843				16775.09		
193.0951				11303.44		
195.8156				10923.76		
197.8128				11751.05		
327.2245			1	23666.22		
369.2358			1	25701.37		
467.2966	467.3014	10.42	1	30116.34	C24 H40 O5	(M+CH3COO)-
468.2996	468.3048	11.26	1	8562.24	C24 H40 O5	(M+CH3COO)-
469.3052	469.3076	5.11	1	3087.67	C24 H40 O5	(M+CH3COO)-

MSMS Spectrum

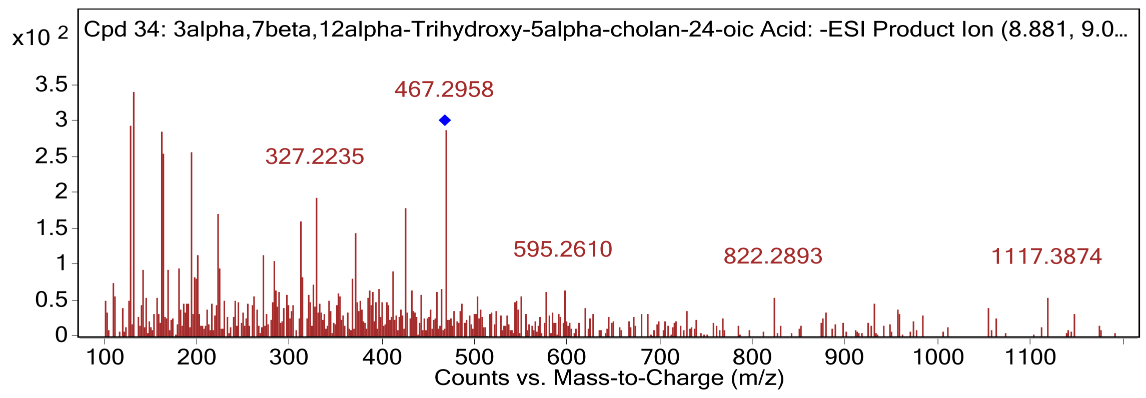

MS/MS Spectrum Peak List

m/z	z	Abund
127.0546		293.3
130.0903		341.88
160.8444		285.07
162.8424		256.14
193.0956		257.96
222.1296		171.86
311.1752	1	161.94
327.2235		194.89
423.3046		179.71
467.2958	1	288.49

Compound Structure

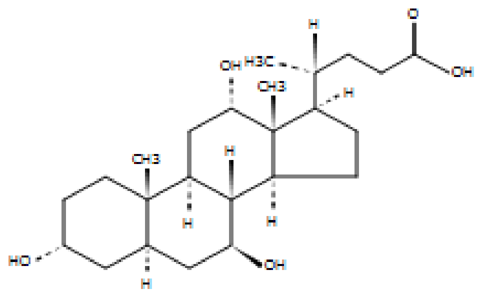

Compound Label	*m/z*	RT	Algorithm
Compound 35	369.2357	9.061	Auto MS/MS

MS Spectrum

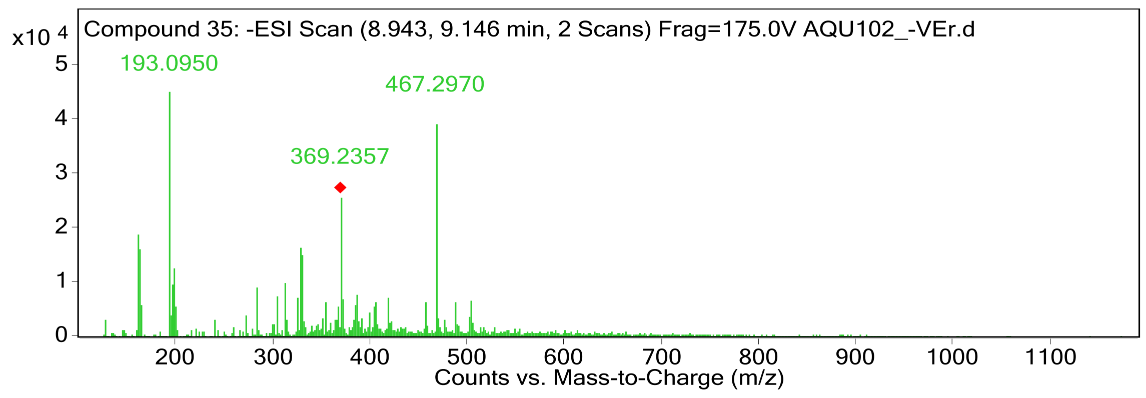

MS Zoomed Spectrum

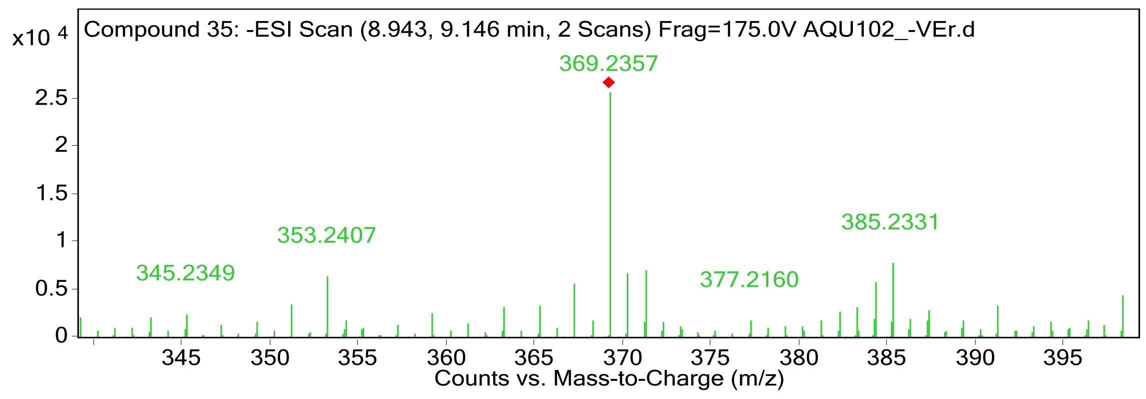

MS Spectrum Peak List

m/z	z	Abund
160.8462		18943.32
162.8432		16151.83
193.095	1	45084.49
197.8126		12573
327.2247	1	16486.51
329.2403	1	15146.41
369.2357	1	25735.18
370.2402	1	6715.95
467.297	1	39029.82

468.2999	1	10649.96

MSMS Spectrum

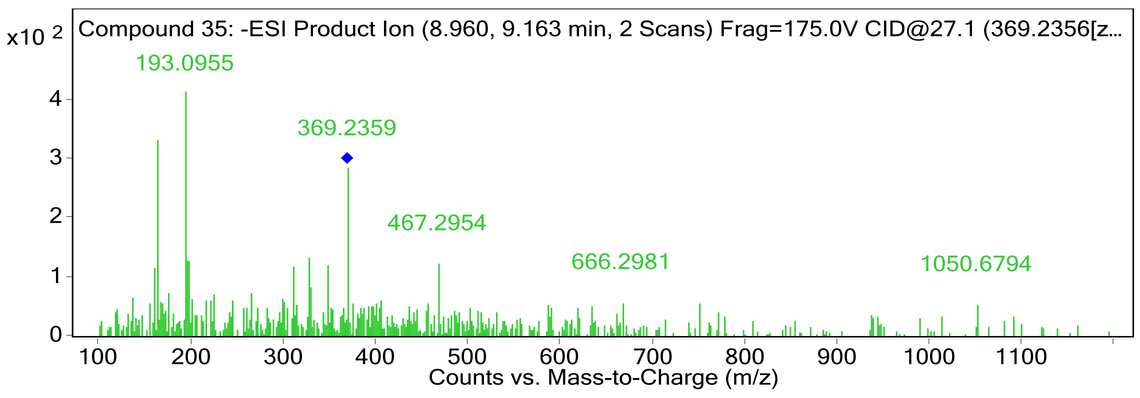

MS/MS Spectrum Peak List

m/z	z	Abund
162.8434		333.1
193.0955	1	415.57
195.8124		130.06
197.8129		129
311.1763		119.44
327.2231		133.64
347.153		120.56
369.2359		284.91
370.2381		177.44
467.2954	1	123.99

Compound Label	*m/z*	RT	Algorithm
Compound 36	327.2248	9.116	Auto MS/MS

MS Spectrum

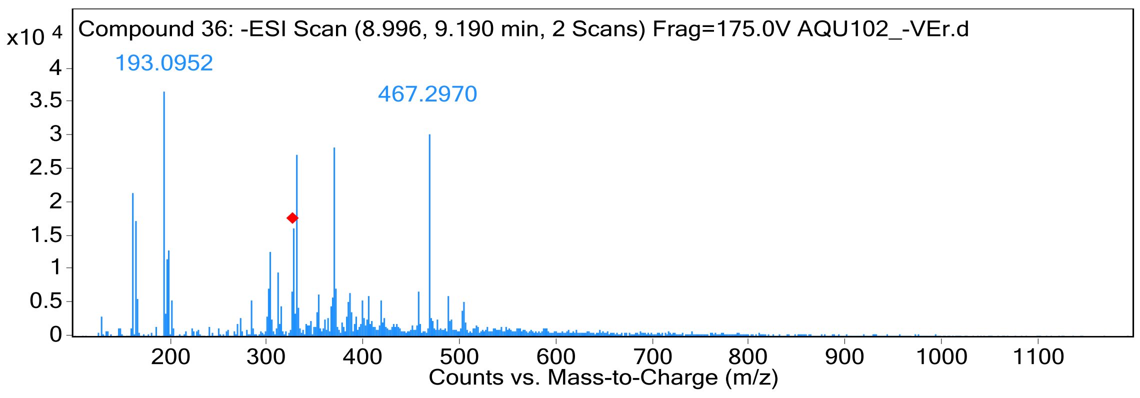

MS Zoomed Spectrum

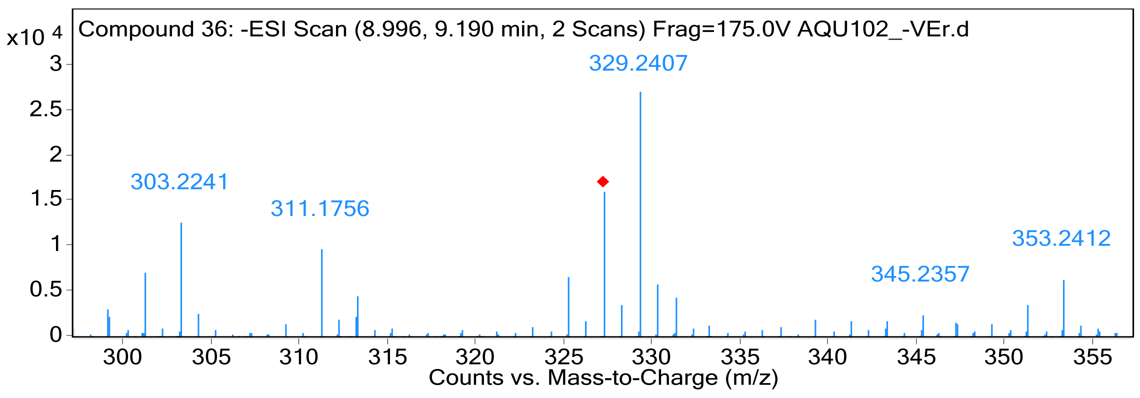

MS Spectrum Peak List

m/z	z	Abund
160.8461		21573.81
162.8432		17402.36
193.0952	1	36663.25
197.8129		12867.79
303.2241	1	12649.76
327.2248	1	16131.52
328.2279	1	3578.14
329.2407	1	27117.15
369.236	1	28259.96
467.297	1	30209.68

MSMS Spectrum

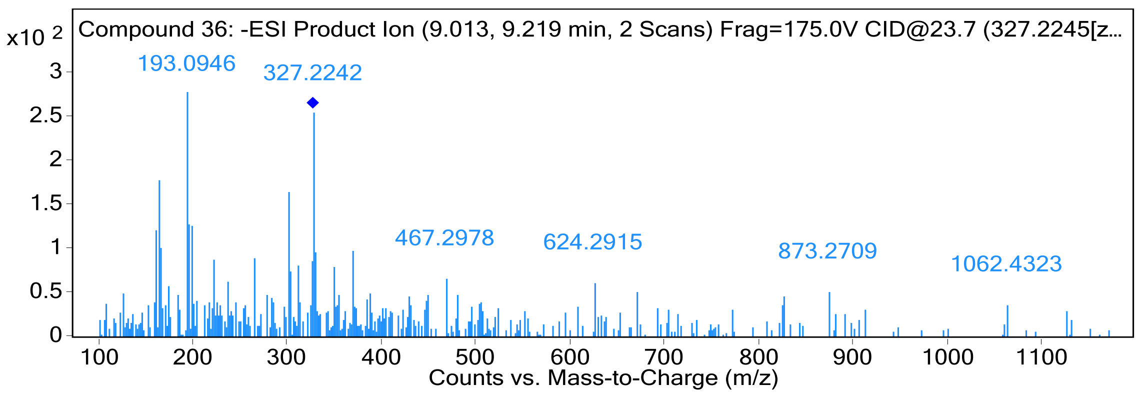

MS/MS Spectrum Peak List

m/z	z	Abund
160.8467		122.36
162.8429		179.19
164.8385		101.79
193.0946		279.41
195.8159		129.29
197.8095		126.67
301.211		165.11
327.2242		254.72
329.2403		97.49
369.2338	1	98.7

Compound Label	Name	*m/z*	RT	Algorithm	Mass
Cpd 37: Laccarin	**Laccarin**	193.095	9.155	Auto MS/MS	194.102

MS Spectrum

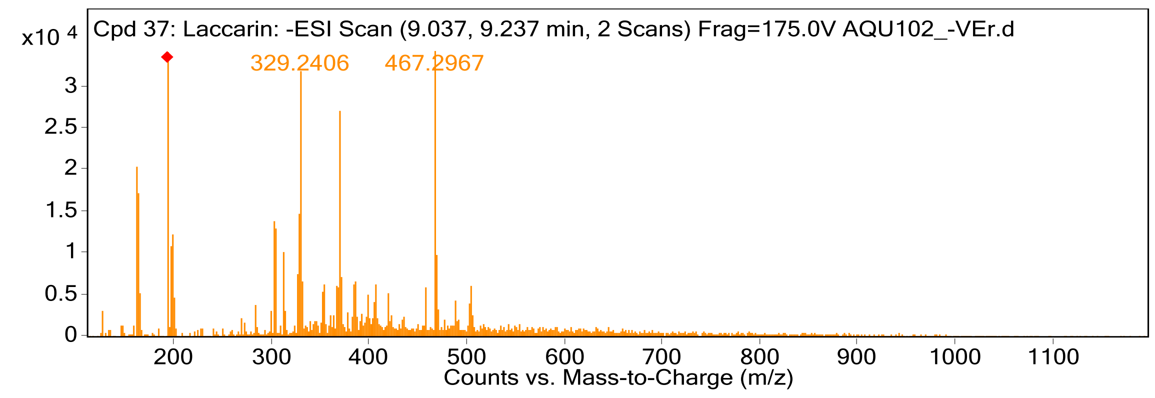

MS Zoomed Spectrum

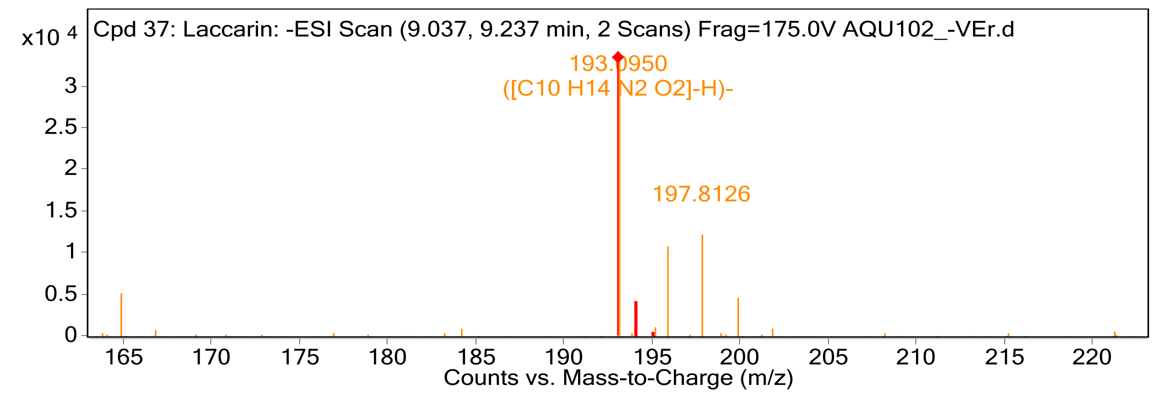

MS Spectrum Peak List

m/z	*Calc m/z*	Diff(ppm)	z	Abund	Formula	Ion
160.8462				20414.38		
162.8432				17218.11		
193.095	193.0983	16.85	1	34164.16	C10 H14 N2 O2	(M-H)-
194.0983	194.1013	15.33	1	3207.68	C10 H14 N2 O2	(M-H)-
195.0911	195.1035	63.76	1	1298.03	C10 H14 N2 O2	(M-H)-
301.208			1	13859.48		
327.2246			1	14769.17		
329.2406			1	31850.07		
369.2357			1	27034.91		
467.2967			1	34670.33		

MSMS Spectrum

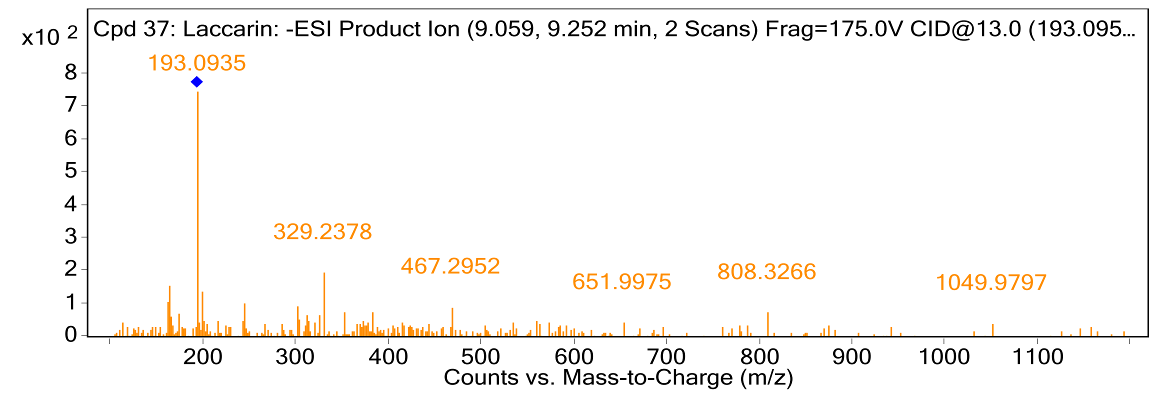

MS/MS Spectrum Peak List

m/z	z	Abund
160.8447		109.02
162.8407		157.74
193.0935	1	747.73
194.0979	1	83.6
197.81		139.17
243.1776		101.52
301.2057		92.73
329.2378		196
381.1852		75
467.2952		91.06

Compound Structure

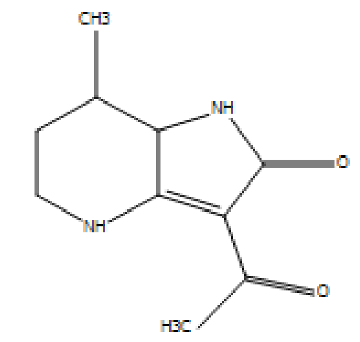

Compound Label	*m/z*	RT	Algorithm
Compound 38	329.2402	9.23	Auto MS/MS

MS Spectrum

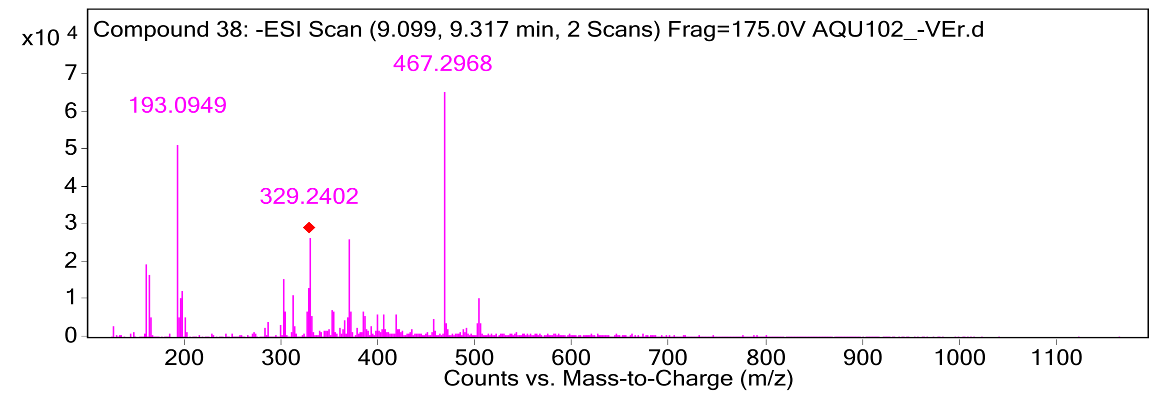

MS Zoomed Spectrum

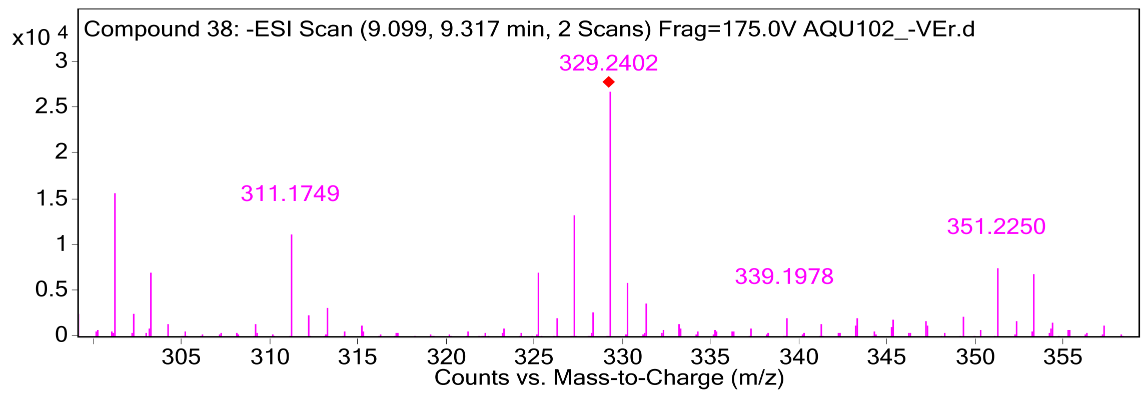

MS Spectrum Peak List

m/z	z	Abund
160.8462		19701.69
162.8431		17017.06
193.0949	1	51202.23
301.2082	1	15745.28
327.2246	1	13345.98
329.2402	1	26853.25
330.2431	1	5885.01
369.2355	1	26244.27
467.2968	1	65562.08
468.2997	1	17299.05

MSMS Spectrum

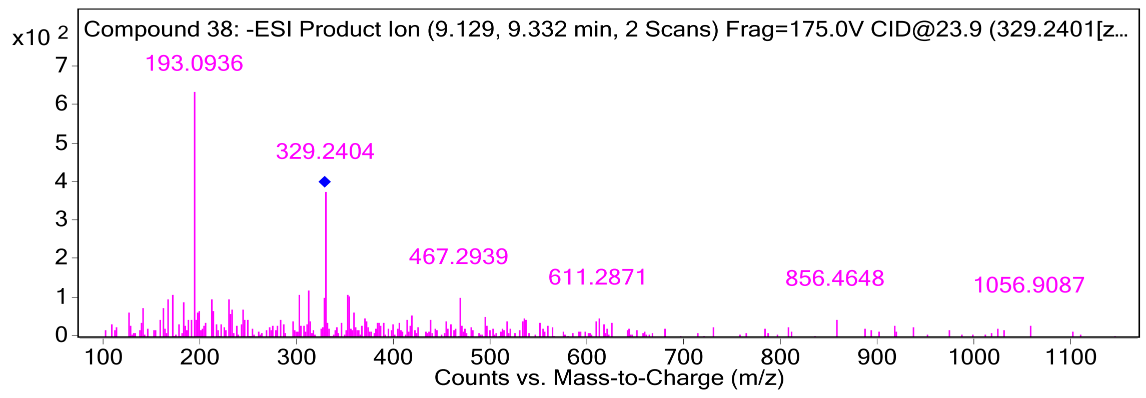

MS/MS Spectrum Peak List

m/z	z	Abund
171.1057		110.99
193.0936	1	636.74
229.1477		100.08
301.2069		111.43
311.1753		121.47
327.2241	1	103.12
329.2404	1	376.26
351.2182		111.3
353.2389		106.98
467.2939	1	102.99

Compound Label	Name	*m/z*	RT	Algorithm	Mass
Cpd 39: sn-3-O-(geranylgeranyl)glycerol 1-phosphate	**sn-3-O-(geranylgeranyl)glycerol 1-phosphate**	503.2741	9.299	Auto MS/MS	444.2604

MS Spectrum

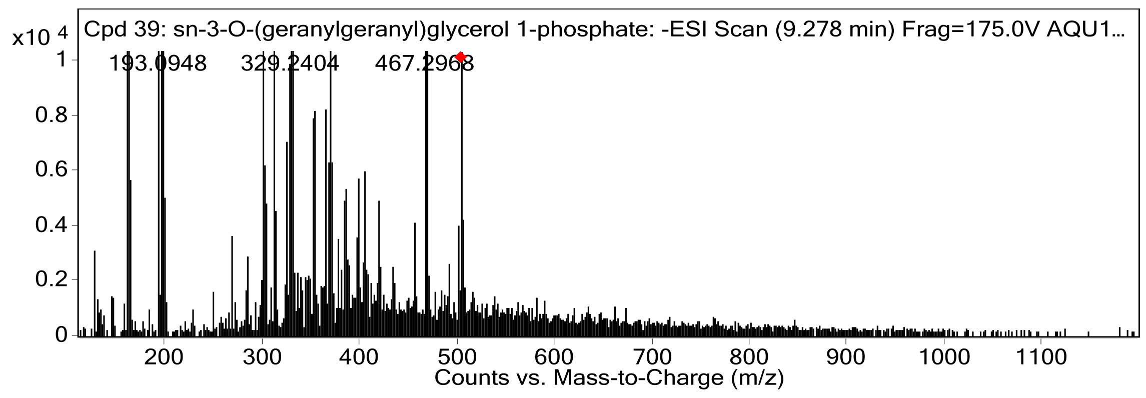

MS Zoomed Spectrum

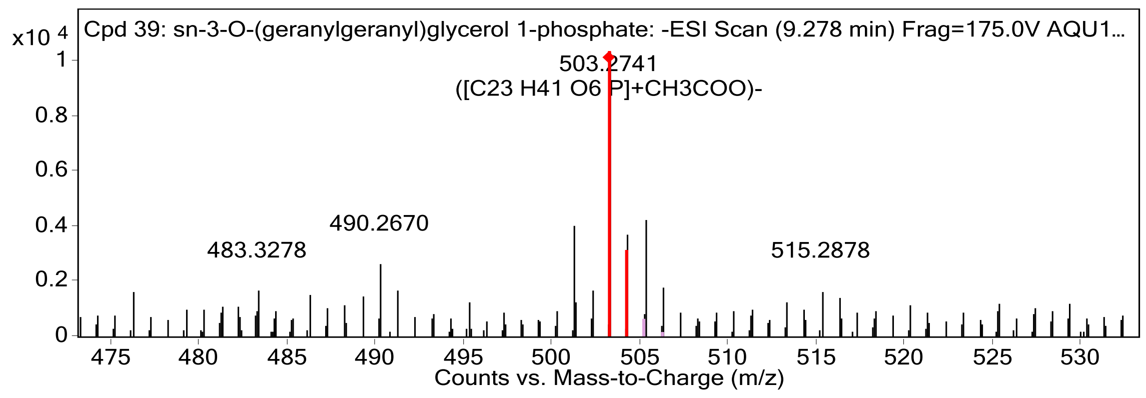

MS Spectrum Peak List

m/z	*Calc m/z*	Diff(ppm)	z	Abund	Formula	Ion
160.8462				21340.16		
162.8432				17747.24		
193.0948			1	30777.77		
301.2083			1	34125.18		
329.2404			1	53557.71		
369.2356			1	28830.66		
467.2968			1	64683.21		
468.3004			1	17953.71		
503.2741	503.2779	7.62	1	10321.74	C23 H41 O6 P	(M+CH3COO)-
504.278	504.2813	6.63	1	3749.7	C23 H41 O6 P	(M+CH3COO)-

MSMS Spectrum

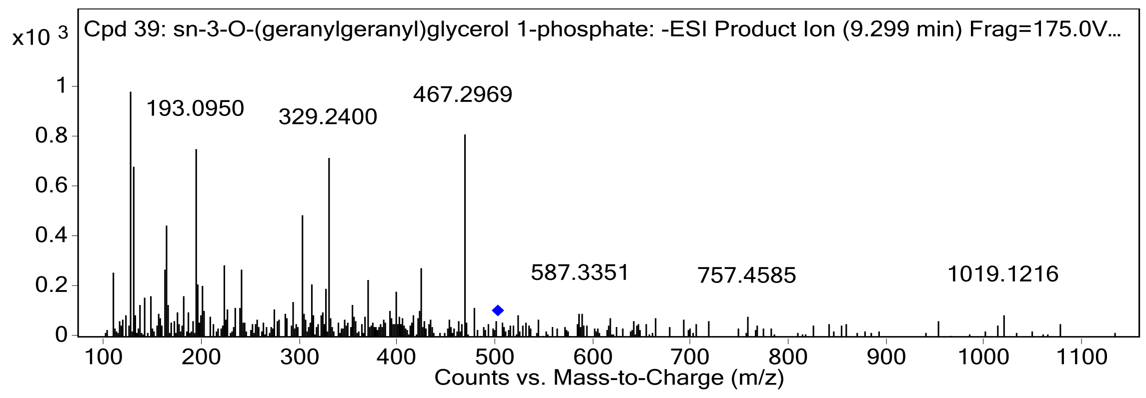

MS/MS Spectrum Peak List

m/z	z	Abund
127.0555		985.82
130.0916	1	686.48
162.8436		449.71
193.095		755.12
222.1293		290.49
240.1397		272.45
301.2072		489.18
329.24	1	721.25
423.3051		276.58
467.2969	1	810.96

Compound Structure

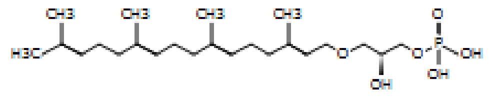

Compound Label	Name	*m/z*	RT	Algorithm	Mass
Cpd 40: 3alpha,7beta,12alpha-Trihydroxy-5alpha-cholan-	**3alpha,7beta,12alpha-Trihydroxy-5alpha-cholan-24-oic Acid**	467.2966	9.352	Auto MS/MS	408.2831

MS Spectrum

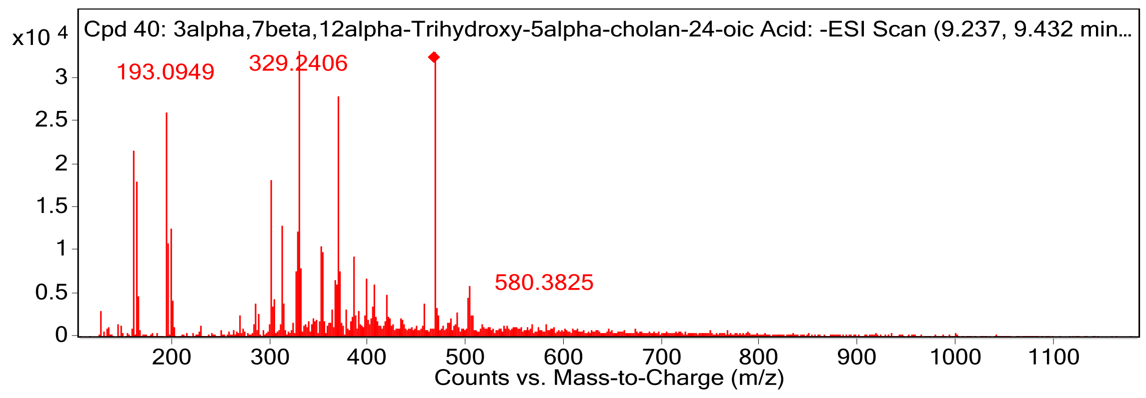

MS Zoomed Spectrum

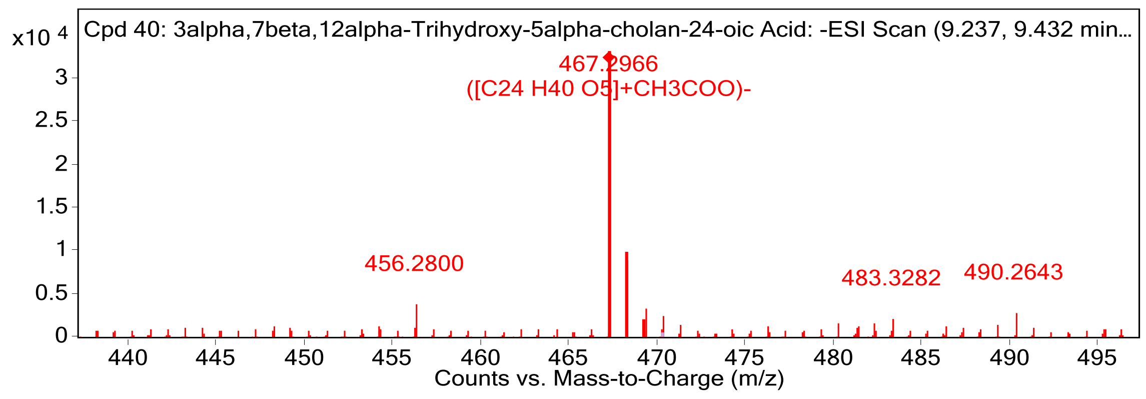

MS Spectrum Peak List

m/z	*Calc m/z*	Diff(ppm)	z	Abund	Formula	Ion
160.8462				21590.84		
162.8432				18001.56		
193.0949			1	26087.75		
301.2081			1	18294.96		
311.1749			1	12872.67		
329.2406			1	38125.57		
369.2356			1	27880.22		
467.2966	467.3014	10.24	1	33063.25	C24 H40 O5	(M+CH3COO)-
468.2998	468.3048	10.75	1	8988.68	C24 H40 O5	(M+CH3COO)-
469.308	469.3076	-1	1	3360.02	C24 H40 O5	(M+CH3COO)-

MSMS Spectrum

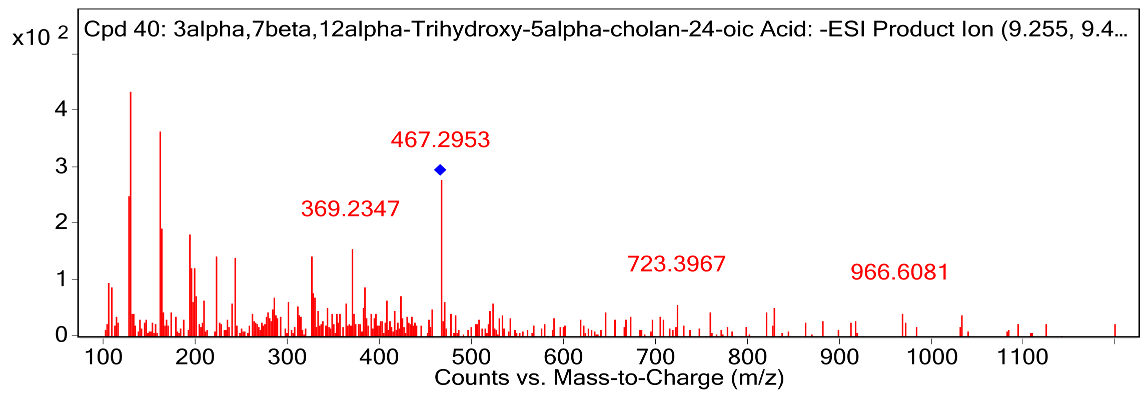

MS/MS Spectrum Peak List

m/z	z	Abund
127.0542		249.57
130.0904	1	434.64
160.8458		364.6
162.8433		191.84
193.0952		181.37
222.1289		142.01
243.1747		140.04
325.1931	1	144.08
369.2347		154.8
467.2953		277.32

Compound Structure

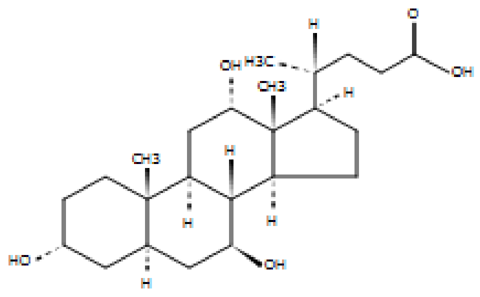

Compound Label	*m/z*	RT	Algorithm
Compound 41	369.2357	9.426	Auto MS/MS

MS Spectrum

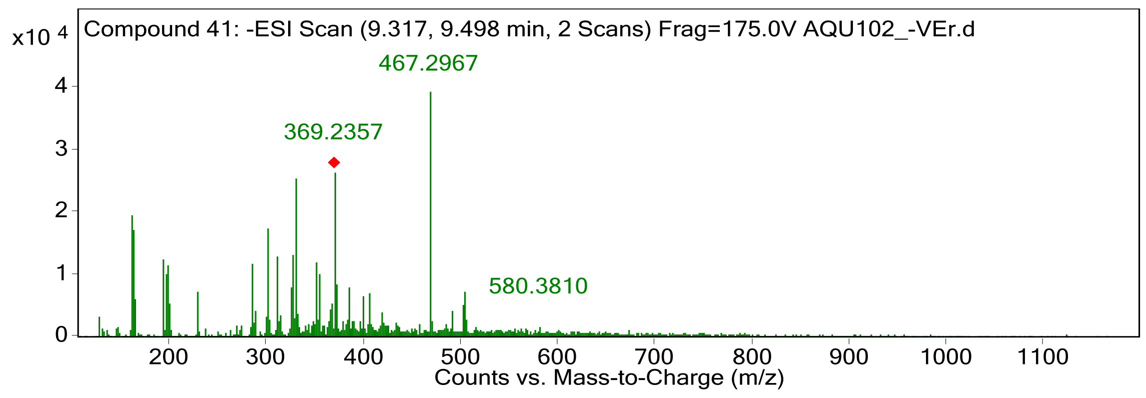

MS Zoomed Spectrum

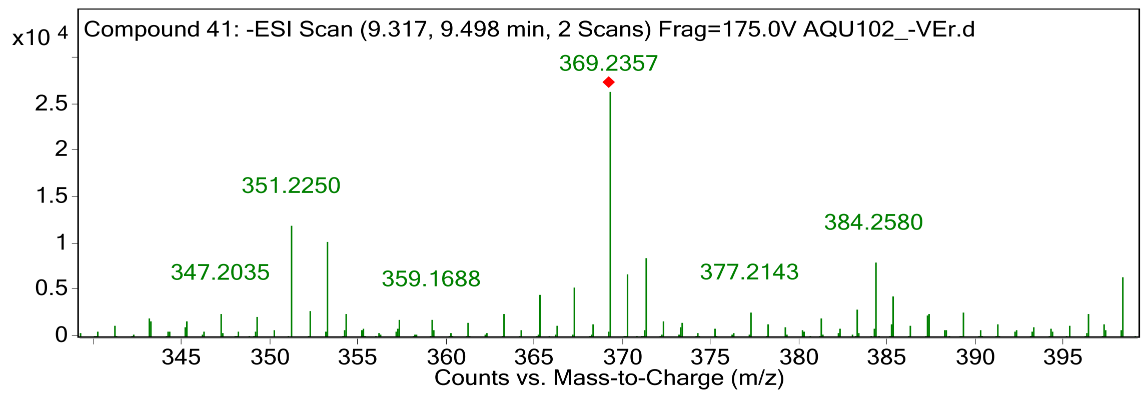

MS Spectrum Peak List

m/z	z	Abund
160.8461		19651.12
162.8428		17160.12
193.0948		12481.45
301.2082	1	17325.82
311.1749	1	12966.65
327.2246	1	13200.93
329.2402	1	25411.18
369.2357	1	26393.48
370.2389	1	6845.62
467.2967	1	39385.03

MSMS Spectrum

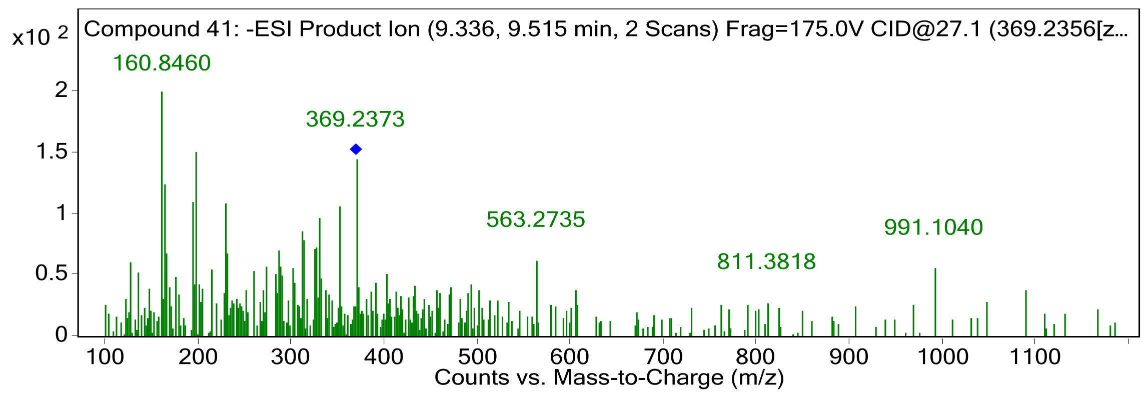

MS/MS Spectrum Peak List

m/z	z	Abund
160.846		200.99
162.841		125.16
193.0935	1	110.13
197.8122		150.7
229.149		109.26
311.1773		86.9
313.184		79.12
329.2408	1	97.52
351.2226		107.23
369.2373		144.81

Compound Label	*m/z*	RT	Algorithm
Compound 42	327.2244	9.493	Auto MS/MS

MS Spectrum

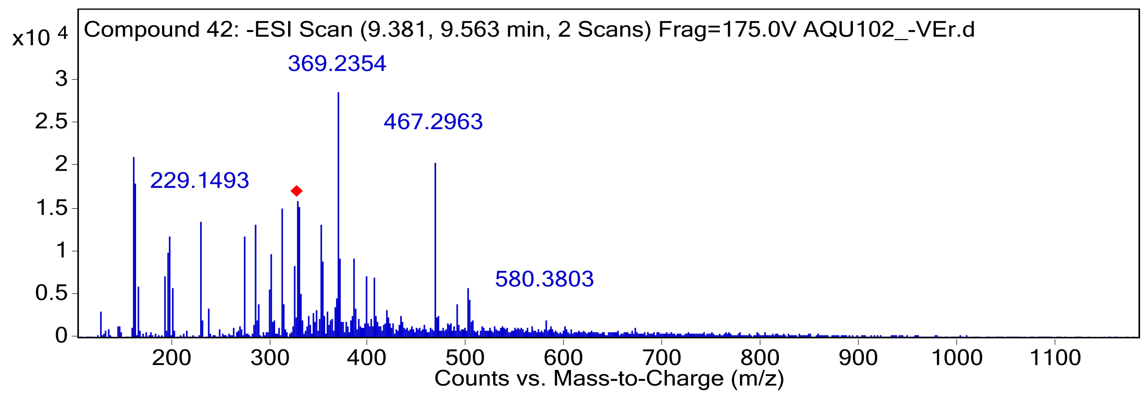

MS Zoomed Spectrum

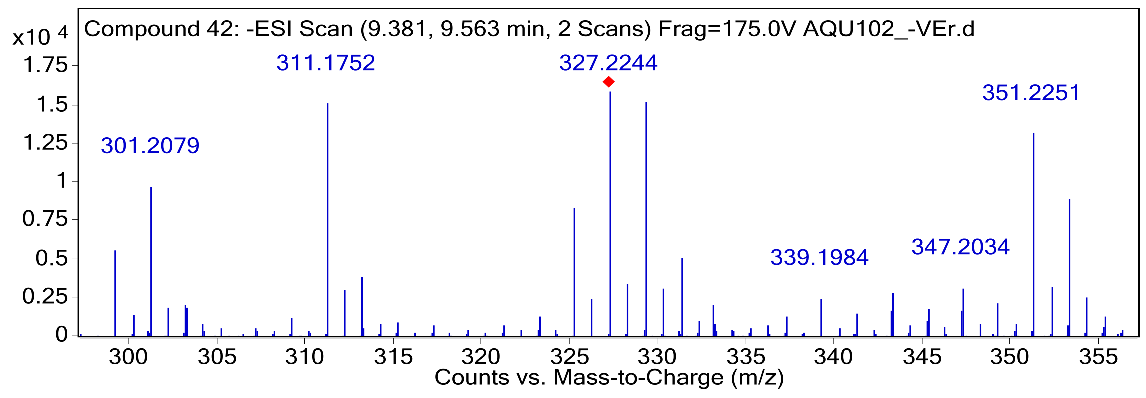

MS Spectrum Peak List

m/z	z	Abund
160.8461		21164.2
162.8428		18074.49
229.1493	1	13605.21
285.1769	1	13214.64
311.1752	1	15113.68
327.2244	1	15943.94
328.2277	1	3402.86
329.24	1	15292.47
369.2354	1	28697.89

467.2963	1	20476.9

MSMS Spectrum

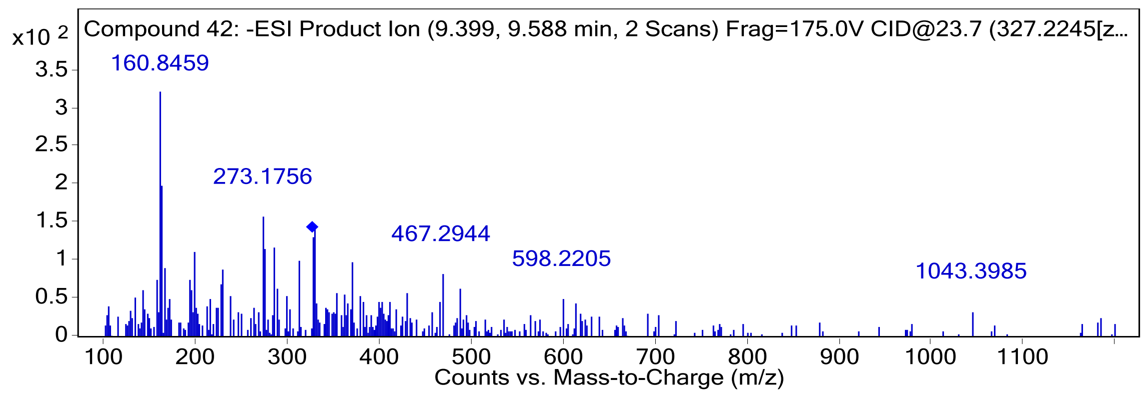

MS/MS Spectrum Peak List

m/z	z	Abund
160.8459		322.93
162.8429		199.11
197.1231		112.18
273.1756		157.42
274.1801		115.64
285.1759		118.38
311.1748		100.62
326.1934	1	125.14
327.2246	1	131.4
329.2408		142.04

Compound Label	*m/z*	RT	Algorithm
Compound 43	273.1766	9.673	Auto MS/MS

MS Spectrum

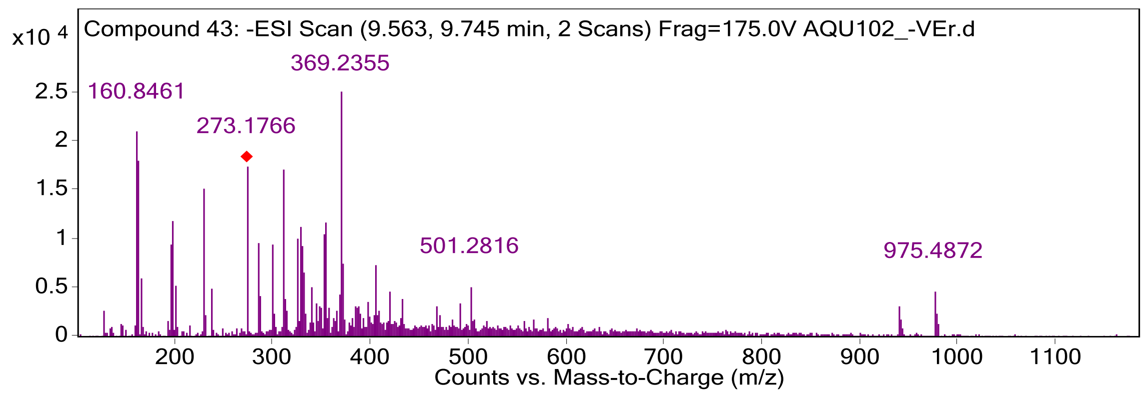

MS Zoomed Spectrum

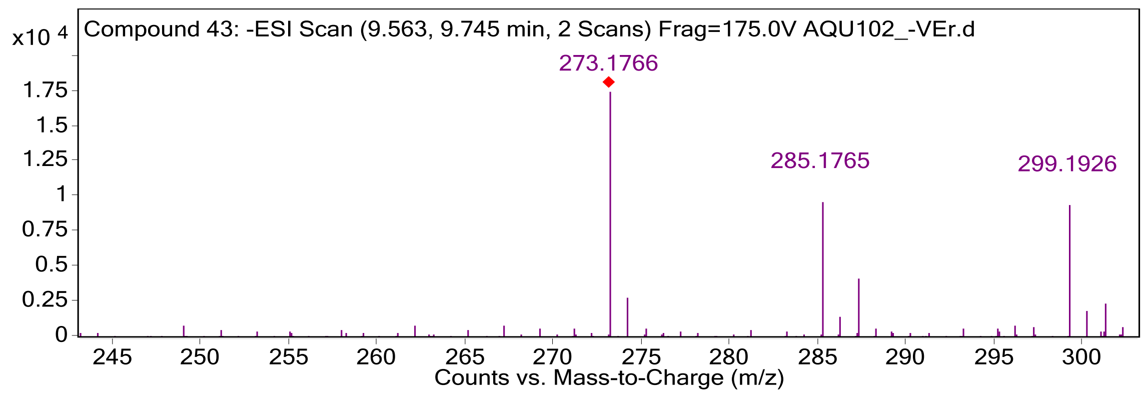

MS Spectrum Peak List

m/z	z	Abund
160.8461		21044.7
162.8429		18011
197.8126		11848.88
229.1493	1	15206.32
273.1766	1	17491.67
274.1803	1	2790.38
275.182	1	584.24
311.1754	1	17236.41
353.24	1	11775.37
369.2355	1	25180.06

MSMS Spectrum

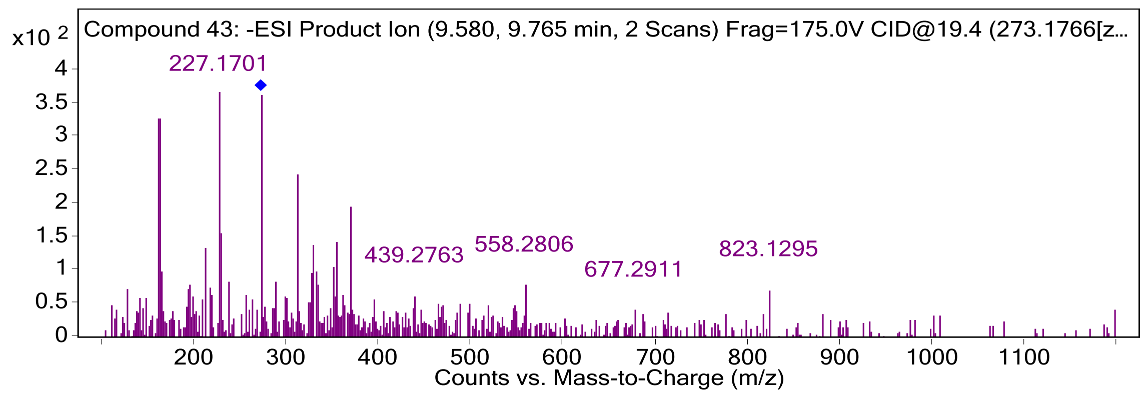

MS/MS Spectrum Peak List

m/z	z	Abund
160.846		328.15
162.8427		327.66
211.1748		134.5
227.1701		367.34
229.1473		156.86
273.1765		363.1
311.175	1	244.73
329.2403		137.58
353.2423		143.45
369.2368	1	195.55

Compound Label	Name	*m/z*	RT	Algorithm	Mass
Cpd 44: 20-Hydroxy-PGF2a	**20-Hydroxy-PGF2a**	369.2354	9.775	Auto MS/MS	370.2421

MS Spectrum

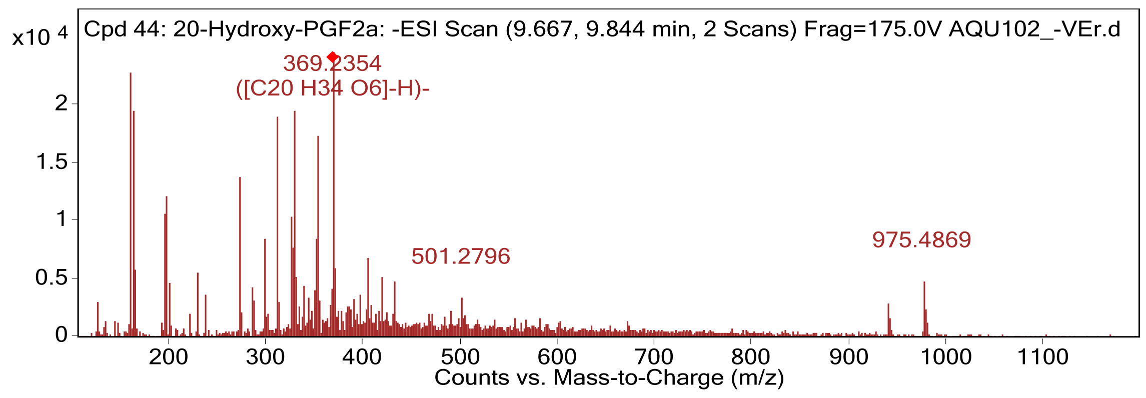

MS Zoomed Spectrum

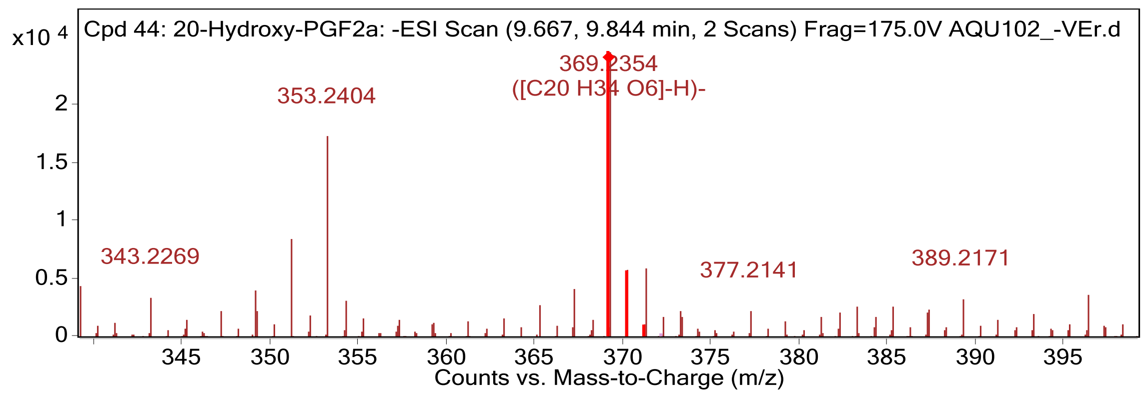

MS Spectrum Peak List

m/z	*Calc m/z*	Diff(ppm)	z	Abund	Formula	Ion
160.846				22766.01		
162.8432				19475.49		
197.8127				12181.87		
273.1768			1	13788.16		
311.1751			1	19066.96		
329.2401			1	19473.62		
353.2404			1	17332.47		
369.2354	369.2283	-19.3	1	24582.78	C20 H34 O6	(M-H)-
370.2389	370.2317	-19.4	1	5839.28	C20 H34 O6	(M-H)-
371.2248	371.2342	25.22	1	1078.6	C20 H34 O6	(M-H)-

MSMS Spectrum

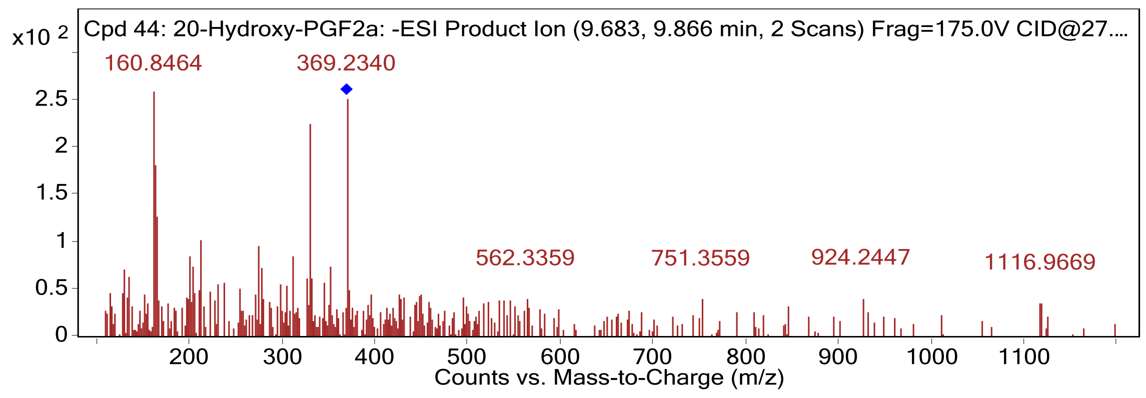

MS/MS Spectrum Peak List

m/z	z	Abund
160.8464		259.72
162.8431		181.94
164.8409		127.58
199.8098		86.06
211.1365		102.59
273.1776		96.92
311.174		85.35
329.2396	1	225.65
351.2251		74.74
369.234	1	252.07

Compound Structure

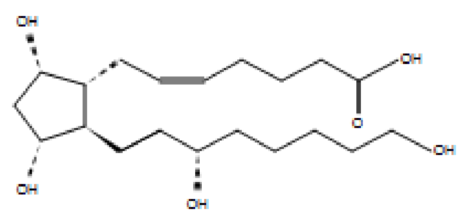

Compound Label	*m/z*	RT	Algorithm
Compound 45	329.2401	9.794	Auto MS/MS

MS Spectrum

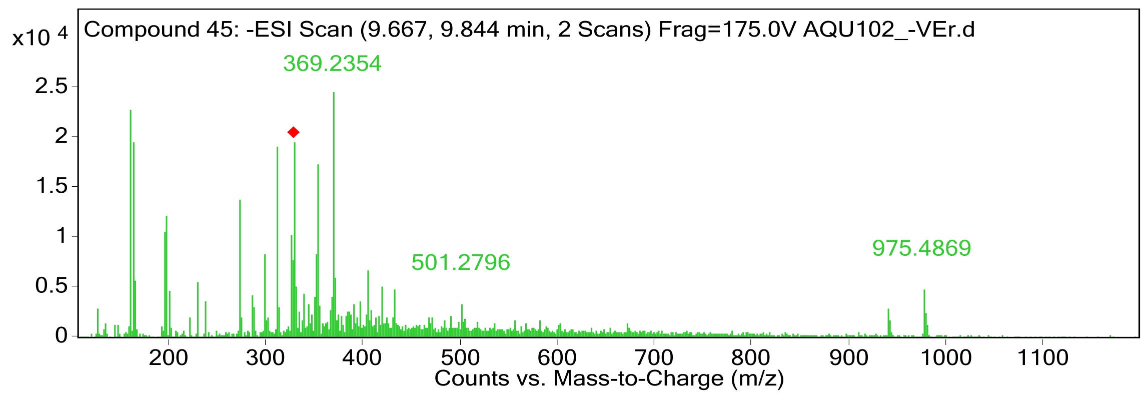

MS Zoomed Spectrum

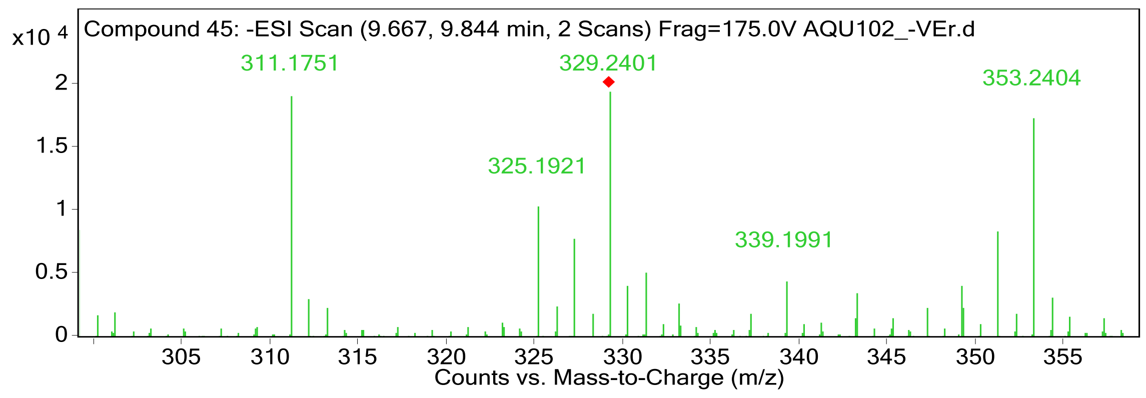

MS Spectrum Peak List

m/z	z	Abund
160.846		22766.01
162.8432		19475.49
195.8154		10645.93
197.8127		12181.87
273.1768	1	13788.16
311.1751	1	19066.96
329.2401	1	19473.62
330.2438	1	4108.31
353.2404	1	17332.47
369.2354	1	24582.78

MSMS Spectrum

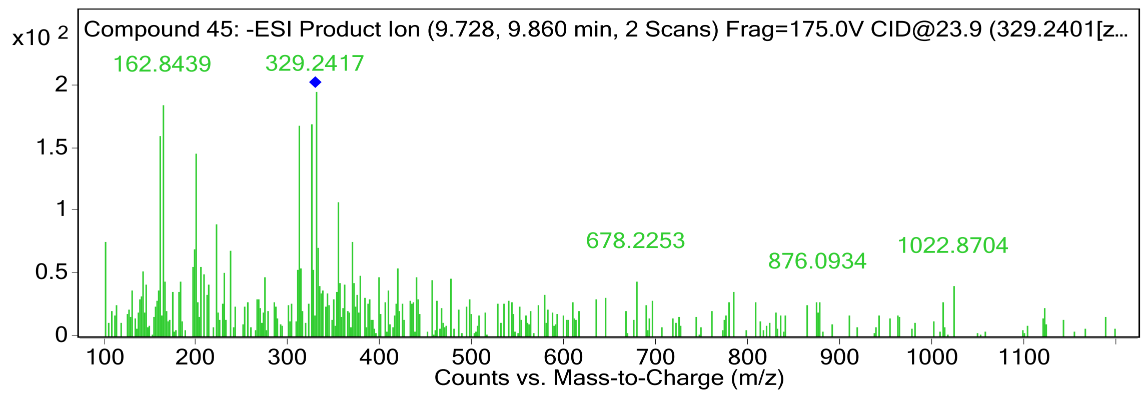

MS/MS Spectrum Peak List

m/z	z	Abund
101.0061		76.31
160.8457		160.49
162.8439		185.1
199.138		145.86
221.1229		89.5
311.1765	1	168.79
325.1896	1	169.53
329.2417	1	195.63
330.2445	1	81.27
353.2416		108.04

Compound Label	*m/z*	RT	Algorithm
Compound 46	353.2407	10.238	Auto MS/MS

MS Spectrum

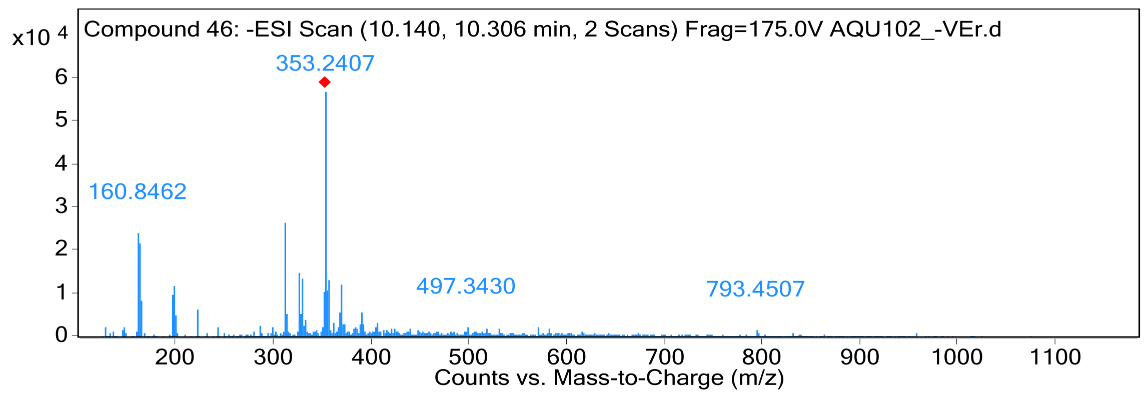

MS Zoomed Spectrum

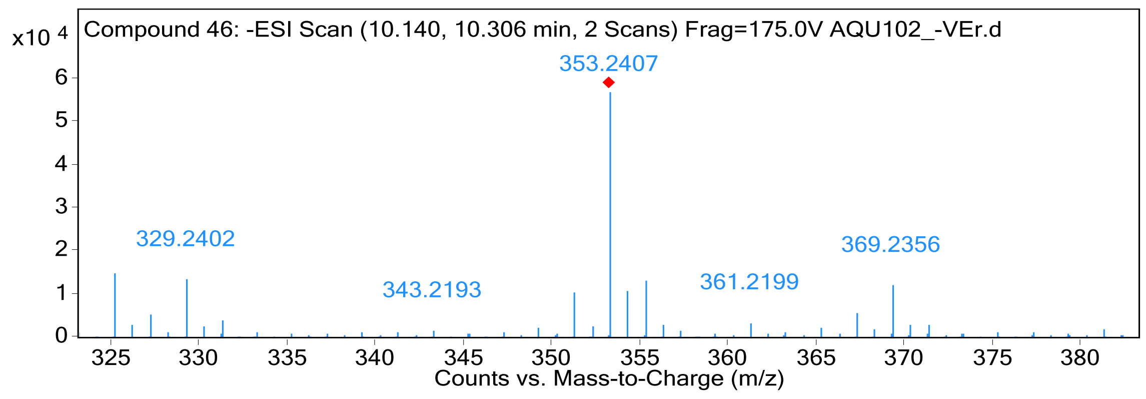

MS Spectrum Peak List

m/z	z	Abund
160.8462		24339.41
162.843		21692.3
197.8128		12082.15
311.1754	1	26734.07
325.1916	1	14999.58
329.2402	1	13487.21
353.2407	1	56984.82
354.2438	1	11063.97
355.256	1	13266.07
369.2356	1	12148.06

MSMS Spectrum

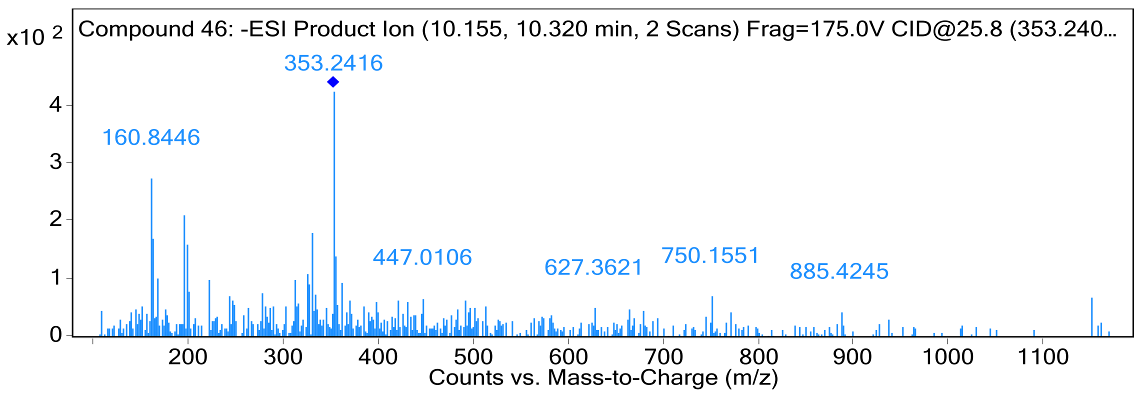

MS/MS Spectrum Peak List

m/z	z	Abund
160.8446		275.12
162.8445		170.45
167.1108		101.63
195.8171		212.18
197.8122		161.34
311.1745		100.01
325.193		108.48
329.2383		180.33
353.2416	1	425.16
354.2455	1	139.46

Compound Label	Name	m/z	RT	Algorithm	Mass
Cpd 47: Tragopogonsaponin B	**Tragopogonsaponin B**	925.4572	10.674	Auto MS/MS	926.4647

MS Spectrum

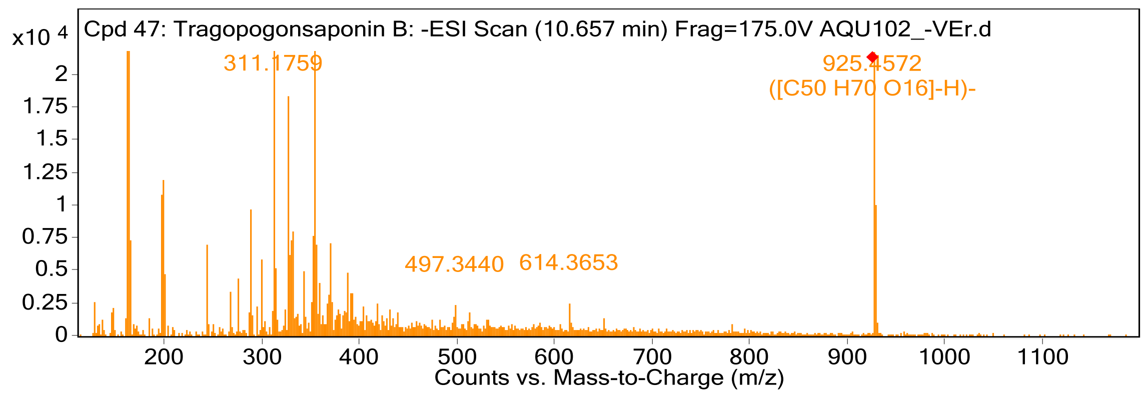

MS Zoomed Spectrum

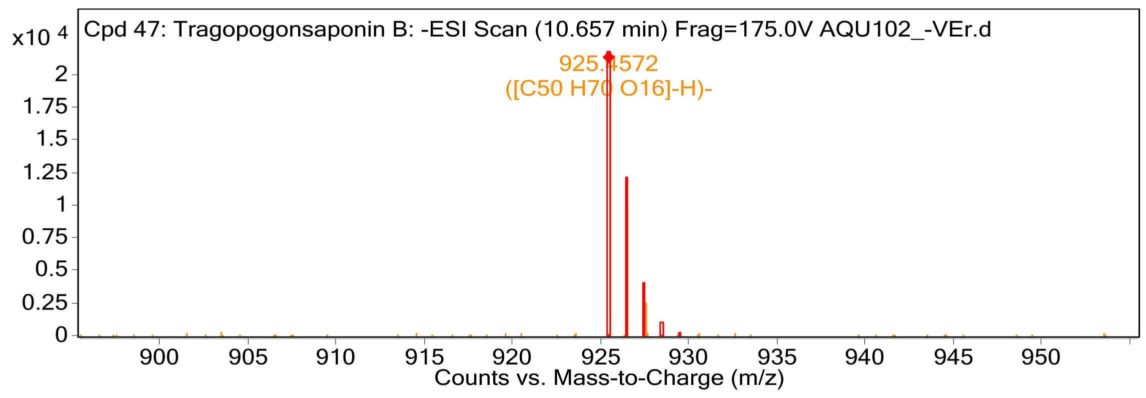

MS Spectrum Peak List

m/z	Calc m/z	Diff(ppm)	z	Abund	Formula	Ion
160.846				26372.77		
162.843				21859.99		
311.1759			1	41471.06		
311.1913				29757.93		
353.2401			1	23091.98		
925.4572	925.4591	2.05	1	21780.05	C50 H70 O16	(M-H)-
926.4618	926.4625	0.77	1	10050.59	C50 H70 O16	(M-H)-
927.4632	927.4655	2.47	1	2656.11	C50 H70 O16	(M-H)-
928.4654	928.4683	3.13	1	1167.64	C50 H70 O16	(M-H)-
929.4625	929.471	9.13	1	405.86	C50 H70 O16	(M-H)-

MSMS Spectrum

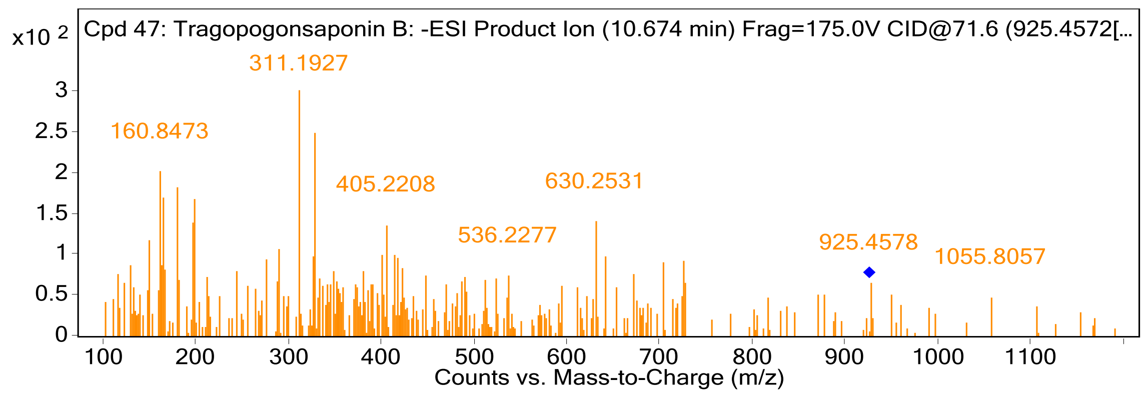

MS/MS Spectrum Peak List

m/z	Abund
160.8473	202.84
164.8395	170.72
179.0925	183.76
195.8145	140.38
197.8115	169.53
311.1761	252.72
311.1927	301.83
327.224	251.02
405.2208	137.67
630.2531	141.5

Compound Structure

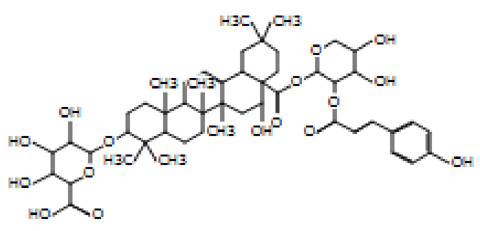

Compound Label	m/z	RT	Algorithm
Compound 48	327.2245	12.034	Auto MS/MS

MS Spectrum

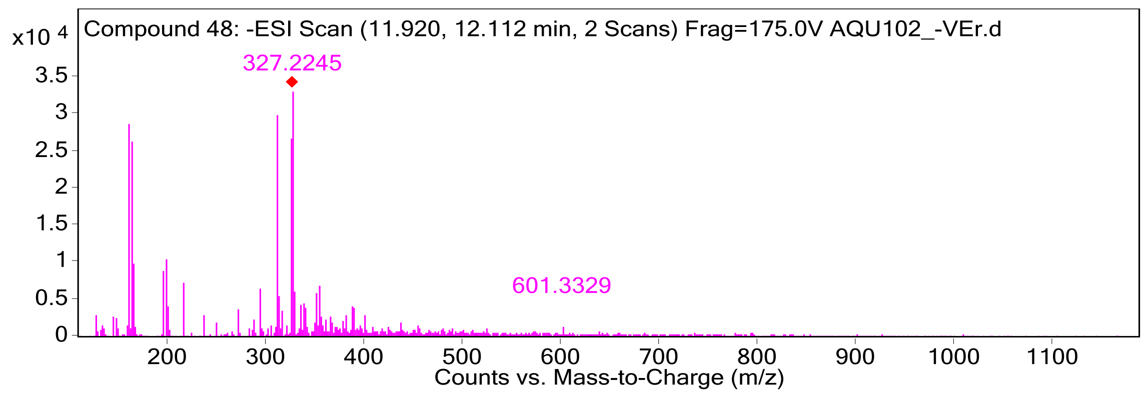

MS Zoomed Spectrum

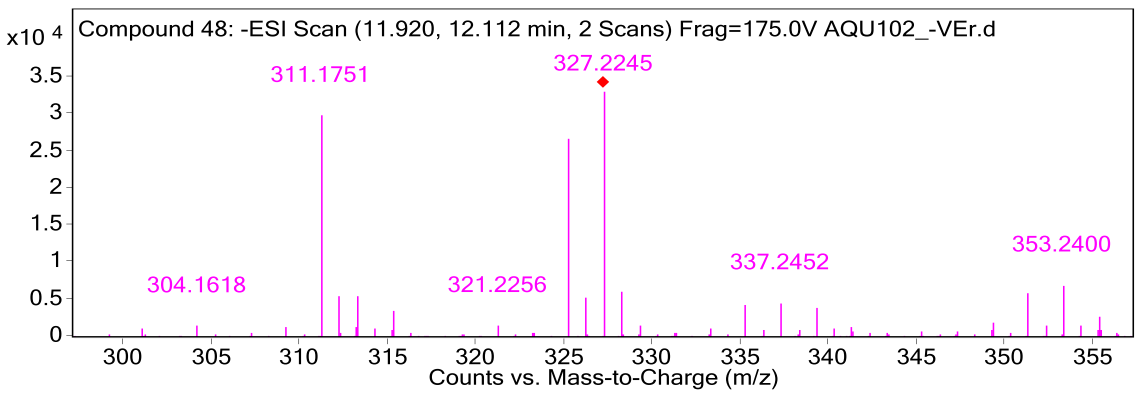

MS Spectrum Peak List

m/z	z	Abund
160.8461	1	28624.98
162.8431	1	26383.87
164.8408		9935.58
197.8128		10485.17
311.1751	1	29902.04
325.1915	1	26692.05
327.2245	1	33066.95
328.2277	1	6205.41
329.2374	1	1582.32
330.2416	1	332.24

MSMS Spectrum

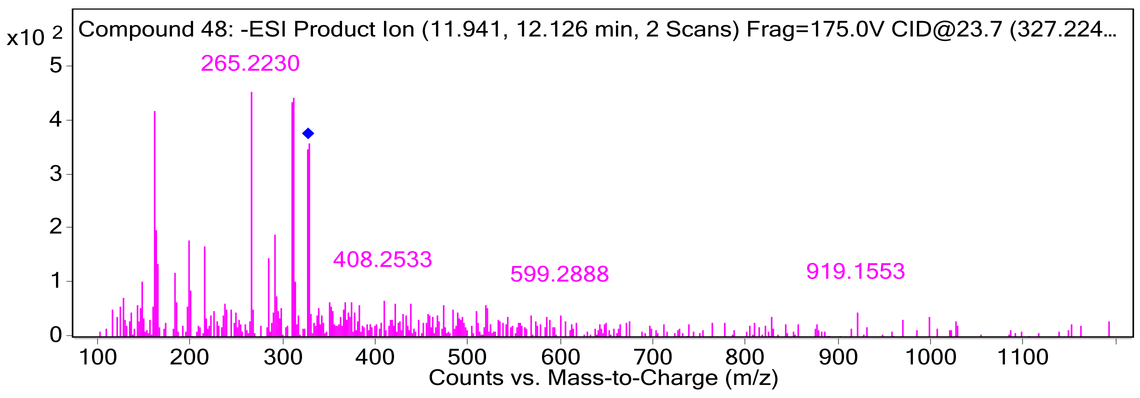

MS/MS Spectrum Peak List

m/z	z	Abund
160.8455	1	418.01
162.8425	1	199.92
265.223		455.2
291.2038	1	191.34
309.2121	1	434.38
311.1746	1	443.45
325.1878		235.51
326.1944		348.84
327.1782		182.43
327.2219	1	358.01

Compound Label	Name	m/z	RT	Algorithm	Mass
Cpd 49: alpha-Tocopherolquinone	**alpha-Tocopherolquinone**	491.3719	12.816	Auto MS/MS	446.3715

MS Spectrum

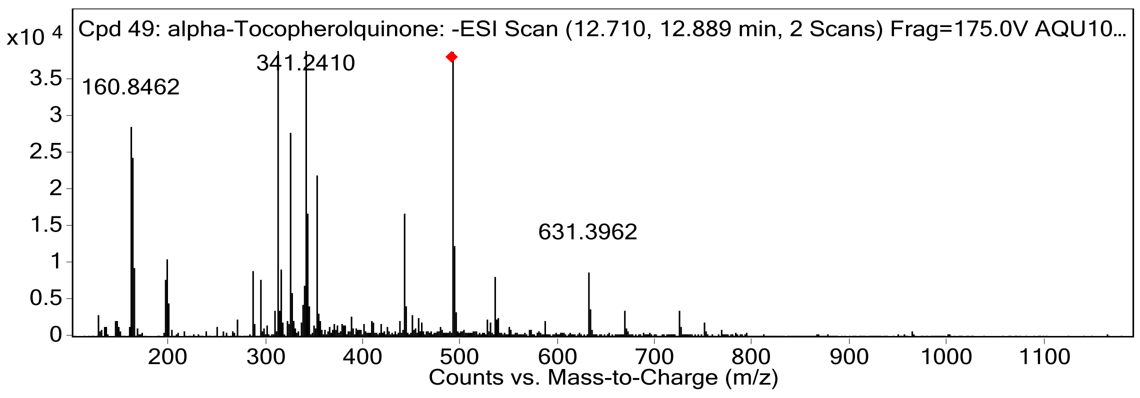

MS Zoomed Spectrum

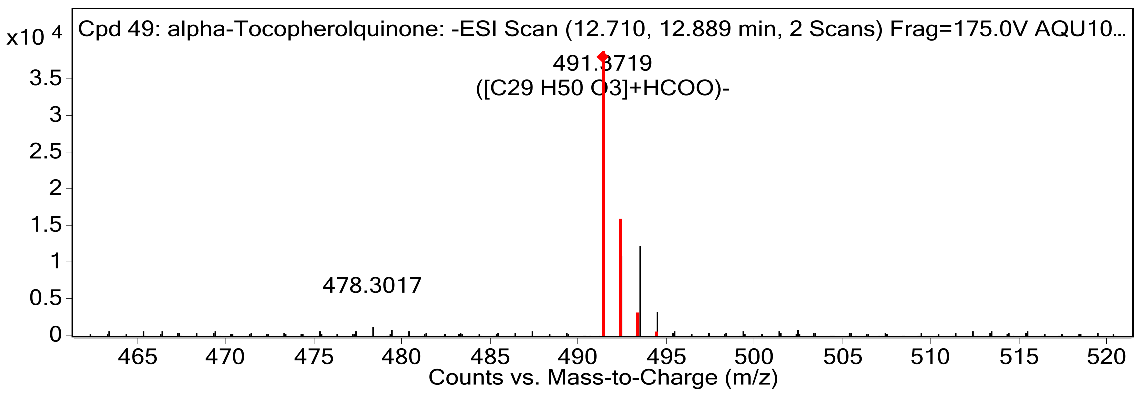

MS Spectrum Peak List

m/z	Calc m/z	Diff(ppm)	z	Abund	Formula	Ion
160.8462				28583.3		
162.8432				24387.64		
311.1754			1	76248.64		
325.1915			1	27809.12		
341.241			1	89364.03		
491.3719	491.3742	4.75	1	38881.12	C29 H50 O3	(M+HCOO)-
492.3752	492.3776	4.98	1	11044.99	C29 H50 O3	(M+HCOO)-
493.3701	493.3806	21.33	1	12433.85	C29 H50 O3	(M+HCOO)-
494.3722	494.3835	22.69	1	3453.46	C29 H50 O3	(M+HCOO)-
495.3699	495.3862	32.99	1	881.81	C29 H50 O3	(M+HCOO)-

MSMS Spectrum

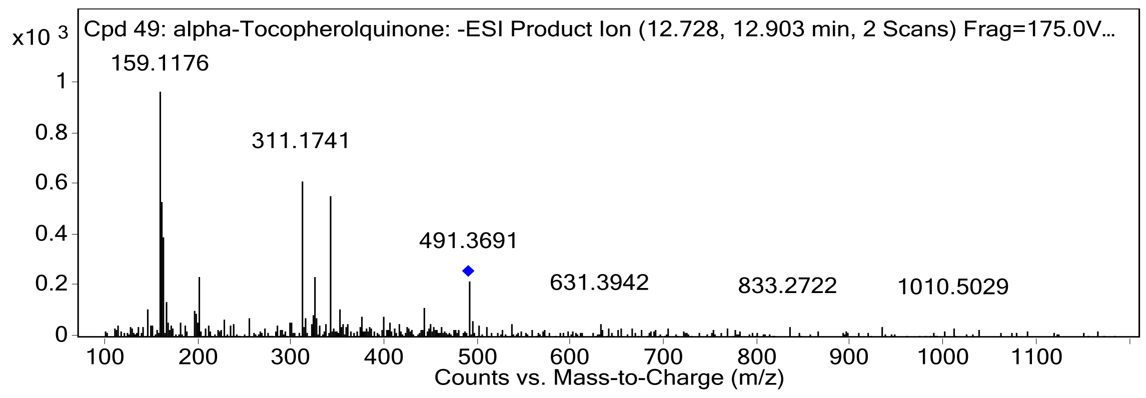

MS/MS Spectrum Peak List

m/z	z	Abund
159.1176	1	969.23
160.846		534.85
162.8441		396.99
164.8406		137.64
201.1284		240.28
311.1741	1	614.69
312.1776	1	266.88
325.1887	1	237.34
341.24	1	556.31
491.3691		219.21

Compound Structure

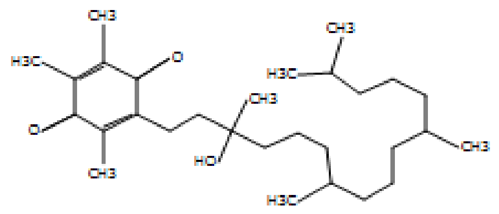

Compound Label	Name	*m/z*	RT	Algorithm	Mass
Cpd 50: alpha-Tocopherolquinone	**alpha-Tocopherolquinone**	491.3714	13.104	Auto MS/MS	446.3708

MS Spectrum

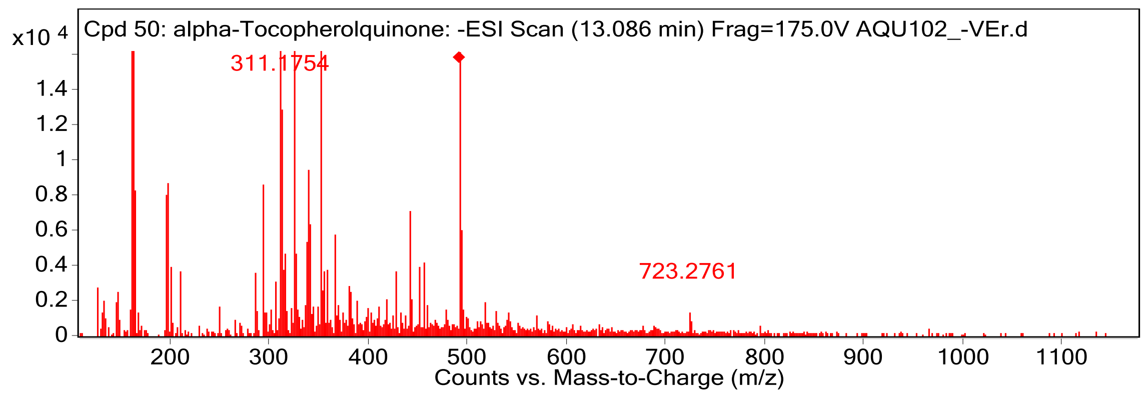

MS Zoomed Spectrum

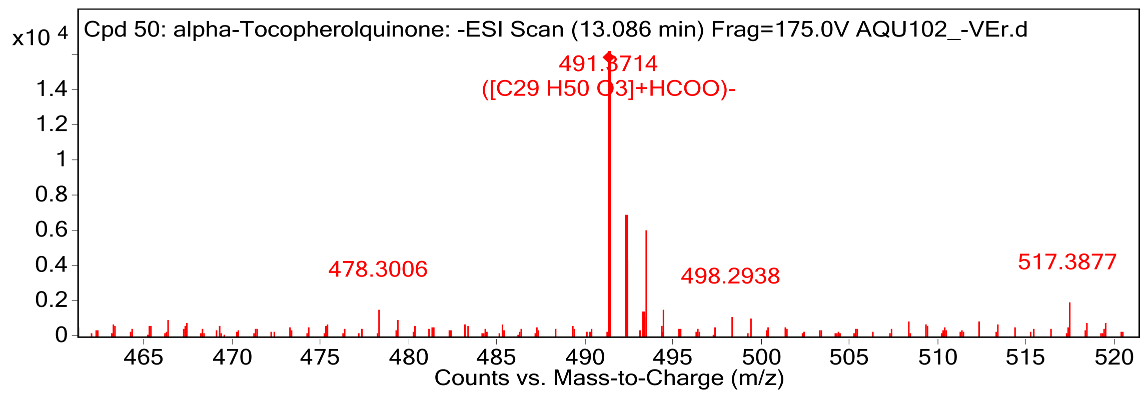

MS Spectrum Peak List

m/z	*Calc m/z*	Diff(ppm)	z	Abund	Formula	Ion
160.8461			1	28091.49		
162.8431			1	27421.62		
311.1754			1	68361.06		
325.1916			1	25280.36		
351.2285			1	28312.52		
491.3714	491.3742	5.66	1	16158.51	C29 H50 O3	(M+HCOO)-
492.375	492.3776	5.38	1	4389.02	C29 H50 O3	(M+HCOO)-
493.3692	493.3806	23.1	1	6048.94	C29 H50 O3	(M+HCOO)-
494.3722	494.3835	22.81	1	1615.25	C29 H50 O3	(M+HCOO)-
495.3747	495.3862	23.26	1	537.22	C29 H50 O3	(M+HCOO)-

MSMS Spectrum

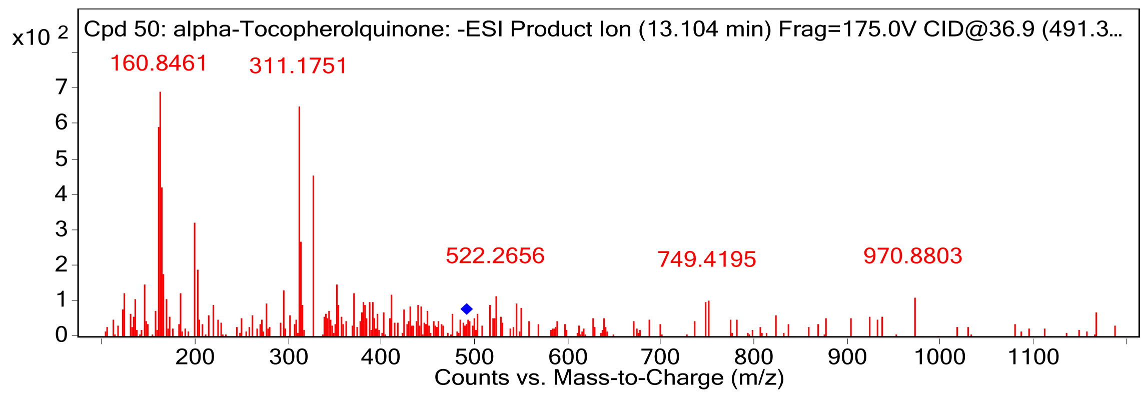

MS/MS Spectrum Peak List

m/z	z	Abund
144.8744		151.36
159.1177		594.77
160.8461		694.35
162.8437		422.71
164.8409		178.3
197.8133		322.63
201.1285		189.34
311.1751	1	652.2

312.1803	1	271.39
325.1909		457.01

Compound Structure

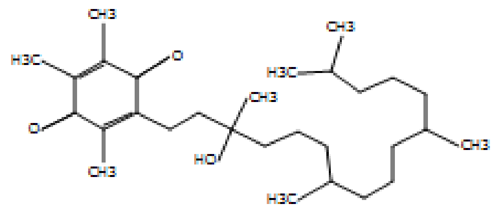

Compound Label	*m/z*	RT	Algorithm
Compound 51	426.9767	13.263	Auto MS/MS

MS Spectrum

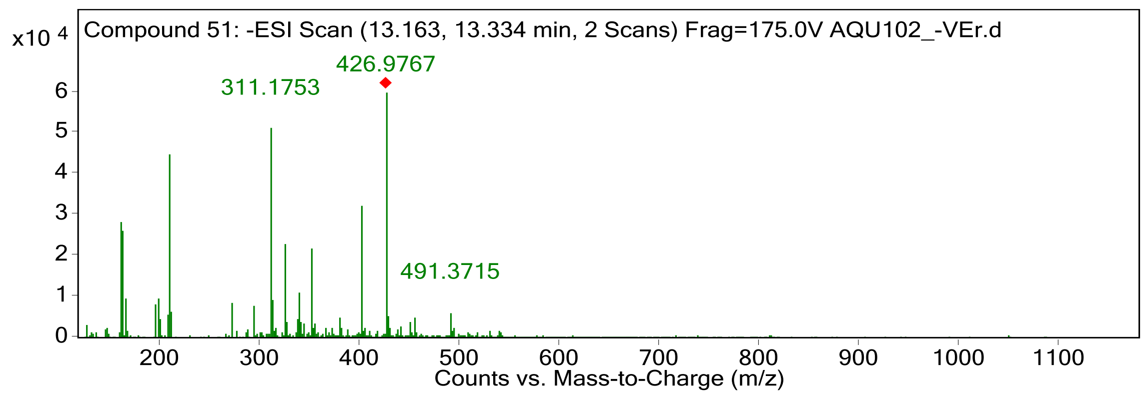

MS Zoomed Spectrum

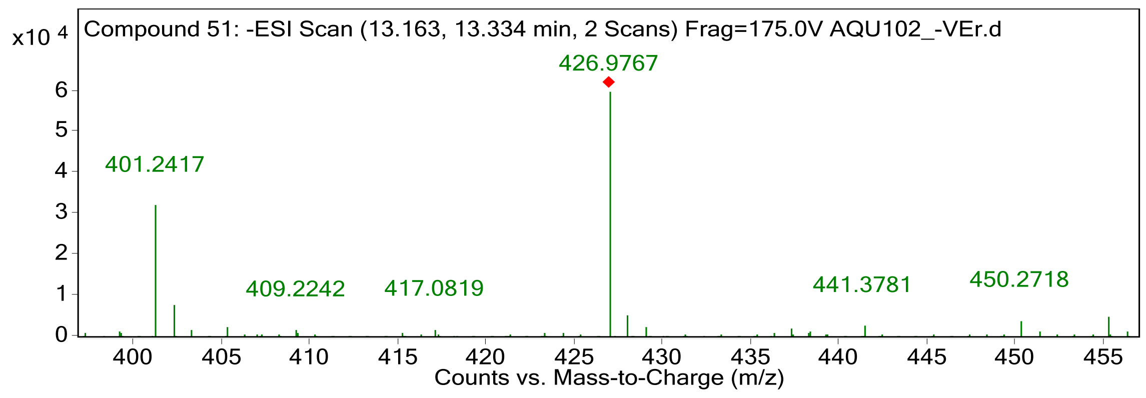

MS Spectrum Peak List

m/z	z	Abund
160.8462		28313.92
162.8433		26329.26
209.1229	1	44654.86
311.1753	1	51268.71
325.1913	1	22803.38
351.2283	1	21957.89
401.2417	1	32222.29
426.9767	1	59923.79
427.9801	1	5405.52
428.9734	1	2523.79

MSMS Spectrum

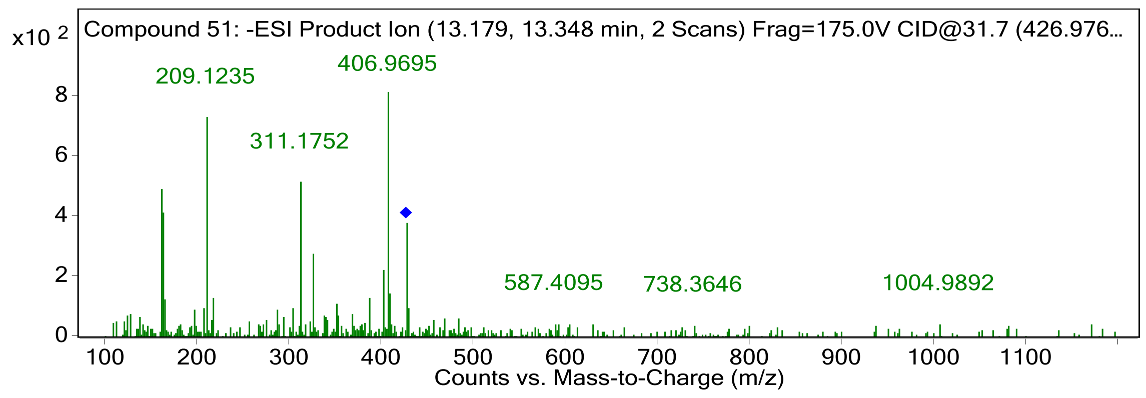

MS/MS Spectrum Peak List

m/z	z	Abund
160.8459		491.46
162.8424		415.4
209.1235	1	732.69
311.1752		516.43
325.1905	1	280.1
386.96		132.88
401.2424		222.81
406.9695	1	815.33
407.976	1	145.35
426.9764	1	380.6

Compound Label	Name	*m/z*	RT	Algorithm	Mass
Cpd 52: Carteolol	**Carteolol**	351.1922	13.363	Auto MS/MS	292.1781

MS Spectrum

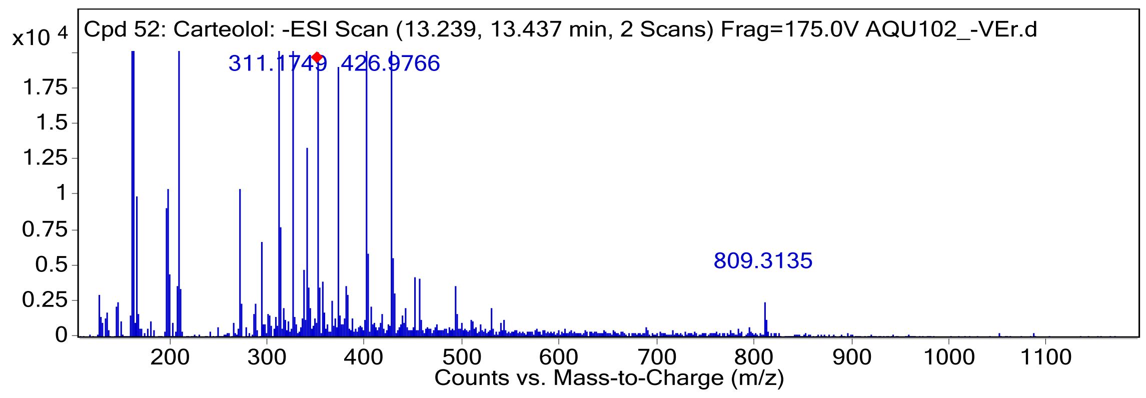

MS Zoomed Spectrum

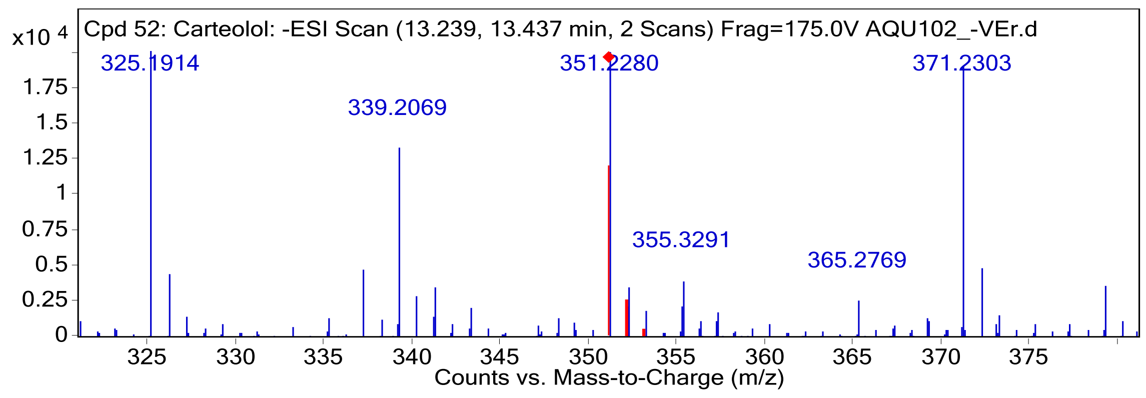

MS Spectrum Peak List

m/z	*Calc m/z*	Diff(ppm)	z	Abund	Formula	Ion
160.8461				28751.43		
162.8432			1	27876.29		
209.1228			1	31132.89		
311.1749			1	45999.92		
325.1914			1	22116.58		
351.1922	351.1925	1.09	1	11796.6	C16 H24 N2 O3	(M+CH3COO)-
352.1954	352.1957	0.8	1	2200	C16 H24 N2 O3	(M+CH3COO)-
353.1946	353.1982	10.18	1	849.5	C16 H24 N2 O3	(M+CH3COO)-
401.2413			1	23891.04		
426.9766			1	64579.05		

MSMS Spectrum

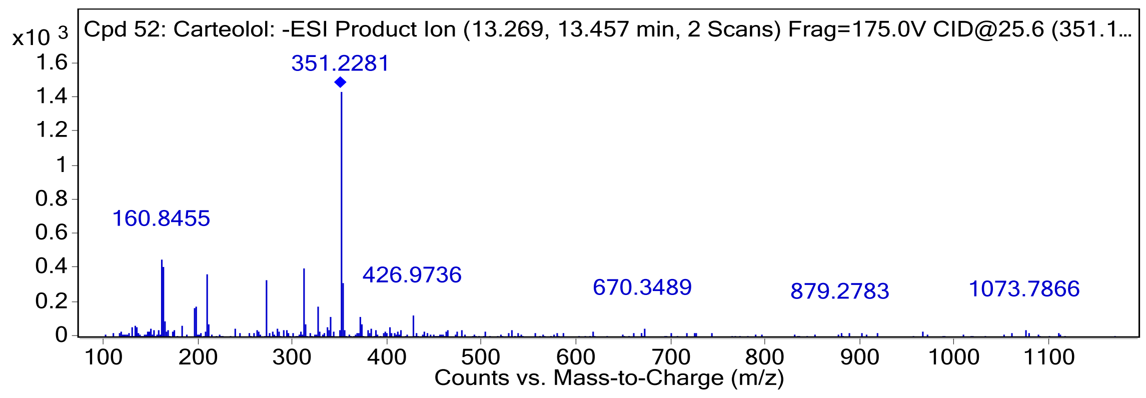

MS/MS Spectrum Peak List

m/z	z	Abund
160.8455		457.04
162.8423		409.35
197.8135		181.34
209.1223	1	367.17
271.2331	1	333.43
311.1737	1	406.3
325.1919		180.11
351.1912		325.46
351.2281	1	1438.36
352.2323	1	314.6

Compound Structure

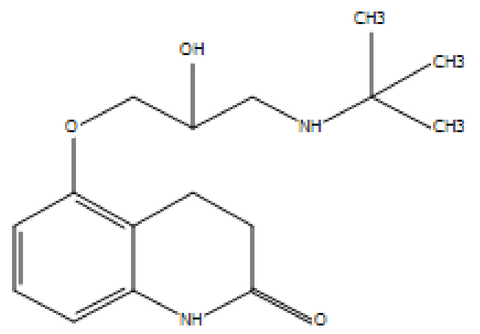

Compound Label	*m/z*	RT	Algorithm
Compound 53	209.123	13.396	Auto MS/MS

MS Spectrum

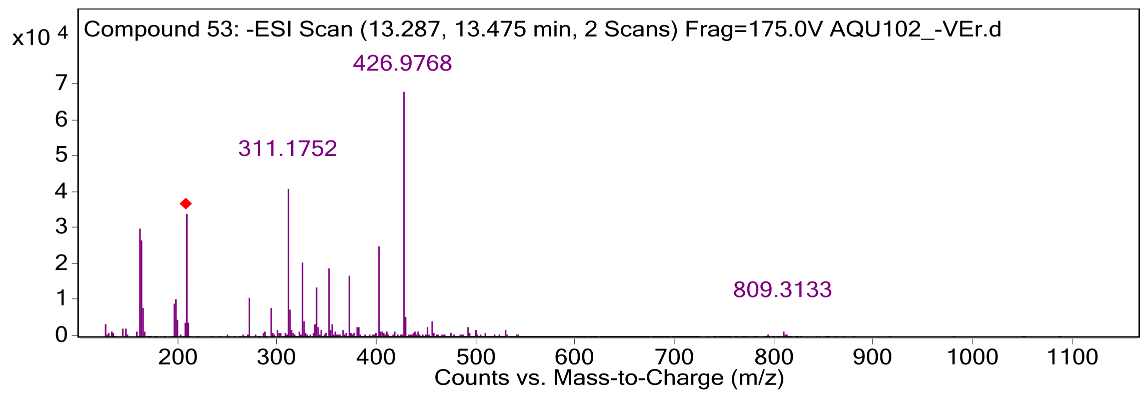

MS Zoomed Spectrum

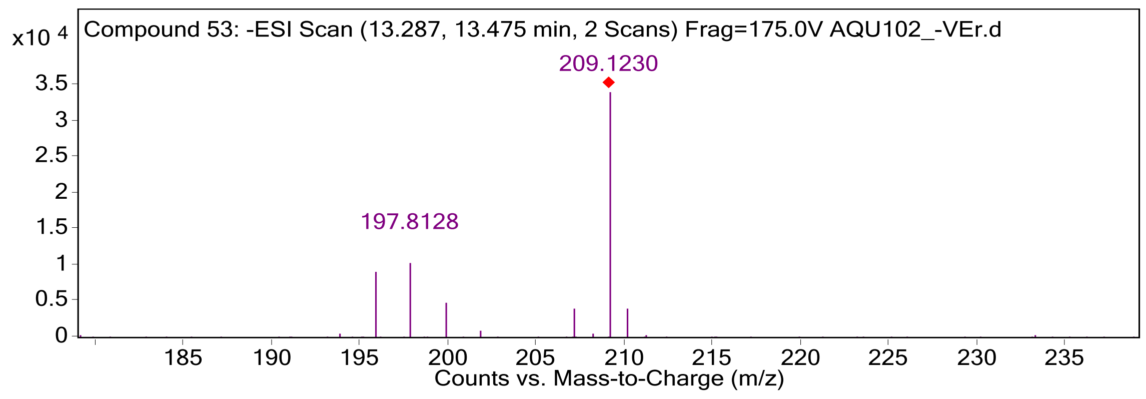

MS Spectrum Peak List

m/z	z	Abund
160.8463	1	30072.51
162.8432	1	27010.39
209.123	1	34114.54
210.1262	1	4092.49
311.1752	1	40990.45
325.1914	1	20554.2
351.2281	1	19146.72
371.231	1	17003.19
401.2413	1	25218.7

426.9768	1	68023.9

MSMS Spectrum

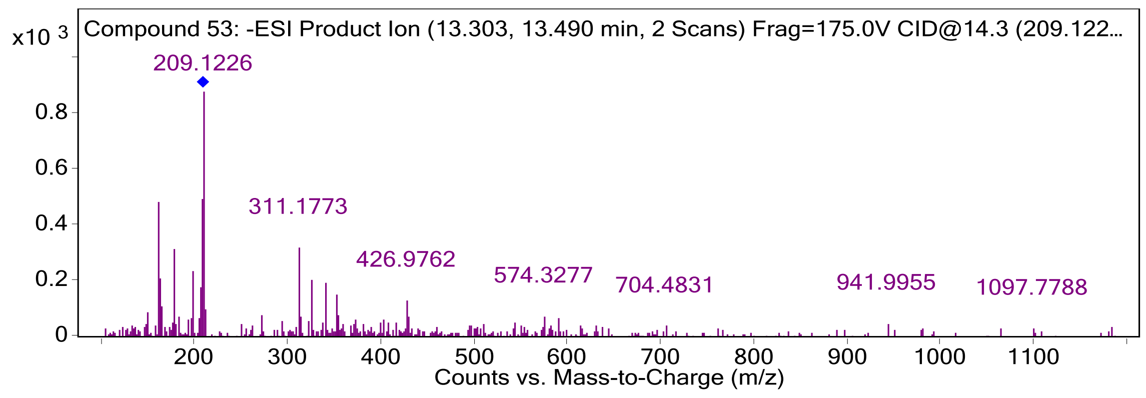

MS/MS Spectrum Peak List

m/z	z	Abund
160.8455		484.13
162.8438		211.31
177.0961		316.25
197.8132		235.18

m/z	z	Abund
205.0935	1	178.73
207.1075	1	493.56
209.1226	1	881.68
311.1773	1	322.17
325.1907		206.03
339.2048	1	195.7

Compound Label	Name	*m/z*	RT	Algorithm	Mass
Cpd 54: Hydroxystenozole	**Hydroxystenozole**	371.2305	13.503	Auto MS/MS	326.232

MS Spectrum

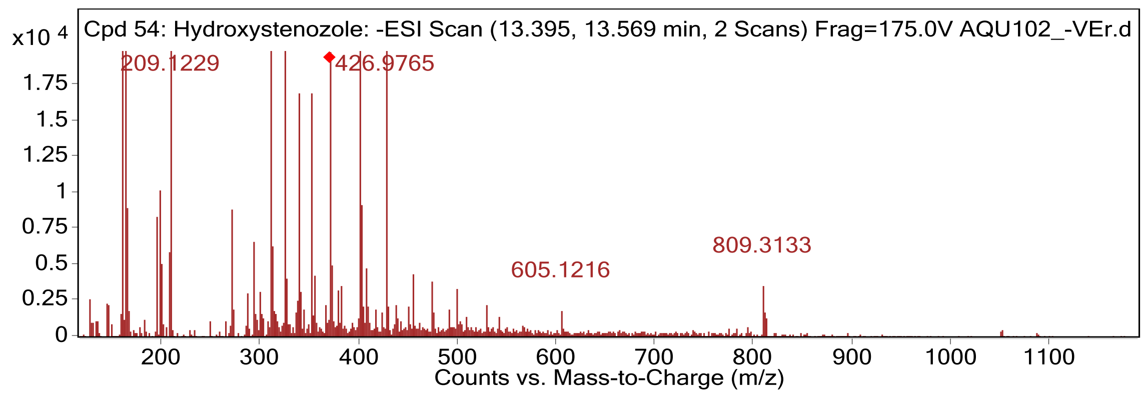

MS Zoomed Spectrum

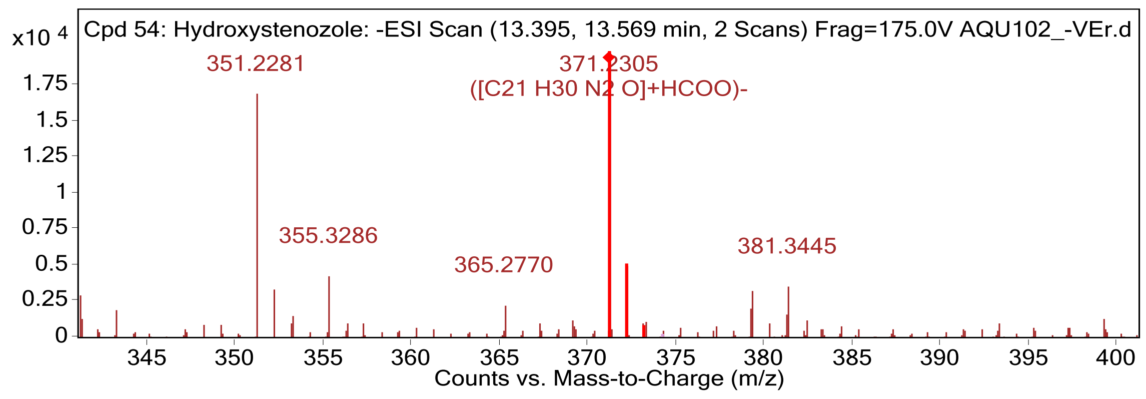

MS Spectrum Peak List

m/z	*Calc m/z*	Diff(ppm)	z	Abund	Formula	Ion
160.8462			1	27776.56		
162.8433			1	25110.04		
209.1229			1	48271.79		
311.1749			1	35035.43		
325.1914			1	21121.76		
371.2305	371.234	9.5	1	19762.14	C21 H30 N2 O	(M+HCOO)-
372.2335	372.2372	9.9	1	5003.47	C21 H30 N2 O	(M+HCOO)-
373.2293	373.2401	28.92	1	1072.48	C21 H30 N2 O	(M+HCOO)-
401.2415			1	38812.72		
426.9765			1	45188.54		

MSMS Spectrum

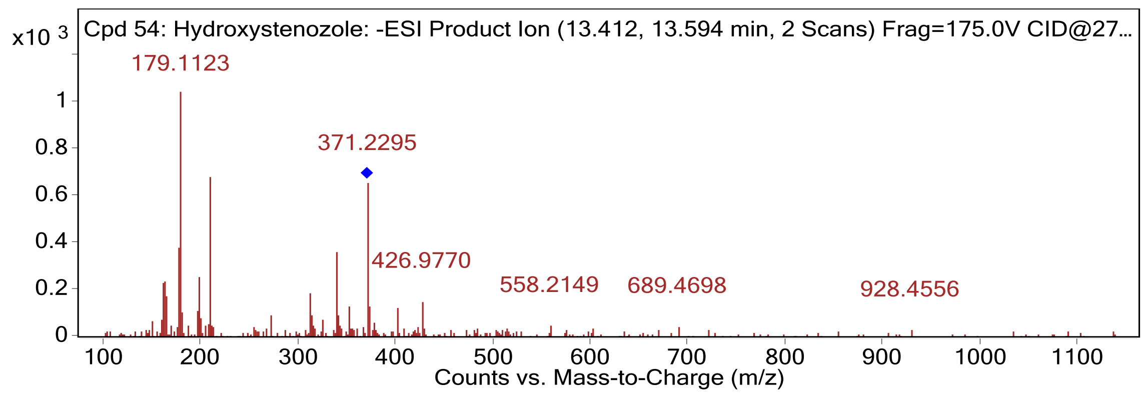

MS/MS Spectrum Peak List

m/z	z	Abund
160.8462		231.74
162.8434		235.19
164.8409		174.11
177.0956		379.48
179.1123	1	1046.28
197.8125		258.72
209.1233	1	681.35
311.175	1	185.92
339.2069	1	365.53
371.2295	1	655.35

Compound Structure

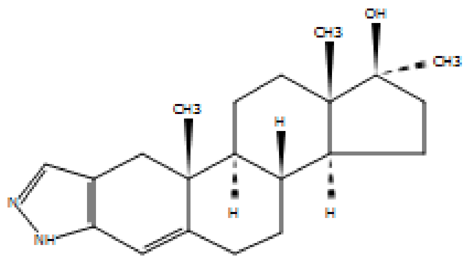

Compound Label	*m/z*	RT	Algorithm
Compound 55	426.9767	13.528	Auto MS/MS

MS Spectrum

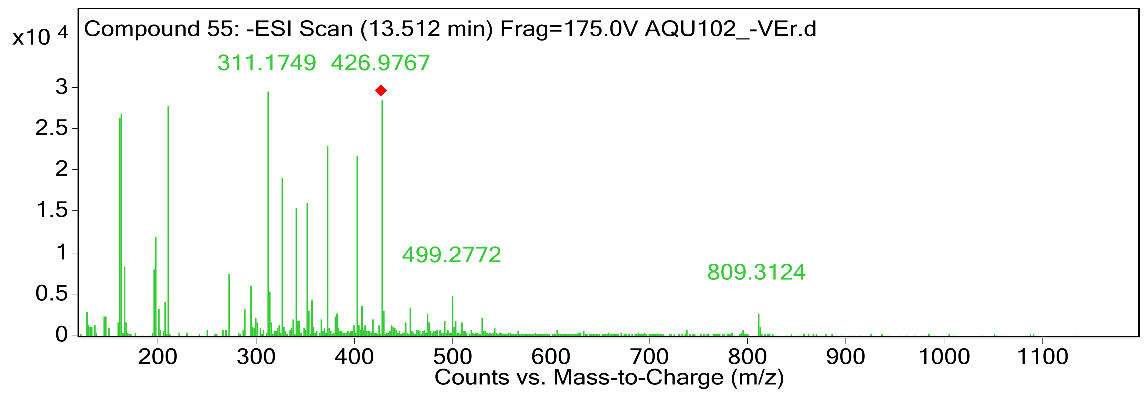

MS Zoomed Spectrum

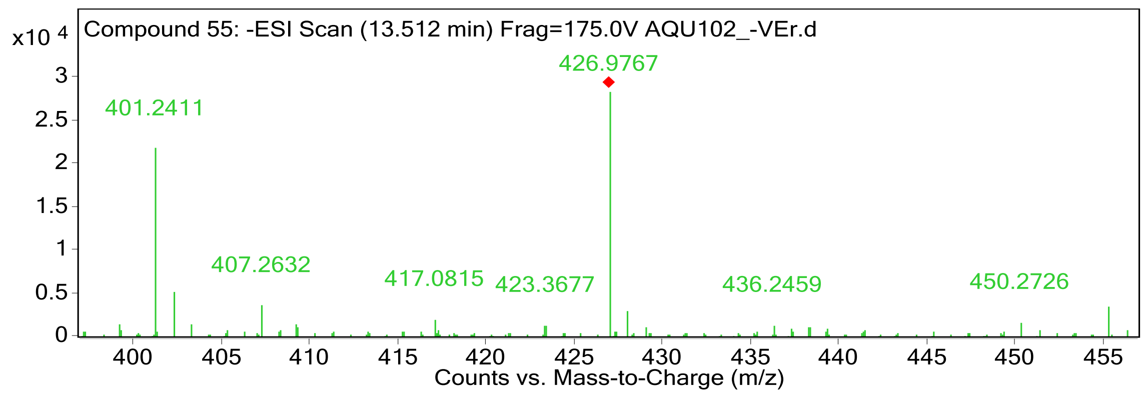

MS Spectrum Peak List

m/z	z	Abund
160.846	1	26473.73
162.8433	1	27005.65
209.123	1	27740.69
311.1749	1	29644.12
325.1913	1	19116.31
371.2306	1	22953.18
401.2411	1	21844.96
426.9767	1	28435.03
427.979	1	3111.26
428.9729	1	1211.42

MSMS Spectrum

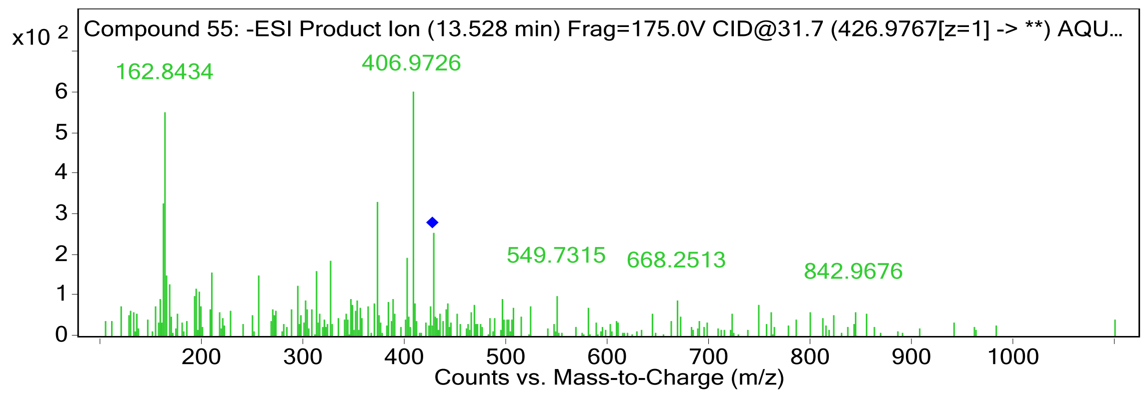

MS/MS Spectrum Peak List

m/z	z	Abund
160.8462		327.36
162.8434		553.47
209.1228		157.67
254.9898		151.5
311.1785		162.99
326.1967		187.3
371.2315		331.9
401.2424		193.92
406.9726	1	605.18
426.9791		257.34

Compound Label	Name	m/z	RT	Algorithm	Mass
Cpd 56: Bryodulcosigenin	**Bryodulcosigenin**	473.3606	13.719	Auto MS/MS	474.3654

MS Spectrum

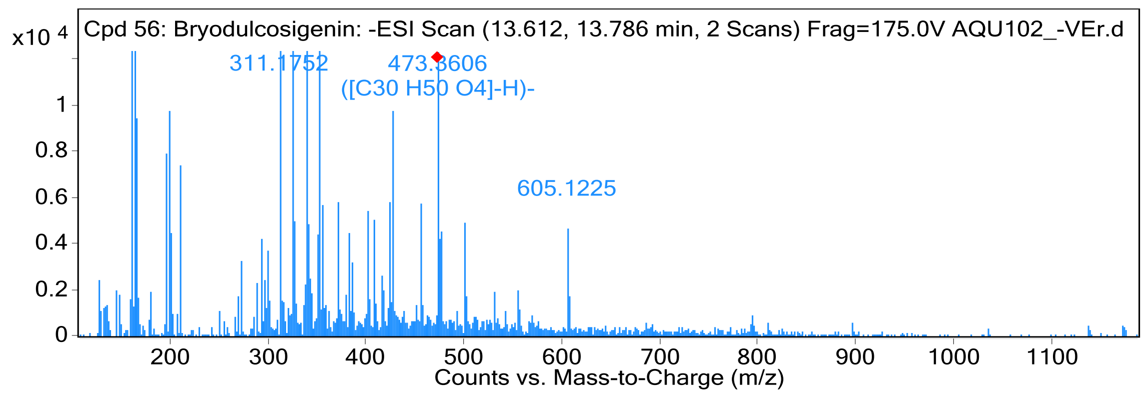

MS Zoomed Spectrum

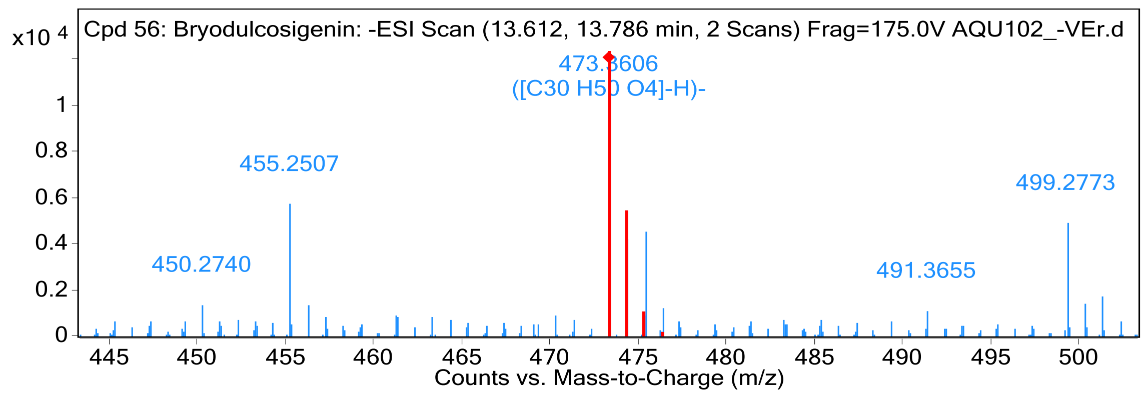

MS Spectrum Peak List

m/z	Calc m/z	Diff(ppm)	z	Abund	Formula	Ion
160.8462			1	29354.14		
162.8431			1	25781.73		
311.1752			1	24842.3		
325.1915			1	24248.82		
339.2069			1	22592.83		
473.3606	473.3636	6.46	1	12327.55	C30 H50 O4	(M-H)-
474.3636	474.367	7.28	1	4232.98	C30 H50 O4	(M-H)-
475.3588	475.3701	23.88	1	4584.68	C30 H50 O4	(M-H)-

476.3603	476.373	26.79	1	1286.02	C30 H50 O4	(M-H)-
477.3619	477.3758	29.24	1	430.83	C30 H50 O4	(M-H)-

MSMS Spectrum

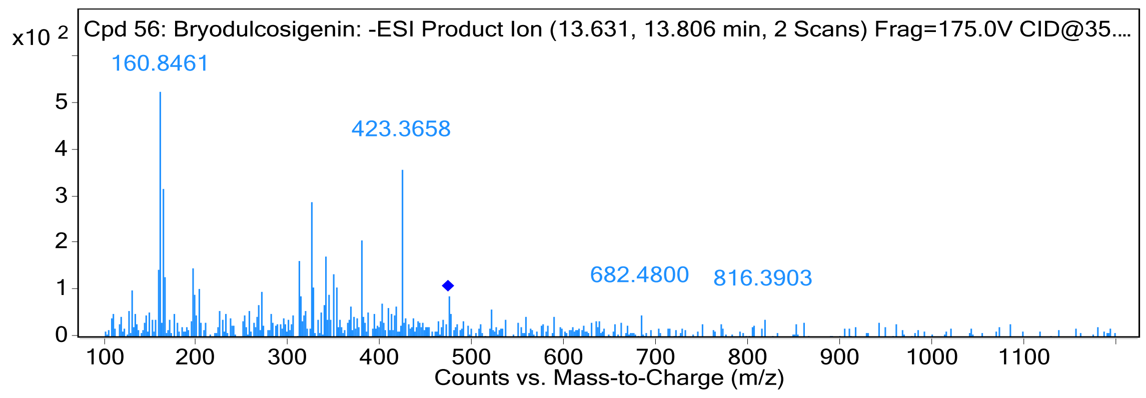

MS/MS Spectrum Peak List

m/z	z	Abund
159.118		145.37
160.8461		526.59
162.8417		318.76
195.8143		148.61
311.174		162.65
325.1905	1	290.82
339.2069		172.87
349.2564		134.78
379.3759	1	208.41
423.3658		359.64

Compound Structure

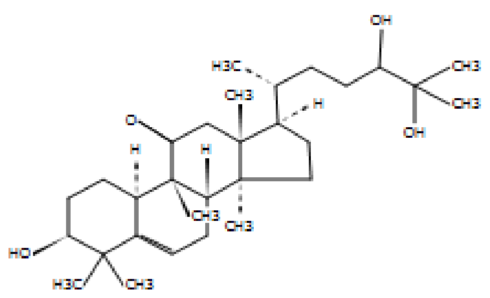

**Compound Label**	**Name**	***m/z***	**RT**	**Algorithm**	**Mass**
Cpd 57: Hydroquinidine	**Hydroquinidine**	325.1914	14.195	Auto MS/MS	326.1984

MS Spectrum

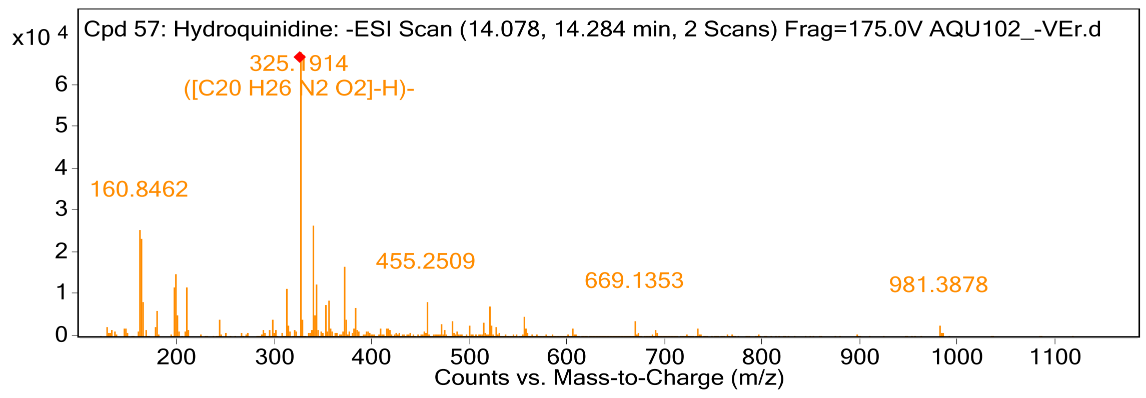

MS Zoomed Spectrum

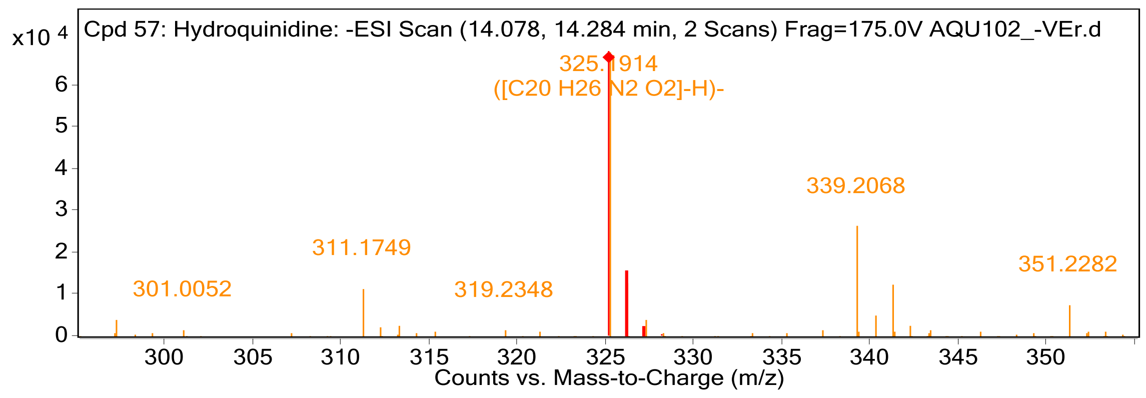

MS Spectrum Peak List

m/z	*Calc m/z*	Diff(ppm)	z	Abund	Formula	Ion
160.8462			1	25587.68		
162.8432			1	23543.05		
197.8128				15127.3		
325.1914	325.1922	2.32	1	68034.74	C20 H26 N2 O2	(M-H)-
326.1943	326.1953	3.11	1	13067.71	C20 H26 N2 O2	(M-H)-
327.1932	327.1982	15.31	1	4232.54	C20 H26 N2 O2	(M-H)-
328.195	328.2009	18.06	1	947.7	C20 H26 N2 O2	(M-H)-
339.2068			1	26478.67		
341.2188			1	12472.84		
371.2306			1	16913.25		

MSMS Spectrum

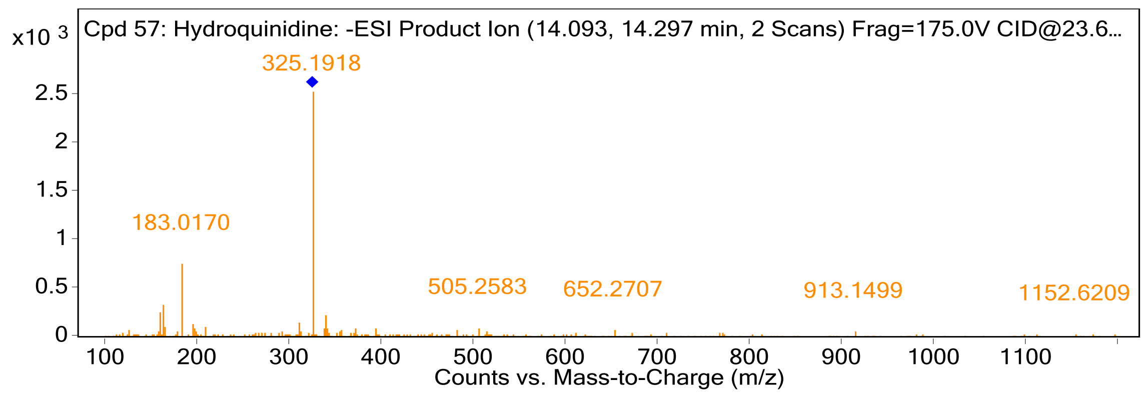

MS/MS Spectrum Peak List

m/z	*Calc m/z*	Diff (ppm)	z	Abund	Formula	Ion
160.8468				259.47		
162.8432				339.31		
183.017			1	762.66		
195.8153				140.95		
209.1228				110.2		
311.1762			1	158.56		

325.1918			1	2540.32		
326.1944	326.1953	3.02	1	525.3	C20 H26 N2 O2	(M-H)-
339.2098				232.11		
340.2079				142.75		

Compound Structure

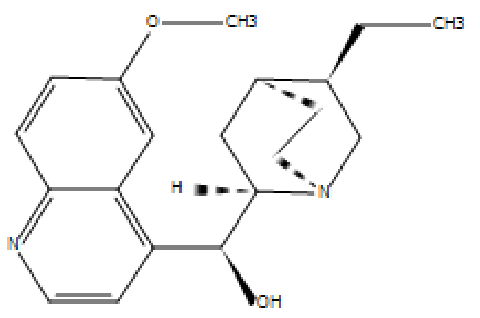

**Compound Label**	**Name**	***m/z***	**RT**	**Algorithm**	**Mass**
Cpd 58: Hydroxystenozole	**Hydroxystenozole**	371.2305	14.279	Auto MS/MS	326.2322

MS Spectrum

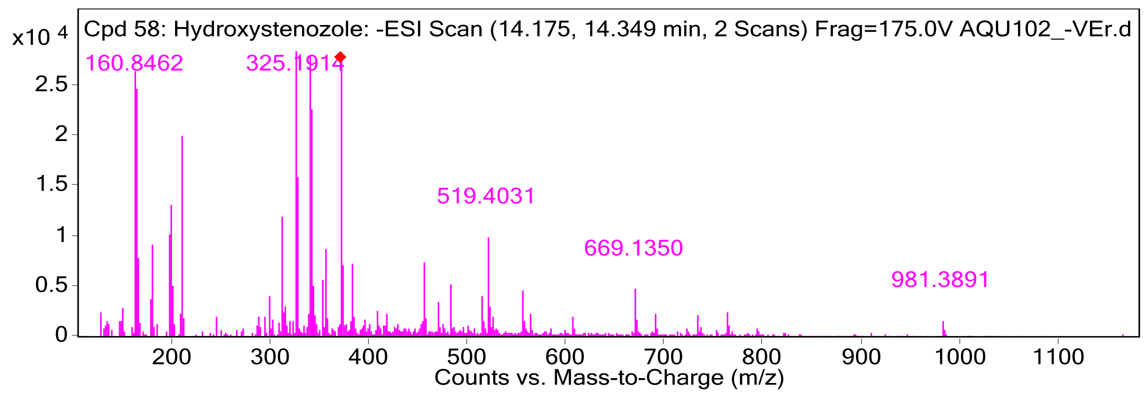

MS Zoomed Spectrum

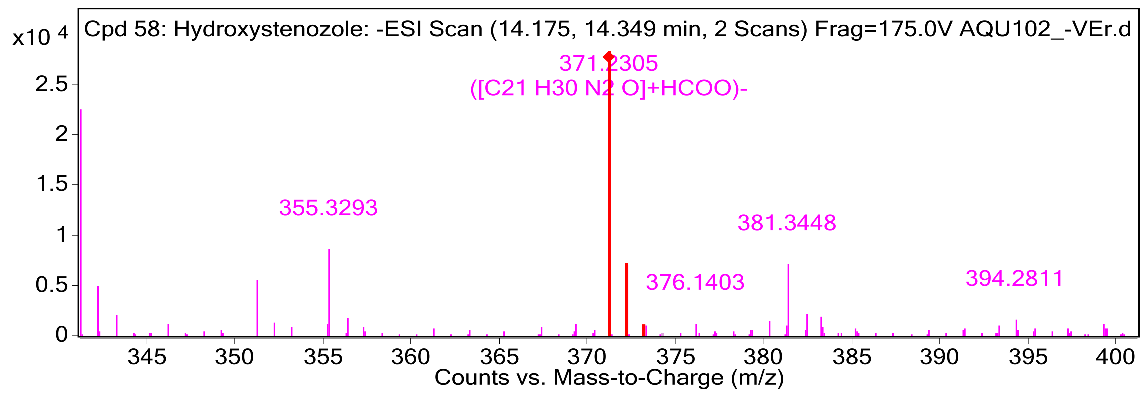

MS Spectrum Peak List

m/z	*Calc m/z*	Diff(ppm)	z	Abund	Formula	Ion
160.8462				26401.66		
162.8434				24761.84		
209.123			1	19949.99		
325.1914			1	77133.98		
326.1942			1	15910.98		
339.2068			1	27876.48		
341.2192			1	22589.09		
371.2305	371.234	9.47	1	28347.23	C21 H30 N2 O	(M+HCOO)-
372.2335	372.2372	10.06	1	7180.16	C21 H30 N2 O	(M+HCOO)-
373.2363	373.2401	10.09	1	1152.59	C21 H30 N2 O	(M+HCOO)-

MSMS Spectrum

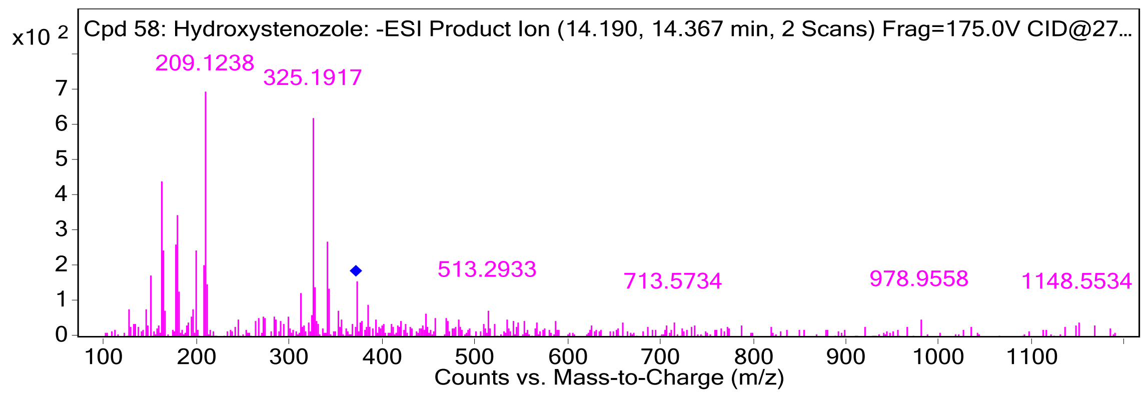

MS/MS Spectrum Peak List

m/z	z	Abund
149.1011		176.36
160.8462		441.87
162.8431	1	246.18
177.0966		262.37
179.1123		345.94
197.813	1	248.04
207.1074	1	205.89
209.1238	1	698.14
325.1917	1	622.16
339.2066		272.83

Compound Structure

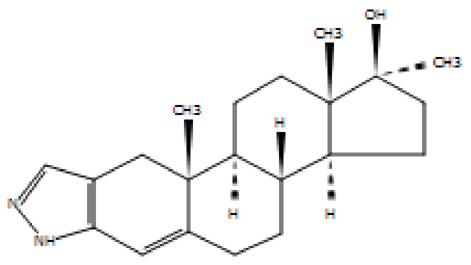

**Compound Label**	***m/z***	**RT**	**Algorithm**	**Mass**
Compound 1	289.0728	14.439	Find By Formula	290.0801

MS Spectrum

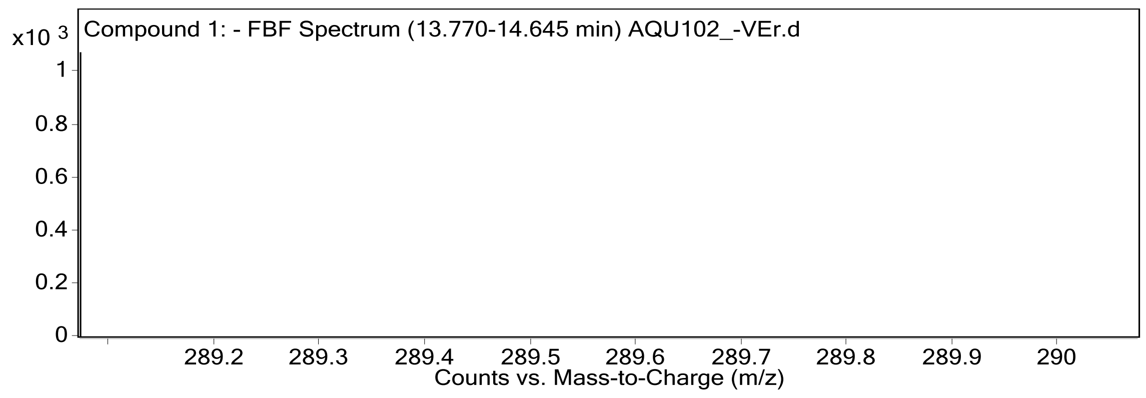

MS Zoomed Spectrum

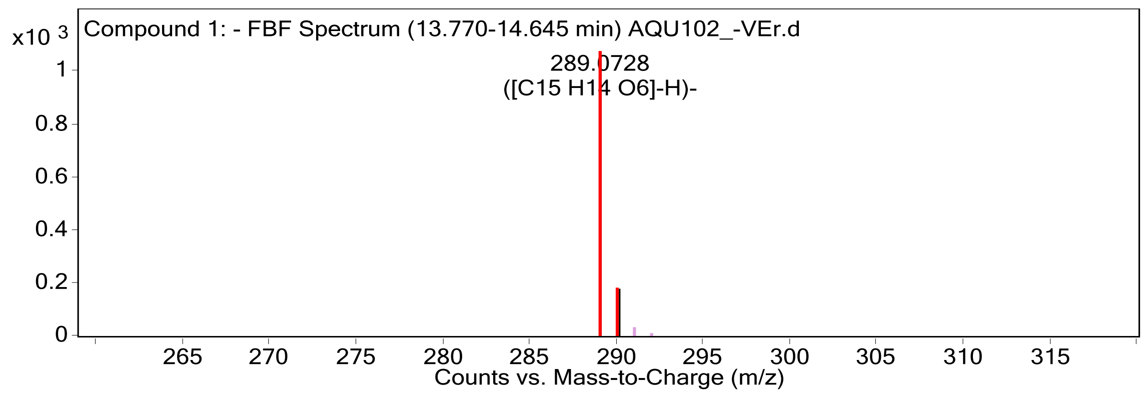

MS Spectrum Peak List

m/z	z	Abund	Formula	Ion
289.0728	1	1074.68	C15 H14 O6	(M-H)-
290.0764	1	184.78	C15 H14 O6	(M-H)-

Compound Label	Name	*m/z*	RT	Algorithm	Mass
Cpd 59: Hydroquinidine	**Hydroquinidine**	325.1914	14.574	Auto MS/MS	326.1983

MS Spectrum

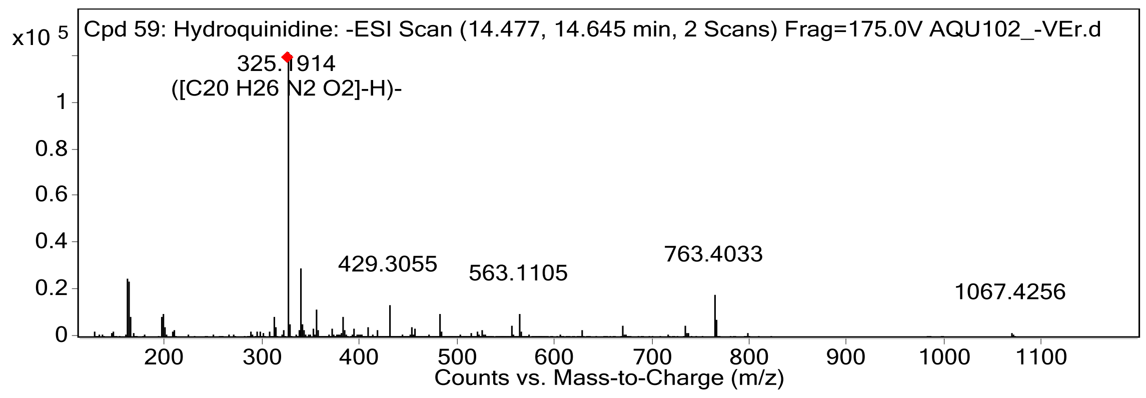

MS Zoomed Spectrum

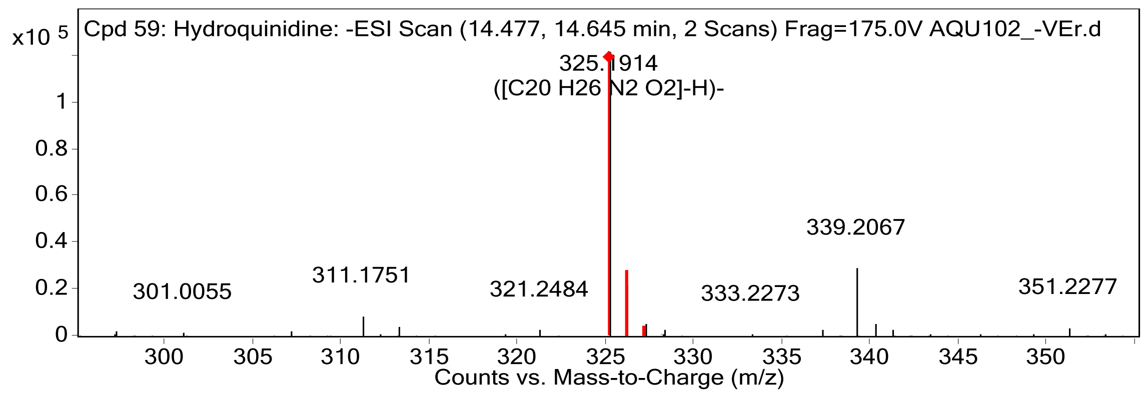

MS Spectrum Peak List

m/z	*Calc m/z*	Diff(ppm)	z	Abund	Formula	Ion
160.8461				24818.39		
162.8432				24101.94		
197.8126				10156.02		
325.1914	325.1922	2.42	1	121863.96	C20 H26 N2 O2	(M-H)-
326.1943	326.1953	3.32	1	21867.82	C20 H26 N2 O2	(M-H)-
327.1905	327.1982	23.75	1	5736.21	C20 H26 N2 O2	(M-H)-
339.2067			1	29454.93		
355.3289			1	11662.04		
429.3055			1	13793.15		
763.4033			1	18072.25		

MSMS Spectrum

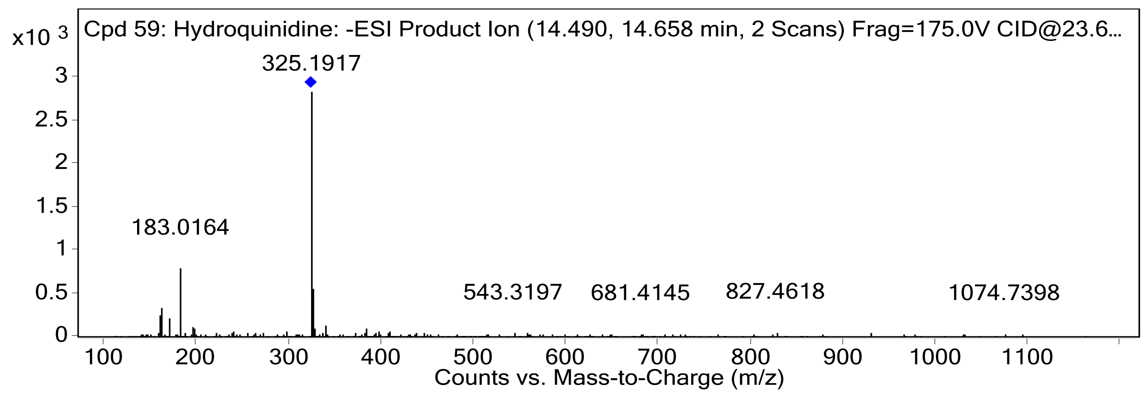

MS/MS Spectrum Peak List

m/z	z	Abund
160.8463		247.84
162.8438		338.42
170.0092	1	216.73
183.0164		799.24
195.815		114
325.1917	1	2841.2
326.1948	1	568.41
328.2566		99.02
339.2103	1	142.84
383.149		105.23

Compound Structure

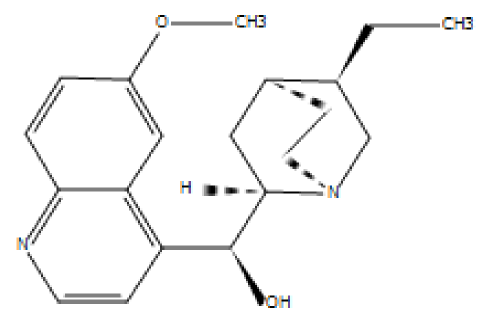
O
CH3
CH3
H
N
N
OH

Compound Label	Name	*m/z*	RT	Algorithm	Mass
Cpd 60: Schidigeragenin C	**Schidigeragenin C**	429.3053	14.628	Auto MS/MS	430.3127

MS Spectrum

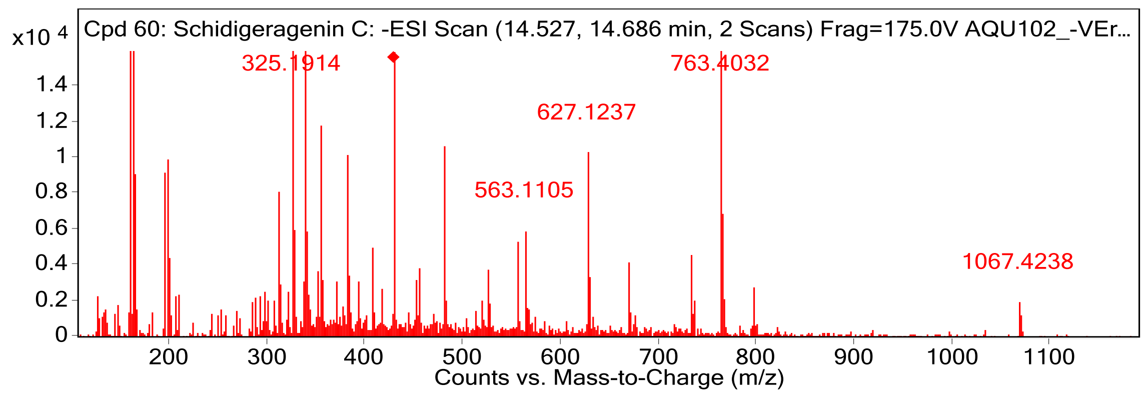

MS Zoomed Spectrum

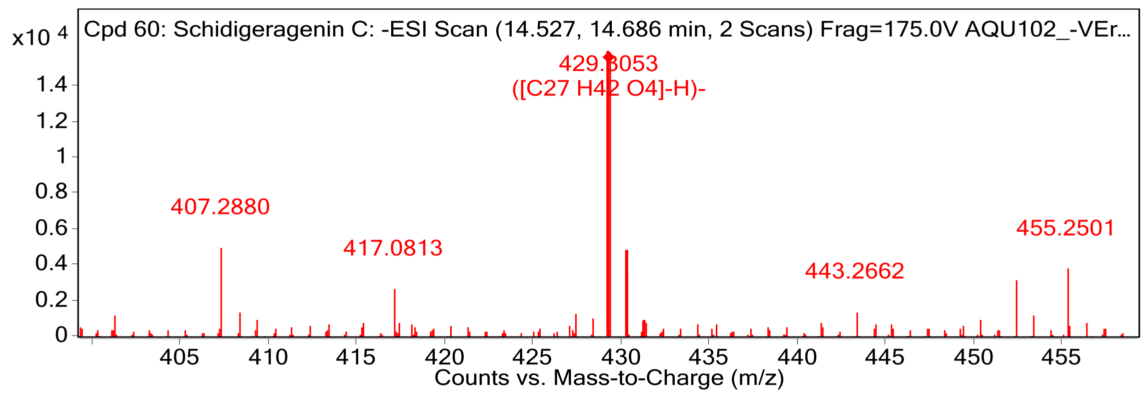

MS Spectrum Peak List

m/z	*Calc m/z*	Diff(ppm)	z	Abund	Formula	Ion
160.8461			1	25841.99		
162.8431			1	23117		
325.1914			1	130811.75		
326.194			1	24194.63		
339.2069			1	28573.83		
429.3053	429.301	-9.87	1	15895.06	C27 H42 O4	(M-H)-
430.3087	430.3044	-9.84	1	3597.83	C27 H42 O4	(M-H)-
431.314	431.3074	-15.21	1	784.69	C27 H42 O4	(M-H)-
432.3164	432.3103	-14.2	1	193.21	C27 H42 O4	(M-H)-
763.4032			1	16698.45		

MSMS Spectrum

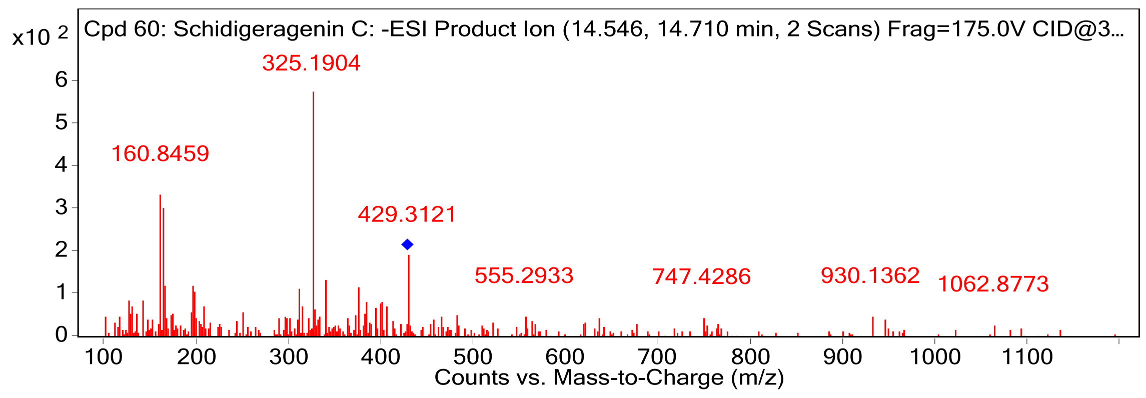

MS/MS Spectrum Peak List

m/z	z	Abund
160.8459		334.78
162.8426		301.88
164.8417		120.8
195.8151		120.33
197.8129		107
311.1735		112.86
325.1904		576.37
339.2083	1	134.03
375.3009		116.72
429.3121		192.02

Compound Structure

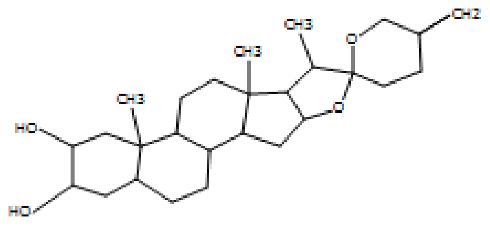

Compound Label	*m/z*	RT	Algorithm
Compound 61	328.2561	14.767	Auto MS/MS

MS Spectrum

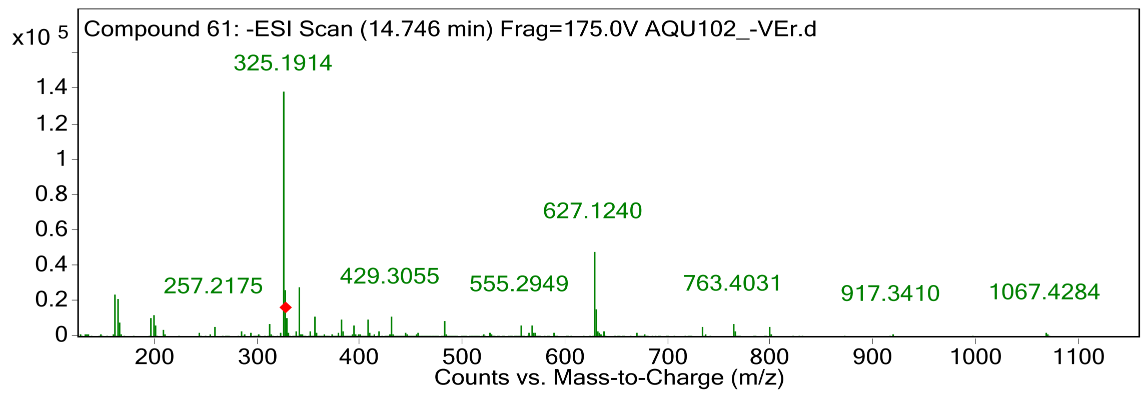

MS Zoomed Spectrum

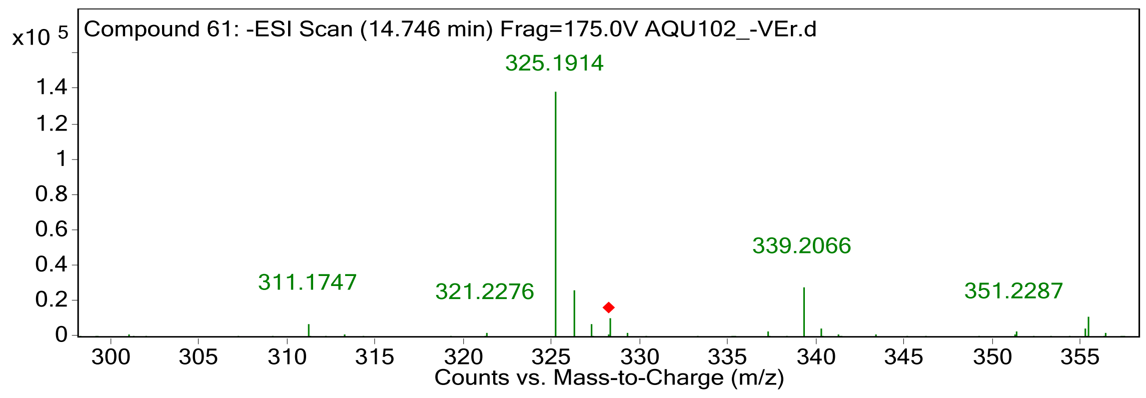

MS Spectrum Peak List

m/z	z	Abund
160.8461		23693.57
162.8433		21752.21
325.1914	1	138868.56
326.194	1	26219.29
328.2561	1	11115
329.2594	1	2395.47
330.2625	1	420.89
339.2066	1	28509.04
627.124	1	48266.32
629.1222	1	15676.19

MSMS Spectrum

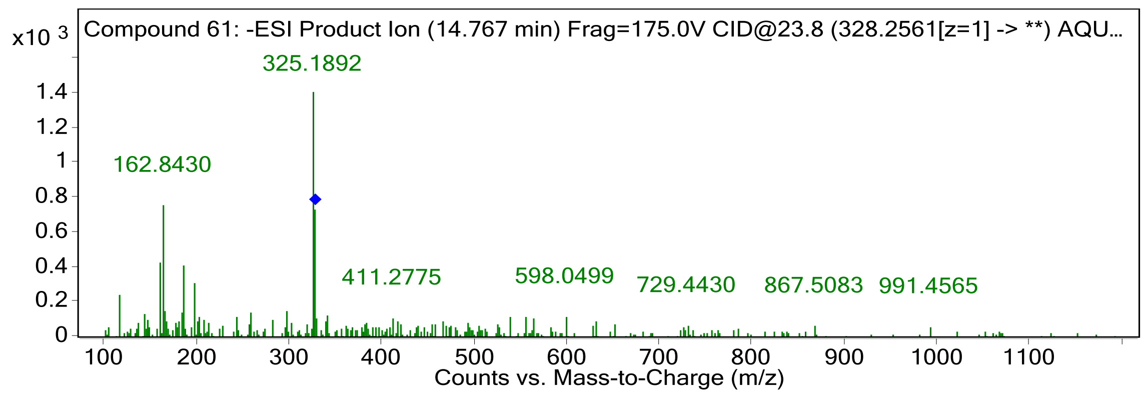

MS/MS Spectrum Peak List

m/z	z	Abund
116.0372		241.53
160.8451		430
162.843		758.65
164.8397		151.77
185.0112		411.44
197.8132		314.08
297.2498		148
325.1892	1	1410.21
326.1927	1	313.57
327.1914	1	734.03

Compound Label	Name	*m/z*	RT	Algorithm	Mass
Cpd 62: 5'-Methoxybilobetin	**5'-Methoxybilobetin**	627.1241	14.812	Auto MS/MS	582.1237

MS Spectrum

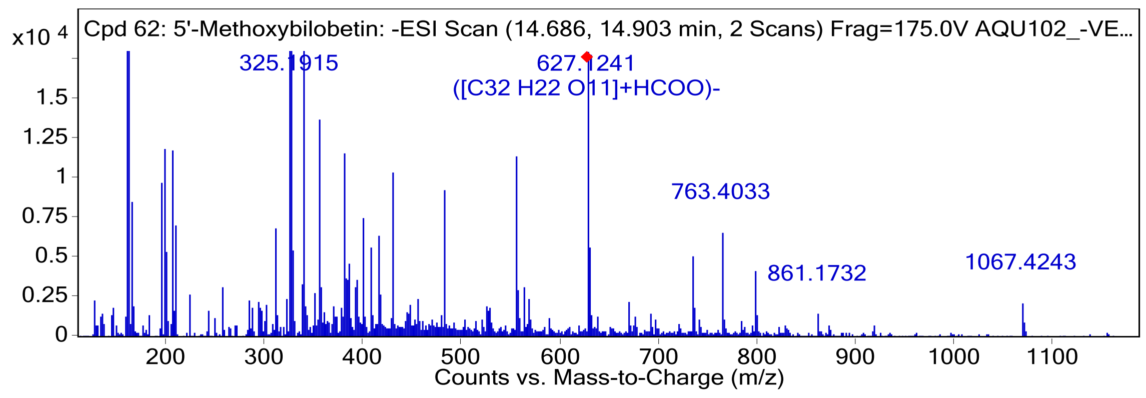

MS Zoomed Spectrum

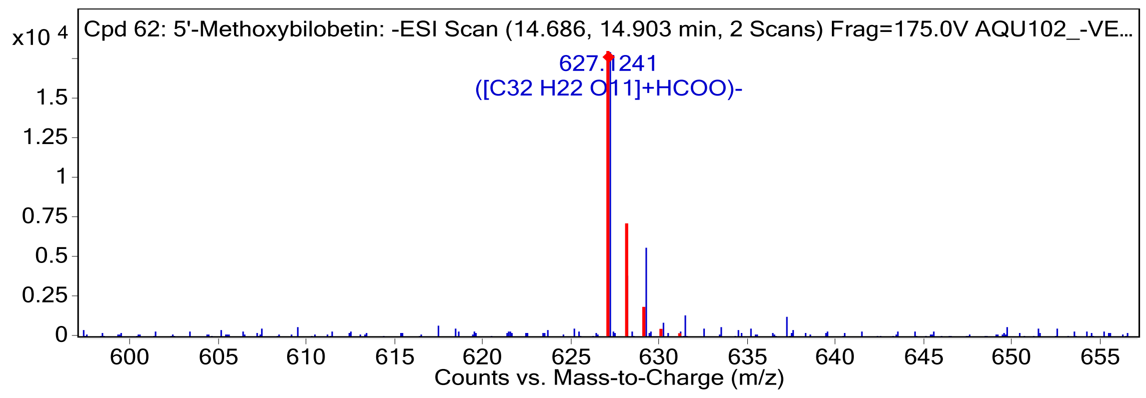

MS Spectrum Peak List

m/z	*Calc m/z*	Diff(ppm)	z	Abund	Formula	Ion
160.8461				23815.61		
162.8432				22519.51		
325.1915			1	143551.05		
326.1941			1	26675.13		
339.2068			1	28813.61		
627.1241	627.1144	-15.44	1	17927.61	C32 H22 O11	(M+HCOO)-
628.1267	628.1178	-14.22	1	3860.01	C32 H22 O11	(M+HCOO)-
629.1215	629.1205	-1.64	1	5598.93	C32 H22 O11	(M+HCOO)-
630.1245	630.1231	-2.22	1	883.26	C32 H22 O11	(M+HCOO)-
631.1279	631.1257	-3.53	1	332.31	C32 H22 O11	(M+HCOO)-

MSMS Spectrum

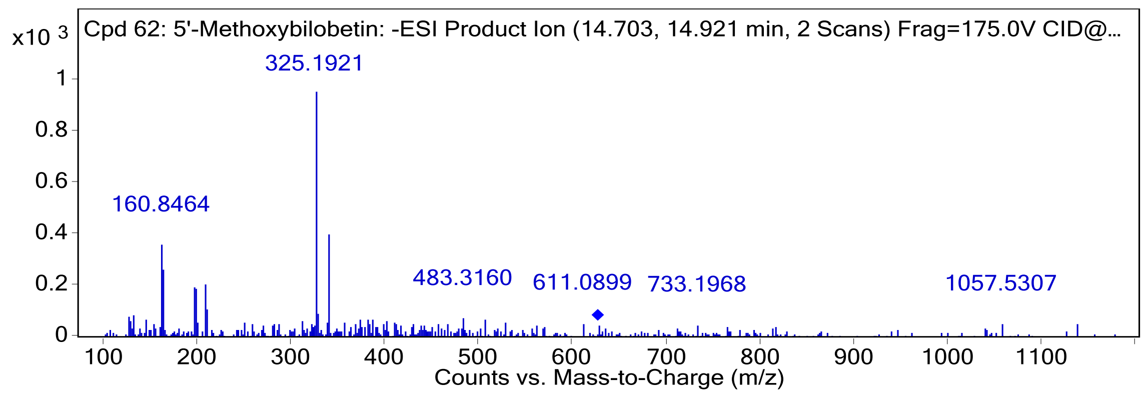

MS/MS Spectrum Peak List

m/z	z	Abund
160.8464		358.13
162.845		261.47
195.8153		193.56
197.8109	1	189.91
207.1074		205.67
209.1236		106.27
325.1921	1	953.3
326.1938	1	226.61
327.1941	1	92.78
339.2056		396.36

Compound Structure

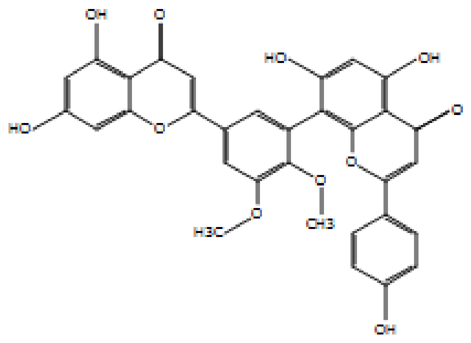

Compound Label	Name	*m/z*	RT	Algorithm	Mass
Cpd 63: Hydroquinidine	**Hydroquinidine**	325.1915	14.944	Auto MS/MS	326.1984

MS Spectrum

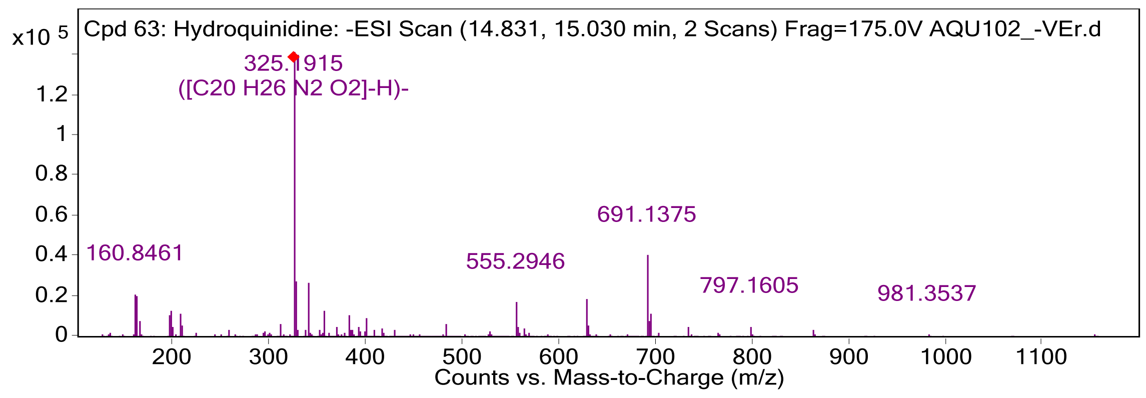

MS Zoomed Spectrum

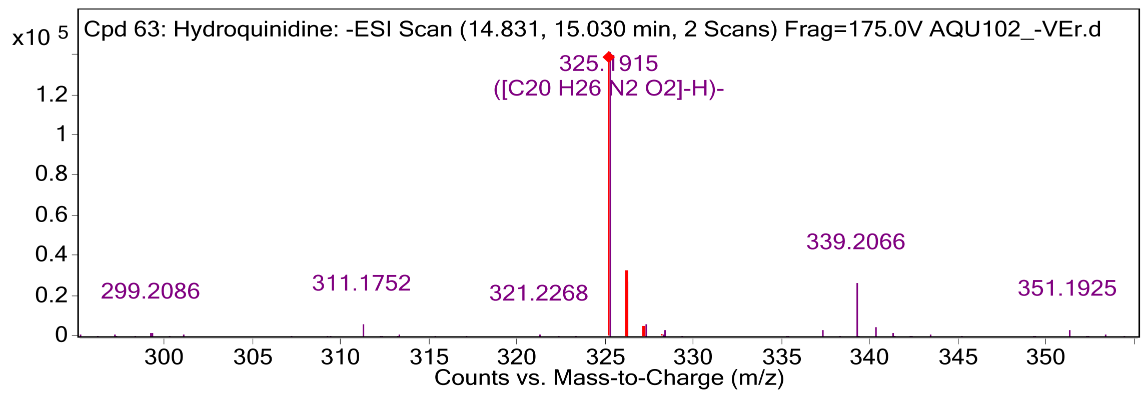

MS Spectrum Peak List

m/z	*Calc m/z*	Diff(ppm)	z	Abund	Formula	Ion
160.8461				21468.09		
162.8432				20259.61		
325.1915	325.1922	2.07	1	141587.11	C20 H26 N2 O2	(M-H)-
326.1943	326.1953	3.27	1	27964.75	C20 H26 N2 O2	(M-H)-
327.1911	327.1982	21.8	1	6802.35	C20 H26 N2 O2	(M-H)-
328.1928	328.2009	24.94	1	1437.52	C20 H26 N2 O2	(M-H)-
339.2066			1	27091.67		
555.2946			1	17277.6		
627.1242			1	18692.12		
691.1375			1	40677.1		

MSMS Spectrum

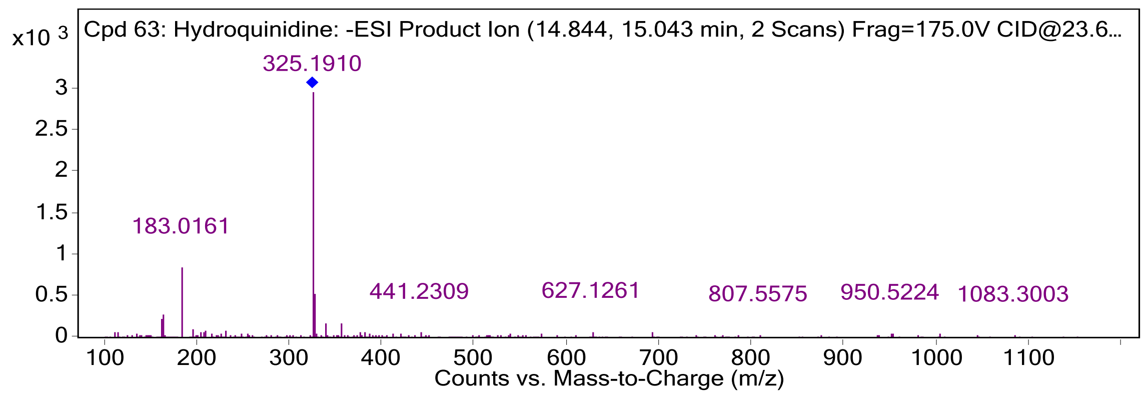

MS/MS Spectrum Peak List

m/z	z	Abund
160.8455		224.86
162.8412		277.36
183.0161	1	856.2
184.0197	1	120.91
195.815		103.86
209.1224		94.23
325.191	1	2970.18
326.1948	1	538.2
339.2054	1	172.79

355.3288	170.03

Compound Structure

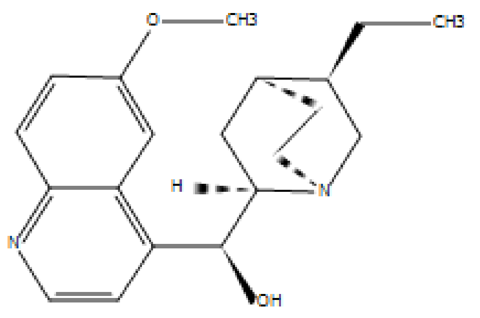

Compound Label	Name	*m/z*	RT	Algorithm	Mass
Cpd 64: UDP-2,4-bis(acetamido)-2,4,6-trideoxy-beta-L-	**UDP-2,4-bis(acetamido)-2,4,6-trideoxy-beta-L-**	691.1374	15.065	Auto MS/MS	632.1216

MS Spectrum

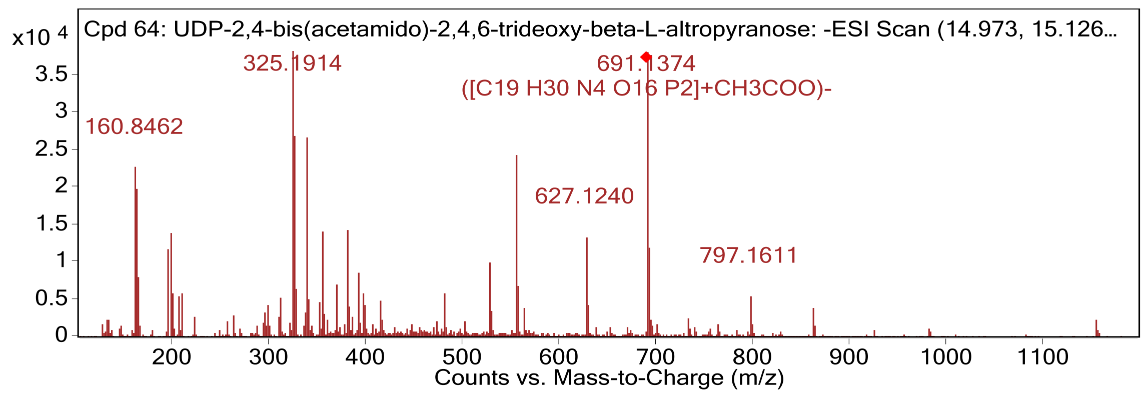

MS Zoomed Spectrum

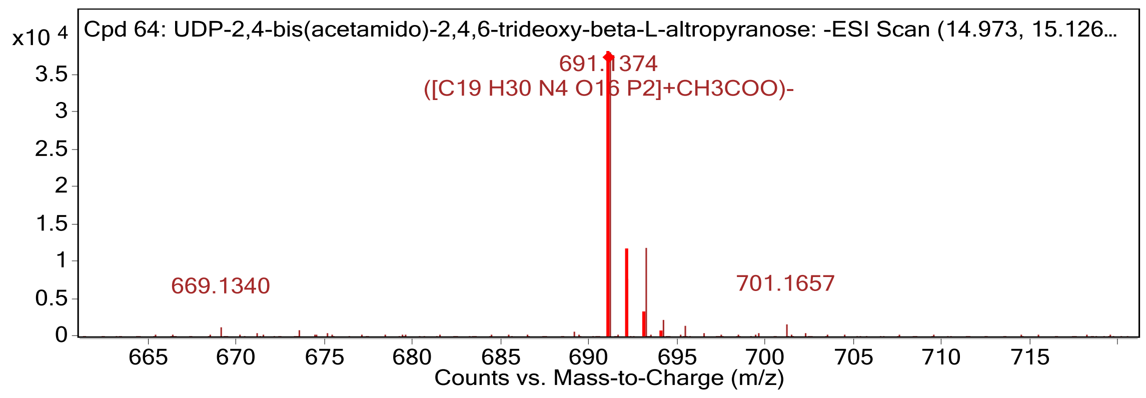

MS Spectrum Peak List

m/z	*Calc m/z*	Diff(ppm)	z	Abund	Formula	Ion
160.8462			1	22770.63		
325.1914			1	142676.13		
326.1941			1	26999.2		
339.2067			1	26821.82		
555.2948			1	24400.77		
691.1374	691.1271	-14.93	1	38146.98	C19 H30 N4 O16 P2	(M+CH3COO)-
692.1404	692.1301	-14.79	1	7954.31	C19 H30 N4 O16 P2	(M+CH3COO)-
693.1345	693.1321	-3.41	1	12077.98	C19 H30 N4 O16 P2	(M+CH3COO)-
694.137	694.1347	-3.29	1	2447.34	C19 H30 N4 O16 P2	(M+CH3COO)-
695.1381	695.1369	-1.73	1	382.41	C19 H30 N4 O16 P2	(M+CH3COO)-

MSMS Spectrum

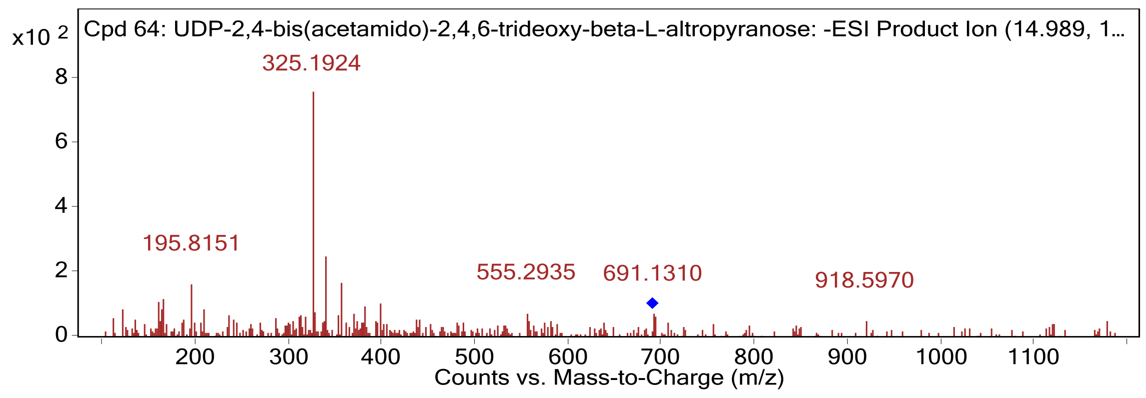

MS/MS Spectrum Peak List

m/z	z	Abund
160.8455		107.4
162.8446		88.1
164.8402		116.25
195.8151		163.95
325.1924	1	759.46
326.1938	1	149.97
339.2079		250.07
355.3289	1	167.46
381.3462		96.39
397.2762	1	102.4

Compound Structure

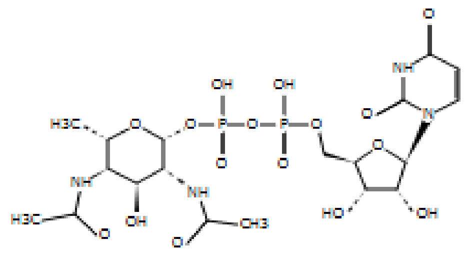

Compound Label	Name	*m/z*	RT	Algorithm	Mass
Cpd 65: 5'-Methoxybilobetin	**5'-Methoxybilobetin**	627.1238	15.177	Auto MS/MS	582.1239

MS Spectrum

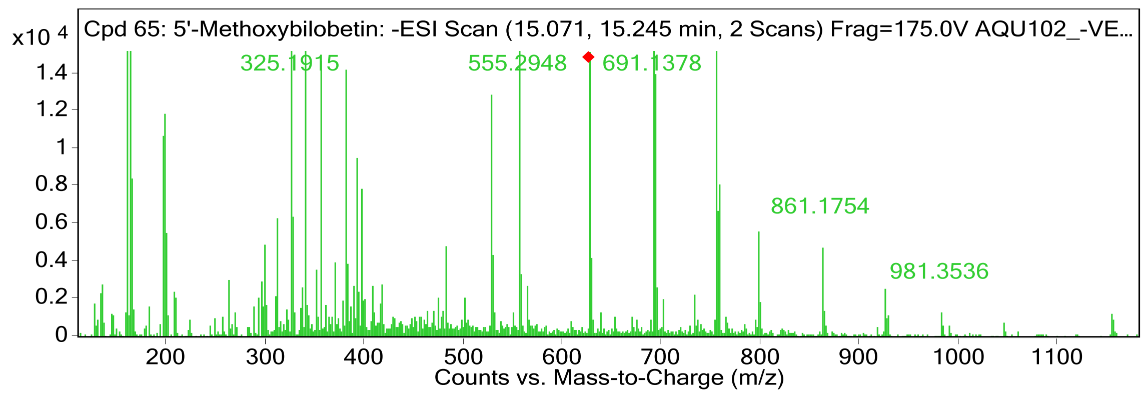

MS Zoomed Spectrum

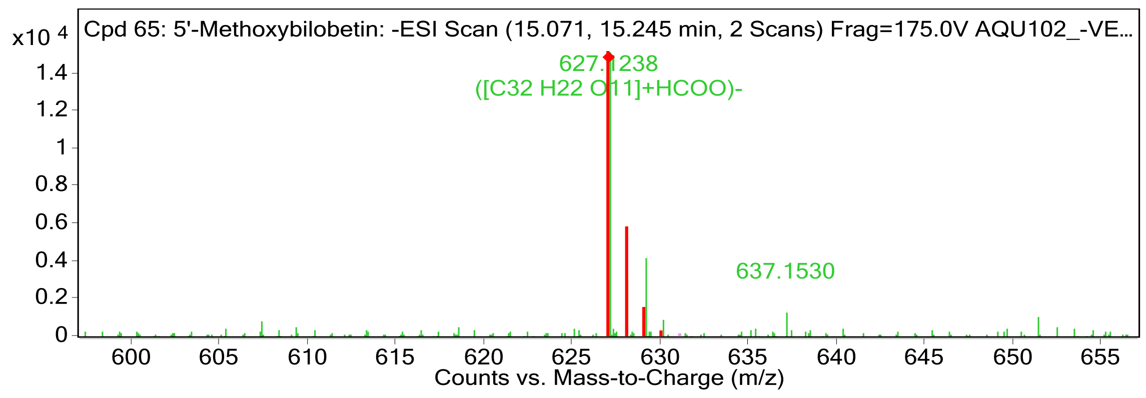

MS Spectrum Peak List

m/z	*Calc m/z*	Diff(ppm)	z	Abund	Formula	Ion
325.1915			1	143954.84		
326.1943			1	27423.63		
339.2068			1	28197.13		
555.2948			1	28759.2		
627.1238	627.1144	-15.03	1	15137.28	C32 H22 O11	(M+HCOO)-
628.1283	628.1178	-16.64	1	3004.21	C32 H22 O11	(M+HCOO)-
629.1217	629.1205	-2.01	1	4213.5	C32 H22 O11	(M+HCOO)-
630.1243	630.1231	-1.9	1	968.03	C32 H22 O11	(M+HCOO)-
691.1378			1	45149.38		
755.151			1	29685.2		

MSMS Spectrum

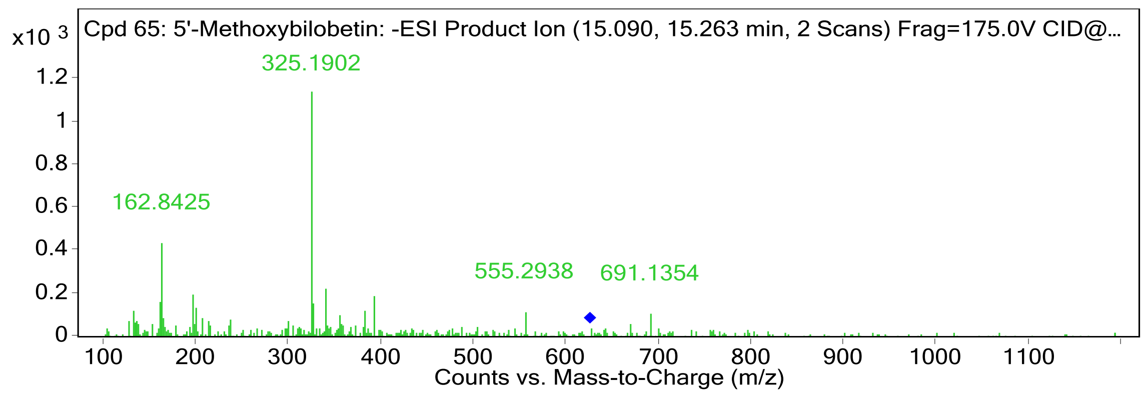

MS/MS Spectrum Peak List

m/z	z	Abund
132.8708		125
160.8454		162.43
162.8425		438.39
195.815		200.37
199.8099		137.1
325.1902	1	1142.89
326.1937	1	156.8
339.2049		227.64
381.3403		120.89
392.2539		193

Compound Structure

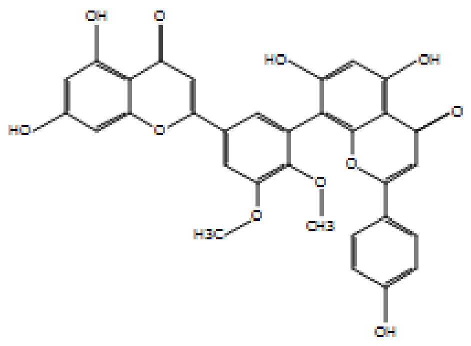

Compound Label	Name	*m/z*	RT	Algorithm	Mass
Cpd 66: Cucurbitacin E	**Cucurbitacin E**	555.2949	15.229	Auto MS/MS	556.3016

MS Spectrum

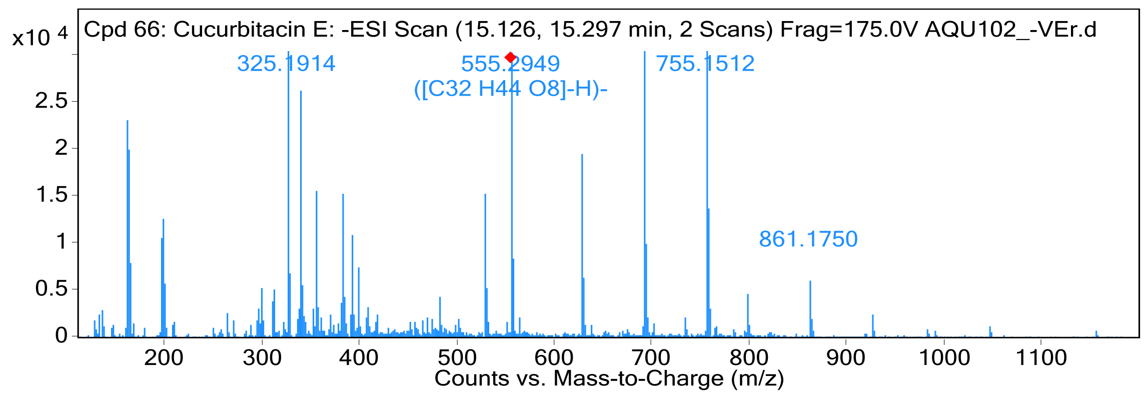

MS Zoomed Spectrum

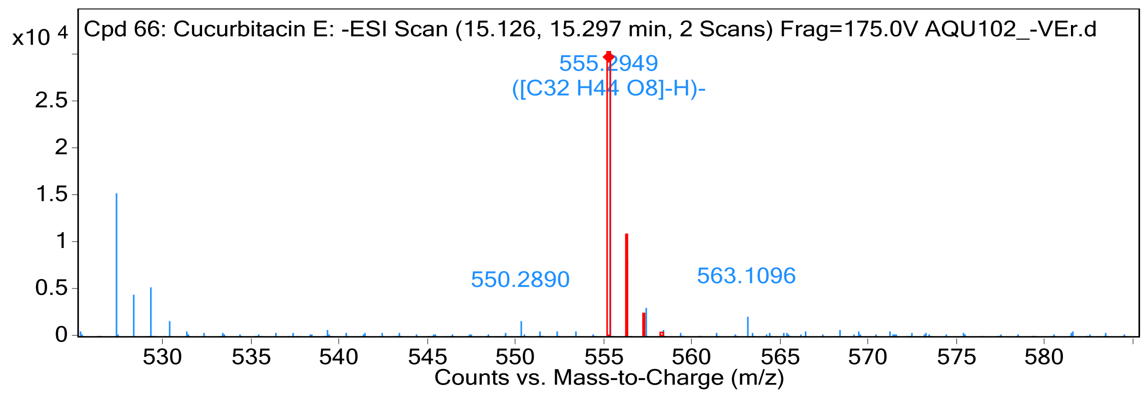

MS Spectrum Peak List

m/z	*Calc m/z*	Diff(ppm)	z	Abund	Formula	Ion
160.8461			1	23189.48		
325.1914			1	143462.3		
326.194			1	27591.75		
339.2069			1	26269.93		
555.2949	555.2963	2.55	1	30337.96	C32 H44 O8	(M-H)-
556.2981	556.2997	2.88	1	8427.9	C32 H44 O8	(M-H)-
557.2956	557.3026	12.61	1	3165.47	C32 H44 O8	(M-H)-
558.297	558.3054	15.05	1	824.36	C32 H44 O8	(M-H)-
691.1371			1	31808.43		
755.1512			1	42389.45		

MSMS Spectrum

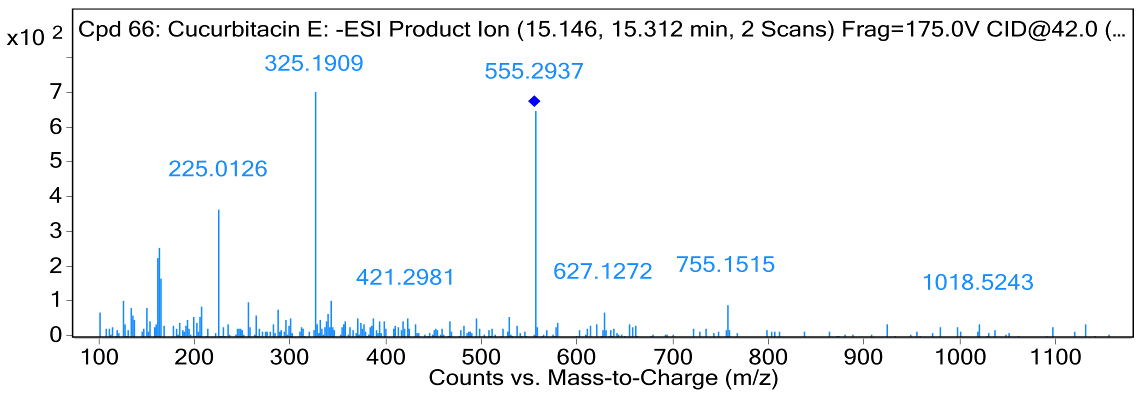

MS/MS Spectrum Peak List

m/z	z	Abund
125.0272		105.84
160.8464		226.74
162.8426		258.49
164.9899		168.39
225.0126		366.63
255.2377		102.53
325.1909	1	705.25
326.1947	1	235.39
342.2719		106.11
555.2937	1	647.92

Compound Structure

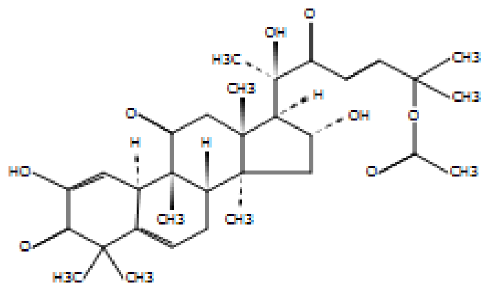

Compound Label	Name	*m/z*	RT	Algorithm	Mass
Cpd 67: Hydroquinidine	**Hydroquinidine**	325.1915	15.286	Auto MS/MS	326.1985

MS Spectrum

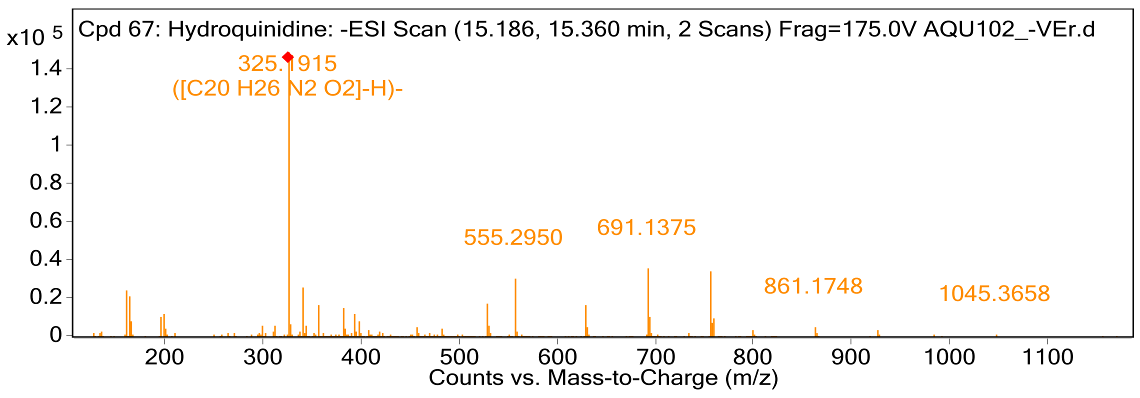

MS Zoomed Spectrum

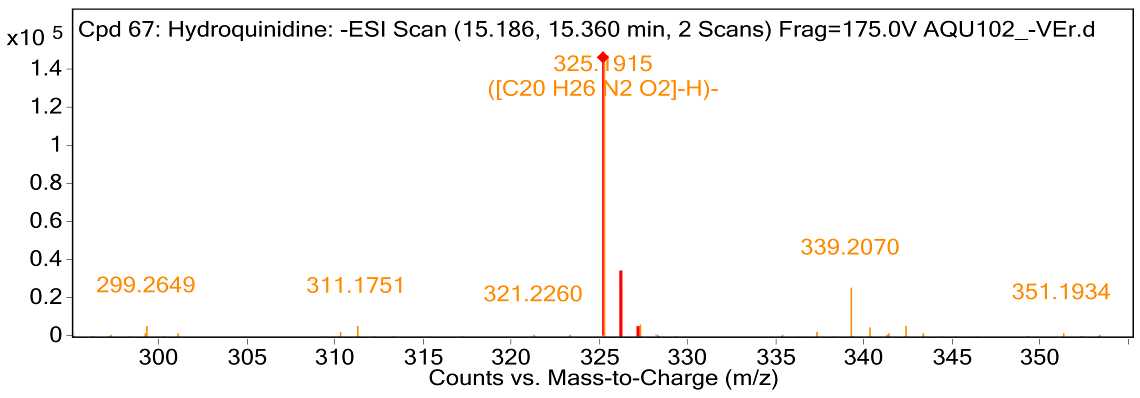

MS Spectrum Peak List

m/z	*Calc m/z*	Diff(ppm)	z	Abund	Formula	Ion
160.8461				24402.67		
162.8432				21716.11		
325.1915	325.1922	2.05	1	149234.38	C20 H26 N2 O2	(M-H)-
326.1943	326.1953	3.25	1	26779.19	C20 H26 N2 O2	(M-H)-
327.1911	327.1982	21.76	1	6715.36	C20 H26 N2 O2	(M-H)-
339.207			1	25881.34		
527.3088			1	17454.21		
555.295			1	30861.13		
691.1375			1	36073.8		
755.1508			1	34446.69		

MSMS Spectrum

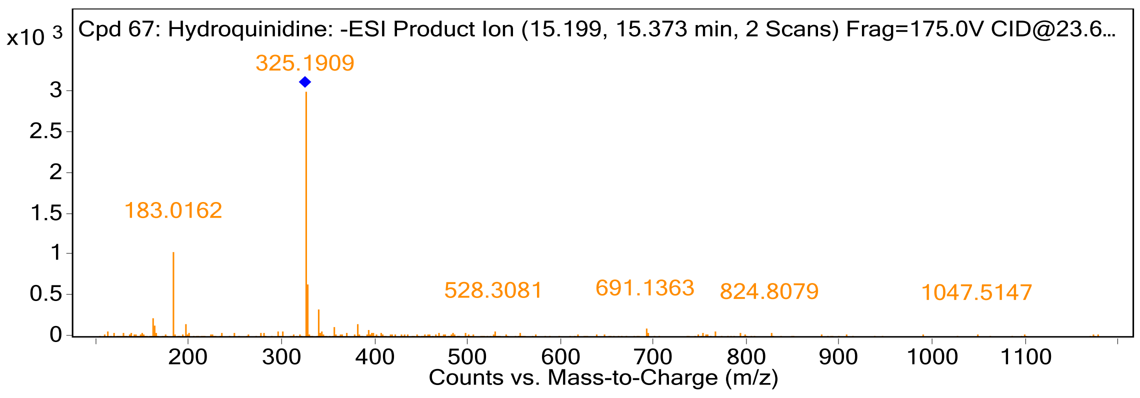

MS/MS Spectrum Peak List

m/z	z	Abund
160.8455		229.27
162.8431		136.16
183.0162	1	1052.23
195.8155		160.56
325.1909	1	3010.2
326.1948	1	650.86
339.2052	1	338.29
355.3272	1	127.13

381.3431		168.8
691.1363	1	99.86

Compound Structure

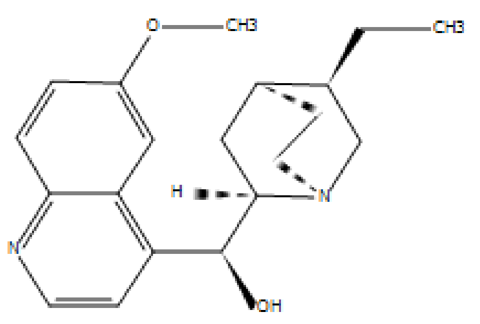

Compound Label	Name	*m/z*	RT	Algorithm	Mass
Cpd 68: UDP-2,4-bis(acetamido)-2,4,6-trideoxy-beta-L-	**UDP-2,4-bis(acetamido)-2,4,6-trideoxy-beta-L-**	691.1376	15.403	Auto MS/MS	632.1217

MS Spectrum

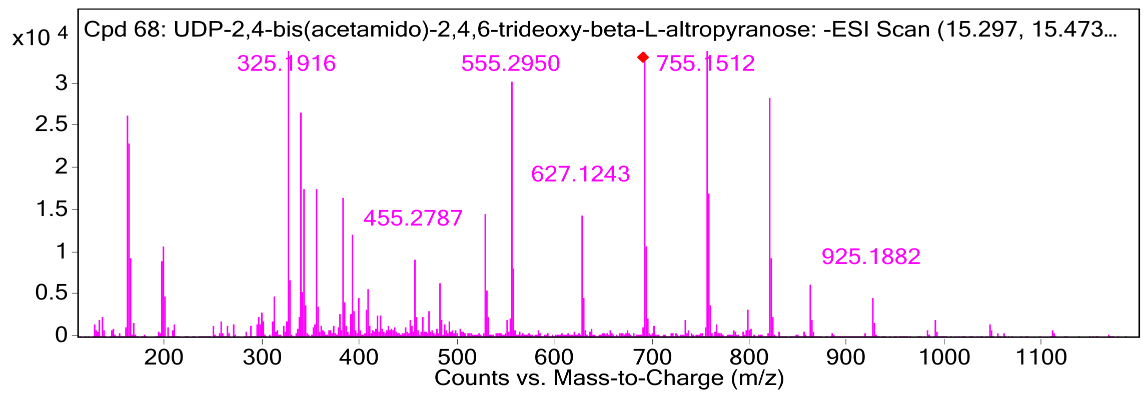

MS Zoomed Spectrum

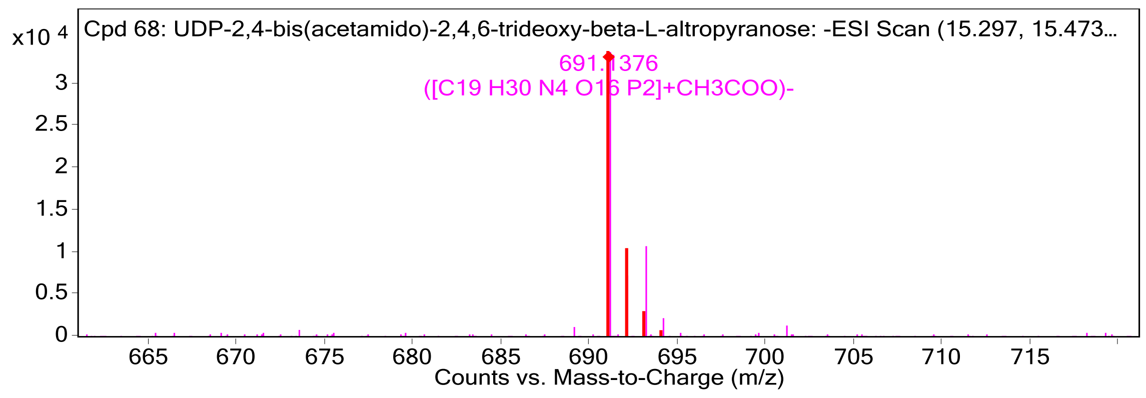

MS Spectrum Peak List

m/z	*Calc m/z*	Diff(ppm)	z	Abund	Formula	Ion
325.1916			1	148496.5		
326.1943			1	28309.95		
555.295			1	30356.8		
691.1376	691.1271	-15.25	1	33778.45	C19 H30 N4 O16 P2	(M+CH3COO)-
692.1408	692.1301	-15.37	1	7334.32	C19 H30 N4 O16 P2	(M+CH3COO)-
693.1349	693.1321	-4.04	1	10719.65	C19 H30 N4 O16 P2	(M+CH3COO)-
694.137	694.1347	-3.34	1	2348.43	C19 H30 N4 O16 P2	(M+CH3COO)-
695.133	695.1369	5.65	1	456.64	C19 H30 N4 O16 P2	(M+CH3COO)-
755.1512			1	54143.53		
819.1644			1	28320.62		

MSMS Spectrum

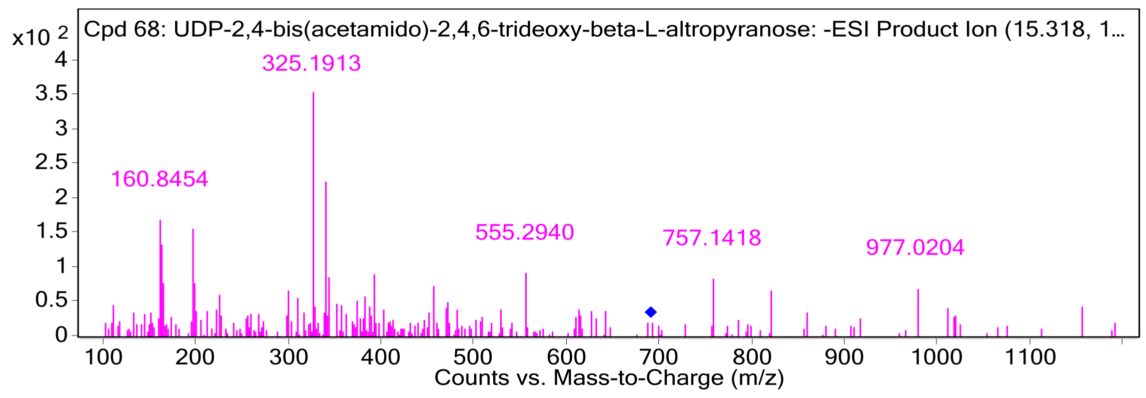

MS/MS Spectrum Peak List

m/z	z	Abund
160.8454		169.84
162.8418		134.43
195.8162		156.64
325.1913		355.23
326.1911		177.93
339.2065	1	225.95
342.2708		87.71
392.2578		90.92
555.294		92.7
757.1418		84.63

Compound Structure

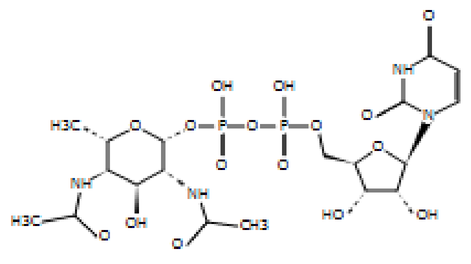

Compound Label	Name	*m/z*	RT	Algorithm	Mass
Cpd 69: Hydroquinidine	**Hydroquinidine**	325.1915	15.63	Auto MS/MS	326.1985

MS Spectrum

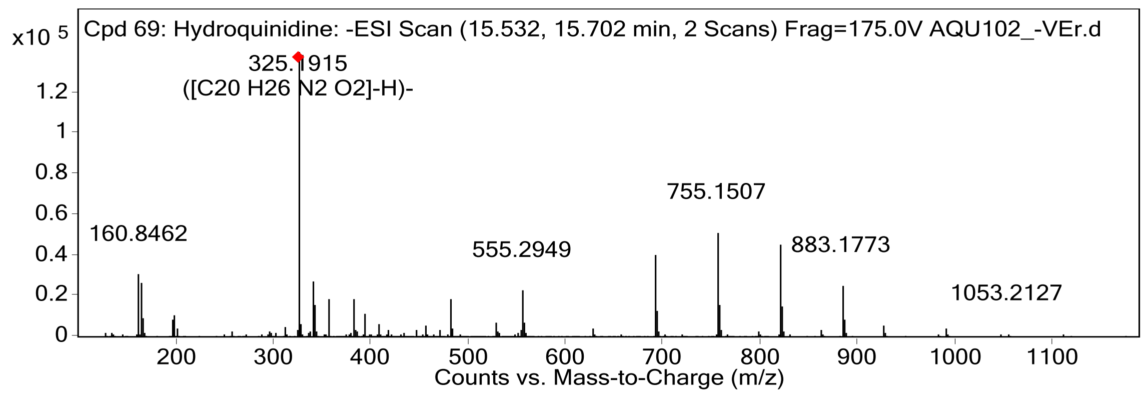

MS Zoomed Spectrum

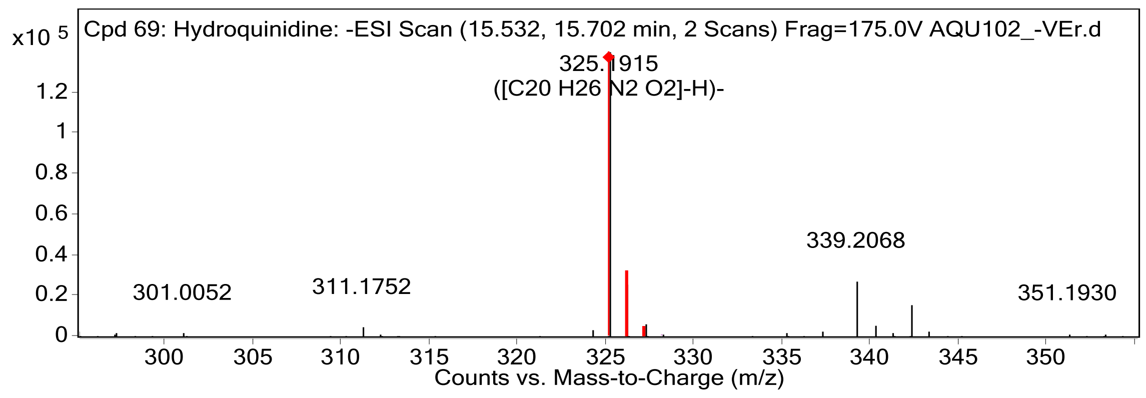

MS Spectrum Peak List

m/z	*Calc m/z*	Diff(ppm)	z	Abund	Formula	Ion
160.8462				30787.11		
162.8433				26868.1		
325.1915	325.1922	1.95	1	139897.03	C20 H26 N2 O2	(M-H)-
326.1943	326.1953	3.22	1	26213.67	C20 H26 N2 O2	(M-H)-
327.1914	327.1982	20.73	1	6434.11	C20 H26 N2 O2	(M-H)-
339.2068			1	27522.27		
691.1376			1	40131.66		
755.1507			1	51503.87		
819.1639			1	45686.18		
883.1773			1	25315.46		

MSMS Spectrum

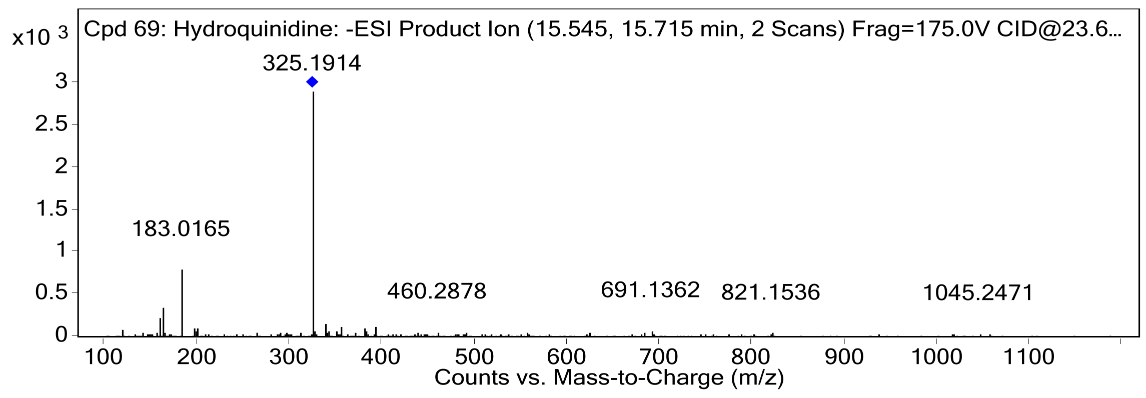

MS/MS Spectrum Peak List

m/z	z	Abund
160.8455		226.48
162.8432		345.85
183.0165		797.96
325.1914	1	2903.04
326.1941	1	516.67
339.2055	1	159.04
355.3291		128.03
356.3339		104.96
381.3427		105.8
392.2541		118.43

Compound Structure

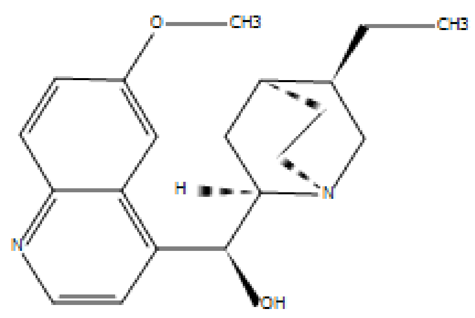

Compound Label	*m/z*	RT	Algorithm
Compound 70	883.1766	15.766	Auto MS/MS

MS Spectrum

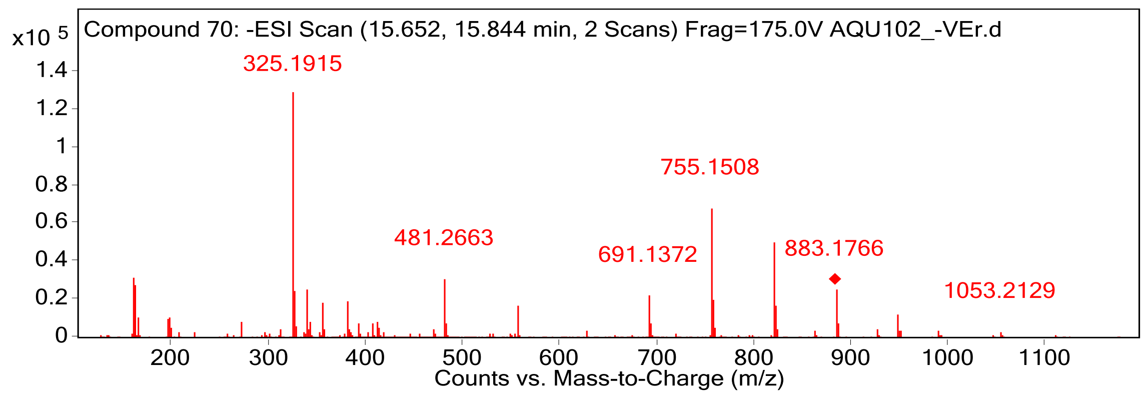

MS Zoomed Spectrum

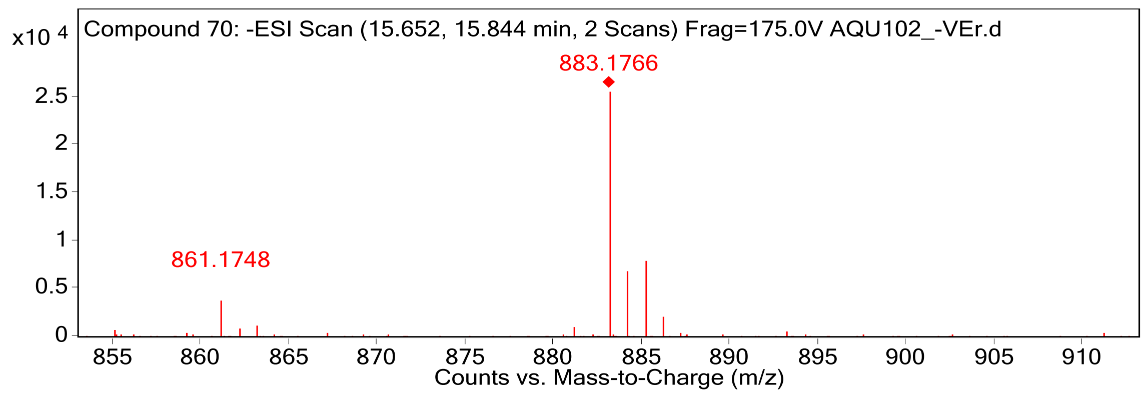

MS Spectrum Peak List

m/z	z	Abund
160.8461	1	32013.7
325.1915	1	129369.63
481.2663	1	31163.96
755.1508	1	68461.72
819.164	1	50336.54
883.1766	1	25587.53
884.1797	1	6950.26
885.1759	1	7919.04
886.1785	1	2123.69
887.1819	1	489.79

MSMS Spectrum

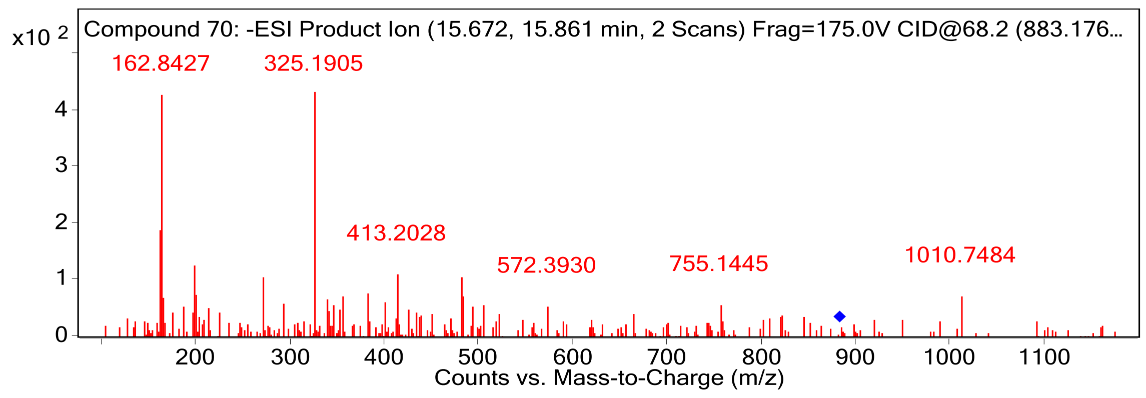

MS/MS Spectrum Peak List

m/z	z	Abund
160.8462		188.51
162.8427		427.82
197.8096		125.88
199.8099		75.88
271.2316		107.29
325.1905	1	433.72
381.3431		77.38
413.2028	1	111.86
481.2655		105.01
482.2624	1	73.47

Compound Label	Name	*m/z*	RT	Algorithm	Mass
Cpd 71: UDP-2,4-bis(acetamido)-2,4,6-trideoxy-beta-L-	**UDP-2,4-bis(acetamido)-2,4,6-trideoxy-beta-L-**	691.1372	15.778	Auto MS/MS	632.1217

MS Spectrum

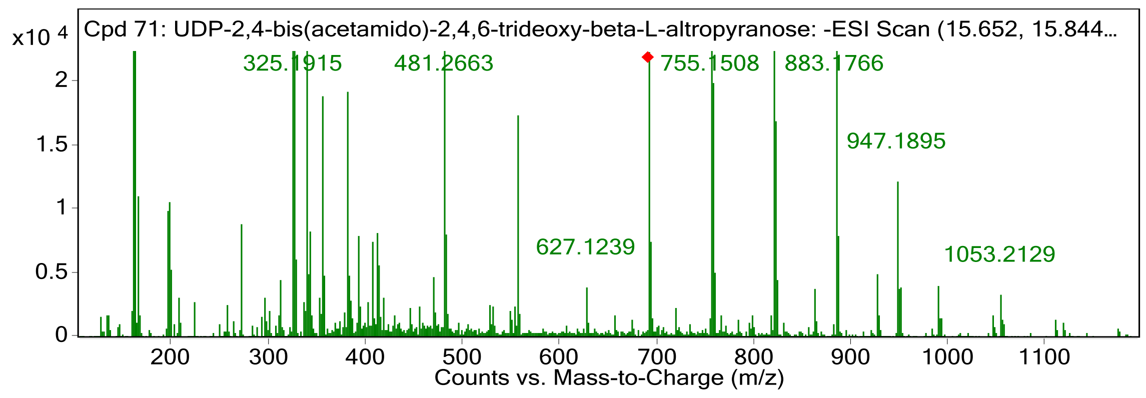

MS Zoomed Spectrum

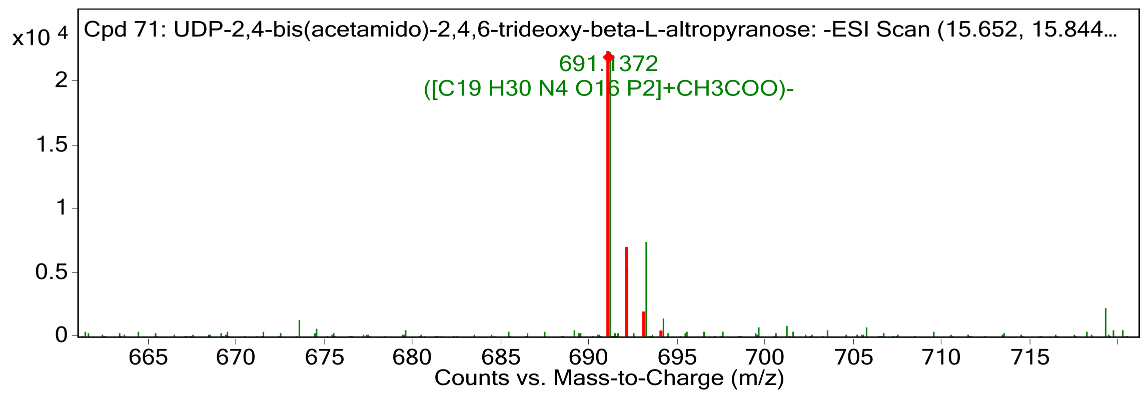

MS Spectrum Peak List

m/z	*Calc m/z*	Diff(ppm)	z	Abund	Formula	Ion
160.8461			1	32013.7		
162.8432			1	27967.64		
325.1915			1	129369.63		
481.2663			1	31163.96		
691.1372	691.1271	-14.74	1	22326.13	C19 H30 N4 O16 P2	(M+CH3COO)-
692.1416	692.1301	-16.57	1	5435.1	C19 H30 N4 O16 P2	(M+CH3COO)-
693.1346	693.1321	-3.59	1	7456.4	C19 H30 N4 O16 P2	(M+CH3COO)-
694.1371	694.1347	-3.37	1	1473.73	C19 H30 N4 O16 P2	(M+CH3COO)-
755.1508			1	68461.72		
819.164			1	50336.54		

MSMS Spectrum

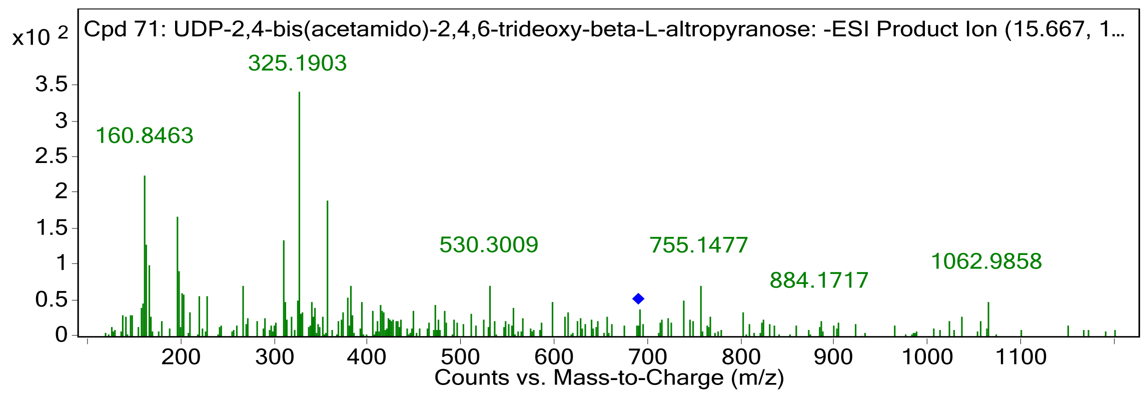

MS/MS Spectrum Peak List

m/z	z	Abund
160.8463		224.61
162.8429		128.93
164.8408		100.85
195.8149	1	168.61
197.8127	1	91.81
265.1563		72.5
309.3228	1	134.72
325.1903	1	343.06
326.193	1	79.72
355.3304	1	190.58

Compound Structure

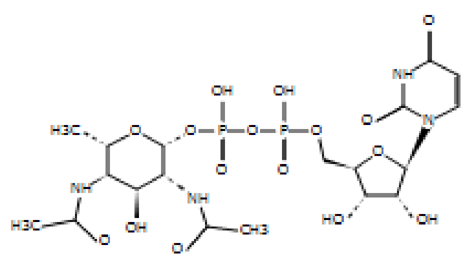

Compound Label	Name	*m/z*	RT	Algorithm	Mass
Cpd 72: Hydroquinidine	**Hydroquinidine**	325.1915	16.016	Auto MS/MS	326.1983

MS Spectrum

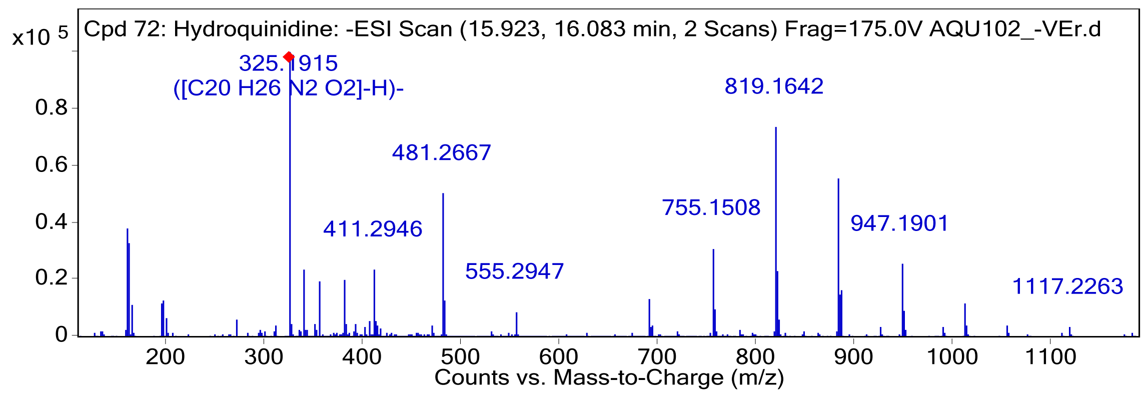

MS Zoomed Spectrum

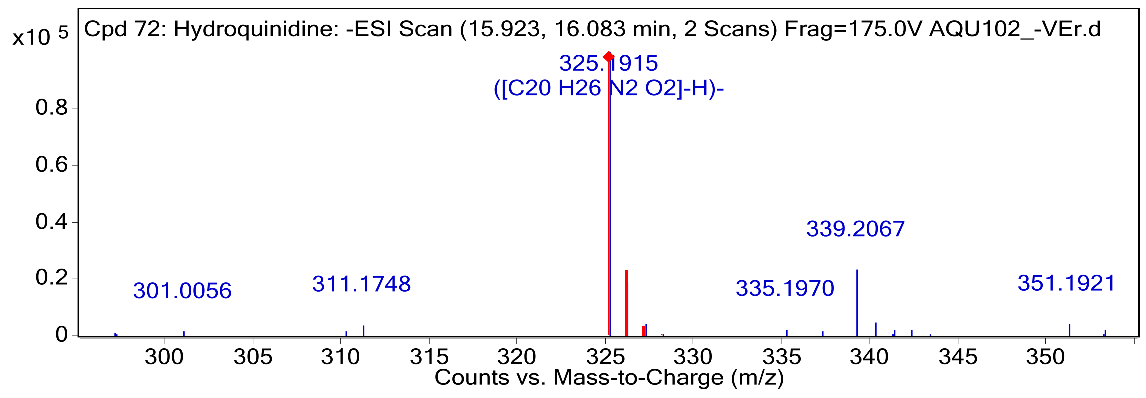

MS Spectrum Peak List

m/z	*Calc m/z*	Diff(ppm)	z	Abund	Formula	Ion
160.8461			1	38023.35		
162.8431			1	32908.35		
325.1915	325.1922	2.12	1	100183.77	C20 H26 N2 O2	(M-H)-
326.1944	326.1953	2.91	1	19106.6	C20 H26 N2 O2	(M-H)-
327.1897	327.1982	25.92	1	4856.11	C20 H26 N2 O2	(M-H)-
328.1924	328.2009	26.07	1	1148.68	C20 H26 N2 O2	(M-H)-
481.2667			1	50519.39		
755.1508			1	31171.91		
819.1642			1	73927.85		
883.1772			1	55916.31		

MSMS Spectrum

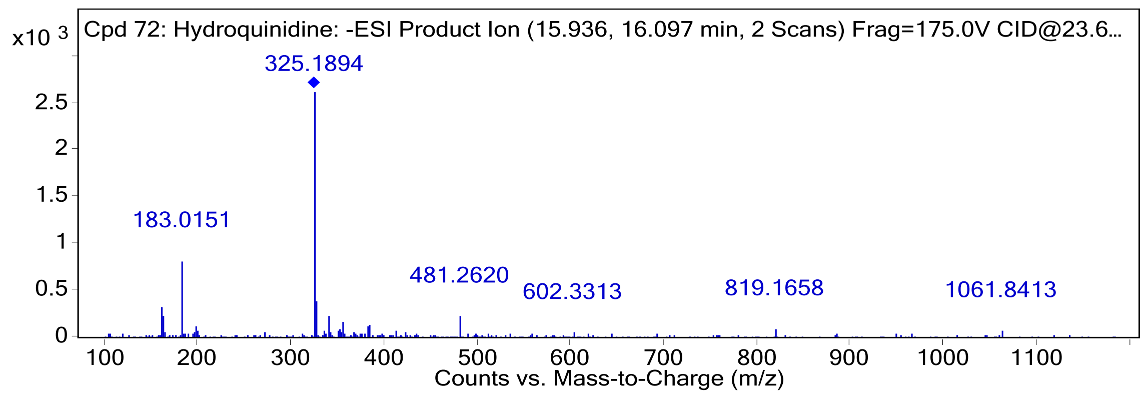

MS/MS Spectrum Peak List

m/z	z	Abund
160.8441		322.81
162.8434		240.91
183.0151	1	822.38
197.8102		125.7
325.1894	1	2621.94
326.1928	1	386.58
339.2038		237.84
355.3261	1	176.94
383.1939		136.84

481.262		231.49

Compound Structure

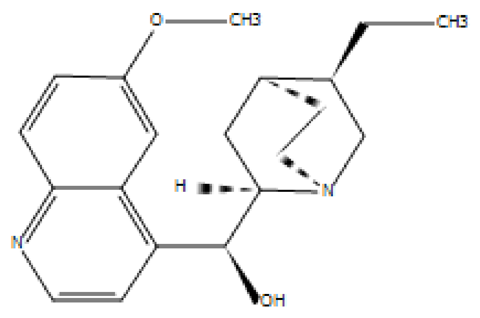

Compound Label	*m/z*	RT	Algorithm
Compound 73	883.1772	16.14	Auto MS/MS

MS Spectrum

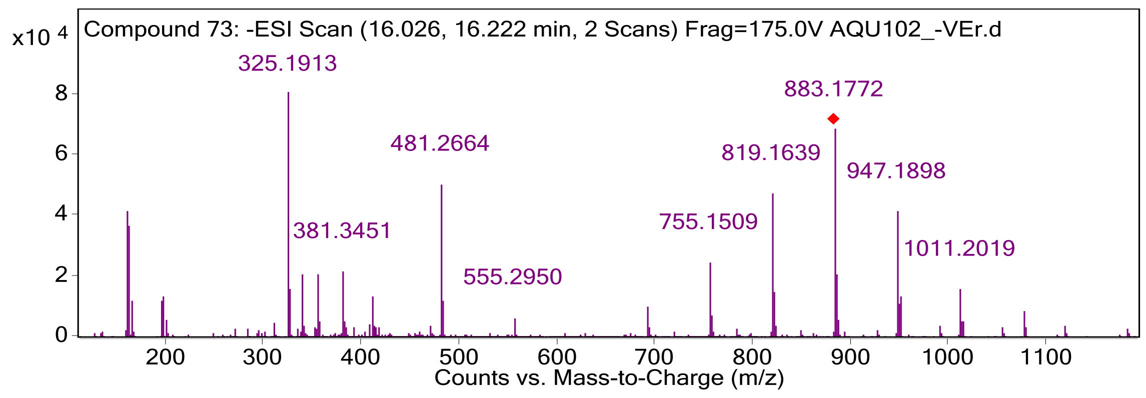

MS Zoomed Spectrum

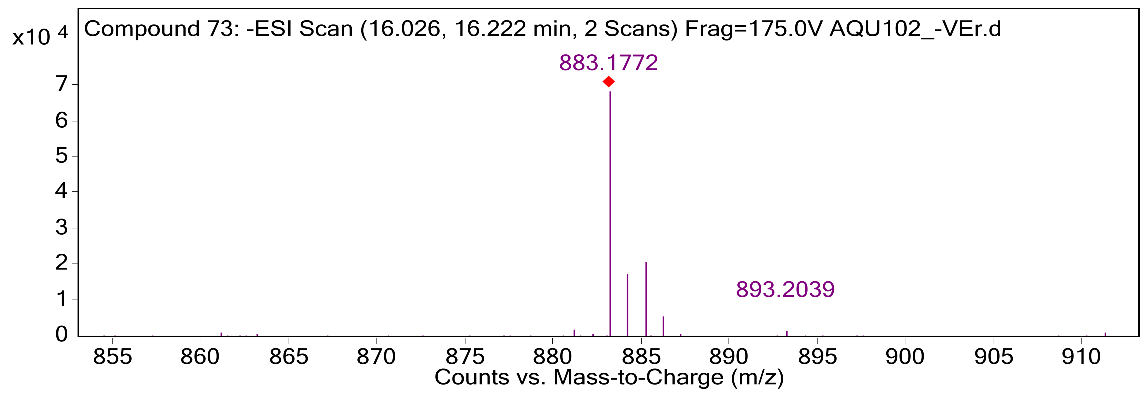

MS Spectrum Peak List

m/z	z	Abund
160.8462		41795.32
325.1913	1	81020.36
481.2664	1	50586.57
819.1639	1	47498.37
883.1772	1	68592.39
884.1802	1	17794.46
885.1752	1	20769.3
886.1774	1	5733.62
887.1807	1	951.39
947.1898	1	41496.41

MSMS Spectrum

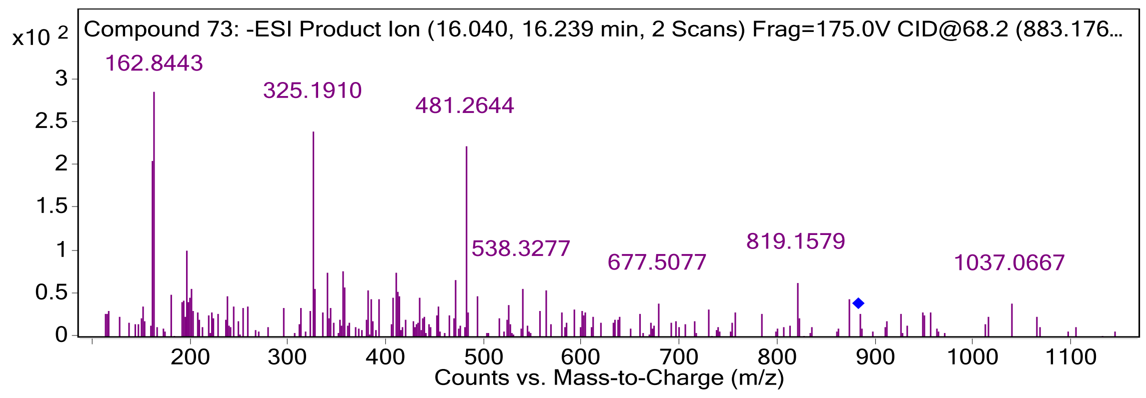

MS/MS Spectrum Peak List

m/z	z	Abund
160.8467	1	205.18
162.8443		286.33
195.8159		101.35
325.191	1	240.17
339.21	1	75.01
355.3271		77.07
409.9355		75.08
470.2694		66.5
481.2644	1	222.58
819.1579	1	64.02

Compound Label	*m/z*	RT	Algorithm
Compound 74	947.1903	16.326	Auto MS/MS

MS Spectrum

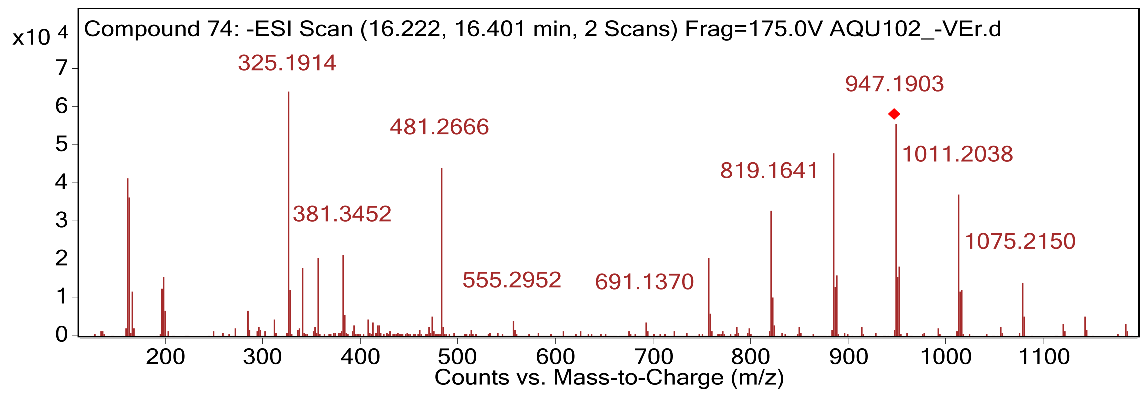

MS Zoomed Spectrum

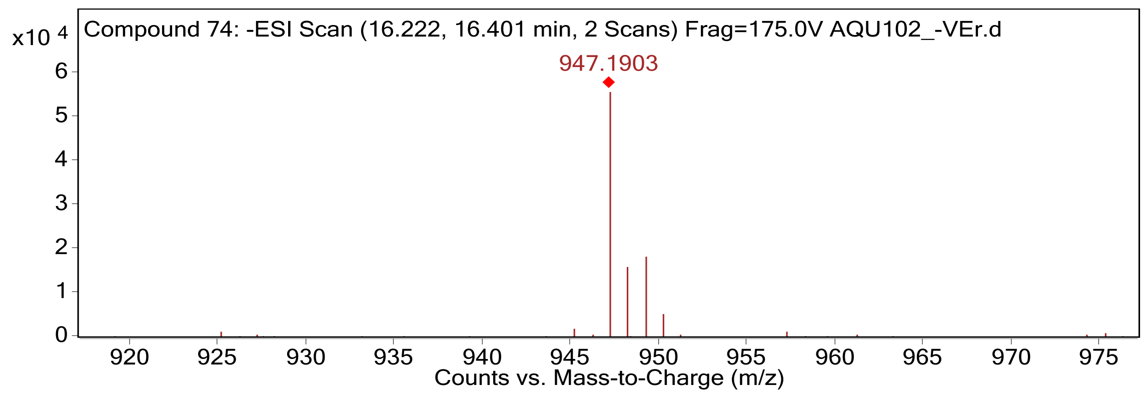

MS Spectrum Peak List

m/z	z	Abund
160.8463		41673.11
325.1914	1	64362.64
481.2666	1	44401.72
883.1774	1	48295.95
947.1903	1	55691.57
948.1934	1	15825.28
949.1887	1	18448.13
950.1907	1	5197.78

951.1962	1	736.73
1011.2038	1	37255.08

MSMS Spectrum

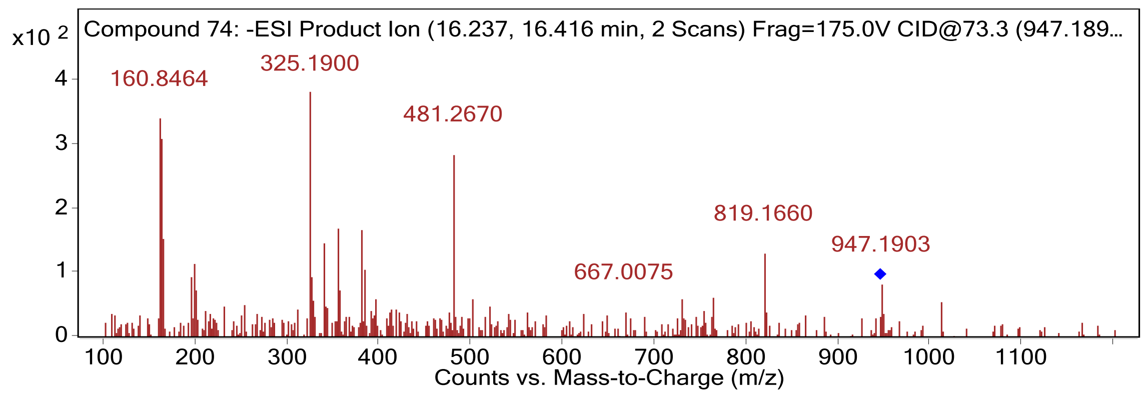

MS/MS Spectrum Peak List

m/z	z	Abund
160.8464		340.12
162.8418		309.57
164.8398		154.14
197.8133		113.96
325.19	1	383.07
339.2068	1	146.94
355.3285	1	170.15
381.3458		166.63
481.267	1	284.72
819.166		129.67

Compound Label	Name	*m/z*	RT	Algorithm	Mass
Cpd 75: Hydroquinidine	**Hydroquinidine**	325.1915	16.371	Auto MS/MS	326.1983

MS Spectrum

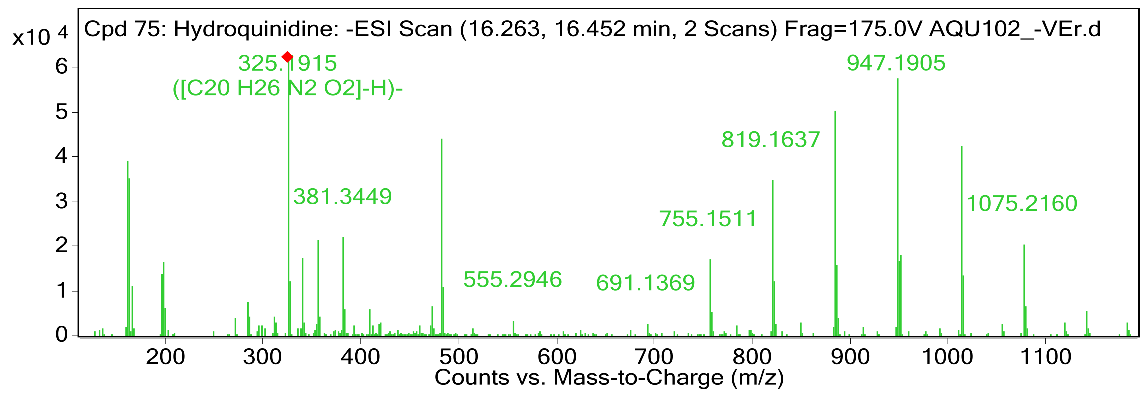

MS Zoomed Spectrum

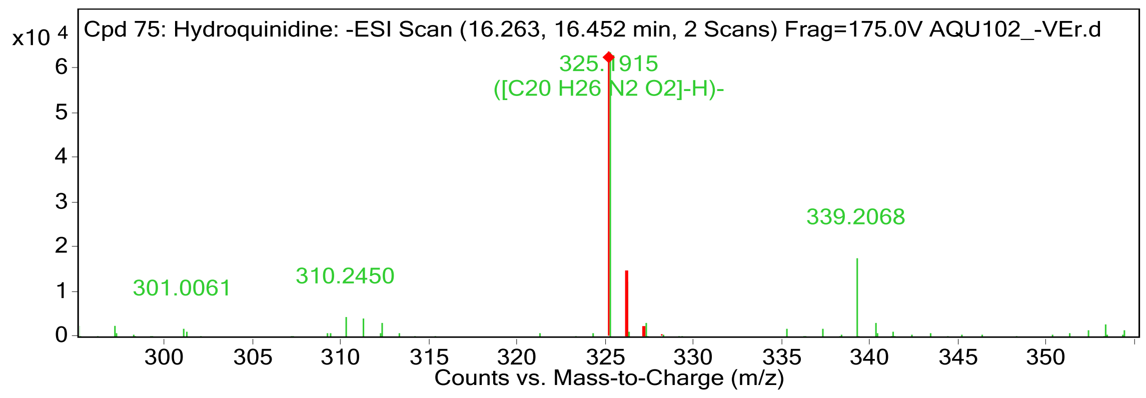

MS Spectrum Peak List

m/z	*Calc m/z*	Diff(ppm)	z	Abund	Formula	Ion
160.8462				39268.59		
162.8432				35387.44		
325.1915	325.1922	2.14	1	63675.09	C20 H26 N2 O2	(M-H)-
326.1943	326.1953	3.19	1	12526.35	C20 H26 N2 O2	(M-H)-
327.1907	327.1982	22.97	1	3389.34	C20 H26 N2 O2	(M-H)-
328.1915	328.2009	28.84	1	653.86	C20 H26 N2 O2	(M-H)-
481.2669			1	44173.45		
883.1773			1	50703.4		
947.1905			1	57720.46		
1011.2035			1	42772.77		

MSMS Spectrum

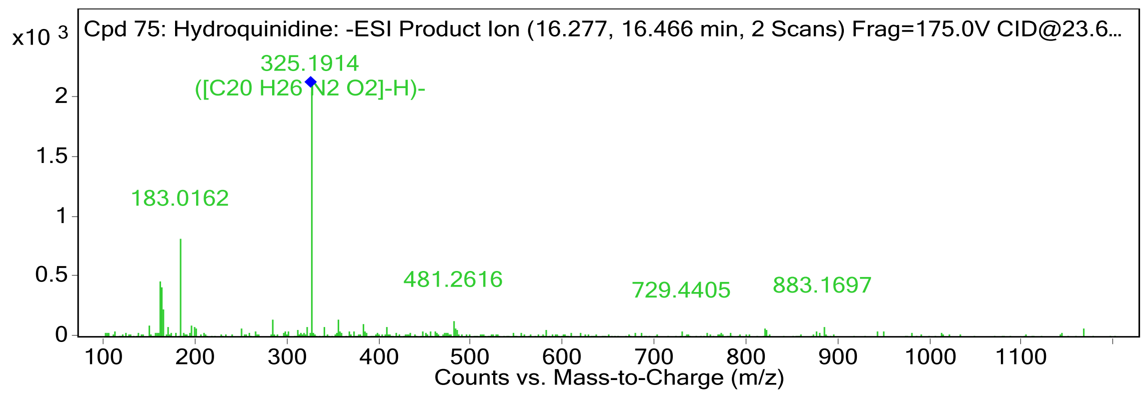

MS/MS Spectrum Peak List

m/z	*Calc m/z*	Diff (ppm)	z	Abund	Formula	Ion
160.8464				468.84		
162.8415			1	415.38		
164.8404			1	228.49		
183.0162			1	818.69		
184.0227			1	119.69		
283.1445				141.71		
325.1914	325.1922	2.18	1	2051.66	C20 H26 N2 O2	(M-H)-
326.1939			1	393.94		
355.3289			1	150.58		
481.2616			1	139.06		

Compound Structure

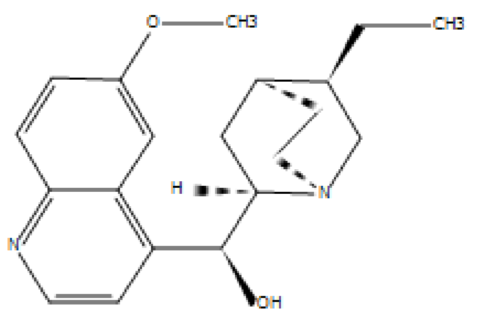

Compound Label	*m/z*	RT	Algorithm
Compound 76	1011.2032	16.438	Auto MS/MS

MS Spectrum

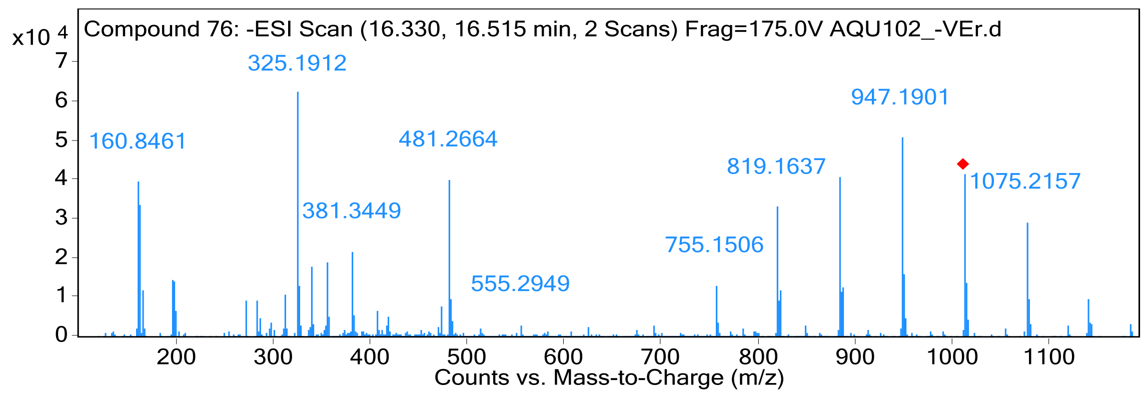

MS Zoomed Spectrum

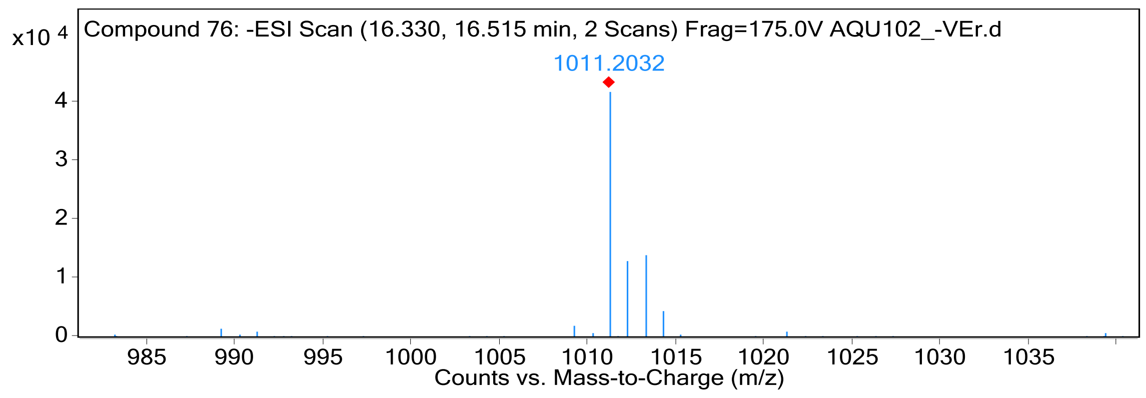

MS Spectrum Peak List

m/z	z	Abund
160.8461	1	39635.78
325.1912	1	62682.86
481.2664	1	40194.44
883.1766	1	40703.43
947.1901	1	50929.09
1011.2032	1	41693.5
1012.2056	1	12862.7
1013.2013	1	13890.79
1014.2024	1	4516.66
1015.2078	1	589.47

MSMS Spectrum

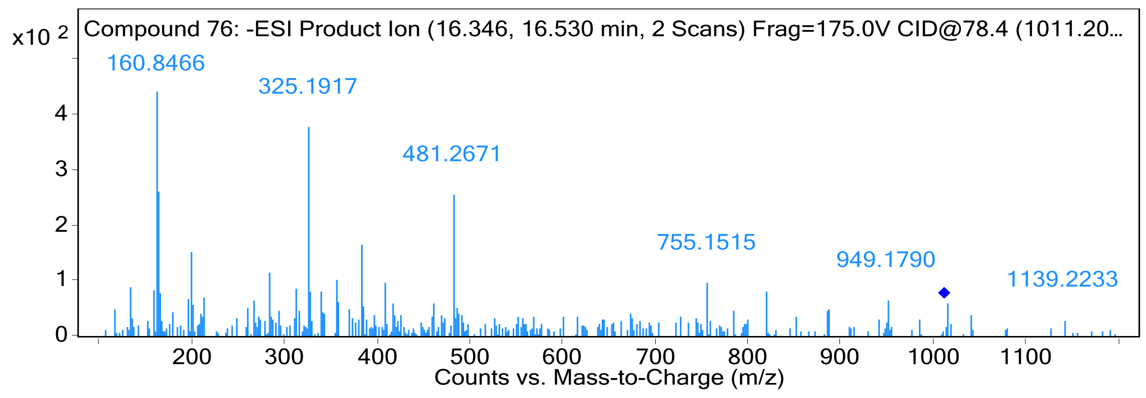

MS/MS Spectrum Peak List

m/z	z	Abund
160.8466		443.2
162.8446		261.66
197.8106		153.53
283.1429		116.73
325.1917	1	379.19
355.3331		104.43
381.3441	1	166.01
407.226	1	97.83
481.2671		257.09
755.1515		97.27

Compound Label	*m/z*	RT	Algorithm
Compound 77	947.19	16.686	Auto MS/MS

MS Spectrum

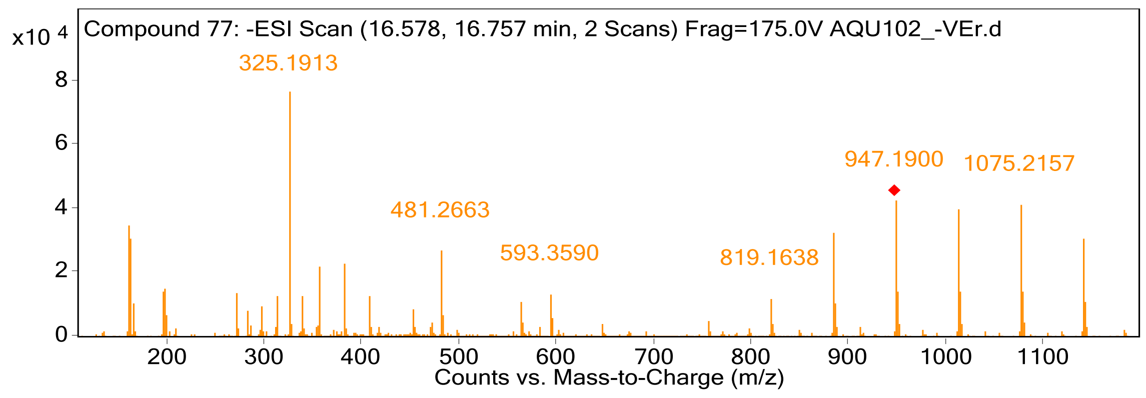

MS Zoomed Spectrum

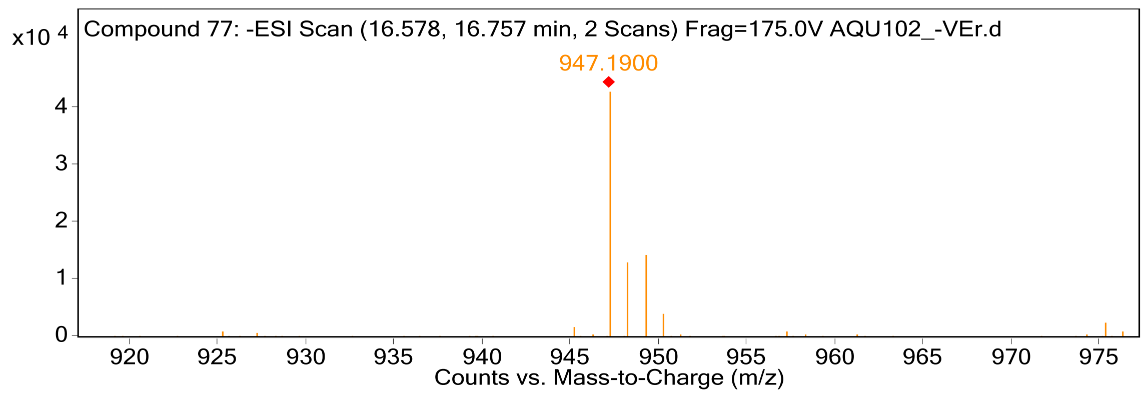

MS Spectrum Peak List

m/z	z	Abund
160.8462		34800.71
325.1913	1	76843.5
883.177	1	32458.13
947.19	1	42937.36
948.1926	1	12990.64
949.1876	1	14460.26
950.1906	1	4181.26
951.1943	1	631.65
1011.2028	1	39872.2
1075.2157	1	41590.8

MSMS Spectrum

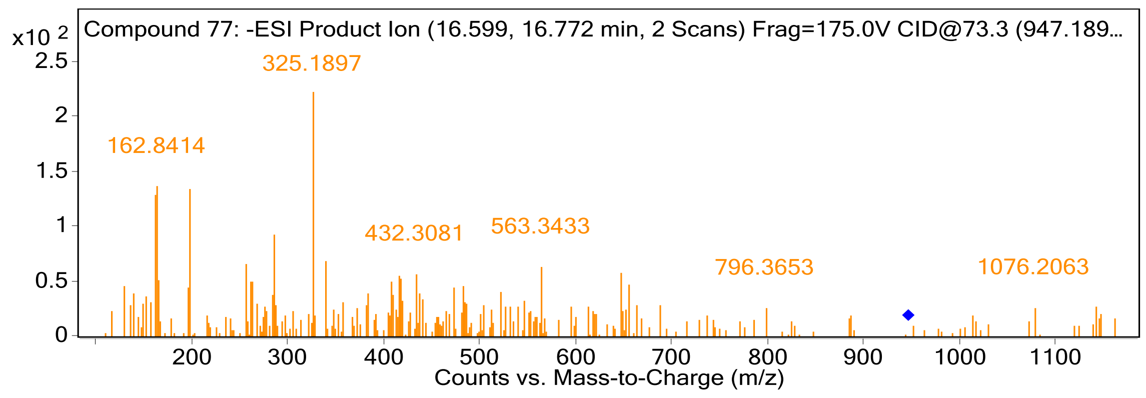

MS/MS Spectrum Peak List

m/z	z	Abund
160.8453		128.88
162.8414	1	137.24
197.8129		134.95
256.0166		66.5
285.2498		93.27
325.1897		223.34
326.1932	1	115.15
338.1448		69.04
563.3433		63.89
645.4232		58.46

Compound Label	*m/z*	RT	Algorithm
Compound 78	1075.2157	16.687	Auto MS/MS

MS Spectrum

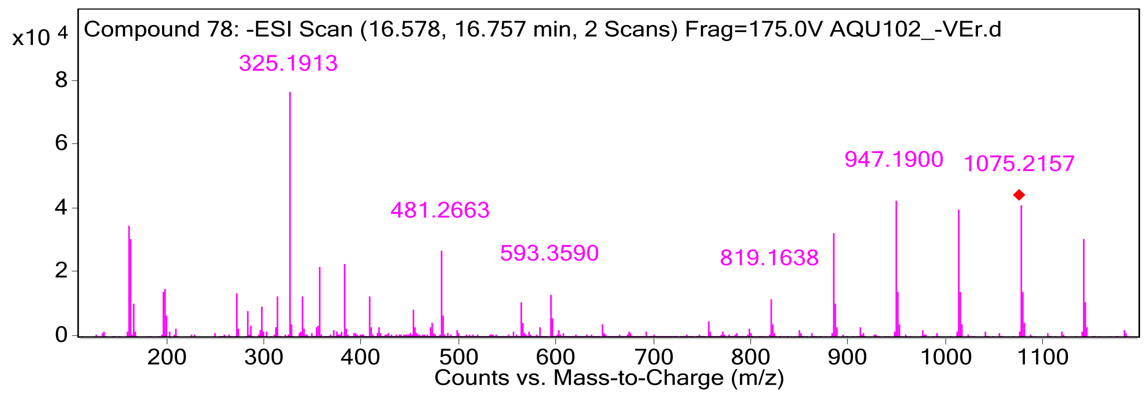

MS Zoomed Spectrum

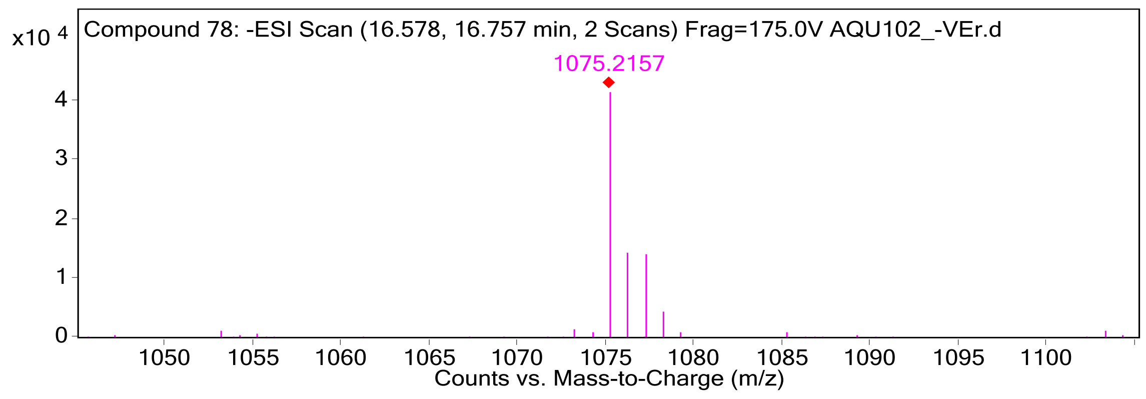

MS Spectrum Peak List

m/z	z	Abund
160.8462		34800.71
325.1913	1	76843.5
883.177	1	32458.13
947.19	1	42937.36
1011.2028	1	39872.2
1075.2157	1	41590.8
1076.2187	1	14321.43
1077.2141	1	14181.01
1078.2158	1	4375.11
1079.2155	1	873.17

MSMS Spectrum

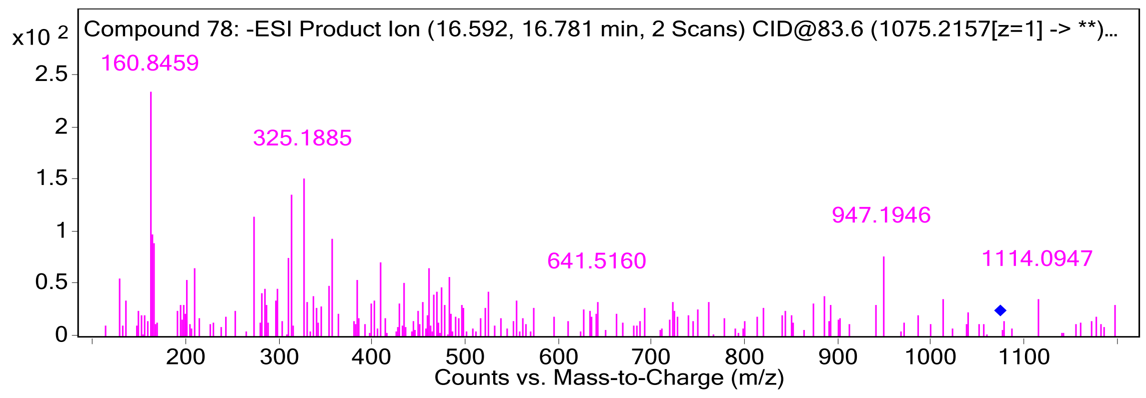

MS/MS Spectrum Peak List

m/z	z	Abund
160.8459		235.33
162.8434		98.85
164.8405		89.32
271.2332	1	115.77
308.2384		76.43
312.2611		135.84
325.1885	1	151.63
355.3287		93.59
407.226		71.71
947.1946		77.65

Compound Label	Name	*m/z*	RT	Algorithm	Mass
Cpd 79: Hydroquinidine	**Hydroquinidine**	325.1913	16.741	Auto MS/MS	326.1983

MS Spectrum

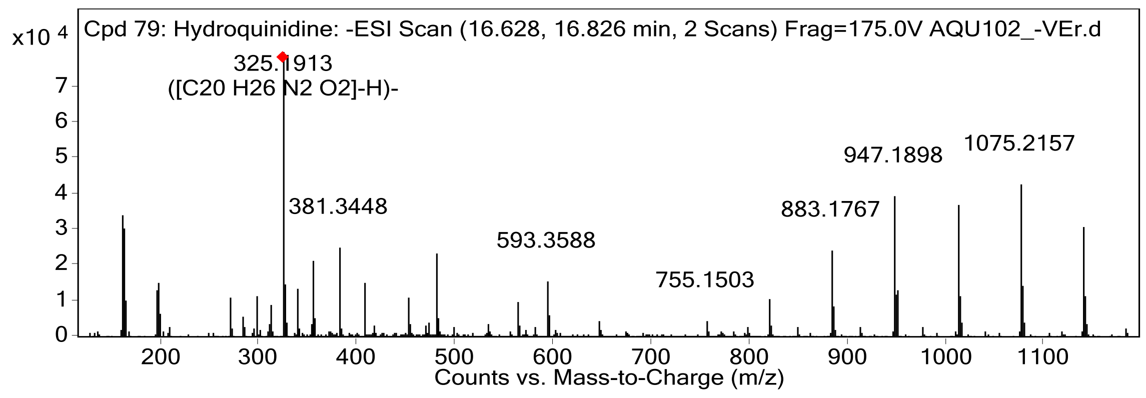

MS Zoomed Spectrum

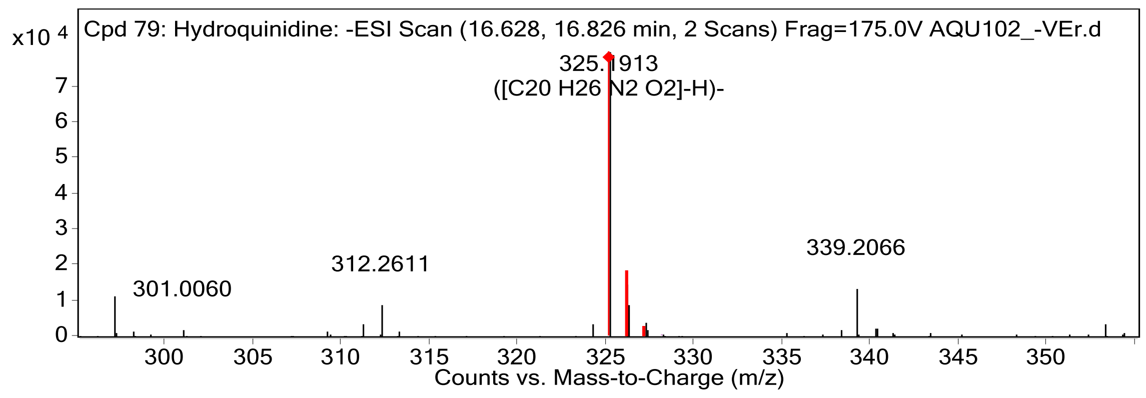

MS Spectrum Peak List

m/z	*Calc m/z*	Diff(ppm)	z	Abund	Formula	Ion
160.8461				34359.5		
162.843				30432.99		
325.1913	325.1922	2.51	1	79831.45	C20 H26 N2 O2	(M-H)-
326.194	326.1953	4	1	14714.07	C20 H26 N2 O2	(M-H)-
327.192	327.1982	18.95	1	4153.04	C20 H26 N2 O2	(M-H)-
381.3448			1	25174		
947.1898			1	39620.89		
1011.2024			1	37065.59		
1075.2157			1	42931.83		
1139.2286			1	30966.42		

MSMS Spectrum

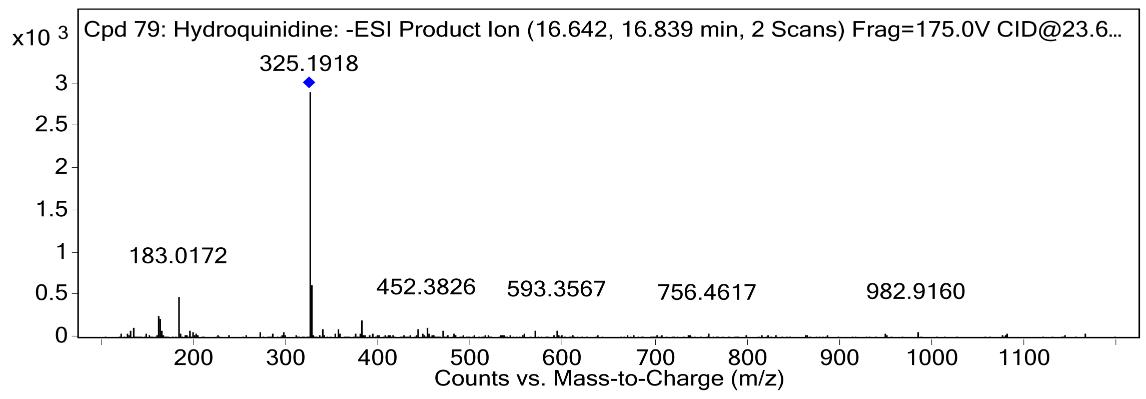

MS/MS Spectrum Peak List

m/z	z	Abund
134.8698		125.94
160.8462		268.97
162.8425		224.75
183.0172	1	487.07
184.0222	1	155.57
325.1918	1	2912.35
326.1942	1	627.95
339.2051		109.55
381.3432	1	207.57
452.3826	1	117.07

Compound Structure

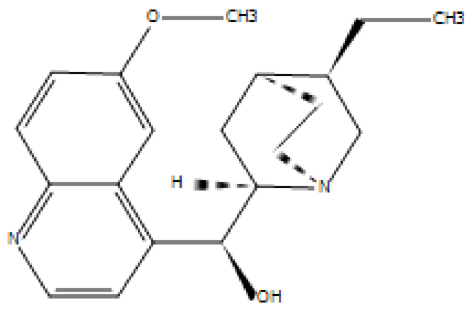

Compound Label	*m/z*	RT	Algorithm
Compound 80	1011.2031	16.82	Auto MS/MS

MS Spectrum

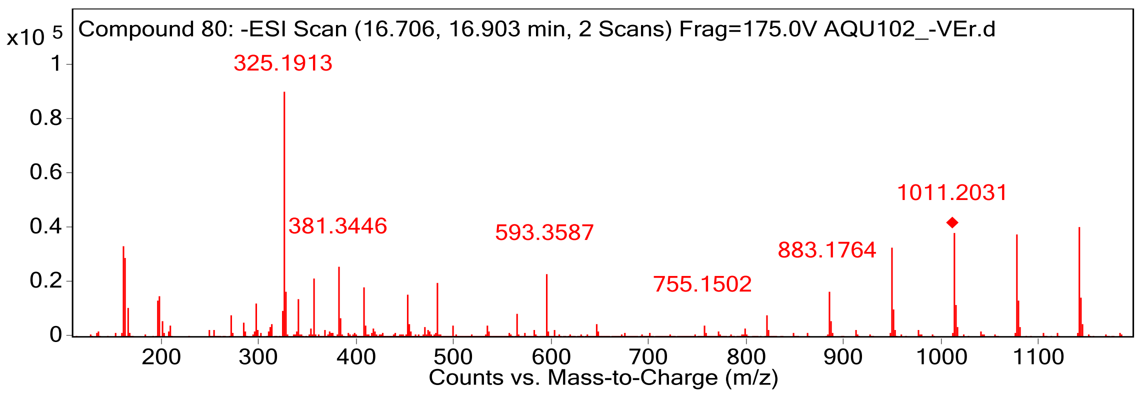

MS Zoomed Spectrum

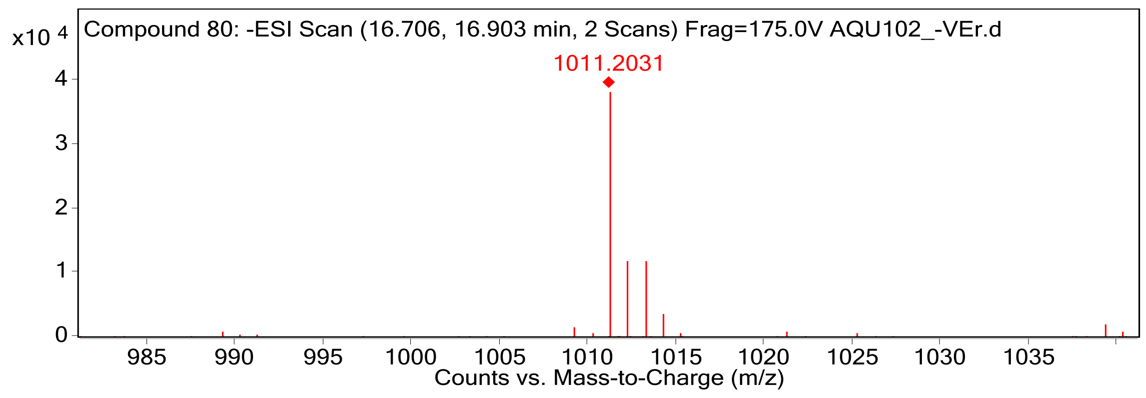

MS Spectrum Peak List

m/z	z	Abund
160.8461		33650.02
325.1913	1	90608.89
947.1898	1	33237.65
1011.2031	1	38268.03
1012.206	1	11917.55
1013.2013	1	11997.12
1014.205	1	3711.1
1015.2073	1	630.37
1075.2155	1	37704.41
1139.2285	1	40404.7

MSMS Spectrum

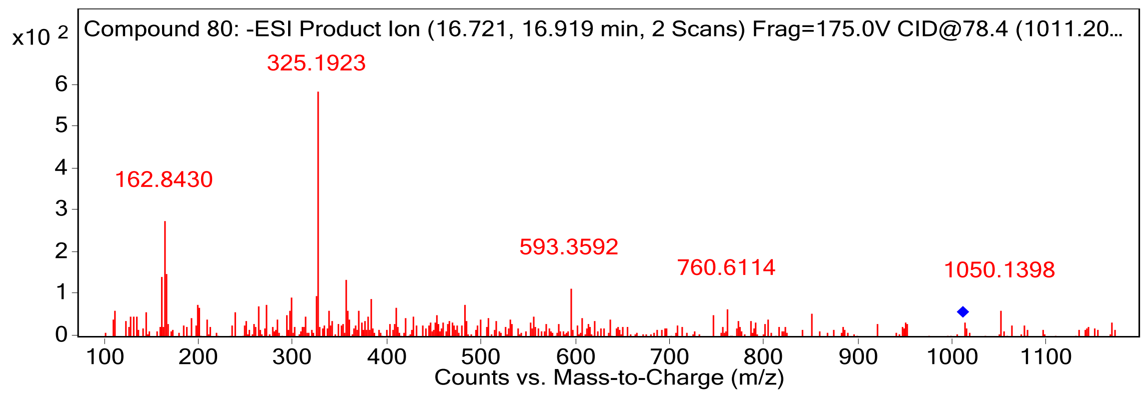

MS/MS Spectrum Peak List

m/z	z	Abund
160.8463		144.35
162.843		278.13
164.8394		149.29
297.1586		94.04
324.2568		96.26
325.1923	1	585.29
326.1937	1	127.56
355.3272		135.06
381.3452		92.26
593.3592	1	117.1

**Compound Label**	***m/z***	**RT**	**Algorithm**
Compound 81	1075.2158	17.053	Auto MS/MS

MS Spectrum

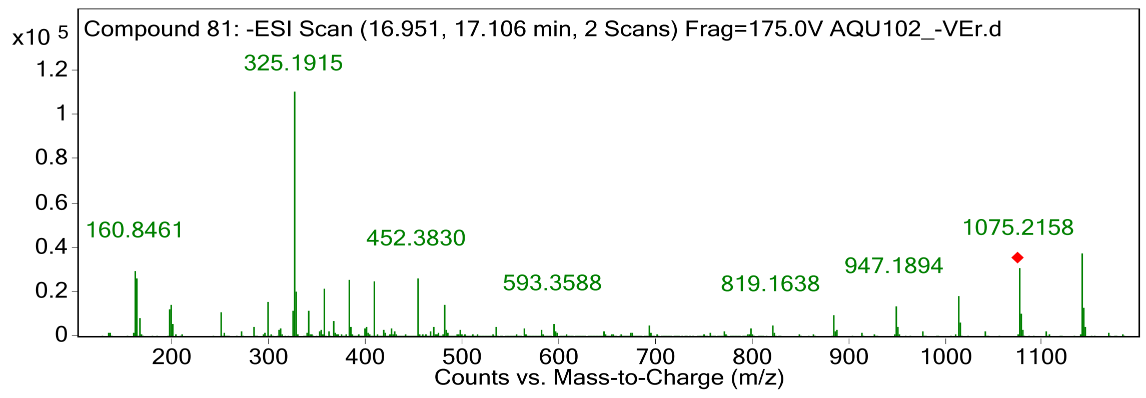

MS Zoomed Spectrum

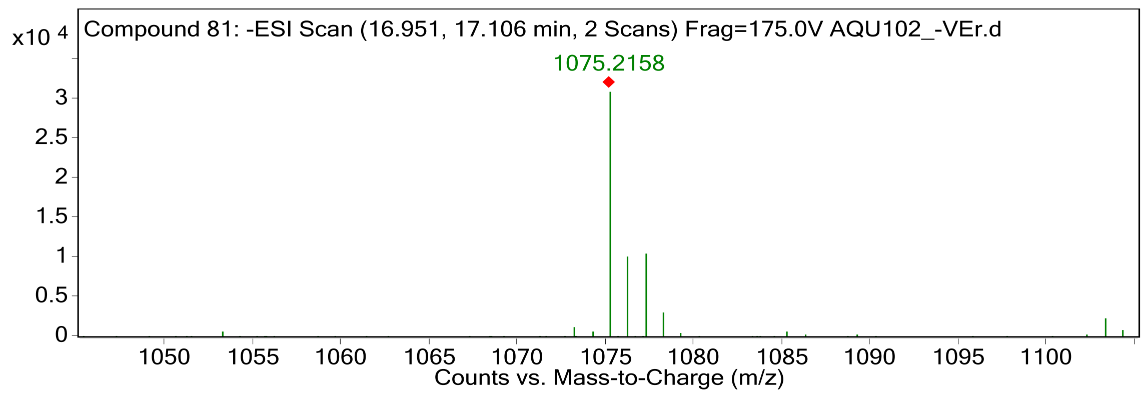

MS Spectrum Peak List

m/z	z	Abund
160.8461		29891.19
162.8432		26287.16
325.1915	1	110963.59
452.383	1	26230.27
1075.2158	1	31032.38
1076.2186	1	10116.27
1077.2134	1	10615.75
1078.2166	1	3236.46
1079.2216	1	622.85
1139.2288	1	37581.82

MSMS Spectrum

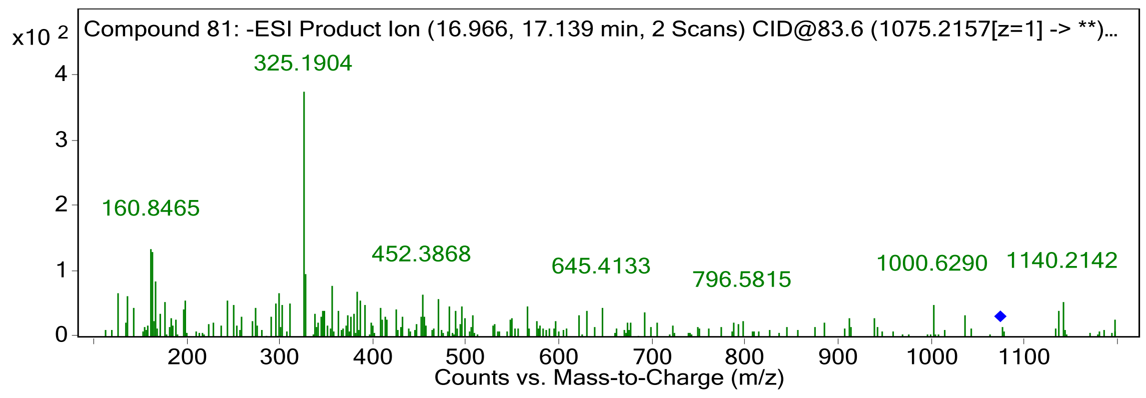

MS/MS Spectrum Peak List

m/z	z	Abund
124.9919		67.84
160.8465		135.02
162.8425		131.19
164.8407		68.31
165.0221		85.39
298.1655		66.94
325.1904	1	376.69
327.1875	1	97.33
355.3263		78.96
382.3015		68.76

Compound Label	Name	*m/z*	RT	Algorithm	Mass
Cpd 82: Hydroquinidine	**Hydroquinidine**	325.1914	17.112	Auto MS/MS	326.1982

MS Spectrum

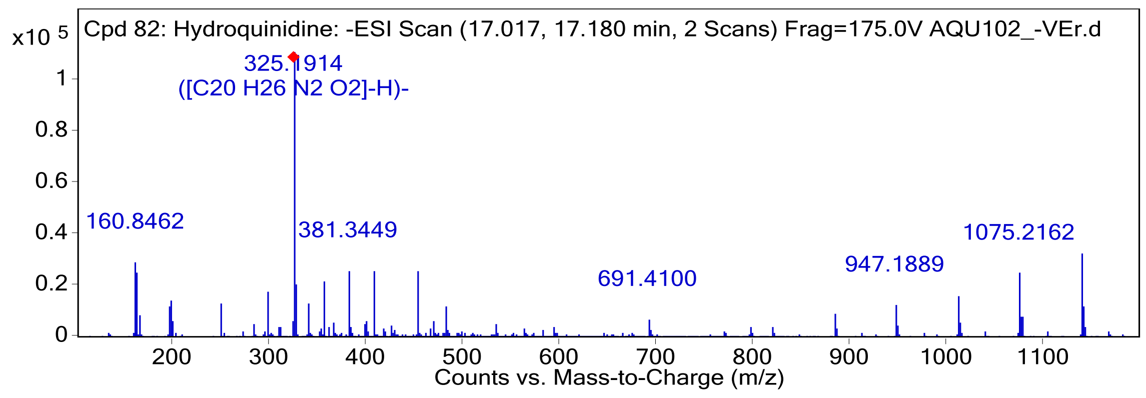

MS Zoomed Spectrum

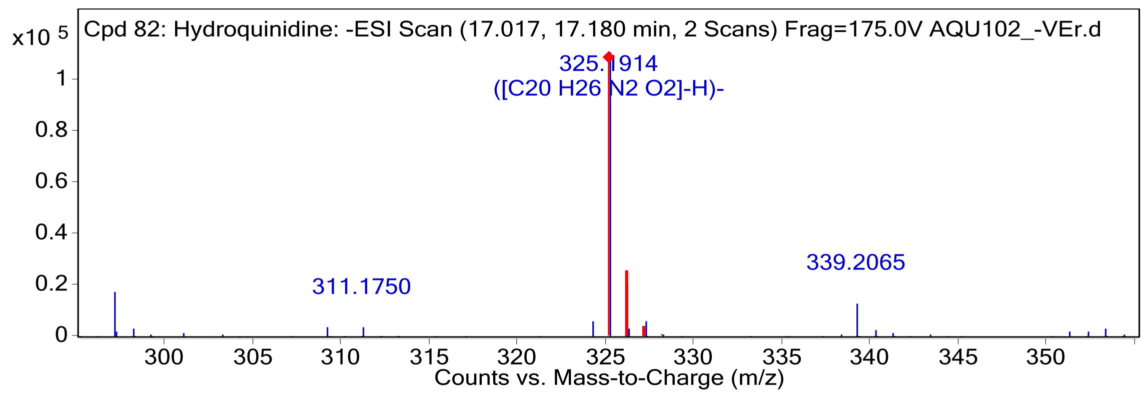

MS Spectrum Peak List

m/z	*Calc m/z*	Diff(ppm)	z	Abund	Formula	Ion
160.8462				29173.67		
162.8431				25287.86		
325.1914	325.1922	2.29	1	110803.19	C20 H26 N2 O2	(M-H)-
326.1944	326.1953	2.86	1	20691.77	C20 H26 N2 O2	(M-H)-
327.1899	327.1982	25.3	1	6078.15	C20 H26 N2 O2	(M-H)-
328.1901	328.2009	33.11	1	1184.45	C20 H26 N2 O2	(M-H)-
381.3449			1	25802.69		
407.2286			1	25642.15		
452.3828			1	25568.88		
1139.229			1	32404.2		

MSMS Spectrum

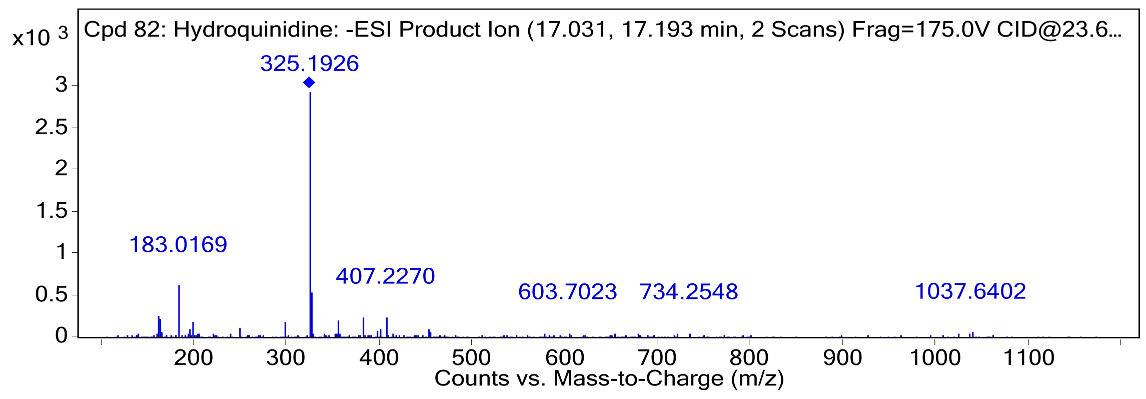

MS/MS Spectrum Peak List

m/z	z	Abund
160.8464		265.86
162.8445	1	220.61
183.0169		624.14
184.0231		357.23
197.813		198.03
325.1926	1	2938.39
326.1955	1	539.31
355.3288	1	209.58
381.3451	1	238.82
407.227		246.51

Compound Structure

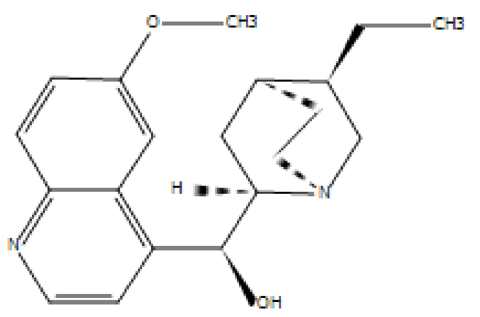

Compound Label	*m/z*	RT	Algorithm
Compound 83	452.3833	17.201	Auto MS/MS

MS Spectrum

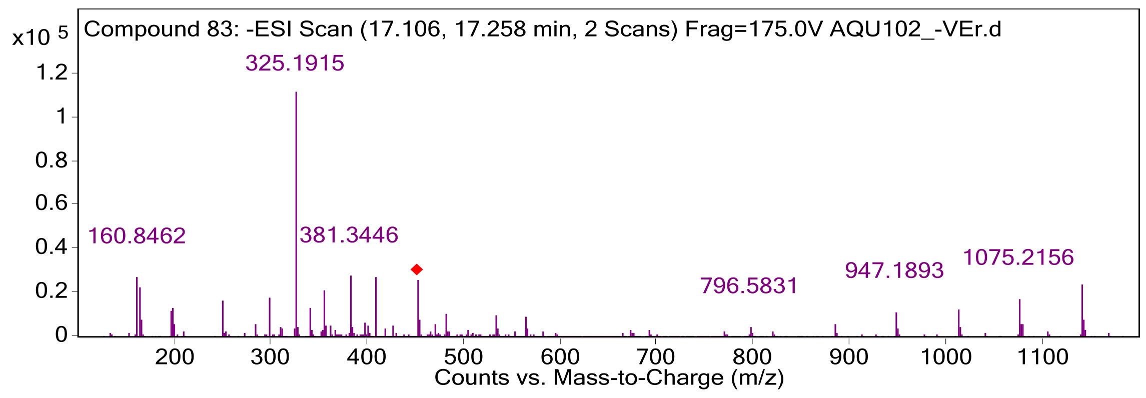

MS Zoomed Spectrum

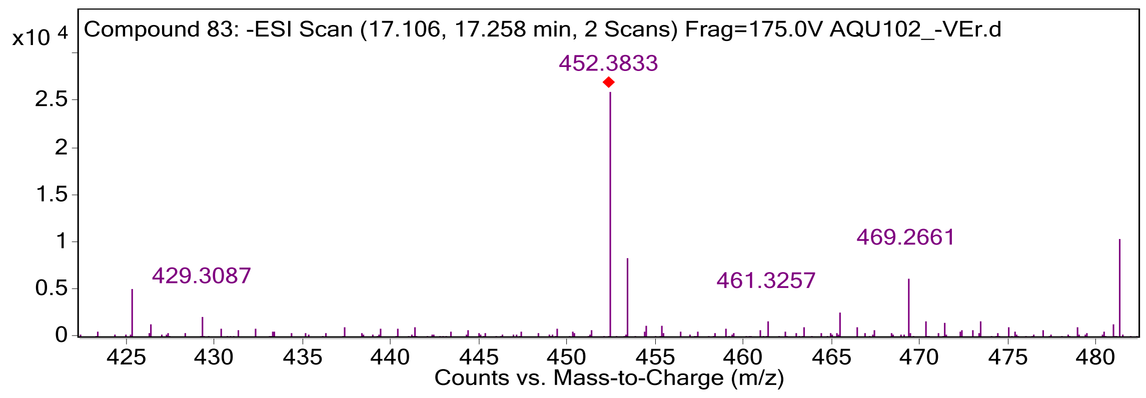

MS Spectrum Peak List

m/z	z	Abund
160.8462		27533.84
162.843		22876.11
325.1915	1	112077.19
355.3293	1	21222.3
381.3446	1	27942.31
407.2283	1	27610.21
452.3833	1	26042.58
453.386	1	8343.65
454.3882	1	1313.78
1139.2293	1	23863.42

MSMS Spectrum

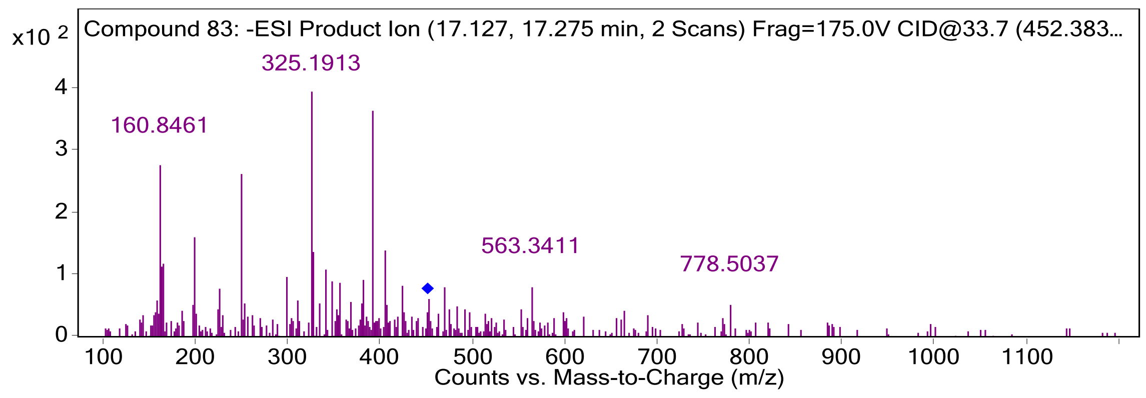

MS/MS Spectrum Peak List

m/z	z	Abund
160.8461		276.11
162.8419		113.19
164.8409		117.62
197.8134		161.5
249.1592	1	264.12
325.1913	1	396.4
326.1938	1	137.81
339.2073		110.03
390.3816	1	364.99
404.3633	1	139.29

Compound Label	*m/z*	RT	Algorithm
Compound 84	1139.2293	17.219	Auto MS/MS

MS Spectrum

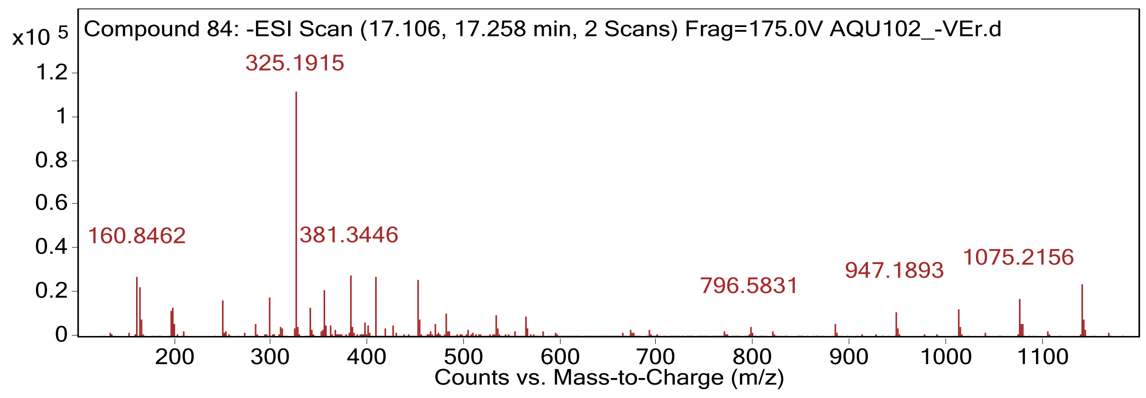

MS Zoomed Spectrum

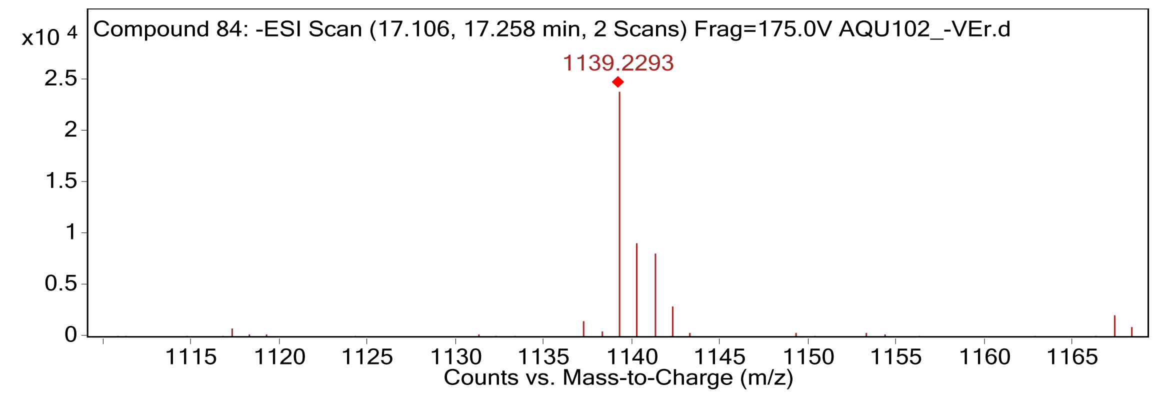

MS Spectrum Peak List

m/z	z	Abund
160.8462		27533.84
325.1915	1	112077.19
381.3446	1	27942.31
407.2283	1	27610.21
452.3833	1	26042.58
1139.2293	1	23863.42
1140.2314	1	9062.97
1141.2275	1	8186.68
1142.2296	1	3043.49
1143.2332	1	402.36

MSMS Spectrum

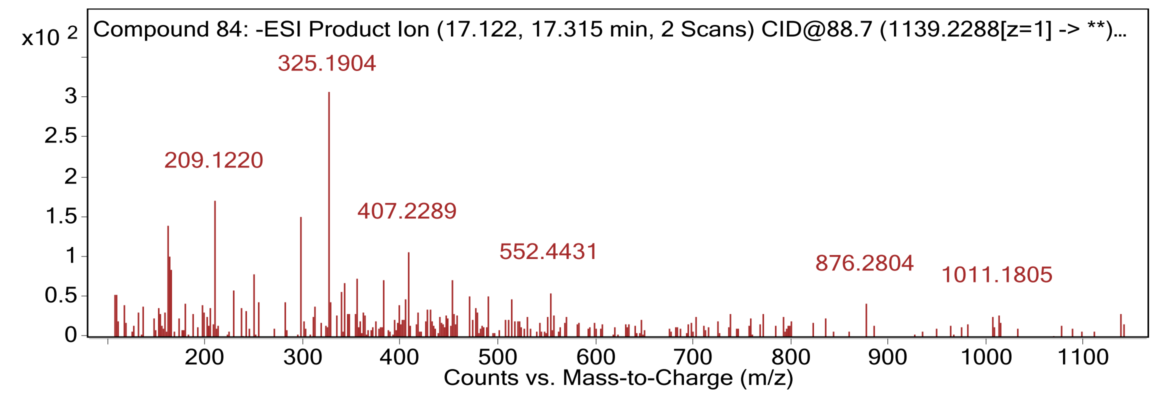

MS/MS Spectrum Peak List

m/z	z	Abund
160.8455	1	140.09
162.8437	1	100.64
164.8439		85.77
209.122		171.22
249.1571		78.56
297.1588		151.13
325.1904	1	308.72
355.3262		73
407.2289		107.17
452.3852		72.75

Compound Label	Name	*m/z*	RT	Algorithm	Mass
Cpd 85: Hydroquinidine	**Hydroquinidine**	325.1917	17.428	Auto MS/MS	326.1986

MS Spectrum

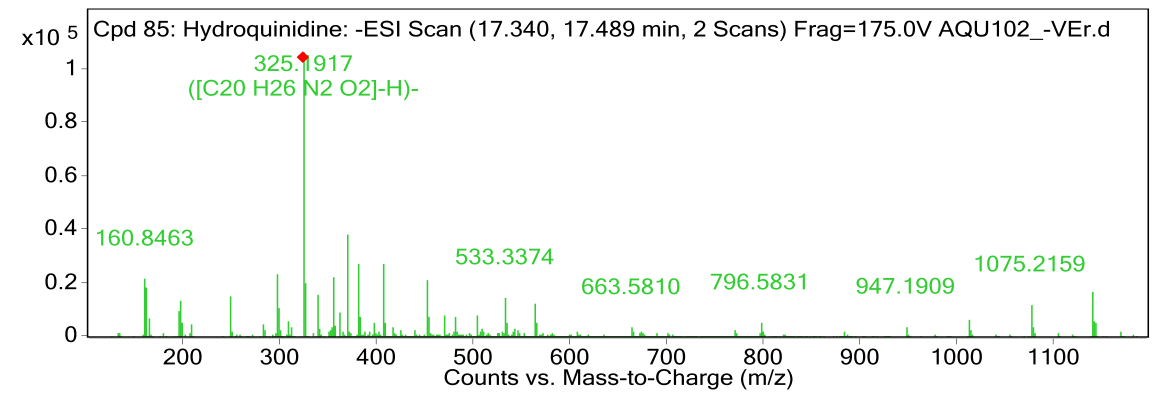

MS Zoomed Spectrum

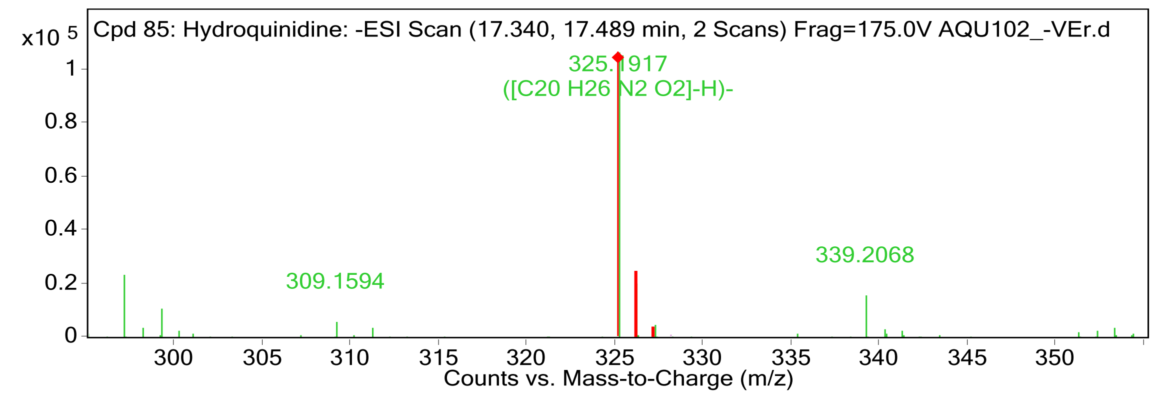

MS Spectrum Peak List

m/z	*Calc m/z*	Diff(ppm)	z	Abund	Formula	Ion
160.8463				21822.9		
297.1594			1	23372.78		
325.1917	325.1922	1.41	1	106573.33	C20 H26 N2 O2	(M-H)-
326.1944	326.1953	2.98	1	20305.66	C20 H26 N2 O2	(M-H)-
327.1913	327.1982	21.06	1	5078.69	C20 H26 N2 O2	(M-H)-
355.3293			1	22355.14		
369.3087			1	38219.09		
381.3455			1	27419.6		
407.2284			1	27246.52		
452.3832			1	21484.92		

MSMS Spectrum

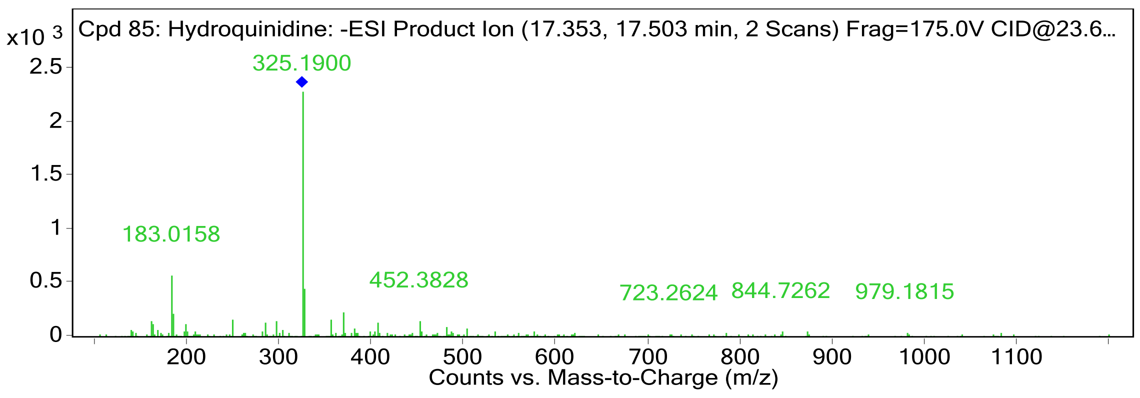

MS/MS Spectrum Peak List

m/z	z	Abund
160.8445		145.26
183.0158		578.16
184.0216	1	220.82
249.159		157.51
297.158		143.78
325.19	1	2288.41
326.1921	1	458.05
355.3273		166
369.3074		234.66
452.3828	1	149.72

Compound Structure

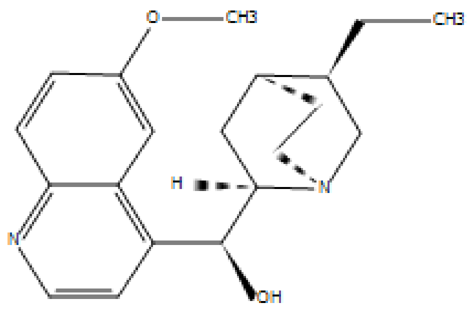

Compound Label	*m/z*	RT	Algorithm
Compound 86	369.3087	17.511	Auto MS/MS

MS Spectrum

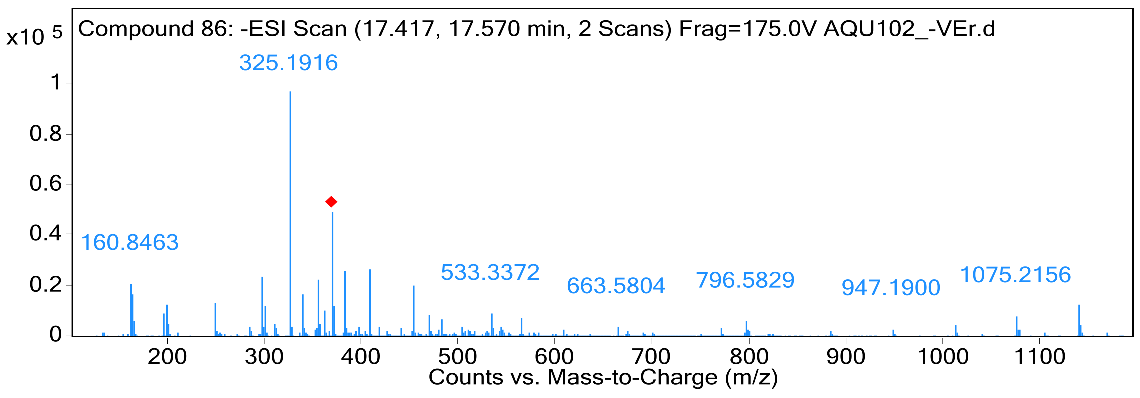

MS Zoomed Spectrum

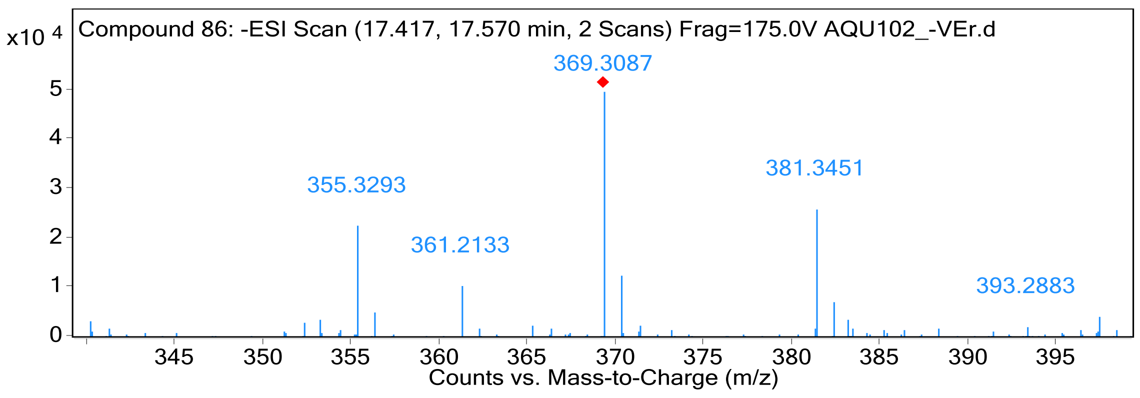

MS Spectrum Peak List

m/z	z	Abund
160.8463		21207.23
297.1594	1	23645.64
325.1916	1	97408.55
355.3293	1	22572.14
369.3087	1	49725.22
370.3119	1	12522.4
371.3204	1	2480.24
372.3229	1	516.92
381.3451	1	26011.49
407.2284	1	27064.05

MSMS Spectrum

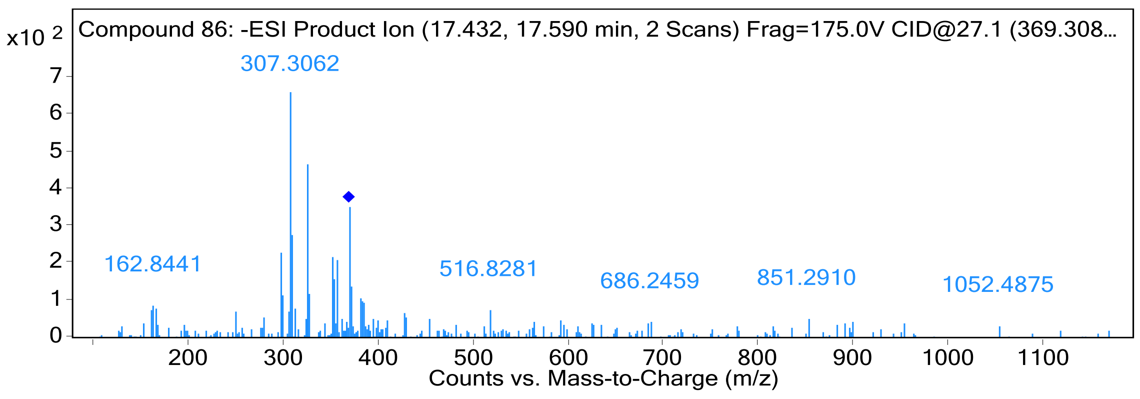

MS/MS Spectrum Peak List

m/z	z	Abund
297.1605	1	228.45
307.3062	1	661.93
308.3103	1	278.38
325.1924	1	468.79
326.195	1	117.11
351.2999		217.76
352.302		157.06
355.331		209.31
369.309	1	351.29

370.31	1	136.84

Compound Label	Name	*m/z*	RT	Algorithm	Mass
Cpd 87: Hydroquinidine	**Hydroquinidine**	325.1915	17.721	Auto MS/MS	326.1985

MS Spectrum

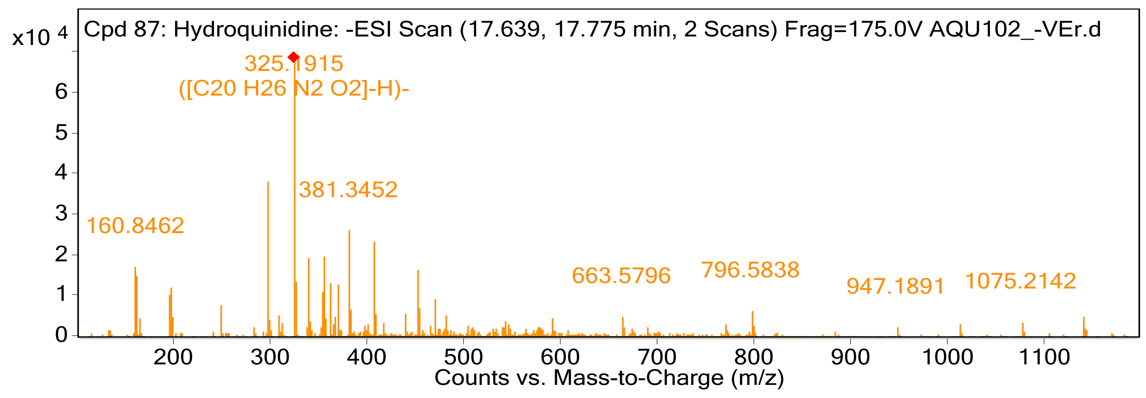

MS Zoomed Spectrum

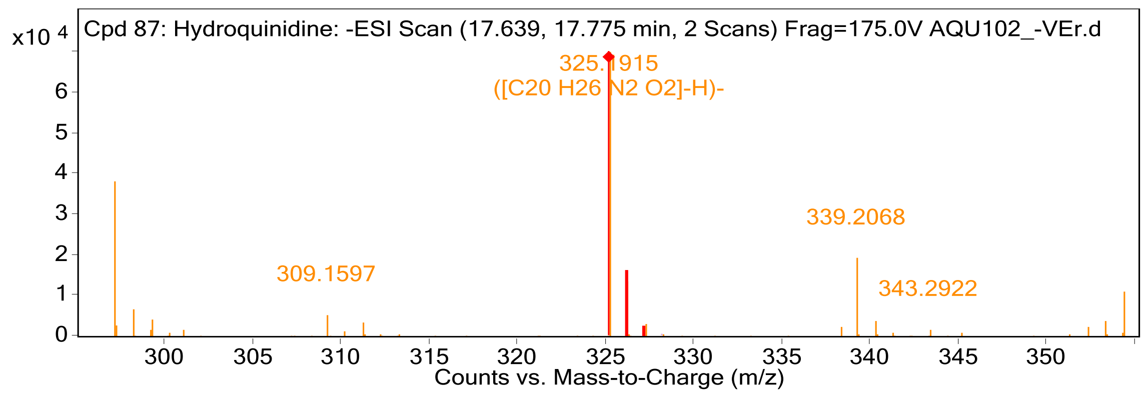

MS Spectrum Peak List

m/z	*Calc m/z*	Diff(ppm)	z	Abund	Formula	Ion
160.8462				17408.03		
297.1593			1	38378.5		
325.1915	325.1922	1.99	1	70107.21	C20 H26 N2 O2	(M-H)-
326.1943	326.1953	3.29	1	13903.96	C20 H26 N2 O2	(M-H)-
327.1909	327.1982	22.29	1	3253.97	C20 H26 N2 O2	(M-H)-

m/z	*Calc m/z*	Diff(ppm)	z	Abund	Formula	Ion
339.2068			1	19463.23		
355.3292			1	19981.64		
381.3452			1	26268.93		
407.2284			1	23315.62		
452.3832			1	16727.9		

MSMS Spectrum

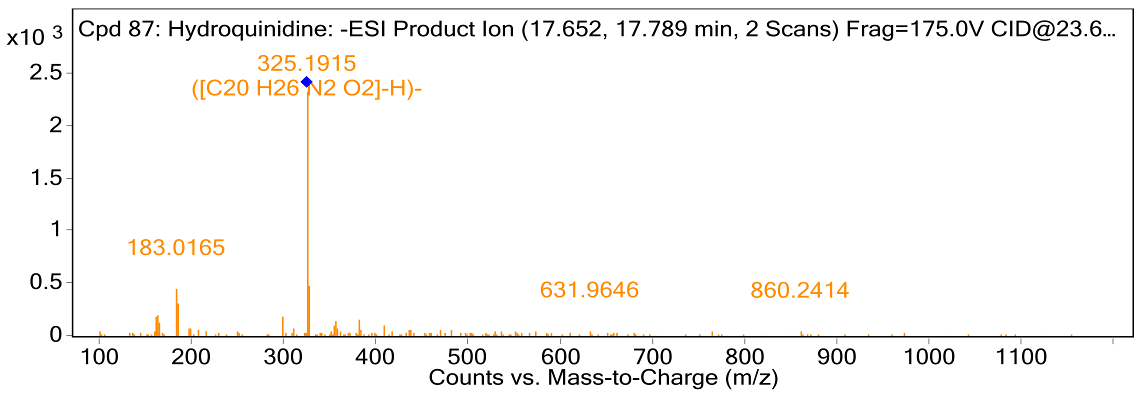

MS/MS Spectrum Peak List

m/z	*Calc m/z*	Diff (ppm)	z	Abund	Formula	Ion
160.8462				190.08		
162.8416				203.51		
164.8402				134.31		
183.0165				462.42		
184.0245				326.47		
297.1576			1	201.3		
325.1915	325.1922	2	1	2336.93	C20 H26 N2 O2	(M-H)-
326.1945			1	484.35		
355.3293				150.68		
381.348			1	167.44		

Compound Structure

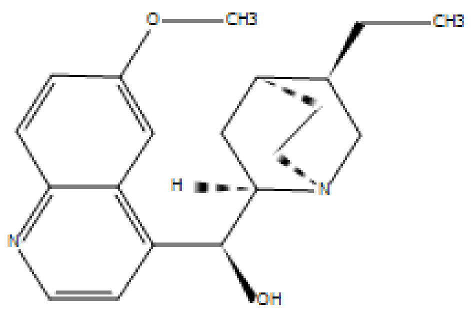

Compound Label	Name	*m/z*	RT	Algorithm	Mass
Cpd 88: 3-O-Mycarosylerythronolide B	**3-O-Mycarosylerythronolid**	591.3433	17.857	Auto MS/MS	546.3457

MS Spectrum

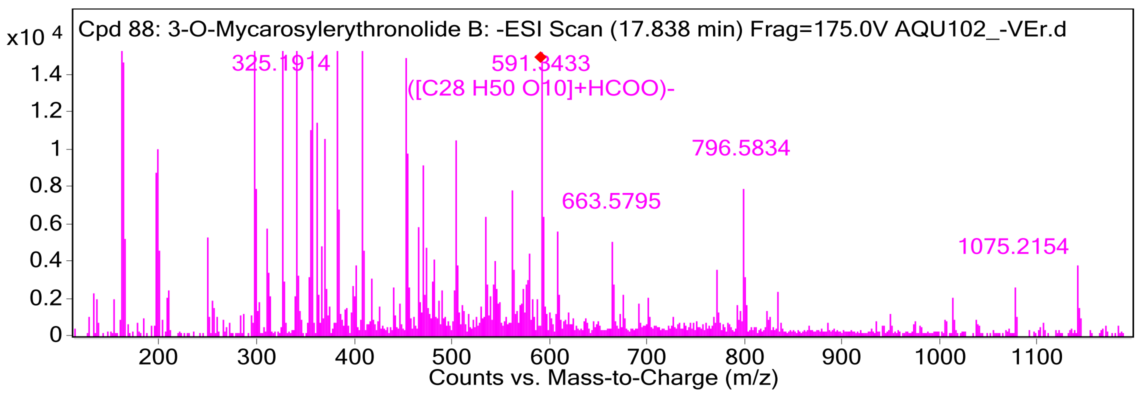

MS Zoomed Spectrum

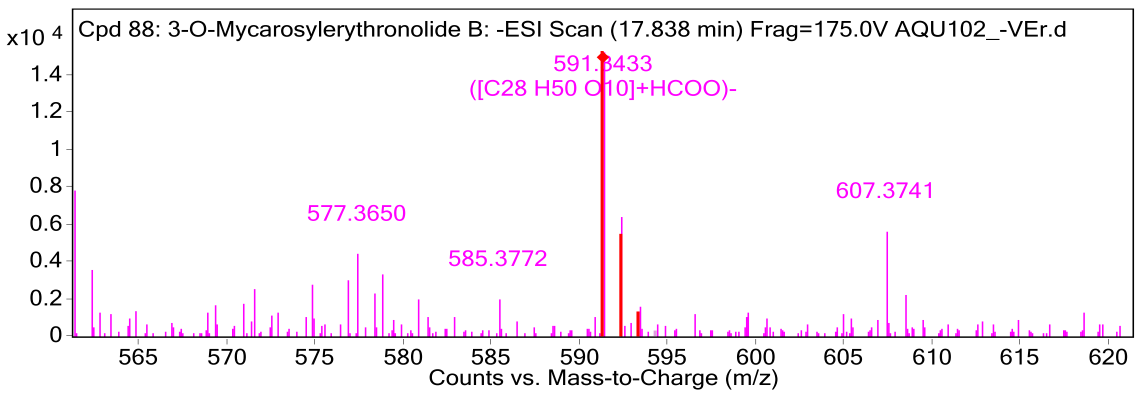

MS Spectrum Peak List

m/z	*Calc m/z*	Diff(ppm)	z	Abund	Formula	Ion
160.8461				16677.38		
297.1595			1	43051.62		
325.1914			1	54164.69		
339.2072			1	21431.89		
355.3291			1	20719.73		
381.3453			1	24813.02		
407.2286			1	19141.67		
591.3433	591.3386	-7.98	1	15235.71	C28 H50 O10	(M+HCOO)-
592.3477	592.342	-9.6	1	6446.97	C28 H50 O10	(M+HCOO)-
593.3543	593.3446	-16.32	1	1643.4	C28 H50 O10	(M+HCOO)-

MSMS Spectrum

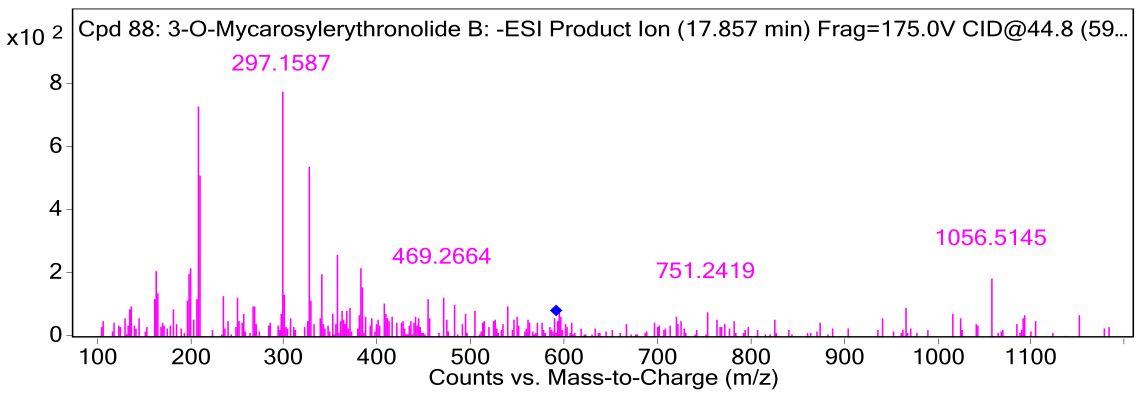

MS/MS Spectrum Peak List

m/z	z	Abund
162.8424		211.17

197.8134		199.63
199.8115		217.11
207.1075		732.02
209.1244		511.26
297.1587	1	779.39
325.192		538.71
339.2116		202.57
355.3309	1	263.06
381.3458		218.8

Compound Structure

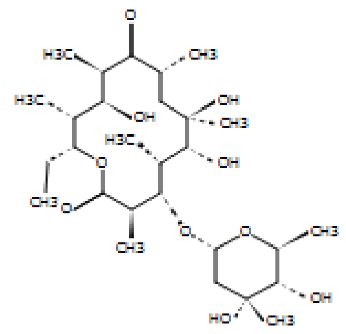

Compound Label	Name	*m/z*	RT	Algorithm	Mass
Cpd 89: 24-Methylcycloart-23-en-3beta-yl acetate	**24-Methylcycloart-23-en-3beta-yl acetate**	481.4105	18.606	Auto MS/MS	482.4176

MS Spectrum

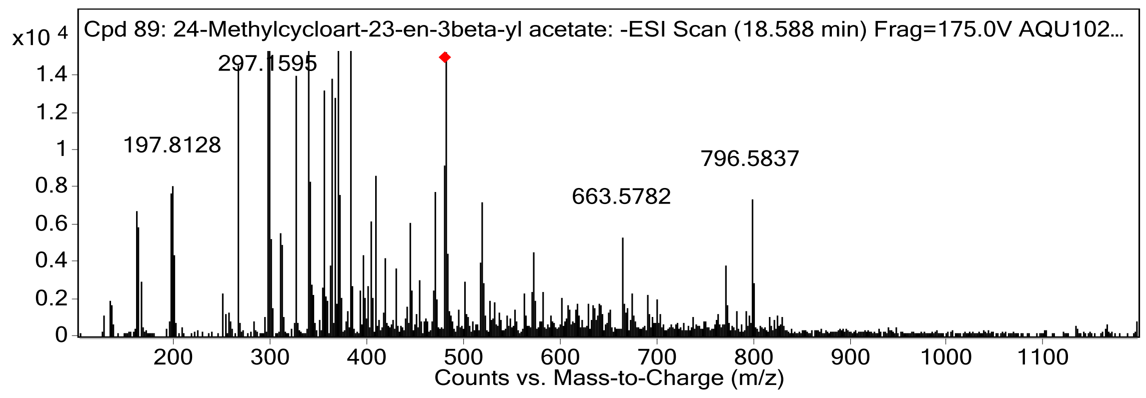

MS Zoomed Spectrum

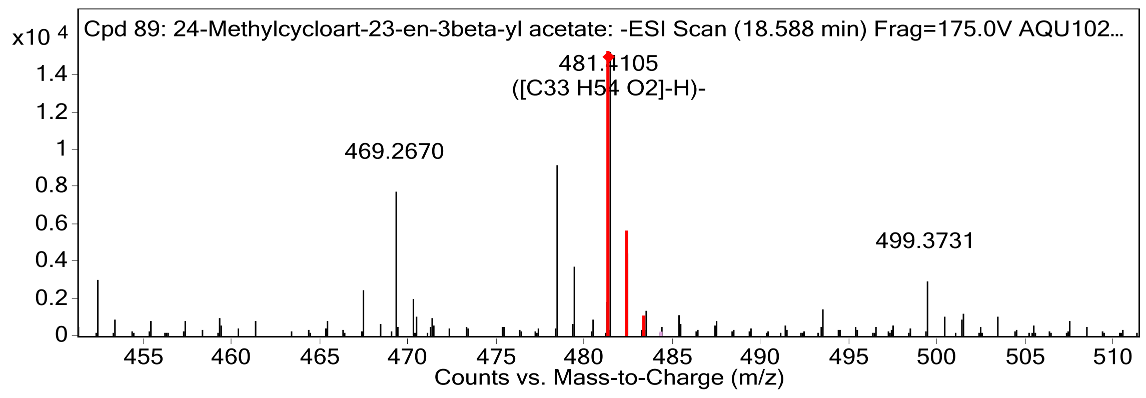

MS Spectrum Peak List

m/z	*Calc m/z*	Diff(ppm)	z	Abund	Formula	Ion
265.1536			1	14604.51		
297.1595			1	99076.46		
298.1626			1	17070.51		
325.1915			1	14042.76		
339.207			1	41114.25		
369.3451			1	28425		
381.3453			1	17359.64		
481.4105	481.4051	-11.11	1	15273.82	C33 H54 O2	(M-H)-
482.4136	482.4085	-10.65	1	4488.82	C33 H54 O2	(M-H)-
483.4158	483.4118	-8.35	1	1394.44	C33 H54 O2	(M-H)-

MSMS Spectrum

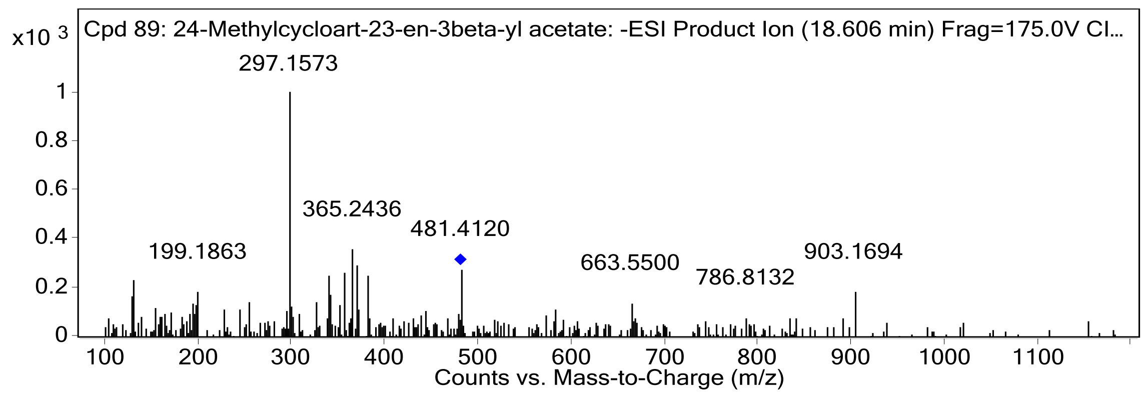

MS/MS Spectrum Peak List

m/z	z	Abund
130.0909		237.13
199.1863		183.75
297.1573	1	1004.72
339.2055		254.41
355.3242		265.33
365.2436		357.73
369.3453	1	294.33
381.2552		249.63
481.412		277.2
903.1694		183.63

Compound Structure

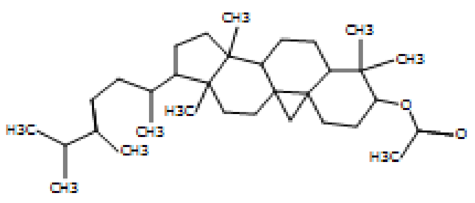

Compound Label	Name	*m/z*	RT	Algorithm	Mass
Cpd 90: Atenolol	**Atenolol**	265.1535	18.648	Auto MS/MS	266.1605

MS Spectrum

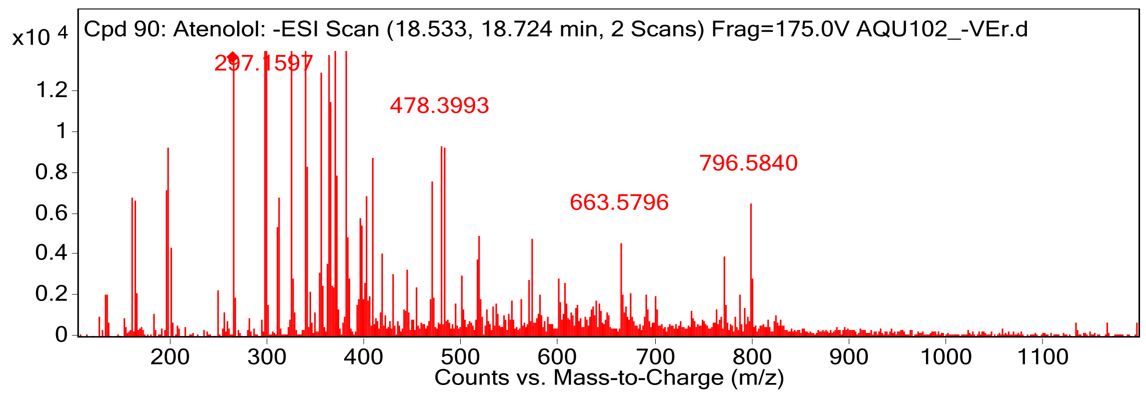

MS Zoomed Spectrum

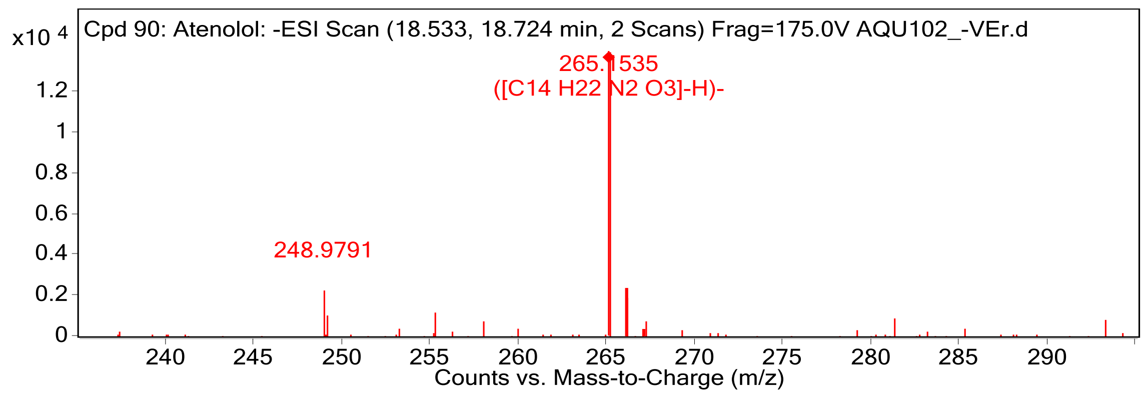

MS Spectrum Peak List

m/z	*Calc m/z*	Diff(ppm)	z	Abund	Formula	Ion
265.1535	265.1558	8.42	1	13956.53	C14 H22 N2 O3	(M-H)-
266.1567	266.1589	8.12	1	1974.33	C14 H22 N2 O3	(M-H)-
267.1524	267.1613	33.6	1	756.21	C14 H22 N2 O3	(M-H)-
297.1597			1	146640.45		
298.1629			1	24787.75		
325.1917			1	16879.79		
339.2071			1	40948.94		
362.2446			1	13839.64		
369.3451			1	27845.15		
381.345			1	17093.64		

MSMS Spectrum

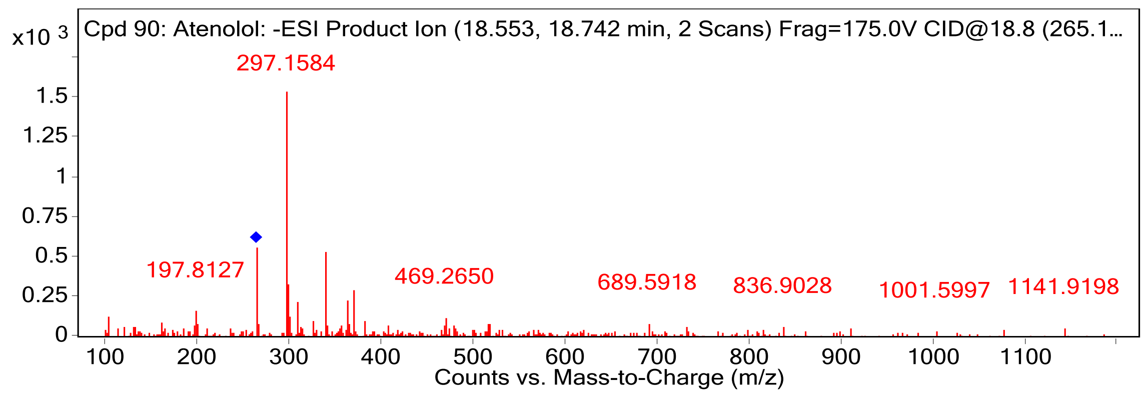

MS/MS Spectrum Peak List

m/z	z	Abund
102.5508		133.63
197.8127		163.78
265.1545	1	558.31
297.1584	1	1541.38
298.1619	1	331.87
299.1598	1	131.88
309.1621		216.98
339.207	1	534.17
362.2437	1	233.95
369.3437	1	294.13

Compound Structure

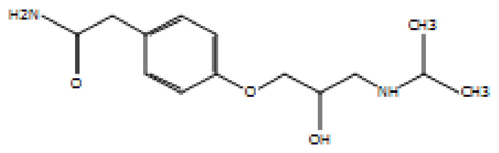

Compound Label	Name	*m/z*	RT	Algorithm	Mass
Cpd 91: 22,23-Dihydroergosterol	**22,23-Dihydroergosterol**	397.3404	18.793	Auto MS/MS	398.3477

MS Spectrum

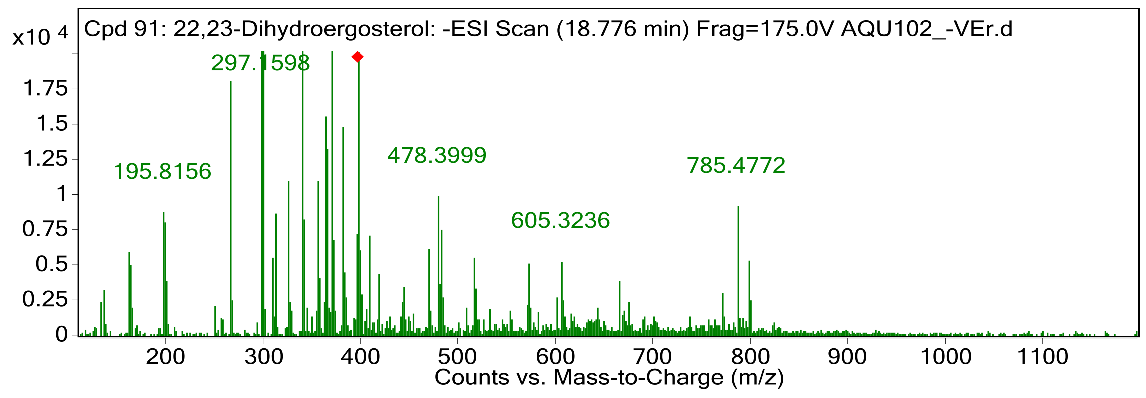

MS Zoomed Spectrum

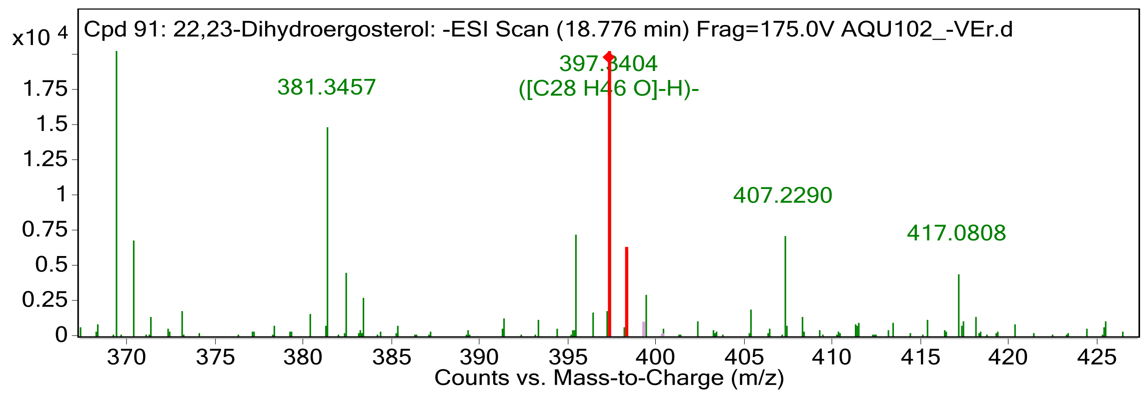

MS Spectrum Peak List

m/z	*Calc m/z*	Diff(ppm)	z	Abund	Formula	Ion
265.1538			1	18113.95		
297.1598			1	276550.78		
298.1629			1	45569.36		
339.2068			1	43239.12		
362.2449			1	15651		
365.2444			1	13361.67		
369.3455			1	29238.77		
381.3457			1	14888.51		
397.3404	397.3476	18.18	1	20232.76	C28 H46 O	(M-H)-
398.344	398.351	17.51	1	6183.43	C28 H46 O	(M-H)-

MSMS Spectrum

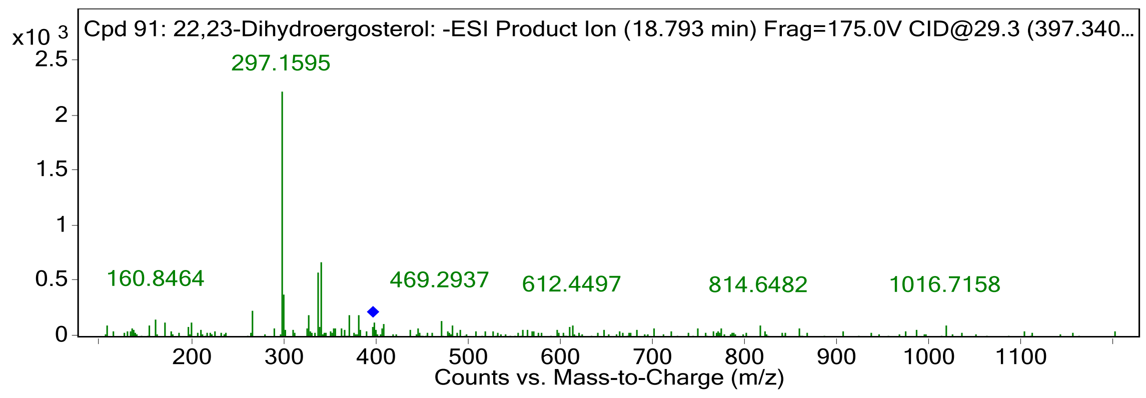

MS/MS Spectrum Peak List

m/z	z	Abund
160.8464		159.01
265.1557		244.25
297.1595	1	2228.44
298.1642	1	389.08
299.1545	1	165.13
325.1917		201
335.3412		587.88
339.2077	1	673.46
369.347	1	196
379.3271	1	193.74

Compound Structure

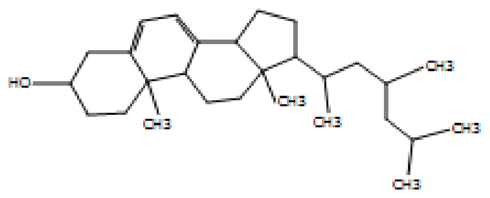

Compound Label	Name	*m/z*	RT	Algorithm	Mass
Cpd 92: Atenolol	**Atenolol**	265.1539	19.009	Auto MS/MS	266.161

MS Spectrum

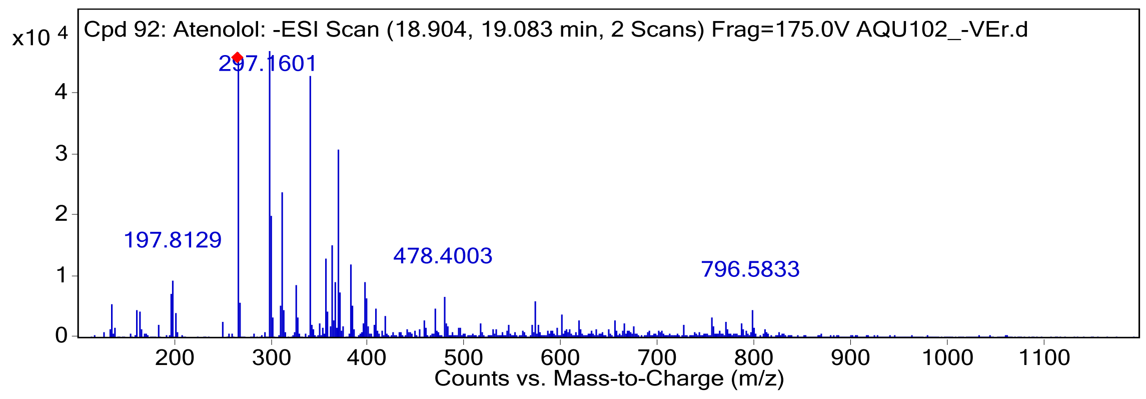

MS Zoomed Spectrum

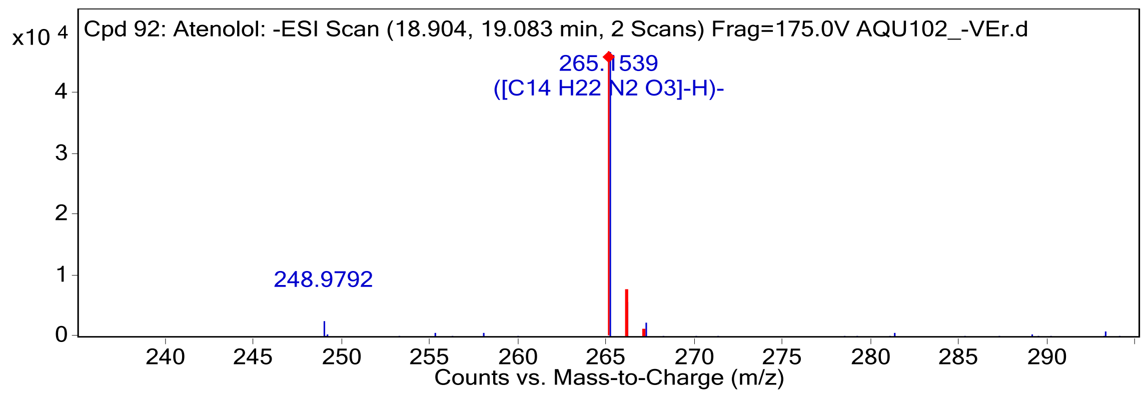

MS Spectrum Peak List

m/z	*Calc m/z*	Diff(ppm)	z	Abund	Formula	Ion
265.1539	265.1558	7	1	46820.56	C14 H22 N2 O3	(M-H)-
266.1573	266.1589	5.92	1	5859.2	C14 H22 N2 O3	(M-H)-
267.1535	267.1613	29.47	1	2324.44	C14 H22 N2 O3	(M-H)-
297.1601			1	464044		
298.163			1	77870.13		
299.1593			1	19923.42		
311.1756			1	24001.31		
339.2072			1	42994.18		
362.2446			1	15215.71		
369.3453			1	30938.43		

MSMS Spectrum

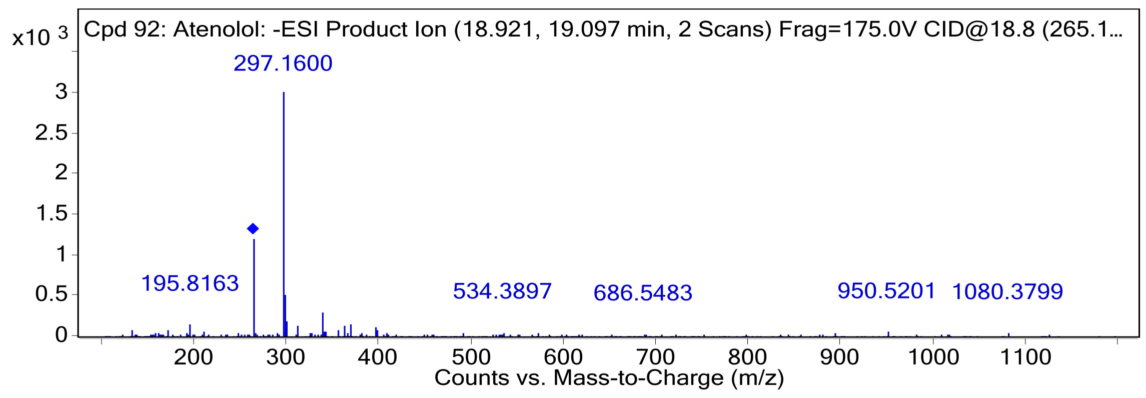

MS/MS Spectrum Peak List

m/z	z	Abund
195.8163		158.75
265.1537	1	1217.31
297.16	1	3017.85
298.1631	1	528.85
299.155	1	200.33
311.1734	1	142.58
339.2067	1	311.53
362.2481		146.41
369.3469		167.03
395.36		122.3

Compound Structure

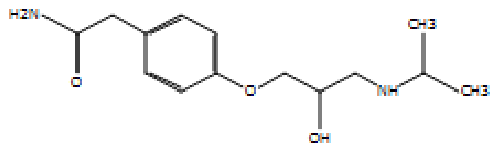

Compound Label	*m/z*	RT	Algorithm
Compound 93	395.3612	19.105	Auto MS/MS

MS Spectrum

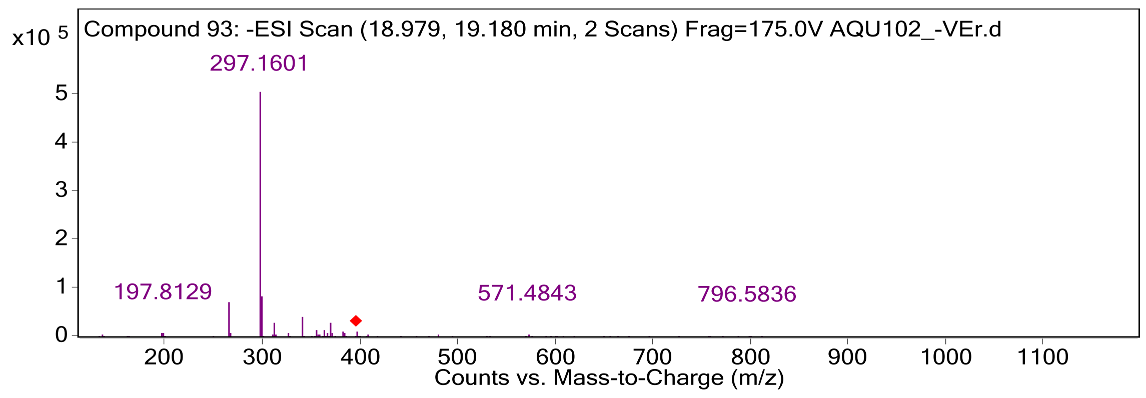

MS Zoomed Spectrum

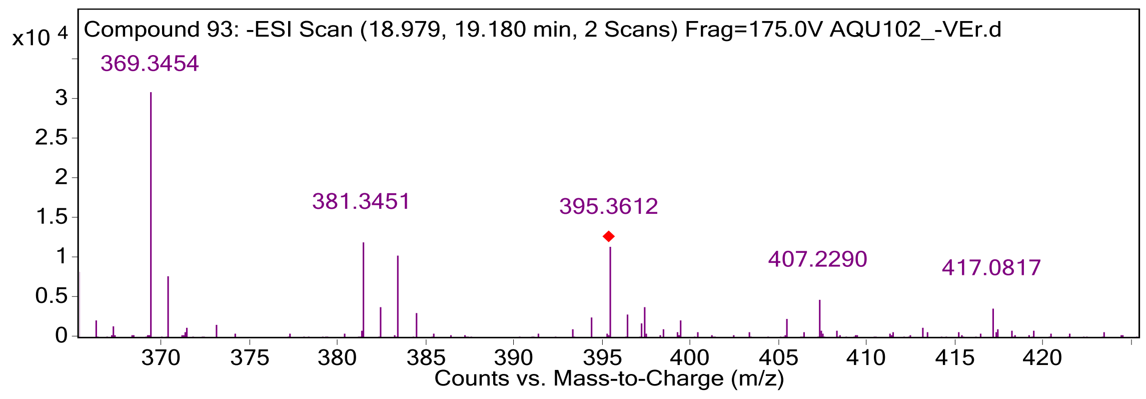

MS Spectrum Peak List

m/z	z	Abund
265.1539	1	71420.38
297.1601	1	506108.13
298.163	1	84763.97
311.1753	1	31546.24
339.2072	1	42030.14
369.3454	1	31046.77
395.3612	1	11431.76
396.3648	1	2902.4
397.3693	1	595.71

398.3734	1	147.98

MSMS Spectrum

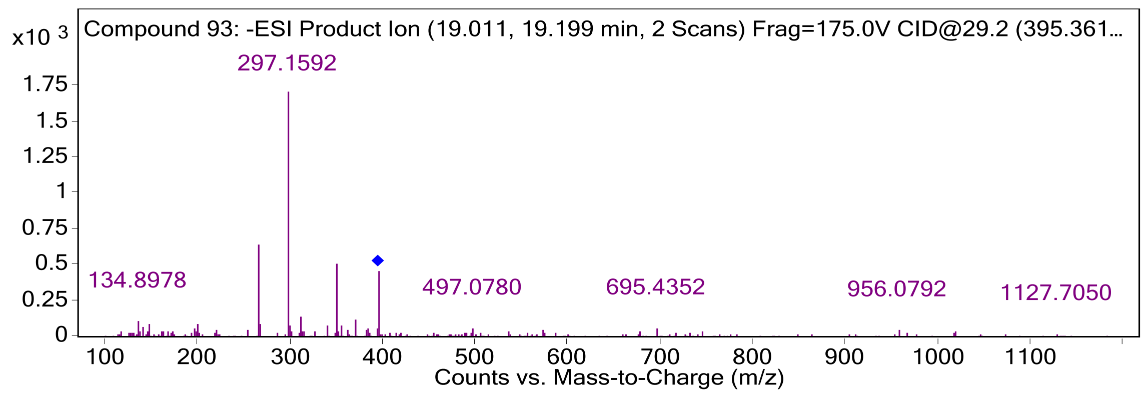

MS/MS Spectrum Peak List

m/z	z	Abund
134.8978		111.21
199.8072		90.38
265.154	1	645.57
297.1592	1	1713.98
298.1625	1	451.15
311.174	1	144.32
349.3553	1	511.1
350.3573	1	196.06
369.3459		124.91
395.3604		457.05

Compound Label	*m/z*	RT	Algorithm
Compound 94	383.3611	19.261	Auto MS/MS

MS Spectrum

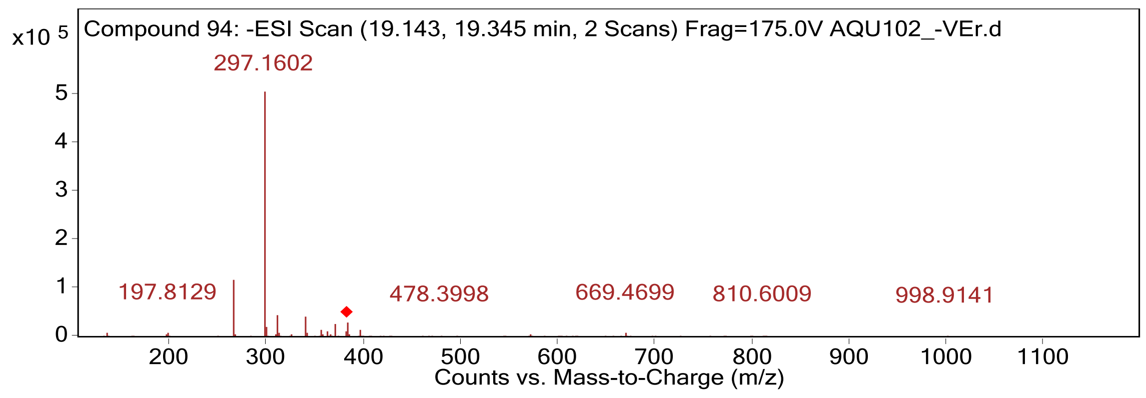

MS Zoomed Spectrum

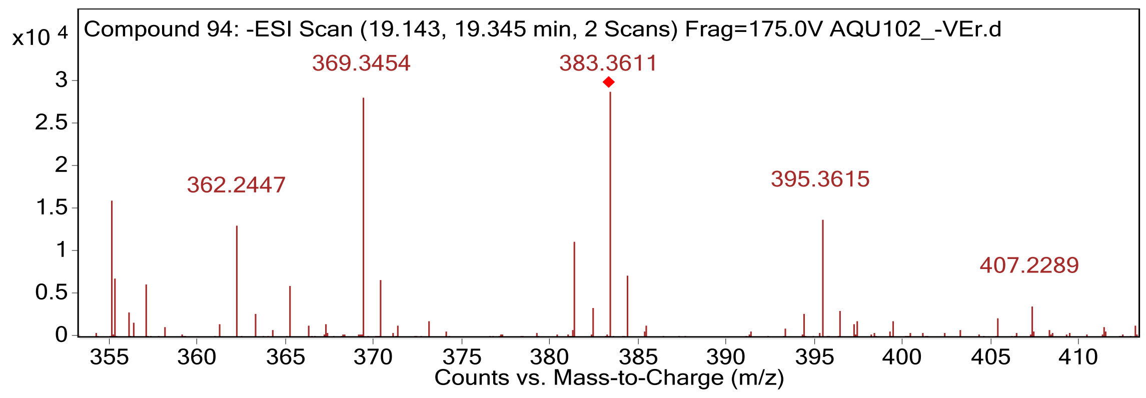

MS Spectrum Peak List

m/z	z	Abund
265.1539	1	117189.13
297.1602	1	505587.5
298.1631	1	82053.63
299.159	1	22175.06
311.1755	1	43975.55
339.2072	1	41713.54
369.3454	1	28045.2
383.3611	1	28812.13
384.3647	1	7166.61
385.3663	1	1366.8

MSMS Spectrum

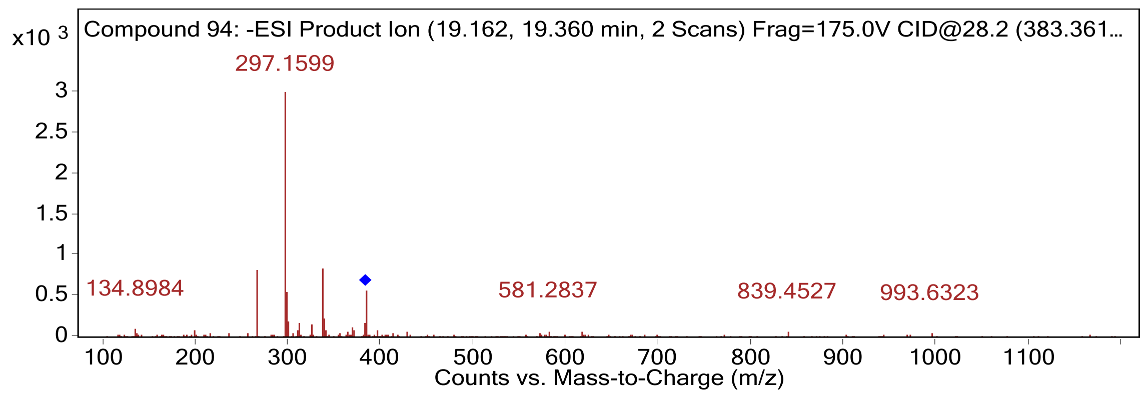

MS/MS Spectrum Peak List

m/z	z	Abund
265.1542	1	829.04
297.1599	1	3012.24
298.1632	1	567.03
299.1582	1	197.34
311.1765	1	178.77
337.3549	1	845.66
339.2073	1	243.21
382.3495		184.17
383.3607	1	578.59
384.3641	1	252.84

Compound Label	Name	*m/z*	RT	Algorithm	Mass
Cpd 95: Atenolol	**Atenolol**	265.1541	19.378	Auto MS/MS	266.1611

MS Spectrum

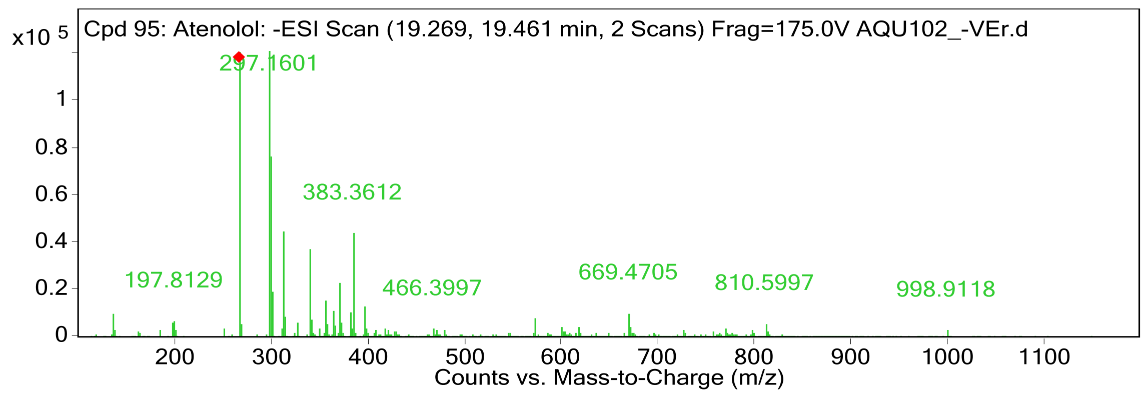

MS Zoomed Spectrum

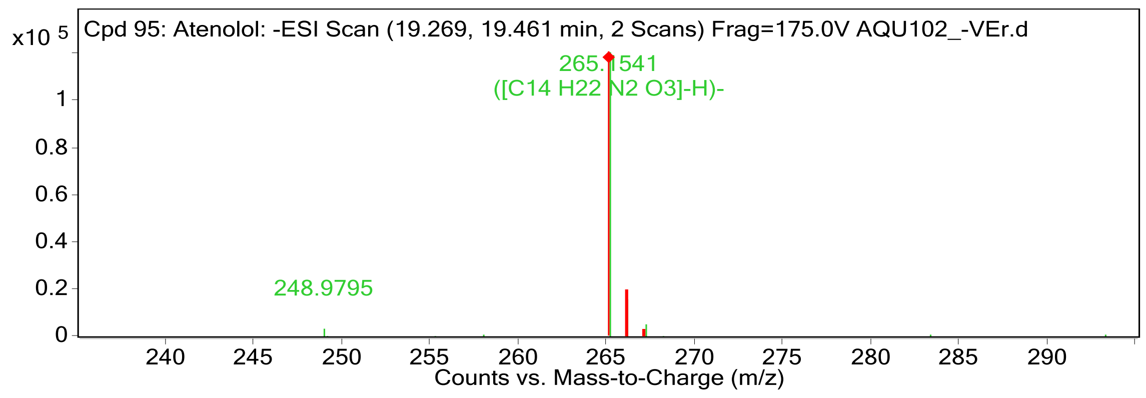

MS Spectrum Peak List

m/z	*Calc m/z*	Diff(ppm)	z	Abund	Formula	Ion
265.1541	265.1558	6.37	1	120822.08	C14 H22 N2 O3	(M-H)-
266.1571	266.1589	6.53	1	16357.28	C14 H22 N2 O3	(M-H)-
267.1522	267.1613	34.19	1	5556.38	C14 H22 N2 O3	(M-H)-
297.1601			1	455586.88		
298.163			1	76905.27		
299.1589			1	19235.49		
311.1756			1	44541.6		
339.2072			1	37228		
369.3452			1	23214.27		
383.3612			1	44197.55		

MSMS Spectrum

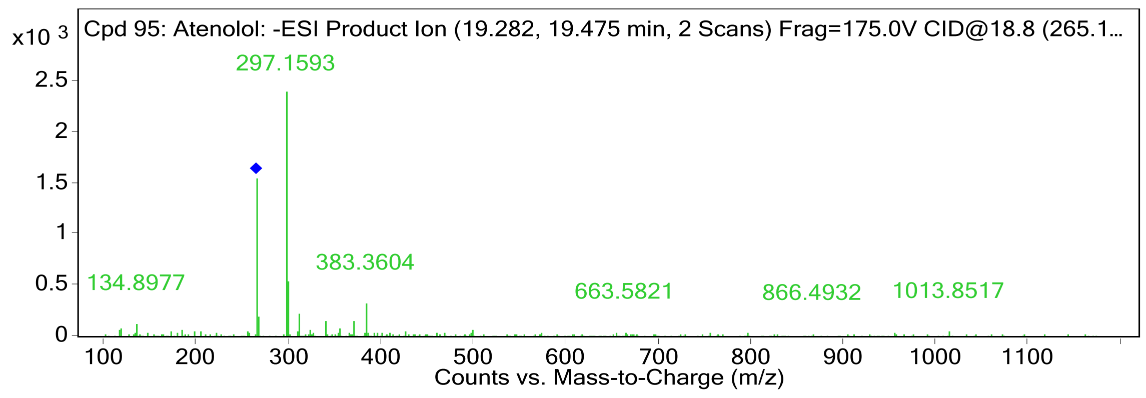

MS/MS Spectrum Peak List

m/z	z	Abund
134.8977		130.55
265.1534	1	1560.43
266.1566	1	206.47
297.1593	1	2404.73
298.1621	1	546.76
299.1577	1	92.47
311.1759		224.66
339.2061	1	152.95
369.3442		162.2
383.3604	1	331.59

Compound Structure

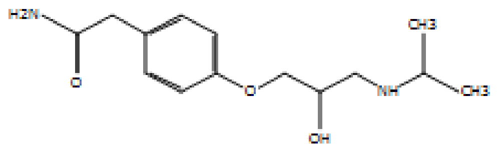

**Compound Label**	***m/z***	**RT**	**Algorithm**
Compound 96	395.3613	19.494	Auto MS/MS

MS Spectrum

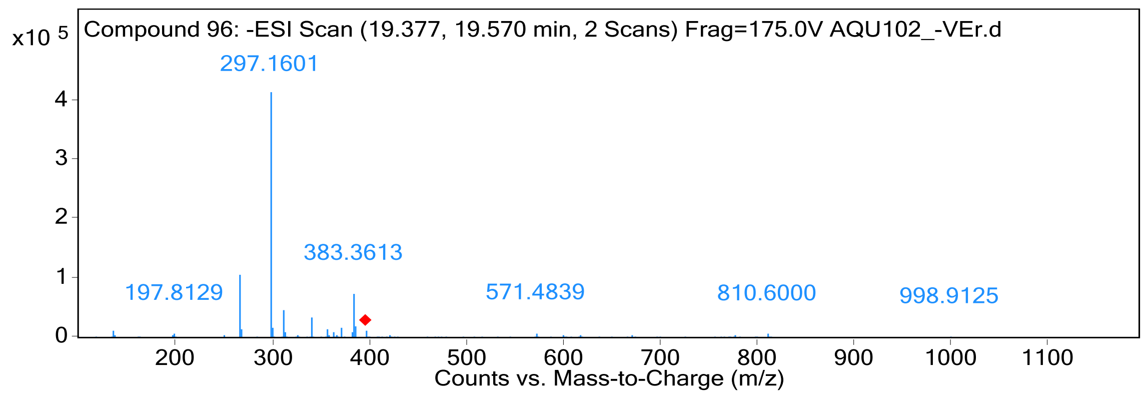

MS Zoomed Spectrum

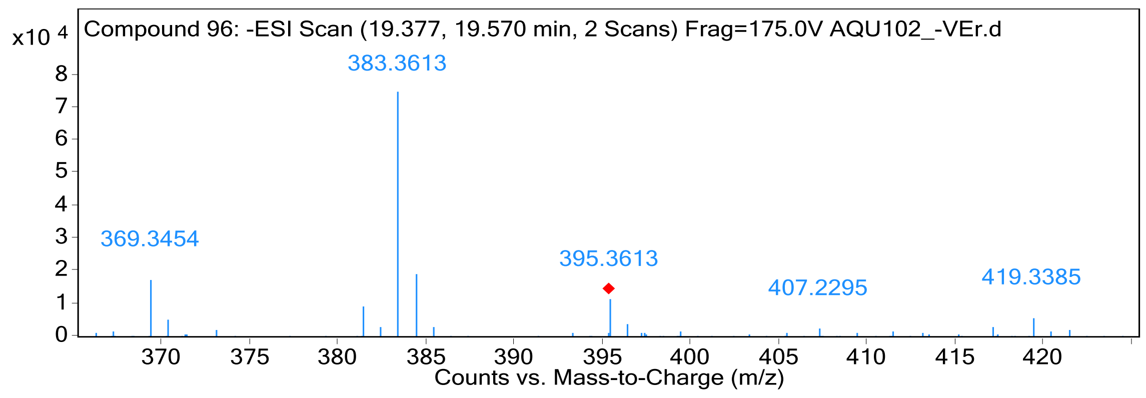

MS Spectrum Peak List

m/z	z	Abund
265.154	1	106402.67
297.1601	1	414124.25
298.1629	1	69269.56
311.1755	1	47588.24
339.2072	1	33918.19
383.3613	1	74940.49
395.3613	1	11471.74
396.363	1	3855.12
397.3668	1	1031.21
398.3759	1	289.98

MSMS Spectrum

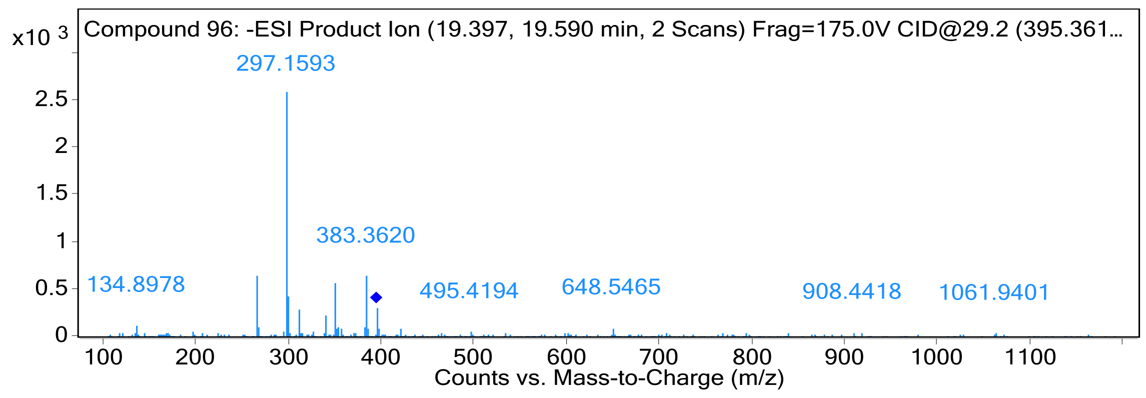

MS/MS Spectrum Peak List

m/z	z	Abund
134.8978		126.52
265.155	1	646.94
297.1593	1	2596.87
298.1628	1	440.92
299.1575	1	123.47
311.1749	1	288.03
339.2075		228.36
349.3559	1	574.58
383.362	1	647.26
395.3623	1	307.94

Compound Label	*m/z*	RT	Algorithm
Compound 97	383.3612	19.659	Auto MS/MS

MS Spectrum

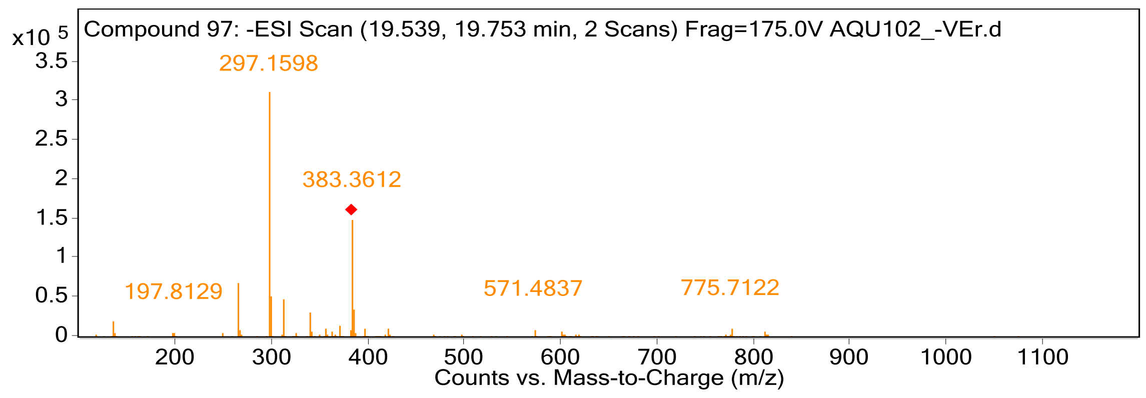

MS Zoomed Spectrum

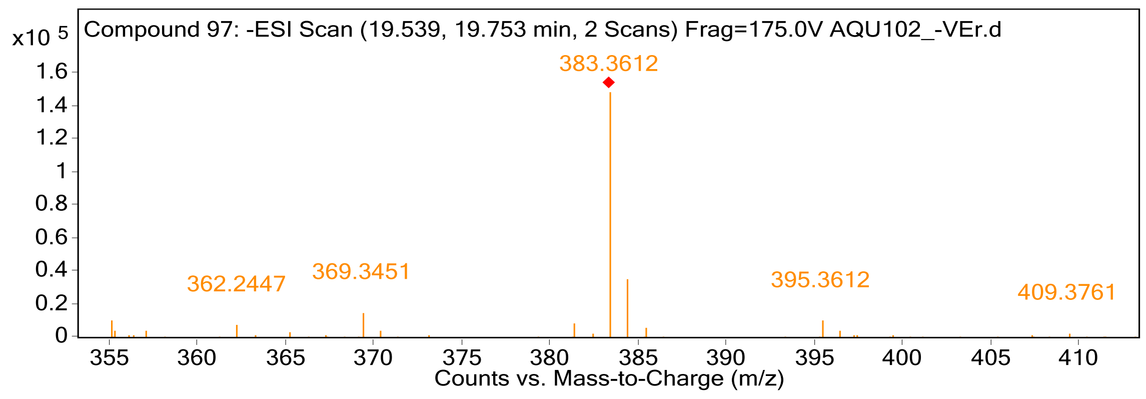

MS Spectrum Peak List

m/z	z	Abund
134.8982	1	21291.34
265.1538	1	69980.13
297.1598	1	312373.88
298.1629	1	52184.76
311.1753	1	47880.59
339.207	1	31467.84
369.3451	1	15280.95
383.3612	1	148795.41
384.3645	1	35650.14
385.3674	1	5860.33

MSMS Spectrum

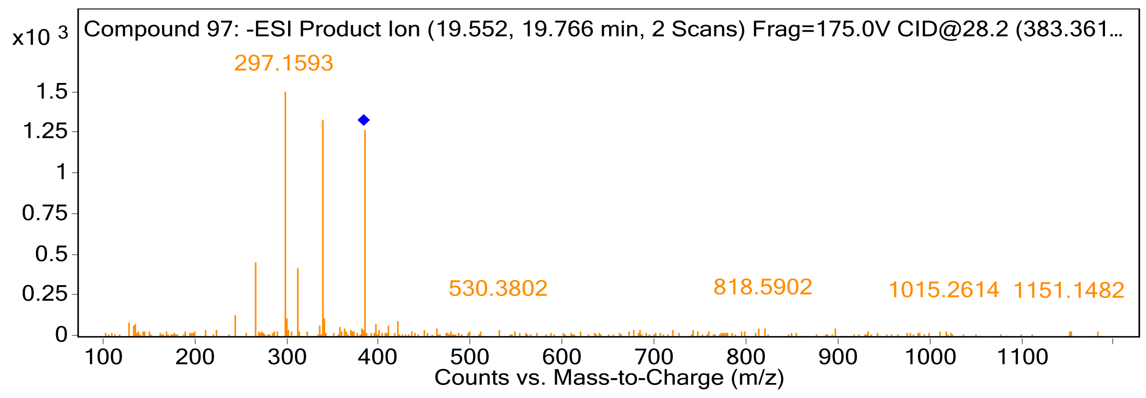

MS/MS Spectrum Peak List

m/z	z	Abund
243.0068		138.35
265.1542		462.47
297.1593	1	1507.85
298.1647	1	259.48
299.16	1	113.76
311.1746	1	427.92
337.3547	1	1334.65
338.3609	1	218.19
383.3614	1	1272.77
384.3632	1	504.59

Compound Label	Name	*m/z*	RT	Algorithm	Mass
Cpd 98: Atenolol	**Atenolol**	265.1538	19.767	Auto MS/MS	266.1609

MS Spectrum

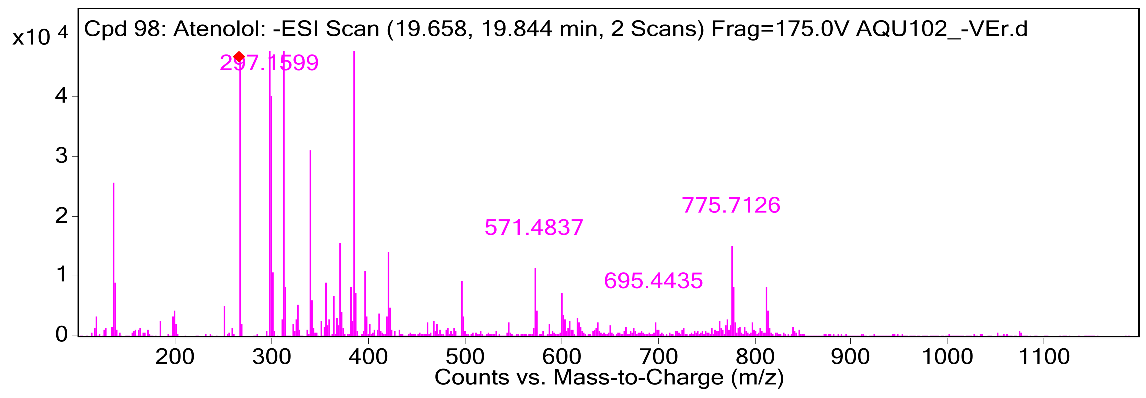

MS Zoomed Spectrum

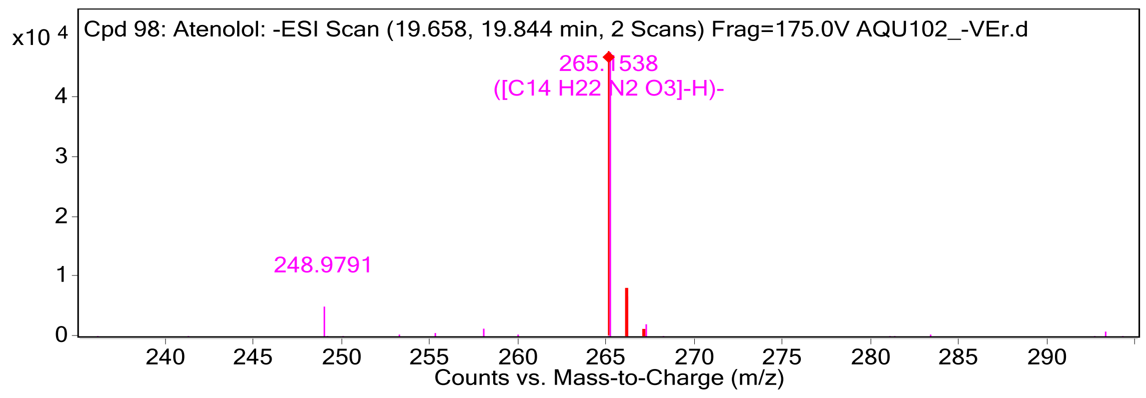

MS Spectrum Peak List

m/z	*Calc m/z*	Diff(ppm)	z	Abund	Formula	Ion
134.8983			1	25876.21		
265.1538	265.1558	7.27	1	47626.09	C14 H22 N2 O3	(M-H)-
266.1572	266.1589	6.38	1	6786.7	C14 H22 N2 O3	(M-H)-
267.1526	267.1613	32.85	1	2282.71	C14 H22 N2 O3	(M-H)-
297.1599			1	251054		
298.1629			1	40299.14		
311.1755			1	49195.34		
339.2071			1	31204.71		
383.3614			1	189856.25		
384.3647			1	47085.37		

MSMS Spectrum

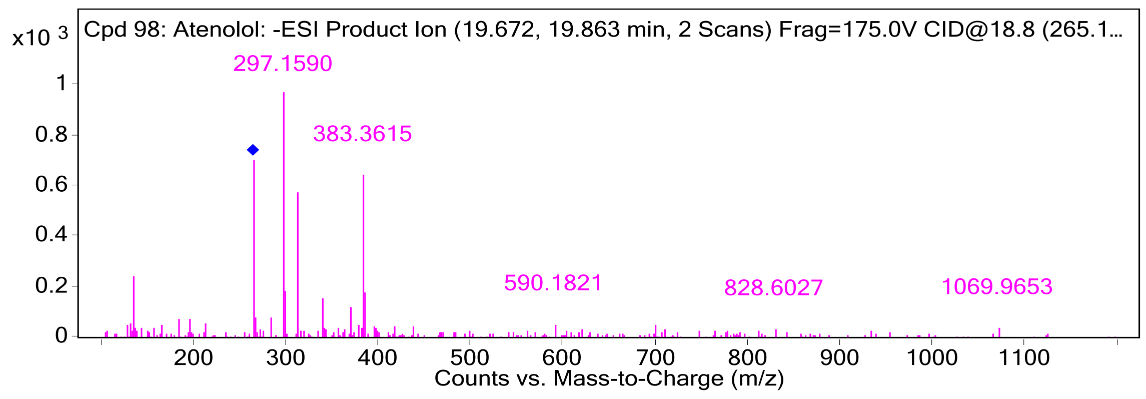

MS/MS Spectrum Peak List

m/z	z	Abund
134.8983	1	247.66
265.1535	1	707.23
297.159	1	974.98
298.1638	1	186.29
311.1754	1	578.39
312.1769	1	135.26
339.2065		159.63
369.3467		124.15
383.3615	1	647.9
384.3622	1	179.75

Compound Structure

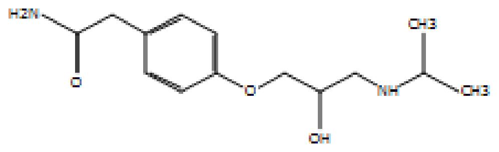

Compound Label	*m/z*	RT	Algorithm
Compound 99	383.3612	20.041	Auto MS/MS

MS Spectrum

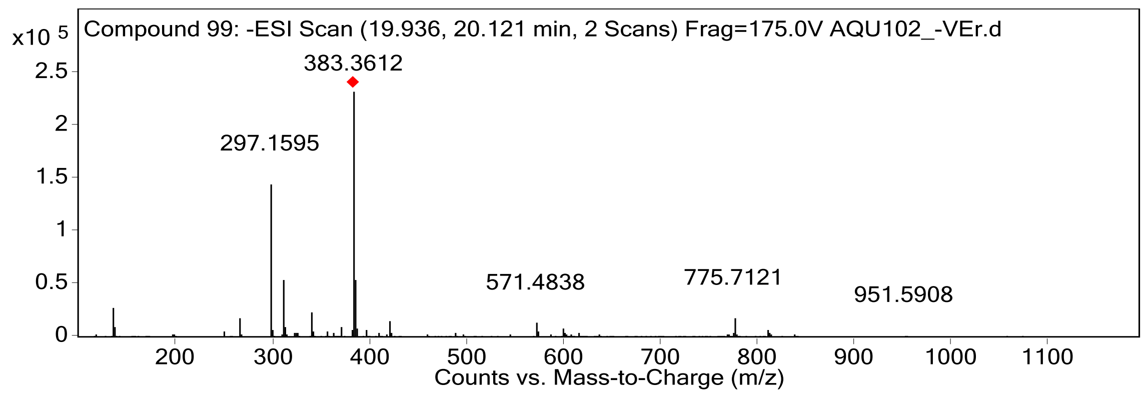

MS Zoomed Spectrum

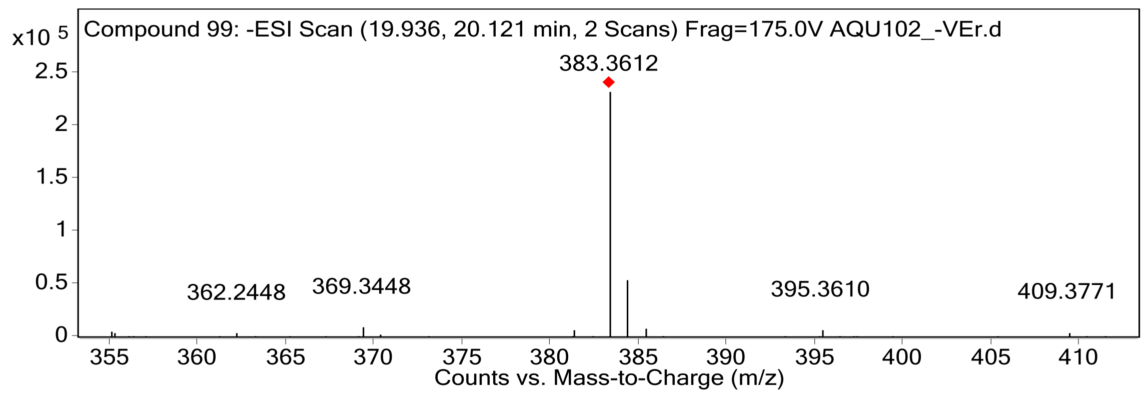

MS Spectrum Peak List

m/z	z	Abund
134.8982	1	27179
265.1537	1	18510.24
297.1595	1	144806.02
298.1627	1	25745.5
311.1754	1	54380.98
339.2069	1	23693.84
383.3612	1	231831.28
384.3643	1	54197.87
385.3674	1	8263.47
775.7121	1	17773.65

MSMS Spectrum

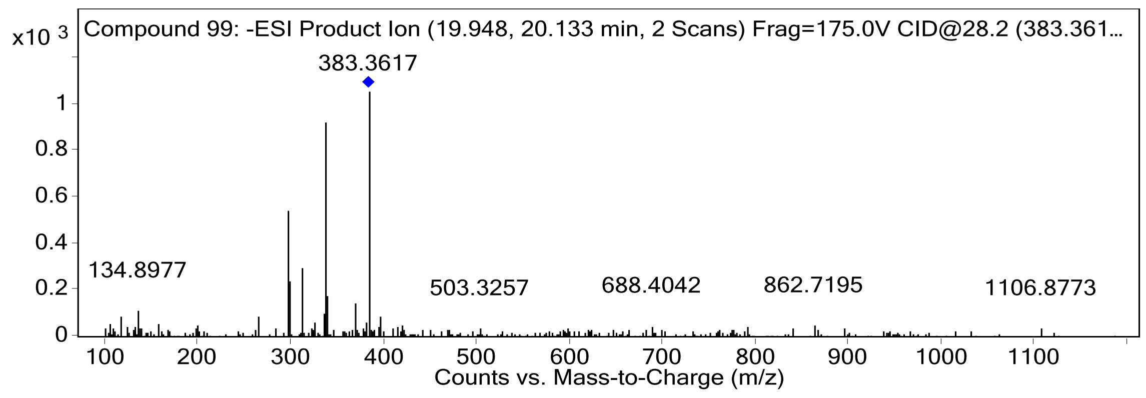

MS/MS Spectrum Peak List

m/z	z	Abund
134.8977		111.9
297.159	1	545.41
298.1617	1	239.96
311.1736	1	297.91
337.3541	1	925.7
338.3556	1	179.88
339.2067		134.58
369.3424		143.28
383.3617	1	1057.31
384.3646	1	326.08

Compound Label	*m/z*	RT	Algorithm
Compound 100	383.3609	20.426	Auto MS/MS

MS Spectrum

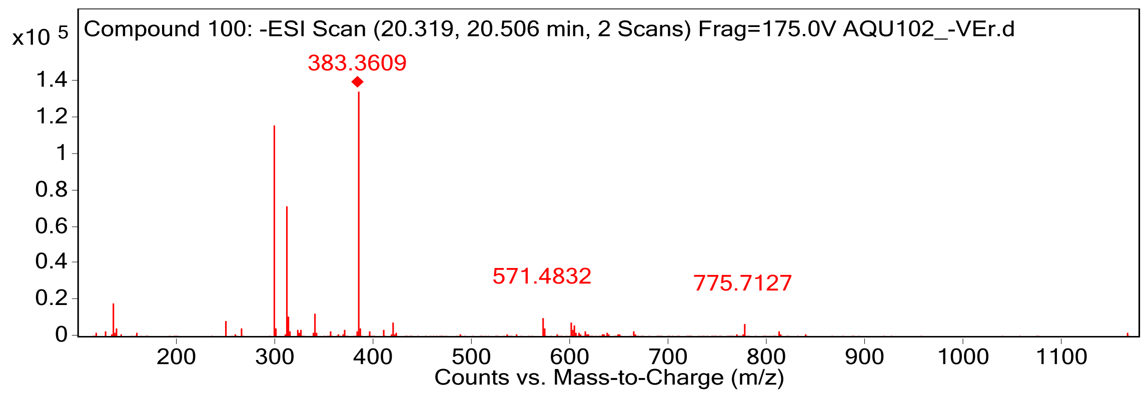

MS Zoomed Spectrum

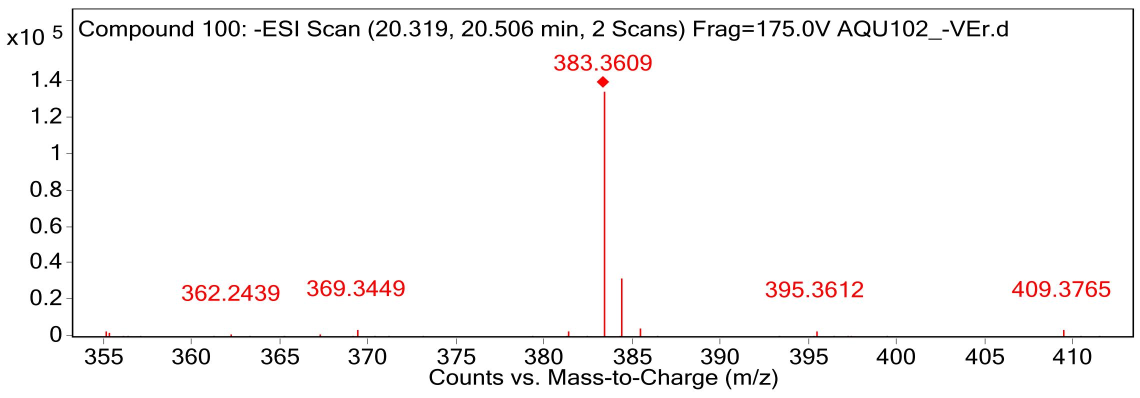

MS Spectrum Peak List

m/z	z	Abund
134.8982	1	18729.79
297.1595	1	115858.22
298.1624	1	21282.29
311.1752	1	71589.19
312.1785	1	11612
339.2071	1	13009.5
383.3609	1	134661.45
384.3643	1	31866.59
385.3669	1	4921.27
571.4832	1	10863.14

MSMS Spectrum

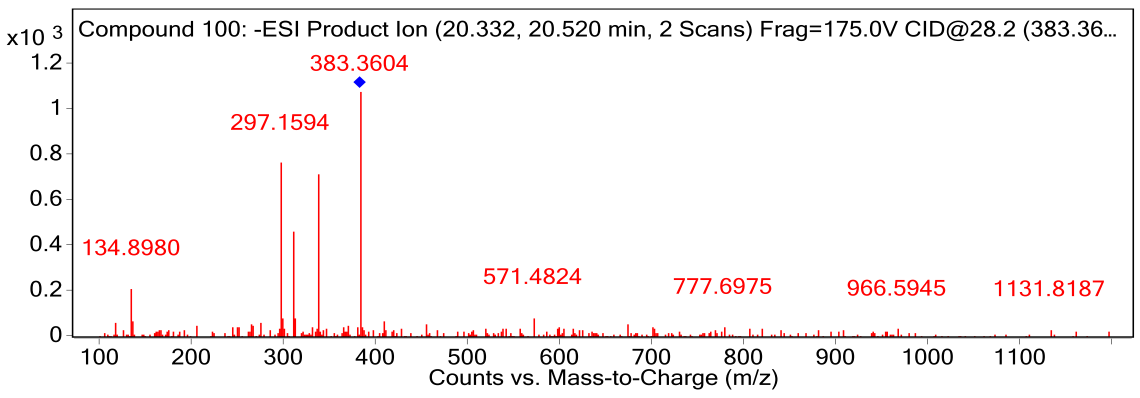

MS/MS Spectrum Peak List

m/z	z	Abund
134.898		213.11
297.1594	1	763.86
299.1543	1	81.39
311.1746	1	465.02
313.1771	1	85.34
337.355	1	714.07
338.3591	1	188.35
383.3604	1	1076.43
384.365	1	271.18
571.4824	1	86.64

**Compound Label**	***m/z***	**RT**	**Algorithm**
Compound 101	383.3605	20.821	Auto MS/MS

MS Spectrum

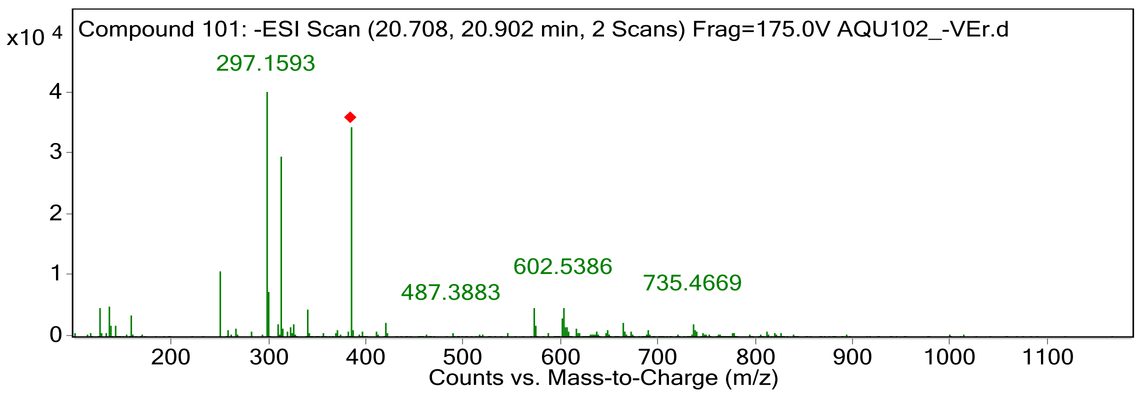

MS Zoomed Spectrum

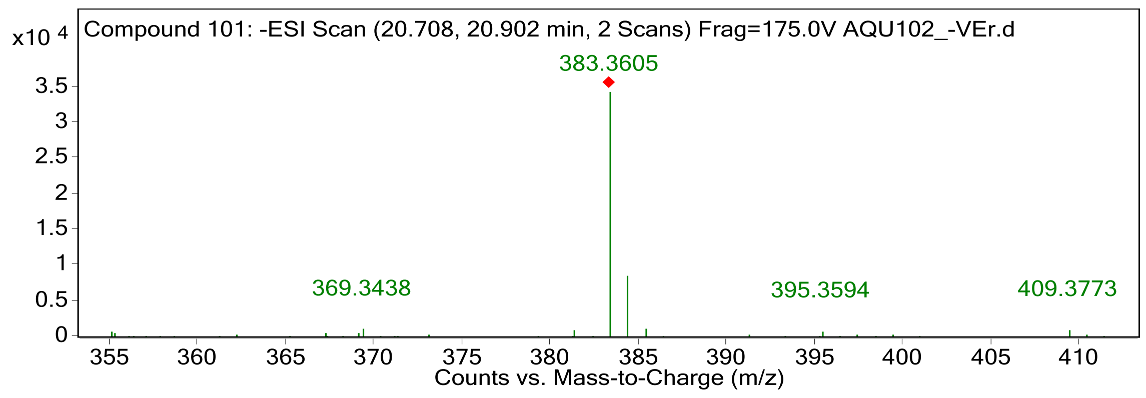

MS Spectrum Peak List

m/z	z	Abund
134.8982	1	5027.97
248.9792		10735.4
297.1593	1	40259.25
298.1625	1	7431.31
311.1751	1	29592.06
312.1784	1	5345.36
383.3605	1	34359.51
384.3643	1	8653.24
385.367	1	1267.33
602.5386	1	4873.48

MSMS Spectrum

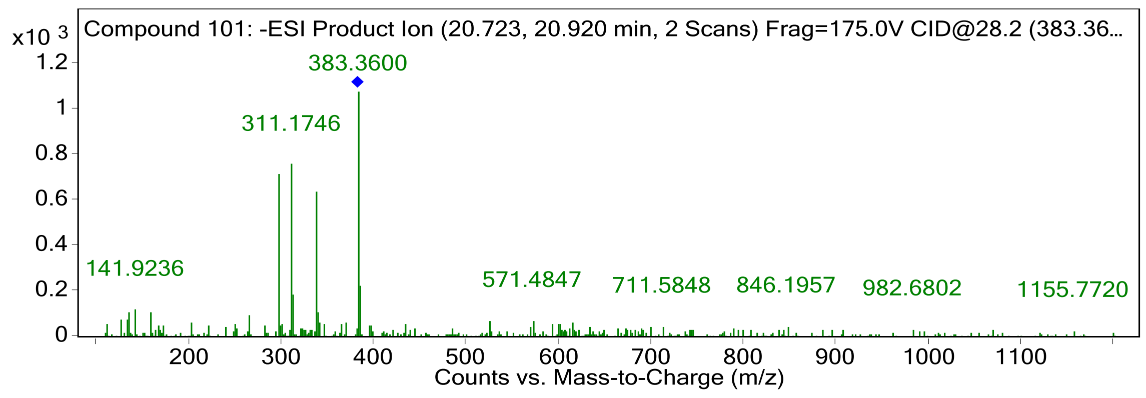

MS/MS Spectrum Peak List

m/z	z	Abund
134.8977		112.35
141.9236		121.4
157.9232		106.76
297.1597	1	718.53
311.1746	1	759.54
312.1802	1	189.41
337.3548	1	639.86
338.3587	1	108.29
383.36	1	1078.17
384.364	1	224.1

--- End Of Report ---

Qualitative Compound Report

**Data File**	AQU202.d	**Sample Name**	AQU202
**Sample Type**	Sample	**Position**	P1-B6
**Instrument Name**	QTOF	**User Name**	
**Acq Method**	metabolite_ESI_+VE_MSMS.m	**Acquired Time**	4/18/2022 8:12:28 PM
**IRM Calibration Status**	Success	**DA Method**	default.m
**Comment**			

**Sample Group**		**Info.**
**Acquisition SW Version**	6200 series TOF/6500 series Q-TOF B.05.01 (B5125.3)	

Compound Table

Compound Label	RT	Mass	Abund	Name	Formula	MFG Formula	DB Formula	DB Diff (ppm)	Hits (DB)
Cpd 1: Retronecine	1.178	155.0945	102689	Retronecine	C8 H13 N O2	C8 H13 N O2	C8 H13 N O2	0.87	3
Compound 2	1.481		15050						
Cpd 3: Retronecine	1.509	155.0934	37749	Retronecine	C8 H13 N O2	C8 H13 N O2	C8 H13 N O2	7.65	3
Cpd 4: Isobutyl N-methylanthranilate	2.329	207.1247	53401	Isobutyl N-methylanthranilate	C12 H17 N O2	C12 H17 N O2	C12 H17 N O2	5.95	8
Cpd 5: Metanephrine	2.407	197.1048	13365	Metanephrine	C10 H15 N O3	C10 H15 N O3	C10 H15 N O3	2.02	10
Cpd 6: d-Dethiobiotin	2.612	214.1311	43373	d-Dethiobiotin	C10 H18 N2 O3	C10 H18 N2 O3	C10 H18 N2 O3	2.86	10
Cpd 7: Isobutyl N-methylanthranilate	2.665	207.1248	46335	Isobutyl N-methylanthranilate	C12 H17 N O2	C12 H17 N O2	C12 H17 N O2	5.39	8
Cpd 8: d-Dethiobiotin	2.881	214.1306	28291	d-Dethiobiotin	C10 H18 N2 O3	C10 H18 N2 O3	C10 H18 N2 O3	5.55	10
Cpd 9: Trihomomethionine	3.06	191.0971	28691	Trihomomethionine	C8 H17 N O2 S	C8 H17 N O2 S	C8 H17 N O2 S	4.9	9
Cpd 10: Butyl 2-aminobenzoate	3.318	193.1093	23715	Butyl 2-aminobenzoate	C11 H15 N O2	C11 H15 N O2	C11 H15 N O2	4.98	10
Cpd 11: Salicylanilide	3.332	213.0789	20580	Salicylanilide	C13 H11 N O2	C13 H11 N O2	C13 H11 N O2	0.6	9
Cpd 12: L-phenylalanyl-L-proline	3.434	262.1304	26095	L-phenylalanyl-L-proline	C14 H18 N2 O3	C14 H18 N2 O3	C14 H18 N2 O3	5.25	10
Cpd 13: Isocarbostyril	3.538	145.0523	88791	Isocarbostyril	C9 H7 N O	C9 H7 N O	C9 H7 N O	3.4	10
Compound 14	3.68		14464						
Cpd 15: 4-Prenyldihydropinosylvin	3.733	282.1647	12208	4-Prenyldihydropinosylvin	C19 H22 O2	C19 H22 O2	C19 H22 O2	-9.51	5
Cpd 16: Fasoracetam	3.898	196.1208	237447	Fasoracetam	C10 H16 N2 O2	C10 H16 N2 O2	C10 H16 N2 O2	1.87	6
Cpd 17: Sinapoylputrescine	3.909	294.1562	48680	Sinapoylputrescine	C15 H22 N2 O4	C15 H22 N2 O4	C15 H22 N2 O4	6.08	10
Cpd 18: 2,4'-Diphenyldiamine	4.409	184.0997	15287	2,4'-Diphenyldiamine	C12 H12 N2	C12 H12 N2	C12 H12 N2	1.72	8
Cpd 19: Pirbuterol	4.454	240.1482	57896	Pirbuterol	C12 H20 N2 O3	C12 H20 N2 O3	C12 H20 N2 O3	-3.21	10
Compound 20	4.584		45608						
Cpd 21: Avocadynofuran	4.606	246.1943	17566	Avocadynofuran	C17 H26 O	C17 H26 O	C17 H26 O	16.34	4
Cpd 22: L,L-Cyclo(leucylprolyl)	4.756	210.1362		L,L-Cyclo(leucylprolyl)	C11 H18 N2 O2	C11 H18 N2 O2	C11 H18 N2 O2	3.21	3
Cpd 23: Istamycin C1	4.83	431.27	129216	Istamycin C1	C19 H37 N5 O6	C19 H37 N5 O6	C19 H37 N5 O6	10.12	1
Cpd 24: Progeldanamycin	5.12	475.296	109222	Progeldanamycin	C27 H41 N O6	C27 H41 N O6	C27 H41 N O6	-5.47	4
Cpd 25: L,L-Cyclo(leucylprolyl)	5.12	210.1362	165462	L,L-Cyclo(leucylprolyl)	C11 H18 N2 O2	C11 H18 N2 O2	C11 H18 N2 O2	3.21	3
Compound 26	5.358								
Compound 27	5.579								
Compound 28	5.727		114288						
Cpd 29: 3-Oxo-12,18-ursadien-28-oic acid	5.8	452.3331	74032	3-Oxo-12,18-ursadien-28-oic acid	C30 H44 O3	C30 H44 O3	C30 H44 O3	-8.95	10
Compound 30	5.947		88356						
Cpd 31: Mycinamicin IV	6.123	695.4262	43092	Mycinamicin IV	C37 H61 N O11	C37 H61 N O11	C37 H61 N O11	-2.51	1
Compound 32	6.598		26191						
Compound 33	6.676								
Compound 34	6.729								
Cpd 35: PE(18:2(9Z,12Z)/15:0)	6.745	700.4824	82347	PE(18:2(9Z,12Z)/15:0)	C38 H72 N O8 P	C38 H72 N O8 P	C38 H72 N O8 P	1452.05	2
Compound 36	6.777		490832						
Compound 37	7.054		41012						
Compound 38	7.174		18220						
Compound 39	7.325		42956						
Compound 40	7.332		81226						
Cpd 41: Dihydrocapsaicin	7.489	307.2126	25647	Dihydrocapsaicin	C18 H29 N O3	C18 H29 N O3	C18 H29 N O3	7.07	3
Compound 42	7.717		18048						
Cpd 43: 1-Phosphatidyl-1D-myo-inositol 3-phosphate	8.168	470.023	24518	1-Phosphatidyl-1D-myo-inositol 3-phosphate	C11 H20 O16 P2	C11 H20 O16 P2	C11 H20 O16 P2	-0.7	2
Cpd 44: Istamycin A1	8.328	417.2603	18182	Istamycin A1	C18 H35 N5 O6	C18 H35 N5 O6	C18 H35 N5 O6	-3.79	2
Cpd 45: Istamycin A1	8.599	415.2542	24201	Istamycin A1	C18 H35 N5 O6	C18 H35 N5 O6	C18 H35 N5 O6	4827.32	2
Cpd 46: Docosatetraenoyl ethanolamide	9.337	375.3089	23129	Docosatetraenoyl ethanolamide	C24 H41 N O2	C24 H41 N O2	C24 H41 N O2	12.75	1
Cpd 47: Fleroxacin	9.925	369.13	25687	Fleroxacin	C17 H18 F3 N3 O3	C17 H18 F3 N3 O3	C17 H18 F3 N3 O3	0.02	2
Cpd 48: Dibenzo[a,e]pyrene	10.115	302.1087	17146	Dibenzo[a,e]pyrene	C24 H14	C24 H14	C24 H14	2.69	10
Cpd 49: Rhipocephalin	10.355	376.1941	102038	Rhipocephalin	C21 H28 O6	C21 H28 O6	C21 H28 O6	-14.54	7
Cpd 50: Enalapril	10.698	376.1949	44189	Enalapril	C20 H28 N2 O5	C20 H28 N2 O5	C20 H28 N2 O5	13.04	7
Cpd 51: Carpaine	11.714	478.3737	27858	Carpaine	C28 H50 N2 O4	C28 H50 N2 O4	C28 H50 N2 O4	6.92	3
Cpd 52: 16-Oxo-palmitate	12.063	270.22	36124	16-Oxo-palmitate	C16 H30 O3	C16 H30 O3	C16 H30 O3	-1.7	10
Cpd 53: 3b,17a,21-Trihydroxypregnenone	12.167	348.2299		3b,17a,21-Trihydroxypregnenone	C21 H32 O4	C21 H32 O4	C21 H32 O4	0.32	10
Cpd 54: 5-Hydroxy-4-methoxy-5-(1-oxo-9,12,15-hexadecatrienyl)-2(5H)-furanone	12.218	362.2045		5-Hydroxy-4-methoxy-5-(1-oxo-9,12,15-hexadecatrienyl)-2(5H)-furanone	C21 H30 O5	C21 H30 O5	C21 H30 O5	13.24	10
Cpd 55: 6-Oxabicyclo[3.1.0]hexane-2-undecanoic acid methyl ester	12.223	282.2202	169020	6-Oxabicyclo[3.1.0]hexane-2-undecanoic acid methyl ester	C17 H30 O3	C17 H30 O3	C17 H30 O3	-2.41	10
Cpd 56: 12-(2,3-Dihydroxycyclopentyl)-2-dodecanone	12.522	284.2362	89967	12-(2,3-Dihydroxycyclopentyl)-2-dodecanone	C17 H32 O3	C17 H32 O3	C17 H32 O3	-3.56	10
Cpd 57: 6-Oxabicyclo[3.1.0]hexane-2-undecanoic acid methyl ester	12.535	282.2203	38228	6-Oxabicyclo[3.1.0]hexane-2-undecanoic acid methyl ester	C17 H30 O3	C17 H30 O3	C17 H30 O3	-3.03	10
Cpd 58: Ximenoylacetone	12.698	434.408		Ximenoylacetone	C29 H54 O2	C29 H54 O2	C29 H54 O2	10.03	2
Cpd 59: Ximenoylacetone	13.006	434.408		Ximenoylacetone	C29 H54 O2	C29 H54 O2	C29 H54 O2	9.98	2

Cpd 60: 5-Pentacosyl-1,3-benzenediol	13.077	460.4229		5-Pentacosyl-1,3-benzenediol	C31 H56 O2	C31 H56 O2	C31 H56 O2	11.1	2
Cpd 61: Ximenoylacetone	13.332	434.4079	81910	Ximenoylacetone	C29 H54 O2	C29 H54 O2	C29 H54 O2	10.42	2
Cpd 62: Stigmastanol	13.403	416.3973	53957	Stigmastanol	C29 H52 O	C29 H52 O	C29 H52 O	10.73	2
Cpd 63: 5-Pentacosyl-1,3-benzenediol	13.405	460.4233	20996	5-Pentacosyl-1,3-benzenediol	C31 H56 O2	C31 H56 O2	C31 H56 O2	10.38	2
Cpd 64: Stigmastanol	13.747	416.3976		Stigmastanol	C29 H52 O	C29 H52 O	C29 H52 O	10.08	2
Cpd 65: (Z)-22-Hentriacontene-2,4-dione	14.043	462.4391		(Z)-22-Hentriacontene-2,4-dione	C31 H58 O2	C31 H58 O2	C31 H58 O2	9.82	1
Cpd 66: Stigmastanol	14.092	416.3972	56120	Stigmastanol	C29 H52 O	C29 H52 O	C29 H52 O	11.02	2
Cpd 67: Hydroflumethiazide	14.31	330.9939		Hydroflumethiazide	C8 H8 F3 N3 O4 S2	C8 H8 F3 N3 O4 S2	C8 H8 F3 N3 O4 S2	-9.23	1
Cpd 68: (Z)-22-Hentriacontene-2,4-dione	14.38	462.439		(Z)-22-Hentriacontene-2,4-dione	C31 H58 O2	C31 H58 O2	C31 H58 O2	10.1	1
Cpd 69: Methyl 2-furoate	14.382	126.0339		Methyl 2-furoate	C6 H6 O3	C6 H6 O3	C6 H6 O3	-17.8	9
Compound 70	14.984		50987						
Cpd 71: Chlorfenvinphos	15.072	357.9707	76772	Chlorfenvinphos	C12 H14 Cl3 O4 P	C12 H14 Cl3 O4 P	C12 H14 Cl3 O4 P	-3.3	1
Compound 72	15.124		21285						
Cpd 73: Idoxuridine	15.318	353.9704	124532	Idoxuridine	C9 H11 I N2 O5	C9 H11 I N2 O5	C9 H11 I N2 O5	2.36	1
Cpd 74: Lumequoylacetone	15.543	490.4699	27718	Lumequoylacetone	C33 H62 O2	C33 H62 O2	C33 H62 O2	10.26	4
Cpd 75: Lumequoylacetone	15.902	490.4699	42328	Lumequoylacetone	C33 H62 O2	C33 H62 O2	C33 H62 O2	10.35	4
Cpd 76: TG(22:5(7Z,10Z,13Z,16Z,19Z)/18:3(9Z,12Z,15Z)/22:5(7Z,10Z,13Z,16Z,19Z))[iso3]	17.914	976.758		TG(22:5(7Z,10Z,13Z,16Z,19Z)/18:3(9Z,12Z,15Z)/22:5(7Z,10Z,13Z,16Z,19Z))[iso3]	C65 H100 O6	C65 H100 O6	C65 H100 O6	-6.16	2
Compound 77	17.987								
Compound 78	17.987								
Compound 79	18.135								
Compound 80	18.19								

Compound Label	Name	m/z	RT	Algorithm	Mass
Cpd 1: Retronecine	**Retronecine**	156.1018	1.178	Auto MS/MS	155.0945

MS Spectrum

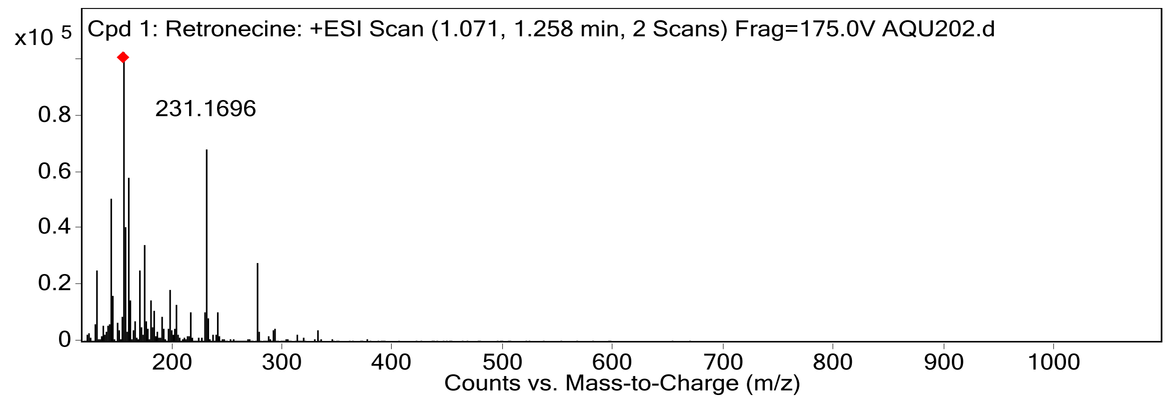

MS Zoomed Spectrum

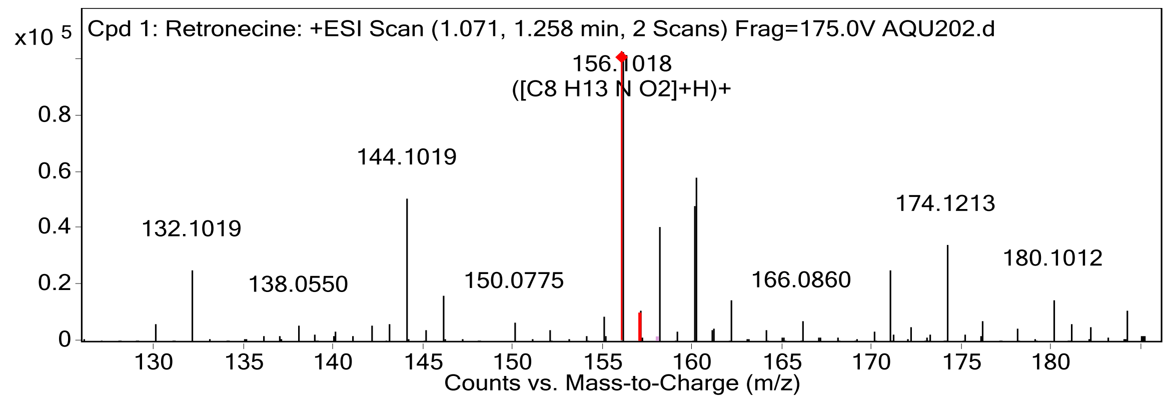

MS Spectrum Peak List

m/z	*Calc m/z*	Diff(ppm)	z	Abund	Formula	Ion
144.1019			1	50911.04		
156.1018	156.1019	0.56	1	102689.3	C8 H13 N O2	(M+H)+
157.1045	157.1051	3.87	1	11150.51	C8 H13 N O2	(M+H)+
158.1173			1	40612.35		
160.0972			1	48095.75		
160.1328			1	58198.52		
170.9695				25632.5		
174.1213			1	34405.2		
231.1696			1	68142.07		
277.0883			1	28318.89		

MSMS Spectrum

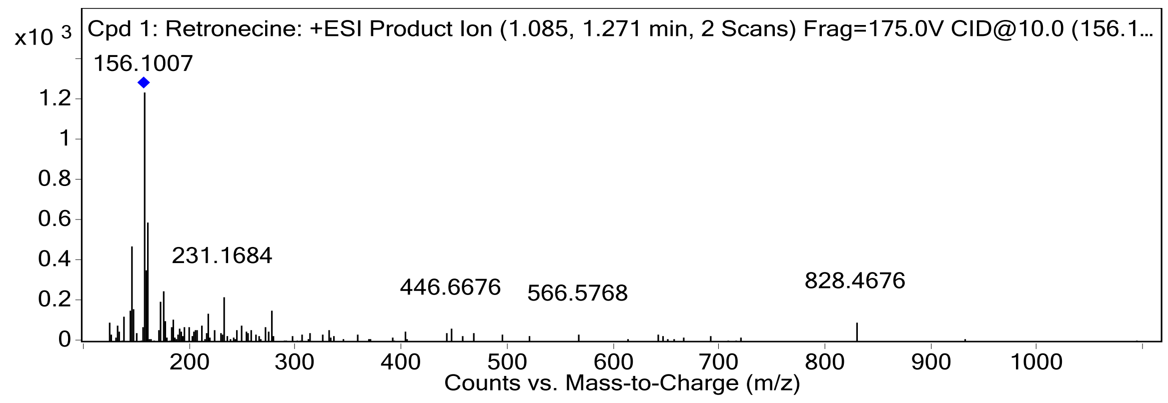

MS/MS Spectrum Peak List

m/z	z	Abund
144.1009	1	472.9
146.1168		163.51
156.1007	1	1236.03
158.1168		352.99
160.097		589.07
160.1328		465.06
172.1074		196.25
174.1232		250.36
231.1684		221.35
277.086	1	157.24

Compound Structure

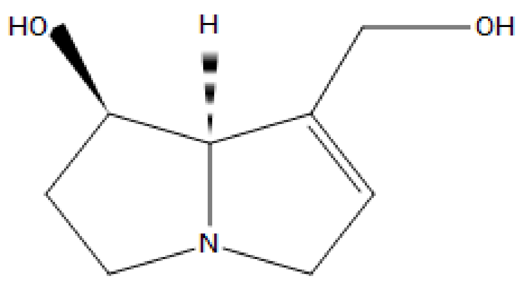

Compound Label	*m/z*	RT	Algorithm
Compound 2	174.1357	1.481	Auto MS/MS

MS Spectrum

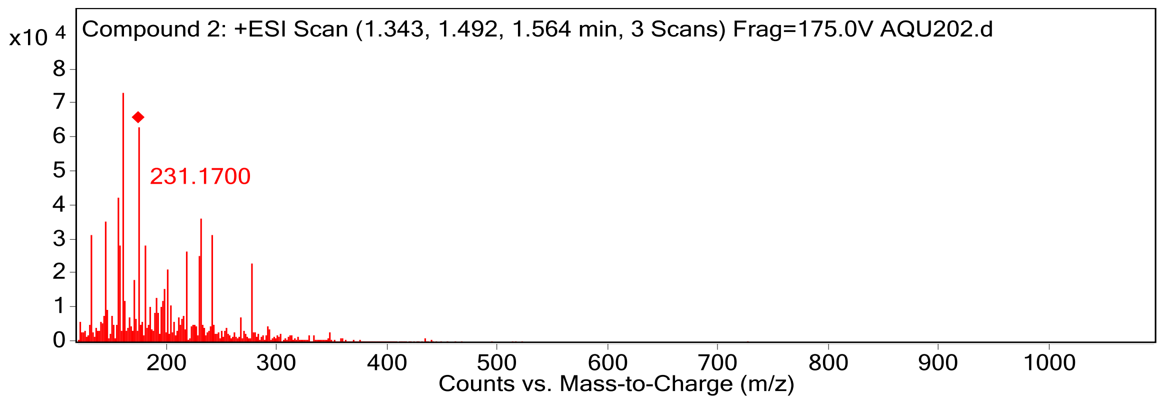

MS Zoomed Spectrum

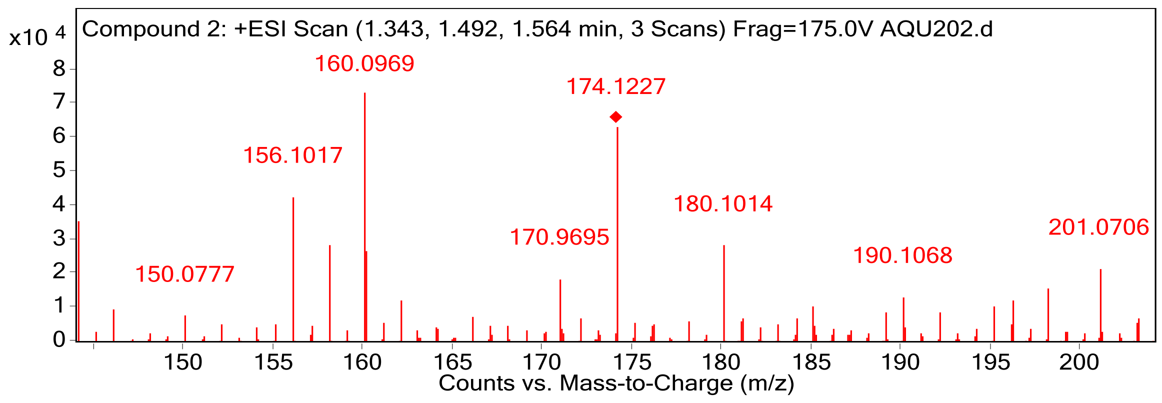

MS Spectrum Peak List

m/z	z	Abund
132.1019		31431.33
144.1017		35542.54
156.1017	1	42704.99
158.1181		28283.13
160.0969	1	73303.06
174.1227	1	62975.14
180.1014	1	28417.33
217.1541	1	26883.87
231.17	1	36590.18
241.1538	1	31603.67

MSMS Spectrum

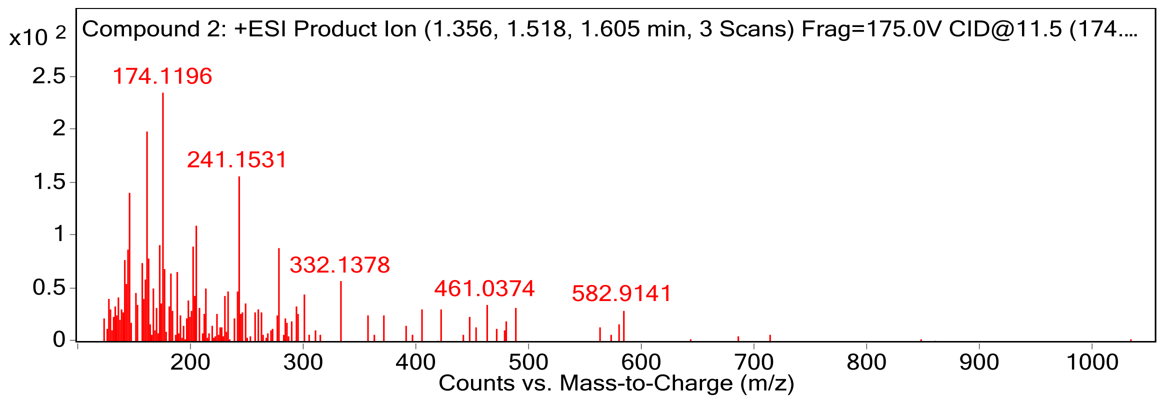

MS/MS Spectrum Peak List

m/z	z	Abund
144.0649		121.06
144.102		140.37
160.0949		198.24
170.9679		91.3
174.1196	1	235.7
174.148		203.84
201.0692		90.06
204.1211		110.34
241.1531	1	156.66
277.0932		88.21

Compound Label	Name	*m/z*	RT	Algorithm	Mass
Cpd 3: Retronecine	**Retronecine**	156.1018	1.509	Auto MS/MS	155.0934

MS Spectrum

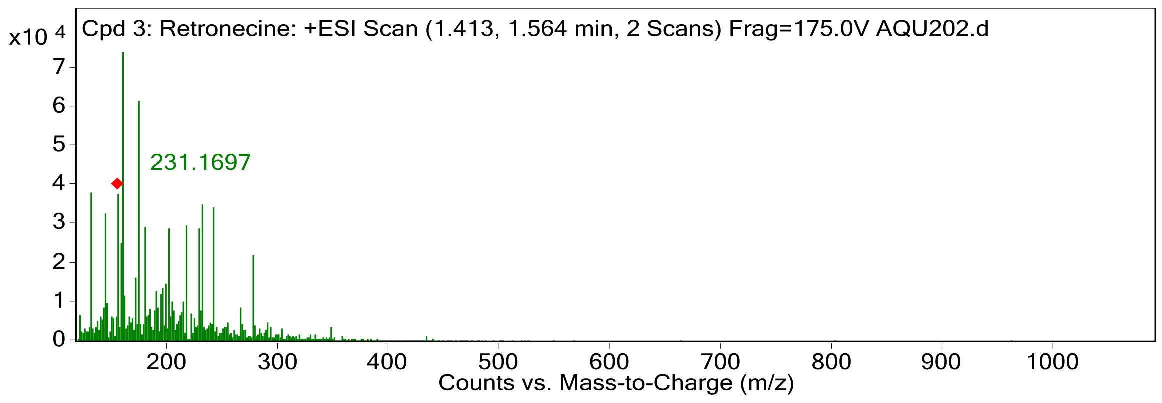

MS Zoomed Spectrum

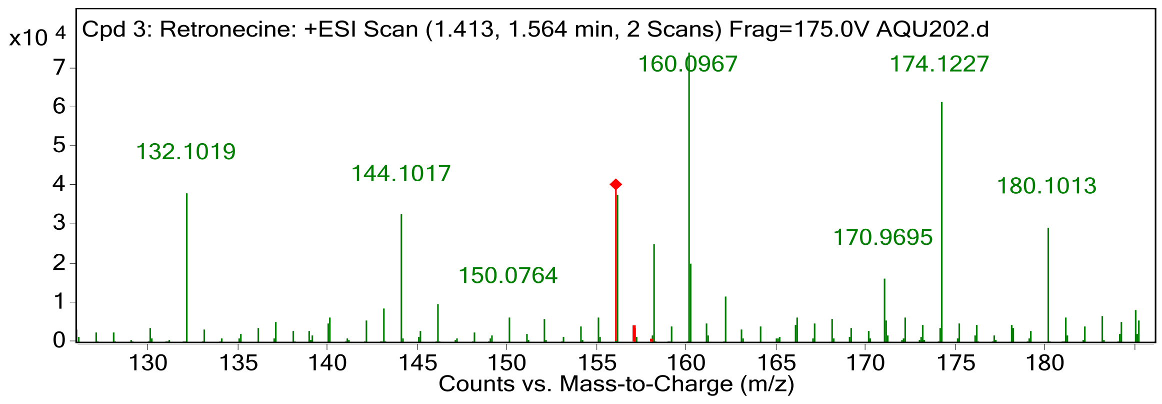

MS Spectrum Peak List

m/z	*Calc m/z*	Diff(ppm)	z	Abund	Formula	Ion
132.1019				38257.16		
144.1017				32679.06		
156.1018	156.1019	0.51		37749.25	C8 H13 N O2	(M+H)+
157.1016	157.1051	22.33	1	3726.04	C8 H13 N O2	(M+H)+
158.0888	158.1071	116.2	1	1921.19	C8 H13 N O2	(M+H)+
160.0967			1	74135.8		
174.1227			1	61681.62		
217.1542			1	29712.44		
231.1697			1	35333.6		
241.1539			1	34222.96		

MSMS Spectrum

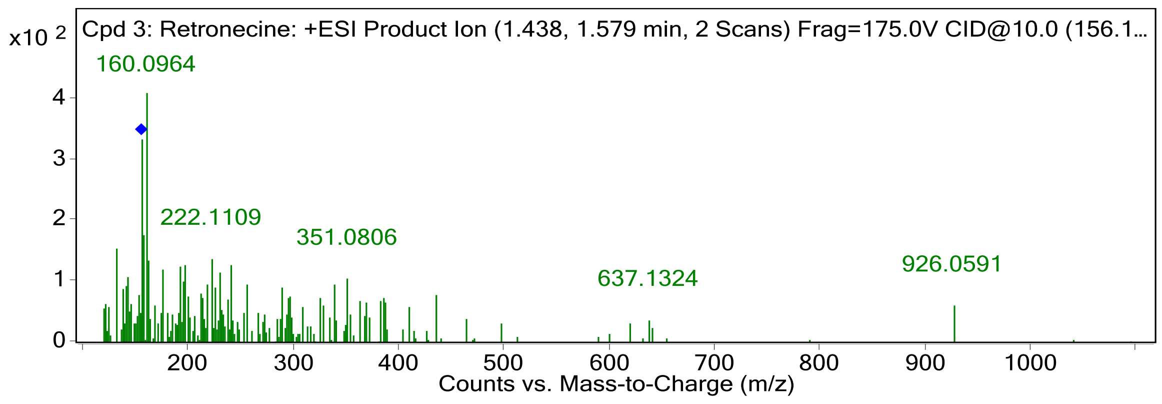

MS/MS Spectrum Peak List

m/z	*Calc m/z*	Diff (ppm)	z	Abund	Formula	Ion
132.1013				153.38		
156.1019	156.1019	0.2	1	333.89	C8 H13 N O2	(M+H)+
158.1169				177.58		
160.0964				410.43		
160.1328				149.13		
162.1119				134.28		
192.0988				125.76		
196.0969				128.44		
222.1109				137.69		
241.154				127.16		

Compound Structure

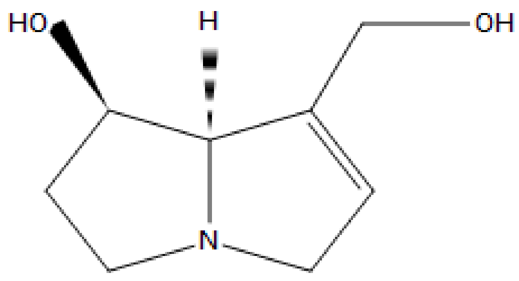

Compound Label	Name	*m/z*	RT	Algorithm	Mass
Cpd 4: Isobutyl N-methylanthranilate	**Isobutyl N-methylanthranilate**	208.1326	2.329	Auto MS/MS	207.1247

MS Spectrum

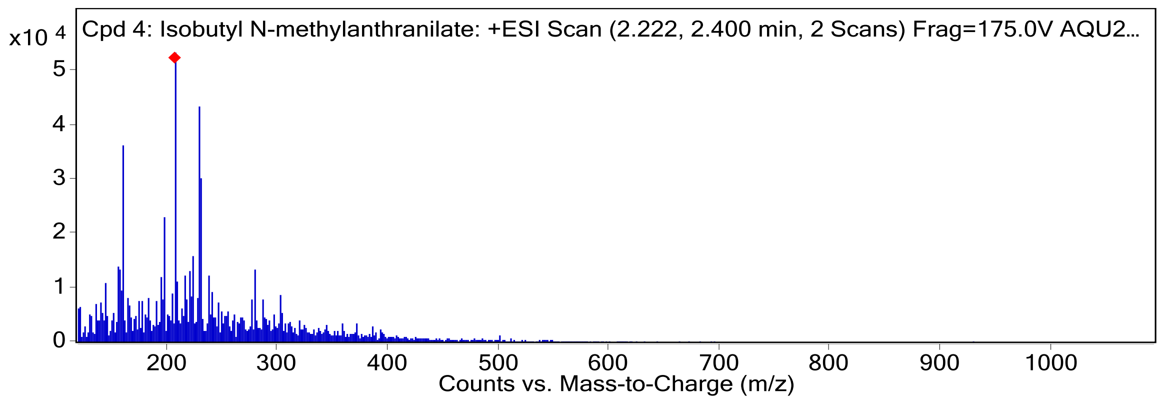

MS Zoomed Spectrum

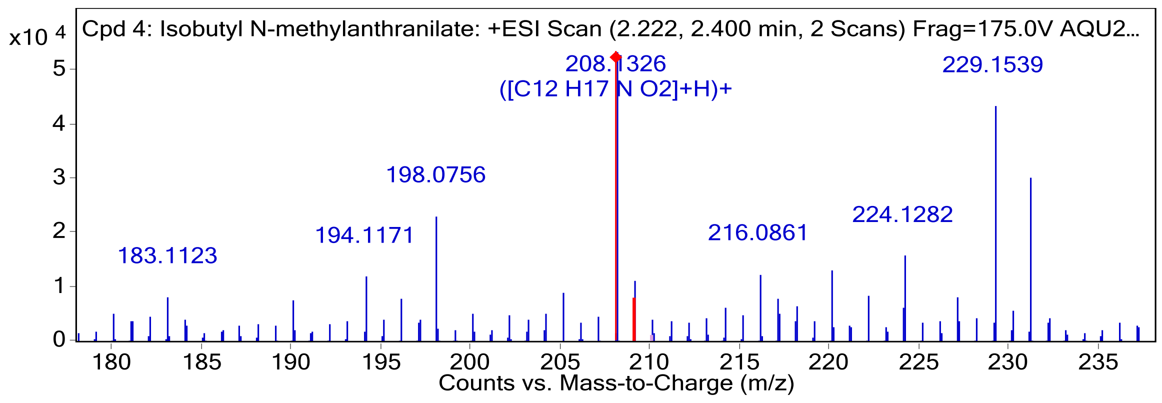

MS Spectrum Peak List

m/z	*Calc m/z*	Diff(ppm)	z	Abund	Formula	Ion
156.1017				13977.21		
160.0967				36410.27		
160.1327				15819.51		
198.0756				23192.6		
208.1326	208.1332	2.99	1	53401.3	C12 H17 N O2	(M+H)+
209.1323	209.1364	19.77	1	11242.26	C12 H17 N O2	(M+H)+
224.1282				15841.13		
229.1539			1	43535.95		
231.1694			1	30302.98		
279.1335			1	13413.9		

MSMS Spectrum

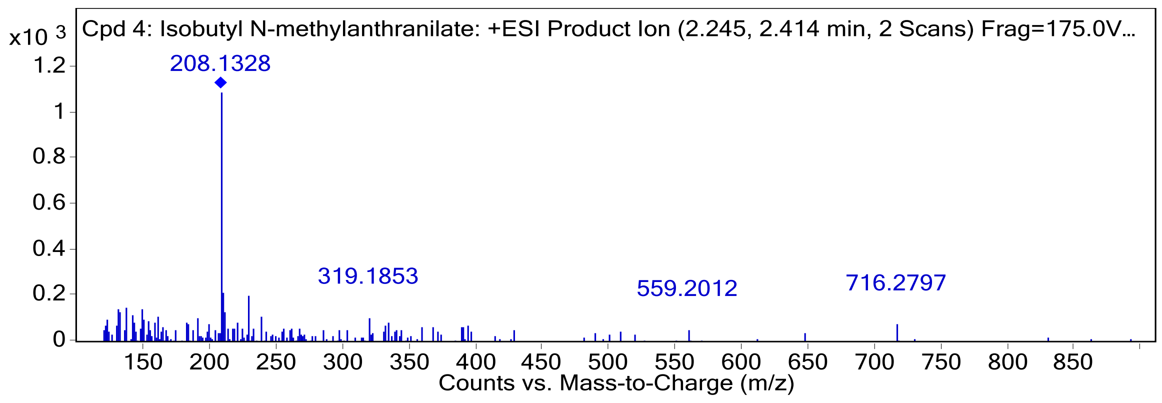

MS/MS Spectrum Peak List

m/z	z	Abund
131.0481		144.09
132.0537		127.65
137.0469		147.96
141.0772		114.52
149.061		146.57
208.1328	1	1090.67
209.1309	1	213.02
210.1109		128.68
229.1527		201.94
238.0694		110.85

Compound Structure

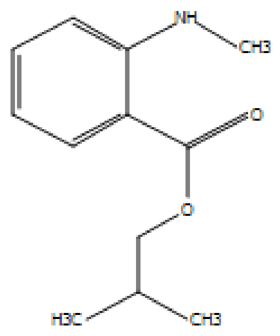

Compound Label	Name	*m/z*	RT	Algorithm	Mass
Cpd 5: Metanephrine	**Metanephrine**	220.0965	2.407	Auto MS/MS	197.1048

MS Spectrum

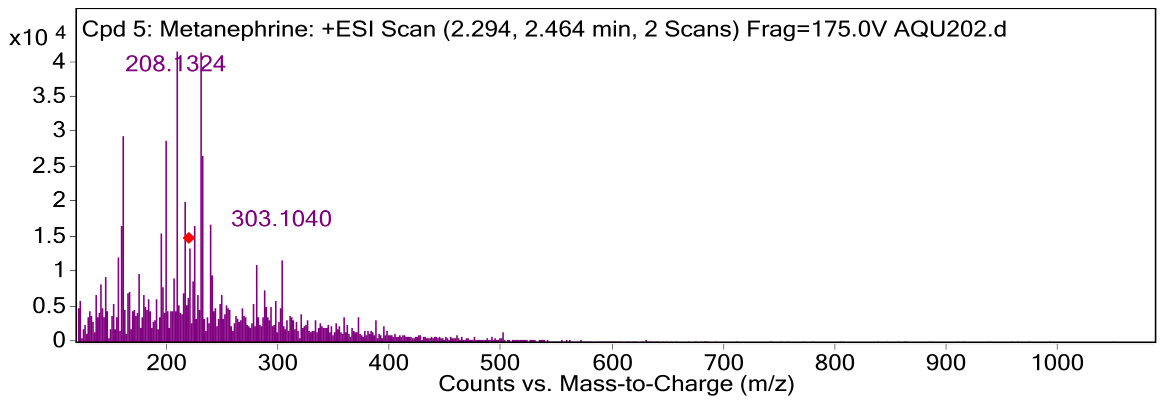

MS Zoomed Spectrum

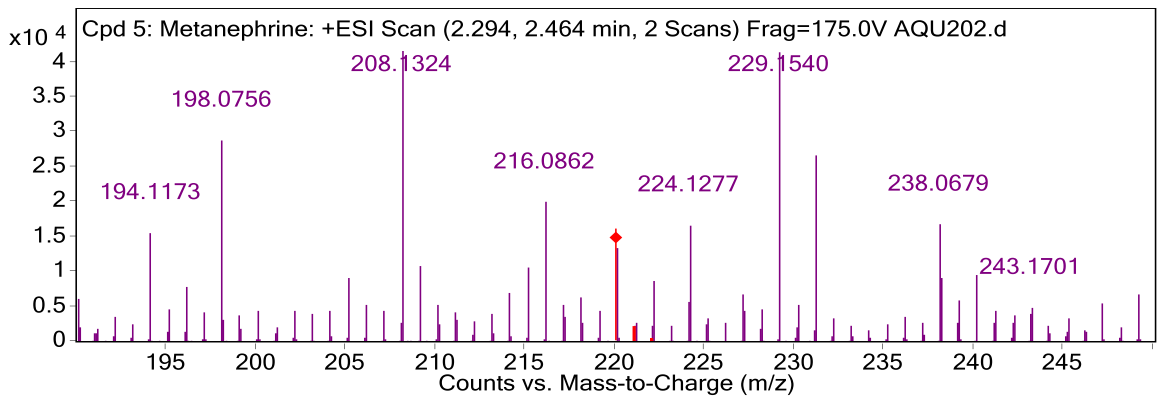

MS Spectrum Peak List

m/z	*Calc m/z*	Diff(ppm)	z	Abund	Formula	Ion
160.0966				29535.06		
198.0756			1	28855.93		

208.1324			1	66815.88		
216.0862				19990.66		
220.0965	220.0944	-9.4		13365	C10 H15 N O3	(M+Na)+
221.0983	221.0976	-2.85	1	2378.22	C10 H15 N O3	(M+Na)+
222.084	222.0997	70.67	1	2314.08	C10 H15 N O3	(M+Na)+
229.154			1	41423.39		
231.1694				26686.07		
238.0679				16806.3		

MSMS Spectrum

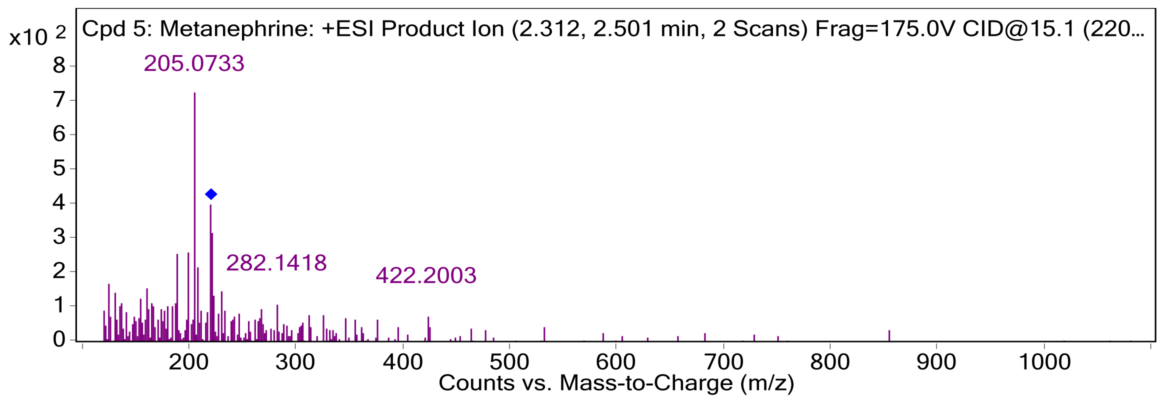

MS/MS Spectrum Peak List

m/z	z	Abund
124.0767		168.11
188.0695		256.03
198.0773		260.41
204.0877		259.4
205.0733		725.25
208.1322	1	215.17
219.1121	1	400.59
220.0976		315.38
220.1346		287.02
221.1274		213.17

Compound Structure

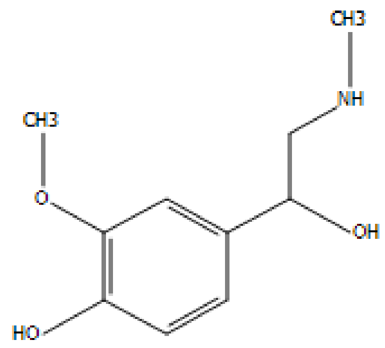

Compound Label	Name	*m/z*	RT	Algorithm	Mass
Cpd 6: d-Dethiobiotin	**d-Dethiobiotin**	215.1383	2.612	Auto MS/MS	214.1311

MS Spectrum

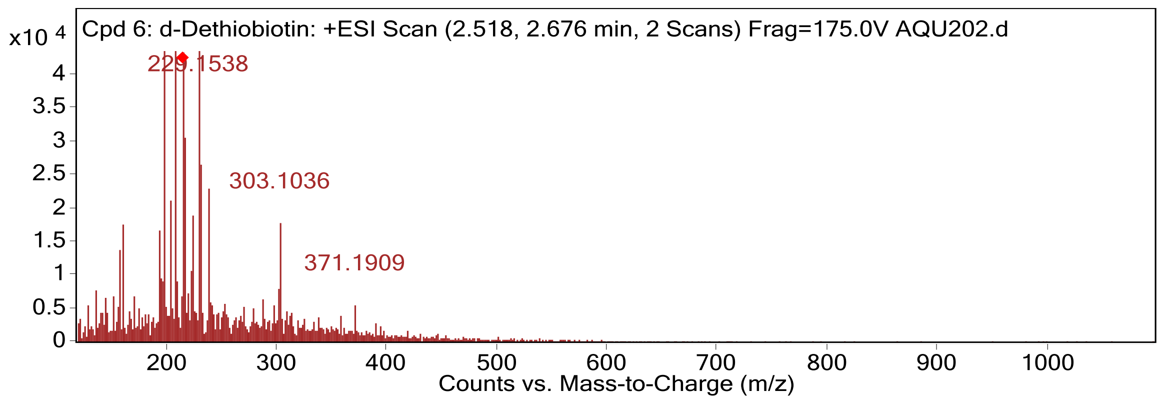

MS Zoomed Spectrum

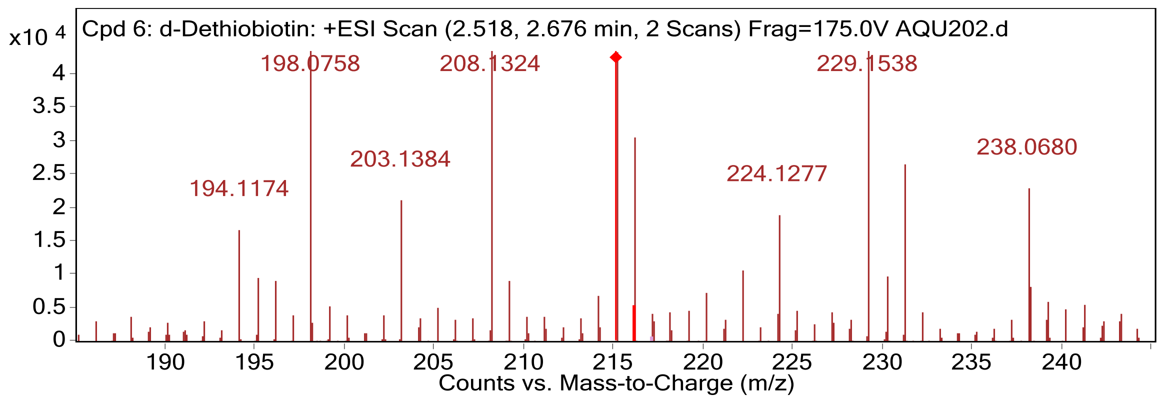

MS Spectrum Peak List

m/z	*Calc m/z*	Diff(ppm)	z	Abund	Formula	Ion
198.0758			1	50845.38		
203.1384				21201.31		
208.1324			1	60004.73		
215.1383	215.139	3.19	1	43373.1	C10 H18 N2 O3	(M+H)+
216.0858			1	30599.37		
216.142	216.142	0.43	1	5932.35	C10 H18 N2 O3	(M+H)+
224.1277			1	19013.37		
229.1538			1	64433.38		
231.1697			1	26698.69		
238.068				23013.93		

MSMS Spectrum

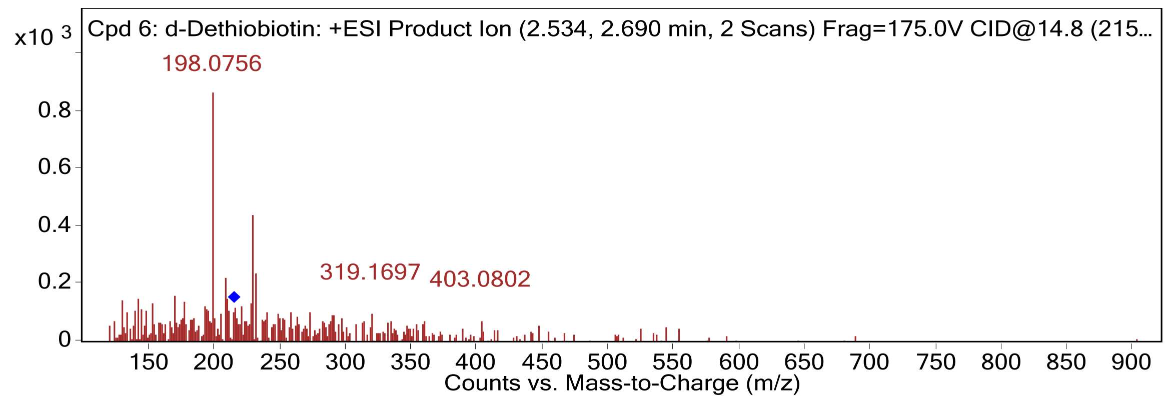

MS/MS Spectrum Peak List

m/z	Abund
130.0874	144.48
142.0937	152.38
153.1257	134.83
170.0827	158.43
177.0708	140.01
198.0756	867.68
208.132	221.8
209.1257	148
229.1551	439.28
231.1695	240.83

Compound Structure

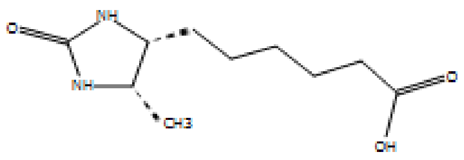

Compound Label	Name	*m/z*	RT	Algorithm	Mass
Cpd 7: Isobutyl N-methylanthranilate	**Isobutyl N-methylanthranilate**	208.1325	2.665	Auto MS/MS	207.1248

MS Spectrum

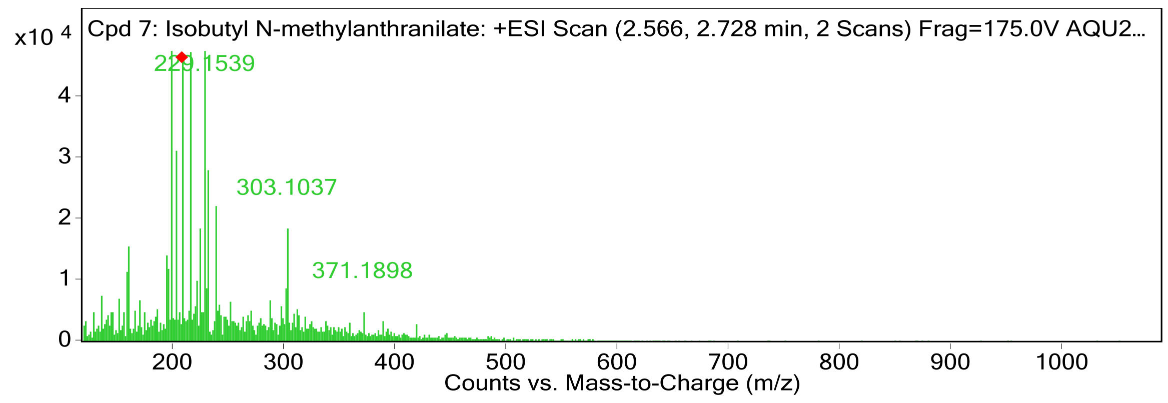

MS Zoomed Spectrum

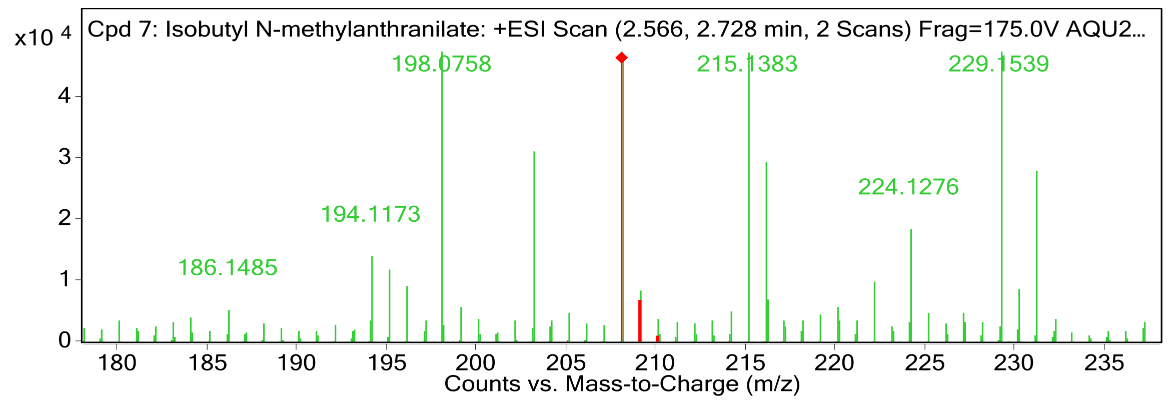

MS Spectrum Peak List

m/z	*Calc m/z*	Diff(ppm)	z	Abund	Formula	Ion
198.0758			1	50203.66		
203.1383			1	31254.28		
208.1325	208.1332	3.49	1	46334.51	C12 H17 N O2	(M+H)+
209.1334	209.1364	14.7	1	8468.71	C12 H17 N O2	(M+H)+
210.1362	210.1389	13.11	1	860.53	C12 H17 N O2	(M+H)+
215.1383			1	47191.09		
216.086			1	29421.22		
229.1539			1	70622.63		
231.1698			1	28072.51		
238.0679				22209.65		

MSMS Spectrum

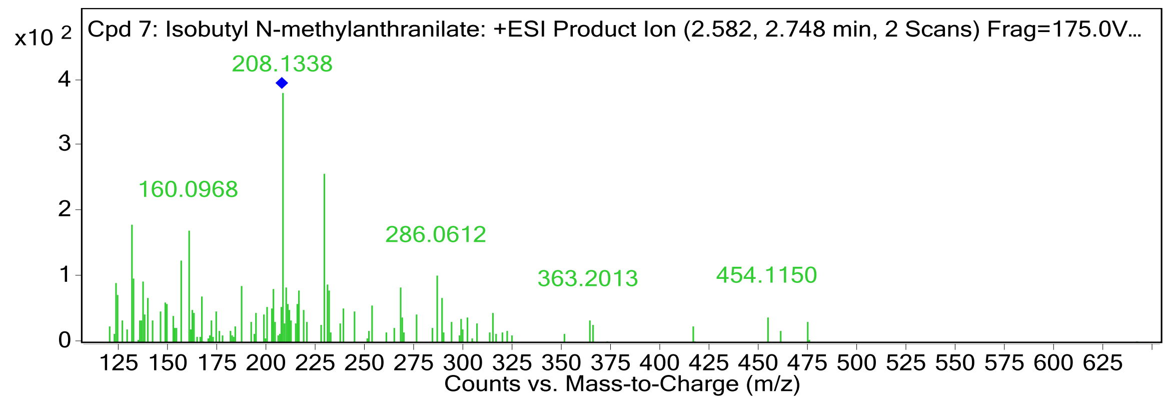

MS/MS Spectrum Peak List

m/z	z	Abund
123.0915		90.87

131.048		179.72
132.1009		98.06
137.0697		93.56
156.0104		126.21
160.0968		170.62
208.1338	1	381.6
229.1553		258.69
230.0951		89.29
286.0612		101.57

Compound Structure

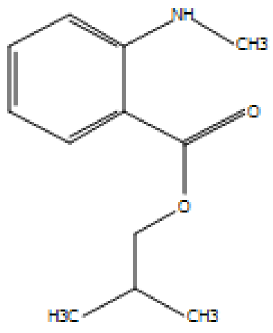

Compound Label	Name	m/z	RT	Algorithm	Mass
Cpd 8: d-Dethiobiotin	**d-Dethiobiotin**	215.1384	2.881	Auto MS/MS	214.1306

MS Spectrum

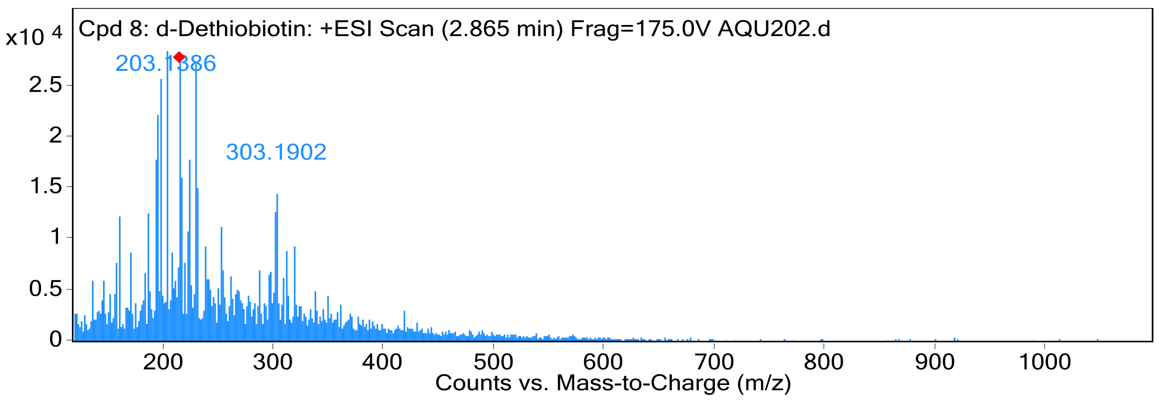

MS Zoomed Spectrum

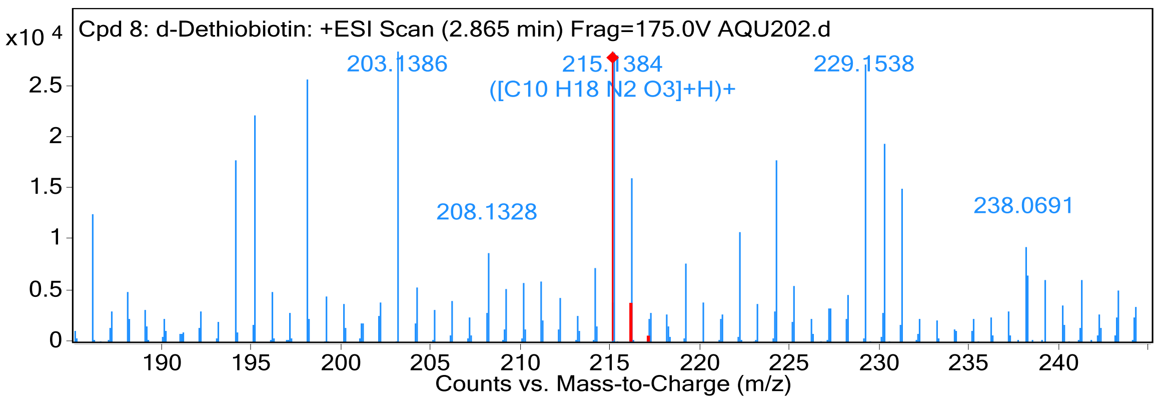

MS Spectrum Peak List

m/z	*Calc m/z*	Diff(ppm)	z	Abund	Formula	Ion
194.1173				17846.33		
195.1126				22185.09		
198.0756			1	25641.37		
203.1386			1	44056.22		
215.1384	215.139	2.76	1	28291.34	C10 H18 N2 O3	(M+H)+
216.1409	216.142	5.34	1	5642.73	C10 H18 N2 O3	(M+H)+
217.1296	217.1442	67.22	1	1278.14	C10 H18 N2 O3	(M+H)+
224.1278			1	17854.81		
229.1538			1	27126.33		
230.1738				19420.53		

MSMS Spectrum

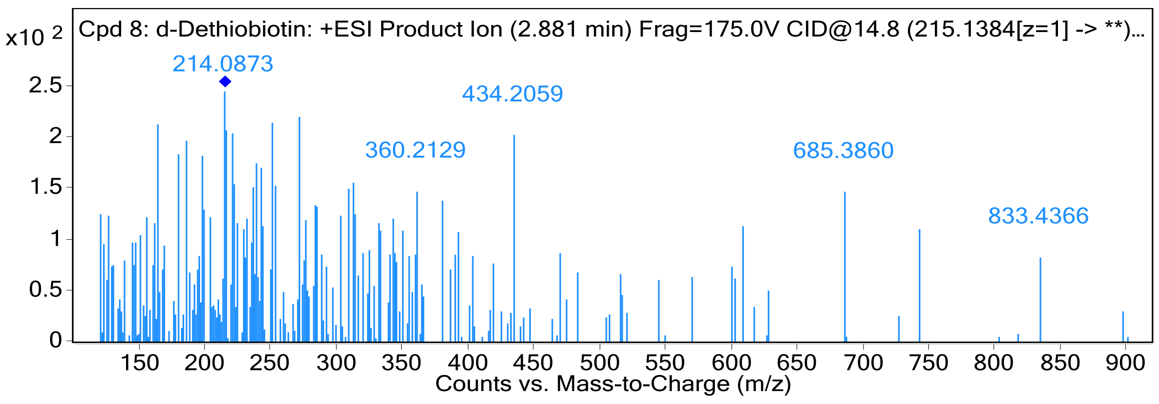

MS/MS Spectrum Peak List

m/z	Abund
164.0685	214.02
179.0823	183.53
186.146	197.01
198.0751	182.5
214.0873	245.13
215.1425	208.02
221.0898	204.4
250.1483	214.8
271.1161	221.02
434.2059	203

Compound Structure

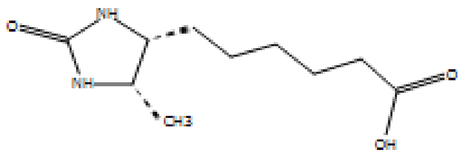

Compound Label	Name	*m/z*	RT	Algorithm	Mass
Cpd 9: Trihomomethionine	**Trihomomethionine**	214.0855	3.06	Auto MS/MS	191.0971

MS Spectrum

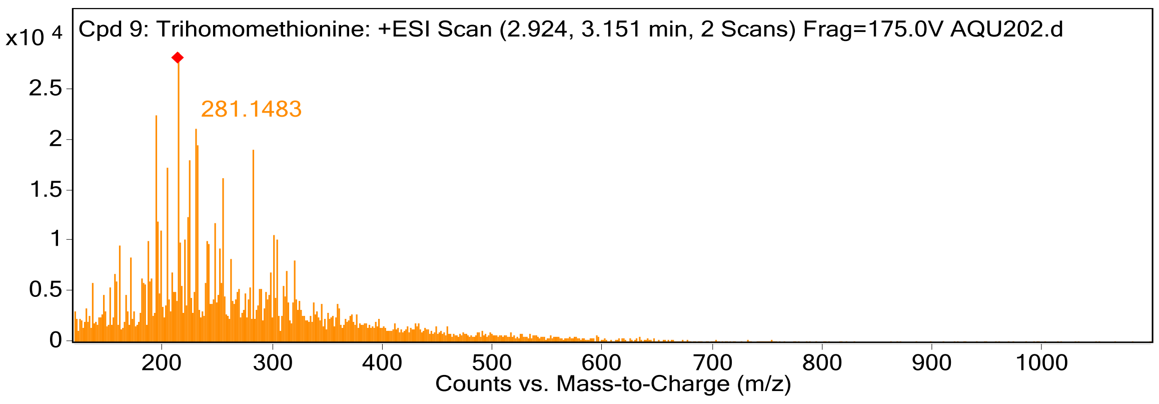

MS Zoomed Spectrum

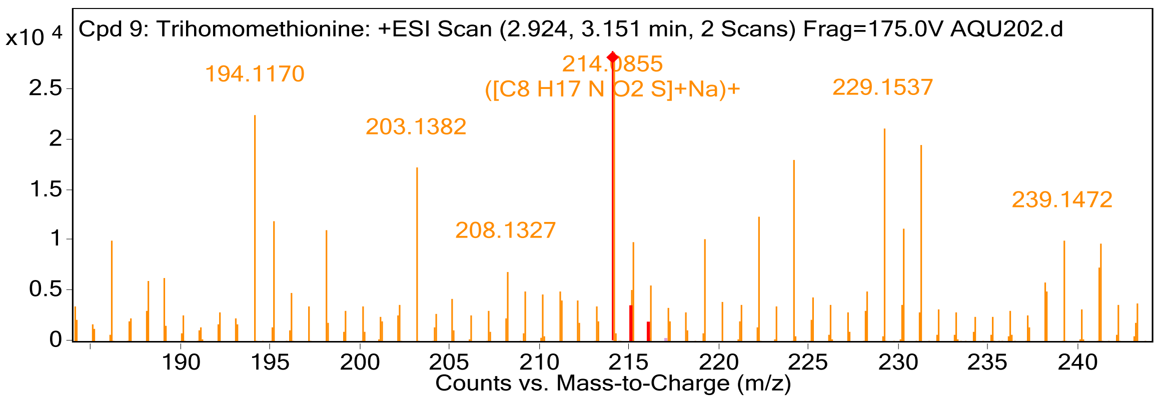

MS Spectrum Peak List

m/z	*Calc m/z*	Diff(ppm)	z	Abund	Formula	Ion
194.117				22491.68		
203.1382				17244.28		
214.0855	214.0872	8.05	1	28691.36	C8 H17 N O2 S	(M+Na)+
215.0895	215.0901	2.92	1	5248.51	C8 H17 N O2 S	(M+Na)+
216.0873	216.0845	-12.92	1	5629.54	C8 H17 N O2 S	(M+Na)+
224.1274			1	18086.4		
229.1537				21167.13		
231.1695				19551.01		
254.1022			1	16333.91		
281.1483			1	19019.56		

MSMS Spectrum

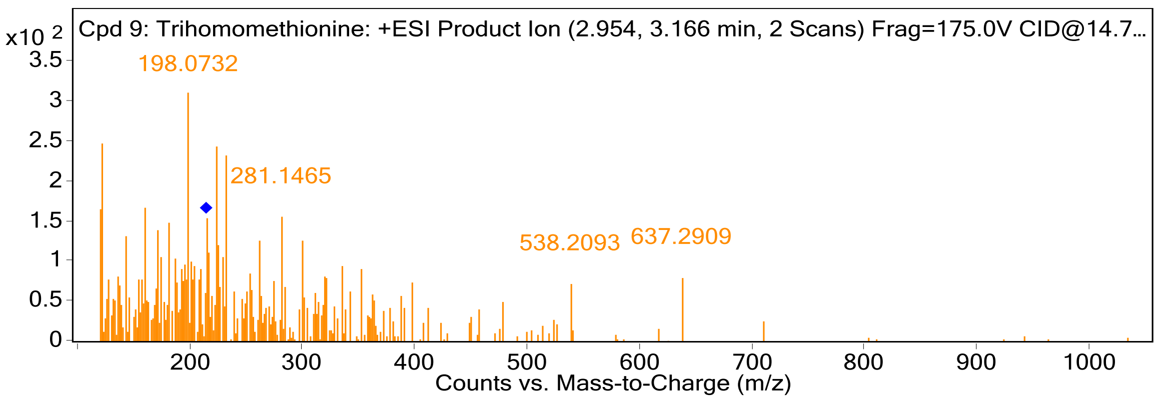

MS/MS Spectrum Peak List

m/z	z	Abund
120.081		166.56
121.0642		248.47
160.0968	1	168.35
170.082		139.13
181.0942		149.36
198.0732		312.01
214.0867		154.54
223.1448		243.64
231.1693		232.96
281.1465		155.98

Compound Structure

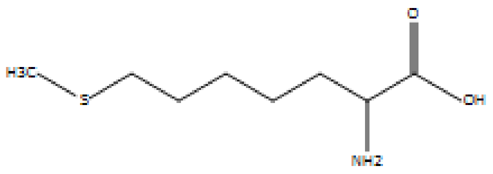

Compound Label	Name	*m/z*	RT	Algorithm	Mass

Cpd 10: Butyl 2-aminobenzoate	**Butyl 2-aminobenzoate**	194.117	3.318	Auto MS/MS	193.1093

MS Spectrum

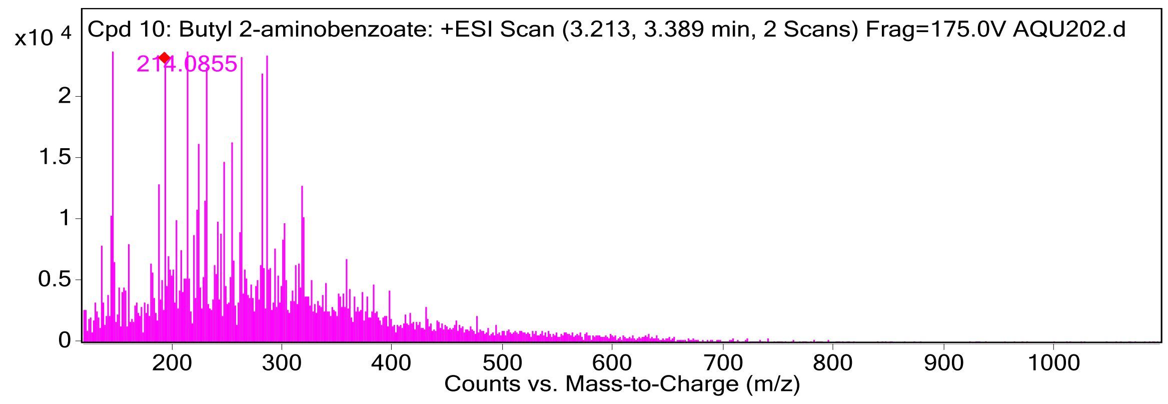

MS Zoomed Spectrum

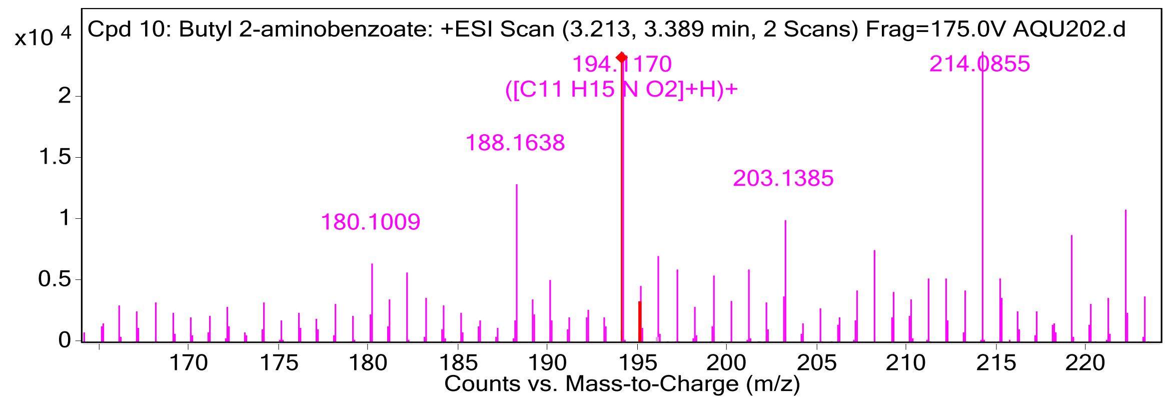

MS Spectrum Peak List

m/z	*Calc m/z*	Diff(ppm)	z	Abund	Formula	Ion
146.0597			1	56901.39		
194.117	194.1176	2.71	1	23714.64	C11 H15 N O2	(M+H)+
195.1175	195.1208	16.54	1	4621.65	C11 H15 N O2	(M+H)+
214.0855			1	24902.3		
224.1279			1	16242.18		
231.1694				22540.17		
254.1021			1	16326.55		
263.138			1	23386.75		
281.1484			1	21991.62		
286.1744			1	23454.79		

MSMS Spectrum

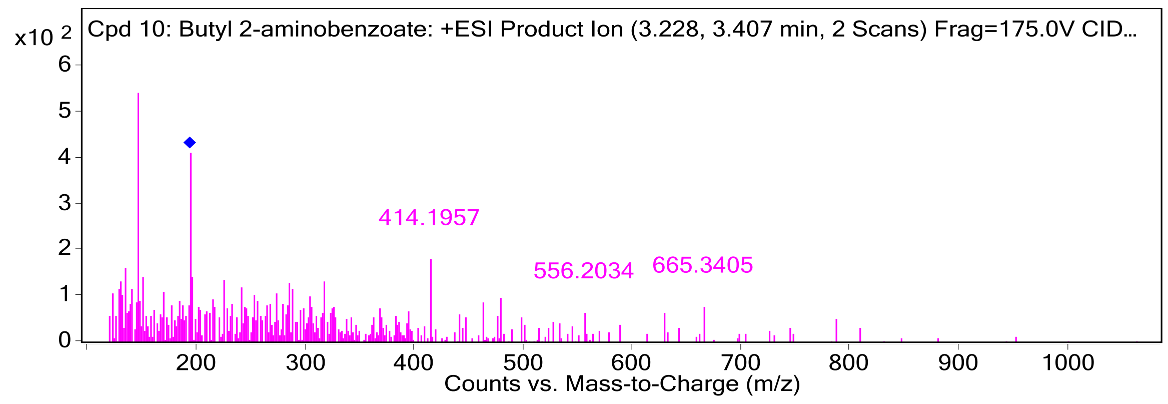

MS/MS Spectrum Peak List

m/z	z	Abund
130.1605		134.01
135.0447		161.23
146.0597	1	542.89
150.9845		144.32
194.1183	1	413.1
195.1485		141.65
224.1259		135.46
284.1133		131.05
316.3691		132.44
414.1957		181.24

Compound Structure

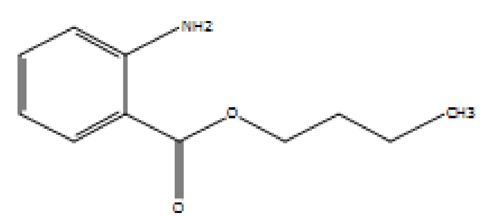

Compound Label	Name	*m/z*	RT	Algorithm	Mass
Cpd 11: Salicylanilide	**Salicylanilide**	214.0856	3.332	Auto MS/MS	213.0789

MS Spectrum

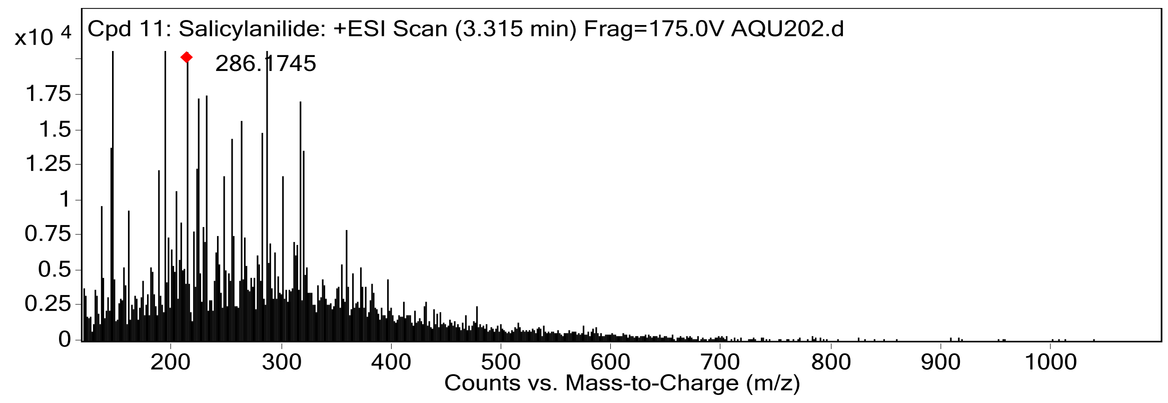

MS Zoomed Spectrum

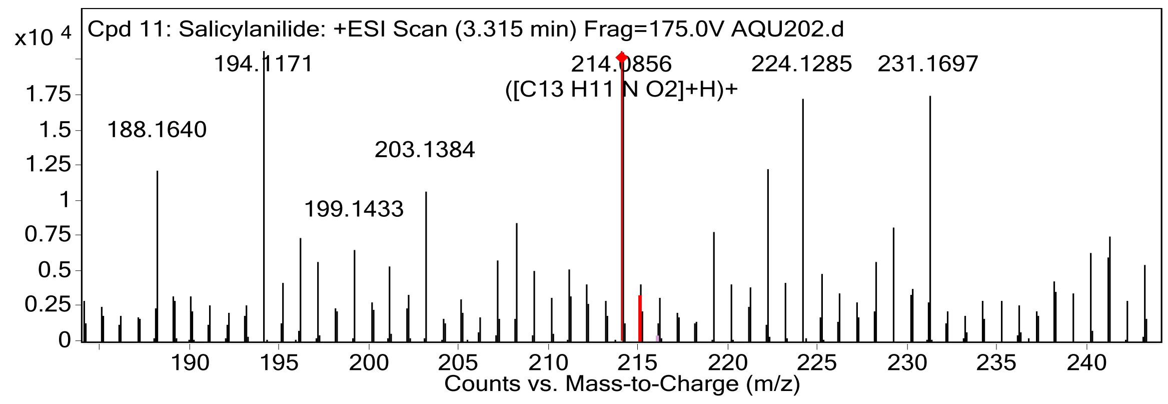

MS Spectrum Peak List

m/z	*Calc m/z*	Diff(ppm)	z	Abund	Formula	Ion
146.0596			1	46356.82		
194.1171			1	20955.1		
214.0856	214.0863	3.11	1	20580.29	C13 H11 N O2	(M+H)+
215.092	215.0895	-11.82	1	4178.22	C13 H11 N O2	(M+H)+
224.1285			1	17309.3		
231.1697				17463.38		
263.1378				15749.24		
281.1483			1	14892.19		
286.1745			1	37150.27		
317.2054				17061.21		

MSMS Spectrum

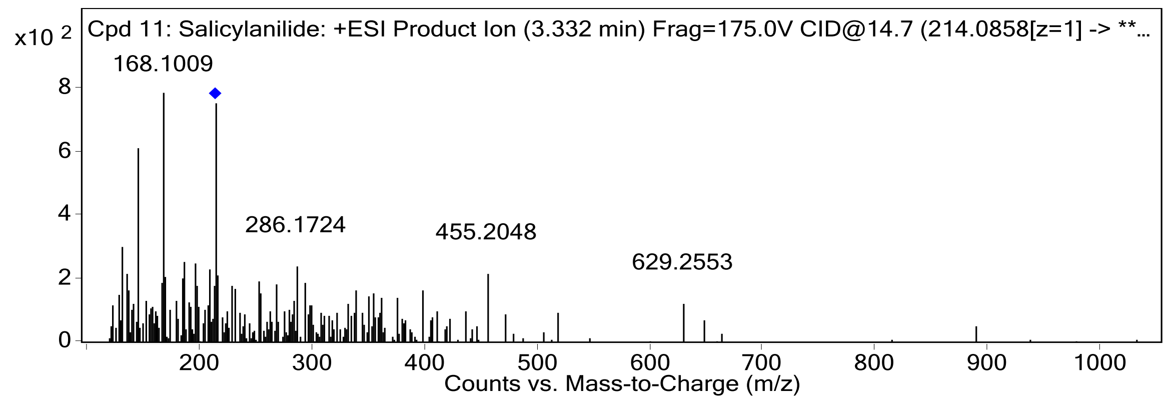

MS/MS Spectrum Peak List

m/z	Abund
132.1004	300.78
145.075	376.11
146.0583	613.96
168.1009	788.73
186.0745	253.06
196.0964	250.8
209.1274	231.37
214.0832	752.47
286.1724	240.46
455.2048	217.92

Compound Structure

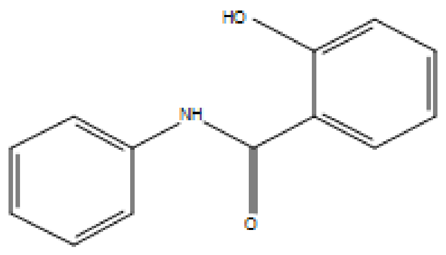

Compound Label	Name	*m/z*	RT	Algorithm	Mass
Cpd 12: L-phenylalanyl-L-proline	**L-phenylalanyl-L-proline**	263.1378	3.434	Auto MS/MS	262.1304

MS Spectrum

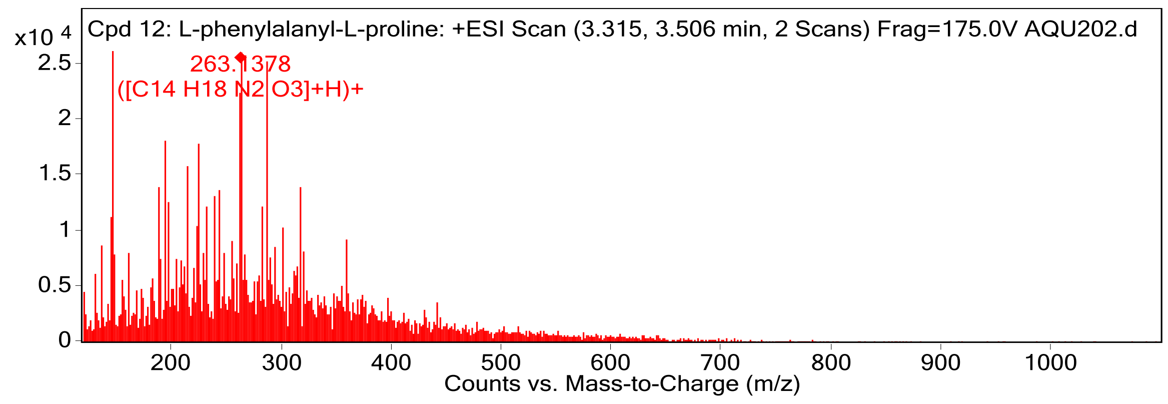

MS Zoomed Spectrum

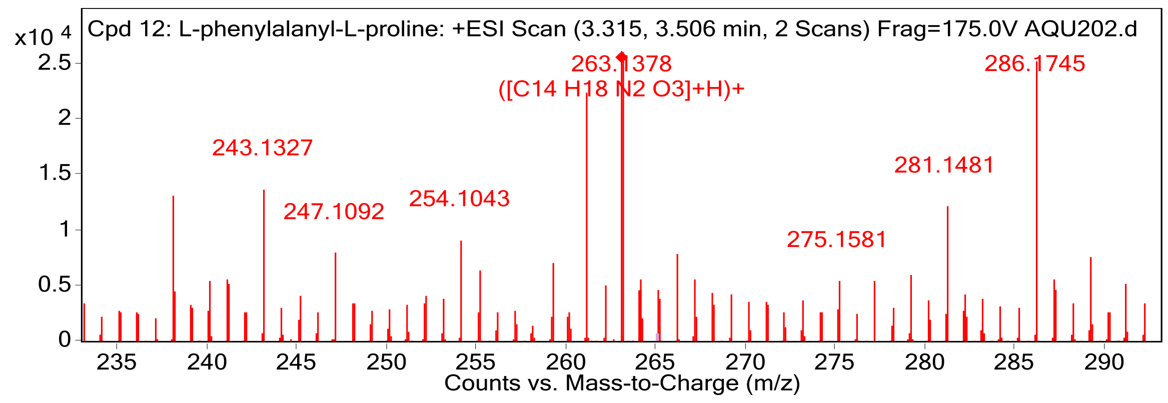

MS Spectrum Peak List

m/z	*Calc m/z*	Diff(ppm)	z	Abund	Formula	Ion

146.0596			1	80121.16		
188.1639				14011.43		
194.1171				18182.83		
214.0855				15881.33		

m/z	*Calc m/z*	Diff(ppm)	z	Abund	Formula	Ion
224.128			1	17942.13		
261.1248			1	22485.42		
263.1378	263.139	4.47	1	26095.12	C14 H18 N2 O3	(M+H)+
264.1398	264.1421	8.85	1	5592.63	C14 H18 N2 O3	(M+H)+
286.1745			1	25230.01		
317.2054				14023.65		

MSMS Spectrum

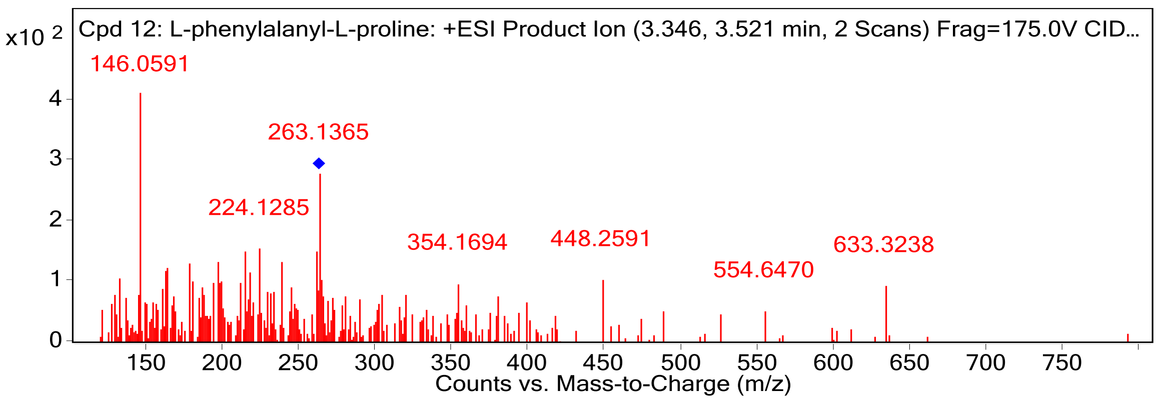

MS/MS Spectrum Peak List

m/z	z	Abund
146.0591		412.24
163.08		122.01
178.1192		131.69
197.1275		133.75
214.1028		151.31
224.0903		131.16
224.1285	1	154.23
238.0691		132.34
261.1446		149.9
263.1365		279.07

Compound Structure

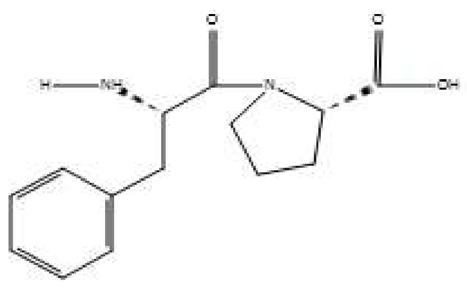

Compound Label	Name	*m/z*	RT	Algorithm	Mass
Cpd 13: Isocarbostyril	**Isocarbostyril**	146.0596	3.538	Auto MS/MS	145.0523

MS Spectrum

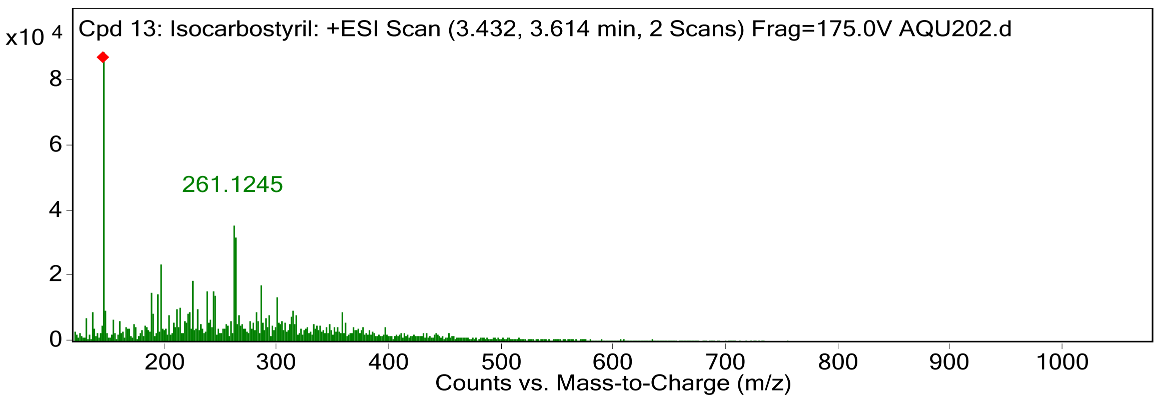

MS Zoomed Spectrum

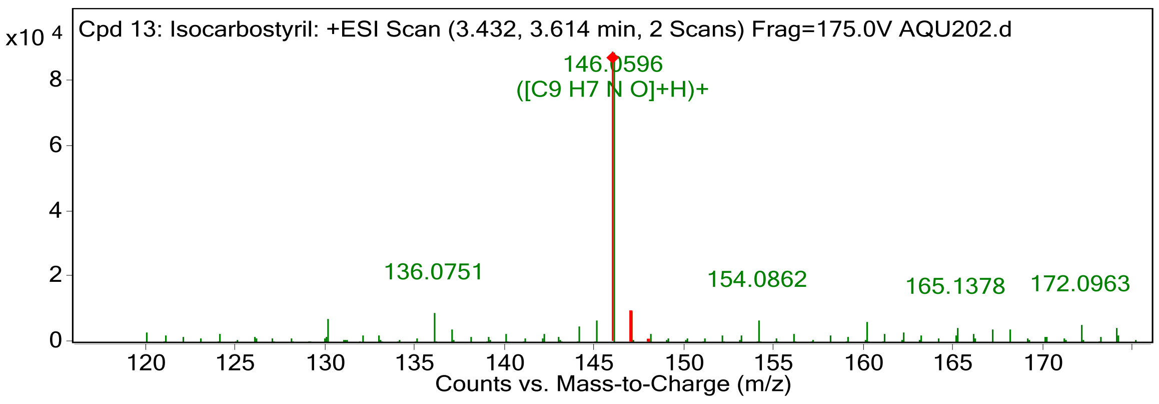

MS Spectrum Peak List

m/z	*Calc m/z*	Diff(ppm)	z	Abund	Formula	Ion
146.0596	146.06	3.26	1	88791.49	C9 H7 N O	(M+H)+
147.0632	147.0632	0.33	1	9530.11	C9 H7 N O	(M+H)+
148.063	148.0657	18.31	1	2844.26	C9 H7 N O	(M+H)+
197.1278				23640.89		
224.1277			1	18768.69		
238.0706				15566.85		
243.1326				15543.03		
261.1245			1	35569.26		
263.1378			1	31870.78		
286.1747				17182.44		

MSMS Spectrum

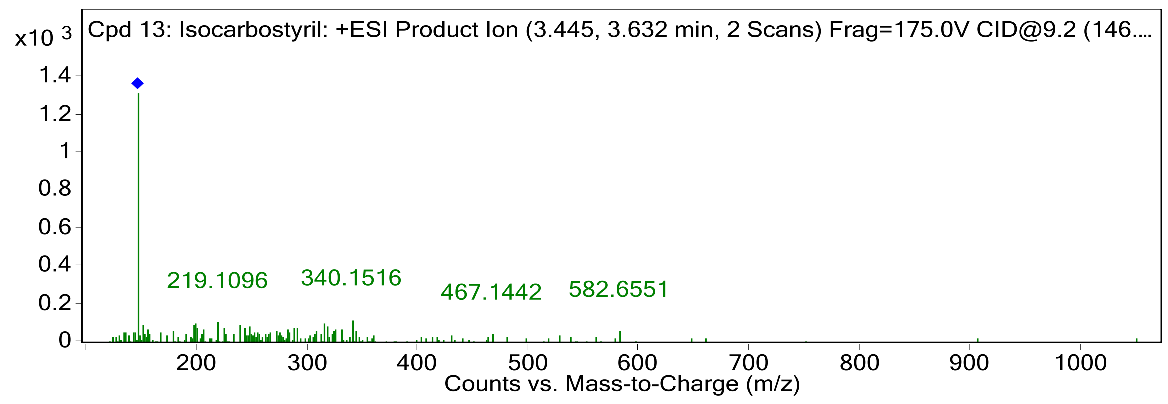

MS/MS Spectrum Peak List

m/z	z	Abund
146.0597	1	1317.26
147.0653	1	212.53
150.9809		92.93
197.1274		92.28
198.1151		105.14
219.1096		108.03
238.0709		91.51
247.1277		89.32
314.2031		105.3
340.1516		122.04

Compound Structure

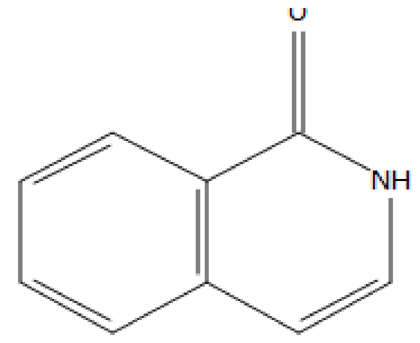

Compound Label	*m/z*	RT	Algorithm
Compound 14	240.1215	3.68	Auto MS/MS

MS Spectrum

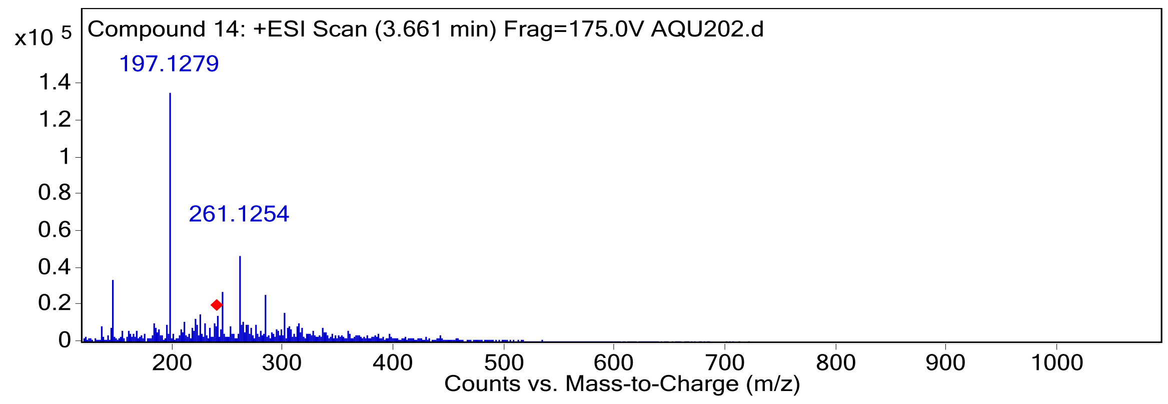

MS Zoomed Spectrum

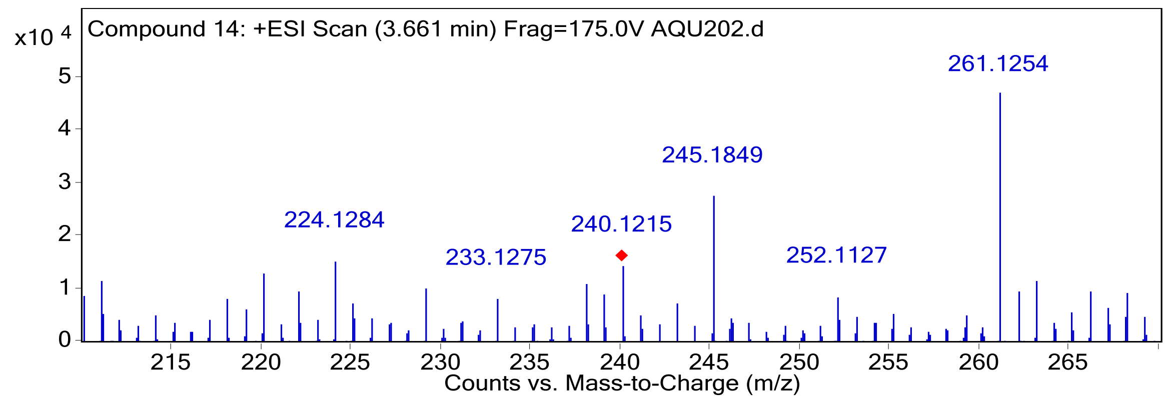

MS Spectrum Peak List

m/z	z	Abund
146.0594		34407.05
197.1279	1	135505.36
198.131	1	18718.76
240.1215	1	14464.13
241.1172	1	5083.93
242.1153	1	2397.1
245.1849		27569.98
261.1254	1	47215.69
283.1733		25649.71
300.1974		16453.75

MSMS Spectrum

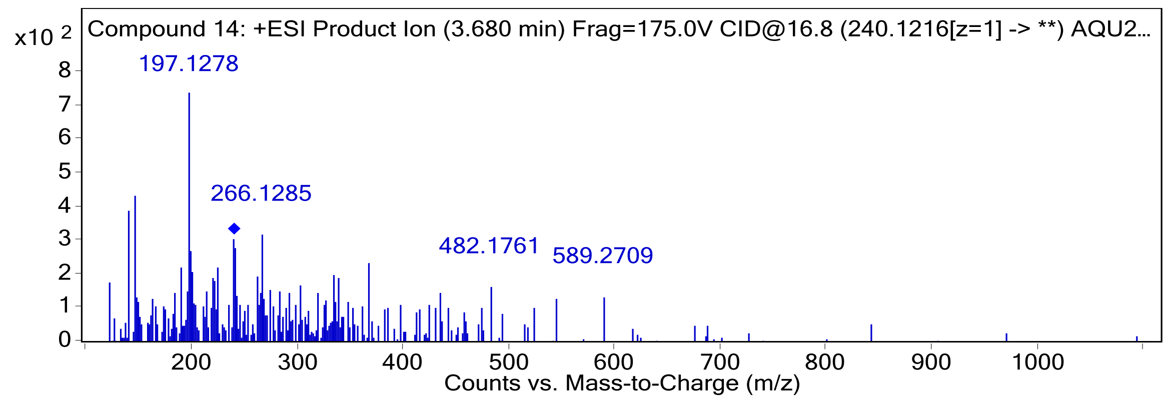

MS/MS Spectrum Peak List

m/z	Abund
140.0721	389.23
146.0591	433.32

190.0823	221.54
197.1278	739.86
198.1294	268.45
224.1275	219.12
239.1413	304.3
240.1356	280.18
266.1285	317.89
367.167	236

Compound Label	Name	*m/z*	RT	Algorithm	Mass
Cpd 15: 4-Prenyldihydropinosylvin	**4-Prenyldihydropinosylvi**	305.1553	3.733	Auto MS/MS	282.1647

MS Spectrum

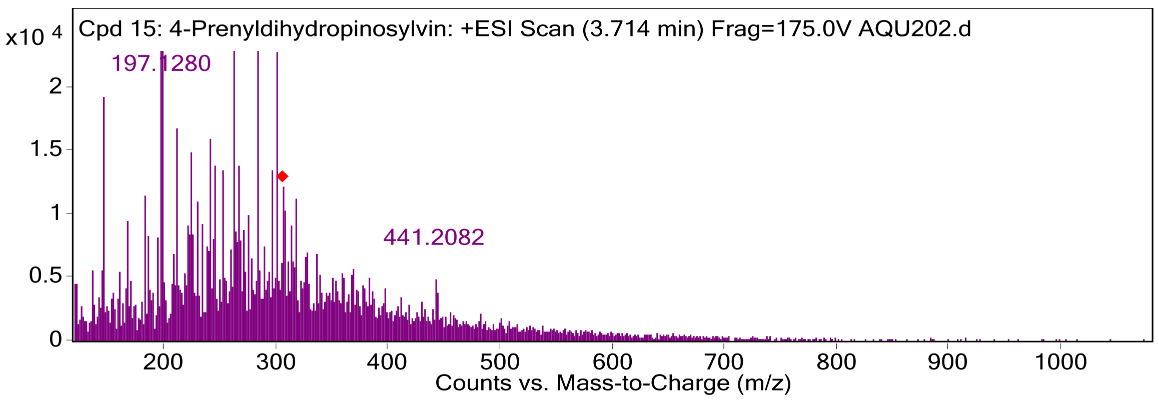

MS Zoomed Spectrum

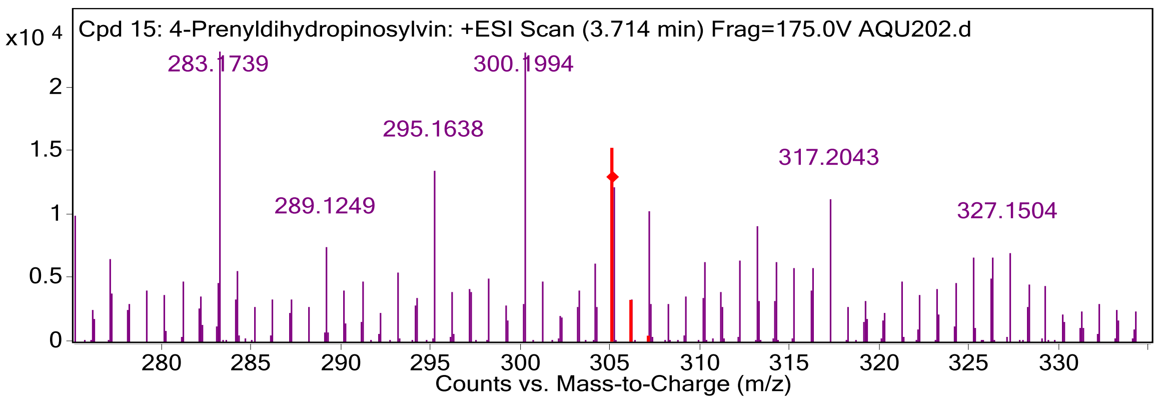

MS Spectrum Peak List

m/z	*Calc m/z*	Diff(ppm)	z	Abund	Formula	Ion
146.0595				19269.45		
197.128			1	345759.44		
198.1312			1	37539.05		
210.1119				16818.86		
261.1259			1	32300.97		
283.1739			1	37800.24		
300.1994				22778.39		
305.1553	305.1512	-13.36		12208.12	C19 H22 O2	(M+Na)+
306.1511	306.1546	11.47	1	3433.45	C19 H22 O2	(M+Na)+
307.1617	307.1576	-13.32	1	3065.59	C19 H22 O2	(M+Na)+

MSMS Spectrum

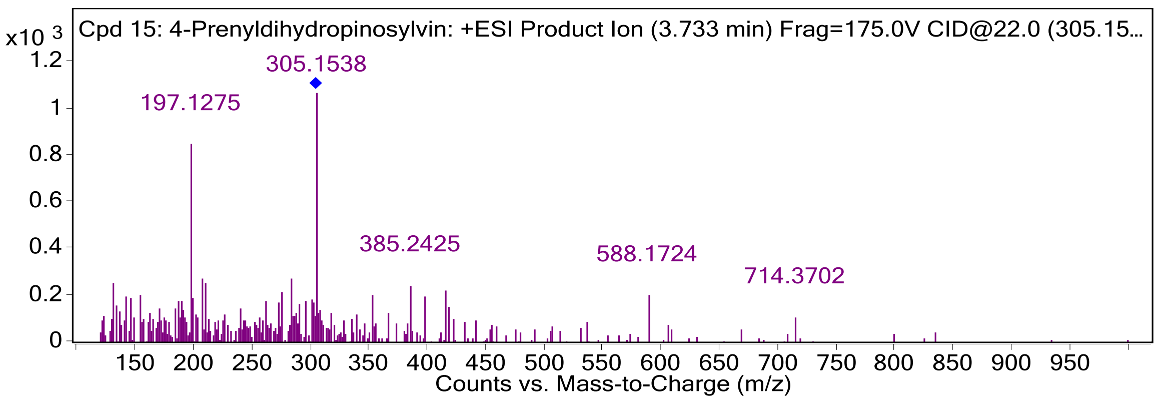

MS/MS Spectrum Peak List

m/z	z	Abund
130.1604		257.28
197.1275	1	850.86
207.0756		272.56
209.0797		255.56
275.1392		220.07
283.1755	1	274.56
304.1325		253.13
305.1538	1	1070.55
385.2425		245.02
415.2317		226.59

Compound Structure

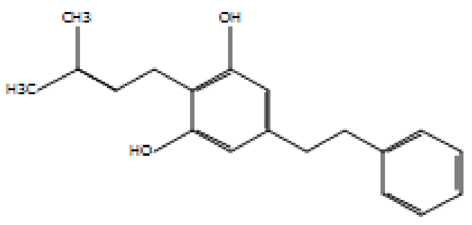

Compound Label	Name	*m/z*	RT	Algorithm	Mass
Cpd 16: Fasoracetam	**Fasoracetam**	197.128	3.898	Auto MS/MS	196.1208

MS Spectrum

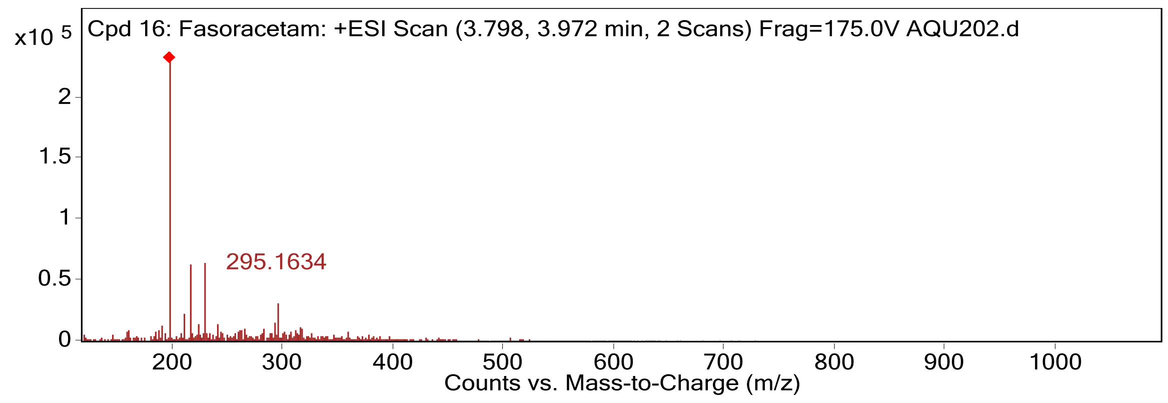

MS Zoomed Spectrum

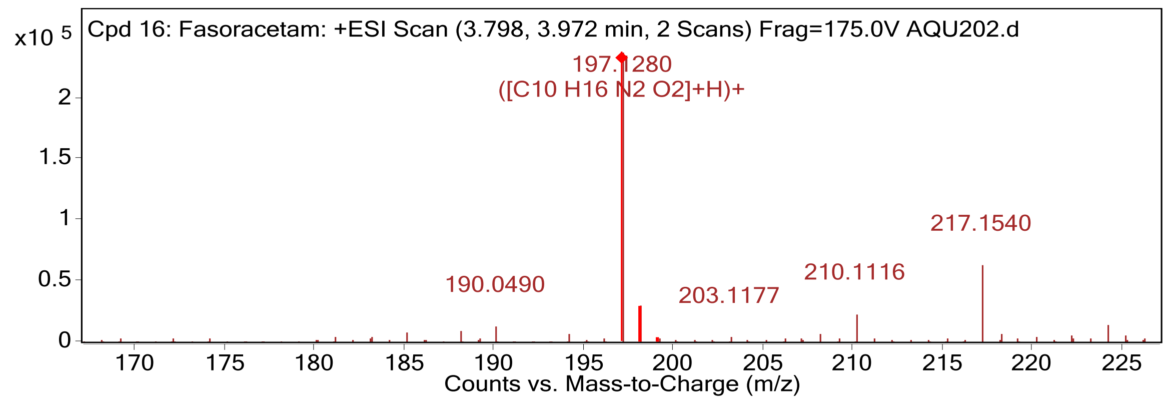

MS Spectrum Peak List

m/z	*Calc m/z*	Diff(ppm)	z	Abund	Formula	Ion
197.128	197.1285	2.35	1	237447.13	C10 H16 N2 O2	(M+H)+
198.131	198.1315	2.46	1	30515.26	C10 H16 N2 O2	(M+H)+
199.1407	199.1338	-35.03	1	3656.76	C10 H16 N2 O2	(M+H)+
210.1116				23774.95		
217.154			1	63860.73		
224.1276				14729.98		
229.1537			1	65480.39		
241.1536				14837.14		
293.1482			1	15356.46		
295.1634			1	31587.76		

MSMS Spectrum

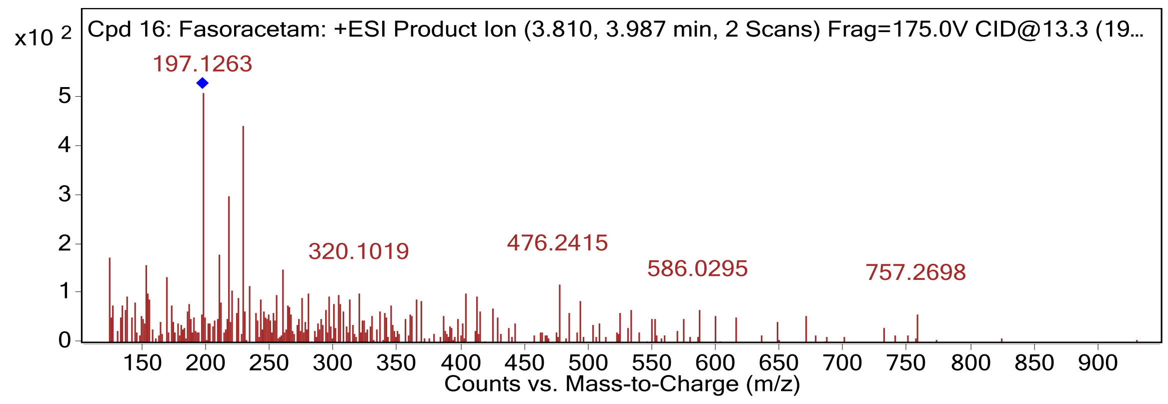

MS/MS Spectrum Peak List

m/z	z	Abund
124.1116		174.06
152.1079		159.18
169.1334		133.63
197.1263	1	511.56
210.1099		180.61
217.1544		300.97
229.1547		443.12
234.0845		115
259.2012	1	150.84
476.2415		118.98

Compound Structure

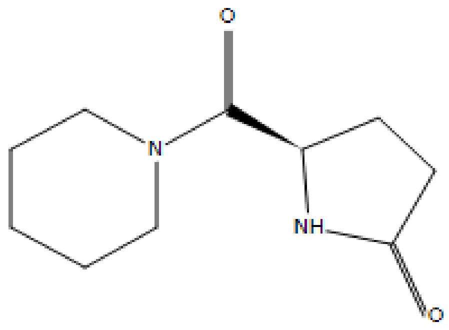

Compound Label	Name	*m/z*	RT	Algorithm	Mass
Cpd 17: Sinapoylputrescine	**Sinapoylputrescine**	295.1636	3.909	Auto MS/MS	294.1562

MS Spectrum

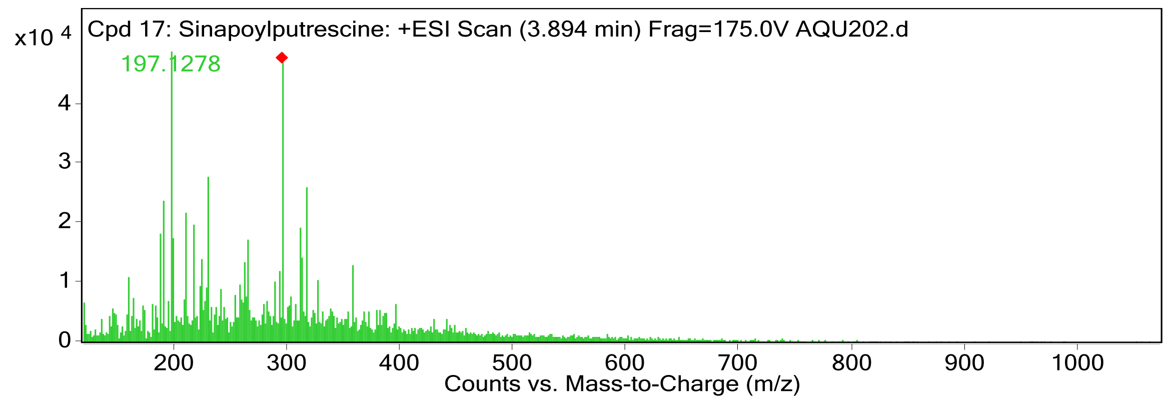

MS Zoomed Spectrum

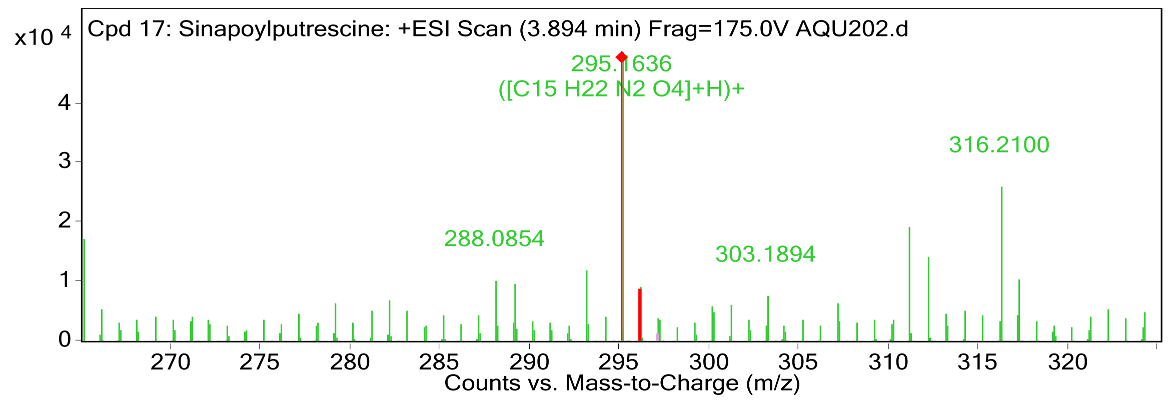

MS Spectrum Peak List

m/z	*Calc m/z*	Diff(ppm)	z	Abund	Formula	Ion
188.0703				18269.23		
190.0493				23714.14		
197.1278			1	150129.8		
210.1118			1	21837.78		
217.1537				19735.63		
229.1536			1	27957.78		
295.1636	295.1652	5.5	1	48679.98	C15 H22 N2 O4	(M+H)+
296.1658	296.1684	8.85	1	9205.22	C15 H22 N2 O4	(M+H)+
311.1212				19376.6		
316.21			1	26191.91		

MSMS Spectrum

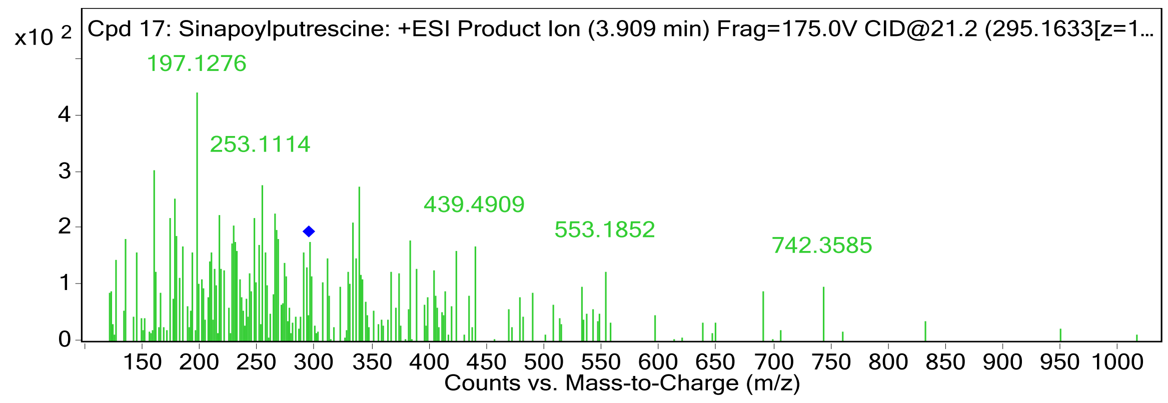

MS/MS Spectrum Peak List

m/z	z	Abund
160.0739		305.02
174.0916		219.17
177.144		255.44
197.1276	1	443.36
216.1044		225.9
247.1452		219.3
253.1114		277.19
265.1545		227.04
332.1319		211.57
338.1734		275.6

Compound Structure

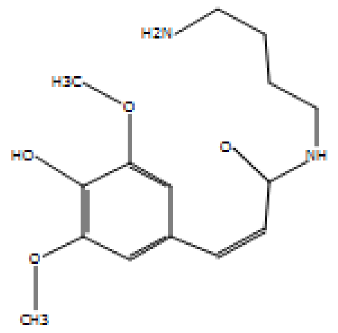

Compound Label	Name	*m/z*	RT	Algorithm	Mass
Cpd 18: 2,4'-Diphenyldiamine	**2,4'-Diphenyldiamine**	185.1067	4.409	Auto MS/MS	184.0997

MS Spectrum

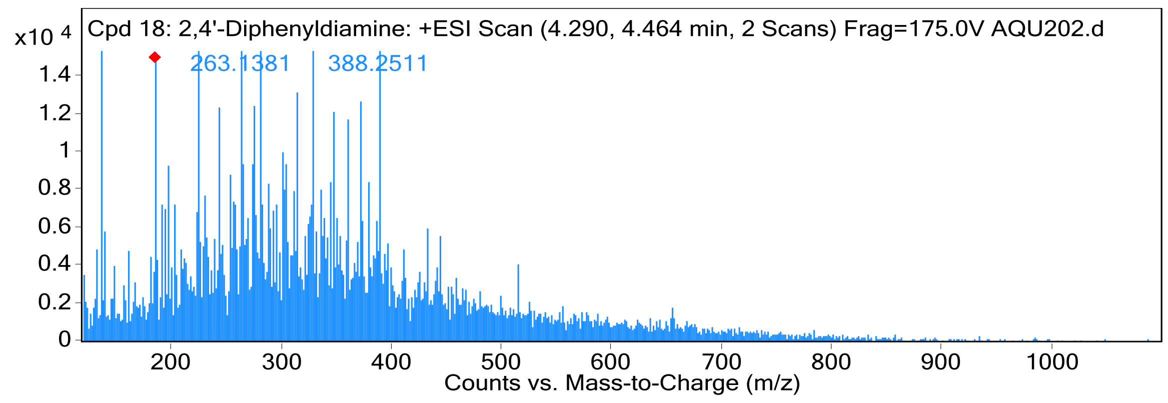

MS Zoomed Spectrum

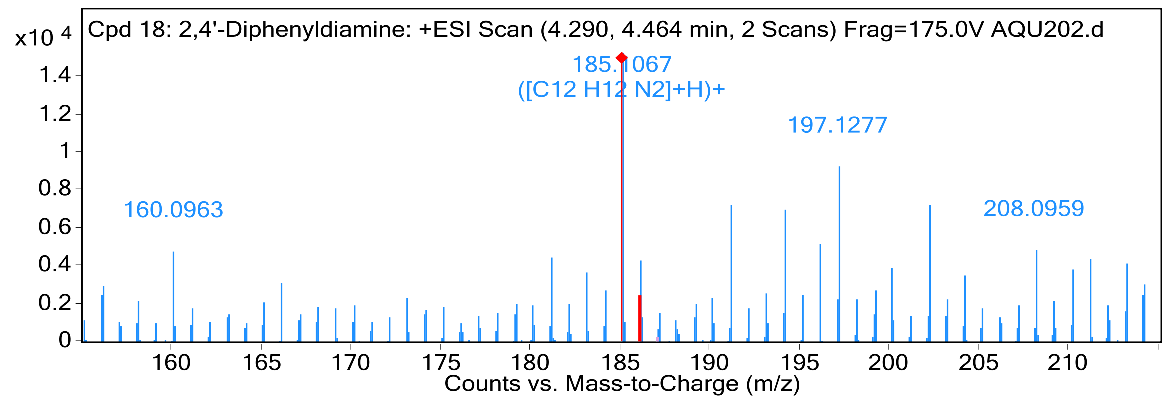

MS Spectrum Peak List

m/z	*Calc m/z*	Diff(ppm)	z	Abund	Formula	Ion
136.0753				18615.7		

185.1067	185.1073	3.42		15286.72	C12 H12 N2	(M+H)+
186.1112	186.1104	-4.3	1	4356.02	C12 H12 N2	(M+H)+
224.1273			1	15937.73		
263.1381			1	66247.09		
280.156			1	21688.81		
314.2052				13135.55		
328.2201				15381.98		
371.2248			1	12659.29		
388.2511			1	35778.45		

MSMS Spectrum

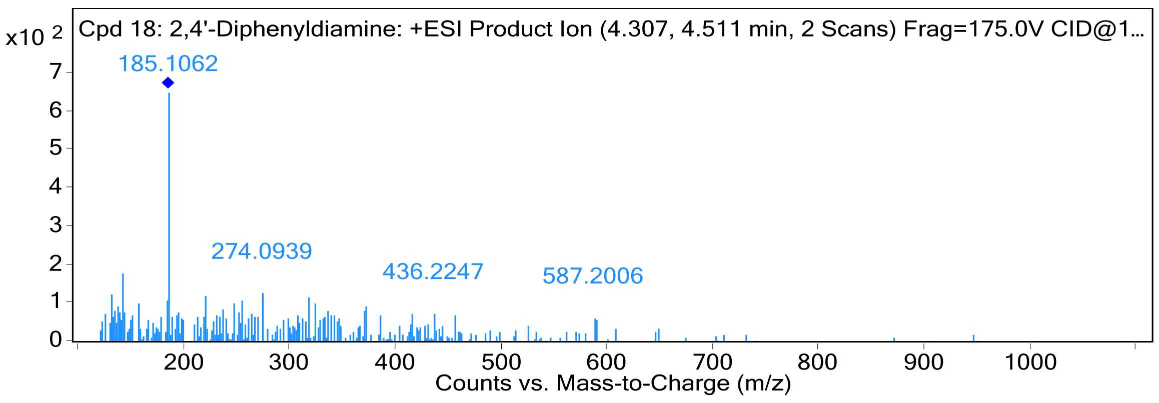

MS/MS Spectrum Peak List

m/z	z	Abund
132.1014		126.1
142.0845		180.65
184.1043		109.25
185.1062	1	650.5
185.1263		136.81
186.107	1	120.47
220.1355		119.83
255.1346		109.45
274.0939		128.15
318.1323		117.3

Compound Structure

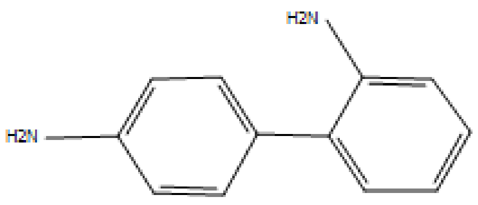

Compound Label	Name	*m/z*	RT	Algorithm	Mass
Cpd 19: Pirbuterol	**Pirbuterol**	263.1376	4.454	Auto MS/MS	240.1482

MS Spectrum

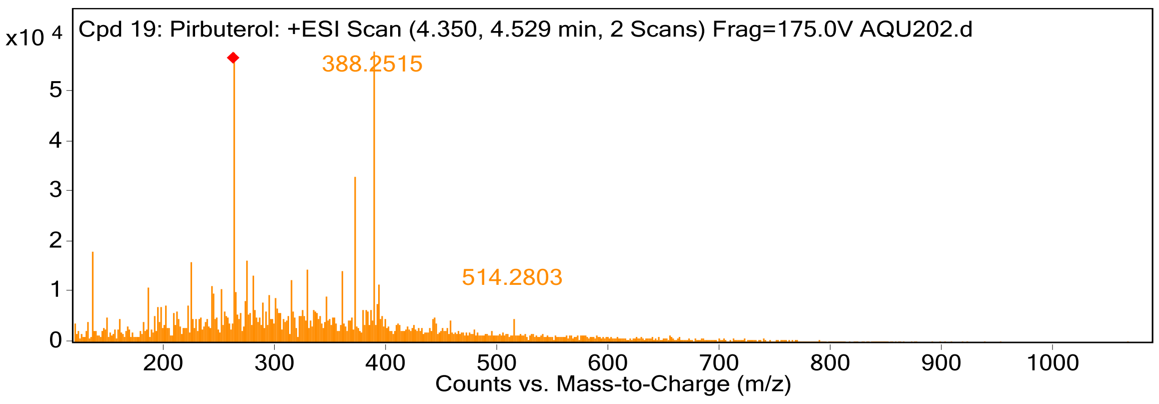

MS Zoomed Spectrum

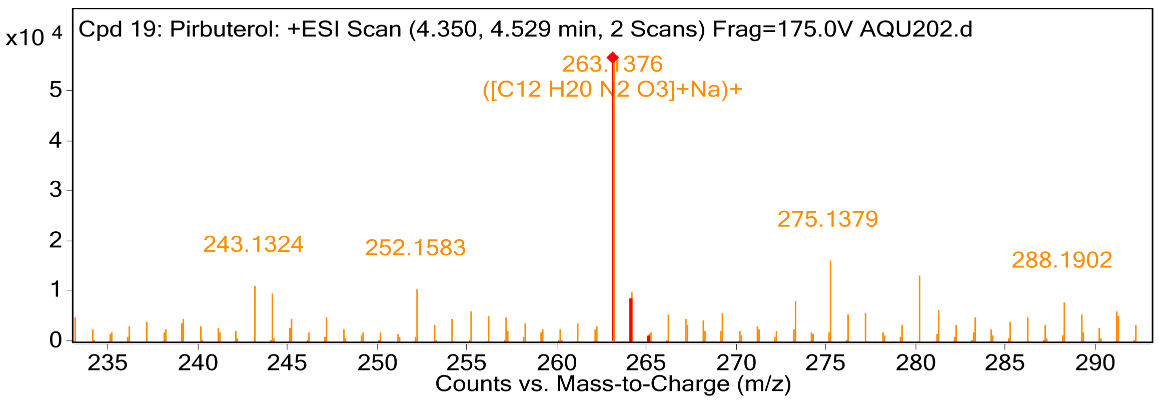

MS Spectrum Peak List

m/z	*Calc m/z*	Diff(ppm)	z	Abund	Formula	Ion
136.0752				18302.06		
224.1275			1	16209.25		
263.1376	263.1366	-3.74	1	57896.4	C12 H20 N2 O3	(M+Na)+
264.141	264.1397	-5.05	1	10044.15	C12 H20 N2 O3	(M+Na)+
265.1324	265.142	36.07	1	1760.8	C12 H20 N2 O3	(M+Na)+
275.1379			1	16357.54		
328.2201				14627.19		
371.2252			1	33144.29		
388.2515			1	92077.11		
389.2538			1	18255.26		

MSMS Spectrum

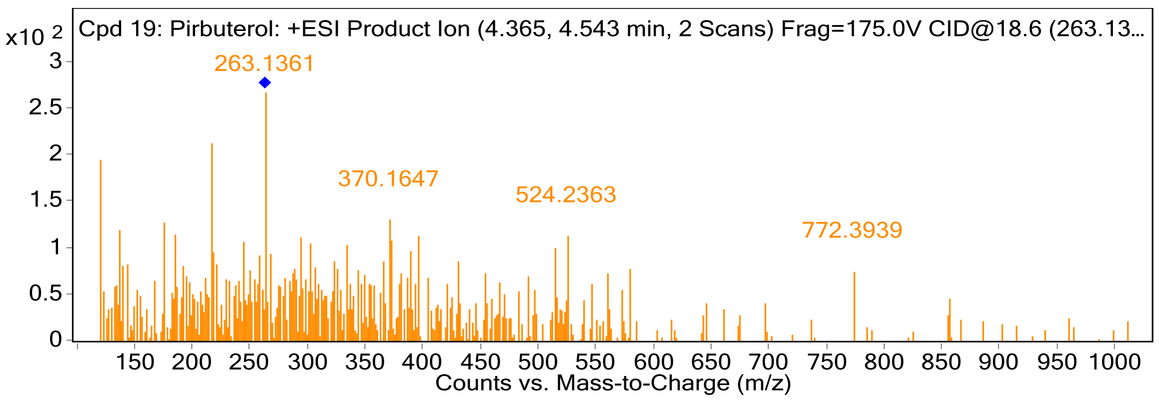

MS/MS Spectrum Peak List

m/z	Abund
120.0803	194.71
136.0741	120.64
175.0642	127.5
184.0748	115.64
216.1025	212.7
263.1361	268.03
293.1297	112.14
370.1647	130.64
395.1793	113.21
524.2363	113.45

Compound Structure

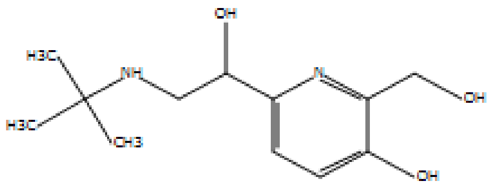

Compound Label	*m/z*	RT	Algorithm
Compound 20	388.2514	4.584	Auto MS/MS

MS Spectrum

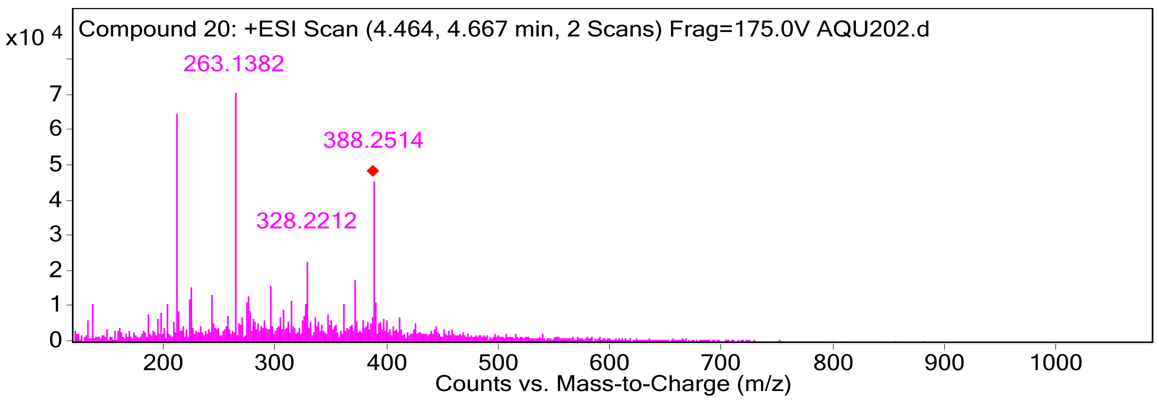

MS Zoomed Spectrum

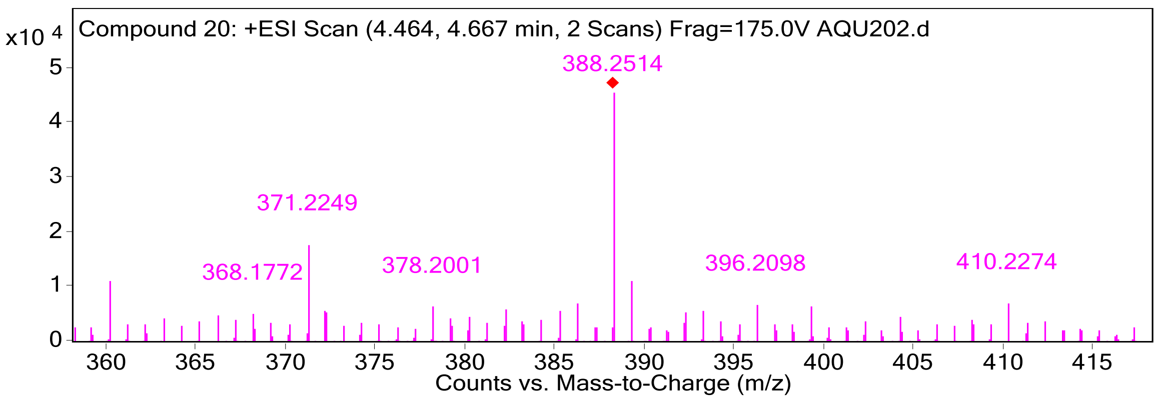

MS Spectrum Peak List

m/z	z	Abund
211.1433	1	64600.33
224.1272		15800.3
243.1323		13677.96
263.1382	1	70819.42
295.1636	1	16248.46
328.2212	1	22701.94
371.2249	1	17864.63
388.2514	1	45608.13
389.2577	1	11227.46
390.2586	1	2698.84

MSMS Spectrum

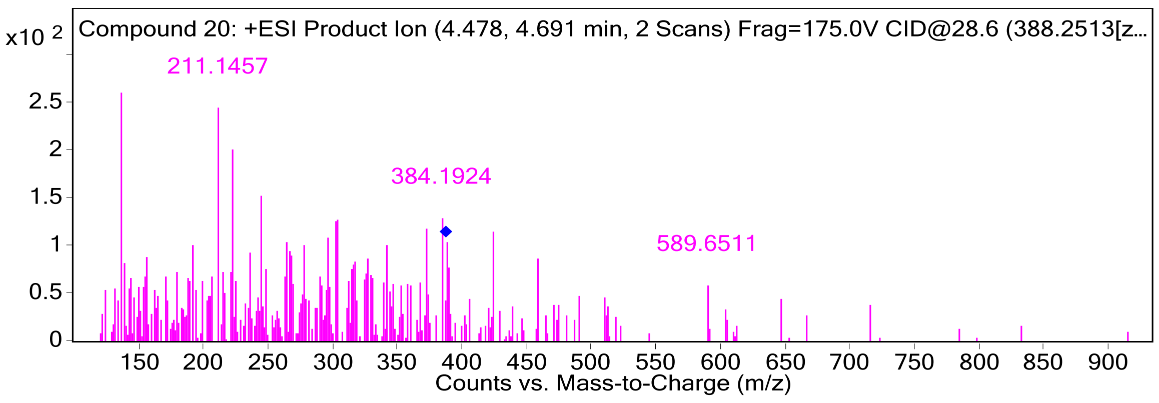

MS/MS Spectrum Peak List

m/z	Abund
136.0745	261.16
211.1457	245.5

222.1108	200.61
244.1318	153.31
296.0897	109.07
302.2035	125.65
303.0445	127.56
371.2297	117.99
384.1924	130.08
423.2115	115.2

Compound Label	Name	*m/z*	RT	Algorithm	Mass
Cpd 21: Avocadynofuran	**Avocadynofuran**	269.1844	4.606	Auto MS/MS	246.1943

MS Spectrum

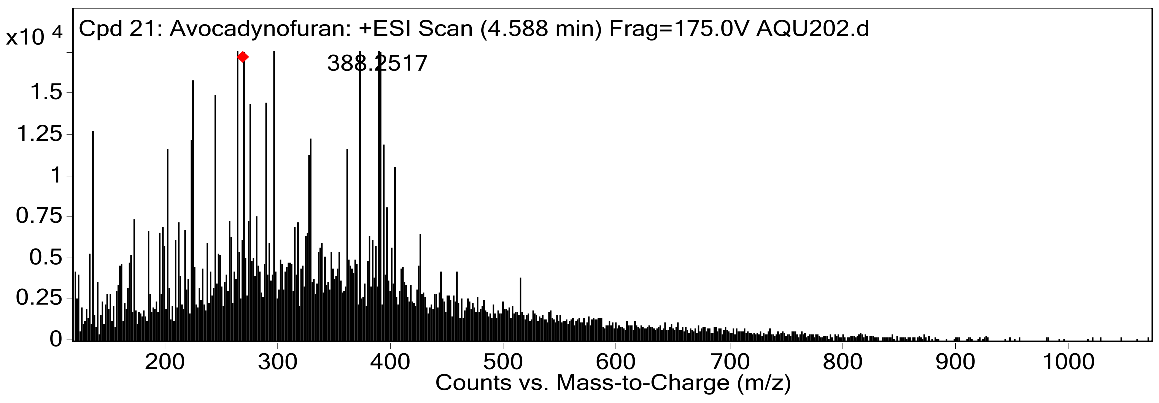

MS Zoomed Spectrum

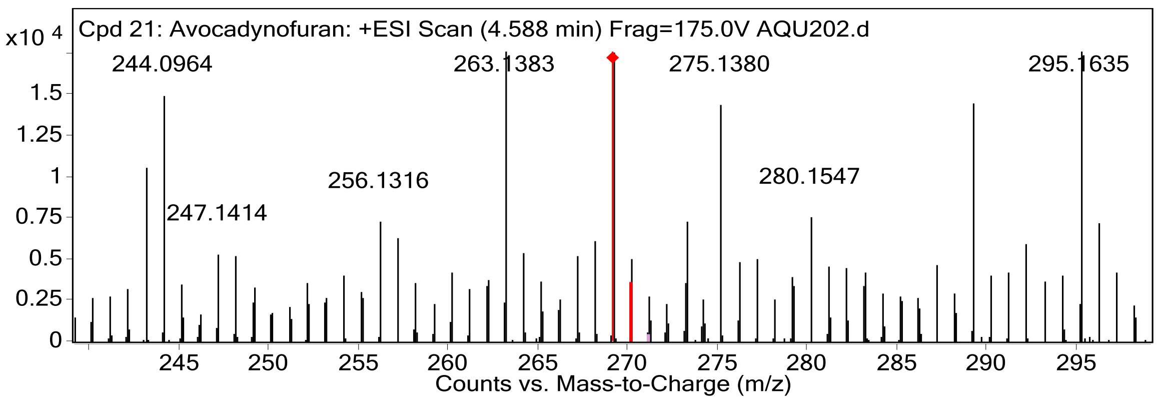

MS Spectrum Peak List

m/z	*Calc m/z*	Diff(ppm)	z	Abund	Formula	Ion
224.1273			1	15878.4		
244.0964				14962.66		
263.1383			1	28007.38		
269.1844	269.1876	11.8	1	17565.65	C17 H26 O	(M+Na)+
270.184	270.191	25.84	1	5094.32	C17 H26 O	(M+Na)+
289.1533				14519.25		
295.1635			1	34623.4		
371.2251			1	37132.24		
388.2517			1	88508.38		
389.2552			1	17565.58		

MSMS Spectrum

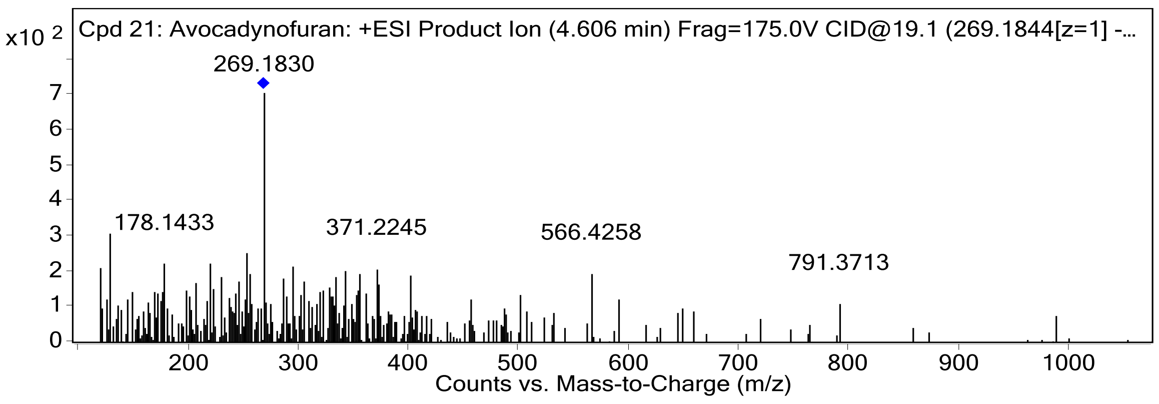

MS/MS Spectrum Peak List

m/z	Abund
120.0808	209.88
128.0666	309.06
178.1433	222.56
219.0946	222.13
252.1914	253
268.1637	251.51
269.183	707.51
295.1661	214.03
342.1962	202.29
371.2245	208.85

Compound Structure

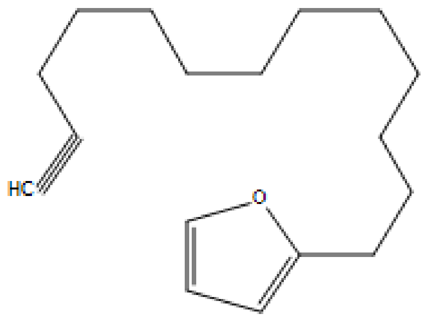

Compound Label	Name	*m/z*	RT	Algorithm	Mass
Cpd 22: L,L-Cyclo(leucylprolyl)	**L,L-Cyclo(leucylprolyl)**	211.1433	4.756	Auto MS/MS	210.1362

MS Spectrum

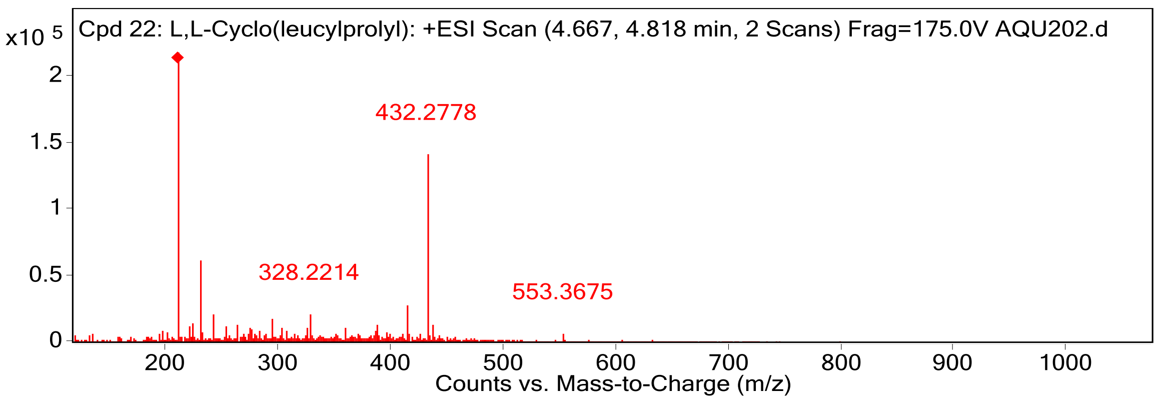

MS Zoomed Spectrum

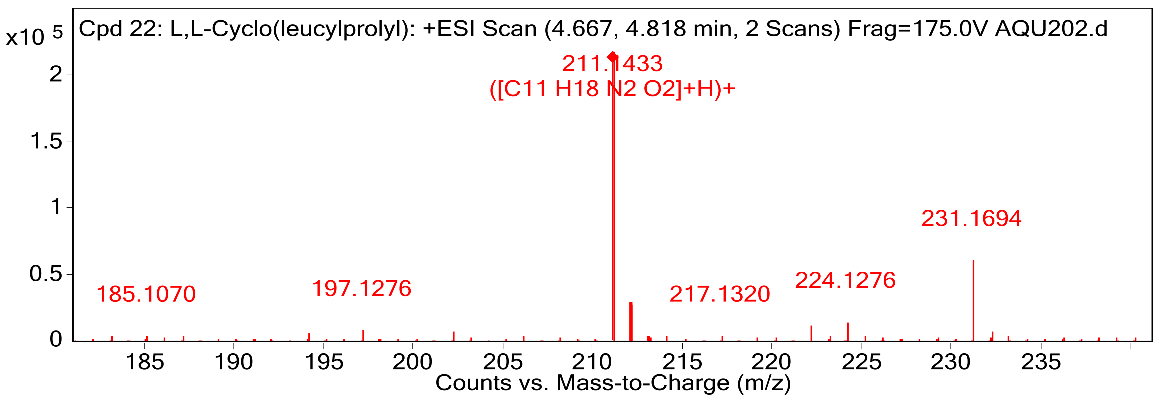

MS Spectrum Peak List

m/z	*Calc m/z*	Diff(ppm)	z	Abund	Formula	Ion
211.1433	211.1441	3.64	1	218017.16	C11 H18 N2 O2	(M+H)+
212.1465	212.1472	3.29	1	27705.83	C11 H18 N2 O2	(M+H)+
213.1542	213.1495	-21.99	1	3759.26	C11 H18 N2 O2	(M+H)+
231.1694			1	61590.21		
243.1325				21633.47		
295.1633			1	18306.49		
328.2214				21462.56		
415.251			1	28073.15		
432.2778			1	142009.16		
433.2805			1	30482.48		

MSMS Spectrum

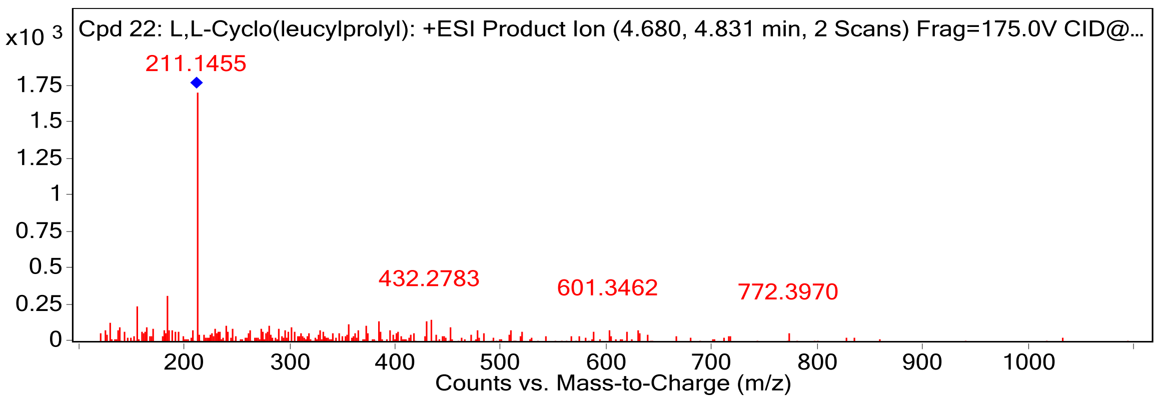

MS/MS Spectrum Peak List

m/z	z	Abund
129.0704		129.77
154.0715		249.93
183.1492		321.42
211.1455	1	1709.69
212.1468	1	266.86
239.0987		113.98
354.1028		121.87
383.1584		138.09
428.258		140.83
432.2783		148.94

Compound Structure

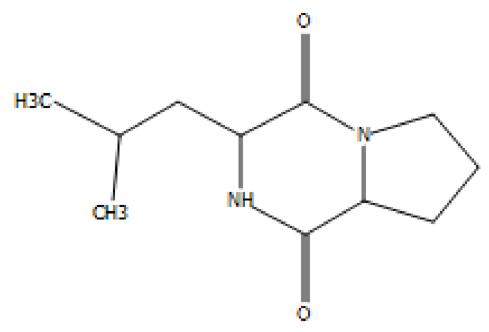

Compound Label	Name	*m/z*	RT	Algorithm	Mass
Cpd 23: Istamycin C1	**Istamycin C1**	432.2772	4.83	Auto MS/MS	431.27

MS Spectrum

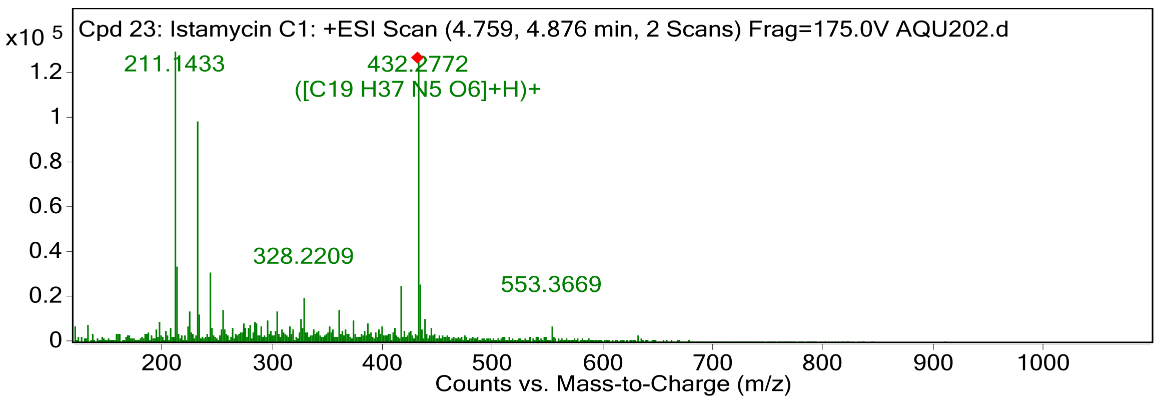

MS Zoomed Spectrum

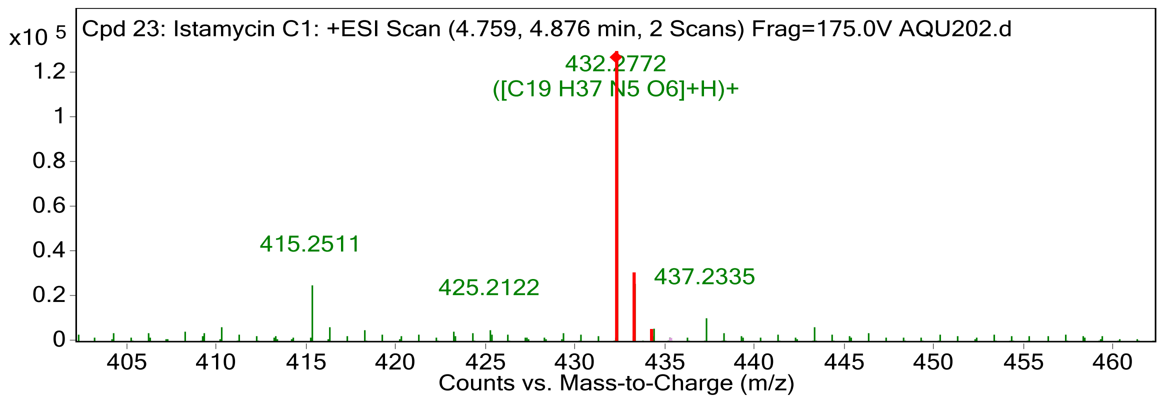

MS Spectrum Peak List

m/z	*Calc m/z*	Diff(ppm)	z	Abund	Formula	Ion
211.1433			1	249204.22		
212.1463			1	33996.36		
231.1694			1	98392.65		
243.1328			1	31445.23		
328.2209			1	19965.38		
360.2094				14673.73		
415.2511			1	25128.28		
432.2772	432.2817	10.2	1	129216.23	C19 H37 N5 O6	(M+H)+
433.2807	433.2846	9.06	1	25643.81	C19 H37 N5 O6	(M+H)+
434.2817	434.287	12.07	1	5742.54	C19 H37 N5 O6	(M+H)+

MSMS Spectrum

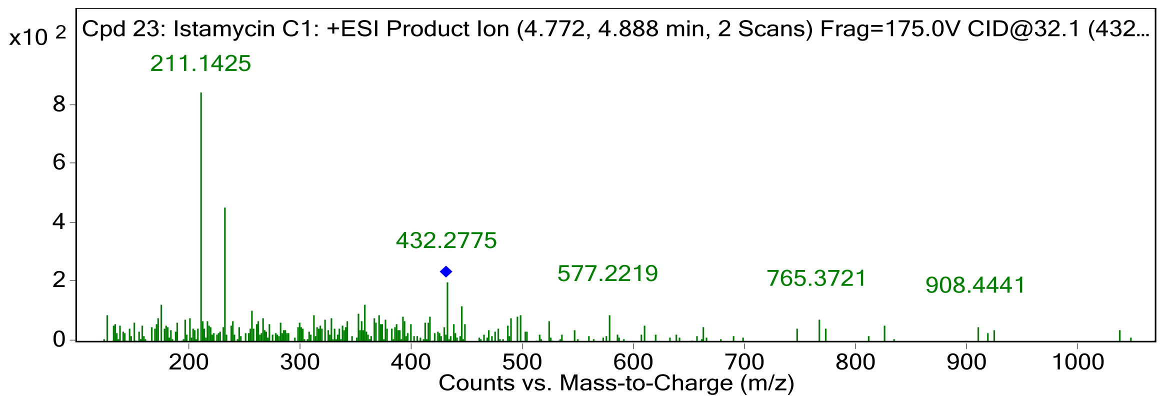

MS/MS Spectrum Peak List

m/z	z	Abund
126.0914		93.17
174.112		126.19
211.1425	1	846.05
231.1694		457.47
256.1268		106.57
351.2176		95.38
357.1739		126.01
370.2111		93.04
432.2775		202.65
445.2625		121.63

Compound Structure

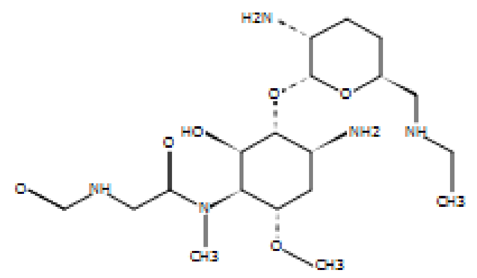

Compound Label	Name	*m/z*	RT	Algorithm	Mass
Cpd 24: Progeldanamycin	**Progeldanamycin**	476.3034	5.12	Auto MS/MS	475.296

MS Spectrum

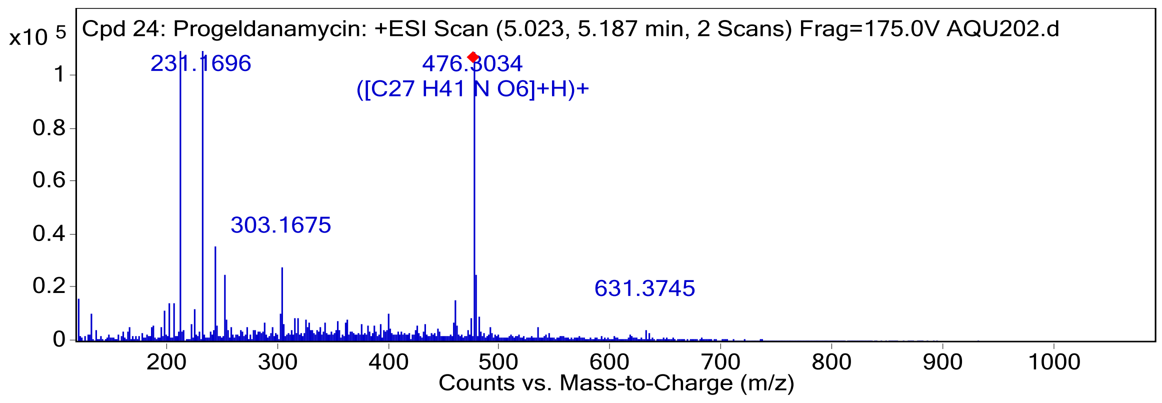

MS Zoomed Spectrum

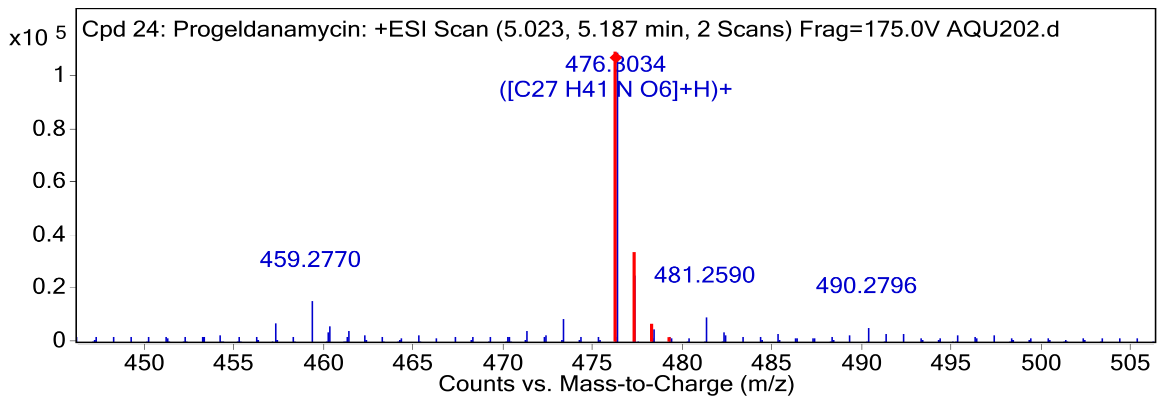

MS Spectrum Peak List

m/z	*Calc m/z*	Diff(ppm)	z	Abund	Formula	Ion
211.1433			1	165461.56		

231.1696			1	174168.13		
232.1728			1	21468.18		
243.1332			1	35853.56		
252.0859			1	25161.53		
303.1675			1	28368.87		
476.3034	476.3007	-5.76	1	109221.86	C27 H41 N O6	(M+H)+
477.3064	477.304	-5.13	1	25109.26	C27 H41 N O6	(M+H)+
478.3077	478.3068	-1.81	1	5345.11	C27 H41 N O6	(M+H)+
479.3102	479.3095	-1.42	1	1540.47	C27 H41 N O6	(M+H)+

MSMS Spectrum

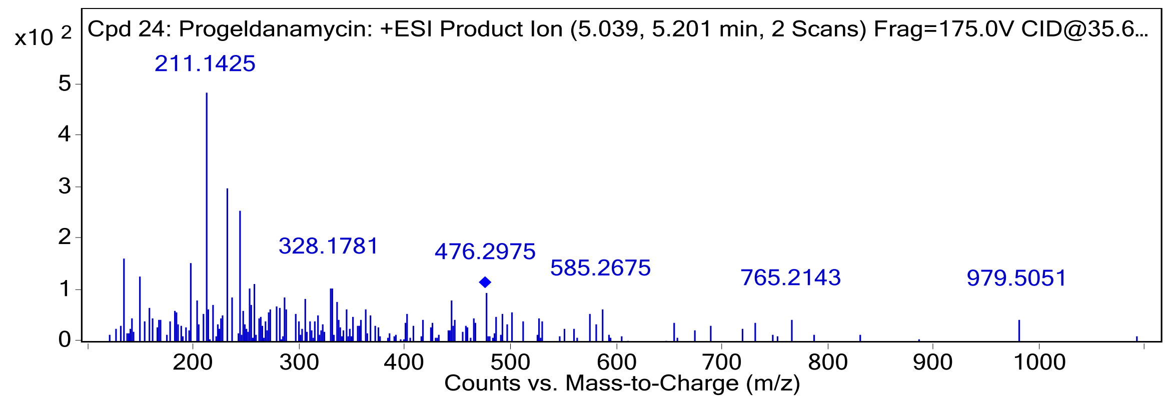

MS/MS Spectrum Peak List

m/z	z	Abund
133.0839	1	163.56
148.0808		128.1
197.1265		153.79
211.1425		486.46
231.1673		300.92
243.1338		255.4
252.1404		104.14
257.11		113.27
328.1781		106.05
330.1418		104.5

Compound Structure

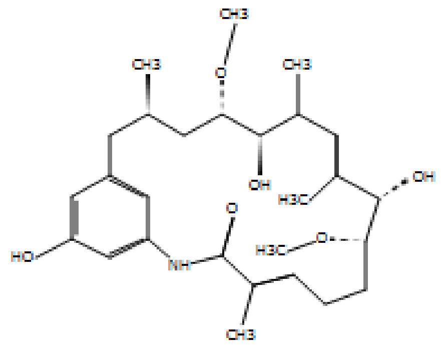

Compound Label	Name	*m/z*	RT	Algorithm	Mass
Cpd 25: L,L-Cyclo(leucylprolyl)	**L,L-Cyclo(leucylprolyl)**	211.1433	5.12	Auto MS/MS	210.1362

MS Spectrum

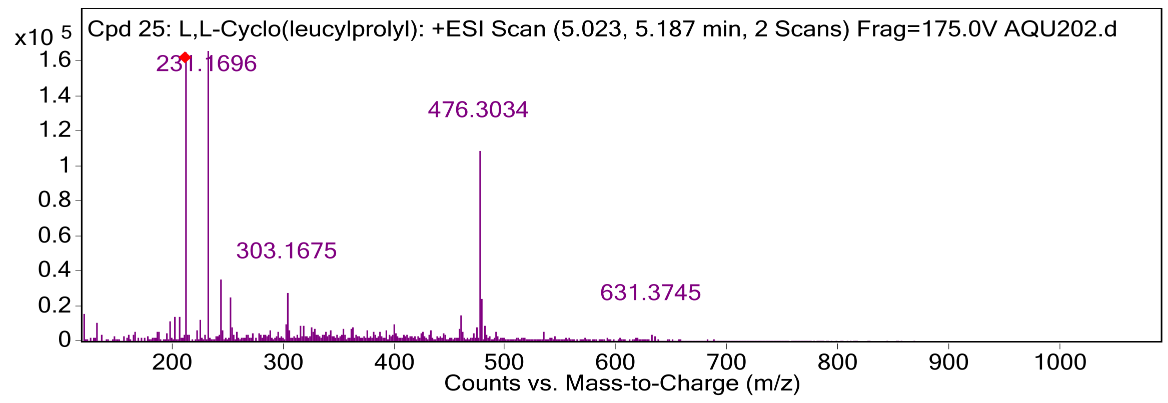

MS Zoomed Spectrum

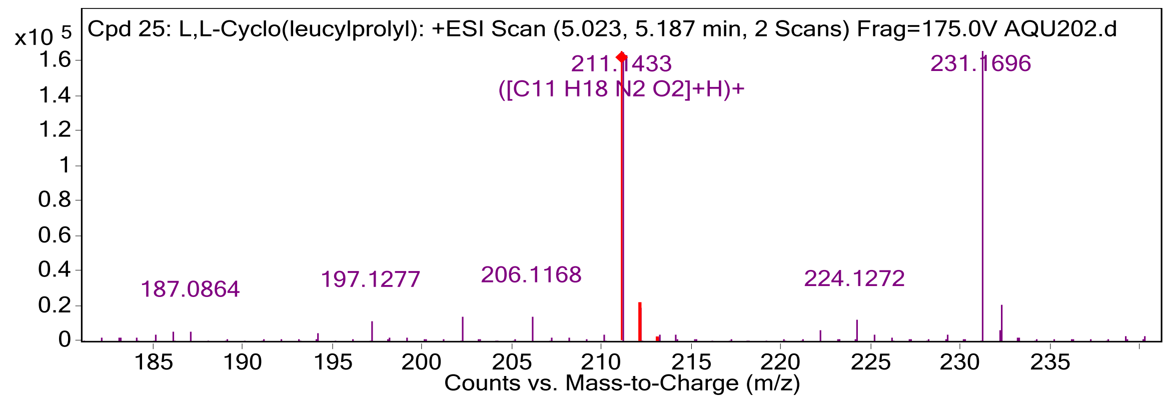

MS Spectrum Peak List

m/z	*Calc m/z*	Diff(ppm)	z	Abund	Formula	Ion
211.1433	211.1441	3.88	1	165461.56	C11 H18 N2 O2	(M+H)+
212.1466	212.1472	2.62	1	20032.68	C11 H18 N2 O2	(M+H)+
213.1542	213.1495	-22.06	1	4142.52	C11 H18 N2 O2	(M+H)+
231.1696			1	174168.13		
232.1728			1	21468.18		
243.1332			1	35853.56		
252.0859			1	25161.53		
303.1675			1	28368.87		
476.3034			1	109221.86		
477.3064			1	25109.26		

MSMS Spectrum

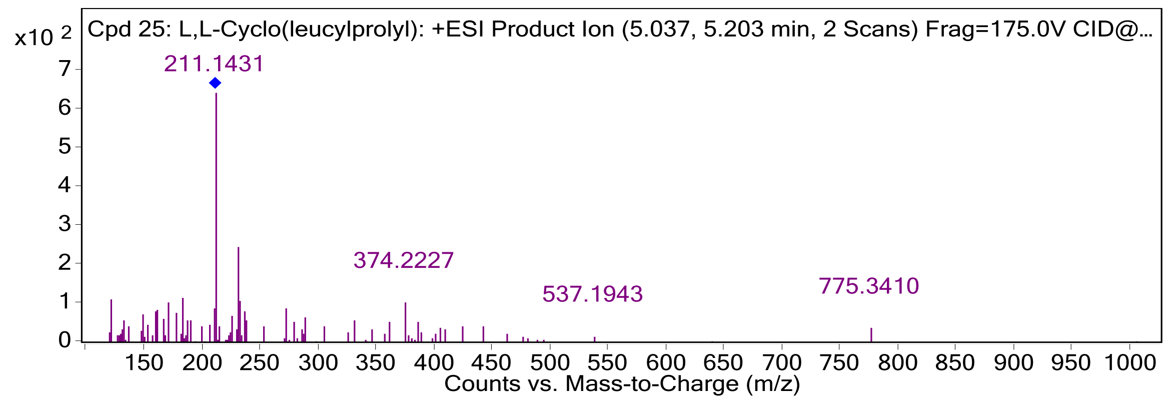

MS/MS Spectrum Peak List

m/z	z	Abund
121.0939		112.51
161.062		85.33
171.1019		103.39
183.1488		115.15
210.1248		89.35
211.1431	1	643.09
231.1704		246.84
232.1734		109.57
271.197		89.15
374.2227		104.21

Compound Structure

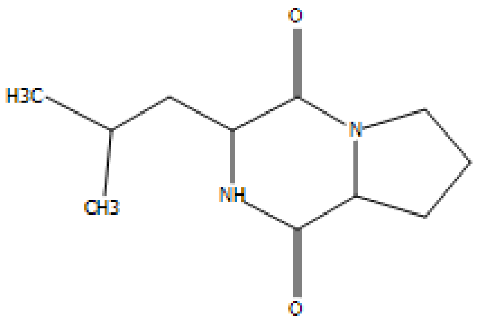

Compound Label	*m/z*	RT	Algorithm
Compound 26	520.3296	5.358	Auto MS/MS

MS Spectrum

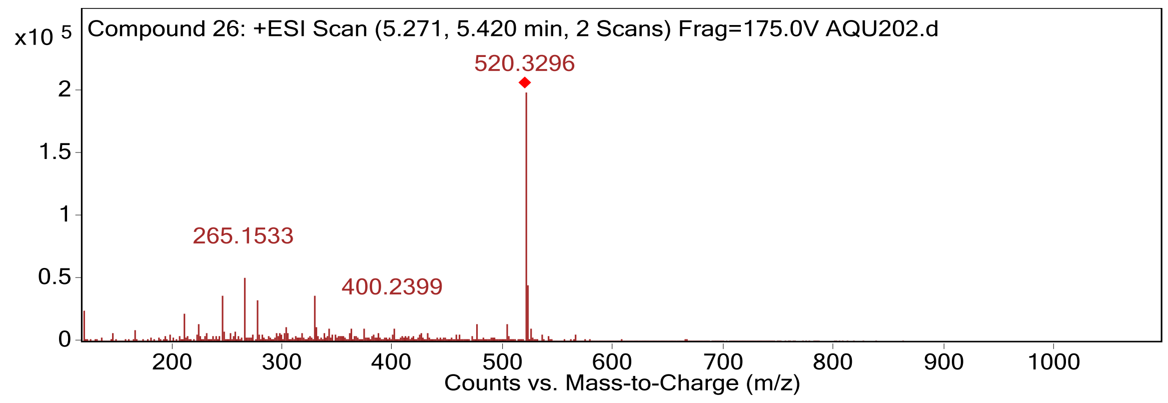

MS Zoomed Spectrum

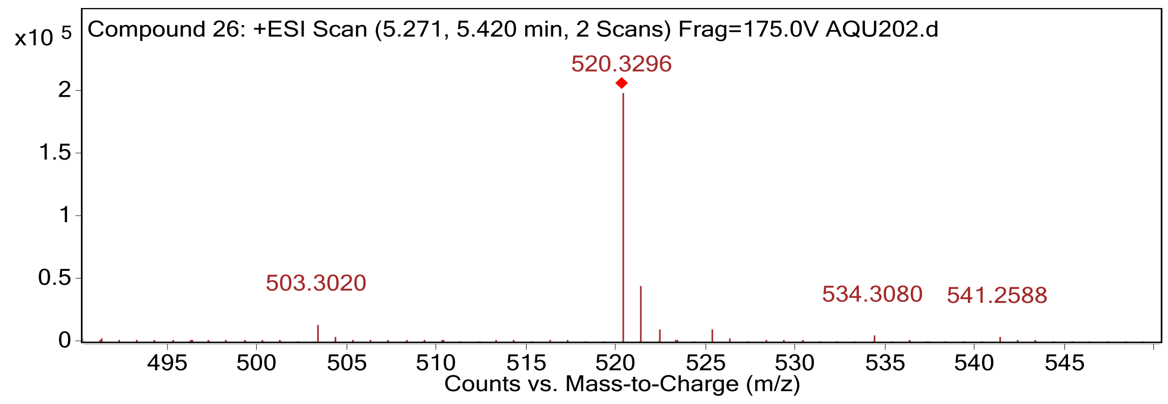

MS Spectrum Peak List

m/z	z	Abund
120.0808		24892.61
211.1431		22046.01
245.1271	1	37226.33
265.1533	1	51424.87
277.117	1	33202.43
328.2215	1	36525.45
520.3296	1	199190.59
521.3328	1	45796.3
522.3348	1	10559.84
523.334	1	2328.7

MSMS Spectrum

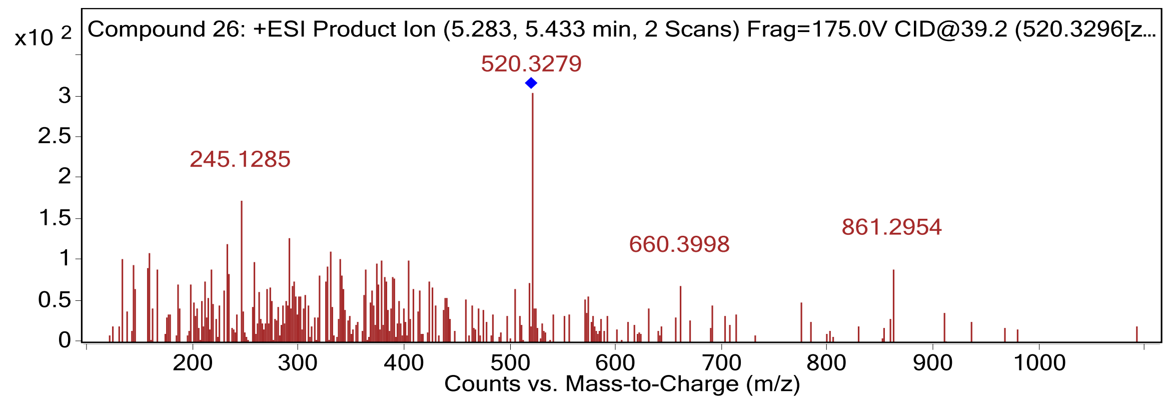

MS/MS Spectrum Peak List

m/z	z	Abund
133.0846		102.61

158.0603		109.44
231.1684		120.73
245.1285		173.47
291.1302		127.19
329.1235		111.52
339.1458		101.8
377.1559		100.45
402.251		100.39
520.3279	1	305.63

Compound Label	*m/z*	RT	Algorithm
Compound 27	564.356	5.579	Auto MS/MS

MS Spectrum

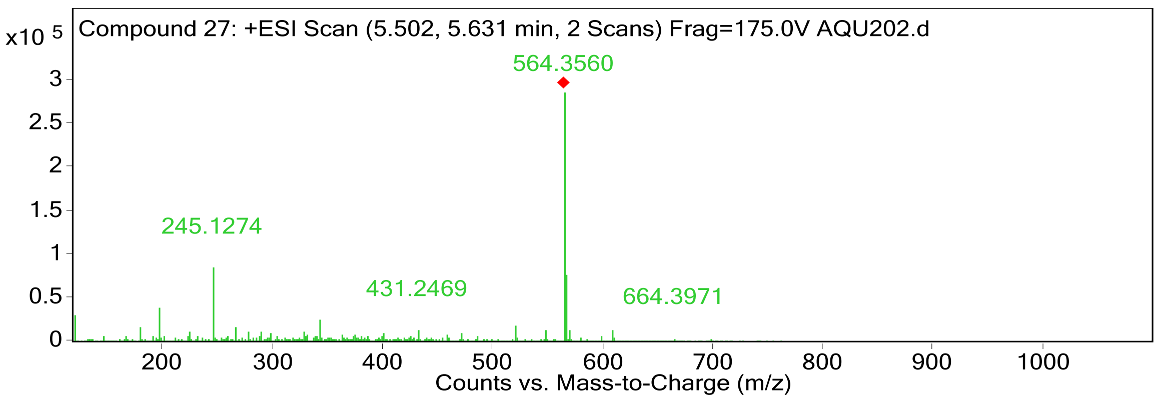

MS Zoomed Spectrum

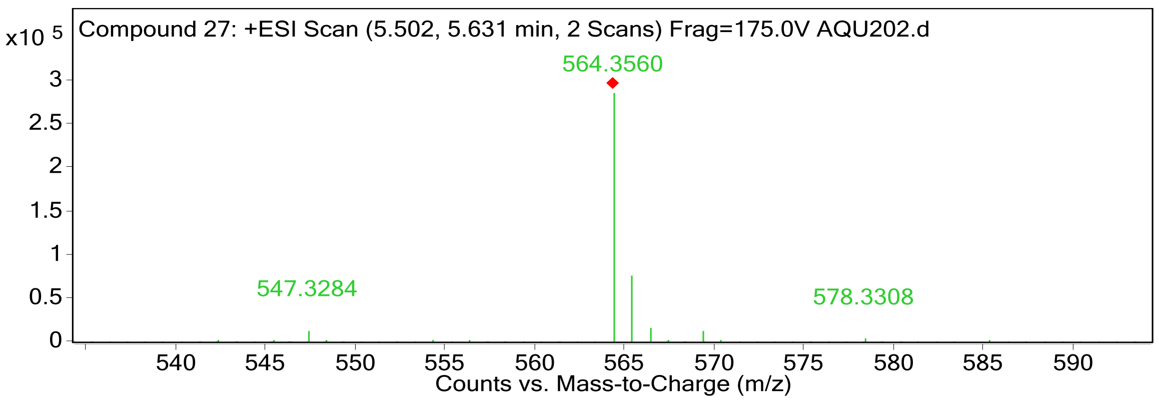

MS Spectrum Peak List

m/z	z	Abund
120.0808		30833.02
197.1169	1	38821.59
245.1274	1	85259.41
265.1533		17383.52
342.2365	1	25638.14
520.3292	1	18083.31
564.356	1	286618.31
565.3589	1	77224.16
566.36	1	17351.35
567.3574	1	3029.19

MSMS Spectrum

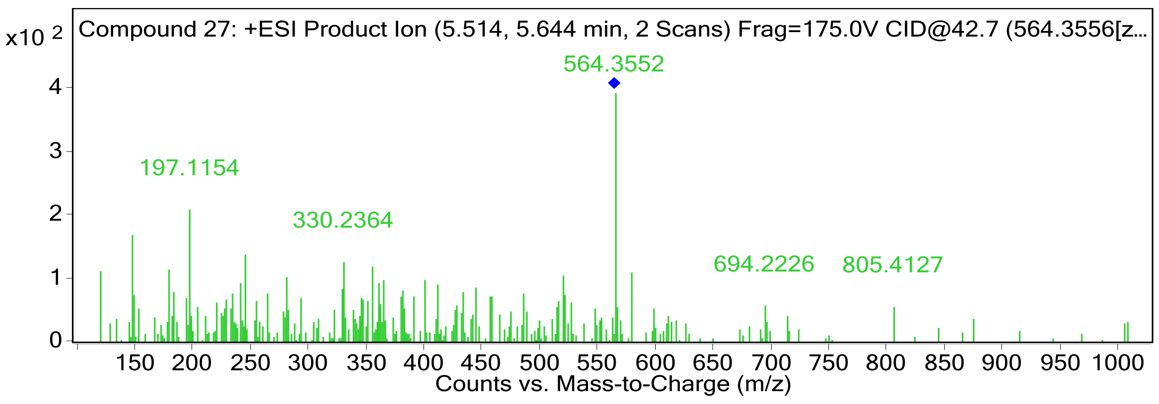

MS/MS Spectrum Peak List

m/z	z	Abund
120.0804		112.6
147.1179		168.45
179.1038		114.74
197.1154	1	210.37
245.1265		137.95
245.1838		130.02
330.2364		127.63
354.1946		119.65
564.3552	1	394.11
578.2553		111.74

Compound Label	*m/z*	RT	Algorithm
Compound 28	608.3821	5.727	Auto MS/MS

MS Spectrum

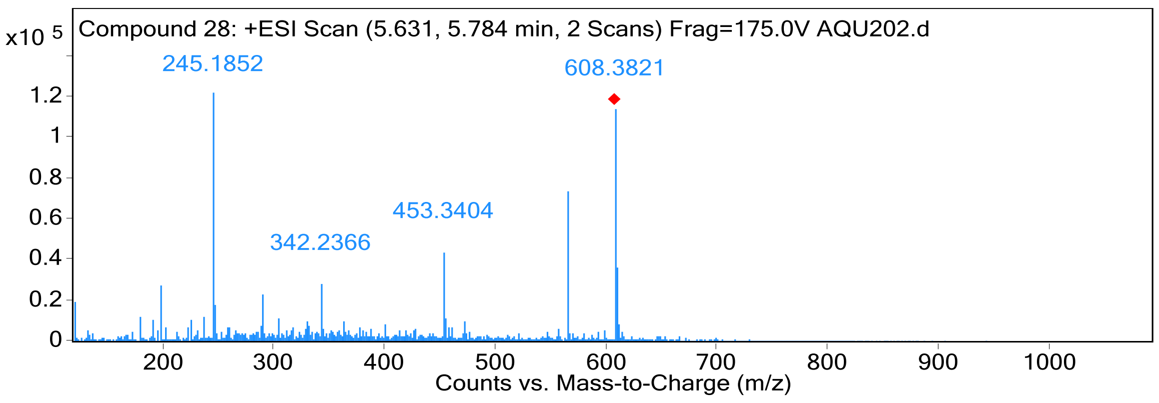

MS Zoomed Spectrum

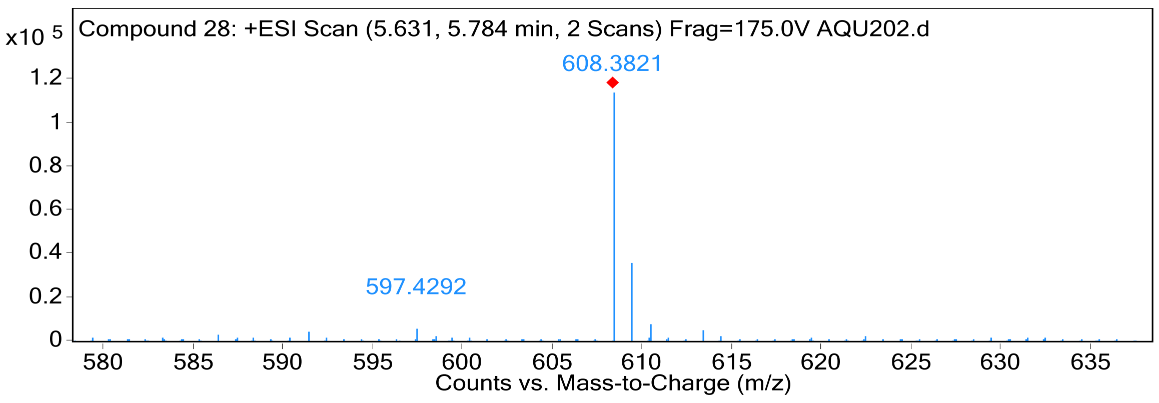

MS Spectrum Peak List

m/z	z	Abund
197.1168		27731.96
245.1272	1	26378.64
245.1852	1	122359.31
342.2366	1	28463.21
453.3404	1	44122.55
564.3558	1	73826.96
608.3821	1	114288.39
609.3847	1	36259.23
610.386	1	8417.18
611.3893	1	2041.94

MSMS Spectrum

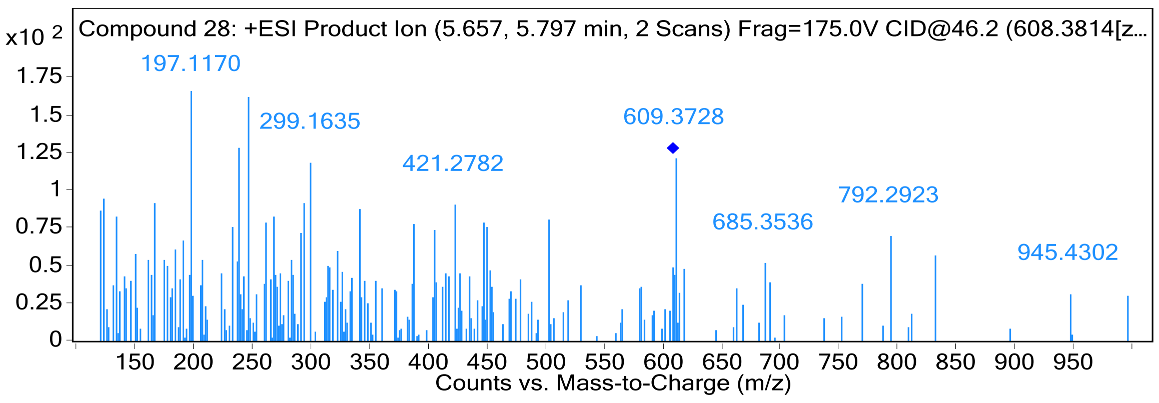

MS/MS Spectrum Peak List

m/z	Abund
123.1149	94.74
166.0473	92.32
197.117	166
238.1685	129.24
245.1814	162.15
246.1834	147.29
293.1446	92.1
299.1635	119.11
421.2782	91.03
609.3728	122.16

Compound Label	Name	*m/z*	RT	Algorithm	Mass
Cpd 29: 3-Oxo-12,18-ursadien-28-oic acid	**3-Oxo-12,18-ursadien-28-oic acid**	453.3404	5.8	Auto MS/MS	452.3331

MS Spectrum

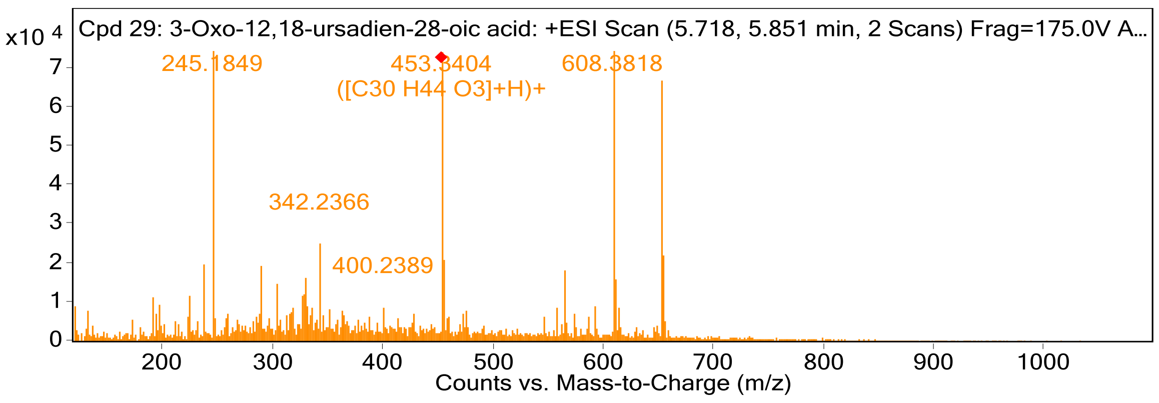

MS Zoomed Spectrum

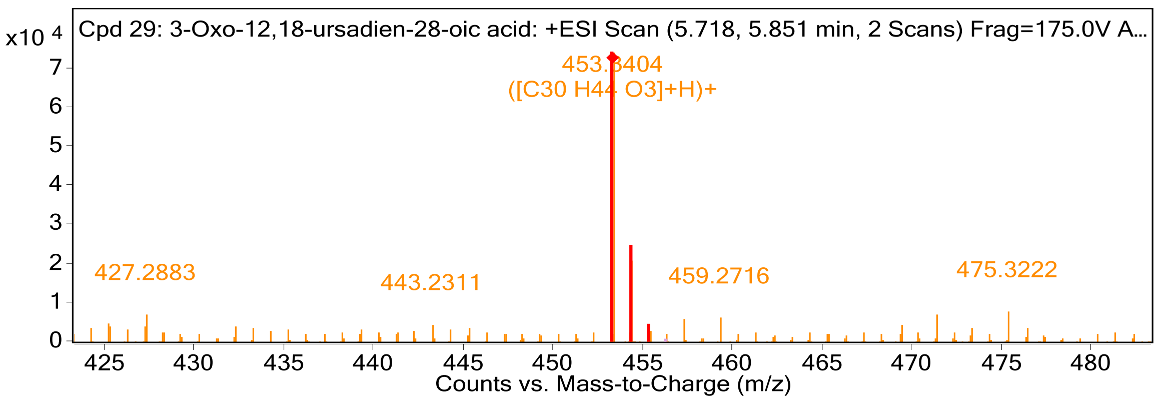

MS Spectrum Peak List

m/z	*Calc m/z*	Diff(ppm)	z	Abund	Formula	Ion
245.1849			1	162152.61		
246.1881			1	22012.54		
342.2366				25149.79		
453.3404	453.3363	-9.1	1	74031.63	C30 H44 O3	(M+H)+
454.3436	454.3397	-8.46	1	20940.54	C30 H44 O3	(M+H)+
455.3463	455.3429	-7.57	1	3199.54	C30 H44 O3	(M+H)+
608.3818			1	240836.44		
609.3847			1	71298.64		
652.4075			1	66762.55		
653.411			1	22087.08		

MSMS Spectrum

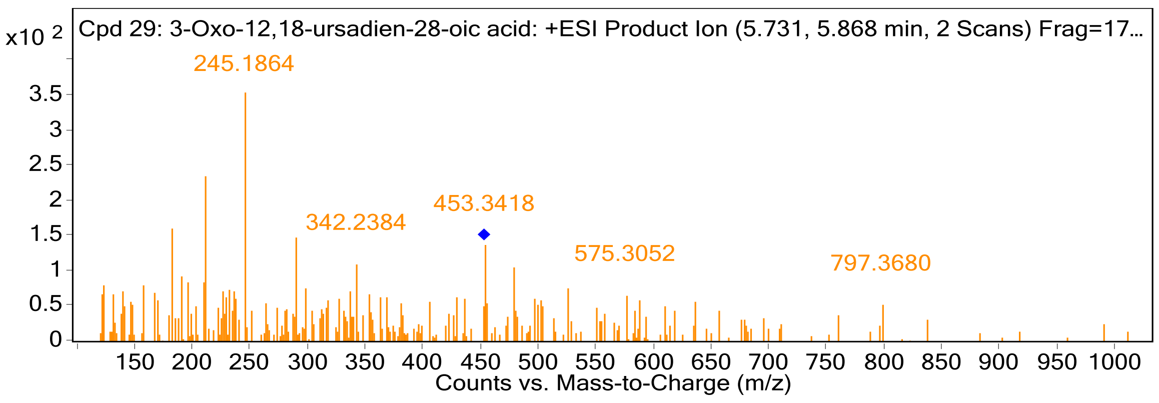

MS/MS Spectrum Peak List

m/z	z	Abund
182.1538		161.65
190.1557		94.04
195.1472		83.86
209.1644		84.15
210.1509	1	235.31
245.1864		355.05
289.1723		149.12
342.2384		110.96
453.3418	1	138.04
478.2236		105.97

Compound Structure

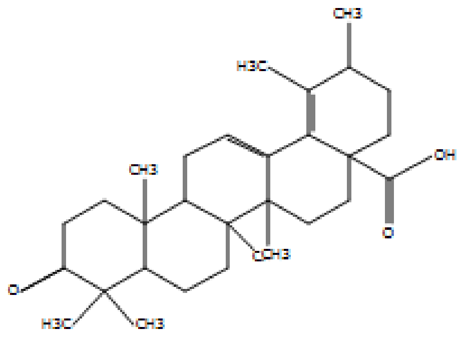

Compound Label	*m/z*	RT	Algorithm
Compound 30	652.4077	5.947	Auto MS/MS

MS Spectrum

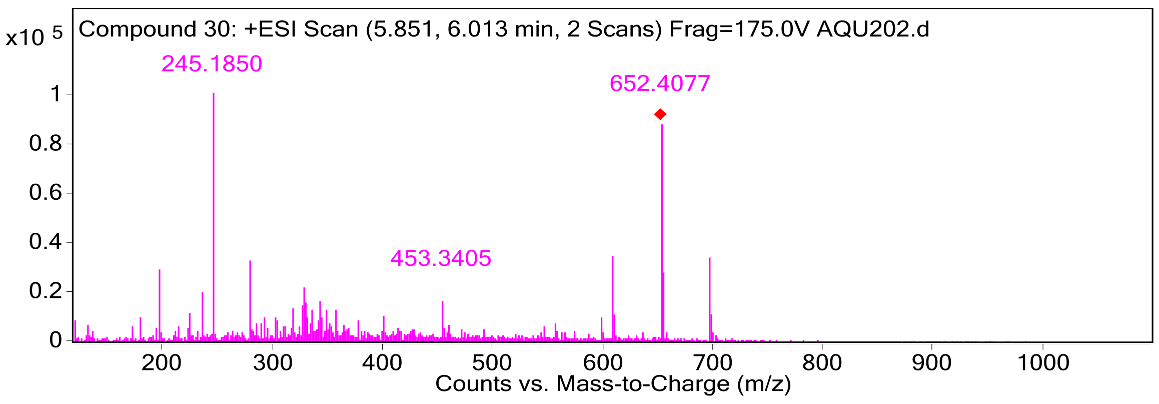

MS Zoomed Spectrum

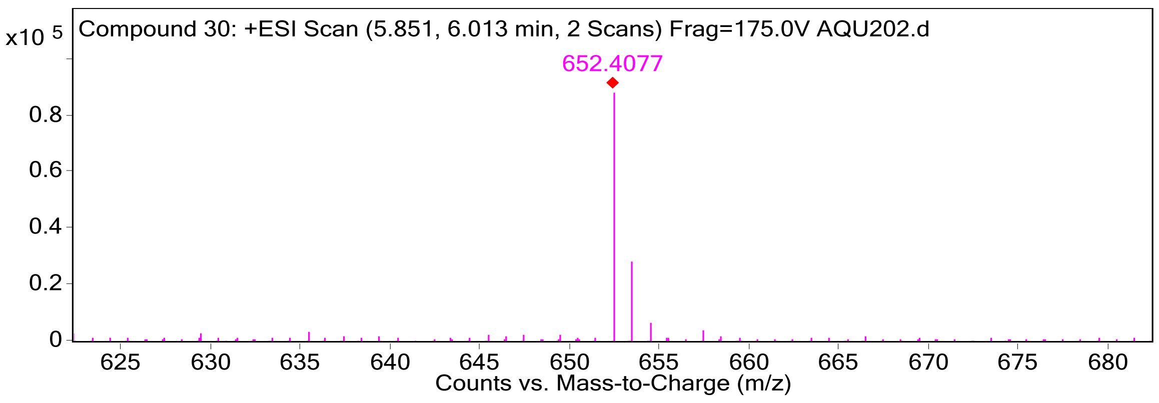

MS Spectrum Peak List

m/z	z	Abund
197.117	1	29716.02
245.185	1	101343.17
279.1688	1	33102.06
328.2211	1	22710.8
608.3809	1	35183.63
652.4077	1	88356.06
653.4111	1	28433.36
654.4099	1	6992.55
655.4133	1	1657.11
696.4333	1	34638.79

MSMS Spectrum

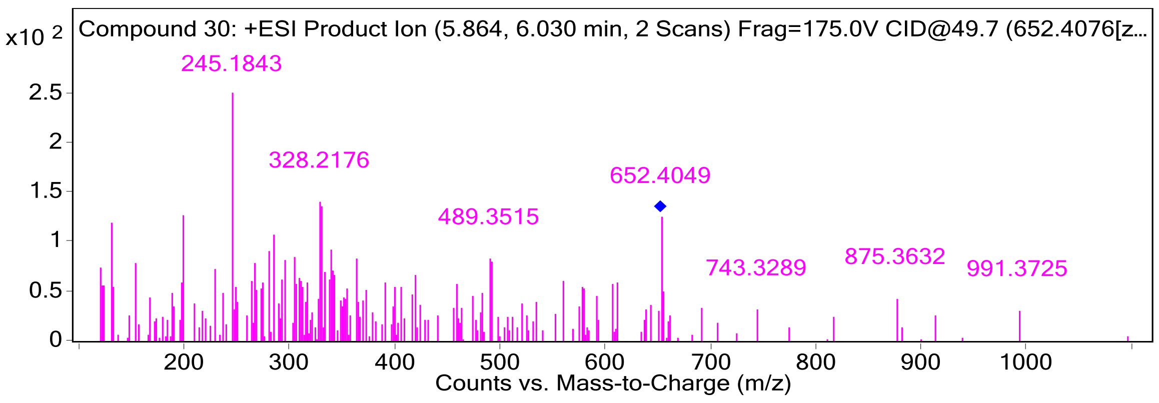

MS/MS Spectrum Peak List

m/z	z	Abund
130.0634		119.74
198.1207		127.55

245.1843	1	251.57
279.1287		90.93
284.137		108.35
303.1871		86.03
328.2176		141.57
329.1642	1	136.34
338.201		92.52
652.4049	1	125.98

Compound Label	Name	*m/z*	RT	Algorithm	Mass
Cpd 31: Mycinamicin IV	**Mycinamicin IV**	696.4335	6.123	Auto MS/MS	695.4262

MS Spectrum

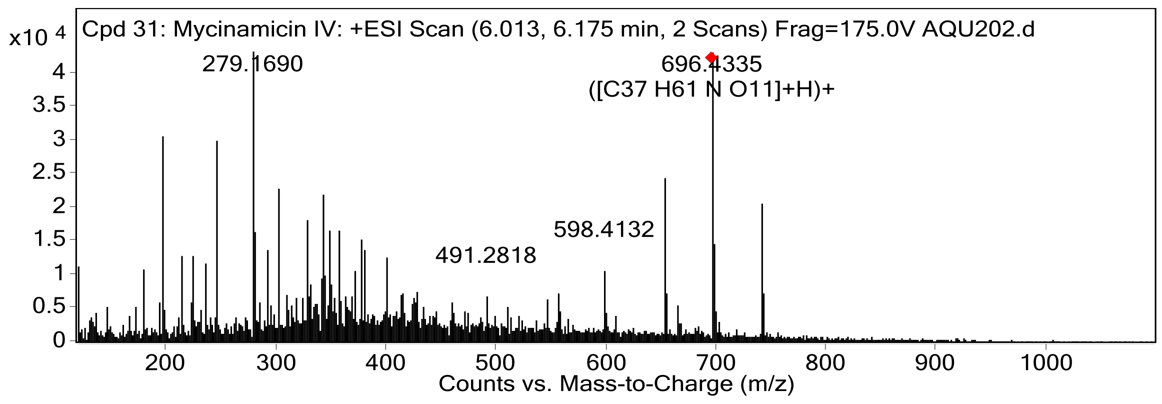

MS Zoomed Spectrum

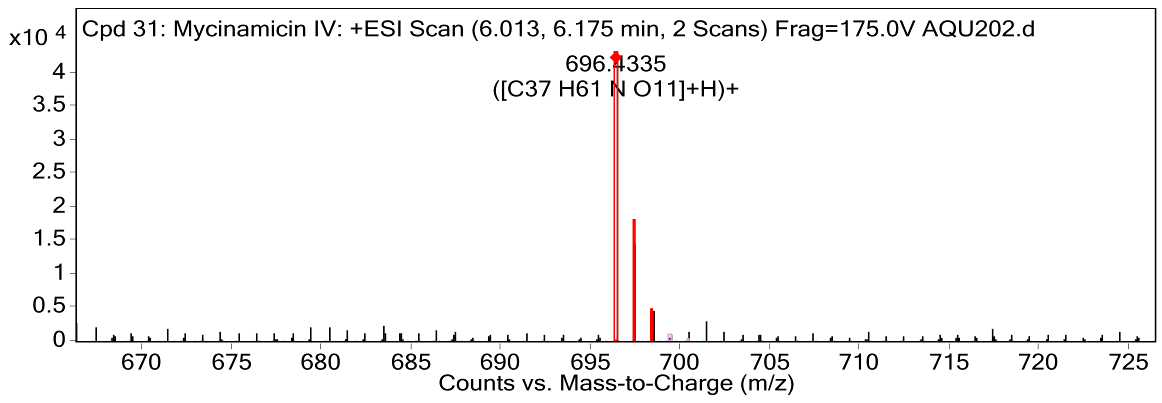

MS Spectrum Peak List

m/z	*Calc m/z*	Diff(ppm)	z	Abund	Formula	Ion
197.1171			1	30690.57		
245.1851				30007.28		
279.169			1	87012.02		
301.2469				22838.02		
342.2368			1	21958.9		
652.4074			1	24466.91		
696.4335	696.4317	-2.57	1	43092.36	C37 H61 N O11	(M+H)+
697.4367	697.4351	-2.25	1	14649.69	C37 H61 N O11	(M+H)+
698.4398	698.4379	-2.73	1	4600.08	C37 H61 N O11	(M+H)+
740.4596			1	20597.18		

MSMS Spectrum

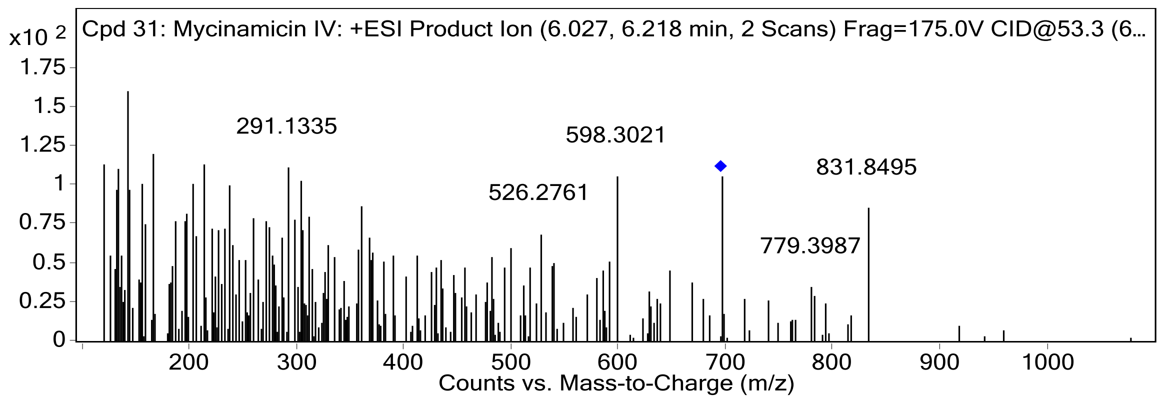

MS/MS Spectrum Peak List

m/z	Abund
120.0808	113.41
133.085	110.98
142.1572	160.01
166.0835	120.49
202.1219	101.55
213.16	113.52
291.1335	111.74
303.19	103.03
598.3021	106.12
696.4322	105.88

Compound Structure

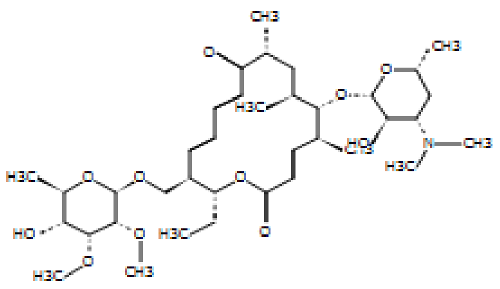

Compound Label	*m/z*	RT	Algorithm
Compound 32	356.2522	6.598	Auto MS/MS

MS Spectrum

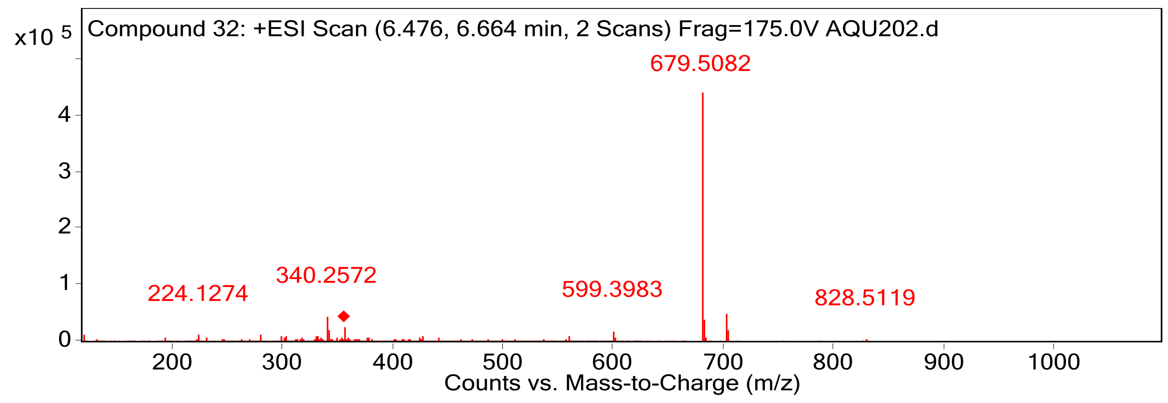

MS Zoomed Spectrum

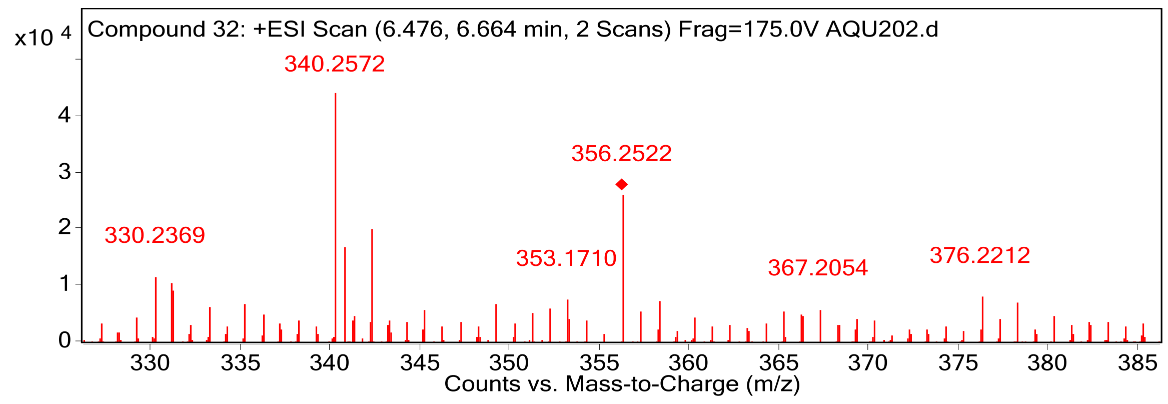

MS Spectrum Peak List

m/z	z	Abund
340.2572	2	44291.49
342.2366	1	20164.79
356.2522	1	26190.97
357.2556	1	5626.12
599.3983	1	19390.65
679.5082	1	442477.44
680.5115	1	178529.75
681.5137	1	38869.54
701.4899	1	51349.05
702.4927	1	20780.25

MSMS Spectrum

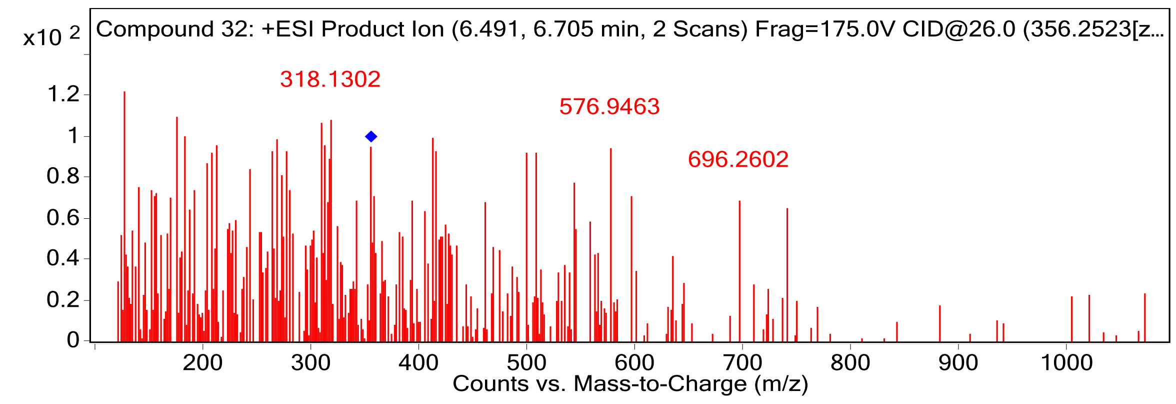

MS/MS Spectrum Peak List

m/z	Abund
126.1255	122.23
175.0787	110.22
183.0813	100.81
211.1801	96.55
268.1261	98.86
309.1844	106.92
311.2103	96.31
318.1302	108.32
355.1575	95.84
411.1726	100

Compound Label	*m/z*	RT	Algorithm
Compound 33	679.5082	6.676	Auto MS/MS

MS Spectrum

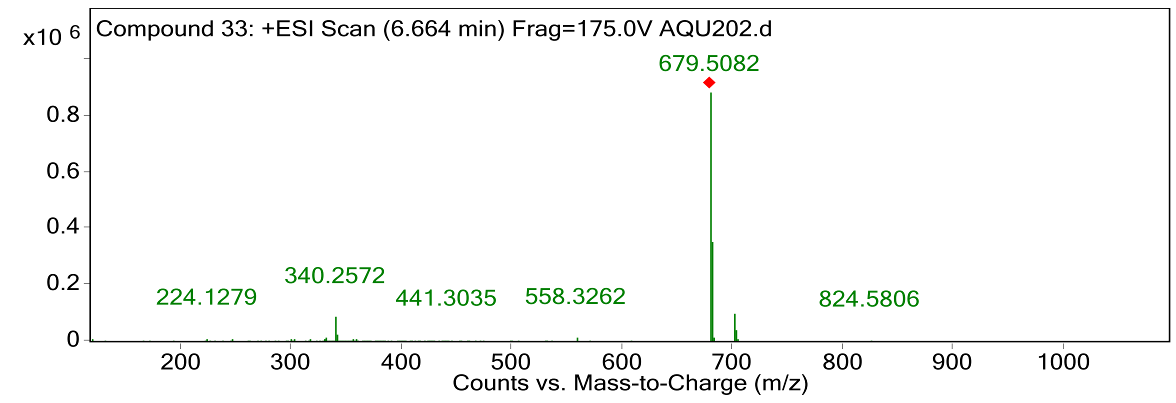

MS Zoomed Spectrum

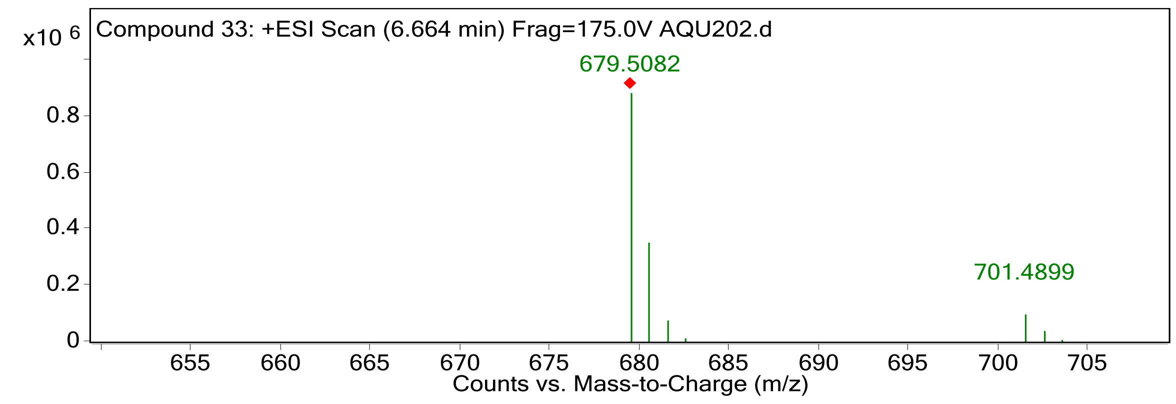

MS Spectrum Peak List

m/z	z	Abund
331.1269		15734.92
340.2572	2	87373.94
340.7596	2	33877.09
342.2365	1	27760.28
679.5082	1	884954.88
680.5115	1	357059.5
681.5137	1	77739.09
682.5161	1	13933.91
701.4899	1	102698.11
702.4927	1	41560.5

MSMS Spectrum

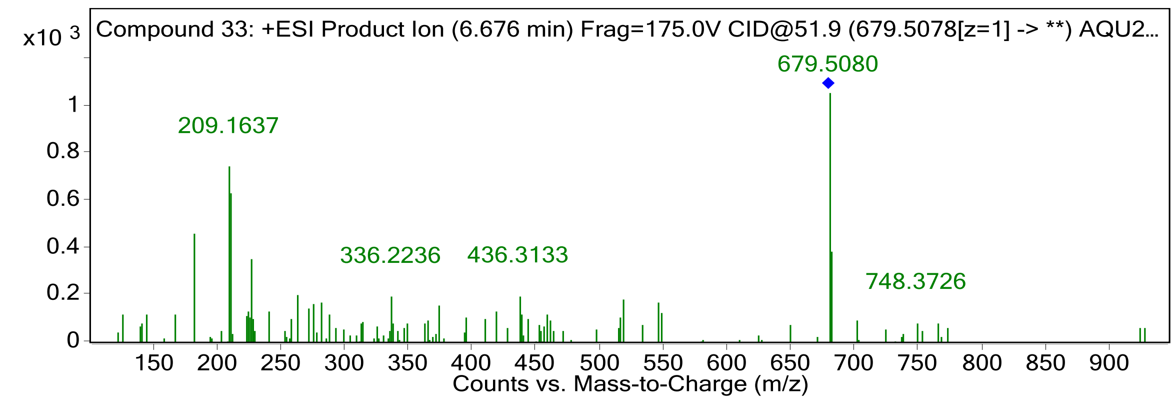

MS/MS Spectrum Peak List

m/z	z	Abund
182.1523		461.01
209.1637		743.14
210.1473	1	630.86
226.1881		350.61
263.1653		203.97
336.2236		193.19
436.3133		195.21
518.1997		184.62
679.508	1	1056.5
680.5057	1	386.4

Compound Label	*m/z*	RT	Algorithm

Compound 34	701.4897	6.729	Auto MS/MS

MS Spectrum

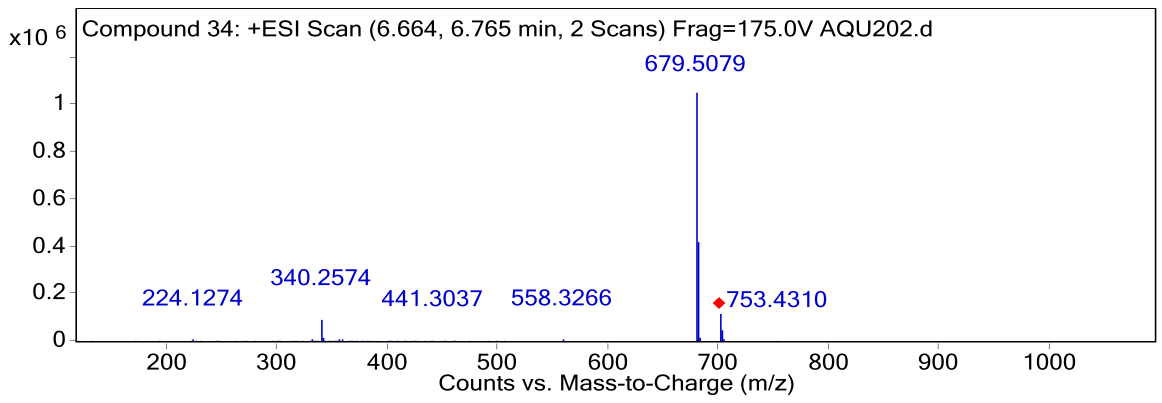

MS Zoomed Spectrum

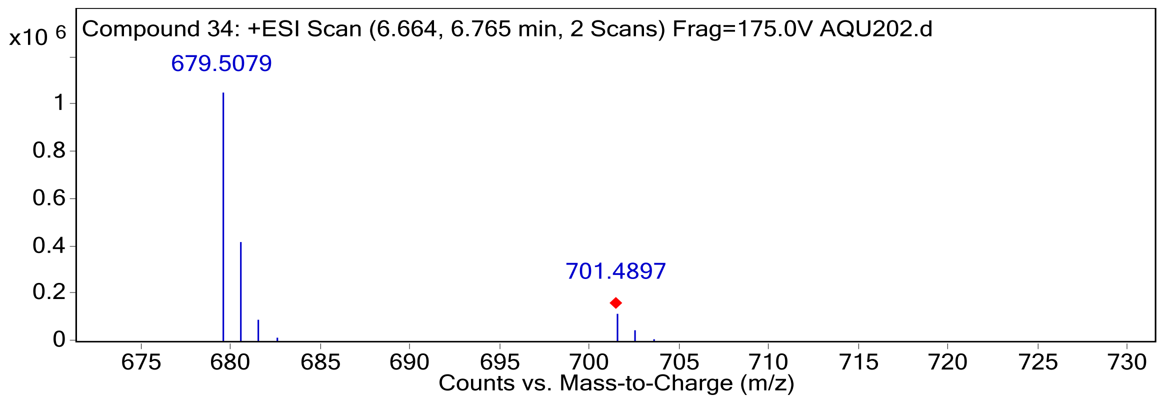

MS Spectrum Peak List

m/z	z	Abund
340.2574	2	96172.16
340.7595	2	37958.54
342.2364	1	19215.14
679.5079	1	1053616.25
680.5115	1	423945.88
681.5139	1	92069.58
701.4897	1	122826.05
702.4925	1	49504.04
703.4946	1	11725.67
704.4991	1	2832.48

MSMS Spectrum

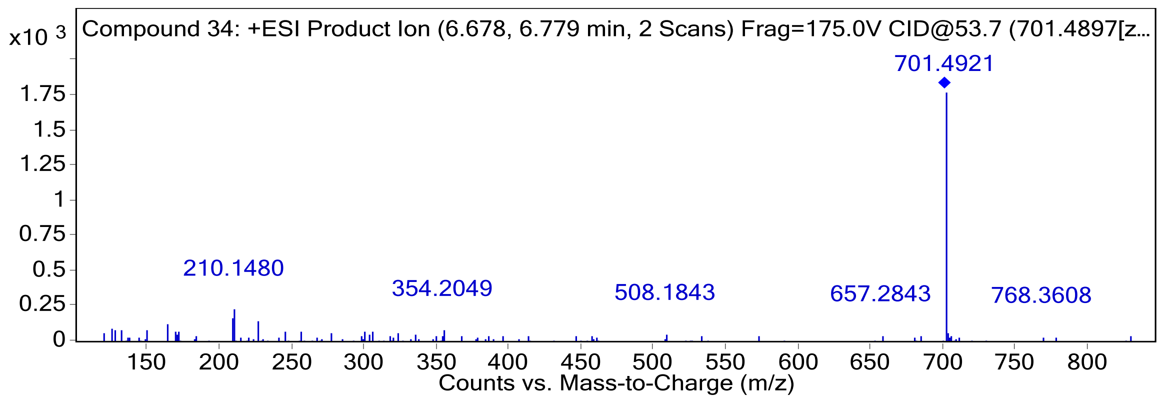

MS/MS Spectrum Peak List

m/z	z	Abund
125.0929		93.5
127.0868		89.72
132.1014		86.92
149.1308		88.39
164.1141		124.27
209.163		174.48
210.148		228.18
226.1921		146.76
354.2049		84
701.4921	1	1772.58

Compound Label	Name	*m/z*	RT	Algorithm	Mass
Cpd 35: PE(18:2(9Z,12Z)/15:0)	**PE(18:2(9Z,12Z)/15:0)**	701.4897	6.745	Auto MS/MS	700.4824

MS Spectrum

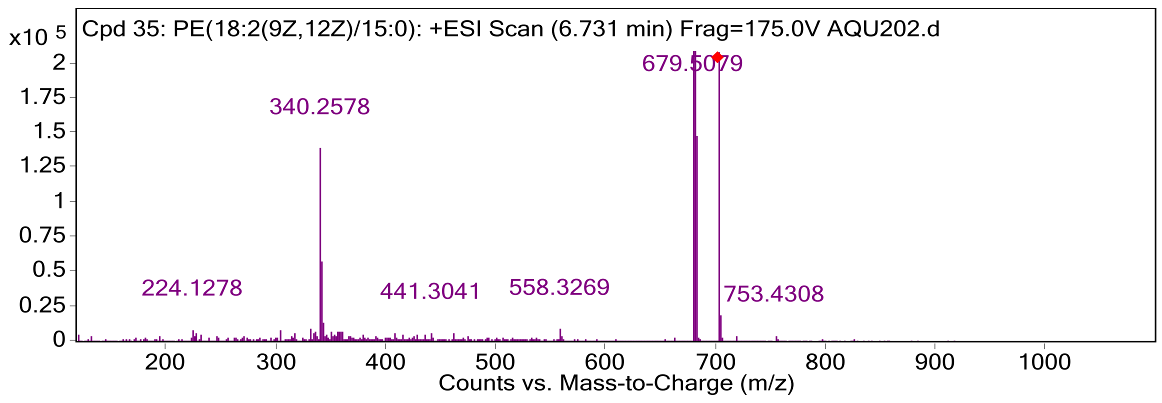

MS Zoomed Spectrum

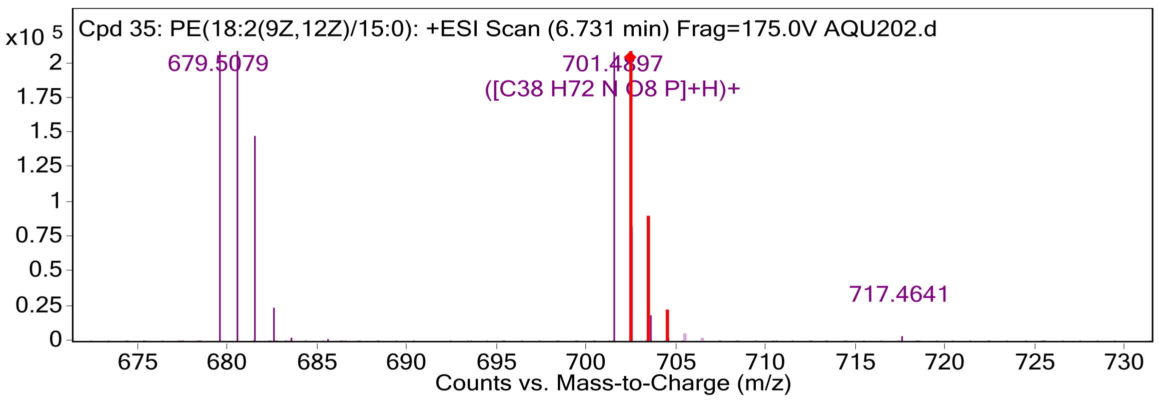

MS Spectrum Peak List

m/z	*Calc m/z*	Diff(ppm)	z	Abund	Formula	Ion
340.2578			2	139469.31		
340.7593			2	58451.92		
679.5079			1	1572654.75		
680.5118			1	648589.44		
681.5143			1	147831.56		
682.5167			1	24221.3		
701.4897	702.5068	1450.01	1	208143.33	C38 H72 N O8 P	(M+H)+
702.493	703.5102	1447.95	1	82347.21	C38 H72 N O8 P	(M+H)+
703.4963	704.5132	1445.42	1	19346.2	C38 H72 N O8 P	(M+H)+
704.4984	705.516	1444.43	1	3273.26	C38 H72 N O8 P	(M+H)+

MSMS Spectrum

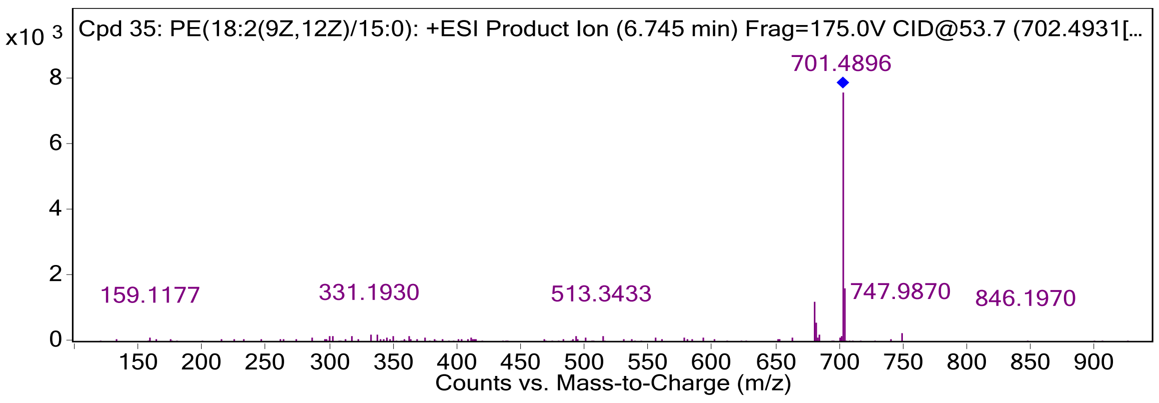

MS/MS Spectrum Peak List

m/z	z	Abund
302.1539		194.84
331.193		237.51
337.1963		236.42
361.1417		197.96
679.5067	1	1224.65
680.5095	1	605.14
683.4742		214.14
701.4896	1	7601.46
702.4903	1	1624.13
747.987		265.38

Compound Structure

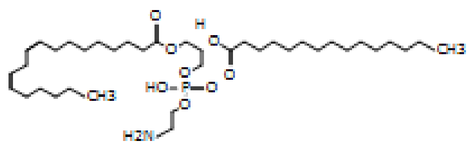

**Compound Label**	***m/z***	**RT**	**Algorithm**
Compound 36	679.5078	6.777	Auto MS/MS

MS Spectrum

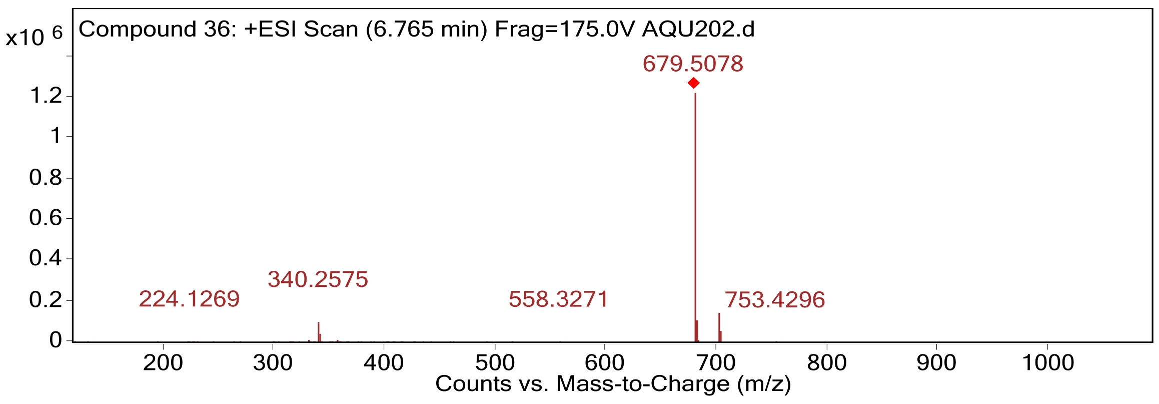

MS Zoomed Spectrum

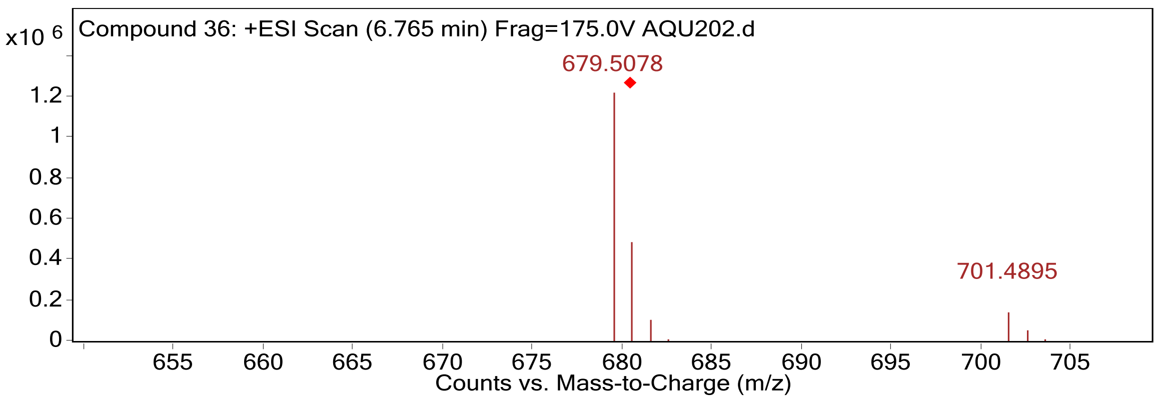

MS Spectrum Peak List

m/z	z	Abund
331.1866	1	14621.6
340.2575	2	104970.37

340.7595	2	42040
341.2594	2	13175.31
679.5078	1	1222277.5
680.5114	1	490832.28
681.514	1	106400.06
682.5165	1	17643.04
701.4895	1	142954
702.4924	1	57447.57

MSMS Spectrum

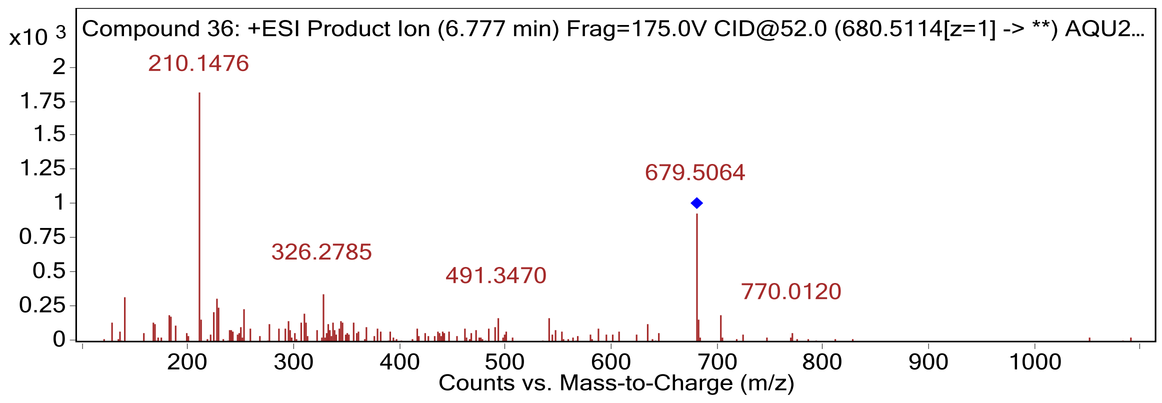

MS/MS Spectrum Peak List

m/z	z	Abund
140.0683		332.14
209.1637		1334.35
210.1476	1	1823.83
223.1341		222.31
226.1923		315.8
227.1727		249.75
251.1718		239.9
308.2631		208.21
326.2785	1	352.36
679.5064	1	934.36

Compound Label	*m/z*	RT	Algorithm
Compound 37	679.5074	7.054	Auto MS/MS

MS Spectrum

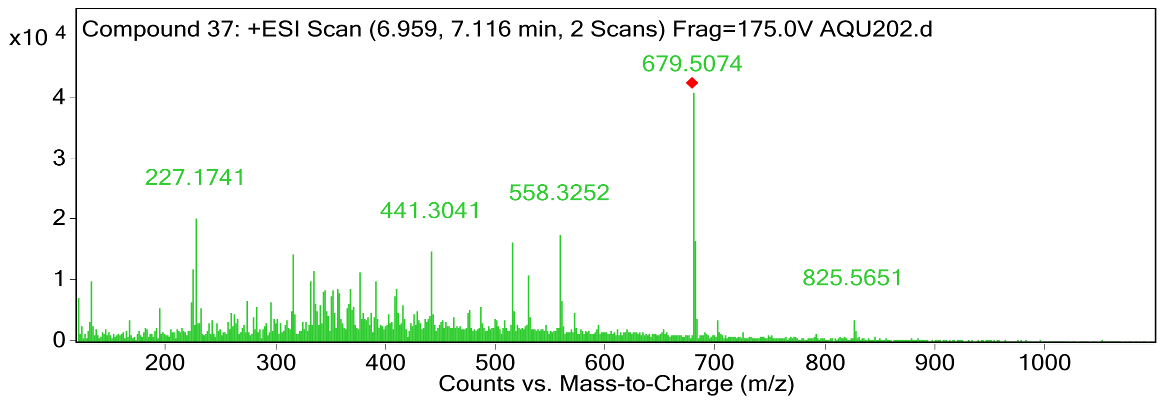

MS Zoomed Spectrum

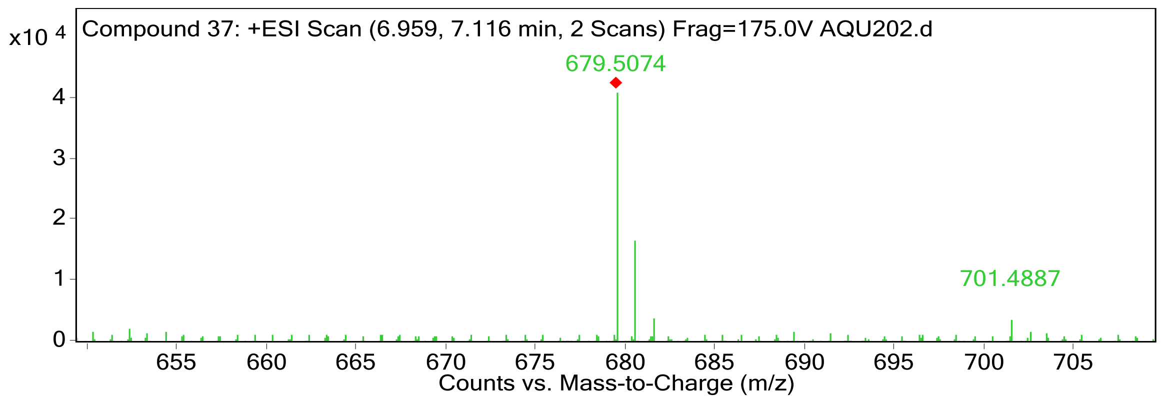

MS Spectrum Peak List

m/z	z	Abund
224.1272		12042.23
227.1741		20312.31
315.2622		14533.03
441.3041	1	14839.41
514.2816	1	16389.7
558.3252	1	17767.84
679.5074	1	41012.16
680.5106	1	16725.4
681.5127	1	3806.71
682.5195	1	571.79

MSMS Spectrum

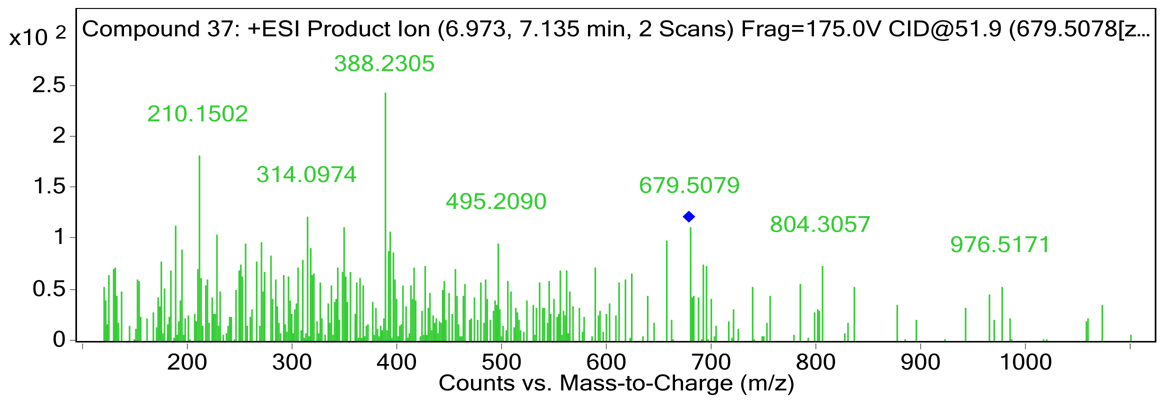

MS/MS Spectrum Peak List

m/z	Abund
188.0735	113.79
210.1502	182.6
228.1596	104.68
270.1417	97.67
314.0974	123.1
349.243	112.94

388.2305	244.05
393.1839	108.5
656.3167	98.57
679.5079	112.52

Compound Label	*m/z*	RT	Algorithm
Compound 38	558.3249	7.174	Auto MS/MS

MS Spectrum

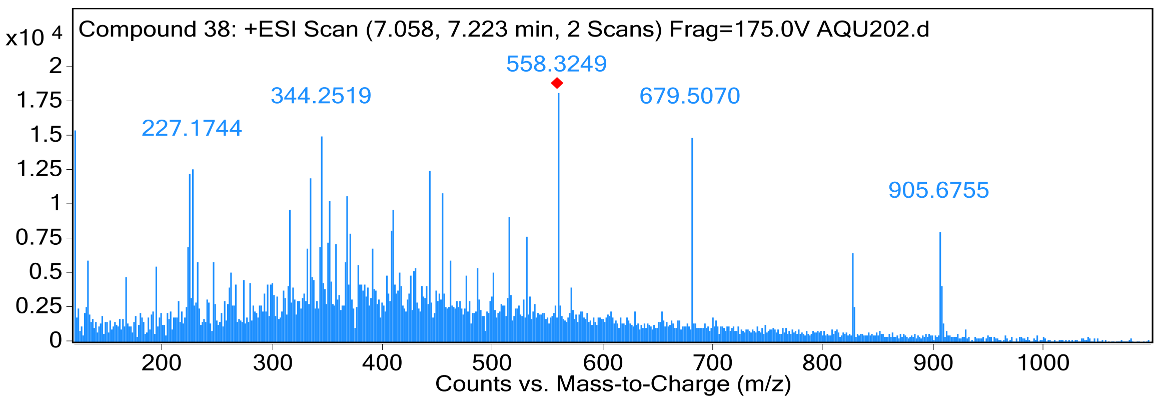

MS Zoomed Spectrum

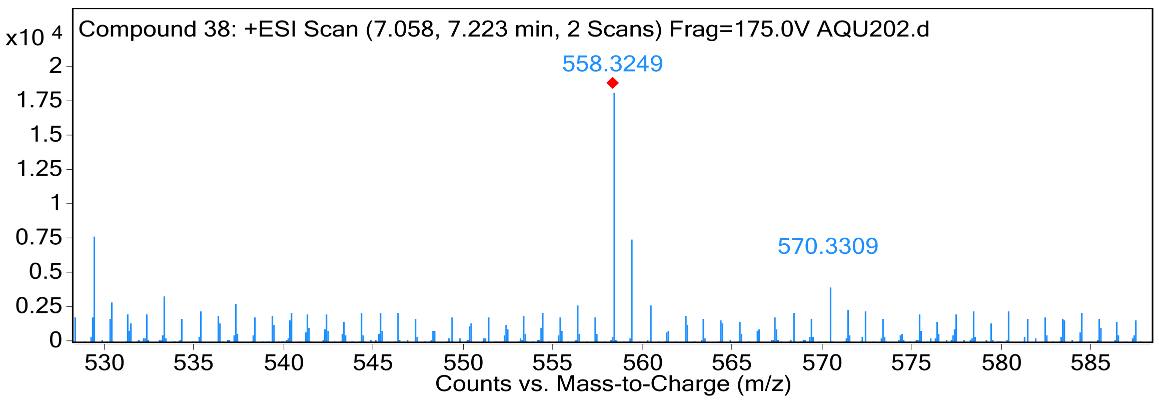

MS Spectrum Peak List

m/z	z	Abund
120.0807		15486.62
224.1274		12267.21
227.1744		12619.06
344.2519		14989.67
441.304	1	12526.97
558.3249	1	18219.63
559.3256	1	7484.94
560.3182	1	2709.94
561.335	1	728.45
679.507	1	14974.39

MSMS Spectrum

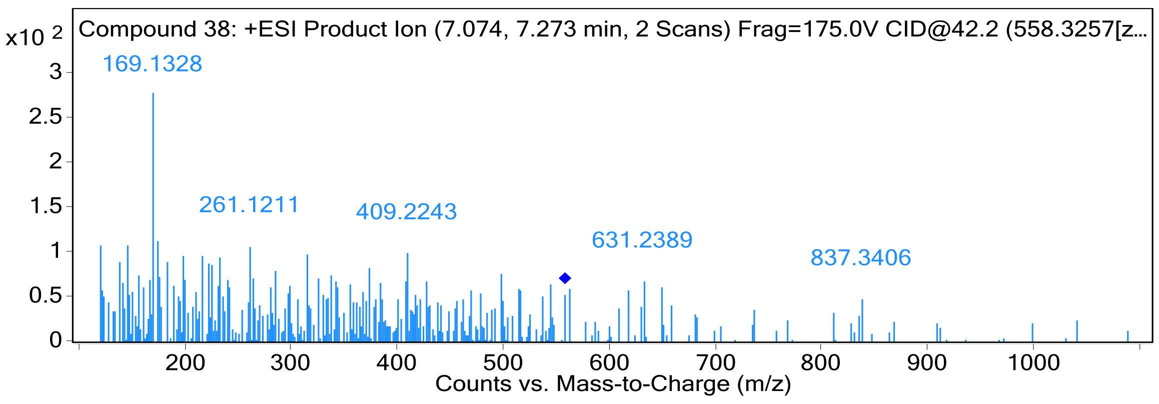

MS/MS Spectrum Peak List

m/z	Abund
120.0797	108.85
145.0613	107.73
169.1328	279.54
173.0934	113.09
197.126	96.41
215.1016	97.53
232.0715	95.59
261.1211	107.7
314.1153	99.09
409.2243	99.77

Compound Label	*m/z*	RT	Algorithm
Compound 39	453.3406	7.325	Auto MS/MS

MS Spectrum

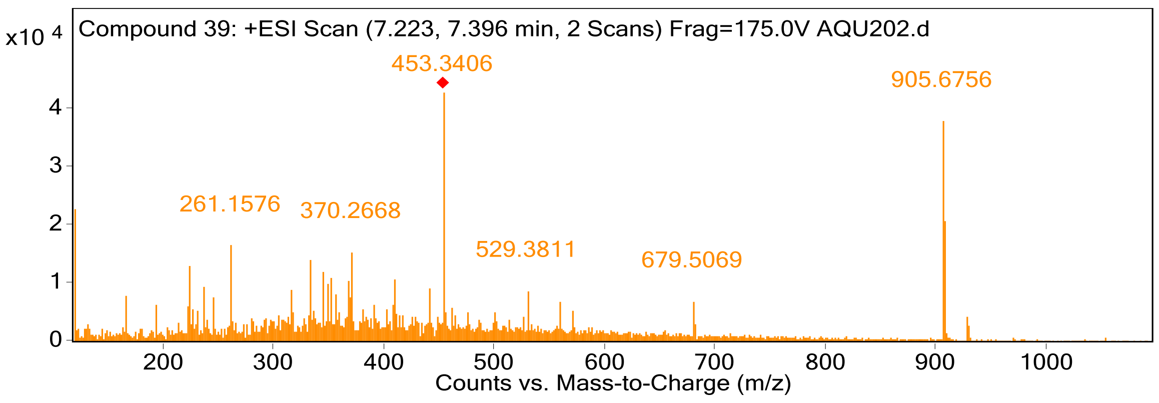

MS Zoomed Spectrum

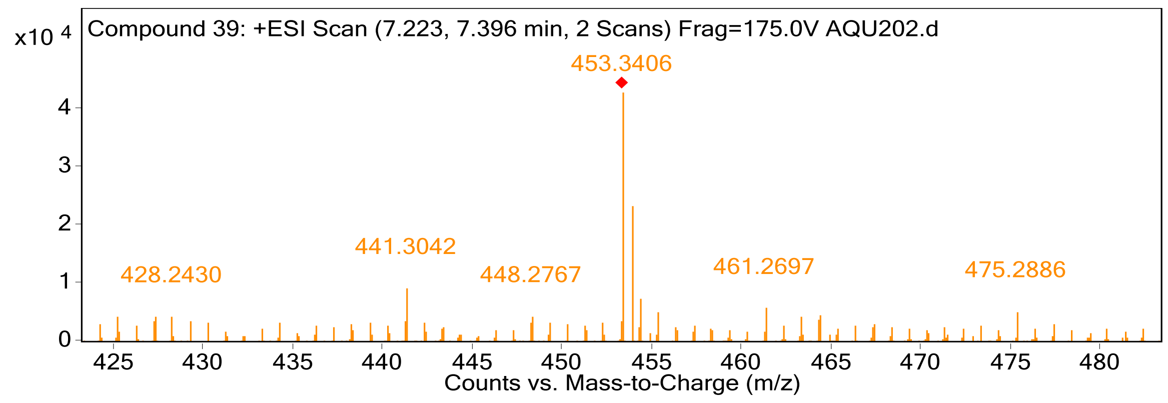

MS Spectrum Peak List

m/z	z	Abund
120.0808		22838.67
261.1576		16612.32
333.204	1	14238.6
370.2668		15479.5
453.3406	2	42955.77
453.8422	2	23398.47
454.3437	2	7464.08
454.847	2	1574.19
905.6756	1	38082.68
906.6783	1	20749.48

MSMS Spectrum

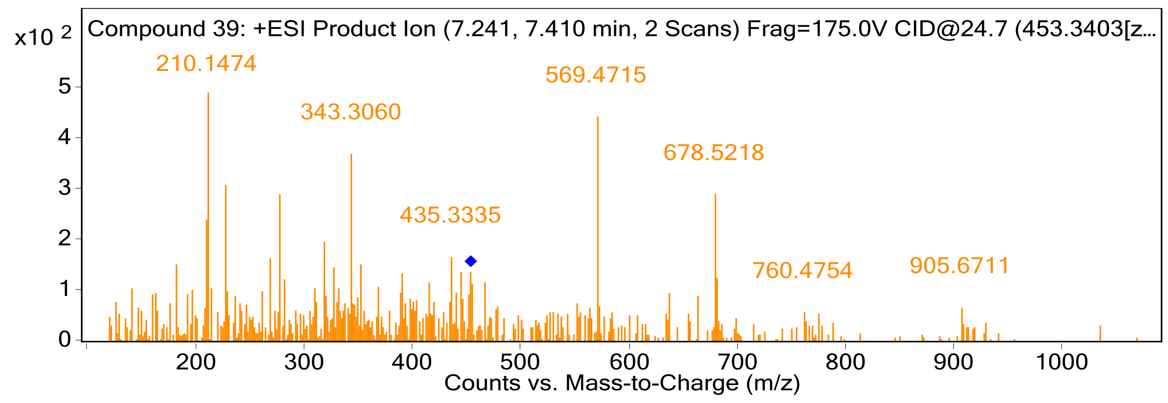

MS/MS Spectrum Peak List

m/z	z	Abund
209.1648		240.96
210.1474	1	491.65
226.1919		280.15
227.178		309.11
276.7257		290.26
318.2122		198.23
343.306	1	371.7
569.4715		444.7
570.4748		330.39
678.5218	1	291.34

Compound Label	*m/z*	RT	Algorithm
Compound 40	453.3412	7.332	Auto MS/MS

MS Spectrum

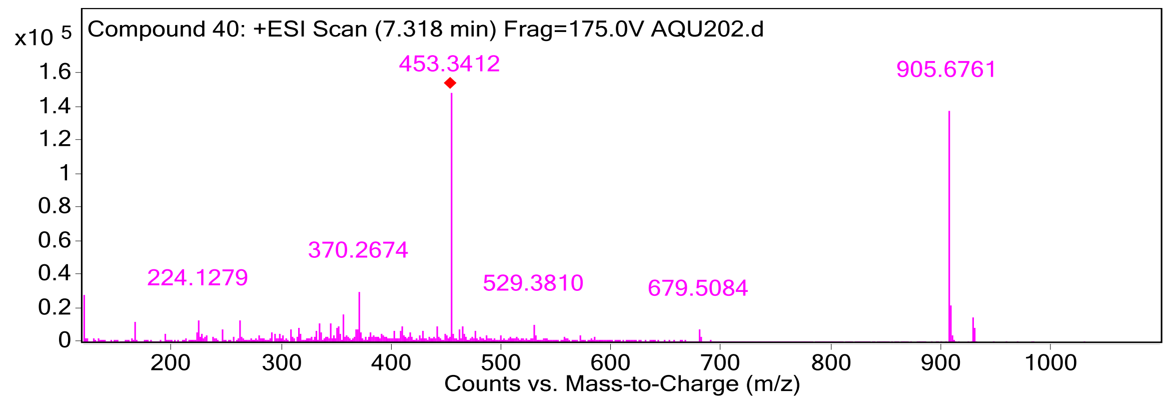

MS Zoomed Spectrum

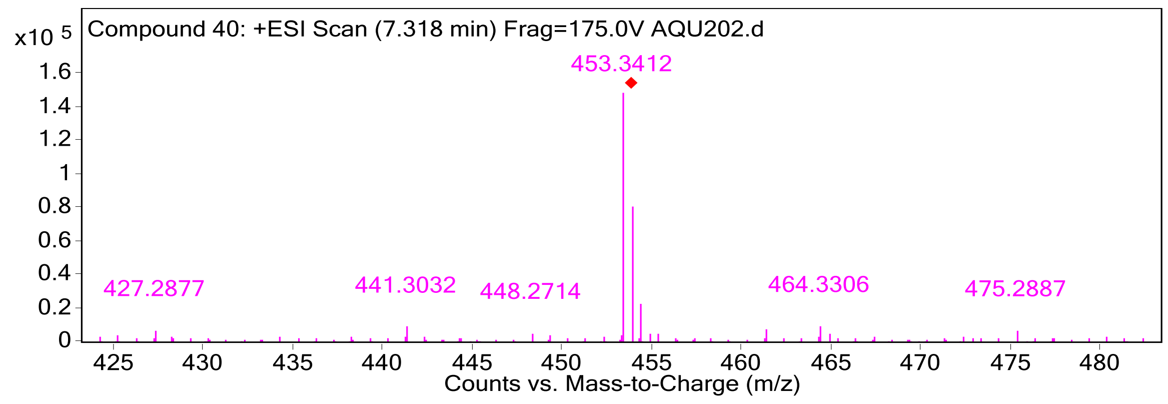

MS Spectrum Peak List

m/z	z	Abund
120.0807		28588.27
356.2524	1	16655.15
370.2674	1	30298.32
453.3412	2	148720.3
453.8424	2	81225.53
454.3436	2	23143.03
454.8454	2	5456.22
905.6761	1	138097.98
906.6795	1	75694.02
907.6819	1	22617.06

MSMS Spectrum

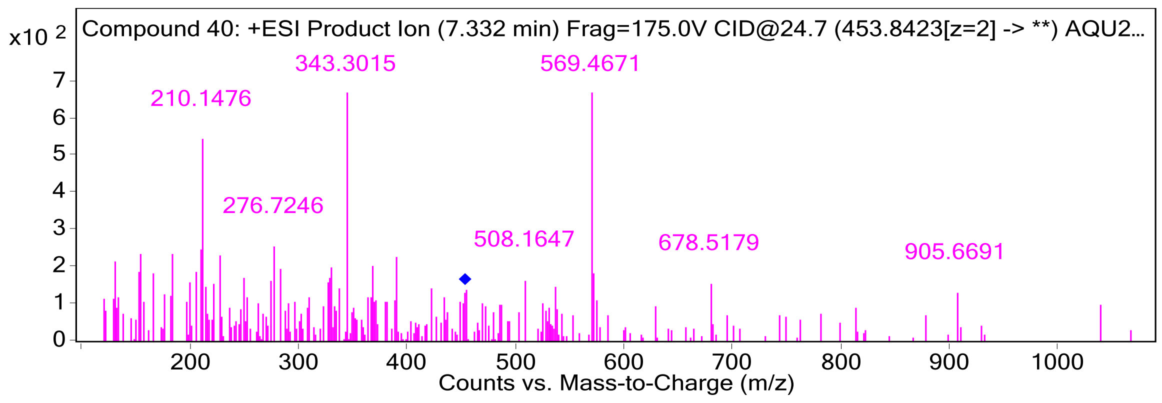

MS/MS Spectrum Peak List

m/z	z	Abund
130.0321		217.98
154.1205		235.01
182.1518		237.31
209.1201		250.06
210.1476		544.16
226.1894		232.24
276.7246	2	255.29
343.3015		668.61
389.8086		228.01
569.4671	1	671.95

Compound Label	Name	*m/z*	RT	Algorithm	Mass
Cpd 41: Dihydrocapsaicin	**Dihydrocapsaicin**	308.2202	7.489	Auto MS/MS	307.2126

MS Spectrum

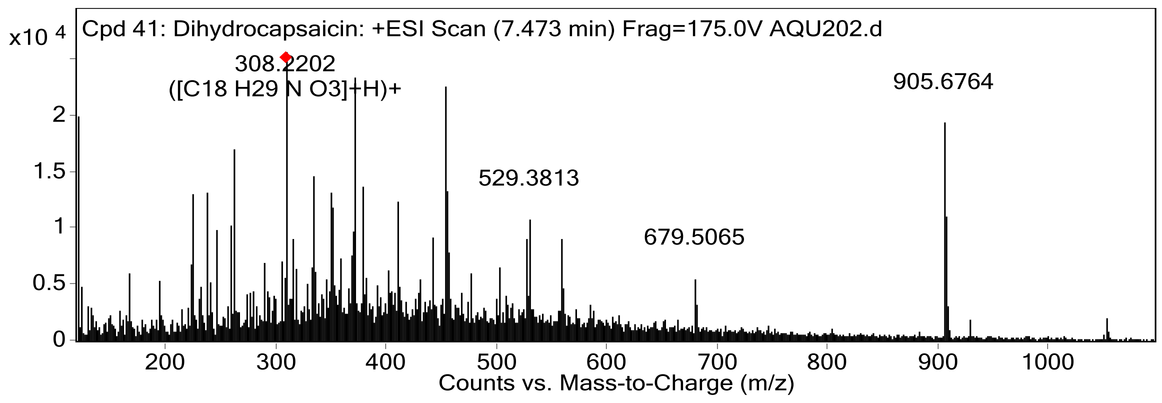

MS Zoomed Spectrum

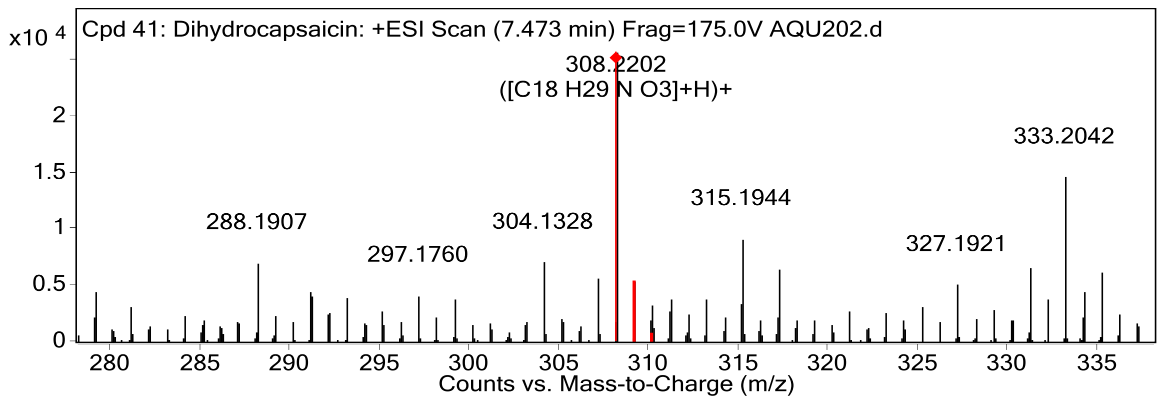

MS Spectrum Peak List

m/z	*Calc m/z*	Diff(ppm)	z	Abund	Formula	Ion
120.0809				19950.26		
261.1586				17007.47		
308.2202	308.222	5.99	1	25646.56	C18 H29 N O3	(M+H)+
309.2215	309.2253	12.37	1	5427.15	C18 H29 N O3	(M+H)+
310.2261	310.228	6.24	1	1326.05	C18 H29 N O3	(M+H)+
333.2042			1	14634.19		
370.2677			1	23433.42		
378.2374				13757.17		
453.3407			2	22557.96		
905.6764			1	19417.2		

MSMS Spectrum

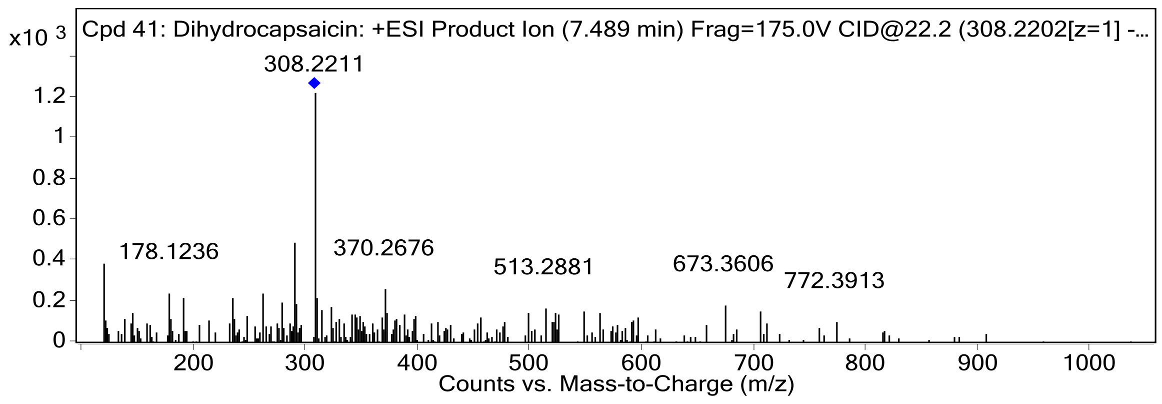

MS/MS Spectrum Peak List

m/z	z	Abund
120.0809		390.55
178.1236		243.17
191.1049		216.78
234.1116		216.39
261.1597		238.03
278.913		195.78
290.2071	1	491.13
308.2211	1	1224.72
309.228	1	220.83
370.2676		260.64

Compound Structure

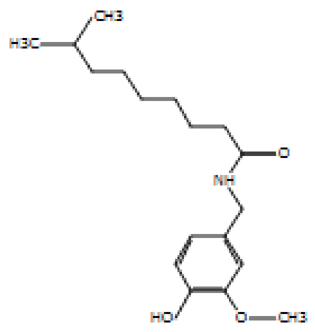

Compound Label	m/z	RT	Algorithm
Compound 42	566.424	7.717	Auto MS/MS

MS Spectrum

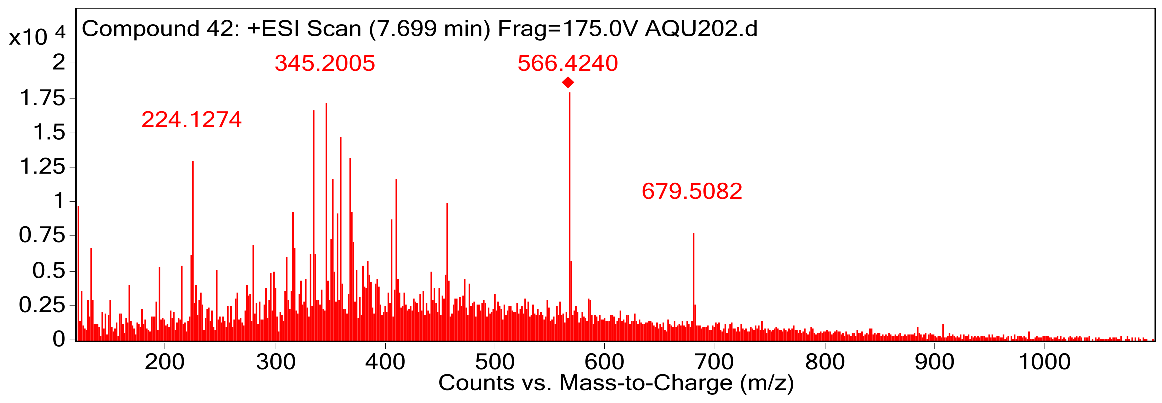

MS Zoomed Spectrum

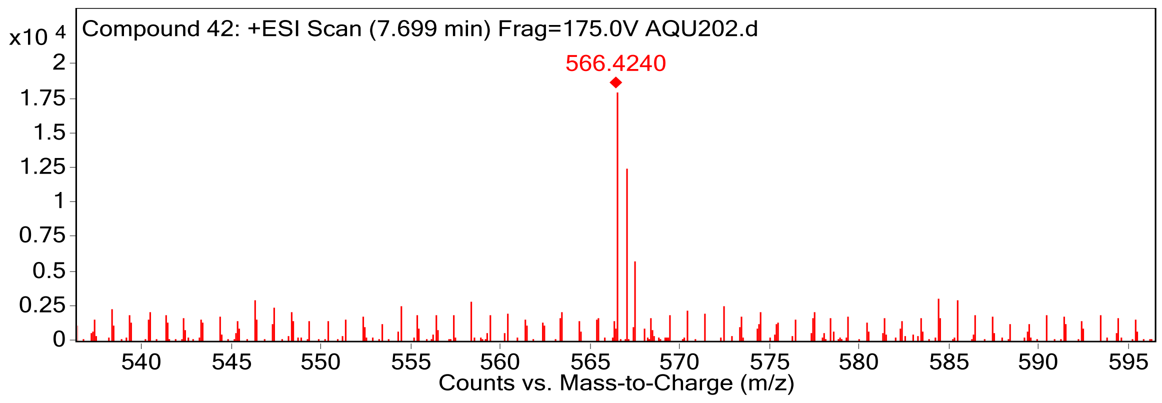

MS Spectrum Peak List

m/z	z	Abund
224.1274		13056.46
333.2043	1	16684.77
345.2005	1	17199.78
358.2677	1	14815.5
367.1824	1	13216.25
409.2178	1	11798.72
566.424	2	18048.13
566.9273	2	12515.28
567.4278	2	5802.08
567.9263	2	939.55

MSMS Spectrum

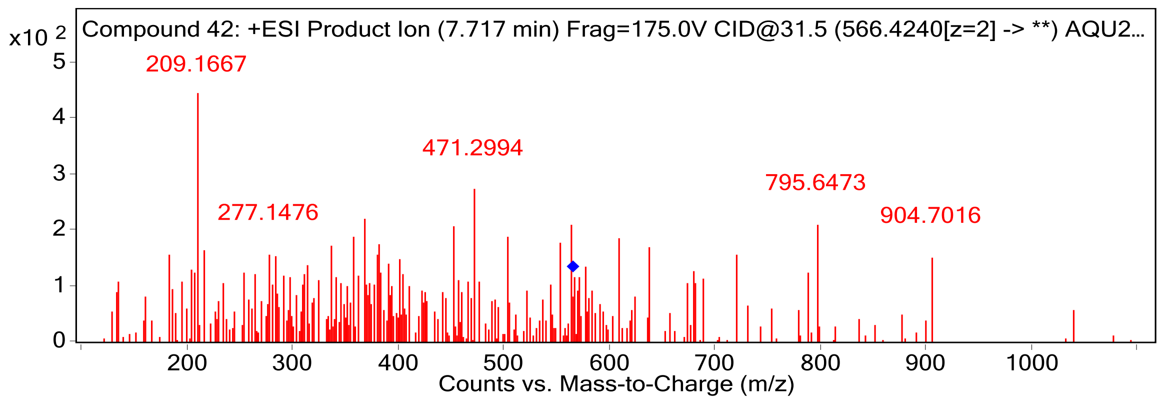

MS/MS Spectrum Peak List

m/z	Abund
209.1667	446.7
210.1475	190.93
356.2144	188.95
368.1517	220.28
452.3492	207.17
471.2994	274.1
471.4083	221.1
502.8893	190.11
562.3983	211.63
795.6473	212.03

Compound Label	Name	m/z	RT	Algorithm	Mass
Cpd 43: 1-Phosphatidyl-1D-myo-inositol 3-phosphate	**1-Phosphatidyl-1D-myo-inositol 3-phosphate**	493.014	8.168	Auto MS/MS	470.023

MS Spectrum

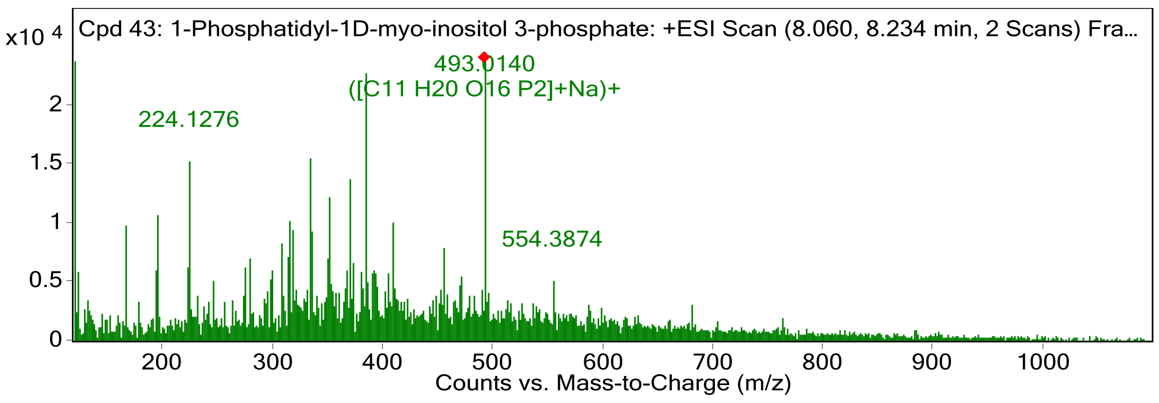

MS Zoomed Spectrum

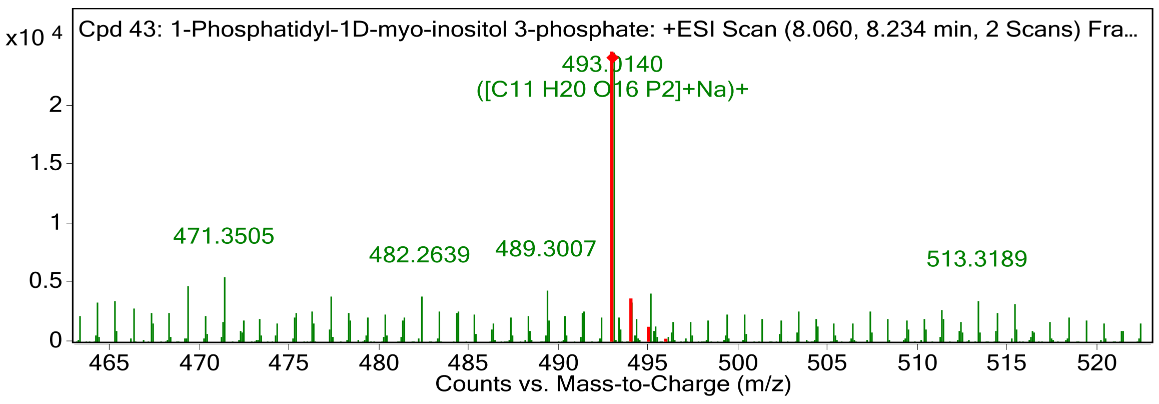

MS Spectrum Peak List

m/z	*Calc m/z*	Diff(ppm)	z	Abund	Formula	Ion
120.0807				23787.39		
224.1276				15347.15		
333.2043			1	15608		
370.2671				13756.57		
384.2829			1	22737.22		
493.014	493.0119	-4.22	1	24518.07	C11 H20 O16 P2	(M+Na)+
494.0153	494.0153	0.07	1	3474.5	C11 H20 O16 P2	(M+Na)+
495.0096	495.0166	14.1	1	4137.82	C11 H20 O16 P2	(M+Na)+
496.0131	496.0197	13.46	1	465.36	C11 H20 O16 P2	(M+Na)+
497.0032	497.0213	36.27	1	426.1	C11 H20 O16 P2	(M+Na)+

MSMS Spectrum

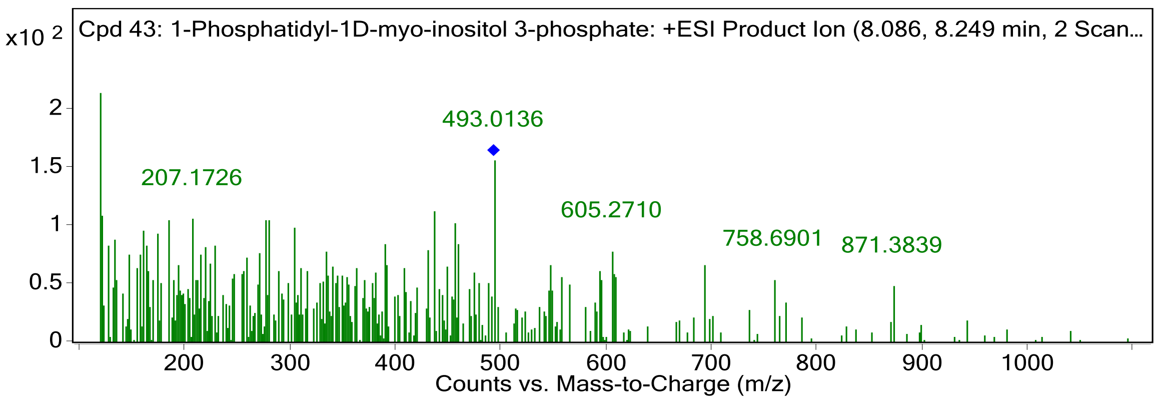

MS/MS Spectrum Peak List

m/z	z	Abund
120.0821	1	215
121.0666		109.17
185.0783		105.88
207.1726		106.22
277.2138		105.46
280.1048		105.8
304.1553		99.04
436.3209		112.36
456.321		102.13
493.0136		156.65

Compound Structure

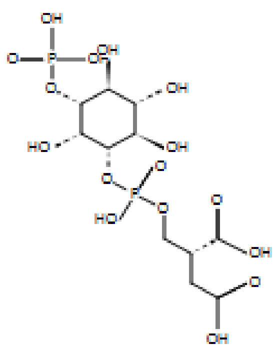

Compound Label	Name	*m/z*	RT	Algorithm	Mass
Cpd 44: Istamycin A1	**Istamycin A1**	418.2672	8.328	Auto MS/MS	417.2603

MS Spectrum

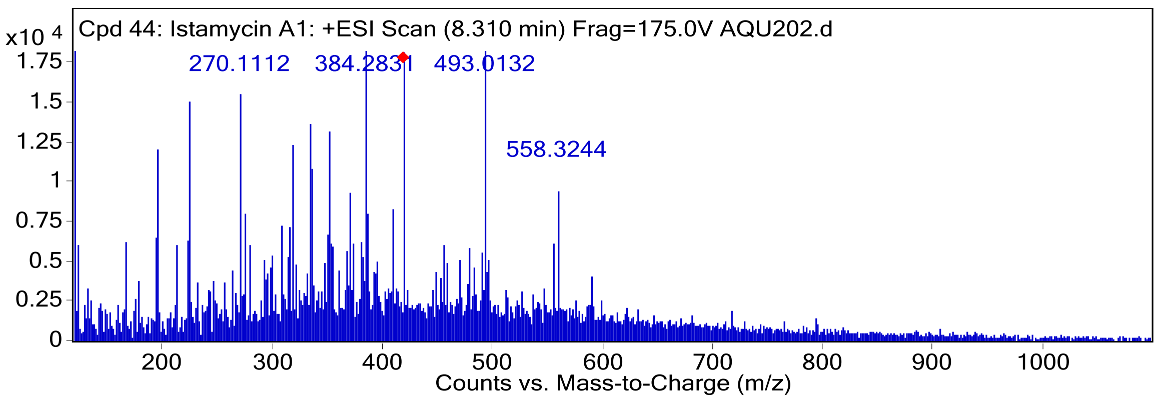

MS Zoomed Spectrum

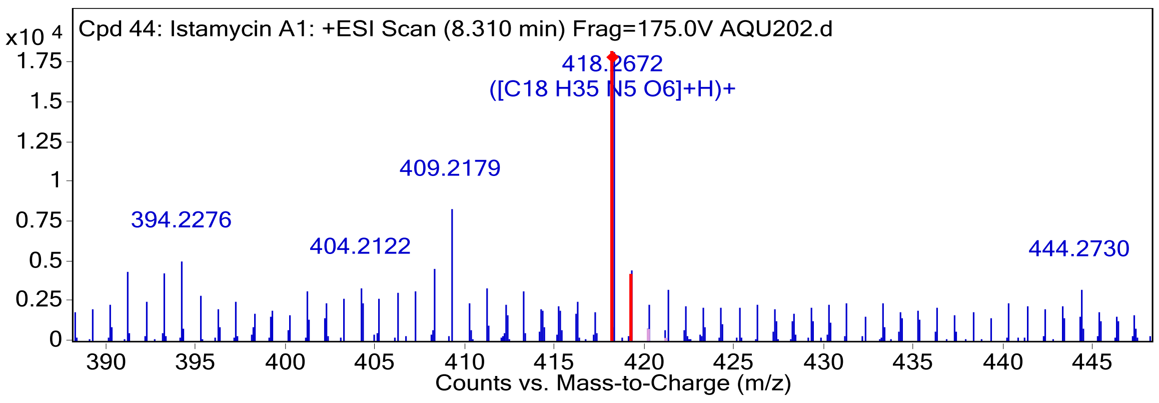

MS Spectrum Peak List

m/z	*Calc m/z*	Diff(ppm)	z	Abund	Formula	Ion
120.0809				19493.38		
224.1273				15064.04		
270.1112				15525.44		
317.2089				12409.71		
333.2033				13706.67		
351.2139			1	13215.27		
384.2831			1	36042.77		
418.2672	418.266	-2.9	1	18182.11	C18 H35 N5 O6	(M+H)+
419.272	419.2689	-7.43	1	4526	C18 H35 N5 O6	(M+H)+
493.0132			1	27977.82		

MSMS Spectrum

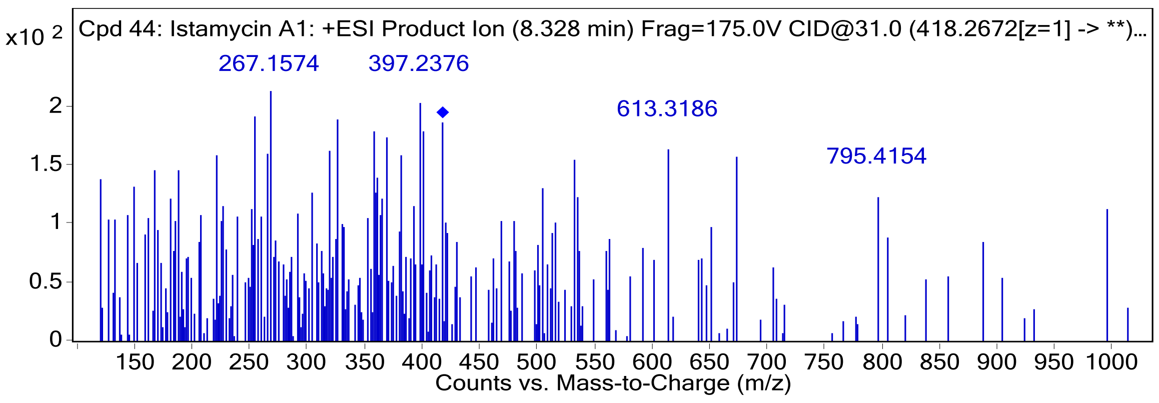

MS/MS Spectrum Peak List

m/z	Abund
254.1544	192.4
267.1574	213.22
318.1584	162.48
325.1769	188.88
357.2334	178.81
368.21	173.57
397.2376	203.84
400.2686	179.66
417.2164	187.46
613.3186	163.5

Compound Structure

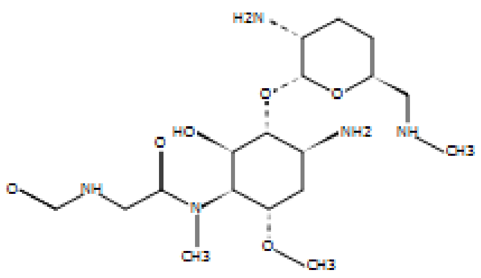

Compound Label	Name	*m/z*	RT	Algorithm	Mass
Cpd 45: Istamycin A1	**Istamycin A1**	416.2594	8.599	Auto MS/MS	415.2542

MS Spectrum

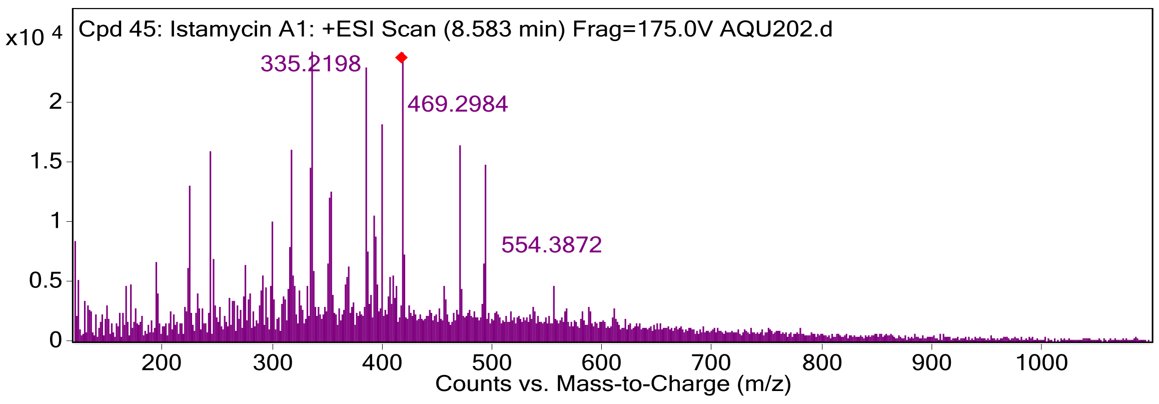

MS Zoomed Spectrum

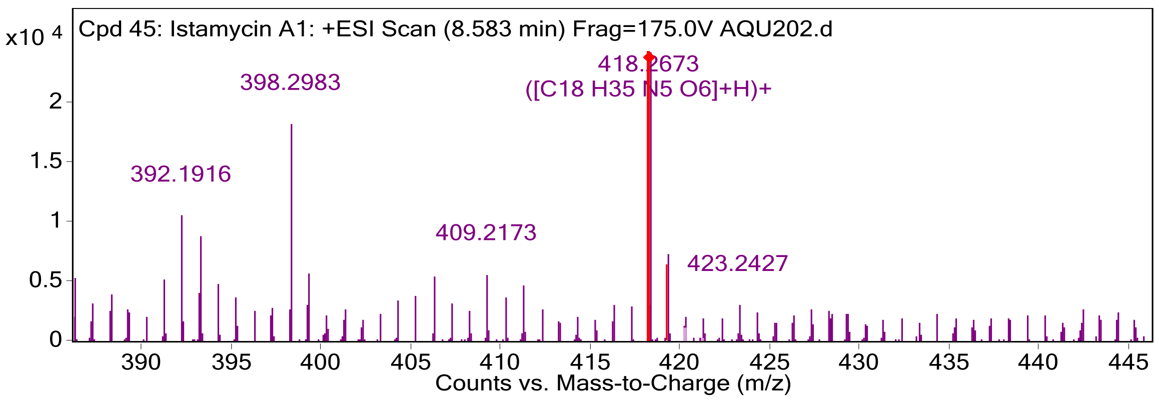

MS Spectrum Peak List

m/z	*Calc m/z*	Diff(ppm)	z	Abund	Formula	Ion
242.2832			1	15946.32		
317.2096			1	16031.3		

335.2198			1	25111.56		
384.2831			1	22950		
398.2983			1	18259.05		
416.2594	418.266	4820.66		3097.32	C18 H35 N5 O6	(M+H)+
417.2608	419.2689	4812.53	1	1723.37	C18 H35 N5 O6	(M+H)+
418.2673	420.2712	4790.96	1	24201.18	C18 H35 N5 O6	(M+H)+
419.2687	421.2737	4782.19	1	7402.64	C18 H35 N5 O6	(M+H)+
469.2984			1	16462.88		

MSMS Spectrum

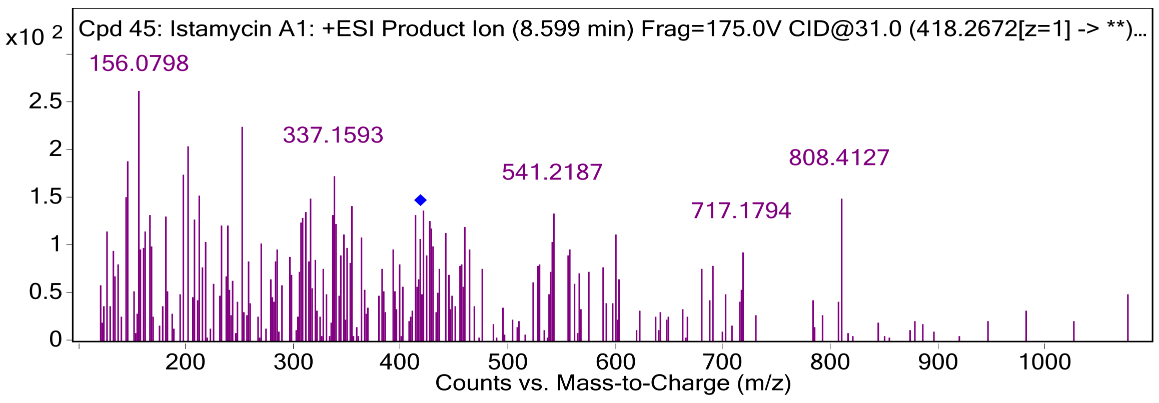

MS/MS Spectrum Peak List

m/z	Abund
143.081	152.2
145.1032	189.68
156.0798	262.41
196.1303	176.02
201.1659	205.72
212.1036	153.09
252.1083	225.07
315.1934	149.67
337.1593	173.53
808.4127	149.81

Compound Structure

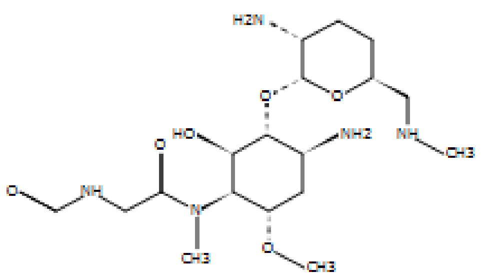

Compound Label	Name	*m/z*	RT	Algorithm	Mass
Cpd 46: Docosatetraenoyl ethanolamide	**Docosatetraenoyl ethanolamide**	398.2981	9.337	Auto MS/MS	375.3089

MS Spectrum

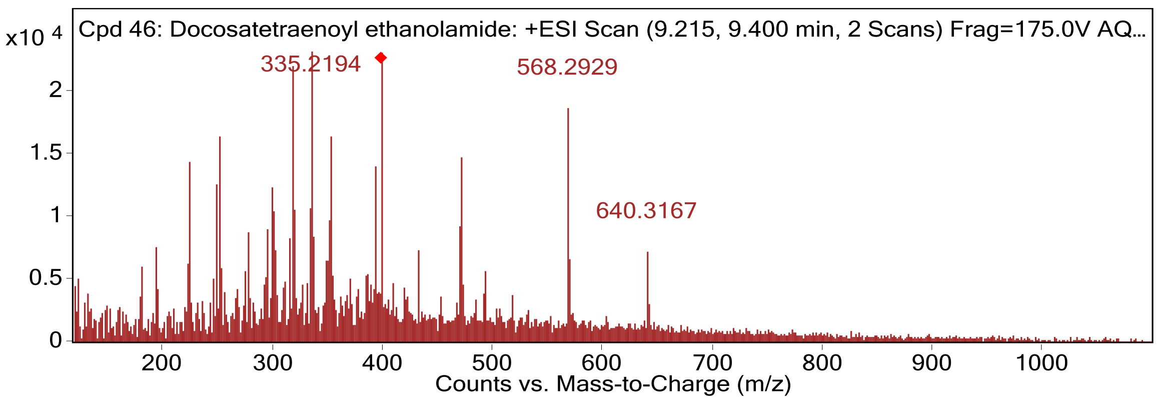

MS Zoomed Spectrum

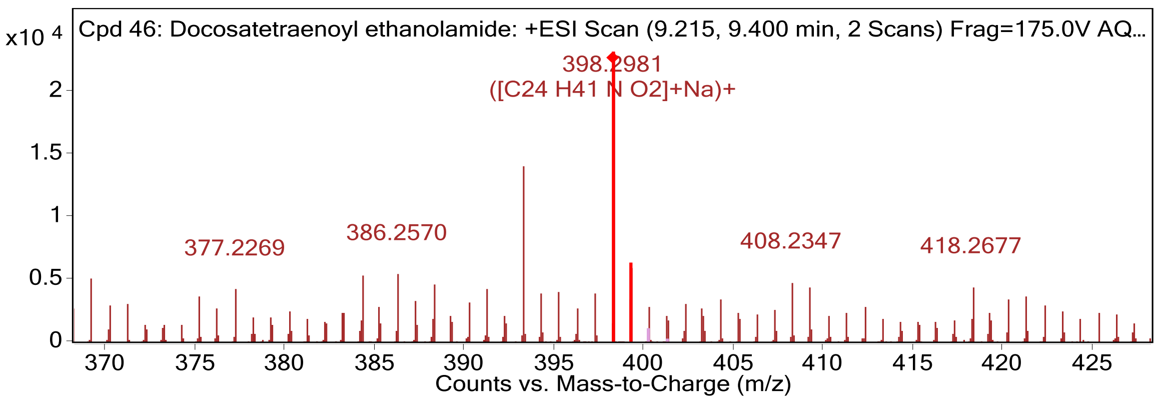

MS Spectrum Peak List

m/z	*Calc m/z*	Diff(ppm)	z	Abund	Formula	Ion
224.1272				14393.72		
251.1988				16511.63		
317.2089			1	22081.63		
335.2194			1	28592.34		
353.2291			1	16501.27		
393.222			1	14103.44		
398.2981	398.303	12.15	1	23128.57	C24 H41 N O2	(M+Na)+
399.3016	399.3063	11.64	1	5967.83	C24 H41 N O2	(M+Na)+
471.3754			1	14842.4		
568.2929			1	18704.95		

MSMS Spectrum

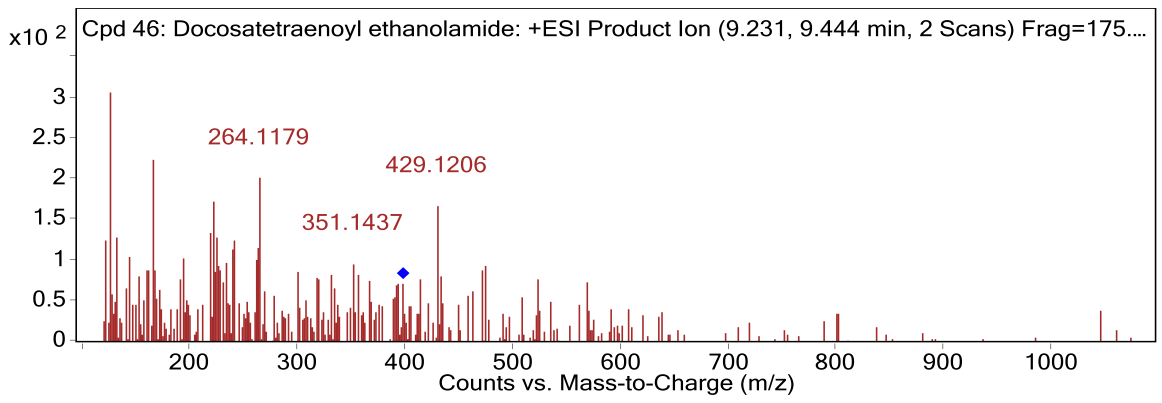

MS/MS Spectrum Peak List

m/z	Abund
121.0943	125.02
126.1281	307.34
132.102	128.18
165.1269	223.73
219.0515	133.19
222.1094	172.4

m/z	Abund
225.1256	128.56
240.1574	124.05
264.1179	201.44
429.1206	167.14

Compound Structure

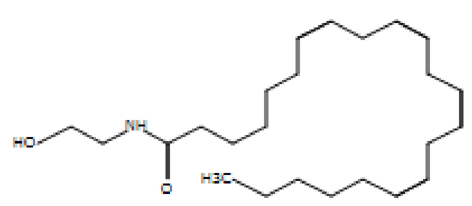

Compound Label	Name	*m/z*	RT	Algorithm	Mass
Cpd 47: Fleroxacin	**Fleroxacin**	370.1368	9.925	Auto MS/MS	369.13

MS Spectrum

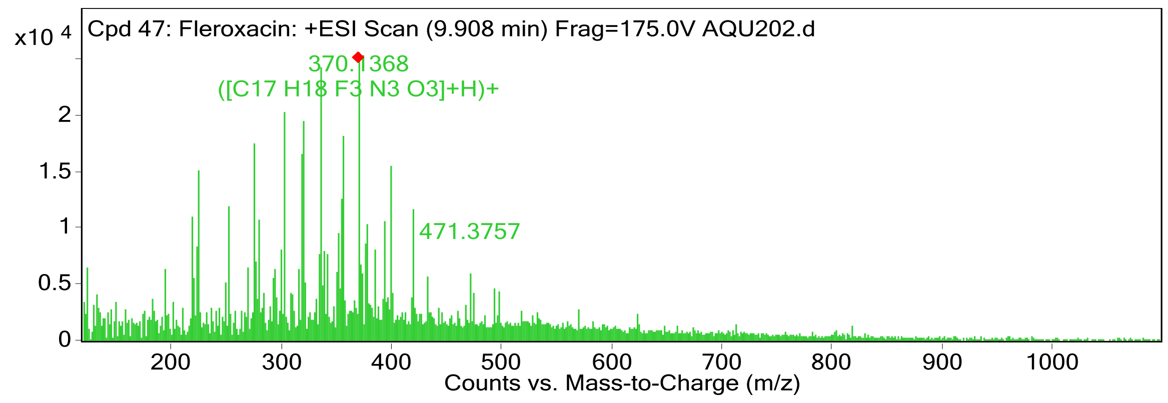

MS Zoomed Spectrum

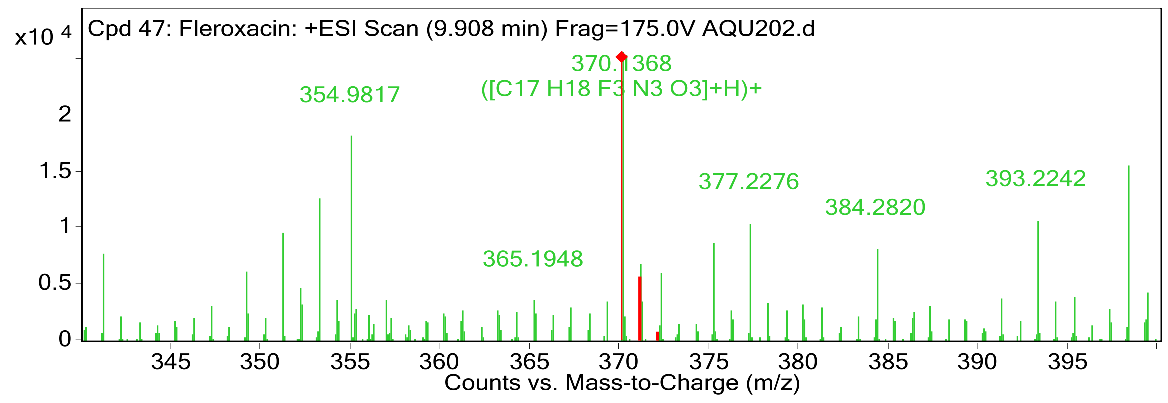

MS Spectrum Peak List

m/z	*Calc m/z*	Diff(ppm)	z	Abund	Formula	Ion
274.2721			1	17643.17		
301.214			1	20436.43		
317.2091			1	16708.37		
319.2247			1	19539.08		
335.2194			1	24366.21		
354.9817				18247.02		
370.1368	370.1373	1.44	1	25686.93	C17 H18 F3 N3 O3	(M+H)+
371.1407	371.1403	-1.04	1	6946.29	C17 H18 F3 N3 O3	(M+H)+
372.15	372.1429	-18.93	1	1511.38	C17 H18 F3 N3 O3	(M+H)+
398.2985			1	15583.39		

MSMS Spectrum

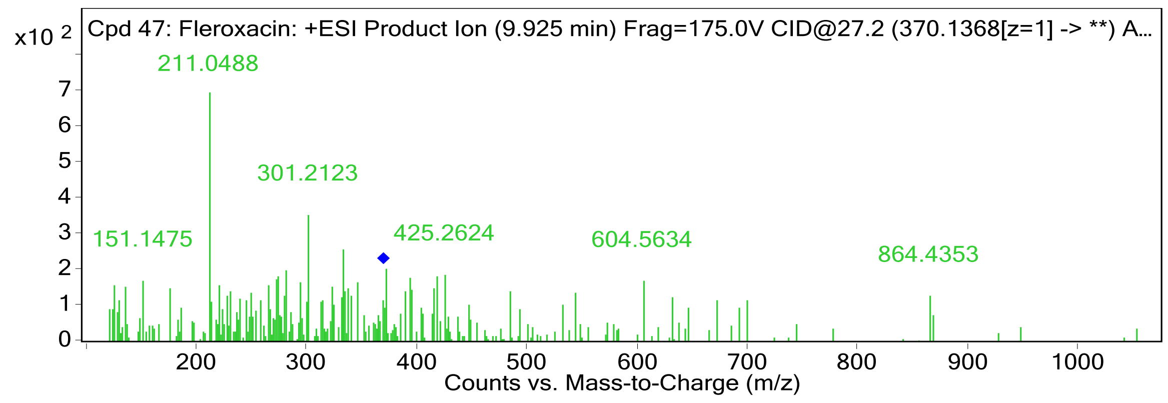

MS/MS Spectrum Peak List

m/z	Abund
211.0488	697.75
272.1294	176.42
274.2694	184.04
280.2056	198.43
301.2123	356.01
333.2034	259.17
371.1776	206.32
393.2189	177.28
418.3017	181.41
425.2624	188.2

Compound Structure

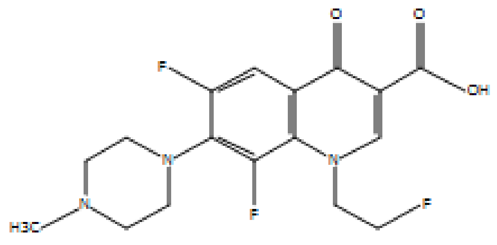

Compound Label	Name	*m/z*	RT	Algorithm	Mass
Cpd 48: Dibenzo[a,e]pyrene	**Dibenzo[a,e]pyrene**	325.0987	10.115	Auto MS/MS	302.1087

MS Spectrum

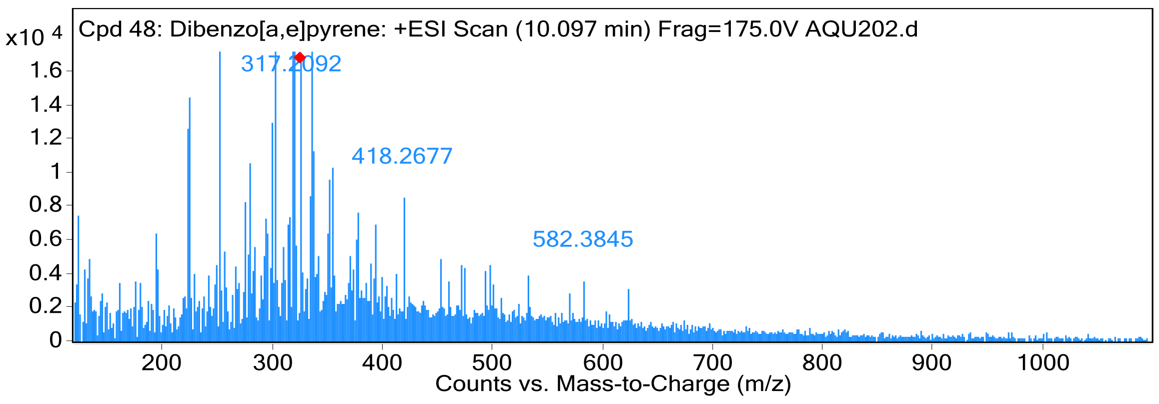

MS Zoomed Spectrum

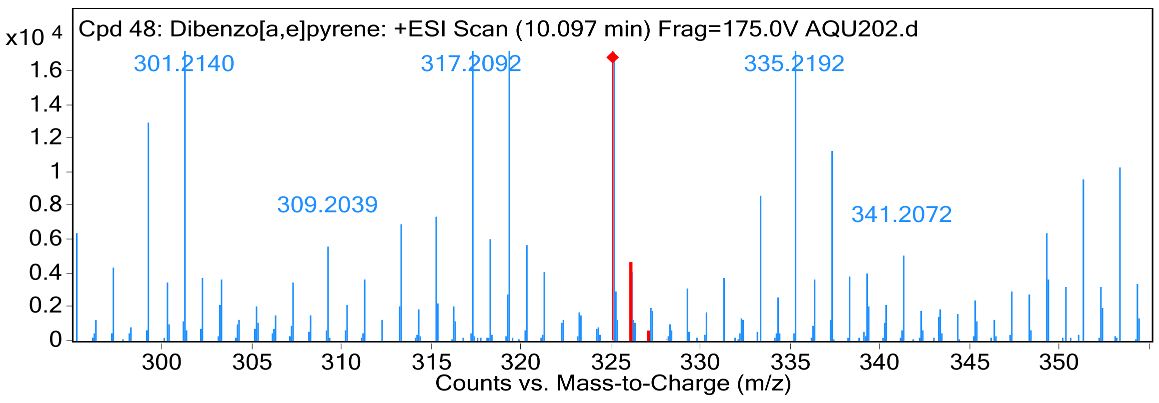

MS Spectrum Peak List

m/z	*Calc m/z*	Diff(ppm)	z	Abund	Formula	Ion
224.1272				14487.16		
251.1989			1	17250.9		
299.1985			1	12960.58		
301.214			1	19237.31		
317.2092			1	22163.14		
319.2246			1	19034.26		
325.0987	325.0988	0.15	1	17145.84	C24 H14	(M+Na)+
326.101	326.1021	3.43	1	4152.97	C24 H14	(M+Na)+
327.0961	327.1055	28.84	1	1375.27	C24 H14	(M+Na)+
335.2192			1	20647.55		

MSMS Spectrum

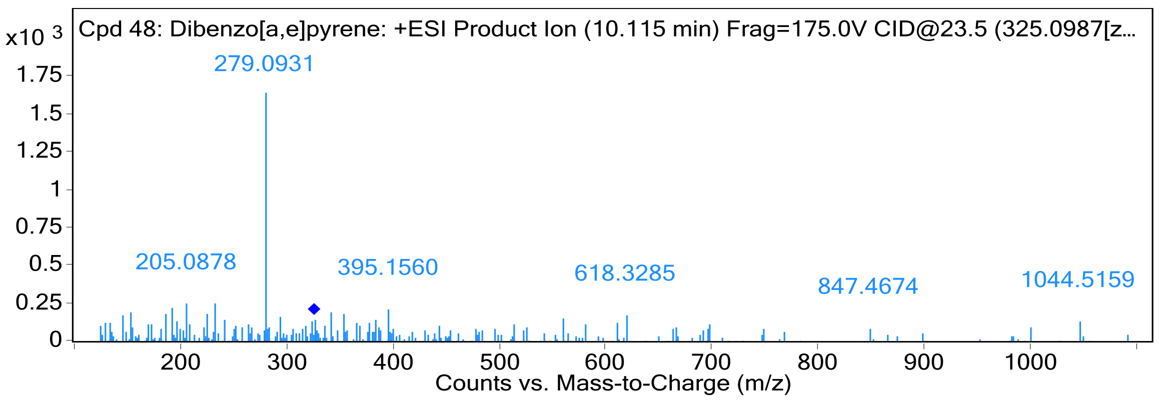

MS/MS Spectrum Peak List

m/z	z	Abund
152.0694		195.37
185.1311		190.72
191.0692		221.84
205.0878		255.18
224.1275		188
231.1375		253.2
279.0931	1	1648.01
340.1895		197.14
353.2028	1	190
395.156	1	218.72

Compound Structure

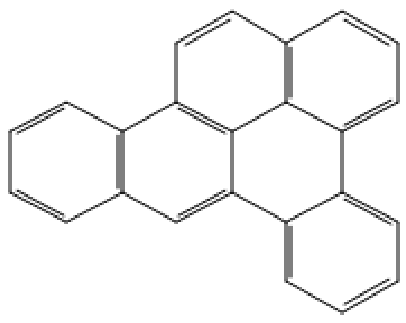

Compound Label	Name	*m/z*	RT	Algorithm	Mass

Cpd 49: Rhipocephalin	**Rhipocephalin**	377.2012	10.355	Auto MS/MS	376.1941

MS Spectrum

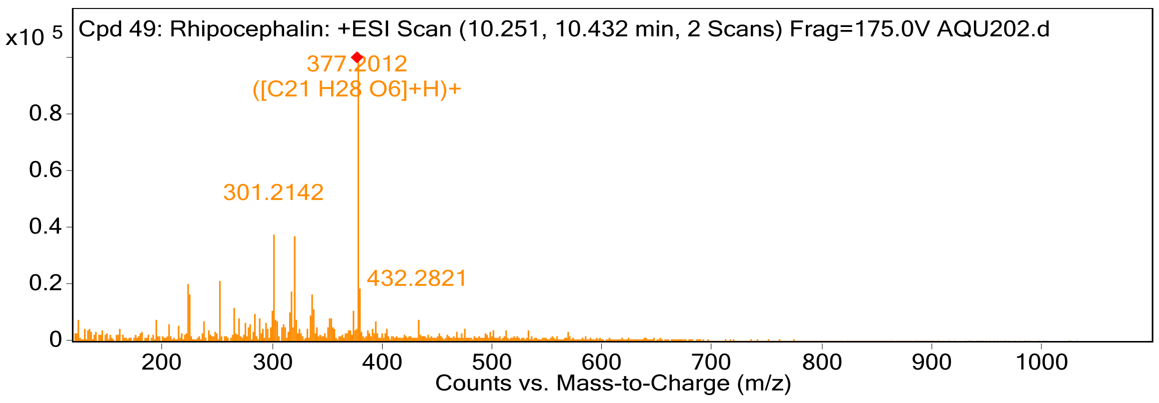

MS Zoomed Spectrum

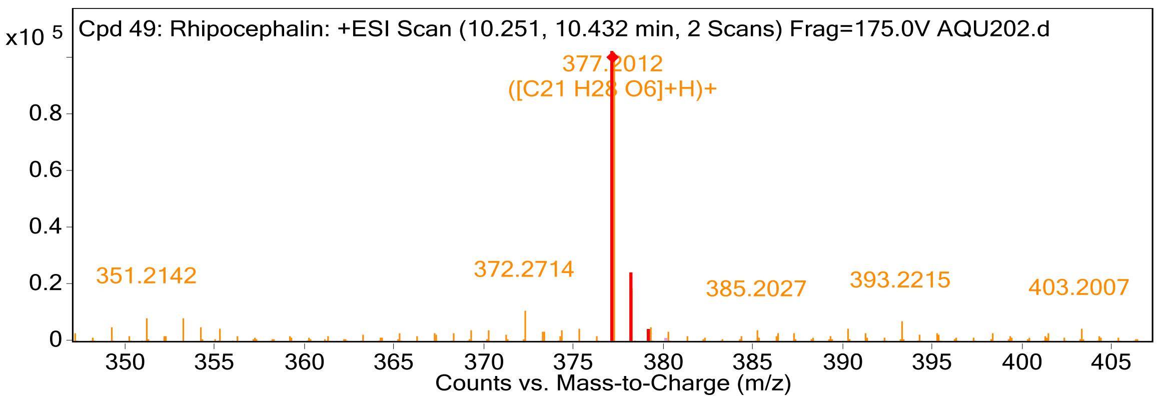

MS Spectrum Peak List

m/z	*Calc m/z*	Diff(ppm)	z	Abund	Formula	Ion
223.1683			1	20706.46		
224.1273				16593.58		
251.1632			1	21786.62		
301.2142			1	38097.01		
317.2092			1	17727.46		
319.2246			1	37274.18		
335.2196			1	16700.44		
377.2012	377.1959	-14.22	1	102038.34	C21 H28 O6	(M+H)+
378.2054	378.1993	-16.27	1	18778.36	C21 H28 O6	(M+H)+
379.2068	379.2019	-13.1	1	4178.5	C21 H28 O6	(M+H)+

MSMS Spectrum

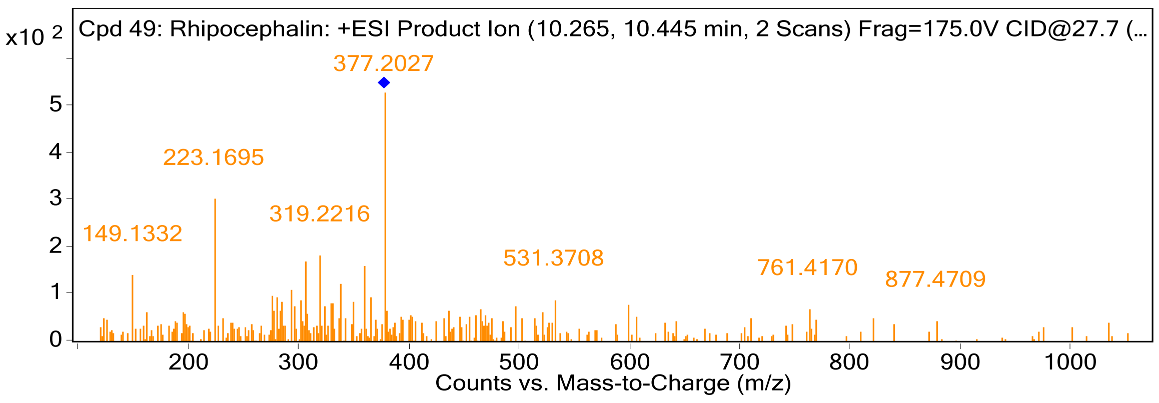

MS/MS Spectrum Peak List

m/z	z	Abund
149.1332		141.77
223.1695		304.03
224.1275		129.54
275.1973		97.79
293.2073		109.55
305.2059		172.14
319.2216		183.88
337.2154		124.01
359.1892		161.74
377.2027	1	530.29

Compound Structure

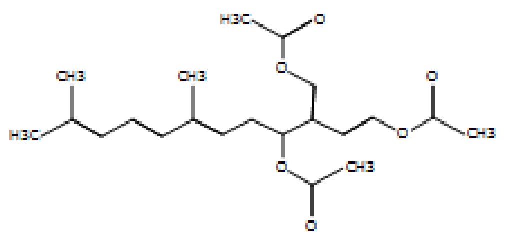

**Compound Label**	**Name**	***m/z***	**RT**	**Algorithm**	**Mass**
Cpd 50: Enalapril	**Enalapril**	377.2011	10.698	Auto MS/MS	376.1949

MS Spectrum

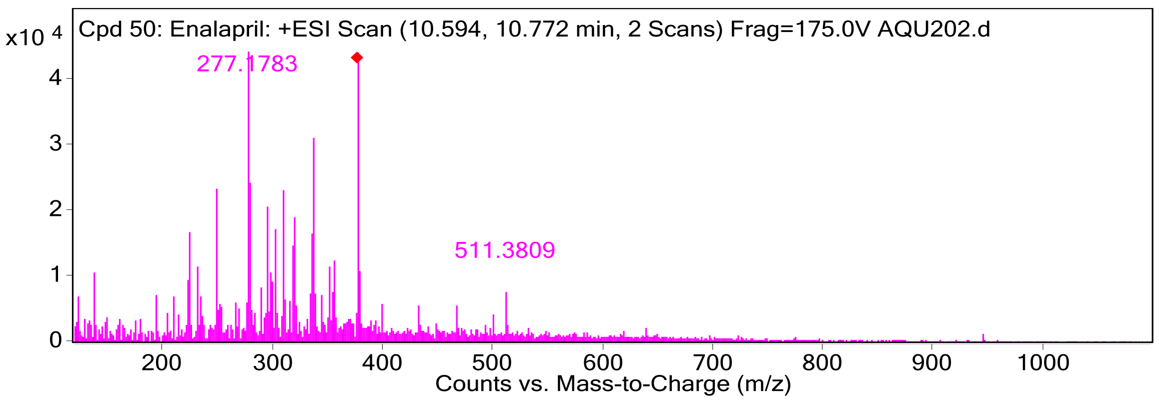

MS Zoomed Spectrum

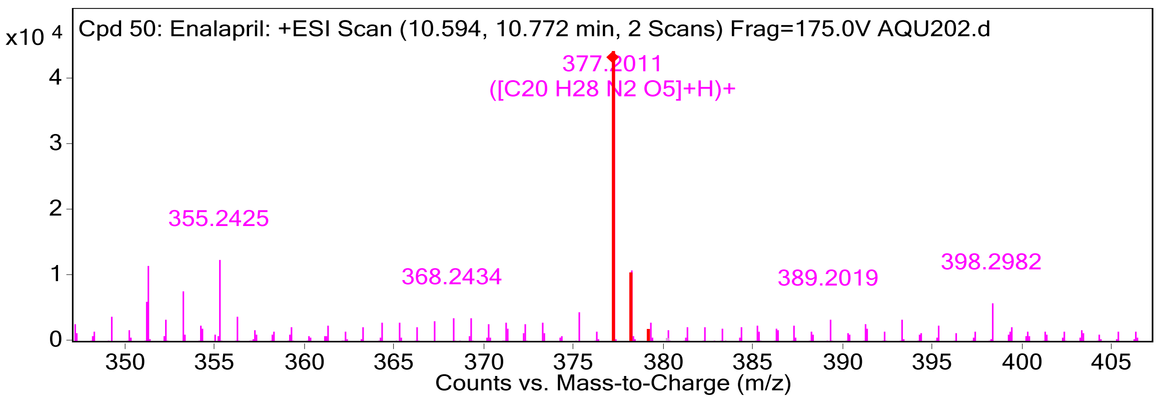

MS Spectrum Peak List

m/z	*Calc m/z*	Diff(ppm)	z	Abund	Formula	Ion
249.1833			1	23516.38		
277.1783			1	125136.14		
278.1816			1	24060.39		
279.2301			1	24450.24		
295.1883			1	20683.3		
309.2042			1	23194.11		
337.2348			1	31096.16		
377.2011	377.2071	15.8	1	44189.44	C20 H28 N2 O5	(M+H)+
378.2094	378.2103	2.3	1	10828.31	C20 H28 N2 O5	(M+H)+
379.2096	379.2129	8.64	1	1624.16	C20 H28 N2 O5	(M+H)+

MSMS Spectrum

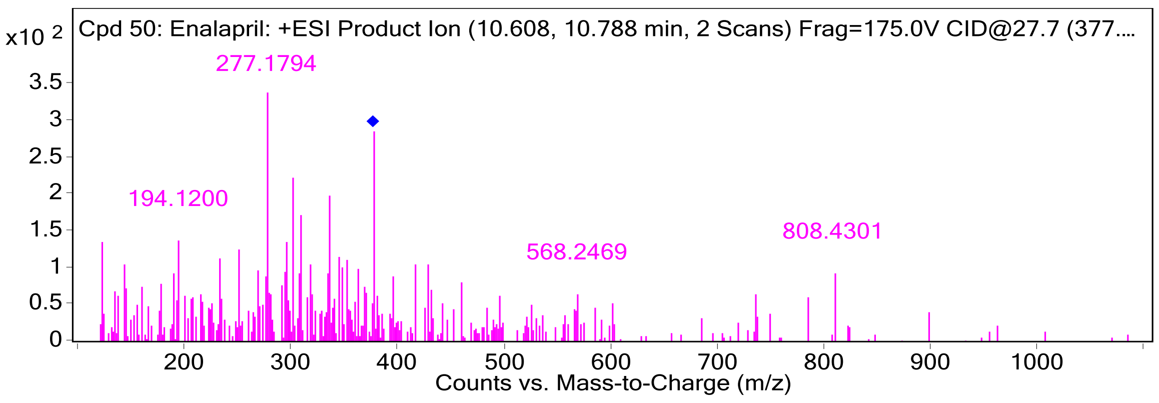

MS/MS Spectrum Peak List

m/z	z	Abund
123.0793		136.13
194.12		138.37
250.1898		126.47
277.1794		339.12
295.1904	1	136.51
301.2161		222.03
309.2046		171.37
335.219		199.31
344.2438		115.49
377.2038		285.65

Compound Structure

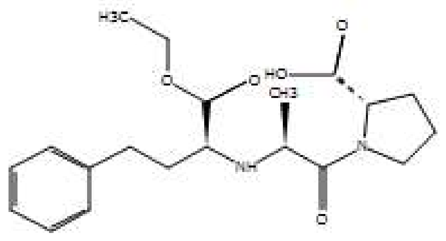

Compound Label	Name	*m/z*	RT	Algorithm	Mass
Cpd 51: Carpaine	**Carpaine**	479.3809	11.714	Auto MS/MS	478.3737

MS Spectrum

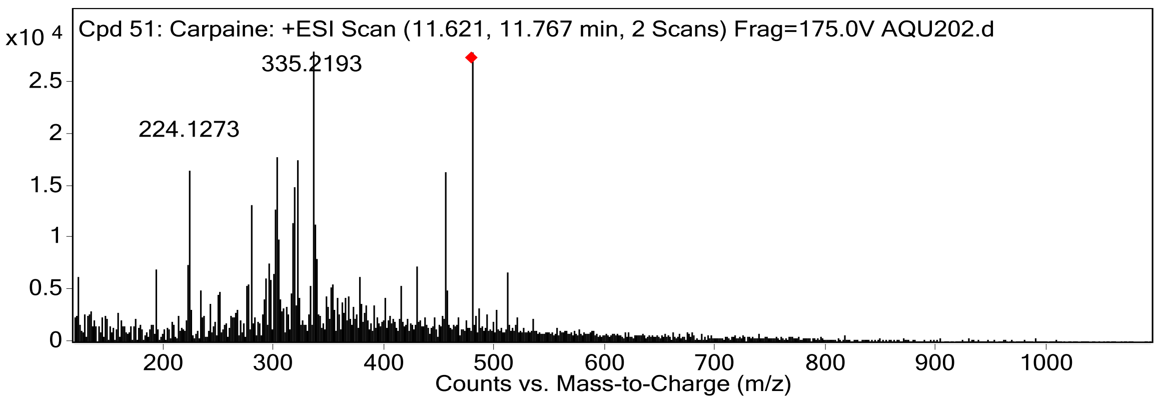

MS Zoomed Spectrum

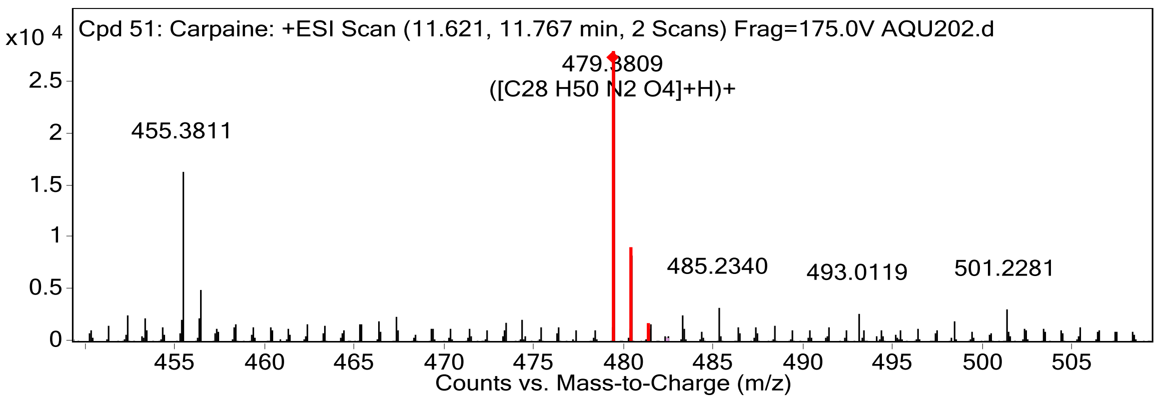

MS Spectrum Peak List

m/z	*Calc m/z*	Diff(ppm)	z	Abund	Formula	Ion
224.1273			1	16521.46		
279.2301				13227.68		

303.23			1	17876.12		
319.2241			1	14987.22		
321.2405			1	17583.88		
335.2193			1	32620.52		
455.3811			1	16346.67		
479.3809	479.3843	7.14	1	27857.56	C28 H50 N2 O4	(M+H)+
480.3845	480.3876	6.53	1	8365.51	C28 H50 N2 O4	(M+H)+
481.3881	481.3905	5.13	1	1700.05	C28 H50 N2 O4	(M+H)+

MSMS Spectrum

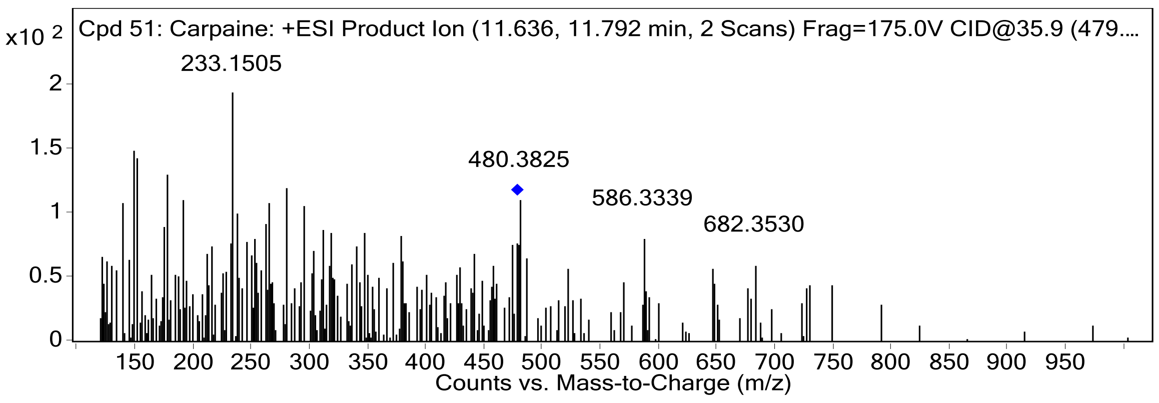

MS/MS Spectrum Peak List

m/z	Abund
139.0567	108.44
149.096	148.62
151.0764	143.5
177.1611	130.81
191.0142	111.17
233.1505	195.02
264.2337	108.36
279.228	119.64
295.1846	105.73
480.3825	110.07

Compound Structure

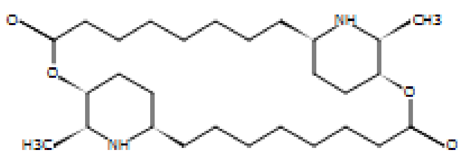

Compound Label	Name	*m/z*	RT	Algorithm	Mass
Cpd 52: 16-Oxo-palmitate	**16-Oxo-palmitate**	293.2092	12.063	Auto MS/MS	270.22

MS Spectrum

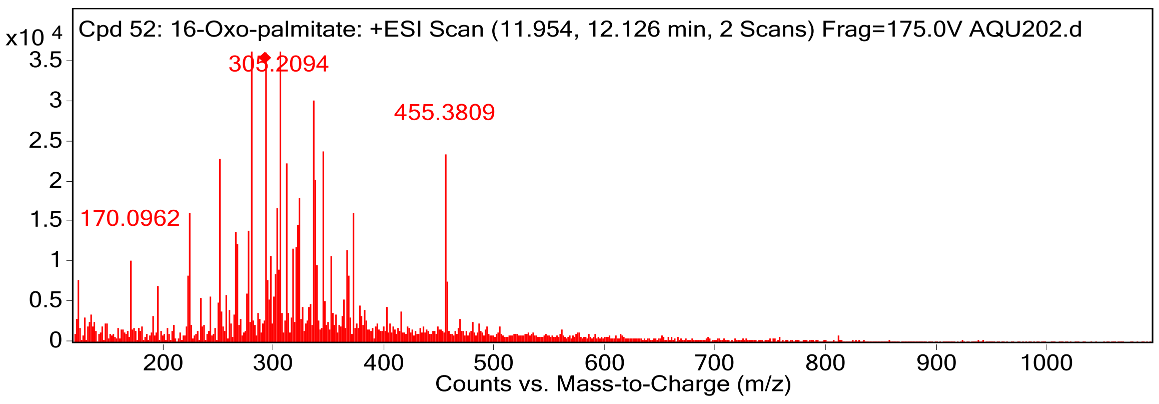

MS Zoomed Spectrum

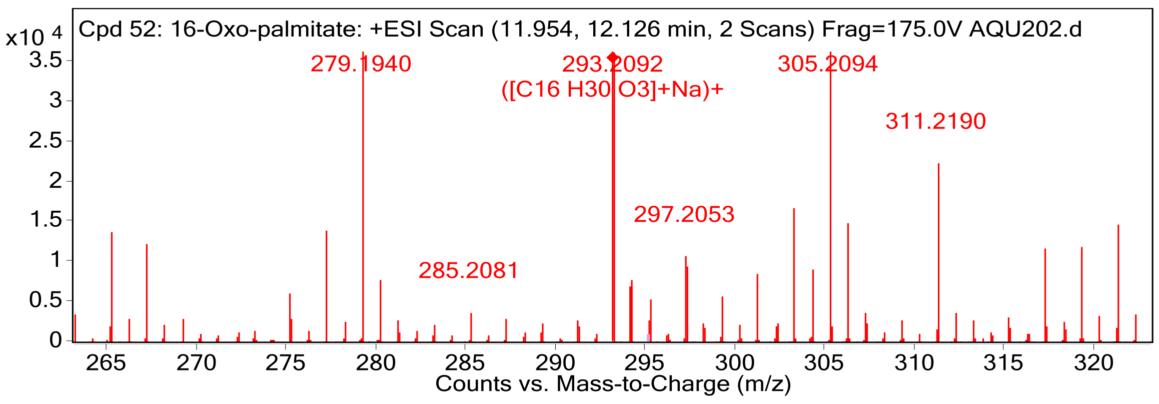

MS Spectrum Peak List

m/z	*Calc m/z*	Diff(ppm)	z	Abund	Formula	Ion
251.1994			1	22859.63		
279.194			1	38982.24		
293.2092	293.2087	-1.61	1	36124.42	C16 H30 O3	(M+Na)+
294.2125	294.2121	-1.24	1	7850.67	C16 H30 O3	(M+Na)+
305.2094			1	65398.61		
311.219			1	22428.64		
335.2195			1	30213.13		
337.2349			1	20241.64		
344.3135			1	23844.69		
455.3809			1	23547.59		

MSMS Spectrum

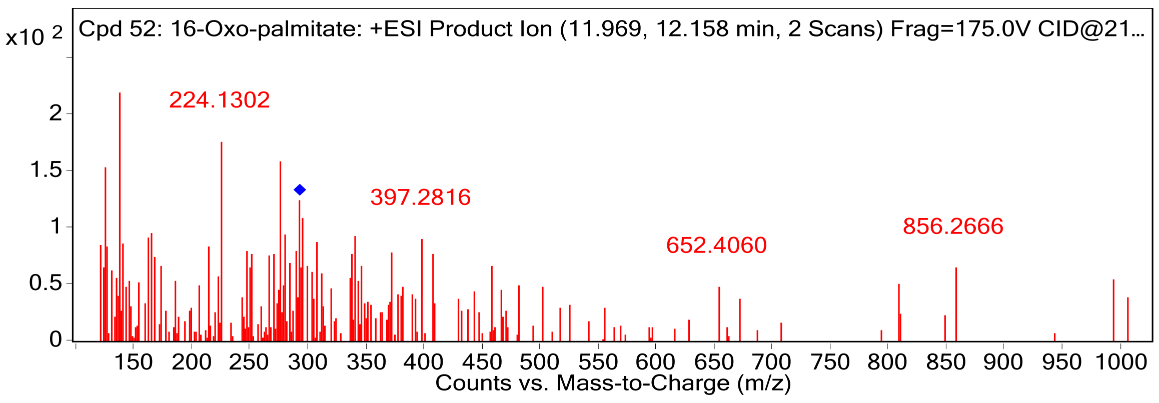

MS/MS Spectrum Peak List

m/z	Abund
125.0601	153.9
137.06	219.77
162.075	91.85
165.1239	95.38
224.1302	176.5
275.1977	159.37
279.232	94.35
292.205	124.62
294.2133	108.45
339.1753	93.51

Compound Structure

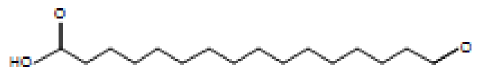

Compound Label	Name	*m/z*	RT	Algorithm	Mass
Cpd 53: 3b,17a,21-Trihydroxypregnenone	**3b,17a,21-Trihydroxypregnenone**	371.2197	12.167	Auto MS/MS	348.2299

MS Spectrum

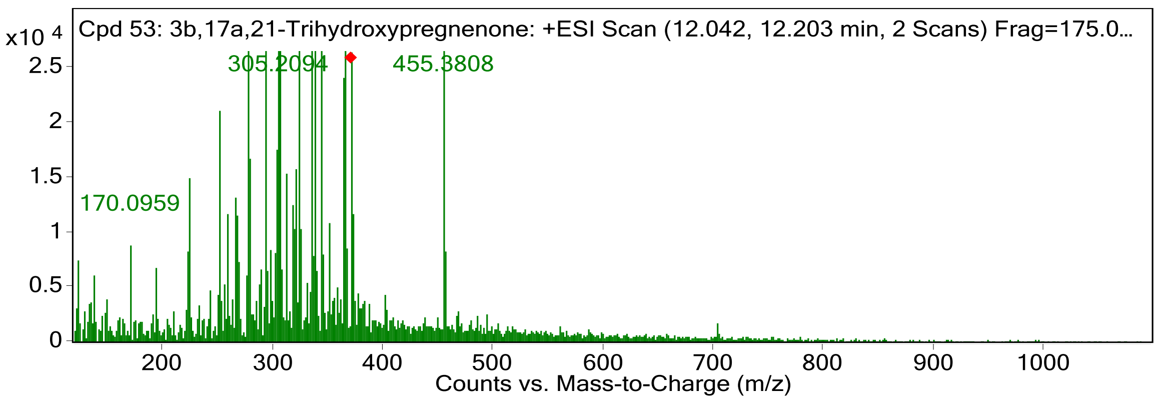

MS Zoomed Spectrum

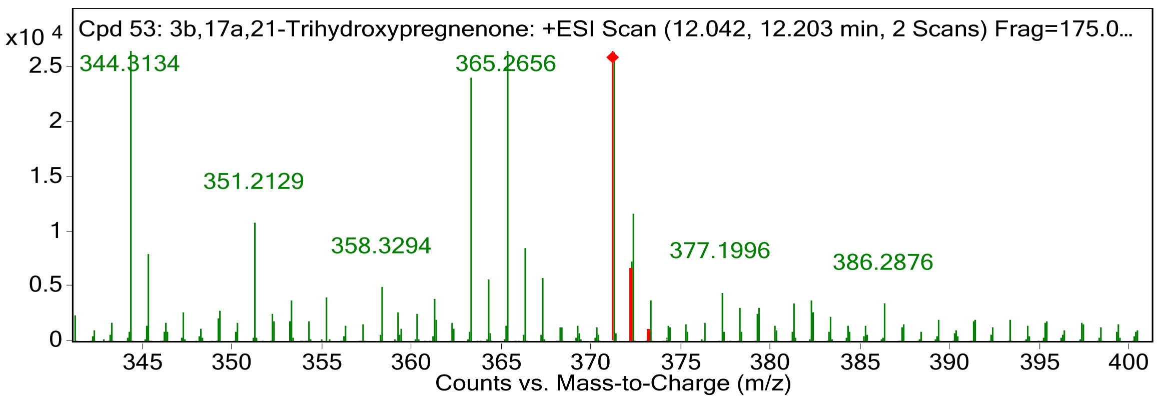

MS Spectrum Peak List

m/z	*Calc m/z*	Diff(ppm)	z	Abund	Formula	Ion
277.2143			1	38710.29		
305.2094			1	211724.31		
306.2125			1	47383.6		
323.2199			1	49784.3		
335.2195			1	40068.59		
337.2349			1	40963.32		
365.2656			1	35292.7		
371.2197	371.2193	-1.04	1	26398.7	C21 H32 O4	(M+Na)+
372.2226	372.2227	0.32	1	7398.59	C21 H32 O4	(M+Na)+
373.2192	373.2255	16.9	1	2099.55	C21 H32 O4	(M+Na)+

MSMS Spectrum

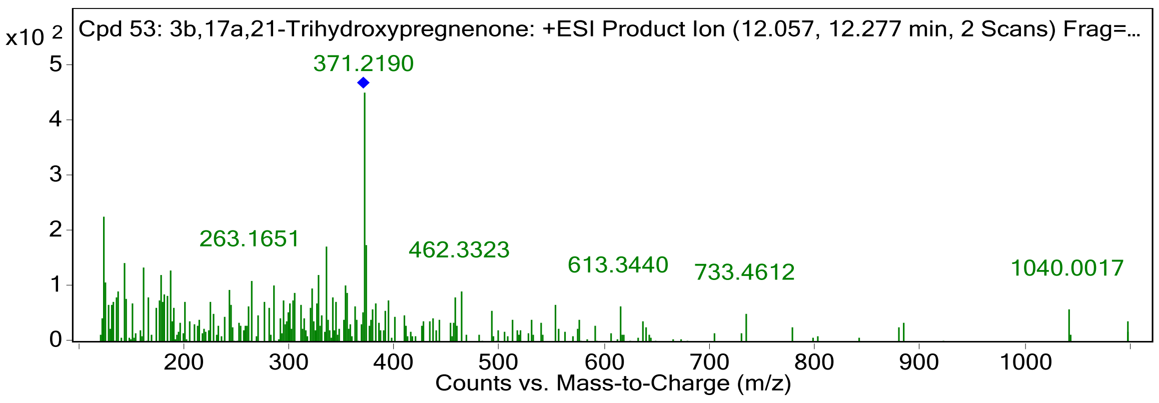

MS/MS Spectrum Peak List

m/z	z	Abund
123.0796		227.25
143.0868		142.15
161.1301		135.36
177.0935		120.79
187.1459	1	130.21
327.0468		120.79
335.2173	1	174.28
370.3066		113.17
371.219	1	452.73
372.2203	1	176.55

Compound Structure

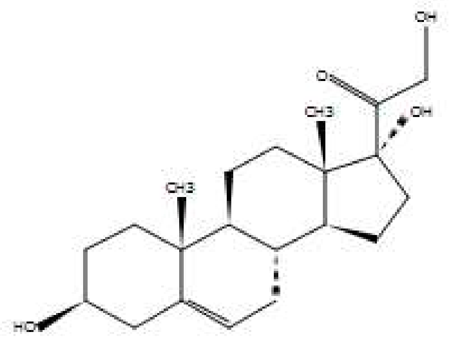

Compound Label	Name	*m/z*	RT	Algorithm	Mass
Cpd 54: 5-Hydroxy-4-methoxy-5-(1-oxo-9,12,15-hexadecatrienyl)-2(5H)-furanone	**5-Hydroxy-4-methoxy-5-(1-oxo-9,12,15-hexadecatrienyl)-2(5H)-furanone**	363.2117	12.218	Auto MS/MS	362.2045

MS Spectrum

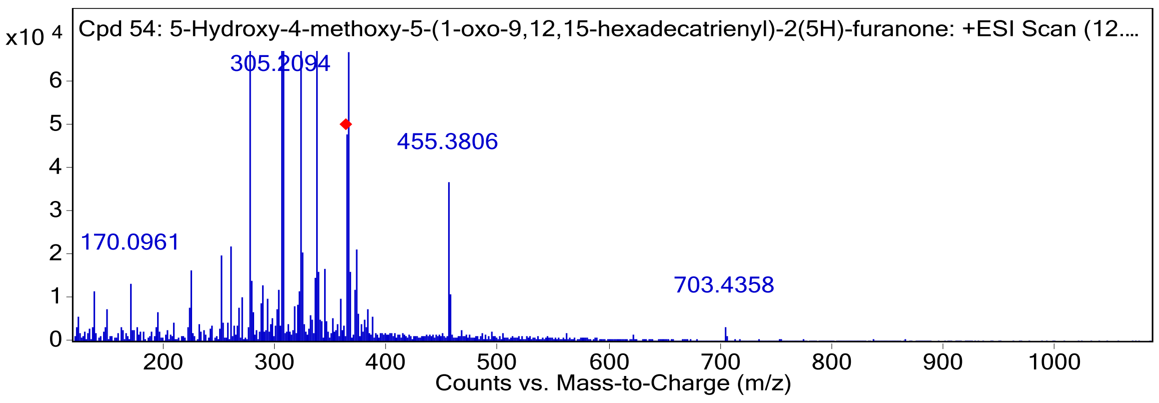

MS Zoomed Spectrum

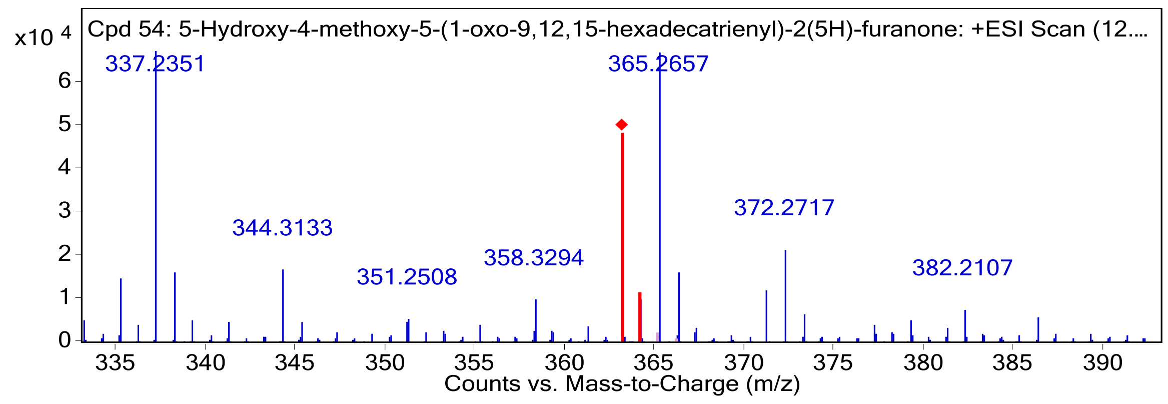

MS Spectrum Peak List

m/z	*Calc m/z*	Diff(ppm)	z	Abund	Formula	Ion
259.204			1	21956.09		
277.2143			1	72132.53		
305.2094			1	417783.16		
306.2126			1	93179.92		
323.2197			1	96211.98		
337.2351			1	72106.92		
363.2117	363.2166	13.54	1	48148.1	C21 H30 O5	(M+H)+
364.2158	364.22	11.42	1	10027.85	C21 H30 O5	(M+H)+
365.2657			1	67066.25		
455.3806			1	36828.47		

MSMS Spectrum

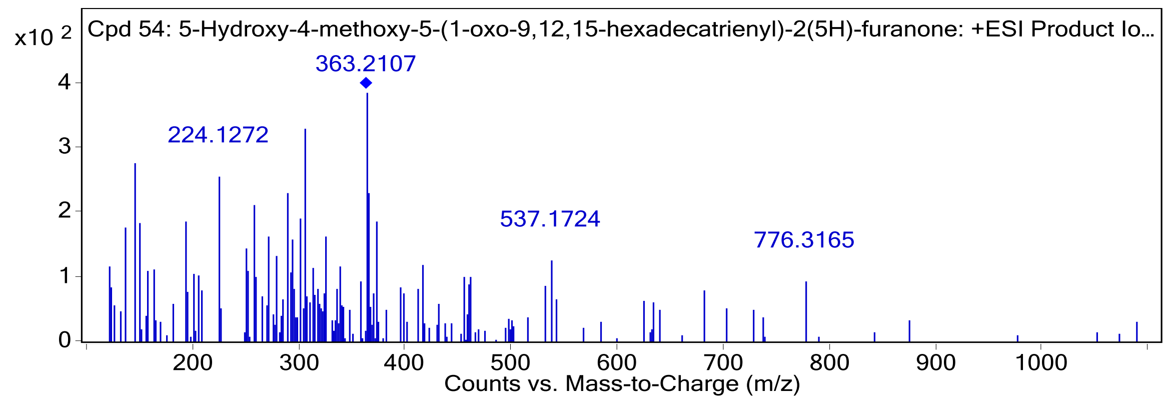

MS/MS Spectrum Peak List

m/z	z	Abund
145.1		277.65
224.1272	1	256.54
257.1107		212.7
288.2484		230.35
301.2124		191.25
305.2111	2	330.47
305.2476		289.64
363.2107		386.11
365.2628	1	229.68
372.2686		187.47

Compound Structure

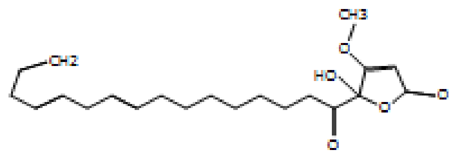

Compound Label	Name	*m/z*	RT	Algorithm	Mass
Cpd 55: 6-Oxabicyclo[3.1.0]hexane-2-undecanoic acid methyl	**6-Oxabicyclo[3.1.0]hexane-2-undecanoic acid**	305.2092	12.223	Auto MS/MS	282.2202

MS Spectrum

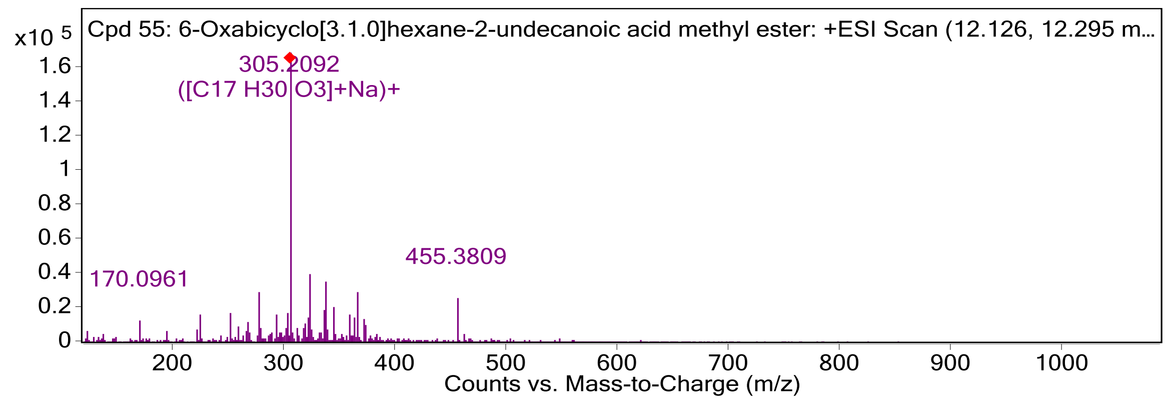

MS Zoomed Spectrum

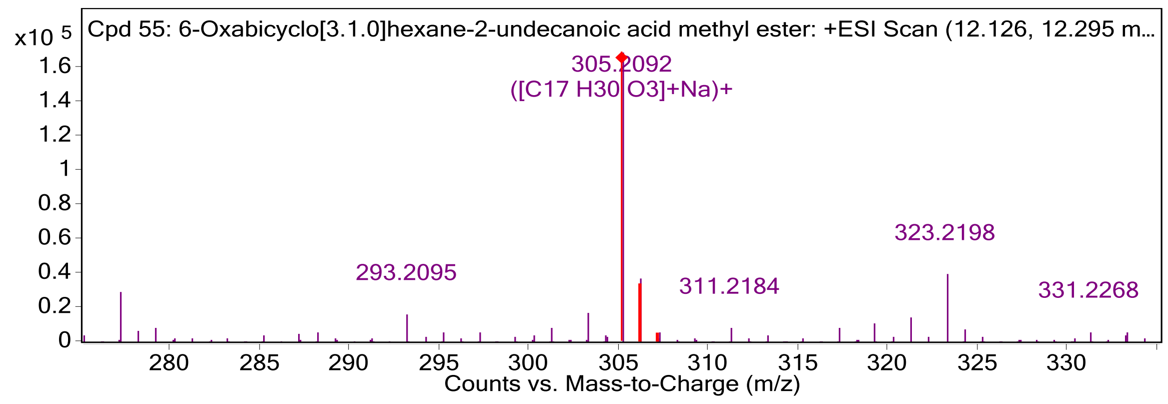

MS Spectrum Peak List

m/z	*Calc m/z*	Diff(ppm)	z	Abund	Formula	Ion
277.2149			1	30021.38		
305.2092	305.2087	-1.74	1	169020.28	C17 H30 O3	(M+Na)+
306.2128	306.2121	-2.08	1	37874.2	C17 H30 O3	(M+Na)+
307.2197	307.2149	-15.63	1	5955.65	C17 H30 O3	(M+Na)+
323.2198			1	39903.24		
335.2192			1	19071.99		
337.2352			1	35511.41		
344.3133			1	20673.57		
365.2661			1	30007.79		
455.3809			1	26034.01		

MSMS Spectrum

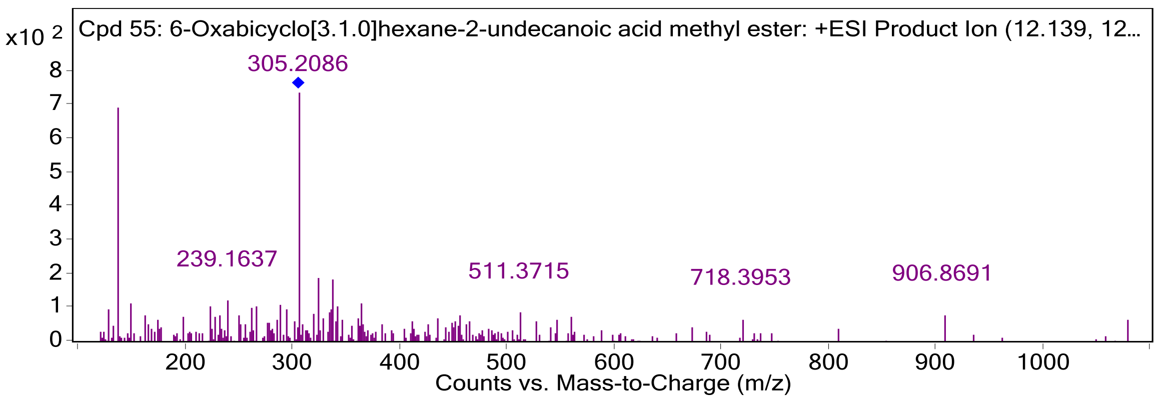

MS/MS Spectrum Peak List

m/z	z	Abund
137.059		690.71
149.1309		112.57
222.1102		105.4
239.1637		122.54
288.2492		109.7
305.2086	1	737.22
306.2137	1	136.62
323.2201	1	190.44
337.2357		185.42
363.2138		113.88

Compound Structure

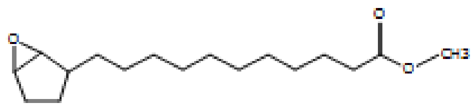

Compound Label	Name	*m/z*	RT	Algorithm	Mass
Cpd 56: 12-(2,3-Dihydroxycyclopentyl)-2-dodecanone	**12-(2,3-Dihydroxycyclopentyl)-2-dodecanone**	307.2253	12.522	Auto MS/MS	284.2362

MS Spectrum

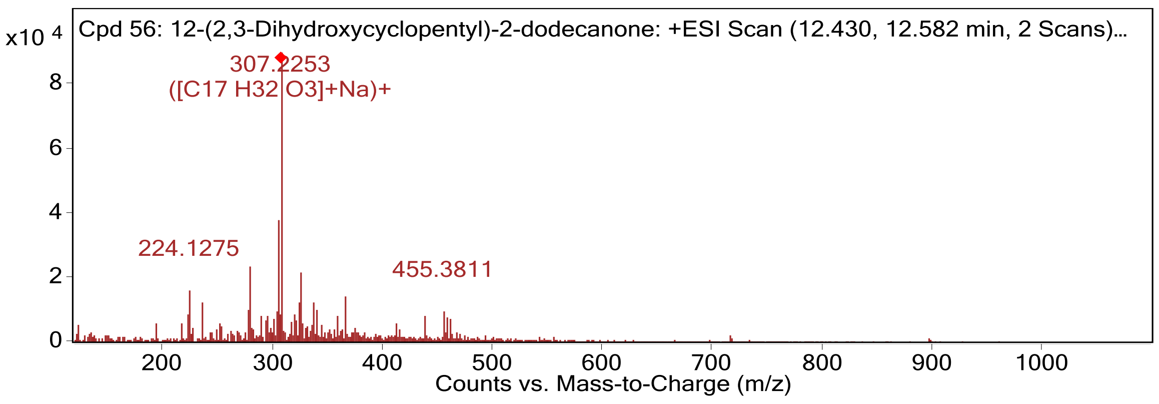

MS Zoomed Spectrum

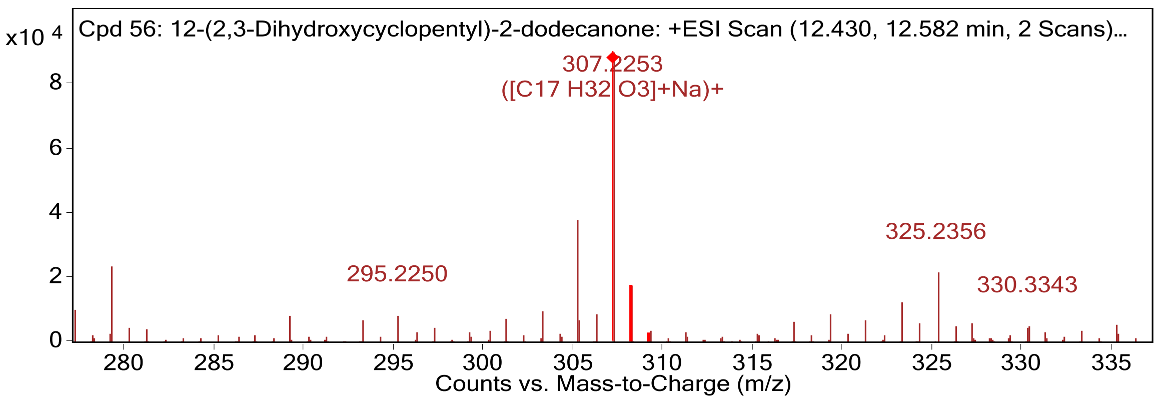

MS Spectrum Peak List

m/z	*Calc m/z*	Diff(ppm)	z	Abund	Formula	Ion
224.1275			1	16339.94		
235.2047				12708.56		
279.2302			1	23596.13		
305.2096			1	38227.81		
307.2253	307.2244	-3.03	1	89966.6	C17 H32 O3	(M+Na)+
308.2281	308.2278	-1.13	1	17669.37	C17 H32 O3	(M+Na)+
309.2362	309.2305	-18.25	1	3850.83	C17 H32 O3	(M+Na)+
325.2356			1	21574		
337.2352			1	12654.52		
365.2284			1	14372.97		

MSMS Spectrum

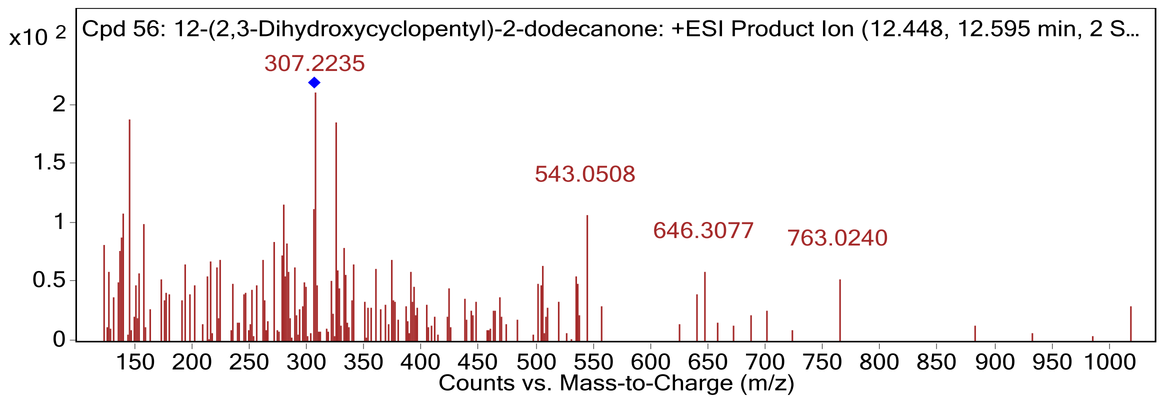

MS/MS Spectrum Peak List

m/z	z	Abund
139.0737		108.35
145.1043		188.94
158.0976		99.95
279.2282		116.02
305.2113		112.85
307.2235	1	211.84
307.2451		110
307.3114		136.88
325.2313	1	185.54
543.0508		107.11

Compound Structure

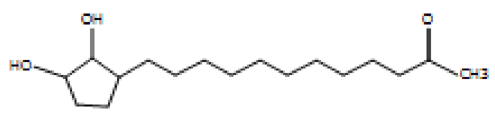

Compound Label	Name	*m/z*	RT	Algorithm	Mass
Cpd 57: 6-Oxabicyclo[3.1.0]hexane-2-undecanoic acid methyl	**6-Oxabicyclo[3.1.0]hexane-2-undecanoic acid**	305.2096	12.535	Auto MS/MS	282.2203

MS Spectrum

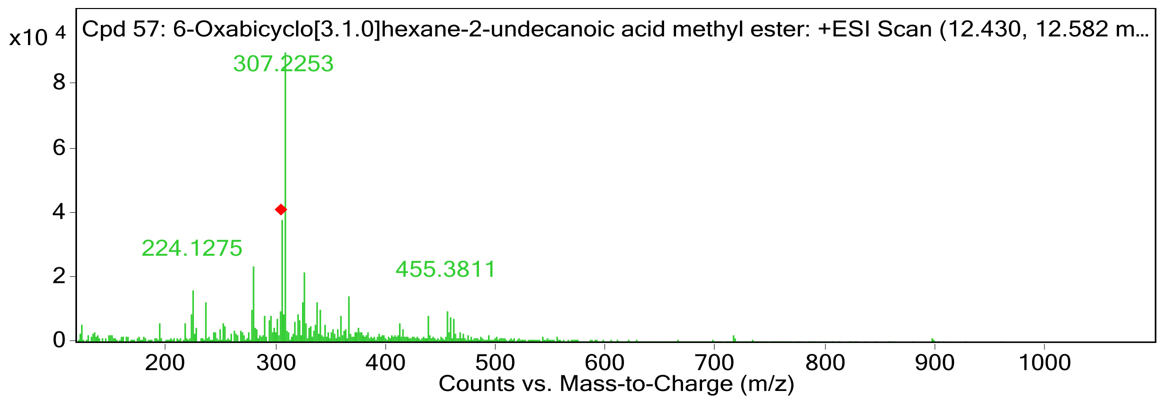

MS Zoomed Spectrum

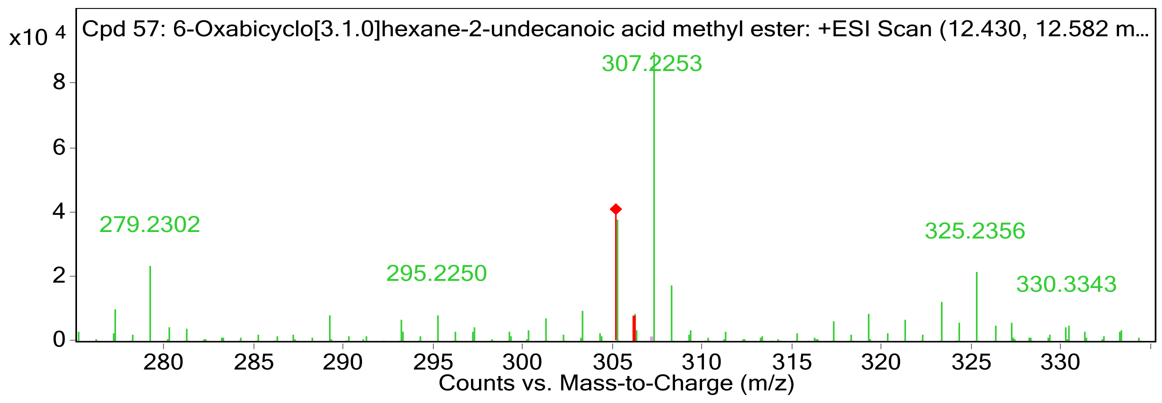

MS Spectrum Peak List

m/z	*Calc m/z*	Diff(ppm)	z	Abund	Formula	Ion
224.1275			1	16339.94		
235.2047				12708.56		
279.2302			1	23596.13		
305.2096	305.2087	-2.75	1	38227.81	C17 H30 O3	(M+Na)+
306.213	306.2121	-2.91	1	9017.36	C17 H30 O3	(M+Na)+
307.2253			1	89966.6		
308.2281			1	17669.37		
325.2356			1	21574		
337.2352			1	12654.52		
365.2284			1	14372.97		

MSMS Spectrum

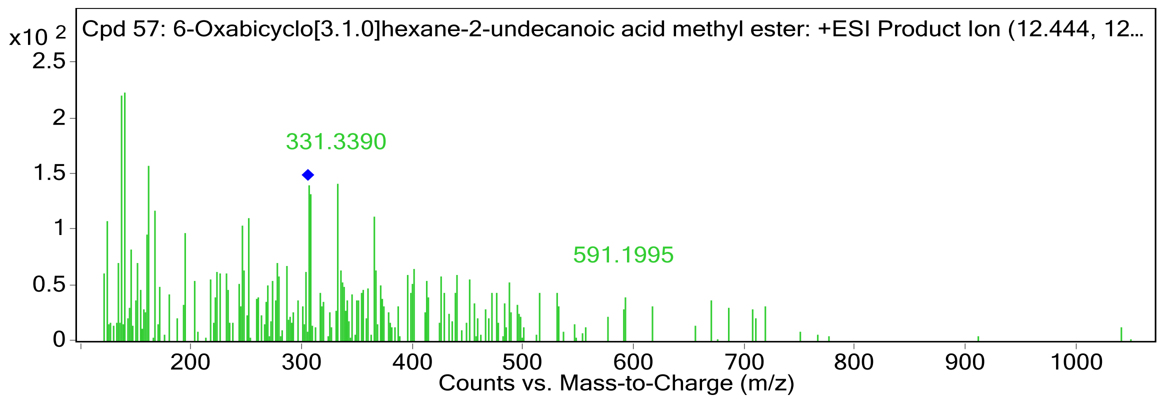

MS/MS Spectrum Peak List

m/z	Abund

123.0785	108.35
137.0589	220.68
139.0751	224.19
161.1309	157.73
166.091	117.53
251.2356	111.65
305.2076	140.41
307.2264	132.4
331.339	142.6
365.2308	113.05

Compound Structure

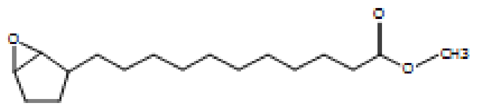

Compound Label	Name	*m/z*	RT	Algorithm	Mass
Cpd 58: Ximenoylacetone	**Ximenoylacetone**	457.3971	12.698	Auto MS/MS	434.408

MS Spectrum

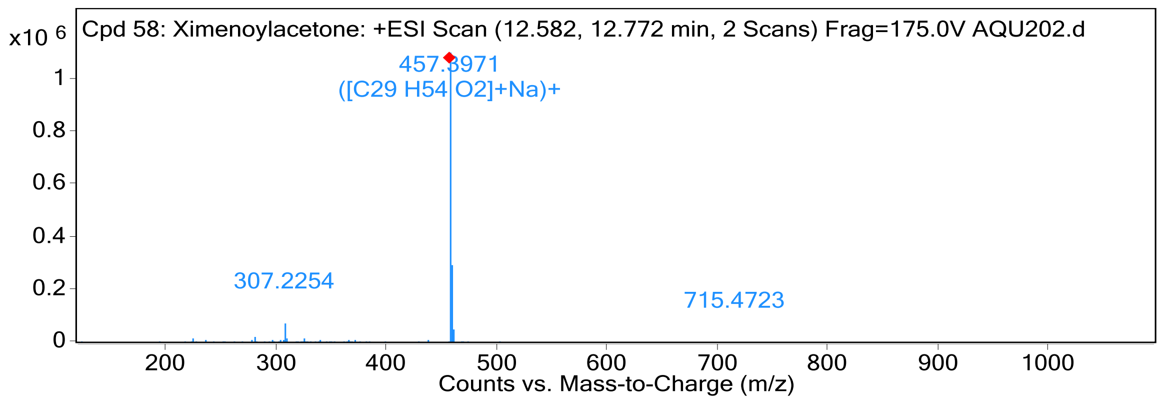

MS Zoomed Spectrum

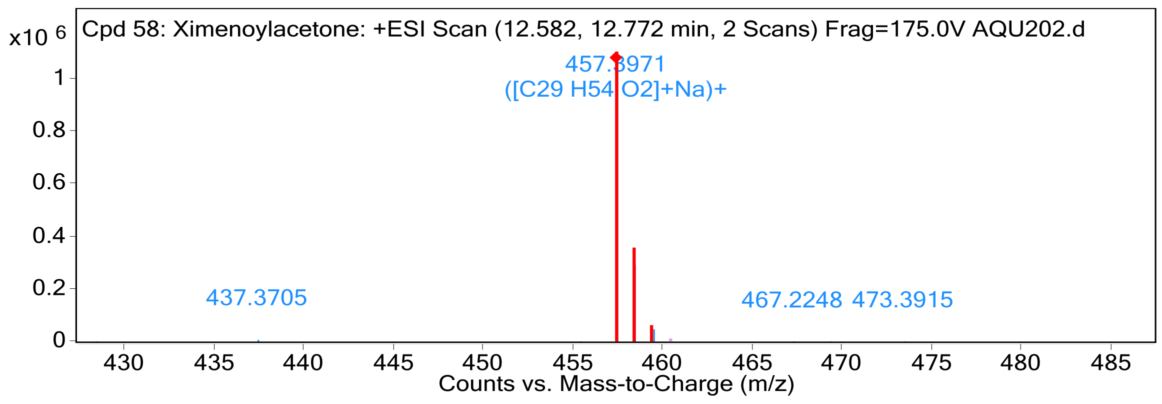

MS Spectrum Peak List

m/z	*Calc m/z*	Diff(ppm)	z	Abund	Formula	Ion
224.1274				14434.84		
235.2045			1	12969.46		
279.2303			1	21319.3		
307.2254			1	76262.85		
308.2284			1	15027.83		
325.2352			1	17213.86		
365.2289			1	12786.34		
457.3971	457.4016	9.75	1	1102466.38	C29 H54 O2	(M+Na)+
458.401	458.405	8.67	1	296686.63	C29 H54 O2	(M+Na)+
459.4036	459.4082	10.08	1	49841.8	C29 H54 O2	(M+Na)+

MSMS Spectrum

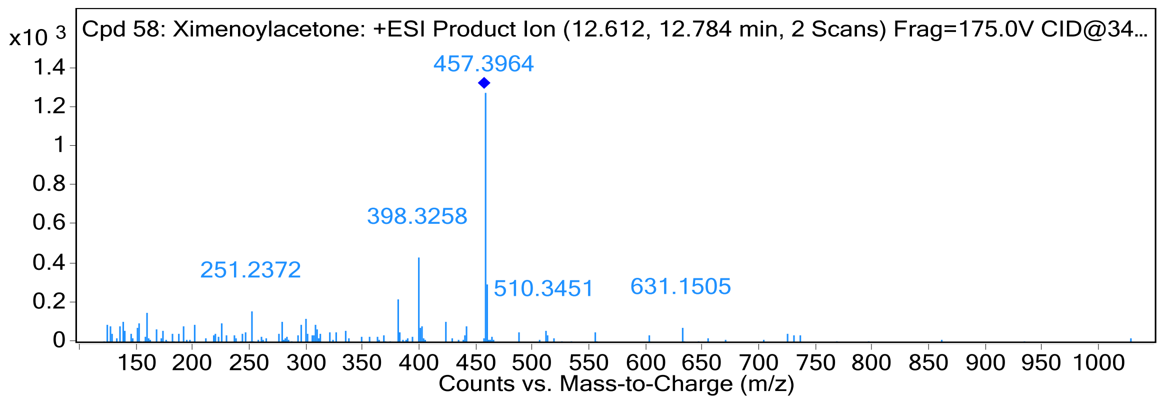

MS/MS Spectrum Peak List

m/z	z	Abund
158.0802	1	151.8
159.1162		124.24
251.2372		157.98
278.2451		109.49
299.2188		125.84
380.3128	1	224.37
398.3258	1	432.81
457.3964	1	1276.43
458.4018	1	294.75
459.408	1	123.36

Compound Structure

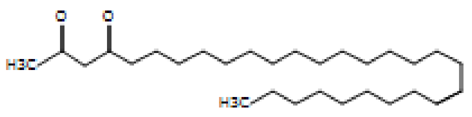

Compound Label	Name	*m/z*	RT	Algorithm	Mass
Cpd 59: Ximenoylacetone	**Ximenoylacetone**	457.3973	13.006	Auto MS/MS	434.408

MS Spectrum

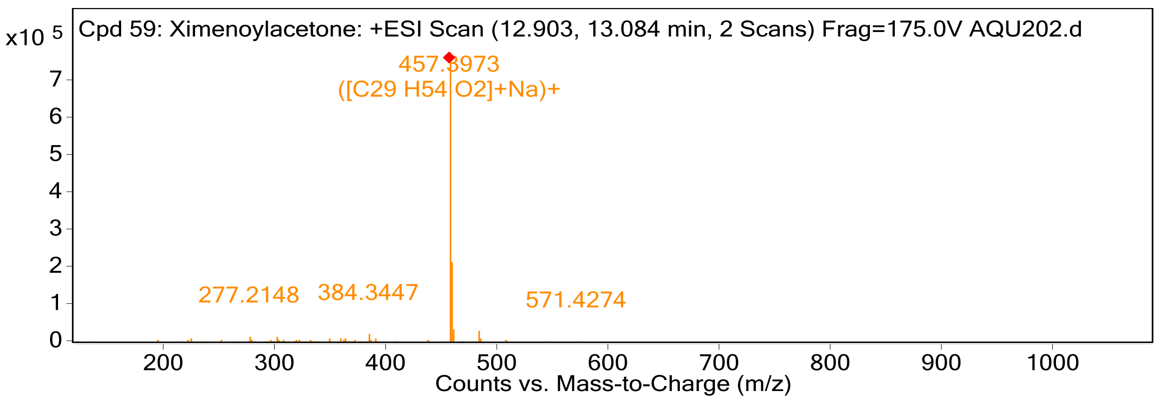

MS Zoomed Spectrum

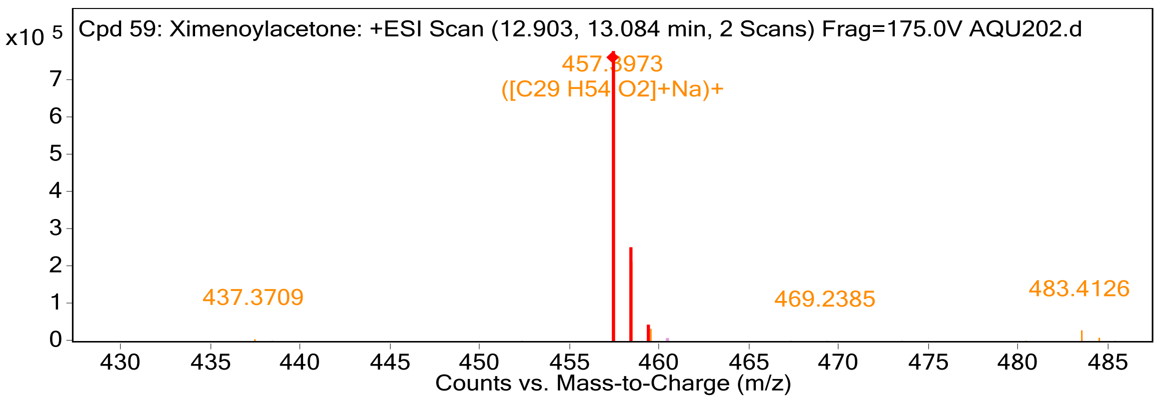

MS Spectrum Peak List

m/z	*Calc m/z*	Diff(ppm)	z	Abund	Formula	Ion
224.1275			1	13882.53		
277.2148			1	15716.79		
301.2148			1	15139.93		
349.2718			1	13106.41		
384.3447			1	22460.24		
390.3193			1	11610.57		
457.3973	457.4016	9.46	1	774810.13	C29 H54 O2	(M+Na)+
458.4007	458.405	9.44	1	213792.08	C29 H54 O2	(M+Na)+
459.4035	459.4082	10.25	1	35537.61	C29 H54 O2	(M+Na)+
483.4126			1	31219.94		

MSMS Spectrum

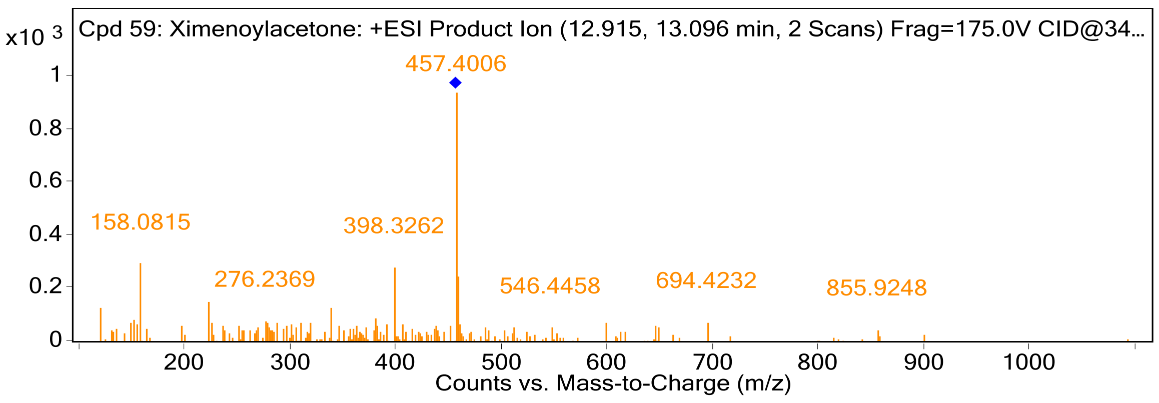

MS/MS Spectrum Peak List

m/z	z	Abund
120.0574		127.6
152.1084		84.42
158.0815		295.73
222.1137		149.73
337.2158		127.11
380.3139		88.82
398.3262		282.02
457.2287		80.33
457.4006	1	940.83
458.4037	1	246.35

Compound Structure

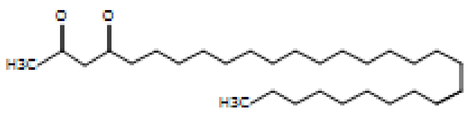

Compound Label	Name	*m/z*	RT	Algorithm	Mass

Cpd 60: 5-Pentacosyl-1,3-benzenediol	**5-Pentacosyl-1,3-benzenediol**	483.4122	13.077	Auto MS/MS	460.4229

MS Spectrum

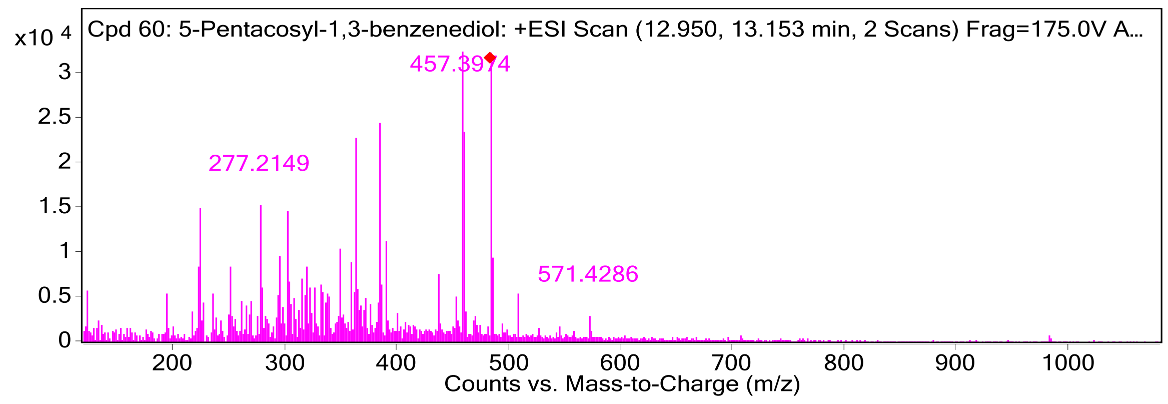

MS Zoomed Spectrum

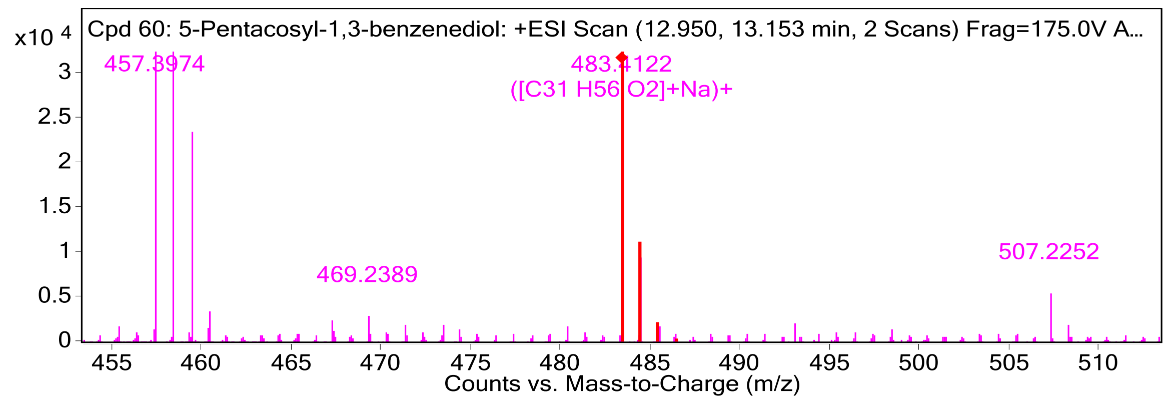

MS Spectrum Peak List

m/z	*Calc m/z*	Diff(ppm)	z	Abund	Formula	Ion
277.2149			1	15358.06		
363.2504			1	22850.69		
384.3448			1	24416.3		
457.3974			1	535457.75		
458.4006			1	145703.36		
459.4033			1	23534.61		
483.4122	483.4173	10.5	1	32279.99	C31 H56 O2	(M+Na)+
484.4154	484.4207	10.86	1	9535.67	C31 H56 O2	(M+Na)+
485.42	485.4239	8.06	1	1767.87	C31 H56 O2	(M+Na)+
486.4181	486.427	18.22	1	411.58	C31 H56 O2	(M+Na)+

MSMS Spectrum

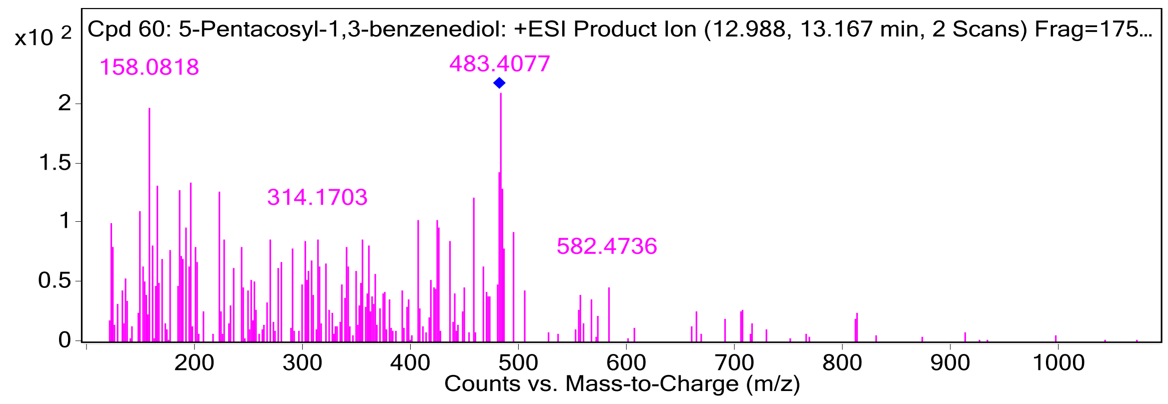

MS/MS Spectrum Peak List

m/z	z	Abund
149.1336		110.7
158.0818		196.9
165.1245		132
185.1317		127.68
196.0775		134.31
222.1183		126.84
457.3972		121.76
481.2523		143.69
483.4077	1	210.41
484.4032	1	128.97

Compound Structure

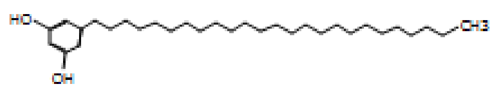

**Compound Label**	**Name**	***m/z***	**RT**	**Algorithm**	**Mass**
Cpd 61: Ximenoylacetone	**Ximenoylacetone**	457.3972	13.332	Auto MS/MS	434.4079

MS Spectrum

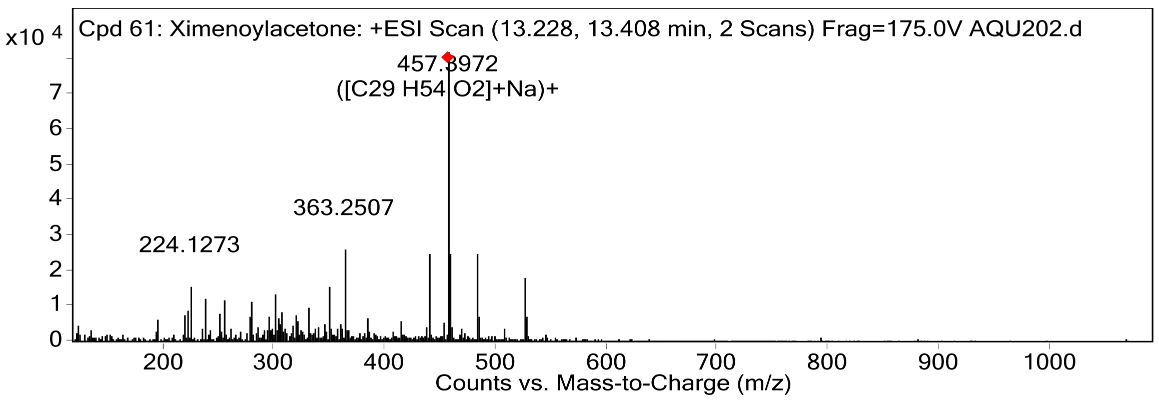

MS Zoomed Spectrum

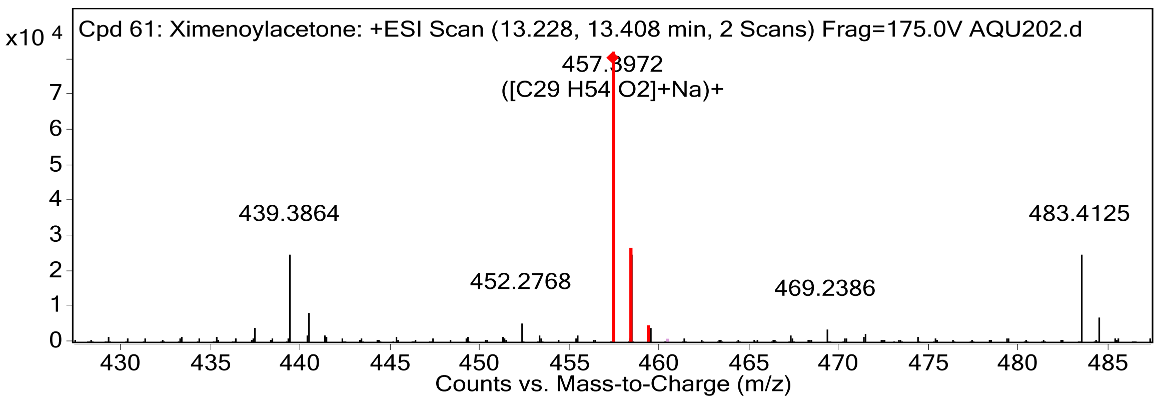

MS Spectrum Peak List

m/z	*Calc m/z*	Diff(ppm)	z	Abund	Formula	Ion
224.1273			1	15749.55		
301.2148			1	13380.63		
349.2712			1	15429.63		
363.2507			1	26262.33		
439.3864			1	24802.94		
457.3972	457.4016	9.57	1	81909.86	C29 H54 O2	(M+Na)+
458.4002	458.405	10.55	1	24738.57	C29 H54 O2	(M+Na)+
459.4027	459.4082	12.03	1	4077.78	C29 H54 O2	(M+Na)+
483.4125			1	24790.68		
526.4219			1	18147.06		

MSMS Spectrum

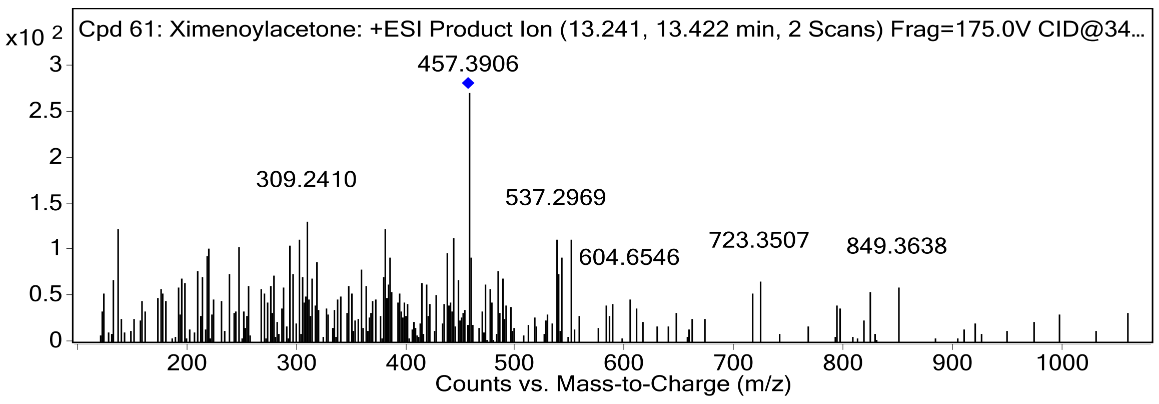

MS/MS Spectrum Peak List

m/z	z	Abund
136.119		123.11
247.168		104.04
293.2715		104.91
302.136		111.73
309.241		132.16
380.3113		123.16
443.3093		113.7
457.3906	1	271.53
537.2969		112.49
550.2165		112.43

Compound Structure

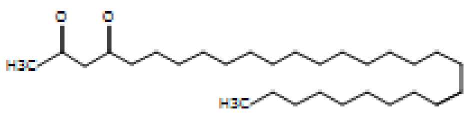

Compound Label	Name	*m/z*	RT	Algorithm	Mass
Cpd 62: Stigmastanol	**Stigmastanol**	439.3865	13.403	Auto MS/MS	416.3973

MS Spectrum

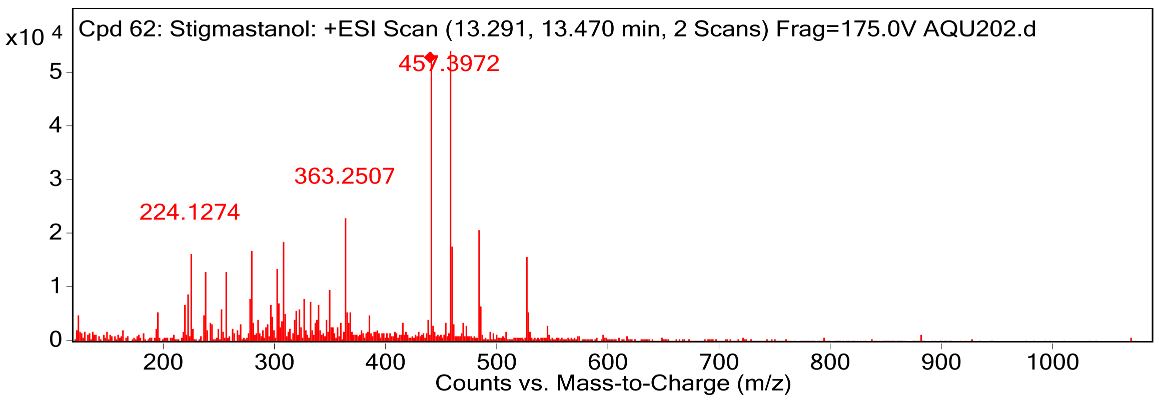

MS Zoomed Spectrum

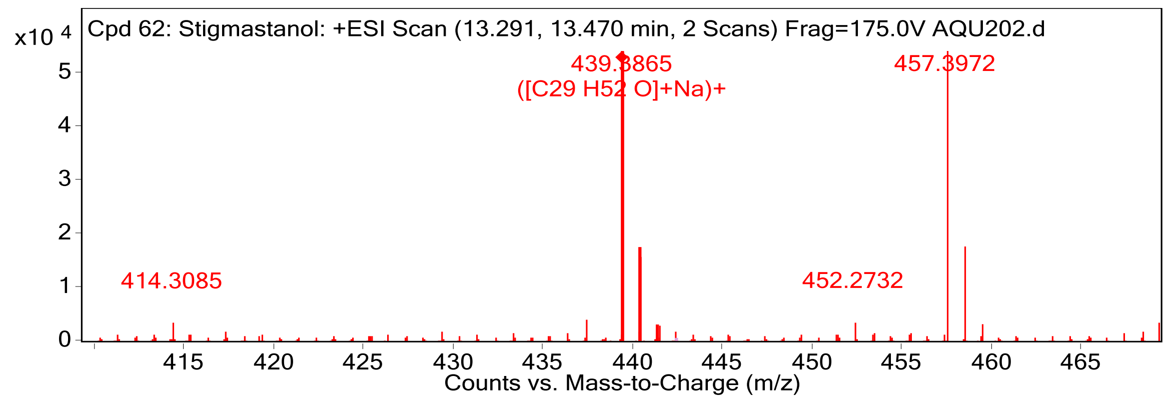

MS Spectrum Peak List

m/z	*Calc m/z*	Diff(ppm)	z	Abund	Formula	Ion
224.1274			1	16465.94		
279.2302			1	17003.13		
307.2253			1	18660.65		
363.2507			1	23192.29		
439.3865	439.391	10.33	1	53957.29	C29 H52 O	(M+Na)+
440.3902	440.3944	9.54	1	15957.51	C29 H52 O	(M+Na)+
441.393	441.3978	10.75	1	3072.87	C29 H52 O	(M+Na)+
457.3972			1	60844.75		
458.4004			1	17915.09		
483.4125			1	20996.09		

MSMS Spectrum

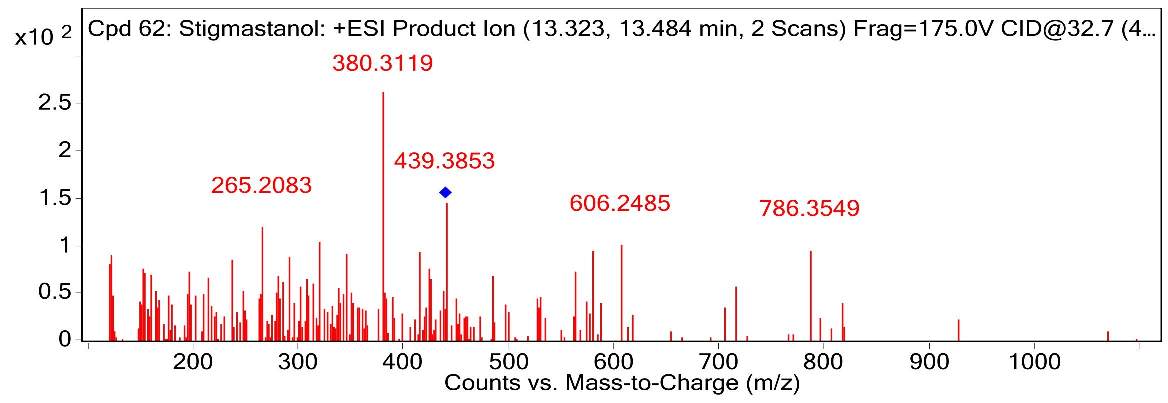

MS/MS Spectrum Peak List

m/z	z	Abund
265.2083		121.27
319.2257		105.13
344.2933		93.01
380.3119	1	263.75
414.2388		94.54
439.3853		147.12
440.3917		94.3
579.3885		96.52
606.2485		102.26
786.3549		96.9

Compound Structure

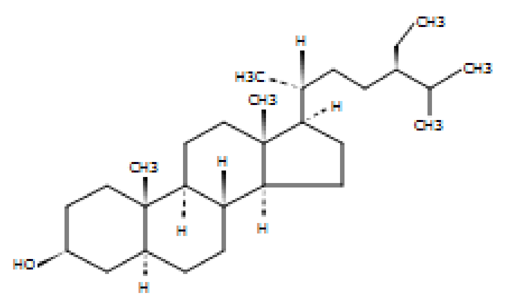

Compound Label	Name	*m/z*	RT	Algorithm	Mass
Cpd 63: 5-Pentacosyl-1,3-benzenediol	**5-Pentacosyl-1,3-benzenediol**	483.4125	13.405	Auto MS/MS	460.4233

MS Spectrum

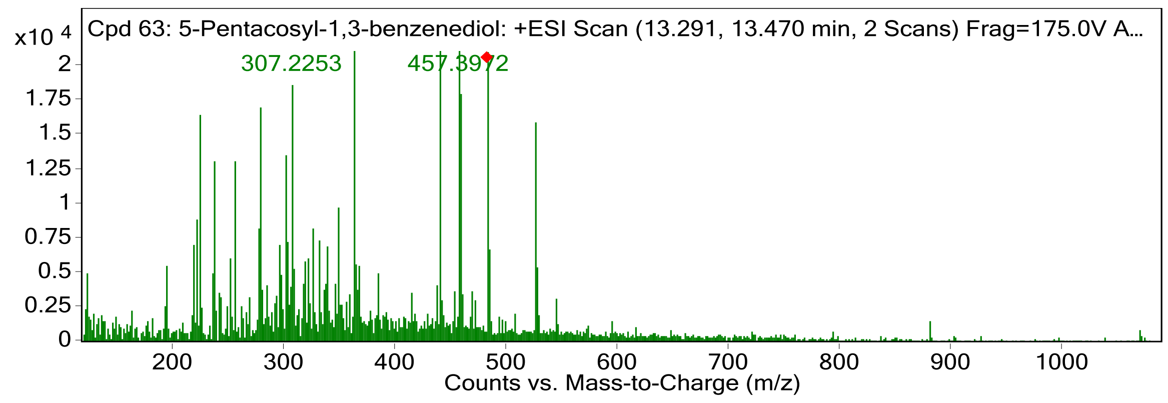

MS Zoomed Spectrum

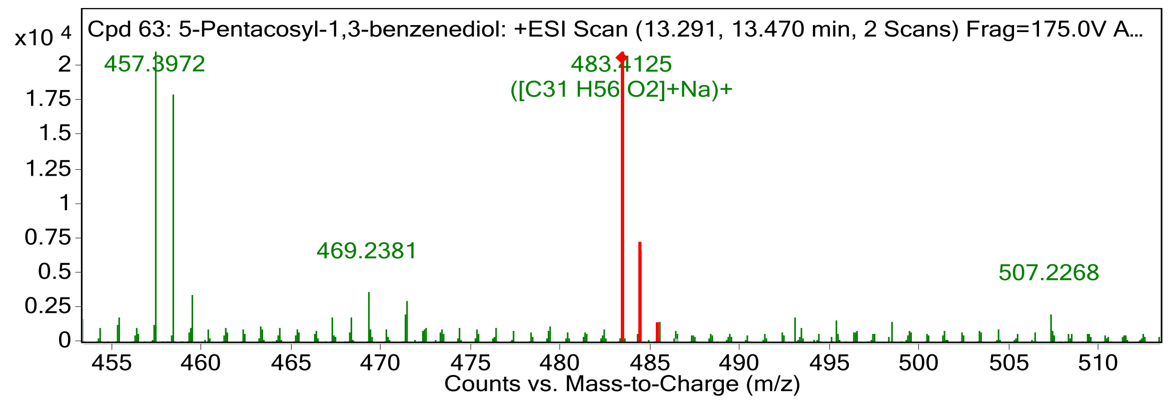

MS Spectrum Peak List

m/z	*Calc m/z*	Diff(ppm)	z	Abund	Formula	Ion
224.1274			1	16465.94		

279.2302			1	17003.13		
307.2253			1	18660.65		
363.2507			1	23192.29		
439.3865			1	53957.29		
457.3972			1	60844.75		
458.4004			1	17915.09		
483.4125	483.4173	9.91	1	20996.09	C31 H56 O2	(M+Na)+
484.416	484.4207	9.56	1	6701.83	C31 H56 O2	(M+Na)+
485.4188	485.4239	10.51	1	1473.09	C31 H56 O2	(M+Na)+

MSMS Spectrum

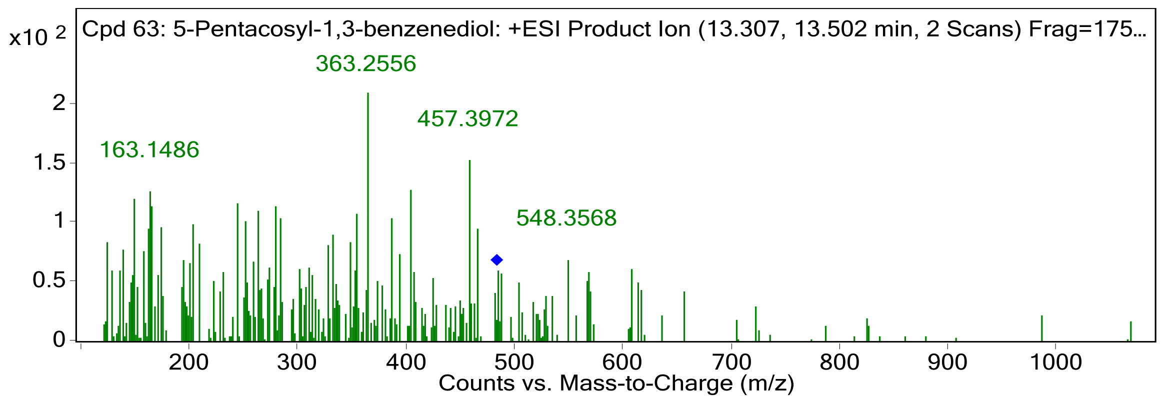

MS/MS Spectrum Peak List

m/z	z	Abund
149.1337		120.36
163.1486		127
165.1284		114.42
243.1327		117.07
263.1986		110.02
279.2285		114.29
354.1876		107.56
363.2556		210.23
403.249		127.91
457.3972	1	153.16

Compound Structure

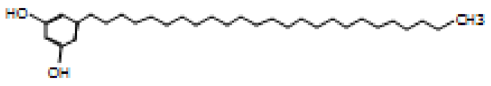

Compound Label	Name	*m/z*	RT	Algorithm	Mass
Cpd 64: Stigmastanol	**Stigmastanol**	439.387	13.747	Auto MS/MS	416.3976

MS Spectrum

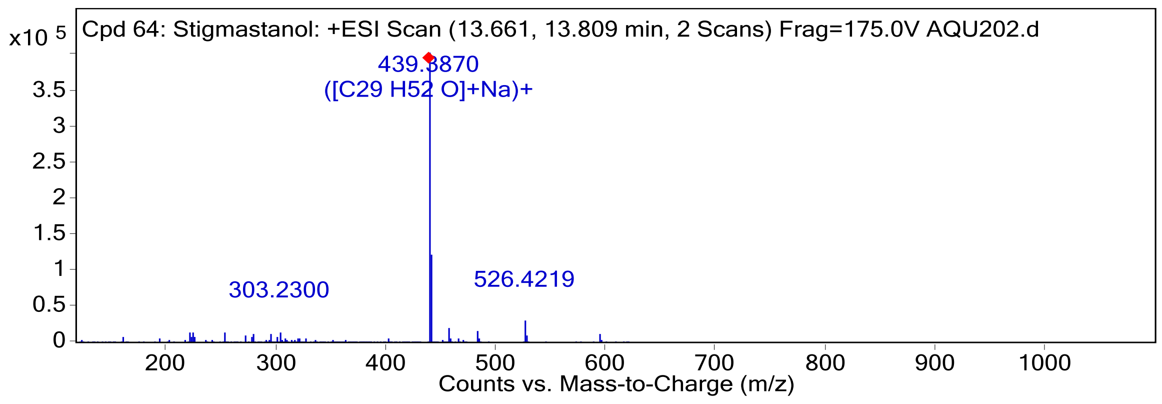

MS Zoomed Spectrum

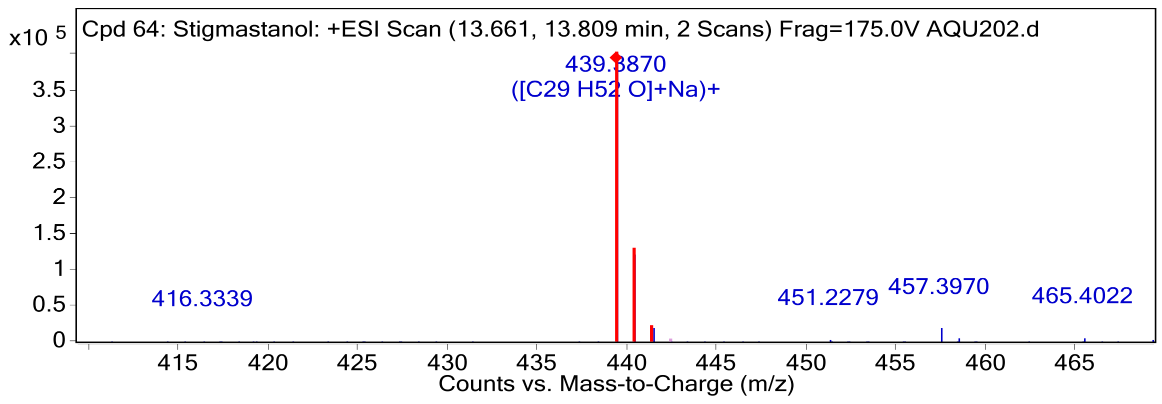

MS Spectrum Peak List

m/z	*Calc m/z*	Diff(ppm)	z	Abund	Formula	Ion
221.1162				15135.61		
224.1273			1	15076.55		
253.2148			1	14746.79		
303.23			1	15437.92		
439.387	439.391	9.29	1	402995.41	C29 H52 O	(M+Na)+
440.39	440.3944	10.2	1	122439.67	C29 H52 O	(M+Na)+
441.3929	441.3978	11	1	20530.56	C29 H52 O	(M+Na)+
457.397			1	20111.6		
483.4115			1	16678.34		
526.4219			1	30308		

MSMS Spectrum

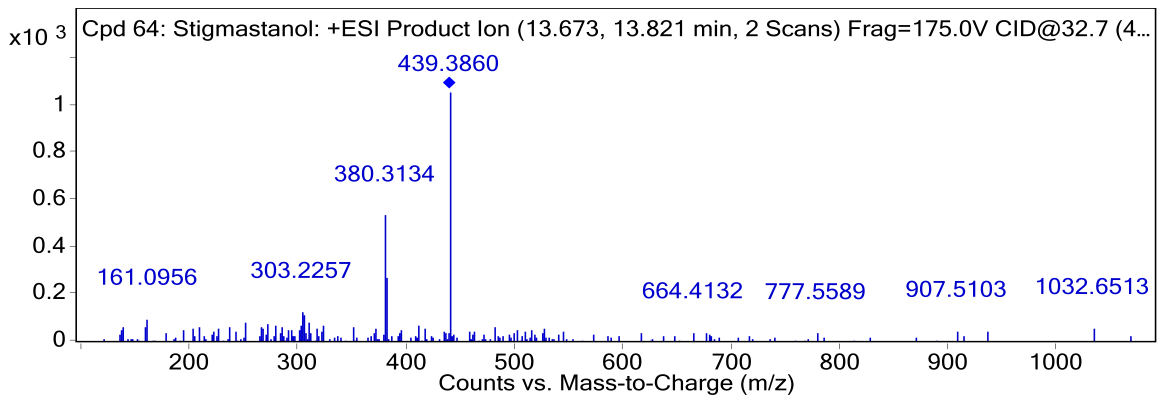

MS/MS Spectrum Peak List

m/z	z	Abund
161.0956		97.53
251.2324		80.01
303.2257	1	126.55
305.2987		112.23
309.2378		84.21
380.3134	1	535.82
381.2553		115.56
381.3163	1	270.06
439.386	1	1056.05
440.3912	1	380.62

Compound Structure

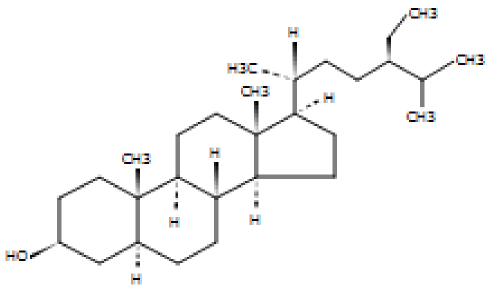

Compound Label	Name	*m/z*	RT	Algorithm	Mass
Cpd 65: (Z)-22-Hentriacontene-2,4-dione	**(Z)-22-Hentriacontene-2,4-dione**	485.4285	14.043	Auto MS/MS	462.4391

MS Spectrum

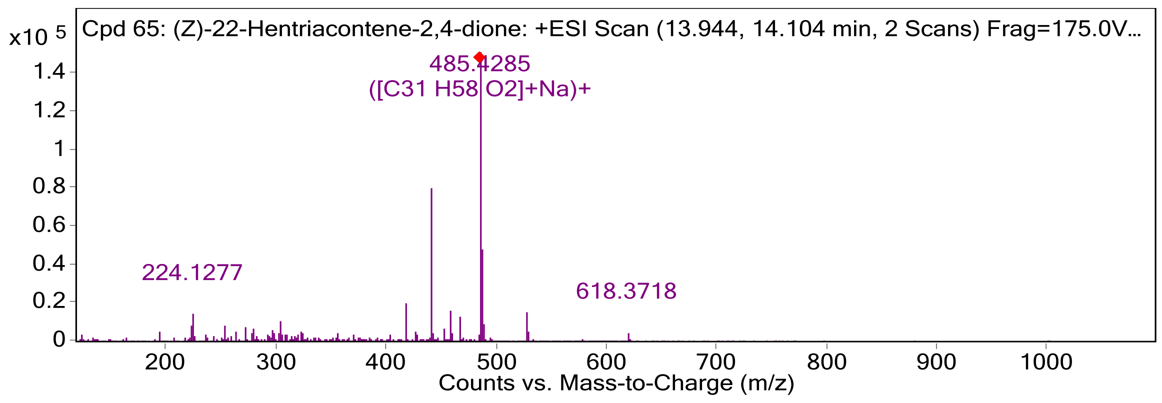

MS Zoomed Spectrum

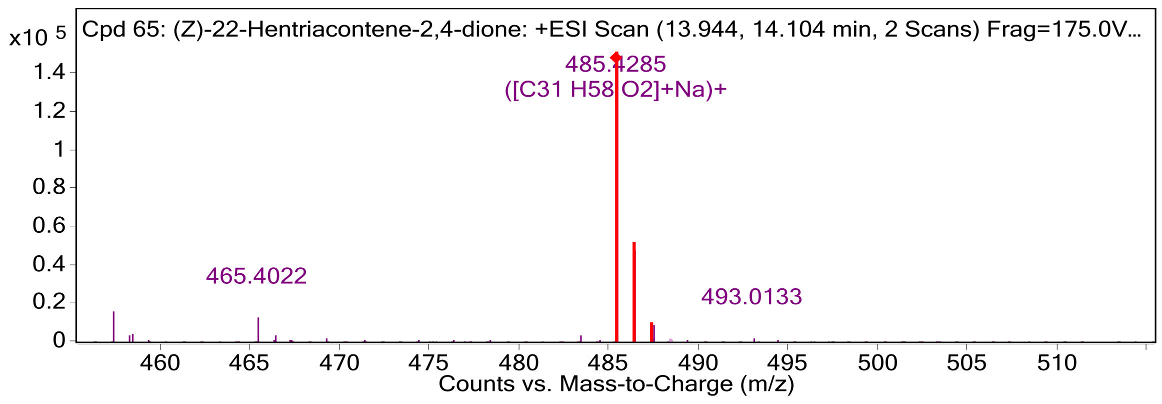

MS Spectrum Peak List

m/z	*Calc m/z*	Diff(ppm)	z	Abund	Formula	Ion
224.1277			1	14446.97		
416.3343			1	20204.87		
439.3866			1	80364.75		
440.3898			1	25138.02		
457.3967			1	16559.37		
465.4022			1	12997.85		
485.4285	485.4329	9.07	1	151165.69	C31 H58 O2	(M+Na)+
486.4314	486.4363	10.06	1	48608.23	C31 H58 O2	(M+Na)+
487.4346	487.4396	10.17	1	9223.56	C31 H58 O2	(M+Na)+
526.4219			1	15884.69		

MSMS Spectrum

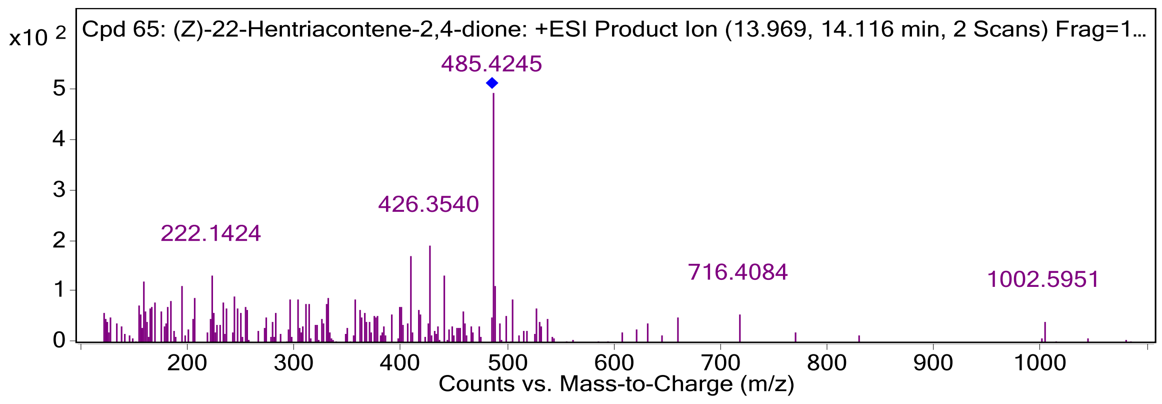

MS/MS Spectrum Peak List

m/z	z	Abund
158.0805		120.82

193.1579		111.51
222.1424		134.28
242.2814		92.83
408.3429		171.18
426.354		192.22
439.3823		133.63

m/z	z	Abund
485.4245	1	496.16
486.431	1	112.45
487.435	1	102.58

Compound Structure

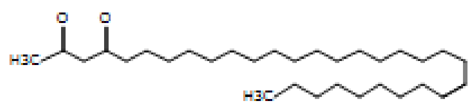

Compound Label	Name	*m/z*	RT	Algorithm	Mass
Cpd 66: Stigmastanol	**Stigmastanol**	439.3864	14.092	Auto MS/MS	416.3972

MS Spectrum

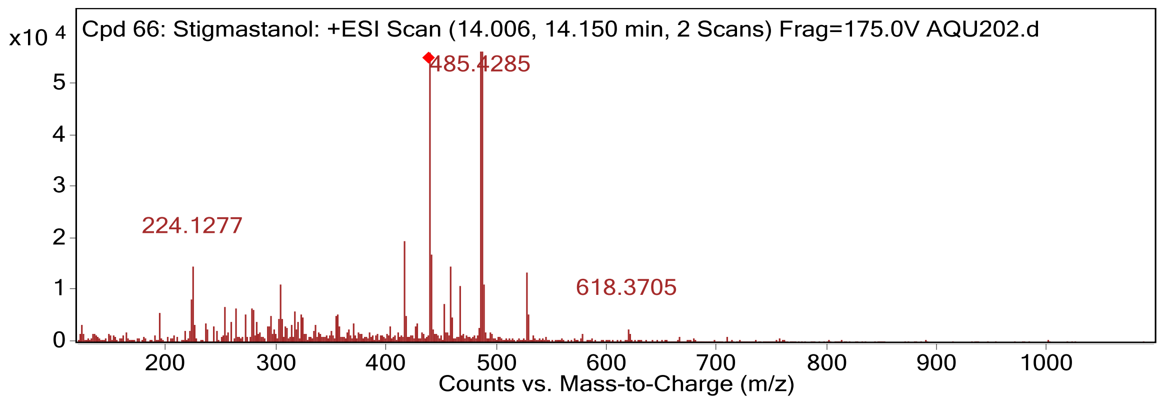

MS Zoomed Spectrum

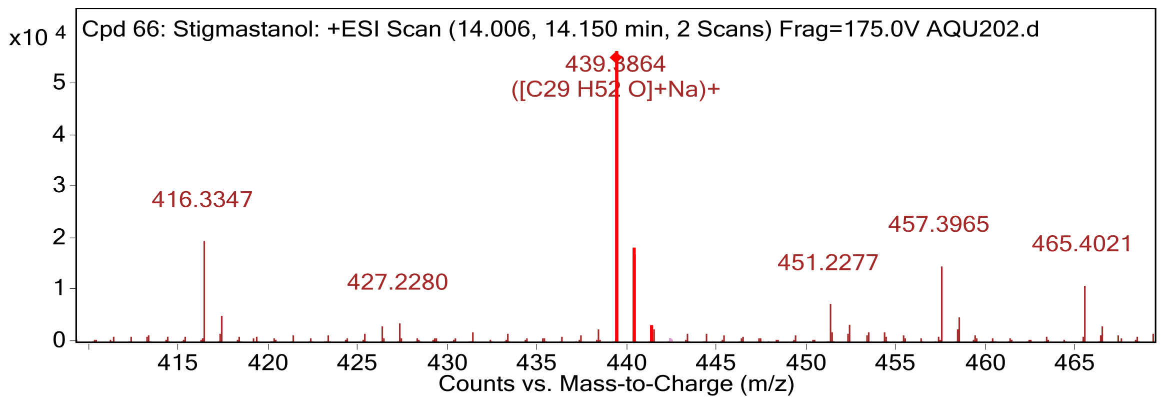

MS Spectrum Peak List

m/z	*Calc m/z*	Diff(ppm)	z	Abund	Formula	Ion
224.1277			1	14618.75		
416.3347			1	19643.96		
439.3864	439.391	10.45	1	56120.41	C29 H52 O	(M+Na)+
440.3898	440.3944	10.49	1	17183.75	C29 H52 O	(M+Na)+
441.3934	441.3978	9.89	1	2583.85	C29 H52 O	(M+Na)+
457.3965			1	14852.02		
485.4285			1	203978.02		
486.4316			1	63830.47		
487.4345			1	11408.21		
526.422			1	13559.11		

MSMS Spectrum

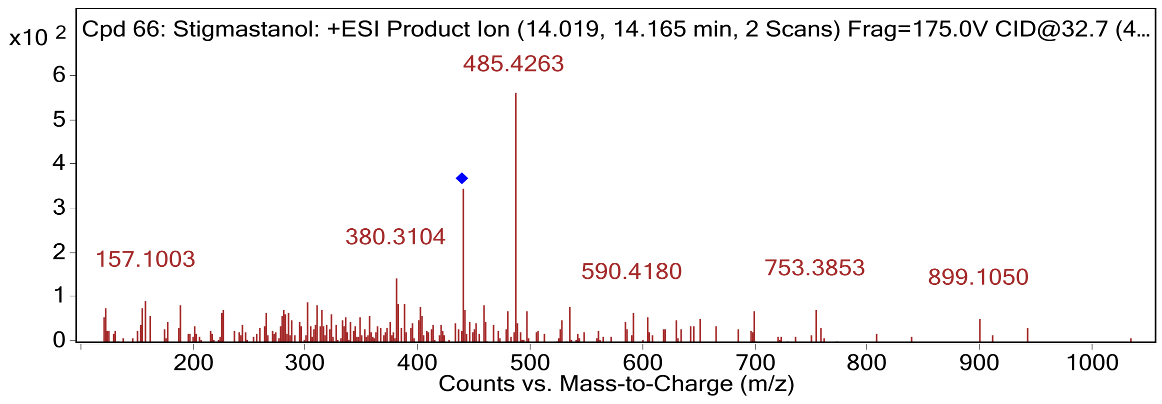

MS/MS Spectrum Peak List

m/z	z	Abund
157.1003		94.14
187.1484		85.12
301.2179		92.03
380.3104		144.77
381.3208		88.92
386.2856		88.33
439.3873		348.63
457.3056		84.6
485.4263	1	564.52
486.4298	1	241.08

Compound Structure

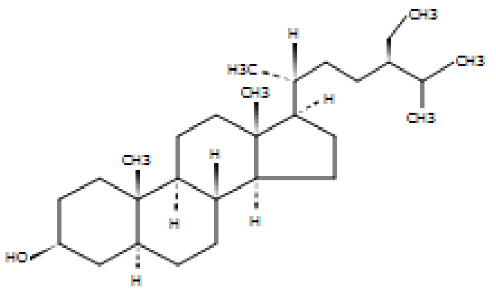

Compound Label	Name	*m/z*	RT	Algorithm	Mass
Cpd 67: Hydroflumethiazide	**Hydroflumethiazide**	353.9832	14.31	Auto MS/MS	330.9939

MS Spectrum

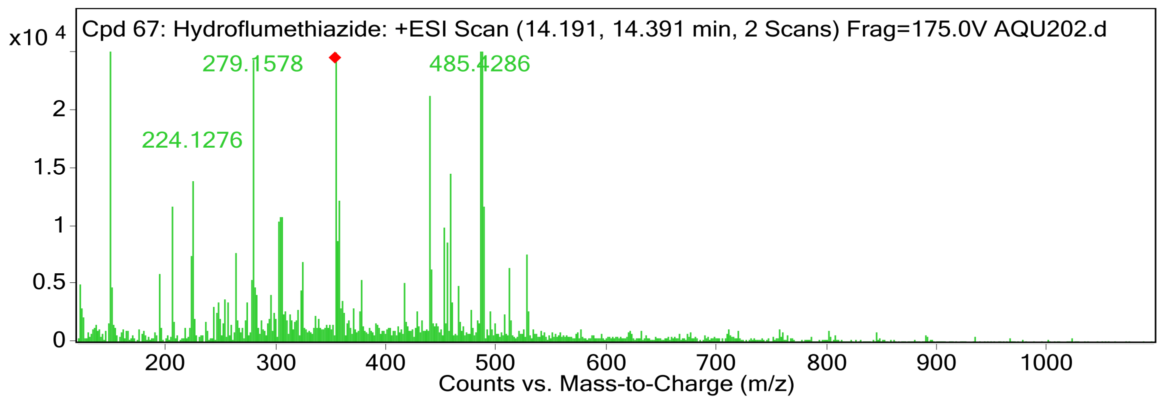

MS Zoomed Spectrum

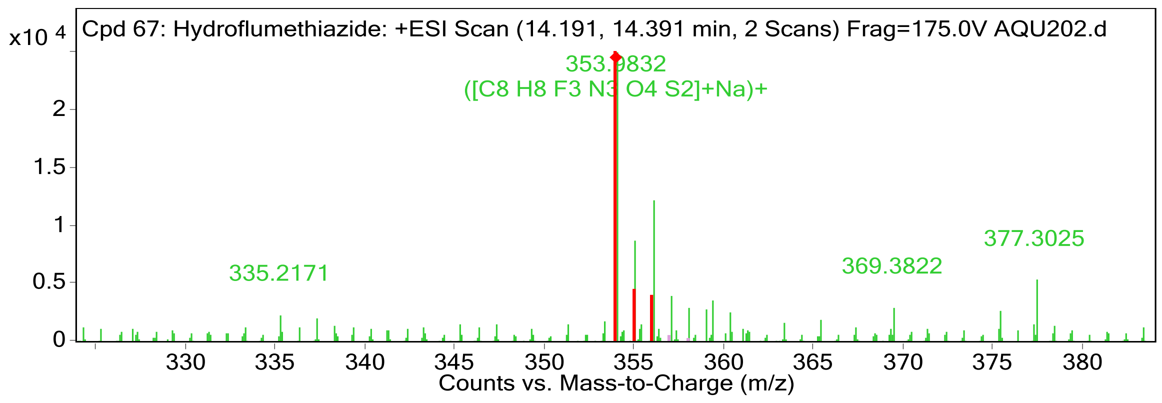

MS Spectrum Peak List

m/z	*Calc m/z*	Diff(ppm)	z	Abund	Formula	Ion
149.0232			1	65996.14		
224.1276			1	13915.3		
279.1578			1	24352.86		
353.9832	353.9801	-8.93	1	25040.8	C8 H8 F3 N3 O4 S2	(M+Na)+
354.9854	354.9823	-8.78	1	8839	C8 H8 F3 N3 O4 S2	(M+Na)+
355.9798	355.977	-7.84	1	12258.58	C8 H8 F3 N3 O4 S2	(M+Na)+
439.3862			1	21342.87		
457.3969			1	14585.12		
485.4286			1	201207.06		
486.4314			1	64320.14		

MSMS Spectrum

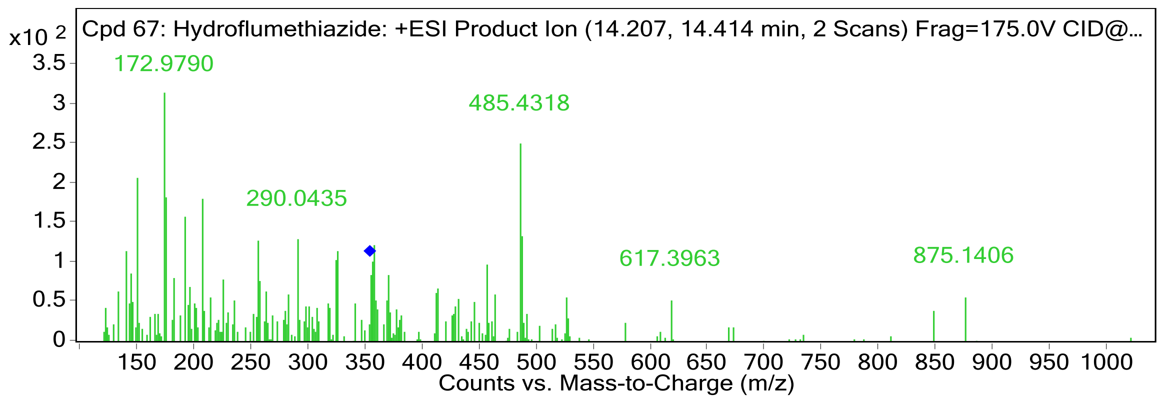

MS/MS Spectrum Peak List

m/z	z	Abund
149.023		206.41
172.979		315.03
173.9862		182.41
190.9934		157.26
206.9656	1	181.32
255.2266		128.7
290.0435	1	128.98
357.3528		122.62
485.4318	1	249.56
486.4279	1	133.5

Compound Structure

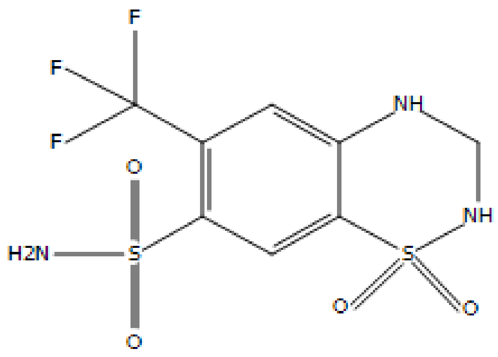

Compound Label	Name	*m/z*	RT	Algorithm	Mass

Cpd 68: (Z)-22-Hentriacontene-2,4-dione	**(Z)-22-Hentriacontene-2,4-dione**	485.4284	14.38	Auto MS/MS	462.439

MS Spectrum

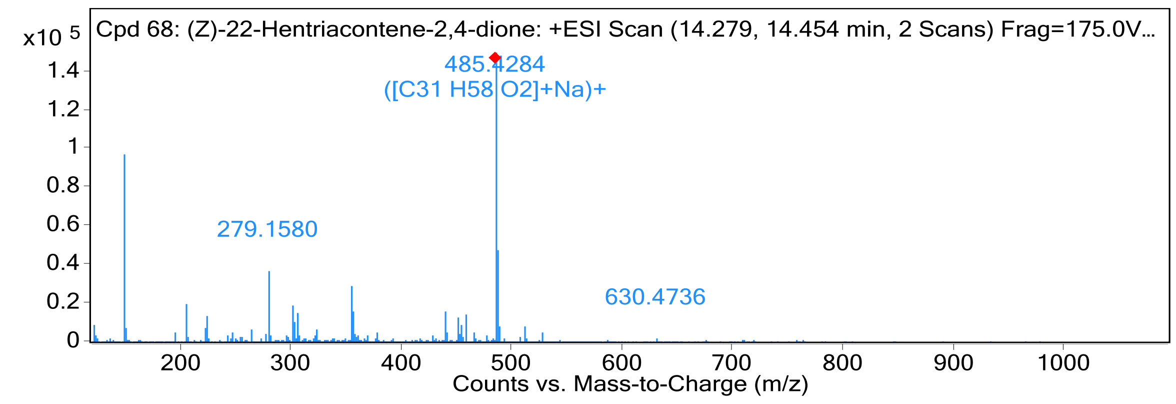

MS Zoomed Spectrum

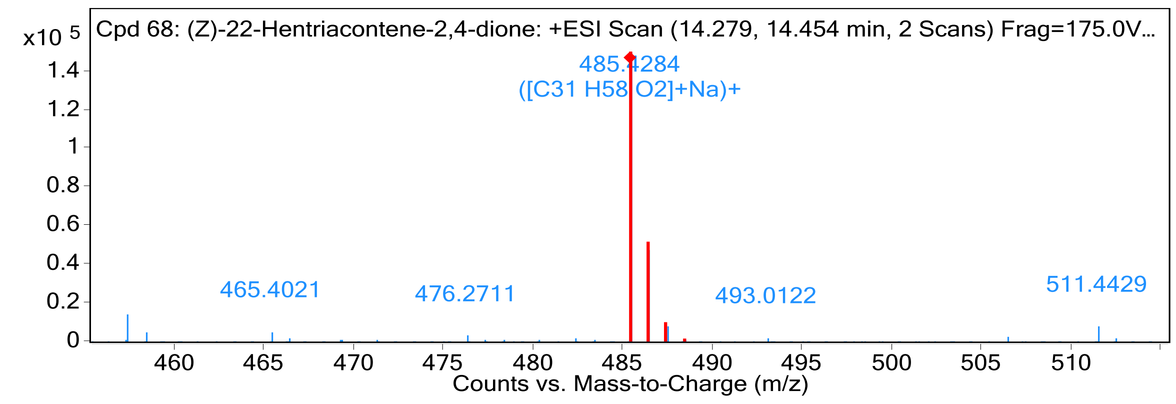

MS Spectrum Peak List

m/z	*Calc m/z*	Diff(ppm)	z	Abund	Formula	Ion
149.0232			1	97361.48		
205.0852			1	20090.27		
279.158			1	37159.82		
301.1394			1	19058.62		
353.9834			1	29167.07		
439.3863			1	16582.78		
485.4284	485.4329	9.25	1	149789.72	C31 H58 O2	(M+Na)+
486.4313	486.4363	10.31	1	48175.59	C31 H58 O2	(M+Na)+
487.4343	487.4396	10.74	1	8107.22	C31 H58 O2	(M+Na)+
488.4352	488.4427	15.33	1	1711.56	C31 H58 O2	(M+Na)+

MSMS Spectrum

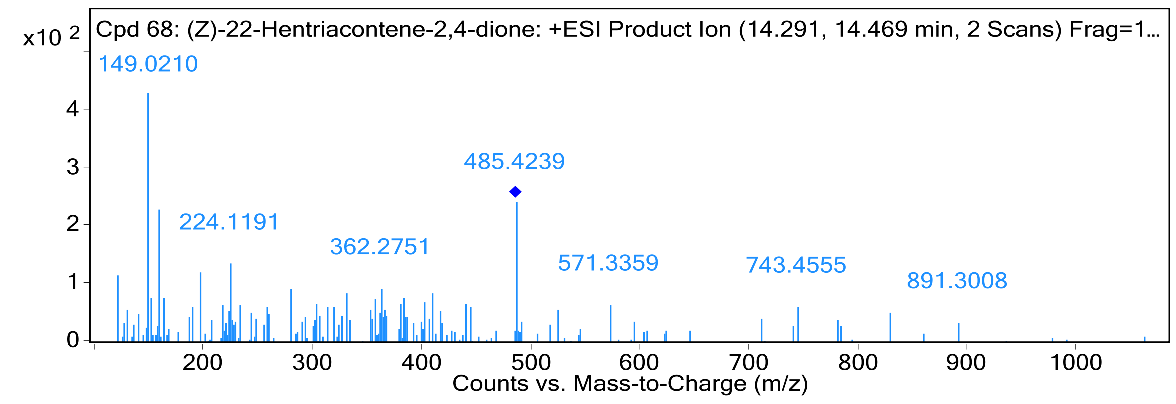

MS/MS Spectrum Peak List

m/z	z	Abund
121.0297		116.66
149.021		431.2
158.0804		228.96
197.0841		120.12
224.1191	1	136.24
279.1592		92.28
362.2751		93.33
408.3411		84.17
485.4239	1	241.03
486.4272	1	132.72

Compound Structure

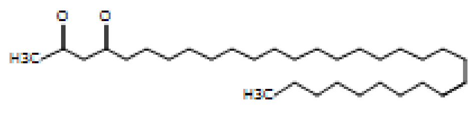

Compound Label	Name	*m/z*	RT	Algorithm	Mass
Cpd 69: Methyl 2-furoate	**Methyl 2-furoate**	149.0232	14.382	Auto MS/MS	126.0339

MS Spectrum

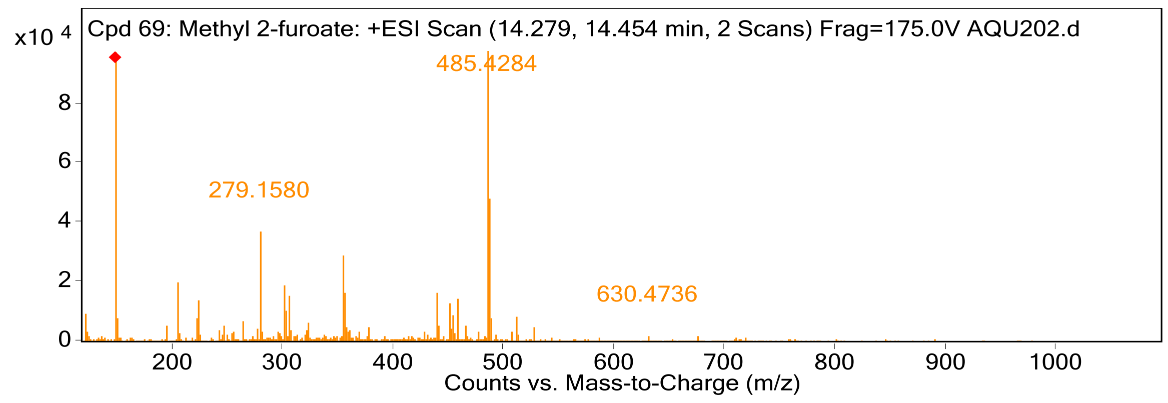

MS Zoomed Spectrum

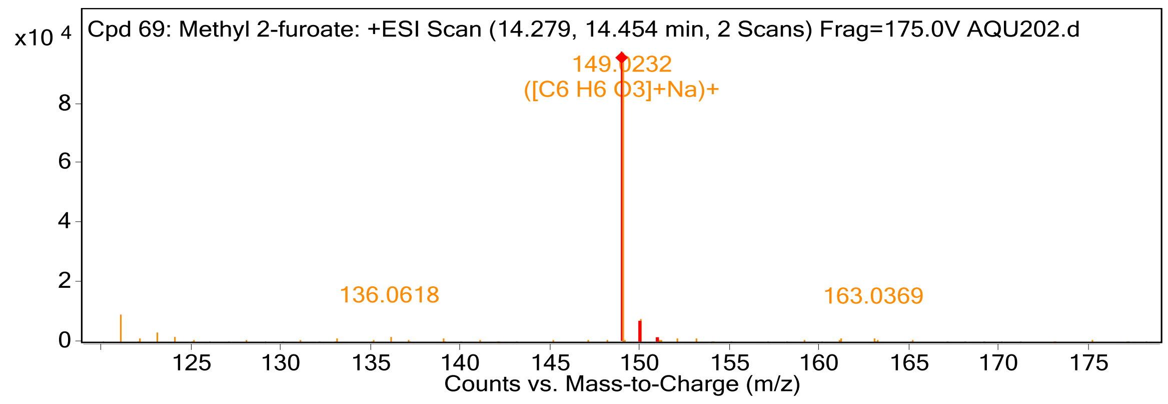

MS Spectrum Peak List

m/z	*Calc m/z*	Diff(ppm)	z	Abund	Formula	Ion
149.0232	149.0209	-15.12	1	97361.48	C6 H6 O3	(M+Na)+
150.0265	150.0243	-14.49	1	7844.48	C6 H6 O3	(M+Na)+
151.0281	151.0258	-15.58	1	1250.78	C6 H6 O3	(M+Na)+
205.0852			1	20090.27		
279.158			1	37159.82		
301.1394			1	19058.62		
353.9834			1	29167.07		
439.3863			1	16582.78		
485.4284			1	149789.72		
486.4313			1	48175.59		

MSMS Spectrum

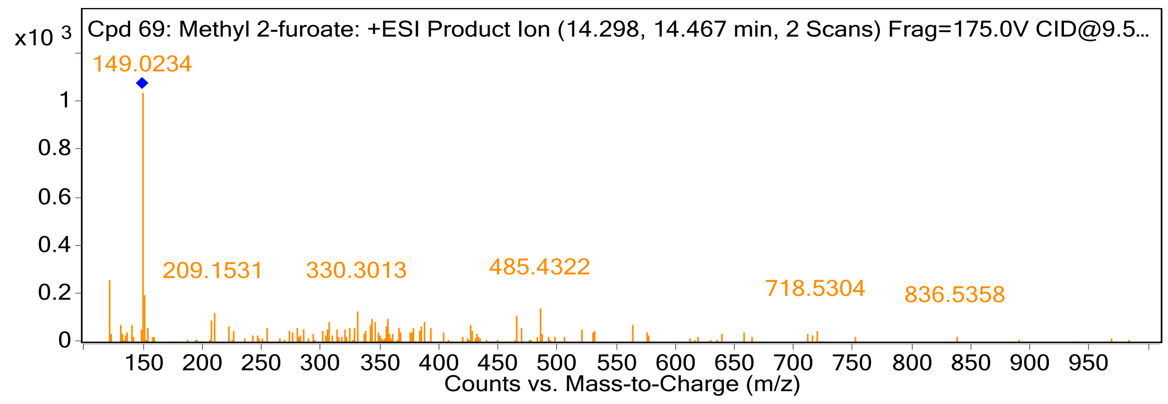

MS/MS Spectrum Peak List

m/z	*Calc m/z*	Diff (ppm)	z	Abund	Formula	Ion
121.0287				263.43		
149.0234			1	1040.86		
150.0265	150.0243	-14.29	1	196.77	C6 H6 O3	(M+Na)+
207.1378				95.55		
209.1531				127.07		
330.3013				128.13		
342.1856				100.05		
355.979				99.74		
464.1978				109.63		
485.4322			1	142.14		

Compound Structure

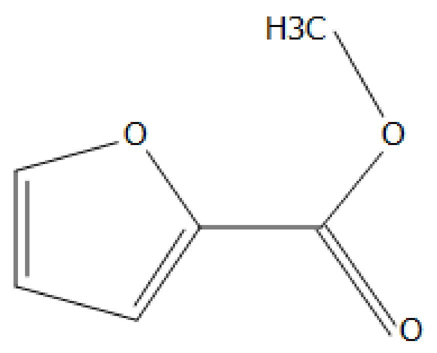

Compound Label	*m/z*	RT	Algorithm
Compound 70	467.4178	14.984	Auto MS/MS

MS Spectrum

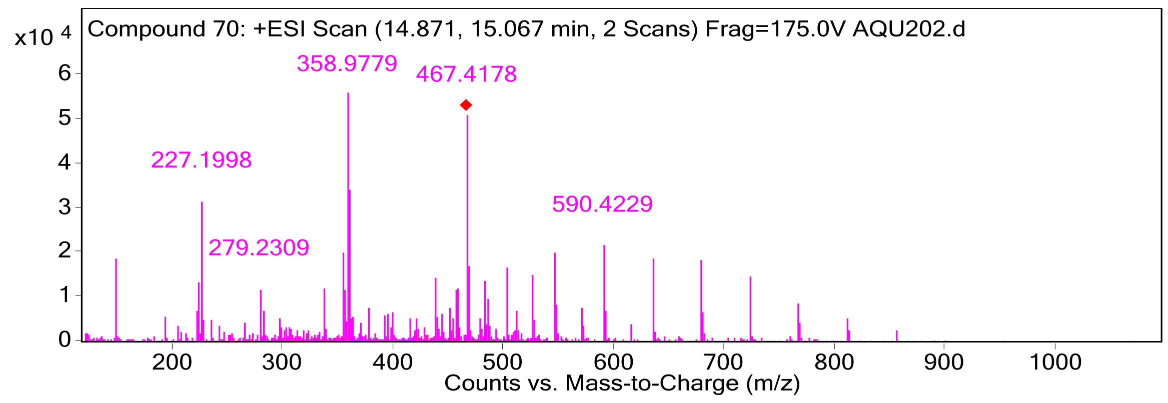

MS Zoomed Spectrum

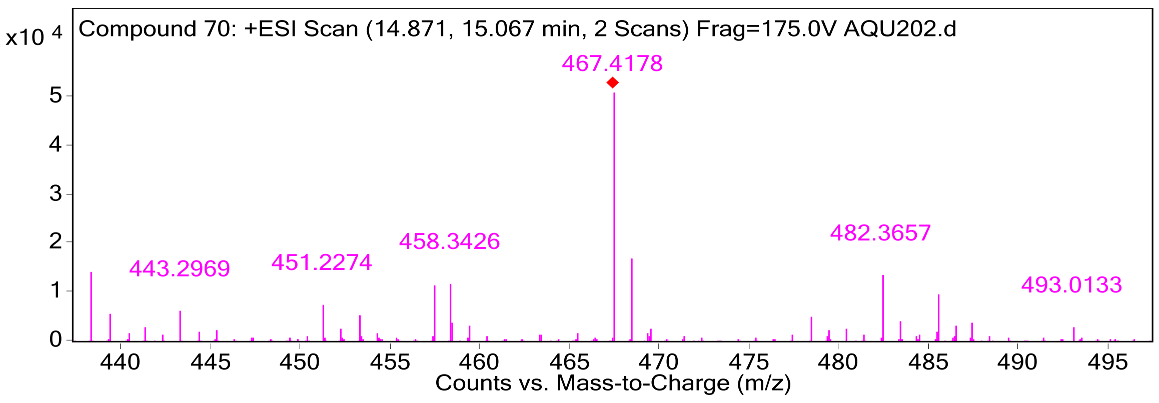

MS Spectrum Peak List

m/z	z	Abund
227.1998	1	31641.97
354.9822		20079.03
358.9779	1	56122.55
360.9758	1	34382.91
467.4178	1	50987.11
468.421	1	17040.32
469.425	1	2658.87
546.397	1	20157.13
590.4229	1	21678.04
634.4488	1	18934.17

MSMS Spectrum

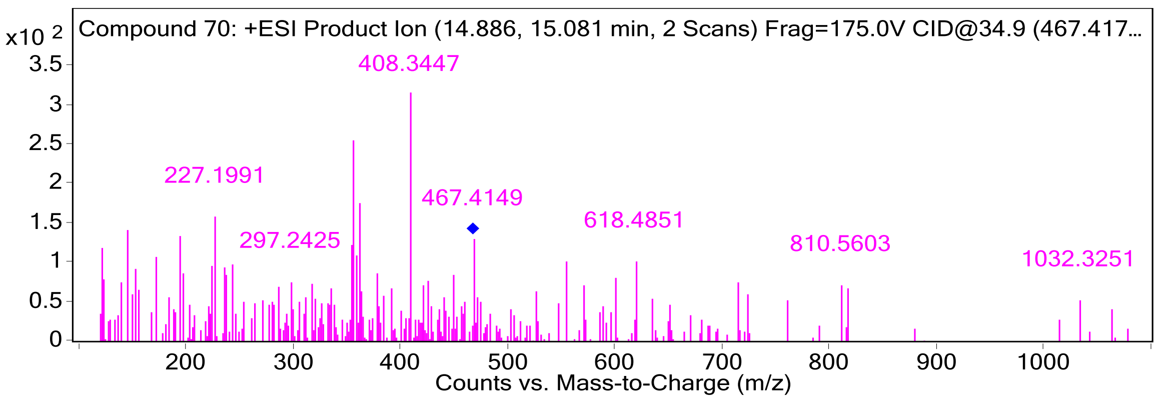

MS/MS Spectrum Peak List

m/z	z	Abund
121.0291		118.68
145.1		141.57
193.1981		133.93
227.1991		158.88
353.987		123.6
354.9853		255.1
360.9749		175.52
408.3447	1	316.84
409.3399	1	109.71
467.4149		130.24

Compound Label	Name	*m/z*	RT	Algorithm	Mass
Cpd 71: Chlorfenvinphos	**Chlorfenvinphos**	358.978	15.072	Auto MS/MS	357.9707

MS Spectrum

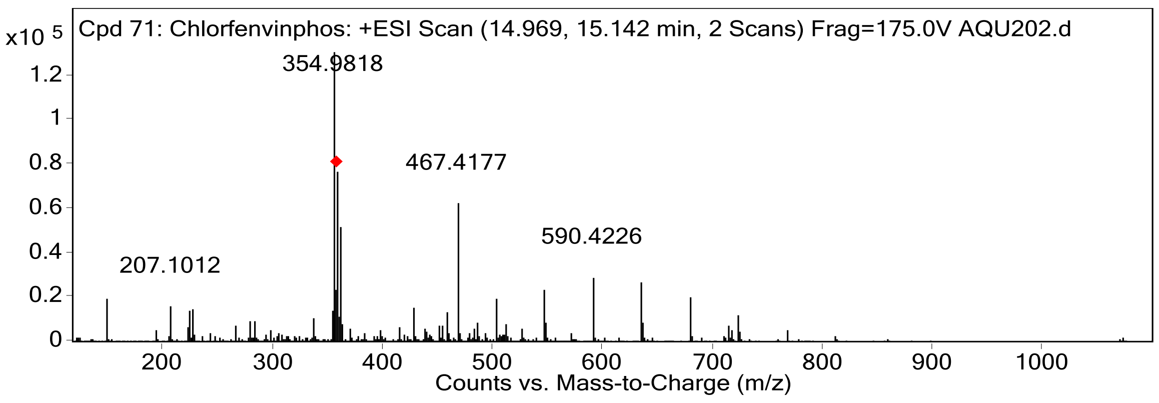

MS Zoomed Spectrum

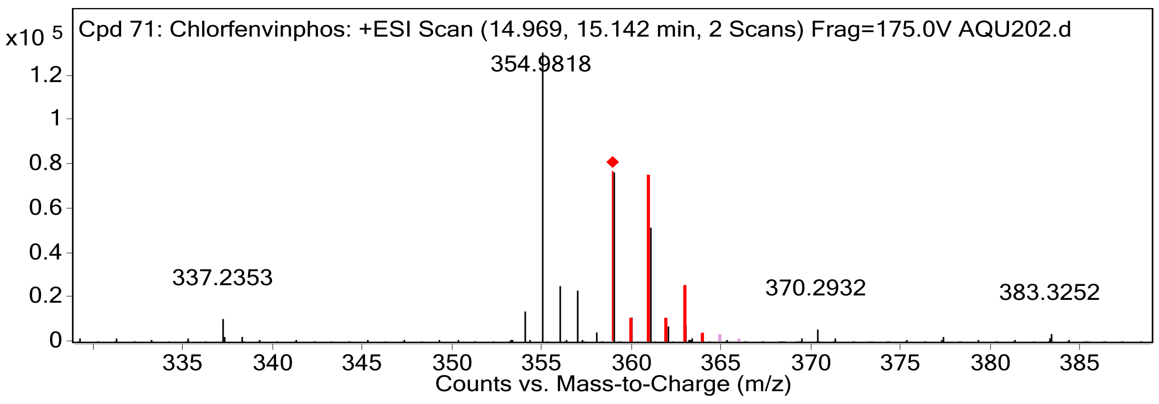

MS Spectrum Peak List

m/z	*Calc m/z*	Diff(ppm)	z	Abund	Formula	Ion
354.9818			1	130531.63		
358.978	358.9768	-3.33	1	76772	C12 H14 Cl3 O4 P	(M+H)+
359.9806	359.9802	-1	1	11628.18	C12 H14 Cl3 O4 P	(M+H)+
360.9756	360.974	-4.49	1	52046.52	C12 H14 Cl3 O4 P	(M+H)+
361.9777	361.9773	-1.08	1	7627.86	C12 H14 Cl3 O4 P	(M+H)+
362.9717	362.9713	-1.1	1	8376.19	C12 H14 Cl3 O4 P	(M+H)+
363.9747	363.9746	-0.42	1	983.12	C12 H14 Cl3 O4 P	(M+H)+
467.4177			1	62358.39		
590.4226			1	28938.92		
634.4487			1	26947.46		

MSMS Spectrum

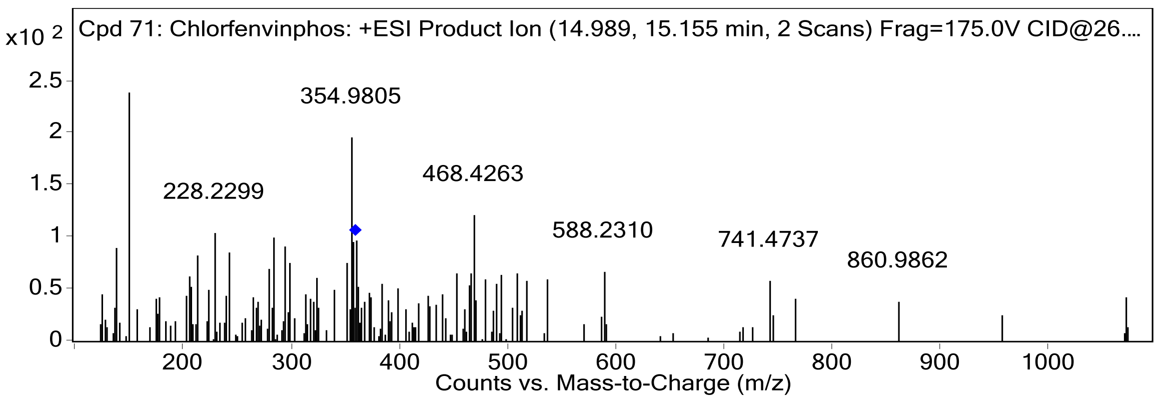

MS/MS Spectrum Peak List

m/z	z	Abund
137.0951		90.53
149.0228	1	240.01
228.2299		104.88
242.2838		85.58
283.2606		100.98
293.1724		91.5
354.9805	1	196.71
355.9822	1	96.37
358.975		97.33
468.4263		121.94

Compound Structure

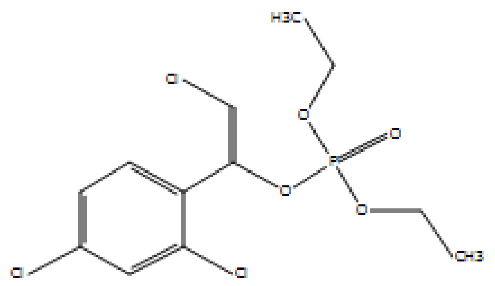

Compound Label	*m/z*	RT	Algorithm
Compound 72	355.9806	15.124	Auto MS/MS

MS Spectrum

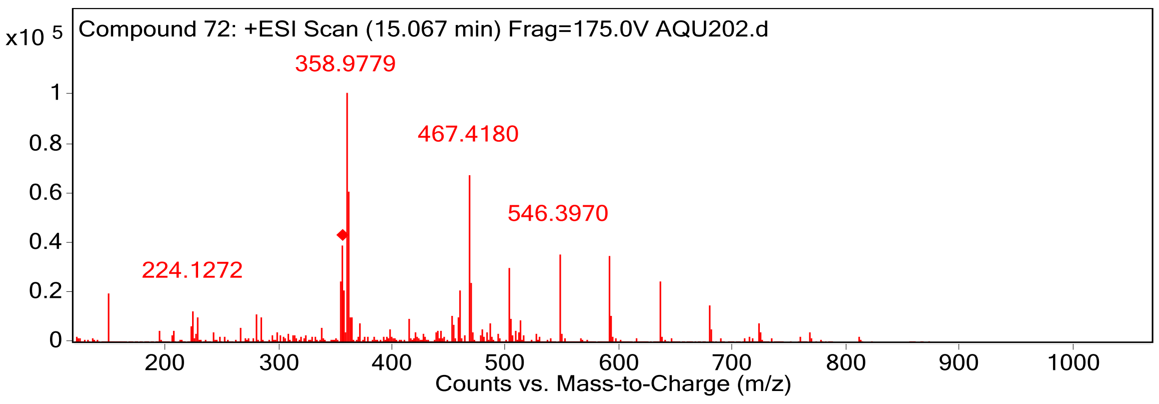

MS Zoomed Spectrum

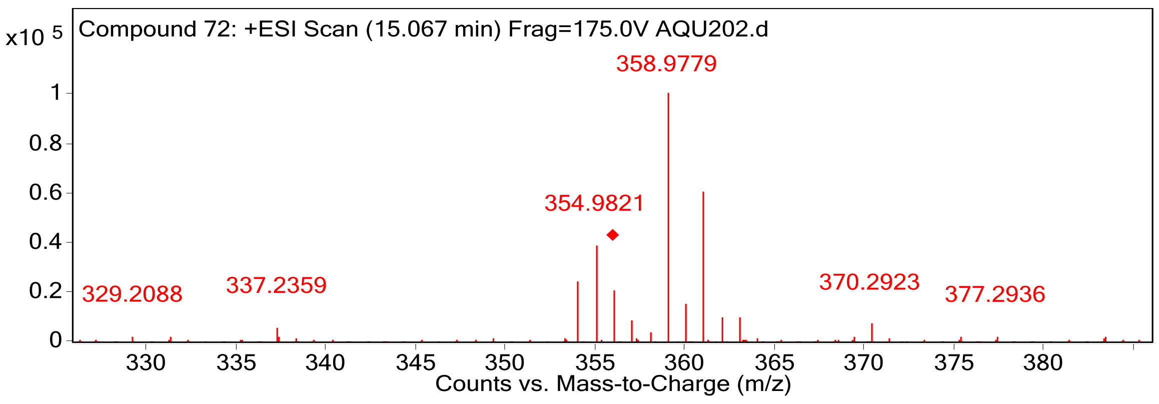

MS Spectrum Peak List

m/z	z	Abund
354.9821		39549.11
355.9806	1	21284.71
356.979	1	8853.41
357.9769	1	4198.87
358.9779	1	101030.48
360.9758	1	61063.84
467.418	1	67612.05
502.3707	1	30392.03
546.397	1	35428.11
590.4229	1	34784.95

MSMS Spectrum

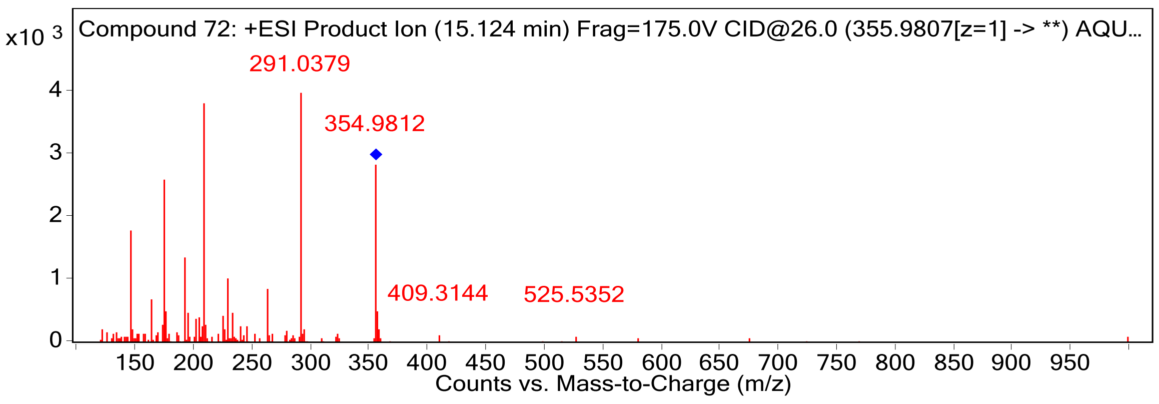

MS/MS Spectrum Peak List

m/z	z	Abund
145.9462	1	1792.31
163.9565		688.97
173.9775	1	2602.57
191.9877		1353.57
207.9652	1	3817.33
229.0191		1015.88
261.9978		795.6
263.0073	1	865.65
291.0379	1	3979.41
354.9812	1	2828.72

Compound Label	Name	*m/z*	RT	Algorithm	Mass
Cpd 73: Idoxuridine	**Idoxuridine**	354.9818	15.318	Auto MS/MS	353.9704

MS Spectrum

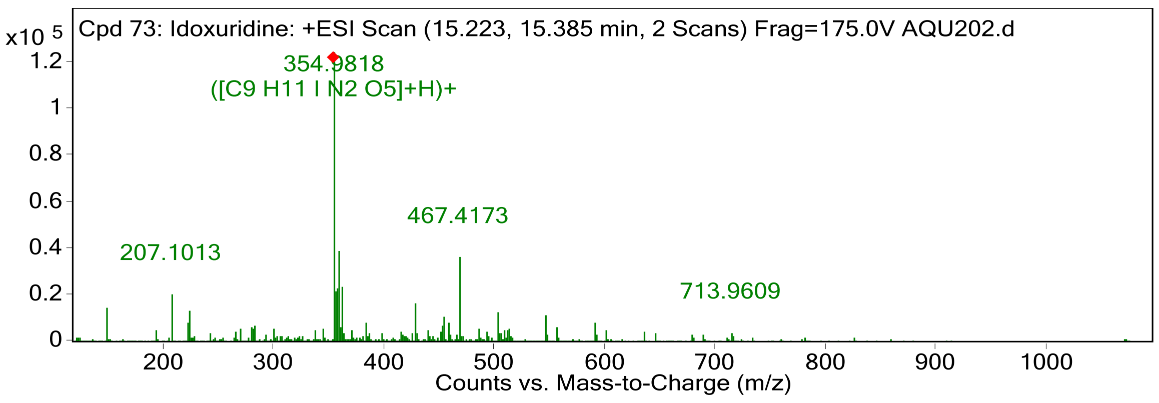

MS Zoomed Spectrum

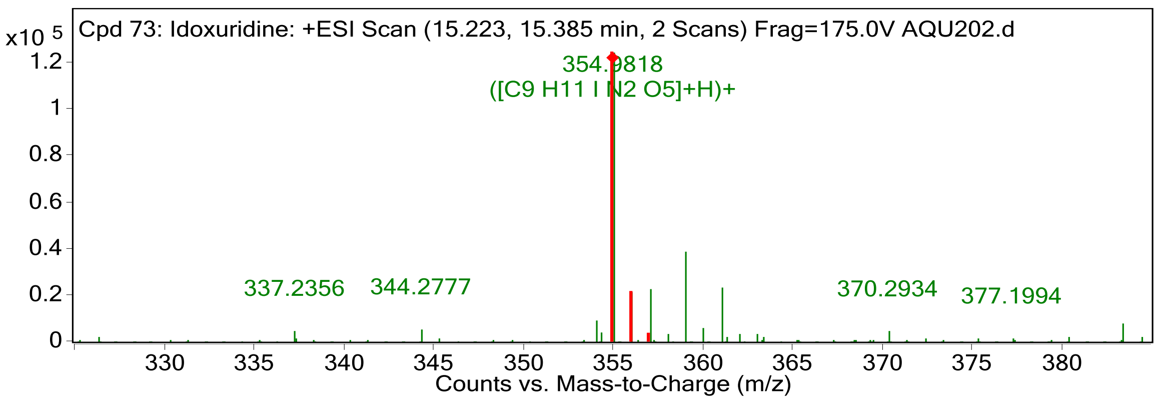

MS Spectrum Peak List

m/z	*Calc m/z*	Diff(ppm)	z	Abund	Formula	Ion
354.9818	354.9785	-9.11	1	124531.82	C9 H11 I N2 O5	(M+H)+
355.9833	355.9815	-4.92	1	22116.58	C9 H11 I N2 O5	(M+H)+
356.9777	356.9834	15.75	1	22947.55	C9 H11 I N2 O5	(M+H)+
357.9795	357.986	18.08	1	4152.34	C9 H11 I N2 O5	(M+H)+
358.9778	358.988	28.42	1	39359.32	C9 H11 I N2 O5	(M+H)+
359.9799	359.9904	29.17	1	6439.19	C9 H11 I N2 O5	(M+H)+
360.9756			1	23515.16		
361.9781			1	3894.5		
362.972			1	3967.16		
467.4173			1	36486.78		

MSMS Spectrum

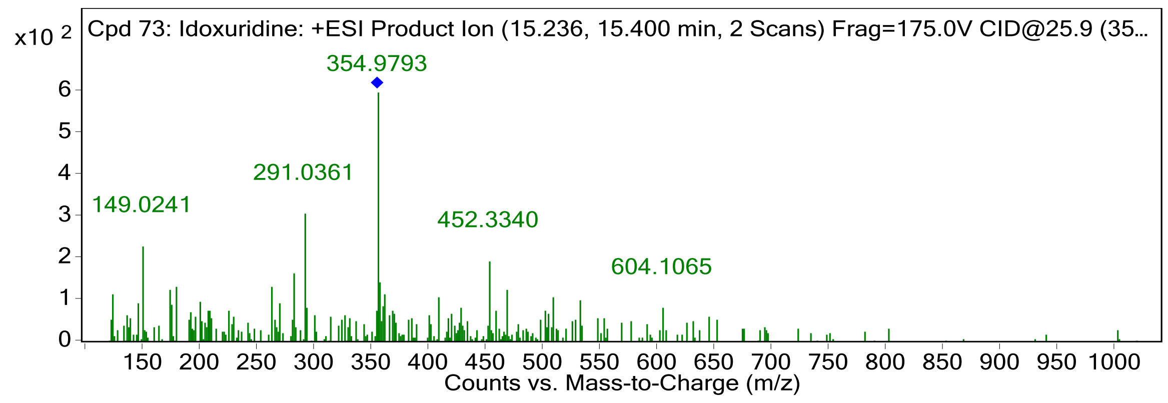

MS/MS Spectrum Peak List

m/z	z	Abund
149.0241		229.24
172.9799		126.42
179.069		131.55
261.9973		132.66
281.2429		164.46
291.0361		306.66
354.9793	1	597.43
355.9874	1	141.75
452.334		193.15
467.4196	1	124.87

Compound Structure

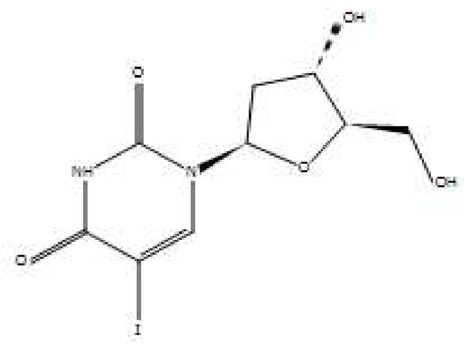

Compound Label	Name	*m/z*	RT	Algorithm	Mass
Cpd 74: Lumequoylacetone	**Lumequoylacetone**	513.4592	15.543	Auto MS/MS	490.4699

MS Spectrum

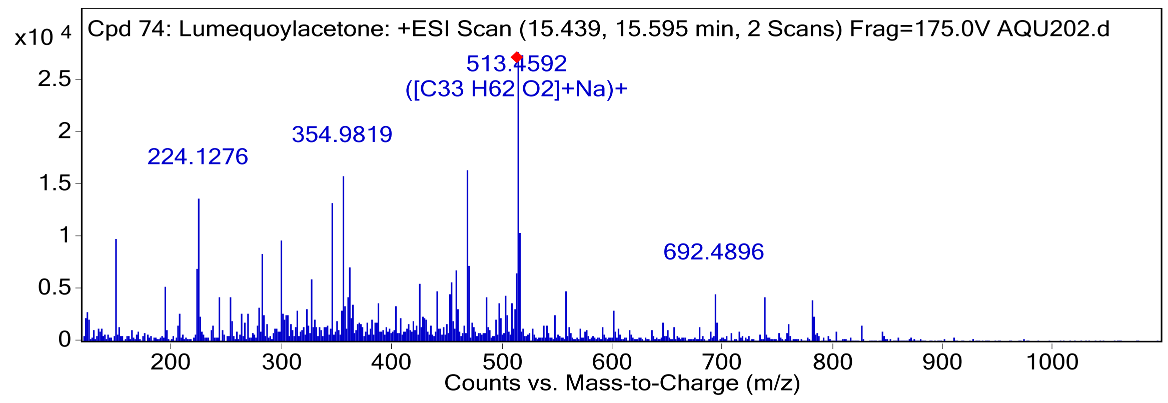

MS Zoomed Spectrum

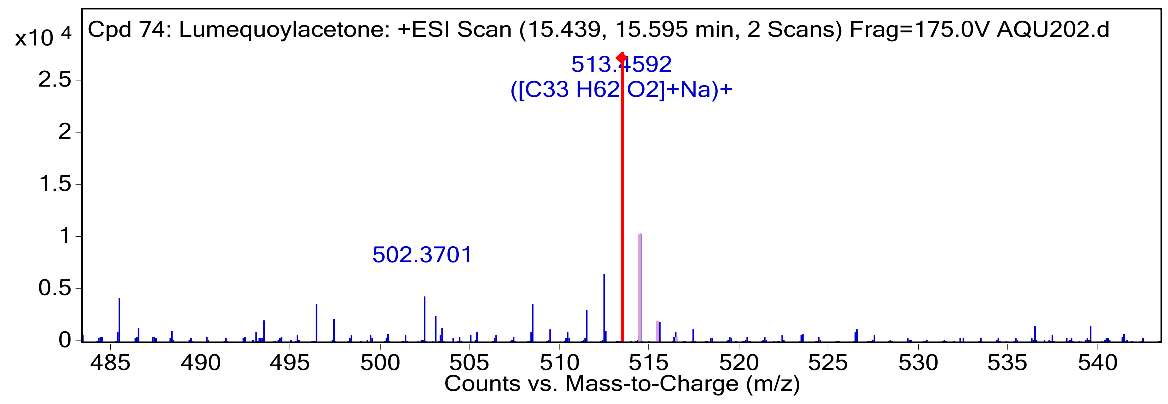

MS Spectrum Peak List

m/z	*Calc m/z*	Diff(ppm)	z	Abund	Formula	Ion
149.0232				9876.42		
224.1276			1	13769.49		
281.2465			1	8406.25		
299.2564			1	9730.58		
344.2776			1	13307.03		
354.9819			1	15894.14		
467.4176			1	16444.14		
468.3854			1	7355.91		
513.4592	513.4642	9.81	1	27717.64	C33 H62 O2	(M+Na)+
514.4625			1	10483.87		

MSMS Spectrum

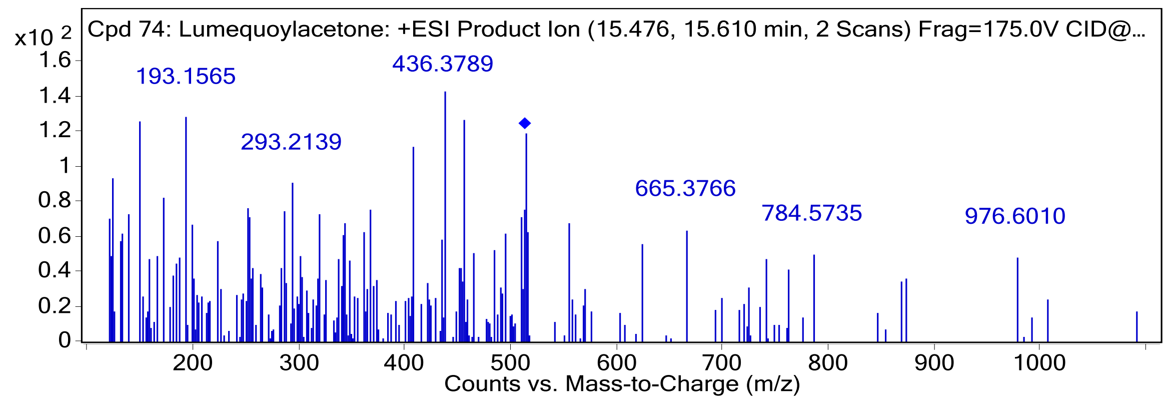

MS/MS Spectrum Peak List

m/z	z	Abund
124.0865		93.43

149.023		126.35
172.1691		82.35
193.1565		128.41
293.2139		90.82
407.2933		111.88
436.3789		142.81
454.3836		119.06
455.3908		126.62
513.4542	1	119.17

Compound Structure

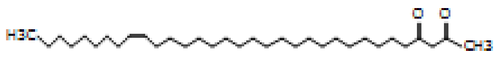

Compound Label	Name	*m/z*	RT	Algorithm	Mass
Cpd 75: Lumequoylacetone	**Lumequoylacetone**	513.4592	15.902	Auto MS/MS	490.4699

MS Spectrum

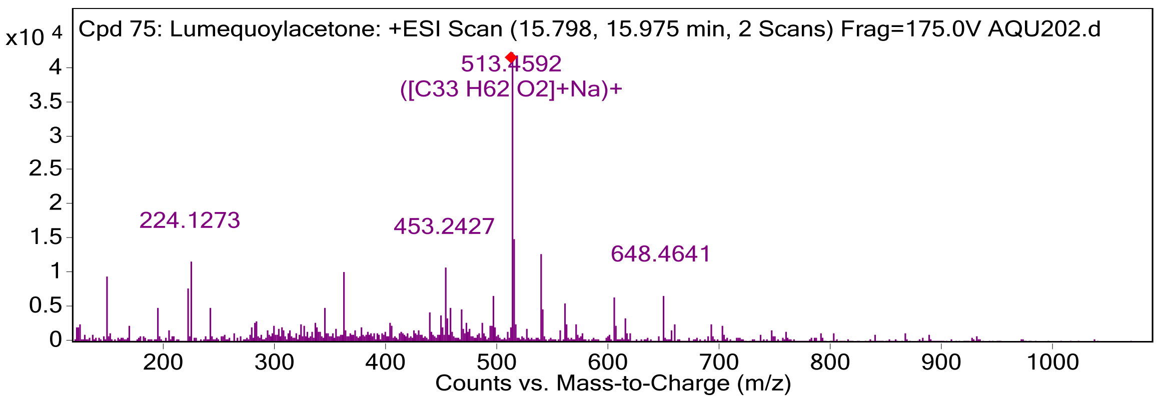

MS Zoomed Spectrum

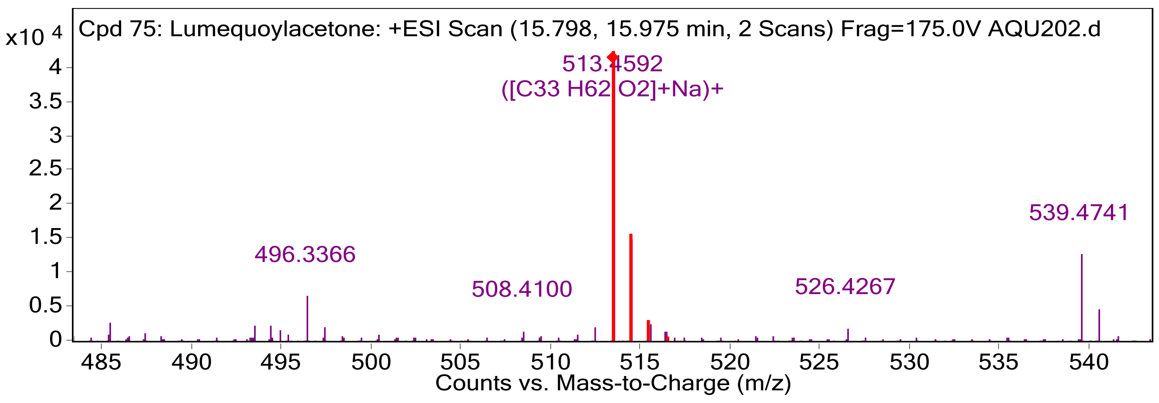

MS Spectrum Peak List

m/z	*Calc m/z*	Diff(ppm)	z	Abund	Formula	Ion
149.0231				9692.64		
222.111				7857.04		
224.1273			1	11720.09		
361.243			1	10363.52		
453.2427			1	10833.5		
513.4592	513.4642	9.82	1	42328.15	C33 H62 O2	(M+Na)+
514.4623	514.4676	10.3	1	14979.73	C33 H62 O2	(M+Na)+
515.4672	515.4709	7.23	1	2598.2	C33 H62 O2	(M+Na)+
516.4651	516.474	17.18	1	637.61	C33 H62 O2	(M+Na)+
539.4741			1	12840.03		

MSMS Spectrum

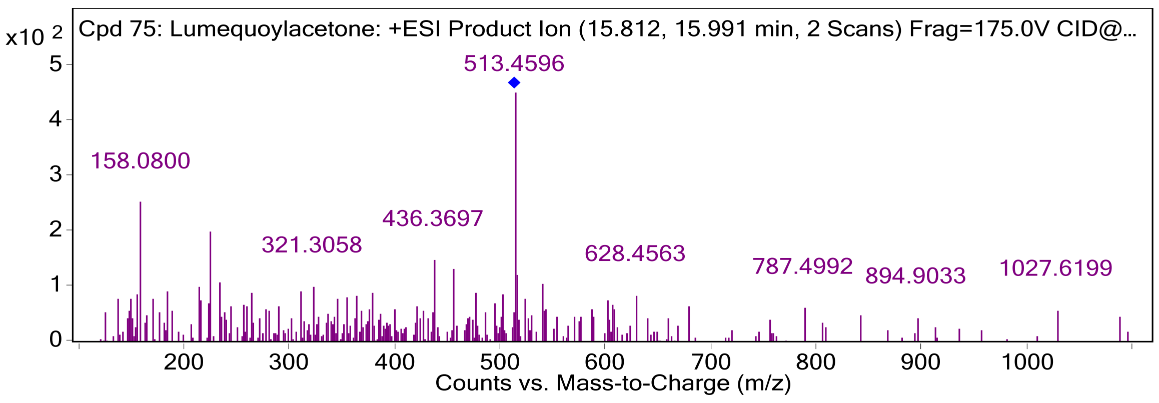

MS/MS Spectrum Peak List

m/z	z	Abund
158.08		252.73
213.1815		100.2
224.1282		199.63
233.1869		109.06
321.3058		100.36
436.3697		148.23
454.3856		132.06
513.4596	1	451.77
514.4585	1	121.46
539.47		104.38

Compound Structure

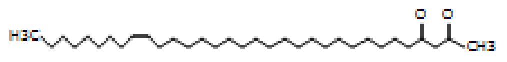

Compound Label	Name	*m/z*	RT	Algorithm	Mass
Cpd 76: TG(22:5(7Z,10Z,13Z,16Z,19Z)/18:3(9Z,12Z,15Z)/22:5(7Z,10Z,13Z,16Z,19Z))[iso3]	**TG(22:5(7Z,10Z,13Z,16Z,19Z)/18:3(9Z,12Z,15Z)/22:5(7Z,10Z,13Z,16Z,19Z))[iso3]**	511.3681	17.914	Auto MS/MS	976.758

MS Spectrum

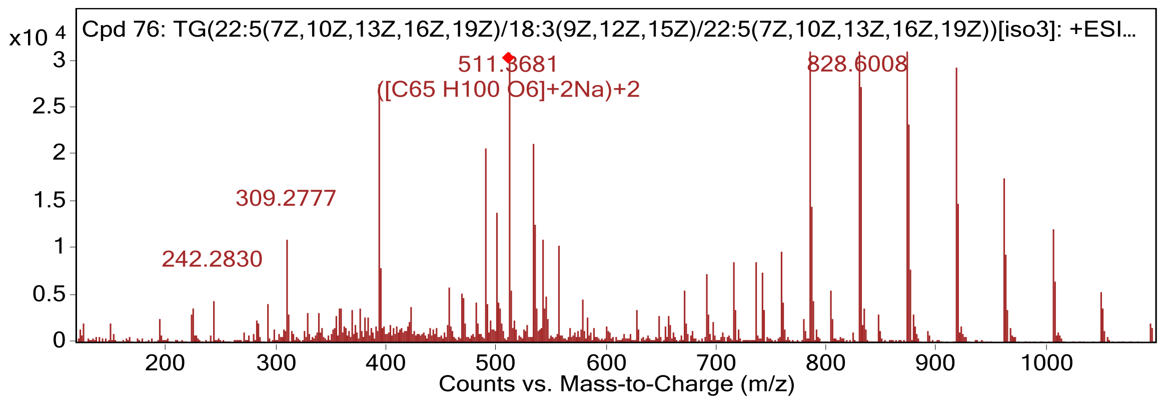

MS Zoomed Spectrum

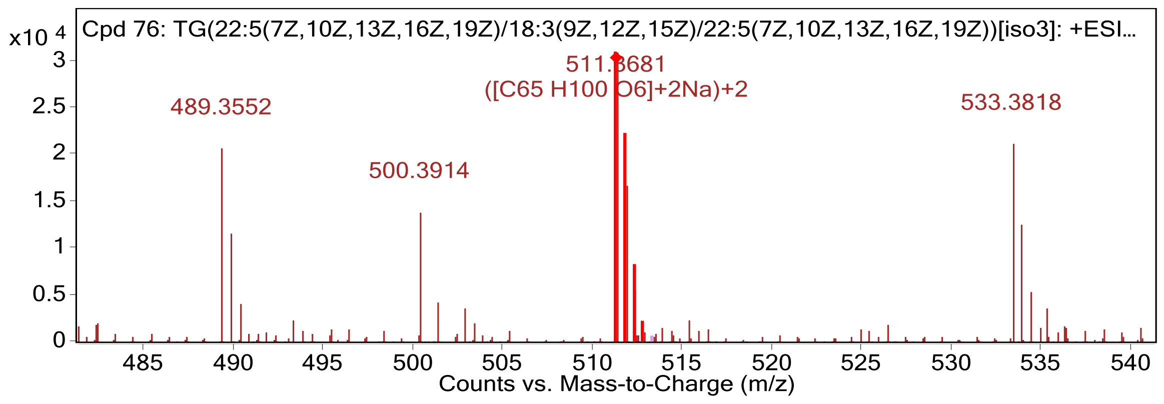

MS Spectrum Peak List

m/z	*Calc m/z*	Diff(ppm)	z	Abund	Formula	Ion
393.2975			1	27584.34		
511.3681	511.3652	-5.69	2	30887.33	C65 H100 O6	(M+2Na)+2
511.8699	511.8669	-5.77	2	16793	C65 H100 O6	(M+2Na)+2
512.3723	512.3686	-7.22	2	5637.51	C65 H100 O6	(M+2Na)+2
512.8731	512.8702	-5.72	2	1091.11	C65 H100 O6	(M+2Na)+2
784.575			1	32278.81		
828.6008			1	62706.89		
829.6043			1	27280.23		
872.6268			1	49299.82		
916.6523			1	29226.8		

MSMS Spectrum

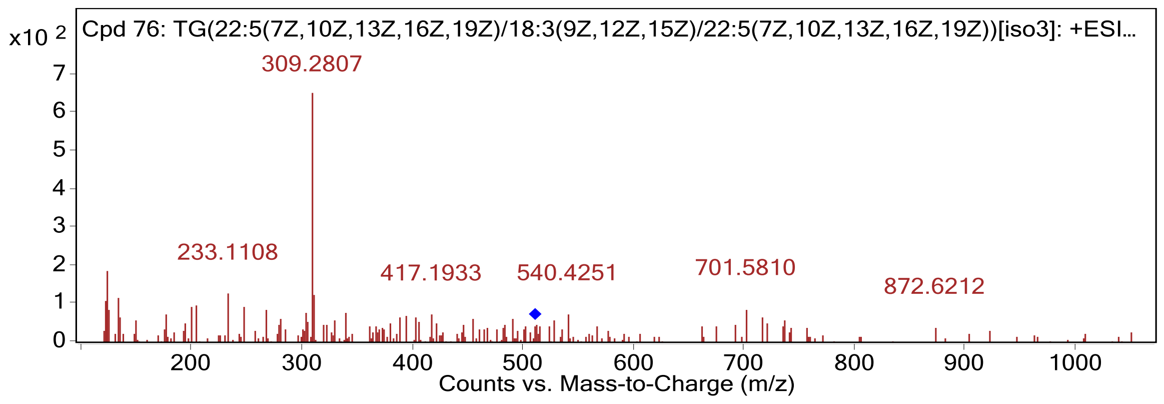

MS/MS Spectrum Peak List

m/z	z	Abund
122.0962		110.13
123.0794		189.02
133.0853		117.52
199.0431		92.04
203.1366		98.22
233.1108		127.69
247.2441		93.01
309.2807	1	653.64
310.2783	1	126.09
701.581		87.38

Compound Structure

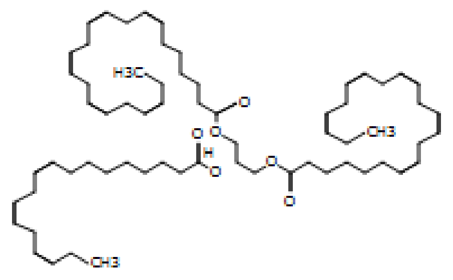

Compound Label	m/z	RT	Algorithm
Compound 77	916.6526	17.987	Auto MS/MS

MS Spectrum

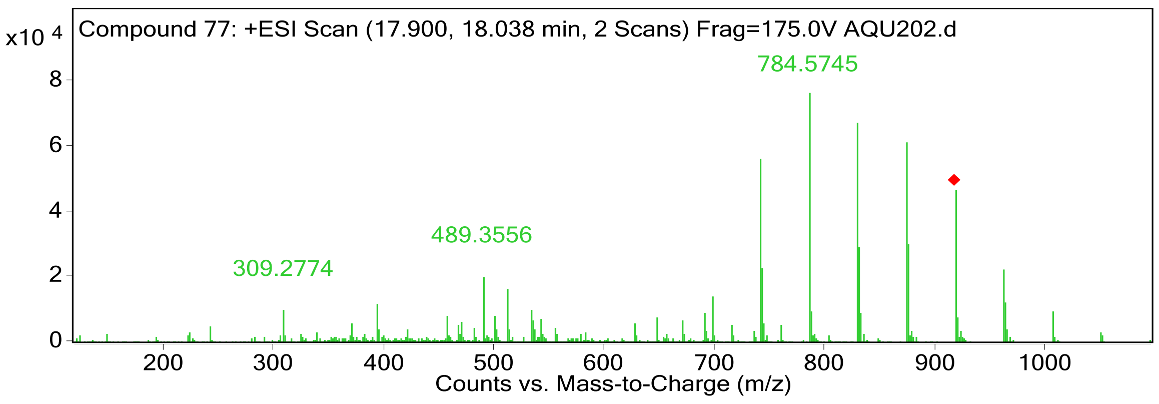

MS Zoomed Spectrum

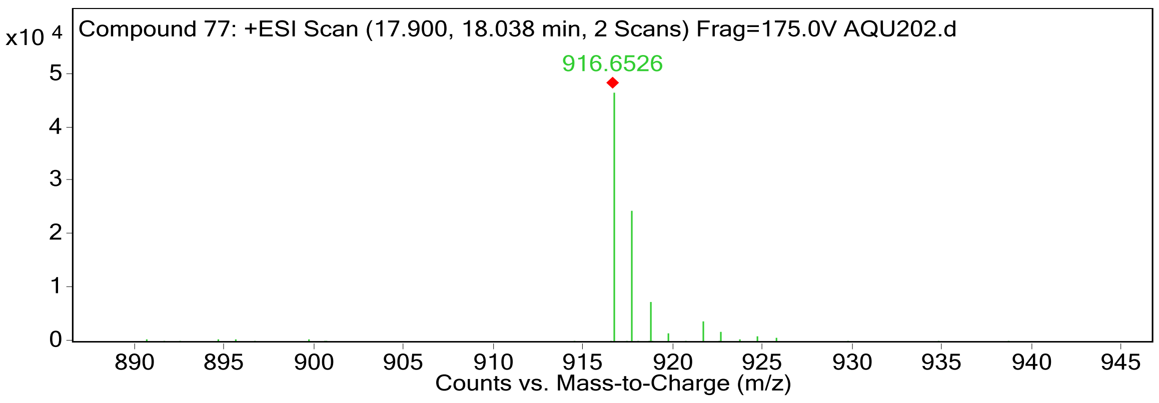

MS Spectrum Peak List

m/z	z	Abund
740.548	1	56320.51
784.5745	1	76611.36
785.5777	1	33627.5
828.5999	1	67143
872.6265	1	61500.53
873.6297	1	30114.66
916.6526	1	46638.57
917.6567	1	24508.33
918.6576	1	7567.75
919.6614	1	1713.08

MSMS Spectrum

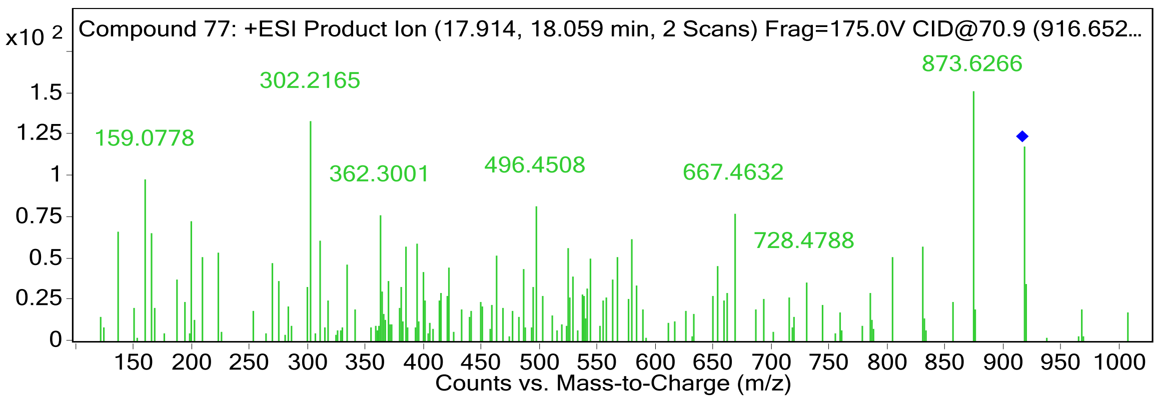

MS/MS Spectrum Peak List

m/z	z	Abund
136.0581		66.57
159.0778		98.33
164.1492		66.26
199.1666		73.19
302.2165		133.31
362.3001	1	76.7
496.4508		82.08
667.4632		77.71
873.6266		151.2
916.6539	1	118.06

Compound Label	m/z	RT	Algorithm
Compound 78	828.5999	17.987	Auto MS/MS

MS Spectrum

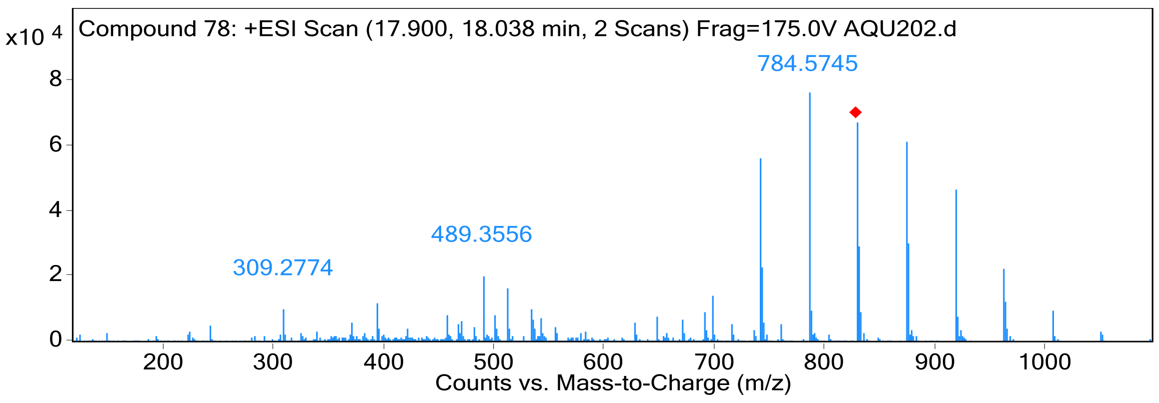

MS Zoomed Spectrum

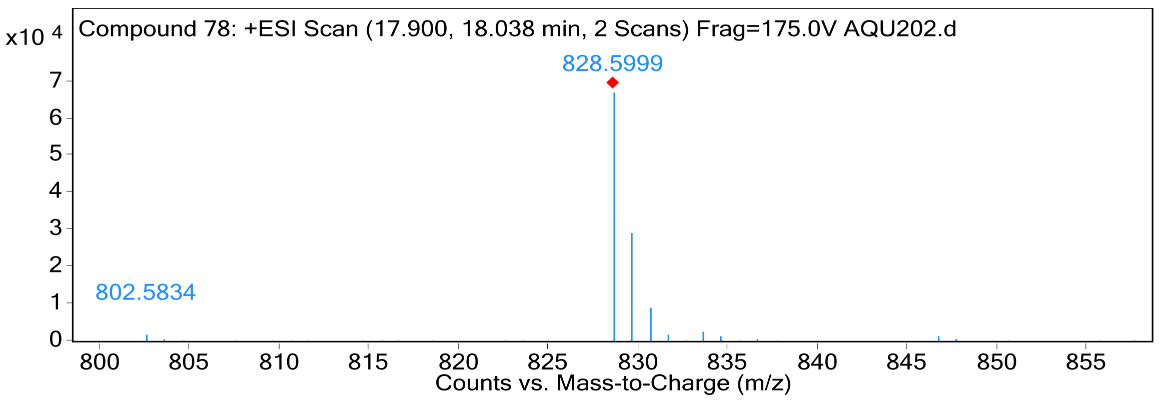

MS Spectrum Peak List

m/z	z	Abund
740.548	1	56320.51
784.5745	1	76611.36
785.5777	1	33627.5
828.5999	1	67143
829.6035	1	29491.92
830.606	1	9156.16
831.6097	1	1981.6
872.6265	1	61500.53
873.6297	1	30114.66
916.6526	1	46638.57

MSMS Spectrum

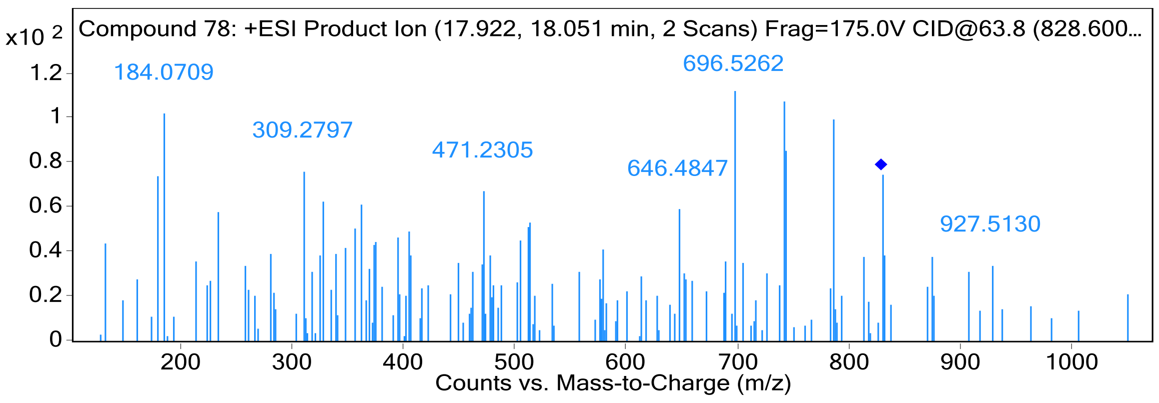

MS/MS Spectrum Peak List

m/z	z	Abund
178.9189		74.28
184.0709		102.39
309.2797		76.41
327.2753		62.61
471.2305		67.43
696.5262		112.12
740.5536	1	107.6
741.5593	1	85.5
784.5741	1	99.76
829.6073	1	75.13

Compound Label	*m/z*	RT	Algorithm
Compound 79	696.5218	18.135	Auto MS/MS

MS Spectrum

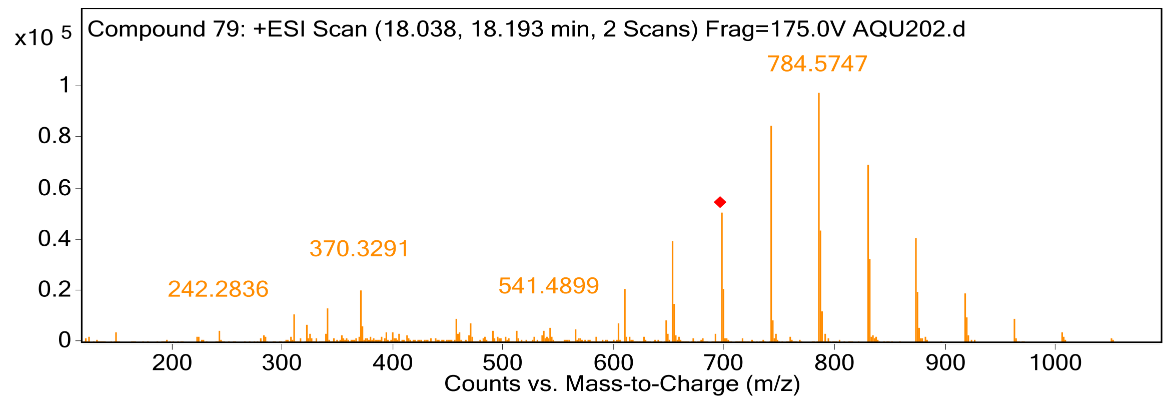

MS Zoomed Spectrum

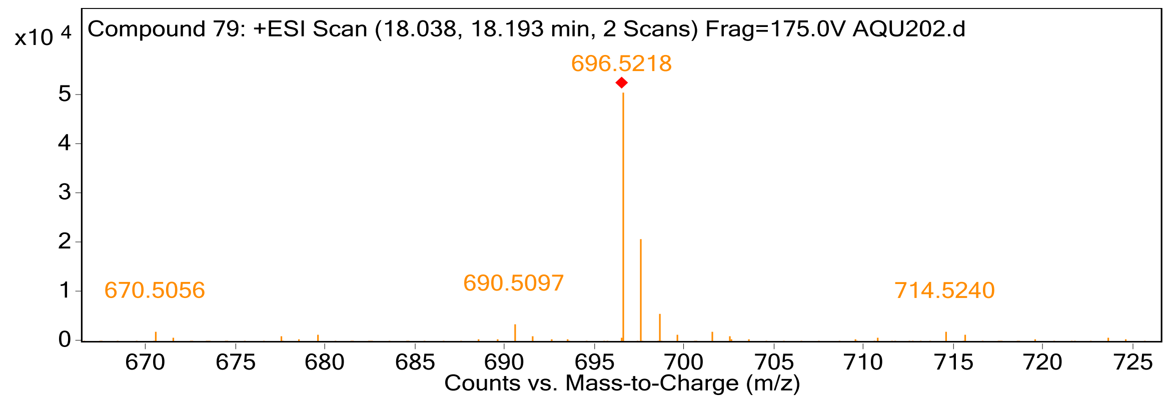

MS Spectrum Peak List

m/z	z	Abund
652.4955	1	39626.32
696.5218	1	50699.64
697.5256	1	20987.31
698.5275	1	5706.09
699.5309	1	1478.24
740.548	1	85219.06
784.5747	1	97963.92
785.5774	1	43759.13
828.5999	1	69893.04
872.6264	1	41064.32

MSMS Spectrum

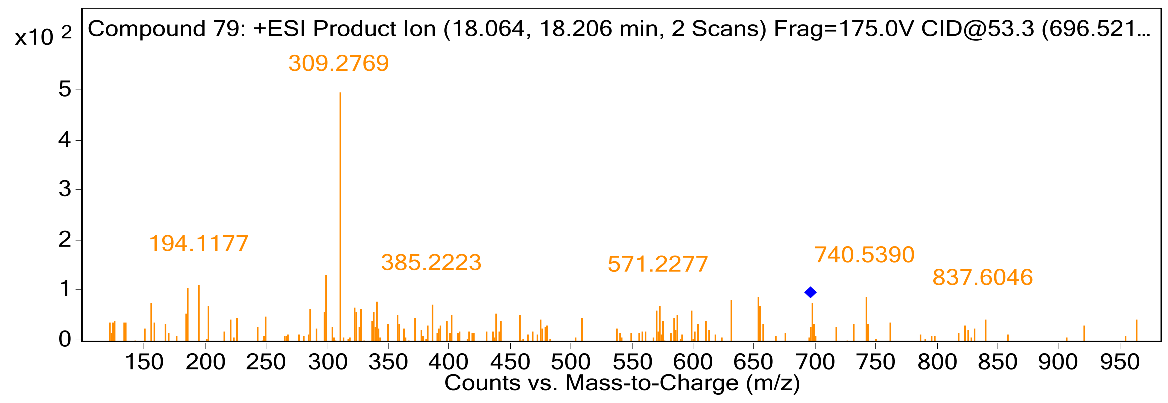

MS/MS Spectrum Peak List

m/z	z	Abund
155.0853		76.64
184.0695		106.97
194.1177		113.47
298.3061		133.95
309.2769		499.2
339.3194	1	80.95
629.428		83.18
652.4868		90.09
696.5203	1	77.5
740.539	1	90.72

Compound Label	*m/z*	RT	Algorithm
Compound 80	652.4957	18.19	Auto MS/MS

MS Spectrum

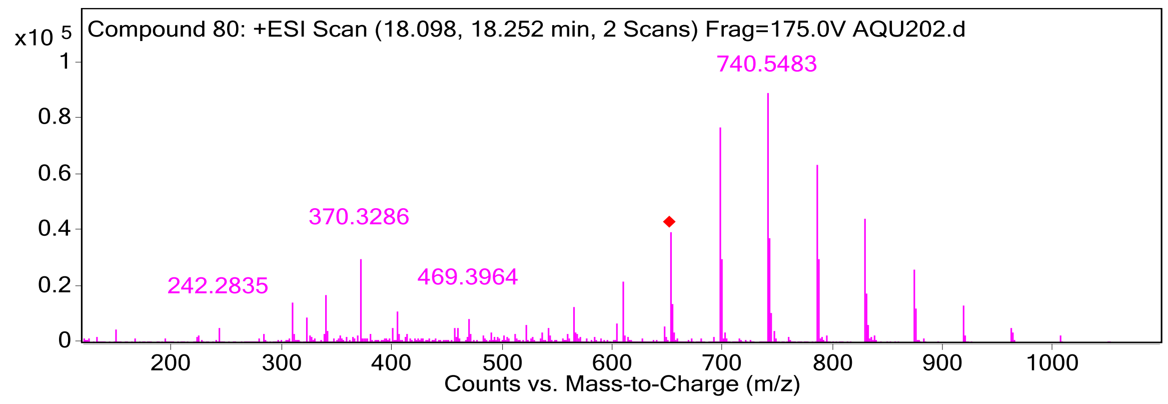

MS Zoomed Spectrum

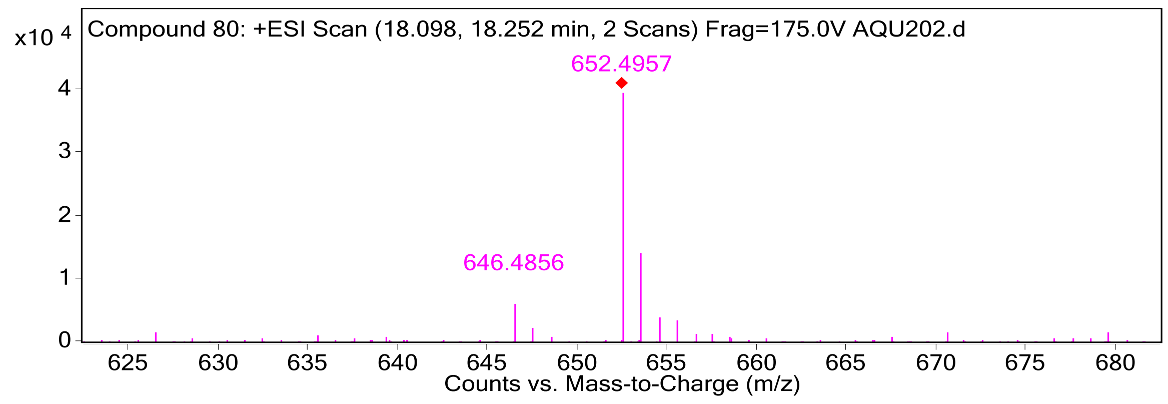

MS Spectrum Peak List

m/z	z	Abund

370.3286	1	30199.48
652.4957	1	39644.25
653.4989	1	14123.99
654.5023	1	3939.09
696.5218	1	77075.13
697.5253	1	30117.74
740.5483	1	89430.89
741.5509	1	37466.77
784.5745	1	63642.69
828.6003	1	44168.63

MSMS Spectrum

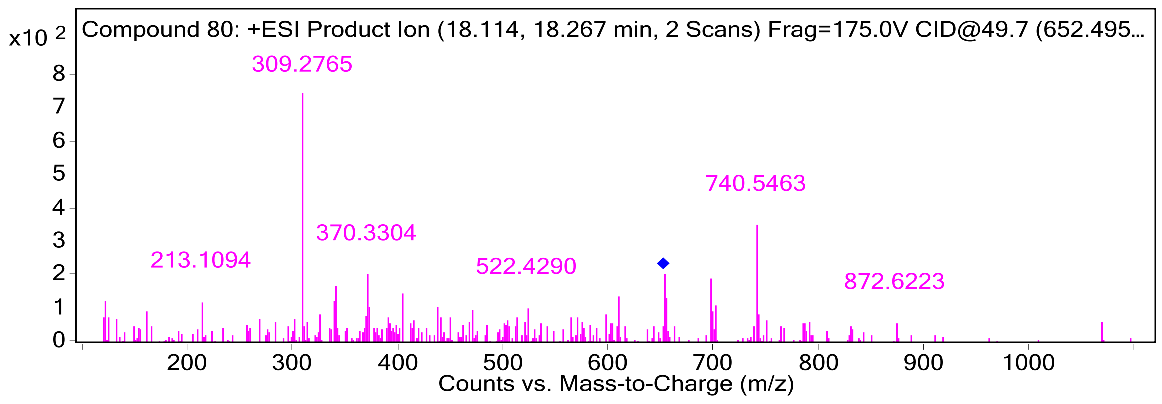

MS/MS Spectrum Peak List

m/z	z	Abund
122.0967		125.99
309.2765		747.71
340.3244		172.07
370.3304	1	204.24
404.3144		145.86
608.4768	1	137.2
652.494	1	207.05
653.4991	1	133.29
696.5229	1	191.13
740.5463	1	352.83

--- End Of Report ---

Qualitative Compound Report

**Data File**	AQU202_-VEr.d	**Sample Name**	AQU202
**Sample Type**	Sample	**Position**	P1-B6
**Instrument Name**	QTOF	**User Name**	
**Acq Method**	30min_-ve_ESI_plant extract_MSMS.m	**Acquired Time**	4/19/2022 2:53:08 PM
**IRM Calibration Status**	Success	**DA Method**	default.m
**Comment**			

Sample Group **Info.**

Acquisition SW Version 6200 series TOF/6500 series Q-TOF B.05.01 (B5125.3)

Compound Table

Compound Label	RT	Mass	Abund	Name	Formula	Tgt Mass	Diff (ppm)	MFG Formula	DB Formula	DB Diff (ppm)	Hits (DB)
Cpd 2: (S)-Menthone 8-thioacetate	3.058	228.1178	15564	(S)-Menthone 8-thioacetate	C12 H20 O2 S			C12 H20 O2 S	C12 H20 O2 S	2.81	10
Cpd 3: (E,E)-1,6-bis(4-methoxyphenyl)-1,5-hexadiene	3.884	294.1643	16506	(E,E)-1,6-bis(4-methoxyphenyl)-1,5-hexadiene	C20 H22 O2			C20 H22 O2	C20 H22 O2	-7.97	3
Cpd 4: 1,3,5,11-Bisabolatetraen-10-one	3.993	216.1528	30228	1,3,5,11-Bisabolatetraen-10-one	C15 H20 O			C15 H20 O	C15 H20 O	-6.45	8
Cpd 5: 4,4'-(2-Methylpropylidene)bisphenol	4.037	242.1323	18195	4,4'-(2-Methylpropylidene)bisphenol	C16 H18 O2			C16 H18 O2	C16 H18 O2	-6.84	8
Cpd 6: Ethylketocyclazocine	4.468	299.1919	12391	Ethylketocyclazocine	C19 H25 N O2			C19 H25 N O2	C19 H25 N O2	-11.39	1
Cpd 7: (E,E)-1,6-bis(4-methoxyphenyl)-1,5-hexadiene	4.599	294.1646	17779	(E,E)-1,6-bis(4-methoxyphenyl)-1,5-hexadiene	C20 H22 O2			C20 H22 O2	C20 H22 O2	-8.77	3
Cpd 8: 4,4'-(2-Methylpropylidene)bisphenol	4.782	242.1323	52397	4,4'-(2-Methylpropylidene)bisphenol	C16 H18 O2			C16 H18 O2	C16 H18 O2	-6.52	8
Cpd 9: Ethylketocyclazocine	4.842	299.1914	14625	Ethylketocyclazocine	C19 H25 N O2			C19 H25 N O2	C19 H25 N O2	-9.57	1
Cpd 10: S-Methyl-1-thio-D-glycerate	5.018	136.0212		S-Methyl-1-thio-D-glycerate	C4 H8 O3 S			C4 H8 O3 S	C4 H8 O3 S	-12.81	8
Cpd 11: 4,4'-(2-Methylpropylidene)bisphenol	5.139	242.1325		4,4'-(2-Methylpropylidene)bisphenol	C16 H18 O2			C16 H18 O2	C16 H18 O2	-7.59	8
Cpd 12: 1,7-Diphenyl-4-hepten-3-one	5.377	264.1537	27743	1,7-Diphenyl-4-hepten-3-one	C19 H20 O			C19 H20 O	C19 H20 O	-8.6	8
Compound 13	5.875		16357								
Cpd 14: Melanostatin	5.97	284.1828	12870	Melanostatin	C13 H24 N4 O3			C13 H24 N4 O3	C13 H24 N4 O3	7.13	10
Compound 1	5.986	212.0691	841		C10 H12 O5	212.0685	2.79	C10 H12 O5	C10 H12 O5		
Cpd 15: Sethoxydim	6.055	327.186	15681	Sethoxydim	C17 H29 N O3 S			C17 H29 N O3 S	C17 H29 N O3 S	2.37	10
Cpd 16: Melanostatin	6.222	284.182	12177	Melanostatin	C13 H24 N4 O3			C13 H24 N4 O3	C13 H24 N4 O3	10.03	10
Cpd 17: Isopentenyl adenosine	6.26	335.1615	11333	Isopentenyl adenosine	C15 H21 N5 O4			C15 H21 N5 O4	C15 H21 N5 O4	-6.3	6
Cpd 18: 2-Deoxystreptidine	6.406	246.144	14218	2-Deoxystreptidine	C8 H18 N6 O3			C8 H18 N6 O3	C8 H18 N6 O3	0.06	4
Cpd 19: Spirasine I	6.494	355.2193	19064	Spirasine I	C22 H29 N O3			C22 H29 N O3	C22 H29 N O3	-12.85	5
Cpd 20: Spirasine I	6.541	355.219	21349	Spirasine I	C22 H29 N O3			C22 H29 N O3	C22 H29 N O3	-12.12	4
Compound 21	6.547		19940								
Cpd 22: Melanostatin	6.785	284.182	55947	Melanostatin	C13 H24 N4 O3			C13 H24 N4 O3	C13 H24 N4 O3	10.12	10
Cpd 23: β-estradiol	6.948	272.1802	45214	β-estradiol	C18 H24 O2			C18 H24 O2	C18 H24 O2	-9.57	9
Cpd 24: Eseramine	7.219	318.1675	21387	Eseramine	C16 H22 N4 O3			C16 H22 N4 O3	C16 H22 N4 O3	5.38	5
Cpd 25: beta-D-Mannosylphosphodecaprenol	7.318	940.6625	26137	beta-D-Mannosylphosphodecaprenol	C56 H93 O9 P			C56 H93 O9 P	C56 H93 O9 P	-7.17	1
Cpd 26: 2-Deoxystreptidine	7.361	246.1441	19297	2-Deoxystreptidine	C8 H18 N6 O3			C8 H18 N6 O3	C8 H18 N6 O3	-0.17	4
Cpd 27: Oxybutynin	7.383	357.2312	25137	Oxybutynin	C22 H31 N O3			C22 H31 N O3	C22 H31 N O3	-2.28	4
Cpd 28: Spirasine I	7.59	355.2182	31522	Spirasine I	C22 H29 N O3			C22 H29 N O3	C22 H29 N O3	-9.59	4
Cpd 29: Spirasine I	7.832	355.2191	54923	Spirasine I	C22 H29 N O3			C22 H29 N O3	C22 H29 N O3	-12.23	4
Cpd 30: β-estradiol	7.834	272.1803		β-estradiol	C18 H24 O2			C18 H24 O2	C18 H24 O2	-9.99	9
Cpd 31: 3alpha,7beta,12alpha-Trihydroxy-5alpha-cholan-24-oic Acid	7.864	408.2832	17415	3alpha,7beta,12alpha-Trihydroxy-5alpha-cholan-24-oic Acid	C24 H40 O5			C24 H40 O5	C24 H40 O5	10.82	10
Cpd 32: β-estradiol	8.122	272.1801	26116	β-estradiol	C18 H24 O2			C18 H24 O2	C18 H24 O2	-9.25	9
Cpd 33: 3beta,7beta,12alpha-Trihydroxy-5alpha-cholan-24-oic Acid	8.171	408.283	11487	3beta,7beta,12alpha-Trihydroxy-5alpha-cholan-24-oic Acid	C24 H40 O5			C24 H40 O5	C24 H40 O5	11.19	10
Cpd 34: (1R,2S,4R,5S)-2,5-Fenchanediol 2-O-b-D-glucoside	8.302	332.1826	20000	(1R,2S,4R,5S)-2,5-Fenchanediol 2-O-b-D-glucoside	C16 H28 O7			C16 H28 O7	C16 H28 O7	2.72	10
Cpd 35: Piperoic acid	8.343	358.2191	15382	Piperoic acid	C22 H30 O4			C22 H30 O4	C22 H30 O4	-13.06	10
Cpd 36: (E,E,E)-Sylvatine	8.542	383.2526	13376	(E,E,E)-Sylvatine	C24 H33 N O3			C24 H33 N O3	C24 H33 N O3	-17.09	1
Cpd 37: Verapamil	8.543	454.2877	11377	Verapamil	C27 H38 N2 O4			C27 H38 N2 O4	C27 H38 N2 O4	-9.99	10
Cpd 38: 3alpha,7beta,12alpha-Trihydroxy-5alpha-cholan-24-oic Acid	8.568	408.2841	29832	3alpha,7beta,12alpha-Trihydroxy-5alpha-cholan-24-oic Acid	C24 H40 O5			C24 H40 O5	C24 H40 O5	8.5	10
Compound 39	8.668		20611								
Cpd 40: 3alpha,7beta,12alpha-Trihydroxy-5alpha-cholan-24-oic Acid	8.88	408.2841	34167	3alpha,7beta,12alpha-Trihydroxy-5alpha-cholan-24-oic Acid	C24 H40 O5			C24 H40 O5	C24 H40 O5	8.47	10
Cpd 41: Fesoterodine	9.084	411.2829	10326	Fesoterodine	C26 H37 N O3			C26 H37 N O3	C26 H37 N O3	-13.61	1
Cpd 42: 3alpha,7beta,12alpha-Trihydroxy-5alpha-cholan-24-oic Acid	9.207	408.2846	78025	3alpha,7beta,12alpha-Trihydroxy-5alpha-cholan-24-oic Acid	C24 H40 O5			C24 H40 O5	C24 H40 O5	7.32	10
Cpd 43: Ovatine	9.441	385.2639	26768	Ovatine	C24 H35 N O3			C24 H35 N O3	C24 H35 N O3	-5.78	3
Compound 44	9.68										
Compound 45	9.796		17249								
Cpd 46: Ethamoxytriphetol	9.848	419.2462	13473	Ethamoxytriphetol	C27 H33 N O3			C27 H33 N O3	C27 H33 N O3	-0.31	6
Compound 47	10.07		18570								
Cpd 48: Cholestane-3,7,12,24,25-pentol	10.125	450.3456	10025	Cholestane-3,7,12,24,25-pentol	C27 H48 O5			C27 H48 O5	C27 H48 O5	4451.27	10
Cpd 49: 1,1'-[1,12-Dodecanediylbis(oxy)]bisbenz	10.266	354.2493	82473	1,1'-[1,12-Dodecanediylbis(oxy)]bisbenz	C24 H34 O2			C24 H34 O2	C24 H34 O2	18.57	2
Cpd 50: Androsterone sulfate	10.426	370.1854	13860	Androsterone sulfate	C19 H30 O5 S			C19 H30 O5 S	C19 H30 O5 S	-10.74	10
Cpd 51: 5beta-Cholestane-3alpha,7alpha,12alpha,25,26-pentol	10.549	452.3469	11508	5beta-Cholestane-3alpha,7alpha,12alpha,25,26-pentol	C27 H48 O5			C27 H48 O5	C27 H48 O5	7.26	10
Compound 52	10.625										
Cpd 53: Propoxycaine	10.866	294.1911		Propoxycaine	C16 H26 N2 O3			C16 H26 N2 O3	C16 H26 N2 O3	10.99	10
Compound 54	11.152		12802								
Cpd 55: Aspidospermine	11.791	354.2312	17262	Aspidospermine	C22 H30 N2 O2			C22 H30 N2 O2	C22 H30 N2 O2	-1.26	5

Compound 56	12.017		56614								
Compound 57	12.171										
Cpd 58: Phlegmarine	12.27	250.237		Phlegmarine	C16 H30 N2			C16 H30 N2	C16 H30 N2	15.68	1
Cpd 59: alpha-Tocopherolquinone	12.81	446.3726	49538	alpha-Tocopherolquinone	C29 H50 O3			C29 H50 O3	C29 H50 O3	7.57	8
Cpd 60: Pithecolobine	12.848	382.3659	24932	Pithecolobine	C22 H46 N4 O			C22 H46 N4 O	C22 H46 N4 O	3.39	10
Cpd 61: alpha-Tocopherolquinone	13.104	446.3729	23321	alpha-Tocopherolquinone	C29 H50 O3			C29 H50 O3	C29 H50 O3	6.88	8
Compound 62	13.234		50398								
Compound 63	13.303										
Compound 64	13.619		23423								
Cpd 65: Bryodulcosigenin	13.708	474.3692	17283	Bryodulcosigenin	C30 H50 O4			C30 H50 O4	C30 H50 O4	3.7	10
Cpd 66: Usambarine	13.783	450.2858	19733	Usambarine	C30 H34 N4			C30 H34 N4	C30 H34 N4	-16.58	3
Compound 67	14.012		15060								
Cpd 68: (3beta,24xi)-Cycloartane-3,24,25-triol	14.212	460.3883	13050	(3beta,24xi)-Cycloartane-3,24,25-triol	C30 H52 O3			C30 H52 O3	C30 H52 O3	7.34	5
Cpd 69: Undecylprodigiosin	14.243	393.275	11873	Undecylprodigiosin	C25 H35 N3 O			C25 H35 N3 O	C25 H35 N3 O	7.68	2
Cpd 70: Undecylprodigiosin	14.634	393.2748	34006	Undecylprodigiosin	C25 H35 N3 O			C25 H35 N3 O	C25 H35 N3 O	8.05	2
Cpd 71: 5'-Methoxybilobetin	14.816	582.1257	27565	5'-Methoxybilobetin	C32 H22 O11			C32 H22 O11	C32 H22 O11	-16.23	3
Compound 72	14.865		16343								
Compound 73	15.045		38160								
Cpd 74: Milbemectin	15.133	528.3168	21832	Milbemectin	C31 H44 O7			C31 H44 O7	C31 H44 O7	-15.37	1
Cpd 75: Maclurin 3-C-(2''-p-hydroxybenzoyl-6''-galloyl-glucoside)	15.318	696.137	33725	Maclurin 3-C-(2''-p-hydroxybenzoyl-6''-galloyl-glucoside)	C33 H28 O17			C33 H28 O17	C33 H28 O17	-6.26	2
Cpd 76: UDP-2,4-bis(acetamido)-2,4,6-trideoxy-beta-L-altropyranose	15.402	632.1237		UDP-2,4-bis(acetamido)-2,4,6-trideoxy-beta-L-altropyranose	C19 H30 N4 O16 P2			C19 H30 N4 O16 P2	C19 H30 N4 O16 P2	-16.67	1
Cpd 77: UDP-2,4-bis(acetamido)-2,4,6-trideoxy-beta-L-altropyranose	15.656	632.1241	34982	UDP-2,4-bis(acetamido)-2,4,6-trideoxy-beta-L-altropyranose	C19 H30 N4 O16 P2			C19 H30 N4 O16 P2	C19 H30 N4 O16 P2	-17.25	1
Cpd 78: Maclurin 3-C-(2''-p-hydroxybenzoyl-6''-galloyl-glucoside)	15.675	696.1374		Maclurin 3-C-(2''-p-hydroxybenzoyl-6''-galloyl-glucoside)	C33 H28 O17			C33 H28 O17	C33 H28 O17	-6.78	2
Cpd 79: Maclurin 3-C-(2''-p-hydroxybenzoyl-6''-galloyl-glucoside)	16.008	696.137		Maclurin 3-C-(2''-p-hydroxybenzoyl-6''-galloyl-glucoside)	C33 H28 O17			C33 H28 O17	C33 H28 O17	-6.3	2
Compound 80	16.174		65120								
Cpd 81: Tricin 7-[sinapoyl-(->2)-glucuronyl-(1->2)-glucuronide]	16.296	888.1767	59384	Tricin 7-[sinapoyl-(->2)-glucuronyl-(1->2)-glucuronide]	C40 H40 O23			C40 H40 O23	C40 H40 O23	21.75	1
Cpd 82: 10-Deoxymethymycin	16.356	453.3102	25641	10-Deoxymethymycin	C25 H43 N O6			C25 H43 N O6	C25 H43 N O6	-2.54	2
Compound 83	16.464		48651								
Compound 84	16.52		43499								
Compound 85	16.583										
Compound 86	16.825		46256								
Compound 87	16.855		38493								
Compound 88	17.193		29289								
Cpd 89: Asterosterol	17.457	370.3172	58845	Asterosterol	C26 H42 O			C26 H42 O	C26 H42 O	17.21	3
Cpd 90: alpha-Amyrin acetate	18.075	468.4028	28256	alpha-Amyrin acetate	C32 H52 O2			C32 H52 O2	C32 H52 O2	-13	8
Compound 91	18.076										
Compound 92	18.179		24276								
Compound 93	18.33		15922								
Compound 94	18.366		27581								
Cpd 95: alpha-Amyrin acetate	18.373	468.4027		alpha-Amyrin acetate	C32 H52 O2			C32 H52 O2	C32 H52 O2	-12.77	8
Cpd 96: N-[2-(1H-Indol-3-yl)ethyl]docosanamide	18.47	482.4186		N-[2-(1H-Indol-3-yl)ethyl]docosanamide	C32 H54 N2 O			C32 H54 N2 O	C32 H54 N2 O	10.4	2
Cpd 97: N-[2-(1H-Indol-3-yl)ethyl]docosanamide	18.72	482.4188		N-[2-(1H-Indol-3-yl)ethyl]docosanamide	C32 H54 N2 O			C32 H54 N2 O	C32 H54 N2 O	10.04	2
Cpd 98: 22,23-Dihydroergosterol	18.864	398.3498		22,23-Dihydroergosterol	C28 H46 O			C28 H46 O	C28 H46 O	12.7	10
Cpd 99: Atenolol	19.04	266.1615		Atenolol	C14 H22 N2 O3			C14 H22 N2 O3	C14 H22 N2 O3	5.86	9
Cpd 100: Atenolol	19.387	266.1618		Atenolol	C14 H22 N2 O3			C14 H22 N2 O3	C14 H22 N2 O3	4.85	9
Cpd 101: Atenolol	19.766	266.1619		Atenolol	C14 H22 N2 O3			C14 H22 N2 O3	C14 H22 N2 O3	4.28	9

**Compound Label**	**Name**	***m/z***	**RT**	**Algorithm**	**Mass**
Cpd 2: (S)-Menthone 8-thioacetate	**(S)-Menthone 8-thioacetate**	273.1155	3.058	Auto MS/MS	228.1178

MS Spectrum

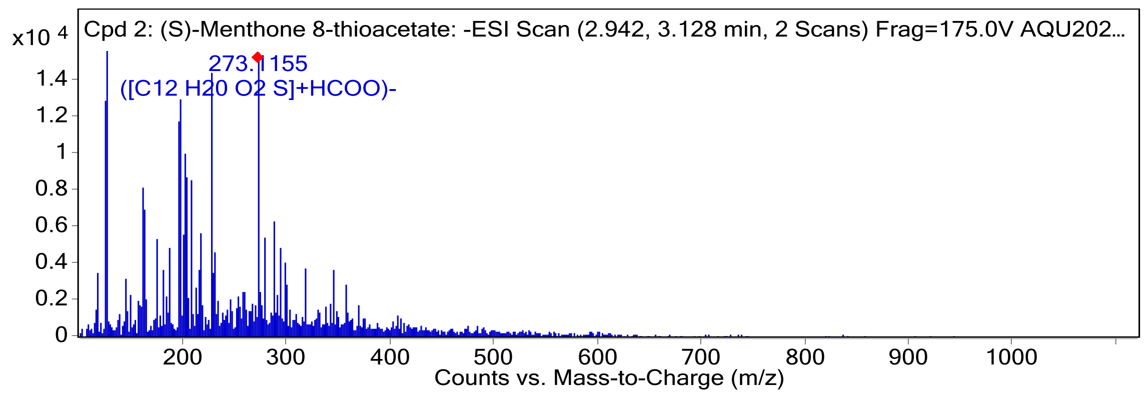

MS Zoomed Spectrum

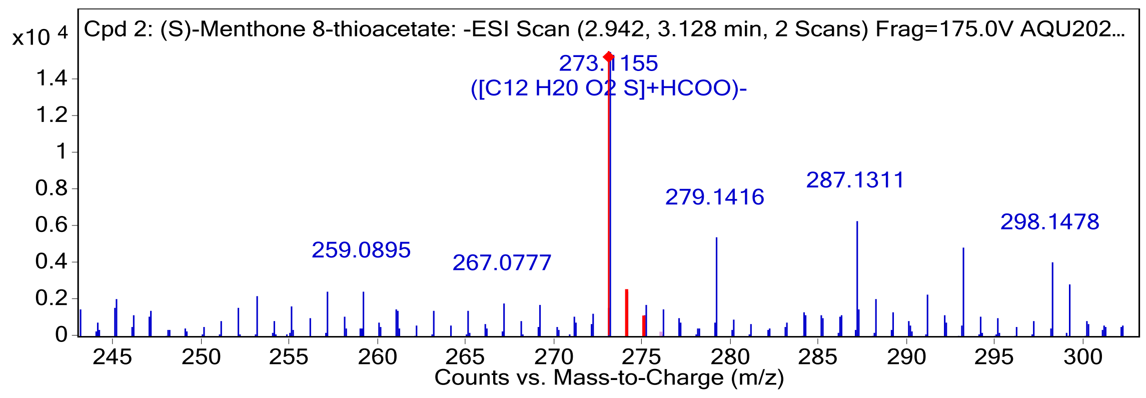

MS Spectrum Peak List

m/z	*Calc m/z*	Diff(ppm)	z	Abund	Formula	Ion
124.9954				12911.85		
126.9086				23000.57		
195.8158				11814.01		

197.8131				12960.65		
201.1293			1	10048.86		
203.0612				8751.28		
227.109			1	14401.94		
273.1155	273.1166	4.14	1	15563.89	C12 H20 O2 S	(M+HCOO)-
274.1179	274.1198	7.11	1	2503.33	C12 H20 O2 S	(M+HCOO)-
275.1207	275.1153	-19.6	1	1795.48	C12 H20 O2 S	(M+HCOO)-

MSMS Spectrum

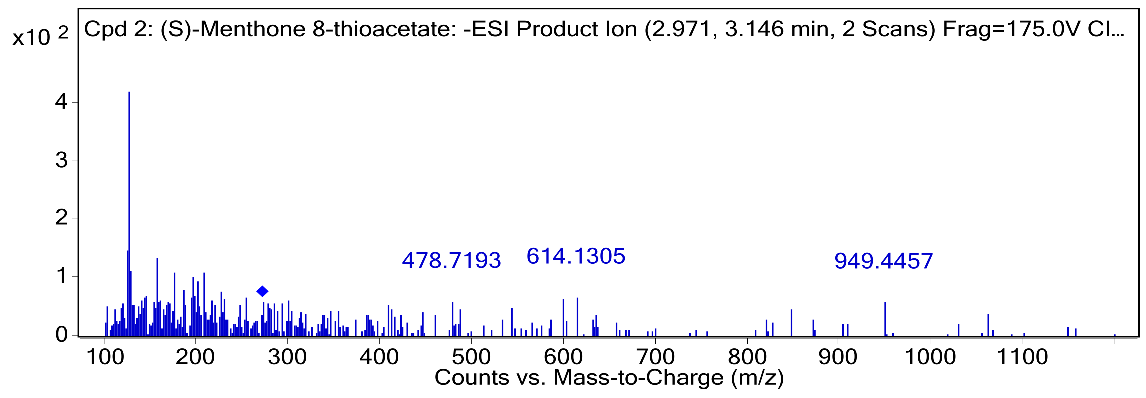

MS/MS Spectrum Peak List

m/z	Abund
124.9958	147.87
126.9098	420.22
128.04	113.55
157.1376	135.71
175.0636	109.97
185.0886	80.1
195.8152	102.63
201.1279	95.83
208.0692	111.06
227.1092	78.46

Compound Structure

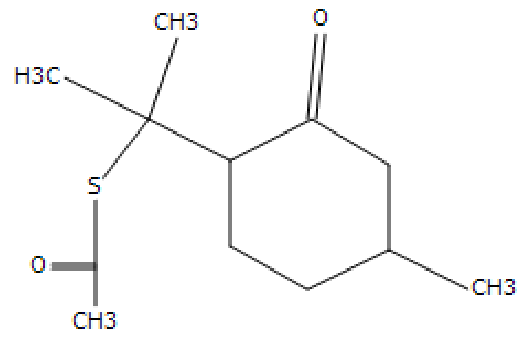

Compound Label	Name	*m/z*	RT	Algorithm	Mass
Cpd 3: (E,E)-1,6-bis(4-methoxyphenyl)-1,5-hexadiene	**(E,E)-1,6-bis(4-methoxyphenyl)-1,5-hexadiene**	293.1573	3.884	Auto MS/MS	294.1643

MS Spectrum

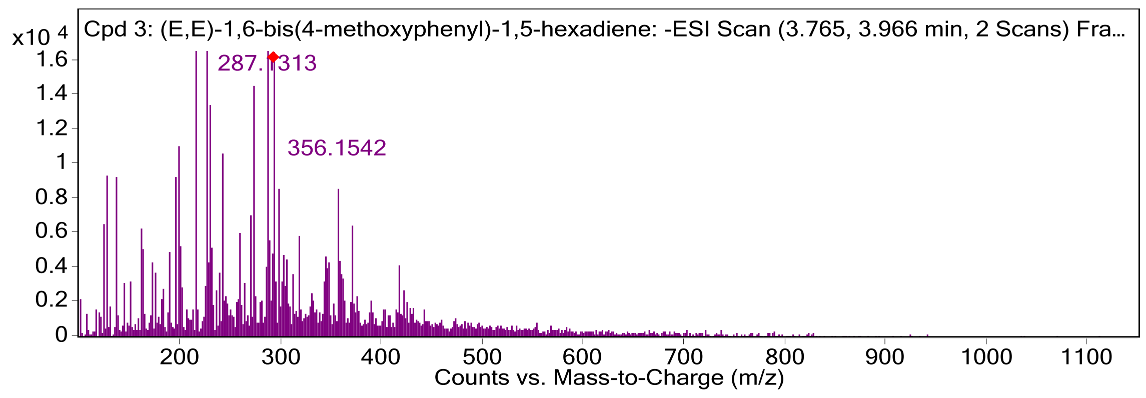

MS Zoomed Spectrum

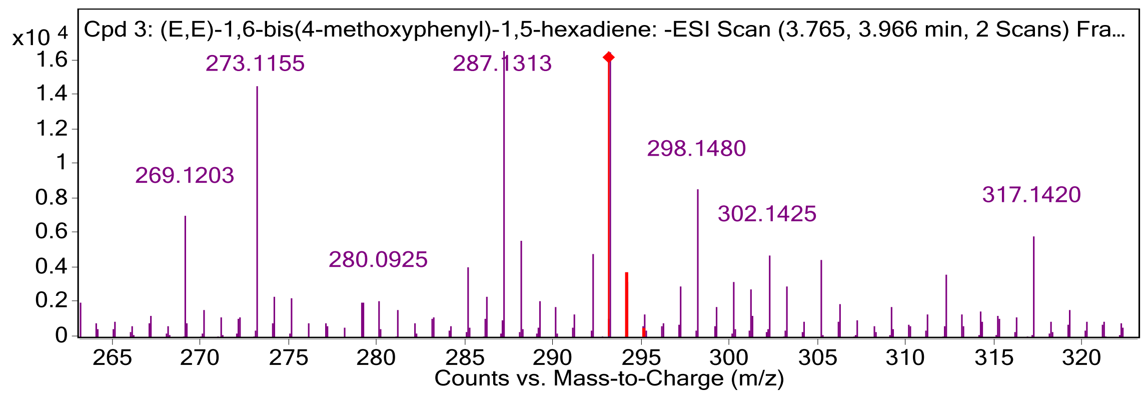

MS Spectrum Peak List

m/z	*Calc m/z*	Diff(ppm)	z	Abund	Formula	Ion
197.8132				11025.4		
215.1088				12041.42		
215.1453			1	30071.37		
227.1088			1	21747.04		
229.1248				13446.24		
273.1155			1	14545.25		
287.1313			1	36711.79		
293.1573	293.1547	-8.76	1	16505.61	C20 H22 O2	(M-H)-
294.1596	294.1581	-5.25	1	3250.72	C20 H22 O2	(M-H)-
295.1601	295.1611	3.32	1	412.39	C20 H22 O2	(M-H)-

MSMS Spectrum

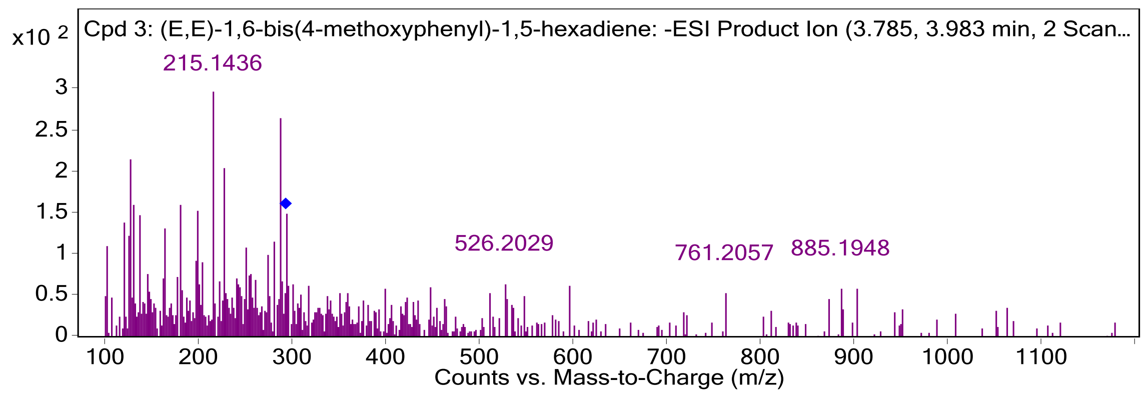

MS/MS Spectrum Peak List

m/z	z	Abund
119.0534		139.15
126.9086		215.28
129.1078		159.57
137.0272		147.34
180.0714		160.07
197.8121		153.89
215.1436		297.88
227.1078	1	205.04
287.1285	1	265.97
293.1559	1	149.54

Compound Structure

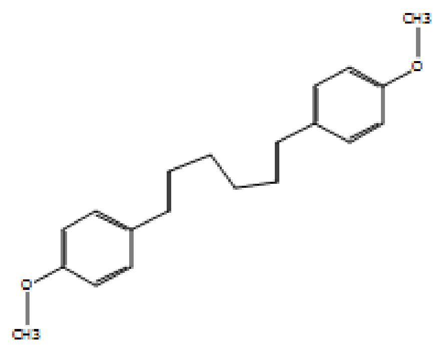

Compound Label	Name	*m/z*	RT	Algorithm	Mass
Cpd 4: 1,3,5,11-Bisabolatetraen-10-one	**1,3,5,11-Bisabolatetraen-10-**	215.1455	3.993	Auto MS/MS	216.1528

MS Spectrum

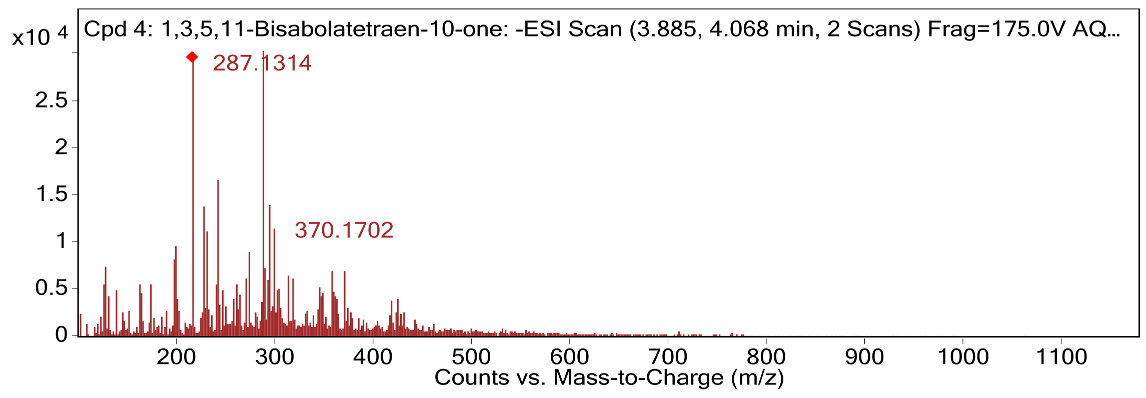

MS Zoomed Spectrum

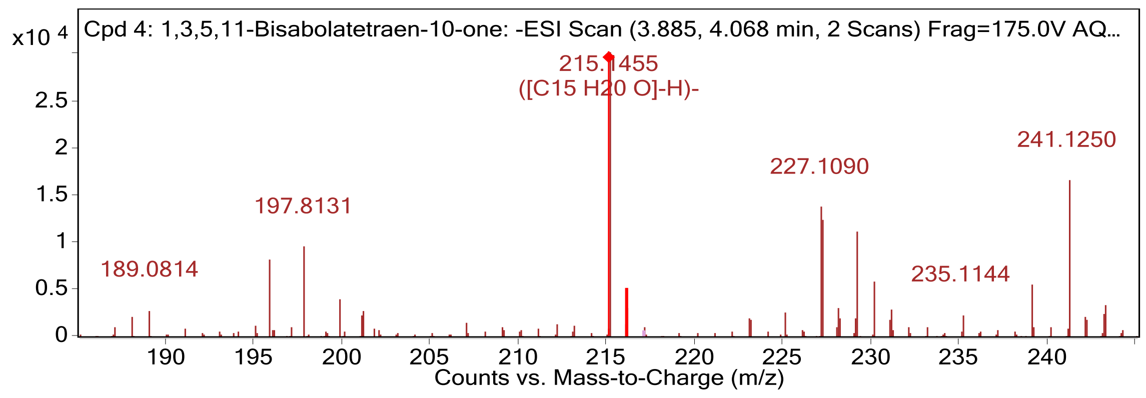

MS Spectrum Peak List

m/z	*Calc m/z*	Diff(ppm)	z	Abund	Formula	Ion
197.8131				9603.54		
215.1455	215.1441	-6.5	1	30228.15	C15 H20 O	(M-H)-
216.1489	216.1475	-6.16	1	3054.55	C15 H20 O	(M-H)-
227.109				13808.73		
227.1454			1	12521.3		
229.1247				11228.83		
241.125				16669.14		
287.1314			1	46583.63		
293.1572			1	13953.03		
298.1482			1	11565.37		

MSMS Spectrum

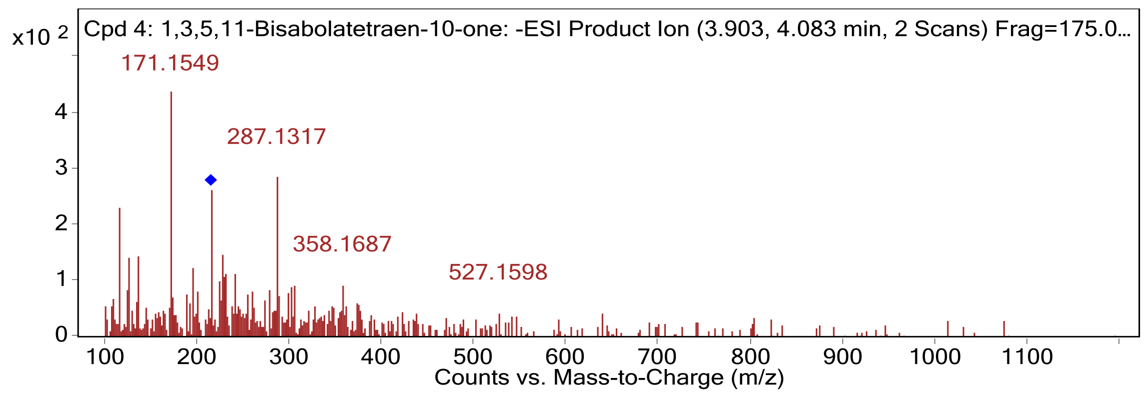

MS/MS Spectrum Peak List

m/z	z	Abund
116.0754		231.3
126.9086		141.51
137.0289		145.46
171.1549		438.9
195.8159		124.11
215.1461	1	263.1
227.1086		146.7
231.0922		113.08
241.1251		113.74
287.1317	1	285.42

Compound Structure

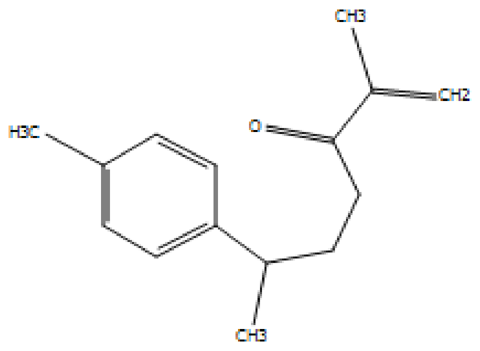

Compound Label	Name	*m/z*	RT	Algorithm	Mass
Cpd 5: 4,4'-(2-Methylpropylidene)bisphe	**4,4'-(2-Methylpropylidene)bis**	241.1251	4.037	Auto MS/MS	242.1323

MS Spectrum

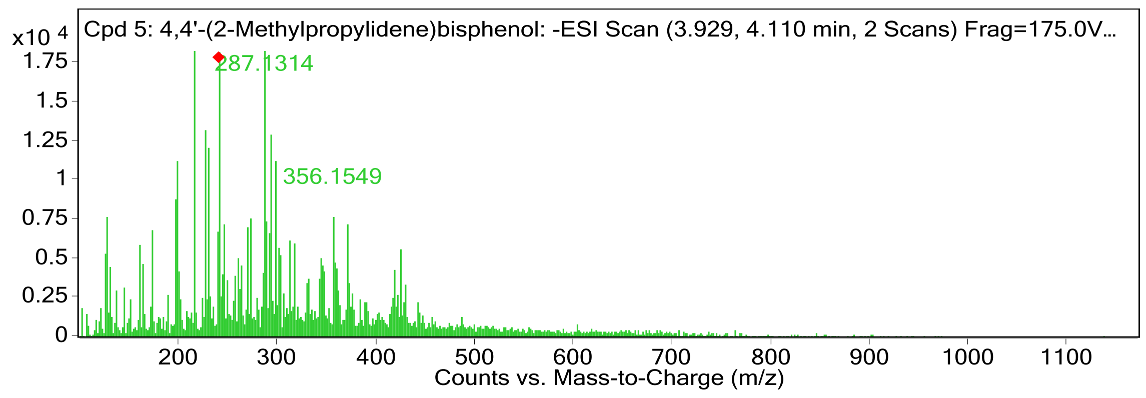

MS Zoomed Spectrum

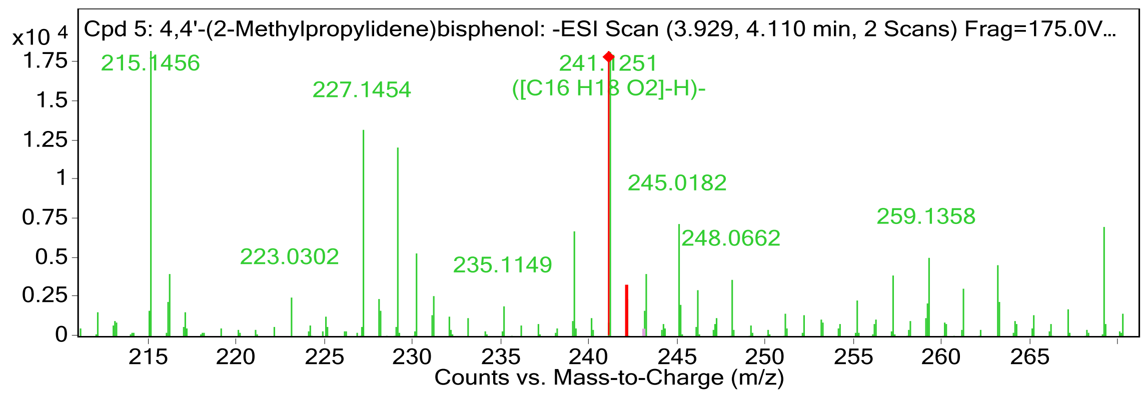

MS Spectrum Peak List

m/z	*Calc m/z*	Diff(ppm)	z	Abund	Formula	Ion
197.8131				11262.28		
215.1456			1	32540.95		
227.1087				9283.82		
227.1454				13201		
229.1251				12114.53		
241.1251	241.1234	-6.93	1	18195.04	C16 H18 O2	(M-H)-
242.1283	242.1268	-6.3	1	2264.24	C16 H18 O2	(M-H)-
287.1314			1	49998.71		
293.1573			1	12960.71		
298.1481			1	11279.27		

MSMS Spectrum

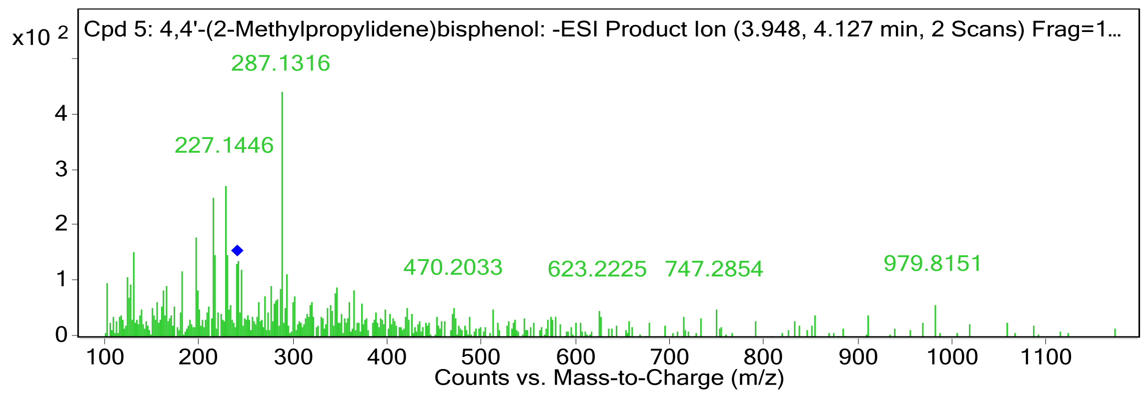

MS/MS Spectrum Peak List

m/z	z	Abund
130.092		152.35
195.8167		180.95
215.1432		250.17
216.1468		149.2
227.1446		272.31
229.1228	1	148.76
239.0624		133.47
241.1244		137.41
245.0172		122.65
287.1316	1	442.46

Compound Structure

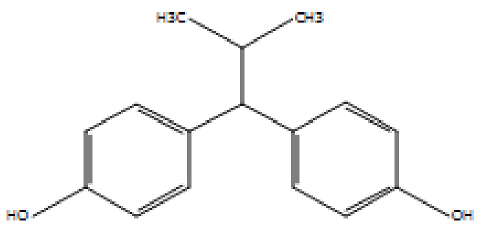

Compound Label	Name	*m/z*	RT	Algorithm	Mass
Cpd 6: Ethylketocyclazocine	**Ethylketocyclazocine**	358.206	4.468	Auto MS/MS	299.1919

MS Spectrum

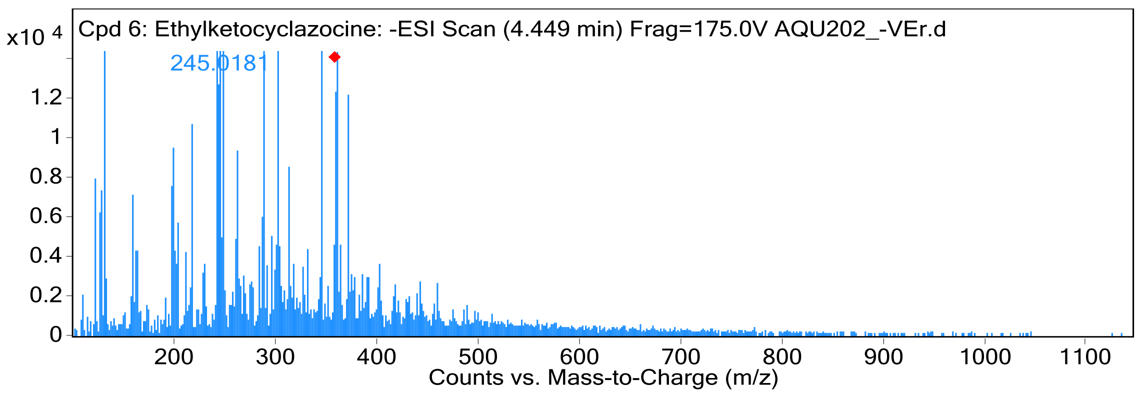

Cpd 6: Ethylketocyclazocine: -ESI Scan (4.449 min) Frag=175.0V AQU202_-VEr.d
x10 4
245.0181
1.2
1
0.8
0.6
0.4
0.2
0
200
300
400
500
600
700
800
900
1000
1100
Counts vs. Mass-to-Charge (m/z)

MS Zoomed Spectrum

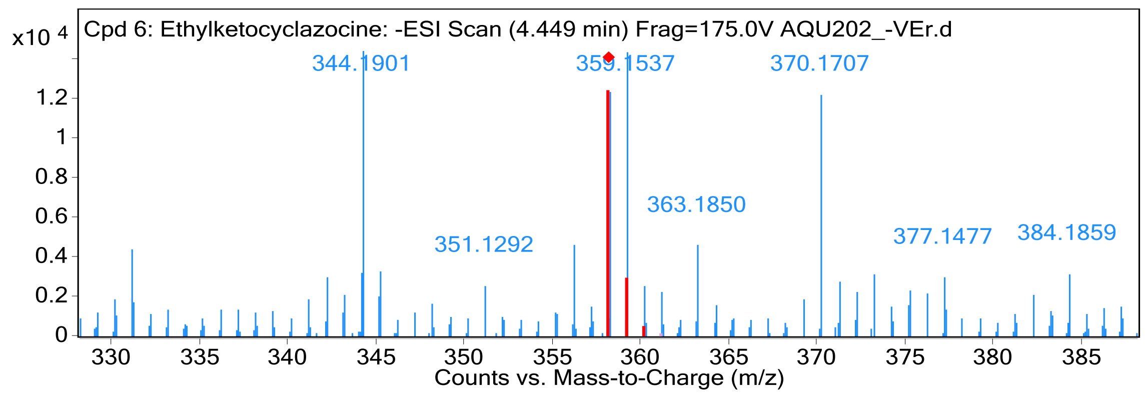

MS Spectrum Peak List

m/z	*Calc m/z*	Diff(ppm)	z	Abund	Formula	Ion
130.0911			1	32971.8		
241.1249			1	31056.22		
245.0181			1	41673.25		
248.0656			1	16412.34		
287.1313			1	23330.15		
301.1472			1	27342.23		
344.1901			1	16949.05		
358.206	358.2024	-10.22	1	12391	C19 H25 N O2	(M+CH3COO)-
359.2084	359.2057	-7.52	1	2317.47	C19 H25 N O2	(M+CH3COO)-
360.2096	360.2084	-3.31	1	742.18	C19 H25 N O2	(M+CH3COO)-

MSMS Spectrum

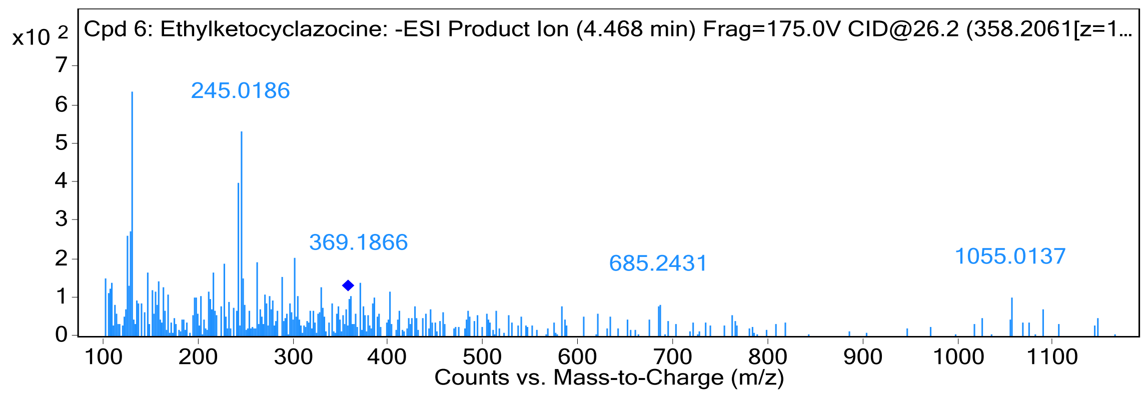

MS/MS Spectrum Peak List

m/z	z	Abund
124.9958	1	262.02
128.0389		274.06
130.0903		637.64
146.0488		166.84
215.1089		167.41
227.1076		188.89
241.1258		401.9
245.0186	1	534.12
261.1293		195.53
301.1451		204.23

Compound Structure

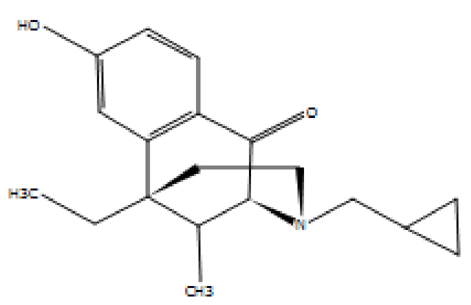

Compound Label	Name	*m/z*	RT	Algorithm	Mass
Cpd 7: (E,E)-1,6-bis(4-methoxyphenyl)-1,5-hexadiene	**(E,E)-1,6-bis(4-methoxyphenyl)-1,5-hexadiene**	293.1574	4.599	Auto MS/MS	294.1646

MS Spectrum

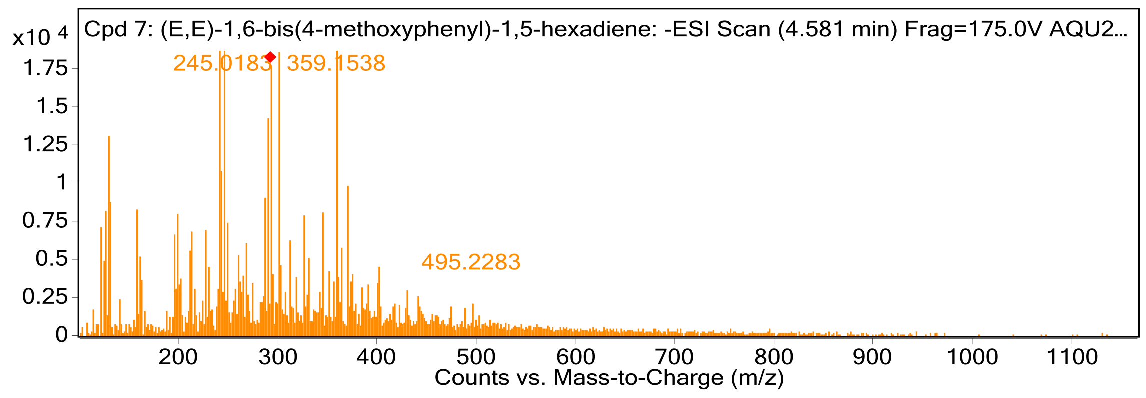

MS Zoomed Spectrum

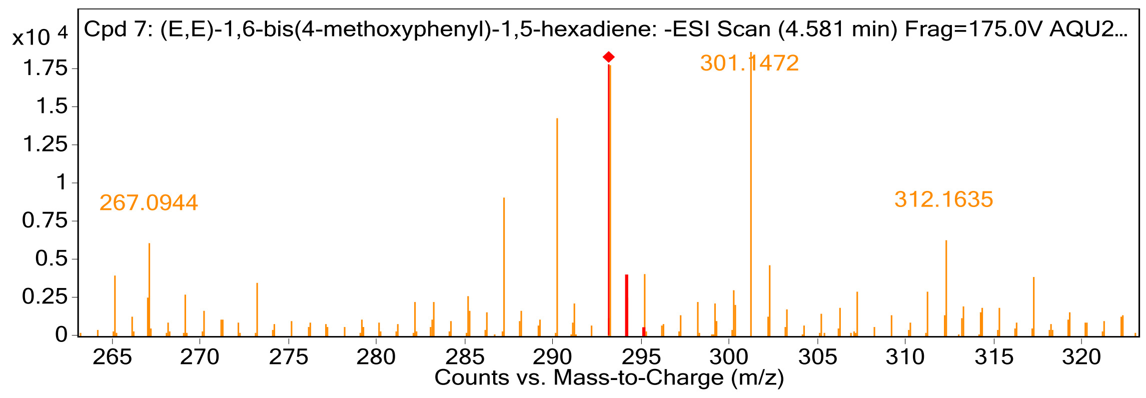

MS Spectrum Peak List

m/z	*Calc m/z*	Diff(ppm)	z	Abund	Formula	Ion
130.0912				13140.29		
241.125			1	20484.67		
243.1407				10914		
245.0183			1	64246.69		
290.1311			1	14353.81		
293.1574	293.1547	-9.19	1	17779.37	C20 H22 O2	(M-H)-

294.1598	294.1581	-5.91	1	2603.05	C20 H22 O2	(M-H)-
295.1636	295.1611	-8.4	1	355.34	C20 H22 O2	(M-H)-
301.1472			1	18662.69		
359.1538			1	19091.38		

MSMS Spectrum

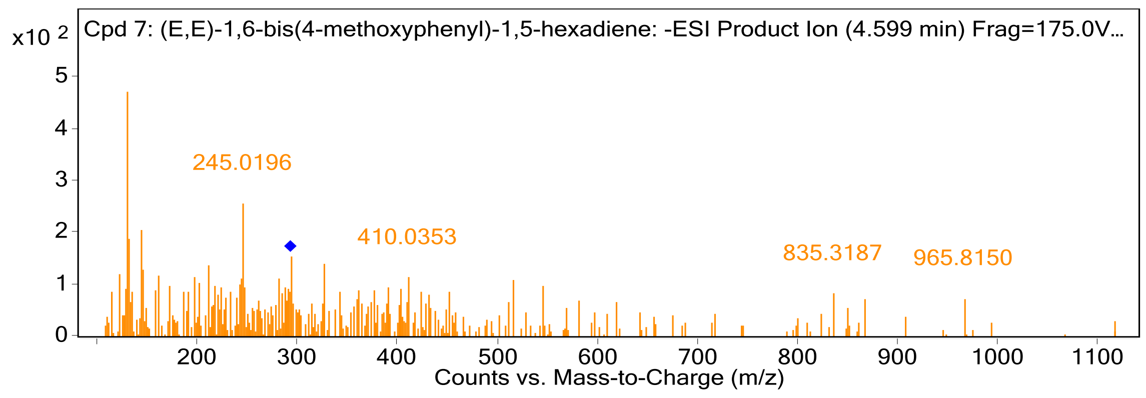

MS/MS Spectrum Peak List

m/z	z	Abund
121.0334		123.04
130.091	1	473.88
131.0758		190.04
143.1218		205.25
144.9433		129.68
211.0339		138.7
245.0196		258.18
245.1182		125.23
293.1605		154.44
326.1786		141.31

Compound Structure

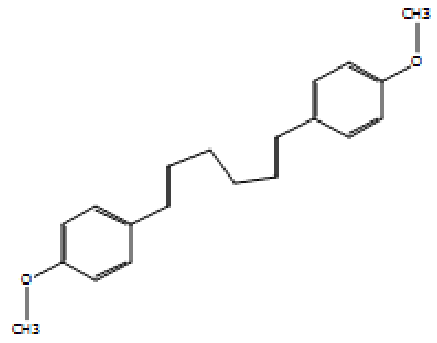

Compound Label	Name	*m/z*	RT	Algorithm	Mass
Cpd 8: 4,4'-(2-Methylpropylidene)bisphe	**4,4'-(2-Methylpropylidene)bis**	241.125	4.782	Auto MS/MS	242.1323

MS Spectrum

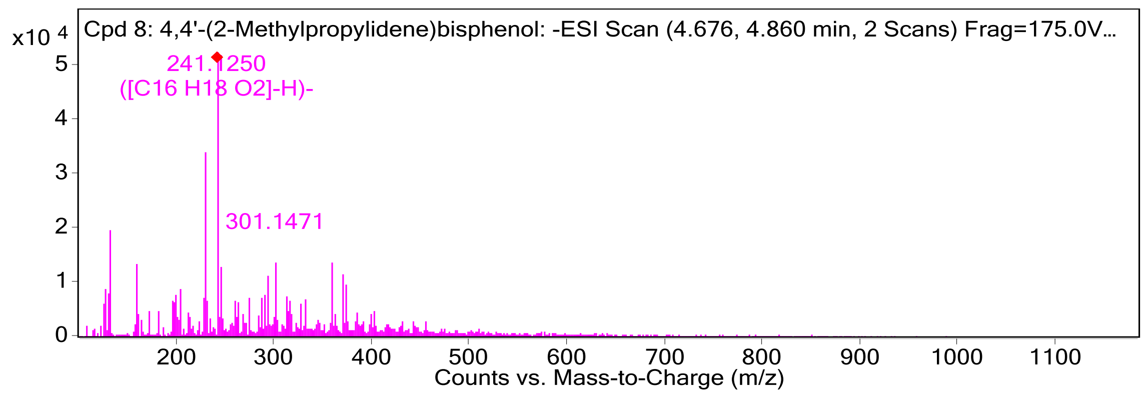

MS Zoomed Spectrum

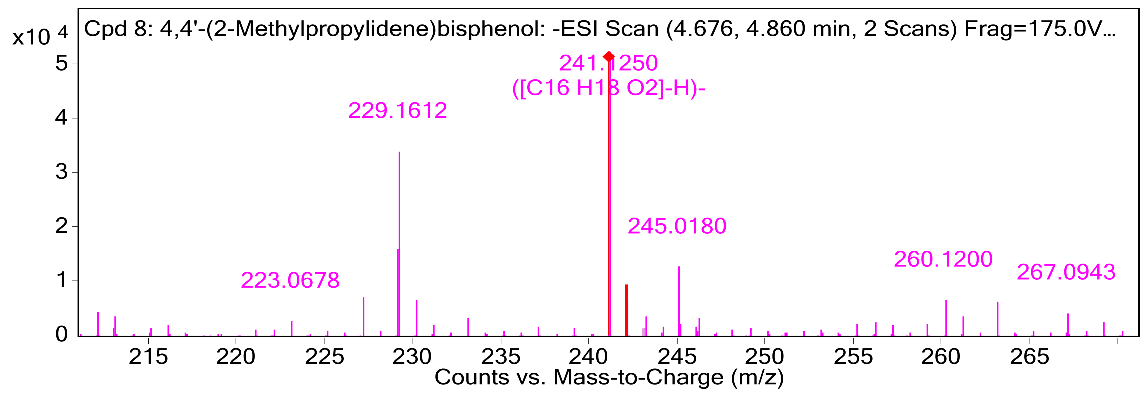

MS Spectrum Peak List

m/z	*Calc m/z*	Diff(ppm)	z	Abund	Formula	Ion
131.0754				19806.6		
158.0861				13483.89		
229.1249			1	16206.72		
229.1612			1	34142.25		
241.125	241.1234	-6.71	1	52396.97	C16 H18 O2	(M-H)-
242.1281	242.1268	-5.45	1	6729.82	C16 H18 O2	(M-H)-
245.018				12837.34		
301.1471			1	13777.53		
358.2063			1	13719.88		
370.1702			1	11546.47		

MSMS Spectrum

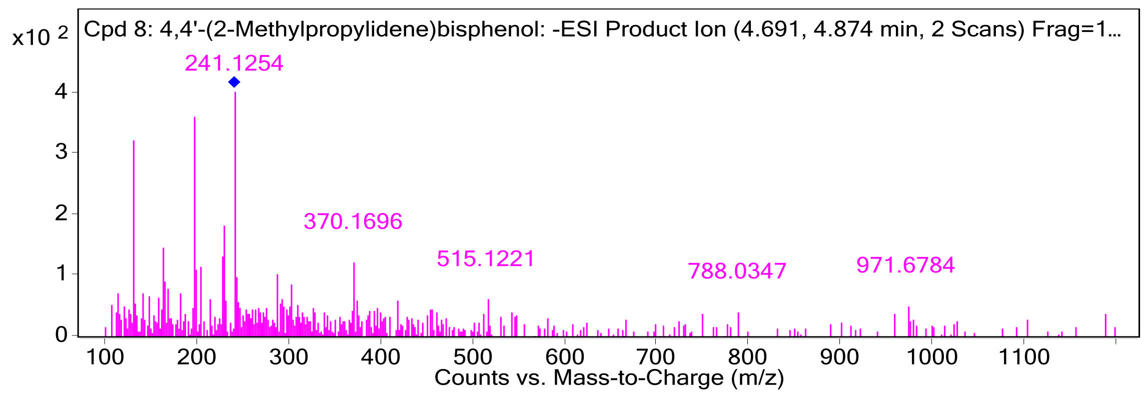

MS/MS Spectrum Peak List

m/z	z	Abund
131.0753	1	322.88
162.8447		148.05
197.1348	1	362.11

198.1374	1	111.08
203.0973		116.72
227.1097		132.87
229.1638		182.53
241.1254	1	402.91
287.1292		103.22
370.1696		122.79

Compound Structure

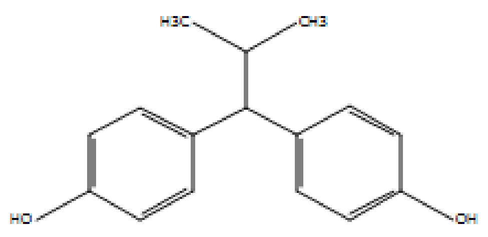

Compound Label	Name	m/z	RT	Algorithm	Mass
Cpd 9: Ethylketocyclazocine	**Ethylketocyclazocine**	358.2059	4.842	Auto MS/MS	299.1914

MS Spectrum

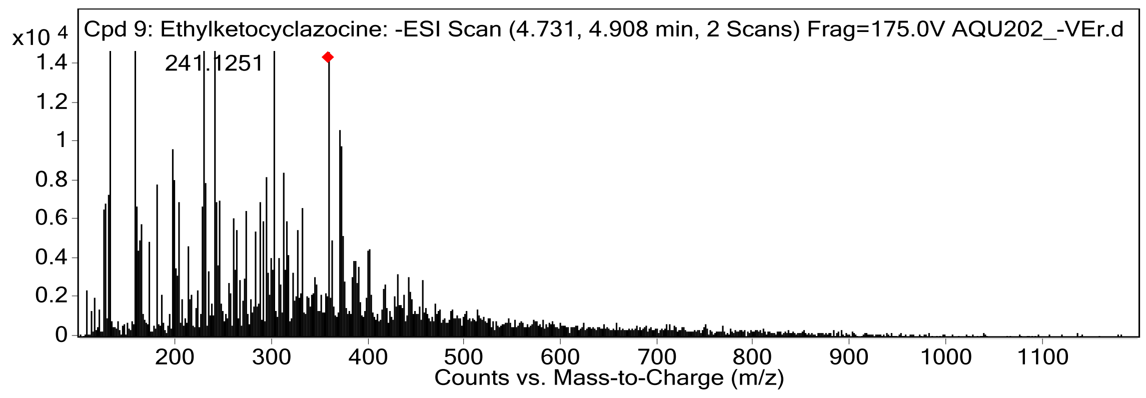

MS Zoomed Spectrum

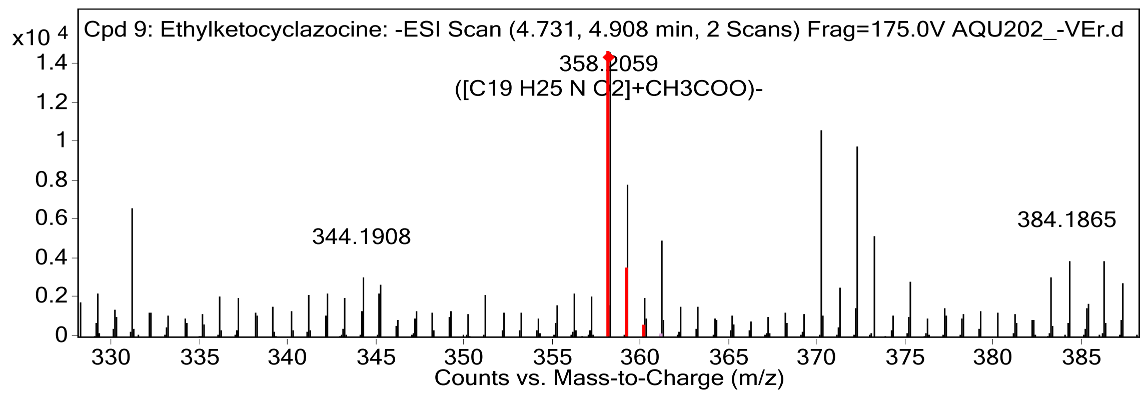

MS Spectrum Peak List

m/z	Calc m/z	Diff(ppm)	z	Abund	Formula	Ion
131.0753				19519.83		
158.086				15199.23		
229.1248				16033.15		
229.1612			1	45747.51		
241.1251			1	64147.2		
301.1471			1	17628.07		
358.2059	358.2024	-9.79	1	14625.2	C19 H25 N O2	(M+CH3COO)-
359.2079	359.2057	-6.12	1	2980.03	C19 H25 N O2	(M+CH3COO)-
360.2033	360.2084	14.36	1	949.76	C19 H25 N O2	(M+CH3COO)-
370.1709			1	10621.31		

MSMS Spectrum

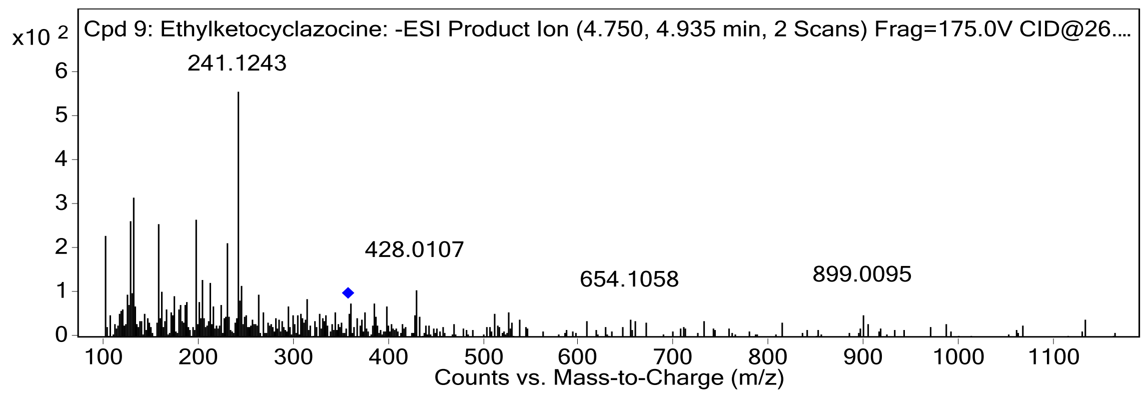

MS/MS Spectrum Peak List

m/z	z	Abund
102.0602		228.29
128.0388		263.95
131.0754	1	315.91
158.0864	1	255.31
197.1336		265.3
203.098		129.84
211.1481		122
229.1618		211.61
241.1243		556.58
245.0176		116.42

Compound Structure

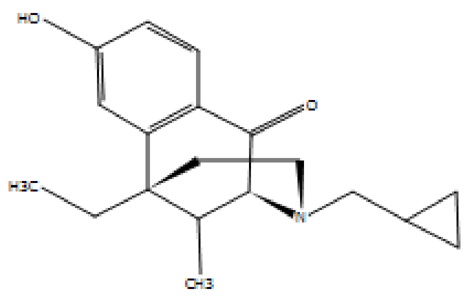

Compound Label	Name	*m/z*	RT	Algorithm	Mass
Cpd 10: S-Methyl-1-thio-D-glycerate	**S-Methyl-1-thio-D-glycerate**	181.0193	5.018	Auto MS/MS	136.0212

MS Spectrum

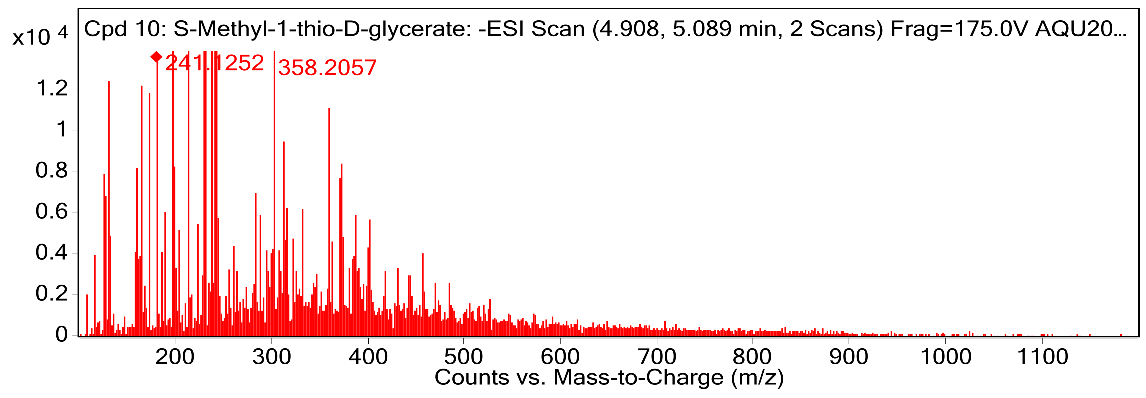

MS Zoomed Spectrum

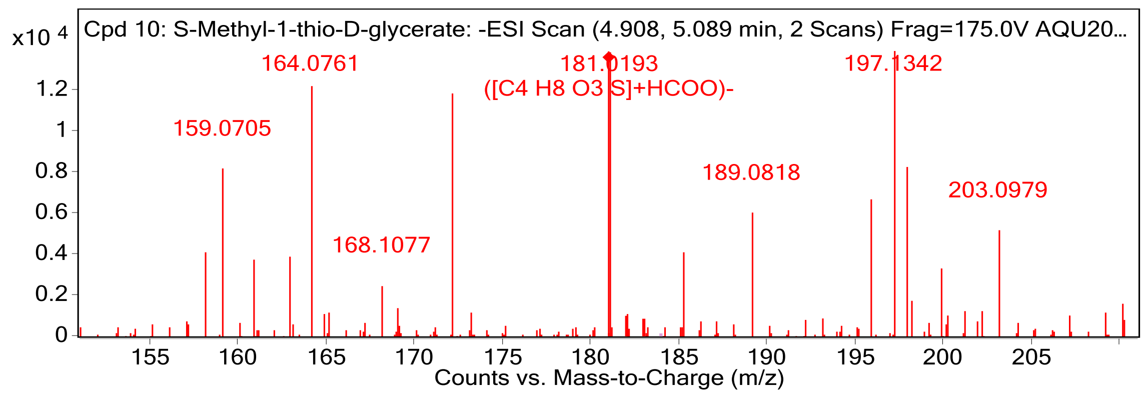

MS Spectrum Peak List

m/z	*Calc m/z*	Diff(ppm)	z	Abund	Formula	Ion
181.0193	181.0176	-9.13		13832.03	C4 H8 O3 S	(M+HCOO)-
182.0226	182.0206	-11.13	1	1118.81	C4 H8 O3 S	(M+HCOO)-
183.0233	183.0153	-44.18	1	143.56	C4 H8 O3 S	(M+HCOO)-
197.1342				16637.57		
212.0982			1	14463.87		
229.1612			1	102055.09		
230.1095				15182.45		
237.0825			1	15081.63		
241.1252			1	126031.3		
301.1472			1	20517.71		

MSMS Spectrum

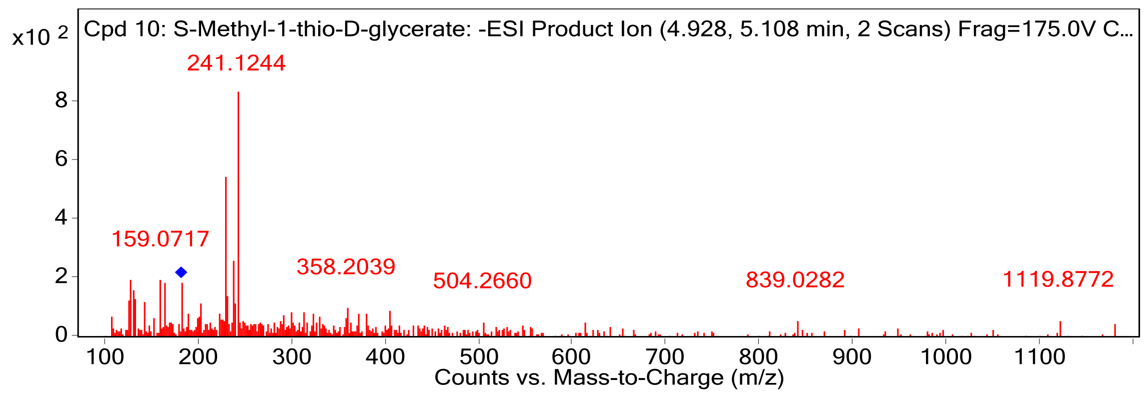

MS/MS Spectrum Peak List

m/z	z	Abund
126.909		195.1
130.0918		161.5
131.0747		130.84
159.0717		195.21
164.0739		185.35
181.0166		185.61
229.1617	1	544.33
230.1667	1	138.24
237.0814		260.74
241.1244	1	838.57

Compound Structure

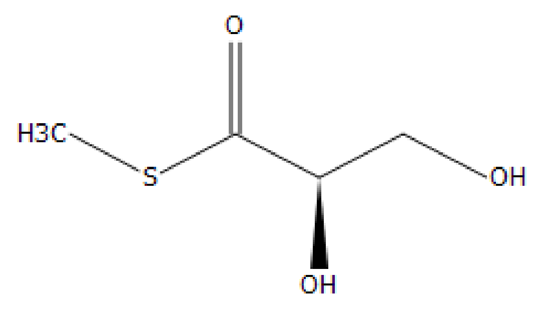

Compound Label	Name	*m/z*	RT	Algorithm	Mass
Cpd 11: 4,4'-(2-Methylpropylidene)bisphe	**4,4'-(2-Methylpropylidene)bis**	241.1253	5.139	Auto MS/MS	242.1325

MS Spectrum

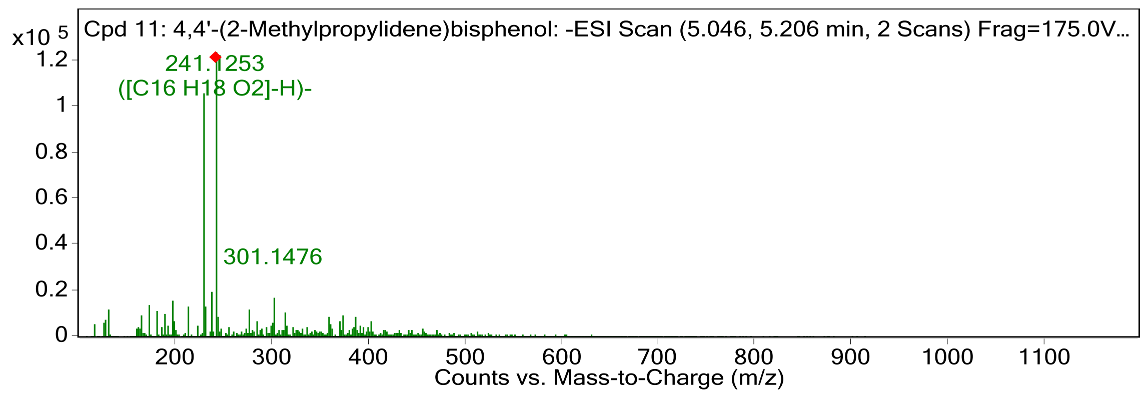

MS Zoomed Spectrum

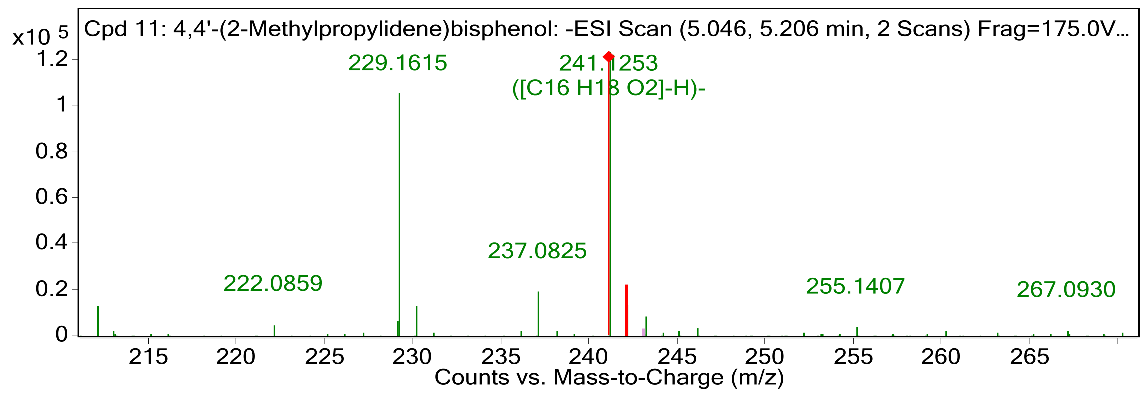

MS Spectrum Peak List

m/z	*Calc m/z*	Diff(ppm)	z	Abund	Formula	Ion
172.1026				14184.64		
197.1343				15778.64		
212.0983				13156.88		
229.1615			1	106015.27		
230.1093				13586.68		
230.165			1	12341.37		
237.0825			1	19588.41		
241.1253	241.1234	-7.71	1	123731.48	C16 H18 O2	(M-H)-
242.1285	242.1268	-6.97	1	13798.17	C16 H18 O2	(M-H)-
301.1476			1	17136.81		

MSMS Spectrum

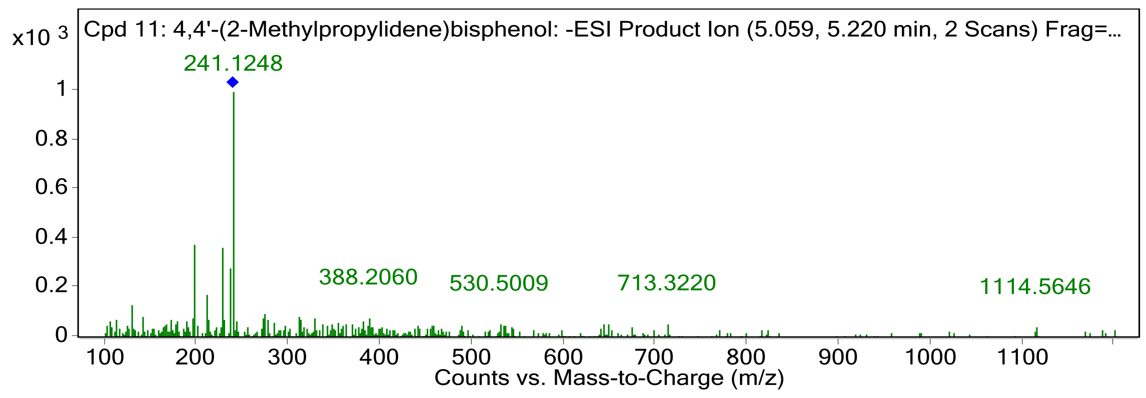

MS/MS Spectrum Peak List

m/z	z	Abund
130.0862		132.56
141.1073		84.34
197.1348	1	377.7
197.8126		114.85
212.099		172.82
229.1612	1	360.51
237.0817		276.98
241.1248		996.1
275.1103		93.75
312.1636		83.23

Compound Structure

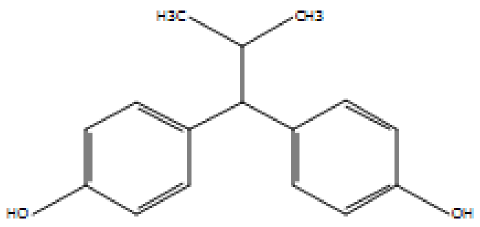

Compound Label	Name	*m/z*	RT	Algorithm	Mass
Cpd 12: 1,7-Diphenyl-4-hepten-3-one	**1,7-Diphenyl-4-hepten-3-one**	263.1465	5.377	Auto MS/MS	264.1537

MS Spectrum

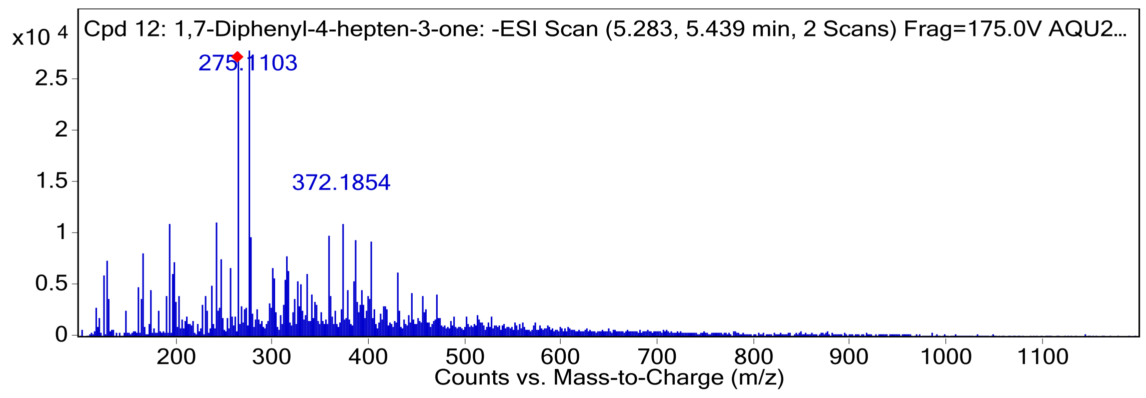

MS Zoomed Spectrum

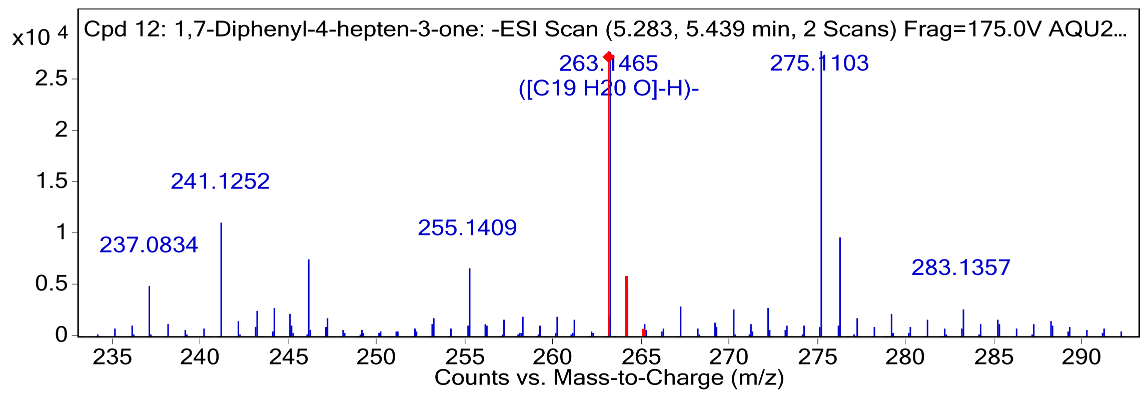

MS Spectrum Peak List

m/z	*Calc m/z*	Diff(ppm)	z	Abund	Formula	Ion
192.0714				10992.62		
241.1252				11200.21		
263.1465	263.1441	-8.9	1	27743.42	C19 H20 O	(M-H)-
264.1494	264.1475	-7.12	1	4922.3	C19 H20 O	(M-H)-
265.1527	265.1507	-7.57	1	646.73	C19 H20 O	(M-H)-
275.1103			1	59881.21		
276.1137			1	9681.31		
358.2058			1	9887.85		

372.1854				11070.87		
372.2222			1	10978.77		

MSMS Spectrum

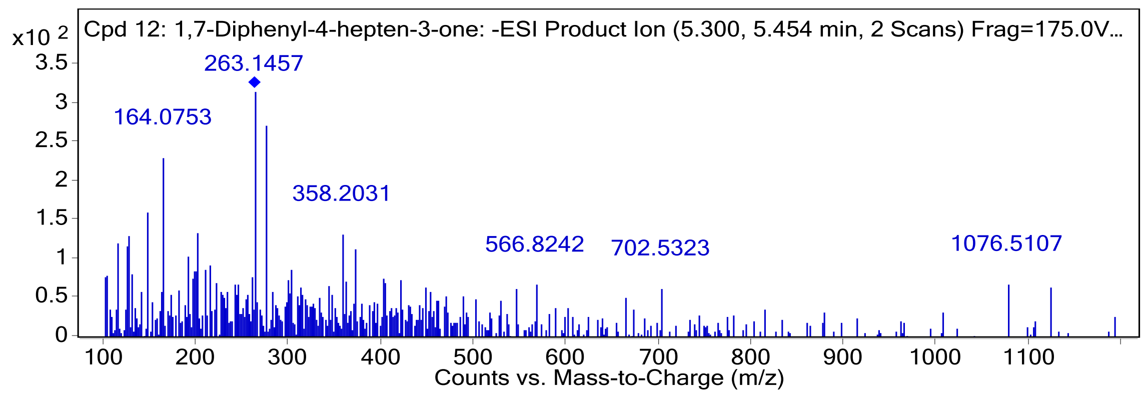

MS/MS Spectrum Peak List

m/z	z	Abund
115.0913		120.37
126.9092		129.82
147.0482		159.52
164.0753		230.24
202.1124		132.94
263.1457	1	314.86
264.1481	1	121.12
275.1084		270.69
276.1114		118.76
358.2031		132.06

Compound Structure

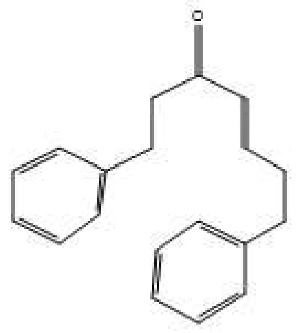

Compound Label	*m/z*	RT	Algorithm
Compound 13	217.1135	5.875	Auto MS/MS

MS Spectrum

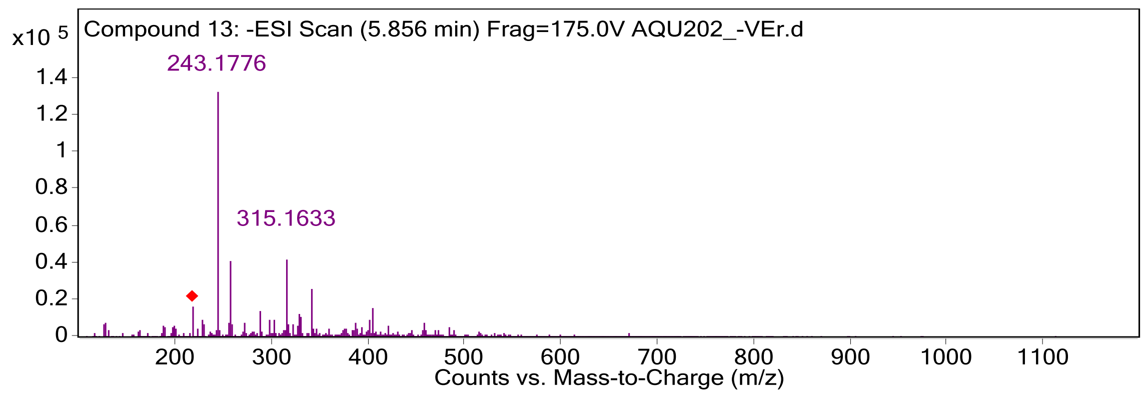

MS Zoomed Spectrum

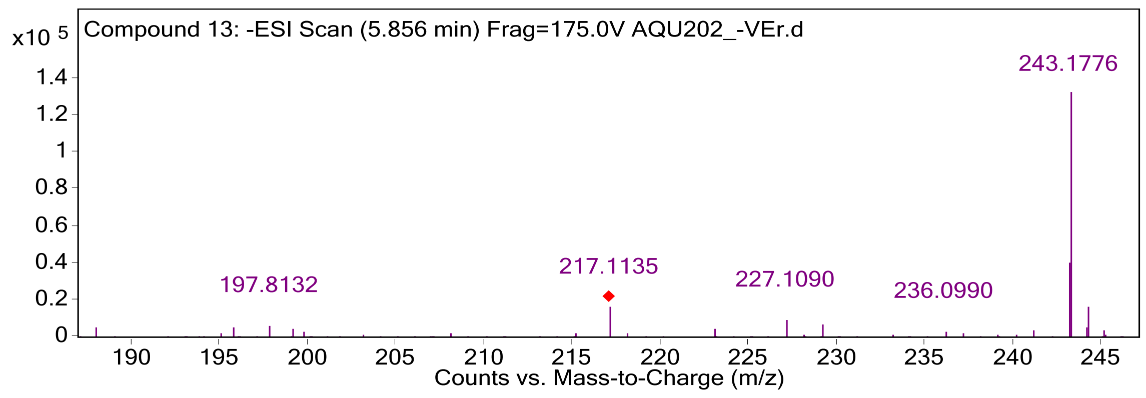

MS Spectrum Peak List

m/z	z	Abund
217.1135		16357.5
218.1169	1	2121.47
219.1241	1	451.15
243.1407	1	40305.12
243.1776	1	132897.16
244.1804	1	16676.61
257.1565	1	41283.76
315.1633	1	42247.32
340.1952	1	26210.71
403.2427	1	16242.48

MSMS Spectrum

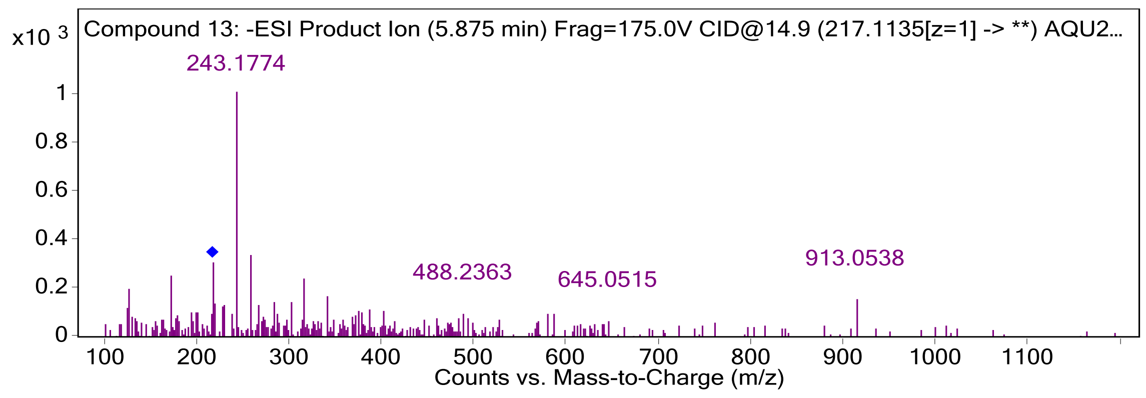

MS/MS Spectrum Peak List

m/z	z	Abund
126.9074		197.79
171.1071		252.09
217.1138	1	305.33
243.1403		223.09
243.1774		1011.29
257.1573	1	337.45
301.1478		147.03
315.161		239.07

340.195		168.35
913.0538		157.01

Compound Label	Name	*m/z*	RT	Algorithm	Mass
Cpd 14: Melanostatin	**Melanostatin**	329.1803	5.97	Auto MS/MS	284.1828

MS Spectrum

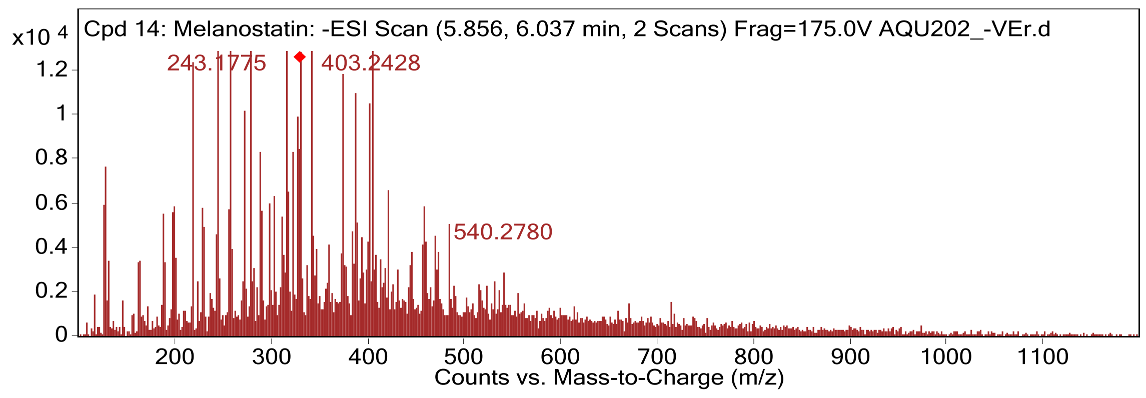

MS Zoomed Spectrum

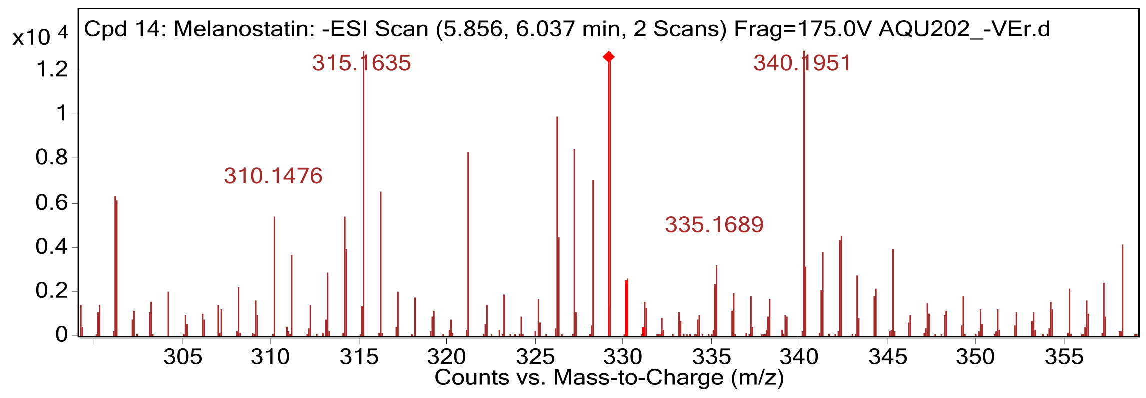

MS Spectrum Peak List

m/z	*Calc m/z*	Diff(ppm)	z	Abund	Formula	Ion
243.1407			1	23345.43		
243.1775			1	90532.62		
257.1564			1	25282.16		
277.1627			1	21733.41		
315.1635			1	25278.25		
329.1803	329.183	8.31	1	12869.84	C13 H24 N4 O3	(M+HCOO)-
330.1834	330.1859	7.53	1	2675.52	C13 H24 N4 O3	(M+HCOO)-
331.192	331.1881	-11.77	1	1325.66	C13 H24 N4 O3	(M+HCOO)-
332.1971	332.1906	-19.81	1	304.01	C13 H24 N4 O3	(M+HCOO)-
403.2428			1	17328.61		

MSMS Spectrum

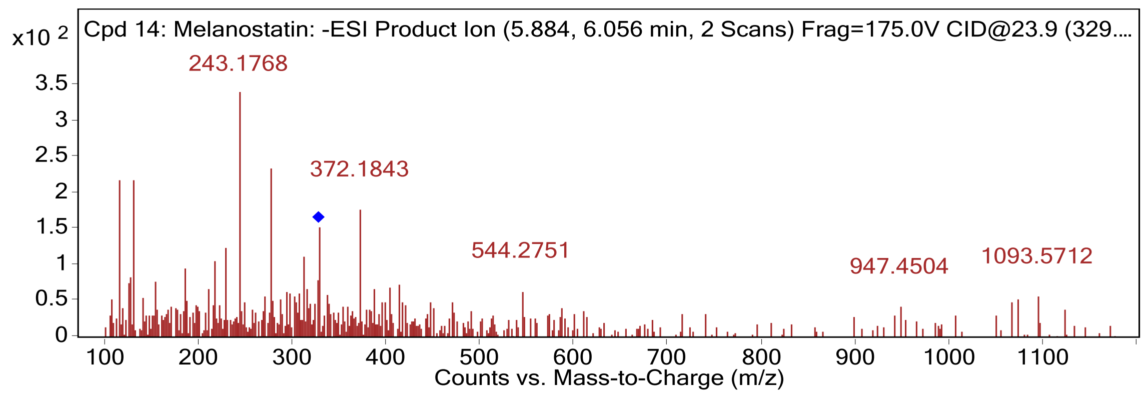

MS/MS Spectrum Peak List

m/z	z	Abund
116.0751		217.97
130.091	1	217.94
229.1597		124.14
243.1401		133.39
243.1768	1	341.13
277.1648	1	233.52
311.1675		112.11
328.1936		126.54
329.1662		152.11
372.1843		177.12

Compound Structure

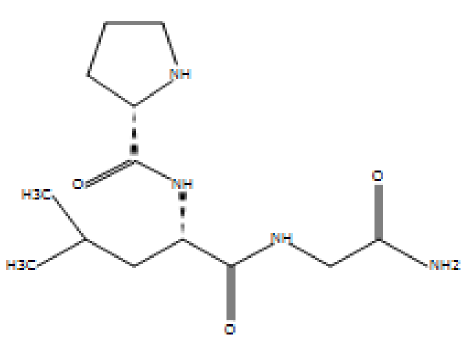

Compound Label	*m/z*	RT	Algorithm	Mass
Compound 1	271.0835	5.986	Find By Formula	212.0691

MS Spectrum

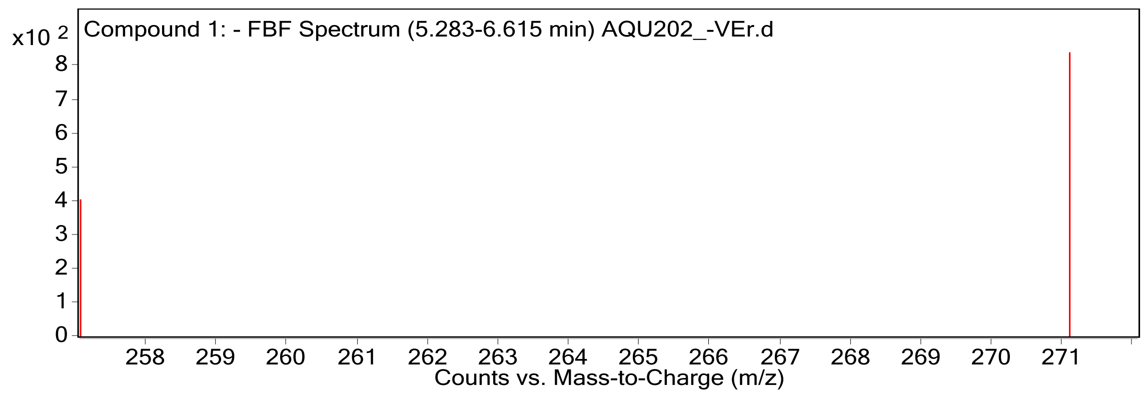

MS Zoomed Spectrum

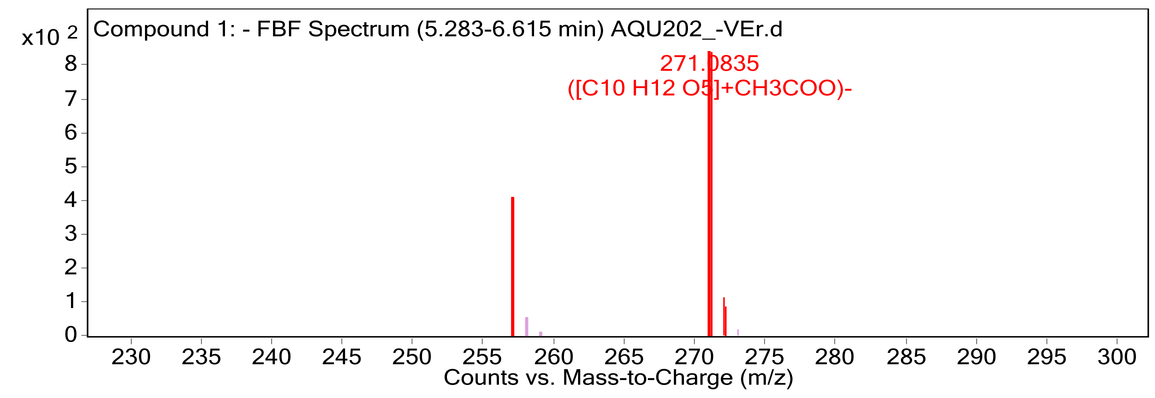

MS Spectrum Peak List

m/z	z	Abund	Formula	Ion
257.0676	1	406.34	C10 H12 O5	(M+HCOO)-
271.0835	1	841.14	C10 H12 O5	(M+CH3COO)-
272.0795	1	92.56	C10 H12 O5	(M+CH3COO)-

Compound Label	Name	*m/z*	RT	Algorithm	Mass
Cpd 15: Sethoxydim	**Sethoxydim**	372.1861	6.055	Auto MS/MS	327.186

MS Spectrum

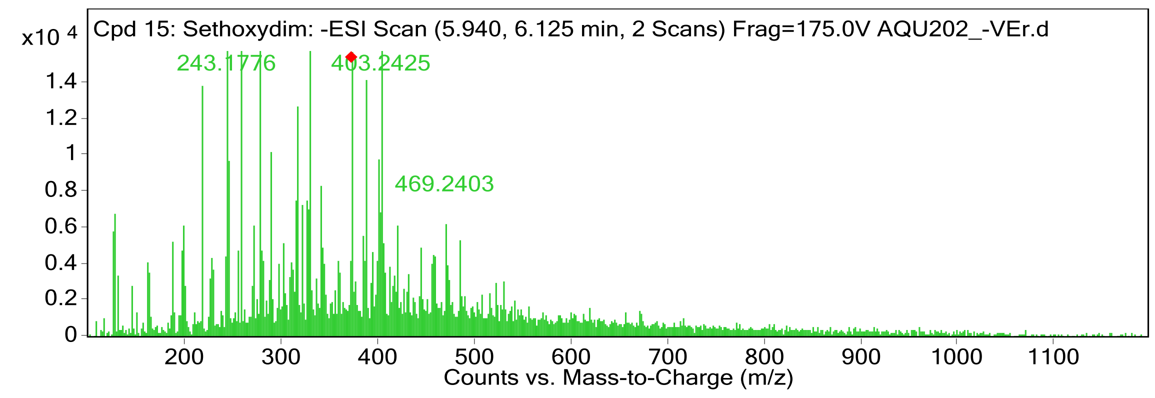

MS Zoomed Spectrum

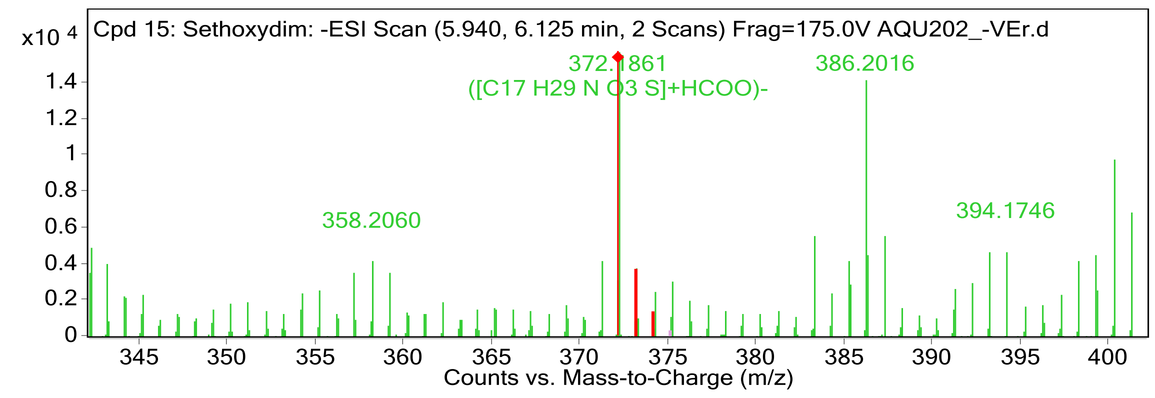

MS Spectrum Peak List

m/z	*Calc m/z*	Diff(ppm)	z	Abund	Formula	Ion
217.1134				13831.13		
243.1776			1	77435.77		
257.1564				16155.39		
277.1627			1	29131.54		
329.18				16718.04		
372.1861	372.185	-2.83	1	15681.3	C17 H29 N O3 S	(M+HCOO)-
373.1834	373.1882	12.81	1	3823.72	C17 H29 N O3 S	(M+HCOO)-
374.1788	374.1849	16.28	1	2500.25	C17 H29 N O3 S	(M+HCOO)-
386.2016			1	14108.22		
403.2425			1	20689.53		

MSMS Spectrum

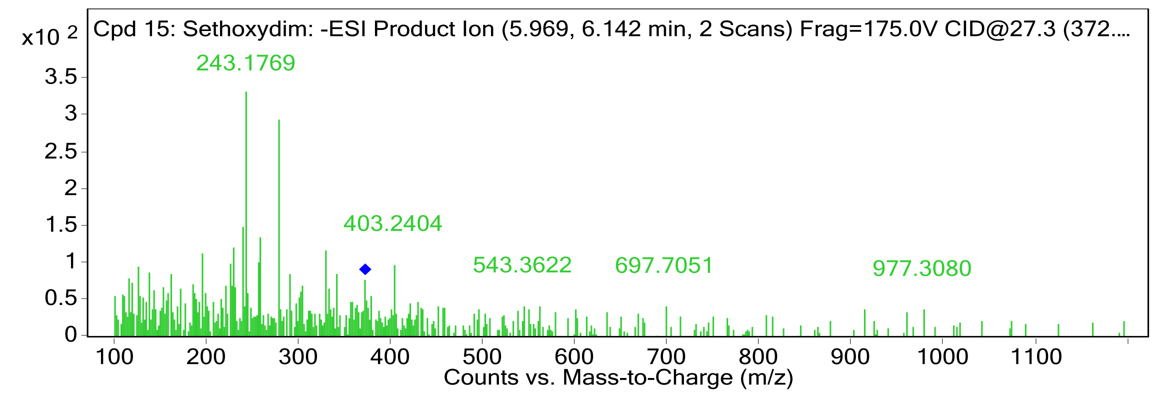

MS/MS Spectrum Peak List

m/z	z	Abund
195.8161		113.1
228.1777		104.48
229.1102		120.76
238.1607		148.79
243.1769	1	333.44
257.1555		135.35
277.1614	1	295.8
278.1665	1	120.04
328.1941		118.46
329.1788		115.71

Compound Structure

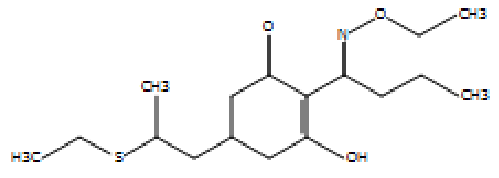

Compound Label	Name	*m/z*	RT	Algorithm	Mass
Cpd 16: Melanostatin	**Melanostatin**	329.1803	6.222	Auto MS/MS	284.182

MS Spectrum

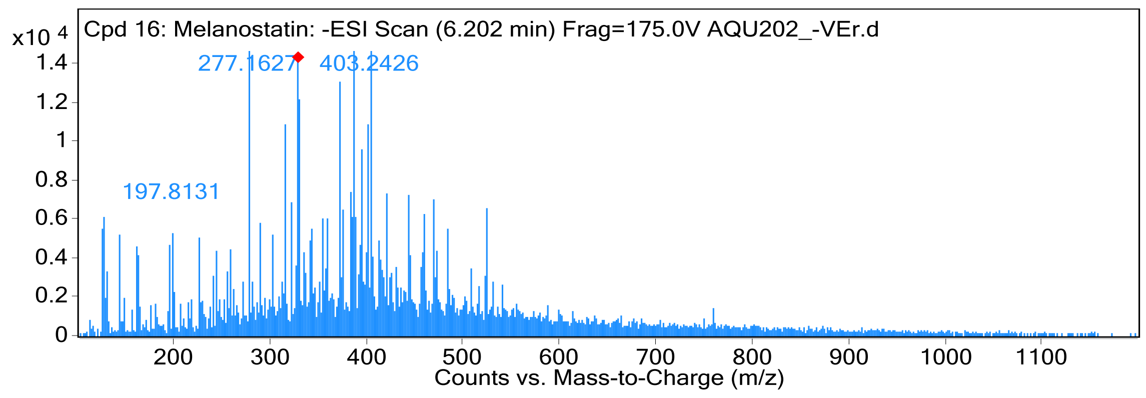

MS Zoomed Spectrum

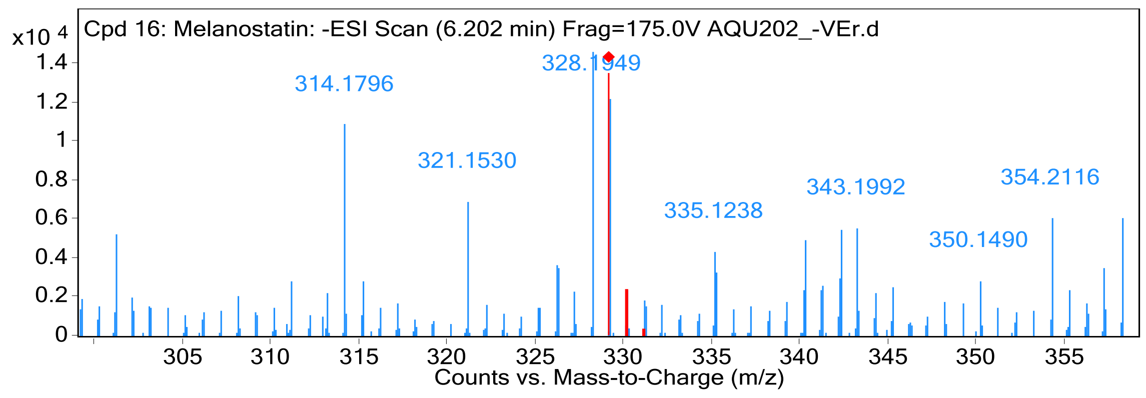

MS Spectrum Peak List

m/z	*Calc m/z*	Diff(ppm)	z	Abund	Formula	Ion
277.1627			1	71853.88		
278.166			1	12750.06		
328.1949				14602.21		
329.1803	329.183	8.47		12177.18	C13 H24 N4 O3	(M+HCOO)-
330.1811	330.1859	14.71	1	2387.64	C13 H24 N4 O3	(M+HCOO)-
331.1878	331.1881	1.07	1	1549.15	C13 H24 N4 O3	(M+HCOO)-
372.1863			1	13066.89		
386.2022			1	21526.02		
400.218				10909.28		
403.2426			1	23067.87		

MSMS Spectrum

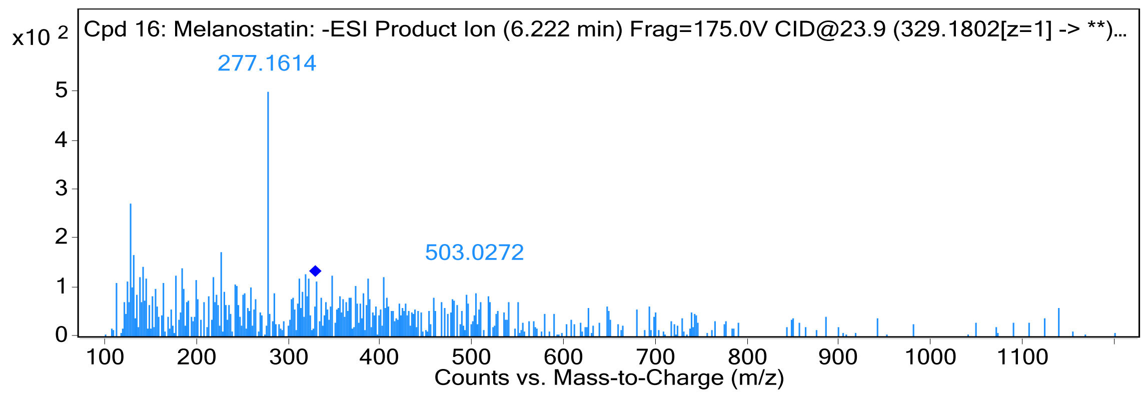

MS/MS Spectrum Peak List

m/z	Abund
126.9072	273.78
130.092	168
141.0951	143.01
176.1061	125.1
183.1396	142.52
217.1095	123.77
226.1144	172.97
277.1614	502.37
317.1767	127.68
347.1651	126.92

Compound Structure

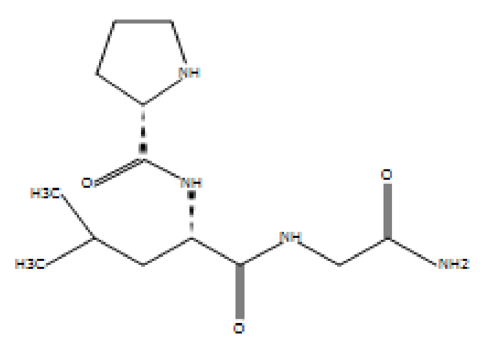

Compound Label	Name	*m/z*	RT	Algorithm	Mass
Cpd 17: Isopentenyl adenosine	**Isopentenyl adenosine**	394.175	6.26	Auto MS/MS	335.1615

MS Spectrum

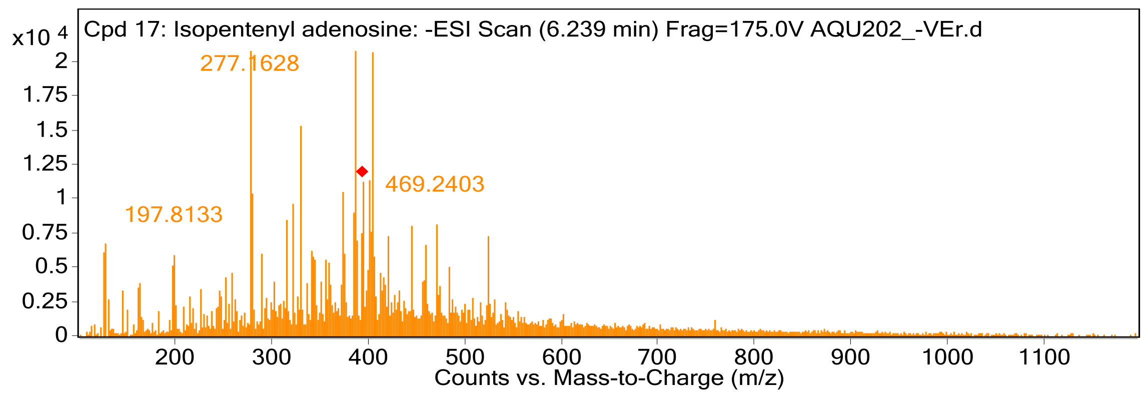

MS Zoomed Spectrum

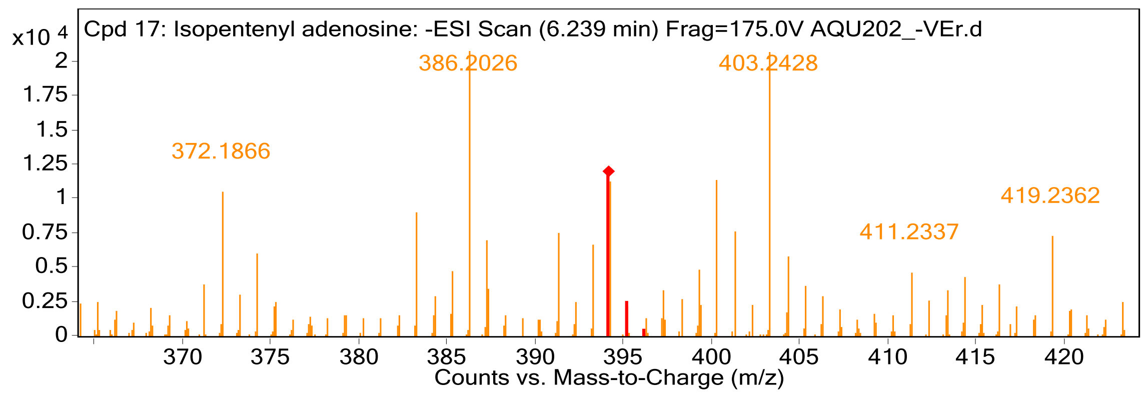

MS Spectrum Peak List

m/z	*Calc m/z*	Diff(ppm)	z	Abund	Formula	Ion
277.1628			1	62718.09		
328.1955				15345.22		
329.1811				11286.37		
372.1866			1	10572.61		
386.2026			1	24198.57		
394.175	394.1732	-4.52		11333.35	C15 H21 N5 O4	(M+CH3COO)-
395.1835	395.1761	-18.73	1	2232.94	C15 H21 N5 O4	(M+CH3COO)-
396.1746	396.1783	9.51	1	1365.07	C15 H21 N5 O4	(M+CH3COO)-
400.2179				11426.17		
403.2428			1	20737.42		

MSMS Spectrum

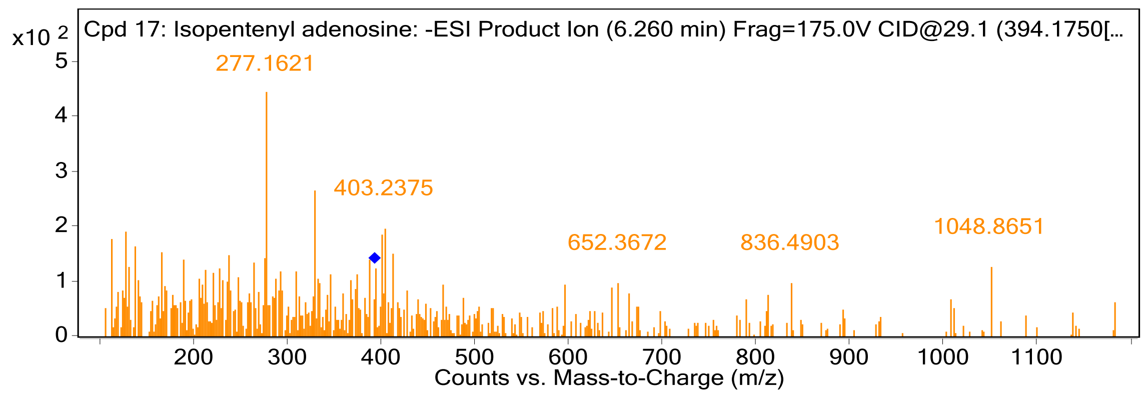

MS/MS Spectrum Peak List

m/z	z	Abund
112.0437		179.53
126.9088		192.88
137.0744		164.98
164.8408		154.5
237.0998		149.17
277.1621		446.75
329.18		266.08
400.2232	1	187.94
403.2375		197.43
411.2348		152.72

Compound Structure

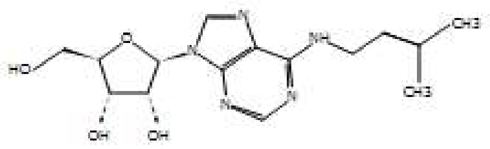

Compound Label	Name	*m/z*	RT	Algorithm	Mass
Cpd 18: 2-Deoxystreptidine	**2-Deoxystreptidine**	291.1417	6.406	Auto MS/MS	246.144

MS Spectrum

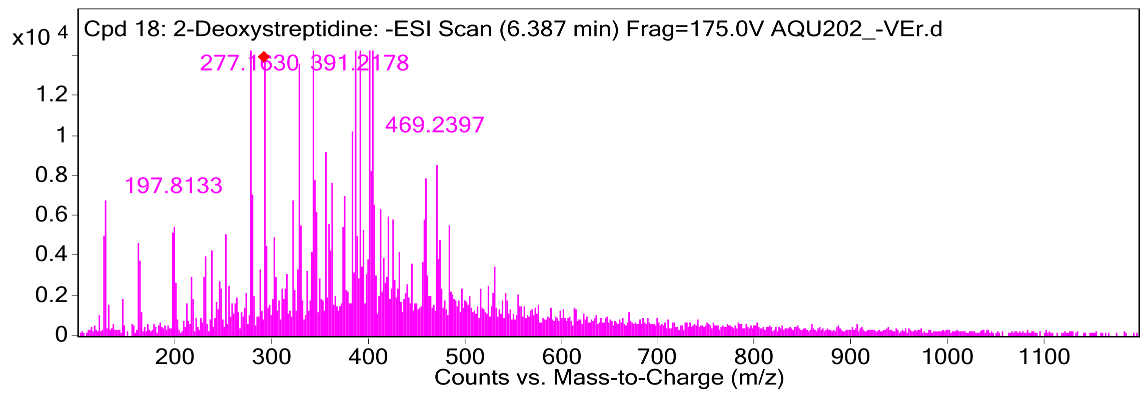

MS Zoomed Spectrum

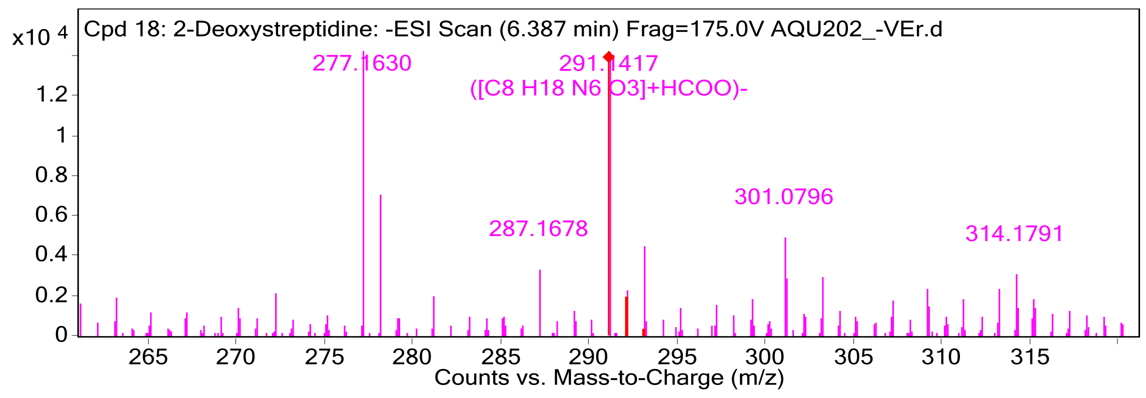

MS Spectrum Peak List

m/z	*Calc m/z*	Diff(ppm)	z	Abund	Formula	Ion
277.163			1	44833.2		
291.1417	291.1422	1.93		14217.92	C8 H18 N6 O3	(M+HCOO)-
292.1452	292.1445	-2.21	1	2359.65	C8 H18 N6 O3	(M+HCOO)-
293.1543	293.1466	-26.24	1	786.21	C8 H18 N6 O3	(M+HCOO)-
328.1955			1	13628.1		
342.2119				15468.86		
386.2019			1	18365.31		
391.2178			1	18773.53		

400.2178			1	15027.2		
403.2428			1	17588.75		

MSMS Spectrum

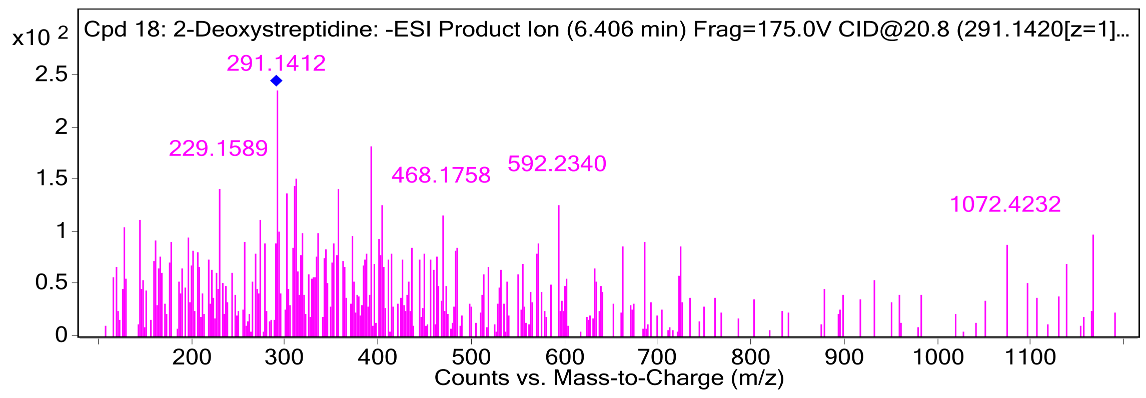

MS/MS Spectrum Peak List

m/z	z	Abund
229.1108		138
229.1589		142.04
291.1412		236.02
301.0778		137.94
310.1246		144.92
311.1489		152.2
356.1374		142.84
391.2177		183.24
403.2414	1	127.09
592.234		127.34

Compound Structure

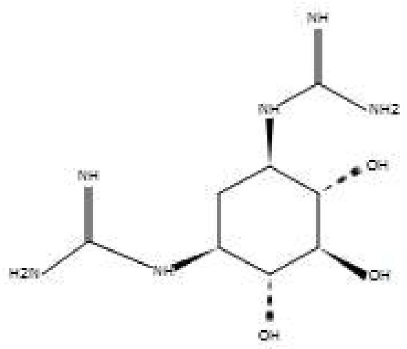

Compound Label	Name	*m/z*	RT	Algorithm	Mass
Cpd 19: Spirasine I	**Spirasine I**	400.2178	6.494	Auto MS/MS	355.2193

MS Spectrum

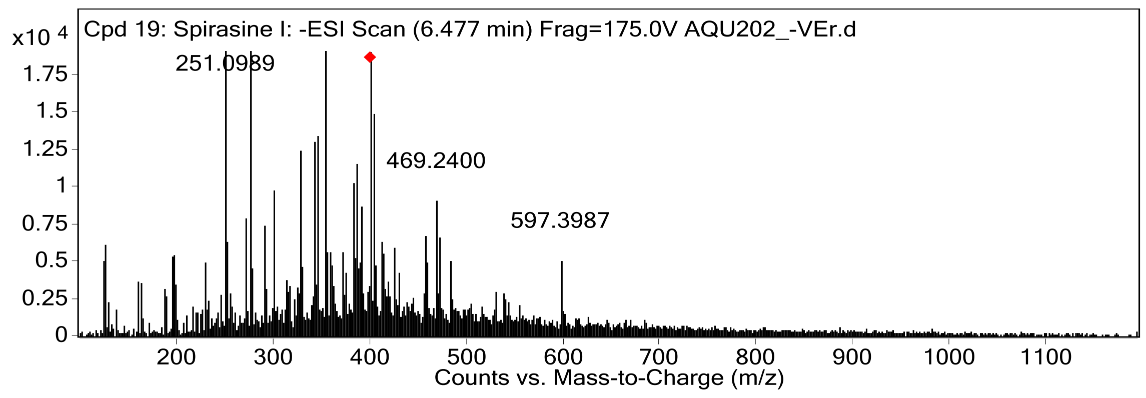

MS Zoomed Spectrum

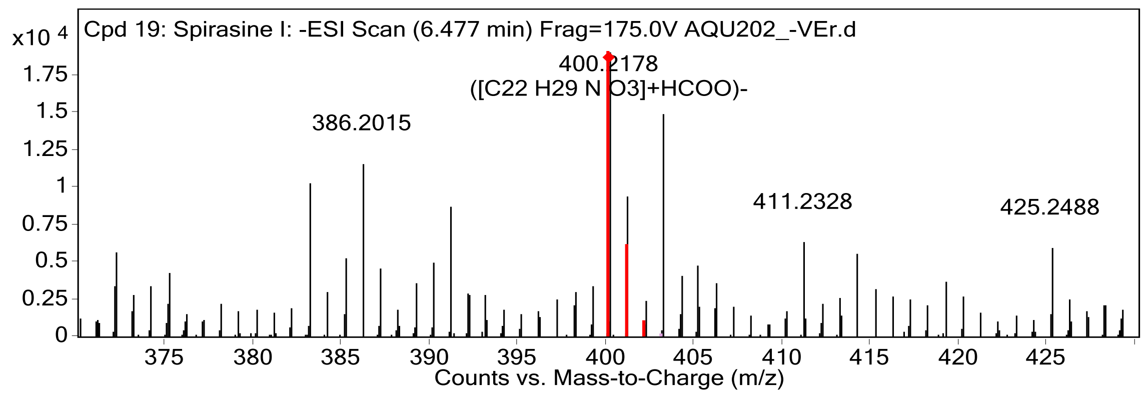

MS Spectrum Peak List

m/z	*Calc m/z*	Diff(ppm)	z	Abund	Formula	Ion
251.0989			1	54623.97		
277.1625			1	23333.4		
328.1952			1	12464.09		
342.2111				13031.54		
345.211			1	13421.52		
354.2113			1	24103.36		
400.2178	400.2129	-12.17	1	19064.14	C22 H29 N O3	(M+HCOO)-
401.2215	401.2163	-13.05	1	9453.33	C22 H29 N O3	(M+HCOO)-
402.2186	402.219	0.98	1	2410.91	C22 H29 N O3	(M+HCOO)-
403.2418			1	14978.85		

MSMS Spectrum

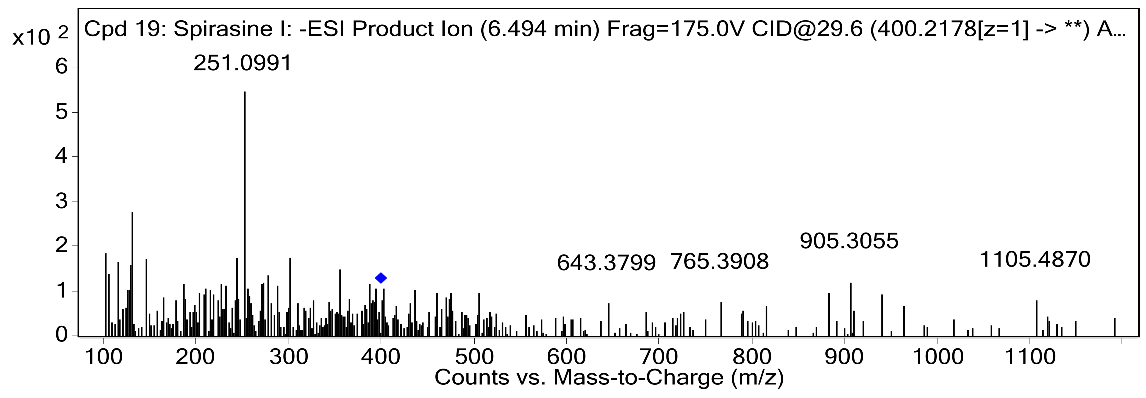

MS/MS Spectrum Peak List

m/z	Abund
102.0597	186.36
104.9897	139.74
116.0747	166.58
128.039	162.08
130.0918	277.77
146.0479	172.79
243.174	177.07
251.0991	549.47

301.1813	177.32
354.2134	151.06

Compound Structure

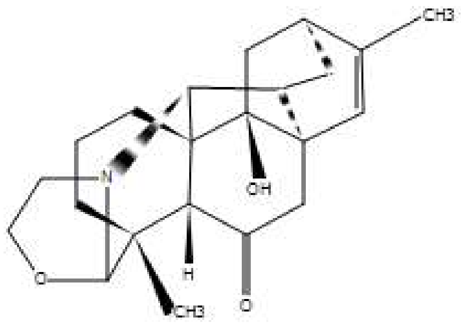

Compound Label	Name	*m/z*	RT	Algorithm	Mass
Cpd 20: Spirasine I	**Spirasine I**	354.2114	6.541	Auto MS/MS	355.219

MS Spectrum

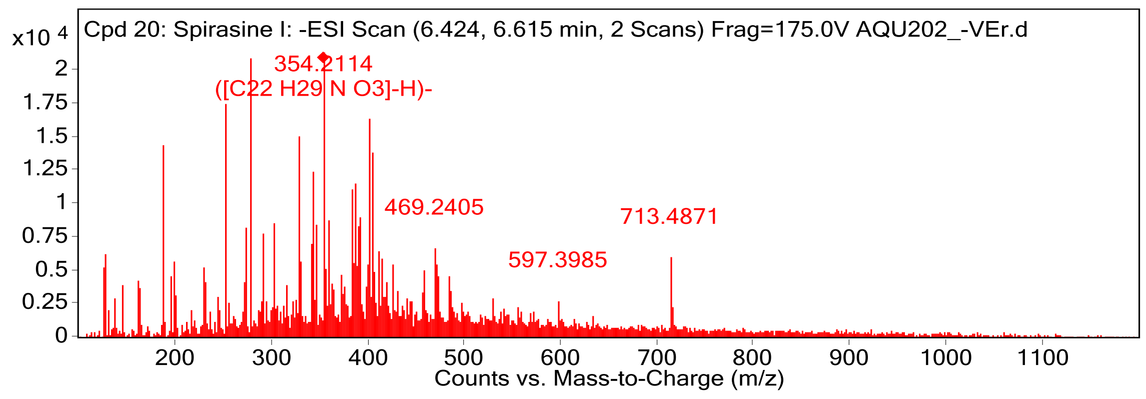

MS Zoomed Spectrum

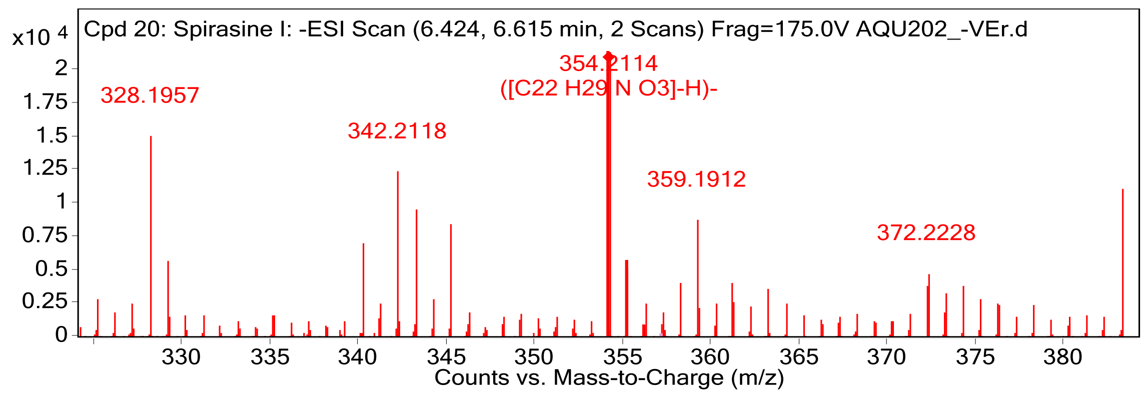

MS Spectrum Peak List

m/z	*Calc m/z*	Diff(ppm)	z	Abund	Formula	Ion
187.1025				14414.73		
251.099			1	17504.13		
277.1628			1	20864.15		
328.1957				15081.76		
342.2118				12402.99		
354.2114	354.2075	-11.22	1	21349.21	C22 H29 N O3	(M-H)-
355.2141	355.2108	-9.36	1	5223.7	C22 H29 N O3	(M-H)-
356.223	356.2137	-26.14	1	2516.64	C22 H29 N O3	(M-H)-
400.2178				16447.95		
403.2421			1	13823.96		

MSMS Spectrum

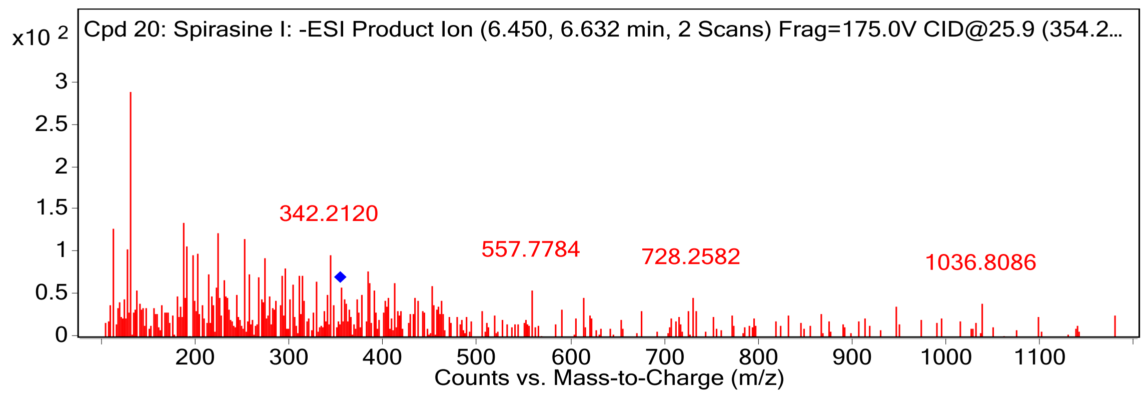

MS/MS Spectrum Peak List

m/z	z	Abund
112.0436		128.16
127.0566		104.25
130.0913		289.83
187.1037		134.92
189.0986		107.17
197.1364		96.47
201.1321		97.94
223.1143	1	122.3
251.0962		115.5
342.212	1	97.78

Compound Structure

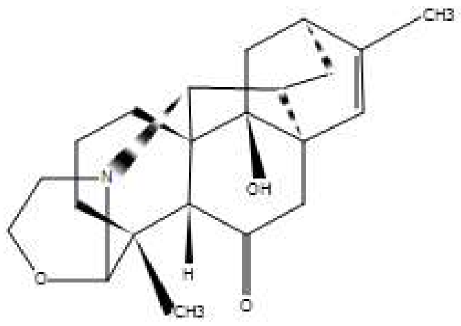

Compound Label	*m/z*	RT	Algorithm
Compound 21	187.1025	6.547	Auto MS/MS

MS Spectrum

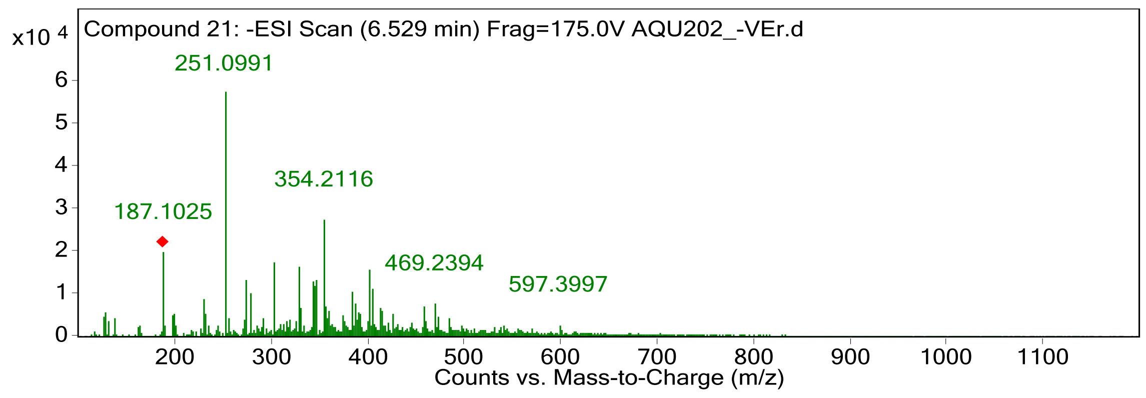

MS Zoomed Spectrum

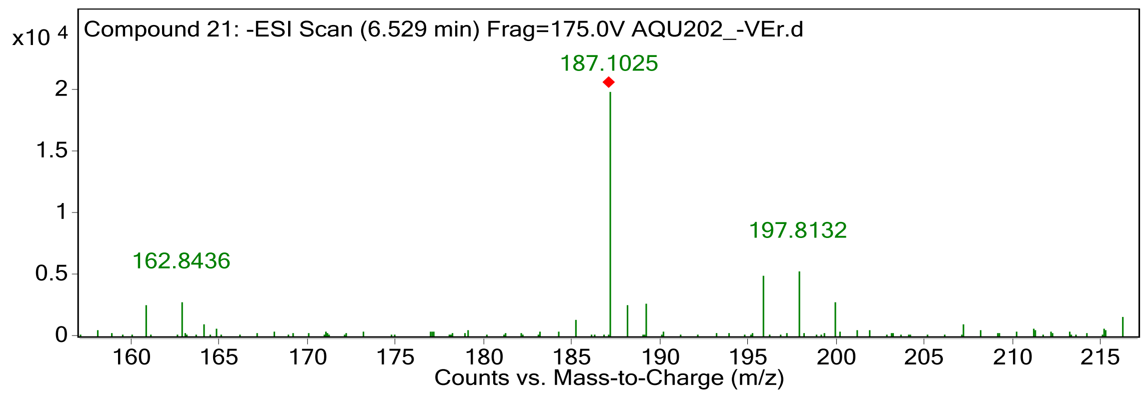

MS Spectrum Peak List

m/z	z	Abund
187.1025		19939.88
188.1067	1	2570.85
189.0974		2744.74
190.0985	1	493.49
251.0991	1	57826.27
301.1838	1	17787.81
328.1953	1	16557.71
345.2105	1	13543.02
354.2116	1	27612.91
400.2184	1	15823.8

MSMS Spectrum

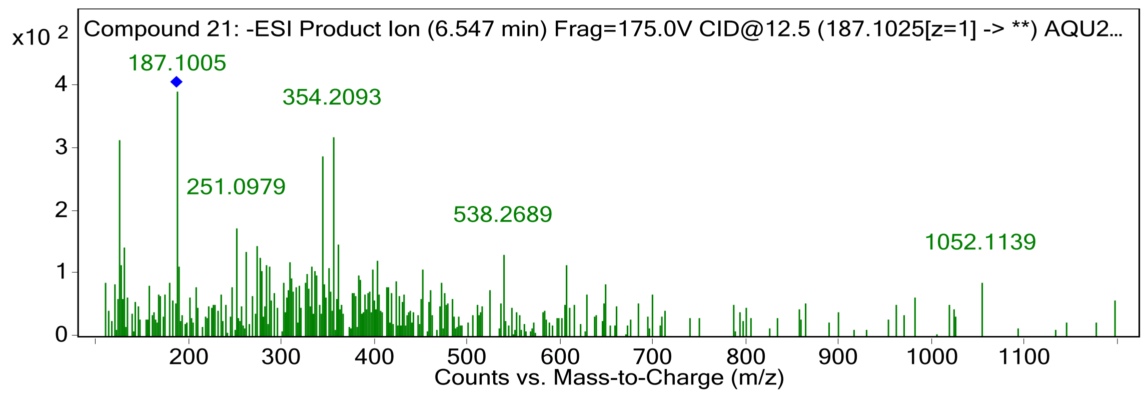

MS/MS Spectrum Peak List

m/z	z	Abund
125.1008	1	313.18
130.0926		142.41
187.1005		392.54
251.0979		174.61
260.108		135.3
273.1637		146.05
343.1956		287.51
354.2093	1	318.08
355.2055	1	159.72
359.1857		147.03

Compound Label	Name	*m/z*	RT	Algorithm	Mass
Cpd 22: Melanostatin	**Melanostatin**	329.18	6.785	Auto MS/MS	284.182

MS Spectrum

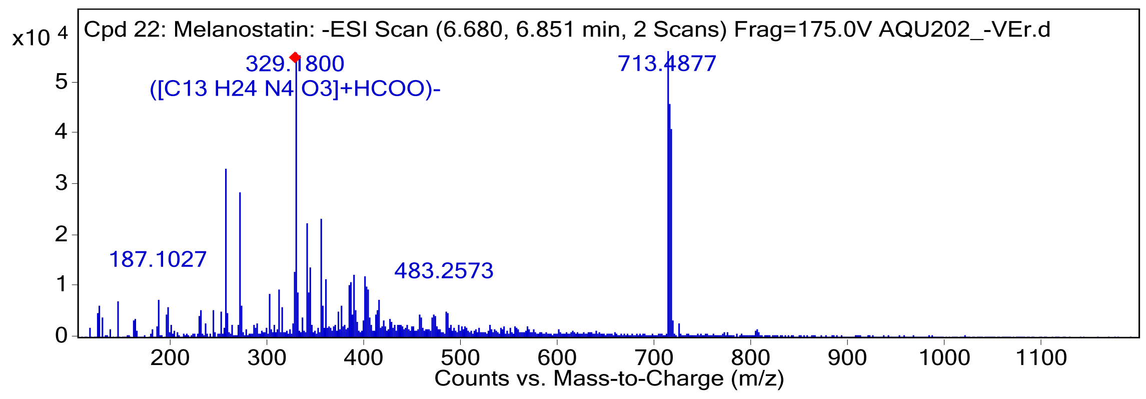

MS Zoomed Spectrum

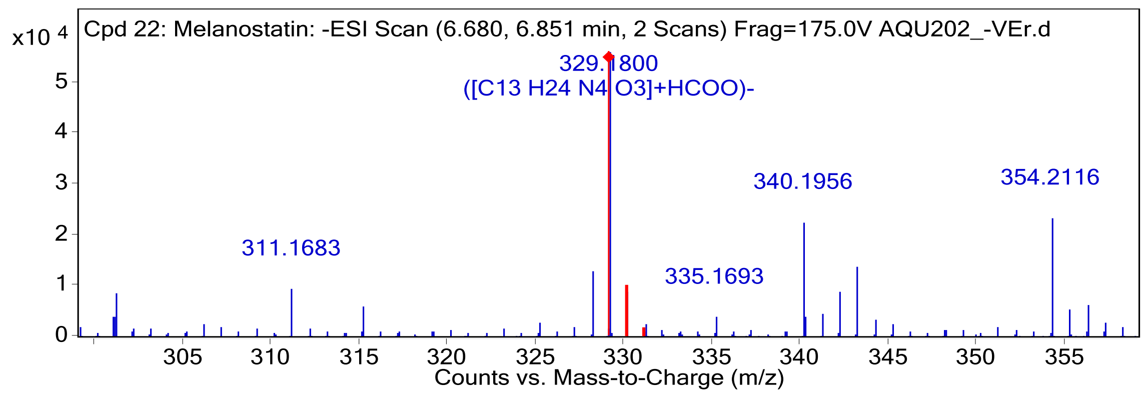

MS Spectrum Peak List

m/z	*Calc m/z*	Diff(ppm)	z	Abund	Formula	Ion
257.1567			1	33157.14		
271.173			1	28548.1		
329.18	329.183	9.24	1	55946.6	C13 H24 N4 O3	(M+HCOO)-
330.1821	330.1859	11.56	1	9041	C13 H24 N4 O3	(M+HCOO)-
331.1898	331.1881	-5.05	1	2584.53	C13 H24 N4 O3	(M+HCOO)-
332.1985	332.1906	-23.97	1	613.62	C13 H24 N4 O3	(M+HCOO)-
354.2116			1	23417.42		
713.4877			1	118543.01		
714.4907			1	45721.64		

715.4866			1	40890.16		

MSMS Spectrum

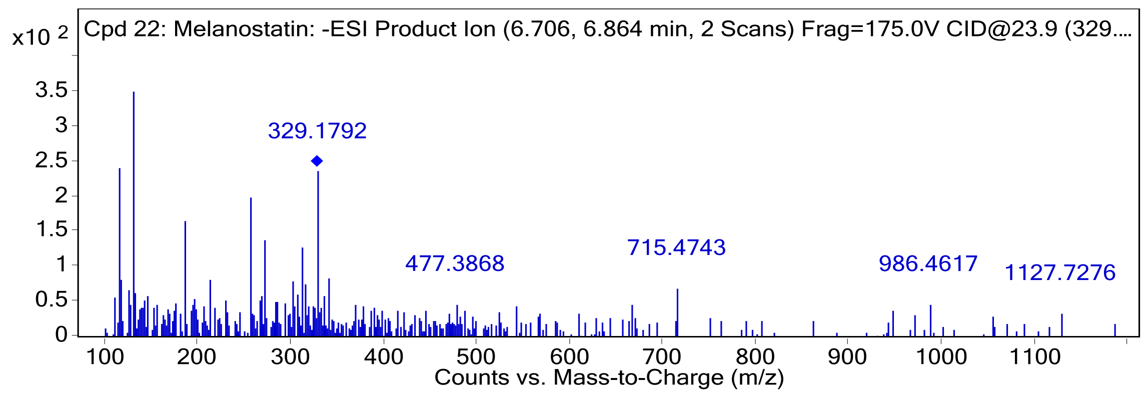

MS/MS Spectrum Peak List

m/z	z	Abund
116.0749		240.34
117.078		82.43
130.0908		350.91
185.1711		166.51
213.0987		82.73
257.1559	1	198.82
271.1722		138.36
311.1643	1	128.48
329.1792	1	236.26
341.1999		83.99

Compound Structure

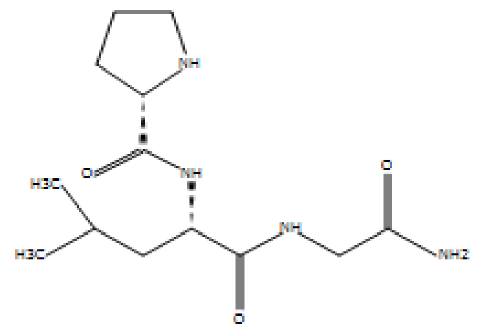

Compound Label	Name	*m/z*	RT	Algorithm	Mass
Cpd 23: β-estradiol	**β-estradiol**	271.1731	6.948	Auto MS/MS	272.1802

MS Spectrum

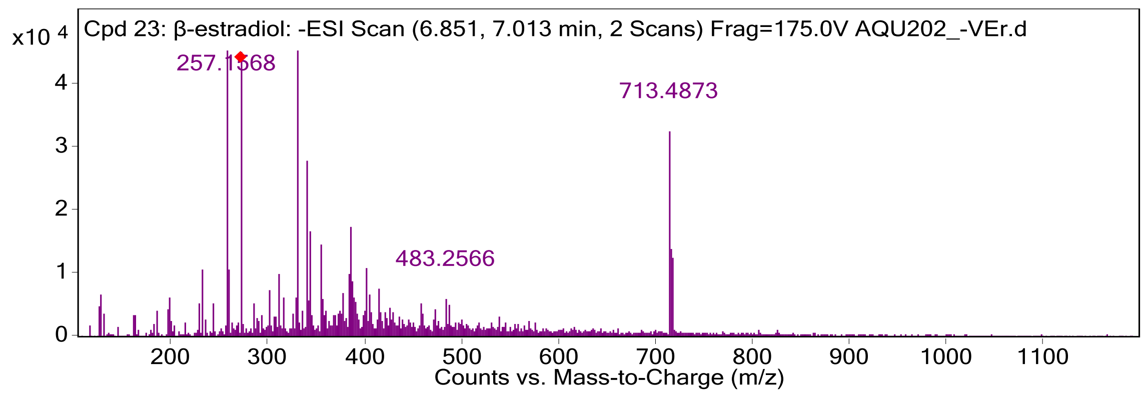

MS Zoomed Spectrum

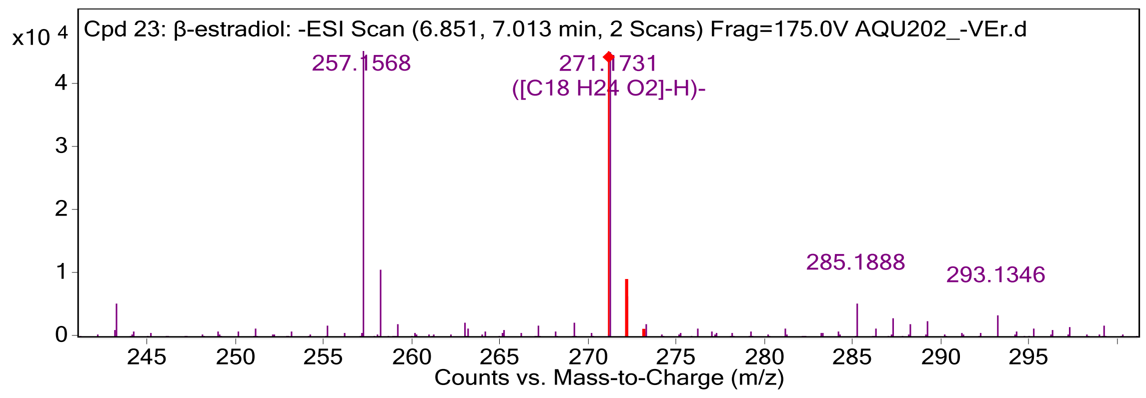

MS Spectrum Peak List

m/z	*Calc m/z*	Diff(ppm)	z	Abund	Formula	Ion
257.1568			1	75306.06		
271.1731	271.1704	-10.04	1	45214.33	C18 H24 O2	(M-H)-
272.176	272.1738	-8.26	1	6477.95	C18 H24 O2	(M-H)-
273.1782	273.1767	-5.51	1	2203.07	C18 H24 O2	(M-H)-
329.1799			1	54748.17		
340.1958			1	27884.24		
343.1964			1	16751.25		
354.2116			1	14602.89		
385.2312			1	17432.42		
713.4873			1	32640.42		

MSMS Spectrum

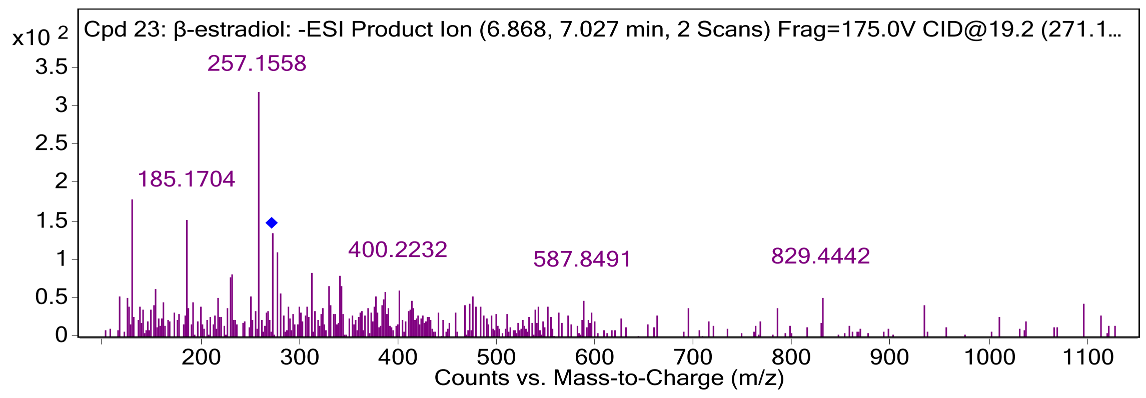

MS/MS Spectrum Peak List

m/z	z	Abund
130.0909		180.65
185.1704	1	153.05
229.1564		78.13
231.1287		81.72
257.1558	1	320.13
258.1601	1	91.56
271.1713		136.77

276.768		111.24
311.168		83.43
340.1955		80.98

Compound Structure

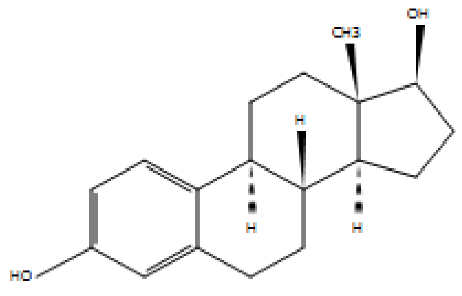

Compound Label	Name	*m/z*	RT	Algorithm	Mass
Cpd 24: Eseramine	**Eseramine**	363.1649	7.219	Auto MS/MS	318.1675

MS Spectrum

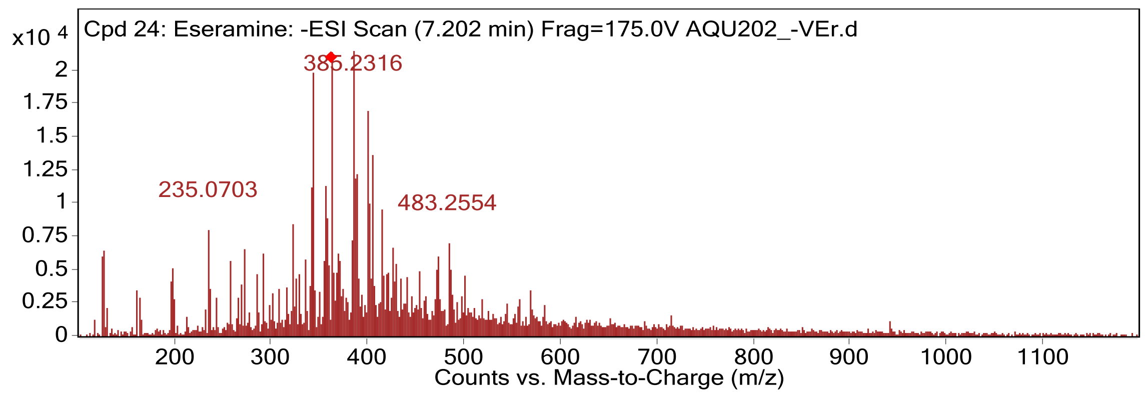

MS Zoomed Spectrum

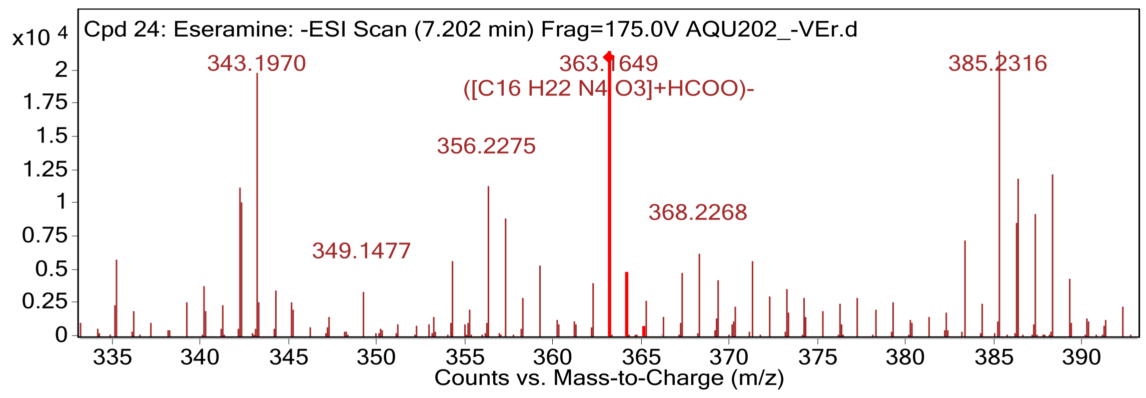

MS Spectrum Peak List

m/z	*Calc m/z*	Diff(ppm)	z	Abund	Formula	Ion
343.197			1	19891.64		
356.2275				11308.38		
363.1649	363.1674	6.83	1	21386.92	C16 H22 N4 O3	(M+HCOO)-
364.1693	364.1703	2.91	1	4903	C16 H22 N4 O3	(M+HCOO)-
365.1758	365.1727	-8.52	1	2733.52	C16 H22 N4 O3	(M+HCOO)-
385.2316			1	25710.28		
386.2367			1	11922.19		
388.1973			1	12216.43		
400.2177				16950.53		
405.2335			1	13672.29		

MSMS Spectrum

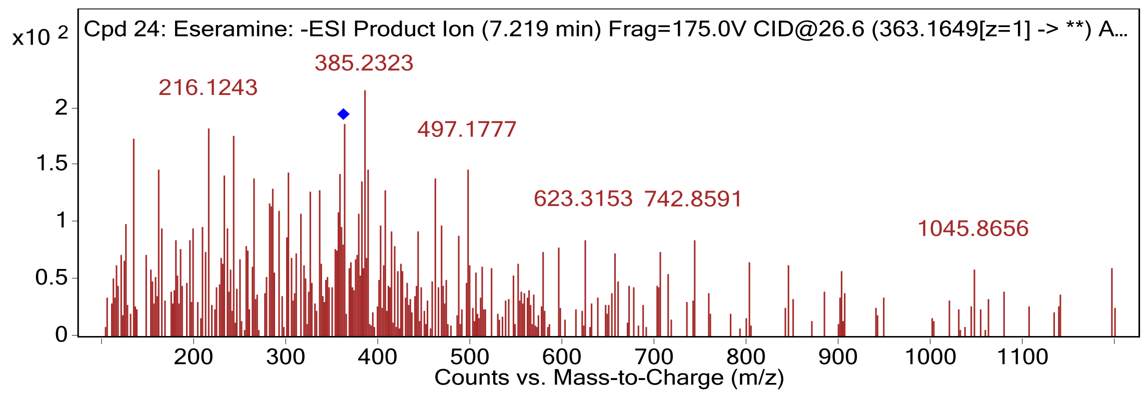

MS/MS Spectrum Peak List

m/z	z	Abund
133.4693		173.1
160.8493		146.88
216.1243		183.08
243.178		176.03
301.182		144.25
357.2236		143.32
363.1631		187
385.2323	1	215.81
388.1991		146.51
497.1777		146.38

Compound Structure

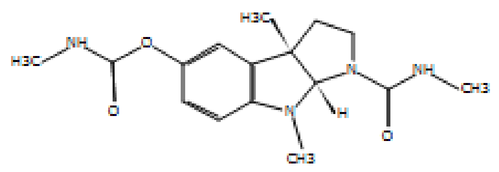

Compound Label	Name	*m/z*	RT	Algorithm	Mass
Cpd 25: beta-D-Mannosylphosphodecapre	**beta-D-Mannosylphosphodec**	939.6576	7.318	Auto MS/MS	940.6625

MS Spectrum

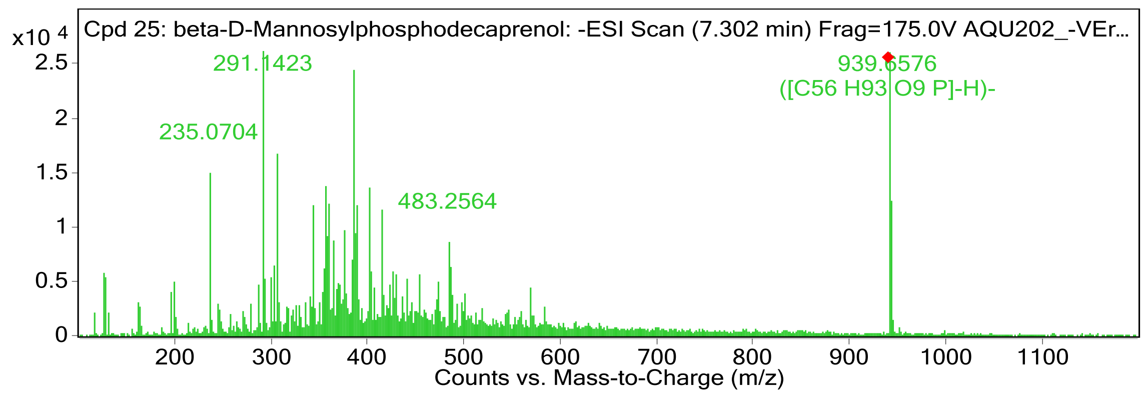

MS Zoomed Spectrum

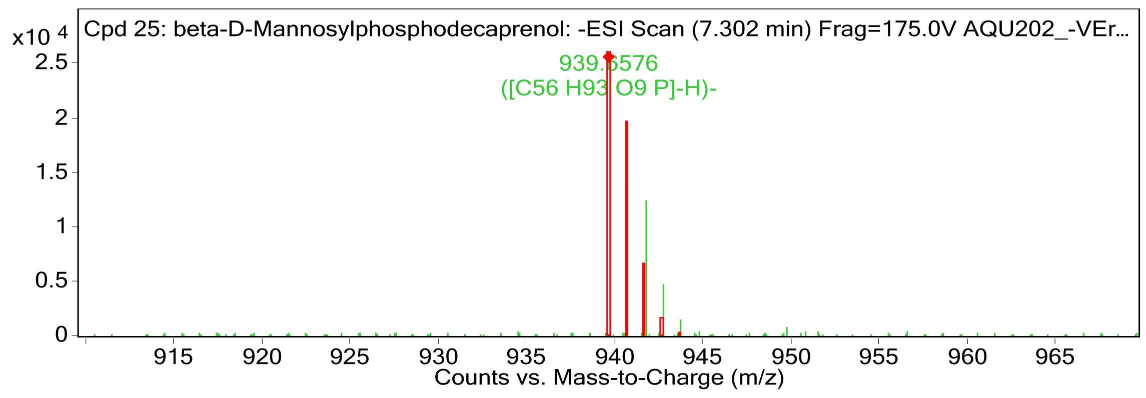

MS Spectrum Peak List

m/z	*Calc m/z*	Diff(ppm)	z	Abund	Formula	Ion
235.0704				15113.28		
291.1423			1	31060.27		
305.1585			1	16865.9		
385.232			1	24568.92		
939.6576	939.6484	-9.72	1	26136.76	C56 H93 O9 P	(M-H)-
940.661	940.6519	-9.76	1	14163.56	C56 H93 O9 P	(M-H)-
941.6577	941.655	-2.79	1	12518.15	C56 H93 O9 P	(M-H)-
942.6587	942.6581	-0.61	1	4851.64	C56 H93 O9 P	(M-H)-
943.6615	943.661	-0.48	1	1612.45	C56 H93 O9 P	(M-H)-
944.6612	944.6639	2.92	1	550.08	C56 H93 O9 P	(M-H)-

MSMS Spectrum

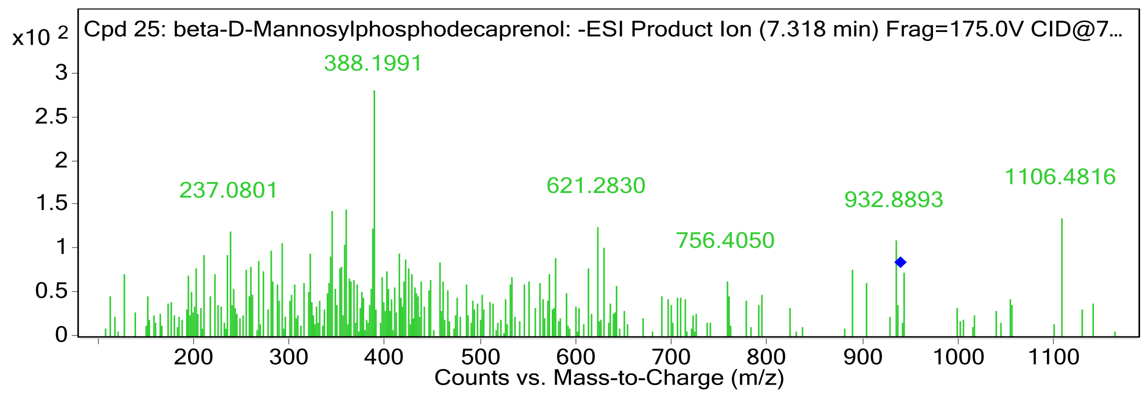

MS/MS Spectrum Peak List

m/z	z	Abund
237.0801		121.01
291.1387		107.04
343.1925	1	143.33
357.2293		104.79
359.2188		146.06
386.2339	1	123.61
388.1991		280.49
621.283		126.33
932.8893		110.25
1106.4816		136.37

Compound Structure

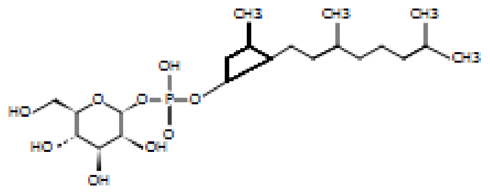

Compound Label	Name	*m/z*	RT	Algorithm	Mass
Cpd 26: 2-Deoxystreptidine	**2-Deoxystreptidine**	291.1422	7.361	Auto MS/MS	246.1441

MS Spectrum

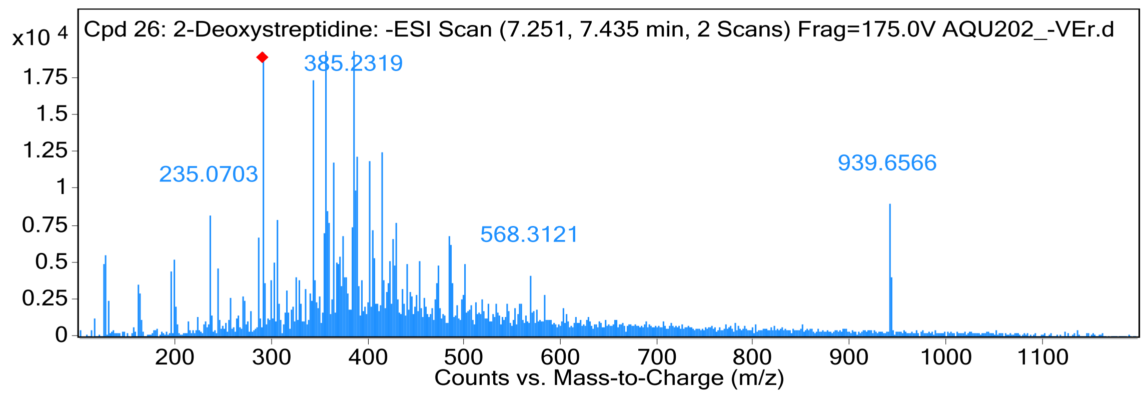

MS Zoomed Spectrum

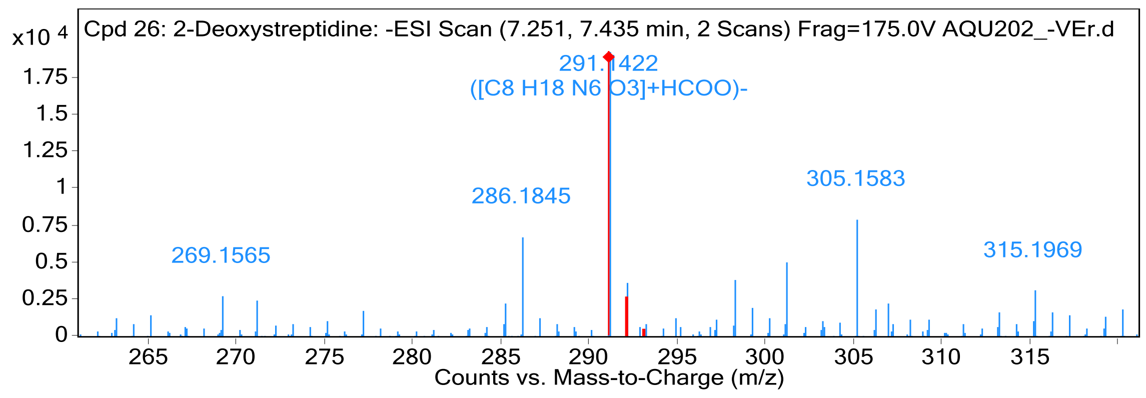

MS Spectrum Peak List

m/z	*Calc m/z*	Diff(ppm)	z	Abund	Formula	Ion
291.1422	291.1422	0.06	1	19297.16	C8 H18 N6 O3	(M+HCOO)-
292.1445	292.1445	0.1	1	3709.44	C8 H18 N6 O3	(M+HCOO)-
293.1485	293.1466	-6.47	1	888.4	C8 H18 N6 O3	(M+HCOO)-
343.1974				17396.87		
356.2275			1	19774.73		
363.1646			1	11824.68		
385.2319			1	23293.57		
387.2463			1	12259.2		
400.218				11977.55		
414.2349			1	12508.82		

MSMS Spectrum

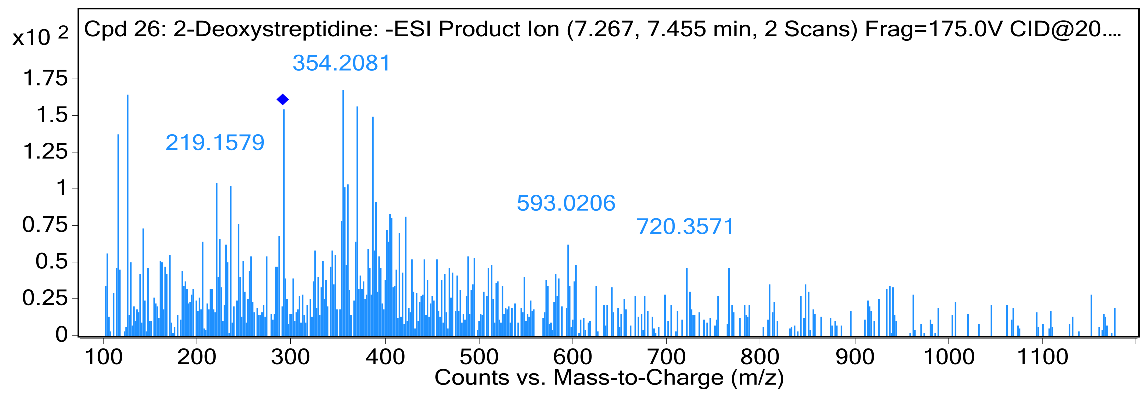

MS/MS Spectrum Peak List

m/z	z	Abund
115.0899		138.55
124.9959		165.71
219.1579		104.93
235.0698		103.45
291.1424	1	154.99
354.2081		167.9
356.2281		102.62
359.23		103.86
369.236		157.57
385.23	1	150.73

Compound Structure

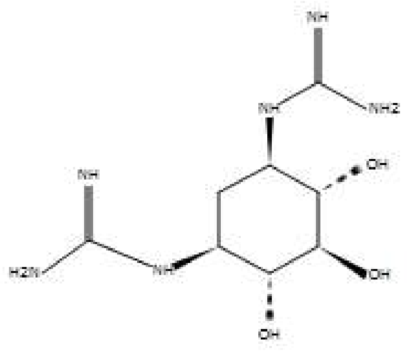

Compound Label	Name	*m/z*	RT	Algorithm	Mass
Cpd 27: Oxybutynin	**Oxybutynin**	356.2276	7.383	Auto MS/MS	357.2312

MS Spectrum

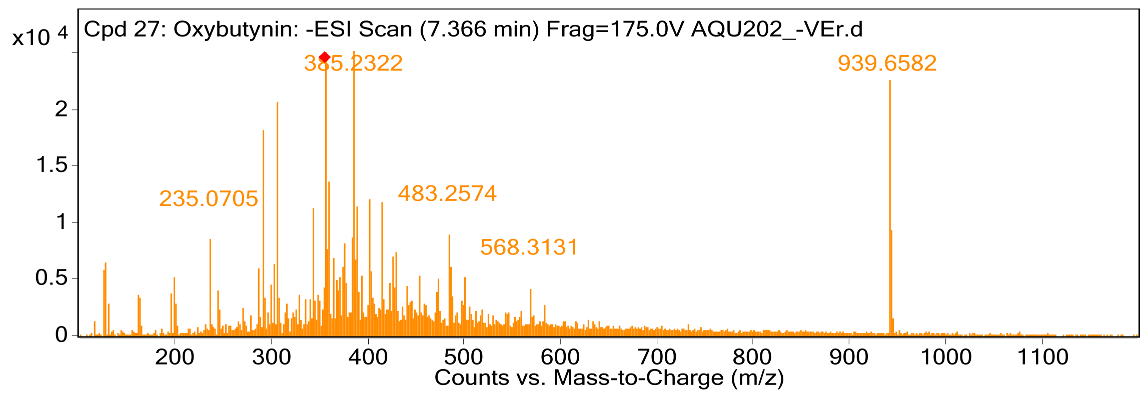

MS Zoomed Spectrum

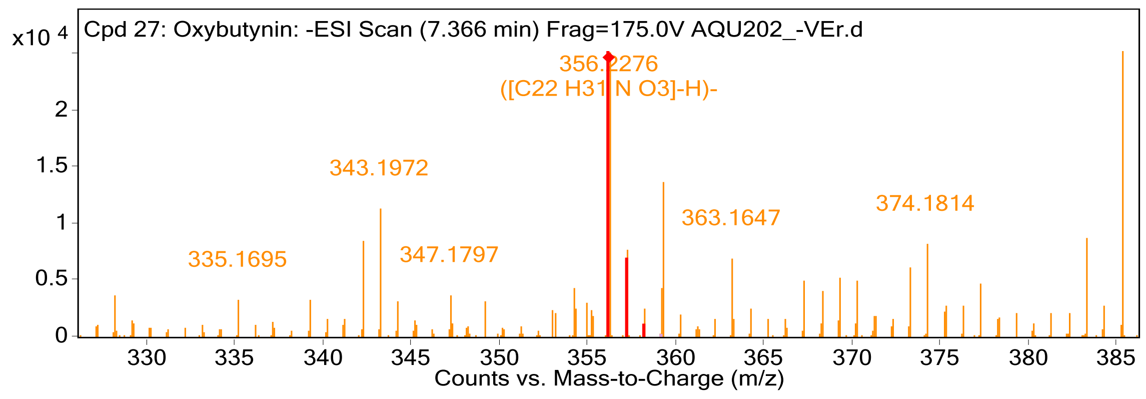

MS Spectrum Peak List

m/z	*Calc m/z*	Diff(ppm)	z	Abund	Formula	Ion
291.1426			1	18249.29		
305.1587			1	20670.76		
356.2276	356.2231	-12.63	1	25136.74	C22 H31 N O3	(M-H)-
357.2195	357.2264	19.27	1	7802.87	C22 H31 N O3	(M-H)-
358.2172	358.2293	33.78	1	2551.66	C22 H31 N O3	(M-H)-
359.2268				13670.99		
385.2322			1	25247.6		
400.2185				12201.89		
414.236			1	11903.21		

939.6582			1	22698.99		

MSMS Spectrum

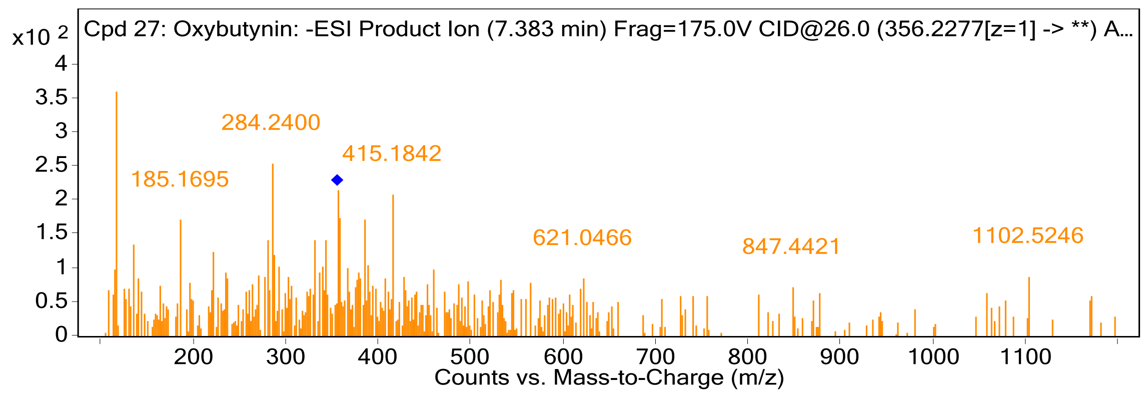

MS/MS Spectrum Peak List

m/z	z	Abund
116.0746		361.24
185.1695		171.88
279.1384		141.47
284.24	1	255.36
331.1888		141.9
342.2456		141.76
356.2255		216.7
357.2149		174.97
385.2256		171.76
415.1842		209.69

Compound Structure

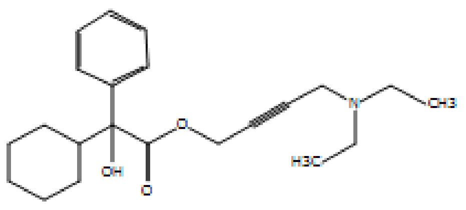

Compound Label	Name	*m/z*	RT	Algorithm	Mass
Cpd 28: Spirasine I	**Spirasine I**	354.2114	7.59	Auto MS/MS	355.2182

MS Spectrum

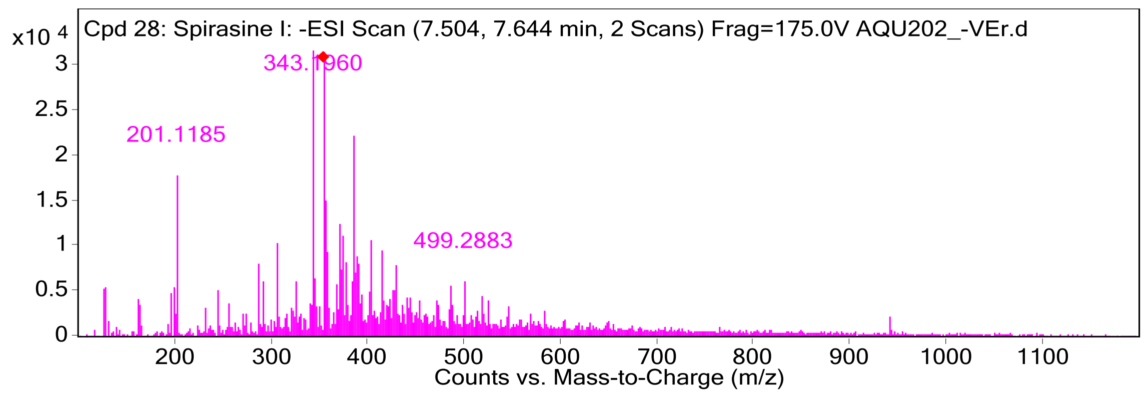

MS Zoomed Spectrum

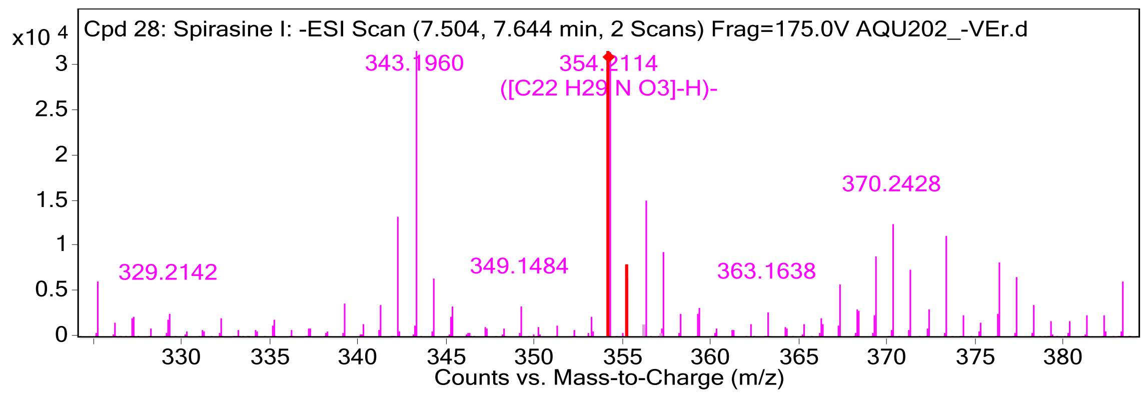

MS Spectrum Peak List

m/z	*Calc m/z*	Diff(ppm)	z	Abund	Formula	Ion
201.1185				17913.94		
342.2113			1	13316.91		
343.196				33614.3		
354.2114	354.2075	-11.23	1	31522.24	C22 H29 N O3	(M-H)-
355.2114	355.2108	-1.74	1	6574.2	C22 H29 N O3	(M-H)-
356.2272				15077.38		
370.2428				12445.48		
373.2068				11279.02		
385.2321			1	22229.96		
403.2179				10727.67		

MSMS Spectrum

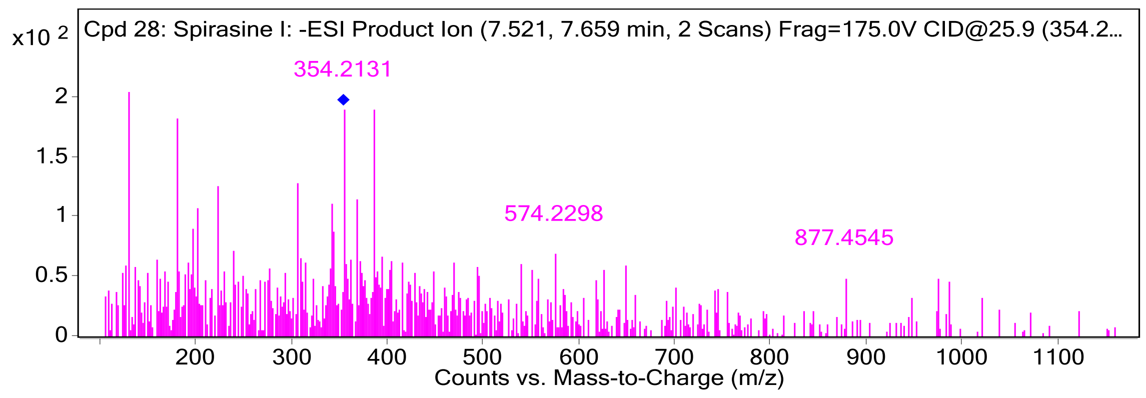

MS/MS Spectrum Peak List

m/z	z	Abund
130.0913		205.34
180.1076		183.08
201.1182		107.59
223.1157		126.04
305.1579		129.01
342.2106		111.22
354.2131		190.14

367.2212		115.63
385.2298	1	189.71
386.2334	1	99.56

Compound Structure

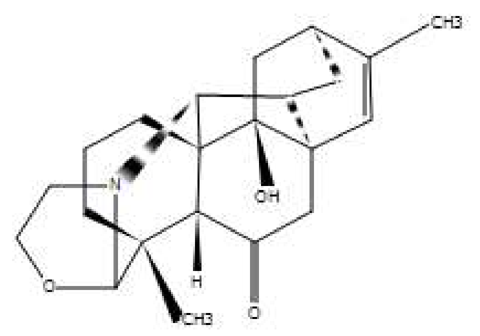

Compound Label	Name	*m/z*	RT	Algorithm	Mass
Cpd 29: Spirasine I	**Spirasine I**	354.212	7.832	Auto MS/MS	355.2191

MS Spectrum

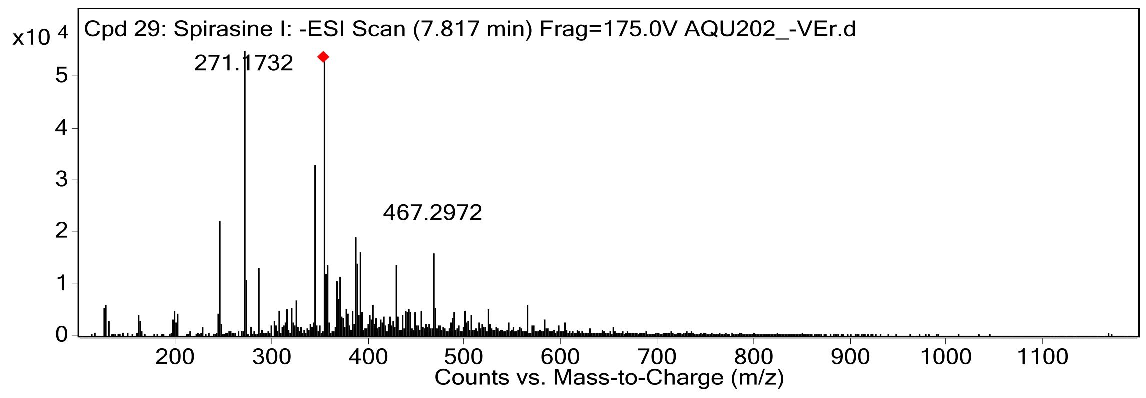

MS Zoomed Spectrum

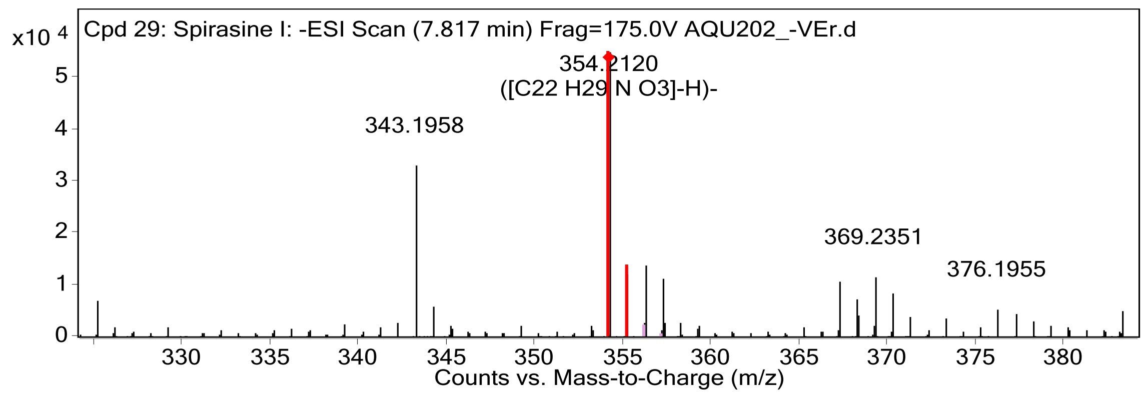

MS Spectrum Peak List

m/z	*Calc m/z*	Diff(ppm)	z	Abund	Formula	Ion
245.1455				22503.73		
271.1732			1	79379.59		
343.1958			1	33052.96		
354.212	354.2075	-12.85	1	54923.33	C22 H29 N O3	(M-H)-
355.2142	355.2108	-9.75	1	12110.33	C22 H29 N O3	(M-H)-
385.2332			1	19160.09		
388.1972			1	14133.41		
390.1889			1	16297.91		
428.25			1	13855.48		
467.2972			1	16107.42		

MSMS Spectrum

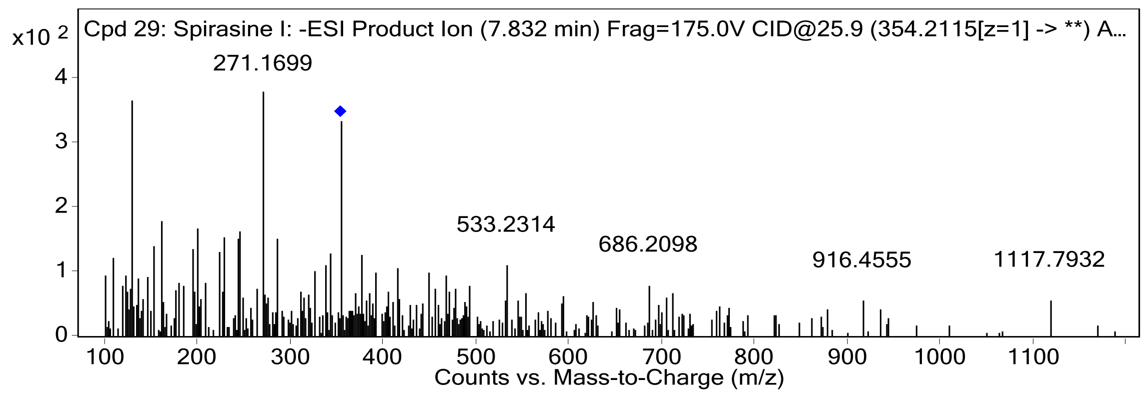

MS/MS Spectrum Peak List

m/z	z	Abund
130.0904		366.25
152.1094		140.87
160.8467		180.93
199.1866		168.17
228.0956		154.07
244.161		152.47
245.144	1	164.89
271.1699	1	381.53
285.1873	1	153.76
354.213	1	334.39

Compound Structure

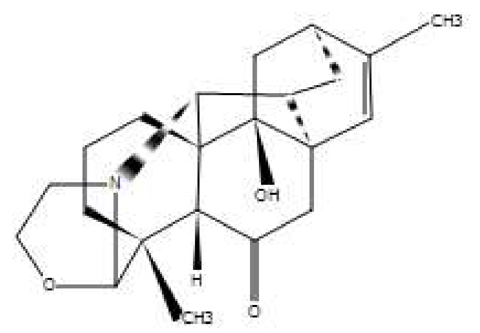

Compound Label	Name	*m/z*	RT	Algorithm	Mass
Cpd 30: β-estradiol	**β-estradiol**	271.1732	7.834	Auto MS/MS	272.1803

MS Spectrum

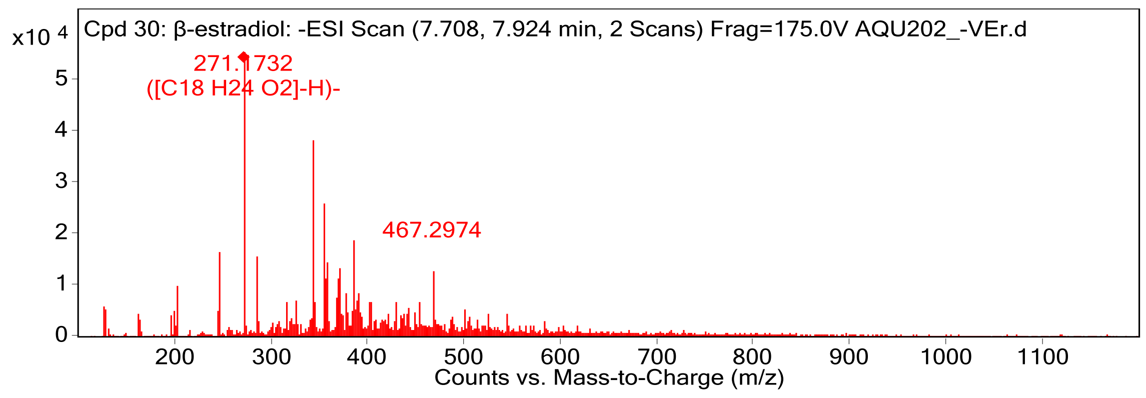

MS Zoomed Spectrum

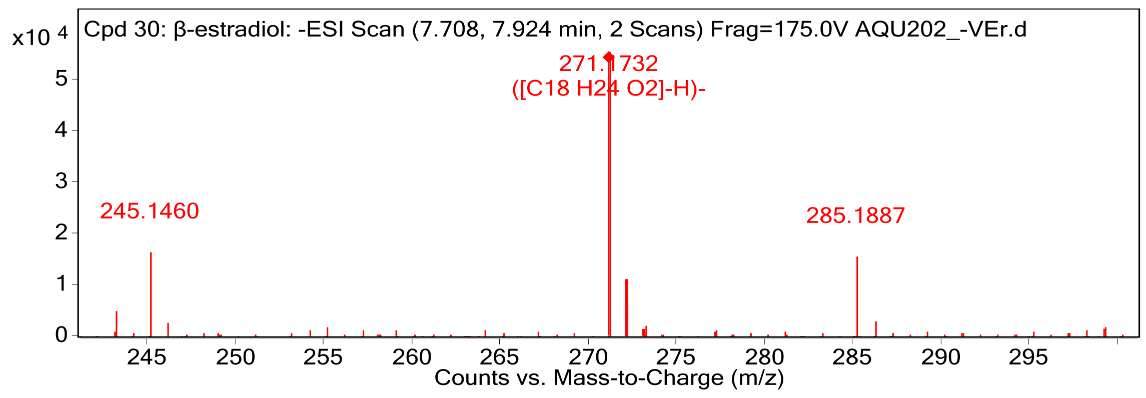

MS Spectrum Peak List

m/z	*Calc m/z*	Diff(ppm)	z	Abund	Formula	Ion
245.146			1	16595.22		
271.1732	271.1704	-10.38	1	55329.53	C18 H24 O2	(M-H)-
272.1763	272.1738	-9.38	1	7564.92	C18 H24 O2	(M-H)-
273.1779	273.1767	-4.52	1	2252.65	C18 H24 O2	(M-H)-
285.1887			1	15720.52		
343.1959			1	38156.67		
354.2117			1	25811.9		
357.2118			1	14519.97		
370.2426			1	13324.05		
385.233			1	18838.56		

MSMS Spectrum

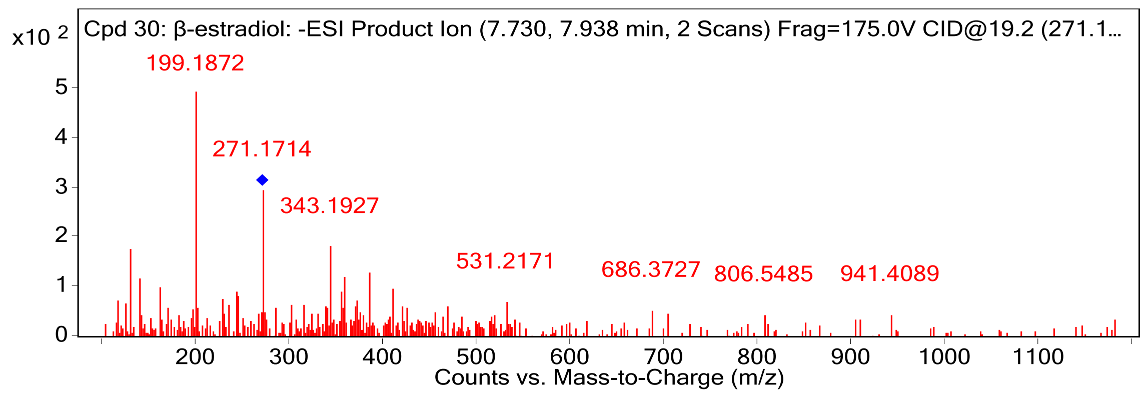

MS/MS Spectrum Peak List

m/z	z	Abund
130.0914		175.96
140.0748		117.63
160.8459		99.77
199.1872	1	494.64
271.1714	1	296.7
343.1927		182.19
354.2131	2	92.52
357.2087	1	120.05
385.2343		129.45
409.1411		97.64

Compound Structure

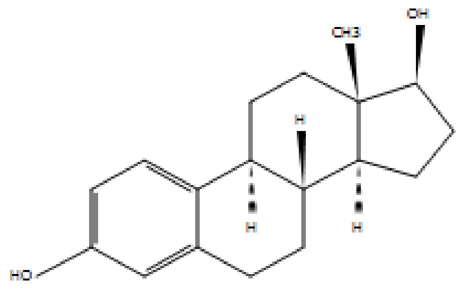

Compound Label	Name	*m/z*	RT	Algorithm	Mass
Cpd 31: 3alpha,7beta,12alpha-Trihydroxy-5alpha-cholan-	**3alpha,7beta,12alpha-Trihydroxy-5alpha-cholan-24-oic Acid**	467.2975	7.864	Auto MS/MS	408.2832

MS Spectrum

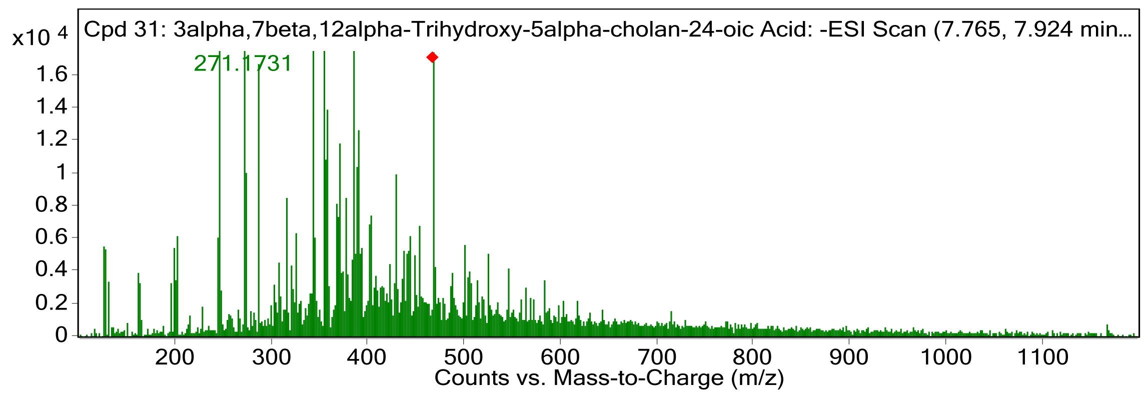

MS Zoomed Spectrum

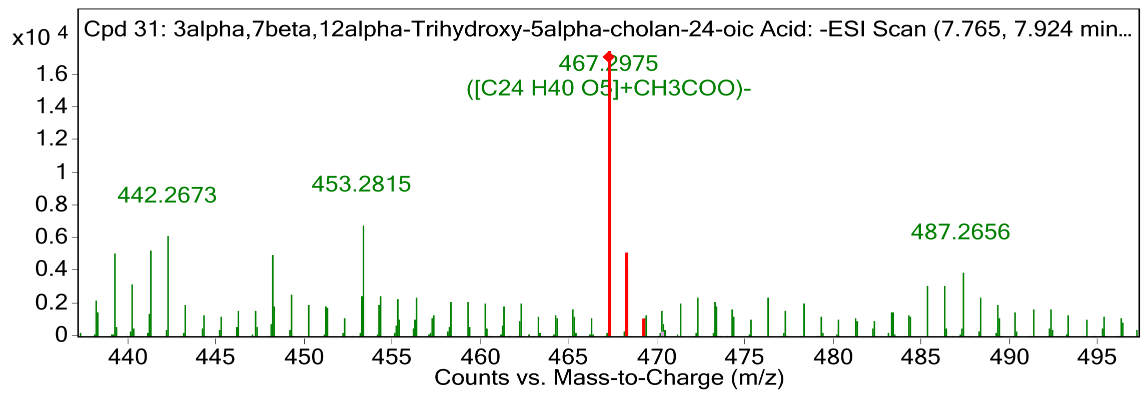

MS Spectrum Peak List

m/z	*Calc m/z*	Diff(ppm)	z	Abund	Formula	Ion
245.146			1	17488.24		
271.1731			1	73165.17		
285.1886			1	16691.47		
343.1957			1	31781.84		
354.2117			1	38622.93		
357.2115			1	13895.89		
385.2329			1	19063.33		

m/z	*Calc m/z*	Diff(ppm)	z	Abund	Formula	Ion
467.2975	467.3014	8.46	1	17415	C24 H40 O5	(M+CH3COO)-
468.2995	468.3048	11.5	1	4314.97	C24 H40 O5	(M+CH3COO)-
469.3001	469.3076	15.84	1	1371.33	C24 H40 O5	(M+CH3COO)-

MSMS Spectrum

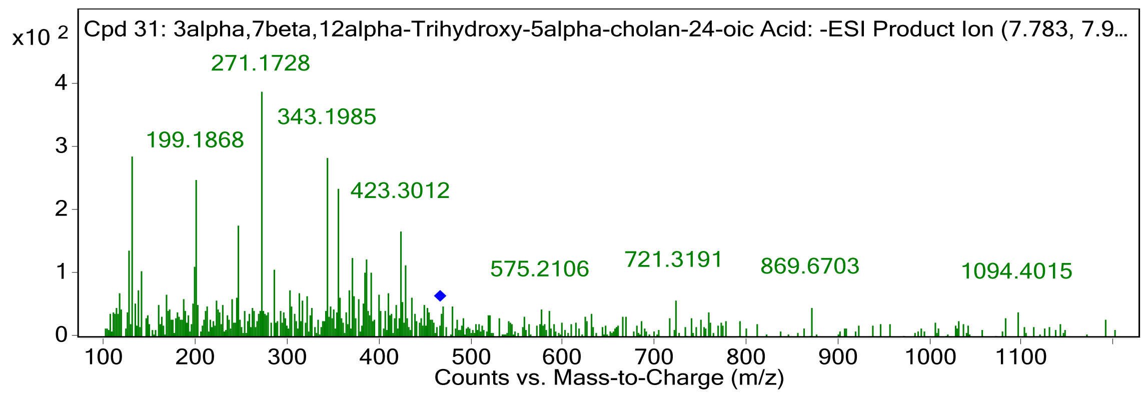

MS/MS Spectrum Peak List

m/z	z	Abund
127.0551		137.23
130.0913		286.2
199.1868		248
245.1467		177.28
271.1728	1	389.29
343.1985		284.82
354.2132		235.73
370.2446		126.11
385.2327		123.13
423.3012		167.55

Compound Structure

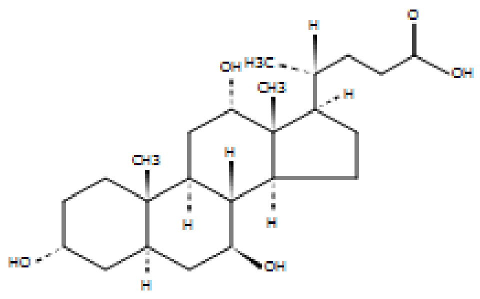

Compound Label	Name	*m/z*	RT	Algorithm	Mass
Cpd 32: β-estradiol	**β-estradiol**	271.1729	8.122	Auto MS/MS	272.1801

MS Spectrum

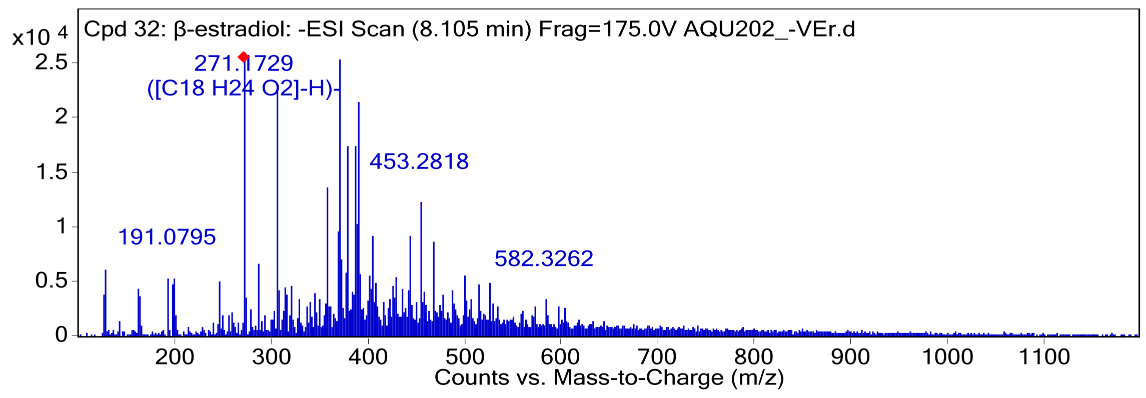

MS Zoomed Spectrum

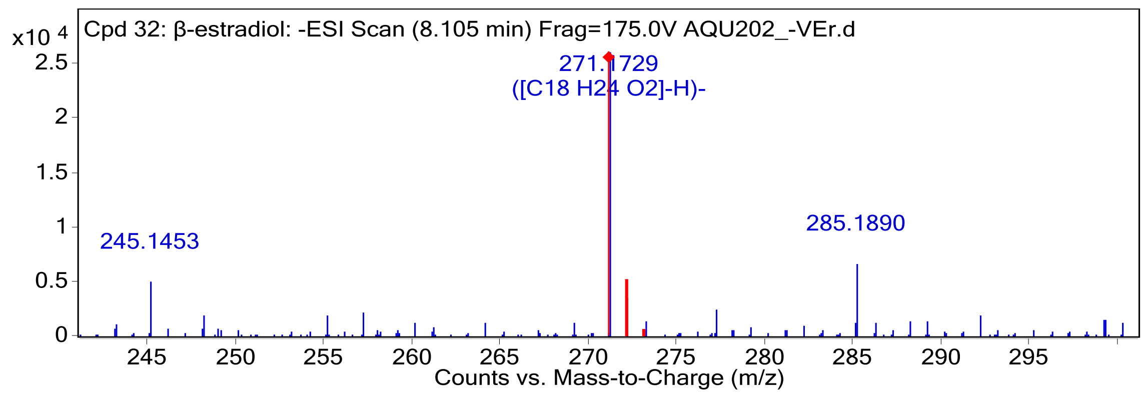

MS Spectrum Peak List

m/z	*Calc m/z*	Diff(ppm)	z	Abund	Formula	Ion
271.1729	271.1704	-9.51	1	26116.05	C18 H24 O2	(M-H)-
272.1764	272.1738	-9.86	1	3571.24	C18 H24 O2	(M-H)-
273.1775	273.1767	-2.95	1	1421.87	C18 H24 O2	(M-H)-
305.1585			1	22506.71		
357.2126			1	13763.44		
369.2364				14588.43		
370.2433			1	25399.88		
377.1808			1	17438.27		
385.2333			1	17489.13		
388.1968			1	21499.84		

MSMS Spectrum

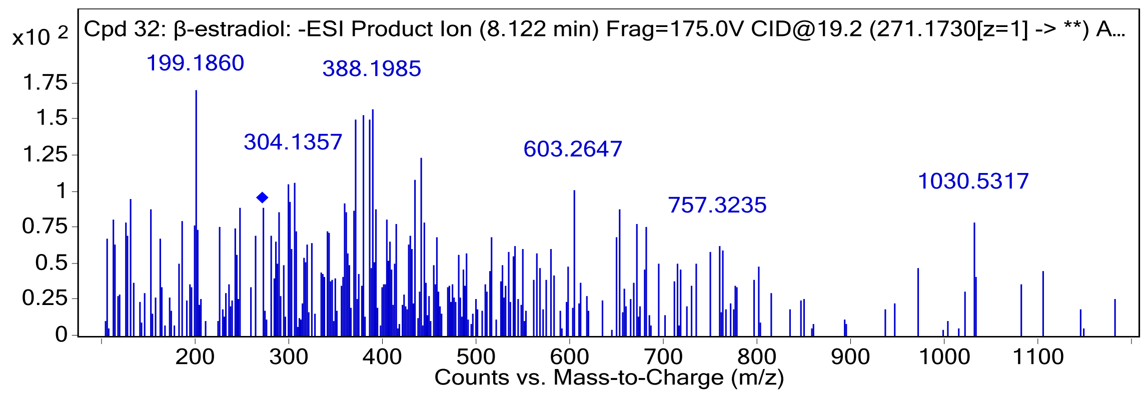

MS/MS Spectrum Peak List

m/z	Abund
199.186	170.54
304.1357	106.72
369.1672	111.63
369.2386	150.23

370.2415	128.92
377.179	153.13
385.2268	150.01
388.1985	157.62
432.2184	109.14
440.2426	124.07

Compound Structure

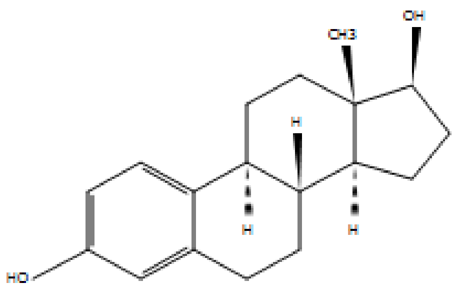

Compound Label	Name	*m/z*	RT	Algorithm	Mass
Cpd 33: 3beta,7beta,12alpha-Trihydroxy-5alpha-cholan-	**3beta,7beta,12alpha-Trihydroxy-5alpha-cholan-24-oic Acid**	453.2821	8.171	Auto MS/MS	408.283

MS Spectrum

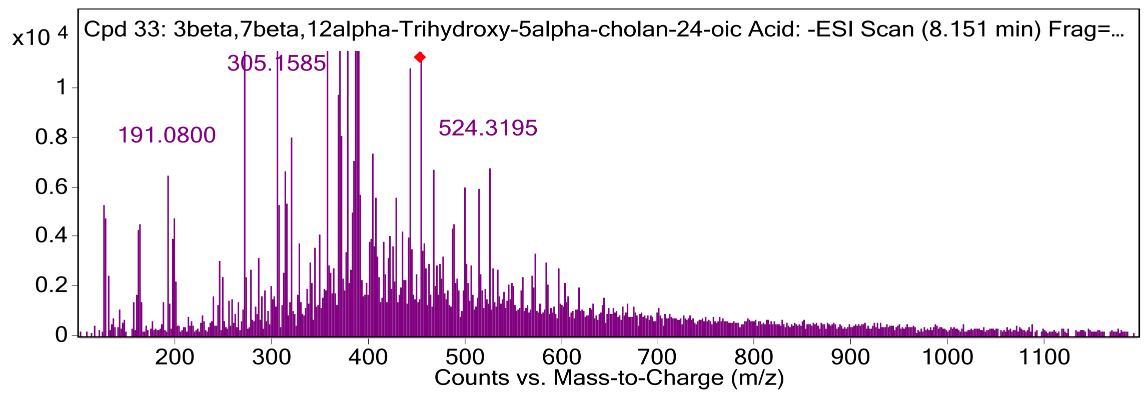

MS Zoomed Spectrum

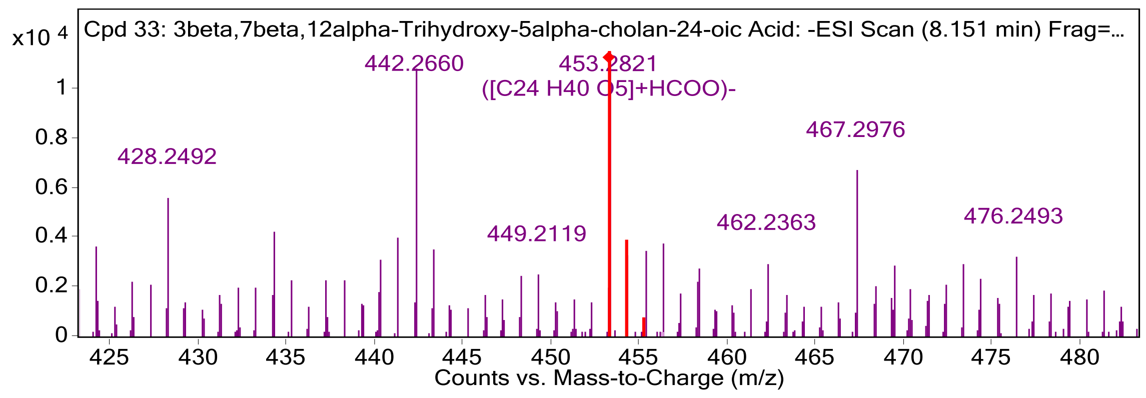

MS Spectrum Peak List

m/z	*Calc m/z*	Diff(ppm)	z	Abund	Formula	Ion
271.1731				16080.03		
305.1585			1	29438.81		
369.2358				15024.27		
370.2433			1	27147.26		
385.2322			1	13837.88		
387.2475			1	16221.4		
388.1969			1	15930.23		
453.2821	453.2858	8.03	1	11487.37	C24 H40 O5	(M+HCOO)-
454.2813	454.2892	17.3	1	3324.05	C24 H40 O5	(M+HCOO)-
455.2874	455.2919	9.9	1	3482.71	C24 H40 O5	(M+HCOO)-

MSMS Spectrum

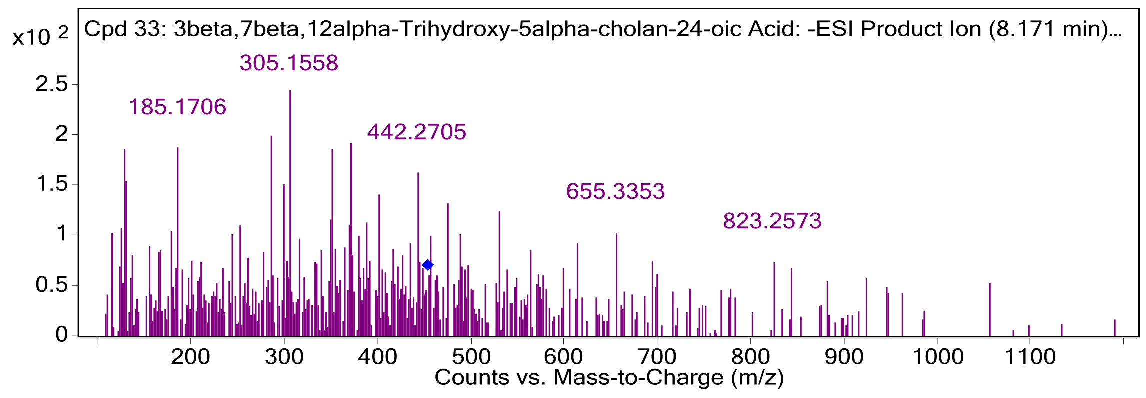

MS/MS Spectrum Peak List

m/z	z	Abund
127.0565		187.01
130.0905		155.2
185.1706		188.16
285.1876		200.39
298.2572		152
305.1558		244.56
350.9773		186.86
370.2427	1	192.45
400.2155		141.68
442.2705		163.22

Compound Structure

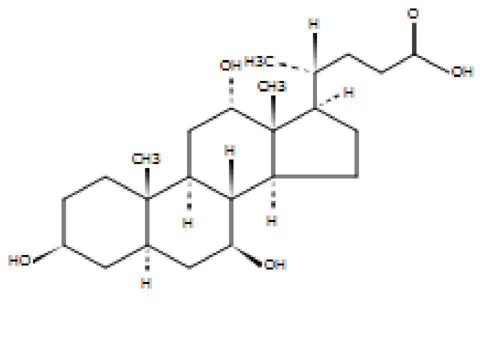

Compound Label	Name	*m/z*	RT	Algorithm	Mass
Cpd 34: (1R,2S,4R,5S)-2,5-Fenchanediol 2-O-b-D-glucoside	**(1R,2S,4R,5S)-2,5-Fenchanediol 2-O-b-D-glucoside**	377.1802	8.302	Auto MS/MS	332.1826

MS Spectrum

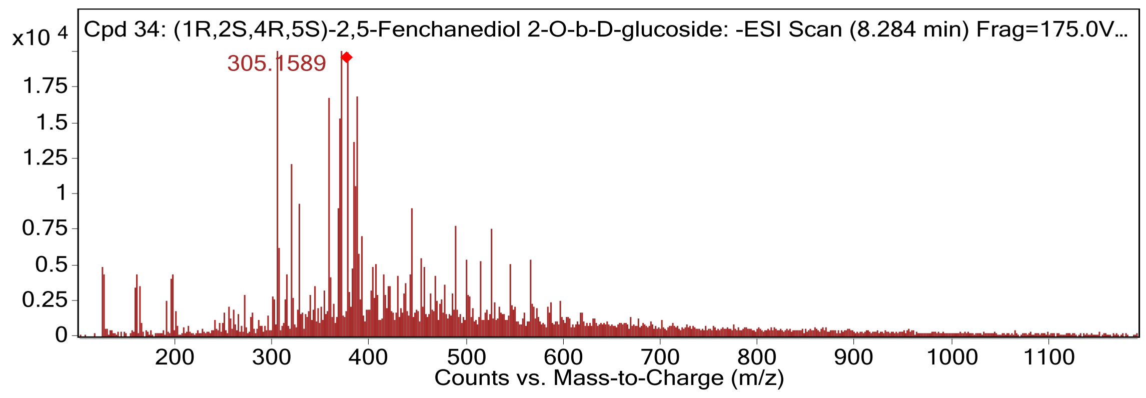

MS Zoomed Spectrum

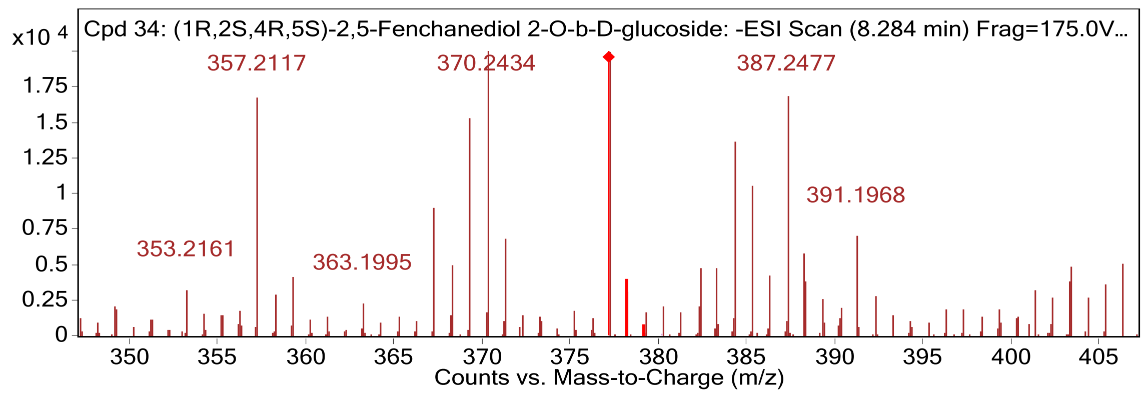

MS Spectrum Peak List

m/z	*Calc m/z*	Diff(ppm)	z	Abund	Formula	Ion
305.1589			1	34836.67		
319.1745			1	12186.81		
357.2117			1	16825.37		
369.2363				15329.3		
370.2434			1	25931.9		
377.1802	377.1817	4.08	1	20000.21	C16 H28 O7	(M+HCOO)-
378.1848	378.1851	0.81	1	3210.24	C16 H28 O7	(M+HCOO)-
379.1923	379.1872	-13.31	1	1789.82	C16 H28 O7	(M+HCOO)-
384.2595				13733.04		
387.2477			1	16922.37		

MSMS Spectrum

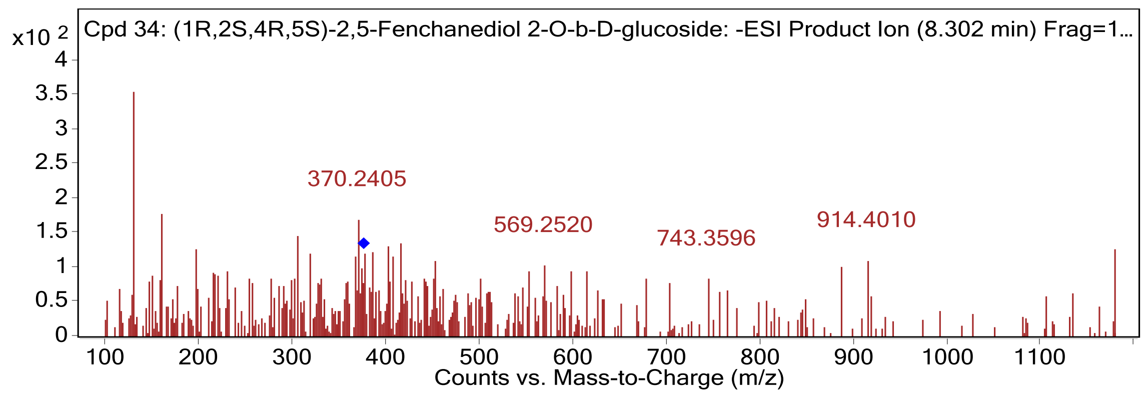

MS/MS Spectrum Peak List

m/z	z	Abund
130.0929		356.07
131.0925		142.32
160.8468		179.05
197.8146		128.22
305.1558		145.81
370.2405	1	170.45
385.2618		124.04
402.2243		132.58
415.2345		136.25
1177.19		127.41

Compound Structure

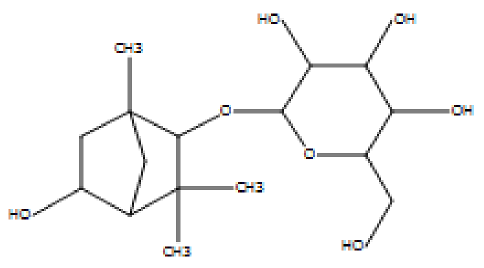

Compound Label	Name	*m/z*	RT	Algorithm	Mass
Cpd 35: Piperoic acid	**Piperoic acid**	357.2118	8.343	Auto MS/MS	358.2191

MS Spectrum

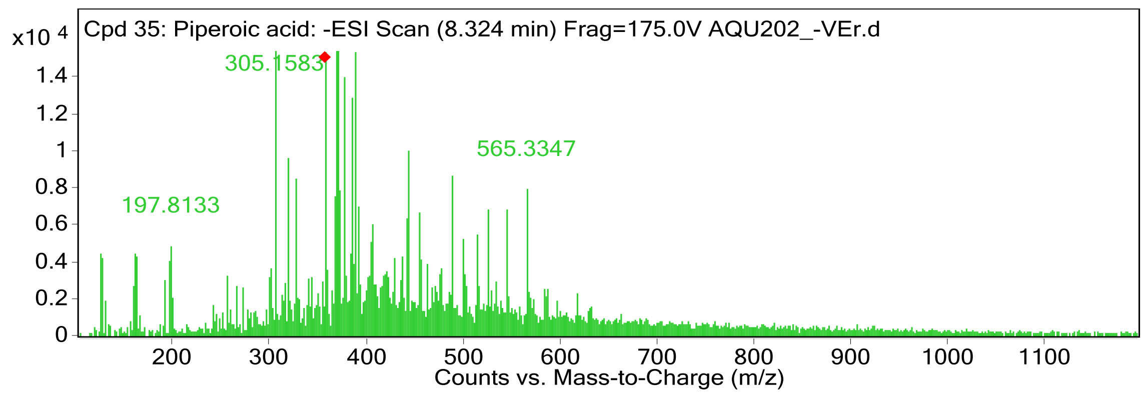

MS Zoomed Spectrum

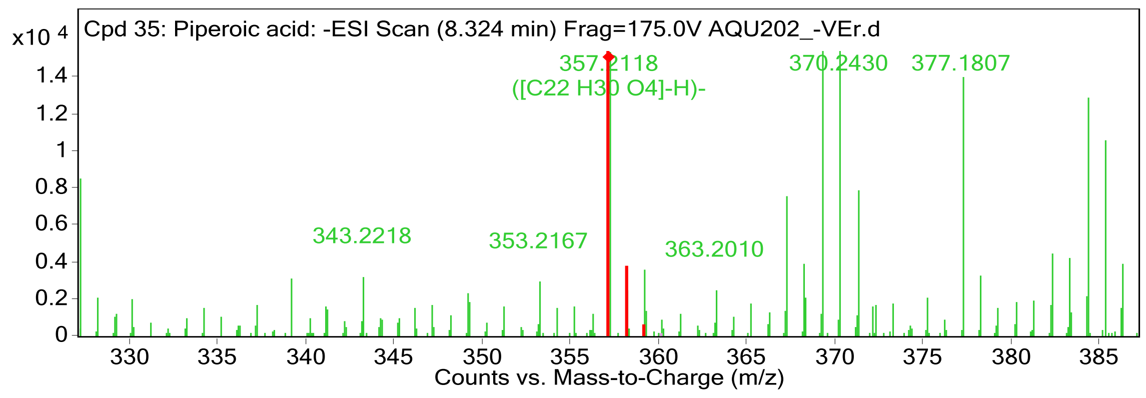

MS Spectrum Peak List

m/z	*Calc m/z*	Diff(ppm)	z	Abund	Formula	Ion
305.1583			1	28380.91		
357.2118	357.2071	-13.05	1	15381.6	C22 H30 O4	(M-H)-
358.2151	358.2105	-12.71	1	2551.87	C22 H30 O4	(M-H)-
359.2185	359.2134	-14.23	1	1436.71	C22 H30 O4	(M-H)-
369.2364				18365.69		
370.243			1	24481.63		
377.1807			1	14006.09		
384.2592				12922.96		
385.2324			1	10616.63		
387.2481			1	15412.31		

MSMS Spectrum

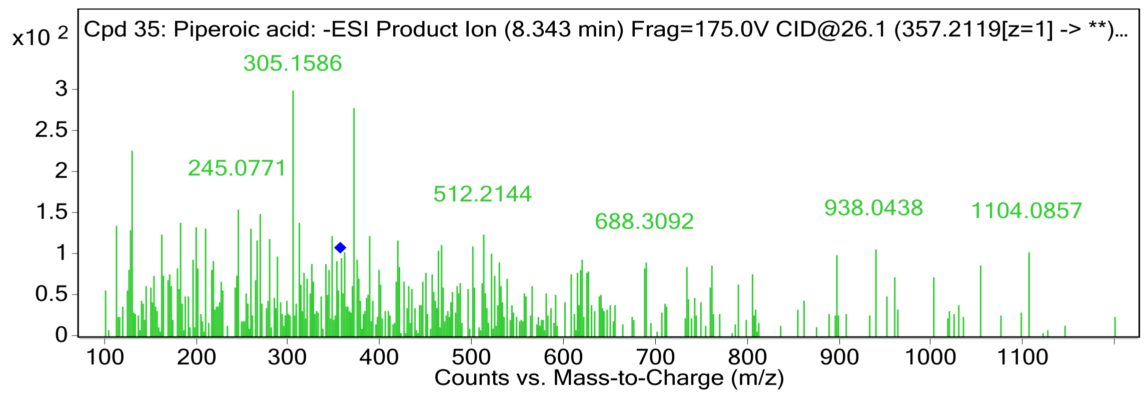

MS/MS Spectrum Peak List

m/z	Abund
112.0439	135.59
130.0886	226.86
182.1235	140.02
199.1869	133.21
245.0771	156.12
268.2327	149.21
305.1586	299.04
311.1522	139.63
370.2117	136.56
370.2437	279.23

Compound Structure

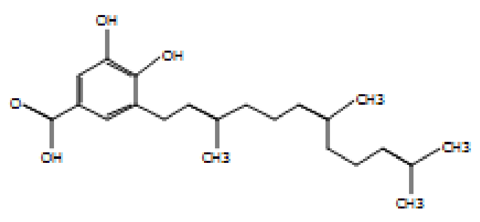

Compound Label	Name	*m/z*	RT	Algorithm	Mass
Cpd 36: (E,E,E)-Sylvatine	**(E,E,E)-Sylvatine**	442.2664	8.542	Auto MS/MS	383.2526

MS Spectrum

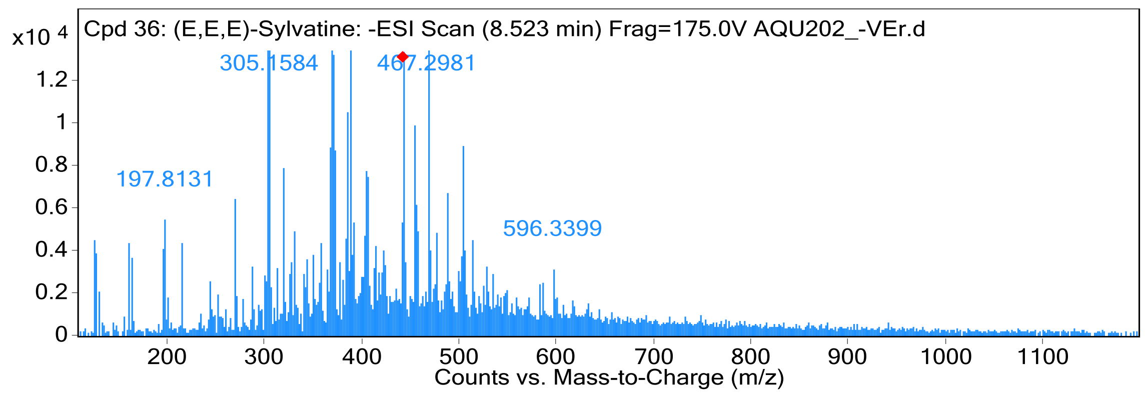

MS Zoomed Spectrum

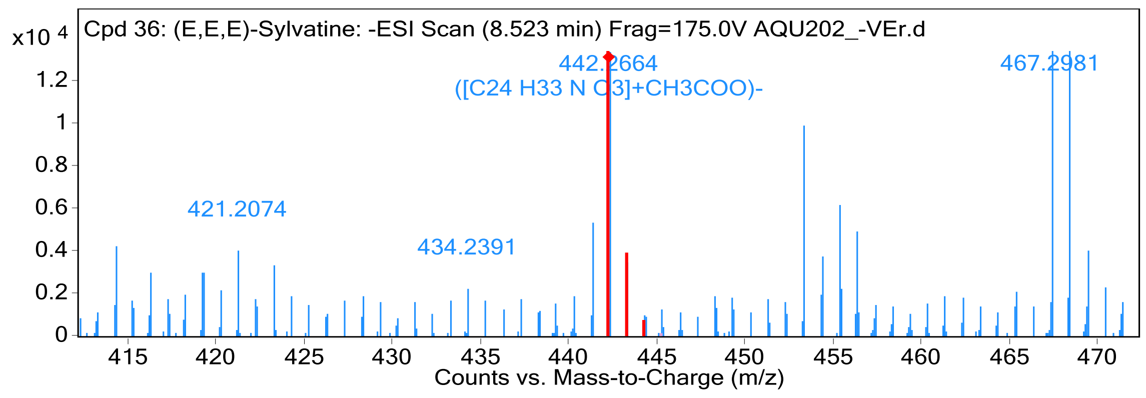

MS Spectrum Peak List

m/z	*Calc m/z*	Diff(ppm)	z	Abund	Formula	Ion
303.225			1	14861.91		
305.1584			1	16028.37		
369.2367			1	34924.43		
370.2408			1	13245.44		
387.2478			1	13440.61		
442.2664	442.2599	-14.67	1	13376.18	C24 H33 N O3	(M+CH3COO)-
443.2694	443.2632	-14.04	1	3532.17	C24 H33 N O3	(M+CH3COO)-
444.2747	444.2661	-19.37	1	945.73	C24 H33 N O3	(M+CH3COO)-

467.2981			1	51932.1		
468.3009			1	13909.2		

MSMS Spectrum

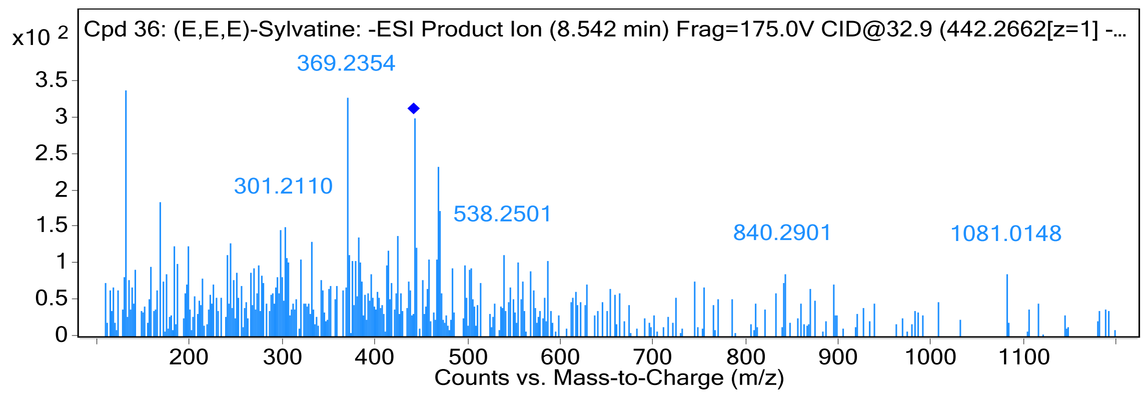

MS/MS Spectrum Peak List

m/z	z	Abund
130.0906		337.03
168.0944		185.75
297.158		146.48
301.211		151.13
369.2354	1	327.76
381.1759		137
423.9428		139.02
442.2672		300.72
467.2984		234.33
468.2945		173.41

Compound Structure

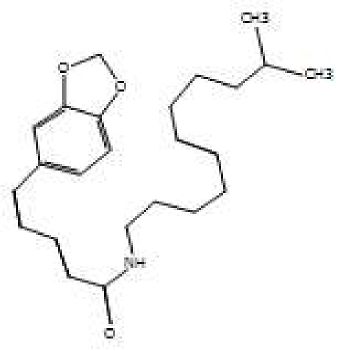

Compound Label	Name	*m/z*	RT	Algorithm	Mass
Cpd 37: Verapamil	**Verapamil**	453.2815	8.543	Auto MS/MS	454.2877

MS Spectrum

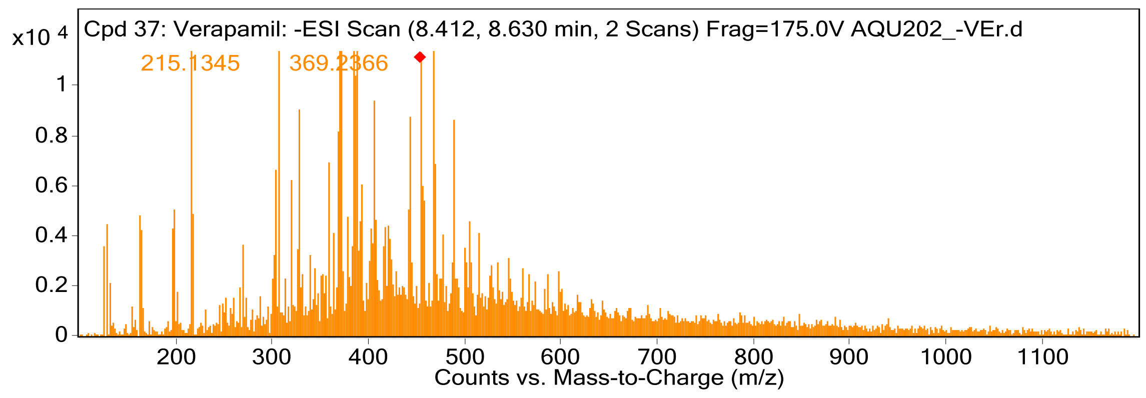

MS Zoomed Spectrum

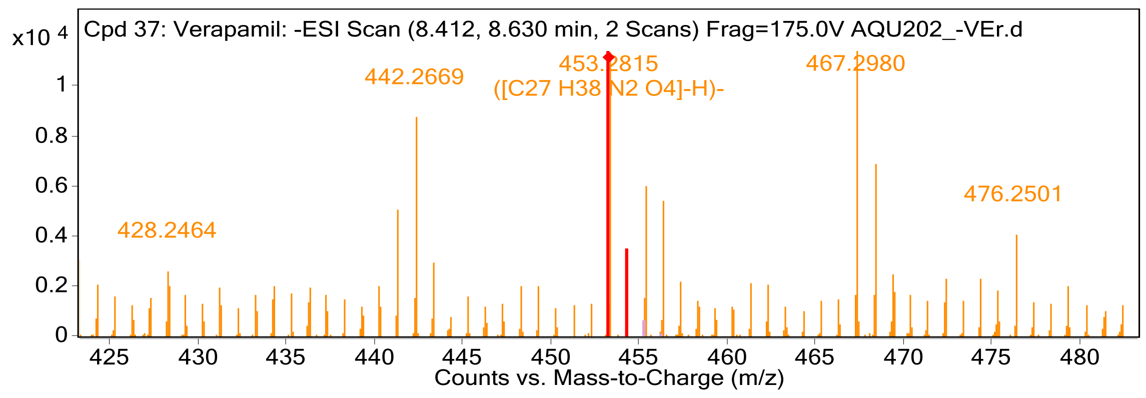

MS Spectrum Peak List

m/z	*Calc m/z*	Diff(ppm)	z	Abund	Formula	Ion
215.1345			1	30906.83		
305.1584			1	22850.84		
369.2366				28265.27		
370.2429				20272.92		
371.2472			1	11694.71		
384.259				12056.2		
387.2476			1	13939.47		
453.2815	453.2759	-12.47	1	11376.55	C27 H38 N2 O4	(M-H)-
454.2801	454.2791	-2.18	1	3538.54	C27 H38 N2 O4	(M-H)-
467.298			1	22835.77		

MSMS Spectrum

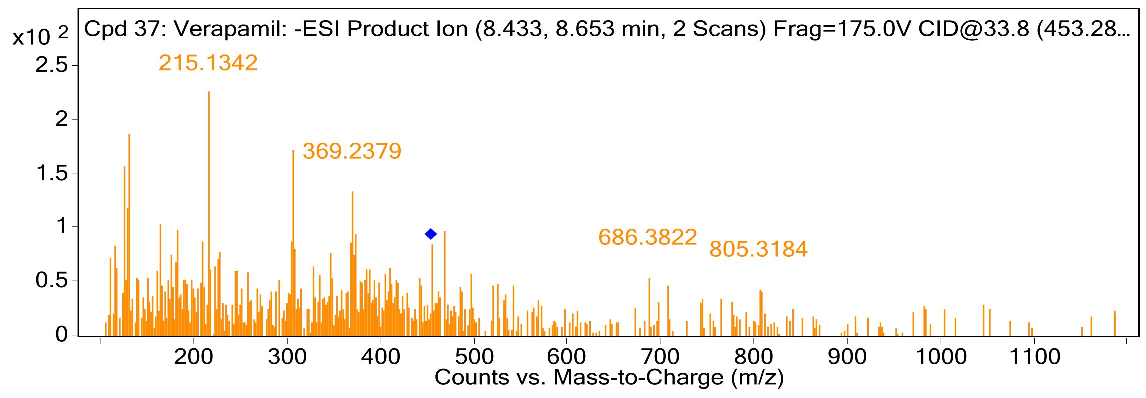

MS/MS Spectrum Peak List

m/z	z	Abund
124.996		157.84
127.056		119.59
130.0907		188.1
162.8443		104.09
181.1049		99.18
215.1342	1	227.65

305.1572		172.72
369.2379		134.21
371.2539		94.83
467.2978		97.28

Compound Structure

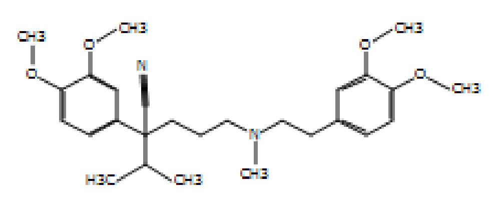

Compound Label	Name	*m/z*	RT	Algorithm	Mass
Cpd 38: 3alpha,7beta,12alpha-Trihydroxy-5alpha-cholan-	**3alpha,7beta,12alpha-Trihydroxy-5alpha-cholan-24-oic Acid**	467.2979	8.568	Auto MS/MS	408.2841

MS Spectrum

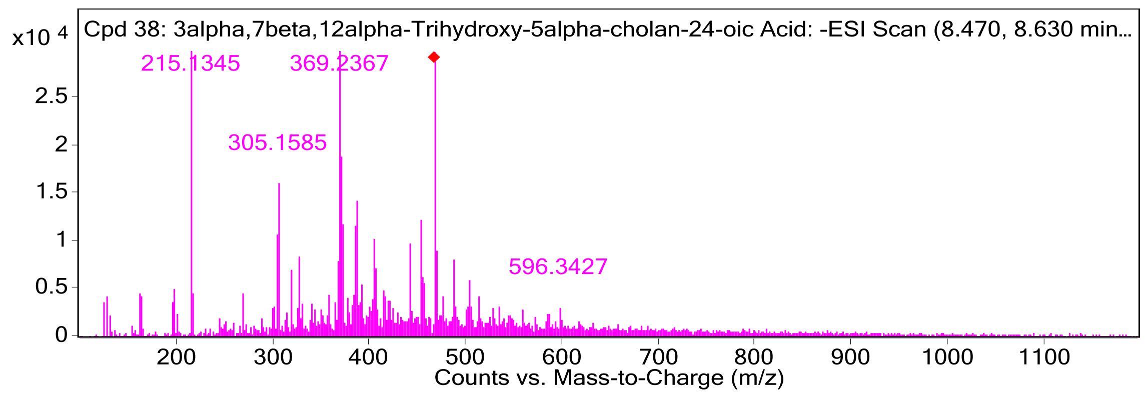

MS Zoomed Spectrum

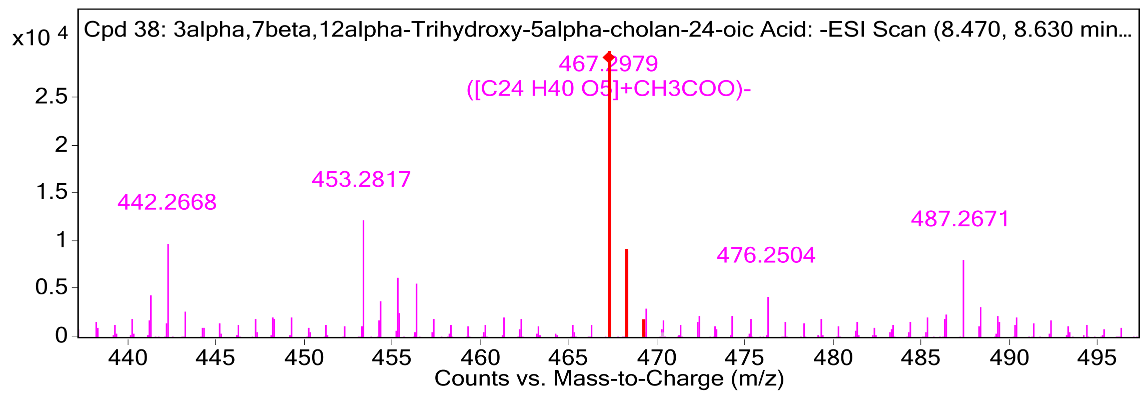

MS Spectrum Peak List

m/z	*Calc m/z*	Diff(ppm)	z	Abund	Formula	Ion
215.1345			1	30828.34		
305.1585			1	16124.38		
369.2367				31980.05		
370.2426				18871.58		
371.2474			1	11786.79		
387.2474			1	14354.26		
453.2817			1	12304.74		
467.2979	467.3014	7.62	1	29832.12	C24 H40 O5	(M+CH3COO)-
468.3009	468.3048	8.5	1	9110.93	C24 H40 O5	(M+CH3COO)-
469.3066	469.3076	2.08	1	3036.39	C24 H40 O5	(M+CH3COO)-

MSMS Spectrum

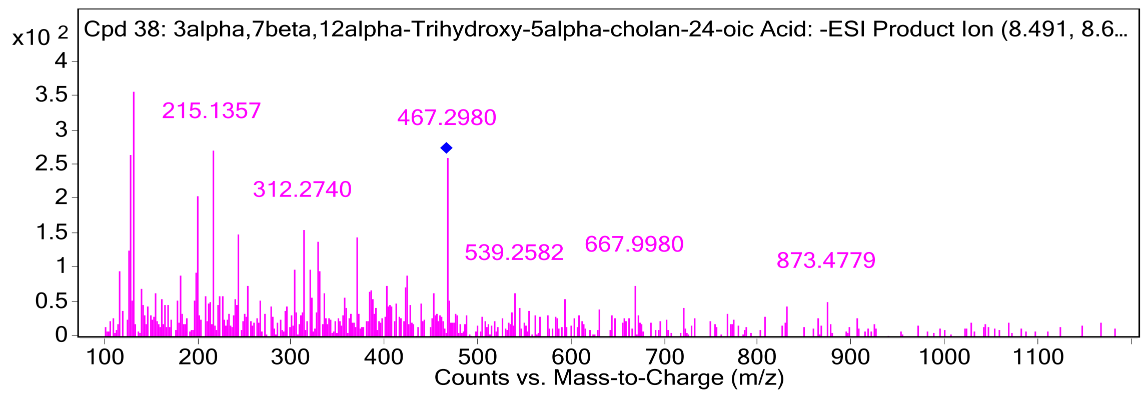

MS/MS Spectrum Peak List

m/z	z	Abund
124.9957		126.32
127.0559		264.21
130.0915		357.63
199.1875		205
215.1357		270.49
243.1783		150.21
312.274		155.64
327.2212		139.93
369.2368		145.33
467.298	1	260.43

Compound Structure

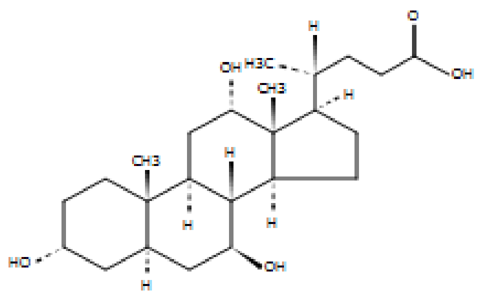

Compound Label	*m/z*	RT	Algorithm
Compound 39	215.1346	8.668	Auto MS/MS

MS Spectrum

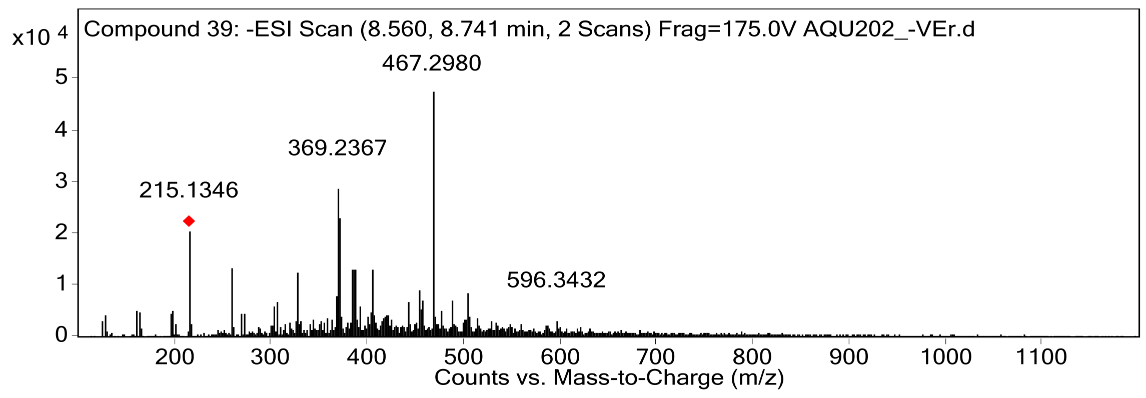

MS Zoomed Spectrum

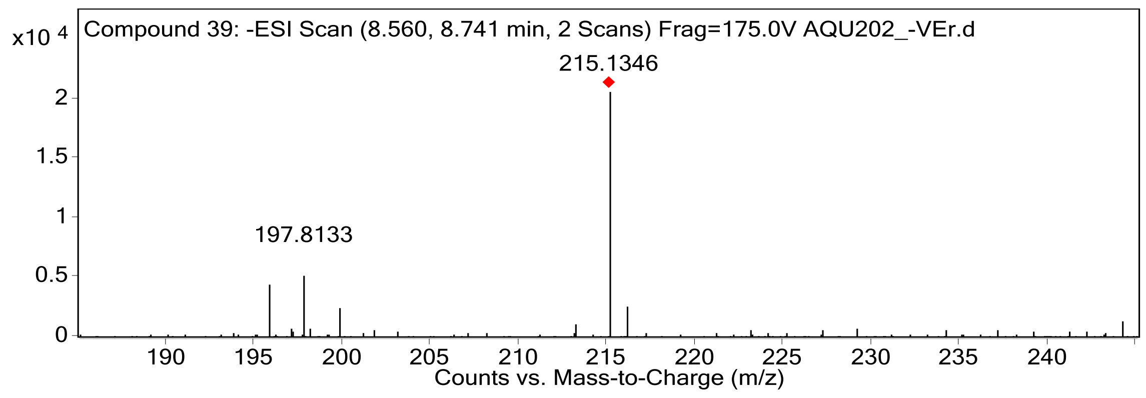

MS Spectrum Peak List

m/z	z	Abund
215.1346	1	20611.34
216.1382	1	2590.89
217.1425	1	406.42
259.1619		13377.26
369.2367		28808.65
370.2428		23051.93
371.2495	1	14975.88
385.2342	1	13203.63
387.2471	1	13102.18
467.298	1	47631.06

MSMS Spectrum

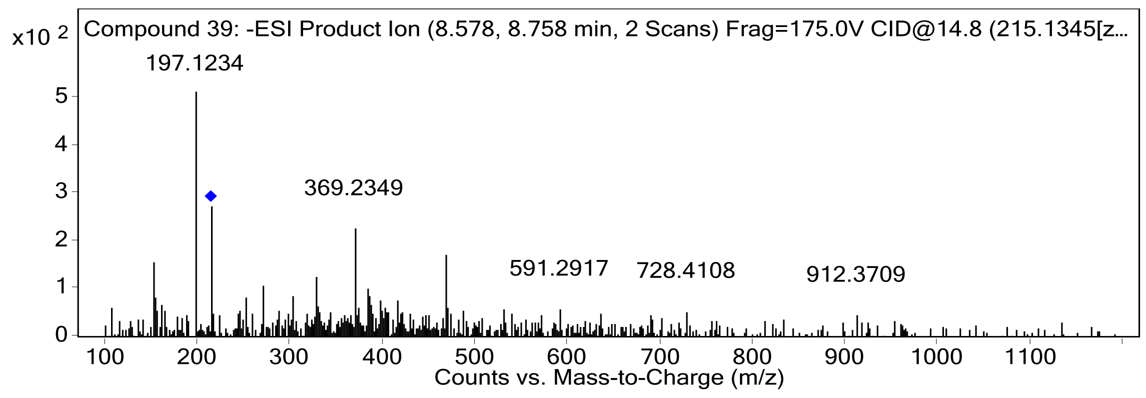

MS/MS Spectrum Peak List

m/z	z	Abund
153.1308		156.28
197.1234		513.4
215.133	1	272.69
271.1595		106.17
327.2243		126.85
369.2349	1	226.55
370.2432	1	107.85
384.255		101.13
467.2938	1	172.35
468.3052	1	92.95

Compound Label	Name	*m/z*	RT	Algorithm	Mass
Cpd 40: 3alpha,7beta,12alpha-Trihydroxy-5alpha-cholan-	**3alpha,7beta,12alpha-Trihydroxy-5alpha-cholan-24-oic Acid**	467.2977	8.88	Auto MS/MS	408.2841

MS Spectrum

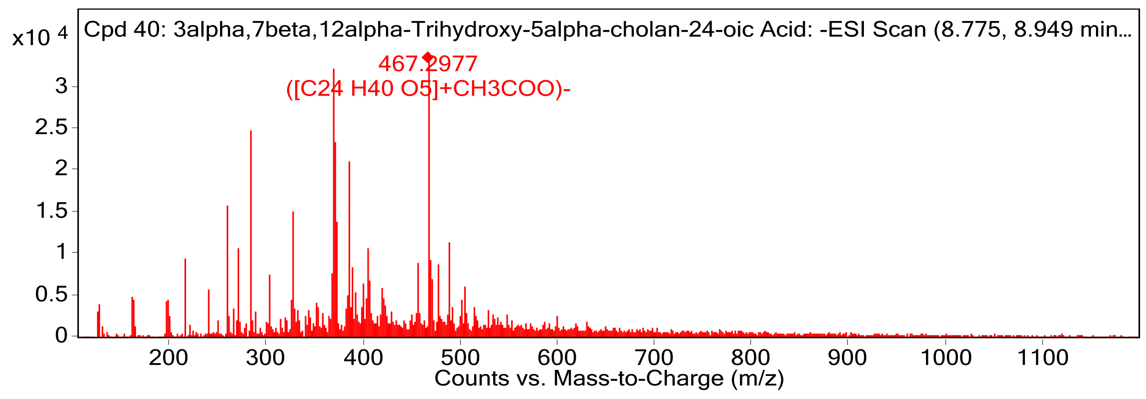

MS Zoomed Spectrum

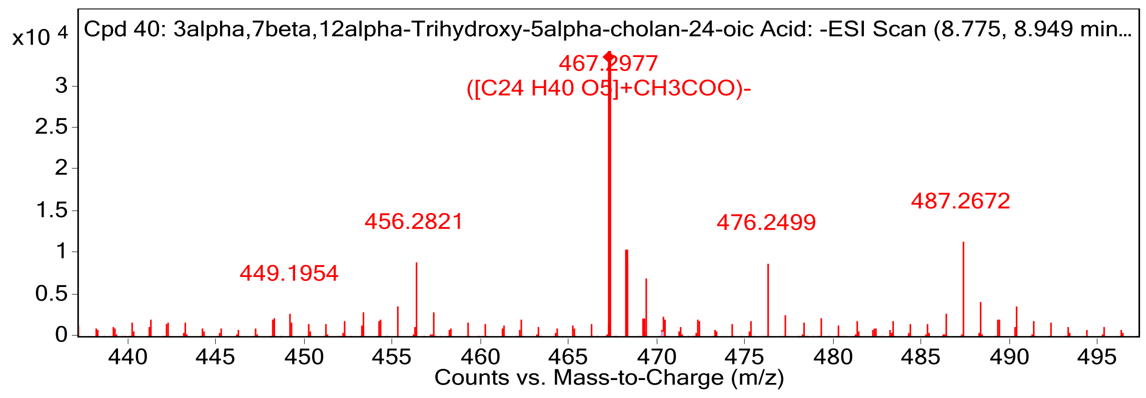

MS Spectrum Peak List

m/z	*Calc m/z*	Diff(ppm)	z	Abund	Formula	Ion
259.1617			1	15839.07		
283.162			1	24783.77		
327.2254			1	15167.71		
369.2368				32247.22		
370.2424				23339.7		
384.2594				21212.35		
385.2356			1	14670.61		

467.2977	467.3014	7.88	1	34167.39	C24 H40 O5	(M+CH3COO)-
468.3007	468.3048	8.83	1	9384.11	C24 H40 O5	(M+CH3COO)-
469.3073	469.3076	0.57	1	4164.45	C24 H40 O5	(M+CH3COO)-

MSMS Spectrum

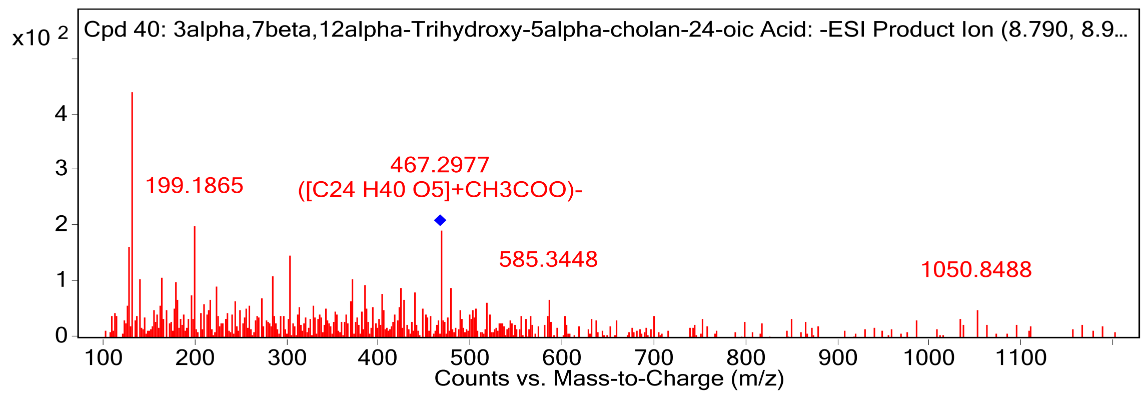

MS/MS Spectrum Peak List

m/z	*Calc m/z*	Diff (ppm)	Abund	Formula	Ion
127.0555			163.56		
130.0913			441.94		
140.0752			104.37		
162.8437			107.83		
179.1244			100.39		
199.1865			200.34		
283.1628			110.46		
303.2234			147.54		
370.2414			106.83		
467.2977	467.3014	8.04	193.05	C24 H40 O5	(M+CH3COO)-

Compound Structure

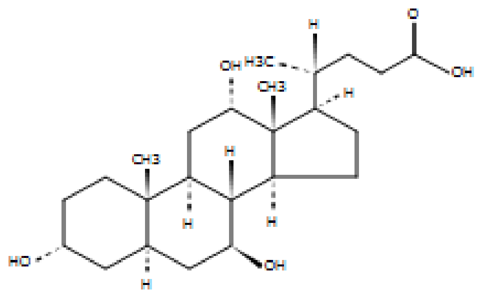

Compound Label	Name	*m/z*	RT	Algorithm	Mass
Cpd 41: Fesoterodine	**Fesoterodine**	456.2818	9.084	Auto MS/MS	411.2829

MS Spectrum

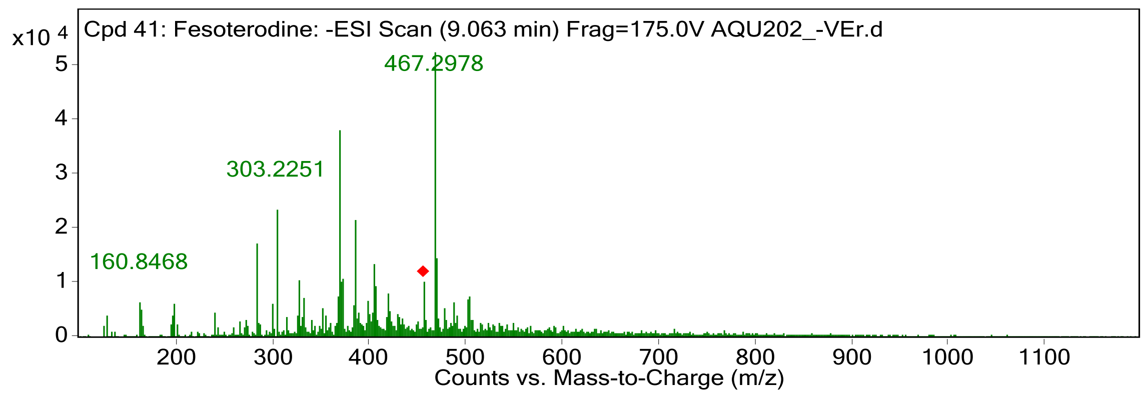

MS Zoomed Spectrum

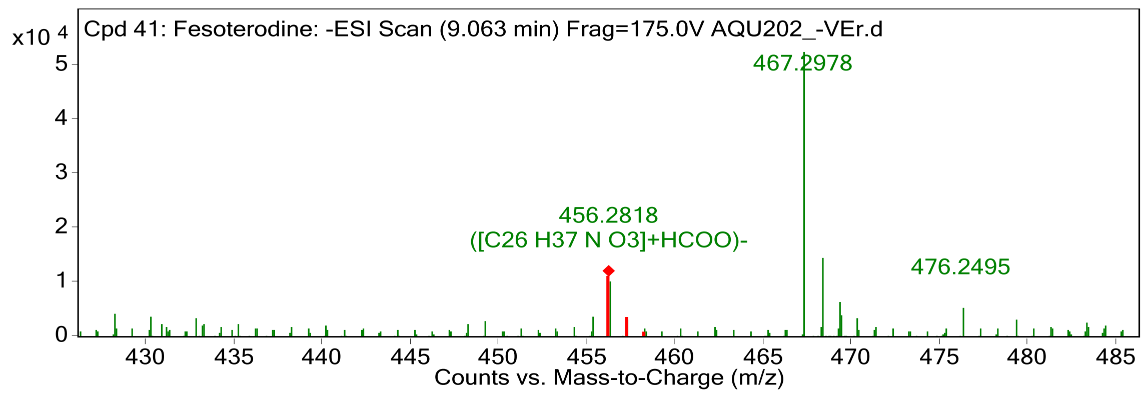

MS Spectrum Peak List

m/z	*Calc m/z*	Diff(ppm)	z	Abund	Formula	Ion
283.1621			1	17297.21		
303.2251			1	23395.75		
369.2369			1	38084.96		
384.2589				21581.98		
404.2278				13571.15		
456.2818	456.2755	-13.7	1	10325.96	C26 H37 N O3	(M+HCOO)-
457.2813	457.2789	-5.26	1	3310.39	C26 H37 N O3	(M+HCOO)-
458.2912	458.2817	-20.62	1	983.68	C26 H37 N O3	(M+HCOO)-
467.2978			1	52300.84		
468.3007			1	14646.39		

MSMS Spectrum

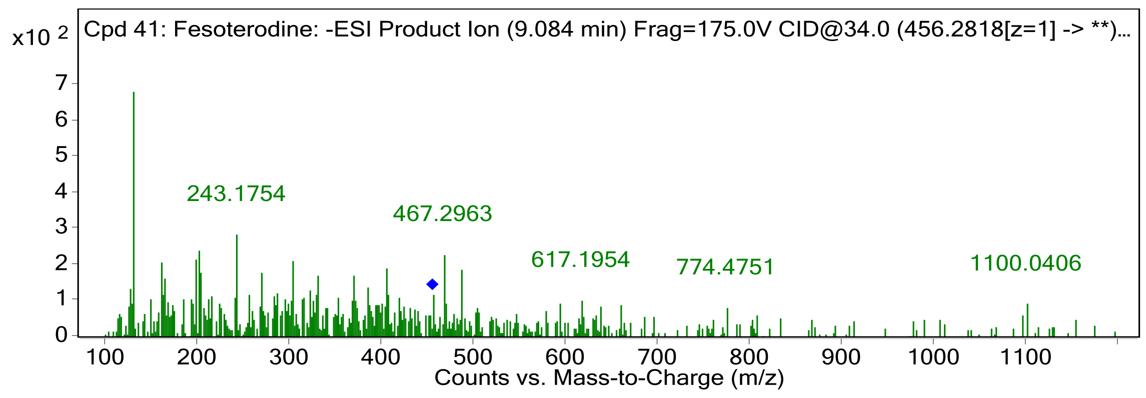

MS/MS Spectrum Peak List

m/z	Abund
130.0909	680.99
160.8457	207.16
197.8095	214.36
201.8057	240.11
243.1754	284.99

270.2253	178.91
303.2246	212.39
405.2129	189.64
467.2963	229.38
487.2669	186.16

Compound Structure

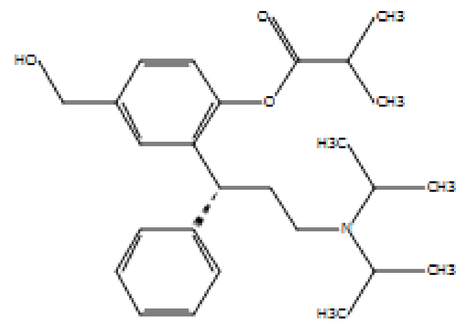

Compound Label	Name	*m/z*	RT	Algorithm	Mass
Cpd 42: 3alpha,7beta,12alpha-Trihydroxy-5alpha-cholan-	**3alpha,7beta,12alpha-Trihydroxy-5alpha-cholan-24-oic Acid**	467.2982	9.207	Auto MS/MS	408.2846

MS Spectrum

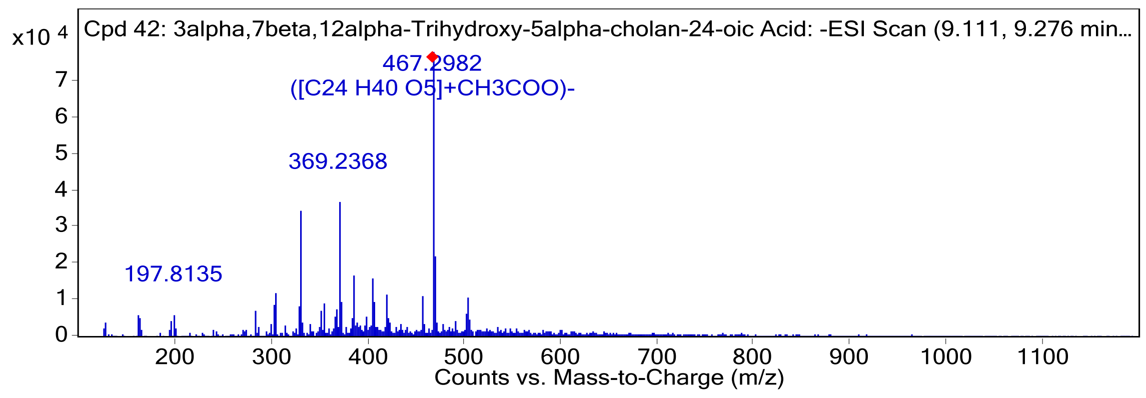

MS Zoomed Spectrum

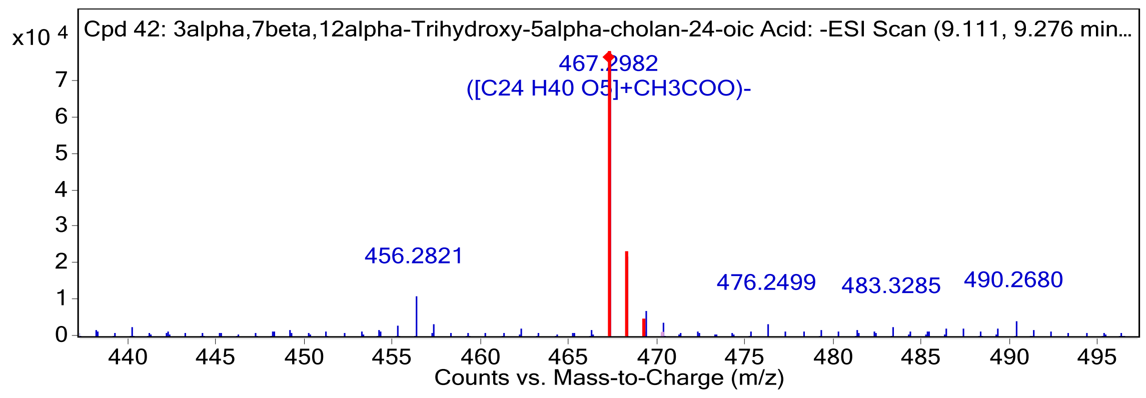

MS Spectrum Peak List

m/z	*Calc m/z*	Diff(ppm)	z	Abund	Formula	Ion
303.2253				11978.68		
329.2417			1	34650.36		
369.2368			1	36946.53		
384.2592			1	16805.12		
404.2286				15960.39		
418.244			1	11607.72		
456.2821			1	11062.95		
467.2982	467.3014	6.95	1	78024.8	C24 H40 O5	(M+CH3COO)-
468.301	468.3048	8.11	1	22126.08	C24 H40 O5	(M+CH3COO)-
469.3098	469.3076	-4.7	1	7177.62	C24 H40 O5	(M+CH3COO)-

MSMS Spectrum

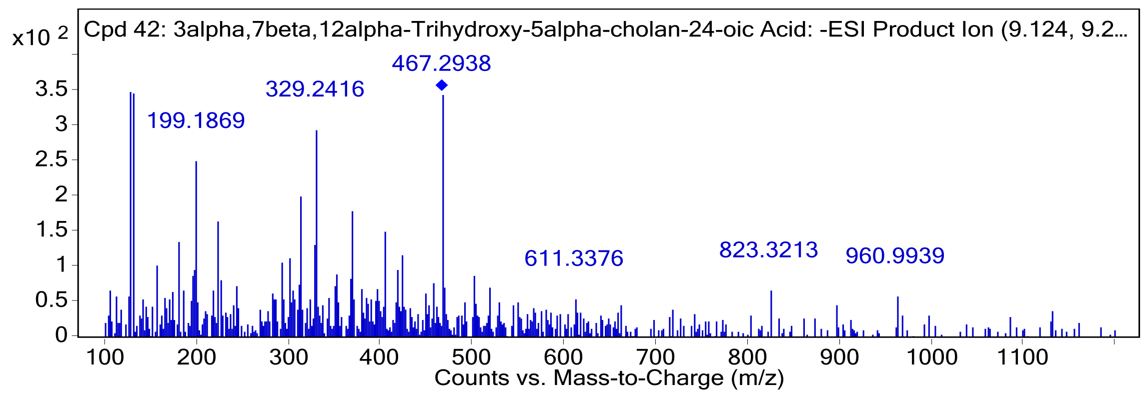

MS/MS Spectrum Peak List

m/z	z	Abund
127.055		348.47
130.0909		346.33
180.1083		135.09
199.1869		249.88
222.129		163.81
312.2706		200.81
329.2416		294.63
369.2337		179.42
404.2254	1	149.76
467.2938	1	344.25

Compound Structure

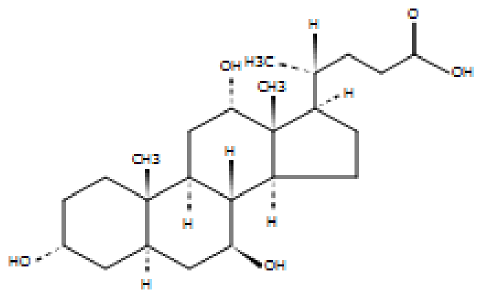

Compound Label	Name	*m/z*	RT	Algorithm	Mass

Cpd 43: Ovatine	**Ovatine**	384.2593	9.441	Auto MS/MS	385.2639

MS Spectrum

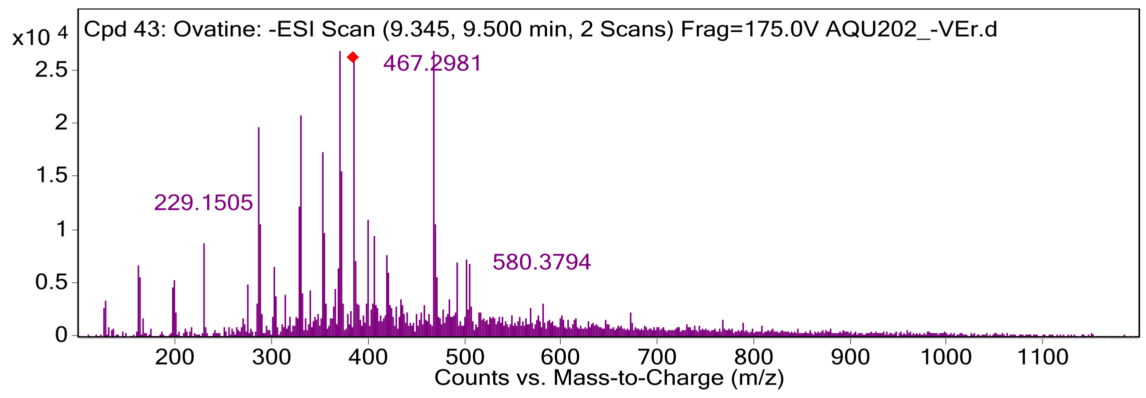

MS Zoomed Spectrum

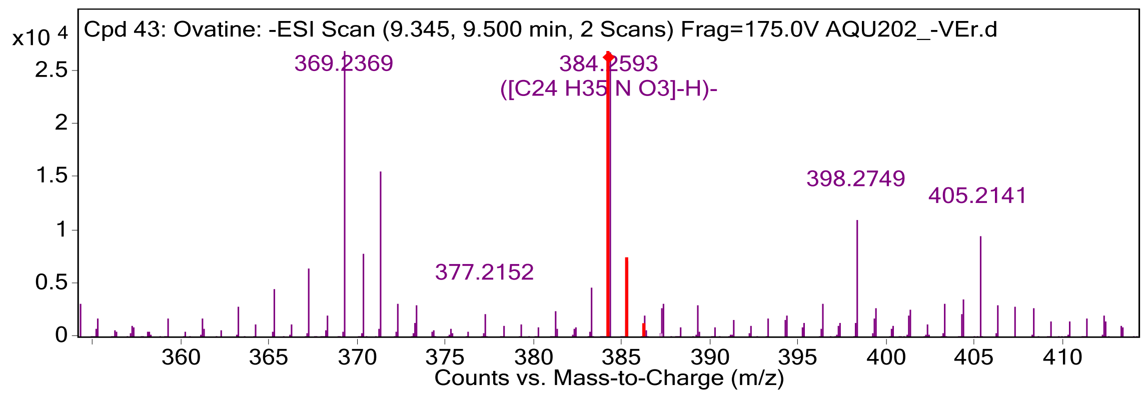

MS Spectrum Peak List

m/z	*Calc m/z*	Diff(ppm)	z	Abund	Formula	Ion
285.1779			1	19749.22		
327.2264			1	12269.59		
329.2415			1	20826.11		
351.226			1	17356.42		
369.2369			1	33631.74		
371.2519			1	15572.3		
384.2593	384.2544	-12.83	1	26768.15	C24 H35 N O3	(M-H)-
385.2559	385.2577	4.76	1	7192.32	C24 H35 N O3	(M-H)-
386.2418	386.2607	48.98	1	2028.51	C24 H35 N O3	(M-H)-
467.2981			1	37138.86		

MSMS Spectrum

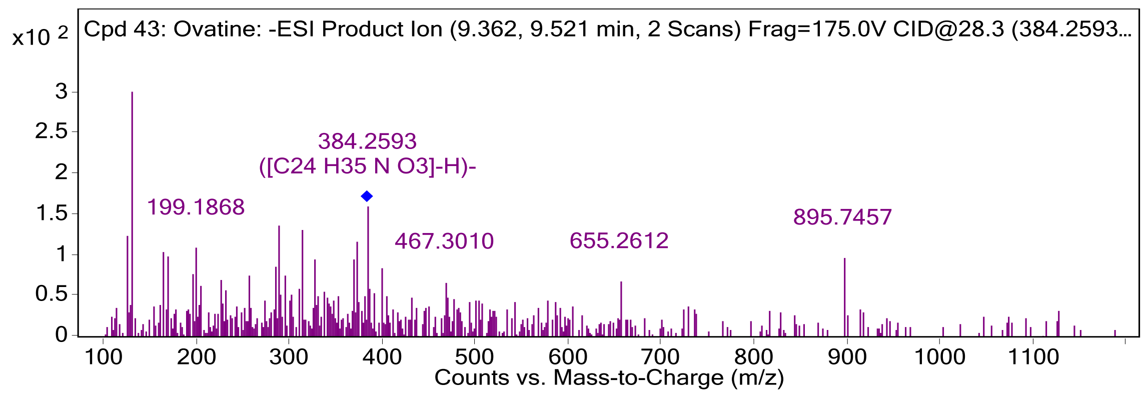

MS/MS Spectrum Peak List

m/z	*Calc m/z*	Diff (ppm)	z	Abund	Formula	Ion
124.994				124.35		
130.0912				301.67		
162.8446				105.33		
168.0962				98.64		
199.1868				109.45		
287.2297				136.27		
312.2726				130.73		
371.2536				116.56		
384.2593	384.2544	-12.77	1	160.05	C24 H35 N O3	(M-H)-
895.7457				97.12		

Compound Structure

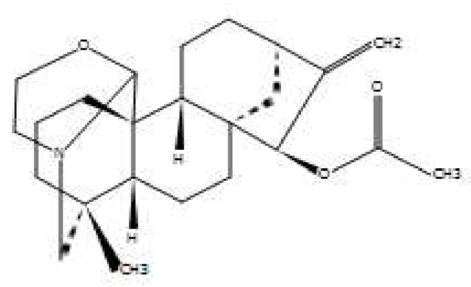

**Compound Label**	***m/z***	**RT**	**Algorithm**
Compound 44	273.1777	9.68	Auto MS/MS

MS Spectrum

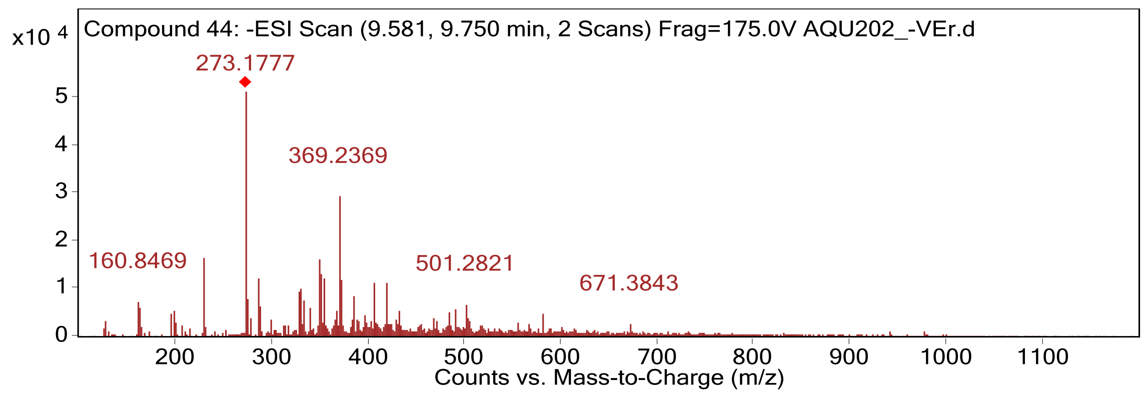

MS Zoomed Spectrum

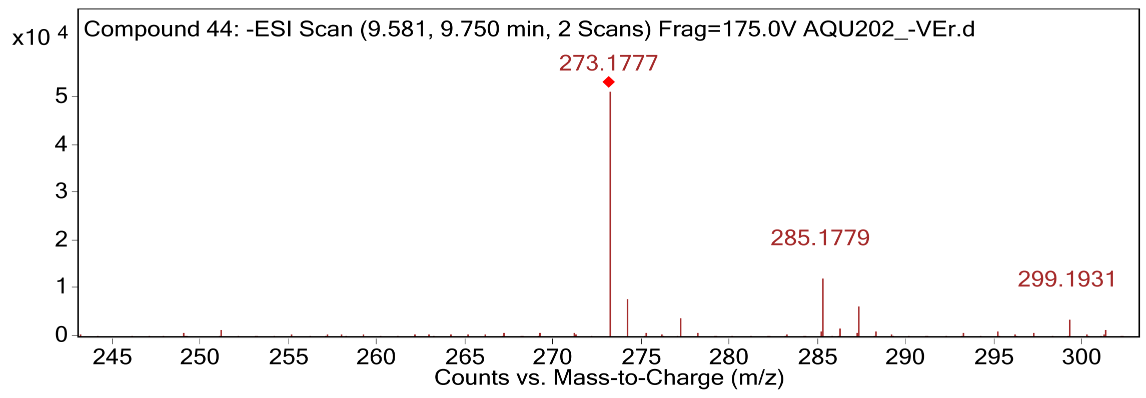

MS Spectrum Peak List

m/z	z	Abund
229.1503	1	16513.49
273.1777	1	51419.75
274.1808	1	7998.94
275.1827	1	837.68
285.1779		12228.78
349.1775	1	16194
351.226	1	13352.29
353.2411	1	12156.14
369.2369	1	29490.08
371.2521	1	11853.37

MSMS Spectrum

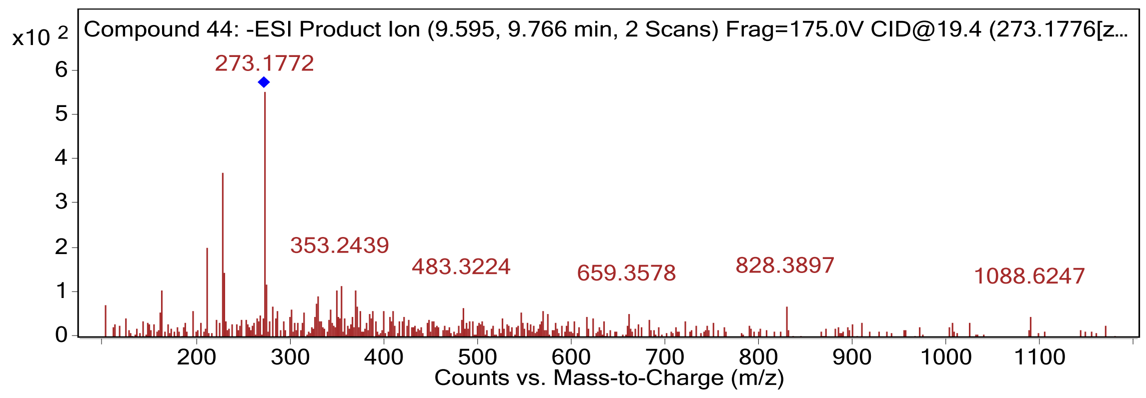

MS/MS Spectrum Peak List

m/z	z	Abund
162.8442		107.51
211.175		202.11
227.1707		371.31
229.1492		145.04
273.1772	1	553.63
274.1801	1	120.59
329.2405	1	94.19
349.1776		106.65
353.2439	1	114.81
369.2375	1	107.4

Compound Label	*m/z*	RT	Algorithm
Compound 45	329.2412	9.796	Auto MS/MS

MS Spectrum

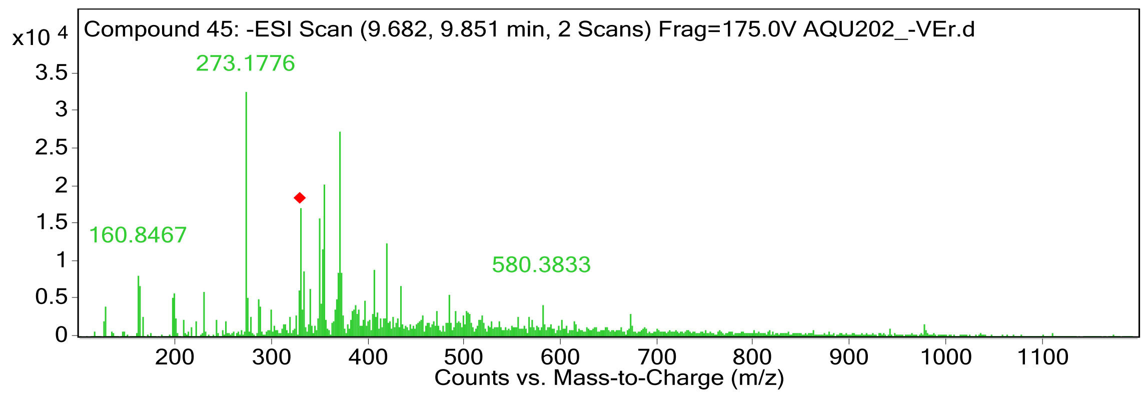

MS Zoomed Spectrum

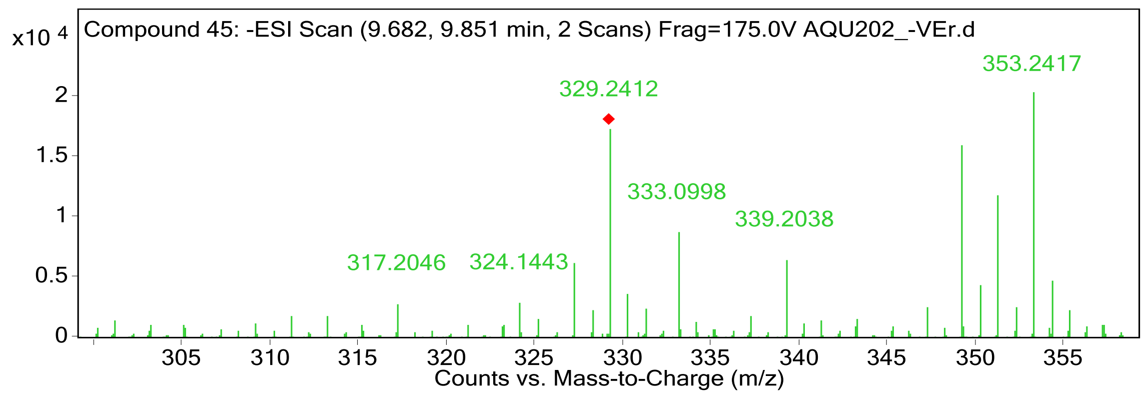

MS Spectrum Peak List

m/z	z	Abund
273.1776	1	32650.31
329.2412	1	17249.24
330.2447	1	3659.28
333.0998		8720.17
349.1773	1	15905.34
351.2257	1	11747.87
353.2417	1	20360.08
369.2367	1	27265.52
405.2136	1	9076.21
418.2435	1	12535.34

MSMS Spectrum

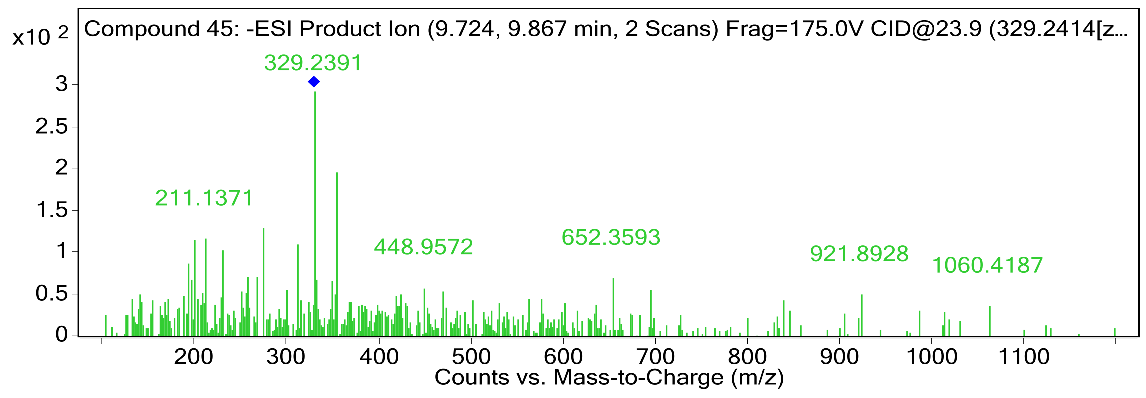

MS/MS Spectrum Peak List

m/z	z	Abund
193.1257		88.24
199.1428		116.03
211.1371		117.19
229.1494		103.03
257.3001		72.56
267.2091		72.59
273.1772		130.8
311.231		111.13
329.2391	1	293.87
353.2403		197

Compound Label	Name	*m/z*	RT	Algorithm	Mass
Cpd 46: Ethamoxytriphetol	**Ethamoxytriphetol**	418.2439	9.848	Auto MS/MS	419.2462

MS Spectrum

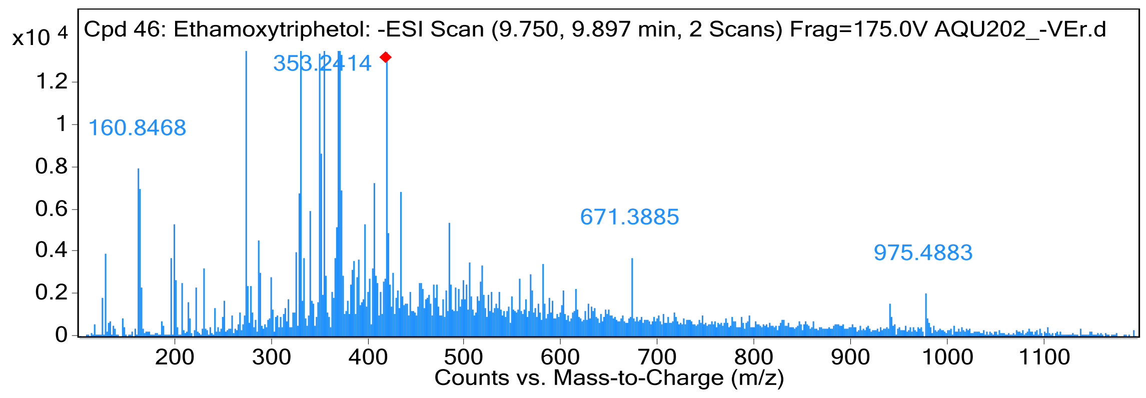

MS Zoomed Spectrum

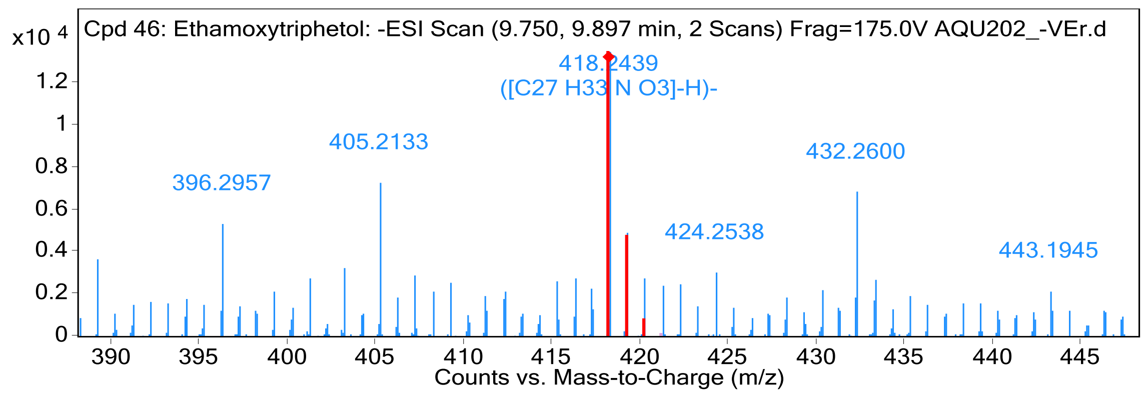

MS Spectrum Peak List

m/z	*Calc m/z*	Diff(ppm)	z	Abund	Formula	Ion
273.1776			1	17448.05		
329.2412			1	16734.27		
349.1777			1	13407.68		
351.226				8662.62		
353.2414			1	29042.96		
368.1339			1	15134.51		
369.2368			1	22947.19		
418.2439	418.2388	-12.17	1	13472.86	C27 H33 N O3	(M-H)-
419.2373	419.2421	11.37	1	4896.86	C27 H33 N O3	(M-H)-
420.23	420.2451	36.04	1	2809.3	C27 H33 N O3	(M-H)-

MSMS Spectrum

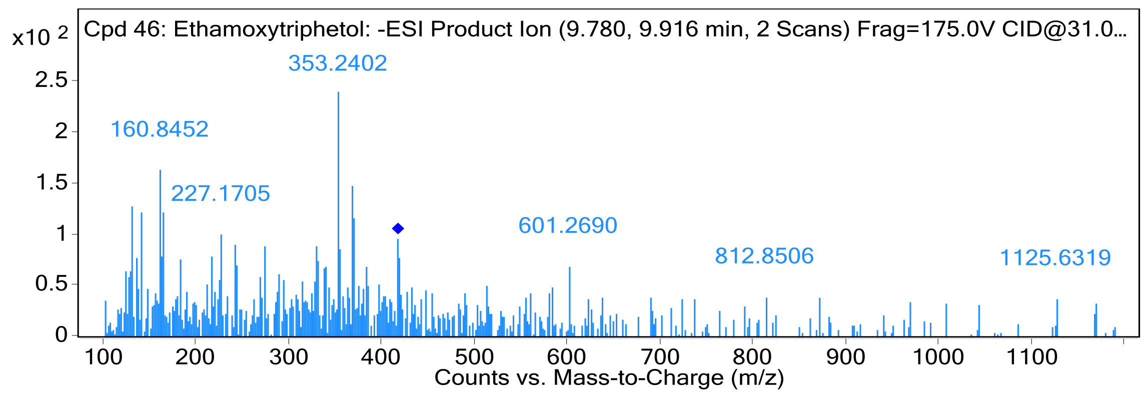

MS/MS Spectrum Peak List

m/z	z	Abund
130.0898		128.23
140.0758		122.26
160.8452		164.06
164.0741		122.04
227.1705		100.88
242.1929		90.79
353.2402	1	240.99
368.1342		147.67
369.2371	1	116.53
417.1976		96.92

Compound Structure

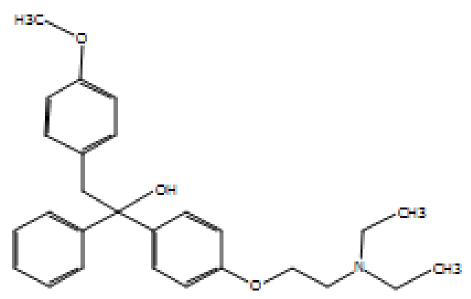

Compound Label	*m/z*	RT	Algorithm
Compound 47	329.2417	10.07	Auto MS/MS

MS Spectrum

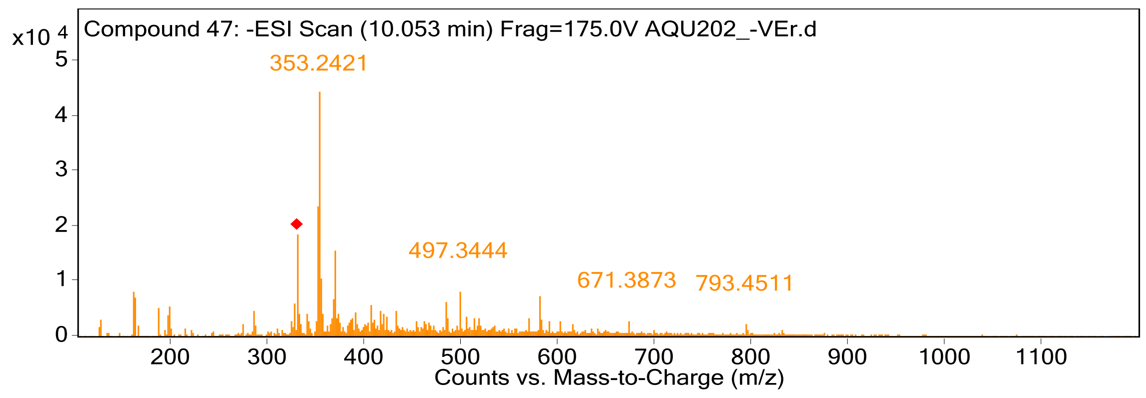

MS Zoomed Spectrum

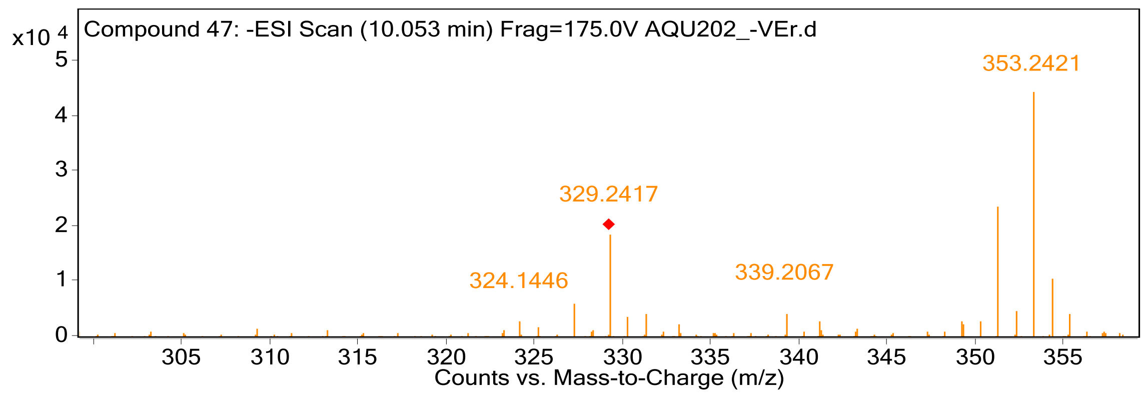

MS Spectrum Peak List

m/z	z	Abund
160.8468		8227.24
329.2417	1	18570.04
330.2454	1	3753.56
351.226	1	23795.34
353.2421	1	44533.7
354.2453	1	10773.25
368.1333	1	10607.82
369.2373	1	15701.99
497.3444	1	8277
580.3843	1	7321.69

MSMS Spectrum

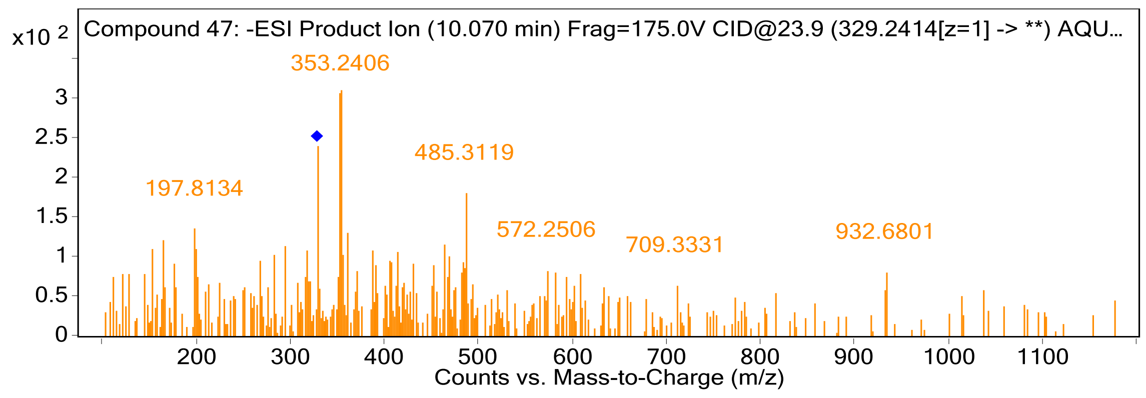

MS/MS Spectrum Peak List

m/z	z	Abund
165.0334		122
197.8134		135.98
199.8119		111.85
293.2268		115.34
329.2415		240.23
351.2267	1	307.04
353.2406	1	309.92
359.206		130.98
463.1979		116
485.3119		181.32

Compound Label	Name	*m/z*	RT	Algorithm	Mass
Cpd 48: Cholestane-3,7,12,24,25-pentol	**Cholestane-3,7,12,24,25-pentol**	495.3657	10.125	Auto MS/MS	450.3456

MS Spectrum

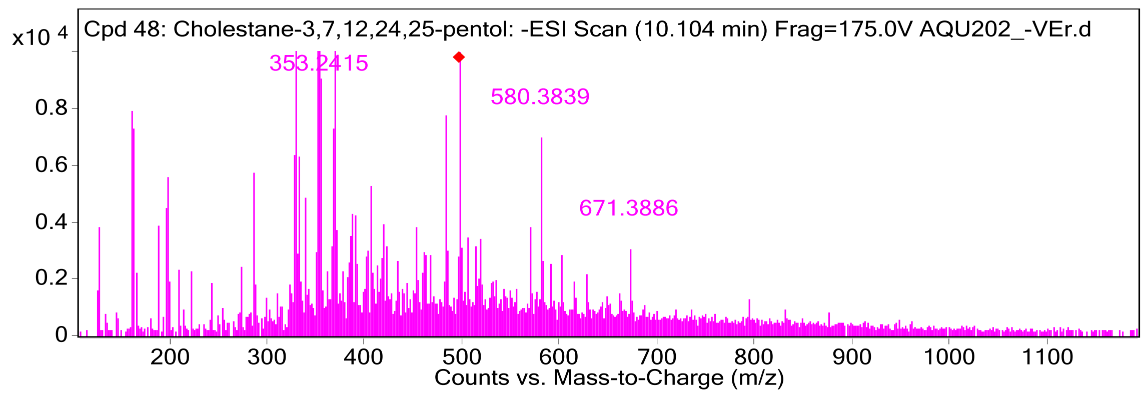

MS Zoomed Spectrum

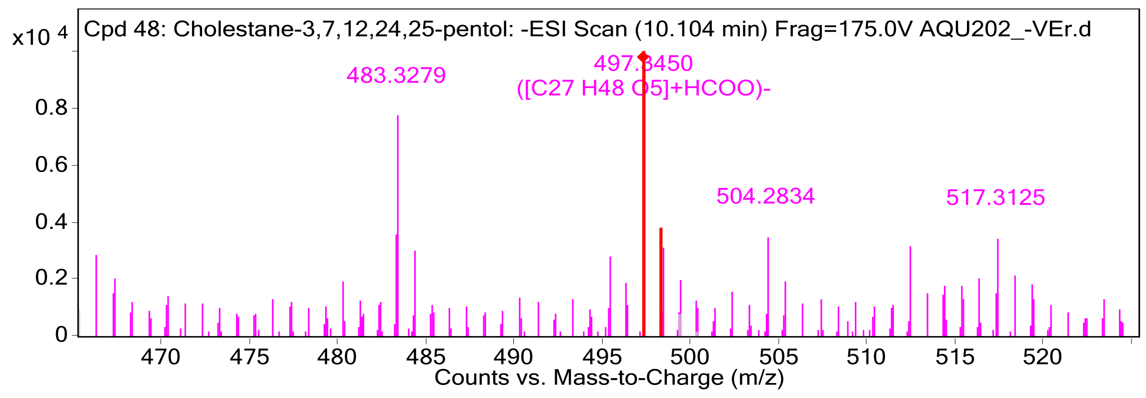

MS Spectrum Peak List

m/z	*Calc m/z*	Diff(ppm)	z	Abund	Formula	Ion
160.8467				7969.74		
329.2414			1	16859.14		
351.2261			1	25756.52		
353.2415			1	40044.52		
354.2447			1	9118.69		
369.2364			1	14772.33		
495.3657	497.3484	4002.5		2852.08	C27 H48 O5	(M+HCOO)-
496.3656	498.3518	4001.56	1	677.75	C27 H48 O5	(M+HCOO)-
497.345	499.3546	4040.66	1	10025.13	C27 H48 O5	(M+HCOO)-
498.3448	500.3573	4038.38	1	3160.44	C27 H48 O5	(M+HCOO)-

MSMS Spectrum

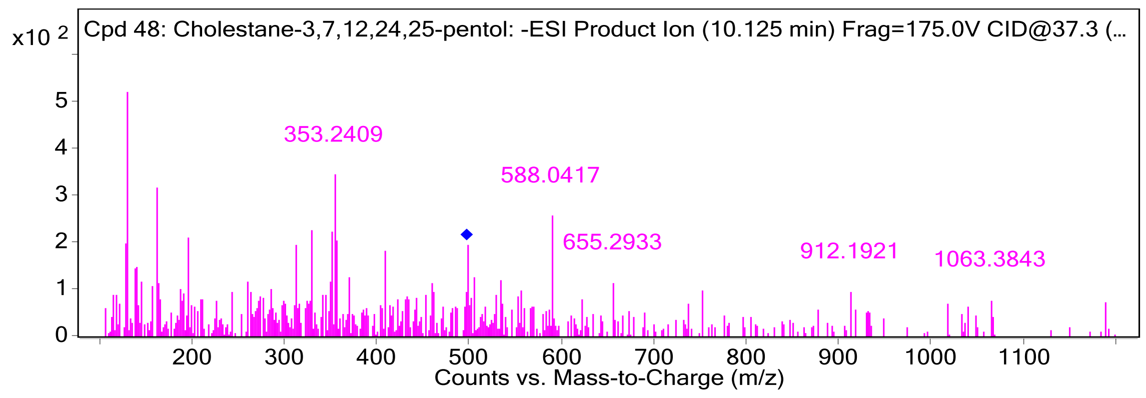

MS/MS Spectrum Peak List

m/z	Abund
126.9088	199.63
130.0912	523.43
160.8458	319.47
195.8151	211.32
312.2709	198.16
329.2423	228.01
351.226	226.25
353.2409	345.76
355.2561	205.57
588.0417	258.68

Compound Structure

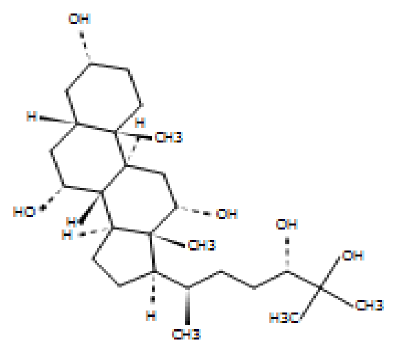

Compound Label	Name	*m/z*	RT	Algorithm	Mass
Cpd 49: 1,1'-[1,12-Dodecanediylbis(oxy)]bis benzene	**1,1'-[1,12-Dodecanediylbis(oxy)] bisbenzene**	353.2421	10.266	Auto MS/MS	354.2493

MS Spectrum

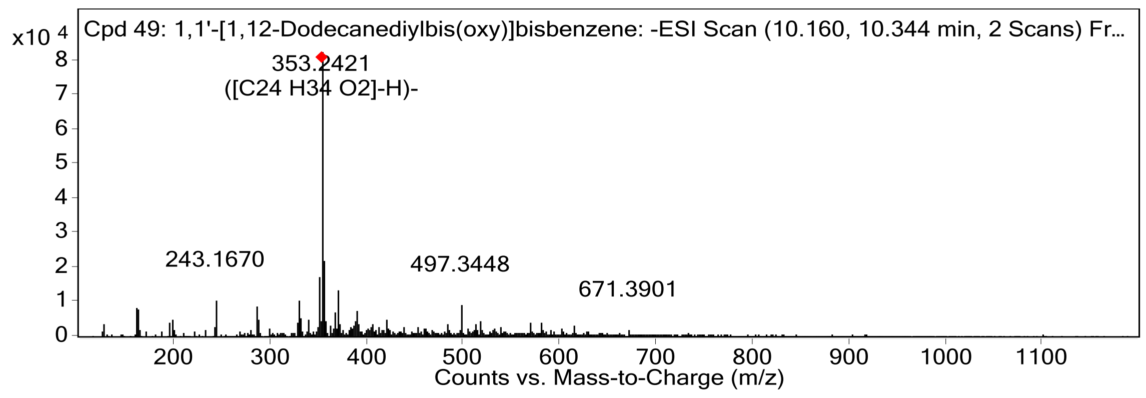

MS Zoomed Spectrum

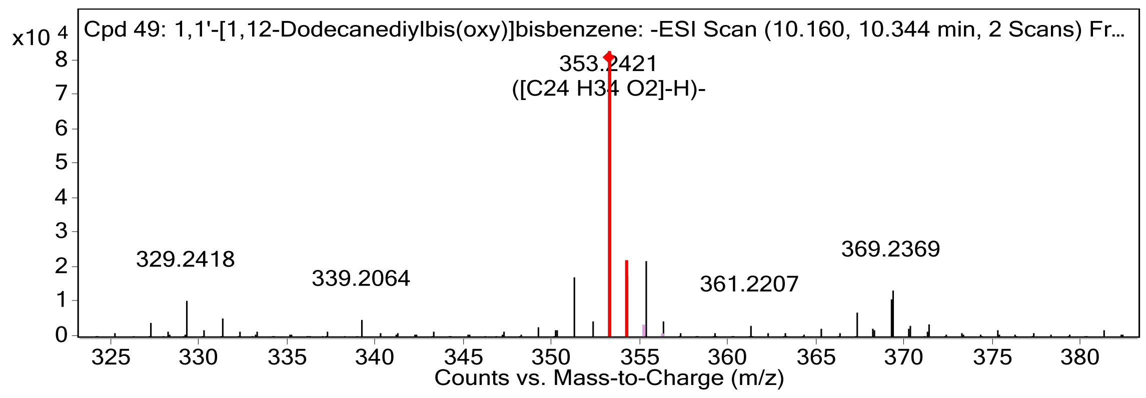

MS Spectrum Peak List

m/z	*Calc m/z*	Diff(ppm)	z	Abund	Formula	Ion
243.167				10696.02		
285.2148				8807.41		
329.2418			1	10740.62		
351.2261			1	17588.89		
353.2421	353.2486	18.5	1	82473.28	C24 H34 O2	(M-H)-
354.2452	354.252	19.1	1	17817.25	C24 H34 O2	(M-H)-
355.2572			1	22152.02		

369.1774			1	11220.95		
369.2369			1	13702.06		
497.3448			1	9294.3		

MSMS Spectrum

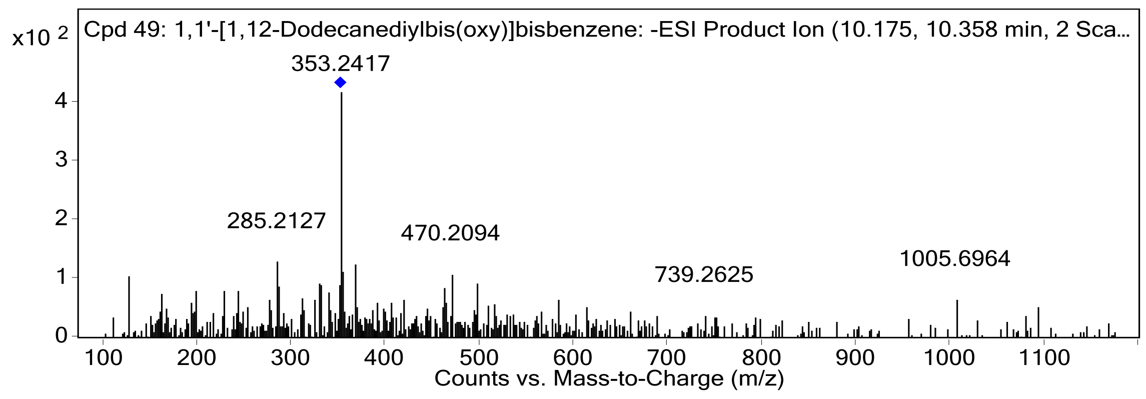

MS/MS Spectrum Peak List

m/z	z	Abund
127.0829		106.04
285.2127		129.84
329.2397		92.35
331.2546		89.45
351.226		90.54
353.2417	1	419.21
355.2556	1	112.11
367.219	1	126.3
470.2094		108.99
497.3416		91.86

Compound Structure

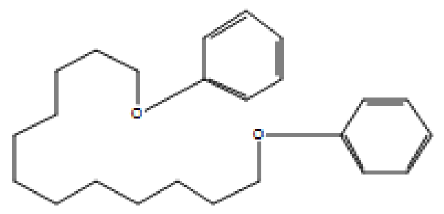

**Compound Label**	**Name**	***m/z***	**RT**	**Algorithm**	**Mass**
Cpd 50: Androsterone sulfate	**Androsterone sulfate**	369.1777	10.426	Auto MS/MS	370.1854

MS Spectrum

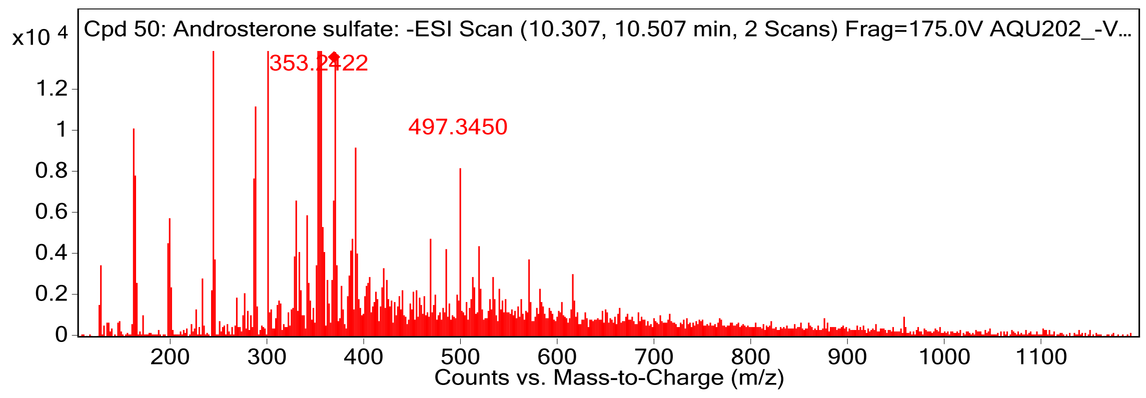

MS Zoomed Spectrum

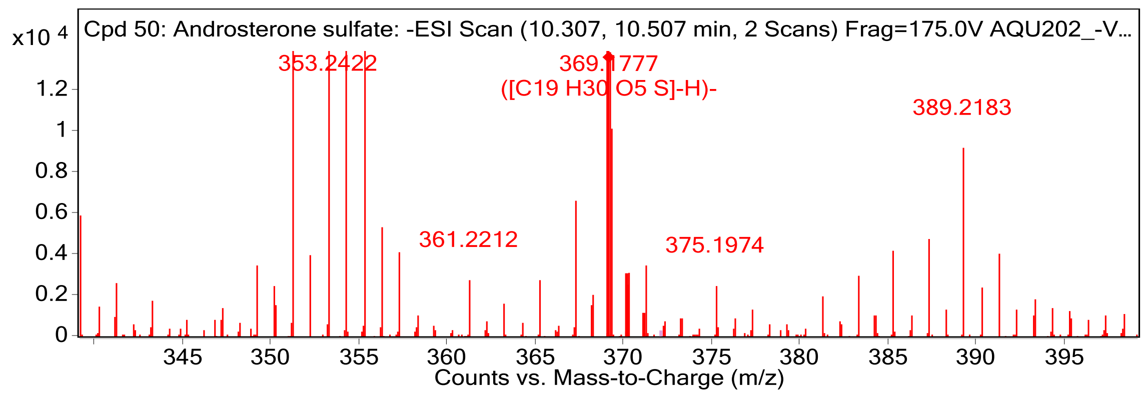

MS Spectrum Peak List

m/z	*Calc m/z*	Diff(ppm)	z	Abund	Formula	Ion
243.1669			1	24886.42		
287.1942				11222.46		
299.1941			1	14685.08		
351.2263			1	15310.42		
353.2422			1	93170.58		
354.2453			1	20361.29		
355.2573			1	24219.88		
369.1777	369.1741	-9.77	1	13860.4	C19 H30 O5 S	(M-H)-
370.182	370.1774	-12.44	1	2556.39	C19 H30 O5 S	(M-H)-
371.1812	371.1742	-18.99	1	1105.24	C19 H30 O5 S	(M-H)-

MSMS Spectrum

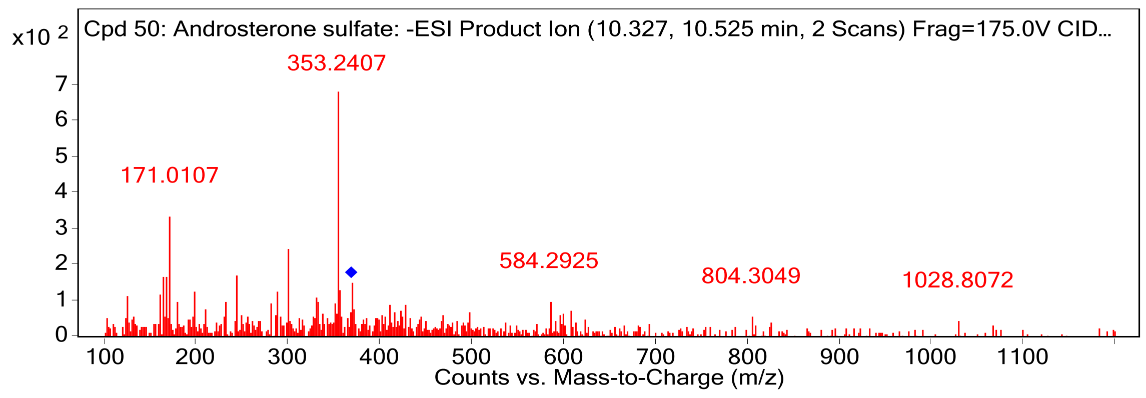

MS/MS Spectrum Peak List

m/z	z	Abund
162.8443		169.14
167.0661	1	168.01
171.0107		337.4
243.1673		173.27

287.2275		125.54
299.1928		245.32
353.2407	1	684.73
354.2463	1	160.05
355.256		130.73
369.2374		151.14

Compound Structure

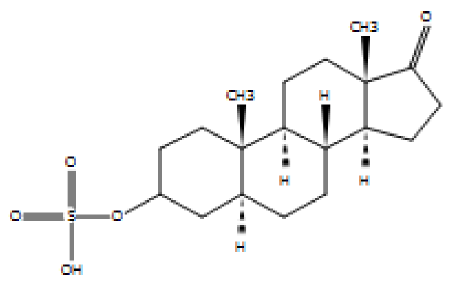

Compound Label	Name	*m/z*	RT	Algorithm	Mass
Cpd 51: 5beta-Cholestane-3alpha,7alpha,12alpha,25,26-pentol	**5beta-Cholestane-3alpha,7alpha,12alpha,25,26-pentol**	497.3451	10.549	Auto MS/MS	452.3469

MS Spectrum

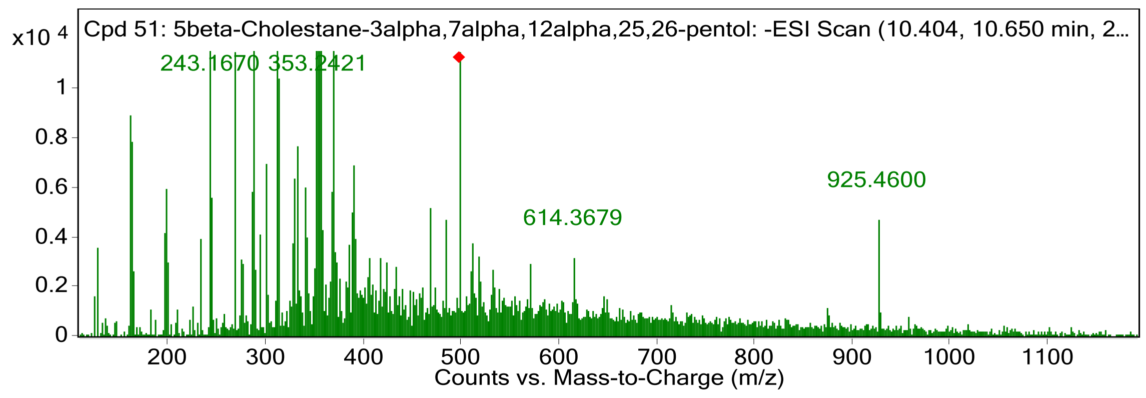

MS Zoomed Spectrum

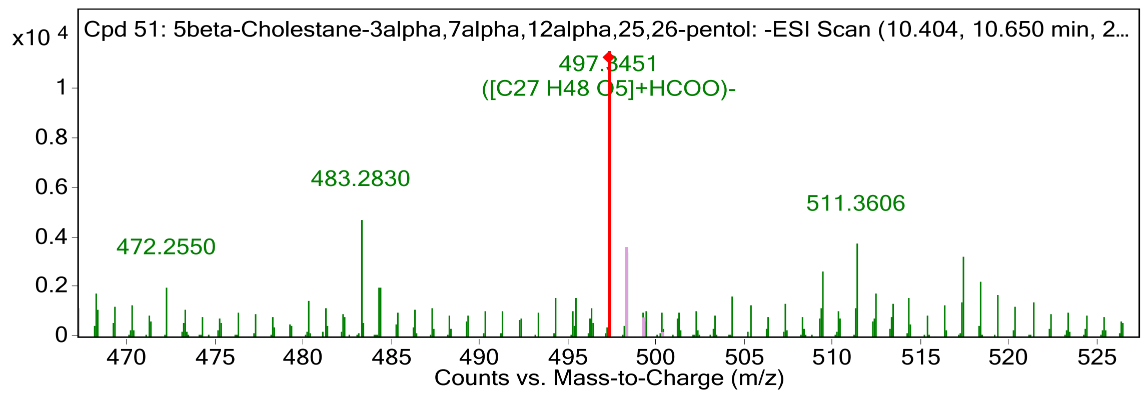

MS Spectrum Peak List

m/z	*Calc m/z*	Diff(ppm)	z	Abund	Formula	Ion
243.167			1	46511.84		
267.2033			1	11503.29		
287.1939			1	16683.54		
311.1943			1	61995.18		
351.2262			1	12040.07		
353.2421			1	66492.01		
354.2453			1	13836.85		
355.2574			1	20765.01		
369.1775			1	16486.76		
497.3451	497.3484	6.63	1	11508.02	C27 H48 O5	(M+HCOO)-

MSMS Spectrum

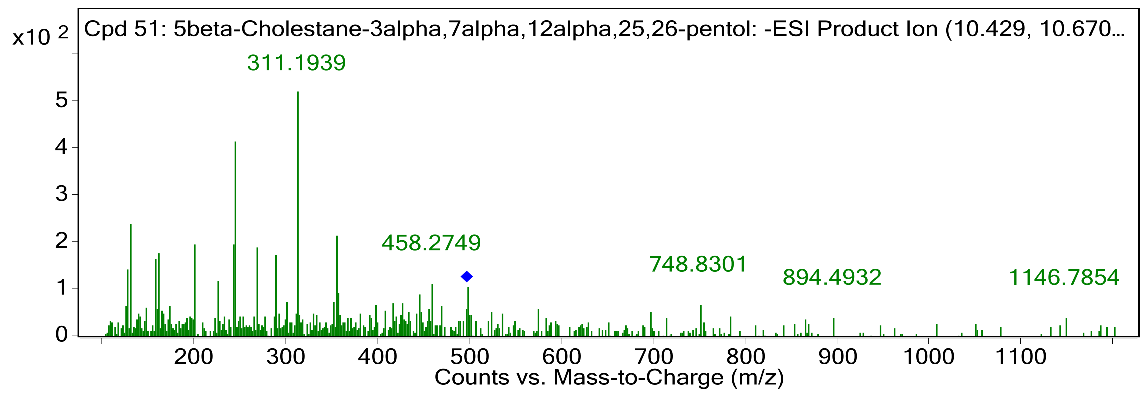

MS/MS Spectrum Peak List

m/z	z	Abund
129.1069		183.18
130.0915		239.52
160.8469		178.23
199.1881	1	196.23
241.1882	1	195.23
243.1695		416.16
267.2025		189.9
287.1925		174.03
311.1939	1	522.06
353.2423		214.73

Compound Structure

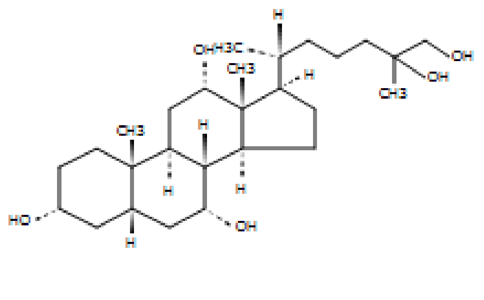

Compound Label	*m/z*	RT	Algorithm
Compound 52	267.2038	10.625	Auto MS/MS

MS Spectrum

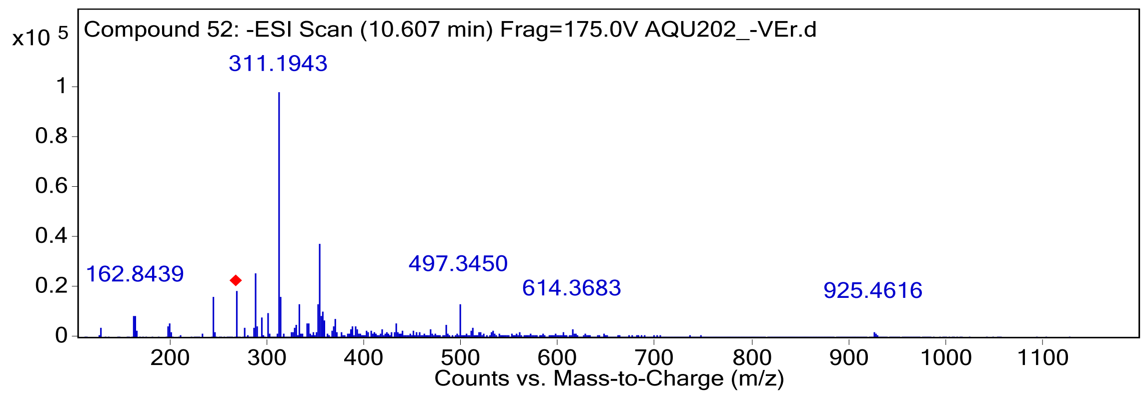

MS Zoomed Spectrum

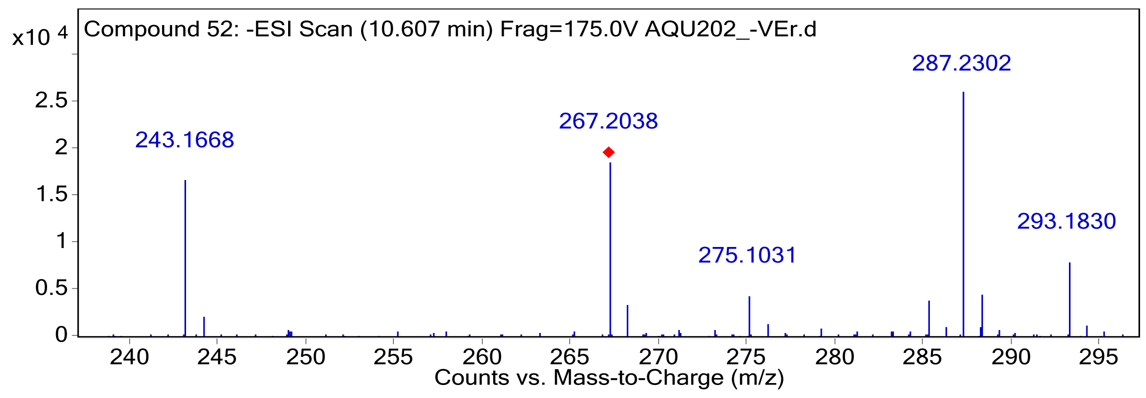

MS Spectrum Peak List

m/z	z	Abund
243.1668	1	16667.51
267.2038	1	18674.65
268.2063	1	3415.7
269.2119	1	513.79
287.2302	1	26163.14
311.1943	1	98382.02
312.1976	1	16413.2
331.2566	1	13532.02
351.2262	1	13451
353.2416	1	37806.34

MSMS Spectrum

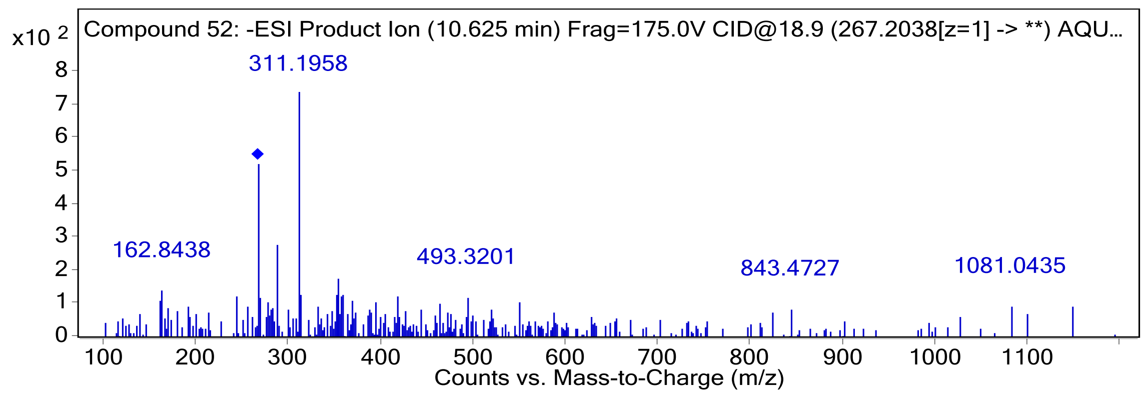

MS/MS Spectrum Peak List

m/z	z	Abund
162.8438		140.39
267.2036	1	520.05
287.2297		278.1
311.1958	1	739.98
313.247		130
352.1093		129.78
353.2425	1	175.31
357.2714		123.43
359.1926		128.14
417.2018		125

Compound Label	Name	*m/z*	RT	Algorithm	Mass
Cpd 53: Propoxycaine	**Propoxycaine**	293.1838	10.866	Auto MS/MS	294.1911

MS Spectrum

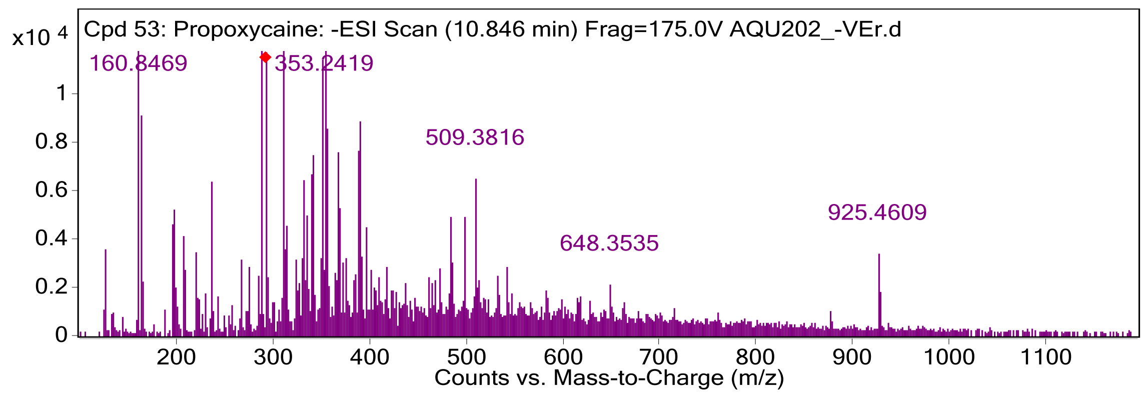

MS Zoomed Spectrum

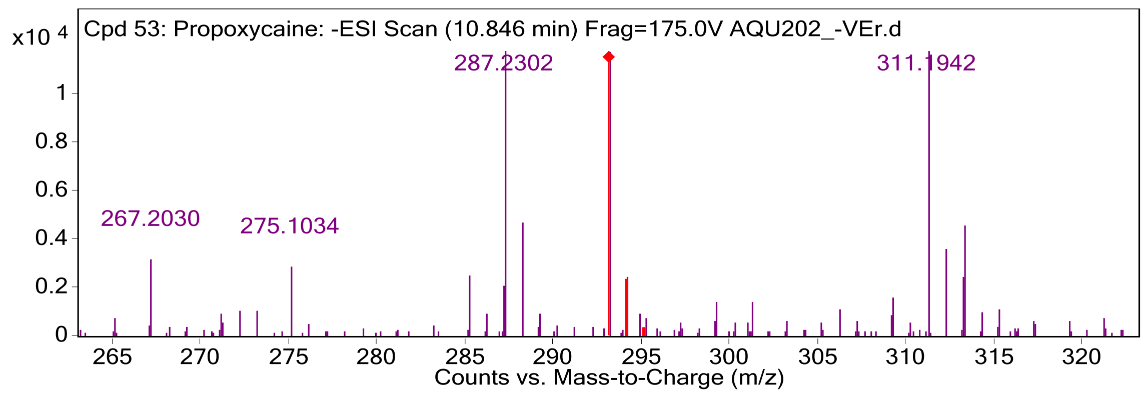

MS Spectrum Peak List

m/z	*Calc m/z*	Diff(ppm)	z	Abund	Formula	Ion
160.8469				12159.82		
162.8437				9135.3		
287.2302			1	25639.26		
293.1838	293.1871	11.11	1	11749.06	C16 H26 N2 O3	(M-H)-
294.1859	294.1902	14.61	1	2471.39	C16 H26 N2 O3	(M-H)-

295.1934	295.1928	-2.09	1	772.67	C16 H26 N2 O3	(M-H)-
311.1942			1	19387.21		
351.2263			1	11466.11		
353.2419			1	76137.5		
354.2452			1	16144.39		

MSMS Spectrum

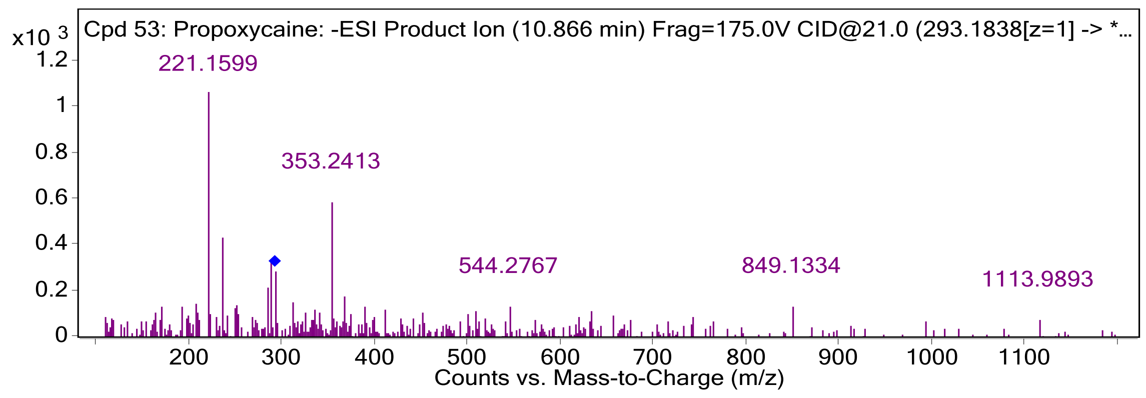

MS/MS Spectrum Peak List

m/z	z	Abund
220.1524		621.56
221.1599	1	1068.67
236.1111		436.46
283.9702		214.67
287.2292	1	326.89
293.1837	1	289.16
353.2413	1	589.71
354.2439	1	223.33
367.2212		181.85
367.2361		173.77

Compound Structure

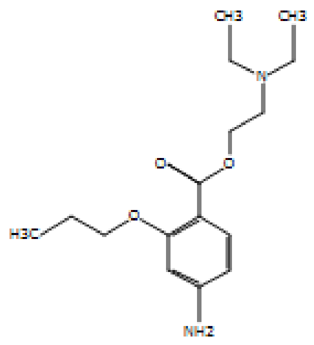

Compound Label	*m/z*	RT	Algorithm
Compound 54	355.2571	11.152	Auto MS/MS

MS Spectrum

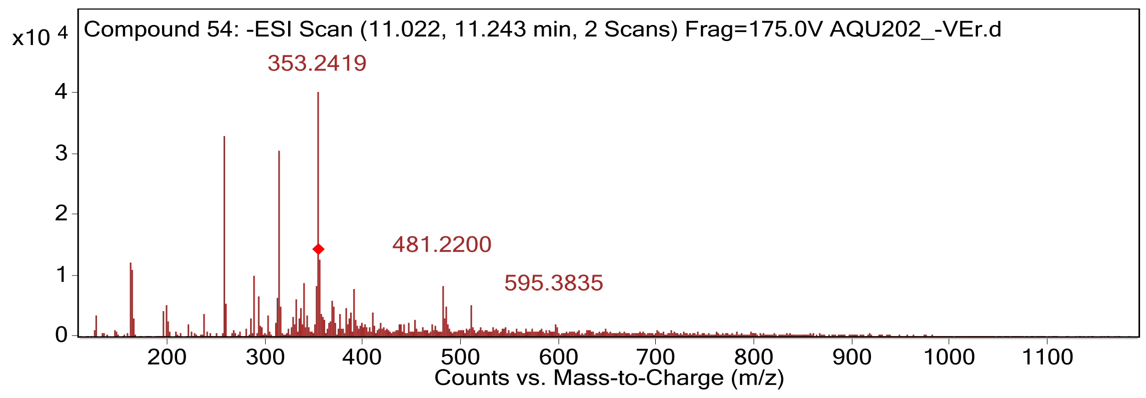

MS Zoomed Spectrum

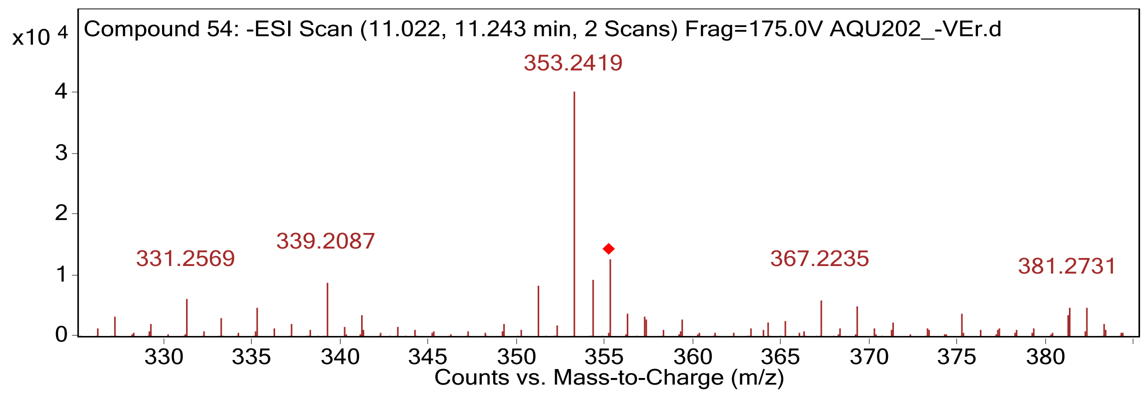

MS Spectrum Peak List

m/z	z	Abund
160.8468		12230.73
162.8439		11121.7
257.1829	1	33135.73
287.2301		10230.31
313.2101	1	30691.11
339.2087	1	9025.32
353.2419	1	40346.45
354.2452	1	9317.08
355.2571	1	12801.93
356.2568	1	3933.78

MSMS Spectrum

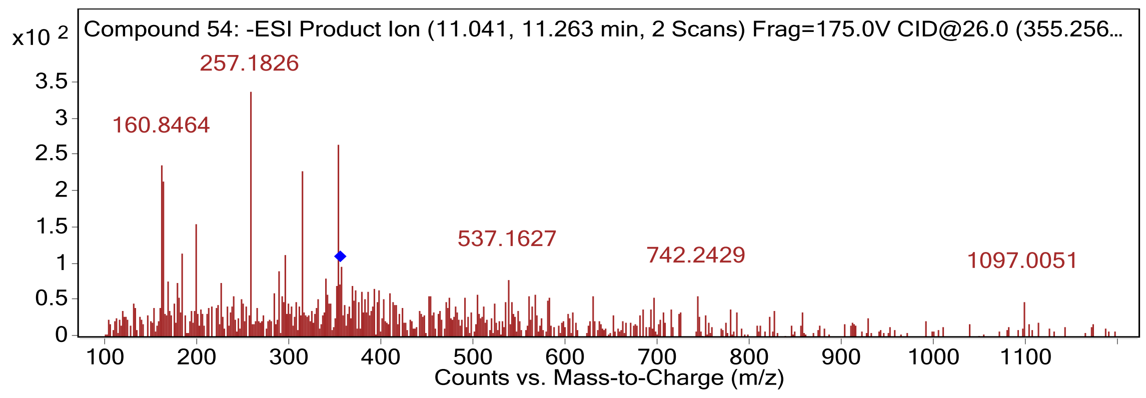

MS/MS Spectrum Peak List

m/z	z	Abund
160.8464		236.05
162.842		215.22
183.1076		115.32

197.8127		155.52
257.1826	1	338.29
287.2281		90.4
294.2512		113.08
313.2113	1	227.85
353.2437	1	265.68
355.26		96.83

Compound Label	Name	*m/z*	RT	Algorithm	Mass
Cpd 55: Aspidospermine	**Aspidospermine**	353.2239	11.791	Auto MS/MS	354.2312

MS Spectrum

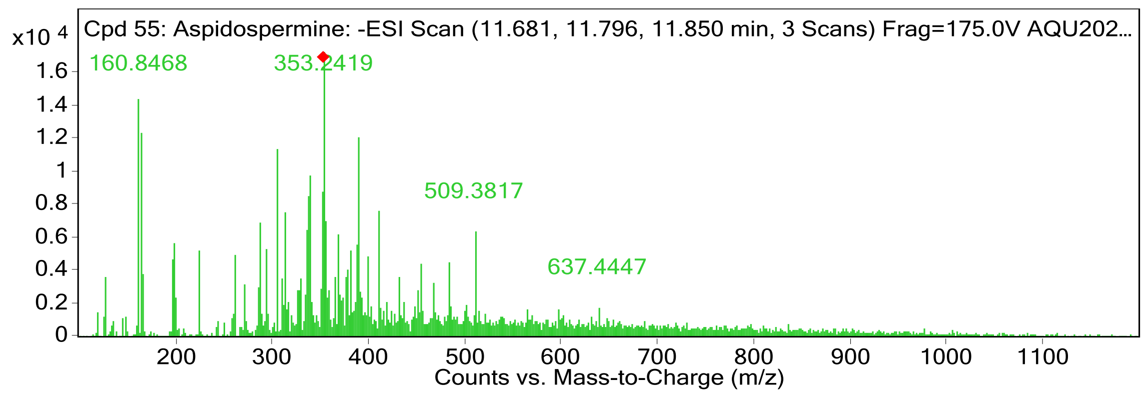

MS Zoomed Spectrum

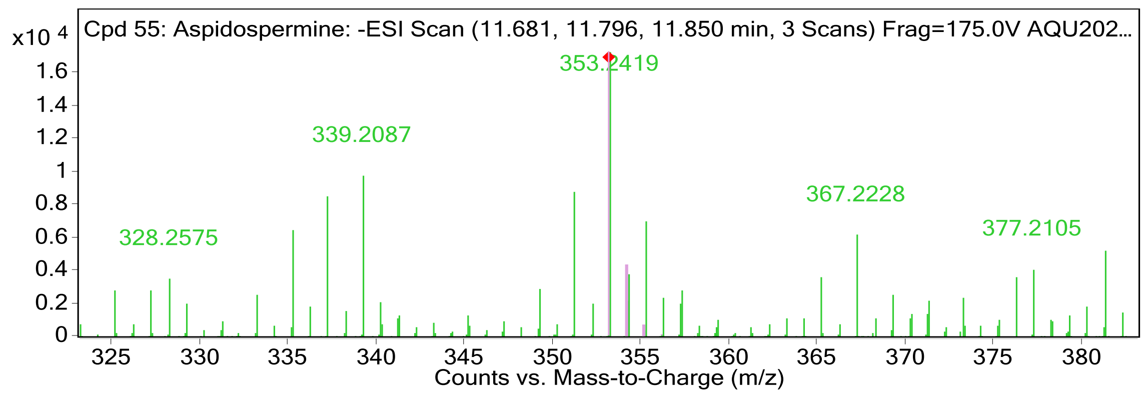

MS Spectrum Peak List

m/z	*Calc m/z*	Diff(ppm)	z	Abund
160.8468				14377.17
162.844				12385.56
304.1631			1	11416.94
337.2464			1	8508.4
339.2087			1	9816.41
351.2261			1	8826.87
353.2058			1	9278.93
353.2419	354.2313	2800.82	1	17261.53
389.2179			1	12132.96
409.2457			1	7687.77

MSMS Spectrum

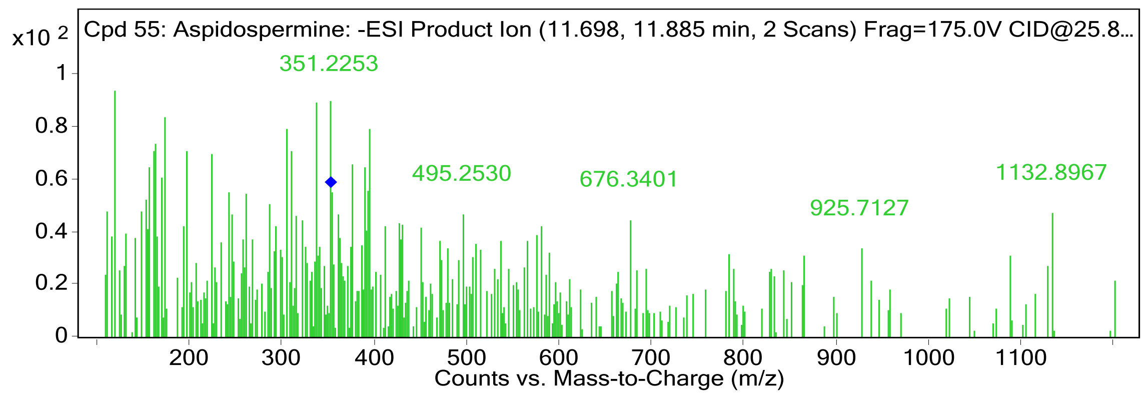

MSMS Spectrum

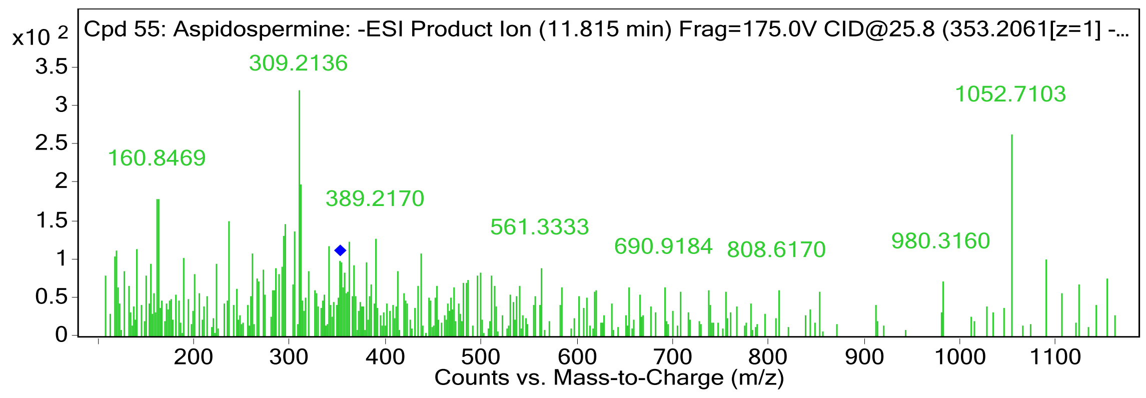

MS/MS Spectrum Peak List

m/z	Abund
118.0682	93.96
160.8462	71.19
162.8436	73.9
172.9641	84
195.8149	71.31
304.1644	79.72
309.2117	71.13
337.2466	89.64
351.2253	90.12
393.2398	79.6
160.8469	180.17
162.8437	179.97
235.1933	152.02
293.1802	132.77
294.1878	148
304.1617	137.19
309.2136	320.44
310.217	198.07
389.217	127.24
1052.7103	263.75

Compound Structure

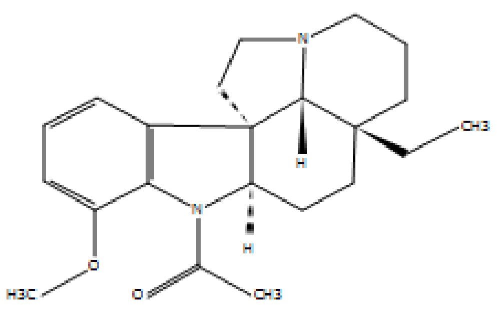

Compound Label	*m/z*	RT	Algorithm
Compound 56	327.2258	12.017	Auto MS/MS

MS Spectrum

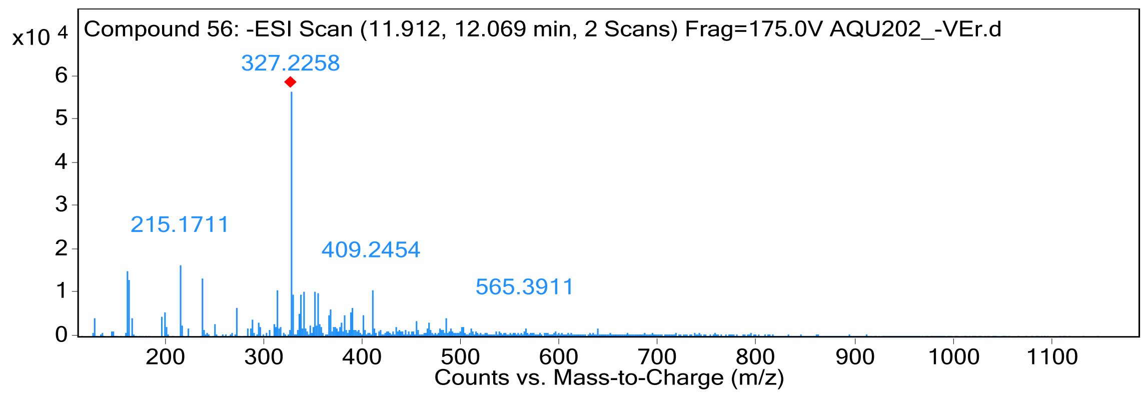

MS Zoomed Spectrum

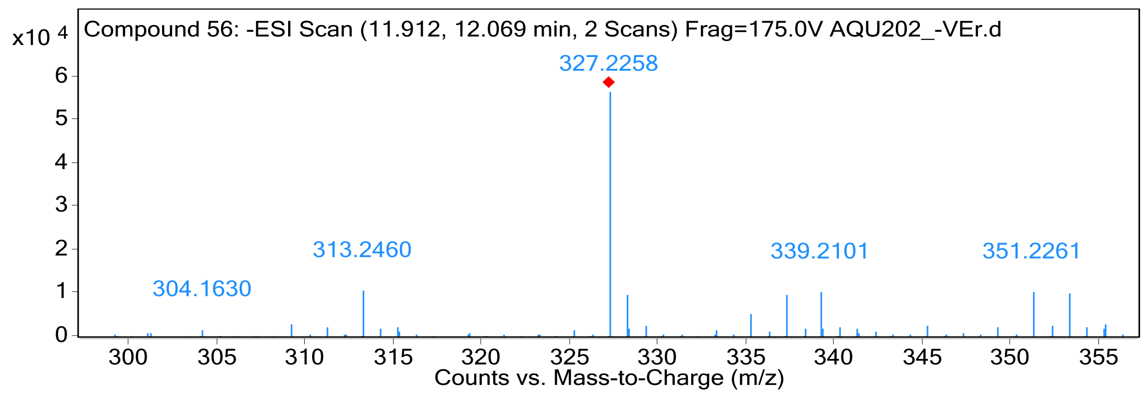

MS Spectrum Peak List

m/z	z	Abund
160.8467		15096.34
162.8439		13081.9
215.1711	1	16528.83
237.1229	1	13632.9
313.246	1	10764.63
327.2258	1	56613.71
328.2294	1	9873.88
329.2369	1	2618.59
330.2448	1	672.49
409.2454	1	10687.21

MSMS Spectrum

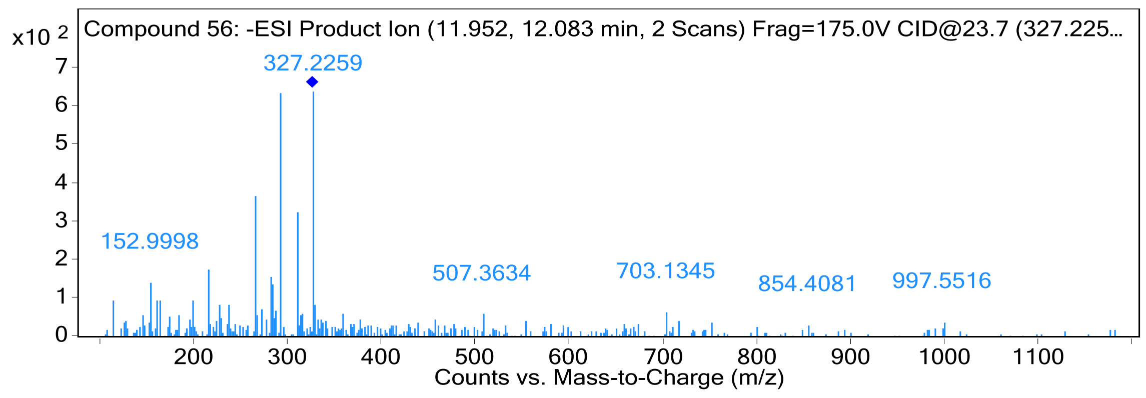

MS/MS Spectrum Peak List

m/z	z	Abund
152.9998		143.03
162.8439		97.25
215.1695	1	176.43
265.2234	1	367.45
281.2177		156.53
283.2355	1	137.24
291.2037	1	636.69
292.2069	1	124.96
309.2149	1	323.91
327.2259	1	640.68

Compound Label	*m/z*	RT	Algorithm
Compound 57	215.1709	12.171	Auto MS/MS

MS Spectrum

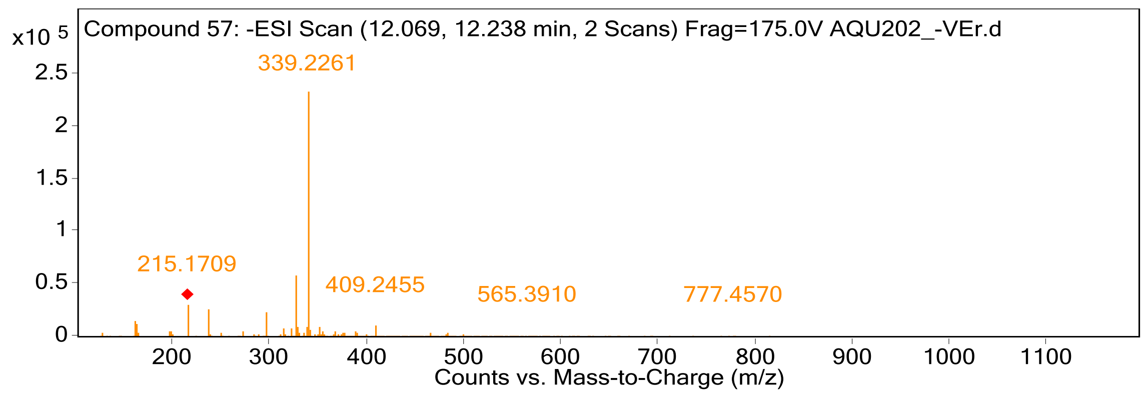

MS Zoomed Spectrum

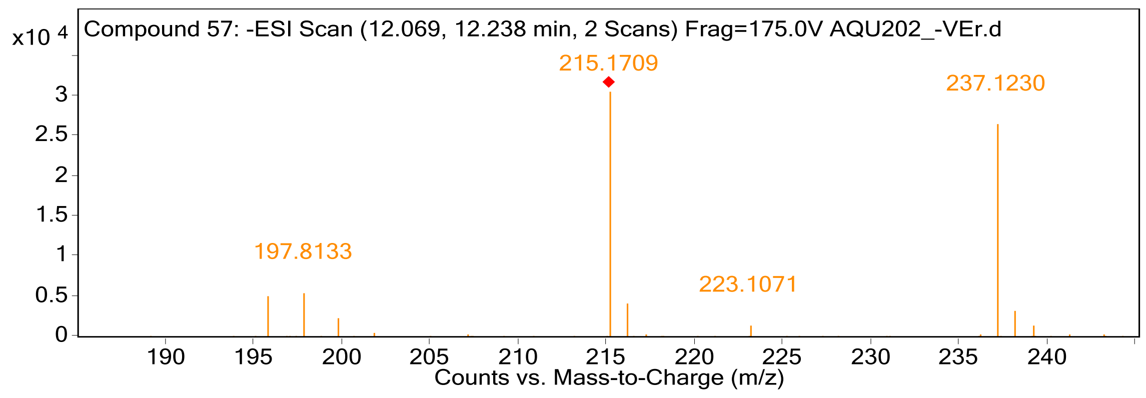

MS Spectrum Peak List

m/z	z	Abund
160.8468		14942.04
162.8439		13211.77
215.1709	1	30588.19
216.174	1	4147.2
217.1779	1	396.08
237.123	1	26579.52
295.2352	1	23449.01
327.2257	1	59119.41
339.2261	1	233811.06
340.2292	1	40164.2

MSMS Spectrum

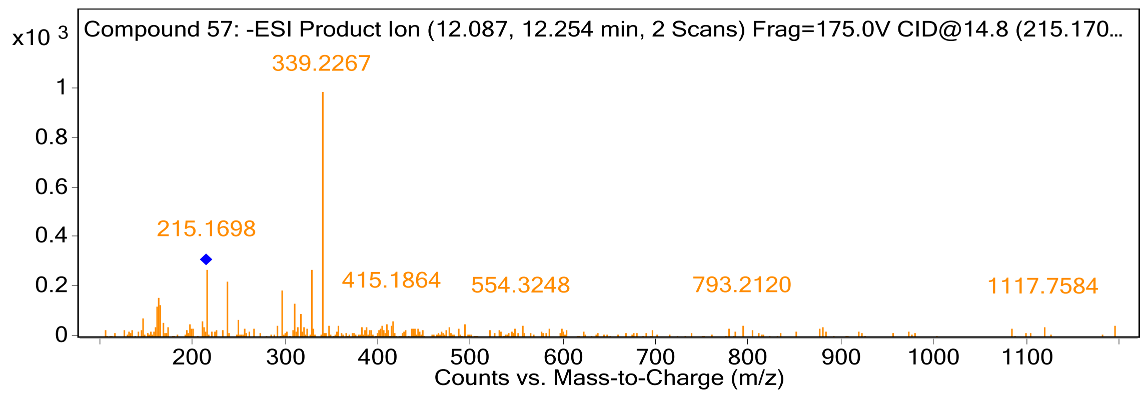

MS/MS Spectrum Peak List

m/z	z	Abund
160.8468		124.81
162.8435		161.83
164.8414		127.78
215.1698		272.44
237.1234		223.06
295.235	1	188.79
309.2127		134.3
327.2261	1	274.06
339.2267	1	988.78
340.2291	1	227.17

Compound Label	Name	*m/z*	RT	Algorithm	Mass
Cpd 58: Phlegmarine	**Phlegmarine**	295.2351	12.27	Auto MS/MS	250.237

MS Spectrum

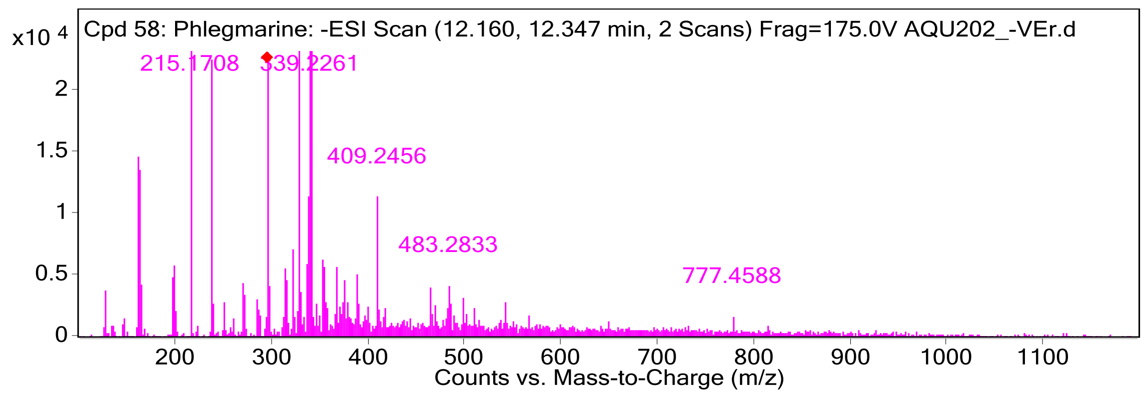

MS Zoomed Spectrum

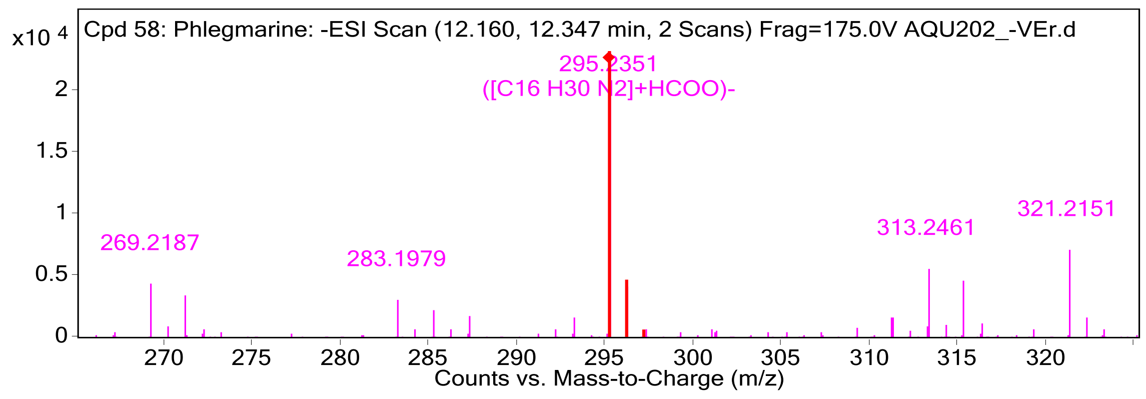

MS Spectrum Peak List

m/z	*Calc m/z*	Diff(ppm)	z	Abund	Formula	Ion
160.8467				14632.33		
162.8437				13508.89		
215.1708			1	36522.03		
237.1229			1	22501.65		
295.2351	295.2391	13.5	1	23085.99	C16 H30 N2	(M+HCOO)-
296.2385	296.2423	12.85	1	4113.68	C16 H30 N2	(M+HCOO)-
297.2428	297.2451	7.55	1	713.78	C16 H30 N2	(M+HCOO)-
327.2254			1	24051.07		
339.2261			1	200931.03		
340.2292			1	35615.64		

MSMS Spectrum

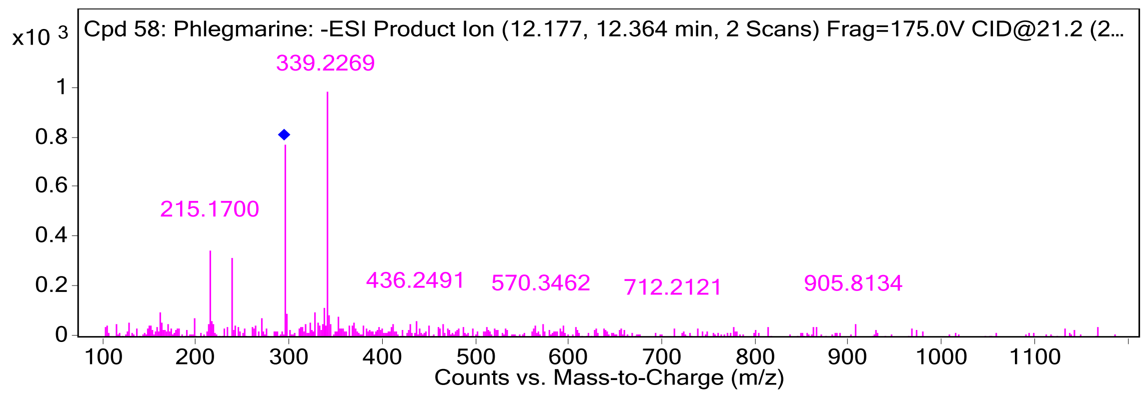

MS/MS Spectrum Peak List

m/z	z	Abund
160.8465		100.97
215.17	1	350.68
237.1233		319.61
295.2348	1	771.33
296.2389	1	92.71
327.2263		99.44
337.2461		119.39
339.2269	1	988.34
340.2315	1	161.2
342.2734		87.35

Compound Structure

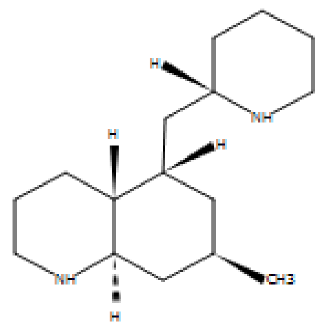

Compound Label	Name	*m/z*	RT	Algorithm	Mass
Cpd 59: alpha-Tocopherolquinone	**alpha-Tocopherolquinone**	491.373	12.81	Auto MS/MS	446.3726

MS Spectrum

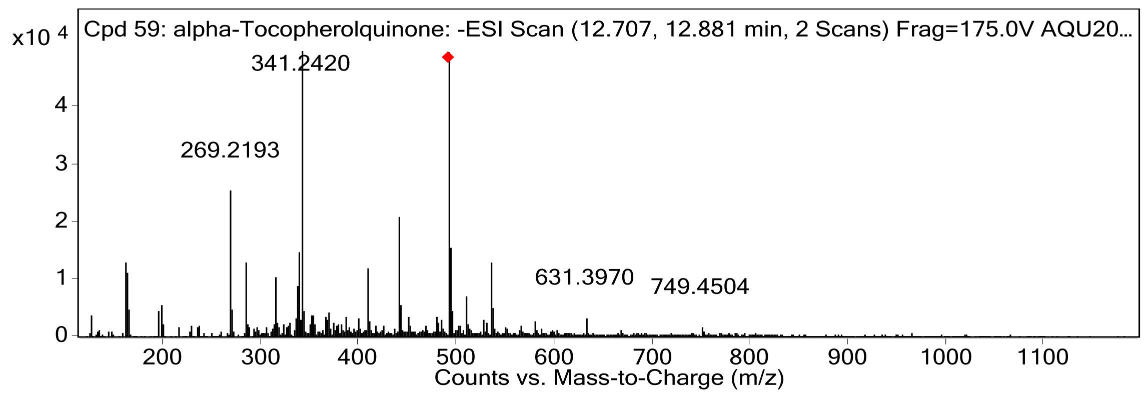

MS Zoomed Spectrum

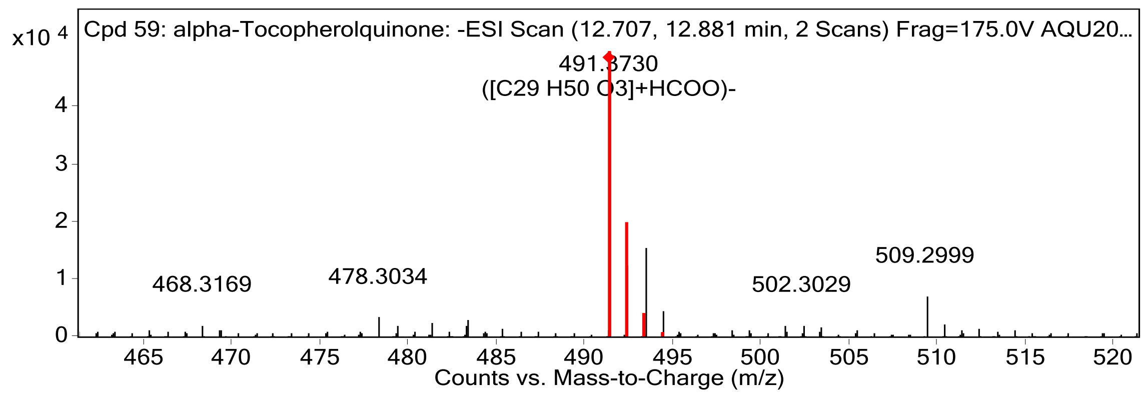

MS Spectrum Peak List

m/z	*Calc m/z*	Diff(ppm)	z	Abund	Formula	Ion
269.2193			1	25467.71		
339.2229			1	14728.67		
341.242			1	91319.79		
342.2453			1	18839.21		
441.3795			1	20965.73		
491.373	491.3742	2.45	1	49537.79	C29 H50 O3	(M+HCOO)-
492.3764	492.3776	2.55	1	12627.36	C29 H50 O3	(M+HCOO)-
493.3709	493.3806	19.69	1	15493.05	C29 H50 O3	(M+HCOO)-
494.3735	494.3835	20.12	1	4629.34	C29 H50 O3	(M+HCOO)-
495.3751	495.3862	22.49	1	689.05	C29 H50 O3	(M+HCOO)-

MSMS Spectrum

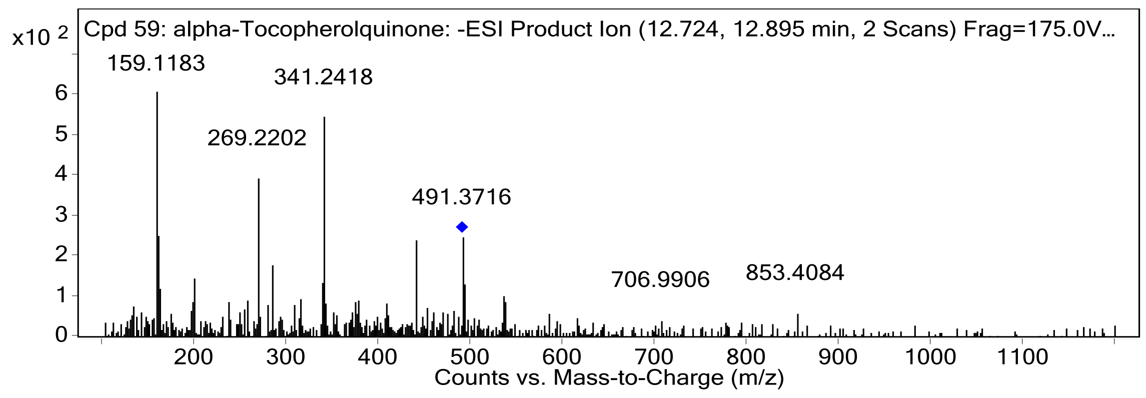

MS/MS Spectrum Peak List

m/z	z	Abund
159.1183		609.18
160.8456		252.61
201.1285		144.17
269.2202	1	393.28
285.2135		179.81
339.224		133.2
341.2418	1	544.79
441.3789		239.04

491.3716			246.51
493.3706			131.13

Compound Structure

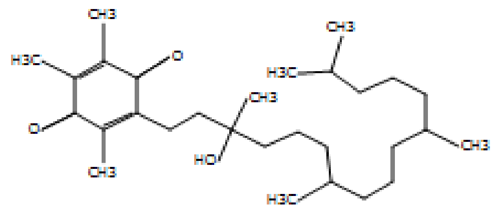

**Compound Label**	**Name**	***m/z***	**RT**	**Algorithm**	**Mass**
Cpd 60: Pithecolobine	**Pithecolobine**	441.3796	12.848	Auto MS/MS	382.3659

MS Spectrum

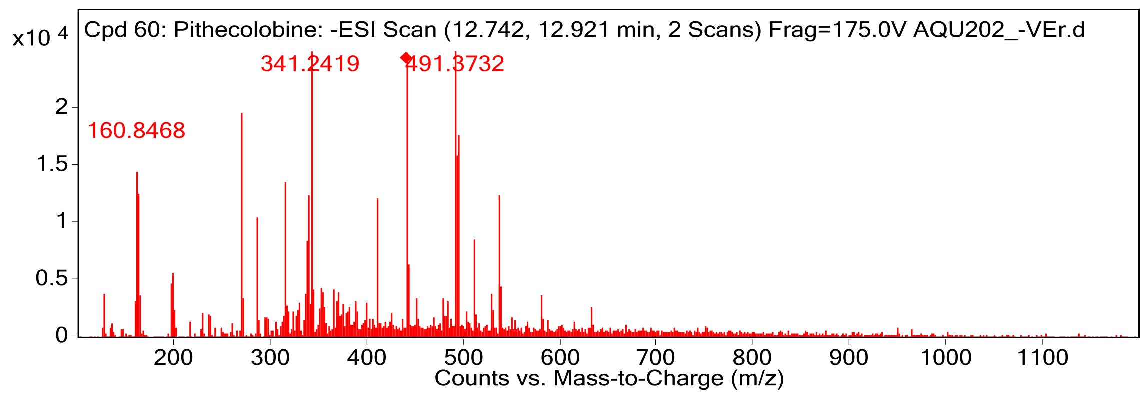

MS Zoomed Spectrum

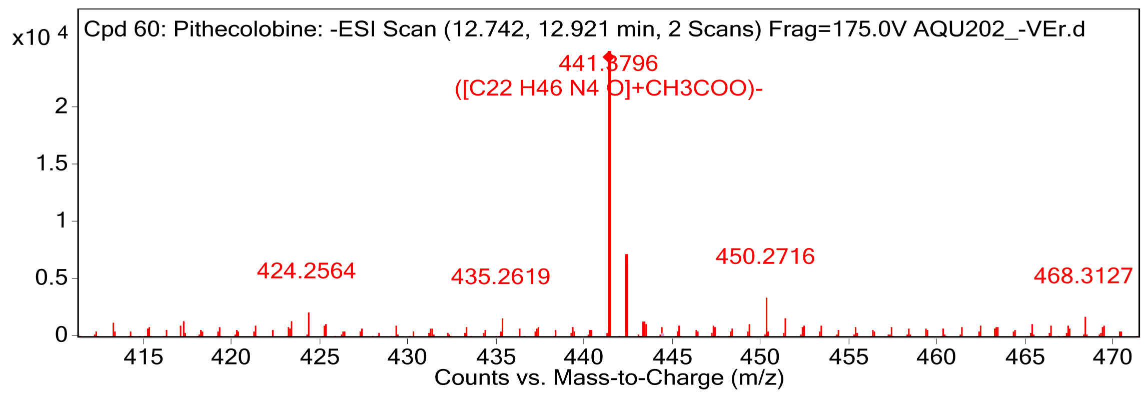

MS Spectrum Peak List

m/z	*Calc m/z*	Diff(ppm)	z	Abund	Formula	Ion
160.8468				14580.93		
269.2193			1	19683.95		
315.2617			1	13675.99		
341.2419			1	66836.82		
441.3796	441.381	3.18	1	24931.54	C22 H46 N4 O	(M+CH3COO)-
442.3833	442.3841	1.74	1	6415.96	C22 H46 N4 O	(M+CH3COO)-
443.3851	443.3869	4.07	1	1130.04	C22 H46 N4 O	(M+CH3COO)-
491.3732			1	54555.06		
492.3762			1	15964.29		
493.3709			1	17792.38		

MSMS Spectrum

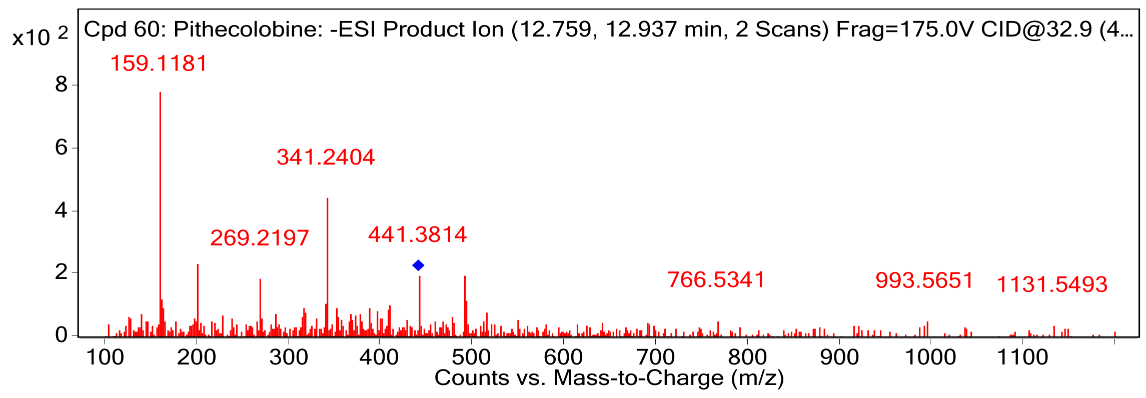

MS/MS Spectrum Peak List

m/z	z	Abund
159.1181	1	782.81
160.1204	1	140
160.8464		121.15
201.1264		234.19
269.2197	1	186.37
341.2404	1	444.51
342.245	1	125.1
441.3814		197.3
491.3708	1	195.75
493.3617	1	118.55

Compound Structure

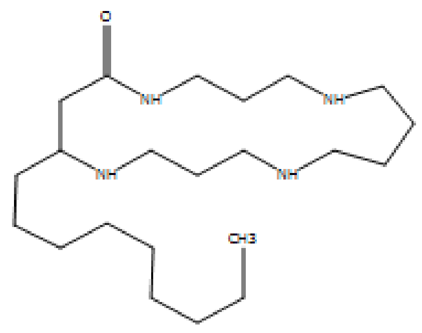

**Compound Label**	**Name**	***m/z***	**RT**	**Algorithm**	**Mass**
Cpd 61: alpha-Tocopherolquinone	**alpha-Tocopherolquinone**	491.3729	13.104	Auto MS/MS	446.3729

MS Spectrum

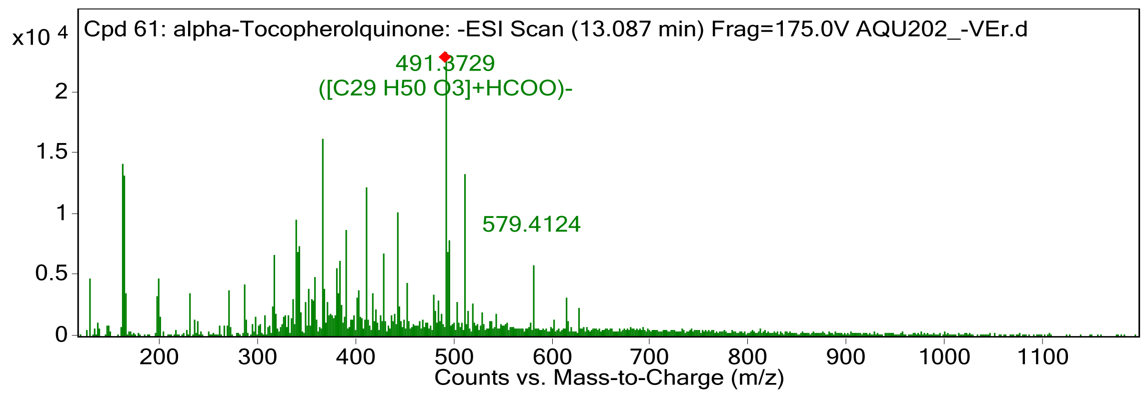

MS Zoomed Spectrum

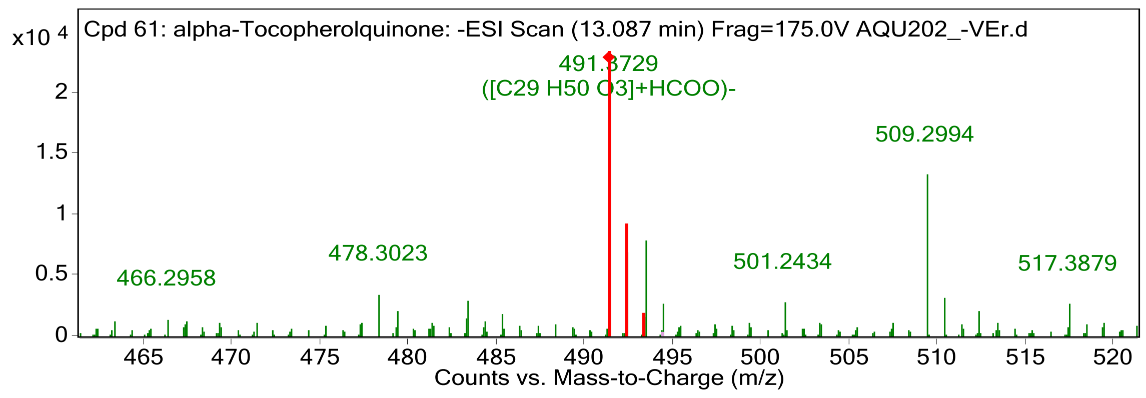

MS Spectrum Peak List

m/z	*Calc m/z*	Diff(ppm)	z	Abund	Formula	Ion
160.8467				14176.78		
162.8439				13195		
337.2467			1	9574.52		
365.2787			1	16240.11		
409.2458			1	12228.31		
441.38			1	10206.19		
491.3729	491.3742	2.7	1	23320.57	C29 H50 O3	(M+HCOO)-
492.3762	492.3776	2.96	1	6944.35	C29 H50 O3	(M+HCOO)-
493.371	493.3806	19.38	1	7966.95	C29 H50 O3	(M+HCOO)-
509.2994			1	13300.04		

MSMS Spectrum

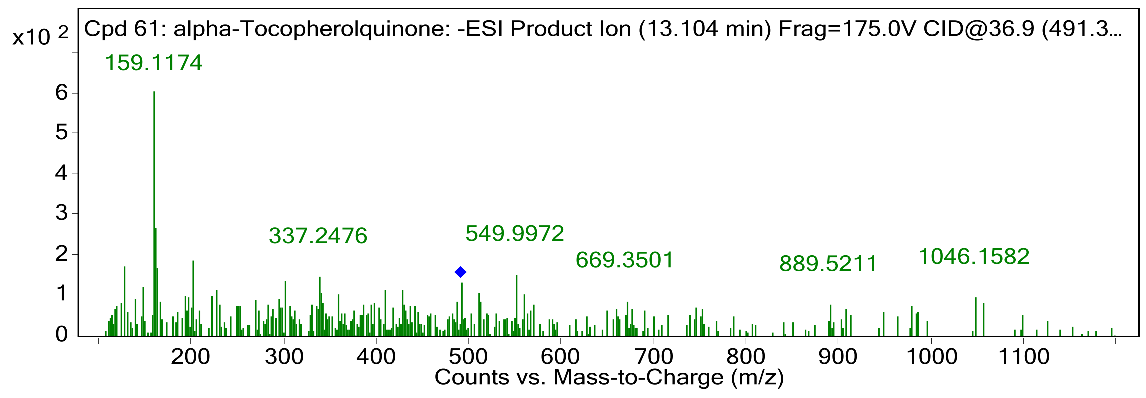

MS/MS Spectrum Peak List

m/z	Abund
126.9068	174.03
146.8722	123.11
159.1174	607.03
160.8466	268.56
162.8449	171.9
201.1306	187.58
301.1853	138.28
337.2476	148.31
491.3727	134
549.9972	154.05

Compound Structure

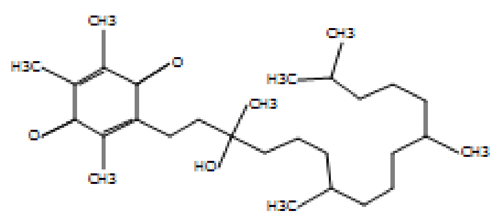

Compound Label	*m/z*	RT	Algorithm
Compound 62	426.9781	13.234	Auto MS/MS

MS Spectrum

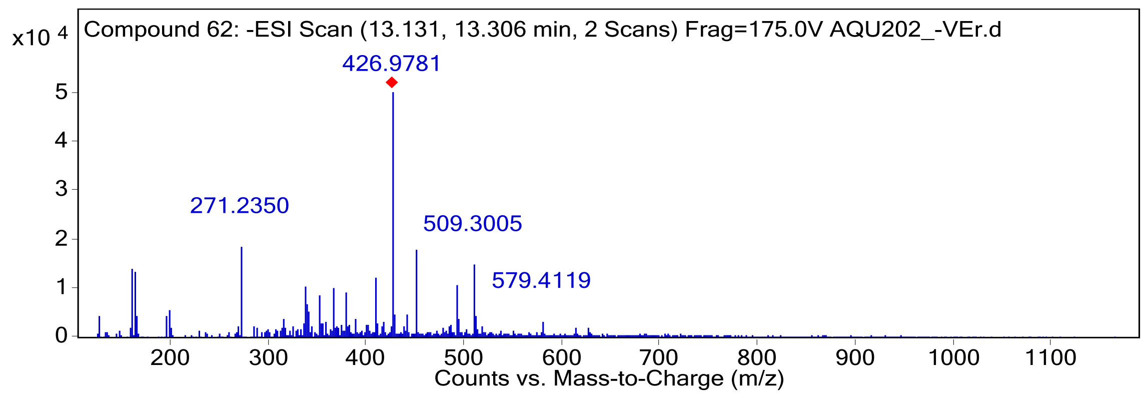

MS Zoomed Spectrum

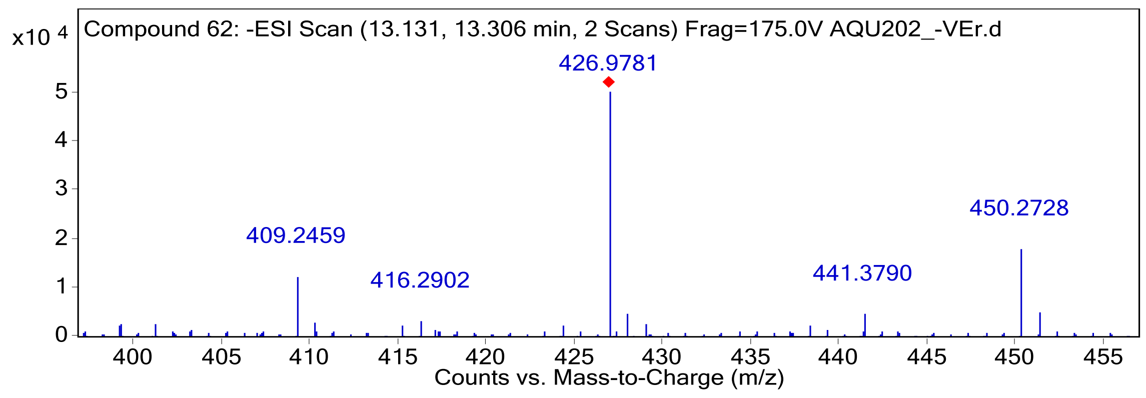

MS Spectrum Peak List

m/z	z	Abund
160.8467		14106.16
162.8438		13527.86
271.235	1	18723.75
409.2459	1	12465.29
426.9781	1	50397.77
427.9814	1	4689
428.9739	1	2618.58
450.2728	1	18121
491.3729	1	10738.99
509.3005	1	15024.09

MSMS Spectrum

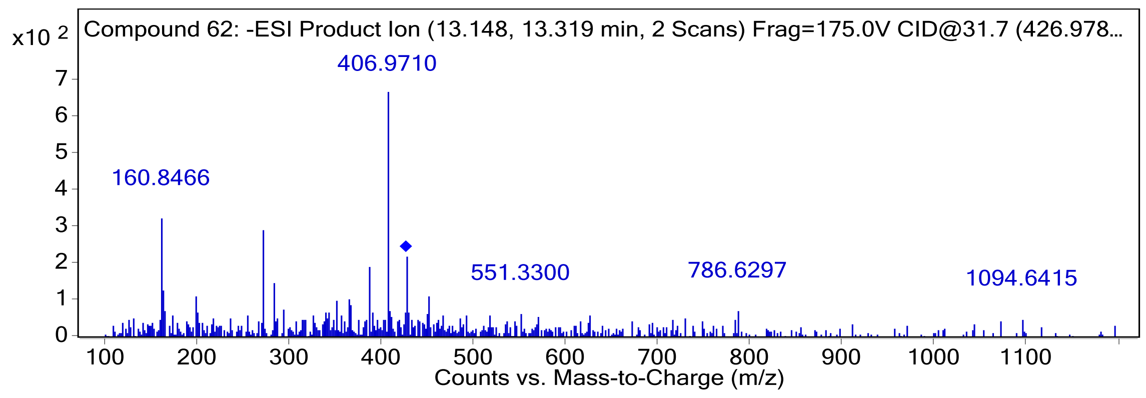

MS/MS Spectrum Peak List

m/z	z	Abund
160.8466		323.1
162.844		128.83
197.8135		113.14
271.2352	1	291.31
282.99		149.18
365.2775		104.27
386.9648		190.24
406.971	1	667.79
426.9772	1	218.62
450.2678		110.79

Compound Label	*m/z*	RT	Algorithm
Compound 63	271.2351	13.303	Auto MS/MS

MS Spectrum

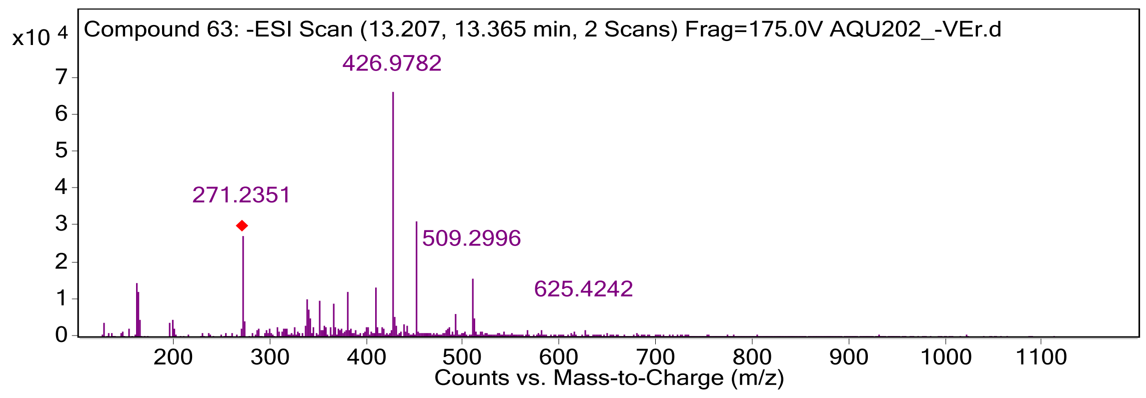

MS Zoomed Spectrum

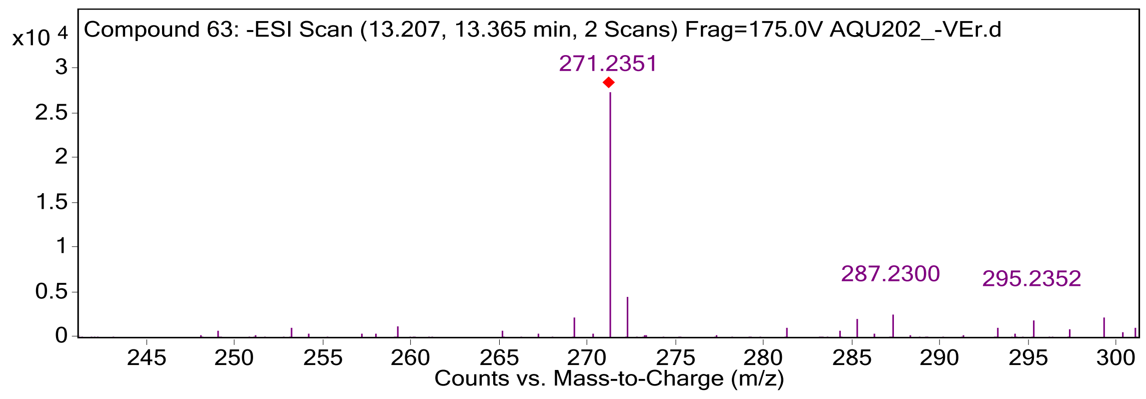

MS Spectrum Peak List

m/z	z	Abund
160.8468		14654.62
162.8439		12357.15
271.2351	1	27435.88
272.2387	1	4551.32
273.24	1	373.2
379.2578	1	12147.25
409.2456	1	13416.78
426.9782	1	66365.08
450.2729	1	31484.98
509.2996	1	15690.81

MSMS Spectrum

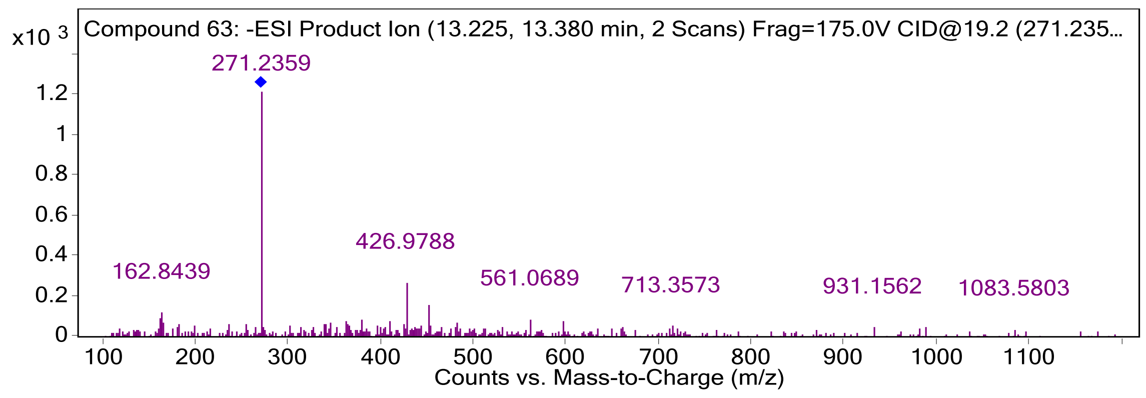

MS/MS Spectrum Peak List

m/z	z	Abund
160.8472		96.74
162.8439		122.75
271.2359	1	1217.09
362.2344		79.14
379.258		85.25
409.2466		78.25
426.9788		271.79
450.2732	1	161
561.0689		89.64
595.3458		77.13

Compound Label	*m/z*	RT	Algorithm
Compound 64	271.235	13.619	Auto MS/MS

MS Spectrum

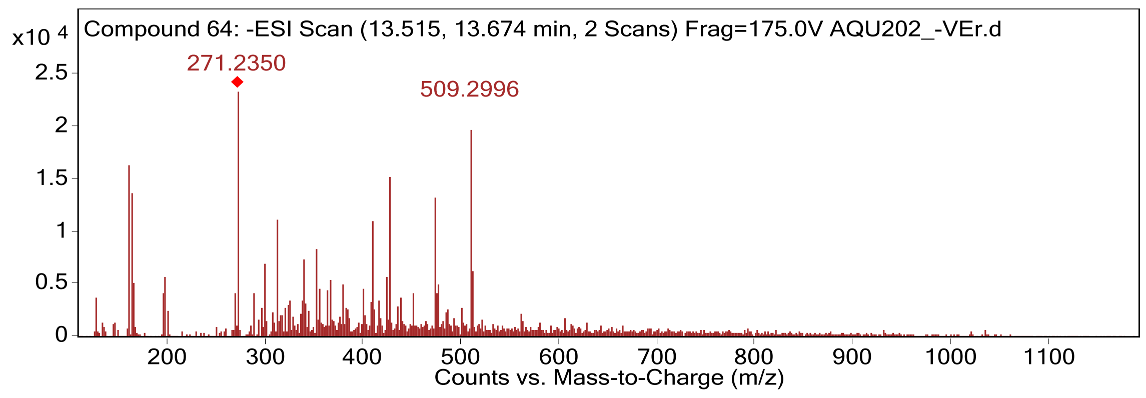

MS Zoomed Spectrum

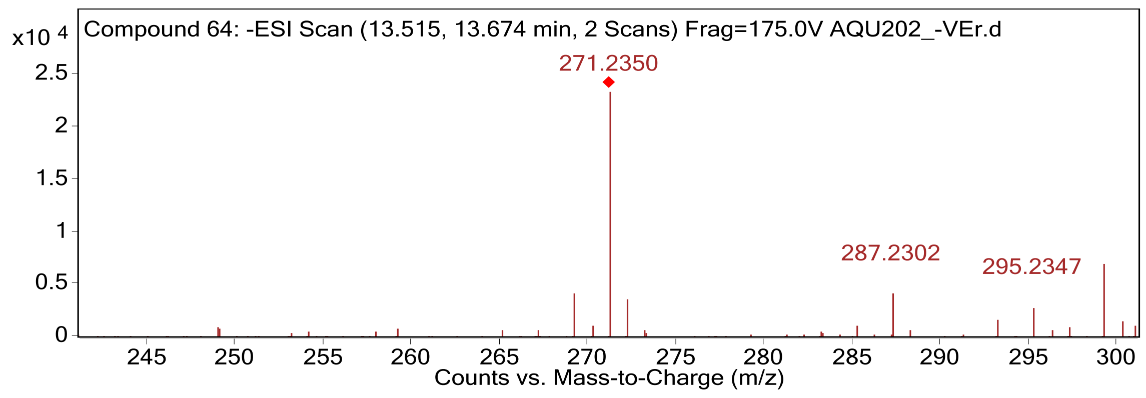

MS Spectrum Peak List

m/z	z	Abund
160.8469		16318.01
162.8441		13730.02
271.235	1	23422.7
272.2381	1	3642.33
273.2389	1	386.62
311.2305	1	11137.81
409.2461	1	11068.87
426.9781		15294.85
473.3622	1	13250.56
509.2996	1	19772.02

MSMS Spectrum

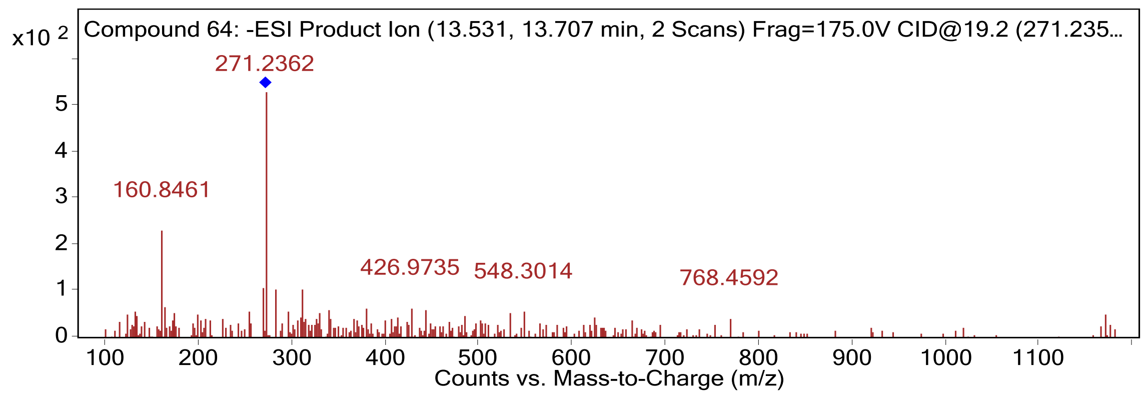

MS/MS Spectrum Peak List

m/z	z	Abund
160.8461		231.68
162.8413		67.84
269.2205		106.63
271.2362	1	530.8
272.2393	1	153.15

m/z	z	Abund
281.2579		103.67
311.2302		104.03
339.2242		60.71
378.2388		62.48
426.9735		63.76

Compound Label	Name	*m/z*	RT	Algorithm	Mass
Cpd 65: Bryodulcosigenin	**Bryodulcosigenin**	473.3621	13.708	Auto MS/MS	474.3692

MS Spectrum

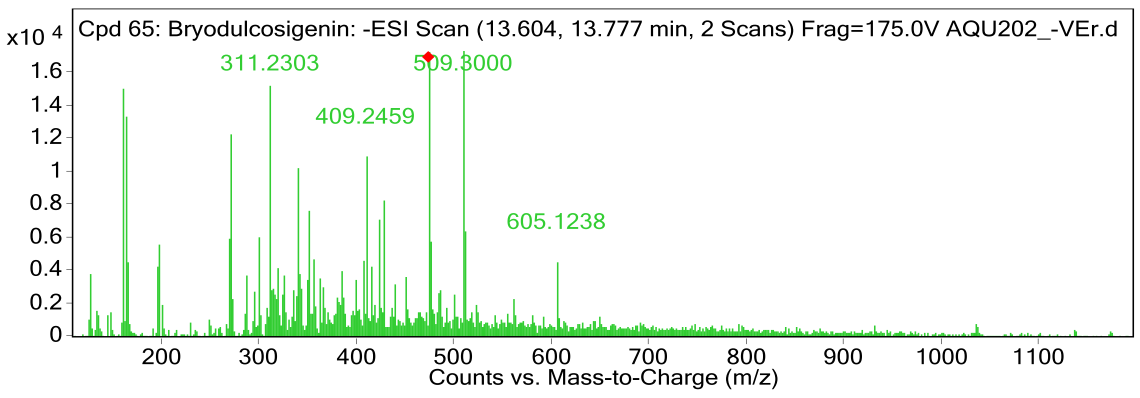

MS Zoomed Spectrum

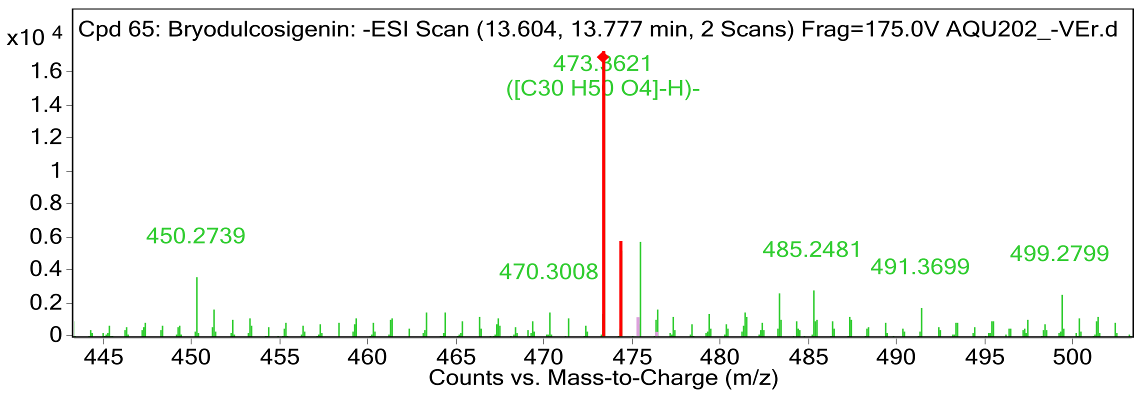

MS Spectrum Peak List

m/z	*Calc m/z*	Diff(ppm)	z	Abund	Formula	Ion
160.8467				15065.77		
162.8441				13348.59		
271.2349			1	12256.46		
311.2303			1	15203.68		
339.2188			1	10211.96		
409.2459			1	10932.65		
426.9785				8251.75		
473.3621	473.3636	3.31	1	17282.81	C30 H50 O4	(M-H)-
474.3647	474.367	4.96	1	5269.89	C30 H50 O4	(M-H)-
509.3			1	19250.66		

MSMS Spectrum

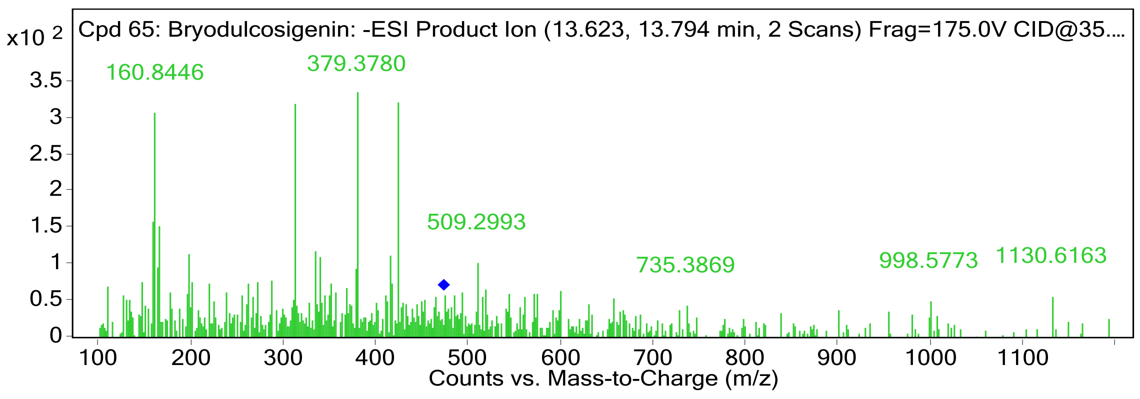

MS/MS Spectrum Peak List

m/z	z	Abund
159.1193	1	159.61
160.8446		307.38
164.8411		152.68
197.8136		113.73
311.2293	1	318.63
334.3187		117.79
339.2193	1	110.6
379.378		336.14
415.2819		112.35
423.3664		321.36

Compound Structure

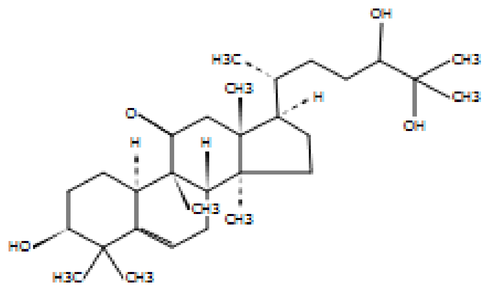

Compound Label	Name	*m/z*	RT	Algorithm	Mass
Cpd 66: Usambarine	**Usambarine**	509.2996	13.783	Auto MS/MS	450.2858

MS Spectrum

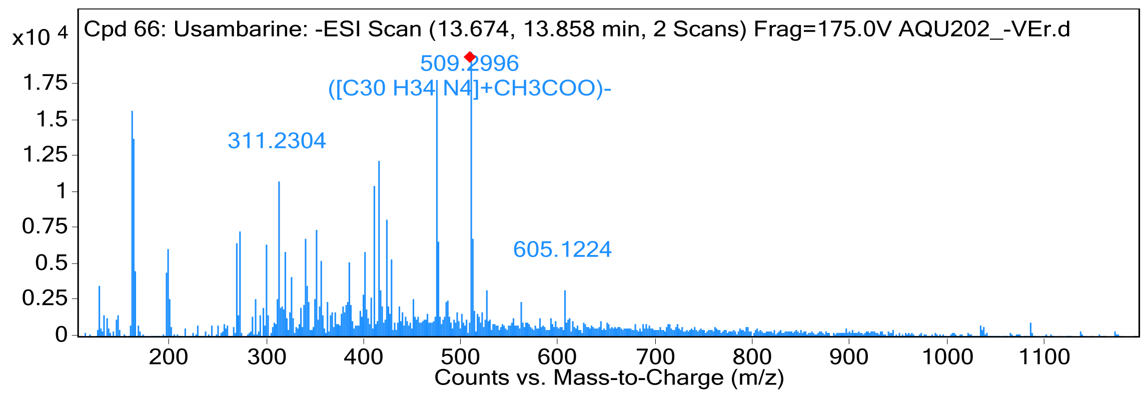

MS Zoomed Spectrum

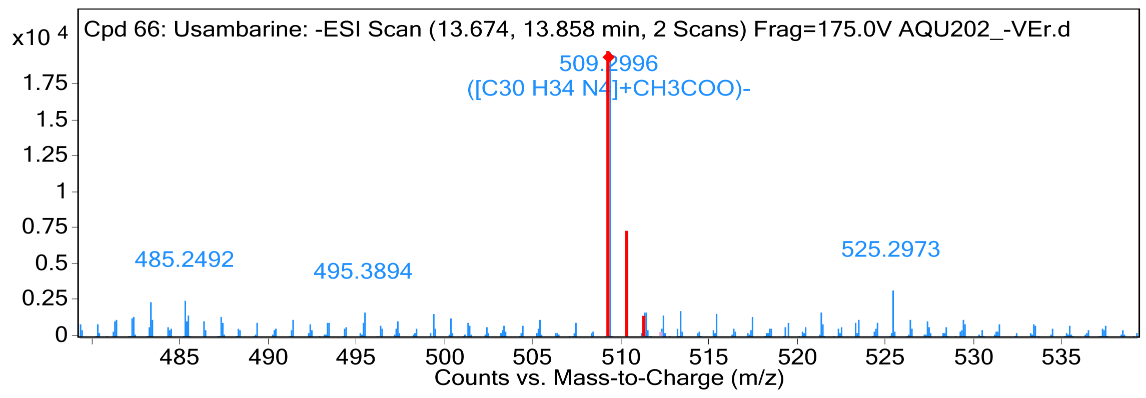

MS Spectrum Peak List

m/z	*Calc m/z*	Diff(ppm)	z	Abund	Formula	Ion
160.8468				15641.12		
162.844				13719.53		
311.2304			1	10781.13		
409.2461			1	10500.86		
415.2908			1	12157.33		
423.369			1	8168.8		
473.3622			1	17797.54		
509.2996	509.2922	-14.44	1	19732.94	C30 H34 N4	(M+CH3COO)-
510.3036	510.2953	-16.24	1	6784.5	C30 H34 N4	(M+CH3COO)-
511.3038	511.2983	-10.69	1	1731.97	C30 H34 N4	(M+CH3COO)-

MSMS Spectrum

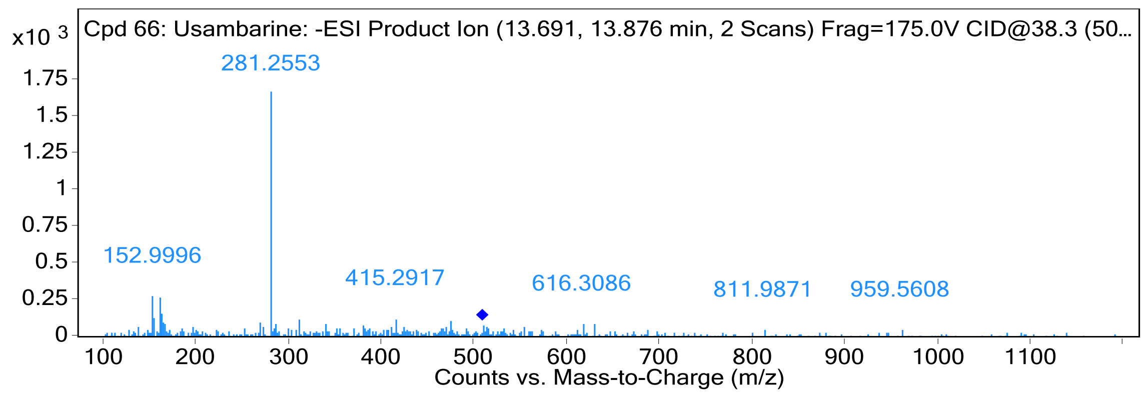

MS/MS Spectrum Peak List

m/z	Abund
152.9996	277.84
153.4926	128.5
160.8472	267.4
162.8438	162.82
164.8405	101.66
269.2251	104.09
281.2553	1674.64
311.231	120.92
415.2917	124.88
473.3636	109.09

Compound Structure

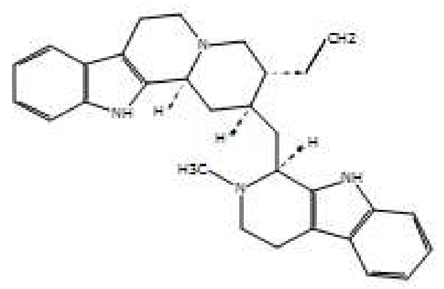

Compound Label	*m/z*	RT	Algorithm
Compound 67	414.3322	14.012	Auto MS/MS

MS Spectrum

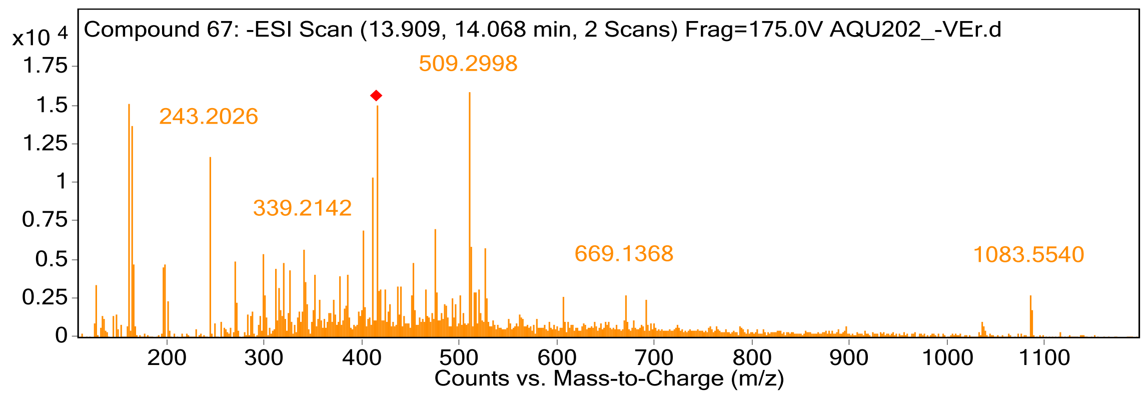

MS Zoomed Spectrum

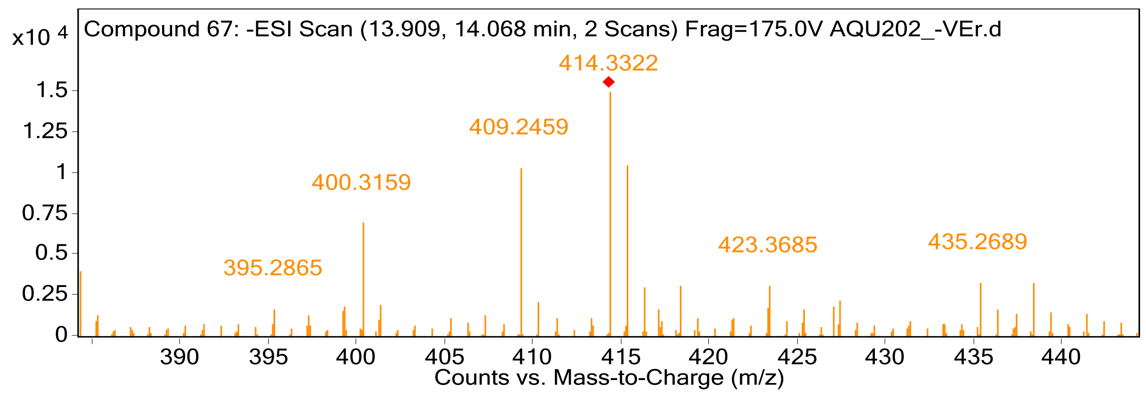

MS Spectrum Peak List

m/z	z	Abund
160.8468		15177.18
162.844		13680.47
243.2026	1	11717.48
409.2459	1	10388.63
414.3322	1	15059.84
415.2908	1	10555.76
415.3342	1	4156.52
416.3344	1	397.22
473.3619	1	7095.95
509.2998	1	15950.51

MSMS Spectrum

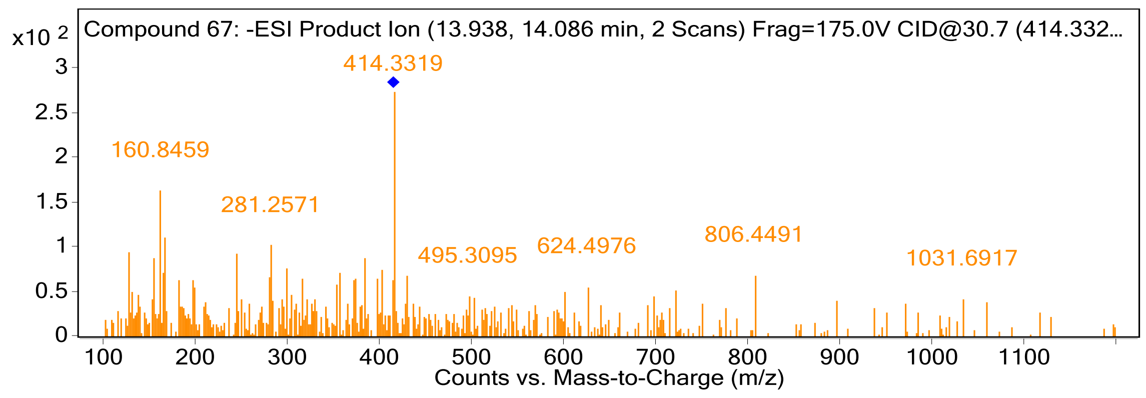

MS/MS Spectrum Peak List

m/z	z	Abund
126.9073		95.94
153		89.18
160.8459		164.4
164.8416		111.15
243.2046		93.02
281.2571		102.74
297.2471		76.75
382.3013		88.06
414.3319	1	275.13
415.3321	1	77.48

Compound Label	Name	*m/z*	RT	Algorithm	Mass
Cpd 68: (3beta,24xi)-Cycloartane-3,24,25-triol	**(3beta,24xi)-Cycloartane-3,24,25-**	519.4044	14.212	Auto MS/MS	460.3883

MS Spectrum

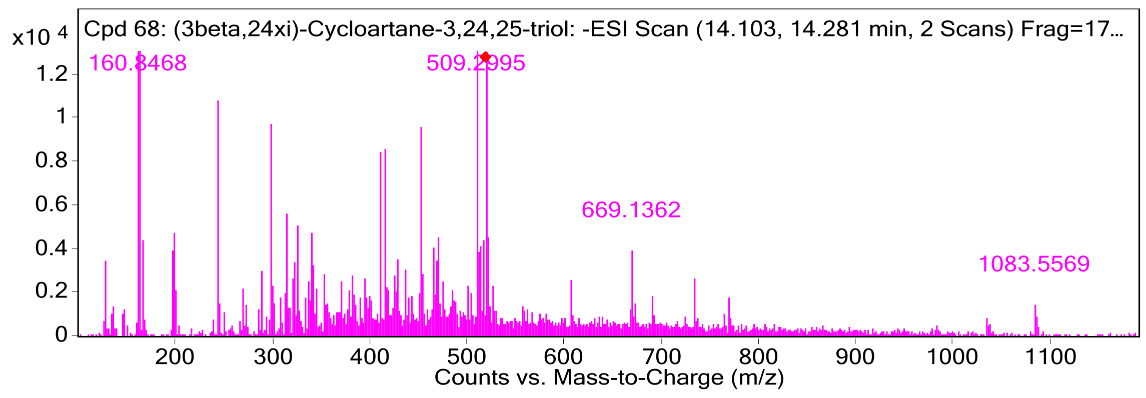

MS Zoomed Spectrum

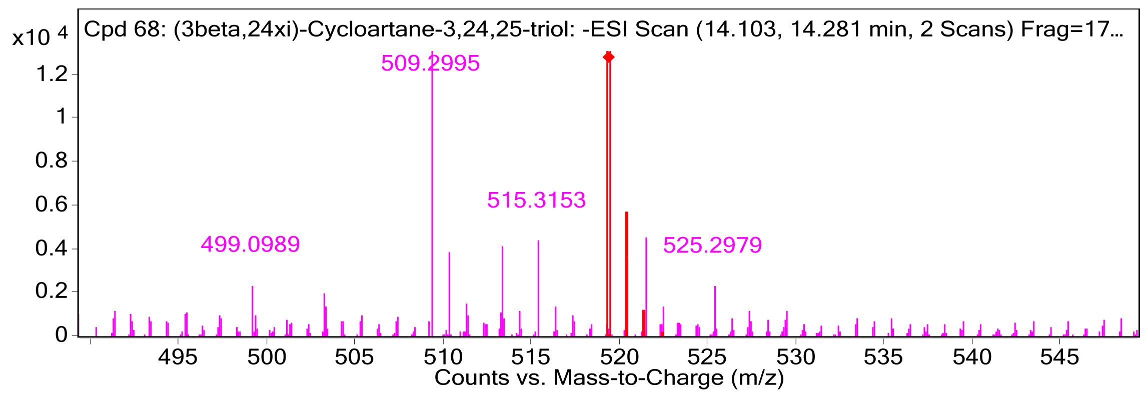

MS Spectrum Peak List

m/z	*Calc m/z*	Diff(ppm)	z	Abund	Formula	Ion
160.8468				14098.99		
162.844				13557.54		
243.2028			1	10820.63		
297.2507			1	9753.1		
509.2995			1	15649.87		
519.4044	519.4055	2.18	1	13049.52	C30 H52 O3	(M+CH3COO)-
520.4077	520.4089	2.41	1	3625.56	C30 H52 O3	(M+CH3COO)-
521.4025	521.412	18.05	1	4542.76	C30 H52 O3	(M+CH3COO)-
522.4057	522.4148	17.56	1	1382.39	C30 H52 O3	(M+CH3COO)-
523.41	523.4176	14.68	1	325.72	C30 H52 O3	(M+CH3COO)-

MSMS Spectrum

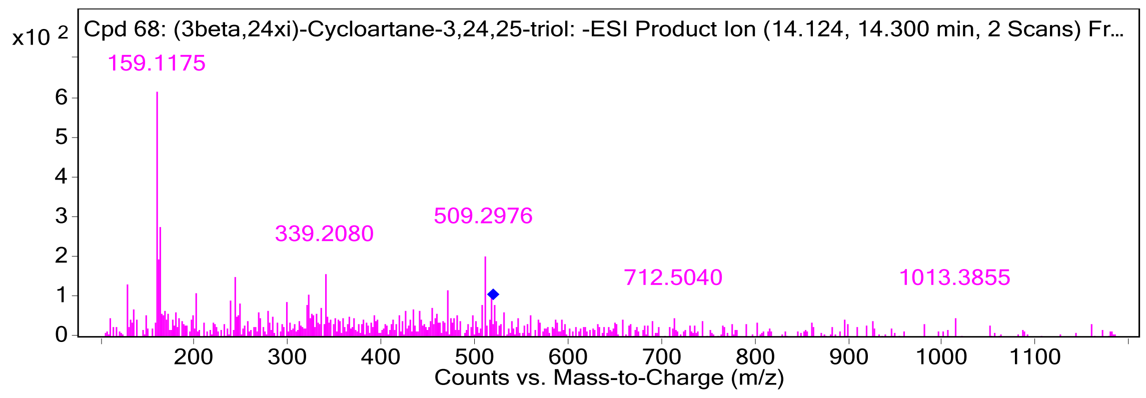

MS/MS Spectrum Peak List

m/z	z	Abund
126.9081		132.98
159.1175		617.72
160.8462		194.62
162.8447		278.49
201.1299		111.12
243.2013	1	150.53
321.2497		108.44
339.208		157.2
469.4102		117.72
509.2976	1	202.15

Compound Structure

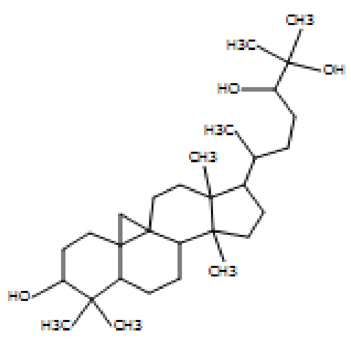

Compound Label	Name	*m/z*	RT	Algorithm	Mass
Cpd 69: Undecylprodigiosin	**Undecylprodigiosin**	452.2888	14.243	Auto MS/MS	393.275

MS Spectrum

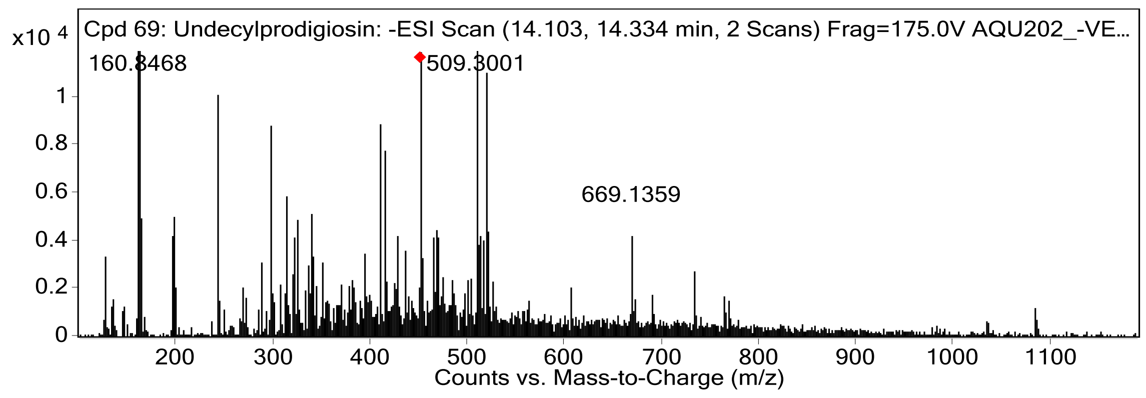

MS Zoomed Spectrum

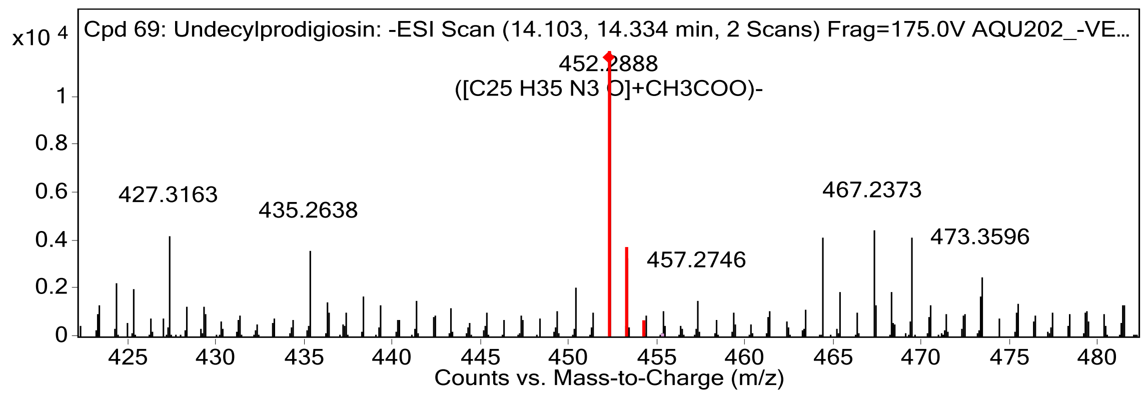

MS Spectrum Peak List

m/z	*Calc m/z*	Diff(ppm)	z	Abund	Formula	Ion
160.8468				13398.01		
162.844				12599.69		
243.2027			1	10082.4		
297.2505			1	8823.29		
409.2463			1	8865.41		
452.2888	452.2919	6.74	1	11873.44	C25 H35 N3 O	(M+CH3COO)-
453.2931	453.295	4.23	1	3305.9	C25 H35 N3 O	(M+CH3COO)-
454.2913	454.298	14.68	1	902.97	C25 H35 N3 O	(M+CH3COO)-
509.3001			1	15283.3		
519.4045			1	10991.43		

MSMS Spectrum

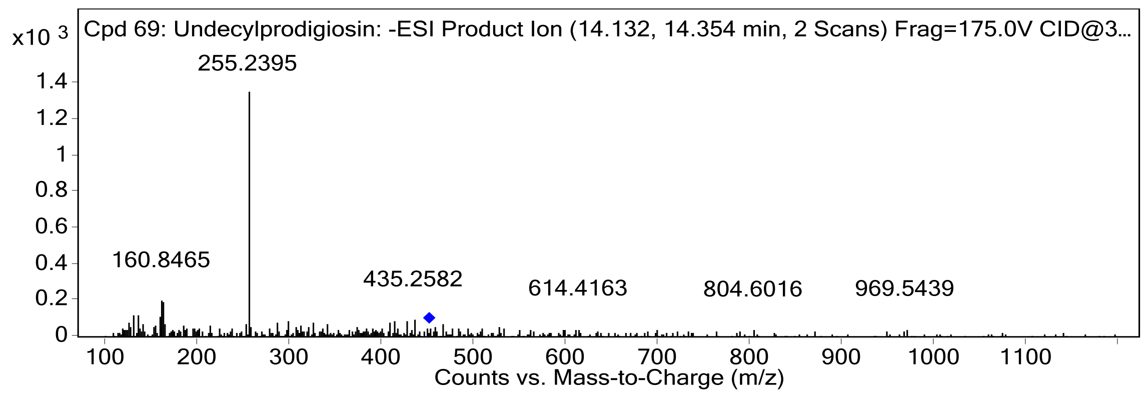

MS/MS Spectrum Peak List

m/z	z	Abund
130.8919		122.97
134.8695		118.73
159.1182		110.06
160.8465		201.04
162.8448		192.04
255.2395	1	1356.86
256.2415	1	175.66
414.3319		92.78

427.3083		89.2
435.2582		94.91

Compound Structure

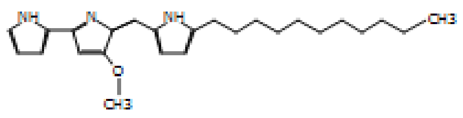

Compound Label	Name	*m/z*	RT	Algorithm	Mass
Cpd 70: Undecylprodigiosin	**Undecylprodigiosin**	452.2887	14.634	Auto MS/MS	393.2748

MS Spectrum

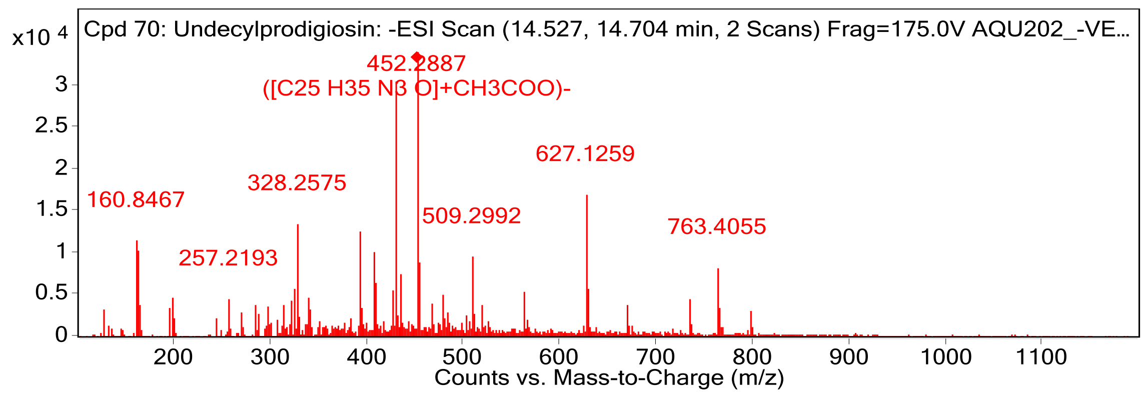

MS Zoomed Spectrum

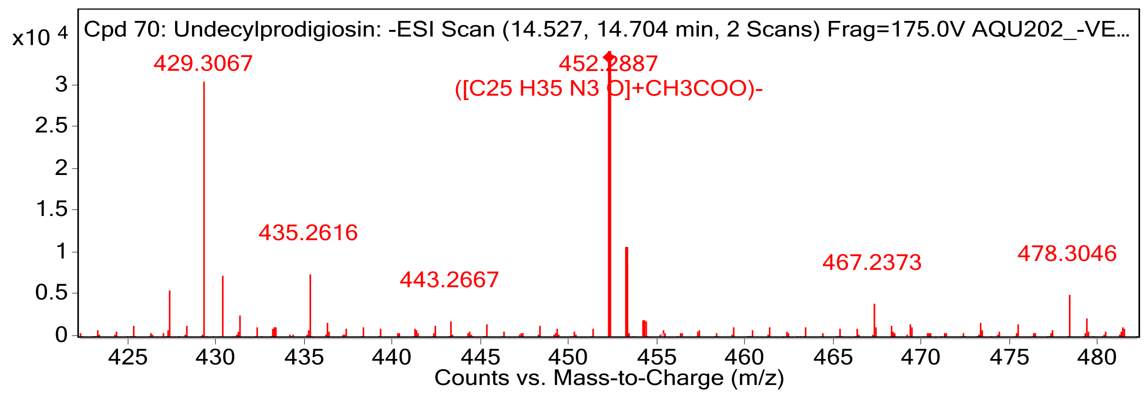

MS Spectrum Peak List

m/z	*Calc m/z*	Diff(ppm)	z	Abund	Formula	Ion
160.8467				11542.83		
162.8441				10381.94		
328.2575			1	13558.59		
393.3105			1	12544.02		
407.2904			1	10153.66		
429.3067			1	30498.67		
452.2887	452.2919	7.02	1	34005.7	C25 H35 N3 O	(M+CH3COO)-
453.2919	453.295	6.82	1	9025.7	C25 H35 N3 O	(M+CH3COO)-
454.2945	454.298	7.72	1	1849.38	C25 H35 N3 O	(M+CH3COO)-
627.1259			1	17043.77		

MSMS Spectrum

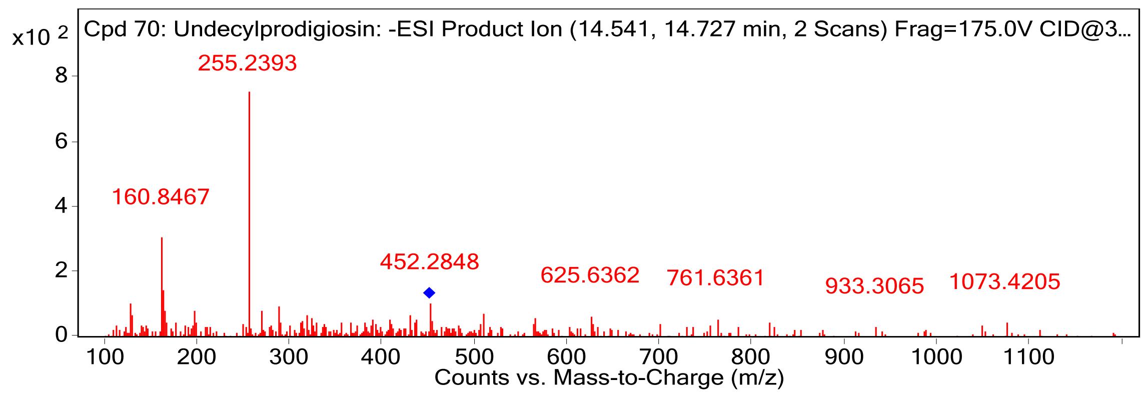

MS/MS Spectrum Peak List

m/z	z	Abund
126.9105		103.07
160.8467		306.61
162.8461		145.95
164.8433		81.74
196.0433		81.35
255.2393	1	757.56
269.2207		80.38
287.2311		94.68
452.2848	1	105.83
509.3077		72.36

Compound Structure

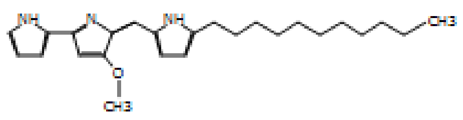

Compound Label	Name	*m/z*	RT	Algorithm	Mass
Cpd 71: 5'-Methoxybilobetin	**5'-Methoxybilobetin**	627.1259	14.816	Auto MS/MS	582.1257

MS Spectrum

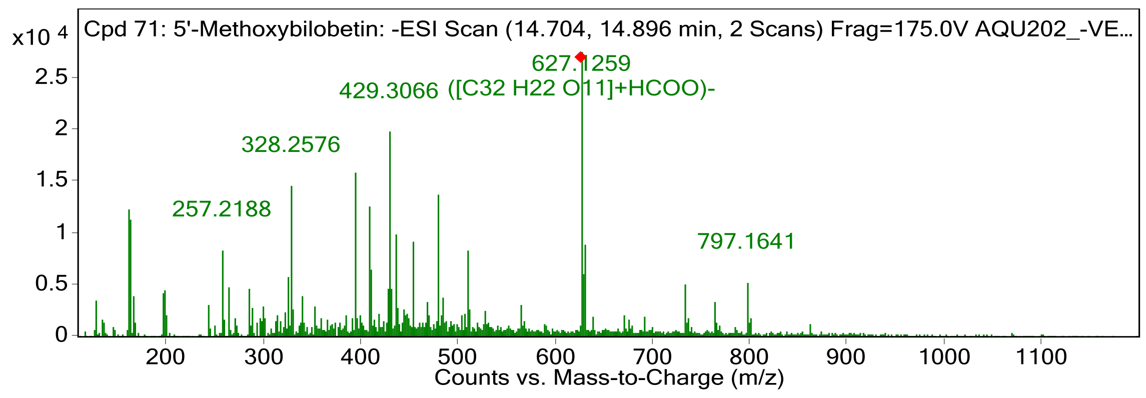

MS Zoomed Spectrum

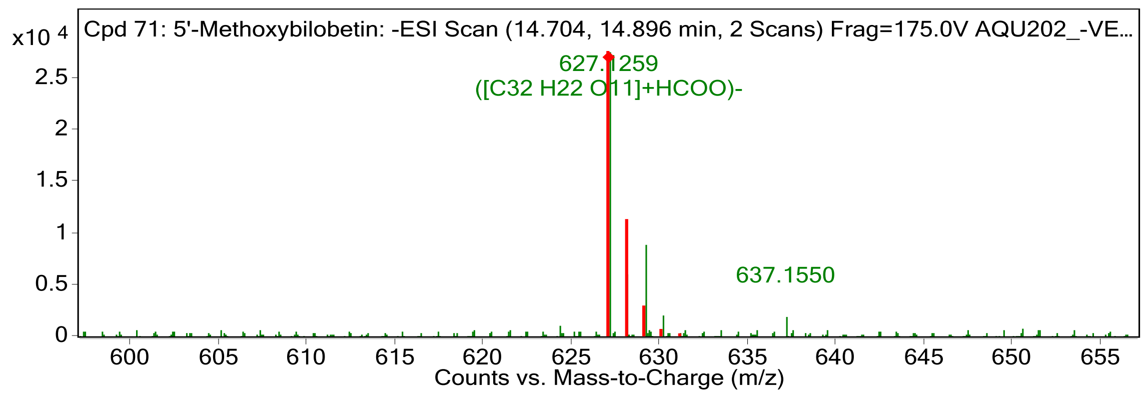

MS Spectrum Peak List

m/z	*Calc m/z*	Diff(ppm)	z	Abund	Formula	Ion
328.2576			1	14692.7		
393.3104			1	15849.93		
407.2903			1	12587.88		
429.3066			1	19903.22		
478.304			1	13802.78		

m/z	*Calc m/z*	Diff(ppm)	z	Abund	Formula	Ion
627.1259	627.1144	-18.39	1	27564.98	C32 H22 O11	(M+HCOO)-
628.1291	628.1178	-17.97	1	6069.4	C32 H22 O11	(M+HCOO)-
629.1236	629.1205	-4.94	1	8969.77	C32 H22 O11	(M+HCOO)-
630.1277	630.1231	-7.29	1	2184.5	C32 H22 O11	(M+HCOO)-
631.1316	631.1257	-9.39	1	476.98	C32 H22 O11	(M+HCOO)-

MSMS Spectrum

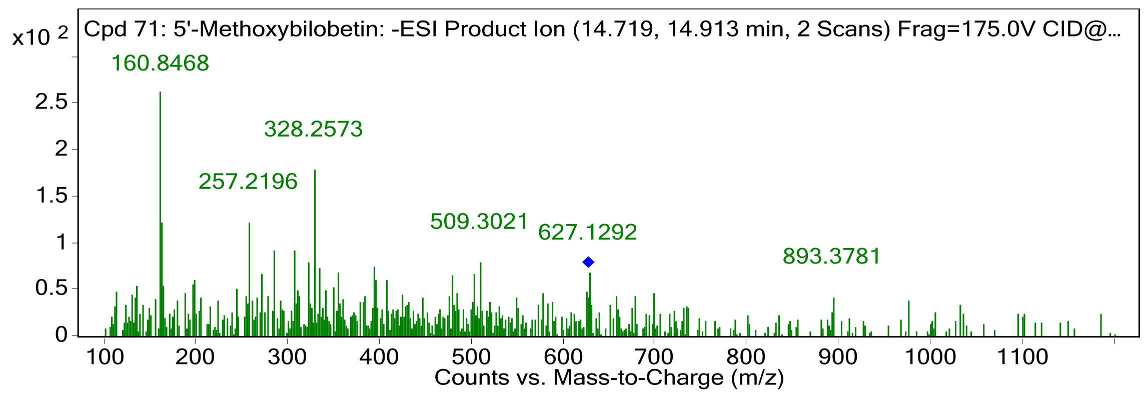

MS/MS Spectrum Peak List

m/z	z	Abund
160.8468		263.64
162.8446		123.11
257.2196		123.17
284.2313	1	92.41
305.7857		92.32
321.247		79.72
328.2573		179.51
334.2493		74.31
393.3071		75.72
509.3021		80.16

Compound Structure

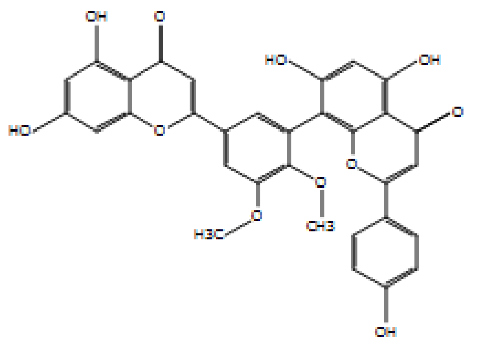

Compound Label	*m/z*	RT	Algorithm
Compound 72	478.3044	14.865	Auto MS/MS

MS Spectrum

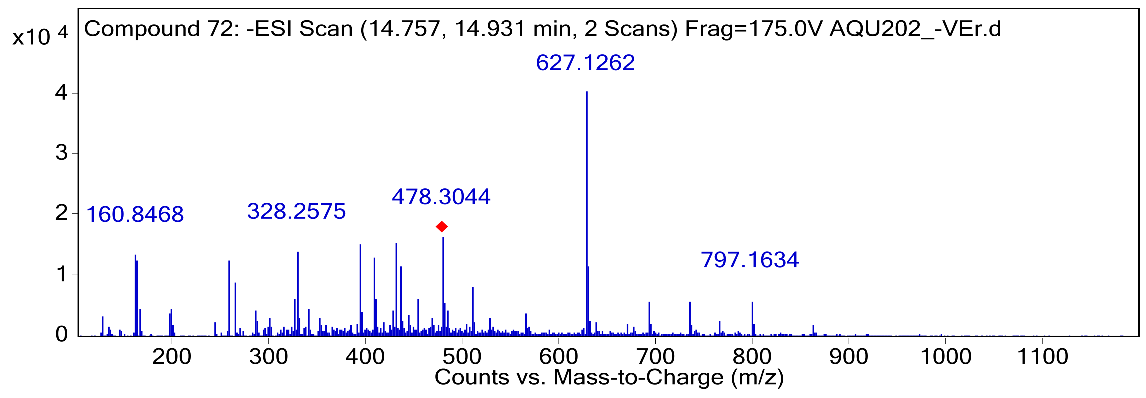

MS Zoomed Spectrum

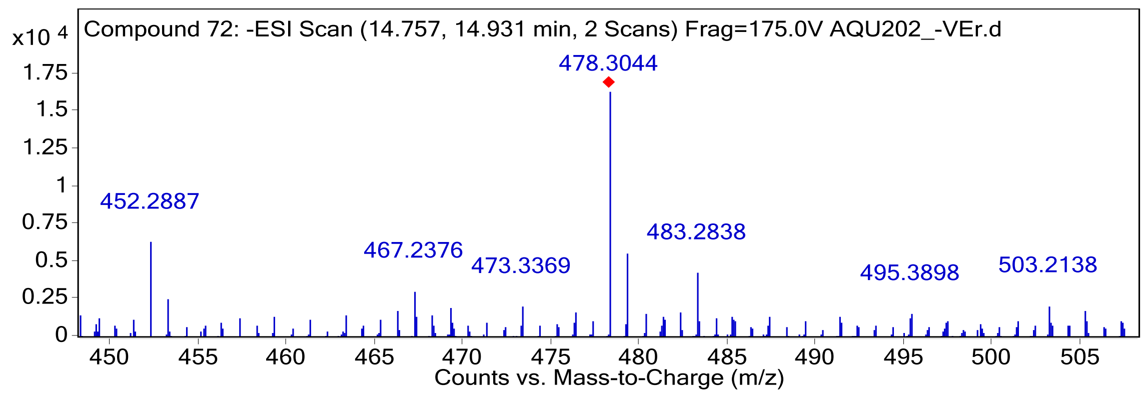

MS Spectrum Peak List

m/z	z	Abund
160.8468		13434.03
257.2185	1	12628.37
328.2575	1	13945.69
393.3102	1	15267
407.2898	1	13152.67
429.3065	1	15371.62
478.3044	1	16343.34
479.3081	1	5525.29
480.3108	1	1595.76
627.1262	1	40475.3

MSMS Spectrum

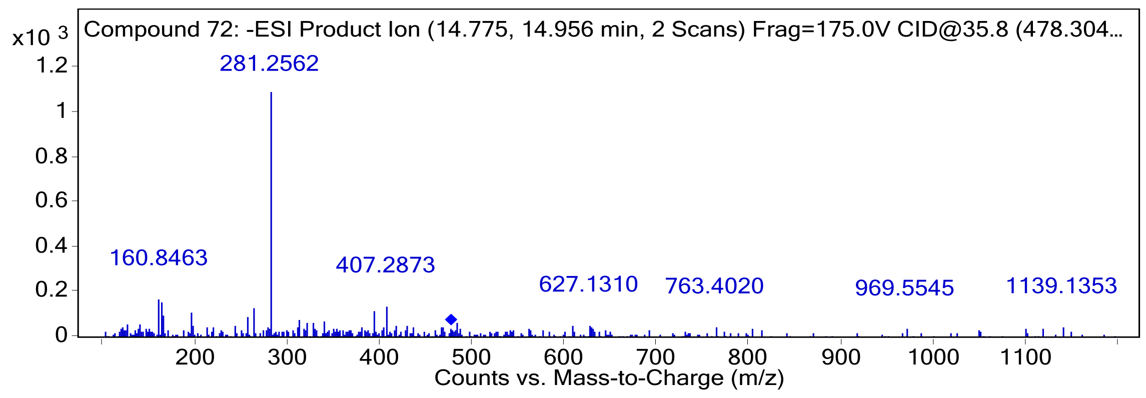

MS/MS Spectrum Peak List

m/z	z	Abund
160.8463		172.1
162.8434		156.32

164.8415		98.98
196.0426		109.92
257.2204		89.65
263.1383	1	132.72
281.2562	1	1093.37
282.2593	1	114.04
393.3085		117.31
407.2873		140.01

Compound Label	*m/z*	RT	Algorithm
Compound 73	691.1398	15.045	Auto MS/MS

MS Spectrum

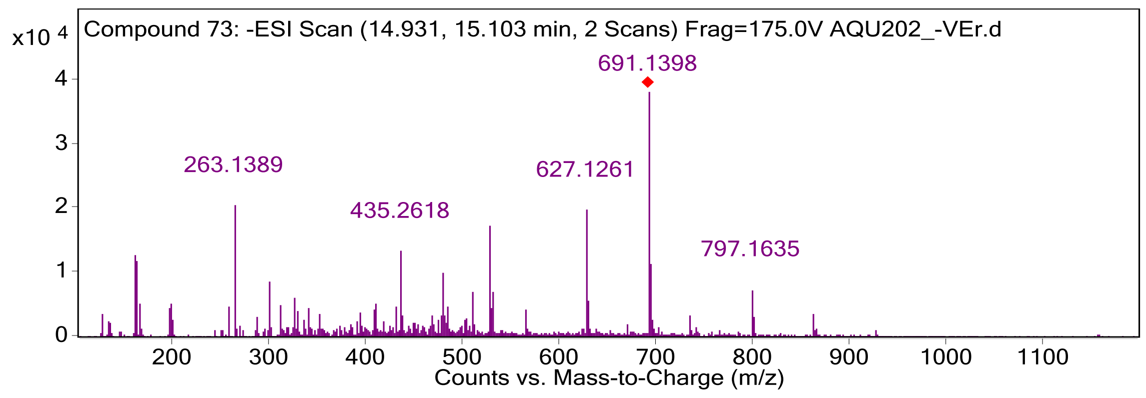

MS Zoomed Spectrum

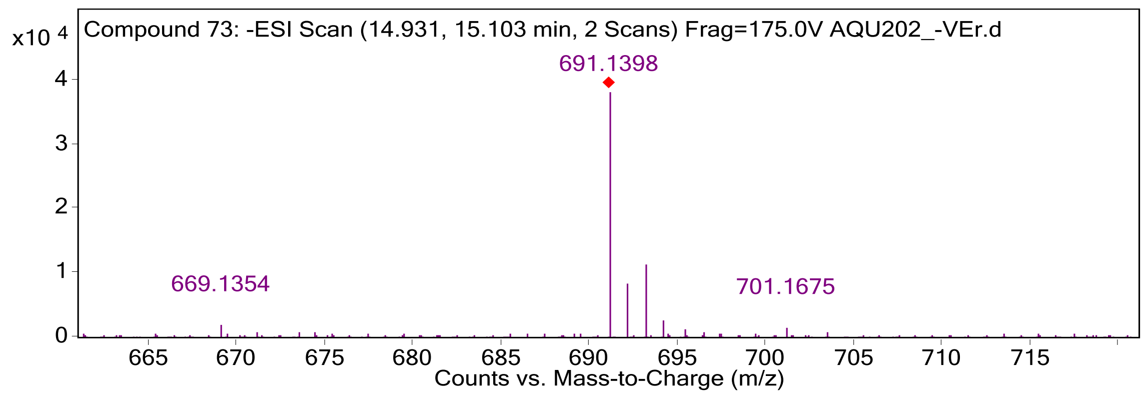

MS Spectrum Peak List

m/z	z	Abund
160.8468		12781.4
162.8441		11829.59
263.1389	1	20488.3
435.2618	1	13356.69
527.3106	1	17232.35
627.1261	1	19760.68
691.1398	1	38160.01
692.1432	1	8481.99
693.1377	1	11377.07
694.14	1	2639.06

MSMS Spectrum

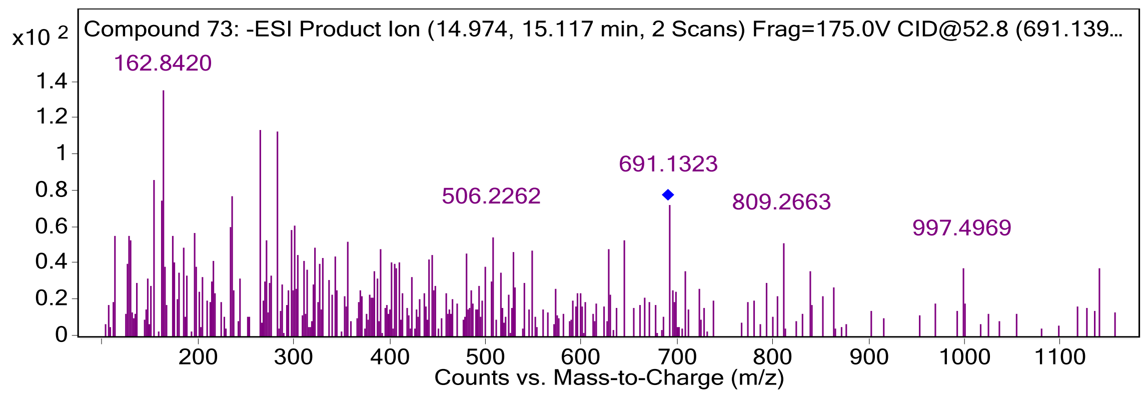

MS/MS Spectrum Peak List

m/z	z	Abund
152.9998		86.78
160.8464		75.05
162.842		135.38
232.1478		60.71
235.1111		77.5
263.1378	1	114.22
281.257		113.2
296.0842		59.33
299.2632		61.52
691.1323		72.83

Compound Label	Name	*m/z*	RT	Algorithm	Mass
Cpd 74: Milbemectin	**Milbemectin**	527.311	15.133	Auto MS/MS	528.3168

MS Spectrum

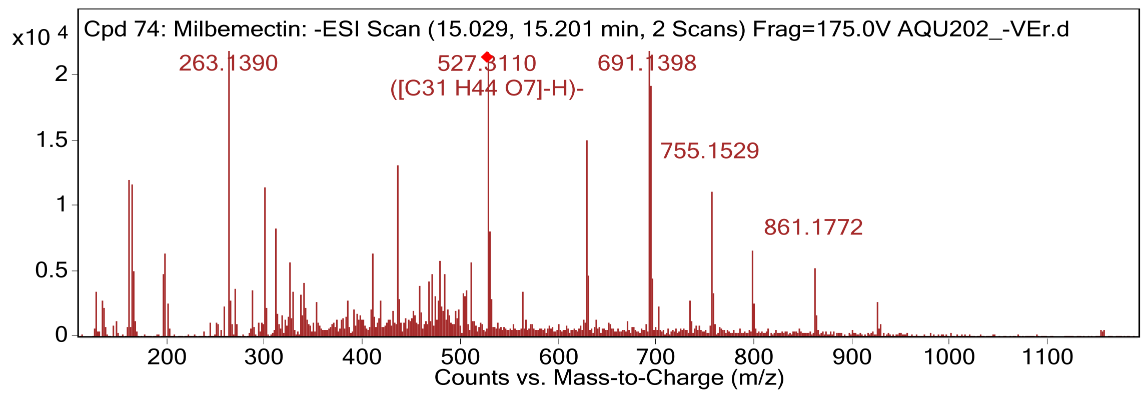

MS Zoomed Spectrum

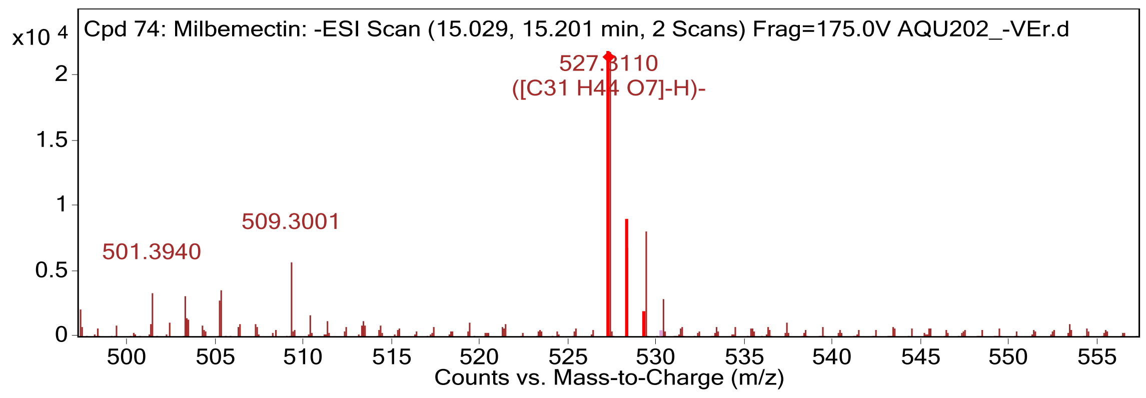

MS Spectrum Peak List

m/z	*Calc m/z*	Diff(ppm)	z	Abund	Formula	Ion
160.8467				12053.85		
263.139			1	22431.3		
435.262			1	13151.33		
527.311	527.3014	-18.1	1	21831.94	C31 H44 O7	(M-H)-
528.3151	528.3048	-19.37	1	6834.07	C31 H44 O7	(M-H)-
529.3102	529.3077	-4.76	1	8125.79	C31 H44 O7	(M-H)-
627.1263			1	15121.63		
691.1398			1	61695.04		
692.1428			1	12921.87		
693.137			1	19279.72		

MSMS Spectrum

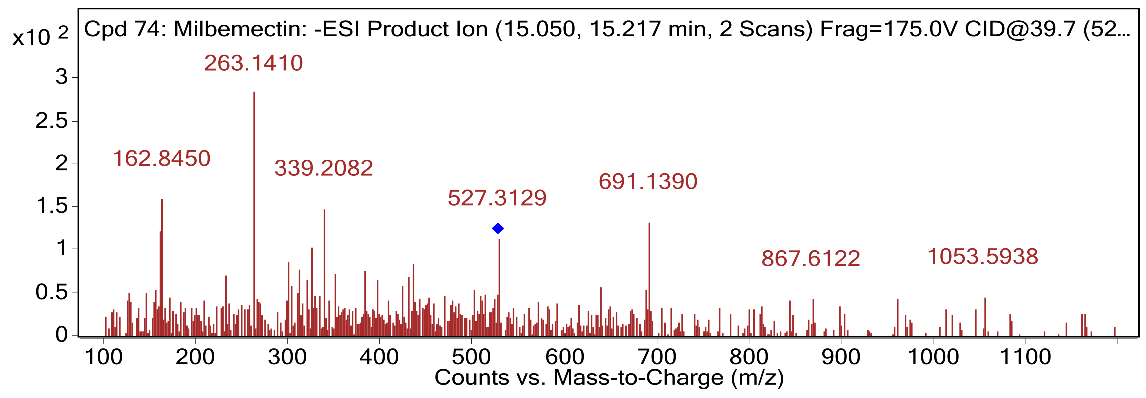

MS/MS Spectrum Peak List

m/z	z	Abund
160.8464		123.12
162.845		160.29
263.141		285.7
299.2631		87.9
311.2507		77.73
325.1916		104.53
339.2082		148.99
435.258	1	85.01
527.3129		114.22
691.139		133.4

Compound Structure

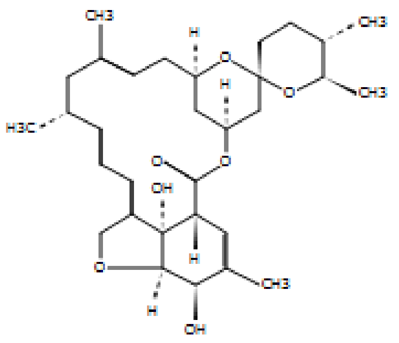

Compound Label	Name	*m/z*	RT	Algorithm	Mass
Cpd 75: Maclurin 3-C-(2''-p-hydroxybenzoyl-6''-galloyl-glucoside)	**Maclurin 3-C-(2''-p-hydroxybenzoyl-6''-galloyl-glucoside)**	755.153	15.318	Auto MS/MS	696.137

MS Spectrum

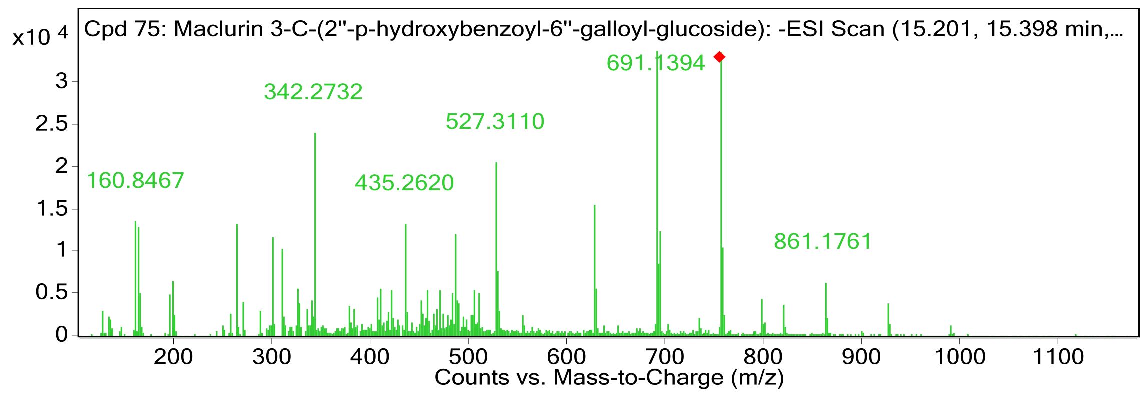

MS Zoomed Spectrum

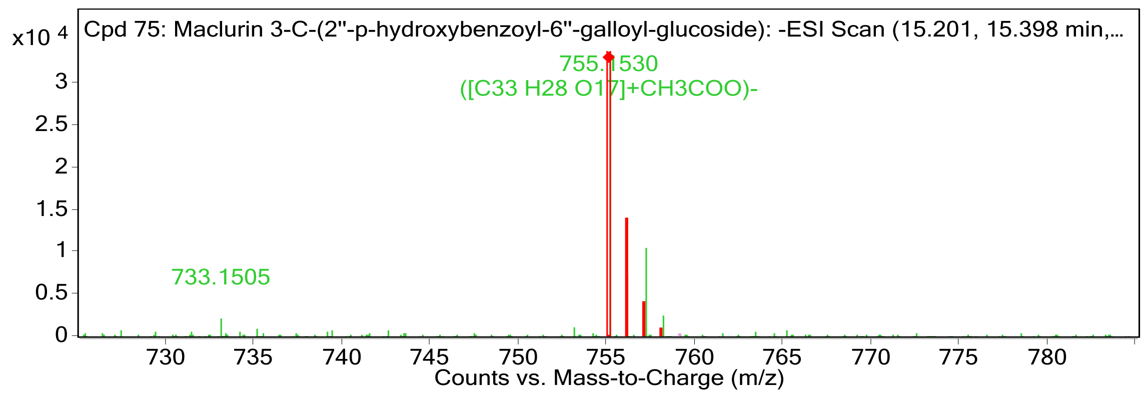

MS Spectrum Peak List

m/z	*Calc m/z*	Diff(ppm)	z	Abund	Formula	Ion
160.8467				13701.07		
342.2732			1	24216.61		
435.262			1	13417.92		
527.311			1	20697.3		
627.1261			1	15690.73		
691.1394			1	42762		
755.153	755.1465	-8.59	1	33724.95	C33 H28 O17	(M+CH3COO)-

756.1559	756.1499	-7.9	1	7519.11	C33 H28 O17	(M+CH3COO)-
757.1503	757.1524	2.77	1	10684.25	C33 H28 O17	(M+CH3COO)-
758.1534	758.1551	2.3	1	2639.82	C33 H28 O17	(M+CH3COO)-

MSMS Spectrum

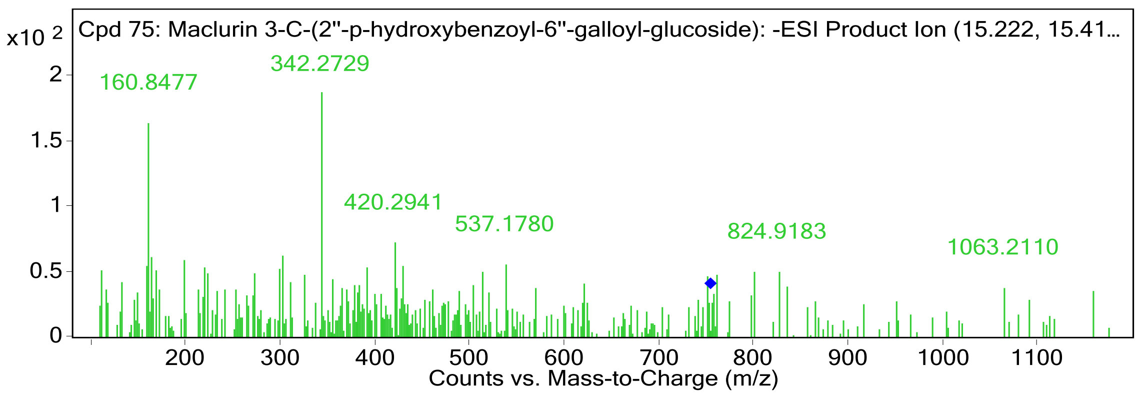

MS/MS Spectrum Peak List

m/z	z	Abund
160.8477		164.76
162.8432		61.96
197.813		60.14
301.0971		62.95
342.2729		188.65
343.2747		94.13
420.2941	1	72.68
420.7159		64.99
428.3132		55.39
537.178		56.02

Compound Structure

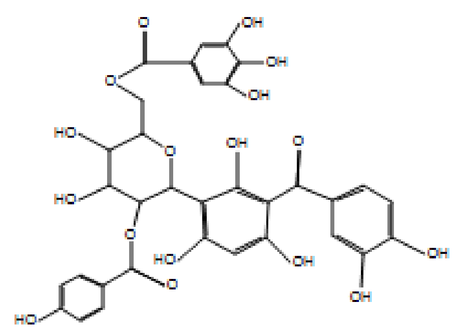

Compound Label	Name	*m/z*	RT	Algorithm	Mass
Cpd 76: UDP-2,4-bis(acetamido)-2,4,6-trideoxy-beta-L-	**UDP-2,4-bis(acetamido)-2,4,6-trideoxy-beta-L-**	691.1395	15.402	Auto MS/MS	632.1237

MS Spectrum

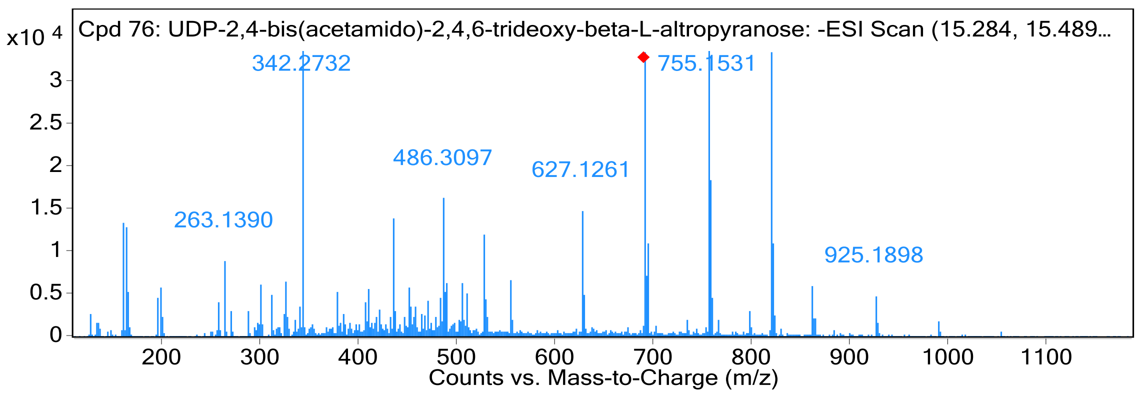

MS Zoomed Spectrum

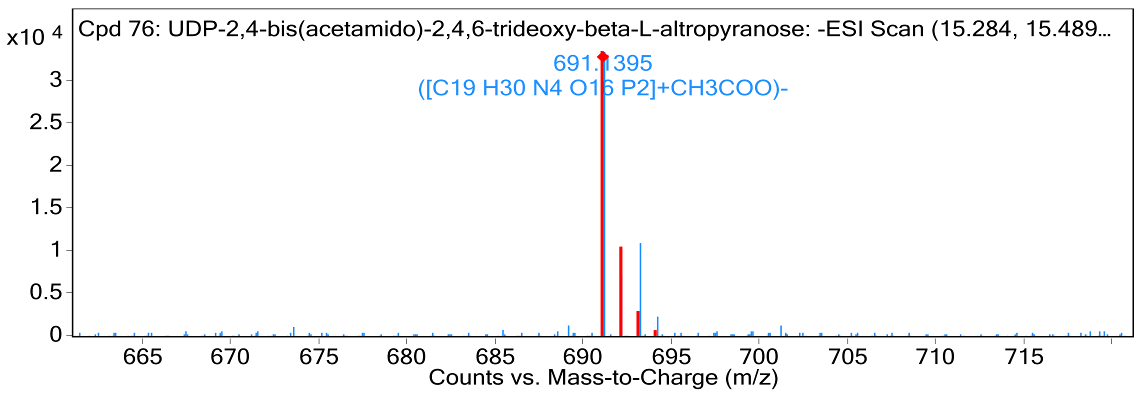

MS Spectrum Peak List

m/z	*Calc m/z*	Diff(ppm)	z	Abund	Formula	Ion
342.2732			1	37442.66		
486.3097			1	16303.81		
691.1395	691.1271	-17.93	1	33438.57	C19 H30 N4 O16 P2	(M+CH3COO)-
692.1428	692.1301	-18.29	1	7199.07	C19 H30 N4 O16 P2	(M+CH3COO)-
693.1369	693.1321	-6.85	1	11047.1	C19 H30 N4 O16 P2	(M+CH3COO)-
694.1401	694.1347	-7.73	1	2401.55	C19 H30 N4 O16 P2	(M+CH3COO)-
695.1447	695.1369	-11.26	1	528.52	C19 H30 N4 O16 P2	(M+CH3COO)-
755.1531			1	60079.25		
757.151			1	18471.41		
819.1665			1	33498.38		

MSMS Spectrum

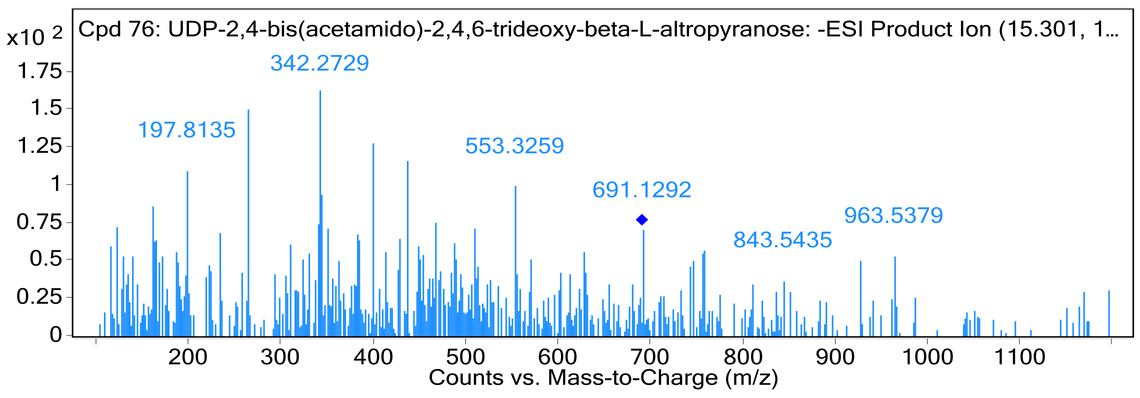

MS/MS Spectrum Peak List

m/z	z	Abund
160.8457		86.28
197.8135		110.07
263.137		150.48

264.1427		86.1
342.2729		162.53
343.2736		93.66
398.3429		127.91
435.2631		116.84
467.2389		75.7
553.3259	1	99.51

Compound Structure

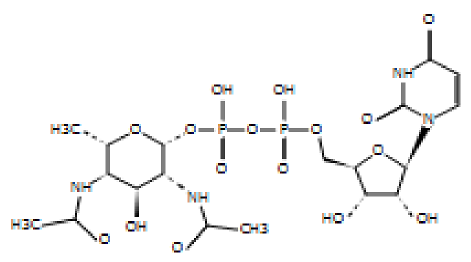

Compound Label	Name	*m/z*	RT	Algorithm	Mass
Cpd 77: UDP-2,4-bis(acetamido)-2,4,6-trideoxy-beta-L-	**UDP-2,4-bis(acetamido)-2,4,6-trideoxy-beta-L-**	691.1396	15.656	Auto MS/MS	632.1241

MS Spectrum

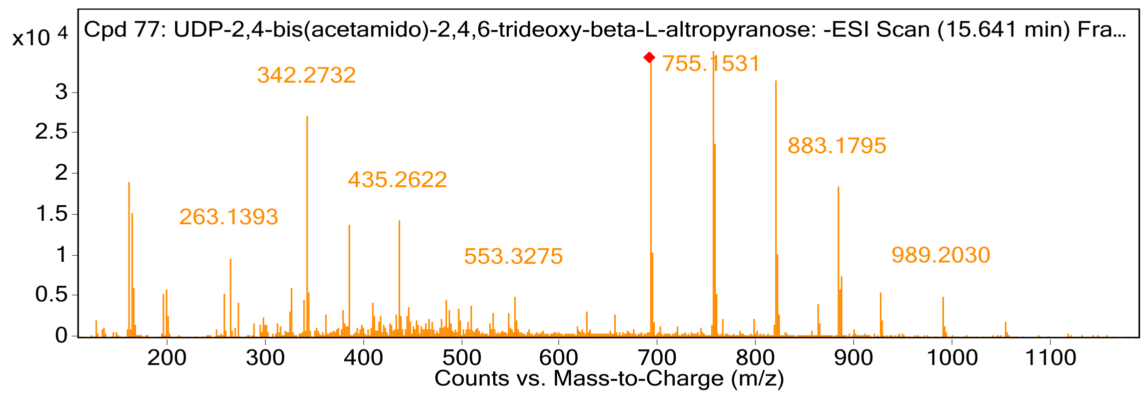

MS Zoomed Spectrum

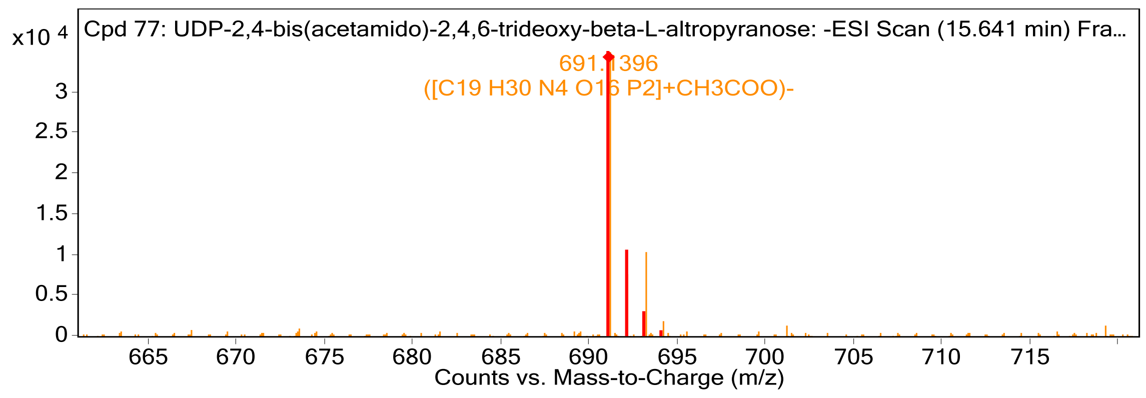

MS Spectrum Peak List

m/z	*Calc m/z*	Diff(ppm)	z	Abund	Formula	Ion
160.8468				19040.87		
342.2732			1	27232.4		
691.1396	691.1271	-18.08	1	34982.29	C19 H30 N4 O16 P2	(M+CH3COO)-
692.1424	692.1301	-17.77	1	7762.64	C19 H30 N4 O16 P2	(M+CH3COO)-
693.1379	693.1321	-8.31	1	10437.91	C19 H30 N4 O16 P2	(M+CH3COO)-
694.1406	694.1347	-8.48	1	1918.32	C19 H30 N4 O16 P2	(M+CH3COO)-
695.1431	695.1369	-8.88	1	438.66	C19 H30 N4 O16 P2	(M+CH3COO)-
755.1531			1	74648.54		
757.1508			1	23834.73		
819.1663			1	31542.82		

MSMS Spectrum

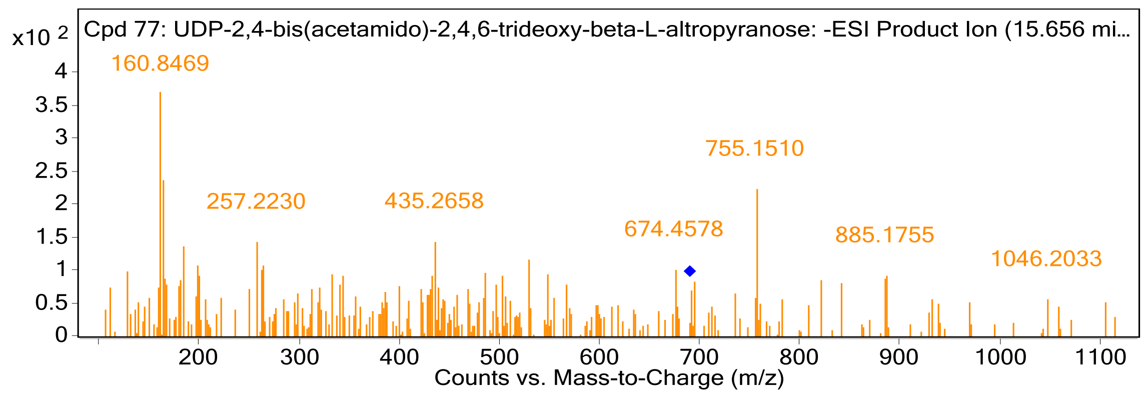

MS/MS Spectrum Peak List

m/z	Abund
160.8469	372.96
162.8475	237.78
184.2323	137.29
197.8132	109.55
257.223	144.31
261.6001	102.98
263.1397	109.38
435.2658	145.11
527.6172	118.8
755.151	224.87

Compound Structure

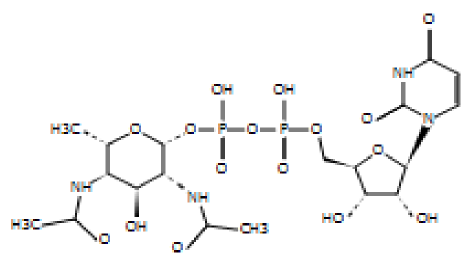

Compound Label	Name	*m/z*	RT	Algorithm	Mass
Cpd 78: Maclurin 3-C-(2''-p-hydroxybenzoyl-6''-galloyl-glucoside)	**Maclurin 3-C-(2''-p-hydroxybenzoyl-6''-galloyl-glucoside)**	755.1532	15.675	Auto MS/MS	696.1374

MS Spectrum

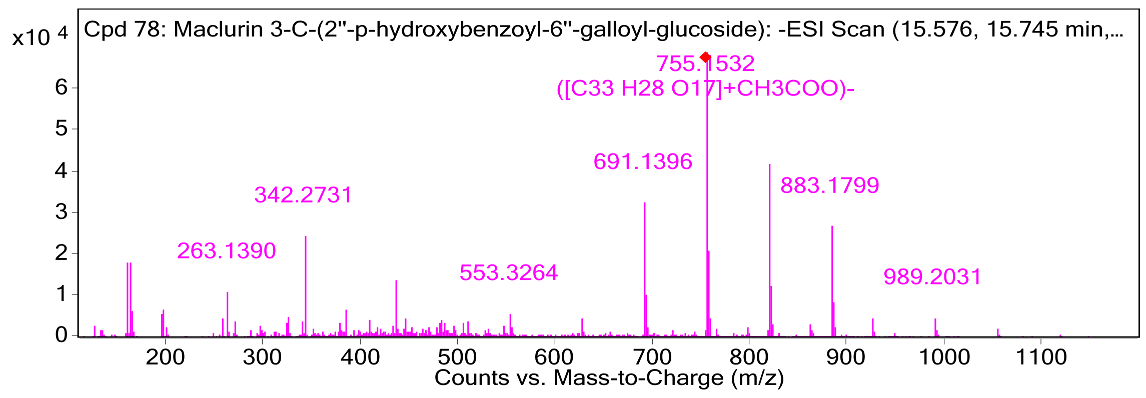

MS Zoomed Spectrum

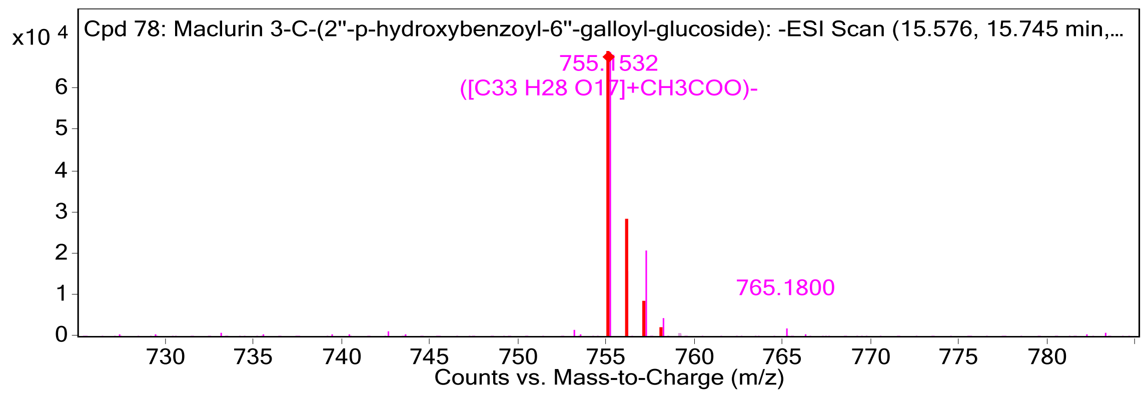

MS Spectrum Peak List

m/z	*Calc m/z*	Diff(ppm)	z	Abund	Formula	Ion
160.8467				18054.16		
162.8441				18170.14		
342.2731			1	24602.47		
691.1396			1	32705.74		
755.1532	755.1465	-8.92	1	69034.48	C33 H28 O17	(M+CH3COO)-
756.1558	756.1499	-7.83	1	15854.76	C33 H28 O17	(M+CH3COO)-
757.1509	757.1524	2.04	1	20817.64	C33 H28 O17	(M+CH3COO)-
758.1534	758.1551	2.25	1	4630.06	C33 H28 O17	(M+CH3COO)-
819.1662			1	42012.21		
883.1799			1	26909.74		

MSMS Spectrum

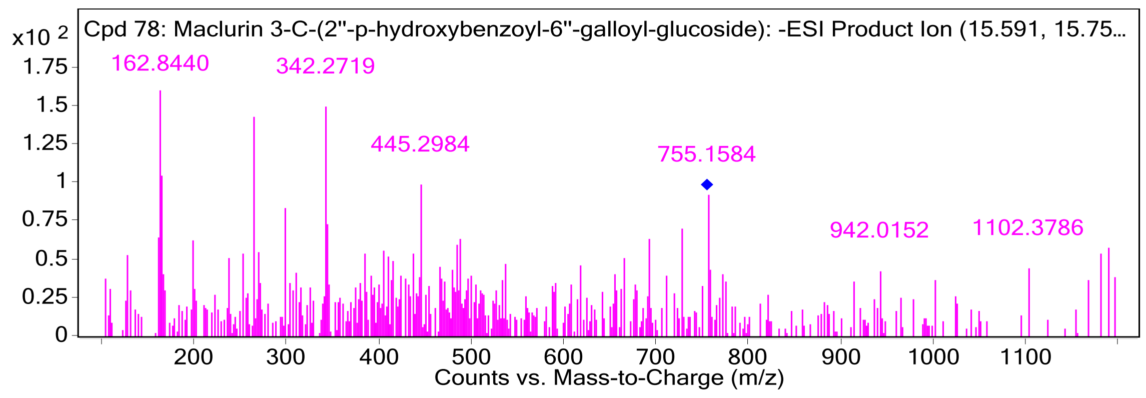

MS/MS Spectrum Peak List

m/z	z	Abund
160.8468		65.36
162.844		159.91
164.8414		104.99
263.1369		143.36
297.2483		83.64
342.2719	2	149.91
342.3099		73.28
445.2984		99.05
726.3694		70.38
755.1584	1	92.52

Compound Structure

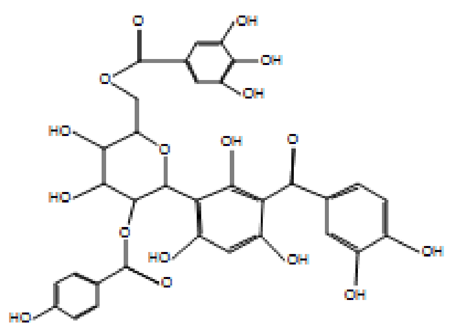

Compound Label	Name	*m/z*	RT	Algorithm	Mass
Cpd 79: Maclurin 3-C-(2''-p-hydroxybenzoyl-6''-galloyl-glucoside)	**Maclurin 3-C-(2''-p-hydroxybenzoyl-6''-galloyl-glucoside)**	755.1529	16.008	Auto MS/MS	696.137

MS Spectrum

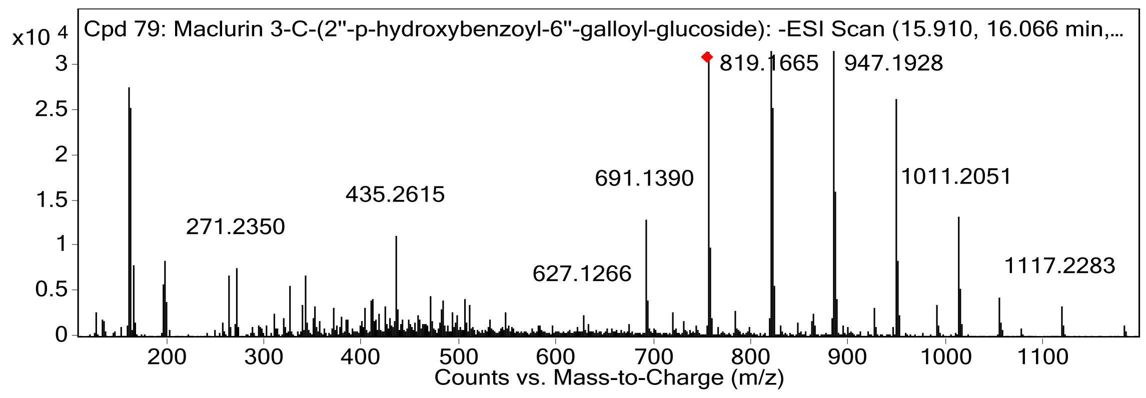

MS Zoomed Spectrum

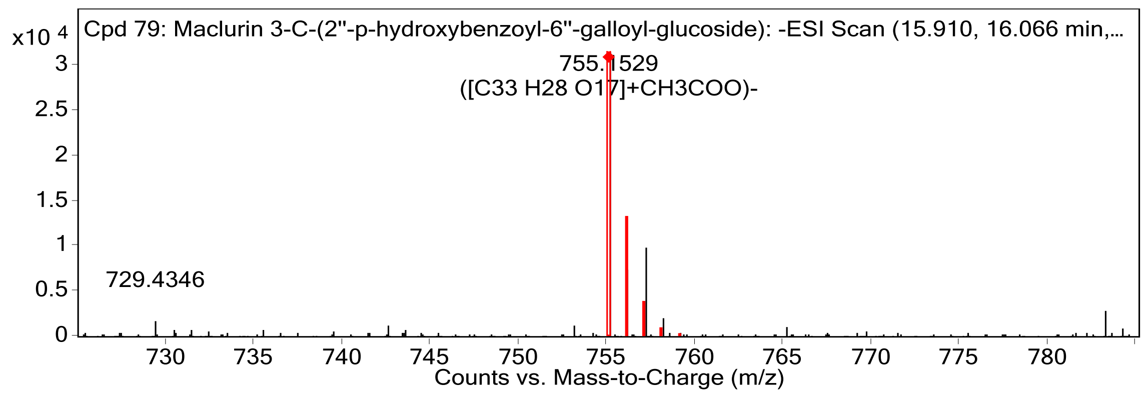

MS Spectrum Peak List

m/z	*Calc m/z*	Diff(ppm)	z	Abund	Formula	Ion
160.8468				27671.21		
162.8442				25335.33		
755.1529	755.1465	-8.44	1	31535.87	C33 H28 O17	(M+CH3COO)-
756.1557	756.1499	-7.69	1	7495.85	C33 H28 O17	(M+CH3COO)-
757.151	757.1524	1.92	1	9896.1	C33 H28 O17	(M+CH3COO)-
758.1539	758.1551	1.61	1	2064.41	C33 H28 O17	(M+CH3COO)-
759.1546	759.1576	3.91	1	552.13	C33 H28 O17	(M+CH3COO)-
819.1665			1	76655.38		
883.1797			1	50052.62		
947.1928			1	26343.13		

MSMS Spectrum

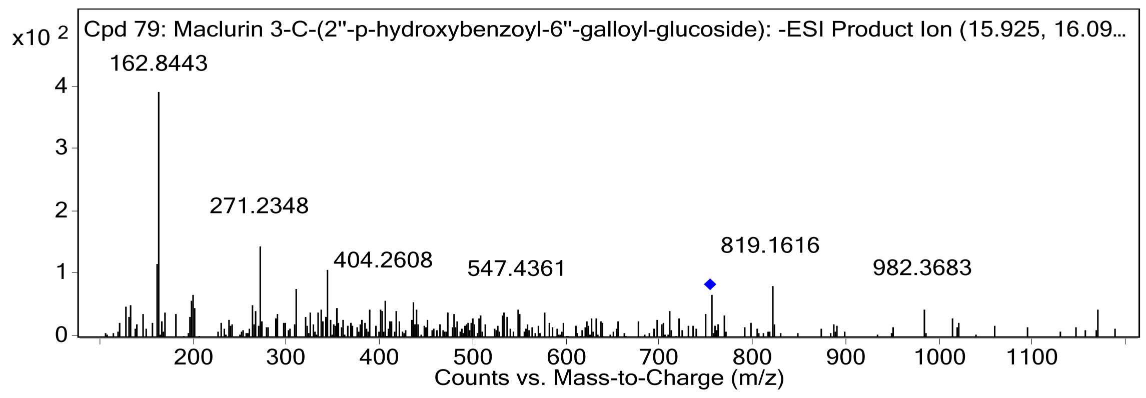

MS/MS Spectrum Peak List

m/z	Abund
160.8464	117.04
162.8443	392.63
197.8128	58.01
199.8076	68.56
271.2348	144.91
309.193	77.18
342.2764	108.84
404.2608	58.06
755.1496	68.08
819.1616	81.6

Compound Structure

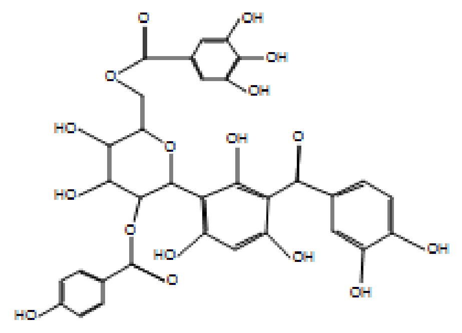

Compound Label	*m/z*	RT	Algorithm
Compound 80	883.1796	16.174	Auto MS/MS

MS Spectrum

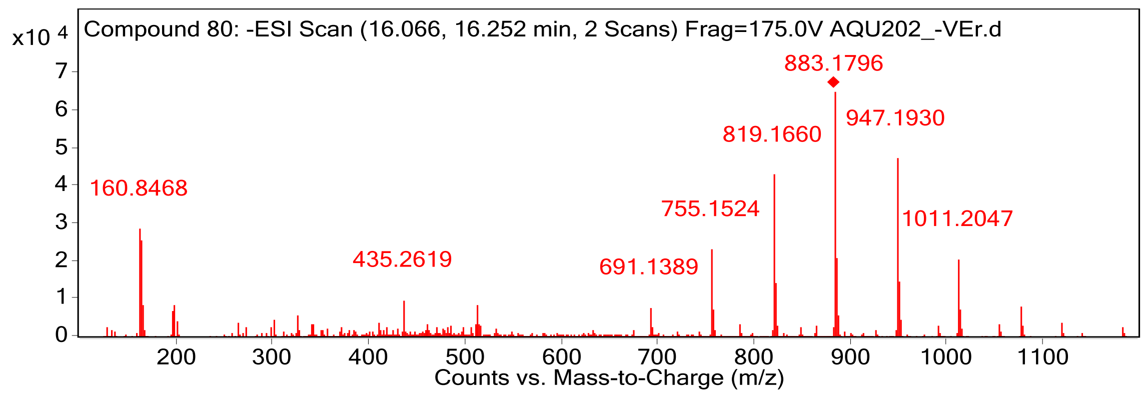

MS Zoomed Spectrum

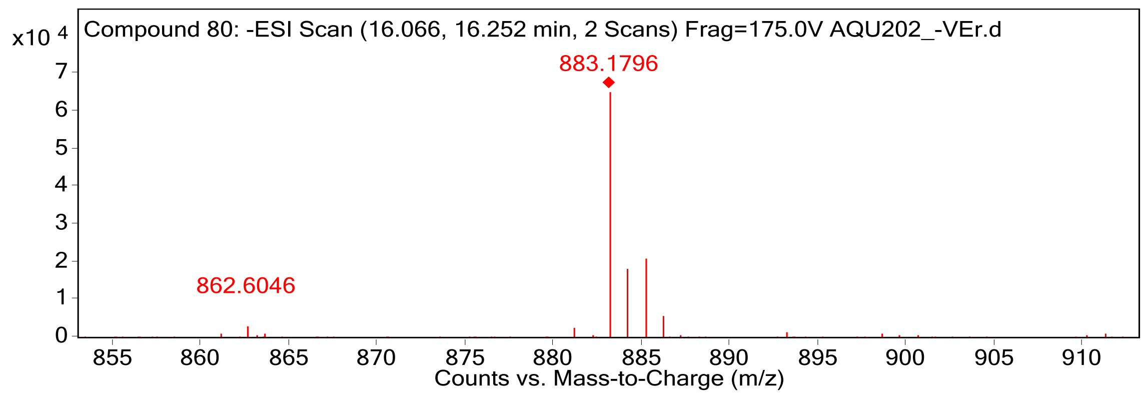

MS Spectrum Peak List

m/z	z	Abund
160.8468		28717.52
162.8441		25683.07
755.1524	1	23254.63
819.166	1	43041.57
883.1796	1	65119.64
884.1824	1	18264.2
885.1769	1	20982.8
886.1799	1	5677.95
887.1846	1	729.85

947.193	1	47370.66

MSMS Spectrum

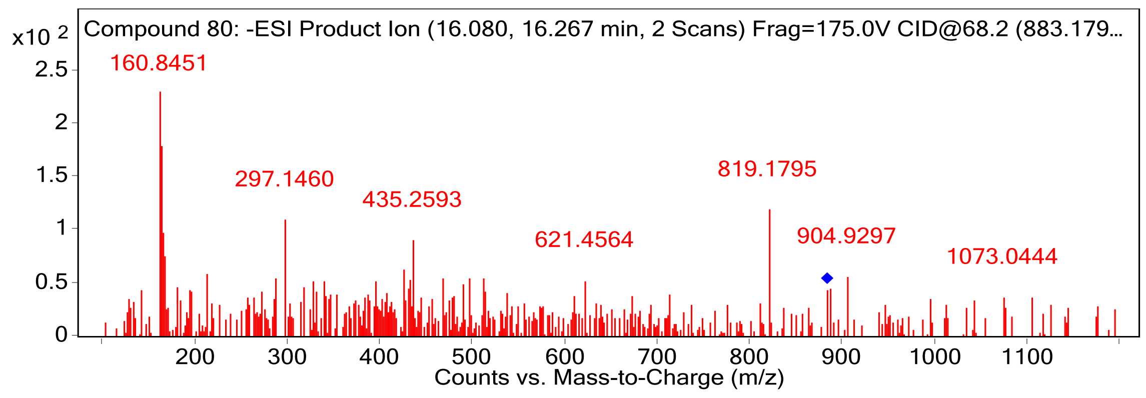

MS/MS Spectrum Peak List

m/z	Abund
160.8451	230.86
162.8444	179.39
164.8417	97.71
166.9852	75.28
212.5248	59.02
297.146	110.12
425.3451	62.83
435.2593	90.8
819.1795	119.59
904.9297	56.57

Compound Label	Name	*m/z*	RT	Algorithm	Mass
Cpd 81: Tricin 7-[sinapoyl-(->2)-glucuronyl-(1->2)-glucuronide]	**Tricin 7-[sinapoyl-(->2)-glucuronyl-(1->2)-glucuronide]**	947.1926	16.296	Auto MS/MS	888.1767

MS Spectrum

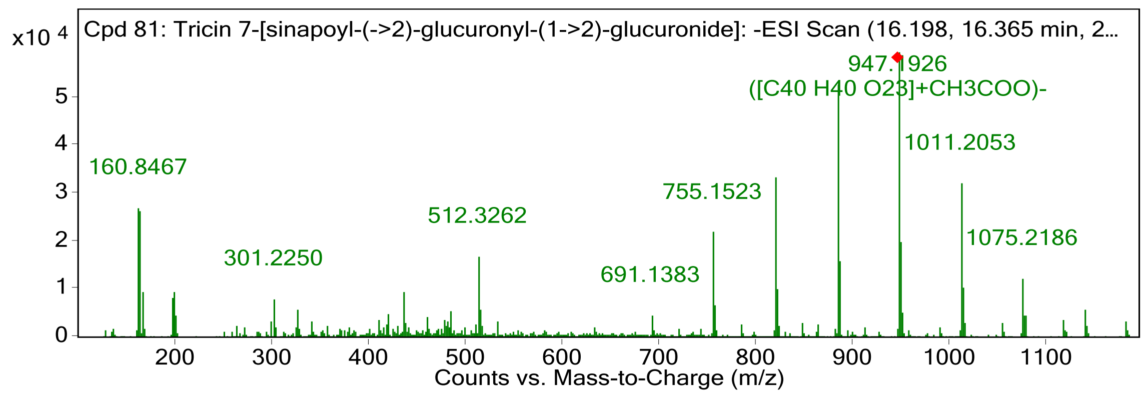

MS Zoomed Spectrum

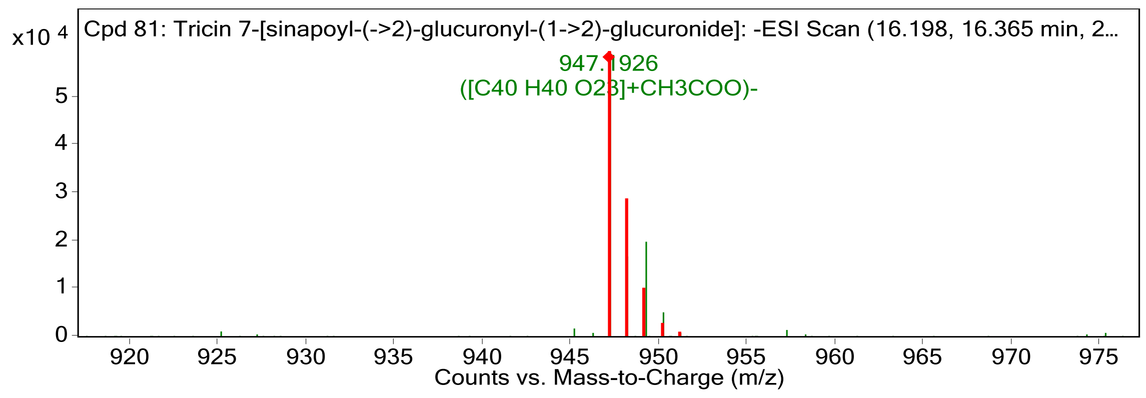

MS Spectrum Peak List

m/z	*Calc m/z*	Diff(ppm)	z	Abund	Formula	Ion
160.8467				27075.88		
162.8441				26271.02		
819.1662			1	33238.9		
883.1794			1	52679.2		
947.1926	947.2099	18.23	1	59384.39	C40 H40 O23	(M+CH3COO)-
948.1955	948.2133	18.73	1	16709.02	C40 H40 O23	(M+CH3COO)-
949.1909	949.2159	26.34	1	19978.71	C40 H40 O23	(M+CH3COO)-
950.1933	950.2186	26.59	1	5330.04	C40 H40 O23	(M+CH3COO)-
951.1985	951.2211	23.78	1	865.57	C40 H40 O23	(M+CH3COO)-
1011.2053			1	32179.02		

MSMS Spectrum

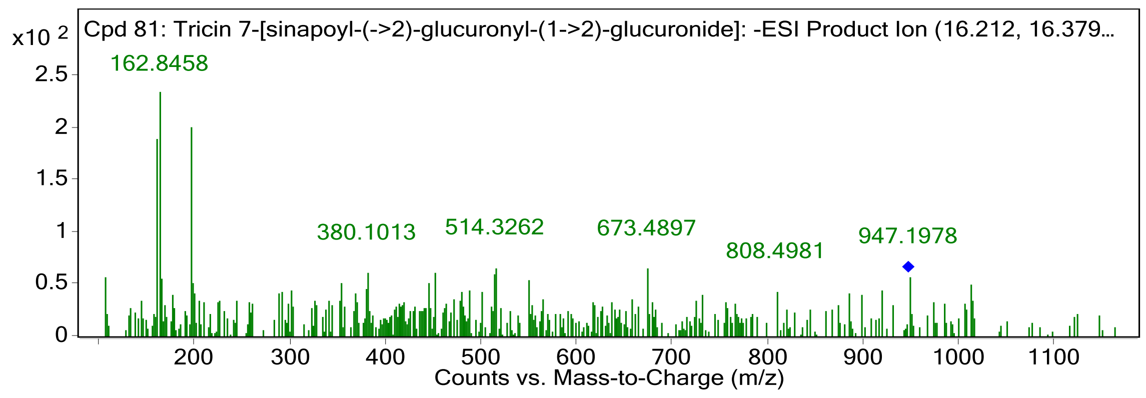

MS/MS Spectrum Peak List

m/z	Abund
106.3463	58.36
160.8461	190.55
162.8458	235.6
195.8159	200.83
380.1013	61.53
451.3513	62.53
512.3249	60.24
514.3262	66.54
673.4897	65.58
947.1978	58.04

Compound Structure

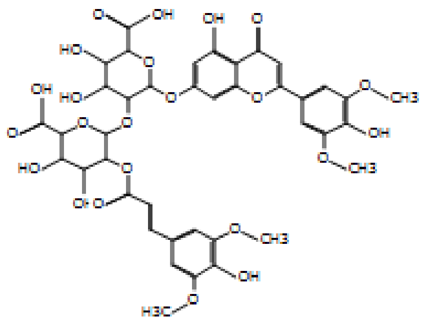

Compound Label	Name	*m/z*	RT	Algorithm	Mass
Cpd 82: 10-Deoxymethymycin	**10-Deoxymethymycin**	512.3261	16.356	Auto MS/MS	453.3102

MS Spectrum

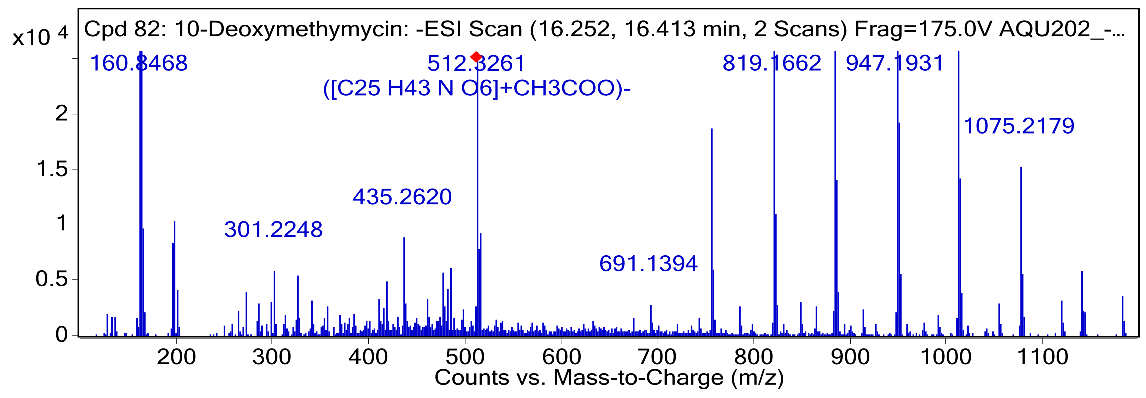

MS Zoomed Spectrum

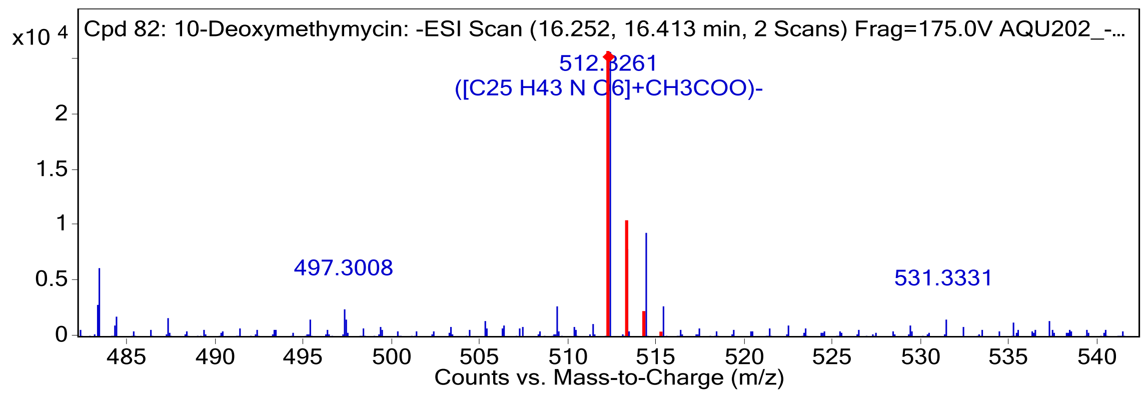

MS Spectrum Peak List

m/z	*Calc m/z*	Diff(ppm)	z	Abund	Formula	Ion
160.8468				29198.61		
512.3261	512.3229	-6.21	1	25640.59	C25 H43 N O6	(M+CH3COO)-
513.3295	513.3262	-6.41	1	7912.59	C25 H43 N O6	(M+CH3COO)-
514.3242	514.3289	9.13	1	9394.47	C25 H43 N O6	(M+CH3COO)-
515.3282	515.3316	6.48	1	2713.47	C25 H43 N O6	(M+CH3COO)-
516.3332	516.3342	1.89	1	682.12	C25 H43 N O6	(M+CH3COO)-
819.1662			1	33912.1		
883.1794			1	43271.09		
947.1931			1	60772.45		
1011.2059			1	45124.82		

MSMS Spectrum

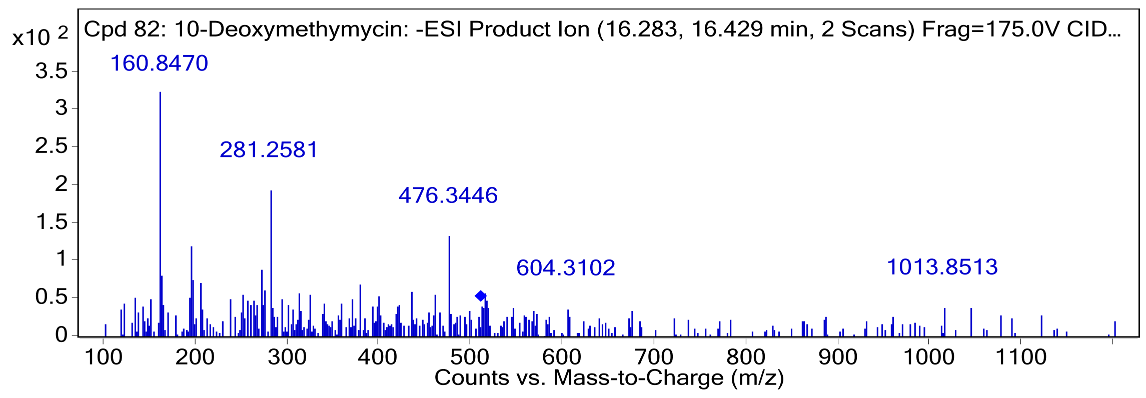

MS/MS Spectrum Peak List

m/z	z	Abund
160.847		324.9
162.8447		81.68
195.8154		120.96
197.8132		76.01
206.0878		71.57
271.2355		89.04
272.2394		81.92
281.2581	1	194.09
379.2231		69.38
476.3446		133.55

Compound Structure

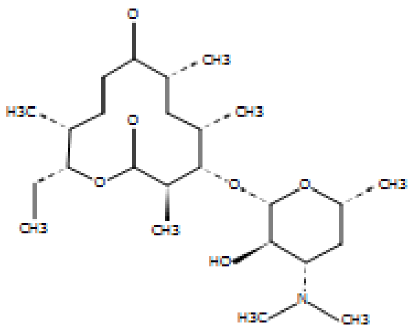

Compound Label	*m/z*	RT	Algorithm
Compound 83	1011.2054	16.464	Auto MS/MS

MS Spectrum

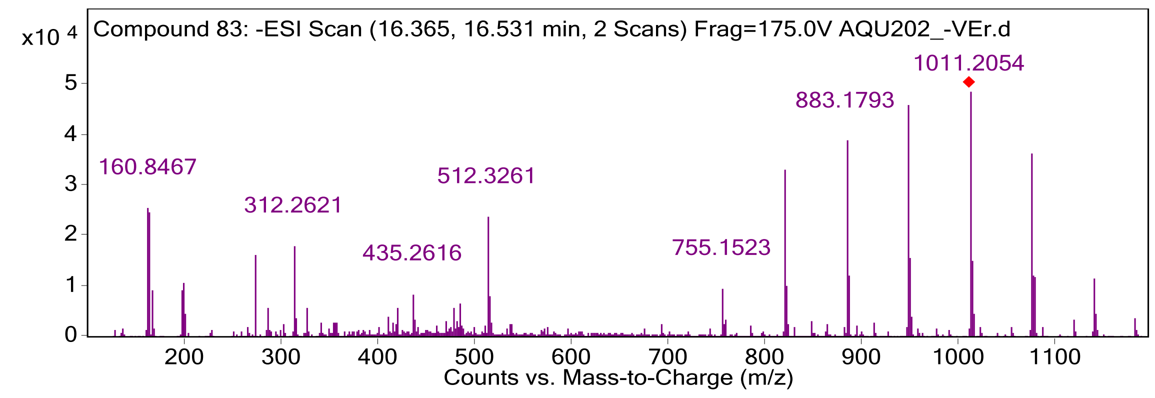

MS Zoomed Spectrum

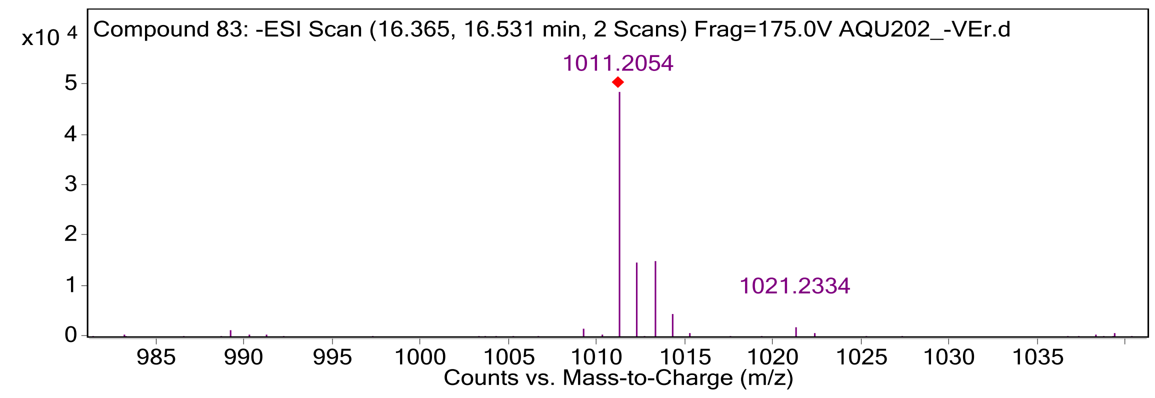

MS Spectrum Peak List

m/z	z	Abund
160.8467		25659.34
819.166	1	33278.84
883.1793	1	38913.23
947.1924	1	46027.95
1011.2054	1	48651.39
1012.2085	1	14821.17
1013.2035	1	15188.6
1014.2048	1	4711.41
1015.2089	1	818.17
1075.2184	1	36333.22

MSMS Spectrum

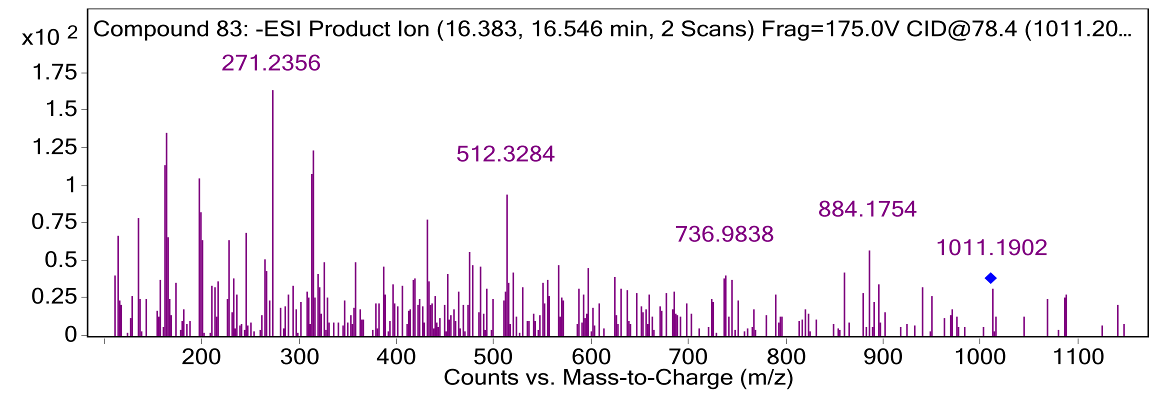

MS/MS Spectrum Peak List

m/z	z	Abund
134.0539		79.53
160.8458		114.48
162.8449		135.45
195.8148		105.01
197.8135		83.21
271.2356		163.51
312.26		108.49
313.2665	1	124.12
430.0413		77.81
512.3284		94.58

Compound Label	*m/z*	RT	Algorithm
Compound 84	1075.2191	16.52	Auto MS/MS

MS Spectrum

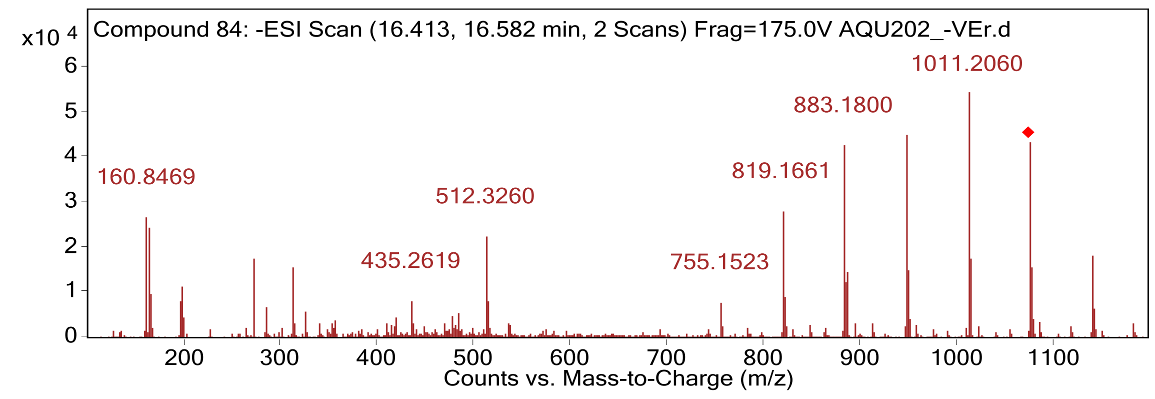

MS Zoomed Spectrum

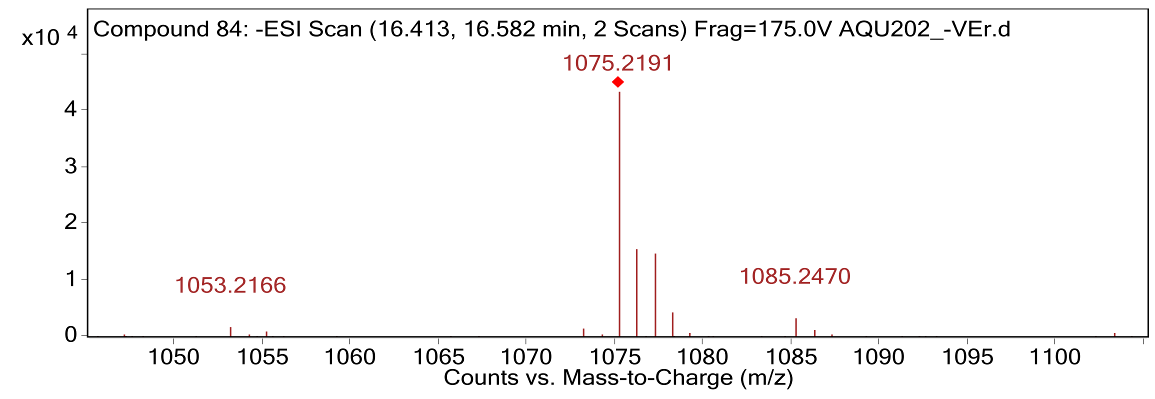

MS Spectrum Peak List

m/z	z	Abund
160.8469		26675.83
819.1661	1	28023.45
883.18	1	42662.07
947.1927	1	45122.39
1011.206	1	54535.13
1075.2191	1	43499.1
1076.2227	1	15511.74
1077.2179	1	14942.54
1078.2184	1	4306.74

1079.2243	1	877.15

MSMS Spectrum

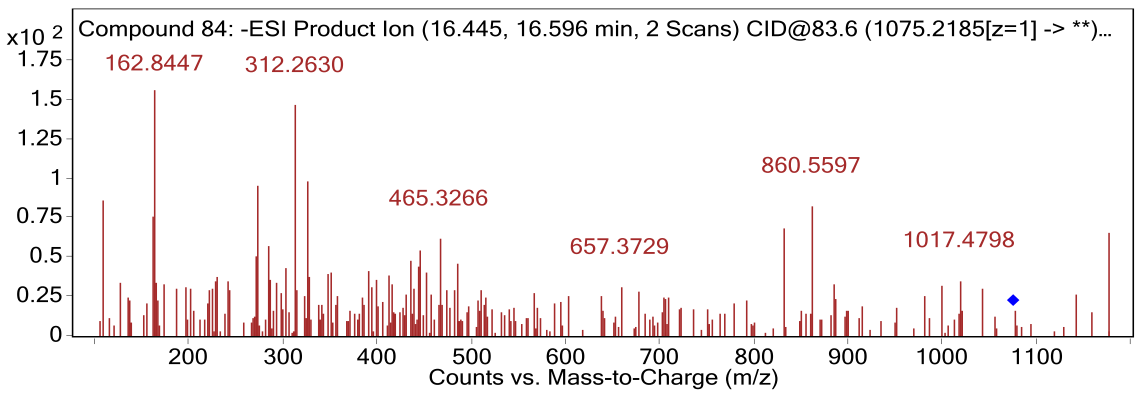

MS/MS Spectrum Peak List

m/z	z	Abund
108.9062		86.67
160.8464		76.82
162.8447		156.01
272.2411		95.9
312.263	1	147.01

m/z	z	Abund
325.1932	1	98.82
465.3266		62.23
831.1059		68.45
860.5597		83.09
1174.4425		65.81

Compound Label	*m/z*	RT	Algorithm
Compound 85	271.2351	16.583	Auto MS/MS

MS Spectrum

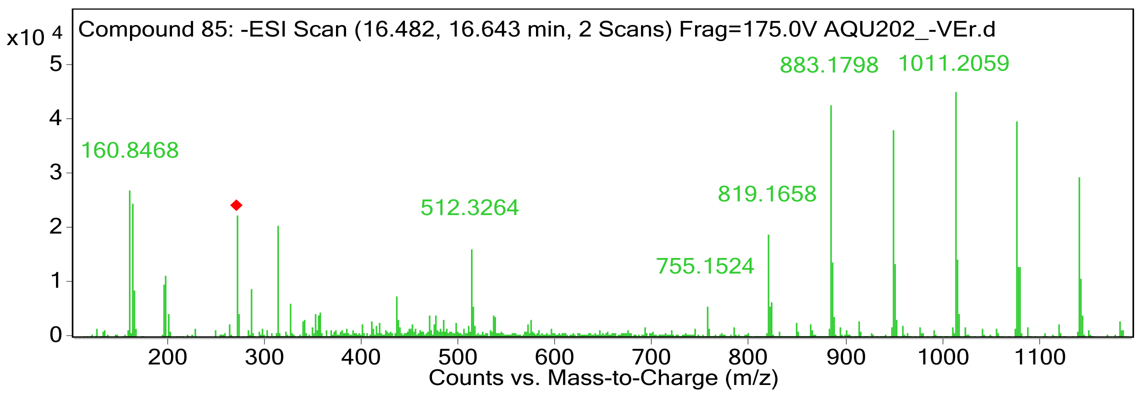

MS Zoomed Spectrum

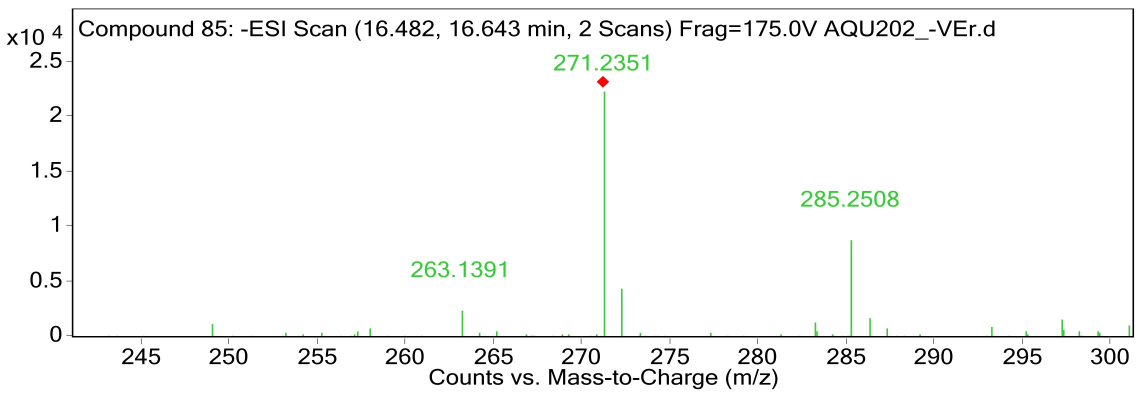

MS Spectrum Peak List

m/z	z	Abund
160.8468		26966.16
162.8441		24570.36
271.2351	1	22377.17
272.2381	1	4383.23
273.241	1	387.5
883.1798	1	42701.04
947.1927	1	38075.64
1011.2059	1	45248.53
1075.2189	1	39861.31
1139.2319	1	29383.72

MSMS Spectrum

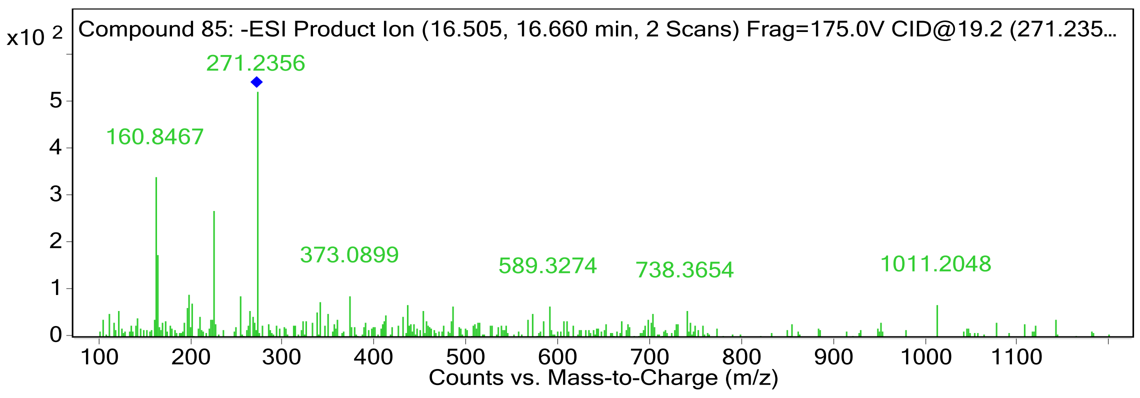

MS/MS Spectrum Peak List

m/z	z	Abund
160.8467		340.72
162.845		173.95
197.8172		89.43
200.4864		71.19
225.2306	1	270.05
253.2217		87.54
271.2356	1	523.88
340.2907		75.04
373.0899		88.16
1011.2048		68.92

Compound Label	*m/z*	RT	Algorithm
Compound 86	1139.2315	16.825	Auto MS/MS

MS Spectrum

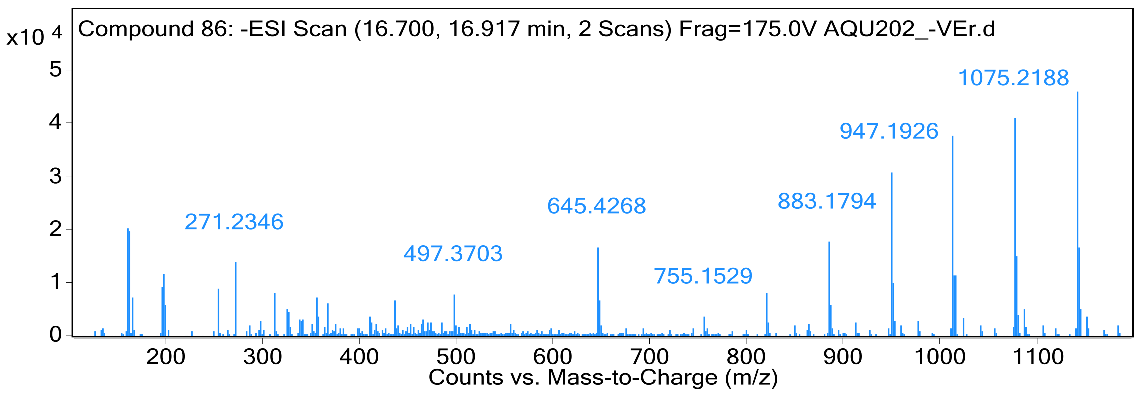

MS Zoomed Spectrum

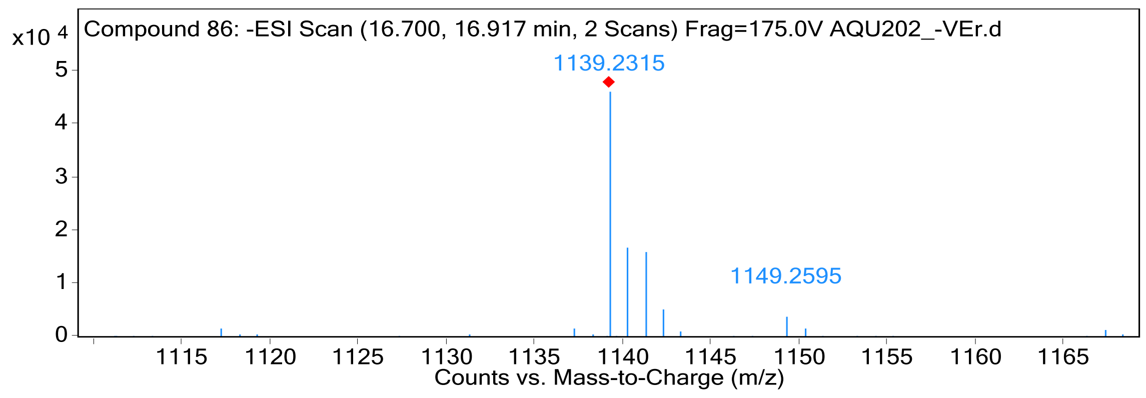

MS Spectrum Peak List

m/z	z	Abund
160.8466		20600.66
162.8441		19914.63
947.1926	1	31101.22
1011.2056	1	38010.69
1075.2188	1	41166.94
1139.2315	1	46255.81
1140.2348	1	16960.37
1141.2302	1	15973.81
1142.233	1	5361.54
1143.2329	1	1180.62

MSMS Spectrum

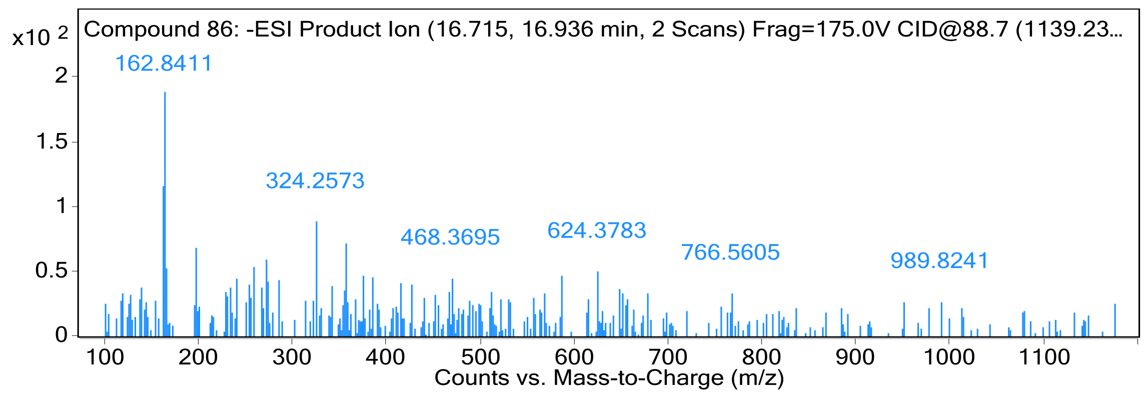

MS/MS Spectrum Peak List

m/z	Abund
160.8453	116.35
162.8411	189.69
164.8393	53.19
195.815	69.4
257.2692	54.2
271.2322	59.87
324.2573	89.28
325.1872	57.24
355.2894	72.53
624.3783	50.88

Compound Label	*m/z*	RT	Algorithm
Compound 87	1075.2193	16.855	Auto MS/MS

MS Spectrum

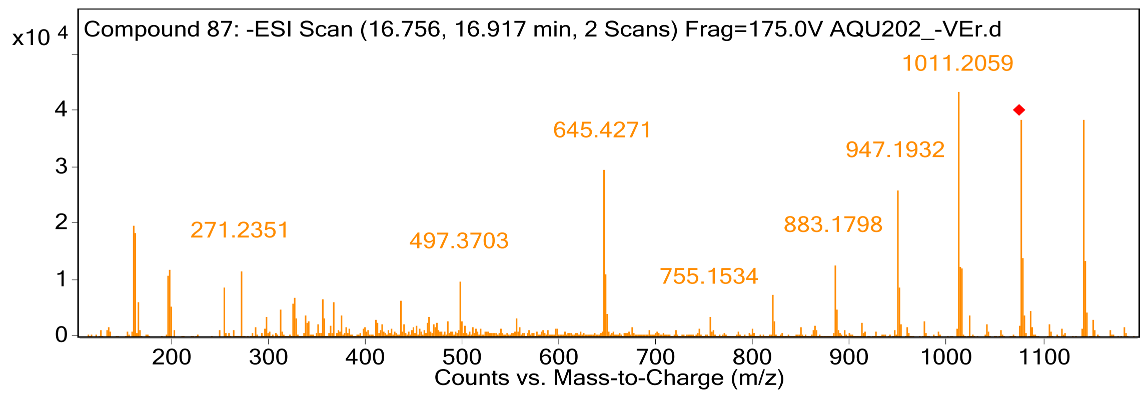

MS Zoomed Spectrum

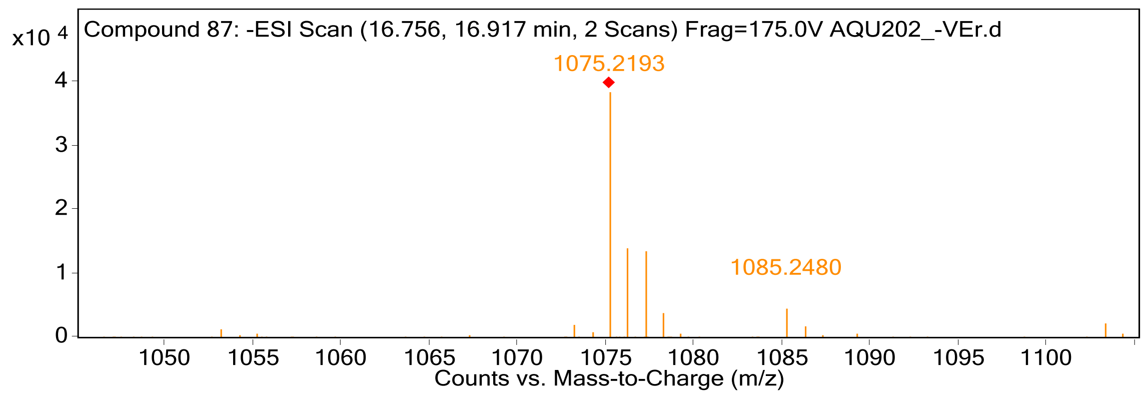

MS Spectrum Peak List

m/z	z	Abund
160.8467		19693.81
645.4271	1	29553.26
947.1932	1	26038.59
1011.2059	1	43534.3
1075.2193	1	38492.8
1076.2222	1	14129.08
1077.2176	1	13523.42
1078.2182	1	3955.88
1079.2203	1	684.58
1139.2315	1	38533.92

MSMS Spectrum

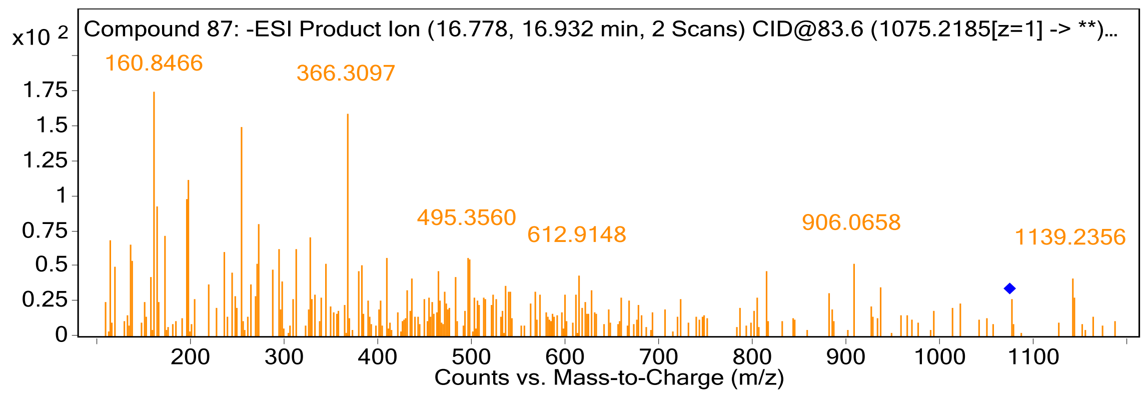

MS/MS Spectrum Peak List

m/z	Abund
113.8539	69.8
160.8466	175.83
162.8442	93.03
172.2228	72.01
195.8182	99
197.8114	112.47
253.2215	149.78
271.2343	80.69
327.2971	71.29
366.3097	159.73

Compound Label	*m/z*	RT	Algorithm
Compound 88	1139.2316	17.193	Auto MS/MS

MS Spectrum

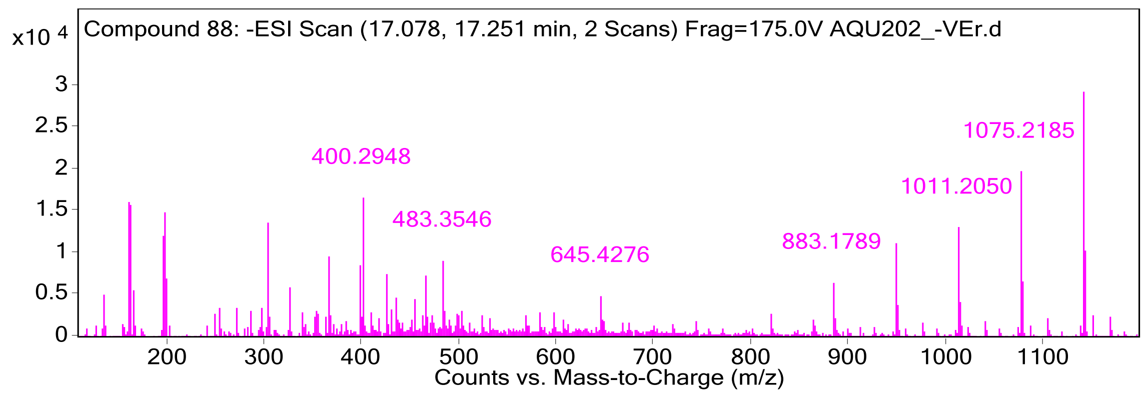

MS Zoomed Spectrum

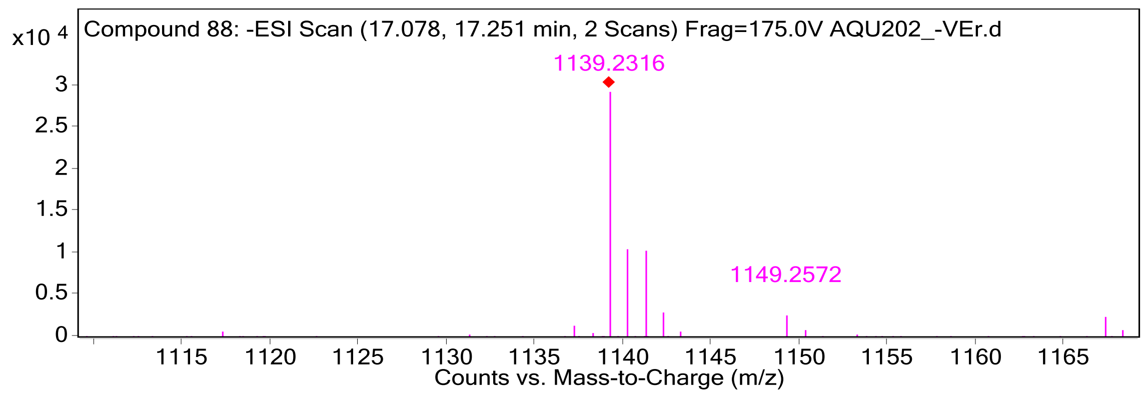

MS Spectrum Peak List

m/z	z	Abund
160.8467		16060.25
162.844		15825.32
197.8135		14803.33
400.2948	1	16688.54
1075.2185	1	19804.24
1139.2316	1	29288.55
1140.2344	1	10589.9
1141.2303	1	10282.96
1142.2329	1	2970.05
1143.2355	1	734.94

MSMS Spectrum

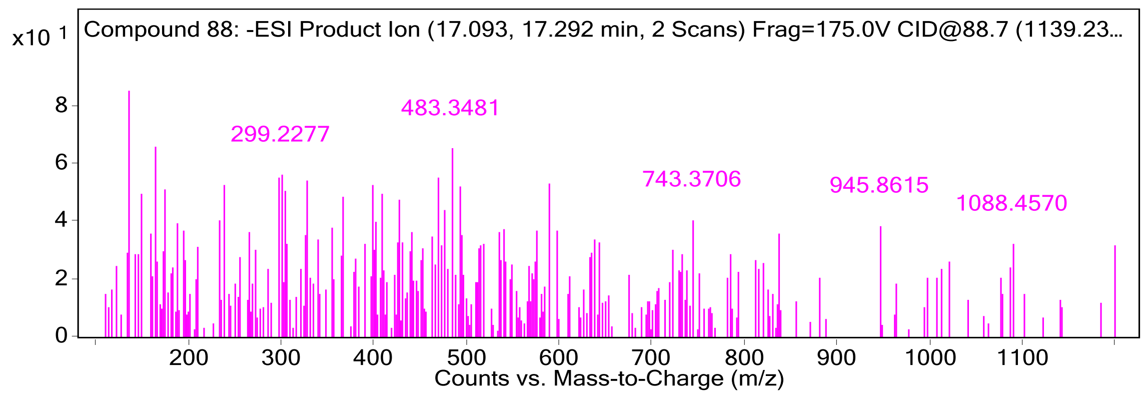

MS/MS Spectrum Peak List

m/z	Abund
134.898	85.49
162.8442	66.17
238.1539	53.05
296.2586	55.56
299.2277	56.49
326.1882	54.65
397.3418	52.82
468.2038	55.5
483.3481	65.6
588.2295	53.63

Compound Label	Name	*m/z*	RT	Algorithm	Mass
Cpd 89: Asterosterol	**Asterosterol**	369.3099	17.457	Auto MS/MS	370.3172

MS Spectrum

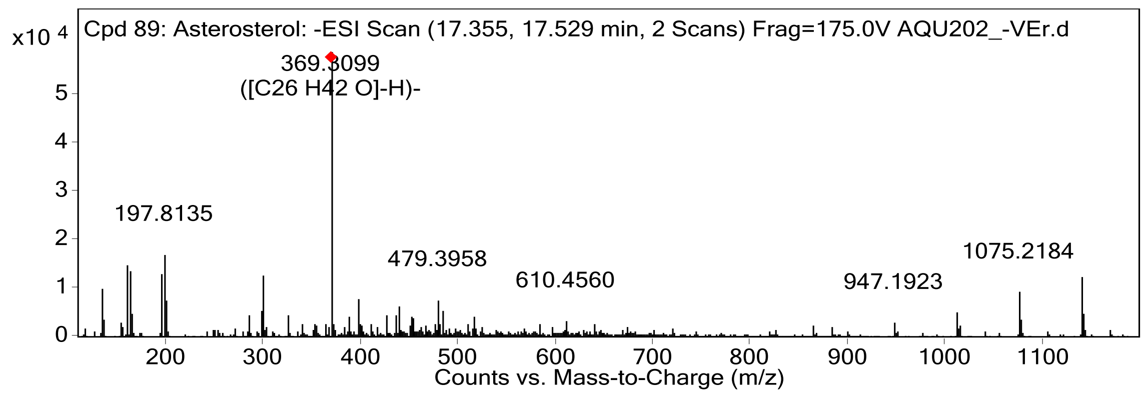

MS Zoomed Spectrum

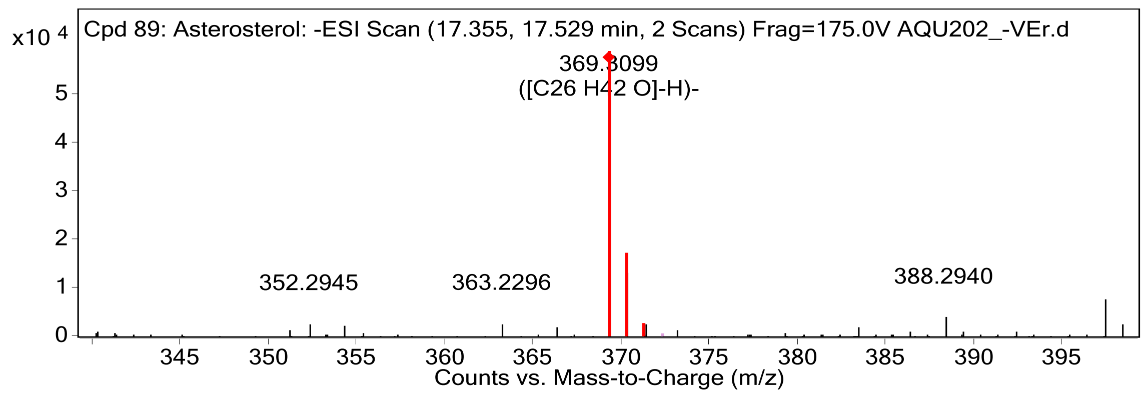

MS Spectrum Peak List

m/z	*Calc m/z*	Diff(ppm)	z	Abund	Formula	Ion
134.8987			1	10078.66		
160.8468				14998.63		
162.8441				13634.77		
195.8164				12999.88		
197.8135				17103.23		
299.2664			1	12759.53		
369.3099	369.3163	17.4	1	58844.6	C26 H42 O	(M-H)-
370.3132	370.3197	17.53	1	13483.72	C26 H42 O	(M-H)-
371.318	371.323	13.29	1	2754.18	C26 H42 O	(M-H)-
1139.2312			1	12439.65		

MSMS Spectrum

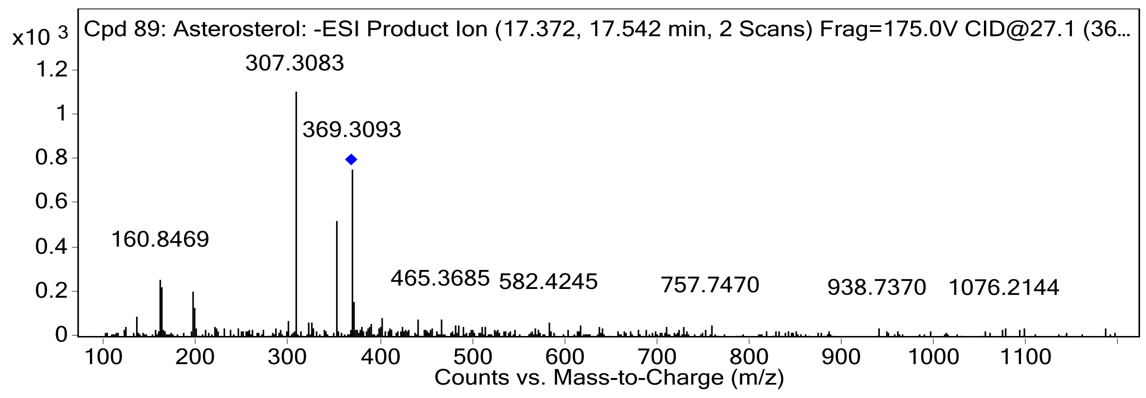

MS/MS Spectrum Peak List

m/z	z	Abund
160.8469		256.06
162.8441		224.83
195.8159		206.73
197.814		133.07
307.3083	1	1106.18
308.3135	1	354.51
351.2987	1	522.99
352.2998	1	204.41
369.3093	1	751.18
370.3126	1	157.78

Compound Structure

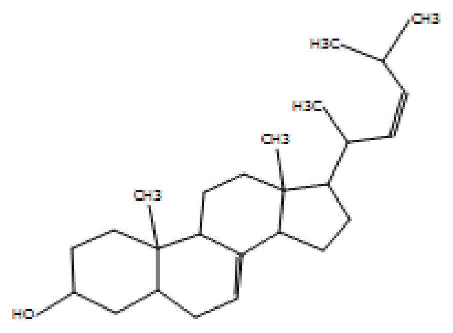

Compound Label	Name	*m/z*	RT	Algorithm	Mass
Cpd 90: alpha-Amyrin acetate	**alpha-Amyrin acetate**	467.3956	18.075	Auto MS/MS	468.4028

MS Spectrum

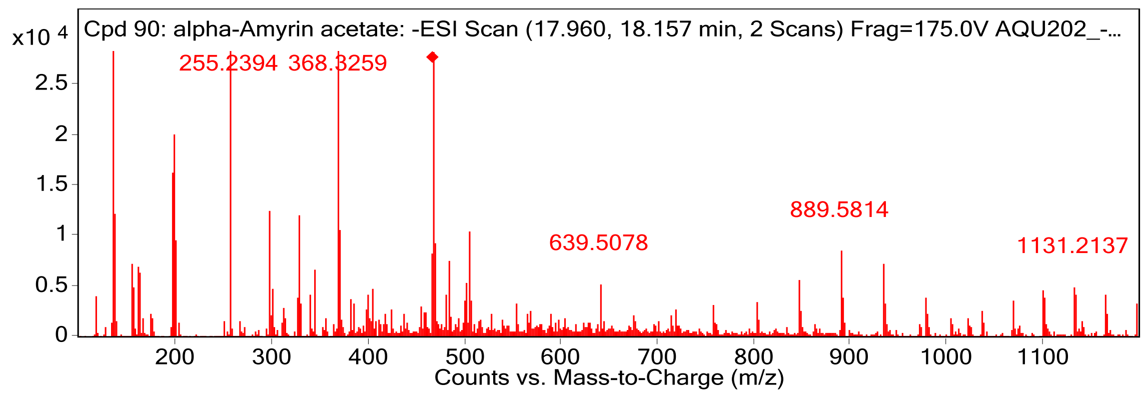

MS Zoomed Spectrum

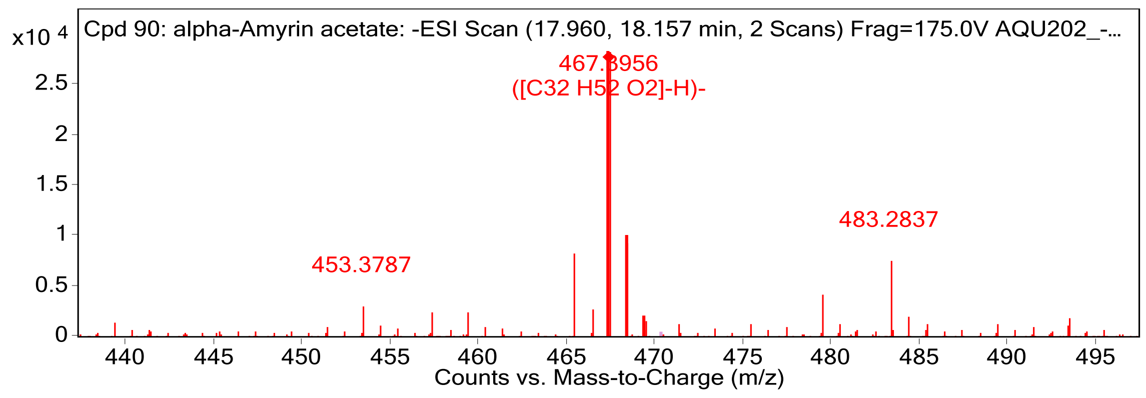

MS Spectrum Peak List

m/z	*Calc m/z*	Diff(ppm)	z	Abund	Formula	Ion
134.8986			1	36688.99		
136.8957			1	12241.49		
195.8161				16254.56		
197.8133				20109.27		
255.2394			1	28661.6		
297.16			1	12469.59		
368.3259			1	46493.46		
467.3956	467.3895	-13.21	1	28255.96	C32 H52 O2	(M-H)-
468.3989	468.3929	-12.85	1	9353.98	C32 H52 O2	(M-H)-
469.401	469.3961	-10.36	1	1560.67	C32 H52 O2	(M-H)-

MSMS Spectrum

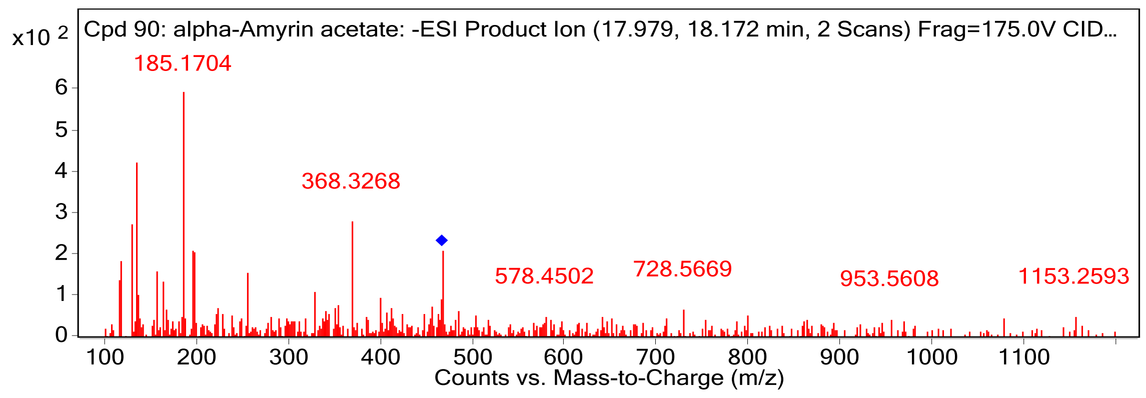

MS/MS Spectrum Peak List

m/z	z	Abund
116.9318		184.71
130.0917		273.36
134.8985		422
155.8692		160.61
185.1704	1	595.54
195.8155		211.5
197.8116		206.23
255.2376		158.14
368.3268	1	279.73
467.3916		210.94

Compound Structure

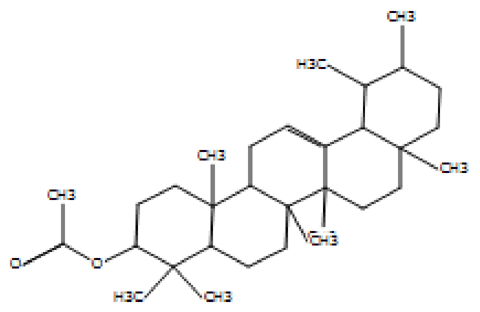

Compound Label	*m/z*	RT	Algorithm
Compound 91	327.2985	18.076	Auto MS/MS

MS Spectrum

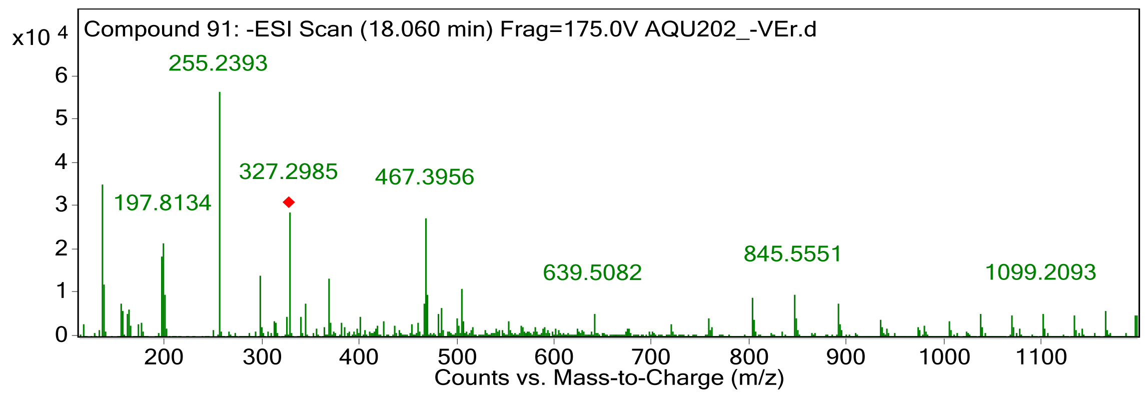

MS Zoomed Spectrum

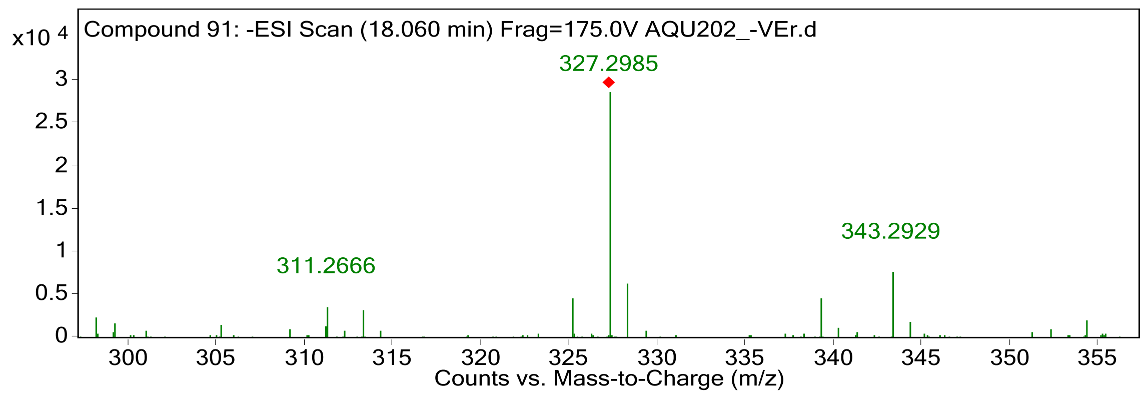

MS Spectrum Peak List

m/z	z	Abund
134.8986	1	35307.38
195.8164		18597.72
197.8134		21539.48
255.2393	1	56547.21
297.1601	1	14195.92
327.2985	1	28748.53
328.3019	1	6389.22
329.3057	1	939.18
368.3256	1	13557.07

467.3956	1	27503.91

MSMS Spectrum

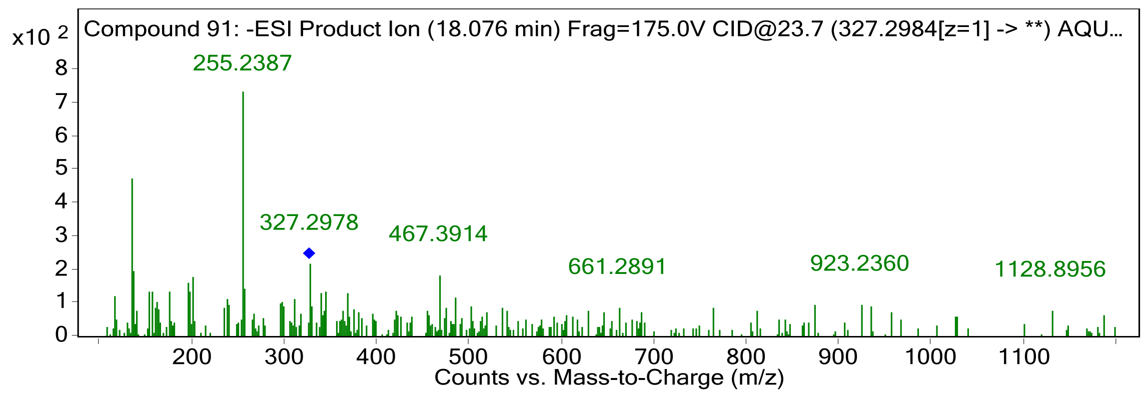

MS/MS Spectrum Peak List

m/z	z	Abund
134.8982		474.37
136.895		198.25
153.8731		137.72
195.8152		162.26
197.8128		138.03
199.8107		178.74
255.2387	1	736.86
256.2427	1	144.2
327.2978	1	221.17
467.3914		187.02

Compound Label	*m/z*	RT	Algorithm
Compound 92	368.3259	18.179	Auto MS/MS

MS Spectrum

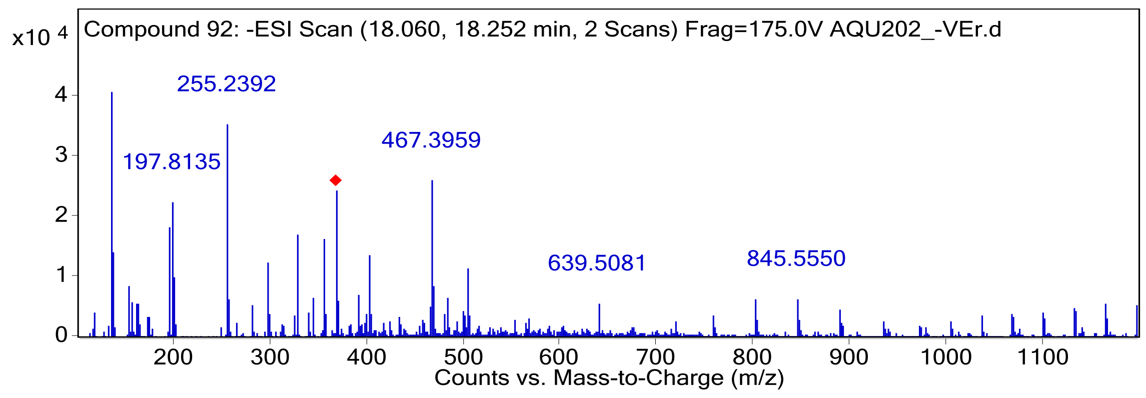

MS Zoomed Spectrum

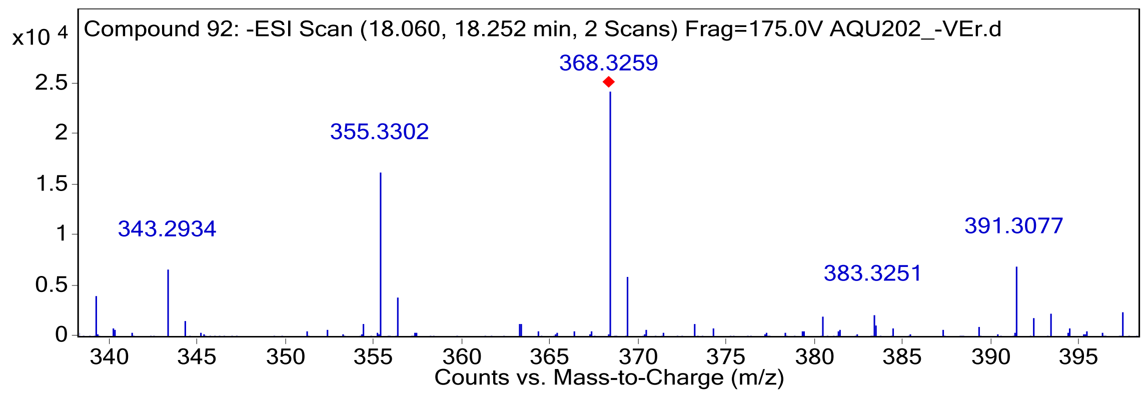

MS Spectrum Peak List

m/z	z	Abund
134.8986	1	40712.54
195.8163		18294.29
197.8135		22356.18
255.2392	1	35293.91
327.2985	1	16946.52
355.3302	1	16272.12
368.3259	1	24276.02
369.3289	1	5967.3
370.3317	1	728.29
467.3959	1	25945.48

MSMS Spectrum

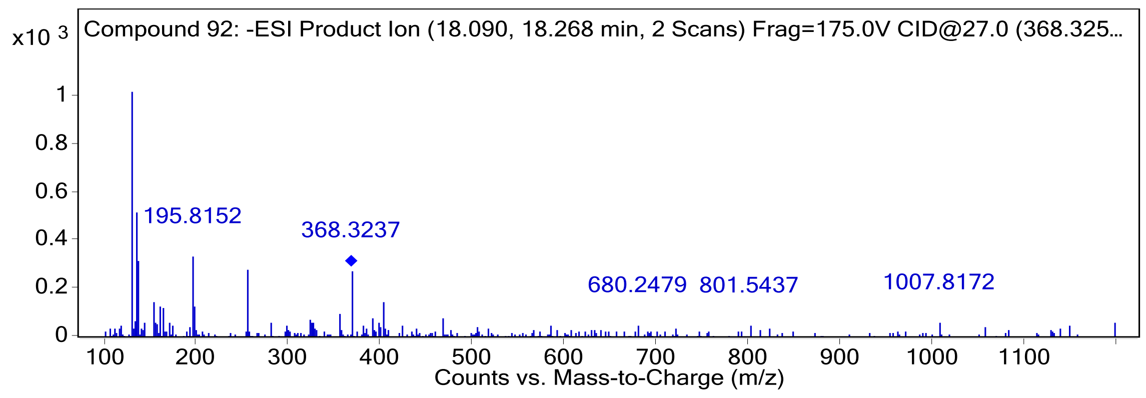

MS/MS Spectrum Peak List

m/z	z	Abund
130.0917	1	1020.77
134.8982	1	520.75
136.8955	1	316.39
153.8724		146.1
160.8472		127.21
195.8152		334.95
197.8115		128.51
255.238		281.27
368.3237	1	276.26
402.3085	1	144.42

Compound Label	m/z	RT	Algorithm
Compound 93	355.3301	18.33	Auto MS/MS

MS Spectrum

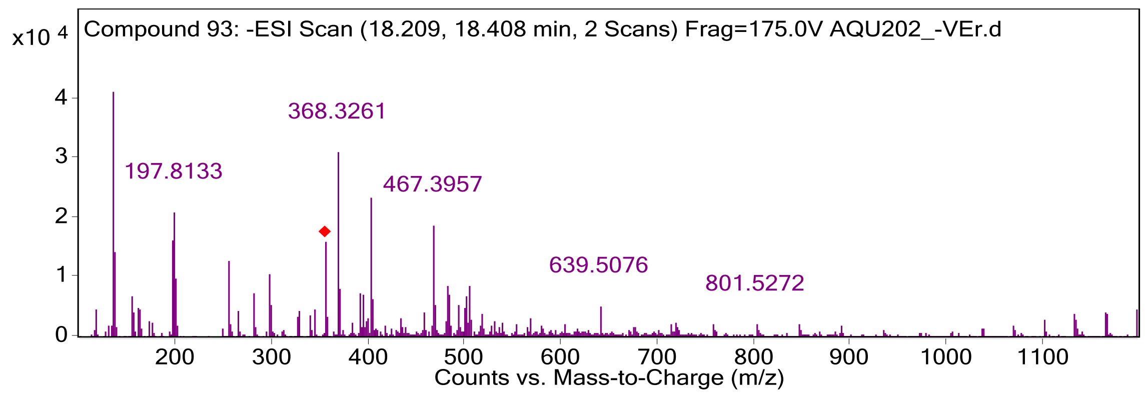

MS Zoomed Spectrum

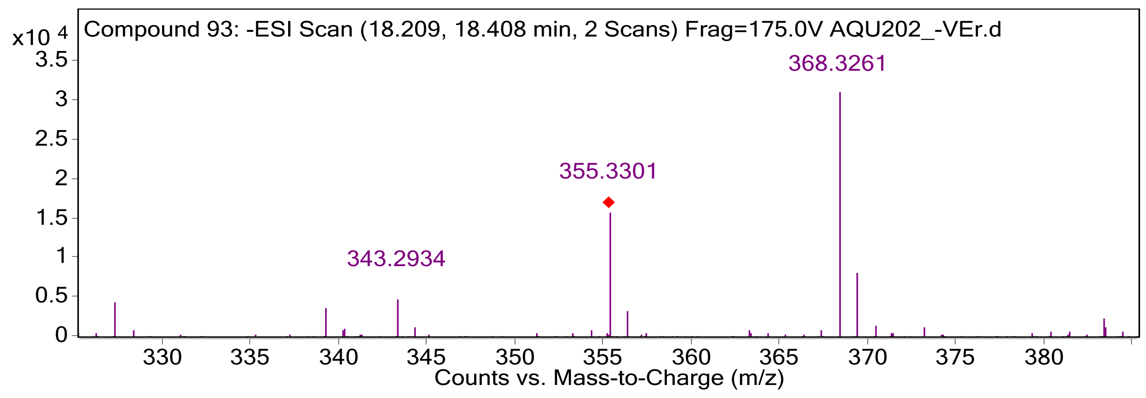

MS Spectrum Peak List

m/z	z	Abund
134.8986	1	41270.07
136.8957	1	14420.25
195.8162		16406.88
197.8133		21045.2
355.3301	1	15922.35
356.3337	1	3347.3
357.3358	1	537.16
368.3261	1	31163.9

402.3107	1	23327.5
467.3957	1	18866.99

MSMS Spectrum

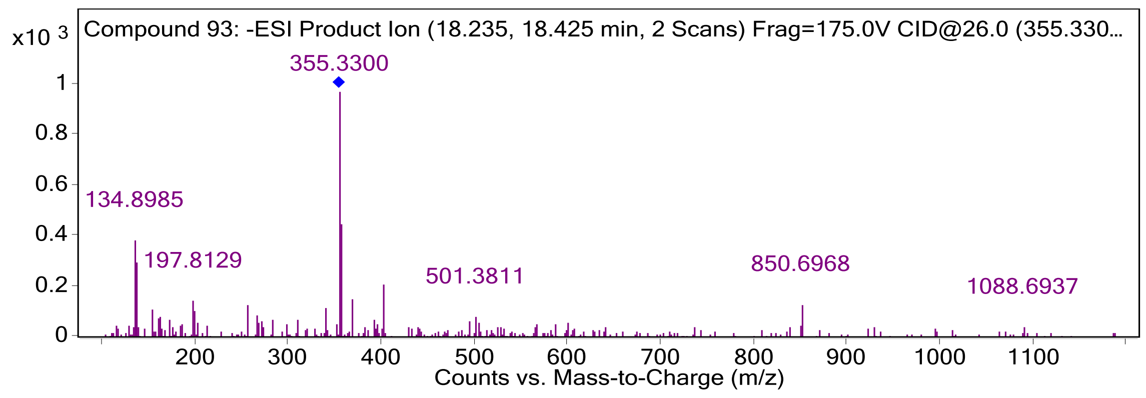

MS/MS Spectrum Peak List

m/z	z	Abund
134.8985		382.71
136.8959	1	294.14
197.8129		142.55
255.2384		125.44
339.2065		115.78
355.33	1	969.83
356.335	1	444.68
368.3254		149.13
402.3101		207.95
850.6968		128.95

Compound Label	*m/z*	RT	Algorithm
Compound 94	402.3109	18.366	Auto MS/MS

MS Spectrum

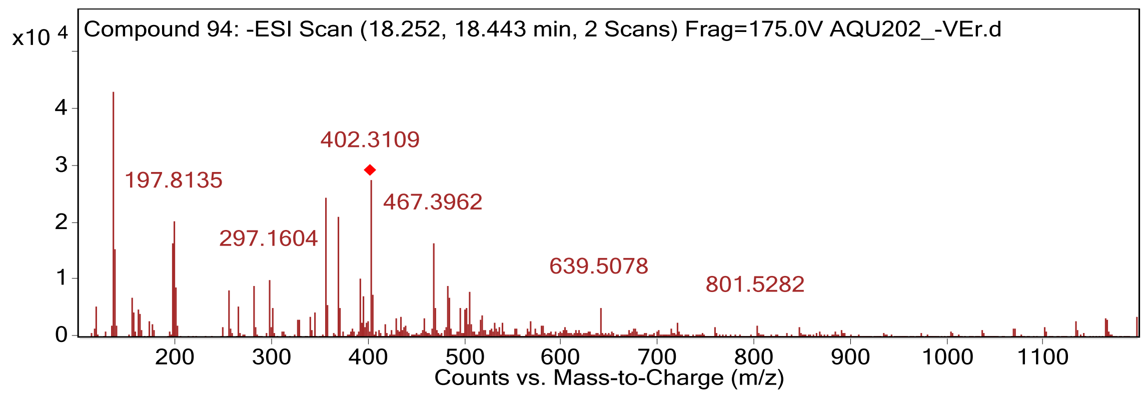

MS Zoomed Spectrum

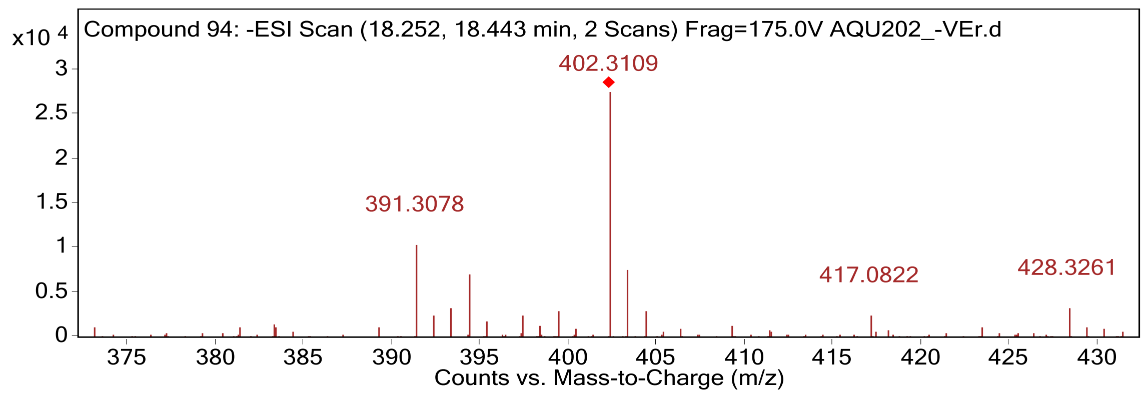

MS Spectrum Peak List

m/z	z	Abund
134.8986	1	43196.38
195.8162		16592.67
197.8135		20449.27
355.3301	1	24544.95
368.3259	1	21057.9
402.3109	1	27581.27
403.314	1	7576.47
404.3071	1	3036.53
405.3086	1	617.92
467.3962	1	16597.1

MSMS Spectrum

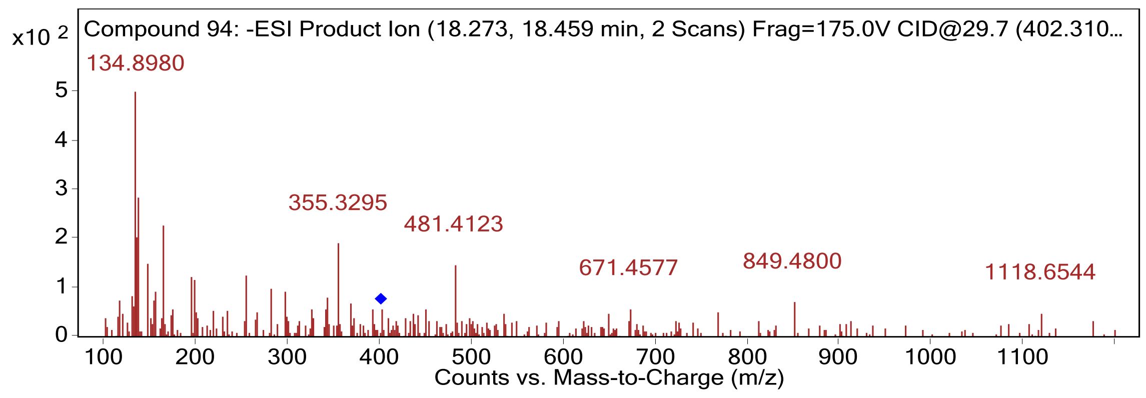

MS/MS Spectrum Peak List

m/z	z	Abund
134.898		501.75
135.8984		202.91
136.8957		286.43
147.0493	1	150.93
164.0761		227.11
195.815		122.5
197.8127		116.06
255.2402		124.85
355.3295		191.13
481.4123		147.51

Compound Label	Name	*m/z*	RT	Algorithm	Mass
Cpd 95: alpha-Amyrin acetate	**alpha-Amyrin acetate**	467.3959	18.373	Auto MS/MS	468.4027

MS Spectrum

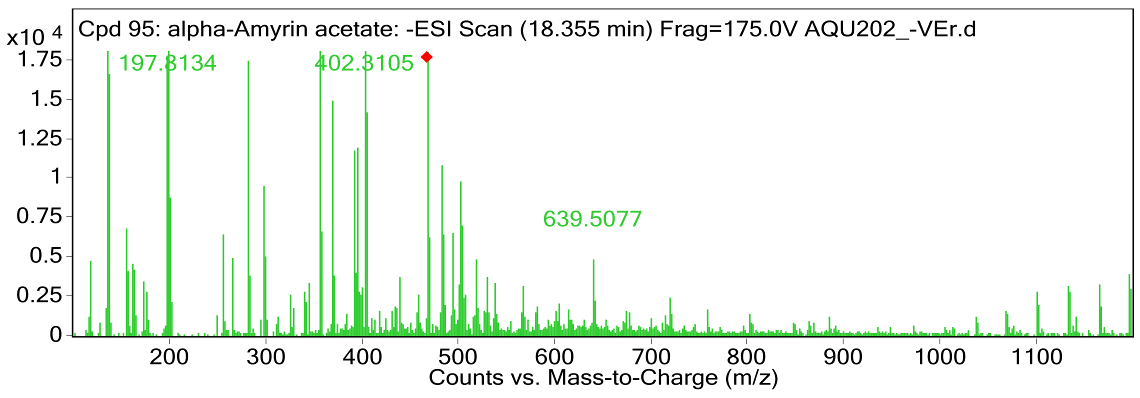

MS Zoomed Spectrum

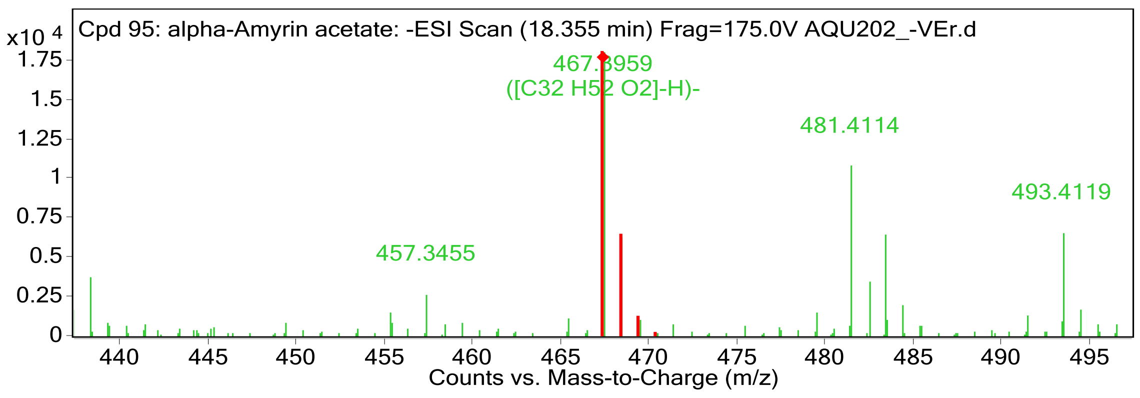

MS Spectrum Peak List

m/z	*Calc m/z*	Diff(ppm)	z	Abund	Formula	Ion
134.8985			1	45741.93		
195.8161				17517.88		
197.8134				22345.96		
281.2554			1	17452.31		
355.3302			1	27108.47		
402.3105			1	55050.03		
467.3959	467.3895	-13.71	1	18039.13	C32 H52 O2	(M-H)-
468.3983	468.3929	-11.54	1	6358.8	C32 H52 O2	(M-H)-
469.4005	469.3961	-9.28	1	1121.87	C32 H52 O2	(M-H)-
470.3972	470.3992	4.38	1	293.77	C32 H52 O2	(M-H)-

MSMS Spectrum

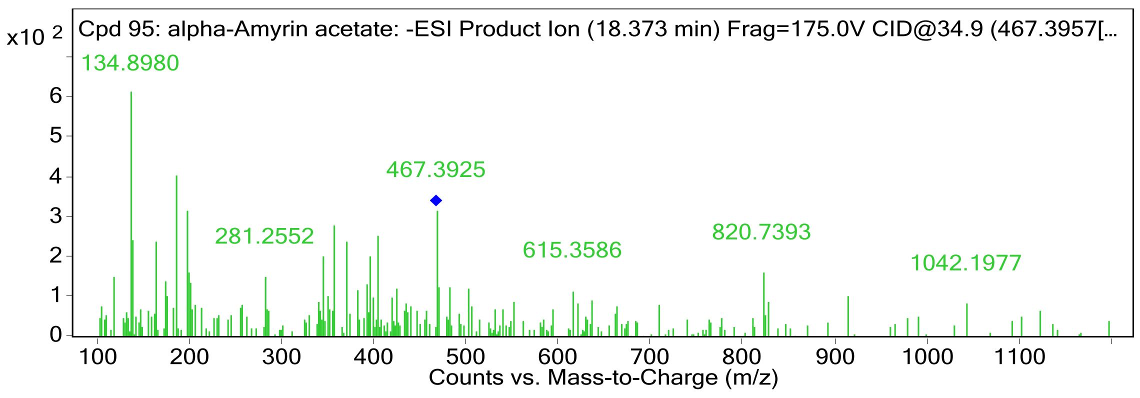

MS/MS Spectrum Peak List

m/z	z	Abund
134.898		617.55
136.8957		242.96
162.8426		241.68
185.1709		404.4
195.8159		317.94
355.328		280.17
368.3238		241.11
394.3376		204.73
402.3081	1	256.01
467.3925		318.22

Compound Structure

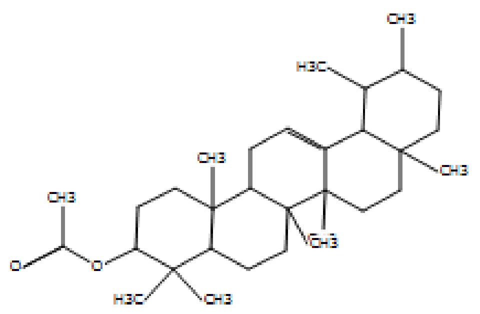

Compound Label	Name	*m/z*	RT	Algorithm	Mass
Cpd 96: N-[2-(1H-Indol-3-yl)ethyl]docosanamide	**N-[2-(1H-Indol-3-yl)ethyl]docosanamide**	481.4115	18.47	Auto MS/MS	482.4186

MS Spectrum

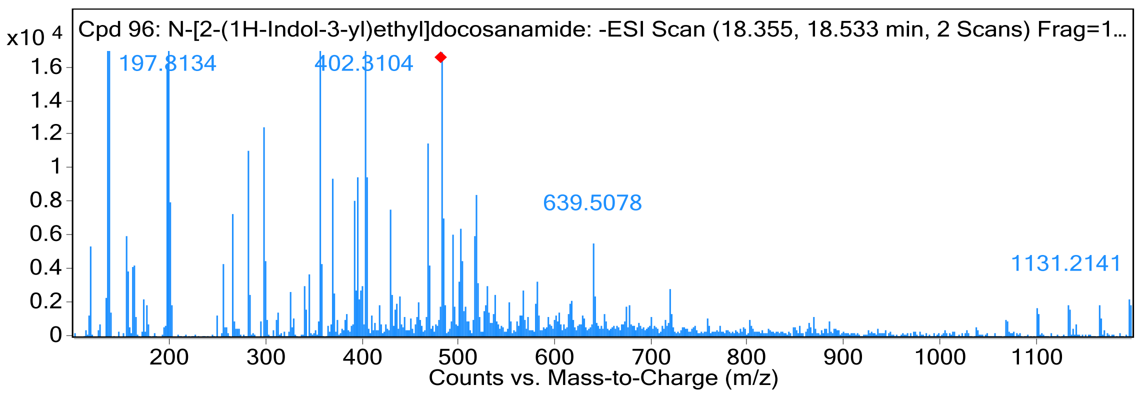

MS Zoomed Spectrum

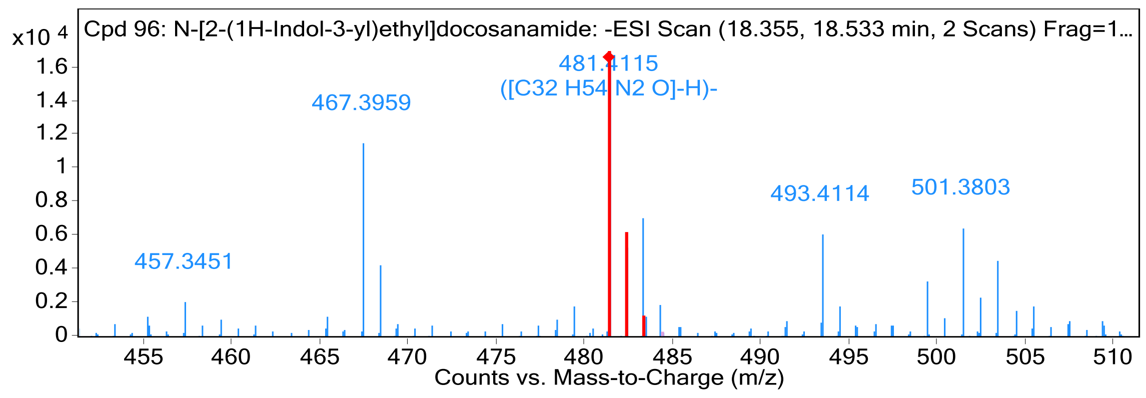

MS Spectrum Peak List

m/z	*Calc m/z*	Diff(ppm)	z	Abund	Formula	Ion
134.8986			1	51469.61		
136.8957			1	17277.17		
195.8161				16206.87		
197.8134				18859.78		
297.1604			1	12473		
355.3302			1	17875.84		
402.3104			1	36415.71		
481.4115	481.4163	10.11	1	16926.55	C32 H54 N2 O	(M-H)-
482.4147	482.4196	10.22	1	5330.89	C32 H54 N2 O	(M-H)-
483.4155	483.4228	15.21	1	1263.67	C32 H54 N2 O	(M-H)-

MSMS Spectrum

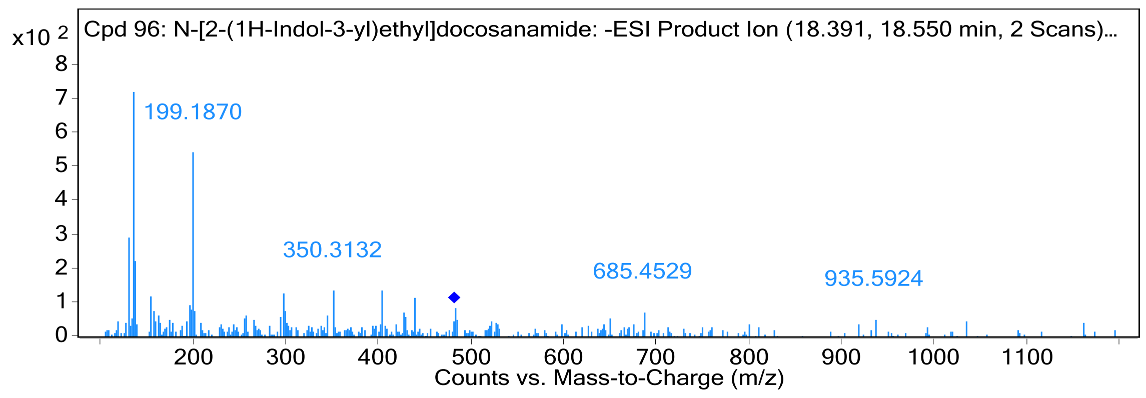

MS/MS Spectrum Peak List

m/z	z	Abund
130.0922	1	292.77
134.8985		721.97
136.8952	1	225
153.8733		120.94
195.8175		94.75
199.187		543.43
297.1595		129.34
350.3132	1	136.81
402.31		136.38
437.4224		117.27

Compound Structure

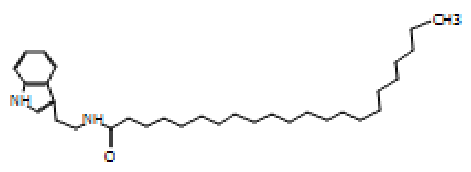

Compound Label	Name	*m/z*	RT	Algorithm	Mass
Cpd 97: N-[2-(1H-Indol-3-yl)ethyl]docosanamide	**N-[2-(1H-Indol-3-yl)ethyl]docosanamide**	481.4115	18.72	Auto MS/MS	482.4188

MS Spectrum

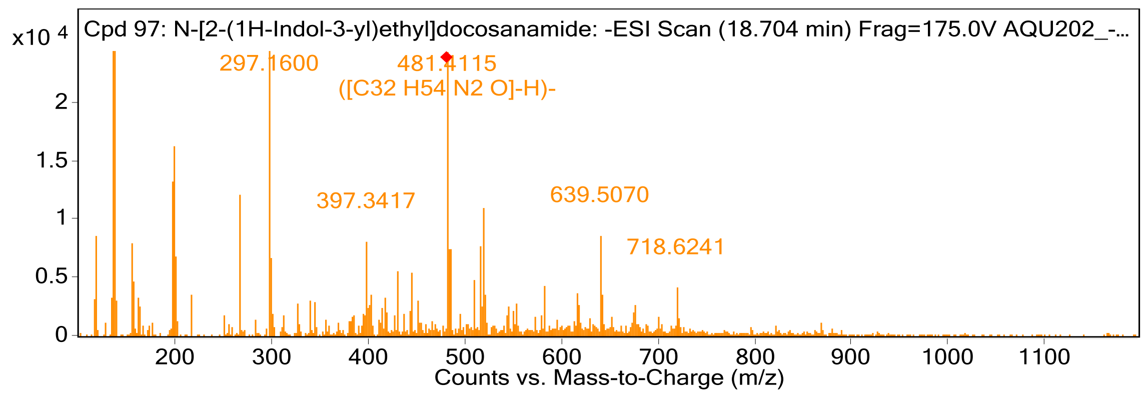

MS Zoomed Spectrum

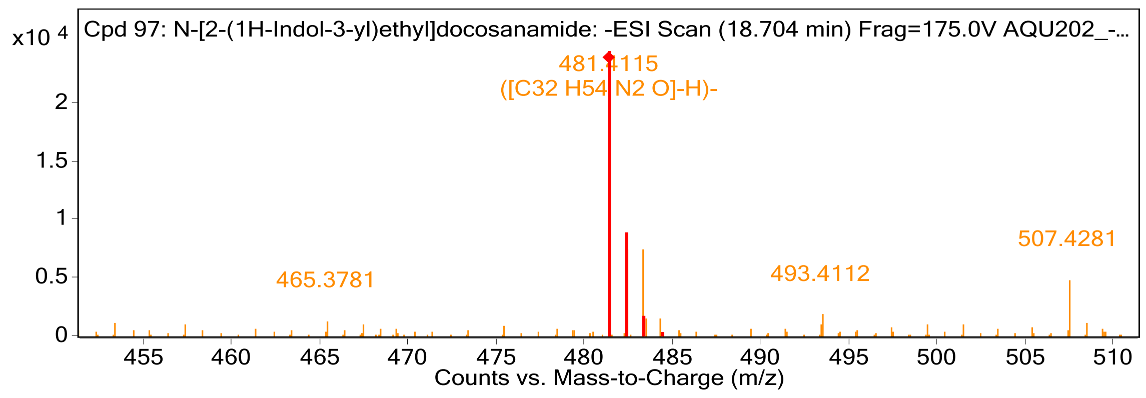

MS Spectrum Peak List

m/z	*Calc m/z*	Diff(ppm)	z	Abund	Formula	Ion
134.8986			1	84345.1		
136.8957			1	30920.66		
195.8161				13390.1		
197.8135				16322.91		
265.1544			1	12202.14		
297.16			1	37376.13		
481.4115	481.4163	9.97	1	24400.29	C32 H54 N2 O	(M-H)-

482.4147	482.4196	10.26	1	7544.42	C32 H54 N2 O	(M-H)-
483.4179	483.4228	10.18	1	1683.75	C32 H54 N2 O	(M-H)-
484.4211	484.4259	9.97	1	342.68	C32 H54 N2 O	(M-H)-

MSMS Spectrum

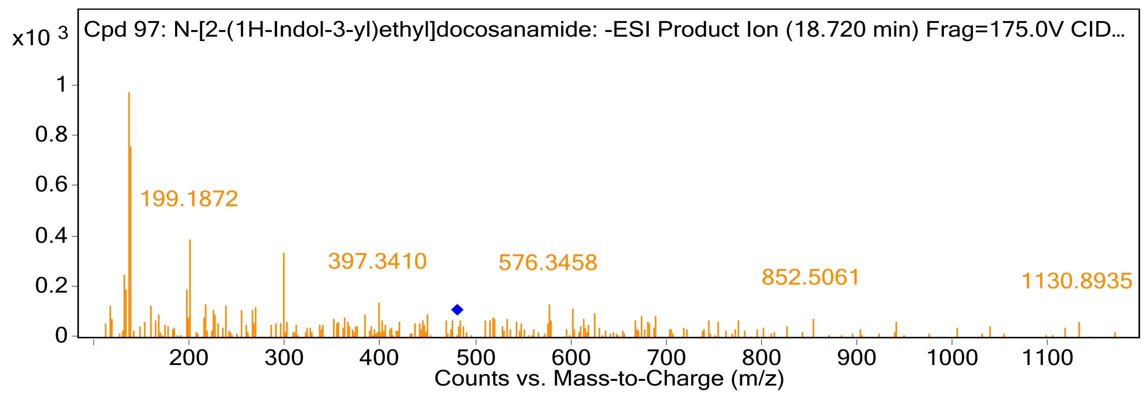

MS/MS Spectrum Peak List

m/z	z	Abund
130.092		254.09
132.9037		190.98
134.8983	1	977.59
135.8988	1	183.53
136.8956	1	758.18
195.8151		191.5
199.1872		390.37
297.1587	1	341.55
397.341		141.55
576.3458		136.21

Compound Structure

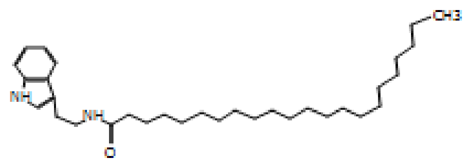

**Compound Label**	**Name**	***m/z***	**RT**	**Algorithm**	**Mass**
Cpd 98: 22,23-Dihydroergosterol	**22,23-Dihydroergosterol**	397.3417	18.864	Auto MS/MS	398.3498

MS Spectrum

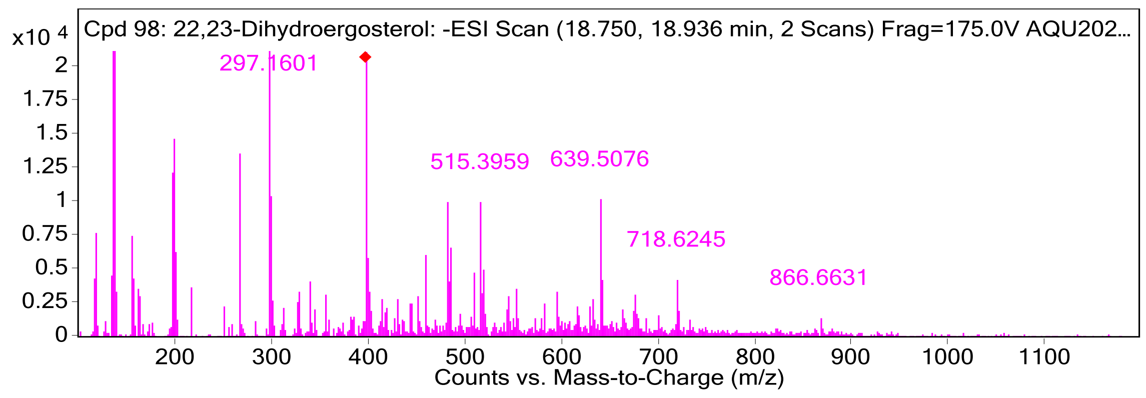

MS Zoomed Spectrum

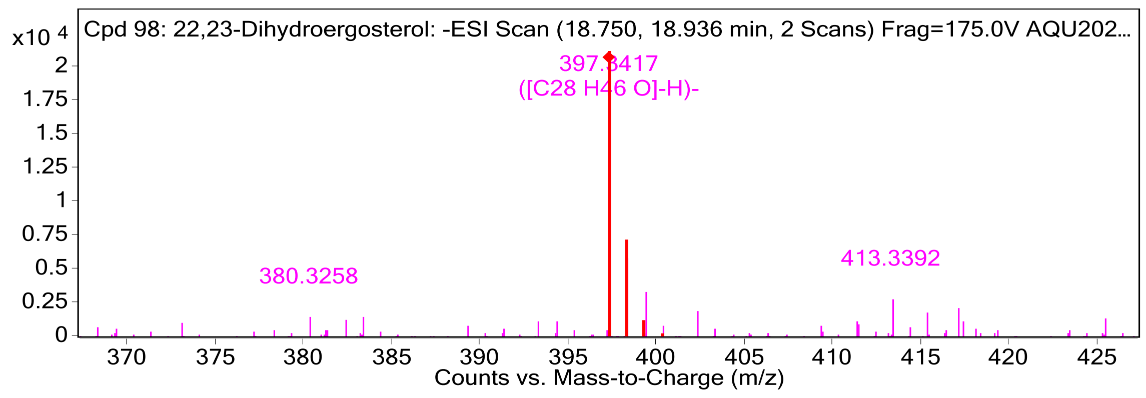

MS Spectrum Peak List

m/z	*Calc m/z*	Diff(ppm)	z	Abund	Formula	Ion
134.8986			1	99024.86		
136.8958			1	32063.38		
195.8163				12153.89		
197.8134				14706.34		
265.1544			1	13559.24		
297.1601			1	61523.48		
397.3417	397.3476	14.76	1	21126.2	C28 H46 O	(M-H)-
398.3444	398.351	16.6	1	5929.02	C28 H46 O	(M-H)-
399.3551	399.3543	-1.98	1	3335.91	C28 H46 O	(M-H)-
400.3598	400.3575	-5.81	1	871.98	C28 H46 O	(M-H)-

MSMS Spectrum

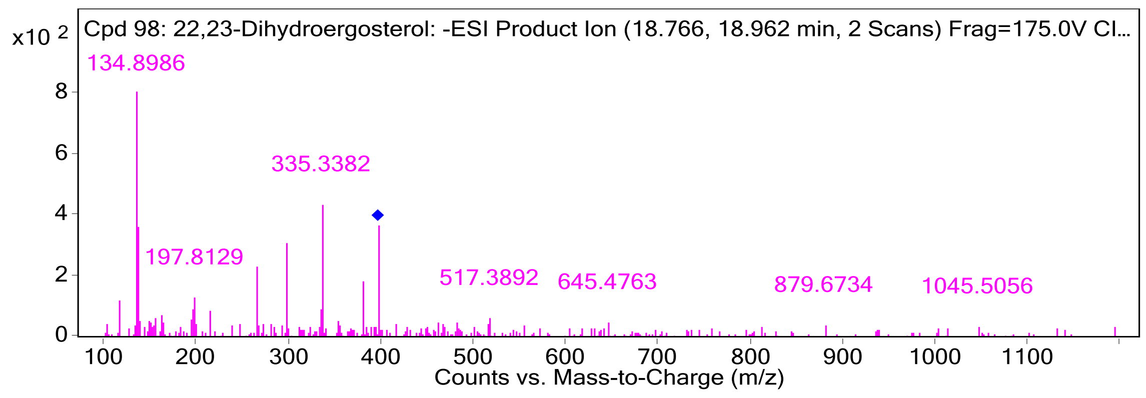

MS/MS Spectrum Peak List

m/z	z	Abund
134.8986	1	807.56
136.8961	1	364.54
197.8129		128.42
265.1556	1	234.12

297.1611	1	310.99
335.3382		435.31
336.3419		241.19
379.3296		153.31
380.3299		184.65
397.3407	1	367

Compound Structure

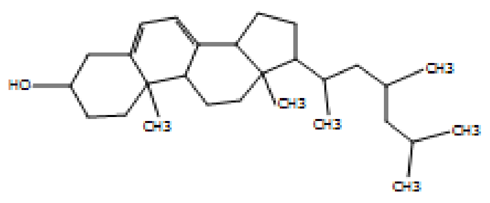

Compound Label	Name	*m/z*	RT	Algorithm	Mass
Cpd 99: Atenolol	**Atenolol**	265.1545	19.04	Auto MS/MS	266.1615

MS Spectrum

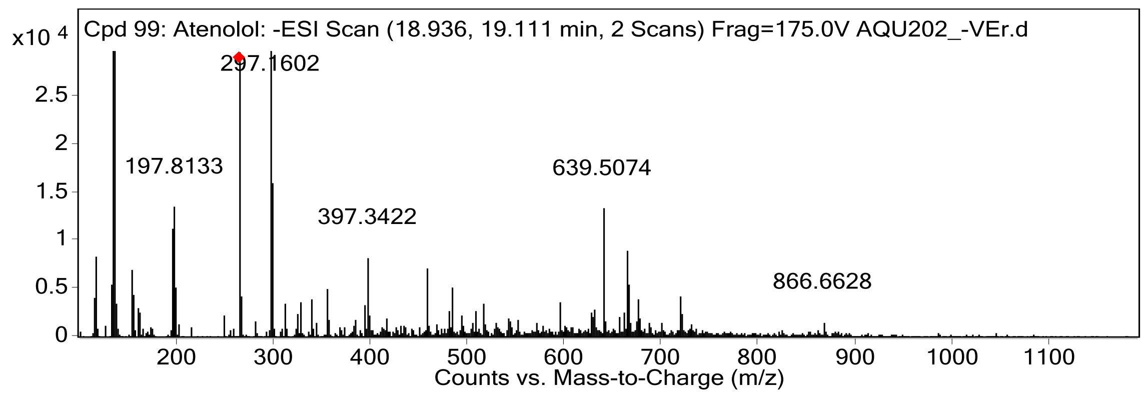

MS Zoomed Spectrum

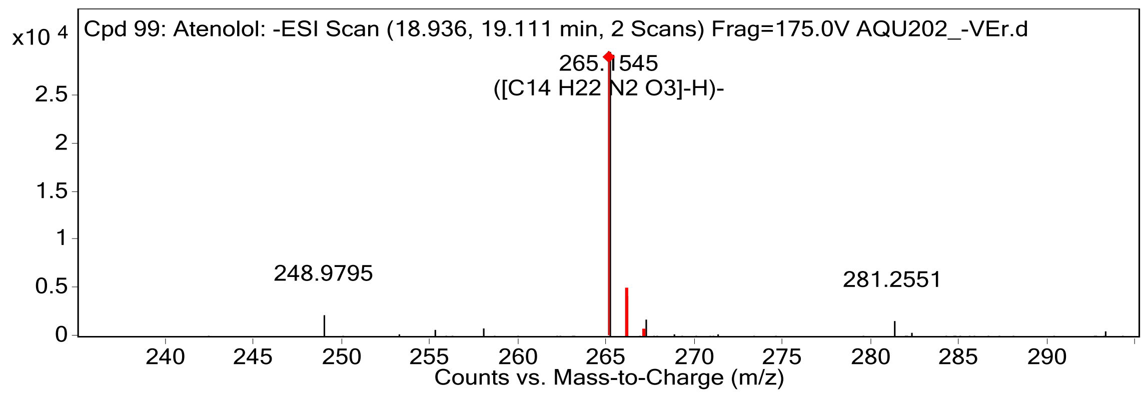

MS Spectrum Peak List

m/z	*Calc m/z*	Diff(ppm)	z	Abund	Formula	Ion
134.8986			1	110296.81		
136.8958			1	36321.89		
195.8161				11341.07		
197.8133				13547.27		
265.1545	265.1558	4.6	1	29580.59	C14 H22 N2 O3	(M-H)-
266.1575	266.1589	5.25	1	4259.2	C14 H22 N2 O3	(M-H)-
267.1537	267.1613	28.41	1	1758.35	C14 H22 N2 O3	(M-H)-
297.1602			1	88256.13		
298.1632			1	16039.73		
639.5074			1	13378.37		

MSMS Spectrum

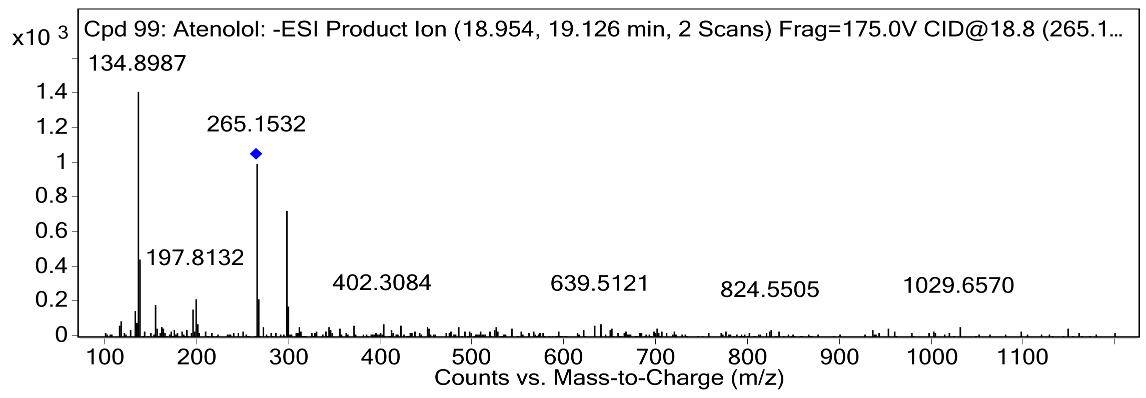

MS/MS Spectrum Peak List

m/z	z	Abund
132.9033		156.18
134.8987	1	1411.82
136.8958	1	447.47
153.8716		189.35
195.8164		161.26
197.8132		221.93
265.1532	1	992.12
266.1581	1	223.01
297.1605	1	729.55
298.1639	1	177.24

Compound Structure

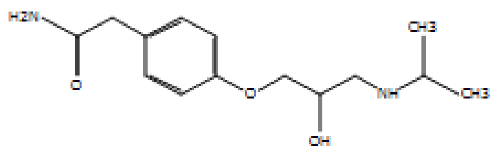

Compound Label	Name	*m/z*	RT	Algorithm	Mass
Cpd 100: Atenolol	**Atenolol**	265.1548	19.387	Auto MS/MS	266.1618

MS Spectrum

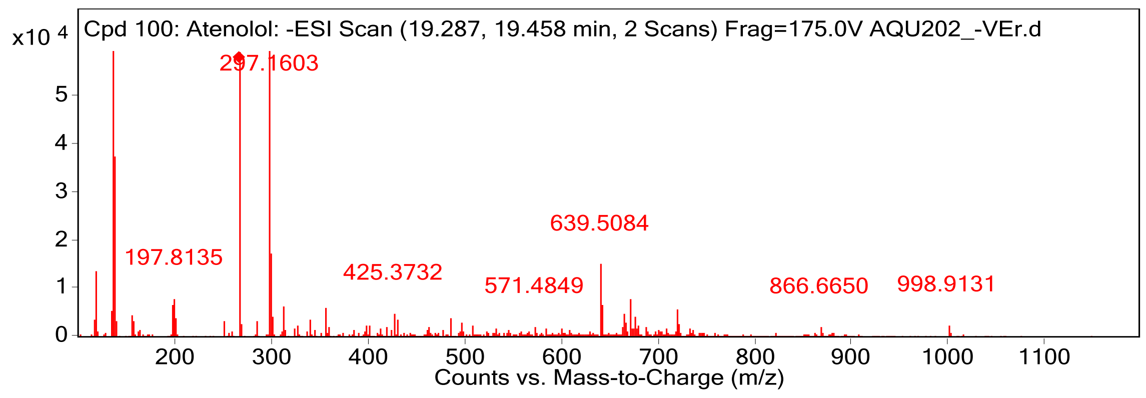

MS Zoomed Spectrum

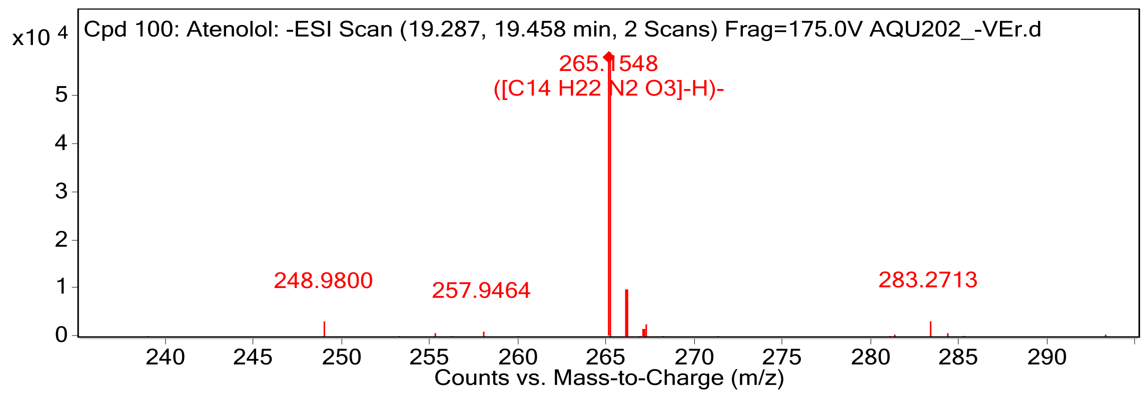

MS Spectrum Peak List

m/z	*Calc m/z*	Diff(ppm)	z	Abund	Formula	Ion
116.9323			1	13620.49		
134.8987			1	114425.27		
135.8989			1	11947.55		
136.8959			1	37635.16		
265.1548	265.1558	3.58	1	59242.6	C14 H22 N2 O3	(M-H)-
266.1577	266.1589	4.36	1	7840.2	C14 H22 N2 O3	(M-H)-
267.1526	267.1613	32.5	1	2846.88	C14 H22 N2 O3	(M-H)-
297.1603			1	96976.47		
298.1636			1	17449.44		
639.5084			1	15196.56		

MSMS Spectrum

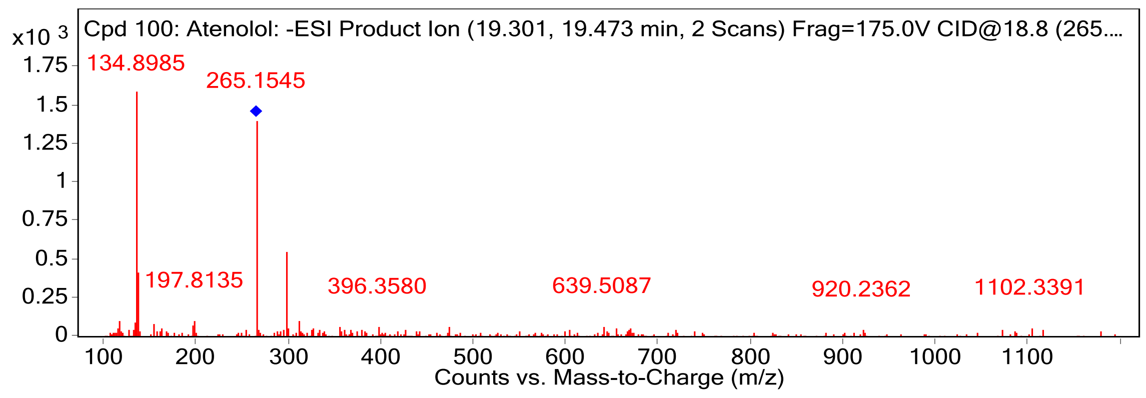

MS/MS Spectrum Peak List

m/z	z	Abund
116.9327		107.39
134.8985	1	1592.81
135.8991	1	158.28
136.8957	1	417.25
197.8135	1	103.13
265.1545	1	1401.08
266.1555	1	156.61
297.1606	1	548.66
298.1653	1	119.05
311.1747	1	109.09

Compound Structure

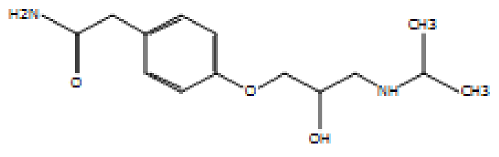

Compound Label	Name	*m/z*	RT	Algorithm	Mass
Cpd 101: Atenolol	**Atenolol**	265.1549	19.766	Auto MS/MS	266.1619

MS Spectrum

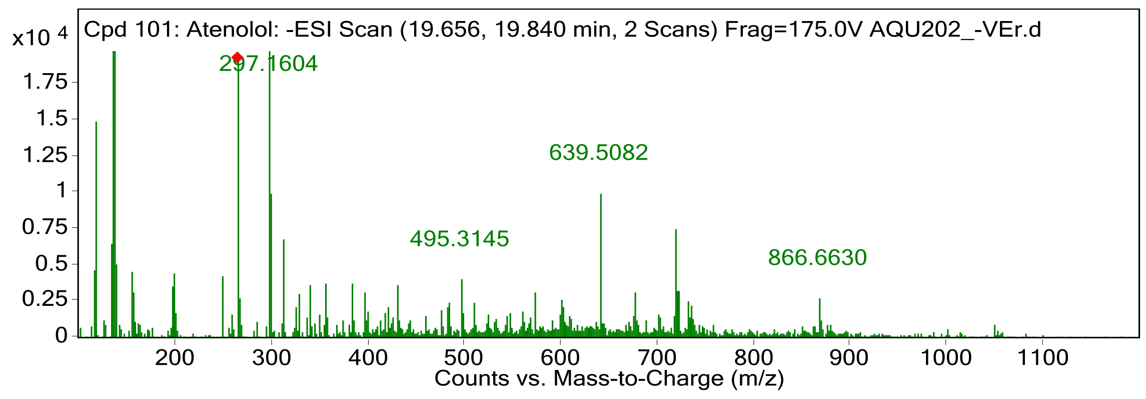

MS Zoomed Spectrum

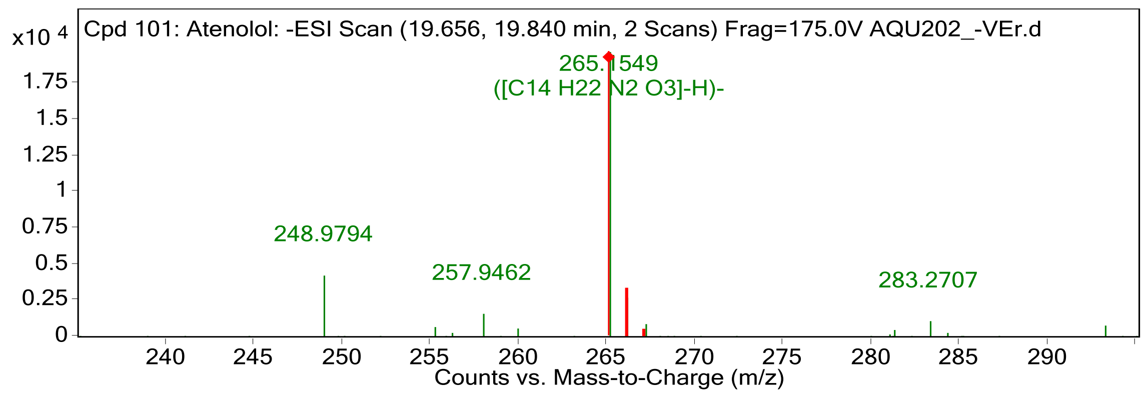

MS Spectrum Peak List

m/z	*Calc m/z*	Diff(ppm)	z	Abund	Formula	Ion
116.9323			1	14887.15		
134.8987			1	141501.59		
135.8991			1	15751.71		
136.8958			1	46441.98		
265.1549	265.1558	3.21	1	19642.91	C14 H22 N2 O3	(M-H)-
266.1582	266.1589	2.75	1	2765.04	C14 H22 N2 O3	(M-H)-
267.1528	267.1613	32.05	1	884.34	C14 H22 N2 O3	(M-H)-
297.1604			1	52591.33		
298.1632			1	9970.41		
639.5082			1	9957.07		

MSMS Spectrum

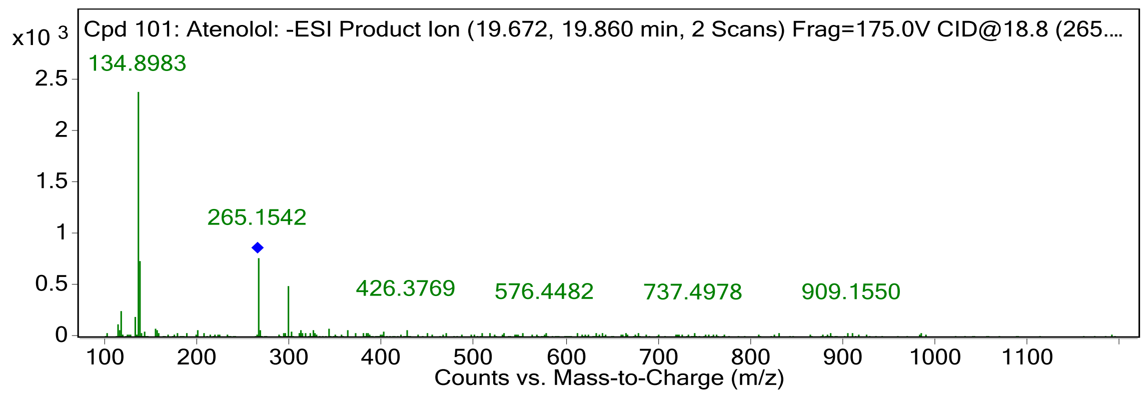

MS/MS Spectrum Peak List

m/z	z	Abund
112.9868		123.81
116.932		257.03
132.9039		194.84
134.8983	1	2391.55
135.8969	1	170.03
136.8954	1	748.38
153.8721		91.96
265.1542	1	770.1
266.157	1	107.14
297.1596	1	497.23

Compound Structure

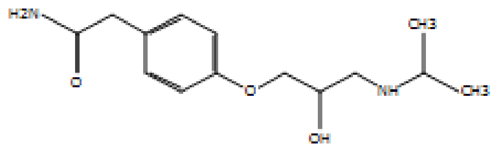

--- End Of Report ---
